# Circadian and light-driven regulation of rod dark adaptation

**DOI:** 10.1038/srep17616

**Published:** 2015-12-02

**Authors:** Yunlu Xue, Susan Q. Shen, Joseph C. Corbo, Vladimir J. Kefalov

**Affiliations:** 1 Washington University School of Medicine, St. Louis, Missouri 63110, USA; 2 Department of Ophthalmology & Visual Sciences, Washington University School of Medicine, St. Louis, Missouri 63110, USA; 3 Department of Pathology & Immunology, Washington University School of Medicine, St. Louis, Missouri 63110, USA; 4 Graduate Program in Division of Biological & Biomedical Sciences, Washington University School of Medicine, St. Louis, Missouri 63110, USA.

## Abstract

Continuous visual perception and the dark adaptation of vertebrate photoreceptors after bright light exposure require recycling of their visual chromophore through a series of reactions in the retinal pigmented epithelium (RPE visual cycle). Light-driven chromophore consumption by photoreceptors is greater in daytime vs. nighttime, suggesting that correspondingly higher activity of the visual cycle may be required. However, as rod photoreceptors are saturated in bright light, the continuous turnover of their chromophore by the visual cycle throughout the day would not contribute to vision. Whether the recycling of chromophore that drives rod dark adaptation is regulated by the circadian clock and light exposure is unknown. Here, we demonstrate that mouse rod dark adaptation is slower during the day or after light pre-exposure. This surprising daytime suppression of the RPE visual cycle was accompanied by light-driven reduction in expression of *Rpe65*, a key enzyme of the RPE visual cycle. Notably, only rods in melatonin-proficient mice were affected by this daily visual cycle modulation. Our results demonstrate that the circadian clock and light exposure regulate the recycling of chromophore in the RPE visual cycle. This daily melatonin-driven modulation of rod dark adaptation could potentially protect the retina from light-induced damage during the day.

The retina provides vertebrate animals with information about the world around them and the overall light intensity. Detailed visual information is generated by rod and cone photoreceptors, which are responsible for dim- and bright-light vision, respectively. The function of the retina is modulated by daily changes in ambient light conditions and by an intrinsic circadian clock^1^. These mechanisms regulate many retinal functions, including melatonin synthesis^2^, the electrical coupling between photoreceptors^3^,^4^, and synaptic transmission^5^, to fine-tune visual processing in the retina^6^. The susceptibility to light-induced retinal damage is also higher in subjective (circadian) night than in subjective day^7^. Although the mechanisms by which the circadian clock regulates this process is not understood, it is likely to be related to the light-sensing visual pigments in photoreceptors.

Light detection is initiated in the retina when a photon is absorbed by the visual pigment in photoreceptors. This causes the conversion of the visual chromophore 11-*cis* retinal to its all-*trans* form, activating the visual pigment and triggering the phototransduction cascade that ultimately results in the electric response of the cell^8^. Resetting of the photoactivated (bleached) visual pigment to its ground state requires removal of the spent all-*trans* chromophore from photoreceptors and its recycling back to its 11-*cis* form in the RPE cells (RPE visual cycle; for both rods and cones) or in the retinal Müller cells (retina visual cycle; for cones only)^9^,^10^. Notably, even though rods are saturated during the day, their visual pigment still continuously cycles through bleaching and regeneration. As a result, in rod-dominant species like mouse and human, rods consume the bulk of the chromophore recycled by the RPE visual cycle^11^, while chromophore recycled by the retina visual cycle allows cones to rapidly regenerate their visual pigment^12^,^13^. The accumulation of retinoid byproducts with age or as a result of a dysfunctional visual cycle can cause retinal degeneration and blindness^14^.

Chromophore consumption varies greatly during the day-night cycle. During the day, the visual pigments in rods and cones are photobleached at a high rate, whereas a minimal amount of chromophore is used and recycled at night. This day/night difference in chromophore consumption prompted us to ask: is pigment regeneration under the regulation of the circadian clock, in accordance with chromophore demand? Does light modulate the efficiency of chromophore recycling? One of the key processes modulated by both the circadian clock and light exposure is melatonin synthesis, which is suppressed during the circadian daytime and by light. Thus, we addressed these questions by electrophysiological recordings and molecular analysis of retinas of melatonin-proficient (C3H/f^+/+^ and CBA/CaJ) and melatonin-deficient (C57BL/6J and 129S2/Sv) mouse strains.

## Results

### Rod dark adaptation in melatonin-proficient mice is regulated by the circadian clock

**Figure 1:**
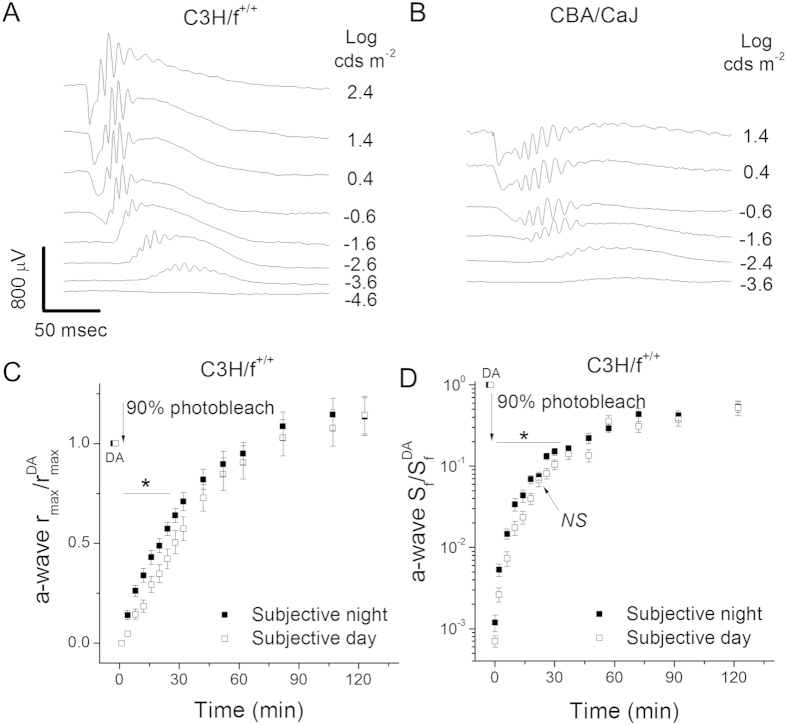
Effect of the circadian clock on rod dark adaptation in melatonin-proficient mice. (**A**) Representative dark-adapted scotopic *in vivo* ERG responses to various light intensities from melatonin-proficient C3H/f^+/+^ mice. (**B**) Representative dark-adapted scotopic *in vivo* ERG responses from melatonin-proficient CBA/CaJ mice revealing b-wave deficit. (**C**) Normalized *in vivo* ERG scotopic a-wave maximal response (a-wave r_max_/r^DA^_max_) recovery in C3H/f^+/+^ mice following 90% pigment bleach at t = 0 at subjective night (solid squares, n = 15) and subjective day (open squares, n = 16) (*p < 0.05). (**D**) Normalized *in vivo* ERG scotopic a-wave sensitivity (a-wave S_f_/a-wave S_f_^DA^) recovery in C3H/f^+/+^ mice following 90% pigment bleach at t = 0 at subjective night (solid squares, n = 15) and subjective day (open squares, n = 16) (*p < 0.05). Here and in all subsequent figures, DA refers to the initial dark-adapted value of the a-wave maximal response or sensitivity.

The goal of our study was to determine if pigment regeneration is regulated by the circadian clock or by light. Each of these two retinal signals strongly regulates the expression of melatonin at night, which in turn affects many processes in the retina^15^,^16^. Thus, we investigated rod dark adaptation in melatonin-proficient (C3H/f^+/+^ and CBA/CaJ) and melatonin-deficient (C57BL/6J and 129S2/Sv) mouse strains. We began with the melatonin-proficient C3H/f^+/+^ strain of mice^15^, first testing their *in vivo* electroretinogram (ERG) responses^13^. We observed robust dark-adapted (scotopic) responses with a normal waveform (Fig. 1A). Measurements of their maximal a-wave amplitudes (r_max_) at subjective night, 6 hours after scheduled lights-off (18 o’clock predicted circadian time, CT 18; 30 hours of actual dark adaptation), and at subjective day, 6 hours after scheduled light-on (CT 6; 18 hours of actual dark adaptation) were comparable (Table 1). Similarly, scotopic a-wave dim flash sensitivity (S_f_) in C3H/f^+/+^ mice was not affected by the time of day of the recordings (Table 1). Thus, our results from dark-adapted C3H/f^+/+^ mice revealed no circadian regulation of their scotopic a-wave responses, indicating that their photoreceptor function in darkness is not modulated by the circadian clock.

In order to determine whether pigment regeneration is regulated by the circadian clock, we next examined the kinetics of rod dark adaptation in C3H/f^+/+^ mice in subjective night and subjective day. The dark adaptation experiments were performed with mice that were dark adapted for 30 hours (for the CT 18 time point) or 18 hours (for the CT 6 time point). As mouse rod pigment regeneration and dark adaptation are typically complete within one hour^11^,^17^, such conditions allowed for full dark-adaptation prior to the experiment for both time points. This notion was also supported by the comparable scotopic a-wave sensitivities at CT 18 and CT 6. A bright 30 seconds light exposure, estimated to bleach >90% of the visual pigment, instantly reduced the a-wave amplitude to near threshold levels (Fig. 1C), while the a-wave sensitivity declined by ~1000-fold, as the flash intensity required to produce a measurable response had to be increased by 3 log units (Fig. 1D). Consecutive measurements of these parameters in darkness over the next two hours revealed the gradual dark adaptation of the rods as their pigment regenerated. Notably, the recovery of both the amplitude and sensitivity of the a-wave over the first ~30 min of dark adaptation was significantly (p < 0.05, two-tailed Student’s t-test) slower for the subjective day group than for the animals in subjective night (Fig. 1C,D). As a result, the initial recovery of rod a-wave response in subjective day was delayed by ~8 minutes compared to that in subjective night (Fig. 1C). Similarly, the rod sensitivity 30 min after the bleach in subjective day was 2-fold lower than that in subjective night (Fig. 1D). Eventually, the recovery levels became comparable, so that the tails of the dark adaptation (final 25% of r_max_ and final 5-fold of S_f_ recovery) were similar in the two groups. These results demonstrate that the time course of rod dark adaptation in C3H/f^+/+^ mice is delayed during the subjective day compared to subjective night. Thus, mouse rod dark adaptation is modulated by the circadian clock.

We sought to establish the regulation of rod dark adaptation in another melatonin-proficient strain, CBA/CaJ^1^. However, the ERG recordings from these mice revealed a prominent b-wave amplitude loss and extended a- and b-wave implicit times (Fig. 1B), reminiscent of the phenotype caused by mutation in *Gpr179*, a G-protein coupled receptor in ON-bipolar cells^18^,^19^. Thus, this strain of CBA/CaJ mice proved unsuitable for our physiological analysis. The abnormal ERG responses from these mice also call for caution when using this strain for molecular analysis of the retinal circadian clock.

### Rod dark adaptation in melatonin-proficient mice is regulated by light history

**Figure 2:**
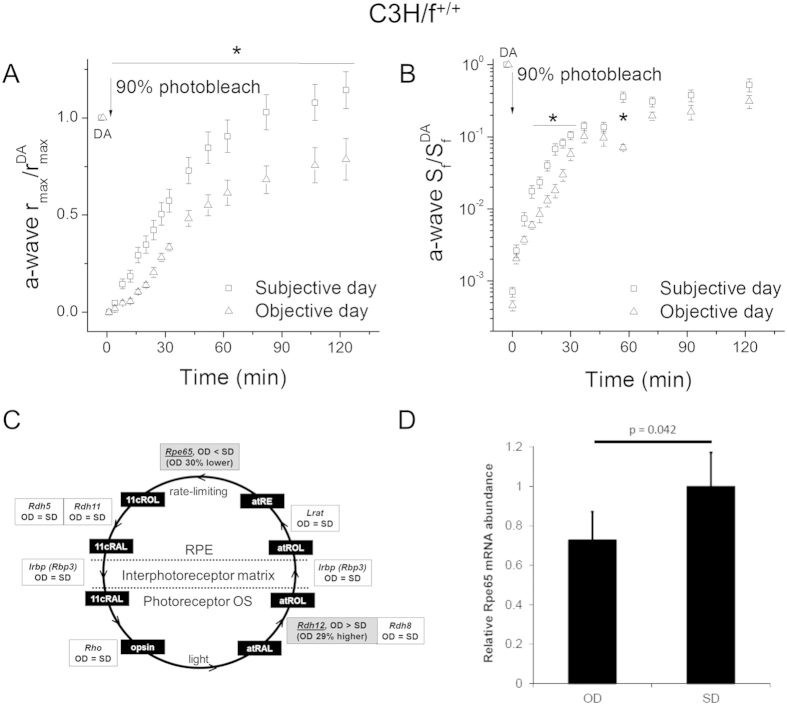
Effect of the light history on rod dark adaptation in melatonin-proficient C3H/f^+/+^ mice. (**A**) Normalized *in vivo* ERG scotopic a-wave maximal response (a-wave r_max_/a-wave r^DA^_max_) recovery in C3H/f^+/+^ mice following 90% pigment bleach at t = 0 at subjective day (replotted from Fig. 1C, open squares) and objective day (open triangles, n = 7) (*p < 0.05). (**B**) Normalized *in vivo* ERG scotopic a-wave sensitivity (a-wave S_f_/a-wave S_f_^DA^) recovery in C3H/f^+/+^ mice following 90% pigment bleach at t = 0 at subjective day (replotted from Fig. 1D, open squares) and objective day (open triangles, n = 7) (*p < 0.05). (**C**) Components of the RPE visual cycle and their expression by RNA-seq in objective day (OD) vs. subjective day (SD). In the photoreceptor outer segment (OS), the absorption of light by rhodopsin (*Rho*) causes conversion of 11-*cis* retinal (11cRAL) to all-*trans* retinal (atRAL). Next, atRAL is reduced to all-*trans* retinol (atROL) by RDH8 or RDH12 and exported to the RPE. There, atROL is converted to all-*trans* retinyl-ester (atRE) by LRAT. Subsequently, atRE is converted to 11-*cis* retinol (11cROL) by RPE65 in a rate-limiting step. Finally, 11cROL is oxidized to 11cRAL by RDH5 or RDH11 and imported back into the OS. IRBP is a binding protein in the interphotoreceptor matrix. Comparison of gene expression levels in OD vs. SD: gray shading indicates a significant difference at FDR  =  0.05. (**D**) *Rpe65* mRNA abundance in objective day (OD) vs. subjective day (SD) in C3H/f^+/+^ mice as quantified by qRT-PCR. Transcript levels of *Rpe65* were normalized to *Gapdh.* Error bars represent standard error of the mean (SEM) across five biological replicates per condition. P-value, two-tailed Student’s t-test.

In addition to the circadian clock, exposure to light may also directly affect animal physiology, particularly in the light-sensitive retina^5^. Thus, we next sought to determine if rod dark adaptation is regulated by light history. To accomplish this, we compared the dark adaptation of C3H/f^+/+^ mouse rods *in vivo* at 12 o’clock (noon) but dark adapted for 30 hours (subjective day, CT 6), or pre-exposed to light in the morning and then dark adapted for 1 hour before the experiment (objective day, zeitgeber time ZT 6). The 1 hour of darkness was sufficient to fully dark-adapt the rods in unanesthetized mice and restore their *in vivo* ERG a-wave sensitivity and maximal response amplitude (Table 1, compare values for subjective day, dark-adapted for 18 hours, and objective day, dark-adapted for 1 hour). Comparison of rod dark adaptation in subjective and objective day demonstrated that both a-wave maximal response (Fig. 2A) and a-wave sensitivity (Fig. 2B) recovered significantly more slowly during the objective day. Thus, our results revealed that rod dark adaptation in melatonin-proficient mice is suppressed by pre-exposure to light. Notably, the effect of light on the rod visual cycle was substantially more prominent than that induced by the circadian clock (Fig. 1).

Given the electrophysiological findings that light exposure suppresses rod dark adaptation, we hypothesized that there was an underlying molecular downregulation of the visual cycle in objective day. Accordingly, we conducted RNA-seq and differential expression analysis of the eyes of subjective day and objective day groups. A total of 1,460 genes were found to be significantly differentially expressed (FDR = 0.05) (Supplemental Table S1), with most of them (1,298 or 89%) expressed at lower levels in objective day than subjective day. Among the genes with altered expression, we identified two known visual cycle genes, *Rpe65* and *Rdh12* (Fig. 2C). RPE65 converts all-*trans* retinal esters (atRE) into 11-*cis* retinol (11cROL) in the RPE^20^, while RDH12 converts all-*trans* retinal (atRAL) to all-*trans* retinol (atROL) in photoreceptors^21^. Interestingly, *Rpe65* levels were 30% lower in objective day than subjective day, whereas *Rdh12* levels were 29% higher in objective day than subjective day. The downregulation of *Rpe65* would delay the recycling of chromophore in the RPE and the overall visual cycle^22^, whereas *Rdh12* upregulation would accelerate the reduction of toxic all-*trans* retinal and its clearance from the rods^23^. To verify the RNA-seq results, we examined *Rpe65* transcript levels by quantitative RT-PCR (qRT-PCR) in an independent set of biological replicates^24^. In good agreement with RNA-seq, we found that *Rpe65* levels were 27% lower in objective day than subjective day by qRT-PCR (Fig. 2D). Overall, these molecular studies suggest that the light-driven slowing of rod dark adaptation may be mediated by *Rpe65*–dependent suppression of the RPE visual cycle.

### Rod dark adaptation in melatonin-deficient C57BL/6 J rods is not affected by the circadian clock or light history

**Figure 3:**
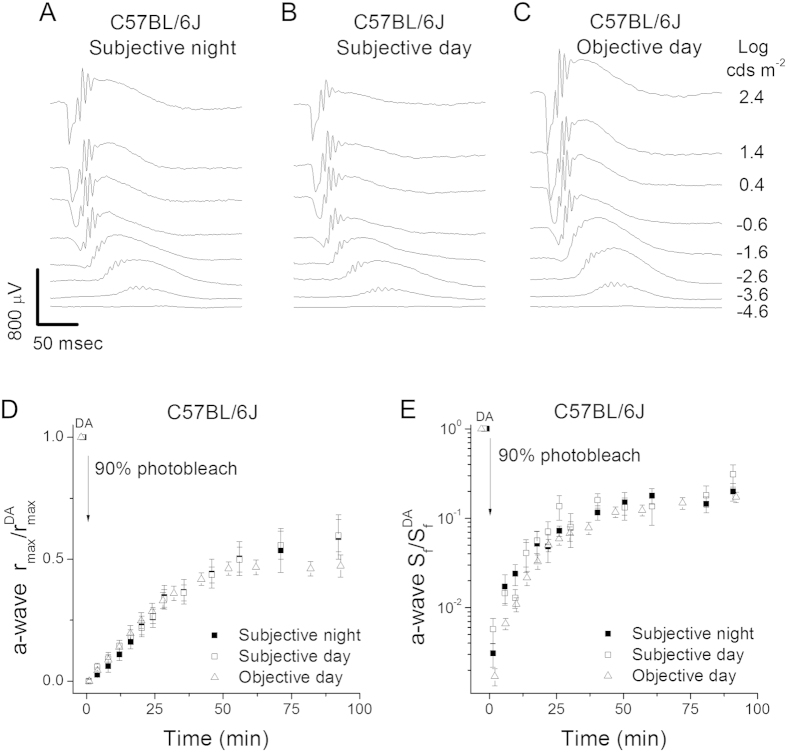
Lack of effect by the circadian clock and light history on rod dark adaptation in melatonin-deficient C57BL/6 J mice. Representative scotopic *in vivo* ERG responses to various light intensities from C57BL/6J mice at (**A**) subjective night, (**B**) subjective day, and (**C**) objective day. (**D**) Normalized *in vivo* ERG scotopic a-wave maximal response (a-wave r_max_/a-wave r^DA^_max_) recovery in C57BL/6J mice following 90% pigment bleach at t = 0 at subjective night (solid squares, n = 7), subjective day (open squares, n = 6), and objective day (open triangles, n = 10). (**E**) Normalized *in vivo* ERG scotopic a-wave sensitivity (a-wave S_f_/a-wave S_f_^DA^) recovery in C57BL/6J mice following 90% pigment bleach at t = 0 at subjective night (solid squares, n = 7), subjective day (open squares, n = 6), and objective day (open triangles, n = 10).

We next investigated whether the changes in dark adaptation we observed in C3H/f^+/+^ mouse rods were mediated by melatonin. To address this question, we examined the function of melatonin-deficient C57BL/6J mouse rods using *in vivo* ERG recordings at subjective day (CT 6), subjective night (CT 18), and objective day (ZT 6). First, we measured the dark-adapted scotopic intensity-response curves at subjective day, subjective night, and objective day (Fig. 3A–C). The results revealed that the ERG a-wave amplitudes were slightly increased in subjective night compared to subjective day (Fig. 3A,B; Table 1). A further increase in a-wave amplitudes was observed in objective day (Fig. 3C). Then we performed the dark adaptation test as above to probe the operation of the C57BL/6J RPE visual cycle at subjective day, subjective night, and objective day. In contrast to the melatonin-proficient C3H strain, we found that the kinetics of C57BL/6J rod dark adaptation at CT 6, CT 18, and ZT 6 were identical as measured by both a-wave amplitude (Fig. 3D) and sensitivity (Fig. 3E).

**Figure 4:**
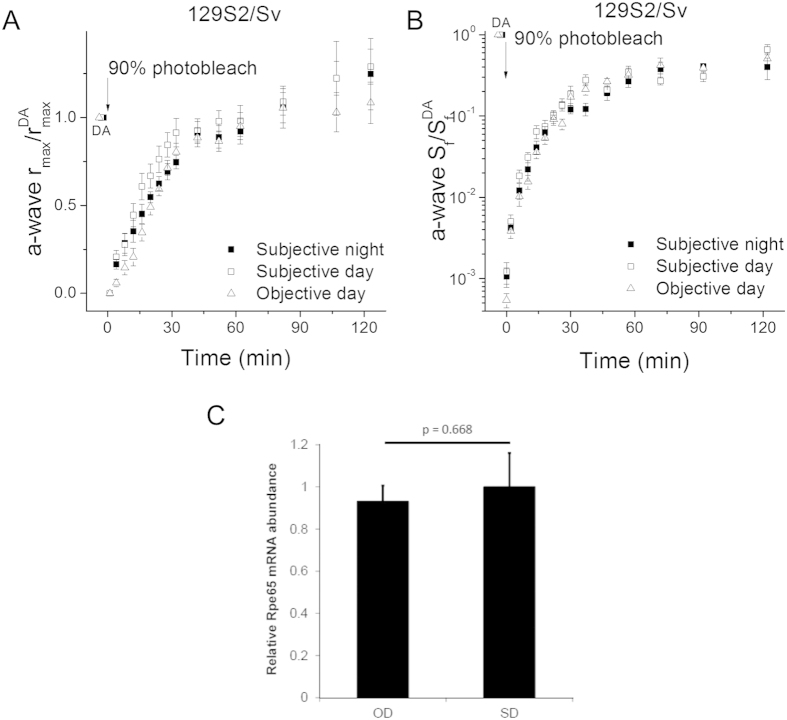
Lack of effects of the circadian clock and light history on rod dark adaptation in melatonin-deficient 129S2/Sv mice. (**A**) Normalized *in vivo* ERG scotopic a-wave maximal response recovery in 129S2/Sv mice following 90% pigment bleach at t = 0 at subjective night (solid squares, n = 6), subjective day (open squares, n = 8), and objective day (open triangles, n = 7). (**B**) Normalized *in vivo* ERG scotopic a-wave sensitivity recovery in 129S2/Sv mice following 90% pigment bleach at t = 0 at subjective night (solid squares, n = 6), subjective day (open squares, n = 8) and objective day (open triangles, n = 7). (**C**) *Rpe65* mRNA abundance in objective day (OD) vs. subjective day (SD) in 129/Sv mice as quantified by qRT-PCR. Transcript levels of *Rpe65* were quantified by qRT-PCR with normalization to *Gapdh.* Error bars represent standard error of the mean (SEM) across five biological replicates per condition. P-value, two-tailed Student’s t-test.

We obtained similar results from another melatonin-deficient mouse strain, 129S2/Sv. As in C3H mice, the scotopic a-wave maximal amplitude and sensitivity of 129S2/Sv mice were comparable among subjective night, subjective day and objective day (Table 1). However, unlike the case of C3H mice and similar to the C57BL/6 J mice, the circadian time or light exposure of 129S2/Sv mice prior to the experiment failed to modulate the dark adaptation of their rods (Fig. 4A,B). Consistent with this observation, we found no significant difference in the transcript levels of *Rpe65* in objective day vs subjective day of 129S2/Sv mice as examined by qRT-PCR (Fig. 4C). Thus, rod dark adaptation in melatonin-deficient C57BL/6J and 129S2/Sv mice was not affected by the circadian clock or light exposure history.

## Discussion

### Melatonin-mediated regulation of mouse rod dark adaptation

The circadian clock and light regulate many processes in the retina^25^. However, despite the large difference in the rates of visual pigment photoactivation at night and during the day, it was not previously known whether the recycling of chromophore and the regeneration of visual pigment are also subject to such regulation. Our results clearly demonstrate that both the circadian clock and light exposure slow down the dark adaptation of rods in melatonin-proficient mice during the day. Thus, rod pigment regeneration in these mice is modulated by the combined effects of the circadian clock and light, such that rod dark adaptation is substantially slowed in the daytime, when rods are largely saturated.

Although the exact mechanism of this regulation is still unclear, our finding that *Rpe65* expression is suppressed by light, together with its previously described diurnal regulation in the melatonin-proficient CBA/CaJ strain^1^, suggest that it likely involves modulation of the efficiency of chromophore recycling by the RPE visual cycle. As this is the only mechanism for regeneration of the rod visual pigment and the rate-limiting step for rod dark adaptation^8^,^11^, slowing the RPE visual cycle would cause a corresponding delay in the regeneration of rod pigment and in the dark adaptation of rods.

Melatonin is produced not only by the pineal gland but also locally in the retina by photoreceptor cells at night^26^, where it regulates many aspects of mammalian retinal physiology (see ref. 16 for review). The rhythmicity of melatonin biosynthesis also drives diurnal retinal dopamine synthesis, which peaks during the day^15^, further amplifying the robustness of the retina-intrinsic circadian clock. Melatonin receptors have been identified in various ocular cell types, including photoreceptors, RPE, and Müller cells^6^,^27^,^28^. However, many commonly used inbred strains of laboratory mice, including C57BL/6J, 129S2/Sv and BALB/c, have lost their ability to produce melatonin due to mutations in key melatonin biosynthesis enzymes^29^,^30^. In contrast, C3H mice still retain their ability to synthesize melatonin. Thus, our observation that rod dark adaptation is subject to regulation by the circadian clock and light in C3H, but by neither the circadian clock nor light in C57BL/6J and 129S2/Sv mice, suggests a role for melatonin in this process. Thus, the simplest explanation for our results is that the efficiency of the RPE visual cycle is modulated by the daily oscillation of melatonin.

### The daily modulation of the RPE visual cycle and rod-mediated vision

The high sensitivity of rods enables them to detect low light levels and mediate dim light vision. However, the high amplification that produces this exquisite rod sensitivity also results in the saturation of the rods at moderately bright light conditions^31^. Despite this fact, the rod visual pigment continues to undergo bleaching and regeneration throughout the day. As this process involves multiple enzymatic reactions both in the rods and in the RPE cells, it imposes a significant metabolic load on the visual system. Therefore, the downregulation of the RPE visual cycle during the day by both the circadian clock and light history would conserve energy without significantly compromising rod-mediated vision. The corresponding acceleration of all-*trans* retinal reduction in the rods, as suggested by the observed upregulation of *Rdh12*, would minimize the toxic effects of this compound^32^ and prevent the formation and accumulation of related toxic byproducts^33^. At the same time, as the cones rely predominantly on the alternative retina visual cycle for the bulk of their dark adaptation^13^,^34^ and for chromophore supply during cone opsin synthesis^35^, the suppression of the RPE visual cycle would not be expected to compromise cone-mediated daytime vision.

Another possible benefit of downregulating the RPE visual cycle during the day is the protection of the retina from light damage. It is known that mice with lower *Rpe65* expression have a slower rod dark adaptation and higher resistance to light-induced rod degeneration^36^, presumably because the slower turnover of visual pigment reduces the accumulation of toxic retinoid byproducts. Similarly, the down-regulation of the RPE visual cycle during the day could be a mechanism to protect photoreceptors from light damage. Consistent with this hypothesis, in rats, retinas are more susceptible to light-induced damage at night^37^,^38^. Our finding that the RPE visual cycle is faster at night provides a mechanistic explanation for this observation. Therefore, a rhythmic melatonin-driven diurnal suppression of the RPE visual cycle may protect the retina from degeneration by lowering the susceptibility of photoreceptors to light damage during the day. Indeed, lack of melatonin-dependent RPE visual cycle regulation could be involved in the enhanced age-dependent retinal degeneration in mice lacking the melatonin receptors MT1 and MT2^6^,^39^. Conversely, enhancing the diurnal suppression of the RPE visual cycle by oral intake of melatonin could be one of the factors that reduce the risk of human age-related macular degeneration (AMD)^40^.

## Methods

### Animals

The maintenance and treatment of the mice was in compliance with the protocols approved by the Washington University Animal Studies Committee. Melatonin-deficient C57BL/6J and melatonin-proficient CBA/CaJ and C3H/f^+/+^ mice were purchased from Jackson Laboratory (Bar Harbor, ME). The C3H/f^+/+^ mice are also known as C3A.BLiA-Pde6b^+^/J, originally generated by Willem J. de Grip (Erasmus Universiteit, Netherlands)^15^,^41^. Melatonin-deficient 129S2/Sv mice were purchased from Charles River Laboratories (Wilmington, MA). Unlike the original C3H strain, the C3H/f^+/+^ mice used in our study were free of the PDE6b mutation that causes retinal degeneration, and had normal retinal morphology and light-driven responses^6^. All animals used in this study were free of the *rd8* mutation^42^. The animals were raised in a 12 hr:12 hr light-dark cycle and entrained in a 420 cd m^−2^ light environment for a week before the experiments. Age-matched animals were grouped into three categories: subjective night, subjective day, and objective day. Subjective night groups were dark-adapted in a light-proof cabinet for 30 hours and tested at 18 circadian time (CT; midnight). Subjective day groups were dark-adapted for 18 hours and tested at CT 6 (noon). Objective day groups were dark-adapted for 1 hour and tested at 6 zeitgeber time (ZT, noon).

### *In vivo* electroretinography (ERG)

The method for studying mouse rod dark adaptation *in vivo* using LKC® ERG system had been described in detail previously^13^. Briefly, dark-adapted animals were anesthetized with ketamine/xylazine cocktail (100/20 mg/kg) by intraperitoneal injection. The pupils of the anesthetized animals were dilated with a drop of 1% atropine sulfate solution and the animals were transferred to a 37 °C heating pad with a feedback anal thermal probe. The reference electrode was inserted subcutaneously beneath the scalp and 2.5% Gonak hypromellose ophthalmic demulcent solution was applied to the cornea. A contact lens electrode was positioned on the cornea of each eye to detect electrical signals from retina. Excessive Gonak solution was removed from the eyes with tissue paper and the animals were allowed to stabilize in darkness for 15 minutes before beginning the recordings. Test flashes from a 530 nm LED, ranging from 2.5 × 10^−5^ cds m^−2^ to the 25 cds m^−2^ limit, were used to elicit photoresponses from each eye, and white Xenon flashes were used to produce saturated photoresponses. Sufficient time was allowed between individual test flashes to allow full recovery of the retina and avoid gradual response run-down due to light adaptation. For dark adaptation testing, a bright green (505 nm) LED light was applied to both eyes for 30 seconds to photobleach an estimated 90% of the visual pigment. The recovery of the ERG responses was monitored at fixed post-bleach time points within 2 hours after the bleach. The maximal response amplitude, r_max_, was recorded at the brightest light intensity, and S_f_ was estimated as the ratio of dim flash response amplitude and the corresponding flash intensity in the linear range of the intensity-response curve, about 20% to 30% of the maximum. The post-bleach maximal amplitude (r_max_) and sensitivity (S_f_) were normalized to their dark adapted pre-bleach level, r^DA^_max_ and S_f_^DA^, respectively.

### RNA-seq

RNA-seq was performed in two biological replicates per condition (objective day vs. subjective day), each consisting of four eyes. Eyes were harvested and rapidly dissected in the dark. The anterior portion of the eye including the lens was removed, and the remaining tissue (posterior sclera, choroid, RPE, and retina) was rinsed in cold sterile HBSS with calcium and magnesium (Gibco) and stored in TRIzol (Invitrogen) at −80 °C. For extraction, the tissue was homogenized with a pestle and then passaged through a needle. Total RNA was extracted and purified using the RNeasy Mini Kit (Qiagen) with on-column DNaseI digestion (Qiagen). Integrity of total RNA was verified on a Agilent 2100 Bioanalyzer. Poly-A selection and synthesis of the cDNA library for sequencing was conducted as described^43^. The four samples were indexed and sequenced on a single lane of a HiSeq 2500 sequencer (1 × 50 bp). To analyze the sequencing data, raw reads were demultiplexed and aligned to *Mus musculus* Ensembl release 72^44^ with Tophat v2.0.9^45^, and Bowtie2 v2.1.0^46^ HTseq^47^ was used to estimate gene abundance, and differential expression analysis was conducted with EdgeR^48^. See Supplementary Table S1 and Gene Expression Omnibus (GEO) accession number GSE68470 for raw and processed RNA-seq data.

### Quantitative RT-PCR (qRT-PCR)

Quantitative RT-PCR was performed in five biological replicates per condition (objective day vs. subjective day), each consisting of two eyes at 5–6 weeks old. Eyes were harvested and dissected and total RNA was prepared as described above for RNA-seq. Complementary DNA (cDNA) was prepared as previously described^24^, and qRT-PCR was conducted with SYBR-Green (Applied Biosystems). *Rpe65* transcript levels were normalized to *Gapdh* transcript levels. *Gapdh* primers^49^ and *Rpe65* primers^50^ were previously published. Three technical replicate PCR reactions were performed for each biological replicate. The ΔCt values from biological replicates were averaged and the SEM across biological replicates was calculated. The ΔCt values were used to calculate statistical significance with a two-tailed Student’s t-test.

### Statistics

Unless noted otherwise, all data is presented as mean ± SEM. Two tailed unpaired Student’s t-test was used to examine the significance of difference between two sample groups. Statistical significance was reported when *p* < 0.05.

## Additional Information

**How to cite this article**: Xue, Y. *et al*. Circadian and light-driven regulation of rod dark adaptation. *Sci. Rep.* **5**, 17616; doi: 10.1038/srep17616 (2015).

## Supplementary Material

Supplementary Information

## Figures and Tables

**Table 1:** Dark-adapted scotopic *in vivo* ERG parameters of C3H/f^+/+^, C57BL/6 J and 129S2/Sv mice at subjective night, subjective day and objective day.

	**Subjective night**	**Subjective day**	**Objective day**
C3H/f+/+	(n = 15)	(n = 16)	(n = 7)
a-wave r^DA^_max_ (μV)	312 ± 21	291 ± 31 (NS)	338 ± 29 (NS)
a-wave S_f_^DA^ (μV m^2^ cds^−1^)	223 ± 30	267 ± 39 (NS)	296 ± 37 (NS)
C57BL/6 J	(n = 9)	(n = 10)	(n = 10)
a-wave r^DA^_max_ (μV)	598 ± 24	532 ± 18 (*)	700 ± 22 (**)
a-wave S_f_^DA^ (μV m^2^ cds^−1^)	751 ± 39	589 ± 15 (**)	856 ± 55 (**)
129S2/Sv	(n = 6)	(n = 8)	(n = 7)
a-wave r^DA^_max_ (μV)	277 ± 29	269 ± 36 (NS)	301 ± 28 (NS)
a-wave S_f_^DA^ (μV m^2^ cds^−1^)	249 ± 46	208 ± 37 (NS)	255 ± 27 (NS)

r^DA^_max_ is the maximal amplitude of a-wave. S_f_^DA^ is the a-wave sensitivity. NS: *p *> 0.05, *p < 0.05, **p < 0.01, subjective day: tested compared to subjective night group, objective day: tested compared to the subjective day group.
