# Supplementary material for: Circadian and light-driven regulation of rod dark adaptation
Source: Sci Rep. 2015 Dec 2;5:17616. doi: 10.1038/srep17616 (PMC4667277; doi:10.1038/srep17616)
Supplement: Supplementary Information [file srep17616-s1.pdf]

Circadian and light-driven regulation of rod dark adaptation

Yunlu Xue[1A,C], Susan Q. Shen[1B,C], Joseph C. Corbo[1B], and Vladimir J. Kefalov[1A]

[A]Department of Ophthalmology & Visual Sciences, [B]Department of Pathology & Immunology, [C]Graduate Program in Division of Biological & Biomedical Sciences, [1]Washington University School of Medicine, St. Louis, Missouri 63110, USA

Corresponding author: Vladimir J. Kefalov, Ph.D, Department of Ophthalmology and Visual Sciences, Washington University in Saint Louis, 660 South Euclid Avenue, Saint Louis, Missouri 63110; Tel: (314) 362-4376; Fax: (314) 747-9046; E-mail: kefalov@wustl.edu

Supplementary Table S1. RNA-seq gene-level differential expression analysis. The table includes all 22,864 genes analyzed for differential expression using EdgeR. The p-value, FDR-adjusted p-value (i.e., q-value), expression estimates for each replicate in units of counts per million (CPM) after fitting and normalization, and $\log_2$ fold change of subjective day vs. objective day are shown. Genes are ranked by significance, with the top 1,460 genes (yellow highlighting) corresponding to significantly differentially expressed genes at FDR = 0.05. Data have been deposited in Gene Expression Omnibus (GEO) accession number GSE68470.

Ensembl_gene_ID	Gene_ID	Description	p-value
ENSMUSG00000038418	Egr1	early growth response 1 [Source:MGI Symbol;Acc:N	1.15E-51
ENSMUSG00000064356	mt-Atp8	mitochondrially encoded ATP synthase 8 [Source:M	7.16E-43
ENSMUSG00000064357	mt-Atp6	mitochondrially encoded ATP synthase 6 [Source:M	8.39E-38
ENSMUSG00000064358	mt-Co3	mitochondrially encoded cytochrome c oxidase III [	1.01E-30
ENSMUSG00000091337	Eid1	EP300 interacting inhibitor of differentiation 1 [Sou	3.19E-25
ENSMUSG00000064345	mt-Nd2	mitochondrially encoded NADH dehydrogenase 2 [S	5.48E-25
ENSMUSG00000037428	Vgf	VGF nerve growth factor inducible [Source:MGI Syn	4.26E-23
ENSMUSG00000035133	Arhgap5	Rho GTPase activating protein 5 [Source:MGI Symb	5.37E-23
ENSMUSG00000031197	Vbp1	von Hippel-Lindau binding protein 1 [Source:MGI Sy	2.85E-22
ENSMUSG00000030208	Emp1	epithelial membrane protein 1 [Source:MGI Symbo	4.17E-21
ENSMUSG00000014956	Ppp1cb	protein phosphatase 1, catalytic subunit, beta isofo	4.47E-20
ENSMUSG00000064339	mt-Rnr2	mitochondrially encoded 16S rRNA [Source:MGI Sy	6.06E-20
ENSMUSG00000064368	mt-Nd6	mitochondrially encoded NADH dehydrogenase 6 [S	1.4E-19
ENSMUSG00000064363	mt-Nd4	mitochondrially encoded NADH dehydrogenase 4 [S	1.69E-19
ENSMUSG00000071266	Zfp946	zinc finger protein 946 [Source:MGI Symbol;Acc:MG	1.45E-18
ENSMUSG00000001891	Ugp2	UDP-glucose pyrophosphorylase 2 [Source:MGI Syn	2.91E-18
ENSMUSG00000079339	Gm14446	predicted gene 14446 [Source:MGI Symbol;Acc:MG	7.04E-18
ENSMUSG00000064349	mt-Tc	mitochondrially encoded tRNA cysteine [Source:MG	1.32E-17
ENSMUSG00000031246	Sh3bgrl	SH3-binding domain glutamic acid-rich protein like	1.81E-17
ENSMUSG00000079620	Muc4	mucin 4 [Source:MGI Symbol;Acc:MGI:2153525]	2.8E-17
ENSMUSG00000039883	Lrrc17	leucine rich repeat containing 17 [Source:MGI Syml	5.05E-17
ENSMUSG00000024227	2610034M16Rik	RIKEN cDNA 2610034M16 gene [Source:MGI Symbo	1.49E-16
ENSMUSG00000053347	Zfp943	zinc finger prtoein 943 [Source:MGI Symbol;Acc:MG	2.48E-16
ENSMUSG00000069892	9930111J21Rik2	RIKEN cDNA 9930111J21 gene 2 [Source:MGI Symb	2.62E-16
ENSMUSG00000025498	Irf7	interferon regulatory factor 7 [Source:MGI Symbol;	4.07E-16
ENSMUSG00000023039	Krt7	keratin 7 [Source:MGI Symbol;Acc:MGI:96704]	5.34E-16
ENSMUSG00000059668	Krt4	keratin 4 [Source:MGI Symbol;Acc:MGI:96701]	8.74E-16
ENSMUSG00000064337	mt-Rnr1	mitochondrially encoded 12S rRNA [Source:MGI Sy	2.15E-15
ENSMUSG00000064369	mt-Te	mitochondrially encoded tRNA glutamic acid [Sourc	2.19E-15
ENSMUSG00000045545	Krt14	keratin 14 [Source:MGI Symbol;Acc:MGI:96688]	4.19E-15
ENSMUSG00000096433	Gm4944	predicted gene 4944 [Source:MGI Symbol;Acc:MGI:	4.63E-15
ENSMUSG00000062014	Gmfb	glia maturation factor, beta [Source:MGI Symbol;Ac	6.2E-15
ENSMUSG00000073427	Gm4924	predicted gene 4924 [Source:MGI Symbol;Acc:MGI:	6.71E-15
ENSMUSG00000042717	Ppp1r3a	protein phosphatase 1, regulatory (inhibitor) subun	7.29E-15
ENSMUSG00000026761	Orc4	origin recognition complex, subunit 4 [Source:MGI	1.03E-14
ENSMUSG00000027375	Mal	myelin and lymphocyte protein, T cell differentiatio	1.26E-14
ENSMUSG00000058093	AA987161	expressed sequence AA987161 [Source:MGI Symbo	1.58E-14
ENSMUSG00000071267	Zfp942	zinc finger protein 942 [Source:MGI Symbol;Acc:MG	1.76E-14
ENSMUSG00000044501	Zfp758	zinc finger protein 758 [Source:MGI Symbol;Acc:MG	1.87E-14
ENSMUSG00000024290	Rock1	Rho-associated coiled-coil containing protein kinase	1.89E-14
ENSMUSG00000025068	Gsto1	glutathione S-transferase omega 1 [Source:MGI Syr	2.24E-14
ENSMUSG00000064360	mt-Nd3	mitochondrially encoded NADH dehydrogenase 3 [S	4.04E-14
ENSMUSG00000091183	Gm5141	predicted gene 5141 [Source:MGI Symbol;Acc:MGI:	4.33E-14
ENSMUSG00000061331	Gm17132	predicted gene 17132 [Source:MGI Symbol;Acc:MG	5.16E-14
ENSMUSG00000044041	Krt13	keratin 13 [Source:MGI Symbol;Acc:MGI:101925]	5.75E-14
ENSMUSG00000002617	Zfp40	zinc finger protein 40 [Source:MGI Symbol;Acc:MGI	5.83E-14
ENSMUSG00000036192	Rorb	RAR-related orphan receptor beta [Source:MGI Syn	7.35E-14
ENSMUSG00000028719	Cmpk1	cytidine monophosphate (UMP-CMP) kinase 1 [Sou	7.42E-14

ENSMUSG00000067006	Serpinb5	serine (or cysteine) peptidase inhibitor, clade B, me	9.41E-14
ENSMUSG00000037475	Thoc2	THO complex 2 [Source:MGI Symbol;Acc:MGI:2442	1.08E-13
ENSMUSG00000024072	Yipf4	Yip1 domain family, member 4 [Source:MGI Symbo	1.78E-13
ENSMUSG00000060988	Galnt13	UDP-N-acetyl-alpha-D-galactosamine:polypeptide N	1.92E-13
ENSMUSG00000031089	Slc6a14	solute carrier family 6 (neurotransmitter transporte	2.08E-13
ENSMUSG00000055373	Fut9	fucosyltransferase 9 [Source:MGI Symbol;Acc:MGI:	2.09E-13
ENSMUSG00000019851	Perp	PERP, TP53 apoptosis effector [Source:MGI Symbol	2.58E-13
ENSMUSG00000024614	Tmx3	thioredoxin-related transmembrane protein 3 [Sou	2.96E-13
ENSMUSG00000063253	Scoc	short coiled-coil protein [Source:MGI Symbol;Acc:M	4.3E-13
ENSMUSG00000033972	Zfp944	zinc finger protein 944 [Source:MGI Symbol;Acc:MG	5.98E-13
ENSMUSG00000079658	Tceb1	transcription elongation factor B (SIII), polypeptide	7.25E-13
ENSMUSG00000021098	4930447C04Rik	RIKEN cDNA 4930447C04 gene [Source:MGI Symbo	8.94E-13
ENSMUSG00000022674	Ube2v2	ubiquitin-conjugating enzyme E2 variant 2 [Source:	9.8E-13
ENSMUSG00000078578	Ube2d3	ubiquitin-conjugating enzyme E2D 3 [Source:MGI S	1.03E-12
ENSMUSG00000059142	Zfp945	zinc finger protein 945 [Source:MGI Symbol;Acc:MG	1.17E-12
ENSMUSG00000047141	Zfp654	zinc finger protein 654 [Source:MGI Symbol;Acc:MG	1.48E-12
ENSMUSG00000032328	Tmem30a	transmembrane protein 30A [Source:MGI Symbol;A	1.74E-12
ENSMUSG00000063383	Zfp947	zinc finger protein 947 [Source:MGI Symbol;Acc:MG	1.81E-12
ENSMUSG00000027009	Itga4	integrin alpha 4 [Source:MGI Symbol;Acc:MGI:9660	1.95E-12
ENSMUSG00000019906	Lin7a	lin-7 homolog A (C. elegans) [Source:MGI Symbol;A	2.1E-12
ENSMUSG00000031333	Abcb7	ATP-binding cassette, sub-family B (MDR/TAP), mer	2.36E-12
ENSMUSG00000019787	Trdn	triadin [Source:MGI Symbol;Acc:MGI:1924007]	2.46E-12
ENSMUSG00000064354	mt-Co2	mitochondrially encoded cytochrome c oxidase II [S	2.58E-12
ENSMUSG00000059898	Dsc3	desmocollin 3 [Source:MGI Symbol;Acc:MGI:11949	2.68E-12
ENSMUSG00000020911	Krt19	keratin 19 [Source:MGI Symbol;Acc:MGI:96693]	3.49E-12
ENSMUSG00000071561	BC100530	cDNA sequence BC100530 [Source:MGI Symbol;Acc	3.66E-12
ENSMUSG00000025900	Rp1	retinitis pigmentosa 1 (human) [Source:MGI Symbo	3.94E-12
ENSMUSG00000027499	Pkia	protein kinase inhibitor, alpha [Source:MGI Symbol	4.83E-12
ENSMUSG00000020794	Ube2g1	ubiquitin-conjugating enzyme E2G 1 [Source:MGI S	5.01E-12
ENSMUSG00000025666	Tmem47	transmembrane protein 47 [Source:MGI Symbol;Ac	5.12E-12
ENSMUSG00000097551	Gm7976	predicted gene 7976 [Source:MGI Symbol;Acc:MGI:	6.48E-12
ENSMUSG00000094472	Gm21897	predicted gene, 21897 [Source:MGI Symbol;Acc:MG	7.62E-12
ENSMUSG00000001833	7-Sep	septin 7 [Source:MGI Symbol;Acc:MGI:1335094]	9.51E-12
ENSMUSG00000078503	Gm13225	predicted gene 13225 [Source:MGI Symbol;Acc:MG	9.96E-12
ENSMUSG00000021510	A530054K11Rik	RIKEN cDNA A530054K11 gene [Source:MGI Symbo	1.15E-11
ENSMUSG00000073062	Zxdb	zinc finger, X-linked, duplicated B [Source:MGI Sym	1.26E-11
ENSMUSG00000058331	Zfp85-rs1	zinc finger protein 85, related sequence 1 [Source:N	1.42E-11
ENSMUSG00000004771	Rab11a	RAB11a, member RAS oncogene family [Source:MG	1.46E-11
ENSMUSG00000056145	AI504432	expressed sequence AI504432 [Source:MGI Symbol	1.68E-11
ENSMUSG00000078546	2210404O09Rik	RIKEN cDNA 2210404O09 gene [Source:MGI Symbo	1.82E-11
ENSMUSG00000034724	Cnot6l	CCR4-NOT transcription complex, subunit 6-like [So	1.89E-11
ENSMUSG00000037112	Sik2	salt inducible kinase 2 [Source:MGI Symbol;Acc:MG	2.15E-11
ENSMUSG00000025321	Itgb8	integrin beta 8 [Source:MGI Symbol;Acc:MGI:1338	2.56E-11
ENSMUSG00000071748	Gm14698	predicted gene 14698 [Source:MGI Symbol;Acc:MG	2.62E-11
ENSMUSG00000055301	Adh7	alcohol dehydrogenase 7 (class IV), mu or sigma po	2.67E-11
ENSMUSG00000064341	mt-Nd1	mitochondrially encoded NADH dehydrogenase 1 [S	2.68E-11
ENSMUSG00000033508	Asprv1	aspartic peptidase, retroviral-like 1 [Source:MGI Sy	3.21E-11
ENSMUSG00000049436	Upk1b	uroplakin 1B [Source:MGI Symbol;Acc:MGI:98912]	3.27E-11
ENSMUSG00000073131	Vma21	VMA21 vacuolar H+-ATPase homolog (S. cerevisiae	3.66E-11

ENSMUSG00000049164	Zfp518a	zinc finger protein 518A [Source:MGI Symbol;Acc:M	3.8E-11
ENSMUSG00000067194	Eif1ax	eukaryotic translation initiation factor 1A, X-linked	4.21E-11
ENSMUSG00000048040	Arxes2	adipocyte-related X-chromosome expressed seque	4.8E-11
ENSMUSG00000046311	Zfp62	zinc finger protein 62 [Source:MGI Symbol;Acc:MGI	5.38E-11
ENSMUSG00000027206	Cops2	COP9 (constitutive photomorphogenic) homolog, s	5.38E-11
ENSMUSG00000024331	Dsc2	desmocollin 2 [Source:MGI Symbol;Acc:MGI:10322	5.39E-11
ENSMUSG00000097375	6720427I07Rik	RIKEN cDNA 6720427I07 gene [Source:MGI Symbol	5.47E-11
ENSMUSG00000039221	Rpl22l1	ribosomal protein L22 like 1 [Source:MGI Symbol;A	6.14E-11
ENSMUSG00000026775	Yme1l1	YME1-like 1 (S. cerevisiae) [Source:MGI Symbol;Acc	6.68E-11
ENSMUSG00000063130	Calml3	calmodulin-like 3 [Source:MGI Symbol;Acc:MGI:191	6.94E-11
ENSMUSG00000035597	Prpf39	PRP39 pre-mRNA processing factor 39 homolog (ye	7.07E-11
ENSMUSG00000062931	Zfp938	zinc finger protein 938 [Source:MGI Symbol;Acc:MG	7.18E-11
ENSMUSG00000027351	Spred1	sprouty protein with EVH-1 domain 1, related sequ	8.78E-11
ENSMUSG00000018907	Alox12e	arachidonate lipoxygenase, epidermal [Source:MGI	8.96E-11
ENSMUSG00000025939	Ube2w	ubiquitin-conjugating enzyme E2W (putative) [Sour	9.2E-11
ENSMUSG00000023892	Zfp51	zinc finger protein 51 [Source:MGI Symbol;Acc:MGI	1.06E-10
ENSMUSG00000020311	Erlec1	endoplasmic reticulum lectin 1 [Source:MGI Symbo	1.07E-10
ENSMUSG00000013663	Pten	phosphatase and tensin homolog [Source:MGI Sym	1.16E-10
ENSMUSG00000056260	Lrif1	ligand dependent nuclear receptor interacting fact	1.19E-10
ENSMUSG00000066798	Zbtb6	zinc finger and BTB domain containing 6 [Source:M	1.26E-10
ENSMUSG00000051412	Vamp7	vesicle-associated membrane protein 7 [Source:MG	1.32E-10
ENSMUSG00000020290	Xpo1	exportin 1, CRM1 homolog (yeast) [Source:MGI Syn	1.36E-10
ENSMUSG00000059897	Zfp930	zinc finger protein 930 [Source:MGI Symbol;Acc:MG	1.38E-10
ENSMUSG00000075700	Selt	selenoprotein T [Source:MGI Symbol;Acc:MGI:1916	1.4E-10
ENSMUSG00000072762	4930522L14Rik	RIKEN cDNA 4930522L14 gene [Source:MGI Symbo	1.73E-10
ENSMUSG00000090641	Zfp712	zinc finger protein 712 [Source:MGI Symbol;Acc:MG	1.81E-10
ENSMUSG00000023041	Krt6b	keratin 6B [Source:MGI Symbol;Acc:MGI:1333768]	2.38E-10
ENSMUSG00000020189	Osbpl8	oxysterol binding protein-like 8 [Source:MGI Symbo	2.48E-10
ENSMUSG00000032293	Ireb2	iron responsive element binding protein 2 [Source:	2.56E-10
ENSMUSG00000020608	Smc6	structural maintenance of chromosomes 6 [Source:	2.58E-10
ENSMUSG00000028676	Srsf10	serine/arginine-rich splicing factor 10 [Source:MGI	2.59E-10
ENSMUSG00000012350	Ehf	ets homologous factor [Source:MGI Symbol;Acc:MG	2.7E-10
ENSMUSG00000091618	H60c	histocompatibility 60c [Source:MGI Symbol;Acc:MG	2.97E-10
ENSMUSG00000069793	Slfn9	schlafen 9 [Source:MGI Symbol;Acc:MGI:2445121]	3.3E-10
ENSMUSG00000072704	2700089E24Rik	RIKEN cDNA 2700089E24 gene [Source:MGI Symbo	3.46E-10
ENSMUSG00000062929	Cfl2	cofilin 2, muscle [Source:MGI Symbol;Acc:MGI:101	3.79E-10
ENSMUSG00000021377	Dek	DEK oncogene (DNA binding) [Source:MGI Symbol;A	3.86E-10
ENSMUSG00000031284	Pak3	p21 protein (Cdc42/Rac)-activated kinase 3 [Source	4.09E-10
ENSMUSG00000042595	Fam199x	family with sequence similarity 199, X-linked [Sourc	4.18E-10
ENSMUSG00000031517	Gpm6a	glycoprotein m6a [Source:MGI Symbol;Acc:MGI:10	4.36E-10
ENSMUSG00000056383	AI987944	expressed sequence AI987944 [Source:MGI Symbol	4.82E-10
ENSMUSG00000054146	Krt15	keratin 15 [Source:MGI Symbol;Acc:MGI:96689]	5.1E-10
ENSMUSG00000021390	Ogn	osteoglycin [Source:MGI Symbol;Acc:MGI:109278]	5.38E-10
ENSMUSG00000061527	Krt5	keratin 5 [Source:MGI Symbol;Acc:MGI:96702]	5.49E-10
ENSMUSG00000033953	Ppp3r1	protein phosphatase 3, regulatory subunit B, alpha	5.6E-10
ENSMUSG00000079834	Tmlhe	trimethyllysine hydroxylase, epsilon [Source:MGI Sy	5.8E-10
ENSMUSG00000031176	Dynlt3	dynein light chain Tctex-type 3 [Source:MGI Symbo	6.46E-10
ENSMUSG00000063663	Brwd3	bromodomain and WD repeat domain containing 3	6.63E-10
ENSMUSG00000067928	Zfp760	zinc finger protein 760 [Source:MGI Symbol;Acc:MG	7.24E-10

ENSMUSG00000068457	Uty	ubiquitously transcribed tetratricopeptide repeat g	7.36E-10
ENSMUSG00000044594	Serpinb3a	serine (or cysteine) peptidase inhibitor, clade B (ova	7.49E-10
ENSMUSG00000020463	Smek2	SMEK homolog 2, suppressor of mek1 (Dictyosteliu	8.19E-10
ENSMUSG00000020954	Strn3	striatin, calmodulin binding protein 3 [Source:MGI S	9.63E-10
ENSMUSG00000017485	Top2b	topoisomerase (DNA) II beta [Source:MGI Symbol;A	1.12E-09
ENSMUSG00000017724	Etv4	ets variant gene 4 (E1A enhancer binding protein, E	1.16E-09
ENSMUSG00000032745	Gpbp1	GC-rich promoter binding protein 1 [Source:MGI Sy	1.18E-09
ENSMUSG00000028391	Wdr31	WD repeat domain 31 [Source:MGI Symbol;Acc:MG	1.21E-09
ENSMUSG00000005802	Slc30a4	solute carrier family 30 (zinc transporter), member	1.39E-09
ENSMUSG00000035236	Scai	suppressor of cancer cell invasion [Source:MGI Sym	1.48E-09
ENSMUSG00000038174	Fam126b	family with sequence similarity 126, member B [Sou	1.49E-09
ENSMUSG00000005547	Cyp2a5	cytochrome P450, family 2, subfamily a, polypeptid	1.52E-09
ENSMUSG00000028212	Ccne2	cyclin E2 [Source:MGI Symbol;Acc:MGI:1329034]	1.56E-09
ENSMUSG00000058624	Gda	guanine deaminase [Source:MGI Symbol;Acc:MGI:9	1.65E-09
ENSMUSG00000027630	Tbl1xr1	transducin (beta)-like 1X-linked receptor 1 [Source:	1.67E-09
ENSMUSG00000066613	Zfp932	zinc finger protein 932 [Source:MGI Symbol;Acc:MG	1.78E-09
ENSMUSG00000031502	Col4a1	collagen, type IV, alpha 1 [Source:MGI Symbol;Acc:	1.81E-09
ENSMUSG00000015733	Capza2	capping protein (actin filament) muscle Z-line, alpha	1.85E-09
ENSMUSG00000027770	Dhx36	DEAH (Asp-Glu-Ala-His) box polypeptide 36 [Source	1.91E-09
ENSMUSG00000031878	Nae1	NEDD8 activating enzyme E1 subunit 1 [Source:MG	2.21E-09
ENSMUSG00000035694	Caps2	calcyphosphine 2 [Source:MGI Symbol;Acc:MGI:244	2.25E-09
ENSMUSG00000042436	Mfap4	microfibrillar-associated protein 4 [Source:MGI Sym	2.44E-09
ENSMUSG00000025979	Mob4	MOB family member 4, phocein [Source:MGI Symb	2.48E-09
ENSMUSG00000021693	Kif2a	kinesin family member 2A [Source:MGI Symbol;Acc	2.73E-09
ENSMUSG00000069919	Hba-a1	hemoglobin alpha, adult chain 1 [Source:MGI Symb	2.91E-09
ENSMUSG00000022523	Fgf12	fibroblast growth factor 12 [Source:MGI Symbol;Ac	3.42E-09
ENSMUSG00000000120	Ngfr	nerve growth factor receptor (TNFR superfamily, m	3.7E-09
ENSMUSG00000055228	Zfp935	zinc finger protein 935 [Source:MGI Symbol;Acc:MG	3.94E-09
ENSMUSG00000022698	Naa50	N(alpha)-acetyltransferase 50, NatE catalytic subun	4.03E-09
ENSMUSG00000037400	Atp11b	ATPase, class VI, type 11B [Source:MGI Symbol;Acc	4.06E-09
ENSMUSG00000031601	Cnot7	CCR4-NOT transcription complex, subunit 7 [Source	4.06E-09
ENSMUSG00000086607	4930511M06Rik	RIKEN cDNA 4930511M06 gene [Source:MGI Symbo	4.11E-09
ENSMUSG00000028180	Zranb2	zinc finger, RAN-binding domain containing 2 [Sour	4.2E-09
ENSMUSG00000025907	Rb1cc1	RB1-inducible coiled-coil 1 [Source:MGI Symbol;Acc	4.3E-09
ENSMUSG00000032496	Ltf	lactotransferrin [Source:MGI Symbol;Acc:MGI:9683	4.69E-09
ENSMUSG00000041935	AW549877	expressed sequence AW549877 [Source:MGI Symb	4.72E-09
ENSMUSG00000049421	Zfp260	zinc finger protein 260 [Source:MGI Symbol;Acc:MG	4.76E-09
ENSMUSG00000041771	Slc24a4	solute carrier family 24 (sodium/potassium/calcium	4.83E-09
ENSMUSG00000064115	Cadm2	cell adhesion molecule 2 [Source:MGI Symbol;Acc:M	5.11E-09
ENSMUSG00000097352	C920009B18Rik	RIKEN cDNA C920009B18 gene [Source:MGI Symbo	5.19E-09
ENSMUSG00000024479	Mal2	mal, T cell differentiation protein 2 [Source:MGI Sy	5.42E-09
ENSMUSG00000089709	Gm16262	predicted gene 16262 [Source:MGI Symbol;Acc:MG	5.45E-09
ENSMUSG00000030729	Pgm2l1	phosphoglucomutase 2-like 1 [Source:MGI Symbol;	5.81E-09
ENSMUSG00000052155	Acvr2a	activin receptor IIA [Source:MGI Symbol;Acc:MGI:1	5.88E-09
ENSMUSG00000097263	Gm26804	predicted gene, 26804 [Source:MGI Symbol;Acc:MG	5.94E-09
ENSMUSG00000062101	Zfp119b	zinc finger protein 119b [Source:MGI Symbol;Acc:M	6.22E-09
ENSMUSG00000037236	Matr3	matrin 3 [Source:MGI Symbol;Acc:MGI:1298379]	6.57E-09
ENSMUSG00000034349	Smc4	structural maintenance of chromosomes 4 [Source:	6.78E-09
ENSMUSG00000076432	Ywhaq	tyrosine 3-monooxygenase/tryptophan 5-monooxy	6.89E-09

ENSMUSG00000028222	Calb1	calbindin 1 [Source:MGI Symbol;Acc:MGI:88248]	6.97E-09
ENSMUSG00000030629	Zfand6	zinc finger, AN1-type domain 6 [Source:MGI Symbo	7.06E-09
ENSMUSG00000041293	Gpr110	G protein-coupled receptor 110 [Source:MGI Symb	7.13E-09
ENSMUSG00000090000	Ier3ip1	immediate early response 3 interacting protein 1 [S	7.23E-09
ENSMUSG00000031367	Ap1s2	adaptor-related protein complex 1, sigma 2 subunit	7.37E-09
ENSMUSG00000078435	AU041133	expressed sequence AU041133 [Source:MGI Symbo	7.41E-09
ENSMUSG00000026021	Sumo1	SMT3 suppressor of mif two 3 homolog 1 (yeast) [S	7.98E-09
ENSMUSG00000089828	Gm16300	predicted gene 16300 [Source:MGI Symbol;Acc:MG	8.36E-09
ENSMUSG00000019878	Hsf2	heat shock factor 2 [Source:MGI Symbol;Acc:MGI:9	8.38E-09
ENSMUSG00000064351	mt-Co1	mitochondrially encoded cytochrome c oxidase I [S	8.56E-09
ENSMUSG00000030759	Far1	fatty acyl CoA reductase 1 [Source:MGI Symbol;Acc	8.78E-09
ENSMUSG00000022105	Rb1	retinoblastoma 1 [Source:MGI Symbol;Acc:MGI:978	9.04E-09
ENSMUSG00000089788	Gm16586	predicted gene 16586 [Source:MGI Symbol;Acc:MG	9.12E-09
ENSMUSG00000026977	7-Mar	membrane-associated ring finger (C3HC4) 7 [Source	9.29E-09
ENSMUSG00000021549	Rasa1	RAS p21 protein activator 1 [Source:MGI Symbol;Ac	9.36E-09
ENSMUSG00000025529	Zfp711	zinc finger protein 711 [Source:MGI Symbol;Acc:MG	9.6E-09
ENSMUSG00000096696	Zfp960	zinc finger protein 960 [Source:MGI Symbol;Acc:MG	9.64E-09
ENSMUSG00000073639	Rab18	RAB18, member RAS oncogene family [Source:MGI	9.8E-09
ENSMUSG00000057396	Zfp759	zinc finger protein 759 [Source:MGI Symbol;Acc:MG	9.9E-09
ENSMUSG00000028557	Rnf11	ring finger protein 11 [Source:MGI Symbol;Acc:MGI	1.02E-08
ENSMUSG00000027677	Ttc14	tetratricopeptide repeat domain 14 [Source:MGI Sy	1.02E-08
ENSMUSG00000062949	Atp11c	ATPase, class VI, type 11C [Source:MGI Symbol;Acc	1.02E-08
ENSMUSG00000020062	Slc5a8	solute carrier family 5 (iodide transporter), membe	1.07E-08
ENSMUSG00000097340	Gm26617	predicted gene, 26617 [Source:MGI Symbol;Acc:MG	1.1E-08
ENSMUSG00000071362	Gm10330	predicted gene 10330 [Source:MGI Symbol;Acc:MG	1.1E-08
ENSMUSG00000086262	A930031H19Rik	RIKEN cDNA A930031H19 gene [Source:MGI Symbo	1.14E-08
ENSMUSG00000078861	Zfp931	zinc finger protein 931 [Source:MGI Symbol;Acc:MG	1.15E-08
ENSMUSG00000024293	Esco1	establishment of cohesion 1 homolog 1 (S. cerevisia	1.19E-08
ENSMUSG00000032013	Trim29	tripartite motif-containing 29 [Source:MGI Symbol;	1.25E-08
ENSMUSG00000033054	Npat	nuclear protein in the AT region [Source:MGI Symb	1.3E-08
ENSMUSG00000027365	Trpm7	transient receptor potential cation channel, subfam	1.33E-08
ENSMUSG00000056579	Tug1	taurine upregulated gene 1 [Source:MGI Symbol;Ac	1.34E-08
ENSMUSG00000057835	Zfp119a	zinc finger protein 119a [Source:MGI Symbol;Acc:M	1.41E-08
ENSMUSG00000078851	Hist3h2a	histone cluster 3, H2a [Source:MGI Symbol;Acc:MG	1.45E-08
ENSMUSG00000047879	Usp14	ubiquitin specific peptidase 14 [Source:MGI Symbo	1.46E-08
ENSMUSG00000090935	Synj2bp	synaptojanin 2 binding protein [Source:MGI Symbo	1.47E-08
ENSMUSG00000059839	Zfp874b	zinc finger protein 874b [Source:MGI Symbol;Acc:M	1.5E-08
ENSMUSG00000027708	Dcun1d1	DCN1, defective in cullin neddylation 1, domain con	1.6E-08
ENSMUSG00000022797	Tfrc	transferrin receptor [Source:MGI Symbol;Acc:MGI:9	1.63E-08
ENSMUSG00000025626	Phf6	PHD finger protein 6 [Source:MGI Symbol;Acc:MGI:	1.76E-08
ENSMUSG00000024276	Zfp397	zinc finger protein 397 [Source:MGI Symbol;Acc:MG	1.78E-08
ENSMUSG00000031201	Brcc3	BRCA1/BRCA2-containing complex, subunit 3 [Sour	1.9E-08
ENSMUSG00000021520	Uqcrb	ubiquinol-cytochrome c reductase binding protein [	2.03E-08
ENSMUSG00000030760	Acer3	alkaline ceramidase 3 [Source:MGI Symbol;Acc:MG	2.04E-08
ENSMUSG00000092400	Gm20469	predicted gene 20469 [Source:MGI Symbol;Acc:MG	2.08E-08
ENSMUSG00000020650	Bcap29	B cell receptor associated protein 29 [Source:MGI S	2.2E-08
ENSMUSG00000085334	Gm12940	predicted gene 12940 [Source:MGI Symbol;Acc:MG	2.28E-08
ENSMUSG00000051285	Pcmtd1	protein-L-isoaspartate (D-aspartate) O-methyltrans	2.31E-08
ENSMUSG00000020687	Cdc27	cell division cycle 27 [Source:MGI Symbol;Acc:MGI:	2.45E-08

ENSMUSG00000035868	3110052M02Rik	RIKEN cDNA 3110052M02 gene [Source:MGI Symb	2.58E-08
ENSMUSG00000000355	Mcts1	malignant T cell amplified sequence 1 [Source:MGI	2.67E-08
ENSMUSG00000017291	Taok1	TAO kinase 1 [Source:MGI Symbol;Acc:MGI:191449	2.69E-08
ENSMUSG00000059423	Zfp933	zinc finger protein 933 [Source:MGI Symbol;Acc:M	2.76E-08
ENSMUSG00000012422	Tmem167	transmembrane protein 167 [Source:MGI Symbol;A	2.8E-08
ENSMUSG00000021448	Shc3	src homology 2 domain-containing transforming pr	2.83E-08
ENSMUSG00000074968	Ano3	anoctamin 3 [Source:MGI Symbol;Acc:MGI:361366	2.89E-08
ENSMUSG00000060149	BC002059	cDNA sequence BC002059 [Source:MGI Symbol;Acc	3.08E-08
ENSMUSG00000048280	Zfp738	zinc finger protein 738 [Source:MGI Symbol;Acc:M	3.12E-08
ENSMUSG00000068130	Zfp442	zinc finger protein 442 [Source:MGI Symbol;Acc:M	3.12E-08
ENSMUSG00000054889	Dsp	desmoplakin [Source:MGI Symbol;Acc:MGI:109611	3.25E-08
ENSMUSG00000028522	Mier1	mesoderm induction early response 1 homolog (Xe	3.32E-08
ENSMUSG00000085328	Gm17131	predicted gene 17131 [Source:MGI Symbol;Acc:MG	3.45E-08
ENSMUSG00000027550	Lrrcc1	leucine rich repeat and coiled-coil domain containi	3.65E-08
ENSMUSG00000026434	Nucks1	nuclear casein kinase and cyclin-dependent kinase	3.66E-08
ENSMUSG00000028211	Trp53inp1	transformation related protein 53 inducible nuclea	3.73E-08
ENSMUSG00000040118	Cacna2d1	calcium channel, voltage-dependent, alpha2/delta	3.73E-08
ENSMUSG00000069755	Zfp125	zinc finger protein 125 [Source:MGI Symbol;Acc:M	3.85E-08
ENSMUSG00000060989	Gm11847	predicted gene 11847 [Source:MGI Symbol;Acc:MG	4.14E-08
ENSMUSG00000042784	Muc1	mucin 1, transmembrane [Source:MGI Symbol;Acc:	4.17E-08
ENSMUSG00000031229	Atrx	alpha thalassemia/mental retardation syndrome X-	4.18E-08
ENSMUSG00000028020	Glrb	glycine receptor, beta subunit [Source:MGI Symbol	4.19E-08
ENSMUSG00000058748	Zfp958	zinc finger protein 958 [Source:MGI Symbol;Acc:M	4.27E-08
ENSMUSG00000020362	Cnot6	CCR4-NOT transcription complex, subunit 6 [Source	4.31E-08
ENSMUSG00000025326	Ube3a	ubiquitin protein ligase E3A [Source:MGI Symbol;A	4.45E-08
ENSMUSG00000032407	U2surp	U2 snRNP-associated SURP domain containing [Sou	4.47E-08
ENSMUSG00000021945	Zmym2	zinc finger, MYM-type 2 [Source:MGI Symbol;Acc:M	4.58E-08
ENSMUSG00000046230	Vps13a	vacuolar protein sorting 13A (yeast) [Source:MGI S	4.8E-08
ENSMUSG00000046351	Zfp322a	zinc finger protein 322A [Source:MGI Symbol;Acc:M	5.11E-08
ENSMUSG00000064344	mt-Tm	mitochondrially encoded tRNA methionine [Source	5.13E-08
ENSMUSG00000066880	Zfp617	zinc finger protein 617 [Source:MGI Symbol;Acc:M	5.32E-08
ENSMUSG00000078597	Cyp4a12b	cytochrome P450, family 4, subfamily a, polypeptid	5.37E-08
ENSMUSG00000073664	Nbeal1	neurobeachin like 1 [Source:MGI Symbol;Acc:MGI:	5.6E-08
ENSMUSG00000024317	Rnf138	ring finger protein 138 [Source:MGI Symbol;Acc:M	5.83E-08
ENSMUSG00000056019	Zfp709	zinc finger protein 709 [Source:MGI Symbol;Acc:M	5.9E-08
ENSMUSG00000064367	mt-Nd5	mitochondrially encoded NADH dehydrogenase 5 [	5.93E-08
ENSMUSG00000025903	Lypla1	lysophospholipase 1 [Source:MGI Symbol;Acc:MGI:	6.02E-08
ENSMUSG00000017418	Arl5b	ADP-ribosylation factor-like 5B [Source:MGI Symbo	6.06E-08
ENSMUSG00000027981	Rnpc3	RNA-binding region (RNP1, RRM) containing 3 [Sou	6.16E-08
ENSMUSG00000066000	2610305D13Rik	RIKEN cDNA 2610305D13 gene [Source:MGI Symbo	6.18E-08
ENSMUSG00000018076	Med13l	mediator complex subunit 13-like [Source:MGI Sym	6.34E-08
ENSMUSG00000018199	Trove2	TROVE domain family, member 2 [Source:MGI Sym	6.4E-08
ENSMUSG00000096795	Zfp433	RIKEN cDNA 1700123A16 gene [Source:MGI Symbo	6.46E-08
ENSMUSG00000023737	1700082M22Rik	RIKEN cDNA 1700082M22 gene [Source:MGI Symb	6.56E-08
ENSMUSG00000057842	Zfp595	zinc finger protein 595 [Source:MGI Symbol;Acc:M	6.61E-08
ENSMUSG00000006369	Fbln1	fibulin 1 [Source:MGI Symbol;Acc:MGI:95487]	7.21E-08
ENSMUSG00000021091	Serpina3n	serine (or cysteine) peptidase inhibitor, clade A, me	7.65E-08
ENSMUSG00000078899	Gm4631	predicted gene 4631 [Source:MGI Symbol;Acc:MGI	7.99E-08
ENSMUSG00000074165	Zfp788	zinc finger protein 788 [Source:MGI Symbol;Acc:M	8.47E-08

ENSMUSG00000039967	Zfp292	zinc finger protein 292 [Source:MGI Symbol;Acc:MG	9.02E-08
ENSMUSG00000043929	Klhl15	kelch-like 15 [Source:MGI Symbol;Acc:MGI:192340	9.99E-08
ENSMUSG00000014905	Dnajb9	DnaJ (Hsp40) homolog, subfamily B, member 9 [Sou	1.01E-07
ENSMUSG00000036469	1-Mar	membrane-associated ring finger (C3HC4) 1 [Source	1.04E-07
ENSMUSG00000063273	Naa15	N(alpha)-acetyltransferase 15, NatA auxiliary subur	1.05E-07
ENSMUSG00000095403	Gm21092	predicted gene, 21092 [Source:MGI Symbol;Acc:MG	1.07E-07
ENSMUSG00000021250	Fos	FBJ osteosarcoma oncogene [Source:MGI Symbol;A	1.1E-07
ENSMUSG00000091509	Gm17066	predicted gene 17066 [Source:MGI Symbol;Acc:MG	1.15E-07
ENSMUSG00000064370	mt-Cytb	mitochondrially encoded cytochrome b [Source:MG	1.16E-07
ENSMUSG00000030704	Rab6a	RAB6A, member RAS oncogene family [Source:MGI	1.16E-07
ENSMUSG00000079084	Ccdc82	coiled-coil domain containing 82 [Source:MGI Symb	1.18E-07
ENSMUSG00000047735	Samd9l	sterile alpha motif domain containing 9-like [Source	1.21E-07
ENSMUSG00000000838	Fmr1	fragile X mental retardation syndrome 1 [Source:M	1.21E-07
ENSMUSG00000007682	Dio2	deiodinase, iodothyronine, type II [Source:MGI Sym	1.22E-07
ENSMUSG00000071379	Hpcal1	hippocalcin-like 1 [Source:MGI Symbol;Acc:MGI:18	1.25E-07
ENSMUSG00000025916	Ppp1r42	protein phosphatase 1, regulatory subunit 42 [Sour	1.28E-07
ENSMUSG00000024659	Anxa1	annexin A1 [Source:MGI Symbol;Acc:MGI:96819]	1.32E-07
ENSMUSG00000025451	Paip1	polyadenylate binding protein-interacting protein 1	1.36E-07
ENSMUSG00000026824	Kcnj3	potassium inwardly-rectifying channel, subfamily J,	1.42E-07
ENSMUSG00000028382	Ptbp3	polypyrimidine tract binding protein 3 [Source:MGI	1.48E-07
ENSMUSG00000019857	Asf1a	ASF1 anti-silencing function 1 homolog A (S. cerevis	1.51E-07
ENSMUSG00000030105	Arl8b	ADP-ribosylation factor-like 8B [Source:MGI Symbo	1.52E-07
ENSMUSG00000020805	Slc13a5	solute carrier family 13 (sodium-dependent citrate	1.52E-07
ENSMUSG00000052681	Rap1b	RAS related protein 1b [Source:MGI Symbol;Acc:MG	1.53E-07
ENSMUSG00000025245	Lztfl1	leucine zipper transcription factor-like 1 [Source:M	1.59E-07
ENSMUSG00000094483	Purb	purine rich element binding protein B [Source:MGI	1.62E-07
ENSMUSG00000020561	Twistnb	TWIST neighbor [Source:MGI Symbol;Acc:MGI:1062	1.65E-07
ENSMUSG00000025862	Stag2	stromal antigen 2 [Source:MGI Symbol;Acc:MGI:10	1.75E-07
ENSMUSG00000019966	Kitl	kit ligand [Source:MGI Symbol;Acc:MGI:96974]	1.76E-07
ENSMUSG00000074272	Ceacam1	carcinoembryonic antigen-related cell adhesion mo	1.9E-07
ENSMUSG00000028435	Aqp3	aquaporin 3 [Source:MGI Symbol;Acc:MGI:1333777	1.91E-07
ENSMUSG00000034317	Trim59	tripartite motif-containing 59 [Source:MGI Symbol;	1.93E-07
ENSMUSG00000033543	Gtf2a2	general transcription factor II A, 2 [Source:MGI Sym	1.94E-07
ENSMUSG00000026361	Cdc73	cell division cycle 73, Paf1/RNA polymerase II comp	1.95E-07
ENSMUSG00000019102	Aldh3a1	aldehyde dehydrogenase family 3, subfamily A1 [So	1.97E-07
ENSMUSG00000061136	Prpf40a	PRP40 pre-mRNA processing factor 40 homolog A (	2.03E-07
ENSMUSG00000053600	Zfp472	zinc finger protein 472 [Source:MGI Symbol;Acc:MG	2.07E-07
ENSMUSG00000025860	Xiap	X-linked inhibitor of apoptosis [Source:MGI Symbol	2.14E-07
ENSMUSG00000021929	Kpna3	karyopherin (importin) alpha 3 [Source:MGI Symbo	2.16E-07
ENSMUSG00000091896	Ube2d2a	ubiquitin-conjugating enzyme E2D 2A [Source:MGI	2.24E-07
ENSMUSG00000034607	Pof1b	premature ovarian failure 1B [Source:MGI Symbol;A	2.24E-07
ENSMUSG00000058291	Zfp68	zinc finger protein 68 [Source:MGI Symbol;Acc:MGI	2.25E-07
ENSMUSG00000095193	Gm20939	predicted gene, 20939 [Source:MGI Symbol;Acc:MG	2.26E-07
ENSMUSG00000053774	Ubxn7	UBX domain protein 7 [Source:MGI Symbol;Acc:MG	2.31E-07
ENSMUSG00000086429	Gt(ROSA)26Sor	gene trap ROSA 26, Philippe Soriano [Source:MGI S	2.33E-07
ENSMUSG00000028035	Dnajb4	DnaJ (Hsp40) homolog, subfamily B, member 4 [Sou	2.34E-07
ENSMUSG00000035615	Frmpd1	FERM and PDZ domain containing 1 [Source:MGI Sy	2.35E-07
ENSMUSG00000069184	Zfp72	zinc finger protein 72 [Source:MGI Symbol;Acc:MGI	2.39E-07
ENSMUSG00000021816	Ppp3cb	protein phosphatase 3, catalytic subunit, beta isofo	2.43E-07

ENSMUSG00000082588	Gm15443	predicted gene 15443 [Source:MGI Symbol;Acc:MG	2.53E-07
ENSMUSG00000031010	Usp9x	ubiquitin specific peptidase 9, X chromosome [Sour	2.56E-07
ENSMUSG00000064061	Dzip3	DAZ interacting protein 3, zinc finger [Source:MGI S	2.59E-07
ENSMUSG00000021548	Ccnh	cyclin H [Source:MGI Symbol;Acc:MGI:1913921]	2.71E-07
ENSMUSG00000041846	Smek1	SMEK homolog 1, suppressor of mek1 (Dictyosteliu	2.72E-07
ENSMUSG00000063145	Bbs5	Bardet-Biedl syndrome 5 (human) [Source:MGI Sym	2.85E-07
ENSMUSG00000056537	Rlim	ring finger protein, LIM domain interacting [Source	3.27E-07
ENSMUSG00000074867	Zfp808	zinc finger protein 80 [Source:MGI Symbol;Acc:MGI	3.33E-07
ENSMUSG00000096808	AC132444.6		3.33E-07
ENSMUSG00000085180	AI838599	expressed sequence AI838599 [Source:MGI Symbol	3.37E-07
ENSMUSG00000033184	Tmed7	transmembrane emp24 protein transport domain c	3.43E-07
ENSMUSG00000060510	Zfp266	zinc finger protein 266 [Source:MGI Symbol;Acc:MC	3.46E-07
ENSMUSG00000032253	Phip	pleckstrin homology domain interacting protein [Sc	3.54E-07
ENSMUSG00000015882	Lcorl	ligand dependent nuclear receptor corepressor-like	3.6E-07
ENSMUSG00000060301	2610008E11Rik	RIKEN cDNA 2610008E11 gene [Source:MGI Symbo	3.7E-07
ENSMUSG00000026696	Vamp4	vesicle-associated membrane protein 4 [Source:MC	3.78E-07
ENSMUSG00000024900	Cpt1a	carnitine palmitoyltransferase 1a, liver [Source:MG	3.79E-07
ENSMUSG00000091764	Zfp964	zinc finger protein 964 [Source:MGI Symbol;Acc:MC	3.97E-07
ENSMUSG00000064366	mt-Tl2	mitochondrially encoded tRNA leucine 2 [Source:M	3.97E-07
ENSMUSG00000057561	Eif1a	eukaryotic translation initiation factor 1A [Source:N	4.21E-07
ENSMUSG00000021072	Tmx1	thioredoxin-related transmembrane protein 1 [Sou	4.22E-07
ENSMUSG00000033849	B3galt2	UDP-Gal:betaGlcNAc beta 1,3-galactosyltransferase	4.41E-07
ENSMUSG00000039478	Micu3	mitochondrial calcium uptake family, member 3 [So	4.45E-07
ENSMUSG00000058354	Krt6a	keratin 6A [Source:MGI Symbol;Acc:MGI:1100845]	4.53E-07
ENSMUSG00000022894	Adamts5	a disintegrin-like and metallopeptidase (reprolysin	4.63E-07
ENSMUSG00000079038	D130040H23Rik	RIKEN cDNA D130040H23 gene [Source:MGI Symbo	4.68E-07
ENSMUSG00000050808	Muc15	mucin 15 [Source:MGI Symbol;Acc:MGI:2442110]	4.69E-07
ENSMUSG00000021832	Psmc6	proteasome (prosome, macropain) 26S subunit, AT	4.74E-07
ENSMUSG00000042670	Immp1l	IMP1 inner mitochondrial membrane peptidase-like	4.76E-07
ENSMUSG00000051341	Zfp52	zinc finger protein 52 [Source:MGI Symbol;Acc:MGI	4.87E-07
ENSMUSG00000095432	Zfp748	zinc finger protein 748 [Source:MGI Symbol;Acc:MC	4.91E-07
ENSMUSG00000027782	Kpna4	karyopherin (importin) alpha 4 [Source:MGI Symbo	4.94E-07
ENSMUSG00000020386	Sar1b	SAR1 gene homolog B (S. cerevisiae) [Source:MGI S	5.04E-07
ENSMUSG00000059811	Atl2	atlastin GTPase 2 [Source:MGI Symbol;Acc:MGI:192	5.04E-07
ENSMUSG00000000058	Cav2	caveolin 2 [Source:MGI Symbol;Acc:MGI:107571]	5.11E-07
ENSMUSG00000032925	Itgbl1	integrin, beta-like 1 [Source:MGI Symbol;Acc:MGI:2	5.29E-07
ENSMUSG00000003051	Elf3	E74-like factor 3 [Source:MGI Symbol;Acc:MGI:110	5.41E-07
ENSMUSG00000029338	Antxr2	anthrax toxin receptor 2 [Source:MGI Symbol;Acc:N	5.62E-07
ENSMUSG00000025531	Chm	choroidermia [Source:MGI Symbol;Acc:MGI:89297	6.01E-07
ENSMUSG00000029816	Gpnmb	glycoprotein (transmembrane) nmb [Source:MGI Sy	6.07E-07
ENSMUSG00000067942	Zfp160	zinc finger protein 160 [Source:MGI Symbol;Acc:MC	6.12E-07
ENSMUSG00000046785	Epm2aip1	EPM2A (laforin) interacting protein 1 [Source:MGI S	6.17E-07
ENSMUSG00000021775	Nr1d2	nuclear receptor subfamily 1, group D, member 2 [S	6.41E-07
ENSMUSG00000030413	Pglyrp1	peptidoglycan recognition protein 1 [Source:MGI Sy	6.77E-07
ENSMUSG00000021282	Eif5	eukaryotic translation initiation factor 5 [Source:MC	6.8E-07
ENSMUSG00000073197	5730507C01Rik	RIKEN cDNA 5730507C01 gene [Source:MGI Symbo	6.93E-07
ENSMUSG00000022800	Fyttd1	forty-two-three domain containing 1 [Source:MGI S	6.96E-07
ENSMUSG00000051469	Zfp191	zinc finger protein 191 [Source:MGI Symbol;Acc:MC	7.02E-07
ENSMUSG00000021831	Ero1l	ERO1-like (S. cerevisiae) [Source:MGI Symbol;Acc:N	7.17E-07

ENSMUSG00000064364	mt-Th	mitochondrially encoded tRNA histidine [Source:MG	7.2E-07
ENSMUSG00000024480	Ap3s1	adaptor-related protein complex 3, sigma 1 subunit	7.42E-07
ENSMUSG00000006262	Mob1b	MOB kinase activator 1B [Source:MGI Symbol;Acc:M	7.89E-07
ENSMUSG00000031696	Vps35	vacuolar protein sorting 35 [Source:MGI Symbol;Ac	7.91E-07
ENSMUSG00000021712	Trim23	tripartite motif-containing 23 [Source:MGI Symbol;	8.08E-07
ENSMUSG00000040123	Zmym5	zinc finger, MYM-type 5 [Source:MGI Symbol;Acc:M	8.31E-07
ENSMUSG00000029823	Luc7l2	LUC7-like 2 (S. cerevisiae) [Source:MGI Symbol;Acc:	8.4E-07
ENSMUSG00000035293	G2e3	G2/M-phase specific E3 ubiquitin ligase [Source:MG	8.67E-07
ENSMUSG00000028716	Pdzk1ip1	PDZK1 interacting protein 1 [Source:MGI Symbol;Ac	8.9E-07
ENSMUSG00000032740	Ccdc88a	coiled coil domain containing 88A [Source:MGI Sym	9.08E-07
ENSMUSG00000031309	Rps6ka3	ribosomal protein S6 kinase polypeptide 3 [Source:	9.31E-07
ENSMUSG00000004364	Cul3	cullin 3 [Source:MGI Symbol;Acc:MGI:1347360]	9.33E-07
ENSMUSG00000036676	Tmtc3	transmembrane and tetratricopeptide repeat conta	9.5E-07
ENSMUSG00000031503	Col4a2	collagen, type IV, alpha 2 [Source:MGI Symbol;Acc:	9.69E-07
ENSMUSG00000016308	Ube2a	ubiquitin-conjugating enzyme E2A [Source:MGI Sym	9.98E-07
ENSMUSG00000027823	Gmps	guanine monophosphate synthetase [Source:MGI S	1.04E-06
ENSMUSG00000052144	Ppp4r2	protein phosphatase 4, regulatory subunit 2 [Sourc	1.06E-06
ENSMUSG00000044067	Gpr22	G protein-coupled receptor 22 [Source:MGI Symbo	1.07E-06
ENSMUSG00000028883	Sema3a	sema domain, immunoglobulin domain (Ig), short b	1.08E-06
ENSMUSG00000025757	Hspa4l	heat shock protein 4 like [Source:MGI Symbol;Acc:M	1.08E-06
ENSMUSG00000026730	Pter	phosphotriesterase related [Source:MGI Symbol;Ac	1.09E-06
ENSMUSG00000048047	Zbtb33	zinc finger and BTB domain containing 33 [Source:M	1.11E-06
ENSMUSG00000016756	Cmah	cytidine monophospho-N-acetylneuraminic acid hy	1.12E-06
ENSMUSG00000056870	Gulp1	GULP, engulfment adaptor PTB domain containing	1.13E-06
ENSMUSG00000018846	Pank3	pantothenate kinase 3 [Source:MGI Symbol;Acc:MG	1.14E-06
ENSMUSG00000062373	Tmem65	transmembrane protein 65 [Source:MGI Symbol;Ac	1.15E-06
ENSMUSG00000079065	BC005561	cDNA sequence BC005561 [Source:MGI Symbol;Acc	1.17E-06
ENSMUSG00000071708	Sms	spermine synthase [Source:MGI Symbol;Acc:MGI:1	1.2E-06
ENSMUSG00000085438	1700020I14Rik	RIKEN cDNA 1700020I14 gene [Source:MGI Symbol	1.25E-06
ENSMUSG00000017548	Suz12	suppressor of zeste 12 homolog (Drosophila) [Sourc	1.27E-06
ENSMUSG00000064352	mt-Ts1	mitochondrially encoded tRNA serine 1 [Source:MG	1.3E-06
ENSMUSG00000025894	Aasdhppt	aminoadipate-semialdehyde dehydrogenase-phosp	1.37E-06
ENSMUSG00000064365	mt-Ts2	mitochondrially encoded tRNA serine 2 [Source:MG	1.38E-06
ENSMUSG00000021270	Hsp90aa1	heat shock protein 90, alpha (cytosolic), class A me	1.43E-06
ENSMUSG00000057924	Gm10033	predicted gene 10033 [Source:MGI Symbol;Acc:MG	1.47E-06
ENSMUSG00000020431	Adcy1	adenylate cyclase 1 [Source:MGI Symbol;Acc:MGI:9	1.48E-06
ENSMUSG00000021669	Col4a3bp	collagen, type IV, alpha 3 (Goodpasture antigen) bi	1.54E-06
ENSMUSG00000019775	Rgs17	regulator of G-protein signaling 17 [Source:MGI Sym	1.56E-06
ENSMUSG00000016534	Lamp2	lysosomal-associated membrane protein 2 [Source:	1.57E-06
ENSMUSG00000032068	1600029D21Rik	RIKEN cDNA 1600029D21 gene [Source:MGI Symbo	1.58E-06
ENSMUSG00000026004	Kansl1l	KAT8 regulatory NSL complex subunit 1-like [Source	1.59E-06
ENSMUSG00000035898	Uba6	ubiquitin-like modifier activating enzyme 6 [Source	1.69E-06
ENSMUSG00000028134	Ptbp2	polypyrimidine tract binding protein 2 [Source:MGI	1.88E-06
ENSMUSG00000060002	Chpt1	choline phosphotransferase 1 [Source:MGI Symbol;	1.9E-06
ENSMUSG00000052974	Cyp2f2	cytochrome P450, family 2, subfamily f, polypeptide	1.91E-06
ENSMUSG00000024597	Slc12a2	solute carrier family 12, member 2 [Source:MGI Sym	1.98E-06
ENSMUSG00000006403	Adamts4	a disintegrin-like and metallopeptidase (reprolysin t	2E-06
ENSMUSG00000021893	Capn7	calpain 7 [Source:MGI Symbol;Acc:MGI:1338030]	2.05E-06
ENSMUSG00000053702	Nebl	nebulette [Source:MGI Symbol;Acc:MGI:1921353]	2.07E-06

ENSMUSG00000025104	Hdgfrp3	hepatoma-derived growth factor, related protein 3	2.13E-06
ENSMUSG00000039470	Zdhhc2	zinc finger, DHHC domain containing 2 [Source:MG	2.15E-06
ENSMUSG00000039841	Zfp800	zinc finger protein 800 [Source:MGI Symbol;Acc:M	2.25E-06
ENSMUSG00000037416	Dmxl1	Dmx-like 1 [Source:MGI Symbol;Acc:MGI:2443926]	2.27E-06
ENSMUSG00000049122	Frmd3	FERM domain containing 3 [Source:MGI Symbol;Ac	2.32E-06
ENSMUSG00000095362	Gm14325	predicted gene 14325 [Source:MGI Symbol;Acc:MG	2.33E-06
ENSMUSG00000047501	Cldn4	claudin 4 [Source:MGI Symbol;Acc:MGI:1313314]	2.34E-06
ENSMUSG00000040651	D14Abb1e	DNA segment, Chr 14, Abbott 1 expressed [Source:	2.41E-06
ENSMUSG00000078867	Gm14418	predicted gene 14418 [Source:MGI Symbol;Acc:MG	2.43E-06
ENSMUSG00000052676	Zmat1	zinc finger, matrin type 1 [Source:MGI Symbol;Acc:	2.44E-06
ENSMUSG00000032091	Tmprss4	transmembrane protease, serine 4 [Source:MGI Sy	2.47E-06
ENSMUSG00000044150	A830080D01Rik	RIKEN cDNA A830080D01 gene [Source:MGI Symbo	2.54E-06
ENSMUSG00000031202	Rab39b	RAB39B, member RAS oncogene family [Source:MG	2.55E-06
ENSMUSG00000031095	Cul4b	cullin 4B [Source:MGI Symbol;Acc:MGI:1919834]	2.57E-06
ENSMUSG00000067430	Zfp763	zinc finger protein 763 [Source:MGI Symbol;Acc:M	2.67E-06
ENSMUSG00000040297	Suco	SUN domain containing ossification factor [Source:	2.77E-06
ENSMUSG00000078566	Bnip3	BCL2/adenovirus E1B interacting protein 3 [Source:	2.77E-06
ENSMUSG00000072761	Gm6712	predicted gene 6712 [Source:MGI Symbol;Acc:MGI	2.82E-06
ENSMUSG00000071281	Zfp71-rs1	zinc finger protein 71, related sequence [Source:M	2.85E-06
ENSMUSG00000021877	Arf4	ADP-ribosylation factor 4 [Source:MGI Symbol;Acc:	2.85E-06
ENSMUSG00000068134	Zfp120	zinc finger protein 120 [Source:MGI Symbol;Acc:M	2.85E-06
ENSMUSG00000034282	Evpl	envoplakin [Source:MGI Symbol;Acc:MGI:107507]	2.85E-06
ENSMUSG00000044807	Zfp354c	zinc finger protein 354C [Source:MGI Symbol;Acc:M	2.86E-06
ENSMUSG00000027900	Dram2	DNA-damage regulated autophagy modulator 2 [So	2.86E-06
ENSMUSG00000007812	Zfp655	zinc finger protein 655 [Source:MGI Symbol;Acc:M	2.87E-06
ENSMUSG00000069045	Ddx3y	DEAD (Asp-Glu-Ala-Asp) box polypeptide 3, Y-linke	2.9E-06
ENSMUSG00000020436	Gabrg2	gamma-aminobutyric acid (GABA) A receptor, subu	2.92E-06
ENSMUSG00000069662	Marcks	myristoylated alanine rich protein kinase C substra	2.96E-06
ENSMUSG00000020063	Sirt1	sirtuin 1 [Source:MGI Symbol;Acc:MGI:2135607]	3E-06
ENSMUSG00000026158	Ogfrl1	opioid growth factor receptor-like 1 [Source:MGI S	3.03E-06
ENSMUSG00000031715	Smarca5	SWI/SNF related, matrix associated, actin depende	3.11E-06
ENSMUSG00000031327	Chic1	cysteine-rich hydrophobic domain 1 [Source:MGI S	3.12E-06
ENSMUSG00000043991	Pura	purine rich element binding protein A [Source:MGI	3.13E-06
ENSMUSG00000005371	Fbxo11	F-box protein 11 [Source:MGI Symbol;Acc:MGI:214	3.15E-06
ENSMUSG00000098239	Pclo	piccolo (presynaptic cytomatrix protein) [Source:M	3.16E-06
ENSMUSG00000026721	Rabgap1l	RAB GTPase activating protein 1-like [Source:MGI S	3.17E-06
ENSMUSG00000027808	Serp1	stress-associated endoplasmic reticulum protein 1	3.18E-06
ENSMUSG00000047714	Ppp1r2	protein phosphatase 1, regulatory (inhibitor) subu	3.29E-06
ENSMUSG00000026365	Cfh	complement component factor h [Source:MGI Sym	3.35E-06
ENSMUSG00000001260	Gabrg1	gamma-aminobutyric acid (GABA) A receptor, subu	3.5E-06
ENSMUSG00000010311	Optc	opticin [Source:MGI Symbol;Acc:MGI:2151113]	3.51E-06
ENSMUSG00000050592	Fam78a	family with sequence similarity 78, member A [Sou	3.69E-06
ENSMUSG00000021709	Erbb2ip	Erbb2 interacting protein [Source:MGI Symbol;Acc:	3.88E-06
ENSMUSG00000022205	Sub1	SUB1 homolog (S. cerevisiae) [Source:MGI Symbol;	4.07E-06
ENSMUSG00000069237	C78339	expressed sequence C78339 [Source:MGI Symbol;A	4.19E-06
ENSMUSG00000024259	Slc25a46	solute carrier family 25, member 46 [Source:MGI S	4.2E-06
ENSMUSG00000019989	Enpp3	ectonucleotide pyrophosphatase/phosphodiestera	4.23E-06
ENSMUSG00000042460	C1galt1	core 1 synthase, glycoprotein-N-acetylgalactosami	4.4E-06
ENSMUSG00000019777	Hdac2	histone deacetylase 2 [Source:MGI Symbol;Acc:MG	4.42E-06

ENSMUSG00000069049	Eif2s3y	eukaryotic translation initiation factor 2, subunit 3,	4.55E-06
ENSMUSG00000022533	Atp13a3	ATPase type 13A3 [Source:MGI Symbol;Acc:MGI:26	4.67E-06
ENSMUSG00000090015	Gm15446	predicted gene 15446 [Source:MGI Symbol;Acc:MG	4.7E-06
ENSMUSG00000047694	Yipf6	Yip1 domain family, member 6 [Source:MGI Symbo	4.77E-06
ENSMUSG00000053897	Slc39a8	solute carrier family 39 (metal ion transporter), me	4.86E-06
ENSMUSG00000031885	Cbfb	core binding factor beta [Source:MGI Symbol;Acc:M	4.9E-06
ENSMUSG00000025496	Drd4	dopamine receptor D4 [Source:MGI Symbol;Acc:MG	5.18E-06
ENSMUSG00000061273	Mmgt1	membrane magnesium transporter 1 [Source:MGI S	5.19E-06
ENSMUSG00000041225	Arhgap12	Rho GTPase activating protein 12 [Source:MGI Sym	5.33E-06
ENSMUSG00000028763	Hspg2	perlecan (heparan sulfate proteoglycan 2) [Source:	5.34E-06
ENSMUSG00000033533	Acsm1	acyl-CoA synthetase medium-chain family member	5.39E-06
ENSMUSG00000040855	Reps2	RALBP1 associated Eps domain containing protein 2	5.82E-06
ENSMUSG00000037722	Gnpnat1	glucosamine-phosphate N-acetyltransferase 1 [Sou	5.95E-06
ENSMUSG00000029322	Plac8	placenta-specific 8 [Source:MGI Symbol;Acc:MGI:2	6.08E-06
ENSMUSG00000048834	Vstm2a	V-set and transmembrane domain containing 2A [S	6.15E-06
ENSMUSG00000031939	Taf1d	TATA box binding protein (Tbp)-associated factor, R	6.16E-06
ENSMUSG00000024411	Aqp4	aquaporin 4 [Source:MGI Symbol;Acc:MGI:107387]	6.26E-06
ENSMUSG00000037071	Scd1	stearoyl-Coenzyme A desaturase 1 [Source:MGI Syr	6.29E-06
ENSMUSG00000036902	Neto2	neuropilin (NRP) and tolloid (TLL)-like 2 [Source:MG	6.39E-06
ENSMUSG00000014226	Cacybp	calcyclin binding protein [Source:MGI Symbol;Acc:M	6.44E-06
ENSMUSG00000027834	Serpini1	serine (or cysteine) peptidase inhibitor, clade I, me	6.48E-06
ENSMUSG00000034723	Tmx4	thioredoxin-related transmembrane protein 4 [Sou	6.54E-06
ENSMUSG00000036202	Rif1	Rap1 interacting factor 1 homolog (yeast) [Source:M	6.65E-06
ENSMUSG00000025981	Coq10b	coenzyme Q10 homolog B (S. cerevisiae) [Source:M	6.72E-06
ENSMUSG00000056055	Sag	S-antigen, retina and pineal gland (arrestin) [Source	6.74E-06
ENSMUSG00000097328	Tnfsf12	tumor necrosis factor (ligand) superfamily, membe	6.91E-06
ENSMUSG00000037239	Spred3	sprouty-related, EVH1 domain containing 3 [Source	7.15E-06
ENSMUSG00000049775	Tmsb4x	thymosin, beta 4, X chromosome [Source:MGI Sym	7.2E-06
ENSMUSG00000021187	Tc2n	tandem C2 domains, nuclear [Source:MGI Symbol;A	7.28E-06
ENSMUSG00000094441	Zfp955a	zinc finger protein 955A [Source:MGI Symbol;Acc:M	7.44E-06
ENSMUSG00000089375	Snord107	small nucleolar RNA, C/D box 107 [Source:MGI Sym	7.45E-06
ENSMUSG00000062511	4930512M02Rik	RIKEN cDNA 4930512M02 gene [Source:MGI Symbo	7.51E-06
ENSMUSG00000047586	Nccrp1	non-specific cytotoxic cell receptor protein 1 homo	7.53E-06
ENSMUSG00000071072	Ptges3	prostaglandin E synthase 3 (cytosolic) [Source:MGI	7.66E-06
ENSMUSG00000045690	Wdr89	WD repeat domain 89 [Source:MGI Symbol;Acc:MG	7.69E-06
ENSMUSG00000003198	Zfp959	zinc finger protein 959 [Source:MGI Symbol;Acc:MG	7.92E-06
ENSMUSG00000043903	Gm22	predicted gene 22 [Source:MGI Symbol;Acc:MGI:26	7.98E-06
ENSMUSG00000026064	Ptp4a1	protein tyrosine phosphatase 4a1 [Source:MGI Sym	8.14E-06
ENSMUSG00000027366	Sppl2a	signal peptide peptidase like 2A [Source:MGI Symb	8.22E-06
ENSMUSG00000019888	Mgat4c	mannosyl (alpha-1,3-)-glycoprotein beta-1,4-N-acet	8.25E-06
ENSMUSG00000034297	Med13	mediator complex subunit 13 [Source:MGI Symbol;	8.31E-06
ENSMUSG00000029571	Tmem106b	transmembrane protein 106B [Source:MGI Symbol;	8.49E-06
ENSMUSG00000020590	Snx13	sorting nexin 13 [Source:MGI Symbol;Acc:MGI:266	8.54E-06
ENSMUSG00000058883	Zfp708	zinc finger protein 708 [Source:MGI Symbol;Acc:MG	8.87E-06
ENSMUSG00000020932	Gfap	glial fibrillary acidic protein [Source:MGI Symbol;Ac	8.92E-06
ENSMUSG00000035840	Lysmd3	LysM, putative peptidoglycan-binding, domain cont	8.93E-06
ENSMUSG00000022788	Fgd4	FYVE, RhoGEF and PH domain containing 4 [Source	8.98E-06
ENSMUSG00000094030	Gm21833	predicted gene, 21833 [Source:MGI Symbol;Acc:MG	9.01E-06
ENSMUSG00000042444	Fam63b	family with sequence similarity 63, member B [Sou	9.03E-06

ENSMUSG00000026667	Uhmk1	U2AF homology motif (UHM) kinase 1 [Source:MGI	9.24E-06
ENSMUSG00000079083	Jrkl	jerky homolog-like (mouse) [Source:MGI Symbol;Ac	9.5E-06
ENSMUSG00000096463	Gm21750	predicted gene, 21750 [Source:MGI Symbol;Acc:MG	9.7E-06
ENSMUSG00000033319	Fem1c	fem-1 homolog c (C.elegans) [Source:MGI Symbol;A	9.7E-06
ENSMUSG00000038702	Dsel	dermatan sulfate epimerase-like [Source:MGI Symb	9.85E-06
ENSMUSG00000060126	Tpt1	tumor protein, translationally-controlled 1 [Source:	9.97E-06
ENSMUSG00000057329	Bcl2	B cell leukemia/lymphoma 2 [Source:MGI Symbol;A	1E-05
ENSMUSG00000097695	Gm26905	predicted gene, 26905 [Source:MGI Symbol;Acc:MG	1.02E-05
ENSMUSG00000043881	Kbtbd7	kelch repeat and BTB (POZ) domain containing 7 [S	1.03E-05
ENSMUSG00000030452	Nipa2	non imprinted in Prader-Willi/Angelman syndrome	1.04E-05
ENSMUSG00000033813	Tcea1	transcription elongation factor A (SII) 1 [Source:MG	1.04E-05
ENSMUSG00000030265	Kras	v-Ki-ras2 Kirsten rat sarcoma viral oncogene homol	1.05E-05
ENSMUSG00000042271	Nxt2	nuclear transport factor 2-like export factor 2 [Sour	1.05E-05
ENSMUSG00000045414	1190002N15Rik	RIKEN cDNA 1190002N15 gene [Source:MGI Symbo	1.06E-05
ENSMUSG00000042599	Jhdm1d	jumonji C domain-containing histone demethylase	1.06E-05
ENSMUSG00000021950	Anxa8	annexin A8 [Source:MGI Symbol;Acc:MGI:1201374]	1.07E-05
ENSMUSG00000078495	Gm13157	predicted gene 13157 [Source:MGI Symbol;Acc:MG	1.09E-05
ENSMUSG00000026331	Slco6c1	solute carrier organic anion transporter family, mer	1.12E-05
ENSMUSG00000069743	Zfp820	zinc finger protein 820 [Source:MGI Symbol;Acc:MG	1.12E-05
ENSMUSG00000026893	Gca	grancalcin [Source:MGI Symbol;Acc:MGI:1918521]	1.13E-05
ENSMUSG00000050334	C130071C03Rik	RIKEN cDNA C130071C03 gene [Source:MGI Symbo	1.14E-05
ENSMUSG00000036854	Hspb6	heat shock protein, alpha-crystallin-related, B6 [Sou	1.19E-05
ENSMUSG00000036093	Arl5a	ADP-ribosylation factor-like 5A [Source:MGI Symbo	1.19E-05
ENSMUSG00000048540	Nhlh2	nescient helix loop helix 2 [Source:MGI Symbol;Acc	1.21E-05
ENSMUSG00000056632	Dsg3	desmoglein 3 [Source:MGI Symbol;Acc:MGI:99499]	1.21E-05
ENSMUSG00000034159	2310007B03Rik	RIKEN cDNA 2310007B03 gene [Source:MGI Symbo	1.25E-05
ENSMUSG00000073940	Hbb-bt	hemoglobin, beta adult t chain [Source:MGI Symbo	1.25E-05
ENSMUSG00000050762	Prss27	protease, serine, 27 [Source:MGI Symbol;Acc:MGI:2	1.27E-05
ENSMUSG00000028188	Spata1	spermatogenesis associated 1 [Source:MGI Symbol	1.32E-05
ENSMUSG00000024750	Zfand5	zinc finger, AN1-type domain 5 [Source:MGI Symbo	1.32E-05
ENSMUSG00000024726	2410127L17Rik	RIKEN cDNA 2410127L17 gene [Source:MGI Symbo	1.33E-05
ENSMUSG00000015961	Adss	adenylosuccinate synthetase, non muscle [Source:M	1.37E-05
ENSMUSG00000040520	Manea	mannosidase, endo-alpha [Source:MGI Symbol;Acc	1.4E-05
ENSMUSG00000067017	Gm3608	predicted gene 3608 [Source:MGI Symbol;Acc:MGI:	1.42E-05
ENSMUSG00000025016	Tm9sf3	transmembrane 9 superfamily member 3 [Source:M	1.43E-05
ENSMUSG00000004698	Hdac9	histone deacetylase 9 [Source:MGI Symbol;Acc:MG	1.49E-05
ENSMUSG00000028174	Rpe65	retinal pigment epithelium 65 [Source:MGI Symbol;	1.5E-05
ENSMUSG00000026327	Serpinb11	serine (or cysteine) peptidase inhibitor, clade B (ova	1.52E-05
ENSMUSG00000066232	Ipo7	importin 7 [Source:MGI Symbol;Acc:MGI:2152414]	1.53E-05
ENSMUSG00000032307	Ube2q2	ubiquitin-conjugating enzyme E2Q (putative) 2 [Sou	1.53E-05
ENSMUSG00000022123	Scel	sciellin [Source:MGI Symbol;Acc:MGI:1891228]	1.55E-05
ENSMUSG00000024511	Rab27b	RAB27b, member RAS oncogene family [Source:MG	1.56E-05
ENSMUSG00000038301	Snx10	sorting nexin 10 [Source:MGI Symbol;Acc:MGI:1919	1.57E-05
ENSMUSG00000064372	mt-Tp	mitochondrially encoded tRNA proline [Source:MG	1.57E-05
ENSMUSG00000058446	Znrf2	zinc and ring finger 2 [Source:MGI Symbol;Acc:MGI	1.59E-05
ENSMUSG00000003418	St8sia6	ST8 alpha-N-acetyl-neuraminide alpha-2,8-sialyltra	1.6E-05
ENSMUSG00000079555	Haus3	HAUS augmin-like complex, subunit 3 [Source:MGI	1.6E-05
ENSMUSG00000060924	Csmd1	CUB and Sushi multiple domains 1 [Source:MGI Syn	1.61E-05
ENSMUSG00000022514	Il1rap	interleukin 1 receptor accessory protein [Source:M	1.62E-05

ENSMUSG00000027965	Olfm3	olfactomedin 3 [Source:MGI Symbol;Acc:MGI:2387	1.63E-05
ENSMUSG00000051397	Tacstd2	tumor-associated calcium signal transducer 2 [Sour	1.66E-05
ENSMUSG00000098206	A430106G13Rik	RIKEN cDNA A430106G13 gene [Source:MGI Symbo	1.7E-05
ENSMUSG00000074829	2010315B03Rik	RIKEN cDNA 2010315B03 gene [Source:MGI Symbo	1.71E-05
ENSMUSG00000078878	Gm14305	predicted gene 14305 [Source:MGI Symbol;Acc:MG	1.72E-05
ENSMUSG00000013523	Bcas1	breast carcinoma amplified sequence 1 [Source:MG	1.73E-05
ENSMUSG00000037720	Tmem33	transmembrane protein 33 [Source:MGI Symbol;Ac	1.73E-05
ENSMUSG00000019987	Arg1	arginase, liver [Source:MGI Symbol;Acc:MGI:88070	1.76E-05
ENSMUSG00000074909	Ranbp6	RAN binding protein 6 [Source:MGI Symbol;Acc:MG	1.77E-05
ENSMUSG00000039457	Ppl	periplakin [Source:MGI Symbol;Acc:MGI:1194898]	1.8E-05
ENSMUSG00000024052	Lpin2	lipin 2 [Source:MGI Symbol;Acc:MGI:1891341]	1.8E-05
ENSMUSG00000062908	Acadm	acyl-Coenzyme A dehydrogenase, medium chain [S	1.84E-05
ENSMUSG00000027253	Lrp4	low density lipoprotein receptor-related protein 4 [	1.86E-05
ENSMUSG00000078872	Gm14401	predicted gene 14401 [Source:MGI Symbol;Acc:MG	1.87E-05
ENSMUSG00000032423	Syncrip	synaptotagmin binding, cytoplasmic RNA interactin	1.9E-05
ENSMUSG00000026753	Ppp6c	protein phosphatase 6, catalytic subunit [Source:M	1.92E-05
ENSMUSG00000047281	Sfn	stratifin [Source:MGI Symbol;Acc:MGI:1891831]	1.95E-05
ENSMUSG00000025958	Creb1	cAMP responsive element binding protein 1 [Sourc	1.97E-05
ENSMUSG00000040321	Zfp770	zinc finger protein 770 [Source:MGI Symbol;Acc:MG	2.03E-05
ENSMUSG00000074735	Gm21994	predicted gene 21994 [Source:MGI Symbol;Acc:MG	2.08E-05
ENSMUSG00000083563	Gm13340	predicted gene 13340 [Source:MGI Symbol;Acc:MG	2.08E-05
ENSMUSG00000038535	Zfp280d	zinc finger protein 280D [Source:MGI Symbol;Acc:M	2.09E-05
ENSMUSG00000006010	BC003331	cDNA sequence BC003331 [Source:MGI Symbol;Acc	2.12E-05
ENSMUSG00000019920	Lims1	LIM and senescent cell antigen-like domains 1 [Sou	2.14E-05
ENSMUSG00000090110	Mtcp1	mature T cell proliferation 1 [Source:MGI Symbol;A	2.15E-05
ENSMUSG00000025171	Ubtd1	ubiquitin domain containing 1 [Source:MGI Symbol	2.15E-05
ENSMUSG00000089417	Gm22009	predicted gene, 22009 [Source:MGI Symbol;Acc:MG	2.16E-05
ENSMUSG00000027855	Sycp1	synaptonemal complex protein 1 [Source:MGI Sym	2.19E-05
ENSMUSG00000040010	Slc7a5	solute carrier family 7 (cationic amino acid transpo	2.23E-05
ENSMUSG00000045210	Vcpip1	valosin containing protein (p97)/p47 complex inter	2.27E-05
ENSMUSG00000034640	Tiparp	TCDD-inducible poly(ADP-ribose) polymerase [Sour	2.28E-05
ENSMUSG00000071172	Srsf3	serine/arginine-rich splicing factor 3 [Source:MGI S	2.29E-05
ENSMUSG00000031592	Pcm1	pericentriolar material 1 [Source:MGI Symbol;Acc:M	2.3E-05
ENSMUSG00000042133	Ppig	peptidyl-prolyl isomerase G (cyclophilin G) [Source:	2.3E-05
ENSMUSG00000030660	Pik3c2a	phosphatidylinositol 3-kinase, C2 domain containin	2.42E-05
ENSMUSG00000021936	Mapk8	mitogen-activated protein kinase 8 [Source:MGI Sy	2.46E-05
ENSMUSG00000028675	Pnrc2	proline-rich nuclear receptor coactivator 2 [Source:	2.56E-05
ENSMUSG00000028572	Hook1	hook homolog 1 (Drosophila) [Source:MGI Symbol;	2.57E-05
ENSMUSG00000066324	Impad1	inositol monophosphatase domain containing 1 [So	2.62E-05
ENSMUSG00000089940	Gm4117	predicted gene 4117 [Source:MGI Symbol;Acc:MGI:	2.65E-05
ENSMUSG00000075376	Rc3h2	ring finger and CCCH-type zinc finger domains 2 [So	2.65E-05
ENSMUSG00000019876	Pkib	protein kinase inhibitor beta, cAMP dependent, tes	2.68E-05
ENSMUSG00000022789	Dnm1l	dynamin 1-like [Source:MGI Symbol;Acc:MGI:19212	2.68E-05
ENSMUSG00000009628	Tex15	testis expressed gene 15 [Source:MGI Symbol;Acc:M	2.73E-05
ENSMUSG00000000568	Hnrnpd	heterogeneous nuclear ribonucleoprotein D [Sourc	2.81E-05
ENSMUSG00000031290	Lrch2	leucine-rich repeats and calponin homology (CH) do	2.89E-05
ENSMUSG00000023074	Mospd1	motile sperm domain containing 1 [Source:MGI Syr	2.92E-05
ENSMUSG00000018379	Srsf1	serine/arginine-rich splicing factor 1 [Source:MGI S	2.97E-05
ENSMUSG00000032238	Rora	RAR-related orphan receptor alpha [Source:MGI Sy	2.99E-05

ENSMUSG00000069206	Zfp874a	zinc finger protein 874a [Source:MGI Symbol;Acc:M	3.12E-05
ENSMUSG00000007617	Homer1	homer homolog 1 (Drosophila) [Source:MGI Symbo	3.13E-05
ENSMUSG00000007655	Cav1	caveolin 1, caveolae protein [Source:MGI Symbol;A	3.22E-05
ENSMUSG00000022338	Eny2	enhancer of yellow 2 homolog (Drosophila) [Source	3.26E-05
ENSMUSG00000030061	Uba3	ubiquitin-like modifier activating enzyme 3 [Source	3.3E-05
ENSMUSG00000026034	Clk1	CDC-like kinase 1 [Source:MGI Symbol;Acc:MGI:107	3.33E-05
ENSMUSG00000036353	P2ry12	purinergic receptor P2Y, G-protein coupled 12 [Sou	3.35E-05
ENSMUSG00000038323	1700066M21Rik	RIKEN cDNA 1700066M21 gene [Source:MGI Symb	3.39E-05
ENSMUSG00000020521	Rnft1	ring finger protein, transmembrane 1 [Source:MGI	3.42E-05
ENSMUSG00000037808	Fam76b	family with sequence similarity 76, member B [Sou	3.42E-05
ENSMUSG00000045573	Penk	preproenkephalin [Source:MGI Symbol;Acc:MGI:10	3.45E-05
ENSMUSG00000022255	Mtdh	metadherin [Source:MGI Symbol;Acc:MGI:1914404	3.45E-05
ENSMUSG00000020580	Rock2	Rho-associated coiled-coil containing protein kinas	3.47E-05
ENSMUSG00000090093	Gm14399	predicted gene 14399 [Source:MGI Symbol;Acc:MG	3.51E-05
ENSMUSG00000055480	Zfp458	zinc finger protein 458 [Source:MGI Symbol;Acc:M	3.57E-05
ENSMUSG00000057454	Lypd3	Ly6/Plaur domain containing 3 [Source:MGI Symbo	3.58E-05
ENSMUSG00000041633	Kctd12b	potassium channel tetramerisation domain contain	3.58E-05
ENSMUSG00000031285	Dcx	doublecortin [Source:MGI Symbol;Acc:MGI:127717	3.61E-05
ENSMUSG00000036282	Naa30	N(alpha)-acetyltransferase 30, NatC catalytic subun	3.66E-05
ENSMUSG00000038024	Dennd4c	DENN/MADD domain containing 4C [Source:MGI Sy	3.76E-05
ENSMUSG00000071533	Pcnp	PEST proteolytic signal containing nuclear protein [	3.78E-05
ENSMUSG00000067929	Gm10226	predicted gene 10226 [Source:MGI Symbol;Acc:MG	3.81E-05
ENSMUSG00000037640	Zfp60	zinc finger protein 60 [Source:MGI Symbol;Acc:MGI	3.93E-05
ENSMUSG00000070709	1700049G17Rik	RIKEN cDNA 1700049G17 gene [Source:MGI Symbo	3.93E-05
ENSMUSG00000056342	Usp34	ubiquitin specific peptidase 34 [Source:MGI Symbo	3.94E-05
ENSMUSG00000047496	Rnf152	ring finger protein 152 [Source:MGI Symbol;Acc:M	3.99E-05
ENSMUSG00000030016	Zfml	zinc finger, matrin-like [Source:MGI Symbol;Acc:M	4.03E-05
ENSMUSG00000036309	Skp1a	S-phase kinase-associated protein 1A [Source:MGI	4.03E-05
ENSMUSG00000078868	Gm14412	predicted gene 14412 [Source:MGI Symbol;Acc:MG	4.07E-05
ENSMUSG00000094707	A830019P07Rik	RIKEN cDNA A830019P07 gene [Source:MGI Symbo	4.07E-05
ENSMUSG00000024287	Thoc1	THO complex 1 [Source:MGI Symbol;Acc:MGI:1919	4.1E-05
ENSMUSG00000060739	Nsa2	NSA2 ribosome biogenesis homolog (S. cerevisiae)	4.11E-05
ENSMUSG00000028544	Slc5a9	solute carrier family 5 (sodium/glucose cotransport	4.15E-05
ENSMUSG00000074158	9830147E19Rik	RIKEN cDNA 9830147E19 gene [Source:MGI Symbo	4.27E-05
ENSMUSG00000021553	Slc28a3	solute carrier family 28 (sodium-coupled nucleoside	4.29E-05
ENSMUSG00000044408	Sptssa	serine palmitoyltransferase, small subunit A [Sourc	4.32E-05
ENSMUSG00000074519	Etohi1	ethanol induced 1 [Source:MGI Symbol;Acc:MGI:12	4.35E-05
ENSMUSG00000071083	Gm10311	predicted gene 10311 [Source:MGI Symbol;Acc:MG	4.36E-05
ENSMUSG00000025059	Gyk	glycerol kinase [Source:MGI Symbol;Acc:MGI:1065	4.38E-05
ENSMUSG00000071528	Usmg5	upregulated during skeletal muscle growth 5 [Sourc	4.49E-05
ENSMUSG00000025402	Nab2	Ngfi-A binding protein 2 [Source:MGI Symbol;Acc:M	4.51E-05
ENSMUSG00000050192	Eif5a2	eukaryotic translation initiation factor 5A2 [Source:	4.56E-05
ENSMUSG00000040473	A330021E22Rik	RIKEN cDNA A330021E22 gene [Source:MGI Symbo	4.66E-05
ENSMUSG00000027555	Car13	carbonic anhydrase 13 [Source:MGI Symbol;Acc:M	4.67E-05
ENSMUSG00000050463	Krt78	keratin 78 [Source:MGI Symbol;Acc:MGI:1917529]	4.69E-05
ENSMUSG00000035638	Muc20	mucin 20 [Source:MGI Symbol;Acc:MGI:2385039]	4.76E-05
ENSMUSG00000063406	Tmed5	transmembrane emp24 protein transport domain c	4.77E-05
ENSMUSG00000024383	Map3k2	mitogen-activated protein kinase kinase kinase 2 [S	4.79E-05
ENSMUSG00000044951	Mylk4	myosin light chain kinase family, member 4 [Source	4.85E-05

ENSMUSG00000039704	Lmbrd2	LMBR1 domain containing 2 [Source:MGI Symbol;A	4.86E-05
ENSMUSG00000094786	Gm14403	predicted gene 14403 [Source:MGI Symbol;Acc:MG	4.9E-05
ENSMUSG00000048015	Neurod4	neurogenic differentiation 4 [Source:MGI Symbol;A	4.93E-05
ENSMUSG00000036446	Lum	lumican [Source:MGI Symbol;Acc:MGI:109347]	5.02E-05
ENSMUSG00000044518	Foxe3	forkhead box E3 [Source:MGI Symbol;Acc:MGI:135	5.06E-05
ENSMUSG00000031647	Mfap3l	microfibrillar-associated protein 3-like [Source:MGI	5.07E-05
ENSMUSG00000048249	Crebrf	CREB3 regulatory factor [Source:MGI Symbol;Acc:M	5.18E-05
ENSMUSG00000022598	Psca	prostate stem cell antigen [Source:MGI Symbol;Acc	5.2E-05
ENSMUSG00000090744	Gm6871	predicted gene 6871 [Source:MGI Symbol;Acc:MGI	5.34E-05
ENSMUSG00000029267	Mtf2	metal response element binding transcription facto	5.35E-05
ENSMUSG00000044647	Csrnp3	cysteine-serine-rich nuclear protein 3 [Source:MGI	5.36E-05
ENSMUSG00000075020	E530001K10Rik	RIKEN cDNA E530001K10 gene [Source:MGI Symbo	5.38E-05
ENSMUSG00000094942	Gm3604	predicted gene 3604 [Source:MGI Symbol;Acc:MGI	5.46E-05
ENSMUSG00000074449	Gm15319	predicted gene 15319 [Source:MGI Symbol;Acc:MG	5.47E-05
ENSMUSG00000062743	Zfp677	zinc finger protein 677 [Source:MGI Symbol;Acc:M	5.67E-05
ENSMUSG00000000787	Ddx3x	DEAD/H (Asp-Glu-Ala-Asp/His) box polypeptide 3, X	5.68E-05
ENSMUSG00000030386	Zfp606	zinc finger protein 606 [Source:MGI Symbol;Acc:M	5.71E-05
ENSMUSG00000032449	Slc25a36	solute carrier family 25, member 36 [Source:MGI Sy	5.75E-05
ENSMUSG00000019971	Cep290	centrosomal protein 290 [Source:MGI Symbol;Acc:I	5.81E-05
ENSMUSG00000020464	Pnpt1	polyribonucleotide nucleotidyltransferase 1 [Source	5.82E-05
ENSMUSG00000021020	Srp54c	signal recognition particle 54C [Source:MGI Symbol	5.96E-05
ENSMUSG00000038070	Cntln	centlein, centrosomal protein [Source:MGI Symbol;	6.04E-05
ENSMUSG00000097751	Gm26690	predicted gene, 26690 [Source:MGI Symbol;Acc:M	6.2E-05
ENSMUSG00000050891	Tatdn1	TatD DNase domain containing 1 [Source:MGI Sym	6.25E-05
ENSMUSG00000036257	Pnpla8	patatin-like phospholipase domain containing 8 [So	6.37E-05
ENSMUSG00000097727	F630040K05Rik	RIKEN cDNA F630040K05 gene [Source:MGI Symbo	6.46E-05
ENSMUSG00000040774	Cept1	choline/ethanolaminephosphotransferase 1 [Sourc	6.47E-05
ENSMUSG00000036256	Igfbp7	insulin-like growth factor binding protein 7 [Source	6.49E-05
ENSMUSG00000061244	Exoc5	exocyst complex component 5 [Source:MGI Symbo	6.51E-05
ENSMUSG00000056216	Cebpg	CCAAT/enhancer binding protein (C/EBP), gamma [	6.54E-05
ENSMUSG00000034362	Csta	cystatin A [Source:MGI Symbol;Acc:MGI:3524930]	6.55E-05
ENSMUSG00000040850	Psme4	proteasome (prosome, macropain) activator subun	6.6E-05
ENSMUSG00000022884	Eif4a2	eukaryotic translation initiation factor 4A2 [Source:	6.61E-05
ENSMUSG00000024146	Cript	cysteine-rich PDZ-binding protein [Source:MGI Sym	6.67E-05
ENSMUSG00000038122	Tbc1d32	TBC1 domain family, member 32 [Source:MGI Sym	6.7E-05
ENSMUSG00000042363	Lgalsl	lectin, galactoside binding-like [Source:MGI Symbol	6.71E-05
ENSMUSG00000094114	Gm21967	predicted gene, 21967 [Source:MGI Symbol;Acc:M	6.72E-05
ENSMUSG00000040455	Usp45	ubiquitin specific petidase 45 [Source:MGI Symbol;	6.77E-05
ENSMUSG00000063108	Zfp26	zinc finger protein 26 [Source:MGI Symbol;Acc:MGI	6.84E-05
ENSMUSG00000027022	Xirp2	xin actin-binding repeat containing 2 [Source:MGI S	6.87E-05
ENSMUSG00000021597	Ankrd32	ankyrin repeat domain 32 [Source:MGI Symbol;Acc	6.9E-05
ENSMUSG00000024789	Jak2	Janus kinase 2 [Source:MGI Symbol;Acc:MGI:96629	6.94E-05
ENSMUSG00000075273	Ttc30b	tetratricopeptide repeat domain 30B [Source:MGI S	6.99E-05
ENSMUSG00000003623	Crot	carnitine O-octanoyltransferase [Source:MGI Symb	7.16E-05
ENSMUSG00000021711	Trappc13	trafficking protein particle complex 13 [Source:MGI	7.24E-05
ENSMUSG00000034560	A230046K03Rik	RIKEN cDNA A230046K03 gene [Source:MGI Symbo	7.44E-05
ENSMUSG00000042063	Zfp386	zinc finger protein 386 (Kruppel-like) [Source:MGI S	7.46E-05
ENSMUSG00000028410	Dnaja1	DnaJ (Hsp40) homolog, subfamily A, member 1 [Sou	7.47E-05
ENSMUSG00000048279	Sacs	sacsin [Source:MGI Symbol;Acc:MGI:1354724]	7.47E-05

ENSMUSG00000004842	Pou1f1	POU domain, class 1, transcription factor 1 [Source	7.59E-05
ENSMUSG00000030446	Zfp273	zinc finger protein 273 [Source:MGI Symbol;Acc:MG	7.66E-05
ENSMUSG00000019877	Serinc1	serine incorporator 1 [Source:MGI Symbol;Acc:MGI	7.66E-05
ENSMUSG00000051079	Rgs13	regulator of G-protein signaling 13 [Source:MGI Sym	7.7E-05
ENSMUSG00000072941	Sod3	superoxide dismutase 3, extracellular [Source:MGI	7.76E-05
ENSMUSG00000036503	Rnf13	ring finger protein 13 [Source:MGI Symbol;Acc:MGI	7.87E-05
ENSMUSG00000028282	Casp8ap2	caspase 8 associated protein 2 [Source:MGI Symbo	8.05E-05
ENSMUSG00000022336	Eif3e	eukaryotic translation initiation factor 3, subunit E	8.05E-05
ENSMUSG00000035958	Tdp2	tyrosyl-DNA phosphodiesterase 2 [Source:MGI Sym	8.46E-05
ENSMUSG00000063894	Zkscan8	zinc finger with KRAB and SCAN domains 8 [Source:	8.47E-05
ENSMUSG00000025544	Tm9sf2	transmembrane 9 superfamily member 2 [Source:M	8.68E-05
ENSMUSG00000073678	Pgap1	post-GPI attachment to proteins 1 [Source:MGI Sym	8.68E-05
ENSMUSG00000085873	Gm12750	predicted gene 12750 [Source:MGI Symbol;Acc:MG	8.85E-05
ENSMUSG00000028232	Tmem68	transmembrane protein 68 [Source:MGI Symbol;Ac	8.87E-05
ENSMUSG00000014932	Yes1	Yamaguchi sarcoma viral (v-yes) oncogene homolog	8.91E-05
ENSMUSG00000023945	Slc5a7	solute carrier family 5 (choline transporter), memb	8.95E-05
ENSMUSG00000094856	Gm21962	predicted gene, 21962 [Source:MGI Symbol;Acc:MG	8.99E-05
ENSMUSG00000084911	Gm16185	predicted gene 16185 [Source:MGI Symbol;Acc:MG	8.99E-05
ENSMUSG00000090963	Gm17655	predicted gene, 17655 [Source:MGI Symbol;Acc:MG	9.06E-05
ENSMUSG00000093385	A330044P14Rik	RIKEN cDNA A330044P14 gene [Source:MGI Symbo	9.29E-05
ENSMUSG00000091474	2610021A01Rik	RIKEN cDNA 2610021A01 gene [Source:MGI Symbo	9.63E-05
ENSMUSG00000027176	Cstf3	cleavage stimulation factor, 3' pre-RNA, subunit 3 [	9.83E-05
ENSMUSG00000022180	Slc7a8	solute carrier family 7 (cationic amino acid transpo	9.88E-05
ENSMUSG00000039219	Arid4b	AT rich interactive domain 4B (RBP1-like) [Source:M	0.000101
ENSMUSG00000067627	Gm10217	predicted gene 10217 [Source:MGI Symbol;Acc:MG	0.000104
ENSMUSG00000091264	Smim13	small integral membrane protein 13 [Source:MGI S	0.000104
ENSMUSG00000037075	Rnf139	ring finger protein 139 [Source:MGI Symbol;Acc:MG	0.000105
ENSMUSG00000028576	Ift74	intraflagellar transport 74 [Source:MGI Symbol;Acc	0.000105
ENSMUSG00000021612	Slc6a18	solute carrier family 6 (neurotransmitter transporte	0.000105
ENSMUSG00000031099	Smarca1	SWI/SNF related, matrix associated, actin depender	0.000106
ENSMUSG00000056380	Gpr50	G-protein-coupled receptor 50 [Source:MGI Symbo	0.000108
ENSMUSG00000040536	Necab1	N-terminal EF-hand calcium binding protein 1 [Sour	0.000108
ENSMUSG00000033964	Zbtb41	zinc finger and BTB domain containing 41 homolog	0.000111
ENSMUSG00000020149	Rab1	RAB1, member RAS oncogene family [Source:MGI S	0.000112
ENSMUSG00000019841	Rev3l	REV3-like, catalytic subunit of DNA polymerase zeta	0.000115
ENSMUSG00000006699	Cdc42	cell division cycle 42 [Source:MGI Symbol;Acc:MGI:	0.000115
ENSMUSG00000042282	Gucy2f	guanylate cyclase 2f [Source:MGI Symbol;Acc:MGI:	0.000115
ENSMUSG00000060681	Slc9a6	solute carrier family 9 (sodium/hydrogen exchange	0.000117
ENSMUSG00000026415	Fcamr	Fc receptor, IgA, IgM, high affinity [Source:MGI Sym	0.000117
ENSMUSG00000042306	S100a14	S100 calcium binding protein A14 [Source:MGI Sym	0.000118
ENSMUSG00000039105	Atp6v1g1	ATPase, H+ transporting, lysosomal V1 subunit G1 [	0.000118
ENSMUSG00000018800	Abca5	ATP-binding cassette, sub-family A (ABC1), membe	0.000118
ENSMUSG00000041763	Tpp2	tripeptidyl peptidase II [Source:MGI Symbol;Acc:MG	0.000119
ENSMUSG00000040037	Negr1	neuronal growth regulator 1 [Source:MGI Symbol;A	0.000119
ENSMUSG00000022237	Ankrd33b	ankyrin repeat domain 33B [Source:MGI Symbol;Ac	0.000121
ENSMUSG00000047648	Fbxo30	F-box protein 30 [Source:MGI Symbol;Acc:MGI:191	0.000121
ENSMUSG00000020300	Cpeb4	cytoplasmic polyadenylation element binding prote	0.000121
ENSMUSG00000020570	Sypl	synaptophysin-like protein [Source:MGI Symbol;Acc	0.000123
ENSMUSG00000022858	Tra2b	transformer 2 beta homolog (Drosophila) [Source:M	0.000123

ENSMUSG00000074794	Arrdc3	arrestin domain containing 3 [Source:MGI Symbol;A	0.000124
ENSMUSG00000093470	Gm20645	predicted gene 20645 [Source:MGI Symbol;Acc:MG	0.000124
ENSMUSG00000074781	Ube2n	ubiquitin-conjugating enzyme E2N [Source:MGI Syr	0.000124
ENSMUSG00000022861	Dgkg	diacylglycerol kinase, gamma [Source:MGI Symbol;A	0.000125
ENSMUSG00000020664	Dld	dihydrolipoamide dehydrogenase [Source:MGI Sym	0.000127
ENSMUSG00000023809	Rps6ka2	ribosomal protein S6 kinase, polypeptide 2 [Source	0.000129
ENSMUSG00000033799	BC016423	cDNA sequence BC016423 [Source:MGI Symbol;Acc	0.000131
ENSMUSG00000028071	Sh2d2a	SH2 domain protein 2A [Source:MGI Symbol;Acc:M	0.000133
ENSMUSG00000029840	Mtpn	myotrophin [Source:MGI Symbol;Acc:MGI:99445]	0.000133
ENSMUSG00000059920	4930453N24Rik	RIKEN cDNA 4930453N24 gene [Source:MGI Symbo	0.000133
ENSMUSG00000026094	Stk17b	serine/threonine kinase 17b (apoptosis-inducing) [S	0.000133
ENSMUSG00000028381	Ugcg	UDP-glucose ceramide glucosyltransferase [Source:	0.000134
ENSMUSG00000021870	Slmap	sarcolemma associated protein [Source:MGI Symbo	0.000135
ENSMUSG00000031278	Acsl4	acyl-CoA synthetase long-chain family member 4 [S	0.000137
ENSMUSG00000069114	Zbtb10	zinc finger and BTB domain containing 10 [Source:N	0.000139
ENSMUSG00000027293	Ehd4	EH-domain containing 4 [Source:MGI Symbol;Acc:M	0.00014
ENSMUSG00000041684	Bivm	basic, immunoglobulin-like variable motif containin	0.00014
ENSMUSG00000037003	Tenc1	tensin like C1 domain-containing phosphatase [Sou	0.000141
ENSMUSG00000043498	9330132A10Rik	RIKEN cDNA 9330132A10 gene [Source:MGI Symbo	0.000141
ENSMUSG00000041685	Fcho2	FCH domain only 2 [Source:MGI Symbol;Acc:MGI:3	0.000141
ENSMUSG00000021099	Six6	sine oculis-related homeobox 6 [Source:MGI Symbo	0.000143
ENSMUSG00000028261	Ndufaf4	NADH dehydrogenase (ubiquinone) 1 alpha subcom	0.000144
ENSMUSG00000016382	Pls3	plastin 3 (T-isoform) [Source:MGI Symbol;Acc:MGI:	0.000147
ENSMUSG00000026921	Egfl7	EGF-like domain 7 [Source:MGI Symbol;Acc:MGI:24	0.000147
ENSMUSG00000029211	Gabra4	gamma-aminobutyric acid (GABA) A receptor, subu	0.00015
ENSMUSG00000001774	Chordc1	cysteine and histidine-rich domain (CHORD)-contai	0.000151
ENSMUSG00000090353	Gm17555	predicted gene, 17555 [Source:MGI Symbol;Acc:MG	0.000152
ENSMUSG00000049908	Gja8	gap junction protein, alpha 8 [Source:MGI Symbol;A	0.000152
ENSMUSG00000054408	Spcs3	signal peptidase complex subunit 3 homolog (S. cer	0.000153
ENSMUSG00000032872	Cyb5r4	cytochrome b5 reductase 4 [Source:MGI Symbol;Ac	0.000155
ENSMUSG00000078624	Olfr613	olfactory receptor 613 [Source:MGI Symbol;Acc:MG	0.000156
ENSMUSG00000021508	Cxcl14	chemokine (C-X-C motif) ligand 14 [Source:MGI Syn	0.000159
ENSMUSG00000057455	Rit2	Ras-like without CAAX 2 [Source:MGI Symbol;Acc:M	0.000162
ENSMUSG00000021969	Zdhhc20	zinc finger, DHHC domain containing 20 [Source:MG	0.000162
ENSMUSG00000041408	Wapal	wings apart-like homolog (Drosophila) [Source:MGI	0.000167
ENSMUSG00000075707	Dio3	deiodinase, iodothyronine type III [Source:MGI Sym	0.000168
ENSMUSG00000027132	Katnbl1	katanin p80 subunit B like 1 [Source:MGI Symbol;Ac	0.000169
ENSMUSG00000028252	Ccnc	cyclin C [Source:MGI Symbol;Acc:MGI:1858199]	0.000169
ENSMUSG00000024993	Fam45a	family with sequence similarity 45, member A [Sou	0.00017
ENSMUSG00000096262	Ap1g1	adaptor protein complex AP-1, gamma 1 subunit [S	0.000172
ENSMUSG00000022752	Tomm70a	translocase of outer mitochondrial membrane 70 h	0.000177
ENSMUSG00000082319	Gm8822	predicted gene 8822 [Source:MGI Symbol;Acc:MGI	0.000178
ENSMUSG00000023882	Zfp54	zinc finger protein 54 [Source:MGI Symbol;Acc:MGI	0.000181
ENSMUSG00000026797	Stxbp1	syntaxin binding protein 1 [Source:MGI Symbol;Acc	0.000181
ENSMUSG00000020719	Ddx5	DEAD (Asp-Glu-Ala-Asp) box polypeptide 5 [Source:	0.000183
ENSMUSG00000025982	Sf3b1	splicing factor 3b, subunit 1 [Source:MGI Symbol;Ac	0.000184
ENSMUSG00000068748	Ptprz1	protein tyrosine phosphatase, receptor type Z, poly	0.000185
ENSMUSG00000030650	Tmc5	transmembrane channel-like gene family 5 [Source	0.000186
ENSMUSG00000034120	Srsf2	serine/arginine-rich splicing factor 2 [Source:MGI S	0.000187

ENSMUSG00000034059	Ypel4	yippee-like 4 (Drosophila) [Source:MGI Symbol;Acc	0.00019
ENSMUSG00000021238	Aldh6a1	aldehyde dehydrogenase family 6, subfamily A1 [Sc	0.000192
ENSMUSG00000033186	Mzt1	mitotic spindle organizing protein 1 [Source:MGI Sy	0.000192
ENSMUSG00000067608	Pcna-ps2	proliferating cell nuclear antigen pseudogene 2 [So	0.000193
ENSMUSG00000063455	D630045J12Rik	RIKEN cDNA D630045J12 gene [Source:MGI Symbo	0.000195
ENSMUSG00000050493	Fam167b	family with sequence similarity 167, member B [Sou	0.000195
ENSMUSG00000028986	Klhl7	kelch-like 7 [Source:MGI Symbol;Acc:MGI:1196453]	0.000199
ENSMUSG00000026204	Ptprn	protein tyrosine phosphatase, receptor type, N [Sou	0.000199
ENSMUSG00000025234	Arih1	ariadne ubiquitin-conjugating enzyme E2 binding p	0.0002
ENSMUSG00000022905	Kpna1	karyopherin (importin) alpha 1 [Source:MGI Symbo	0.0002
ENSMUSG00000023868	Pde10a	phosphodiesterase 10A [Source:MGI Symbol;Acc:M	0.000203
ENSMUSG00000036916	Zfp280c	zinc finger protein 280C [Source:MGI Symbol;Acc:M	0.000203
ENSMUSG00000025742	Prps2	phosphoribosyl pyrophosphate synthetase 2 [Sourc	0.000204
ENSMUSG00000054752	Fsd1l	fibronectin type III and SPRY domain containing 1-li	0.000204
ENSMUSG00000007613	Tgfbr1	transforming growth factor, beta receptor I [Source	0.000205
ENSMUSG00000031762	Mt2	metallothionein 2 [Source:MGI Symbol;Acc:MGI:97	0.000206
ENSMUSG00000044393	Dsg2	desmoglein 2 [Source:MGI Symbol;Acc:MGI:119646	0.000206
ENSMUSG00000032058	Ppp2r1b	protein phosphatase 2 (formerly 2A), regulatory su	0.000207
ENSMUSG00000052131	Akr1b7	aldo-keto reductase family 1, member B7 [Source:M	0.000208
ENSMUSG00000021388	Aspn	asporin [Source:MGI Symbol;Acc:MGI:1913945]	0.000211
ENSMUSG00000027828	Ssr3	signal sequence receptor, gamma [Source:MGI Sym	0.000212
ENSMUSG00000025064	Col17a1	collagen, type XVII, alpha 1 [Source:MGI Symbol;Ac	0.000214
ENSMUSG00000019944	Rhobtb1	Rho-related BTB domain containing 1 [Source:MGI	0.000215
ENSMUSG00000032502	Stac	src homology three (SH3) and cysteine rich domain	0.000215
ENSMUSG00000090622	A930033H14Rik	RIKEN cDNA A930033H14 gene [Source:MGI Symbo	0.000216
ENSMUSG00000023025	Larp4	La ribonucleoprotein domain family, member 4 [So	0.000216
ENSMUSG00000031342	Gpm6b	glycoprotein m6b [Source:MGI Symbol;Acc:MGI:10	0.000216
ENSMUSG00000097469	Gm26733	predicted gene, 26733 [Source:MGI Symbol;Acc:MG	0.000221
ENSMUSG00000028015	Ctso	cathepsin O [Source:MGI Symbol;Acc:MGI:2139628	0.000223
ENSMUSG00000054459	Vsnl1	visinin-like 1 [Source:MGI Symbol;Acc:MGI:134945	0.000229
ENSMUSG00000022472	Desi1	desumoylating isopeptidase 1 [Source:MGI Symbol	0.000229
ENSMUSG00000021733	Slc4a7	solute carrier family 4, sodium bicarbonate cotrans	0.000232
ENSMUSG00000027162	Lin7c	lin-7 homolog C (C. elegans) [Source:MGI Symbol;A	0.000232
ENSMUSG00000001435	Col18a1	collagen, type XVIII, alpha 1 [Source:MGI Symbol;A	0.000232
ENSMUSG00000039238	Zfp750	zinc finger protein 750 [Source:MGI Symbol;Acc:MG	0.000233
ENSMUSG00000024759	Atl3	atlastin GTPase 3 [Source:MGI Symbol;Acc:MGI:192	0.000235
ENSMUSG00000052562	Slc22a30	solute carrier family 22, member 30 [Source:MGI Sy	0.000237
ENSMUSG00000035861	Tmprss11bnl	transmembrane protease, serine 11b N terminal lik	0.000237
ENSMUSG00000052305	Hbb-bs	hemoglobin, beta adult s chain [Source:MGI Symbo	0.000238
ENSMUSG00000036810	Cnep1r1	CTD nuclear envelope phosphatase 1 regulatory sul	0.000238
ENSMUSG00000056665	Them6	thioesterase superfamily member 6 [Source:MGI Sy	0.00024
ENSMUSG00000028484	Psip1	PC4 and SFRS1 interacting protein 1 [Source:MGI Sy	0.000241
ENSMUSG00000038128	Camk4	calcium/calmodulin-dependent protein kinase IV [S	0.000242
ENSMUSG00000084913	Gm7616	predicted gene 7616 [Source:MGI Symbol;Acc:MGI:	0.000242
ENSMUSG00000018924	Alox15	arachidonate 15-lipoxygenase [Source:MGI Symbol	0.000242
ENSMUSG00000055561	Spink5	serine peptidase inhibitor, Kazal type 5 [Source:MG	0.000243
ENSMUSG00000022722	Arl6	ADP-ribosylation factor-like 6 [Source:MGI Symbol;	0.000243
ENSMUSG00000020572	Nampt	nicotinamide phosphoribosyltransferase [Source:M	0.000244
ENSMUSG00000085678	Gm16349	predicted gene 16349 [Source:MGI Symbol;Acc:MG	0.000246

ENSMUSG00000098041	Gm26981	predicted gene, 26981 [Source:MGI Symbol;Acc:M	0.00025
ENSMUSG00000038679	Trps1	trichorhinophalangeal syndrome I (human) [Source	0.000255
ENSMUSG00000031242	2610002M06Rik	RIKEN cDNA 2610002M06 gene [Source:MGI Symb	0.000255
ENSMUSG00000021303	Gng4	guanine nucleotide binding protein (G protein), gar	0.000257
ENSMUSG00000003929	Zfp81	zinc finger protein 81 [Source:MGI Symbol;Acc:MG	0.000258
ENSMUSG00000034488	Edil3	EGF-like repeats and discoidin I-like domains 3 [Sou	0.000258
ENSMUSG00000060427	Zfp868	zinc finger protein 868 [Source:MGI Symbol;Acc:M	0.000259
ENSMUSG00000069727	Gm5595	predicted gene 5595 [Source:MGI Symbol;Acc:MGI:	0.000259
ENSMUSG00000031765	Mt1	metallothionein 1 [Source:MGI Symbol;Acc:MGI:97	0.00026
ENSMUSG00000049583	Grm5	glutamate receptor, metabotropic 5 [Source:MGI S	0.000264
ENSMUSG00000020385	Clk4	CDC like kinase 4 [Source:MGI Symbol;Acc:MGI:109	0.000265
ENSMUSG00000013662	Atad1	ATPase family, AAA domain containing 1 [Source:M	0.000269
ENSMUSG00000028228	Cpne3	copine III [Source:MGI Symbol;Acc:MGI:1917818]	0.00027
ENSMUSG00000025777	Gdap1	ganglioside-induced differentiation-associated-prot	0.000272
ENSMUSG00000028560	Usp1	ubiquitin specific peptidase 1 [Source:MGI Symbol;	0.000273
ENSMUSG00000027018	Hat1	histone aminotransferase 1 [Source:MGI Symbol;A	0.000276
ENSMUSG00000027792	Bche	butyrylcholinesterase [Source:MGI Symbol;Acc:MG	0.000281
ENSMUSG00000021930	Spryd7	SPRY domain containing 7 [Source:MGI Symbol;Acc	0.000281
ENSMUSG00000020015	Cdk17	cyclin-dependent kinase 17 [Source:MGI Symbol;A	0.000283
ENSMUSG00000073563	Csnk1g3	casein kinase 1, gamma 3 [Source:MGI Symbol;Acc:	0.000283
ENSMUSG00000030846	Tial1	Tia1 cytotoxic granule-associated RNA binding prot	0.000285
ENSMUSG00000031425	Plp1	proteolipid protein (myelin) 1 [Source:MGI Symbol;	0.000286
ENSMUSG00000041828	Abca8a	ATP-binding cassette, sub-family A (ABC1), membe	0.000288
ENSMUSG00000035640	Dos	downstream of Stk11 [Source:MGI Symbol;Acc:MG	0.00029
ENSMUSG00000087370	Tmem170b	transmembrane protein 170B [Source:MGI Symbol;	0.000291
ENSMUSG00000036478	Btg1	B cell translocation gene 1, anti-proliferative [Sourc	0.000291
ENSMUSG00000062627	Mysm1	myb-like, SWIRM and MPN domains 1 [Source:MGI	0.000294
ENSMUSG00000049112	Oxtr	oxytocin receptor [Source:MGI Symbol;Acc:MGI:10	0.000295
ENSMUSG00000031196	F8	coagulation factor VIII [Source:MGI Symbol;Acc:MG	0.000295
ENSMUSG00000064340	mt-Tl1	mitochondrially encoded tRNA leucine 1 [Source:M	0.000298
ENSMUSG00000044217	Aqp5	aquaporin 5 [Source:MGI Symbol;Acc:MGI:106215]	0.0003
ENSMUSG00000060090	Rp2h	retinitis pigmentosa 2 homolog (human) [Source:M	0.000301
ENSMUSG00000016409	Nkap	NFKB activating protein [Source:MGI Symbol;Acc:M	0.000301
ENSMUSG00000026103	Gls	glutaminase [Source:MGI Symbol;Acc:MGI:95752]	0.000305
ENSMUSG00000052783	Grk4	G protein-coupled receptor kinase 4 [Source:MGI S	0.000309
ENSMUSG00000031326	Cdx4	caudal type homeobox 4 [Source:MGI Symbol;Acc:I	0.00031
ENSMUSG00000046699	Slitrk4	SLIT and NTRK-like family, member 4 [Source:MGI S	0.00031
ENSMUSG00000020516	Rps6kb1	ribosomal protein S6 kinase, polypeptide 1 [Source	0.000313
ENSMUSG00000020671	Rab10	RAB10, member RAS oncogene family [Source:MGI	0.000314
ENSMUSG00000054945	Gm9958	predicted gene 9958 [Source:MGI Symbol;Acc:MGI:	0.000315
ENSMUSG00000050714	Zbtb26	zinc finger and BTB domain containing 26 [Source:N	0.000318
ENSMUSG00000055531	Cpsf6	cleavage and polyadenylation specific factor 6 [Sou	0.000319
ENSMUSG00000035498	Cdcp1	CUB domain containing protein 1 [Source:MGI Sym	0.000321
ENSMUSG00000040929	Rfx3	regulatory factor X, 3 (influences HLA class II expres	0.000322
ENSMUSG00000025240	Sacm1l	SAC1 (suppressor of actin mutations 1, homolog)-li	0.000323
ENSMUSG00000090553	Snrpe	small nuclear ribonucleoprotein E [Source:MGI Sym	0.000326
ENSMUSG00000003363	Pld3	phospholipase D family, member 3 [Source:MGI Sy	0.000326
ENSMUSG00000032002	Dcun1d5	DCN1, defective in cullin neddylation 1, domain cor	0.00033
ENSMUSG00000028161	Ppp3ca	protein phosphatase 3, catalytic subunit, alpha isof	0.000332

ENSMUSG00000020620	Abca8b	ATP-binding cassette, sub-family A (ABC1), membe	0.000335
ENSMUSG00000025893	Kbtbd3	kelch repeat and BTB (POZ) domain containing 3 [S	0.000338
ENSMUSG00000079317	Trappc2	trafficking protein particle complex 2 [Source:MGI	0.000338
ENSMUSG00000024298	Zfp871	zinc finger protein 871 [Source:MGI Symbol;Acc:M	0.000342
ENSMUSG00000029208	Guf1	GUF1 GTPase homolog (S. cerevisiae) [Source:MGI	0.000343
ENSMUSG00000017412	Cacnb4	calcium channel, voltage-dependent, beta 4 subuni	0.000344
ENSMUSG00000033417	Cacul1	CDK2 associated, cullin domain 1 [Source:MGI Sym	0.000346
ENSMUSG00000031613	Hpgd	hydroxyprostaglandin dehydrogenase 15 (NAD) [So	0.00035
ENSMUSG00000040693	Slco4c1	solute carrier organic anion transporter family, me	0.000351
ENSMUSG00000044748	Defb1	defensin beta 1 [Source:MGI Symbol;Acc:MGI:1096	0.000354
ENSMUSG00000021730	Hcn1	hyperpolarization-activated, cyclic nucleotide-gate	0.000358
ENSMUSG00000027011	Ube2e3	ubiquitin-conjugating enzyme E2E 3 [Source:MGI S	0.000366
ENSMUSG00000034739	Mfrp	membrane-type frizzled-related protein [Source:M	0.000367
ENSMUSG00000002222	Rmnd5a	required for meiotic nuclear division 5 homolog A (	0.00037
ENSMUSG00000006143	Upk3bl	uroplakin 3B-like [Source:MGI Symbol;Acc:MGI:191	0.000371
ENSMUSG00000015757	Ppil4	peptidylprolyl isomerase (cyclophilin)-like 4 [Source	0.000374
ENSMUSG00000037185	Krt80	keratin 80 [Source:MGI Symbol;Acc:MGI:1921377]	0.000375
ENSMUSG00000096401	Gm21811	predicted gene, 21811 [Source:MGI Symbol;Acc:M	0.000376
ENSMUSG00000087278	A930006I01Rik	RIKEN cDNA A930006I01 gene [Source:MGI Symbol	0.000377
ENSMUSG00000021519	Mterfd1	MTERF domain containing 1 [Source:MGI Symbol;A	0.000377
ENSMUSG00000022016	Akap11	A kinase (PRKA) anchor protein 11 [Source:MGI Sy	0.000377
ENSMUSG00000026739	Bmi1	Bmi1 polycomb ring finger oncogene [Source:MGI	0.000383
ENSMUSG00000022707	Gbe1	glucan (1,4-alpha-), branching enzyme 1 [Source:M	0.000387
ENSMUSG00000002428	Hltf	helicase-like transcription factor [Source:MGI Symb	0.000389
ENSMUSG00000027835	Pdcd10	programmed cell death 10 [Source:MGI Symbol;Ac	0.00039
ENSMUSG00000014725	Adam28	a disintegrin and metallopeptidase domain 28 [Sou	0.000391
ENSMUSG00000025766	D3Ertd751e	DNA segment, Chr 3, ERATO Doi 751, expressed [S	0.000395
ENSMUSG00000048373	Fgfbp1	fibroblast growth factor binding protein 1 [Source:	0.000395
ENSMUSG00000002265	Peg3	paternally expressed 3 [Source:MGI Symbol;Acc:M	0.000395
ENSMUSG00000029787	Avl9	AVL9 homolog (S. cerevisiase) [Source:MGI Symbol	0.000398
ENSMUSG00000066595	Mfsd7b	major facilitator superfamily domain containing 7B	0.000399
ENSMUSG00000033411	Ctdspl2	CTD (carboxy-terminal domain, RNA polymerase II,	0.000402
ENSMUSG00000001025	S100a6	S100 calcium binding protein A6 (calcyclin) [Source	0.000404
ENSMUSG00000051037	Zfp455	zinc finger protein 455 [Source:MGI Symbol;Acc:M	0.000406
ENSMUSG00000029361	Nos1	nitric oxide synthase 1, neuronal [Source:MGI Symb	0.00041
ENSMUSG00000028552	Eps15	epidermal growth factor receptor pathway substra	0.000412
ENSMUSG00000028156	Eif4e	eukaryotic translation initiation factor 4E [Source:M	0.000415
ENSMUSG00000025407	Gli1	GLI-Kruppel family member GLI1 [Source:MGI Symb	0.000416
ENSMUSG00000089824	Rbm12	RNA binding motif protein 12 [Source:MGI Symbol;	0.000416
ENSMUSG00000092335	Gm7221	predicted gene 7221 [Source:MGI Symbol;Acc:MGI	0.000417
ENSMUSG00000057457	Phex	phosphate regulating gene with homologies to end	0.000417
ENSMUSG00000020326	Ccng1	cyclin G1 [Source:MGI Symbol;Acc:MGI:102890]	0.000418
ENSMUSG00000023982	Guca1a	guanylate cyclase activator 1a (retina) [Source:MGI	0.000418
ENSMUSG00000020745	Pafah1b1	platelet-activating factor acetylhydrolase, isoform 1	0.000419
ENSMUSG00000091625	Lsm5	LSM5 homolog, U6 small nuclear RNA associated (S	0.000422
ENSMUSG00000012640	Zfp715	zinc finger protein 715 [Source:MGI Symbol;Acc:M	0.000428
ENSMUSG00000022096	Hr	hairless [Source:MGI Symbol;Acc:MGI:96223]	0.000429
ENSMUSG00000016477	E2f3	E2F transcription factor 3 [Source:MGI Symbol;Acc:	0.000432
ENSMUSG00000035049	Rrp12	ribosomal RNA processing 12 homolog (S. cerevisia	0.000435

ENSMUSG00000054814	Usp46	ubiquitin specific peptidase 46 [Source:MGI Symbo	0.000438
ENSMUSG00000026097	Ormdl1	ORM1-like 1 (S. cerevisiae) [Source:MGI Symbol;Ac	0.000443
ENSMUSG00000026380	Tfcp2l1	transcription factor CP2-like 1 [Source:MGI Symbol	0.000444
ENSMUSG00000033981	Gria2	glutamate receptor, ionotropic, AMPA2 (alpha 2) [S	0.000444
ENSMUSG00000040254	Sema3d	sema domain, immunoglobulin domain (Ig), short b	0.000446
ENSMUSG00000031304	Il2rg	interleukin 2 receptor, gamma chain [Source:MGI S	0.000449
ENSMUSG00000025001	Hells	helicase, lymphoid specific [Source:MGI Symbol;Ac	0.000453
ENSMUSG00000051111	Sv2c	synaptic vesicle glycoprotein 2c [Source:MGI Symb	0.000455
ENSMUSG00000066090	Insl5	insulin-like 5 [Source:MGI Symbol;Acc:MGI:134608	0.000456
ENSMUSG00000032231	Anxa2	annexin A2 [Source:MGI Symbol;Acc:MGI:88246]	0.000459
ENSMUSG00000034551	Hdx	highly divergent homeobox [Source:MGI Symbol;Ac	0.00046
ENSMUSG00000079451	Tmprss11g	transmembrane protease, serine 11g [Source:MGI	0.000468
ENSMUSG00000059820	AU019823	expressed sequence AU019823 [Source:MGI Symbo	0.00047
ENSMUSG00000039577	Nphp4	nephronophthisis 4 (juvenile) homolog (human) [So	0.000474
ENSMUSG00000068823	Csde1	cold shock domain containing E1, RNA binding [Sou	0.000477
ENSMUSG00000020077	Srgn	serglycin [Source:MGI Symbol;Acc:MGI:97756]	0.000477
ENSMUSG00000025380	Fscn2	fascin homolog 2, actin-bundling protein, retinal (S	0.000478
ENSMUSG00000029821	Dfna5	deafness, autosomal dominant 5 (human) [Source:	0.000478
ENSMUSG00000000600	Krit1	KRIT1, ankyrin repeat containing [Source:MGI Symb	0.000479
ENSMUSG00000090588	Gm9573	predicted gene 9573 [Source:MGI Symbol;Acc:MGI	0.000484
ENSMUSG00000008333	Snrpb2	U2 small nuclear ribonucleoprotein B [Source:MGI	0.000484
ENSMUSG00000045103	Dmd	dystrophin, muscular dystrophy [Source:MGI Symb	0.000486
ENSMUSG00000050103	Agmo	alkylglycerol monooxygenase [Source:MGI Symbol;	0.000491
ENSMUSG00000082765	Gm14411	predicted gene 14411 [Source:MGI Symbol;Acc:MG	0.000492
ENSMUSG00000028293	Slc35a1	solute carrier family 35 (CMP-sialic acid transporter	0.000502
ENSMUSG00000005360	Slc1a3	solute carrier family 1 (glial high affinity glutamate	0.000503
ENSMUSG00000038777	Sema6c	sema domain, transmembrane domain (TM), and c	0.000505
ENSMUSG00000027822	Slc33a1	solute carrier family 33 (acetyl-CoA transporter), m	0.000505
ENSMUSG00000027805	Pfn2	profilin 2 [Source:MGI Symbol;Acc:MGI:97550]	0.000505
ENSMUSG00000005034	Prkacb	protein kinase, cAMP dependent, catalytic, beta [So	0.000506
ENSMUSG00000089756	Gm8898	predicted gene 8898 [Source:MGI Symbol;Acc:MGI	0.00051
ENSMUSG00000070923	Klhl9	kelch-like 9 [Source:MGI Symbol;Acc:MGI:2180122	0.000513
ENSMUSG00000028217	Cdh17	cadherin 17 [Source:MGI Symbol;Acc:MGI:1095414	0.000514
ENSMUSG00000033685	Ucp2	uncoupling protein 2 (mitochondrial, proton carrier	0.000515
ENSMUSG00000038121	Fam210a	family with sequence similarity 210, member A [So	0.000517
ENSMUSG00000092416	Zfp141	zinc finger protein 141 [Source:MGI Symbol;Acc:M	0.000518
ENSMUSG00000068882	Ssb	Sjogren syndrome antigen B [Source:MGI Symbol;A	0.000519
ENSMUSG00000025630	Hprt	hypoxanthine guanine phosphoribosyl transferase	0.000526
ENSMUSG00000046229	Scand1	SCAN domain-containing 1 [Source:MGI Symbol;Ac	0.000526
ENSMUSG00000041459	Tardbp	TAR DNA binding protein [Source:MGI Symbol;Acc:	0.000527
ENSMUSG00000042742	B630005N14Rik	RIKEN cDNA B630005N14 gene [Source:MGI Symbo	0.000528
ENSMUSG00000079184	Mphosph8	M-phase phosphoprotein 8 [Source:MGI Symbol;Ac	0.000533
ENSMUSG00000029377	Ereg	epiregulin [Source:MGI Symbol;Acc:MGI:107508]	0.000534
ENSMUSG00000035126	Wdr78	WD repeat domain 78 [Source:MGI Symbol;Acc:MG	0.000535
ENSMUSG00000006740	Kif5b	kinesin family member 5B [Source:MGI Symbol;Acc	0.000538
ENSMUSG00000019779	Frk	fyn-related kinase [Source:MGI Symbol;Acc:MGI:10	0.000541
ENSMUSG00000057367	Birc2	baculoviral IAP repeat-containing 2 [Source:MGI Sy	0.000543
ENSMUSG00000042208	0610010F05Rik	RIKEN cDNA 0610010F05 gene [Source:MGI Symbo	0.000544
ENSMUSG00000017057	Il13ra1	interleukin 13 receptor, alpha 1 [Source:MGI Symb	0.000546

ENSMUSG00000018425	Dhx40	DEAH (Asp-Glu-Ala-His) box polypeptide 40 [Source	0.000547
ENSMUSG00000030077	Chl1	cell adhesion molecule with homology to L1CAM [S	0.000555
ENSMUSG00000040782	Rfwd2	ring finger and WD repeat domain 2 [Source:MGI Sy	0.000559
ENSMUSG00000038738	Shank1	SH3/ankyrin domain gene 1 [Source:MGI Symbol;Ac	0.000568
ENSMUSG00000040370	Lyrm5	LYR motif containing 5 [Source:MGI Symbol;Acc:MG	0.000569
ENSMUSG00000014164	Klhl3	kelch-like 3 [Source:MGI Symbol;Acc:MGI:2445185]	0.000578
ENSMUSG00000041378	Cldn5	claudin 5 [Source:MGI Symbol;Acc:MGI:1276112]	0.000587
ENSMUSG00000052581	Lrrtm4	leucine rich repeat transmembrane neuronal 4 [Sou	0.000589
ENSMUSG00000071107	4732465J04Rik	RIKEN cDNA 4732465J04 gene [Source:MGI Symbol	0.000602
ENSMUSG00000021302	Ggps1	geranylgeranyl diphosphate synthase 1 [Source:MG	0.000602
ENSMUSG00000034810	Scn7a	sodium channel, voltage-gated, type VII, alpha [Sou	0.000608
ENSMUSG00000020152	Actr2	ARP2 actin-related protein 2 [Source:MGI Symbol;A	0.000608
ENSMUSG00000029229	Chic2	cysteine-rich hydrophobic domain 2 [Source:MGI Sy	0.00061
ENSMUSG00000058799	Nap1l1	nucleosome assembly protein 1-like 1 [Source:MGI	0.000623
ENSMUSG00000028527	Ak4	adenylate kinase 4 [Source:MGI Symbol;Acc:MGI:87	0.000625
ENSMUSG00000030031	Kbtbd8	kelch repeat and BTB (POZ) domain containing 8 [So	0.000626
ENSMUSG00000033871	Ppargc1b	peroxisome proliferative activated receptor, gamm	0.000632
ENSMUSG00000030465	Psd3	pleckstrin and Sec7 domain containing 3 [Source:M	0.000634
ENSMUSG00000060336	Zfp937	zinc finger protein 937 [Source:MGI Symbol;Acc:MG	0.000642
ENSMUSG00000078862	Gm14326	predicted gene 14326 [Source:MGI Symbol;Acc:MG	0.000643
ENSMUSG00000038836	Agbl3	ATP/GTP binding protein-like 3 [Source:MGI Symbo	0.000643
ENSMUSG00000034158	Lrrc58	leucine rich repeat containing 58 [Source:MGI Symb	0.000654
ENSMUSG00000079508	Apoo	apolipoprotein O [Source:MGI Symbol;Acc:MGI:191	0.000655
ENSMUSG00000030967	Zranb1	zinc finger, RAN-binding domain containing 1 [Sour	0.000655
ENSMUSG00000057337	Chst3	carbohydrate (chondroitin 6/keratan) sulfotransfer	0.000664
ENSMUSG00000022129	Dct	dopachrome tautomerase [Source:MGI Symbol;Acc	0.000665
ENSMUSG00000021221	Dpf3	D4, zinc and double PHD fingers, family 3 [Source:M	0.000666
ENSMUSG00000025584	Pde8a	phosphodiesterase 8A [Source:MGI Symbol;Acc:MG	0.000666
ENSMUSG00000044365	Cxxc4	CXXC finger 4 [Source:MGI Symbol;Acc:MGI:244211	0.000667
ENSMUSG00000019936	Epyc	epiphycan [Source:MGI Symbol;Acc:MGI:107942]	0.000667
ENSMUSG00000025431	Crisp1	cysteine-rich secretory protein 1 [Source:MGI Symb	0.00067
ENSMUSG00000078898	Gm4723	predicted gene 4723 [Source:MGI Symbol;Acc:MGI:	0.000677
ENSMUSG00000060188	Cxcl17	chemokine (C-X-C motif) ligand 17 [Source:MGI Sym	0.000679
ENSMUSG00000026535	Ifi202b	interferon activated gene 202B [Source:MGI Symbo	0.00068
ENSMUSG00000071337	Tia1	cytotoxic granule-associated RNA binding protein 1	0.000699
ENSMUSG00000064348	mt-Tn	mitochondrially encoded tRNA asparagine [Source:	0.000701
ENSMUSG00000047383	Als2cr11	amyotrophic lateral sclerosis 2 (juvenile) chromoso	0.000703
ENSMUSG00000043542	Zc2hc1a	zinc finger, C2HC-type containing 1A [Source:MGI S	0.000712
ENSMUSG00000016559	H3f3b	H3 histone, family 3B [Source:MGI Symbol;Acc:MGI	0.000715
ENSMUSG00000027315	Spint1	serine protease inhibitor, Kunitz type 1 [Source:MG	0.000715
ENSMUSG00000060073	Psma3	proteasome (prosome, macropain) subunit, alpha t	0.000722
ENSMUSG00000034574	Daam1	dishevelled associated activator of morphogenesis	0.000725
ENSMUSG00000031137	Fgf13	fibroblast growth factor 13 [Source:MGI Symbol;Ac	0.000727
ENSMUSG00000054604	Cggbp1	CGG triplet repeat binding protein 1 [Source:MGI Sy	0.000731
ENSMUSG00000021770	Samd8	sterile alpha motif domain containing 8 [Source:MG	0.000735
ENSMUSG00000001773	Folh1	folate hydrolase 1 [Source:MGI Symbol;Acc:MGI:18	0.000741
ENSMUSG00000057894	Zfp329	zinc finger protein 329 [Source:MGI Symbol;Acc:MG	0.000742
ENSMUSG00000057440	Mpp7	membrane protein, palmitoylated 7 (MAGUK p55 s	0.000747
ENSMUSG00000067377	Tspan6	tetraspanin 6 [Source:MGI Symbol;Acc:MGI:192626	0.000748

ENSMUSG00000097121	D130020L05Rik	RIKEN cDNA D130020L05 gene [Source:MGI Symbo	0.000749
ENSMUSG00000060935	AI597468	expressed sequence AI597468 [Source:MGI Symbol	0.000751
ENSMUSG00000024231	Cul2	cullin 2 [Source:MGI Symbol;Acc:MGI:1918995]	0.000754
ENSMUSG00000036943	Rab8b	RAB8B, member RAS oncogene family [Source:MGI	0.000754
ENSMUSG00000043705	Capn13	calpain 13 [Source:MGI Symbol;Acc:MGI:2685789]	0.000762
ENSMUSG00000090286	Gm17615	predicted gene, 17615 [Source:MGI Symbol;Acc:MG	0.000767
ENSMUSG00000020458	Rtn4	reticulon 4 [Source:MGI Symbol;Acc:MGI:1915835]	0.000795
ENSMUSG00000002881	Nab1	Ngfi-A binding protein 1 [Source:MGI Symbol;Acc:M	0.000795
ENSMUSG00000028647	Mycbp	c-myc binding protein [Source:MGI Symbol;Acc:MG	0.000797
ENSMUSG00000049606	Zfp644	zinc finger protein 644 [Source:MGI Symbol;Acc:MG	0.000806
ENSMUSG00000031433	Rbm41	RNA binding motif protein 41 [Source:MGI Symbol;	0.000806
ENSMUSG00000035476	Tab3	TGF-beta activated kinase 1/MAP3K7 binding prote	0.000806
ENSMUSG00000002728	Naa20	N(alpha)-acetyltransferase 20, NatB catalytic subun	0.000806
ENSMUSG00000041058	Wwp1	WW domain containing E3 ubiquitin protein ligase	0.000813
ENSMUSG00000040818	Dennd6a	DENN/MADD domain containing 6A [Source:MGI Sy	0.000822
ENSMUSG00000048368	Omd	osteomodulin [Source:MGI Symbol;Acc:MGI:13509	0.000839
ENSMUSG00000033809	Alg3	asparagine-linked glycosylation 3 (alpha-1,3-mann	0.00085
ENSMUSG00000027530	Fabp12	fatty acid binding protein 12 [Source:MGI Symbol;A	0.00085
ENSMUSG00000046449	C77370	expressed sequence C77370 [Source:MGI Symbol;A	0.000863
ENSMUSG00000036745	Ttll7	tubulin tyrosine ligase-like family, member 7 [Sourc	0.000867
ENSMUSG00000028233	Tgs1	trimethylguanosine synthase homolog (S. cerevisia	0.000873
ENSMUSG00000035910	Dcdc2a	doublecortin domain containing 2a [Source:MGI Sy	0.000888
ENSMUSG00000055491	Pprc1	peroxisome proliferative activated receptor, gamm	0.00089
ENSMUSG00000077191	Snord64	small nucleolar RNA, C/D box 64 [Source:MGI Symb	0.000891
ENSMUSG00000071862	Lrrtm2	leucine rich repeat transmembrane neuronal 2 [Sou	0.000892
ENSMUSG00000032932	Hspa13	heat shock protein 70 family, member 13 [Source:M	0.000894
ENSMUSG00000029405	G3bp2	GTPase activating protein (SH3 domain) binding pro	0.000904
ENSMUSG00000034382	AI661453	expressed sequence AI661453 [Source:MGI Symbol	0.000916
ENSMUSG00000020053	Igf1	insulin-like growth factor 1 [Source:MGI Symbol;Ac	0.000916
ENSMUSG00000045394	Epcam	epithelial cell adhesion molecule [Source:MGI Symb	0.000918
ENSMUSG00000018008	Cyth4	cytohesin 4 [Source:MGI Symbol;Acc:MGI:2441702	0.000927
ENSMUSG00000037608	Bclaf1	BCL2-associated transcription factor 1 [Source:MGI	0.000929
ENSMUSG00000037857	Nufip2	nuclear fragile X mental retardation protein interac	0.000938
ENSMUSG00000061665	Cd2ap	CD2-associated protein [Source:MGI Symbol;Acc:M	0.000945
ENSMUSG00000056962	Jmjd6	jumonji domain containing 6 [Source:MGI Symbol;A	0.000948
ENSMUSG00000020029	Nudt4	nudix (nucleoside diphosphate linked moiety X)-typ	0.000949
ENSMUSG00000035161	Ints6	integrator complex subunit 6 [Source:MGI Symbol;A	0.000953
ENSMUSG00000034163	Zfc3h1	zinc finger, C3H1-type containing [Source:MGI Sym	0.000954
ENSMUSG00000039782	Cpeb2	cytoplasmic polyadenylation element binding prote	0.000956
ENSMUSG00000031754	Nudt21	nudix (nucleoside diphosphate linked moiety X)-typ	0.000959
ENSMUSG00000031600	Vps37a	vacuolar protein sorting 37A (yeast) [Source:MGI Sy	0.000965
ENSMUSG00000038187	Btbd10	BTB (POZ) domain containing 10 [Source:MGI Symb	0.000965
ENSMUSG00000024500	Ppp2r2b	protein phosphatase 2 (formerly 2A), regulatory su	0.000971
ENSMUSG00000041827	Oasl1	2'-5' oligoadenylate synthetase-like 1 [Source:MGI S	0.000975
ENSMUSG00000020592	Sdc1	syndecan 1 [Source:MGI Symbol;Acc:MGI:1349162]	0.000979
ENSMUSG00000058355	Abce1	ATP-binding cassette, sub-family E (OABP), membe	0.000981
ENSMUSG00000021796	Bmpr1a	bone morphogenetic protein receptor, type 1A [Sou	0.000983
ENSMUSG00000097743	Gm16973	predicted gene, 16973 [Source:MGI Symbol;Acc:MG	0.000983
ENSMUSG00000057092	Fxyd3	FXYD domain-containing ion transport regulator 3 [	0.000986

ENSMUSG00000038510	Rpf2	ribosome production factor 2 homolog (S. cerevisia	0.000992
ENSMUSG00000036480	Prss56	protease, serine 56 [Source:MGI Symbol;Acc:MGI:1	0.000995
ENSMUSG00000021301	Hecw1	HECT, C2 and WW domain containing E3 ubiquitin	0.000998
ENSMUSG00000054178	Gm9938	predicted gene 9938 [Source:MGI Symbol;Acc:MGI:	0.001006
ENSMUSG00000042501	Cpa6	carboxypeptidase A6 [Source:MGI Symbol;Acc:MGI	0.001011
ENSMUSG00000027894	Slc6a17	solute carrier family 6 (neurotransmitter transporte	0.00102
ENSMUSG00000047412	Zbtb44	zinc finger and BTB domain containing 44 [Source:N	0.001026
ENSMUSG00000074093	Svip	small VCP/p97-interacting protein [Source:MGI Syn	0.001027
ENSMUSG00000024261	Syt4	synaptotagmin IV [Source:MGI Symbol;Acc:MGI:10	0.001028
ENSMUSG00000031004	Mki67	antigen identified by monoclonal antibody Ki 67 [Sc	0.00103
ENSMUSG00000043154	Ppp2r3a	protein phosphatase 2, regulatory subunit B'', alpha	0.001031
ENSMUSG00000021123	Rdh12	retinol dehydrogenase 12 [Source:MGI Symbol;Acc	0.001031
ENSMUSG00000068479	Mfap1a	microfibrillar-associated protein 1A [Source:MGI Sy	0.001034
ENSMUSG00000032030	Cul5	cullin 5 [Source:MGI Symbol;Acc:MGI:1922967]	0.001036
ENSMUSG00000084834	4930565N06Rik	RIKEN cDNA 4930565N06 gene [Source:MGI Symbo	0.001036
ENSMUSG00000036790	Slitrk2	SLIT and NTRK-like family, member 2 [Source:MGI S	0.001038
ENSMUSG00000031112	2610018G03Rik	RIKEN cDNA 2610018G03 gene [Source:MGI Symbo	0.001039
ENSMUSG00000023979	Guca1b	guanylate cyclase activator 1B [Source:MGI Symbol	0.001062
ENSMUSG00000031174	Rpgr	retinitis pigmentosa GTPase regulator [Source:MGI	0.001071
ENSMUSG00000086450	Gm14077	predicted gene 14077 [Source:MGI Symbol;Acc:MG	0.001076
ENSMUSG00000036898	Zfp157	zinc finger protein 157 [Source:MGI Symbol;Acc:MG	0.001079
ENSMUSG00000001855	Nup214	nucleoporin 214 [Source:MGI Symbol;Acc:MGI:109	0.001086
ENSMUSG00000089790	Gm16588	predicted gene 16588 [Source:MGI Symbol;Acc:MG	0.001094
ENSMUSG00000047793	Sned1	sushi, nidogen and EGF-like domains 1 [Source:MGI	0.001097
ENSMUSG00000019818	Cd164	CD164 antigen [Source:MGI Symbol;Acc:MGI:18595	0.001098
ENSMUSG00000026830	Ermn	ermin, ERM-like protein [Source:MGI Symbol;Acc:M	0.001098
ENSMUSG00000047213	Ythdf3	YTH domain family 3 [Source:MGI Symbol;Acc:MGI:	0.001101
ENSMUSG00000063887	Nlgn1	neuroligin 1 [Source:MGI Symbol;Acc:MGI:2179435	0.001104
ENSMUSG00000031381	Piga	phosphatidylinositol glycan anchor biosynthesis, cla	0.001109
ENSMUSG00000045903	Npas4	neuronal PAS domain protein 4 [Source:MGI Symbo	0.001122
ENSMUSG00000079436	Kcnj13	potassium inwardly-rectifying channel, subfamily J,	0.001124
ENSMUSG00000015189	Casd1	CAS1 domain containing 1 [Source:MGI Symbol;Acc	0.001136
ENSMUSG00000084989	E030010N08Rik	RIKEN cDNA E030010N08 gene [Source:MGI Symbo	0.001138
ENSMUSG00000029669	Tspan12	tetraspanin 12 [Source:MGI Symbol;Acc:MGI:18898	0.00115
ENSMUSG00000040569	Slc26a7	solute carrier family 26, member 7 [Source:MGI Syr	0.001162
ENSMUSG00000031337	Mtm1	X-linked myotubular myopathy gene 1 [Source:MGI	0.001168
ENSMUSG00000039789	Zfp597	zinc finger protein 597 [Source:MGI Symbol;Acc:MG	0.001169
ENSMUSG00000020251	Glt8d2	glycosyltransferase 8 domain containing 2 [Source:	0.001175
ENSMUSG00000068547	Clca6	chloride channel calcium activated 6 [Source:MGI S	0.001184
ENSMUSG00000097488	4732487G21Rik	RIKEN cDNA 4732487G21 gene [Source:MGI Symbo	0.001196
ENSMUSG00000053453	Thoc7	THO complex 7 homolog (Drosophila) [Source:MGI	0.0012
ENSMUSG00000021749	Oit1	oncoprotein induced transcript 1 [Source:MGI Sym	0.001209
ENSMUSG00000030237	Slco1a4	solute carrier organic anion transporter family, mer	0.001223
ENSMUSG00000044933	Sstr3	somatostatin receptor 3 [Source:MGI Symbol;Acc:N	0.001224
ENSMUSG00000032348	Gsta4	glutathione S-transferase, alpha 4 [Source:MGI Sym	0.001235
ENSMUSG00000086503	Xist	inactive X specific transcripts [Source:MGI Symbol;/	0.001243
ENSMUSG00000000708	Kat2b	K(lysine) acetyltransferase 2B [Source:MGI Symbol;	0.00125
ENSMUSG00000038736	Nudcd1	NudC domain containing 1 [Source:MGI Symbol;Acc	0.00125
ENSMUSG00000020283	Pex13	peroxisomal biogenesis factor 13 [Source:MGI Sym	0.001252

ENSMUSG00000034647	Ankrd12	ankyrin repeat domain 12 [Source:MGI Symbol;Acc	0.001261
ENSMUSG00000037286	Stag1	stromal antigen 1 [Source:MGI Symbol;Acc:MGI:10	0.001262
ENSMUSG00000032643	Fhl3	four and a half LIM domains 3 [Source:MGI Symbol	0.00127
ENSMUSG00000016344	Ppdpf	pancreatic progenitor cell differentiation and prolif	0.001271
ENSMUSG00000090602	Gm5611	predicted gene 5611 [Source:MGI Symbol;Acc:MGI	0.001272
ENSMUSG00000043004	Gng2	guanine nucleotide binding protein (G protein), gar	0.001274
ENSMUSG00000067352	Gm14149	predicted gene 14149 [Source:MGI Symbol;Acc:MG	0.001297
ENSMUSG00000056078	Lipm	lipase, family member M [Source:MGI Symbol;Acc:I	0.001303
ENSMUSG00000037062	Sh3glb1	SH3-domain GRB2-like B1 (endophilin) [Source:MG	0.001307
ENSMUSG00000025592	Dach2	dachshund 2 (Drosophila) [Source:MGI Symbol;Acc	0.00131
ENSMUSG00000028249	Sdcbp	syndecan binding protein [Source:MGI Symbol;Acc:	0.001316
ENSMUSG00000027248	Pdia3	protein disulfide isomerase associated 3 [Source:M	0.001319
ENSMUSG00000034845	Plvap	plasmalemma vesicle associated protein [Source:M	0.00133
ENSMUSG00000027620	Rbm39	RNA binding motif protein 39 [Source:MGI Symbol;	0.001345
ENSMUSG00000035200	Chrnb4	cholinergic receptor, nicotinic, beta polypeptide 4 [	0.001349
ENSMUSG00000036572	Upf3b	UPF3 regulator of nonsense transcripts homolog B	0.001351
ENSMUSG00000097230	Gm26853	predicted gene, 26853 [Source:MGI Symbol;Acc:MG	0.001351
ENSMUSG00000096972	Gm26883	predicted gene, 26883 [Source:MGI Symbol;Acc:MG	0.001356
ENSMUSG00000022899	Slc15a2	solute carrier family 15 (H+/peptide transporter), m	0.001357
ENSMUSG00000046096	BC030336	cDNA sequence BC030336 [Source:MGI Symbol;Acc	0.001359
ENSMUSG00000074292	Gm10660	predicted gene 10660 [Source:MGI Symbol;Acc:MG	0.001359
ENSMUSG00000073176	Zfp449	zinc finger protein 449 [Source:MGI Symbol;Acc:MG	0.001362
ENSMUSG00000074733	5830428H23Rik	RIKEN cDNA 5830428H23 gene [Source:MGI Symbo	0.00137
ENSMUSG00000021537	Cetn3	centrin 3 [Source:MGI Symbol;Acc:MGI:1097706]	0.001376
ENSMUSG00000055963	Triqk	triple QxxK/R motif containing [Source:MGI Symbol	0.001377
ENSMUSG00000021022	Ppp2r3c	protein phosphatase 2, regulatory subunit B'', gam	0.001378
ENSMUSG00000042622	Maff	v-maf musculoaponeurotic fibrosarcoma oncogene	0.001385
ENSMUSG00000035798	Zdhhc17	zinc finger, DHHC domain containing 17 [Source:MG	0.001385
ENSMUSG00000032413	Rasa2	RAS p21 protein activator 2 [Source:MGI Symbol;Ac	0.001396
ENSMUSG00000024727	Trpm6	transient receptor potential cation channel, subfam	0.001396
ENSMUSG00000025232	Hexa	hexosaminidase A [Source:MGI Symbol;Acc:MGI:96	0.001399
ENSMUSG00000028037	Ifi44	interferon-induced protein 44 [Source:MGI Symbol	0.001403
ENSMUSG00000097225	A330076H08Rik	RIKEN cDNA A330076H08 gene [Source:MGI Symbo	0.001406
ENSMUSG00000025094	Slc18a2	solute carrier family 18 (vesicular monoamine), me	0.001411
ENSMUSG00000064346	mt-Tw	mitochondrially encoded tRNA tryptophan [Source:	0.001413
ENSMUSG00000089787	Gm15825	predicted gene 15825 [Source:MGI Symbol;Acc:MG	0.001417
ENSMUSG00000028977	Casz1	castor zinc finger 1 [Source:MGI Symbol;Acc:MGI:1	0.001424
ENSMUSG00000072847	A530017D24Rik	RIKEN cDNA A530017D24 gene [Source:MGI Symbo	0.001426
ENSMUSG00000070808	Gltscr1	glioma tumor suppressor candidate region gene 1 [	0.001426
ENSMUSG00000022594	Lynx1	Ly6/neurotoxin 1 [Source:MGI Symbol;Acc:MGI:134	0.001436
ENSMUSG00000035818	Plekhs1	pleckstrin homology domain containing, family S m	0.001459
ENSMUSG00000031139	Mcf2	mcf.2 transforming sequence [Source:MGI Symbol;	0.001463
ENSMUSG00000021806	Nid2	nidogen 2 [Source:MGI Symbol;Acc:MGI:1298229]	0.001469
ENSMUSG00000039968	Rsbn1l	round spermatid basic protein 1-like [Source:MGI S	0.001473
ENSMUSG00000031245	Hmgn5	high-mobility group nucleosome binding domain 5	0.001476
ENSMUSG00000040710	St8sia4	ST8 alpha-N-acetyl-neuraminide alpha-2,8-sialyltra	0.001478
ENSMUSG00000085084	4930570G19Rik	RIKEN cDNA 4930570G19 gene [Source:MGI Symbo	0.001487
ENSMUSG00000019917	10-Sep	septin 10 [Source:MGI Symbol;Acc:MGI:1918110]	0.001492
ENSMUSG00000016984	Etaa1	Ewing's tumor-associated antigen 1 [Source:MGI Sy	0.001492

ENSMUSG00000040322	Slc25a24	solute carrier family 25 (mitochondrial carrier, phos	0.001495
ENSMUSG00000054693	Adam10	a disintegrin and metallopeptidase domain 10 [Sou	0.001504
ENSMUSG00000022811	Zfp148	zinc finger protein 148 [Source:MGI Symbol;Acc:MG	0.001515
ENSMUSG00000047139	Cd24a	CD24a antigen [Source:MGI Symbol;Acc:MGI:88323	0.001521
ENSMUSG00000040728	Esrp1	epithelial splicing regulatory protein 1 [Source:MGI	0.001525
ENSMUSG00000007908	Hmgcll1	3-hydroxymethyl-3-methylglutaryl-Coenzyme A lya	0.001531
ENSMUSG00000020128	Vps54	vacuolar protein sorting 54 (yeast) [Source:MGI Syr	0.001535
ENSMUSG00000032712	2810474O19Rik	RIKEN cDNA 2810474O19 gene [Source:MGI Symbo	0.00154
ENSMUSG00000021706	Zfyve16	zinc finger, FYVE domain containing 16 [Source:MG	0.001542
ENSMUSG00000028218	Fam92a	family with sequence similarity 92, member A [Sou	0.001552
ENSMUSG00000000605	Clcn4-2	chloride channel 4-2 [Source:MGI Symbol;Acc:MGI:	0.00156
ENSMUSG00000027239	Mdk	midkine [Source:MGI Symbol;Acc:MGI:96949]	0.001564
ENSMUSG00000027287	Snap23	synaptosomal-associated protein 23 [Source:MGI S	0.001583
ENSMUSG00000042410	Agps	alkylglycerone phosphate synthase [Source:MGI Sy	0.001591
ENSMUSG00000074513	Arfip1	ADP-ribosylation factor interacting protein 1 [Sourc	0.001601
ENSMUSG00000057113	Npm1	nucleophosmin 1 [Source:MGI Symbol;Acc:MGI:10	0.00161
ENSMUSG00000026483	Fam129a	family with sequence similarity 129, member A [So	0.001612
ENSMUSG00000029188	Slc34a2	solute carrier family 34 (sodium phosphate), memb	0.001613
ENSMUSG00000074652	Myh7b	myosin, heavy chain 7B, cardiac muscle, beta [Sour	0.001614
ENSMUSG00000085963	Gm15249	predicted gene 15249 [Source:MGI Symbol;Acc:MG	0.001619
ENSMUSG00000031995	St14	suppression of tumorigenicity 14 (colon carcinoma)	0.001621
ENSMUSG00000033760	Rbm4b	RNA binding motif protein 4B [Source:MGI Symbol;	0.001622
ENSMUSG00000037355	Uvssa	UV stimulated scaffold protein A [Source:MGI Symb	0.001627
ENSMUSG00000011179	Odc1	ornithine decarboxylase, structural 1 [Source:MGI S	0.001629
ENSMUSG00000055341	Zfp457	zinc finger protein 457 [Source:MGI Symbol;Acc:MG	0.00164
ENSMUSG00000010608	Rbm25	RNA binding motif protein 25 [Source:MGI Symbol;	0.001664
ENSMUSG00000044768	D1Ertd622e	DNA segment, Chr 1, ERATO Doi 622, expressed [So	0.001664
ENSMUSG00000097151	Gm26514	predicted gene, 26514 [Source:MGI Symbol;Acc:MG	0.001668
ENSMUSG00000005362	Crbn	cereblon [Source:MGI Symbol;Acc:MGI:1913277]	0.001683
ENSMUSG00000049969	Plekhf2	pleckstrin homology domain containing, family F (w	0.001688
ENSMUSG00000022026	Olfm4	olfactomedin 4 [Source:MGI Symbol;Acc:MGI:2685	0.001696
ENSMUSG00000032839	Trpc1	transient receptor potential cation channel, subfam	0.001703
ENSMUSG00000039904	Gpr37	G protein-coupled receptor 37 [Source:MGI Symbo	0.001715
ENSMUSG00000032845	Alpk2	alpha-kinase 2 [Source:MGI Symbol;Acc:MGI:24494	0.001718
ENSMUSG00000038546	Ranbp9	RAN binding protein 9 [Source:MGI Symbol;Acc:MG	0.001723
ENSMUSG00000021596	Mctp1	multiple C2 domains, transmembrane 1 [Source:MG	0.001725
ENSMUSG00000013089	Etv5	ets variant gene 5 [Source:MGI Symbol;Acc:MGI:10	0.001735
ENSMUSG00000002944	Cd36	CD36 antigen [Source:MGI Symbol;Acc:MGI:107899	0.001741
ENSMUSG00000005268	Prlr	prolactin receptor [Source:MGI Symbol;Acc:MGI:97	0.00175
ENSMUSG00000098233	Gm26954	predicted gene, 26954 [Source:MGI Symbol;Acc:MG	0.001759
ENSMUSG00000026502	Desi2	desumoylating isopeptidase 2 [Source:MGI Symbol;	0.001761
ENSMUSG00000074863	Gm5665	predicted gene 5665 [Source:MGI Symbol;Acc:MGI:	0.001761
ENSMUSG00000053012	Krcc1	lysine-rich coiled-coil 1 [Source:MGI Symbol;Acc:MG	0.001782
ENSMUSG00000085453	Gm12018	predicted gene 12018 [Source:MGI Symbol;Acc:MG	0.001783
ENSMUSG00000031493	Ggn	gametogenetin [Source:MGI Symbol;Acc:MGI:2181	0.001784
ENSMUSG00000040452	Cdh12	cadherin 12 [Source:MGI Symbol;Acc:MGI:109503]	0.001787
ENSMUSG00000005649	Cabp5	calcium binding protein 5 [Source:MGI Symbol;Acc:	0.001789
ENSMUSG00000008601	Rab25	RAB25, member RAS oncogene family [Source:MGI	0.00179
ENSMUSG00000047036	Zfp445	zinc finger protein 445 [Source:MGI Symbol;Acc:MG	0.001806

ENSMUSG00000085234	Gm15614	predicted gene 15614 [Source:MGI Symbol;Acc:MG	0.001806
ENSMUSG00000022890	Atp5j	ATP synthase, H+ transporting, mitochondrial F0 co	0.001806
ENSMUSG00000097055	Gm4419	predicted gene 4419 [Source:MGI Symbol;Acc:MGI:	0.001809
ENSMUSG00000069456	Rdh16	retinol dehydrogenase 16 [Source:MGI Symbol;Acc	0.001816
ENSMUSG00000030469	Zfp719	zinc finger protein 719 [Source:MGI Symbol;Acc:MG	0.001819
ENSMUSG00000024757	Slc22a19	solute carrier family 22 (organic anion transporter),	0.001823
ENSMUSG00000095595	Fam177a	family with sequence similarity 177, member A [Sou	0.001824
ENSMUSG00000024143	Rhoq	ras homolog gene family, member Q [Source:MGI S	0.001825
ENSMUSG00000025150	Cbr2	carbonyl reductase 2 [Source:MGI Symbol;Acc:MGI	0.001831
ENSMUSG00000002105	Slc39a13	solute carrier family 39 (metal ion transporter), me	0.001834
ENSMUSG00000050029	Rap2c	RAP2C, member of RAS oncogene family [Source:M	0.001851
ENSMUSG00000028777	Gnat3	guanine nucleotide binding protein, alpha transduc	0.001858
ENSMUSG00000053870	Fpgt	fucose-1-phosphate guanylyltransferase [Source:M	0.001863
ENSMUSG00000027109	Sp3	trans-acting transcription factor 3 [Source:MGI Sym	0.001876
ENSMUSG00000022361	Zhx1	zinc fingers and homeoboxes 1 [Source:MGI Symbo	0.001878
ENSMUSG00000026643	Nmt2	N-myristoyltransferase 2 [Source:MGI Symbol;Acc:I	0.001889
ENSMUSG00000029088	Kcnip4	Kv channel interacting protein 4 [Source:MGI Symb	0.001909
ENSMUSG00000041949	Tango6	transport and golgi organization 6 [Source:MGI Sym	0.001913
ENSMUSG00000022431	Ribc2	RIB43A domain with coiled-coils 2 [Source:MGI Sym	0.001915
ENSMUSG00000083805	Gm14848	predicted gene 14848 [Source:MGI Symbol;Acc:MG	0.001915
ENSMUSG00000051223	Bzw1	basic leucine zipper and W2 domains 1 [Source:MG	0.001923
ENSMUSG00000092072	Gm4540	predicted gene 4540 [Source:MGI Symbol;Acc:MGI:	0.001925
ENSMUSG00000017607	Tns4	tensin 4 [Source:MGI Symbol;Acc:MGI:2144377]	0.001926
ENSMUSG00000001175	Calm1	calmodulin 1 [Source:MGI Symbol;Acc:MGI:88251]	0.00193
ENSMUSG00000020811	Wscd1	WSC domain containing 1 [Source:MGI Symbol;Acc	0.001938
ENSMUSG00000053935	Atf7ip	activating transcription factor 7 interacting protein	0.001956
ENSMUSG00000019802	Sec63	SEC63-like (S. cerevisiae) [Source:MGI Symbol;Acc:I	0.00196
ENSMUSG00000010461	Eya4	eyes absent 4 homolog (Drosophila) [Source:MGI S	0.001971
ENSMUSG00000016921	Srsf6	serine/arginine-rich splicing factor 6 [Source:MGI S	0.001976
ENSMUSG00000069208	Zfp825	zinc finger protein 825 [Source:MGI Symbol;Acc:MG	0.001984
ENSMUSG00000020407	Upp1	uridine phosphorylase 1 [Source:MGI Symbol;Acc:N	0.001985
ENSMUSG00000031590	Frg1	FSHD region gene 1 [Source:MGI Symbol;Acc:MGI:8	0.00199
ENSMUSG00000070544	Top1	topoisomerase (DNA) I [Source:MGI Symbol;Acc:MG	0.001997
ENSMUSG00000013878	Rnf170	ring finger protein 170 [Source:MGI Symbol;Acc:MG	0.002008
ENSMUSG00000039770	Ypel5	yippee-like 5 (Drosophila) [Source:MGI Symbol;Acc	0.002011
ENSMUSG00000027882	Stxbp3a	syntaxin binding protein 3A [Source:MGI Symbol;Ac	0.002022
ENSMUSG00000079605	Zbtb9	zinc finger and BTB domain containing 9 [Source:M	0.002048
ENSMUSG00000049353	Rd3	retinal degeneration 3 [Source:MGI Symbol;Acc:MG	0.002051
ENSMUSG00000087354	4930404I05Rik	RIKEN cDNA 4930404I05 gene [Source:MGI Symbol	0.002052
ENSMUSG00000079450	3110007F17Rik	RIKEN cDNA 3110007F17 gene [Source:MGI Symbo	0.002056
ENSMUSG00000032727	Mier3	mesoderm induction early response 1, family mem	0.002078
ENSMUSG00000031093	Dock11	dedicator of cytokinesis 11 [Source:MGI Symbol;Ac	0.002079
ENSMUSG00000090100	Ttbk2	tau tubulin kinase 2 [Source:MGI Symbol;Acc:MGI:2	0.002086
ENSMUSG00000062901	Klhl24	kelch-like 24 [Source:MGI Symbol;Acc:MGI:192303	0.002091
ENSMUSG00000087694	A530058N18Rik	RIKEN cDNA A530058N18 gene [Source:MGI Symbo	0.002105
ENSMUSG00000098063	Gm26930	predicted gene, 26930 [Source:MGI Symbol;Acc:MG	0.002127
ENSMUSG00000073448	Gm10509	predicted gene 10509 [Source:MGI Symbol;Acc:MG	0.002152
ENSMUSG00000027961	Lrrc39	leucine rich repeat containing 39 [Source:MGI Sym	0.002153
ENSMUSG00000082568	Gm14683	predicted gene 14683 [Source:MGI Symbol;Acc:MG	0.002162

ENSMUSG00000065126	Snord104	small nucleolar RNA, C/D box 104 [Source:MGI Sym	0.002162
ENSMUSG00000026771	Spopl	speckle-type POZ protein-like [Source:MGI Symbol;	0.002167
ENSMUSG00000062604	Srpk2	serine/arginine-rich protein specific kinase 2 [Sourc	0.002167
ENSMUSG00000038646	Fam103a1	family with sequence similarity 103, member A1 [S	0.002167
ENSMUSG00000047216	Cdh19	cadherin 19, type 2 [Source:MGI Symbol;Acc:MGI:3	0.002186
ENSMUSG00000074264	Amy1	amylase 1, salivary [Source:MGI Symbol;Acc:MGI:8	0.002188
ENSMUSG00000022329	Stk3	serine/threonine kinase 3 [Source:MGI Symbol;Acc	0.002196
ENSMUSG00000023845	Lnpep	leucyl/cystinyl aminopeptidase [Source:MGI Symbo	0.002205
ENSMUSG00000027167	Elp4	elongation protein 4 homolog (S. cerevisiae) [Sourc	0.002209
ENSMUSG00000078963	Hsbp1l1	heat shock factor binding protein 1-like 1 [Source:N	0.002223
ENSMUSG00000024583	Txnl1	thioredoxin-like 1 [Source:MGI Symbol;Acc:MGI:18	0.002231
ENSMUSG00000074865	Zfp934	zinc finger protein 934 [Source:MGI Symbol;Acc:MG	0.002234
ENSMUSG00000054099	Slc25a40	solute carrier family 25, member 40 [Source:MGI Sy	0.002237
ENSMUSG00000074273	Gm10657	predicted gene 10657 [Source:MGI Symbol;Acc:MG	0.002262
ENSMUSG00000062376	2010012O05Rik	RIKEN cDNA 2010012O05 gene [Source:MGI Symbo	0.002268
ENSMUSG00000068101	Cenpm	centromere protein M [Source:MGI Symbol;Acc:MG	0.002274
ENSMUSG00000028207	Asph	aspartate-beta-hydroxylase [Source:MGI Symbol;Ac	0.002274
ENSMUSG00000021824	Ap3m1	adaptor-related protein complex 3, mu 1 subunit [S	0.002287
ENSMUSG00000039809	Gabbr2	gamma-aminobutyric acid (GABA) B receptor, 2 [So	0.002288
ENSMUSG00000070803	Cited4	Cbp/p300-interacting transactivator, with Glu/Asp-	0.002302
ENSMUSG00000021218	Gdi2	guanosine diphosphate (GDP) dissociation inhibitor	0.002313
ENSMUSG00000030057	Cnbp	cellular nucleic acid binding protein [Source:MGI Sy	0.002316
ENSMUSG00000041559	Fmod	fibromodulin [Source:MGI Symbol;Acc:MGI:132836	0.002318
ENSMUSG00000047446	Arl4a	ADP-ribosylation factor-like 4A [Source:MGI Symbo	0.00232
ENSMUSG00000091045	Vmn2r55	vomeronasal 2, receptor 55 [Source:MGI Symbol;Ac	0.002325
ENSMUSG00000021112	Mpp5	membrane protein, palmitoylated 5 (MAGUK p55 s	0.002335
ENSMUSG00000033721	Vav3	vav 3 oncogene [Source:MGI Symbol;Acc:MGI:1888	0.002339
ENSMUSG00000022831	Hcls1	hematopoietic cell specific Lyn substrate 1 [Source:	0.002348
ENSMUSG00000026399	Cd55	CD55 antigen [Source:MGI Symbol;Acc:MGI:104850	0.002351
ENSMUSG00000034780	B3galt1	UDP-Gal:betaGlcNAc beta 1,3-galactosyltransferase	0.002357
ENSMUSG00000042250	Pglyrp4	peptidoglycan recognition protein 4 [Source:MGI Sy	0.002359
ENSMUSG00000021690	Jmy	junction-mediating and regulatory protein [Source:	0.002361
ENSMUSG00000005225	Plekha8	pleckstrin homology domain containing, family A (p	0.002365
ENSMUSG00000009907	Vps4b	vacuolar protein sorting 4b (yeast) [Source:MGI Syr	0.002373
ENSMUSG00000045519	Zfp560	zinc finger protein 560 [Source:MGI Symbol;Acc:MG	0.002376
ENSMUSG00000027346	Gpcpd1	glycerophosphocholine phosphodiesterase GDE1 h	0.002377
ENSMUSG00000054976	Nyap2	neuronal tyrosine-phophorylated phosphoinositide	0.00238
ENSMUSG00000015134	Aldh1a3	aldehyde dehydrogenase family 1, subfamily A3 [So	0.002407
ENSMUSG00000072501	Phf20l1	PHD finger protein 20-like 1 [Source:MGI Symbol;A	0.002413
ENSMUSG00000090078	Gm16147	predicted gene 16147 [Source:MGI Symbol;Acc:MG	0.002418
ENSMUSG00000040331	Nsmce4a	non-SMC element 4 homolog A (S. cerevisiae) [Sour	0.00242
ENSMUSG00000040760	Appl1	adaptor protein, phosphotyrosine interaction, PH d	0.002432
ENSMUSG00000020677	Ddx52	DEAD (Asp-Glu-Ala-Asp) box polypeptide 52 [Sourc	0.002433
ENSMUSG00000017344	Vtn	vitronectin [Source:MGI Symbol;Acc:MGI:98940]	0.002435
ENSMUSG00000084421	Gm25107	predicted gene, 25107 [Source:MGI Symbol;Acc:MG	0.002438
ENSMUSG00000028637	Ccdc30	coiled-coil domain containing 30 [Source:MGI Symb	0.002438
ENSMUSG00000024827	Gldc	glycine decarboxylase [Source:MGI Symbol;Acc:MG	0.002447
ENSMUSG00000060180	Myh13	myosin, heavy polypeptide 13, skeletal muscle [Sou	0.002454
ENSMUSG00000085685	Gm15253	predicted gene 15253 [Source:MGI Symbol;Acc:MG	0.002455

ENSMUSG00000017776	Crk	v-crk sarcoma virus CT10 oncogene homolog (avian	0.002456
ENSMUSG00000035557	Krt17	keratin 17 [Source:MGI Symbol;Acc:MGI:96691]	0.002457
ENSMUSG00000072437	Nanos1	nanos homolog 1 (Drosophila) [Source:MGI Symbol	0.002459
ENSMUSG00000047037	Nipa1	non imprinted in Prader-Willi/Angelman syndrome	0.002465
ENSMUSG00000035964	Tmem59l	transmembrane protein 59-like [Source:MGI Symbo	0.002492
ENSMUSG00000041328	Pcf11	cleavage and polyadenylation factor subunit homol	0.0025
ENSMUSG00000009575	Cbx5	chromobox 5 [Source:MGI Symbol;Acc:MGI:109372	0.002505
ENSMUSG00000072763	5430403G16Rik	RIKEN cDNA 5430403G16 gene [Source:MGI Symbo	0.002514
ENSMUSG00000019894	Slc6a15	solute carrier family 6 (neurotransmitter transporte	0.002522
ENSMUSG00000049422	Chchd10	coiled-coil-helix-coiled-coil-helix domain containing	0.002525
ENSMUSG00000038602	Slc35f1	solute carrier family 35, member F1 [Source:MGI Sy	0.002527
ENSMUSG00000027957	Slc35a3	solute carrier family 35 (UDP-N-acetylglucosamine	0.002531
ENSMUSG00000003948	Mmd	monocyte to macrophage differentiation-associate	0.002534
ENSMUSG00000096910	Zfp955b	zinc finger protein 955B [Source:MGI Symbol;Acc:M	0.002541
ENSMUSG00000059742	Kcnh7	potassium voltage-gated channel, subfamily H (eag	0.002546
ENSMUSG00000047227	Gm527	predicted gene 527 [Source:MGI Symbol;Acc:MGI:2	0.002551
ENSMUSG00000024054	Smchd1	SMC hinge domain containing 1 [Source:MGI Symb	0.002554
ENSMUSG00000055436	Srsf11	serine/arginine-rich splicing factor 11 [Source:MGI	0.002555
ENSMUSG00000060475	Wtap	Wilms' tumour 1-associating protein [Source:MGI S	0.002576
ENSMUSG00000024294	Mib1	mindbomb homolog 1 (Drosophila) [Source:MGI Sy	0.00258
ENSMUSG00000034320	Slc26a2	solute carrier family 26 (sulfate transporter), memb	0.002586
ENSMUSG00000027398	Il1b	interleukin 1 beta [Source:MGI Symbol;Acc:MGI:96	0.002606
ENSMUSG00000052479	A330008L17Rik	RIKEN cDNA A330008L17 gene [Source:MGI Symbo	0.002623
ENSMUSG00000070003	Ssbp4	single stranded DNA binding protein 4 [Source:MGI	0.002645
ENSMUSG00000071855	Ccdc112	coiled-coil domain containing 112 [Source:MGI Sym	0.002655
ENSMUSG00000061544	Zfp229	zinc finger protein 229 [Source:MGI Symbol;Acc:MG	0.002658
ENSMUSG00000027193	Api5	apoptosis inhibitor 5 [Source:MGI Symbol;Acc:MGI:	0.002661
ENSMUSG00000021546	Hnrnpk	heterogeneous nuclear ribonucleoprotein K [Source	0.00267
ENSMUSG00000035967	Ddx26b	DEAD/H (Asp-Glu-Ala-Asp/His) box polypeptide 26B	0.002672
ENSMUSG00000057614	Gnai1	guanine nucleotide binding protein (G protein), alp	0.002677
ENSMUSG00000060771	Tsga10	testis specific 10 [Source:MGI Symbol;Acc:MGI:268	0.002678
ENSMUSG00000057409	Zfp53	zinc finger protein 53 [Source:MGI Symbol;Acc:MGI	0.002692
ENSMUSG00000024943	Smc5	structural maintenance of chromosomes 5 [Source:	0.002699
ENSMUSG00000071291	Zfp58	zinc finger protein 58 [Source:MGI Symbol;Acc:MGI	0.002702
ENSMUSG00000022299	Slc25a32	solute carrier family 25, member 32 [Source:MGI Sy	0.002703
ENSMUSG00000047161	Chst9	carbohydrate (N-acetylgalactosamine 4-0) sulfotran	0.002703
ENSMUSG00000030108	Slc6a13	solute carrier family 6 (neurotransmitter transporte	0.002708
ENSMUSG00000037458	Azin1	antizyme inhibitor 1 [Source:MGI Symbol;Acc:MGI:	0.00271
ENSMUSG00000074170	Plekhf1	pleckstrin homology domain containing, family F (w	0.002711
ENSMUSG00000028132	Tmem56	transmembrane protein 56 [Source:MGI Symbol;Ac	0.002715
ENSMUSG00000028496	Mllt3	myeloid/lymphoid or mixed-lineage leukemia (trith	0.002732
ENSMUSG00000036499	Eea1	early endosome antigen 1 [Source:MGI Symbol;Acc	0.002739
ENSMUSG00000045427	Hnrnph2	heterogeneous nuclear ribonucleoprotein H2 [Sour	0.002752
ENSMUSG00000035992	Fnip1	folliculin interacting protein 1 [Source:MGI Symbol;	0.002754
ENSMUSG00000048355	Arxes1	adipocyte-related X-chromosome expressed seque	0.002779
ENSMUSG00000041782	Lad1	ladinin [Source:MGI Symbol;Acc:MGI:109343]	0.002793
ENSMUSG00000052525	Spdya	speedy homolog A (Xenopus laevis) [Source:MGI Sy	0.002795
ENSMUSG00000025912	Mybl1	myeloblastosis oncogene-like 1 [Source:MGI Symbo	0.002805
ENSMUSG00000089857	Zfp882	zinc finger protein 882 [Source:MGI Symbol;Acc:MG	0.00284

ENSMUSG00000053279	Aldh1a1	aldehyde dehydrogenase family 1, subfamily A1 [So	0.002842
ENSMUSG00000072774	Zfp951	zinc finger protein 951 [Source:MGI Symbol;Acc:MG	0.002849
ENSMUSG00000022122	Ednrb	endothelin receptor type B [Source:MGI Symbol;Ac	0.002859
ENSMUSG00000035329	Fbxo33	F-box protein 33 [Source:MGI Symbol;Acc:MGI:191	0.002885
ENSMUSG00000056673	Kdm5d	lysine (K)-specific demethylase 5D [Source:MGI Sym	0.002888
ENSMUSG00000029265	Dr1	down-regulator of transcription 1 [Source:MGI Sym	0.002922
ENSMUSG00000051674	Dcun1d4	DCN1, defective in cullin neddylation 1, domain cor	0.002924
ENSMUSG00000039285	Azi2	5-azacytidine induced gene 2 [Source:MGI Symbol;A	0.002928
ENSMUSG00000089804	Gm16136	predicted gene 16136 [Source:MGI Symbol;Acc:MG	0.002945
ENSMUSG00000027660	Skil	SKI-like [Source:MGI Symbol;Acc:MGI:106203]	0.002945
ENSMUSG00000089296	Gm23205	predicted gene, 23205 [Source:MGI Symbol;Acc:MG	0.002945
ENSMUSG00000051379	Flrt3	fibronectin leucine rich transmembrane protein 3 [S	0.002963
ENSMUSG00000057387	4922502B01Rik	RIKEN cDNA 4922502B01 gene [Source:MGI Symbo	0.00297
ENSMUSG00000026822	Lcn2	lipocalin 2 [Source:MGI Symbol;Acc:MGI:96757]	0.002972
ENSMUSG00000064361	mt-Tr	mitochondrially encoded tRNA arginine [Source:MG	0.002973
ENSMUSG00000055240	Zfp101	zinc finger protein 101 [Source:MGI Symbol;Acc:MG	0.002976
ENSMUSG00000021280	Exoc3l4	exocyst complex component 3-like 4 [Source:MGI S	0.002978
ENSMUSG00000022307	Oxr1	oxidation resistance 1 [Source:MGI Symbol;Acc:MG	0.002994
ENSMUSG00000040423	Rc3h1	RING CCCH (C3H) domains 1 [Source:MGI Symbol;A	0.003004
ENSMUSG00000036099	Vezt	vezatin, adherens junctions transmembrane protei	0.003012
ENSMUSG00000091537	Tma7	translational machinery associated 7 homolog (S. ce	0.003022
ENSMUSG00000024576	Csnk1a1	casein kinase 1, alpha 1 [Source:MGI Symbol;Acc:M	0.003033
ENSMUSG00000024806	Mlana	melan-A [Source:MGI Symbol;Acc:MGI:108454]	0.003034
ENSMUSG00000002983	Relb	avian reticuloendotheliosis viral (v-rel) oncogene re	0.003038
ENSMUSG00000021214	Akr1c18	aldo-keto reductase family 1, member C18 [Source:	0.003066
ENSMUSG00000033910	Gucy1a3	guanylate cyclase 1, soluble, alpha 3 [Source:MGI S	0.003096
ENSMUSG00000074674	Gm10742	predicted gene 10742 [Source:MGI Symbol;Acc:MG	0.003107
ENSMUSG00000066415	Msl2	male-specific lethal 2 homolog (Drosophila) [Source	0.003108
ENSMUSG00000041571	Sepw1	selenoprotein W, muscle 1 [Source:MGI Symbol;Ac	0.003109
ENSMUSG00000022261	Sdc2	syndecan 2 [Source:MGI Symbol;Acc:MGI:1349165]	0.003112
ENSMUSG00000097589	Dleu2	deleted in lymphocytic leukemia, 2 [Source:MGI Sy	0.003115
ENSMUSG00000051375	Pcdh1	protocadherin 1 [Source:MGI Symbol;Acc:MGI:104	0.003129
ENSMUSG00000049321	Zfp2	zinc finger protein 2 [Source:MGI Symbol;Acc:MGI:	0.003141
ENSMUSG00000034472	Rasd2	RASD family, member 2 [Source:MGI Symbol;Acc:M	0.003143
ENSMUSG00000024091	Vapa	vesicle-associated membrane protein, associated p	0.003148
ENSMUSG00000041817	Fam169a	family with sequence similarity 169, member A [Sou	0.003153
ENSMUSG00000037610	Kcnmb2	potassium large conductance calcium-activated cha	0.003157
ENSMUSG00000045591	Olig3	oligodendrocyte transcription factor 3 [Source:MGI	0.003165
ENSMUSG00000041120	Nbl1	neuroblastoma, suppression of tumorigenicity 1 [So	0.003175
ENSMUSG00000043463	Rab9b	RAB9B, member RAS oncogene family [Source:MGI	0.003178
ENSMUSG00000036769	Wdr44	WD repeat domain 44 [Source:MGI Symbol;Acc:MG	0.003191
ENSMUSG00000026767	Fam188a	family with sequence similarity 188, member A [Sou	0.003203
ENSMUSG00000063382	Bcl9l	B cell CLL/lymphoma 9-like [Source:MGI Symbol;Ac	0.003228
ENSMUSG00000043557	Mdga1	MAM domain containing glycosylphosphatidylinosi	0.003229
ENSMUSG00000051154	Commd3	COMM domain containing 3 [Source:MGI Symbol;A	0.003234
ENSMUSG00000009670	Tex11	testis expressed gene 11 [Source:MGI Symbol;Acc:M	0.003245
ENSMUSG00000042197	Zfp451	zinc finger protein 451 [Source:MGI Symbol;Acc:MG	0.003249
ENSMUSG00000026020	Nop58	NOP58 ribonucleoprotein [Source:MGI Symbol;Acc	0.003267

ENSMUSG00000047368	Abhd17b	abhydrolase domain containing 17B [Source:MGI Sy	0.003285
ENSMUSG00000026639	Lamb3	laminin, beta 3 [Source:MGI Symbol;Acc:MGI:9991	0.003293
ENSMUSG00000019373	Cops3	COP9 (constitutive photomorphogenic) homolog, s	0.003307
ENSMUSG00000059588	Calcrl	calcitonin receptor-like [Source:MGI Symbol;Acc:M	0.003313
ENSMUSG00000037126	Psd	pleckstrin and Sec7 domain containing [Source:MG	0.003321
ENSMUSG00000041483	Zfp281	zinc finger protein 281 [Source:MGI Symbol;Acc:M	0.003331
ENSMUSG00000090386	2810055G20Rik	RIKEN cDNA 2810055G20 gene [Source:MGI Symbo	0.003346
ENSMUSG00000085900	A930041C12Rik	RIKEN cDNA A930041C12 gene [Source:MGI Symbo	0.003361
ENSMUSG00000037492	Zmat4	zinc finger, matrin type 4 [Source:MGI Symbol;Acc:	0.003366
ENSMUSG00000022228	Zscan26	zinc finger and SCAN domain containing 26 [Source	0.003386
ENSMUSG00000053293	Pom121	nuclear pore membrane protein 121 [Source:MGI S	0.003395
ENSMUSG00000038936	Sccpdh	saccharopine dehydrogenase (putative) [Source:M	0.003398
ENSMUSG00000007653	Gabrb2	gamma-aminobutyric acid (GABA) A receptor, subu	0.003401
ENSMUSG00000039220	Ppp1r10	protein phosphatase 1, regulatory subunit 10 [Sour	0.003419
ENSMUSG00000049225	Pdp1	pyruvate dehyrogenase phosphatase catalytic subu	0.00342
ENSMUSG00000048388	Fam171b	family with sequence similarity 171, member B [Sou	0.003443
ENSMUSG00000037999	Arap2	ArfGAP with RhoGAP domain, ankyrin repeat and P	0.003443
ENSMUSG00000028291	Akirin2	akirin 2 [Source:MGI Symbol;Acc:MGI:1889364]	0.003454
ENSMUSG00000032517	Mobp	myelin-associated oligodendrocytic basic protein [S	0.003473
ENSMUSG00000089726	Mir17hg	Mir17 host gene 1 (non-protein coding) [Source:M	0.00348
ENSMUSG00000031828	Klhl36	kelch-like 36 [Source:MGI Symbol;Acc:MGI:238530	0.003508
ENSMUSG00000021567	Nkd2	naked cuticle 2 homolog (Drosophila) [Source:MGI	0.003516
ENSMUSG00000022253	Nadk2	NAD kinase 2, mitochondrial [Source:MGI Symbol;A	0.003519
ENSMUSG00000048379	Socs4	suppressor of cytokine signaling 4 [Source:MGI Sym	0.003524
ENSMUSG00000025759	Mfsd8	major facilitator superfamily domain containing 8 [	0.003533
ENSMUSG00000022076	Klhl1	kelch-like 1 [Source:MGI Symbol;Acc:MGI:2136335	0.00354
ENSMUSG00000003131	Pafah1b2	platelet-activating factor acetylhydrolase, isoform 1	0.003557
ENSMUSG00000040339	Fam102b	family with sequence similarity 102, member B [Sou	0.003565
ENSMUSG00000050697	Prkaa1	protein kinase, AMP-activated, alpha 1 catalytic sub	0.003577
ENSMUSG00000031703	Itfg1	integrin alpha FG-GAP repeat containing 1 [Source:	0.003583
ENSMUSG00000014353	Tmem87b	transmembrane protein 87B [Source:MGI Symbol;A	0.003604
ENSMUSG00000026904	Slc4a10	solute carrier family 4, sodium bicarbonate cotrans	0.003609
ENSMUSG00000049232	Tigd2	tigger transposable element derived 2 [Source:MGI	0.003611
ENSMUSG00000028568	Btf3l4	basic transcription factor 3-like 4 [Source:MGI Sym	0.003622
ENSMUSG00000079523	Tmsb10	thymosin, beta 10 [Source:MGI Symbol;Acc:MGI:10	0.003624
ENSMUSG00000056153	Socs6	suppressor of cytokine signaling 6 [Source:MGI Sym	0.003646
ENSMUSG00000020863	Luc7l3	LUC7-like 3 (S. cerevisiae) [Source:MGI Symbol;Acc:	0.00368
ENSMUSG00000027180	Fbxo3	F-box protein 3 [Source:MGI Symbol;Acc:MGI:1929	0.00369
ENSMUSG00000033454	Zbtb1	zinc finger and BTB domain containing 1 [Source:M	0.003704
ENSMUSG00000032324	Tspan3	tetraspanin 3 [Source:MGI Symbol;Acc:MGI:192809	0.00371
ENSMUSG00000068250	Amn1	antagonist of mitotic exit network 1 [Source:MGI Sy	0.003725
ENSMUSG00000037318	Traf3ip3	TRAF3 interacting protein 3 [Source:MGI Symbol;Ac	0.003729
ENSMUSG00000052372	Il1rapl1	interleukin 1 receptor accessory protein-like 1 [Sou	0.003733
ENSMUSG00000036242	3632451O06Rik	RIKEN cDNA 3632451O06 gene [Source:MGI Symbo	0.003736
ENSMUSG00000090498	Kcnb2	potassium voltage gated channel, Shab-related sub	0.003741
ENSMUSG00000040170	Fmo2	flavin containing monooxygenase 2 [Source:MGI Sy	0.003741
ENSMUSG00000036990	Otud4	OTU domain containing 4 [Source:MGI Symbol;Acc:	0.003747

ENSMUSG00000026781	Acbd5	acyl-Coenzyme A binding domain containing 5 [Sou	0.003774
ENSMUSG00000029569	Tmem168	transmembrane protein 168 [Source:MGI Symbol;A	0.003782
ENSMUSG00000024027	Glp1r	glucagon-like peptide 1 receptor [Source:MGI Symb	0.003815
ENSMUSG00000051098	Mblac2	metallo-beta-lactamase domain containing 2 [Sourc	0.003816
ENSMUSG00000019998	Stx7	syntaxin 7 [Source:MGI Symbol;Acc:MGI:1858210]	0.003831
ENSMUSG00000070644	Etnk2	ethanolamine kinase 2 [Source:MGI Symbol;Acc:MG	0.003833
ENSMUSG00000029390	Tmed2	transmembrane emp24 domain trafficking protein	0.003835
ENSMUSG00000097660	Gm26762	predicted gene, 26762 [Source:MGI Symbol;Acc:MG	0.003848
ENSMUSG00000026424	Gpr37l1	G protein-coupled receptor 37-like 1 [Source:MGI S	0.003854
ENSMUSG00000094870	Zfp131	zinc finger protein 131 [Source:MGI Symbol;Acc:MG	0.003855
ENSMUSG00000039361	Picalm	phosphatidylinositol binding clathrin assembly prot	0.003871
ENSMUSG00000030029	Lrig1	leucine-rich repeats and immunoglobulin-like doma	0.003875
ENSMUSG00000053226	Dand5	DAN domain family, member 5 [Source:MGI Symbo	0.003887
ENSMUSG00000015002	Efr3a	EFR3 homolog A (S. cerevisiae) [Source:MGI Symbo	0.003894
ENSMUSG00000051149	Adnp	activity-dependent neuroprotective protein [Source	0.003923
ENSMUSG00000038039	Gcc2	GRIP and coiled-coil domain containing 2 [Source:M	0.003948
ENSMUSG00000027122	Arl14ep	ADP-ribosylation factor-like 14 effector protein [Sou	0.003953
ENSMUSG00000031239	Itm2a	integral membrane protein 2A [Source:MGI Symbo	0.003954
ENSMUSG00000011034	Slc5a1	solute carrier family 5 (sodium/glucose cotransport	0.003957
ENSMUSG00000038292	Ccdc155	coiled-coil domain containing 155 [Source:MGI Sym	0.003966
ENSMUSG00000048416	Mlf1	myeloid leukemia factor 1 [Source:MGI Symbol;Acc	0.003972
ENSMUSG00000054519	Zfp867	zinc finger protein 867 [Source:MGI Symbol;Acc:MG	0.003976
ENSMUSG00000059857	Ntng1	netrin G1 [Source:MGI Symbol;Acc:MGI:1934028]	0.003979
ENSMUSG00000089842	Gm16338	predicted gene 16338 [Source:MGI Symbol;Acc:MG	0.003983
ENSMUSG00000022024	Sugt1	SGT1, suppressor of G2 allele of SKP1 (S. cerevisiae	0.004008
ENSMUSG00000028788	Ptp4a2	protein tyrosine phosphatase 4a2 [Source:MGI Sym	0.004023
ENSMUSG00000044201	Cdc25c	cell division cycle 25C [Source:MGI Symbol;Acc:MG	0.004026
ENSMUSG00000038147	Cd84	CD84 antigen [Source:MGI Symbol;Acc:MGI:133688	0.004037
ENSMUSG00000067297	2010002M12Rik	RIKEN cDNA 2010002M12 gene [Source:MGI Symbo	0.004037
ENSMUSG00000032551	1110059G10Rik	RIKEN cDNA 1110059G10 gene [Source:MGI Symbo	0.004043
ENSMUSG00000028563	Tm2d1	TM2 domain containing 1 [Source:MGI Symbol;Acc	0.004044
ENSMUSG00000054065	Pkp3	plakophilin 3 [Source:MGI Symbol;Acc:MGI:189183	0.004081
ENSMUSG00000029406	Pitpnm2	phosphatidylinositol transfer protein, membrane-a	0.004109
ENSMUSG00000034484	Snx2	sorting nexin 2 [Source:MGI Symbol;Acc:MGI:19150	0.004115
ENSMUSG00000027285	Haus2	HAUS augmin-like complex, subunit 2 [Source:MGI	0.004124
ENSMUSG00000018830	Myh11	myosin, heavy polypeptide 11, smooth muscle [Sou	0.004125
ENSMUSG00000086228	Gm514	predicted gene 514 [Source:MGI Symbol;Acc:MGI:2	0.004126
ENSMUSG00000085743	8430419K02Rik	RIKEN cDNA 8430419K02 gene [Source:MGI Symbo	0.004184
ENSMUSG00000017400	Stac2	SH3 and cysteine rich domain 2 [Source:MGI Symbo	0.004207
ENSMUSG00000026866	Kynu	kynureninase (L-kynurenine hydrolase) [Source:MG	0.004208
ENSMUSG00000045871	Slitrk6	SLIT and NTRK-like family, member 6 [Source:MGI S	0.004213
ENSMUSG00000040359	Ufl1	UFM1 specific ligase 1 [Source:MGI Symbol;Acc:MG	0.004214
ENSMUSG00000028256	Odf2l	outer dense fiber of sperm tails 2-like [Source:MGI	0.004216
ENSMUSG00000025350	Rdh5	retinol dehydrogenase 5 [Source:MGI Symbol;Acc:M	0.00422
ENSMUSG00000027199	Gatm	glycine amidinotransferase (L-arginine:glycine amid	0.004223
ENSMUSG00000020102	Slc16a7	solute carrier family 16 (monocarboxylic acid transp	0.004255
ENSMUSG00000040396	Abhd13	abhydrolase domain containing 13 [Source:MGI Syn	0.004261

ENSMUSG00000085139	A730046J19Rik	RIKEN cDNA A730046J19 gene [Source:MGI Symbo	0.004262
ENSMUSG00000020064	Herc4	hect domain and RLD 4 [Source:MGI Symbol;Acc:M	0.004264
ENSMUSG00000078773	Rad54b	RAD54 homolog B (S. cerevisiae) [Source:MGI Symb	0.004268
ENSMUSG00000023068	Nus1	nuclear undecaprenyl pyrophosphate synthase 1 ho	0.004278
ENSMUSG00000060445	Sycp2	synaptonemal complex protein 2 [Source:MGI Sym	0.004282
ENSMUSG00000016833	Mrps18c	mitochondrial ribosomal protein S18C [Source:MGI	0.004287
ENSMUSG00000000581	C1d	C1D nuclear receptor co-repressor [Source:MGI Syr	0.004288
ENSMUSG00000086022	Rad51ap2	RAD51 associated protein 2 [Source:MGI Symbol;A	0.004291
ENSMUSG00000025437	Usp33	ubiquitin specific peptidase 33 [Source:MGI Symbo	0.004308
ENSMUSG00000035234	Fam175a	family with sequence similarity 175, member A [So	0.004311
ENSMUSG00000060467	Gm10080	predicted gene 10080 [Source:MGI Symbol;Acc:MG	0.004313
ENSMUSG00000030291	Med21	mediator complex subunit 21 [Source:MGI Symbol;	0.00435
ENSMUSG00000045493	Bhlhe23	basic helix-loop-helix family, member e23 [Source:I	0.004354
ENSMUSG00000039585	Myo9a	myosin IXa [Source:MGI Symbol;Acc:MGI:107735]	0.004359
ENSMUSG00000033906	Zdhhc15	zinc finger, DHHC domain containing 15 [Source:M	0.004367
ENSMUSG00000068614	Actc1	actin, alpha, cardiac muscle 1 [Source:MGI Symbol;	0.004379
ENSMUSG00000059447	Hadhb	hydroxyacyl-Coenzyme A dehydrogenase/3-ketoac	0.004381
ENSMUSG00000022661	Cd200	CD200 antigen [Source:MGI Symbol;Acc:MGI:1196	0.004402
ENSMUSG00000054162	Spock3	sparc/osteonectin, cwcv and kazal-like domains pro	0.004411
ENSMUSG00000085307	Gm11525	predicted gene 11525 [Source:MGI Symbol;Acc:MG	0.00442
ENSMUSG00000019817	Plagl1	pleiomorphic adenoma gene-like 1 [Source:MGI Sy	0.004428
ENSMUSG00000032454	Rbp2	retinol binding protein 2, cellular [Source:MGI Sym	0.004435
ENSMUSG00000026107	Nabp1	nucleic acid binding protein 1 [Source:MGI Symbol;	0.00444
ENSMUSG00000035504	Reep6	receptor accessory protein 6 [Source:MGI Symbol;A	0.004478
ENSMUSG00000024068	Spast	spastin [Source:MGI Symbol;Acc:MGI:1858896]	0.004484
ENSMUSG00000072683	Gm7457	predicted gene 7457 [Source:MGI Symbol;Acc:MGI:	0.004495
ENSMUSG00000032621	Srek1	splicing regulatory glutamine/lysine-rich protein 1 [	0.004498
ENSMUSG00000054792	Klhl18	kelch-like 18 [Source:MGI Symbol;Acc:MGI:214331	0.004512
ENSMUSG00000057766	Ankrd29	ankyrin repeat domain 29 [Source:MGI Symbol;Acc	0.004534
ENSMUSG00000084883	Ccdc85c	coiled-coil domain containing 85C [Source:MGI Sym	0.004558
ENSMUSG00000028698	Pik3r3	phosphatidylinositol 3 kinase, regulatory subunit, p	0.004595
ENSMUSG00000090958	Lrrc32	leucine rich repeat containing 32 [Source:MGI Sym	0.004615
ENSMUSG00000035946	Gsx2	GS homeobox 2 [Source:MGI Symbol;Acc:MGI:9584	0.004648
ENSMUSG00000040653	Ppp1r14c	protein phosphatase 1, regulatory (inhibitor) subun	0.004658
ENSMUSG00000025040	Fundc1	FUN14 domain containing 1 [Source:MGI Symbol;A	0.004666
ENSMUSG00000000214	Th	tyrosine hydroxylase [Source:MGI Symbol;Acc:MGI	0.004674
ENSMUSG00000084842	Pabpc1l2b-ps	poly(A) binding protein, cytoplasmic 1-like 2B, pseu	0.004675
ENSMUSG00000031467	Agpat5	1-acylglycerol-3-phosphate O-acyltransferase 5 (lys	0.004683
ENSMUSG00000022337	Emc2	ER membrane protein complex subunit 2 [Source:M	0.004704
ENSMUSG00000033825	Tpsb2	tryptase beta 2 [Source:MGI Symbol;Acc:MGI:9694	0.004709
ENSMUSG00000037965	Zc3h7a	zinc finger CCCH type containing 7 A [Source:MGI S	0.004722
ENSMUSG00000010492	Gm16119	predicted gene 16119 [Source:MGI Symbol;Acc:MG	0.004726
ENSMUSG00000055485	Soga1	suppressor of glucose, autophagy associated 1 [Sou	0.004733
ENSMUSG00000021987	Mtmr6	myotubularin related protein 6 [Source:MGI Symbo	0.004736
ENSMUSG00000021189	Atxn3	ataxin 3 [Source:MGI Symbol;Acc:MGI:1099442]	0.004737
ENSMUSG00000038007	Acer2	alkaline ceramidase 2 [Source:MGI Symbol;Acc:MG	0.004738
ENSMUSG00000029802	Abcg2	ATP-binding cassette, sub-family G (WHITE), memb	0.004743

ENSMUSG00000018589	Glra2	glycine receptor, alpha 2 subunit [Source:MGI Symb	0.004768
ENSMUSG00000041313	Slc7a1	solute carrier family 7 (cationic amino acid transpo	0.004775
ENSMUSG00000029403	Cdkl2	cyclin-dependent kinase-like 2 (CDC2-related kinas	0.004789
ENSMUSG00000025588	Nat1	N-acetyl transferase 1 [Source:MGI Symbol;Acc:MG	0.004791
ENSMUSG00000019230	Lhx9	LIM homeobox protein 9 [Source:MGI Symbol;Acc:	0.004801
ENSMUSG00000020889	Nr1d1	nuclear receptor subfamily 1, group D, member 1 [	0.004805
ENSMUSG00000062078	Qk	quaking [Source:MGI Symbol;Acc:MGI:97837]	0.004819
ENSMUSG00000045767	B230219D22Rik	RIKEN cDNA B230219D22 gene [Source:MGI Symbo	0.004863
ENSMUSG00000040229	Gpr34	G protein-coupled receptor 34 [Source:MGI Symbo	0.004867
ENSMUSG00000073374	C030034I22Rik	RIKEN cDNA C030034I22 gene [Source:MGI Symbol	0.004867
ENSMUSG00000031952	Chst5	carbohydrate (N-acetylglucosamine 6-O) sulfotrans	0.004896
ENSMUSG00000026655	Fam107b	family with sequence similarity 107, member B [Sou	0.004955
ENSMUSG00000039178	Tbc1d19	TBC1 domain family, member 19 [Source:MGI Symb	0.004974
ENSMUSG00000031922	Cep57	centrosomal protein 57 [Source:MGI Symbol;Acc:M	0.004981
ENSMUSG00000061371	Zfp873	zinc finger protein 873 [Source:MGI Symbol;Acc:M	0.005015
ENSMUSG00000007872	Id3	inhibitor of DNA binding 3 [Source:MGI Symbol;Acc	0.005029
ENSMUSG00000074196	Gm6289	predicted gene 6289 [Source:MGI Symbol;Acc:MGI	0.005031
ENSMUSG00000061080	Lsamp	limbic system-associated membrane protein [Sourc	0.005049
ENSMUSG00000037316	Bag4	BCL2-associated athanogene 4 [Source:MGI Symbo	0.00506
ENSMUSG00000025384	2310003H01Rik	RIKEN cDNA 2310003H01 gene [Source:MGI Symbo	0.00506
ENSMUSG00000000266	Mid2	midline 2 [Source:MGI Symbol;Acc:MGI:1344333]	0.005097
ENSMUSG00000009207	Lnp	limb and neural patterns [Source:MGI Symbol;Acc:	0.005097
ENSMUSG00000011831	Evi5	ecotropic viral integration site 5 [Source:MGI Symb	0.005117
ENSMUSG00000022829	Stxbp5l	syntaxin binding protein 5-like [Source:MGI Symbol	0.005144
ENSMUSG00000006435	Neurl1a	neuralized homolog 1A (Drosophila) [Source:MGI S	0.00519
ENSMUSG00000027649	Ctnnbl1	catenin, beta like 1 [Source:MGI Symbol;Acc:MGI:1	0.005192
ENSMUSG00000030583	Sipa1l3	signal-induced proliferation-associated 1 like 3 [Sou	0.005231
ENSMUSG00000040543	Pitpnm3	PITPNM family member 3 [Source:MGI Symbol;Acc	0.005267
ENSMUSG00000022064	Pibf1	progesterone immunomodulatory binding factor 1	0.005274
ENSMUSG00000069833	Ahnak	AHNAK nucleoprotein (desmoyokin) [Source:MGI S	0.00528
ENSMUSG00000042851	Zc3h6	zinc finger CCCH type containing 6 [Source:MGI Sym	0.005281
ENSMUSG00000039103	Nexn	nexilin [Source:MGI Symbol;Acc:MGI:1916060]	0.005284
ENSMUSG00000038541	Srd5a2	steroid 5 alpha-reductase 2 [Source:MGI Symbol;Ac	0.005309
ENSMUSG00000038127	Ccdc50	coiled-coil domain containing 50 [Source:MGI Symb	0.005311
ENSMUSG00000074212	Dnajb14	DnaJ (Hsp40) homolog, subfamily B, member 14 [So	0.005336
ENSMUSG00000020309	Chac2	ChaC, cation transport regulator 2 [Source:MGI Sym	0.005346
ENSMUSG00000002020	Ltbp2	latent transforming growth factor beta binding pro	0.005371
ENSMUSG00000033740	St18	suppression of tumorigenicity 18 [Source:MGI Symb	0.0054
ENSMUSG00000028378	Ptgr1	prostaglandin reductase 1 [Source:MGI Symbol;Acc	0.005406
ENSMUSG00000023883	Phf10	PHD finger protein 10 [Source:MGI Symbol;Acc:MG	0.005411
ENSMUSG00000031357	Syap1	synapse associated protein 1 [Source:MGI Symbol;	0.005426
ENSMUSG00000049265	Kcnk3	potassium channel, subfamily K, member 3 [Source	0.005459
ENSMUSG00000036815	Dpp10	dipeptidylpeptidase 10 [Source:MGI Symbol;Acc:M	0.005469
ENSMUSG00000026961	Lrrc26	leucine rich repeat containing 26 [Source:MGI Syml	0.005494
ENSMUSG00000078906	Gm14444	predicted gene 14444 [Source:MGI Symbol;Acc:MG	0.005494
ENSMUSG00000090459	Gm17210	predicted gene 17210 [Source:MGI Symbol;Acc:MG	0.005494
ENSMUSG00000098061	Gm26945	predicted gene, 26945 [Source:MGI Symbol;Acc:M	0.005509

ENSMUSG00000078190	Dnm3os	dynamin 3, opposite strand [Source:MGI Symbol;A	0.005529
ENSMUSG00000028871	Rspo1	R-spondin homolog (Xenopus laevis) [Source:MGI S	0.005539
ENSMUSG00000084592	Mir1898	microRNA 1898 [Source:MGI Symbol;Acc:MGI:3811	0.00565
ENSMUSG00000097924	A730020E08Rik	RIKEN cDNA A730020E08 gene [Source:MGI Symbo	0.005716
ENSMUSG00000042515	Mum1l1	melanoma associated antigen (mutated) 1-like 1 [S	0.005732
ENSMUSG00000004221	Ikbkg	inhibitor of kappaB kinase gamma [Source:MGI Syn	0.005732
ENSMUSG00000046618	Olfml2a	olfactomedin-like 2A [Source:MGI Symbol;Acc:MGI	0.005745
ENSMUSG00000031438	Rnf128	ring finger protein 128 [Source:MGI Symbol;Acc:M	0.005748
ENSMUSG00000036667	Fam115a	family with sequence similarity 115, member A [So	0.005767
ENSMUSG00000091594	Gm17067	predicted gene 17067 [Source:MGI Symbol;Acc:MG	0.005773
ENSMUSG00000037206	Islr	immunoglobulin superfamily containing leucine-ric	0.005775
ENSMUSG00000044934	Zfp367	zinc finger protein 367 [Source:MGI Symbol;Acc:M	0.00578
ENSMUSG00000026915	Strbp	spermatid perinuclear RNA binding protein [Source	0.005846
ENSMUSG00000020594	Pum2	pumilio 2 (Drosophila) [Source:MGI Symbol;Acc:M	0.005853
ENSMUSG00000030017	Reg3g	regenerating islet-derived 3 gamma [Source:MGI S	0.005864
ENSMUSG00000030653	Pde2a	phosphodiesterase 2A, cGMP-stimulated [Source:M	0.005889
ENSMUSG00000074698	Csnk2a1	casein kinase 2, alpha 1 polypeptide [Source:MGI S	0.005914
ENSMUSG00000007034	Slc44a4	solute carrier family 44, member 4 [Source:MGI Sy	0.005943
ENSMUSG00000053560	Ier2	immediate early response 2 [Source:MGI Symbol;A	0.005951
ENSMUSG00000032475	Nck1	non-catalytic region of tyrosine kinase adaptor pro	0.005955
ENSMUSG00000032854	Ugt8a	UDP galactosyltransferase 8A [Source:MGI Symbol;	0.005959
ENSMUSG00000044098	Rsbn1	rosbin, round spermatid basic protein 1 [Source:M	0.005961
ENSMUSG00000049313	Sorl1	sortilin-related receptor, LDLR class A repeats-cont	0.005972
ENSMUSG00000037386	Rims2	regulating synaptic membrane exocytosis 2 [Source	0.006025
ENSMUSG00000014444	Piezo1	piezo-type mechanosensitive ion channel compone	0.00604
ENSMUSG00000078307	AI593442	expressed sequence AI593442 [Source:MGI Symbol	0.006051
ENSMUSG00000006494	Pdk1	pyruvate dehydrogenase kinase, isoenzyme 1 [Sour	0.006076
ENSMUSG00000020859	Spag9	sperm associated antigen 9 [Source:MGI Symbol;A	0.006079
ENSMUSG00000036856	Wnt4	wingless-related MMTV integration site 4 [Source:M	0.006093
ENSMUSG00000026095	Asnsd1	asparagine synthetase domain containing 1 [Source	0.006094
ENSMUSG00000028270	Gbp2	guanylate binding protein 2 [Source:MGI Symbol;A	0.006095
ENSMUSG00000049502	Dtx3l	deltex 3-like (Drosophila) [Source:MGI Symbol;Acc:	0.006105
ENSMUSG00000002718	Cse1l	chromosome segregation 1-like (S. cerevisiae) [Sou	0.006132
ENSMUSG00000059552	Trp53	transformation related protein 53 [Source:MGI Sym	0.006143
ENSMUSG00000052428	Tmco1	transmembrane and coiled-coil domains 1 [Source:	0.00616
ENSMUSG00000034098	Fstl5	follistatin-like 5 [Source:MGI Symbol;Acc:MGI:2442	0.006183
ENSMUSG00000043631	Ecm2	extracellular matrix protein 2, female organ and ad	0.006189
ENSMUSG00000027002	Nckap1	NCK-associated protein 1 [Source:MGI Symbol;Acc:	0.006208
ENSMUSG00000033706	Smyd5	SET and MYND domain containing 5 [Source:MGI S	0.006239
ENSMUSG00000025491	Ifitm1	interferon induced transmembrane protein 1 [Sour	0.006247
ENSMUSG00000097532	Gm4349	predicted gene 4349 [Source:MGI Symbol;Acc:MGI:	0.006253
ENSMUSG00000058914	C1qtnf3	C1q and tumor necrosis factor related protein 3 [So	0.006253
ENSMUSG00000042743	Sgtb	small glutamine-rich tetratricopeptide repeat (TPR)	0.006278
ENSMUSG00000064138	Fam172a	family with sequence similarity 172, member A [So	0.006282
ENSMUSG00000063694	Cycs	cytochrome c, somatic [Source:MGI Symbol;Acc:M	0.006292
ENSMUSG00000010066	Cacna2d2	calcium channel, voltage-dependent, alpha 2/delta	0.006307
ENSMUSG00000079011	Gm11033	predicted gene 11033 [Source:MGI Symbol;Acc:MG	0.006322

ENSMUSG00000025332	Kdm5c	lysine (K)-specific demethylase 5C [Source:MGI Sym	0.006339
ENSMUSG00000020091	Eif4ebp2	eukaryotic translation initiation factor 4E binding p	0.00635
ENSMUSG00000031273	Col4a6	collagen, type IV, alpha 6 [Source:MGI Symbol;Acc:	0.006367
ENSMUSG00000038344	Txlng	taxilin gamma [Source:MGI Symbol;Acc:MGI:35906	0.006369
ENSMUSG00000028879	Stx12	syntaxin 12 [Source:MGI Symbol;Acc:MGI:1931027	0.006408
ENSMUSG00000036634	Mag	myelin-associated glycoprotein [Source:MGI Symbo	0.006411
ENSMUSG00000008855	Hdac5	histone deacetylase 5 [Source:MGI Symbol;Acc:MG	0.006444
ENSMUSG00000066735	Vkorc1l1	vitamin K epoxide reductase complex, subunit 1-lik	0.006462
ENSMUSG00000020130	Tbc1d15	TBC1 domain family, member 15 [Source:MGI Symb	0.006469
ENSMUSG00000032010	Usp2	ubiquitin specific peptidase 2 [Source:MGI Symbol;	0.006494
ENSMUSG00000025986	Slc39a10	solute carrier family 39 (zinc transporter), member	0.006498
ENSMUSG00000023460	Rab12	RAB12, member RAS oncogene family [Source:MGI	0.006499
ENSMUSG00000001280	Sp1	trans-acting transcription factor 1 [Source:MGI Sym	0.006515
ENSMUSG00000040253	Gbp7	guanylate binding protein 7 [Source:MGI Symbol;A	0.006521
ENSMUSG00000029154	Cwh43	cell wall biogenesis 43 C-terminal homolog (S. cere	0.006526
ENSMUSG00000047414	Flrt2	fibronectin leucine rich transmembrane protein 2 [	0.006528
ENSMUSG00000070565	Rasal2	RAS protein activator like 2 [Source:MGI Symbol;Ac	0.006562
ENSMUSG00000023267	Gabrr2	gamma-aminobutyric acid (GABA) C receptor, subu	0.006566
ENSMUSG00000028229	Rmdn1	regulator of microtubule dynamics 1 [Source:MGI S	0.006571
ENSMUSG00000022200	Golph3	golgi phosphoprotein 3 [Source:MGI Symbol;Acc:M	0.006575
ENSMUSG00000026981	Il1rn	interleukin 1 receptor antagonist [Source:MGI Sym	0.006577
ENSMUSG00000078887	Gm6710	predicted gene 6710 [Source:MGI Symbol;Acc:MGI:	0.006588
ENSMUSG00000066058	Cldn19	claudin 19 [Source:MGI Symbol;Acc:MGI:3033992]	0.006617
ENSMUSG00000020681	Ace	angiotensin I converting enzyme (peptidyl-dipeptid	0.006639
ENSMUSG00000047022	Mipol1	mirror-image polydactyly gene 1 homolog (human)	0.006645
ENSMUSG00000074415	2610203C20Rik	RIKEN cDNA 2610203C20 gene [Source:MGI Symbo	0.006648
ENSMUSG00000000804	Usp32	ubiquitin specific peptidase 32 [Source:MGI Symbo	0.006649
ENSMUSG00000085811	Gm11669	predicted gene 11669 [Source:MGI Symbol;Acc:MG	0.006649
ENSMUSG00000043131	Mob1a	MOB kinase activator 1A [Source:MGI Symbol;Acc:I	0.00666
ENSMUSG00000006373	Pgrmc1	progesterone receptor membrane component 1 [S	0.006668
ENSMUSG00000026885	Ttll11	tubulin tyrosine ligase-like family, member 11 [Sou	0.006677
ENSMUSG00000057182	Scn3a	sodium channel, voltage-gated, type III, alpha [Sour	0.006683
ENSMUSG00000028403	Zdhhc21	zinc finger, DHHC domain containing 21 [Source:M	0.006684
ENSMUSG00000059482	2610301B20Rik	RIKEN cDNA 2610301B20 gene [Source:MGI Symbo	0.006687
ENSMUSG00000027829	Ccnl1	cyclin L1 [Source:MGI Symbol;Acc:MGI:1922664]	0.006705
ENSMUSG00000059355	BC056474	cDNA sequence BC056474 [Source:MGI Symbol;Acc	0.006707
ENSMUSG00000021509	Slc25a48	solute carrier family 25, member 48 [Source:MGI S	0.006715
ENSMUSG00000061778	Mospd2	motile sperm domain containing 2 [Source:MGI Syr	0.006717
ENSMUSG00000067288	Rps28	ribosomal protein S28 [Source:MGI Symbol;Acc:MG	0.006725
ENSMUSG00000073791	Efcab7	EF-hand calcium binding domain 7 [Source:MGI Syr	0.006729
ENSMUSG00000046138	9930021J03Rik	RIKEN cDNA 9930021J03 gene [Source:MGI Symbol	0.006741
ENSMUSG00000046873	Mbtps2	membrane-bound transcription factor peptidase, si	0.006745
ENSMUSG00000032878	Ccdc85a	coiled-coil domain containing 85A [Source:MGI Sym	0.006747
ENSMUSG00000022749	Tbc1d23	TBC1 domain family, member 23 [Source:MGI Symb	0.006817
ENSMUSG00000093117	Gm22699	predicted gene, 22699 [Source:MGI Symbol;Acc:M	0.006835
ENSMUSG00000037152	Ndufc1	NADH dehydrogenase (ubiquinone) 1, subcomplex	0.006852
ENSMUSG00000044628	Rnf208	ring finger protein 208 [Source:MGI Symbol;Acc:M	0.006855

ENSMUSG00000035845	Alg12	asparagine-linked glycosylation 12 (alpha-1,6-mann	0.006866
ENSMUSG00000022051	Bnip3l	BCL2/adenovirus E1B interacting protein 3-like [Sou	0.006878
ENSMUSG00000067931	Zfp948	zinc finger protein 948 [Source:MGI Symbol;Acc:MG	0.006903
ENSMUSG00000028926	Cdk14	cyclin-dependent kinase 14 [Source:MGI Symbol;Ac	0.006904
ENSMUSG00000079043	Fastkd5	FAST kinase domains 5 [Source:MGI Symbol;Acc:MG	0.006914
ENSMUSG00000025609	Mkln1	muskelin 1, intracellular mediator containing kelch	0.006929
ENSMUSG00000025754	Agbl1	ATP/GTP binding protein-like 1 [Source:MGI Symbo	0.006934
ENSMUSG00000072966	Gprasp2	G protein-coupled receptor associated sorting prot	0.006952
ENSMUSG00000062609	Kcnj15	potassium inwardly-rectifying channel, subfamily J,	0.006983
ENSMUSG00000025287	Acot9	acyl-CoA thioesterase 9 [Source:MGI Symbol;Acc:M	0.006984
ENSMUSG00000062794	Zfp599	zinc finger protein 599 [Source:MGI Symbol;Acc:MG	0.006985
ENSMUSG00000037826	Ppm1k	protein phosphatase 1K (PP2C domain containing)	0.006999
ENSMUSG00000020634	Ubxn2a	UBX domain protein 2A [Source:MGI Symbol;Acc:M	0.007006
ENSMUSG00000056682	4930465K10Rik	RIKEN cDNA 4930465K10 gene [Source:MGI Symbo	0.007072
ENSMUSG00000045613	Chrm2	cholinergic receptor, muscarinic 2, cardiac [Source:	0.007075
ENSMUSG00000035649	Zcchc7	zinc finger, CCHC domain containing 7 [Source:MGI	0.007102
ENSMUSG00000043313	Pcdhb19	protocadherin beta 19 [Source:MGI Symbol;Acc:MG	0.007129
ENSMUSG00000008859	Rala	v-ral simian leukemia viral oncogene homolog A (ra	0.007133
ENSMUSG00000094834	Gm25472	predicted gene, 25472 [Source:MGI Symbol;Acc:MG	0.00714
ENSMUSG00000028013	Ppa2	pyrophosphatase (inorganic) 2 [Source:MGI Symbo	0.007141
ENSMUSG00000017721	Pigt	phosphatidylinositol glycan anchor biosynthesis, cla	0.00716
ENSMUSG00000095913	Gm21844	predicted gene, 21844 [Source:MGI Symbol;Acc:MG	0.00719
ENSMUSG00000035439	Haus8	4HAUS augmin-like complex, subunit 8 [Source:MG	0.007196
ENSMUSG00000027004	Frzb	frizzled-related protein [Source:MGI Symbol;Acc:M	0.007234
ENSMUSG00000092202	Stk19-ps1	serine/threonine kinase 19, pseudogene 1 [Source:	0.007261
ENSMUSG00000089951	Gm14435	predicted gene 14435 [Source:MGI Symbol;Acc:MG	0.007302
ENSMUSG00000037058	Paip2	polyadenylate-binding protein-interacting protein 2	0.007327
ENSMUSG00000087196	Gm13373	predicted gene 13373 [Source:MGI Symbol;Acc:MG	0.007342
ENSMUSG00000085423	Gm15735	predicted gene 15735 [Source:MGI Symbol;Acc:MG	0.007369
ENSMUSG00000038351	Sgsm2	small G protein signaling modulator 2 [Source:MGI	0.00737
ENSMUSG00000027680	Fxr1	fragile X mental retardation gene 1, autosomal hom	0.007405
ENSMUSG00000056296	Synpr	synaptoporin [Source:MGI Symbol;Acc:MGI:191925	0.007427
ENSMUSG00000069132	Nxph2	neurexophilin 2 [Source:MGI Symbol;Acc:MGI:1074	0.007428
ENSMUSG00000097171	Gm17644	predicted gene, 17644 [Source:MGI Symbol;Acc:MG	0.00744
ENSMUSG00000021413	Prpf4b	PRP4 pre-mRNA processing factor 4 homolog B (yea	0.007466
ENSMUSG00000009470	Tnpo1	transportin 1 [Source:MGI Symbol;Acc:MGI:268152	0.007522
ENSMUSG00000024188	Luc7l	Luc7 homolog (S. cerevisiae)-like [Source:MGI Symb	0.007535
ENSMUSG00000031591	Asah1	N-acylsphingosine amidohydrolase 1 [Source:MGI S	0.007541
ENSMUSG00000033111	3830406C13Rik	RIKEN cDNA 3830406C13 gene [Source:MGI Symbo	0.007564
ENSMUSG00000044139	Prss53	protease, serine, 53 [Source:MGI Symbol;Acc:MGI:1	0.007593
ENSMUSG00000055370	Gm9968	predicted gene 9968 [Source:MGI Symbol;Acc:MGI:	0.00762
ENSMUSG00000045648	Vwc2l	von Willebrand factor C domain-containing protein	0.007626
ENSMUSG00000023951	Vegfa	vascular endothelial growth factor A [Source:MGI S	0.007627
ENSMUSG00000096606	Gm4980	predicted gene 4980 [Source:MGI Symbol;Acc:MGI:	0.007635
ENSMUSG00000032409	Atr	ataxia telangiectasia and Rad3 related [Source:MGI	0.007639
ENSMUSG00000030768	Disp1	dispatched homolog 1 (Drosophila) [Source:MGI Sy	0.007664
ENSMUSG00000004667	Polr2e	polymerase (RNA) II (DNA directed) polypeptide E [	0.007669

ENSMUSG00000038733	Wdr26	WD repeat domain 26 [Source:MGI Symbol;Acc:MG	0.007676
ENSMUSG00000024810	Il33	interleukin 33 [Source:MGI Symbol;Acc:MGI:19243	0.007682
ENSMUSG00000024811	Tnks2	tankyrase, TRF1-interacting ankyrin-related ADP-rib	0.007692
ENSMUSG00000024360	Etf1	eukaryotic translation termination factor 1 [Source	0.007694
ENSMUSG00000026417	Pigr	polymeric immunoglobulin receptor [Source:MGI S	0.007708
ENSMUSG00000025246	Tbl1x	transducin (beta)-like 1 X-linked [Source:MGI Symb	0.007726
ENSMUSG00000027479	Mapre1	microtubule-associated protein, RP/EB family, mem	0.007738
ENSMUSG00000022378	Fam49b	family with sequence similarity 49, member B [Sour	0.007741
ENSMUSG00000020390	Ube2b	ubiquitin-conjugating enzyme E2B [Source:MGI Sym	0.007759
ENSMUSG00000064329	Scn1a	sodium channel, voltage-gated, type I, alpha [Sourc	0.007777
ENSMUSG00000029655	N4bp2l2	NEDD4 binding protein 2-like 2 [Source:MGI Symbo	0.007783
ENSMUSG00000017421	Zfp207	zinc finger protein 207 [Source:MGI Symbol;Acc:MG	0.007784
ENSMUSG00000018189	Uchl5	ubiquitin carboxyl-terminal esterase L5 [Source:MG	0.007789
ENSMUSG00000050711	Scg2	secretogranin II [Source:MGI Symbol;Acc:MGI:1030	0.007799
ENSMUSG00000074340	Ovgp1	oviductal glycoprotein 1 [Source:MGI Symbol;Acc:M	0.007816
ENSMUSG00000067219	Nipal1	NIPA-like domain containing 1 [Source:MGI Symbol	0.007825
ENSMUSG00000052917	Senp7	SUMO1/sentrin specific peptidase 7 [Source:MGI Sy	0.007835
ENSMUSG00000055541	Lair1	leukocyte-associated Ig-like receptor 1 [Source:MG	0.007837
ENSMUSG00000021732	Fgf10	fibroblast growth factor 10 [Source:MGI Symbol;Ac	0.007837
ENSMUSG00000044349	Snhg11	small nucleolar RNA host gene 11 [Source:MGI Sym	0.007858
ENSMUSG00000010936	Vac14	Vac14 homolog (S. cerevisiae) [Source:MGI Symbol	0.007883
ENSMUSG00000048163	Selplg	selectin, platelet (p-selectin) ligand [Source:MGI Sy	0.007884
ENSMUSG00000059474	Mbtd1	mbt domain containing 1 [Source:MGI Symbol;Acc:	0.007911
ENSMUSG00000097258	Gm26767	predicted gene, 26767 [Source:MGI Symbol;Acc:MG	0.007932
ENSMUSG00000097554	Gm26825	predicted gene, 26825 [Source:MGI Symbol;Acc:MG	0.007976
ENSMUSG00000025351	Cd63	CD63 antigen [Source:MGI Symbol;Acc:MGI:99529]	0.00799
ENSMUSG00000050088	1600012H06Rik	RIKEN cDNA 1600012H06 gene [Source:MGI Symbo	0.008002
ENSMUSG00000022510	Trp63	transformation related protein 63 [Source:MGI Sym	0.008005
ENSMUSG00000027806	Tsc22d2	TSC22 domain family, member 2 [Source:MGI Symb	0.008045
ENSMUSG00000030187	Klra2	killer cell lectin-like receptor, subfamily A, member	0.008048
ENSMUSG00000048424	Ranbp3l	RAN binding protein 3-like [Source:MGI Symbol;Acc	0.008059
ENSMUSG00000038112	AW551984	expressed sequence AW551984 [Source:MGI Symb	0.008098
ENSMUSG00000035378	Shq1	SHQ1 homolog (S. cerevisiae) [Source:MGI Symbol;	0.008101
ENSMUSG00000082229	Nap1l2	nucleosome assembly protein 1-like 2 [Source:MGI	0.008102
ENSMUSG00000025036	Sfxn2	sideroflexin 2 [Source:MGI Symbol;Acc:MGI:213767	0.008106
ENSMUSG00000021076	Actr10	ARP10 actin-related protein 10 [Source:MGI Symbo	0.008113
ENSMUSG00000010205	Raver1	ribonucleoprotein, PTB-binding 1 [Source:MGI Sym	0.008122
ENSMUSG00000040084	Bub1b	budding uninhibited by benzimidazoles 1 homolog,	0.008145
ENSMUSG00000041153	Osgin2	oxidative stress induced growth inhibitor family me	0.00815
ENSMUSG00000041609	Ccdc64	coiled-coil domain containing 64 [Source:MGI Symb	0.008158
ENSMUSG00000013629	Cad	carbamoyl-phosphate synthetase 2, aspartate trans	0.00819
ENSMUSG00000051339	2900026A02Rik	RIKEN cDNA 2900026A02 gene [Source:MGI Symbo	0.008193
ENSMUSG00000004319	Clcn3	chloride channel 3 [Source:MGI Symbol;Acc:MGI:10	0.008195
ENSMUSG00000040268	Plekha1	pleckstrin homology domain containing, family A (p	0.008229
ENSMUSG00000053205	Styx	serine/threonine/tyrosine interaction protein [Sour	0.00824
ENSMUSG00000024002	Brd4	bromodomain containing 4 [Source:MGI Symbol;Ac	0.008241
ENSMUSG00000066829	Zfp810	zinc finger protein 810 [Source:MGI Symbol;Acc:MG	0.008254

ENSMUSG00000030341	Tnfrsf1a	tumor necrosis factor receptor superfamily, membe	0.008292
ENSMUSG00000031617	Tmem184c	transmembrane protein 184C [Source:MGI Symbol;	0.008326
ENSMUSG00000037217	Syn1	synapsin I [Source:MGI Symbol;Acc:MGI:98460]	0.008334
ENSMUSG00000041028	Ghitm	growth hormone inducible transmembrane protein	0.008346
ENSMUSG00000024613	Tcof1	Treacher Collins Franceschetti syndrome 1, homolo	0.008372
ENSMUSG00000031644	Nek1	NIMA (never in mitosis gene a)-related expressed k	0.008383
ENSMUSG00000060890	Arr3	arrestin 3, retinal [Source:MGI Symbol;Acc:MGI:21	0.008394
ENSMUSG00000016763	Scube1	signal peptide, CUB domain, EGF-like 1 [Source:MG	0.008417
ENSMUSG00000059187	Fam19a1	family with sequence similarity 19, member A1 [So	0.008465
ENSMUSG00000097335	Gm26563	predicted gene, 26563 [Source:MGI Symbol;Acc:M	0.008466
ENSMUSG00000036768	Kif15	kinesin family member 15 [Source:MGI Symbol;Acc	0.008466
ENSMUSG00000030137	Tuba8	tubulin, alpha 8 [Source:MGI Symbol;Acc:MGI:1858	0.008469
ENSMUSG00000025997	Ikzf2	IKAROS family zinc finger 2 [Source:MGI Symbol;Ac	0.008473
ENSMUSG00000022912	Pros1	protein S (alpha) [Source:MGI Symbol;Acc:MGI:109	0.00848
ENSMUSG00000024592	C330018D20Rik	RIKEN cDNA C330018D20 gene [Source:MGI Symbo	0.008489
ENSMUSG00000044499	Hs3st5	heparan sulfate (glucosamine) 3-O-sulfotransferase	0.008491
ENSMUSG00000097140	Gm26779	predicted gene, 26779 [Source:MGI Symbol;Acc:M	0.008495
ENSMUSG00000027170	Eif3m	eukaryotic translation initiation factor 3, subunit M	0.008558
ENSMUSG00000039850	Endov	endonuclease V [Source:MGI Symbol;Acc:MGI:2444	0.008615
ENSMUSG00000097734	B930082K07Rik	RIKEN cDNA B930082K07 gene [Source:MGI Symbo	0.008622
ENSMUSG00000030754	Copb1	coatomer protein complex, subunit beta 1 [Source:	0.008626
ENSMUSG00000030393	Zik1	zinc finger protein interacting with K protein 1 [Sou	0.008646
ENSMUSG00000032252	Glce	glucuronyl C5-epimerase [Source:MGI Symbol;Acc:	0.00865
ENSMUSG00000032939	Nup93	nucleoporin 93 [Source:MGI Symbol;Acc:MGI:1919	0.008654
ENSMUSG00000074796	Slc4a11	solute carrier family 4, sodium bicarbonate transpo	0.008655
ENSMUSG00000026638	Irf6	interferon regulatory factor 6 [Source:MGI Symbol;	0.008662
ENSMUSG00000006522	Itih3	inter-alpha trypsin inhibitor, heavy chain 3 [Source:	0.008666
ENSMUSG00000044147	Arf6	ADP-ribosylation factor 6 [Source:MGI Symbol;Acc:	0.008683
ENSMUSG00000037940	Inpp4b	inositol polyphosphate-4-phosphatase, type II [Sou	0.008691
ENSMUSG00000057265	Ccdc176	coiled-coil domain containing 176 [Source:MGI Sym	0.00871
ENSMUSG00000079465	Col4a3	collagen, type IV, alpha 3 [Source:MGI Symbol;Acc:	0.008718
ENSMUSG00000023904	Hcfc1r1	host cell factor C1 regulator 1 (XPO1-dependent) [S	0.00873
ENSMUSG00000064293	Cntn4	contactin 4 [Source:MGI Symbol;Acc:MGI:1095737]	0.008737
ENSMUSG00000028796	Phc2	polyhomeotic-like 2 (Drosophila) [Source:MGI Sym	0.00876
ENSMUSG00000073643	Wdfy1	WD repeat and FYVE domain containing 1 [Source:	0.008773
ENSMUSG00000020437	Myo1g	myosin IG [Source:MGI Symbol;Acc:MGI:1927091]	0.008783
ENSMUSG00000054134	Umodl1	uromodulin-like 1 [Source:MGI Symbol;Acc:MGI:19	0.008791
ENSMUSG00000097445	Gm26631	predicted gene, 26631 [Source:MGI Symbol;Acc:M	0.008802
ENSMUSG00000036879	Phkb	phosphorylase kinase beta [Source:MGI Symbol;Acc	0.008808
ENSMUSG00000004360	9330159F19Rik	RIKEN cDNA 9330159F19 gene [Source:MGI Symbo	0.008826
ENSMUSG00000043635	Adamts3	a disintegrin-like and metallopeptidase (reprolysin	0.008832
ENSMUSG00000063052	Lrrc40	leucine rich repeat containing 40 [Source:MGI Sym	0.008878
ENSMUSG00000028343	Erp44	endoplasmic reticulum protein 44 [Source:MGI Sym	0.008883
ENSMUSG00000038604	Fam65a	family with sequence similarity 65, member A [Sou	0.008891
ENSMUSG00000023034	Nr4a1	nuclear receptor subfamily 4, group A, member 1 [	0.008905
ENSMUSG00000018341	Il12rb2	interleukin 12 receptor, beta 2 [Source:MGI Symbo	0.008923
ENSMUSG00000045624	Esf1	ESF1, nucleolar pre-rRNA processing protein, homo	0.00894

ENSMUSG00000043165	Lor	loricrin [Source:MGI Symbol;Acc:MGI:96816]	0.008944
ENSMUSG00000031559	4930555F03Rik	RIKEN cDNA 4930555F03 gene [Source:MGI Symbo	0.00895
ENSMUSG00000031950	Gabarapl2	gamma-aminobutyric acid (GABA) A receptor-assoc	0.008966
ENSMUSG00000089707	4921525B02Rik	RIKEN cDNA 4921525B02 gene [Source:MGI Symbo	0.008972
ENSMUSG00000032757	Bet1	blocked early in transport 1 homolog (S. cerevisiae)	0.008974
ENSMUSG00000033014	Trim33	tripartite motif-containing 33 [Source:MGI Symbol;	0.008982
ENSMUSG00000067424	Zfp563	zinc finger protein 563 [Source:MGI Symbol;Acc:MG	0.008988
ENSMUSG00000053192	Mllt11	myeloid/lymphoid or mixed-lineage leukemia (trith	0.009007
ENSMUSG00000030373	Psg28	pregnancy-specific glycoprotein 28 [Source:MGI Sy	0.00905
ENSMUSG00000052819	Best2	bestrophin 2 [Source:MGI Symbol;Acc:MGI:238758	0.009063
ENSMUSG00000001506	Col1a1	collagen, type I, alpha 1 [Source:MGI Symbol;Acc:M	0.009073
ENSMUSG00000030510	Cers3	ceramide synthase 3 [Source:MGI Symbol;Acc:MGI:	0.009087
ENSMUSG00000025925	Terf1	telomeric repeat binding factor 1 [Source:MGI Sym	0.009127
ENSMUSG00000029165	Agbl5	ATP/GTP binding protein-like 5 [Source:MGI Symbo	0.009136
ENSMUSG00000023075	Akirin1	akirin 1 [Source:MGI Symbol;Acc:MGI:1915300]	0.009141
ENSMUSG00000075318	Scn2a1	sodium channel, voltage-gated, type II, alpha 1 [Sou	0.009157
ENSMUSG00000033364	Usp37	ubiquitin specific peptidase 37 [Source:MGI Symbo	0.009161
ENSMUSG00000025809	Itgb1	integrin beta 1 (fibronectin receptor beta) [Source:	0.009162
ENSMUSG00000032006	Pdgfd	platelet-derived growth factor, D polypeptide [Sour	0.009215
ENSMUSG00000020305	Asb3	ankyrin repeat and SOCS box-containing 3 [Source:	0.009229
ENSMUSG00000050310	Rictor	RPTOR independent companion of MTOR, complex	0.009246
ENSMUSG00000016664	Pacsin2	protein kinase C and casein kinase substrate in neu	0.009254
ENSMUSG00000037621	Atoh8	atonal homolog 8 (Drosophila) [Source:MGI Symbo	0.009255
ENSMUSG00000075044	Slc22a29	solute carrier family 22. member 29 [Source:MGI Sy	0.009322
ENSMUSG00000034361	Cpne2	copine II [Source:MGI Symbol;Acc:MGI:2387578]	0.009356
ENSMUSG00000049047	Armcx3	armadillo repeat containing, X-linked 3 [Source:MG	0.009394
ENSMUSG00000049878	Rlf	rearranged L-myc fusion sequence [Source:MGI Syn	0.009395
ENSMUSG00000032776	Mctp2	multiple C2 domains, transmembrane 2 [Source:MG	0.009398
ENSMUSG00000091747	D17H6S56E-5	DNA segment, Chr 17, human D6S56E 5 [Source:MG	0.009408
ENSMUSG00000078877	Gm14295	predicted gene 14295 [Source:MGI Symbol;Acc:MG	0.009408
ENSMUSG00000023015	Racgap1	Rac GTPase-activating protein 1 [Source:MGI Symb	0.00942
ENSMUSG00000026748	Plxdc2	plexin domain containing 2 [Source:MGI Symbol;Ac	0.009426
ENSMUSG00000045776	Lrtm1	leucine-rich repeats and transmembrane domains 1	0.009436
ENSMUSG00000083116	Gm13410	predicted gene 13410 [Source:MGI Symbol;Acc:MG	0.009449
ENSMUSG00000046049	Rp1l1	retinitis pigmentosa 1 homolog (human)-like 1 [Sou	0.009453
ENSMUSG00000039163	Cmc1	COX assembly mitochondrial protein 1 [Source:MG	0.009488
ENSMUSG00000022634	Yaf2	YY1 associated factor 2 [Source:MGI Symbol;Acc:M	0.009491
ENSMUSG00000070883	Ccdc173	coiled-coil domain containing 173 [Source:MGI Sym	0.009497
ENSMUSG00000021109	Hif1a	hypoxia inducible factor 1, alpha subunit [Source:M	0.009503
ENSMUSG00000039191	Rbpj	recombination signal binding protein for immunogl	0.009509
ENSMUSG00000085558	4930412C18Rik	RIKEN cDNA 4930412C18 gene [Source:MGI Symbo	0.009526
ENSMUSG00000043629	1700019D03Rik	RIKEN cDNA 1700019D03 gene [Source:MGI Symbo	0.009527
ENSMUSG00000059708	Akap17b	A kinase (PRKA) anchor protein 17B [Source:MGI Sy	0.009558
ENSMUSG00000009535	Rnmt	RNA (guanine-7-) methyltransferase [Source:MGI S	0.009582
ENSMUSG00000029684	Wasl	Wiskott-Aldrich syndrome-like (human) [Source:MG	0.009609
ENSMUSG00000097312	Gm26870	predicted gene, 26870 [Source:MGI Symbol;Acc:MG	0.009611
ENSMUSG00000022664	Slc35a5	solute carrier family 35, member A5 [Source:MGI Sy	0.009667

ENSMUSG00000055704	Gm9978	predicted gene 9978 [Source:MGI Symbol;Acc:MGI:	0.009668
ENSMUSG00000029213	Commd8	COMM domain containing 8 [Source:MGI Symbol;A	0.009699
ENSMUSG00000087305	A430035B10Rik	RIKEN cDNA A430035B10 gene [Source:MGI Symbo	0.009719
ENSMUSG00000037625	Cldn11	claudin 11 [Source:MGI Symbol;Acc:MGI:106925]	0.009795
ENSMUSG00000038074	Fkbp14	FK506 binding protein 14 [Source:MGI Symbol;Acc:	0.009818
ENSMUSG00000033578	Tmem35	transmembrane protein 35 [Source:MGI Symbol;Ac	0.00982
ENSMUSG00000041695	Kcnj2	potassium inwardly-rectifying channel, subfamily J,	0.009822
ENSMUSG00000030275	Etnk1	ethanolamine kinase 1 [Source:MGI Symbol;Acc:M	0.009822
ENSMUSG00000031343	Gabra3	gamma-aminobutyric acid (GABA) A receptor, subu	0.009864
ENSMUSG00000059005	Hnrnpa3	heterogeneous nuclear ribonucleoprotein A3 [Sour	0.009875
ENSMUSG00000087813	Gm22490	predicted gene, 22490 [Source:MGI Symbol;Acc:M	0.009877
ENSMUSG00000055733	Nap1l3	nucleosome assembly protein 1-like 3 [Source:MGI	0.009903
ENSMUSG00000023959	Clic5	chloride intracellular channel 5 [Source:MGI Symbo	0.009938
ENSMUSG00000028497	Ptplad2	protein tyrosine phosphatase-like A domain contai	0.009997
ENSMUSG00000063851	Rnf183	ring finger protein 183 [Source:MGI Symbol;Acc:M	0.010019
ENSMUSG00000029859	Epha1	Eph receptor A1 [Source:MGI Symbol;Acc:MGI:107	0.010028
ENSMUSG00000042182	Bend6	BEN domain containing 6 [Source:MGI Symbol;Acc:	0.01003
ENSMUSG00000000732	Icosl	icos ligand [Source:MGI Symbol;Acc:MGI:1354701]	0.010049
ENSMUSG00000030657	Xylt1	xylosyltransferase 1 [Source:MGI Symbol;Acc:MGI:	0.010086
ENSMUSG00000056536	Pign	phosphatidylinositol glycan anchor biosynthesis, cla	0.0101
ENSMUSG00000038000	Acd	adrenocortical dysplasia [Source:MGI Symbol;Acc:M	0.010144
ENSMUSG00000074656	Eif2s2	eukaryotic translation initiation factor 2, subunit 2	0.010167
ENSMUSG00000025038	Efhc2	EF-hand domain (C-terminal) containing 2 [Source:	0.010181
ENSMUSG00000040721	Zfhx2	zinc finger homeobox 2 [Source:MGI Symbol;Acc:M	0.010204
ENSMUSG00000091623	Gm17092	predicted gene 17092 [Source:MGI Symbol;Acc:MG	0.010205
ENSMUSG00000040463	Mybbp1a	MYB binding protein (P160) 1a [Source:MGI Symbo	0.010214
ENSMUSG00000032375	Aph1b	anterior pharynx defective 1b homolog (C. elegans)	0.010219
ENSMUSG00000042269	Fam92b	family with sequence similarity 92, member B [Sou	0.010221
ENSMUSG00000052488	Cherp	calcium homeostasis endoplasmic reticulum protei	0.010222
ENSMUSG00000028906	Epb4.1	erythrocyte protein band 4.1 [Source:MGI Symbol;	0.010225
ENSMUSG00000044519	Zfp488	zinc finger protein 488 [Source:MGI Symbol;Acc:M	0.010227
ENSMUSG00000053477	Tcf4	transcription factor 4 [Source:MGI Symbol;Acc:MGI	0.010245
ENSMUSG00000023987	Pgc	progastricsin (pepsinogen C) [Source:MGI Symbol;A	0.010249
ENSMUSG00000021009	Ptpn21	protein tyrosine phosphatase, non-receptor type 2	0.010256
ENSMUSG00000022663	Atg3	autophagy related 3 [Source:MGI Symbol;Acc:MGI:	0.010301
ENSMUSG00000042111	Ccdc115	coiled-coil domain containing 115 [Source:MGI Sym	0.010308
ENSMUSG00000025049	Taf5	TAF5 RNA polymerase II, TATA box binding protein	0.010313
ENSMUSG00000071252	2210408I21Rik	RIKEN cDNA 2210408I21 gene [Source:MGI Symbol	0.01032
ENSMUSG00000029878	Dbpht2	DNA binding protein with his-thr domain [Source:M	0.010326
ENSMUSG00000022020	Naa16	N(alpha)-acetyltransferase 16, NatA auxiliary subur	0.010332
ENSMUSG00000055884	Fancm	Fanconi anemia, complementation group M [Sourc	0.010336
ENSMUSG00000033943	Mga	MAX gene associated [Source:MGI Symbol;Acc:MG	0.010391
ENSMUSG00000097766	5730420D15Rik	RIKEN cDNA 5730420D15 gene [Source:MGI Symbo	0.010396
ENSMUSG00000097021	4933433G15Rik	RIKEN cDNA 4933433G15 gene [Source:MGI Symbo	0.010457
ENSMUSG00000084283	Gm14914	predicted gene 14914 [Source:MGI Symbol;Acc:MG	0.010497
ENSMUSG00000041476	Smpx	small muscle protein, X-linked [Source:MGI Symbol	0.010519
ENSMUSG00000036402	Gng12	guanine nucleotide binding protein (G protein), gar	0.010545

ENSMUSG00000089742	Gm15898	predicted gene 15898 [Source:MGI Symbol;Acc:MG	0.010597
ENSMUSG00000055660	Mettl4	methyltransferase like 4 [Source:MGI Symbol;Acc:M	0.010627
ENSMUSG00000027787	Nmd3	NMD3 homolog (S. cerevisiae) [Source:MGI Symbol	0.010639
ENSMUSG00000079056	Kcnip3	Kv channel interacting protein 3, calsenilin [Source:	0.010697
ENSMUSG00000039166	Akap7	A kinase (PRKA) anchor protein 7 [Source:MGI Sym	0.01071
ENSMUSG00000032463	Faim	Fas apoptotic inhibitory molecule [Source:MGI Sym	0.010717
ENSMUSG00000040280	Ndufa4l2	NADH dehydrogenase (ubiquinone) 1 alpha subcom	0.010733
ENSMUSG00000020193	Zpbp	zona pellucida binding protein [Source:MGI Symbol	0.010737
ENSMUSG00000032336	Nptn	neuroplastin [Source:MGI Symbol;Acc:MGI:108077	0.010739
ENSMUSG00000055493	Epm2a	epilepsy, progressive myoclonic epilepsy, type 2 ge	0.01075
ENSMUSG00000051627	Hist1h1e	histone cluster 1, H1e [Source:MGI Symbol;Acc:MG	0.010767
ENSMUSG00000062826	Ces2f	carboxylesterase 2F [Source:MGI Symbol;Acc:MGI:1	0.010787
ENSMUSG00000011263	Exoc3l2	exocyst complex component 3-like 2 [Source:MGI S	0.010793
ENSMUSG00000036372	Tmem258	transmembrane protein 258 [Source:MGI Symbol;A	0.010809
ENSMUSG00000021147	Wdr37	WD repeat domain 37 [Source:MGI Symbol;Acc:MG	0.010816
ENSMUSG00000037266	D4Wsu53e	DNA segment, Chr 4, Wayne State University 53, ex	0.010817
ENSMUSG00000062391	4932435O22Rik	RIKEN cDNA 4932435O22 gene [Source:MGI Symbo	0.01087
ENSMUSG00000021552	Gkap1	G kinase anchoring protein 1 [Source:MGI Symbol;A	0.010911
ENSMUSG00000034312	Iqsec1	IQ motif and Sec7 domain 1 [Source:MGI Symbol;Ac	0.010947
ENSMUSG00000026496	Parp1	poly (ADP-ribose) polymerase family, member 1 [So	0.010955
ENSMUSG00000074207	Adh1	alcohol dehydrogenase 1 (class I) [Source:MGI Sym	0.010963
ENSMUSG00000042275	Pelo	pelota homolog (Drosophila) [Source:MGI Symbol;A	0.010968
ENSMUSG00000036151	Tm6sf2	transmembrane 6 superfamily member 2 [Source:M	0.010988
ENSMUSG00000031325	4930519F16Rik	RIKEN cDNA 4930519F16 gene [Source:MGI Symbo	0.011002
ENSMUSG00000090035	Galnt4	UDP-N-acetyl-alpha-D-galactosamine:polypeptide N	0.011006
ENSMUSG00000042109	Csdc2	cold shock domain containing C2, RNA binding [Sou	0.01101
ENSMUSG00000034160	Ogt	O-linked N-acetylglucosamine (GlcNAc) transferase	0.011051
ENSMUSG00000027108	Ola1	Obg-like ATPase 1 [Source:MGI Symbol;Acc:MGI:19	0.011067
ENSMUSG00000018411	Mapt	microtubule-associated protein tau [Source:MGI Sy	0.011094
ENSMUSG00000044707	Ccnjl	cyclin J-like [Source:MGI Symbol;Acc:MGI:2685723	0.011105
ENSMUSG00000068798	Rap1a	RAS-related protein-1a [Source:MGI Symbol;Acc:M	0.011106
ENSMUSG00000042507	Elmsan1	ELM2 and Myb/SANT-like domain containing 1 [Sou	0.011118
ENSMUSG00000027356	Fermt1	fermitin family homolog 1 (Drosophila) [Source:MG	0.011123
ENSMUSG00000027810	Eif2a	eukaryotic translation initiation factor 2A [Source:M	0.011136
ENSMUSG00000081042	Psmb7-ps2	proteasome (prosome, macropain) subunit, beta ty	0.01116
ENSMUSG00000027130	Slc12a6	solute carrier family 12, member 6 [Source:MGI Sy	0.011176
ENSMUSG00000097971	Gm26917	predicted gene, 26917 [Source:MGI Symbol;Acc:MG	0.011205
ENSMUSG00000062257	Opcml	opioid binding protein/cell adhesion molecule-like	0.011209
ENSMUSG00000021010	Npas3	neuronal PAS domain protein 3 [Source:MGI Symbo	0.011247
ENSMUSG00000019948	Actr6	ARP6 actin-related protein 6 [Source:MGI Symbol;A	0.011262
ENSMUSG00000026581	Sell	selectin, lymphocyte [Source:MGI Symbol;Acc:MGI:	0.011286
ENSMUSG00000021470	Ercc6l2	excision repair cross-complementing rodent repair	0.011295
ENSMUSG00000029174	Tbc1d1	TBC1 domain family, member 1 [Source:MGI Symbo	0.011295
ENSMUSG00000034021	Pds5b	PDS5, regulator of cohesion maintenance, homolog	0.011316
ENSMUSG00000034248	Slc25a37	solute carrier family 25, member 37 [Source:MGI Sy	0.011326
ENSMUSG00000053693	Mast1	microtubule associated serine/threonine kinase 1 [	0.011361
ENSMUSG00000027868	Tbx15	T-box 15 [Source:MGI Symbol;Acc:MGI:1277234]	0.011382

ENSMUSG00000030867	Plk1	polo-like kinase 1 [Source:MGI Symbol;Acc:MGI:97(	0.011386
ENSMUSG00000032422	Snx14	sorting nexin 14 [Source:MGI Symbol;Acc:MGI:215!	0.011389
ENSMUSG00000001750	Tcirg1	T cell, immune regulator 1, ATPase, H+ transporting	0.01145
ENSMUSG00000026872	Zeb2	zinc finger E-box binding homeobox 2 [Source:MGI	0.011484
ENSMUSG00000026814	Eng	endoglin [Source:MGI Symbol;Acc:MGI:95392]	0.011489
ENSMUSG00000008575	Nfib	nuclear factor I/B [Source:MGI Symbol;Acc:MGI:10	0.011514
ENSMUSG00000055053	Nfic	nuclear factor I/C [Source:MGI Symbol;Acc:MGI:10	0.011523
ENSMUSG00000045658	Pid1	phosphotyrosine interaction domain containing 1 [	0.011529
ENSMUSG00000097026	Gm26786	predicted gene, 26786 [Source:MGI Symbol;Acc:M(	0.011535
ENSMUSG00000033697	Arhgap39	Rho GTPase activating protein 39 [Source:MGI Sym	0.011569
ENSMUSG00000027377	Mall	mal, T cell differentiation protein-like [Source:MGI	0.011584
ENSMUSG00000029810	Tmem176b	transmembrane protein 176B [Source:MGI Symbol;	0.011605
ENSMUSG00000063109	A930013B10Rik	RIKEN cDNA A930013B10 gene [Source:MGI Symbo	0.011655
ENSMUSG00000041577	Prelp	proline arginine-rich end leucine-rich repeat [Sourc	0.011719
ENSMUSG00000035683	Melk	maternal embryonic leucine zipper kinase [Source:I	0.011723
ENSMUSG00000064350	mt-Ty	mitochondrially encoded tRNA tyrosine [Source:M(	0.011726
ENSMUSG00000048960	Prex2	phosphatidylinositol-3,4,5-trisphosphate-depender	0.011736
ENSMUSG00000051297	2410124H12Rik	RIKEN cDNA 2410124H12 gene [Source:MGI Symbo	0.011748
ENSMUSG00000082361	Btc	betacellulin, epidermal growth factor family memb	0.011769
ENSMUSG00000043230	Fam124b	family with sequence similarity 124, member B [Sou	0.011781
ENSMUSG00000086171	C630020P19Rik	RIKEN cDNA C630020P19 gene [Source:MGI Symbo	0.011784
ENSMUSG00000038178	Slc43a2	solute carrier family 43, member 2 [Source:MGI Syr	0.011798
ENSMUSG00000045205	Dpy19l4	dpy-19-like 4 (C. elegans) [Source:MGI Symbol;Acc:	0.011834
ENSMUSG00000044906	4930503L19Rik	RIKEN cDNA 4930503L19 gene [Source:MGI Symbo	0.01185
ENSMUSG00000027763	Mbnl1	muscleblind-like 1 (Drosophila) [Source:MGI Symbo	0.011854
ENSMUSG00000042254	Cilp	cartilage intermediate layer protein, nucleotide pyr	0.011885
ENSMUSG00000008999	Bmp7	bone morphogenetic protein 7 [Source:MGI Symbo	0.011898
ENSMUSG00000089708	5330439A09Rik	RIKEN cDNA 5330439A09 gene [Source:MGI Symbo	0.011929
ENSMUSG00000090965	Gm17203	predicted gene 17203 [Source:MGI Symbol;Acc:MG	0.011962
ENSMUSG00000032899	Styk1	serine/threonine/tyrosine kinase 1 [Source:MGI Syı	0.011973
ENSMUSG00000087431	Gm16054	predicted gene 16054 [Source:MGI Symbol;Acc:MG	0.011976
ENSMUSG00000058385	Hist1h2bg	histone cluster 1, H2bg [Source:MGI Symbol;Acc:M	0.011986
ENSMUSG00000045991	Onecut2	one cut domain, family member 2 [Source:MGI Sym	0.012024
ENSMUSG00000020908	Myh3	myosin, heavy polypeptide 3, skeletal muscle, emb	0.012026
ENSMUSG00000033157	Abhd10	abhydrolase domain containing 10 [Source:MGI Syr	0.012026
ENSMUSG00000038280	Ostm1	osteopetrosis associated transmembrane protein 1	0.012028
ENSMUSG00000025229	Pitx3	paired-like homeodomain transcription factor 3 [So	0.012033
ENSMUSG00000041245	Wnk3	WNK lysine deficient protein kinase 3 [Source:MGI	0.012038
ENSMUSG00000061306	Slc38a10	solute carrier family 38, member 10 [Source:MGI S	0.012061
ENSMUSG00000061171	Slc38a11	solute carrier family 38, member 11 [Source:MGI S	0.012071
ENSMUSG00000023973	Cnpy3	canopy 3 homolog (zebrafish) [Source:MGI Symbol;	0.012077
ENSMUSG00000035151	Elmod2	ELMO/CED-12 domain containing 2 [Source:MGI Sy	0.012088
ENSMUSG00000091994	E130317F20Rik	RIKEN cDNA E130317F20 gene [Source:MGI Symbo	0.012102
ENSMUSG00000031024	St5	suppression of tumorigenicity 5 [Source:MGI Symb	0.012111
ENSMUSG00000036097	Fam178a	family with sequence similarity 178, member A [Sou	0.012151
ENSMUSG00000097152	Gm26515	predicted gene, 26515 [Source:MGI Symbol;Acc:M(	0.012203
ENSMUSG00000042508	Dmtf1	cyclin D binding myb-like transcription factor 1 [Sou	0.012207

ENSMUSG00000020607	Fam84a	family with sequence similarity 84, member A [Sou	0.012217
ENSMUSG00000024766	Lipo1	lipase, member O1 [Source:MGI Symbol;Acc:MGI:2	0.012235
ENSMUSG00000000869	Il4	interleukin 4 [Source:MGI Symbol;Acc:MGI:96556]	0.012248
ENSMUSG00000080966	Gm6263	predicted gene 6263 [Source:MGI Symbol;Acc:MGI:	0.012283
ENSMUSG00000097051	Gm26836	predicted gene, 26836 [Source:MGI Symbol;Acc:M	0.012297
ENSMUSG00000033287	Kctd17	potassium channel tetramerisation domain contain	0.012303
ENSMUSG00000031701	Dnaja2	DnaJ (Hsp40) homolog, subfamily A, member 2 [So	0.012311
ENSMUSG00000029594	Rbm19	RNA binding motif protein 19 [Source:MGI Symbol;	0.012323
ENSMUSG00000020150	Gamt	guanidinoacetate methyltransferase [Source:MGI S	0.01236
ENSMUSG00000042198	Chchd7	coiled-coil-helix-coiled-coil-helix domain containing	0.012417
ENSMUSG00000045435	Tmem60	transmembrane protein 60 [Source:MGI Symbol;Ac	0.012428
ENSMUSG00000097828	6430562O15Rik	RIKEN cDNA 6430562O15 gene [Source:MGI Symbo	0.012431
ENSMUSG00000079057	Cyp4v3	cytochrome P450, family 4, subfamily v, polypeptid	0.012445
ENSMUSG00000098135	Gm6946	predicted gene 6946 [Source:MGI Symbol;Acc:MGI:	0.012447
ENSMUSG00000040351	Ankib1	ankyrin repeat and IBR domain containing 1 [Sourc	0.012448
ENSMUSG00000079105	C7	complement component 7 [Source:MGI Symbol;Ac	0.012449
ENSMUSG00000070465	Gm9696	predicted gene 9696 [Source:MGI Symbol;Acc:MGI:	0.012504
ENSMUSG00000075296	Aldh3b2	aldehyde dehydrogenase 3 family, member B2 [Sou	0.012509
ENSMUSG00000090098	Alms1-ps2	Alstrom syndrome 1, pseudogene 2 [Source:MGI Sy	0.012515
ENSMUSG00000087213	2810408I11Rik	RIKEN cDNA 2810408I11 gene [Source:MGI Symbol	0.012518
ENSMUSG00000025899	Alkbh8	alkB, alkylation repair homolog 8 (E. coli) [Source:M	0.012528
ENSMUSG00000043090	Zfp866	zinc finger protein 866 [Source:MGI Symbol;Acc:M	0.012533
ENSMUSG00000045106	Ccdc73	coiled-coil domain containing 73 [Source:MGI Symb	0.012535
ENSMUSG00000020277	Pfkl	phosphofructokinase, liver, B-type [Source:MGI Sym	0.012568
ENSMUSG00000005732	Ranbp1	RAN binding protein 1 [Source:MGI Symbol;Acc:MG	0.012576
ENSMUSG00000051451	Crebzf	CREB/ATF bZIP transcription factor [Source:MGI Sy	0.012606
ENSMUSG00000027230	Creb3l1	cAMP responsive element binding protein 3-like 1 [	0.012626
ENSMUSG00000031673	Cdh11	cadherin 11 [Source:MGI Symbol;Acc:MGI:99217]	0.012646
ENSMUSG00000024404	Riok3	RIO kinase 3 [Source:MGI Symbol;Acc:MGI:191412	0.012665
ENSMUSG00000079487	Med12	mediator of RNA polymerase II transcription, subur	0.012665
ENSMUSG00000040514	4921511H03Rik	RIKEN cDNA 4921511H03 gene [Source:MGI Symbo	0.012683
ENSMUSG00000027901	Dennd2d	DENN/MADD domain containing 2D [Source:MGI S	0.012688
ENSMUSG00000090919	Pabpc4l	poly(A) binding protein, cytoplasmic 4-like [Source:	0.012692
ENSMUSG00000031565	Fgfr1	fibroblast growth factor receptor 1 [Source:MGI Sy	0.01272
ENSMUSG00000028137	Celf3	CUGBP, Elav-like family member 3 [Source:MGI Syn	0.012732
ENSMUSG00000020120	Plek	pleckstrin [Source:MGI Symbol;Acc:MGI:1860485]	0.01274
ENSMUSG00000004843	Chmp2b	charged multivesicular body protein 2B [Source:MG	0.012748
ENSMUSG00000053604	Rpia	ribose 5-phosphate isomerase A [Source:MGI Symb	0.012759
ENSMUSG00000002104	Rapsn	receptor-associated protein of the synapse [Source	0.012784
ENSMUSG00000068523	Gng5	guanine nucleotide binding protein (G protein), gar	0.012803
ENSMUSG00000030589	Rasgrp4	RAS guanyl releasing protein 4 [Source:MGI Symbo	0.012823
ENSMUSG00000029189	Sel1l3	sel-1 suppressor of lin-12-like 3 (C. elegans) [Source	0.012859
ENSMUSG00000036291	Ap5m1	adaptor-related protein complex 5, mu 1 subunit [S	0.012871
ENSMUSG00000084984	1700006P19Rik	RIKEN cDNA 1700006P19 gene [Source:MGI Symbo	0.012892
ENSMUSG00000087612	A230005M16Rik	RIKEN cDNA A230005M16 gene [Source:MGI Symb	0.012922
ENSMUSG00000024889	Rce1	RCE1 homolog, prenyl protein peptidase (S. cerevis	0.012933
ENSMUSG00000034075	Zdhhc5	zinc finger, DHHC domain containing 5 [Source:MG	0.012939

ENSMUSG00000000001	Gnai3	guanine nucleotide binding protein (G protein), alp	0.012946
ENSMUSG00000024070	Prkd3	protein kinase D3 [Source:MGI Symbol;Acc:MGI:19	0.012959
ENSMUSG00000040022	Rab11fip2	RAB11 family interacting protein 2 (class I) [Source:	0.012968
ENSMUSG00000044071	Fam19a2	family with sequence similarity 19, member A2 [Sou	0.012992
ENSMUSG00000025479	Cyp2e1	cytochrome P450, family 2, subfamily e, polypeptid	0.013
ENSMUSG00000008763	Man1a2	mannosidase, alpha, class 1A, member 2 [Source:M	0.013026
ENSMUSG00000074037	Mc1r	melanocortin 1 receptor [Source:MGI Symbol;Acc:M	0.013029
ENSMUSG00000084915	C230037L18Rik	RIKEN cDNA C230037L18 gene [Source:MGI Symbo	0.013029
ENSMUSG00000036840	Siah1a	seven in absentia 1A [Source:MGI Symbol;Acc:MGI:	0.013056
ENSMUSG00000040128	Pnrc1	proline-rich nuclear receptor coactivator 1 [Source:	0.013067
ENSMUSG00000041124	Msantd4	Myb/SANT-like DNA-binding domain containing 4 w	0.013071
ENSMUSG00000054263	Lifr	leukemia inhibitory factor receptor [Source:MGI Sy	0.013096
ENSMUSG00000007050	Lsm2	LSM2 homolog, U6 small nuclear RNA associated (S	0.013097
ENSMUSG00000038774	Ascc3	activating signal cointegrator 1 complex subunit 3 [	0.013128
ENSMUSG00000020396	Nefh	neurofilament, heavy polypeptide [Source:MGI Sym	0.013152
ENSMUSG00000024319	Vps52	vacuolar protein sorting 52 (yeast) [Source:MGI Syr	0.013155
ENSMUSG00000021748	Pdhb	pyruvate dehydrogenase (lipoamide) beta [Source:	0.013163
ENSMUSG00000050229	Pigm	phosphatidylinositol glycan anchor biosynthesis, cla	0.013178
ENSMUSG00000031451	Gas6	growth arrest specific 6 [Source:MGI Symbol;Acc:M	0.013216
ENSMUSG00000030605	Mfge8	milk fat globule-EGF factor 8 protein [Source:MGI S	0.013229
ENSMUSG00000060244	Alyref2	Aly/REF export factor 2 [Source:MGI Symbol;Acc:M	0.013302
ENSMUSG00000083356	Gm11708	predicted gene 11708 [Source:MGI Symbol;Acc:MG	0.013321
ENSMUSG00000034252	Senp6	SUMO/sentrin specific peptidase 6 [Source:MGI Syr	0.013328
ENSMUSG00000028172	Tacr3	tachykinin receptor 3 [Source:MGI Symbol;Acc:MGI	0.013329
ENSMUSG00000026925	Inpp5e	inositol polyphosphate-5-phosphatase E [Source:M	0.013351
ENSMUSG00000004961	Syt5	synaptotagmin V [Source:MGI Symbol;Acc:MGI:192	0.01336
ENSMUSG00000007877	Tcap	titin-cap [Source:MGI Symbol;Acc:MGI:1330233]	0.013392
ENSMUSG00000022564	Grina	glutamate receptor, ionotropic, N-methyl D-asparta	0.013431
ENSMUSG00000021028	Mbip	MAP3K12 binding inhibitory protein 1 [Source:MGI	0.013444
ENSMUSG00000033032	Afap1l1	actin filament associated protein 1-like 1 [Source:M	0.013516
ENSMUSG00000036398	Ppp1r11	protein phosphatase 1, regulatory (inhibitor) subur	0.013534
ENSMUSG00000085819	A930028C08Rik	RIKEN cDNA A930028C08 gene [Source:MGI Symbo	0.013536
ENSMUSG00000070047	Fat1	FAT tumor suppressor homolog 1 (Drosophila) [Sou	0.013544
ENSMUSG00000039997	Ifi203	interferon activated gene 203 [Source:MGI Symbol;	0.013547
ENSMUSG00000028693	Nasp	nuclear autoantigenic sperm protein (histone-bindi	0.013551
ENSMUSG00000027006	Dnajc10	DnaJ (Hsp40) homolog, subfamily C, member 10 [Sc	0.013575
ENSMUSG00000049327	Setd8	SET domain containing (lysine methyltransferase) 8	0.013583
ENSMUSG00000020962	Gtf2a1	general transcription factor II A, 1 [Source:MGI Sym	0.013594
ENSMUSG00000052656	Rnf103	ring finger protein 103 [Source:MGI Symbol;Acc:MC	0.013635
ENSMUSG00000093459	5730480H06Rik	RIKEN cDNA 5730480H06 gene [Source:MGI Symbo	0.013651
ENSMUSG00000031079	Zfp300	zinc finger protein 300 [Source:MGI Symbol;Acc:MC	0.013659
ENSMUSG00000040152	Thbs1	thrombospondin 1 [Source:MGI Symbol;Acc:MGI:98	0.013768
ENSMUSG00000024976	Shoc2	soc-2 (suppressor of clear) homolog (C. elegans) [Sc	0.013785
ENSMUSG00000029919	Hpgds	hematopoietic prostaglandin D synthase [Source:M	0.013799
ENSMUSG00000022837	Iqcb1	IQ calmodulin-binding motif containing 1 [Source:M	0.013816
ENSMUSG00000078622	Ccdc47	coiled-coil domain containing 47 [Source:MGI Symb	0.013887
ENSMUSG00000024395	Lims2	LIM and senescent cell antigen like domains 2 [Sou	0.013891

ENSMUSG00000085583	Gm15676	predicted gene 15676 [Source:MGI Symbol;Acc:MG	0.013912
ENSMUSG00000092464	Gm8902	predicted gene 8902 [Source:MGI Symbol;Acc:MGI:	0.01396
ENSMUSG00000021684	Pde8b	phosphodiesterase 8B [Source:MGI Symbol;Acc:MG	0.013964
ENSMUSG00000043424	Eif3j2	eukaryotic translation initiation factor 3, subunit J2	0.013966
ENSMUSG00000021807	2700060E02Rik	RIKEN cDNA 2700060E02 gene [Source:MGI Symbo	0.01399
ENSMUSG00000030304	Ergic2	ERGIC and golgi 2 [Source:MGI Symbol;Acc:MGI:19	0.013994
ENSMUSG00000023846	Riok2	RIO kinase 2 (yeast) [Source:MGI Symbol;Acc:MGI:1	0.013997
ENSMUSG00000023067	Cdkn1a	cyclin-dependent kinase inhibitor 1A (P21) [Source:	0.014005
ENSMUSG00000018167	Stard3	START domain containing 3 [Source:MGI Symbol;Ac	0.014071
ENSMUSG00000021835	Bmp4	bone morphogenetic protein 4 [Source:MGI Symbo	0.014076
ENSMUSG00000034557	Zfyve9	zinc finger, FYVE domain containing 9 [Source:MGI	0.014077
ENSMUSG00000022025	Lect1	leukocyte cell derived chemotaxin 1 [Source:MGI Sy	0.01411
ENSMUSG00000028399	Ptprd	protein tyrosine phosphatase, receptor type, D [Sou	0.014124
ENSMUSG00000071796	6820431F20Rik	RIKEN cDNA 6820431F20 gene [Source:MGI Symbo	0.014131
ENSMUSG00000036845	Lin37	lin-37 homolog (C. elegans) [Source:MGI Symbol;Ac	0.014184
ENSMUSG00000028681	Ptch2	patched homolog 2 [Source:MGI Symbol;Acc:MGI:1	0.014225
ENSMUSG00000026959	Grin1	glutamate receptor, ionotropic, NMDA1 (zeta 1) [So	0.014267
ENSMUSG00000036225	Kctd1	potassium channel tetramerisation domain contain	0.014289
ENSMUSG00000055737	Ghr	growth hormone receptor [Source:MGI Symbol;Acc	0.014313
ENSMUSG00000040125	Gpr26	G protein-coupled receptor 26 [Source:MGI Symbo	0.014386
ENSMUSG00000030230	Plcz1	phospholipase C, zeta 1 [Source:MGI Symbol;Acc:M	0.014423
ENSMUSG00000021061	Sptb	spectrin beta, erythrocytic [Source:MGI Symbol;Acc	0.014425
ENSMUSG00000083798	Gm14584	predicted gene 14584 [Source:MGI Symbol;Acc:MG	0.014431
ENSMUSG00000018339	Gpx3	glutathione peroxidase 3 [Source:MGI Symbol;Acc:I	0.014456
ENSMUSG00000034480	Diap2	diaphanous homolog 2 (Drosophila) [Source:MGI Sy	0.014471
ENSMUSG00000005233	Spc25	SPC25, NDC80 kinetochore complex component, ho	0.014511
ENSMUSG00000038740	Mvb12b	multivesicular body subunit 12B [Source:MGI Symb	0.014567
ENSMUSG00000031645	F11	coagulation factor XI [Source:MGI Symbol;Acc:MGI	0.014584
ENSMUSG00000031608	Galnt7	UDP-N-acetyl-alpha-D-galactosamine: polypeptide	0.014592
ENSMUSG00000002608	Ccdc97	coiled-coil domain containing 97 [Source:MGI Symb	0.014639
ENSMUSG00000024529	Lox	lysyl oxidase [Source:MGI Symbol;Acc:MGI:96817]	0.014646
ENSMUSG00000091570	Gm7707	predicted gene 7707 [Source:MGI Symbol;Acc:MGI:	0.014646
ENSMUSG00000042258	Isl1	ISL1 transcription factor, LIM/homeodomain [Sourc	0.014663
ENSMUSG00000032400	Zwilch	zwilch kinetochore protein [Source:MGI Symbol;Ac	0.014685
ENSMUSG00000038023	Atp6v0a2	ATPase, H+ transporting, lysosomal V0 subunit A2 [	0.014701
ENSMUSG00000028483	Snapc3	small nuclear RNA activating complex, polypeptide	0.014703
ENSMUSG00000032384	Csnk1g1	casein kinase 1, gamma 1 [Source:MGI Symbol;Acc:	0.014719
ENSMUSG00000029603	Dtx1	deltex 1 homolog (Drosophila) [Source:MGI Symbol	0.014727
ENSMUSG00000027993	Trim2	tripartite motif-containing 2 [Source:MGI Symbol;A	0.014729
ENSMUSG00000022110	Sucla2	succinate-Coenzyme A ligase, ADP-forming, beta su	0.014759
ENSMUSG00000090034	Gm15917	predicted gene 15917 [Source:MGI Symbol;Acc:MG	0.014776
ENSMUSG00000063800	Prpf38a	PRP38 pre-mRNA processing factor 38 (yeast) doma	0.014779
ENSMUSG00000023132	Gzma	granzyme A [Source:MGI Symbol;Acc:MGI:109266]	0.014782
ENSMUSG00000060568	Fam78b	family with sequence similarity 78, member B [Sour	0.014789
ENSMUSG00000050565	Tor1aip2	torsin A interacting protein 2 [Source:MGI Symbol;A	0.014861
ENSMUSG00000022052	Ppp2r2a	protein phosphatase 2 (formerly 2A), regulatory su	0.014868
ENSMUSG00000037818	3110057O12Rik	RIKEN cDNA 3110057O12 gene [Source:MGI Symbo	0.014869

ENSMUSG00000020022	Ndufa12	NADH dehydrogenase (ubiquinone) 1 alpha subcom	0.014888
ENSMUSG00000020364	Zfp354a	zinc finger protein 354A [Source:MGI Symbol;Acc:M	0.014903
ENSMUSG00000005148	Klf5	Kruppel-like factor 5 [Source:MGI Symbol;Acc:MGI:	0.014911
ENSMUSG00000055960	Skint4	selection and upkeep of intraepithelial T cells 4 [Sou	0.014925
ENSMUSG00000022034	Esco2	establishment of cohesion 1 homolog 2 (S. cerevisia	0.015001
ENSMUSG00000089774	Slc5a3	solute carrier family 5 (inositol transporters), meml	0.015006
ENSMUSG00000058407	Txndc9	thioredoxin domain containing 9 [Source:MGI Symb	0.015024
ENSMUSG00000022321	Cdh10	cadherin 10 [Source:MGI Symbol;Acc:MGI:107436]	0.015034
ENSMUSG00000000305	Cdh4	cadherin 4 [Source:MGI Symbol;Acc:MGI:99218]	0.015074
ENSMUSG00000021027	Ralgapa1	Ral GTPase activating protein, alpha subunit 1 [Sou	0.015082
ENSMUSG00000034218	Atm	ataxia telangiectasia mutated homolog (human) [So	0.015089
ENSMUSG00000075420	Smim6	small integral membrane protein 6 [Source:MGI Sy	0.015107
ENSMUSG00000022262	Dnah5	dynein, axonemal, heavy chain 5 [Source:MGI Symb	0.015113
ENSMUSG00000030498	Gas2	growth arrest specific 2 [Source:MGI Symbol;Acc:M	0.015156
ENSMUSG00000046314	Stxbp6	syntaxin binding protein 6 (amisyn) [Source:MGI Sy	0.015159
ENSMUSG00000043391	2510009E07Rik	RIKEN cDNA 2510009E07 gene [Source:MGI Symbo	0.015201
ENSMUSG00000062785	Kcnc3	potassium voltage gated channel, Shaw-related sub	0.015215
ENSMUSG00000048921	Zfp689	zinc finger protein 689 [Source:MGI Symbol;Acc:MG	0.015222
ENSMUSG00000041886	Macc1	metastasis associated in colon cancer 1 [Source:MG	0.015281
ENSMUSG00000030615	Tmem126a	transmembrane protein 126A [Source:MGI Symbol;	0.015305
ENSMUSG00000015653	Steap2	six transmembrane epithelial antigen of prostate 2	0.015312
ENSMUSG00000034473	Sec22a	SEC22 vesicle trafficking protein homolog A (S. cere	0.015318
ENSMUSG00000008489	Elavl2	ELAV (embryonic lethal, abnormal vision, Drosophil	0.015343
ENSMUSG00000003354	Ccdc65	coiled-coil domain containing 65 [Source:MGI Symb	0.015376
ENSMUSG00000095241	Gm5478	predicted pseudogene 5478 [Source:MGI Symbol;A	0.01539
ENSMUSG00000041777	Cir1	corepressor interacting with RBPJ, 1 [Source:MGI Sy	0.015414
ENSMUSG00000020349	Ppp2ca	protein phosphatase 2 (formerly 2A), catalytic subu	0.015438
ENSMUSG00000025551	Fgf14	fibroblast growth factor 14 [Source:MGI Symbol;Ac	0.015439
ENSMUSG00000021640	Naip1	NLR family, apoptosis inhibitory protein 1 [Source:M	0.01544
ENSMUSG00000097777	Gm16794	predicted gene, 16794 [Source:MGI Symbol;Acc:MG	0.015454
ENSMUSG00000025930	Msc	musculin [Source:MGI Symbol;Acc:MGI:1333884]	0.015471
ENSMUSG00000064493	Snora28	small nucleolar RNA, H/ACA box 28 [Source:MGI Sy	0.015528
ENSMUSG00000034459	Ifit1	interferon-induced protein with tetratricopeptide r	0.015542
ENSMUSG00000097164	Ccdc41os1	Ccdc41 opposite strand 1 [Source:MGI Symbol;Acc:	0.015544
ENSMUSG00000024695	Zfp91	zinc finger protein 91 [Source:MGI Symbol;Acc:MGI	0.015561
ENSMUSG00000041247	Lamp3	lysosomal-associated membrane protein 3 [Source:	0.015594
ENSMUSG00000028760	Eif4g3	eukaryotic translation initiation factor 4 gamma, 3	0.015596
ENSMUSG00000039735	Fnbp1l	formin binding protein 1-like [Source:MGI Symbol;A	0.015601
ENSMUSG00000091091	Kcnmb3	potassium large conductance calcium-activated cha	0.015625
ENSMUSG00000028069	Gpatch4	G patch domain containing 4 [Source:MGI Symbol;A	0.015628
ENSMUSG00000050953	Gja1	gap junction protein, alpha 1 [Source:MGI Symbol;A	0.015647
ENSMUSG00000003452	Bicd1	bicaudal D homolog 1 (Drosophila) [Source:MGI Syr	0.015673
ENSMUSG00000024774	Ankrd22	ankyrin repeat domain 22 [Source:MGI Symbol;Acc	0.015688
ENSMUSG00000031103	Elf4	E74-like factor 4 (ets domain transcription factor) [S	0.015737
ENSMUSG00000042655	Fam159b	family with sequence similarity 159, member B [Sou	0.015759
ENSMUSG00000020019	Ntn4	netrin 4 [Source:MGI Symbol;Acc:MGI:1888978]	0.015787
ENSMUSG00000050587	Lrrc4c	leucine rich repeat containing 4C [Source:MGI Symb	0.015809

ENSMUSG00000000303	Cdh1	cadherin 1 [Source:MGI Symbol;Acc:MGI:88354]	0.015832
ENSMUSG00000097103	Gm2885	predicted gene 2885 [Source:MGI Symbol;Acc:MGI:	0.015883
ENSMUSG00000074994	Qser1	glutamine and serine rich 1 [Source:MGI Symbol;Ac	0.015956
ENSMUSG00000030782	Tgfb1i1	transforming growth factor beta 1 induced transcri	0.015971
ENSMUSG00000073987	Ggh	gamma-glutamyl hydrolase [Source:MGI Symbol;Ac	0.015978
ENSMUSG00000092260	Zfp963	zinc finger protein 963 [Source:MGI Symbol;Acc:MG	0.016
ENSMUSG00000025401	Myo1a	myosin IA [Source:MGI Symbol;Acc:MGI:107732]	0.016028
ENSMUSG00000051579	Tceal8	transcription elongation factor A (SII)-like 8 [Source	0.016032
ENSMUSG00000025185	Loxl4	lysyl oxidase-like 4 [Source:MGI Symbol;Acc:MGI:19	0.016076
ENSMUSG00000040212	Emp3	epithelial membrane protein 3 [Source:MGI Symbo	0.016093
ENSMUSG00000035762	Tmem161b	transmembrane protein 161B [Source:MGI Symbol;	0.016109
ENSMUSG00000044712	Slc38a6	solute carrier family 38, member 6 [Source:MGI Syr	0.01612
ENSMUSG00000012126	Ubxn11	UBX domain protein 11 [Source:MGI Symbol;Acc:M	0.016127
ENSMUSG00000037379	Spon2	spondin 2, extracellular matrix protein [Source:MG	0.016129
ENSMUSG00000003161	Sri	sorcin [Source:MGI Symbol;Acc:MGI:98419]	0.016164
ENSMUSG00000095139	Pou3f2	POU domain, class 3, transcription factor 2 [Source	0.016164
ENSMUSG00000002504	Slc9a3r2	solute carrier family 9 (sodium/hydrogen exchange	0.016201
ENSMUSG00000049536	Tceal1	transcription elongation factor A (SII)-like 1 [Source	0.016212
ENSMUSG00000050520	Cldn8	claudin 8 [Source:MGI Symbol;Acc:MGI:1859286]	0.016269
ENSMUSG00000026986	Hnmt	histamine N-methyltransferase [Source:MGI Symbo	0.016308
ENSMUSG00000049630	C1ql3	C1q-like 3 [Source:MGI Symbol;Acc:MGI:2387350]	0.016373
ENSMUSG00000025793	Hgs	HGF-regulated tyrosine kinase substrate [Source:M	0.016431
ENSMUSG00000023170	Gps2	G protein pathway suppressor 2 [Source:MGI Symb	0.016474
ENSMUSG00000025076	Casp7	caspase 7 [Source:MGI Symbol;Acc:MGI:109383]	0.016483
ENSMUSG00000036270	Edc4	enhancer of mRNA decapping 4 [Source:MGI Symbo	0.016513
ENSMUSG00000006007	Pdc	phosducin [Source:MGI Symbol;Acc:MGI:98090]	0.016519
ENSMUSG00000067878	Mtap7d3	MAP7 domain containing 3 [Source:MGI Symbol;Ac	0.016534
ENSMUSG00000075217	4833423E24Rik	RIKEN cDNA 4833423E24 gene [Source:MGI Symbo	0.016536
ENSMUSG00000039852	Rere	arginine glutamic acid dipeptide (RE) repeats [Sourc	0.016608
ENSMUSG00000055296	Tmem245	transmembrane protein 245 [Source:MGI Symbol;A	0.016623
ENSMUSG00000021589	Rhobtb3	Rho-related BTB domain containing 3 [Source:MGI	0.016645
ENSMUSG00000031007	Atp6ap2	ATPase, H+ transporting, lysosomal accessory prote	0.01667
ENSMUSG00000078143	Gm17344	predicted gene, 17344 [Source:MGI Symbol;Acc:MG	0.016688
ENSMUSG00000056531	Ccdc18	coiled-coil domain containing 18 [Source:MGI Symb	0.016708
ENSMUSG00000033342	4833424O15Rik	RIKEN cDNA 4833424O15 gene [Source:MGI Symbo	0.016719
ENSMUSG00000071637	Cebpd	CCAAT/enhancer binding protein (C/EBP), delta [So	0.016789
ENSMUSG00000015890	Amdhd1	amidohydrolase domain containing 1 [Source:MGI S	0.016815
ENSMUSG00000004591	Pkn2	protein kinase N2 [Source:MGI Symbol;Acc:MGI:10	0.016866
ENSMUSG00000027202	Slc12a1	solute carrier family 12, member 1 [Source:MGI Syr	0.016875
ENSMUSG00000028098	Rnf115	ring finger protein 115 [Source:MGI Symbol;Acc:MG	0.016876
ENSMUSG00000024691	Fam111a	family with sequence similarity 111, member A [Sou	0.016901
ENSMUSG00000029245	Epha5	Eph receptor A5 [Source:MGI Symbol;Acc:MGI:996	0.016931
ENSMUSG00000072568	Fam84b	family with sequence similarity 84, member B [Sour	0.016961
ENSMUSG00000035783	Acta2	actin, alpha 2, smooth muscle, aorta [Source:MGI S	0.016968
ENSMUSG00000096935	1700113A16Rik	RIKEN cDNA 1700113A16 gene [Source:MGI Symbo	0.017002
ENSMUSG00000092495	Gm20405	predicted gene 20405 [Source:MGI Symbol;Acc:MG	0.017087
ENSMUSG00000047187	Rab2a	RAB2A, member RAS oncogene family [Source:MGI	0.017146

ENSMUSG00000097211	BC065403	cDNA sequence BC065403 [Source:MGI Symbol;Acc	0.017159
ENSMUSG00000060227	Casc4	cancer susceptibility candidate 4 [Source:MGI Symb	0.01716
ENSMUSG00000039278	Pcsk1n	proprotein convertase subtilisin/kexin type 1 inhibi	0.017174
ENSMUSG00000059895	Ptp4a3	protein tyrosine phosphatase 4a3 [Source:MGI Sym	0.017216
ENSMUSG00000097123	Gm6297	predicted gene 6297 [Source:MGI Symbol;Acc:MGI:	0.017261
ENSMUSG00000024913	Lrp5	low density lipoprotein receptor-related protein 5 [	0.017271
ENSMUSG00000073672	Rnf223	ring finger 223 [Source:MGI Symbol;Acc:MGI:35881	0.017279
ENSMUSG00000071041	Gm15210	predicted gene 15210 [Source:MGI Symbol;Acc:MG	0.017326
ENSMUSG00000006800	Sulf2	sulfatase 2 [Source:MGI Symbol;Acc:MGI:1919293]	0.017343
ENSMUSG00000060950	Trmt61a	tRNA methyltransferase 61A [Source:MGI Symbol;A	0.01735
ENSMUSG00000054909	Wbscr25	Williams Beuren syndrome chromosome region 25	0.017389
ENSMUSG00000030249	Abcc9	ATP-binding cassette, sub-family C (CFTR/MRP), me	0.017436
ENSMUSG00000007837	Prrg2	proline-rich Gla (G-carboxyglutamic acid) polypepti	0.017438
ENSMUSG00000032292	Nr2e3	nuclear receptor subfamily 2, group E, member 3 [S	0.017449
ENSMUSG00000054057	A930004D18Rik	RIKEN cDNA A930004D18 gene [Source:MGI Symbo	0.017479
ENSMUSG00000031352	Hccs	holocytochrome c synthetase [Source:MGI Symbol;	0.017483
ENSMUSG00000008604	Ubqln4	ubiquilin 4 [Source:MGI Symbol;Acc:MGI:2150152]	0.017505
ENSMUSG00000046275	Tusc5	tumor suppressor candidate 5 [Source:MGI Symbol	0.017513
ENSMUSG00000030064	Frmd4b	FERM domain containing 4B [Source:MGI Symbol;A	0.017521
ENSMUSG00000032087	Dscaml1	Down syndrome cell adhesion molecule like 1 [Sour	0.017581
ENSMUSG00000036501	Fam13b	family with sequence similarity 13, member B [Sour	0.017581
ENSMUSG00000000563	Atp5f1	ATP synthase, H+ transporting, mitochondrial F0 co	0.017588
ENSMUSG00000046470	Sox18	SRY-box containing gene 18 [Source:MGI Symbol;Ac	0.01761
ENSMUSG00000059273	Zc3h4	zinc finger CCCH-type containing 4 [Source:MGI Syn	0.017611
ENSMUSG00000088185	Scarna2	small Cajal body-specific RNA 2 [Source:MGI Symbo	0.017615
ENSMUSG00000084884	Gm12289	predicted gene 12289 [Source:MGI Symbol;Acc:MG	0.017616
ENSMUSG00000085093	Gm12737	predicted gene 12737 [Source:MGI Symbol;Acc:MG	0.017635
ENSMUSG00000089979	Gm15954	predicted gene 15954 [Source:MGI Symbol;Acc:MG	0.017639
ENSMUSG00000074922	Fam122a	family with sequence similarity 122, member A [Sou	0.01766
ENSMUSG00000066150	Slc31a1	solute carrier family 31, member 1 [Source:MGI Syn	0.01767
ENSMUSG00000049092	Gpr137c	G protein-coupled receptor 137C [Source:MGI Sym	0.017738
ENSMUSG00000025482	Odf3	outer dense fiber of sperm tails 3 [Source:MGI Sym	0.017741
ENSMUSG00000025665	Rps6ka6	ribosomal protein S6 kinase polypeptide 6 [Source:	0.017751
ENSMUSG00000039195	1110008P14Rik	RIKEN cDNA 1110008P14 gene [Source:MGI Symbo	0.017754
ENSMUSG00000035203	Epn1	epsin 1 [Source:MGI Symbol;Acc:MGI:1333763]	0.017762
ENSMUSG00000024085	Man2a1	mannosidase 2, alpha 1 [Source:MGI Symbol;Acc:M	0.017784
ENSMUSG00000003271	Sult2b1	sulfotransferase family, cytosolic, 2B, member 1 [So	0.017798
ENSMUSG00000084797	Gm14321	predicted gene 14321 [Source:MGI Symbol;Acc:MG	0.017807
ENSMUSG00000074211	Sdhaf1	succinate dehydrogenase complex assembly factor	0.017808
ENSMUSG00000046865	Fbl	fibrillarin [Source:MGI Symbol;Acc:MGI:95486]	0.017833
ENSMUSG00000097666	A330094K24Rik	RIKEN cDNA A330094K24 gene [Source:MGI Symbo	0.017886
ENSMUSG00000030423	Pop4	processing of precursor 4, ribonuclease P/MRP fam	0.01789
ENSMUSG00000026426	Arl8a	ADP-ribosylation factor-like 8A [Source:MGI Symbo	0.017901
ENSMUSG00000023064	Sncg	synuclein, gamma [Source:MGI Symbol;Acc:MGI:12	0.017917
ENSMUSG00000040907	Atp1a3	ATPase, Na+/K+ transporting, alpha 3 polypeptide [	0.017935
ENSMUSG00000025400	Tac2	tachykinin 2 [Source:MGI Symbol;Acc:MGI:98476]	0.017985
ENSMUSG00000050621	Gm9846	predicted gene 9846 [Source:MGI Symbol;Acc:MGI:	0.017987

ENSMUSG00000085263	1700125G02Rik	RIKEN cDNA 1700125G02 gene [Source:MGI Symbo	0.01799
ENSMUSG00000094651	Gal3st2	galactose-3-O-sulfotransferase 2 [Source:MGI Symb	0.01801
ENSMUSG00000041417	Pik3r1	phosphatidylinositol 3-kinase, regulatory subunit, p	0.018013
ENSMUSG00000024601	Isoc1	isochorismatase domain containing 1 [Source:MGI	0.018039
ENSMUSG00000036295	Lrrn3	leucine rich repeat protein 3, neuronal [Source:MG	0.018041
ENSMUSG00000089901	Gm8113	predicted gene 8113 [Source:MGI Symbol;Acc:MGI:	0.018051
ENSMUSG00000032570	Atp2c1	ATPase, Ca++-sequestering [Source:MGI Symbol;Ac	0.018055
ENSMUSG00000013367	Iglon5	IgLON family member 5 [Source:MGI Symbol;Acc:M	0.018075
ENSMUSG00000020887	A230052G05Rik	RIKEN cDNA A230052G05 gene [Source:MGI Symbo	0.018197
ENSMUSG00000025374	Nabp2	nucleic acid binding protein 2 [Source:MGI Symbol;	0.018231
ENSMUSG00000042148	Cox10	cytochrome c oxidase assembly protein 10 [Source:	0.018291
ENSMUSG00000028251	Tstd3	thiosulfate sulfurtransferase (rhodanese)-like doma	0.01833
ENSMUSG00000087228	Gm12827	predicted gene 12827 [Source:MGI Symbol;Acc:MG	0.018349
ENSMUSG00000090546	Cdr1	cerebellar degeneration related antigen 1 [Source:M	0.018359
ENSMUSG00000039844	Rapgef1	Rap guanine nucleotide exchange factor (GEF) 1 [So	0.018379
ENSMUSG00000032511	Scn5a	sodium channel, voltage-gated, type V, alpha [Sour	0.018426
ENSMUSG00000036905	C1qb	complement component 1, q subcomponent, beta	0.018435
ENSMUSG00000046404	Yod1	YOD1 OTU deubiquitinating enzyme 1 homologue (	0.018448
ENSMUSG00000041649	Klf8	Kruppel-like factor 8 [Source:MGI Symbol;Acc:MGI:	0.018494
ENSMUSG00000029544	Cabp1	calcium binding protein 1 [Source:MGI Symbol;Acc:	0.018516
ENSMUSG00000021111	Papola	poly (A) polymerase alpha [Source:MGI Symbol;Acc	0.018535
ENSMUSG00000057176	Gm166	predicted gene 166 [Source:MGI Symbol;Acc:MGI:2	0.018553
ENSMUSG00000062115	Rai1	retinoic acid induced 1 [Source:MGI Symbol;Acc:MG	0.018578
ENSMUSG00000065362	Gm24411	predicted gene, 24411 [Source:MGI Symbol;Acc:MG	0.018608
ENSMUSG00000084207	Gm14070	predicted gene 14070 [Source:MGI Symbol;Acc:MG	0.018611
ENSMUSG00000028300	3110043O21Rik	RIKEN cDNA 3110043O21 gene [Source:MGI Symbo	0.018626
ENSMUSG00000035890	Rnf126	ring finger protein 126 [Source:MGI Symbol;Acc:MG	0.018662
ENSMUSG00000040738	Ints8	integrator complex subunit 8 [Source:MGI Symbol;A	0.018668
ENSMUSG00000056629	Fkbp2	FK506 binding protein 2 [Source:MGI Symbol;Acc:M	0.018677
ENSMUSG00000060257	Scrt2	scratch homolog 2, zinc finger protein (Drosophila)	0.018684
ENSMUSG00000000317	Bcl6b	B cell CLL/lymphoma 6, member B [Source:MGI Syn	0.018686
ENSMUSG00000006442	Srm	spermidine synthase [Source:MGI Symbol;Acc:MGI	0.018766
ENSMUSG00000004677	Myo9b	myosin IXb [Source:MGI Symbol;Acc:MGI:106624]	0.018788
ENSMUSG00000062760	1810041L15Rik	RIKEN cDNA 1810041L15 gene [Source:MGI Symbo	0.018806
ENSMUSG00000024101	Wash	WAS protein family homolog [Source:MGI Symbol;A	0.018829
ENSMUSG00000052512	Nav2	neuron navigator 2 [Source:MGI Symbol;Acc:MGI:2	0.018841
ENSMUSG00000081648	Gm13423	predicted gene 13423 [Source:MGI Symbol;Acc:MG	0.018912
ENSMUSG00000092349	BC033916	cDNA sequence BC033916 [Source:MGI Symbol;Acc	0.018978
ENSMUSG00000034610	Zcchc11	zinc finger, CCHC domain containing 11 [Source:MG	0.019029
ENSMUSG00000032855	Pkd1	polycystic kidney disease 1 homolog [Source:MGI S	0.019048
ENSMUSG00000022865	Cxadr	coxsackie virus and adenovirus receptor [Source:M	0.01905
ENSMUSG00000021638	Ocln	occludin [Source:MGI Symbol;Acc:MGI:106183]	0.019091
ENSMUSG00000052276	Ostn	osteocrin [Source:MGI Symbol;Acc:MGI:2677164]	0.019099
ENSMUSG00000039100	6-Mar	membrane-associated ring finger (C3HC4) 6 [Source	0.019109
ENSMUSG00000032561	Acpp	acid phosphatase, prostate [Source:MGI Symbol;Ac	0.019154
ENSMUSG00000033633	Clec18a	C-type lectin domain family 18, member A [Source:	0.019161
ENSMUSG00000040525	Cblc	Casitas B-lineage lymphoma c [Source:MGI Symbol;	0.019165

ENSMUSG00000039477	Tnrc18	trinucleotide repeat containing 18 [Source:MGI Syn	0.019184
ENSMUSG00000006362	Cbfa2t3	core-binding factor, runt domain, alpha subunit 2, t	0.019206
ENSMUSG00000056459	Zbtb25	zinc finger and BTB domain containing 25 [Source:M	0.019277
ENSMUSG00000037795	N4bp2	NEDD4 binding protein 2 [Source:MGI Symbol;Acc:I	0.019278
ENSMUSG00000020014	Gm872	RIKEN cDNA 4930485B16 gene [Source:MGI Symbo	0.019303
ENSMUSG00000025144	Stra13	stimulated by retinoic acid 13 [Source:MGI Symbol;	0.019316
ENSMUSG00000027221	Chst1	carbohydrate (keratan sulfate Gal-6) sulfotransfera	0.019346
ENSMUSG00000021700	Rab3c	RAB3C, member RAS oncogene family [Source:MGI	0.019363
ENSMUSG00000048489	8430408G22Rik	RIKEN cDNA 8430408G22 gene [Source:MGI Symbo	0.019402
ENSMUSG00000078772	Gm12353	predicted gene 12353 [Source:MGI Symbol;Acc:MG	0.019493
ENSMUSG00000071103	1700029J07Rik	RIKEN cDNA 1700029J07 gene [Source:MGI Symbol	0.019501
ENSMUSG00000076281	Gm24270	predicted gene, 24270 [Source:MGI Symbol;Acc:MG	0.019513
ENSMUSG00000022900	Ildr1	immunoglobulin-like domain containing receptor 1	0.019527
ENSMUSG00000034656	Cacna1a	calcium channel, voltage-dependent, P/Q type, alp	0.019554
ENSMUSG00000079427	Gm2382	predicted gene 2382 [Source:MGI Symbol;Acc:MGI:	0.019646
ENSMUSG00000067869	Tcea1-ps1	transcription elongation factor A (SII) 1, pseudogen	0.019702
ENSMUSG00000024384	Iws1	IWS1 homolog (S. cerevisiae) [Source:MGI Symbol;A	0.019705
ENSMUSG00000070473	Cldn3	claudin 3 [Source:MGI Symbol;Acc:MGI:1329044]	0.019796
ENSMUSG00000020420	Zfp607	zinc finger proten 607 [Source:MGI Symbol;Acc:MG	0.01984
ENSMUSG00000056481	Cd248	CD248 antigen, endosialin [Source:MGI Symbol;Acc	0.01984
ENSMUSG00000085982	9530051G07Rik	RIKEN cDNA 9530051G07 gene [Source:MGI Symbo	0.019852
ENSMUSG00000029860	Zyx	zyxin [Source:MGI Symbol;Acc:MGI:103072]	0.019853
ENSMUSG00000055799	Tcf7l1	transcription factor 7 like 1 (T cell specific, HMG bo	0.019874
ENSMUSG00000025950	Idh1	isocitrate dehydrogenase 1 (NADP+), soluble [Sourc	0.019883
ENSMUSG00000034793	G6pc3	glucose 6 phosphatase, catalytic, 3 [Source:MGI Syr	0.019897
ENSMUSG00000034177	Rnf43	ring finger protein 43 [Source:MGI Symbol;Acc:MG	0.019933
ENSMUSG00000023988	Bysl	bystin-like [Source:MGI Symbol;Acc:MGI:1858419]	0.019941
ENSMUSG00000028433	Ubap2	ubiquitin-associated protein 2 [Source:MGI Symbol	0.019954
ENSMUSG00000020273	Papolg	poly(A) polymerase gamma [Source:MGI Symbol;Ac	0.019968
ENSMUSG00000042390	Gatad2b	GATA zinc finger domain containing 2B [Source:MG	0.019971
ENSMUSG00000026782	Abi2	abl-interactor 2 [Source:MGI Symbol;Acc:MGI:1069	0.019972
ENSMUSG00000029486	Mrpl1	mitochondrial ribosomal protein L1 [Source:MGI Sy	0.019994
ENSMUSG00000033722	BC034090	cDNA sequence BC034090 [Source:MGI Symbol;Acc	0.020002
ENSMUSG00000069135	Fgfr1op	Fgfr1 oncogene partner [Source:MGI Symbol;Acc:M	0.020029
ENSMUSG00000028991	Mtor	mechanistic target of rapamycin (serine/threonine	0.020045
ENSMUSG00000037643	Prkci	protein kinase C, iota [Source:MGI Symbol;Acc:MGI	0.020117
ENSMUSG00000046675	Tmem251	transmembrane protein 251 [Source:MGI Symbol;A	0.020121
ENSMUSG00000073656	Gm10558	predicted gene 10558 [Source:MGI Symbol;Acc:MG	0.020127
ENSMUSG00000002996	Hbp1	high mobility group box transcription factor 1 [Sour	0.020175
ENSMUSG00000045180	Shroom2	shroom family member 2 [Source:MGI Symbol;Acc:	0.020177
ENSMUSG00000074397	Foxr1	forkhead box R1 [Source:MGI Symbol;Acc:MGI:268	0.02018
ENSMUSG00000042190	Cmklr1	chemokine-like receptor 1 [Source:MGI Symbol;Acc	0.020194
ENSMUSG00000020723	Cacng4	calcium channel, voltage-dependent, gamma subur	0.020216
ENSMUSG00000048399	Tprg	transformation related protein 63 regulated [Sourc	0.020264
ENSMUSG00000042042	Csgalnact2	chondroitin sulfate N-acetylgalactosaminyltransfera	0.020282
ENSMUSG00000010601	Apol7a	apolipoprotein L 7a [Source:MGI Symbol;Acc:MGI:1	0.020301
ENSMUSG00000028127	Abcd3	ATP-binding cassette, sub-family D (ALD), member	0.020318

ENSMUSG00000026779	Mastl	microtubule associated serine/threonine kinase-lik	0.020394
ENSMUSG00000036371	Serbp1	serpine1 mRNA binding protein 1 [Source:MGI Sym	0.020411
ENSMUSG00000031376	Atp2b3	ATPase, Ca++ transporting, plasma membrane 3 [S	0.020427
ENSMUSG00000024975	Pdcd4	programmed cell death 4 [Source:MGI Symbol;Acc:	0.020469
ENSMUSG00000043410	Hfm1	HFM1, ATP-dependent DNA helicase homolog (S. c	0.020508
ENSMUSG00000023919	Cenpq	centromere protein Q [Source:MGI Symbol;Acc:MG	0.020531
ENSMUSG00000090946	Ccdc71l	coiled-coil domain containing 71 like [Source:MGI S	0.020549
ENSMUSG00000036438	Calm2	calmodulin 2 [Source:MGI Symbol;Acc:MGI:103250	0.020593
ENSMUSG00000044927	H1fx	H1 histone family, member X [Source:MGI Symbol;	0.020601
ENSMUSG00000037979	Ccdc92	coiled-coil domain containing 92 [Source:MGI Symb	0.020661
ENSMUSG00000022483	Col2a1	collagen, type II, alpha 1 [Source:MGI Symbol;Acc:N	0.020674
ENSMUSG00000068617	Efcab1	EF hand calcium binding domain 1 [Source:MGI Syn	0.020738
ENSMUSG00000082935	Gm7658	predicted gene 7658 [Source:MGI Symbol;Acc:MGI:	0.020755
ENSMUSG00000052727	Map1b	microtubule-associated protein 1B [Source:MGI Sy	0.020756
ENSMUSG00000006219	Fblim1	filamin binding LIM protein 1 [Source:MGI Symbol;	0.020791
ENSMUSG00000039633	Lonrf1	LON peptidase N-terminal domain and ring finger 1	0.020805
ENSMUSG00000030921	Trim30a	tripartite motif-containing 30A [Source:MGI Symbo	0.020826
ENSMUSG00000017723	Wfdc2	WAP four-disulfide core domain 2 [Source:MGI Sym	0.020834
ENSMUSG00000089768	Tmsb15b1	thymosin beta 15b1 [Source:MGI Symbol;Acc:MGI:	0.020857
ENSMUSG00000037234	Hook3	hook homolog 3 (Drosophila) [Source:MGI Symbol;	0.020862
ENSMUSG00000033382	Trappc8	trafficking protein particle complex 8 [Source:MGI	0.020878
ENSMUSG00000033676	Gabrb3	gamma-aminobutyric acid (GABA) A receptor, subu	0.02099
ENSMUSG00000062151	Unc13c	unc-13 homolog C (C. elegans) [Source:MGI Symbol	0.021004
ENSMUSG00000041607	Mbp	myelin basic protein [Source:MGI Symbol;Acc:MGI:	0.021028
ENSMUSG00000039519	Cyp7b1	cytochrome P450, family 7, subfamily b, polypeptid	0.021071
ENSMUSG00000025764	Phf17	PHD finger protein 17 [Source:MGI Symbol;Acc:MG	0.021077
ENSMUSG00000022844	Pdia5	protein disulfide isomerase associated 5 [Source:M	0.021084
ENSMUSG00000021065	Fut8	fucosyltransferase 8 [Source:MGI Symbol;Acc:MGI:	0.021089
ENSMUSG00000074847	Gm10775	predicted gene 10775 [Source:MGI Symbol;Acc:MG	0.021104
ENSMUSG00000082189	Gm4784	predicted gene 4784 [Source:MGI Symbol;Acc:MGI:	0.021122
ENSMUSG00000026088	Mitd1	MIT, microtubule interacting and transport, domai	0.021125
ENSMUSG00000003824	Syce2	synaptonemal complex central element protein 2 [	0.021129
ENSMUSG00000052459	Atp6v1a	ATPase, H+ transporting, lysosomal V1 subunit A [S	0.021157
ENSMUSG00000022463	Srebf2	sterol regulatory element binding factor 2 [Source:	0.0212
ENSMUSG00000035914	Cd276	CD276 antigen [Source:MGI Symbol;Acc:MGI:2183	0.021204
ENSMUSG00000032334	Loxl1	lysyl oxidase-like 1 [Source:MGI Symbol;Acc:MGI:1	0.021212
ENSMUSG00000020589	Fam49a	family with sequence similarity 49, member A [Sou	0.02123
ENSMUSG00000043430	Psapl1	prosaposin-like 1 [Source:MGI Symbol;Acc:MGI:192	0.021235
ENSMUSG00000009487	Otog	otogelin [Source:MGI Symbol;Acc:MGI:1202064]	0.021246
ENSMUSG00000038379	Ttk	Ttk protein kinase [Source:MGI Symbol;Acc:MGI:11	0.021269
ENSMUSG00000027737	Slc7a11	solute carrier family 7 (cationic amino acid transpo	0.021286
ENSMUSG00000035365	Parpbp	PARP1 binding protein [Source:MGI Symbol;Acc:M	0.021324
ENSMUSG00000076441	Ass1	argininosuccinate synthetase 1 [Source:MGI Symbo	0.021329
ENSMUSG00000026163	Sphkap	SPHK1 interactor, AKAP domain containing [Source	0.021332
ENSMUSG00000032578	Cish	cytokine inducible SH2-containing protein [Source:	0.021398
ENSMUSG00000052539	Magi3	membrane associated guanylate kinase, WW and P	0.021402
ENSMUSG00000042473	Tbc1d8b	TBC1 domain family, member 8B [Source:MGI Sym	0.021424

ENSMUSG00000031628	Casp3	caspase 3 [Source:MGI Symbol;Acc:MGI:107739]	0.021447
ENSMUSG00000021728	Emb	embigin [Source:MGI Symbol;Acc:MGI:95321]	0.021462
ENSMUSG00000052748	Swt1	SWT1 RNA endoribonuclease homolog (S. cerevisia	0.021473
ENSMUSG00000078808	Vmn1r58	vomeronasal 1 receptor 58 [Source:MGI Symbol;Ac	0.021503
ENSMUSG00000051234	Rnf7	ring finger protein 7 [Source:MGI Symbol;Acc:MGI:	0.02153
ENSMUSG00000048004	Tmem196	transmembrane protein 196 [Source:MGI Symbol;A	0.021697
ENSMUSG00000026864	Hspa5	heat shock protein 5 [Source:MGI Symbol;Acc:MGI:	0.021699
ENSMUSG00000032420	Nt5e	5' nucleotidase, ecto [Source:MGI Symbol;Acc:MGI	0.021699
ENSMUSG00000085037	4933421O10Rik	RIKEN cDNA 4933421O10 gene [Source:MGI Symbo	0.021723
ENSMUSG00000026395	Ptprc	protein tyrosine phosphatase, receptor type, C [Sou	0.021727
ENSMUSG00000021719	Rgs7bp	regulator of G-protein signalling 7 binding protein [	0.021728
ENSMUSG00000029402	Snrnp35	small nuclear ribonucleoprotein 35 (U11/U12) [Sou	0.021747
ENSMUSG00000079509	Zfx	zinc finger protein X-linked [Source:MGI Symbol;Ac	0.021832
ENSMUSG00000050914	Ankrd37	ankyrin repeat domain 37 [Source:MGI Symbol;Acc	0.021855
ENSMUSG00000051920	Rspo2	R-spondin 2 homolog (Xenopus laevis) [Source:MGI	0.021897
ENSMUSG00000028268	Gbp3	guanylate binding protein 3 [Source:MGI Symbol;A	0.021929
ENSMUSG00000030835	Nomo1	nodal modulator 1 [Source:MGI Symbol;Acc:MGI:2	0.021929
ENSMUSG00000048617	Rtbdn	retbindin [Source:MGI Symbol;Acc:MGI:2443686]	0.021974
ENSMUSG00000085287	4833418N02Rik	RIKEN cDNA 4833418N02 gene [Source:MGI Symbo	0.022021
ENSMUSG00000024640	Psat1	phosphoserine aminotransferase 1 [Source:MGI Sy	0.022062
ENSMUSG00000004535	Tax1bp1	Tax1 (human T cell leukemia virus type I) binding pr	0.022088
ENSMUSG00000039835	Nhsl1	NHS-like 1 [Source:MGI Symbol;Acc:MGI:106390]	0.02211
ENSMUSG00000026833	Olfm1	olfactomedin 1 [Source:MGI Symbol;Acc:MGI:1860	0.02215
ENSMUSG00000024795	Kif20b	kinesin family member 20B [Source:MGI Symbol;Ac	0.022219
ENSMUSG00000051224	Tceanc	transcription elongation factor A (SII) N-terminal ar	0.022244
ENSMUSG00000061689	Dlgap4	discs, large homolog-associated protein 4 (Drosoph	0.022255
ENSMUSG00000074024	4632427E13Rik	RIKEN cDNA 4632427E13 gene [Source:MGI Symbo	0.022264
ENSMUSG00000019699	Akt3	thymoma viral proto-oncogene 3 [Source:MGI Sym	0.022436
ENSMUSG00000074149	Gm10634	predicted gene 10634 [Source:MGI Symbol;Acc:MG	0.022472
ENSMUSG00000026712	Mrc1	mannose receptor, C type 1 [Source:MGI Symbol;A	0.022478
ENSMUSG00000038991	Txndc5	thioredoxin domain containing 5 [Source:MGI Symb	0.022491
ENSMUSG00000052040	Klf13	Kruppel-like factor 13 [Source:MGI Symbol;Acc:MG	0.02255
ENSMUSG00000024260	Sap130	Sin3A associated protein [Source:MGI Symbol;Acc:I	0.022566
ENSMUSG00000042763	Maneal	mannosidase, endo-alpha-like [Source:MGI Symbol	0.022592
ENSMUSG00000000078	Klf6	Kruppel-like factor 6 [Source:MGI Symbol;Acc:MGI:	0.022631
ENSMUSG00000022323	Hrsp12	heat-responsive protein 12 [Source:MGI Symbol;Ac	0.022652
ENSMUSG00000039323	Igfbp2	insulin-like growth factor binding protein 2 [Source	0.022664
ENSMUSG00000006205	Htra1	HtrA serine peptidase 1 [Source:MGI Symbol;Acc:M	0.022706
ENSMUSG00000047731	Wbp1l	WW domain binding protein 1 like [Source:MGI Syr	0.022725
ENSMUSG00000034636	Zyg11b	zyg-ll family member B, cell cycle regulator [Source	0.022795
ENSMUSG00000039697	Ncoa7	nuclear receptor coactivator 7 [Source:MGI Symbol	0.022884
ENSMUSG00000022641	Bbx	bobby sox homolog (Drosophila) [Source:MGI Symb	0.022895
ENSMUSG00000024431	Nr3c1	nuclear receptor subfamily 3, group C, member 1 [S	0.022902
ENSMUSG00000032719	Sbspon	somatomedin B and thrombospondin, type 1 doma	0.023025
ENSMUSG00000068205	Macrod2	MACRO domain containing 2 [Source:MGI Symbol;A	0.023028
ENSMUSG00000042298	Ttc19	tetratricopeptide repeat domain 19 [Source:MGI Sy	0.023057
ENSMUSG00000017978	Cadps2	Ca2+-dependent activator protein for secretion 2 [S	0.023059

ENSMUSG00000064343	mt-Tq	mitochondrially encoded tRNA glutamine [Source:N	0.023121
ENSMUSG00000022986	Krt75	keratin 75 [Source:MGI Symbol;Acc:MGI:1923500]	0.023124
ENSMUSG00000031347	Cetn2	centrin 2 [Source:MGI Symbol;Acc:MGI:1347085]	0.023145
ENSMUSG00000020358	Hnrnpab	heterogeneous nuclear ribonucleoprotein A/B [Sou	0.023147
ENSMUSG00000007029	Vars	valyl-tRNA synthetase [Source:MGI Symbol;Acc:MG	0.0232
ENSMUSG00000021819	Zswim8	zinc finger SWIM-type containing 8 [Source:MGI Sy	0.02322
ENSMUSG00000024098	Twsg1	twisted gastrulation homolog 1 (Drosophila) [Sourc	0.02325
ENSMUSG00000031016	Wee1	WEE 1 homolog 1 (S. pombe) [Source:MGI Symbol;	0.023253
ENSMUSG00000026202	Tuba4a	tubulin, alpha 4A [Source:MGI Symbol;Acc:MGI:109	0.023282
ENSMUSG00000029478	Ncor2	nuclear receptor co-repressor 2 [Source:MGI Symb	0.023284
ENSMUSG00000020137	Thap2	THAP domain containing, apoptosis associated prot	0.023301
ENSMUSG00000040797	Iqsec3	IQ motif and Sec7 domain 3 [Source:MGI Symbol;A	0.023324
ENSMUSG00000023150	Ivns1abp	influenza virus NS1A binding protein [Source:MGI S	0.023338
ENSMUSG00000041623	D11Wsu47e	DNA segment, Chr 11, Wayne State University 47, e	0.023338
ENSMUSG00000074171	Gm6658	predicted gene 6658 [Source:MGI Symbol;Acc:MGI:	0.023399
ENSMUSG00000020238	Ncln	nicalin homolog (zebrafish) [Source:MGI Symbol;Ac	0.023418
ENSMUSG00000000555	Itga5	integrin alpha 5 (fibronectin receptor alpha) [Sourc	0.023437
ENSMUSG00000029925	Tbxas1	thromboxane A synthase 1, platelet [Source:MGI Sy	0.023449
ENSMUSG00000062184	Hs6st2	heparan sulfate 6-O-sulfotransferase 2 [Source:MG	0.023455
ENSMUSG00000020078	Vps26a	vacuolar protein sorting 26 homolog A (yeast) [Sou	0.023459
ENSMUSG00000092060	Bend4	BEN domain containing 4 [Source:MGI Symbol;Acc:	0.023479
ENSMUSG00000026743	Mllt10	myeloid/lymphoid or mixed-lineage leukemia (trith	0.023505
ENSMUSG00000055333	Fat2	FAT tumor suppressor homolog 2 (Drosophila) [Sou	0.023631
ENSMUSG00000027977	Ndst3	N-deacetylase/N-sulfotransferase (heparan glucosa	0.023723
ENSMUSG00000028943	Espn	espin [Source:MGI Symbol;Acc:MGI:1861630]	0.023727
ENSMUSG00000047034	Ankrd33	ankyrin repeat domain 33 [Source:MGI Symbol;Acc	0.023753
ENSMUSG00000038094	Atp13a4	ATPase type 13A4 [Source:MGI Symbol;Acc:MGI:19	0.023768
ENSMUSG00000022543	4930451G09Rik	RIKEN cDNA 4930451G09 gene [Source:MGI Symbo	0.023872
ENSMUSG00000044795	Cyb5d1	cytochrome b5 domain containing 1 [Source:MGI S	0.02392
ENSMUSG00000015053	Gata2	GATA binding protein 2 [Source:MGI Symbol;Acc:M	0.023929
ENSMUSG00000035173	A630007B06Rik	RIKEN cDNA A630007B06 gene [Source:MGI Symbo	0.02394
ENSMUSG00000042272	Sestd1	SEC14 and spectrin domains 1 [Source:MGI Symbol	0.02396
ENSMUSG00000032352	Lrrc1	leucine rich repeat containing 1 [Source:MGI Symb	0.02401
ENSMUSG00000032479	Map4	microtubule-associated protein 4 [Source:MGI Sym	0.024024
ENSMUSG00000084999	Gm15839	predicted gene 15839 [Source:MGI Symbol;Acc:MG	0.024049
ENSMUSG00000032377	Plscr4	phospholipid scramblase 4 [Source:MGI Symbol;Ac	0.02405
ENSMUSG00000018459	Slc13a3	solute carrier family 13 (sodium-dependent dicarbo	0.024092
ENSMUSG00000058135	Gstm1	glutathione S-transferase, mu 1 [Source:MGI Symb	0.024103
ENSMUSG00000031217	Efnb1	ephrin B1 [Source:MGI Symbol;Acc:MGI:102708]	0.024118
ENSMUSG00000050663	Trhde	TRH-degrading enzyme [Source:MGI Symbol;Acc:M	0.024124
ENSMUSG00000026219	Trip12	thyroid hormone receptor interactor 12 [Source:M	0.024151
ENSMUSG00000098066	Gm26944	predicted gene, 26944 [Source:MGI Symbol;Acc:M	0.024192
ENSMUSG00000083646	Gm6088	predicted gene 6088 [Source:MGI Symbol;Acc:MGI:	0.024217
ENSMUSG00000054199	Gon4l	gon-4-like (C.elegans) [Source:MGI Symbol;Acc:MG	0.024218
ENSMUSG00000058546	Rpl23a	ribosomal protein L23A [Source:MGI Symbol;Acc:M	0.024223
ENSMUSG00000086859	2810008D09Rik	RIKEN cDNA 2810008D09 gene [Source:MGI Symbo	0.024258
ENSMUSG00000027282	Mtch2	mitochondrial carrier homolog 2 (C. elegans) [Sourc	0.024284

ENSMUSG00000092043	Gm5494	predicted gene 5494 [Source:MGI Symbol;Acc:MGI:	0.024299
ENSMUSG00000020993	Trappc6b	trafficking protein particle complex 6B [Source:MGI	0.024341
ENSMUSG00000021619	Atg10	autophagy related 10 [Source:MGI Symbol;Acc:MG	0.02435
ENSMUSG00000090551	A730015C16Rik	RIKEN cDNA A730015C16 gene [Source:MGI Symbo	0.024394
ENSMUSG00000000552	Zfp385a	zinc finger protein 385A [Source:MGI Symbol;Acc:M	0.024398
ENSMUSG00000022836	Mylk	myosin, light polypeptide kinase [Source:MGI Symb	0.024402
ENSMUSG00000021194	Chga	chromogranin A [Source:MGI Symbol;Acc:MGI:883	0.024425
ENSMUSG00000006204	5430419D17Rik	RIKEN cDNA 5430419D17 gene [Source:MGI Symbo	0.02443
ENSMUSG00000012405	Rpl15	ribosomal protein L15 [Source:MGI Symbol;Acc:MG	0.02445
ENSMUSG00000033883	D3Ertd254e	DNA segment, Chr 3, ERATO Doi 254, expressed [So	0.024481
ENSMUSG00000035451	Foxa1	forkhead box A1 [Source:MGI Symbol;Acc:MGI:134	0.024481
ENSMUSG00000046768	Rhoj	ras homolog gene family, member J [Source:MGI Sy	0.024502
ENSMUSG00000039568	Ubald1	UBA-like domain containing 1 [Source:MGI Symbol;	0.024511
ENSMUSG00000040687	Madd	MAP-kinase activating death domain [Source:MGI S	0.024513
ENSMUSG00000058704	Memo1	mediator of cell motility 1 [Source:MGI Symbol;Acc	0.024521
ENSMUSG00000097452	Gm2824	predicted gene 2824 [Source:MGI Symbol;Acc:MGI:	0.024536
ENSMUSG00000029863	Casp2	caspase 2 [Source:MGI Symbol;Acc:MGI:97295]	0.024573
ENSMUSG00000041044	Lrit1	leucine-rich repeat, immunoglobulin-like and trans	0.024598
ENSMUSG00000071722	Spin4	spindlin family, member 4 [Source:MGI Symbol;Acc	0.024602
ENSMUSG00000031391	L1cam	L1 cell adhesion molecule [Source:MGI Symbol;Acc	0.024663
ENSMUSG00000035849	Krt222	keratin 222 [Source:MGI Symbol;Acc:MGI:2442728	0.024672
ENSMUSG00000003282	Plag1	pleiomorphic adenoma gene 1 [Source:MGI Symbo	0.024692
ENSMUSG00000028389	Zfp37	zinc finger protein 37 [Source:MGI Symbol;Acc:MGI	0.024693
ENSMUSG00000024654	Asrgl1	asparaginase like 1 [Source:MGI Symbol;Acc:MGI:1	0.024743
ENSMUSG00000021814	Anxa7	annexin A7 [Source:MGI Symbol;Acc:MGI:88031]	0.024781
ENSMUSG00000023110	Prmt5	protein arginine N-methyltransferase 5 [Source:MG	0.024787
ENSMUSG00000027599	Armc1	armadillo repeat containing 1 [Source:MGI Symbol;	0.024797
ENSMUSG00000085953	Gm15496	predicted gene 15496 [Source:MGI Symbol;Acc:MG	0.024813
ENSMUSG00000028546	Elavl4	ELAV (embryonic lethal, abnormal vision, Drosophil	0.024839
ENSMUSG00000097379	Gm26873	predicted gene, 26873 [Source:MGI Symbol;Acc:M	0.024927
ENSMUSG00000020741	Cluh	clustered mitochondria (cluA/CLU1) homolog [Sour	0.024943
ENSMUSG00000018196	Glrx2	glutaredoxin 2 (thioltransferase) [Source:MGI Symb	0.024987
ENSMUSG00000038151	Prdm1	PR domain containing 1, with ZNF domain [Source:	0.02499
ENSMUSG00000042505	Acn9	ACN9 homolog (S. cerevisiae) [Source:MGI Symbol;	0.025031
ENSMUSG00000002320	Tm9sf1	transmembrane 9 superfamily member 1 [Source:N	0.025056
ENSMUSG00000030671	Pde3b	phosphodiesterase 3B, cGMP-inhibited [Source:MG	0.025079
ENSMUSG00000020661	Dnmt3a	DNA methyltransferase 3A [Source:MGI Symbol;Ac	0.025083
ENSMUSG00000070166	Gm25821	predicted gene, 25821 [Source:MGI Symbol;Acc:M	0.025206
ENSMUSG00000038489	Polr2l	polymerase (RNA) II (DNA directed) polypeptide L [	0.02521
ENSMUSG00000044387	2410080I02Rik	RIKEN cDNA 2410080I02 gene [Source:MGI Symbol	0.025228
ENSMUSG00000073418	C4b	complement component 4B (Chido blood group) [S	0.02523
ENSMUSG00000088984	Gm25604	predicted gene, 25604 [Source:MGI Symbol;Acc:M	0.02531
ENSMUSG00000048347	Pcdhb18	protocadherin beta 18 [Source:MGI Symbol;Acc:M	0.025311
ENSMUSG00000046574	Prr12	proline rich 12 [Source:MGI Symbol;Acc:MGI:2679	0.025362
ENSMUSG00000027936	Crtc2	CREB regulated transcription coactivator 2 [Source:	0.025405
ENSMUSG00000049807	Arhgap23	Rho GTPase activating protein 23 [Source:MGI Sym	0.025416
ENSMUSG00000033902	Mapkbp1	mitogen-activated protein kinase binding protein 1	0.02544

ENSMUSG00000036053	Fmnl2	formin-like 2 [Source:MGI Symbol;Acc:MGI:191865	0.025488
ENSMUSG00000027254	Map1a	microtubule-associated protein 1 A [Source:MGI Sy	0.025491
ENSMUSG00000027820	Mme	membrane metallo endopeptidase [Source:MGI Sy	0.025529
ENSMUSG00000043687	1190005I06Rik	RIKEN cDNA 1190005I06 gene [Source:MGI Symbol	0.025548
ENSMUSG00000020651	Slc26a4	solute carrier family 26, member 4 [Source:MGI Sy	0.025552
ENSMUSG00000008193	Spib	Spi-B transcription factor (Spi-1/PU.1 related) [Sour	0.025553
ENSMUSG00000035150	Eif2s3x	eukaryotic translation initiation factor 2, subunit 3,	0.025562
ENSMUSG00000024941	Scyl1	SCY1-like 1 (S. cerevisiae) [Source:MGI Symbol;Acc:	0.025572
ENSMUSG00000031848	Lsm4	LSM4 homolog, U6 small nuclear RNA associated (S	0.025573
ENSMUSG00000057914	Cacnb2	calcium channel, voltage-dependent, beta 2 subuni	0.025594
ENSMUSG00000019809	Pex3	peroxisomal biogenesis factor 3 [Source:MGI Symb	0.025627
ENSMUSG00000057530	Ece1	endothelin converting enzyme 1 [Source:MGI Symb	0.025632
ENSMUSG00000025262	Fam120c	family with sequence similarity 120, member C [So	0.025651
ENSMUSG00000041930	Fam222a	family with sequence similarity 222, member A [So	0.025666
ENSMUSG00000086359	9630013K17Rik	RIKEN cDNA 9630013K17 gene [Source:MGI Symbo	0.025684
ENSMUSG00000020935	Dcakd	dephospho-CoA kinase domain containing [Source:	0.025685
ENSMUSG00000028439	Fam219a	family with sequence similarity 219, member A [So	0.025693
ENSMUSG00000090698	Apold1	apolipoprotein L domain containing 1 [Source:MGI	0.025736
ENSMUSG00000022951	Rcan1	regulator of calcineurin 1 [Source:MGI Symbol;Acc:	0.025768
ENSMUSG00000024413	Npc1	Niemann Pick type C1 [Source:MGI Symbol;Acc:MG	0.025768
ENSMUSG00000086905	Gm13716	predicted gene 13716 [Source:MGI Symbol;Acc:MG	0.025772
ENSMUSG00000043987	Cep164	centrosomal protein 164 [Source:MGI Symbol;Acc:I	0.025775
ENSMUSG00000064125	BC068157	cDNA sequence BC068157 [Source:MGI Symbol;Acc	0.025789
ENSMUSG00000020682	Mmp28	matrix metallopeptidase 28 (epilysin) [Source:MGI	0.025804
ENSMUSG00000044308	Ubr3	ubiquitin protein ligase E3 component n-recognin 3	0.025893
ENSMUSG00000097234	Gm26518	predicted gene, 26518 [Source:MGI Symbol;Acc:M	0.025926
ENSMUSG00000022148	Fyb	FYN binding protein [Source:MGI Symbol;Acc:MGI:	0.025957
ENSMUSG00000074912	Gm14207	predicted gene 14207 [Source:MGI Symbol;Acc:MG	0.025972
ENSMUSG00000096344	Gm6408	predicted gene 6408 [Source:MGI Symbol;Acc:MGI:	0.026025
ENSMUSG00000024312	Wdr46	WD repeat domain 46 [Source:MGI Symbol;Acc:MG	0.026031
ENSMUSG00000037408	Cnnm4	cyclin M4 [Source:MGI Symbol;Acc:MGI:2151060]	0.026104
ENSMUSG00000047534	Mis18bp1	MIS18 binding protein 1 [Source:MGI Symbol;Acc:M	0.026121
ENSMUSG00000029430	Ran	RAN, member RAS oncogene family [Source:MGI Sy	0.026122
ENSMUSG00000045502	Niacr1	niacin receptor 1 [Source:MGI Symbol;Acc:MGI:193	0.026161
ENSMUSG00000020948	Klhl28	kelch-like 28 [Source:MGI Symbol;Acc:MGI:191393	0.026173
ENSMUSG00000006471	Ndor1	NADPH dependent diflavin oxidoreductase 1 [Sourc	0.026179
ENSMUSG00000026259	Ngef	neuronal guanine nucleotide exchange factor [Sour	0.026196
ENSMUSG00000030579	Tyrobp	TYRO protein tyrosine kinase binding protein [Sour	0.026262
ENSMUSG00000060904	Arl1	ADP-ribosylation factor-like 1 [Source:MGI Symbol;	0.026272
ENSMUSG00000026239	Pde6d	phosphodiesterase 6D, cGMP-specific, rod, delta [S	0.026343
ENSMUSG00000050079	Rspry1	ring finger and SPRY domain containing 1 [Source:N	0.02638
ENSMUSG00000029422	Rsrc2	arginine/serine-rich coiled-coil 2 [Source:MGI Symb	0.026398
ENSMUSG00000031422	Morf4l2	mortality factor 4 like 2 [Source:MGI Symbol;Acc:M	0.026398
ENSMUSG00000045027	Prss22	protease, serine, 22 [Source:MGI Symbol;Acc:MGI:	0.026453
ENSMUSG00000086863	Gm15543	predicted gene 15543 [Source:MGI Symbol;Acc:MG	0.026471
ENSMUSG00000056737	Capg	capping protein (actin filament), gelsolin-like [Sourc	0.026482
ENSMUSG00000059361	Nrsn2	neurensin 2 [Source:MGI Symbol;Acc:MGI:2684969	0.0265

ENSMUSG00000035062	Zc4h2	zinc finger, C4H2 domain containing [Source:MGI Sy	0.02651
ENSMUSG00000026579	F5	coagulation factor V [Source:MGI Symbol;Acc:MGI:	0.026517
ENSMUSG00000001627	Ifrd1	interferon-related developmental regulator 1 [Sour	0.026536
ENSMUSG00000028082	Sh3d19	SH3 domain protein D19 [Source:MGI Symbol;Acc:M	0.026536
ENSMUSG00000012114	Med15	mediator complex subunit 15 [Source:MGI Symbol;	0.026613
ENSMUSG00000027184	Caprin1	cell cycle associated protein 1 [Source:MGI Symbol;	0.02662
ENSMUSG00000082545	Gm12196	predicted gene 12196 [Source:MGI Symbol;Acc:MG	0.026697
ENSMUSG00000035623	Rsf1	remodeling and spacing factor 1 [Source:MGI Symb	0.02671
ENSMUSG00000044533	Rps2	ribosomal protein S2 [Source:MGI Symbol;Acc:MGI	0.026712
ENSMUSG00000032532	Cck	cholecystokinin [Source:MGI Symbol;Acc:MGI:8829	0.026734
ENSMUSG00000048550	Thnsl1	threonine synthase-like 1 (bacterial) [Source:MGI Sy	0.026748
ENSMUSG00000000167	Pih1d2	PIH1 domain containing 2 [Source:MGI Symbol;Acc	0.02675
ENSMUSG00000023403	Stk31	serine threonine kinase 31 [Source:MGI Symbol;Acc	0.026765
ENSMUSG00000027012	Dync1i2	dynein cytoplasmic 1 intermediate chain 2 [Source:	0.026771
ENSMUSG00000043822	Adamtsl5	ADAMTS-like 5 [Source:MGI Symbol;Acc:MGI:1913	0.026772
ENSMUSG00000024974	Smc3	structural maintenance of chromosomes 3 [Source:	0.026794
ENSMUSG00000037129	Tmprss13	transmembrane protease, serine 13 [Source:MGI Sy	0.026821
ENSMUSG00000063810	Alms1	Alstrom syndrome 1 [Source:MGI Symbol;Acc:MGI:	0.026879
ENSMUSG00000057858	D19Ertd737e	DNA segment, Chr 19, ERATO Doi 737, expressed [S	0.026941
ENSMUSG00000020955	Ap4s1	adaptor-related protein complex AP-4, sigma 1 [Sou	0.026949
ENSMUSG00000025915	Sgk3	serum/glucocorticoid regulated kinase 3 [Source:M	0.026985
ENSMUSG00000040877	Wdr25	WD repeat domain 25 [Source:MGI Symbol;Acc:MG	0.027009
ENSMUSG00000028630	Dyrk2	dual-specificity tyrosine-(Y)-phosphorylation regula	0.027024
ENSMUSG00000086029	Pax6os1	Pax6 opposite strand transcript 1 [Source:MGI Sym	0.027038
ENSMUSG00000005656	Snx6	sorting nexin 6 [Source:MGI Symbol;Acc:MGI:1919	0.027054
ENSMUSG00000071477	Zfp777	zinc finger protein 777 [Source:MGI Symbol;Acc:MG	0.027073
ENSMUSG00000067786	Nnat	neuronatin [Source:MGI Symbol;Acc:MGI:104716]	0.027108
ENSMUSG00000049556	Lingo1	leucine rich repeat and Ig domain containing 1 [Sou	0.027113
ENSMUSG00000042388	Dlgap3	discs, large (Drosophila) homolog-associated protei	0.027271
ENSMUSG00000001151	Pcnt	pericentrin (kendrin) [Source:MGI Symbol;Acc:MGI	0.027273
ENSMUSG00000030302	Atp2b2	ATPase, Ca++ transporting, plasma membrane 2 [So	0.027285
ENSMUSG00000034807	Colgalt1	collagen beta(1-O)galactosyltransferase 1 [Source:M	0.0273
ENSMUSG00000023092	Fhl1	four and a half LIM domains 1 [Source:MGI Symbol	0.027302
ENSMUSG00000019647	Sema6a	sema domain, transmembrane domain (TM), and cy	0.027332
ENSMUSG00000080717	B230307C23Rik	RIKEN cDNA B230307C23 gene [Source:MGI Symbo	0.027376
ENSMUSG00000038822	Hace1	HECT domain and ankyrin repeat containing, E3 ubi	0.027378
ENSMUSG00000048930	Tada3	transcriptional adaptor 3 [Source:MGI Symbol;Acc:	0.02739
ENSMUSG00000039116	Gpr126	G protein-coupled receptor 126 [Source:MGI Symb	0.027392
ENSMUSG00000021054	Sgpp1	sphingosine-1-phosphate phosphatase 1 [Source:M	0.027399
ENSMUSG00000030852	Tacc2	transforming, acidic coiled-coil containing protein 2	0.027413
ENSMUSG00000070394	Tmem256	transmembrane protein 256 [Source:MGI Symbol;A	0.027417
ENSMUSG00000017760	Ctsa	cathepsin A [Source:MGI Symbol;Acc:MGI:97748]	0.027491
ENSMUSG00000068079	Tcf15	transcription factor 15 [Source:MGI Symbol;Acc:MG	0.027494
ENSMUSG00000026586	Prrx1	paired related homeobox 1 [Source:MGI Symbol;Ac	0.0275
ENSMUSG00000002930	Ppp1r17	protein phosphatase 1, regulatory subunit 17 [Sour	0.027507
ENSMUSG00000029647	Pan3	PAN3 polyA specific ribonuclease subunit homolog	0.027517
ENSMUSG00000023232	Serinc2	serine incorporator 2 [Source:MGI Symbol;Acc:MGI	0.027532

ENSMUSG00000015568	Lpl	lipoprotein lipase [Source:MGI Symbol;Acc:MGI:96	0.027535
ENSMUSG00000024487	Yipf5	Yip1 domain family, member 5 [Source:MGI Symbo	0.027535
ENSMUSG00000021255	Esrrb	estrogen related receptor, beta [Source:MGI Symb	0.027579
ENSMUSG00000097494	4933406C10Rik	RIKEN cDNA 4933406C10 gene [Source:MGI Symbo	0.027619
ENSMUSG00000032245	Cln6	ceroid-lipofuscinosis, neuronal 6 [Source:MGI Symb	0.027627
ENSMUSG00000003617	Cp	ceruloplasmin [Source:MGI Symbol;Acc:MGI:88476	0.027633
ENSMUSG00000032374	Plod2	procollagen lysine, 2-oxoglutarate 5-dioxygenase 2	0.027654
ENSMUSG00000088929	Gm24299	predicted gene, 24299 [Source:MGI Symbol;Acc:M	0.027654
ENSMUSG00000084791	Gm13667	predicted gene 13667 [Source:MGI Symbol;Acc:MG	0.027668
ENSMUSG00000053253	Ndfip2	Nedd4 family interacting protein 2 [Source:MGI Sy	0.027684
ENSMUSG00000035559	Mpv17l2	MPV17 mitochondrial membrane protein-like 2 [So	0.027741
ENSMUSG00000055633	Zfp580	zinc finger protein 580 [Source:MGI Symbol;Acc:M	0.027745
ENSMUSG00000078794	Dact3	dapper homolog 3, antagonist of beta-catenin (xen	0.027758
ENSMUSG00000040242	Fgfr1op2	FGFR1 oncogene partner 2 [Source:MGI Symbol;Ac	0.027797
ENSMUSG00000025571	Tnrc6c	trinucleotide repeat containing 6C [Source:MGI Sy	0.027802
ENSMUSG00000027281	Slx4ip	SLX4 interacting protein [Source:MGI Symbol;Acc:M	0.027839
ENSMUSG00000004317	Clcn5	chloride channel 5 [Source:MGI Symbol;Acc:MGI:9	0.027858
ENSMUSG00000050919	Zfp366	zinc finger protein 366 [Source:MGI Symbol;Acc:M	0.027913
ENSMUSG00000069631	Strada	STE20-related kinase adaptor alpha [Source:MGI S	0.027921
ENSMUSG00000022544	Fam86	family with sequence similarity 86 [Source:MGI Sy	0.027938
ENSMUSG00000021256	Vash1	vasohibin 1 [Source:MGI Symbol;Acc:MGI:2442543	0.027939
ENSMUSG00000025971	9430016H08Rik	RIKEN cDNA 9430016H08 gene [Source:MGI Symbo	0.027977
ENSMUSG00000027656	Wisp2	WNT1 inducible signaling pathway protein 2 [Sourc	0.028026
ENSMUSG00000017664	Slc35c2	solute carrier family 35, member C2 [Source:MGI S	0.028055
ENSMUSG00000084788	Gm11342	predicted gene 11342 [Source:MGI Symbol;Acc:MG	0.028059
ENSMUSG00000083833	Gm13841	predicted gene 13841 [Source:MGI Symbol;Acc:MG	0.028113
ENSMUSG00000029462	Vps29	vacuolar protein sorting 29 (S. pombe) [Source:MG	0.028142
ENSMUSG00000027739	Rab33b	RAB33B, member of RAS oncogene family [Source:	0.028198
ENSMUSG00000069785	Gm11444	predicted gene 11444 [Source:MGI Symbol;Acc:MG	0.028213
ENSMUSG00000031883	Car7	carbonic anhydrase 7 [Source:MGI Symbol;Acc:MG	0.028242
ENSMUSG00000061436	Hipk2	homeodomain interacting protein kinase 2 [Source	0.028325
ENSMUSG00000040680	Kremen2	kringle containing transmembrane protein 2 [Sourc	0.028353
ENSMUSG00000024472	Dcp2	DCP2 decapping enzyme homolog (S. cerevisiae) [S	0.028359
ENSMUSG00000033688	1300017J02Rik	RIKEN cDNA 1300017J02 gene [Source:MGI Symbol	0.028368
ENSMUSG00000054387	Mdm4	transformed mouse 3T3 cell double minute 4 [Sour	0.028402
ENSMUSG00000059363	Fxn	frataxin [Source:MGI Symbol;Acc:MGI:1096879]	0.028443
ENSMUSG00000026924	Sec16a	SEC16 homolog A (S. cerevisiae) [Source:MGI Symb	0.028444
ENSMUSG00000031634	Ufsp2	UFM1-specific peptidase 2 [Source:MGI Symbol;Ac	0.028448
ENSMUSG00000044362	Ccdc89	coiled-coil domain containing 89 [Source:MGI Symb	0.028478
ENSMUSG00000025545	Clybl	citrate lyase beta like [Source:MGI Symbol;Acc:MG	0.028482
ENSMUSG00000038628	Polr3k	polymerase (RNA) III (DNA directed) polypeptide K	0.028495
ENSMUSG00000045045	Lrfn4	leucine rich repeat and fibronectin type III domain	0.028508
ENSMUSG00000024187	Itfg3	integrin alpha FG-GAP repeat containing 3 [Source:	0.028595
ENSMUSG00000040462	Os9	amplified in osteosarcoma [Source:MGI Symbol;Ac	0.028603
ENSMUSG00000019961	Tmpo	thymopoietin [Source:MGI Symbol;Acc:MGI:10692	0.028655
ENSMUSG00000038611	Phrf1	PHD and ring finger domains 1 [Source:MGI Symbo	0.02877
ENSMUSG00000065208	Gm24616	predicted gene, 24616 [Source:MGI Symbol;Acc:M	0.028775

ENSMUSG00000088224	Gm25406	predicted gene, 25406 [Source:MGI Symbol;Acc:MG	0.028778
ENSMUSG00000090468	Gm2840	predicted gene 2840 [Source:MGI Symbol;Acc:MGI:	0.028829
ENSMUSG00000026878	Rab14	RAB14, member RAS oncogene family [Source:MGI	0.028831
ENSMUSG00000020884	Asgr1	asialoglycoprotein receptor 1 [Source:MGI Symbol;	0.02888
ENSMUSG00000002107	Celf2	CUGBP, Elav-like family member 2 [Source:MGI Syn	0.028881
ENSMUSG00000032280	Tle3	transducin-like enhancer of split 3, homolog of Dro	0.028884
ENSMUSG00000031708	Tecr	trans-2,3-enoyl-CoA reductase [Source:MGI Symbo	0.028931
ENSMUSG00000087571	Gm15202	predicted gene 15202 [Source:MGI Symbol;Acc:MG	0.028933
ENSMUSG00000025020	Slit1	slit homolog 1 (Drosophila) [Source:MGI Symbol;Ac	0.028936
ENSMUSG00000042203	Tbc1d22b	TBC1 domain family, member 22B [Source:MGI Syn	0.028944
ENSMUSG00000040035	Disp2	dispatched homolog 2 (Drosophila) [Source:MGI Sy	0.028986
ENSMUSG00000009687	Fxyd5	FXYD domain-containing ion transport regulator 5 [	0.029015
ENSMUSG00000028360	Slc44a5	solute carrier family 44, member 5 [Source:MGI Syn	0.029056
ENSMUSG00000026471	Mr1	major histocompatibility complex, class I-related [S	0.029093
ENSMUSG00000021680	Crhbp	corticotropin releasing hormone binding protein [S	0.02913
ENSMUSG00000088835	Gm23547	predicted gene, 23547 [Source:MGI Symbol;Acc:MG	0.02915
ENSMUSG00000097122	Gm26624	predicted gene, 26624 [Source:MGI Symbol;Acc:MG	0.029196
ENSMUSG00000038539	Atf5	activating transcription factor 5 [Source:MGI Symbo	0.029204
ENSMUSG00000024050	Wiz	widely-interspaced zinc finger motifs [Source:MGI S	0.029248
ENSMUSG00000029203	Ube2k	ubiquitin-conjugating enzyme E2K [Source:MGI Syn	0.029277
ENSMUSG00000026384	Ptpn4	protein tyrosine phosphatase, non-receptor type 4	0.029282
ENSMUSG00000039765	Cc2d2a	coiled-coil and C2 domain containing 2A [Source:M	0.029284
ENSMUSG00000051951	Xkr4	X Kell blood group precursor related family membe	0.029399
ENSMUSG00000049717	Lig4	ligase IV, DNA, ATP-dependent [Source:MGI Symbo	0.029409
ENSMUSG00000029381	Shroom3	shroom family member 3 [Source:MGI Symbol;Acc:	0.029418
ENSMUSG00000040586	Ofd1	oral-facial-digital syndrome 1 gene homolog (huma	0.029424
ENSMUSG00000084250	Gm13550	predicted gene 13550 [Source:MGI Symbol;Acc:MG	0.02951
ENSMUSG00000013921	Clip3	CAP-GLY domain containing linker protein 3 [Source	0.029515
ENSMUSG00000038773	Kdm3b	KDM3B lysine (K)-specific demethylase 3B [Source:I	0.029523
ENSMUSG00000049848	Ceacam19	carcinoembryonic antigen-related cell adhesion mo	0.029691
ENSMUSG00000030079	Ruvbl1	RuvB-like protein 1 [Source:MGI Symbol;Acc:MGI:1	0.029748
ENSMUSG00000047721	Bola2	bolA-like 2 (E. coli) [Source:MGI Symbol;Acc:MGI:19	0.029765
ENSMUSG00000081514	Gm14673	predicted gene 14673 [Source:MGI Symbol;Acc:MG	0.029787
ENSMUSG00000021850	1700011H14Rik	RIKEN cDNA 1700011H14 gene [Source:MGI Symbo	0.029808
ENSMUSG00000078870	Gm14410	predicted gene 14410 [Source:MGI Symbol;Acc:MG	0.029809
ENSMUSG00000078122	F630028O10Rik	RIKEN cDNA F630028O10 gene [Source:MGI Symbo	0.02981
ENSMUSG00000020122	Egfr	epidermal growth factor receptor [Source:MGI Sym	0.029825
ENSMUSG00000030104	Edem1	ER degradation enhancer, mannosidase alpha-like 1	0.029828
ENSMUSG00000002413	Braf	Braf transforming gene [Source:MGI Symbol;Acc:M	0.029907
ENSMUSG00000075307	Klhl41	kelch-like 41 [Source:MGI Symbol;Acc:MGI:268385	0.029914
ENSMUSG00000043019	Edem3	ER degradation enhancer, mannosidase alpha-like 3	0.029922
ENSMUSG00000030956	Fam53b	family with sequence similarity 53, member B [Sour	0.029928
ENSMUSG00000037072	15-Sep	selenoprotein [Source:MGI Symbol;Acc:MGI:19279	0.029965
ENSMUSG00000043252	Tmem64	transmembrane protein 64 [Source:MGI Symbol;Ac	0.029967
ENSMUSG00000073594	Gm10545	predicted gene 10545 [Source:MGI Symbol;Acc:MG	0.029975
ENSMUSG00000022491	Glycam1	glycosylation dependent cell adhesion molecule 1 [	0.03003
ENSMUSG00000044820	AY074887	cDNA sequence AY074887 [Source:MGI Symbol;Acc	0.030078

ENSMUSG00000018486	Wnt9b	wingless-type MMTV integration site 9B [Source:M	0.030088
ENSMUSG00000085862	Gm13483	predicted gene 13483 [Source:MGI Symbol;Acc:MG	0.030107
ENSMUSG00000031379	Pir	pirin [Source:MGI Symbol;Acc:MGI:1916906]	0.030132
ENSMUSG00000029780	Nt5c3	5'-nucleotidase, cytosolic III [Source:MGI Symbol;A	0.030145
ENSMUSG00000021392	Nol8	nucleolar protein 8 [Source:MGI Symbol;Acc:MGI:1	0.030198
ENSMUSG00000086735	Gm13977	predicted gene 13977 [Source:MGI Symbol;Acc:MG	0.030296
ENSMUSG00000066189	Cacng3	calcium channel, voltage-dependent, gamma subur	0.03031
ENSMUSG00000047773	Ankfn1	ankyrin-repeat and fibronectin type III domain cont	0.030325
ENSMUSG00000019792	Trmt11	tRNA methyltransferase 11 [Source:MGI Symbol;Ac	0.030379
ENSMUSG00000025582	Nptx1	neuronal pentraxin 1 [Source:MGI Symbol;Acc:MGI	0.030394
ENSMUSG00000030536	Iqgap1	IQ motif containing GTPase activating protein 1 [So	0.030404
ENSMUSG00000059486	Kbtbd2	kelch repeat and BTB (POZ) domain containing 2 [S	0.030425
ENSMUSG00000029366	Dck	deoxycytidine kinase [Source:MGI Symbol;Acc:MGI	0.030436
ENSMUSG00000086324	Gm15564	predicted gene 15564 [Source:MGI Symbol;Acc:MG	0.030444
ENSMUSG00000024130	Abca3	ATP-binding cassette, sub-family A (ABC1), membe	0.030482
ENSMUSG00000036095	Dgkb	diacylglycerol kinase, beta [Source:MGI Symbol;Acc	0.030538
ENSMUSG00000021629	Slc30a5	solute carrier family 30 (zinc transporter), member	0.030541
ENSMUSG00000085615	A330035P11Rik	RIKEN cDNA A330035P11 gene [Source:MGI Symbo	0.030547
ENSMUSG00000027706	Sec62	SEC62 homolog (S. cerevisiae) [Source:MGI Symbol	0.03056
ENSMUSG00000021375	Kif13a	kinesin family member 13A [Source:MGI Symbol;Ac	0.030566
ENSMUSG00000001247	Lsr	lipolysis stimulated lipoprotein receptor [Source:M	0.030569
ENSMUSG00000089872	Rps6kc1	ribosomal protein S6 kinase polypeptide 1 [Source:	0.03058
ENSMUSG00000042429	Adora1	adenosine A1 receptor [Source:MGI Symbol;Acc:M	0.030584
ENSMUSG00000040276	Pacsin1	protein kinase C and casein kinase substrate in neu	0.030751
ENSMUSG00000026276	2-Sep	septin 2 [Source:MGI Symbol;Acc:MGI:97298]	0.030789
ENSMUSG00000040563	BC018242	cDNA sequence BC018242 [Source:MGI Symbol;Acc	0.030922
ENSMUSG00000024393	Prrc2a	proline-rich coiled-coil 2A [Source:MGI Symbol;Acc	0.030953
ENSMUSG00000026450	Chit1	chitinase 1 (chitotriosidase) [Source:MGI Symbol;A	0.030954
ENSMUSG00000032997	Chpf	chondroitin polymerizing factor [Source:MGI Symb	0.030958
ENSMUSG00000031955	Bcar1	breast cancer anti-estrogen resistance 1 [Source:M	0.030961
ENSMUSG00000007656	Arpp19	cAMP-regulated phosphoprotein 19 [Source:MGI S	0.030968
ENSMUSG00000041997	Tlk1	tousled-like kinase 1 [Source:MGI Symbol;Acc:MGI:	0.030981
ENSMUSG00000075014	Gm10800	predicted gene 10800 [Source:MGI Symbol;Acc:MG	0.031005
ENSMUSG00000068122	Agtr2	angiotensin II receptor, type 2 [Source:MGI Symbol	0.031059
ENSMUSG00000020949	Fkbp3	FK506 binding protein 3 [Source:MGI Symbol;Acc:M	0.031064
ENSMUSG00000029397	Rchy1	ring finger and CHY zinc finger domain containing 1	0.031096
ENSMUSG00000029674	Limk1	LIM-domain containing, protein kinase [Source:MG	0.031156
ENSMUSG00000043036	Ccdc63	coiled-coil domain containing 63 [Source:MGI Symb	0.031157
ENSMUSG00000028041	Adam15	a disintegrin and metallopeptidase domain 15 (met	0.031205
ENSMUSG00000031353	Rbbp7	retinoblastoma binding protein 7 [Source:MGI Sym	0.031214
ENSMUSG00000032243	Itga11	integrin alpha 11 [Source:MGI Symbol;Acc:MGI:244	0.031238
ENSMUSG00000031986	Sprtn	SprT-like N-terminal domain [Source:MGI Symbol;A	0.03126
ENSMUSG00000046000	Naa11	N(alpha)-acetyltransferase 11, NatA catalytic subur	0.031283
ENSMUSG00000027447	Cst3	cystatin C [Source:MGI Symbol;Acc:MGI:102519]	0.031322
ENSMUSG00000030307	Slc6a11	solute carrier family 6 (neurotransmitter transporte	0.031365
ENSMUSG00000039715	Wdr34	WD repeat domain 34 [Source:MGI Symbol;Acc:MG	0.031396
ENSMUSG00000089890	Gm16055	predicted gene 16055 [Source:MGI Symbol;Acc:MG	0.031399

ENSMUSG00000022054	Nefm	neurofilament, medium polypeptide [Source:MGI S	0.031443
ENSMUSG00000058153	Sez6l	seizure related 6 homolog like [Source:MGI Symbol	0.031528
ENSMUSG00000084847	Gm15507	predicted gene 15507 [Source:MGI Symbol;Acc:MG	0.03154
ENSMUSG00000017999	Ddx27	DEAD (Asp-Glu-Ala-Asp) box polypeptide 27 [Source	0.031556
ENSMUSG00000051431	Gpr87	G protein-coupled receptor 87 [Source:MGI Symbo	0.031563
ENSMUSG00000034595	Ppp1r18	protein phosphatase 1, regulatory subunit 18 [Sour	0.031636
ENSMUSG00000076439	Mog	myelin oligodendrocyte glycoprotein [Source:MGI S	0.031643
ENSMUSG00000048385	Scrt1	scratch homolog 1, zinc finger protein (Drosophila)	0.031647
ENSMUSG00000022636	Alcam	activated leukocyte cell adhesion molecule [Source	0.031649
ENSMUSG00000048126	Col6a3	collagen, type VI, alpha 3 [Source:MGI Symbol;Acc:	0.031691
ENSMUSG00000020614	Fam20a	family with sequence similarity 20, member A [Sour	0.031712
ENSMUSG00000022940	Pigp	phosphatidylinositol glycan anchor biosynthesis, cla	0.03174
ENSMUSG00000035878	Hykk	hydroxylysine kinase 1 [Source:MGI Symbol;Acc:MG	0.031825
ENSMUSG00000026156	B3gat2	beta-1,3-glucuronyltransferase 2 (glucuronosyltran	0.031867
ENSMUSG00000059834	Sclt1	sodium channel and clathrin linker 1 [Source:MGI S	0.03187
ENSMUSG00000071302	2610044O15Rik8	RIKEN cDNA 2610044O15 gene [Source:MGI Symbo	0.03187
ENSMUSG00000026407	Cacna1s	calcium channel, voltage-dependent, L type, alpha	0.031907
ENSMUSG00000040044	Orc3	origin recognition complex, subunit 3 [Source:MGI	0.031962
ENSMUSG00000025323	Sp4	trans-acting transcription factor 4 [Source:MGI Sym	0.031967
ENSMUSG00000036368	Rmdn2	regulator of microtubule dynamics 2 [Source:MGI S	0.032004
ENSMUSG00000051606	2010001K21Rik	RIKEN cDNA 2010001K21 gene [Source:MGI Symbo	0.032023
ENSMUSG00000090841	Myl6	myosin, light polypeptide 6, alkali, smooth muscle a	0.032035
ENSMUSG00000032011	Thy1	thymus cell antigen 1, theta [Source:MGI Symbol;A	0.032085
ENSMUSG00000022451	Twf1	twinfilin, actin-binding protein, homolog 1 (Drosop	0.032179
ENSMUSG00000041740	Rnf10	ring finger protein 10 [Source:MGI Symbol;Acc:MGI	0.032222
ENSMUSG00000029683	Lmod2	leiomodin 2 (cardiac) [Source:MGI Symbol;Acc:MGI	0.032225
ENSMUSG00000025189	Cnnm1	cyclin M1 [Source:MGI Symbol;Acc:MGI:1891366]	0.03225
ENSMUSG00000020393	Kremen1	kringle containing transmembrane protein 1 [Sourc	0.032276
ENSMUSG00000021830	Txndc16	thioredoxin domain containing 16 [Source:MGI Sym	0.0323
ENSMUSG00000045036	Tmem232	transmembrane protein 232 [Source:MGI Symbol;A	0.032336
ENSMUSG00000020329	Polrmt	polymerase (RNA) mitochondrial (DNA directed) [So	0.032371
ENSMUSG00000031398	Plxna3	plexin A3 [Source:MGI Symbol;Acc:MGI:107683]	0.032493
ENSMUSG00000050232	Cxcr3	chemokine (C-X-C motif) receptor 3 [Source:MGI Sy	0.032513
ENSMUSG00000069670	Nkain2	Na+/K+ transporting ATPase interacting 2 [Source:M	0.032528
ENSMUSG00000029554	Mad1l1	MAD1 mitotic arrest deficient 1-like 1 [Source:MGI	0.032561
ENSMUSG00000087679	C330006A16Rik	RIKEN cDNA C330006A16 gene [Source:MGI Symbo	0.032633
ENSMUSG00000028670	Lypla2	lysophospholipase 2 [Source:MGI Symbol;Acc:MGI:	0.032661
ENSMUSG00000084957	Bbip1	BBSome interacting protein 1 [Source:MGI Symbol;	0.032707
ENSMUSG00000098006	Gm8565	predicted gene 8565 [Source:MGI Symbol;Acc:MGI:	0.03271
ENSMUSG00000075585	6330403L08Rik	RIKEN cDNA 6330403L08 gene [Source:MGI Symbo	0.032715
ENSMUSG00000029491	Pde6b	phosphodiesterase 6B, cGMP, rod receptor, beta po	0.032727
ENSMUSG00000068487	Gm4943	predicted pseudogene 4943 [Source:MGI Symbol;A	0.032761
ENSMUSG00000001424	Snd1	staphylococcal nuclease and tudor domain contain	0.032769
ENSMUSG00000050761	Gp1bb	glycoprotein Ib, beta polypeptide [Source:MGI Sym	0.032792
ENSMUSG00000025224	Gbf1	golgi-specific brefeldin A-resistance factor 1 [Sourc	0.032845
ENSMUSG00000029334	Prkg2	protein kinase, cGMP-dependent, type II [Source:M	0.032856
ENSMUSG00000029617	Ccz1	CCZ1 vacuolar protein trafficking and biogenesis as	0.03287

ENSMUSG00000032599	Ip6k2	inositol hexaphosphate kinase 2 [Source:MGI Symb	0.032952
ENSMUSG00000027236	Eif3j1	eukaryotic translation initiation factor 3, subunit J1	0.032999
ENSMUSG00000020577	Tspan13	tetraspanin 13 [Source:MGI Symbol;Acc:MGI:19133	0.033041
ENSMUSG00000024862	Klc2	kinesin light chain 2 [Source:MGI Symbol;Acc:MGI:1	0.033063
ENSMUSG00000045968	5830403L16Rik	RIKEN cDNA 5830403L16 gene [Source:MGI Symbo	0.033091
ENSMUSG00000023018	Smarcd1	SWI/SNF related, matrix associated, actin depender	0.0331
ENSMUSG00000022715	Cldn26	claudin 26 [Source:MGI Symbol;Acc:MGI:1921970]	0.033101
ENSMUSG00000027435	Cd93	CD93 antigen [Source:MGI Symbol;Acc:MGI:106664	0.033133
ENSMUSG00000031778	Cx3cl1	chemokine (C-X3-C motif) ligand 1 [Source:MGI Syn	0.033159
ENSMUSG00000022623	Shank3	SH3/ankyrin domain gene 3 [Source:MGI Symbol;A	0.033205
ENSMUSG00000045934	Mtmr11	myotubularin related protein 11 [Source:MGI Symb	0.033298
ENSMUSG00000032089	Il10ra	interleukin 10 receptor, alpha [Source:MGI Symbol;	0.033465
ENSMUSG00000040747	Cd53	CD53 antigen [Source:MGI Symbol;Acc:MGI:88341]	0.033508
ENSMUSG00000001985	Grik3	glutamate receptor, ionotropic, kainate 3 [Source:N	0.033527
ENSMUSG00000096603	Gm7694	predicted gene 7694 [Source:MGI Symbol;Acc:MGI:	0.03355
ENSMUSG00000031824	6430548M08Rik	RIKEN cDNA 6430548M08 gene [Source:MGI Symbo	0.033561
ENSMUSG00000036887	C1qa	complement component 1, q subcomponent, alpha	0.033621
ENSMUSG00000028518	Prkaa2	protein kinase, AMP-activated, alpha 2 catalytic sub	0.033627
ENSMUSG00000028454	Pigo	phosphatidylinositol glycan anchor biosynthesis, cla	0.033634
ENSMUSG00000091732	Gm17541	predicted gene, 17541 [Source:MGI Symbol;Acc:MG	0.033666
ENSMUSG00000037270	4932438A13Rik	RIKEN cDNA 4932438A13 gene [Source:MGI Symbo	0.033668
ENSMUSG00000027198	Ext2	exostoses (multiple) 2 [Source:MGI Symbol;Acc:MG	0.033669
ENSMUSG00000029484	Anxa3	annexin A3 [Source:MGI Symbol;Acc:MGI:1201378]	0.033684
ENSMUSG00000055612	Cdca7	cell division cycle associated 7 [Source:MGI Symbol	0.033709
ENSMUSG00000041268	Dmxl2	Dmx-like 2 [Source:MGI Symbol;Acc:MGI:2444630]	0.033752
ENSMUSG00000042348	Arl15	ADP-ribosylation factor-like 15 [Source:MGI Symbo	0.033777
ENSMUSG00000038914	Dido1	death inducer-obliterator 1 [Source:MGI Symbol;Ac	0.033794
ENSMUSG00000037369	Kdm6a	lysine (K)-specific demethylase 6A [Source:MGI Sym	0.0338
ENSMUSG00000036617	Etl4	enhancer trap locus 4 [Source:MGI Symbol;Acc:MG	0.033869
ENSMUSG00000096967	Gm26621	predicted gene, 26621 [Source:MGI Symbol;Acc:MG	0.033961
ENSMUSG00000000276	Dgke	diacylglycerol kinase, epsilon [Source:MGI Symbol;A	0.033988
ENSMUSG00000078784	1810022K09Rik	RIKEN cDNA 1810022K09 gene [Source:MGI Symbo	0.03399
ENSMUSG00000062591	Tubb4a	tubulin, beta 4A class IVA [Source:MGI Symbol;Acc:	0.03406
ENSMUSG00000055720	Ubl7	ubiquitin-like 7 (bone marrow stromal cell-derived)	0.034091
ENSMUSG00000050856	Atp5k	ATP synthase, H+ transporting, mitochondrial F1F0	0.034096
ENSMUSG00000038295	Atg9b	autophagy related 9B [Source:MGI Symbol;Acc:MG	0.034125
ENSMUSG00000026383	Epb4.1l5	erythrocyte protein band 4.1-like 5 [Source:MGI Sy	0.034131
ENSMUSG00000028173	Wls	wntless homolog (Drosophila) [Source:MGI Symbol	0.034236
ENSMUSG00000041977	Arhgef11	Rho guanine nucleotide exchange factor (GEF) 11 [S	0.034241
ENSMUSG00000097958	Gm8492	predicted gene 8492 [Source:MGI Symbol;Acc:MGI:	0.034255
ENSMUSG00000040537	Adam22	a disintegrin and metallopeptidase domain 22 [Sou	0.034256
ENSMUSG00000056267	Cep70	centrosomal protein 70 [Source:MGI Symbol;Acc:M	0.034279
ENSMUSG00000060450	Rnf14	ring finger protein 14 [Source:MGI Symbol;Acc:MGI	0.034346
ENSMUSG00000028577	Plaa	phospholipase A2, activating protein [Source:MGI S	0.034381
ENSMUSG00000056494	Cngb3	cyclic nucleotide gated channel beta 3 [Source:MGI	0.034441
ENSMUSG00000039621	Prex1	phosphatidylinositol-3,4,5-trisphosphate-depender	0.034477
ENSMUSG00000028578	Caap1	caspase activity and apoptosis inhibitor 1 [Source:N	0.034496

ENSMUSG00000045639	Zfp629	zinc finger protein 629 [Source:MGI Symbol;Acc:MG	0.034498
ENSMUSG00000024397	Aif1	allograft inflammatory factor 1 [Source:MGI Symbo	0.034521
ENSMUSG00000009293	Ube2g2	ubiquitin-conjugating enzyme E2G 2 [Source:MGI S	0.034663
ENSMUSG00000092341	Malat1	metastasis associated lung adenocarcinoma transcr	0.034672
ENSMUSG00000046329	Slc25a23	solute carrier family 25 (mitochondrial carrier; phos	0.034734
ENSMUSG00000043719	Col6a6	collagen, type VI, alpha 6 [Source:MGI Symbol;Acc:	0.034771
ENSMUSG00000059518	Znhit1	zinc finger, HIT domain containing 1 [Source:MGI Sy	0.03479
ENSMUSG00000027306	Nusap1	nucleolar and spindle associated protein 1 [Source:	0.034794
ENSMUSG00000001911	Nfix	nuclear factor I/X [Source:MGI Symbol;Acc:MGI:97	0.034798
ENSMUSG00000021721	Htr1a	5-hydroxytryptamine (serotonin) receptor 1A [Sour	0.034818
ENSMUSG00000027676	Ccdc39	coiled-coil domain containing 39 [Source:MGI Symb	0.03482
ENSMUSG00000079018	Ly6c1	lymphocyte antigen 6 complex, locus C1 [Source:M	0.034914
ENSMUSG00000068323	Slc4a5	solute carrier family 4, sodium bicarbonate cotrans	0.034922
ENSMUSG00000020541	Tom1l1	target of myb1-like 1 (chicken) [Source:MGI Symbo	0.034926
ENSMUSG00000018166	Erbb3	v-erb-b2 erythroblastic leukemia viral oncogene ho	0.035005
ENSMUSG00000022781	Pak2	p21 protein (Cdc42/Rac)-activated kinase 2 [Source	0.035023
ENSMUSG00000002885	Cd97	CD97 antigen [Source:MGI Symbol;Acc:MGI:134709	0.035031
ENSMUSG00000046020	Pofut1	protein O-fucosyltransferase 1 [Source:MGI Symbo	0.03509
ENSMUSG00000096847	Tmem151b	transmembrane protein 151B [Source:MGI Symbol;	0.035096
ENSMUSG00000028848	Gpn2	GPN-loop GTPase 2 [Source:MGI Symbol;Acc:MGI:2	0.035097
ENSMUSG00000025270	Alas2	aminolevulinic acid synthase 2, erythroid [Source:M	0.0352
ENSMUSG00000036800	Fam135b	family with sequence similarity 135, member B [Sou	0.035205
ENSMUSG00000020212	Mdm1	transformed mouse 3T3 cell double minute 1 [Sour	0.03521
ENSMUSG00000088625	Gm23318	predicted gene, 23318 [Source:MGI Symbol;Acc:MG	0.035274
ENSMUSG00000024451	Arap3	ArfGAP with RhoGAP domain, ankyrin repeat and P	0.035308
ENSMUSG00000028703	Lrrc41	leucine rich repeat containing 41 [Source:MGI Symb	0.035413
ENSMUSG00000027534	Snx16	sorting nexin 16 [Source:MGI Symbol;Acc:MGI:1921	0.035423
ENSMUSG00000025579	Gaa	glucosidase, alpha, acid [Source:MGI Symbol;Acc:M	0.035539
ENSMUSG00000078902	Gm14443	predicted gene 14443 [Source:MGI Symbol;Acc:MG	0.03554
ENSMUSG00000029234	Tmem165	transmembrane protein 165 [Source:MGI Symbol;A	0.035543
ENSMUSG00000054716	Zfp771	zinc finger protein 771 [Source:MGI Symbol;Acc:MG	0.035606
ENSMUSG00000038967	Pdk2	pyruvate dehydrogenase kinase, isoenzyme 2 [Sour	0.035616
ENSMUSG00000039377	Hlx	H2.0-like homeobox [Source:MGI Symbol;Acc:MGI:	0.035643
ENSMUSG00000087256	Gm15990	predicted gene 15990 [Source:MGI Symbol;Acc:MG	0.035713
ENSMUSG00000023284	Zfp605	zinc finger protein 605 [Source:MGI Symbol;Acc:MG	0.035786
ENSMUSG00000058881	Zfp516	zinc finger protein 516 [Source:MGI Symbol;Acc:MG	0.035887
ENSMUSG00000058661	Gm7452	predicted pseudogene 7452 [Source:MGI Symbol;A	0.035901
ENSMUSG00000031299	Pdha1	pyruvate dehydrogenase E1 alpha 1 [Source:MGI Sy	0.035933
ENSMUSG00000044447	Dock5	dedicator of cytokinesis 5 [Source:MGI Symbol;Acc:	0.035947
ENSMUSG00000020668	Kif3c	kinesin family member 3C [Source:MGI Symbol;Acc	0.03599
ENSMUSG00000050830	Vwc2	von Willebrand factor C domain containing 2 [Sourc	0.035996
ENSMUSG00000004098	Col5a3	collagen, type V, alpha 3 [Source:MGI Symbol;Acc:M	0.036011
ENSMUSG00000019158	Tmem160	transmembrane protein 160 [Source:MGI Symbol;A	0.036099
ENSMUSG00000027335	Adra1d	adrenergic receptor, alpha 1d [Source:MGI Symbol;	0.036112
ENSMUSG00000039753	Fbxl5	F-box and leucine-rich repeat protein 5 [Source:MG	0.036159
ENSMUSG00000086631	Gm12784	predicted gene 12784 [Source:MGI Symbol;Acc:MG	0.036196
ENSMUSG00000096351	Samd11	sterile alpha motif domain containing 11 [Source:M	0.036199

ENSMUSG00000025158	Rfng	RFNG O-fucosylpeptide 3-beta-N-acetylglucosamin	0.036201
ENSMUSG00000050272	Dscam	Down syndrome cell adhesion molecule [Source:M	0.036243
ENSMUSG00000052928	Ctif	CBP80/20-dependent translation initiation factor [S	0.036263
ENSMUSG00000037845	Fdxacb1	ferredoxin-fold anticodon binding domain containi	0.036283
ENSMUSG00000015090	Ptgds	prostaglandin D2 synthase (brain) [Source:MGI Sym	0.036296
ENSMUSG00000025047	Pdcd11	programmed cell death 11 [Source:MGI Symbol;Acc	0.036298
ENSMUSG00000033209	Ttc28	tetratricopeptide repeat domain 28 [Source:MGI Sy	0.036427
ENSMUSG00000027341	Tmem230	transmembrane protein 230 [Source:MGI Symbol;A	0.036429
ENSMUSG00000068762	Gstm6	glutathione S-transferase, mu 6 [Source:MGI Symb	0.036433
ENSMUSG00000027624	Epb4.1l1	erythrocyte protein band 4.1-like 1 [Source:MGI Sy	0.036436
ENSMUSG00000022309	Angpt1	angiopoietin 1 [Source:MGI Symbol;Acc:MGI:10844	0.036506
ENSMUSG00000097392	D930016D06Rik	RIKEN cDNA D930016D06 gene [Source:MGI Symbo	0.036532
ENSMUSG00000085495	Gm16796	predicted gene, 16796 [Source:MGI Symbol;Acc:M	0.036541
ENSMUSG00000034949	Zfr2	zinc finger RNA binding protein 2 [Source:MGI Sym	0.03657
ENSMUSG00000021639	Gtf2h2	general transcription factor II H, polypeptide 2 [Sou	0.036579
ENSMUSG00000090163	Gm16231	predicted gene 16231 [Source:MGI Symbol;Acc:MG	0.036685
ENSMUSG00000028345	Tex10	testis expressed gene 10 [Source:MGI Symbol;Acc:M	0.036702
ENSMUSG00000033386	Frrs1	ferric-chelate reductase 1 [Source:MGI Symbol;Acc	0.036702
ENSMUSG00000020773	Trim47	tripartite motif-containing 47 [Source:MGI Symbol;	0.03672
ENSMUSG00000054720	Lrrc8c	leucine rich repeat containing 8 family, member C [	0.036771
ENSMUSG00000041645	Ddx24	DEAD (Asp-Glu-Ala-Asp) box polypeptide 24 [Sourc	0.036777
ENSMUSG00000033705	Stard9	START domain containing 9 [Source:MGI Symbol;Ac	0.036779
ENSMUSG00000037331	Larp1	La ribonucleoprotein domain family, member 1 [So	0.036824
ENSMUSG00000096144	B020004C17Rik	RIKEN cDNA B020004C17 gene [Source:MGI Symbo	0.036883
ENSMUSG00000090112	Shprh	SNF2 histone linker PHD RING helicase [Source:MG	0.036891
ENSMUSG00000020787	P2rx1	purinergic receptor P2X, ligand-gated ion channel,	0.036943
ENSMUSG00000030954	Gp2	glycoprotein 2 (zymogen granule membrane) [Sour	0.036954
ENSMUSG00000063142	Kcnma1	potassium large conductance calcium-activated cha	0.036963
ENSMUSG00000036537	Rnf113a1	ring finger protein 113A1 [Source:MGI Symbol;Acc:	0.036967
ENSMUSG00000026177	Slc11a1	solute carrier family 11 (proton-coupled divalent m	0.037024
ENSMUSG00000000093	Tbx2	T-box 2 [Source:MGI Symbol;Acc:MGI:98494]	0.03703
ENSMUSG00000028187	Rpf1	ribosome production factor 1 homolog (S. cerevisia	0.037036
ENSMUSG00000031543	Ank1	ankyrin 1, erythroid [Source:MGI Symbol;Acc:MGI:8	0.037049
ENSMUSG00000049709	Nlrp10	NLR family, pyrin domain containing 10 [Source:MG	0.03706
ENSMUSG00000050359	Sprr1a	small proline-rich protein 1A [Source:MGI Symbol;A	0.037072
ENSMUSG00000024083	Pja2	praja 2, RING-H2 motif containing [Source:MGI Sym	0.037092
ENSMUSG00000025576	Rbfox3	RNA binding protein, fox-1 homolog (C. elegans) 3 [	0.037097
ENSMUSG00000021134	Srsf5	serine/arginine-rich splicing factor 5 [Source:MGI S	0.037127
ENSMUSG00000030471	Zdhhc13	zinc finger, DHHC domain containing 13 [Source:M	0.037151
ENSMUSG00000028124	Gclm	glutamate-cysteine ligase, modifier subunit [Source	0.037191
ENSMUSG00000029016	Clcn6	chloride channel 6 [Source:MGI Symbol;Acc:MGI:13	0.037355
ENSMUSG00000087481	Gm15608	predicted gene 15608 [Source:MGI Symbol;Acc:MG	0.037387
ENSMUSG00000087006	Gm13889	predicted gene 13889 [Source:MGI Symbol;Acc:MG	0.037395
ENSMUSG00000077493	Snord91a	small nucleolar RNA, C/D box 91A [Source:MGI Sym	0.037405
ENSMUSG00000053490	Trim60	tripartite motif-containing 60 [Source:MGI Symbol;	0.037422
ENSMUSG00000073761	4933427I04Rik	Riken cDNA 4933427I04 gene [Source:MGI Symbol;	0.03743
ENSMUSG00000056073	Grik2	glutamate receptor, ionotropic, kainate 2 (beta 2) [	0.037438

ENSMUSG00000060477	Irak2	interleukin-1 receptor-associated kinase 2 [Source:	0.037439
ENSMUSG00000056832	Ttc26	tetratricopeptide repeat domain 26 [Source:MGI Sy	0.037441
ENSMUSG00000037098	Rab11fip3	RAB11 family interacting protein 3 (class II) [Source	0.037476
ENSMUSG00000094551	Gm22392	predicted gene, 22392 [Source:MGI Symbol;Acc:MG	0.037489
ENSMUSG00000004263	Atn1	atrophin 1 [Source:MGI Symbol;Acc:MGI:104725]	0.037547
ENSMUSG00000093677	Gm20712	predicted gene 20712 [Source:MGI Symbol;Acc:MG	0.037576
ENSMUSG00000026109	Tmeff2	transmembrane protein with EGF-like and two follis	0.037581
ENSMUSG00000096361	Gm5814	predicted pseudogene 5814 [Source:MGI Symbol;A	0.037589
ENSMUSG00000058402	Zfp420	zinc finger protein 420 [Source:MGI Symbol;Acc:MG	0.037592
ENSMUSG00000077617	Gm25501	predicted gene, 25501 [Source:MGI Symbol;Acc:MG	0.037638
ENSMUSG00000029176	Anapc4	anaphase promoting complex subunit 4 [Source:MG	0.037639
ENSMUSG00000051146	Camk2n2	calcium/calmodulin-dependent protein kinase II inh	0.037647
ENSMUSG00000058638	Zfp110	zinc finger protein 110 [Source:MGI Symbol;Acc:MG	0.037655
ENSMUSG00000097355	Gm26662	predicted gene, 26662 [Source:MGI Symbol;Acc:MG	0.037681
ENSMUSG00000024622	Hmgxb3	HMG box domain containing 3 [Source:MGI Symbo	0.037759
ENSMUSG00000033632	AW554918	expressed sequence AW554918 [Source:MGI Symb	0.037878
ENSMUSG00000029221	Slc30a9	solute carrier family 30 (zinc transporter), member	0.037904
ENSMUSG00000006586	Runx1t1	runt-related transcription factor 1; translocated to,	0.037905
ENSMUSG00000027870	Hao2	hydroxyacid oxidase 2 [Source:MGI Symbol;Acc:MG	0.037928
ENSMUSG00000028830	AU040320	expressed sequence AU040320 [Source:MGI Symbo	0.03794
ENSMUSG00000032312	Csk	c-src tyrosine kinase [Source:MGI Symbol;Acc:MGI:	0.038011
ENSMUSG00000031283	Chrdl1	chordin-like 1 [Source:MGI Symbol;Acc:MGI:193317	0.03802
ENSMUSG00000026356	Dars	aspartyl-tRNA synthetase [Source:MGI Symbol;Acc:	0.038022
ENSMUSG00000030839	Sergef	secretion regulating guanine nucleotide exchange f	0.038052
ENSMUSG00000061068	Mcpt4	mast cell protease 4 [Source:MGI Symbol;Acc:MGI:	0.038073
ENSMUSG00000035735	Dagla	diacylglycerol lipase, alpha [Source:MGI Symbol;Acc	0.038102
ENSMUSG00000070576	Mn1	meningioma 1 [Source:MGI Symbol;Acc:MGI:12618	0.038108
ENSMUSG00000097292	A230107N01Rik	RIKEN cDNA A230107N01 gene [Source:MGI Symbo	0.038174
ENSMUSG00000020080	Hkdc1	hexokinase domain containing 1 [Source:MGI Symb	0.038193
ENSMUSG00000056608	Chd9	chromodomain helicase DNA binding protein 9 [Sou	0.038217
ENSMUSG00000029598	Plbd2	phospholipase B domain containing 2 [Source:MGI	0.038231
ENSMUSG00000026413	Pkp1	plakophilin 1 [Source:MGI Symbol;Acc:MGI:132835	0.038318
ENSMUSG00000032589	Bsn	bassoon [Source:MGI Symbol;Acc:MGI:1277955]	0.038343
ENSMUSG00000030824	Nucb1	nucleobindin 1 [Source:MGI Symbol;Acc:MGI:97388	0.038365
ENSMUSG00000091971	Hspa1a	heat shock protein 1A [Source:MGI Symbol;Acc:MG	0.03837
ENSMUSG00000032327	Stra6	stimulated by retinoic acid gene 6 [Source:MGI Sym	0.038383
ENSMUSG00000051343	Rab11fip5	RAB11 family interacting protein 5 (class I) [Source:	0.038435
ENSMUSG00000031149	Praf2	PRA1 domain family 2 [Source:MGI Symbol;Acc:MG	0.038473
ENSMUSG00000079139	Gm4204	predicted gene 4204 [Source:MGI Symbol;Acc:MGI:	0.038615
ENSMUSG00000050812	AI314180	expressed sequence AI314180 [Source:MGI Symbol	0.038647
ENSMUSG00000022174	Dad1	defender against cell death 1 [Source:MGI Symbol;A	0.03869
ENSMUSG00000000168	Dlat	dihydrolipoamide S-acetyltransferase (E2 compone	0.038753
ENSMUSG00000063077	Kif1b	kinesin family member 1B [Source:MGI Symbol;Acc	0.038762
ENSMUSG00000087168	Gm15983	predicted gene 15983 [Source:MGI Symbol;Acc:MG	0.038847
ENSMUSG00000084885	3010001F23Rik	RIKEN cDNA 3010001F23 gene [Source:MGI Symbo	0.038873
ENSMUSG00000040841	Six5	sine oculis-related homeobox 5 [Source:MGI Symbo	0.038878
ENSMUSG00000071001	Hrct1	histidine rich carboxyl terminus 1 [Source:MGI Sym	0.038893

ENSMUSG00000028549	Itgb3bp	integrin beta 3 binding protein (beta3-endonexin) [	0.038927
ENSMUSG00000033278	Ptprm	protein tyrosine phosphatase, receptor type, M [So	0.038951
ENSMUSG00000049537	Tecrl	trans-2,3-enoyl-CoA reductase-like [Source:MGI Sy	0.038969
ENSMUSG00000020163	Uqcr11	ubiquinol-cytochrome c reductase, complex III subu	0.03897
ENSMUSG00000056498	Tmem154	transmembrane protein 154 [Source:MGI Symbol;A	0.039018
ENSMUSG00000044816	D630023F18Rik	RIKEN cDNA D630023F18 gene [Source:MGI Symbo	0.039084
ENSMUSG00000028799	Zfp362	zinc finger protein 362 [Source:MGI Symbol;Acc:M	0.039106
ENSMUSG00000032187	Smarca4	SWI/SNF related, matrix associated, actin depende	0.039138
ENSMUSG00000041741	Pde3a	phosphodiesterase 3A, cGMP inhibited [Source:MG	0.03914
ENSMUSG00000041261	Car8	carbonic anhydrase 8 [Source:MGI Symbol;Acc:MG	0.039174
ENSMUSG00000078012	Gm23487	predicted gene, 23487 [Source:MGI Symbol;Acc:M	0.039175
ENSMUSG00000028745	Capzb	capping protein (actin filament) muscle Z-line, beta	0.039189
ENSMUSG00000047747	Rnf150	ring finger protein 150 [Source:MGI Symbol;Acc:M	0.039203
ENSMUSG00000064451	Snora23	small nucleolar RNA, H/ACA box 23 [Source:MGI Sy	0.039223
ENSMUSG00000032085	Tagln	transgelin [Source:MGI Symbol;Acc:MGI:106012]	0.039263
ENSMUSG00000062209	Erbb4	v-erb-a erythroblastic leukemia viral oncogene hom	0.039283
ENSMUSG00000038206	Fbxo8	F-box protein 8 [Source:MGI Symbol;Acc:MGI:1354	0.039328
ENSMUSG00000020196	Cabin1	calcineurin binding protein 1 [Source:MGI Symbol;A	0.03934
ENSMUSG00000040722	Scamp5	secretory carrier membrane protein 5 [Source:MGI	0.03938
ENSMUSG00000096944	Gm26722	predicted gene, 26722 [Source:MGI Symbol;Acc:M	0.039384
ENSMUSG00000058260	Serpina9	serine (or cysteine) peptidase inhibitor, clade A (alp	0.039439
ENSMUSG00000022760	Thap7	THAP domain containing 7 [Source:MGI Symbol;Ac	0.039492
ENSMUSG00000000489	Pdgfb	platelet derived growth factor, B polypeptide [Sour	0.039508
ENSMUSG00000074579	Lekr1	leucine, glutamate and lysine rich 1 [Source:MGI Sy	0.039551
ENSMUSG00000052299	Ltn1	listerin E3 ubiquitin protein ligase 1 [Source:MGI Sy	0.039593
ENSMUSG00000066720	Cldn9	claudin 9 [Source:MGI Symbol;Acc:MGI:1913100]	0.039672
ENSMUSG00000036264	Fstl4	follistatin-like 4 [Source:MGI Symbol;Acc:MGI:2443	0.039673
ENSMUSG00000003421	Nosip	nitric oxide synthase interacting protein [Source:M	0.039737
ENSMUSG00000020366	Mapk9	mitogen-activated protein kinase 9 [Source:MGI Sy	0.039772
ENSMUSG00000024758	Rtn3	reticulon 3 [Source:MGI Symbol;Acc:MGI:1339970]	0.039776
ENSMUSG00000085201	Gm13507	predicted gene 13507 [Source:MGI Symbol;Acc:MG	0.039847
ENSMUSG00000039929	Urb1	URB1 ribosome biogenesis 1 homolog (S. cerevisiae	0.039857
ENSMUSG00000042284	Itga1	integrin alpha 1 [Source:MGI Symbol;Acc:MGI:9659	0.039859
ENSMUSG00000064193	Gm4735	predicted gene 4735 [Source:MGI Symbol;Acc:MGI:	0.039911
ENSMUSG00000019929	Dcn	decorin [Source:MGI Symbol;Acc:MGI:94872]	0.03993
ENSMUSG00000037254	Itih2	inter-alpha trypsin inhibitor, heavy chain 2 [Source:	0.039973
ENSMUSG00000067818	Myl9	myosin, light polypeptide 9, regulatory [Source:MG	0.040001
ENSMUSG00000059412	Fxyd2	FXYD domain-containing ion transport regulator 2 [	0.040056
ENSMUSG00000021978	Extl3	exostoses (multiple)-like 3 [Source:MGI Symbol;Acc	0.040067
ENSMUSG00000022119	Rbm26	RNA binding motif protein 26 [Source:MGI Symbol;	0.040091
ENSMUSG00000043099	Hic1	hypermethylated in cancer 1 [Source:MGI Symbol;A	0.040149
ENSMUSG00000028468	Rgp1	RGP1 retrograde golgi transport homolog (S. cerevi	0.040154
ENSMUSG00000097547	B230110C06Rik	RIKEN cDNA B230110C06 gene [Source:MGI Symbo	0.040182
ENSMUSG00000020087	Tysnd1	trypsin domain containing 1 [Source:MGI Symbol;A	0.040203
ENSMUSG00000001240	Ramp2	receptor (calcitonin) activity modifying protein 2 [S	0.040239
ENSMUSG00000091387	Gcnt4	glucosaminyl (N-acetyl) transferase 4, core 2 (beta-	0.040263
ENSMUSG00000022330	Osr2	odd-skipped related 2 [Source:MGI Symbol;Acc:MG	0.040375

ENSMUSG00000041263	Rusc1	RUN and SH3 domain containing 1 [Source:MGI Syr	0.040432
ENSMUSG00000041781	Cpsf2	cleavage and polyadenylation specific factor 2 [Sou	0.040456
ENSMUSG00000034457	Eda2r	ectodysplasin A2 receptor [Source:MGI Symbol;Acc	0.040467
ENSMUSG00000025892	Gria4	glutamate receptor, ionotropic, AMPA4 (alpha 4) [S	0.040472
ENSMUSG00000071584	Atf7	activating transcription factor 7 [Source:MGI Symbo	0.040494
ENSMUSG00000077212	Snord69	small nucleolar RNA, C/D box 69 [Source:MGI Symb	0.040512
ENSMUSG00000074595	Wfdc6a	WAP four-disulfide core domain 6A [Source:MGI Sy	0.040676
ENSMUSG00000036733	Rbm42	RNA binding motif protein 42 [Source:MGI Symbol;	0.040714
ENSMUSG00000022286	Grhl2	grainyhead-like 2 (Drosophila) [Source:MGI Symbol	0.04072
ENSMUSG00000059493	Nhs	Nance-Horan syndrome (human) [Source:MGI Symb	0.040764
ENSMUSG00000029636	Wasf3	WAS protein family, member 3 [Source:MGI Symbo	0.04077
ENSMUSG00000031387	Renbp	renin binding protein [Source:MGI Symbol;Acc:MGI	0.04077
ENSMUSG00000032349	Elovl5	ELOVL family member 5, elongation of long chain fa	0.040839
ENSMUSG00000055761	Nkain3	Na+/K+ transporting ATPase interacting 3 [Source:N	0.040852
ENSMUSG00000025525	Apool	apolipoprotein O-like [Source:MGI Symbol;Acc:MGI	0.0409
ENSMUSG00000063358	Mapk1	mitogen-activated protein kinase 1 [Source:MGI Sy	0.040926
ENSMUSG00000096929	A330023F24Rik	RIKEN cDNA A330023F24 gene [Source:MGI Symbo	0.040966
ENSMUSG00000016552	Foxred2	FAD-dependent oxidoreductase domain containing	0.040973
ENSMUSG00000017167	Cntnap1	contactin associated protein-like 1 [Source:MGI Syr	0.04101
ENSMUSG00000083876	Gm11439	predicted gene 11439 [Source:MGI Symbol;Acc:MG	0.041076
ENSMUSG00000097265	Gm26803	predicted gene, 26803 [Source:MGI Symbol;Acc:M(	0.041144
ENSMUSG00000039205	Ciz1	CDKN1A interacting zinc finger protein 1 [Source:M	0.041161
ENSMUSG00000048978	Nrsn1	neurensin 1 [Source:MGI Symbol;Acc:MGI:894662]	0.041201
ENSMUSG00000026469	Xpr1	xenotropic and polytropic retrovirus receptor 1 [So	0.041222
ENSMUSG00000072623	Zfp9	zinc finger protein 9 [Source:MGI Symbol;Acc:MGI:9	0.041229
ENSMUSG00000028165	Cisd2	CDGSH iron sulfur domain 2 [Source:MGI Symbol;A	0.041264
ENSMUSG00000054648	Zfp869	zinc finger protein 869 [Source:MGI Symbol;Acc:M(	0.04128
ENSMUSG00000074088	Snrnp40	small nuclear ribonucleoprotein 40 (U5) [Source:M(	0.041319
ENSMUSG00000026037	Orc2	origin recognition complex, subunit 2 [Source:MGI	0.041355
ENSMUSG00000048779	P2ry6	pyrimidinergic receptor P2Y, G-protein coupled, 6 [	0.041355
ENSMUSG00000045160	Bola3	bolA-like 3 (E. coli) [Source:MGI Symbol;Acc:MGI:1	0.041386
ENSMUSG00000060098	Prmt7	protein arginine N-methyltransferase 7 [Source:MG	0.041396
ENSMUSG00000026393	Nek7	NIMA (never in mitosis gene a)-related expressed k	0.041426
ENSMUSG00000087557	Gm15869	predicted gene 15869 [Source:MGI Symbol;Acc:MG	0.041436
ENSMUSG00000029290	Zfp326	zinc finger protein 326 [Source:MGI Symbol;Acc:M(	0.041536
ENSMUSG00000024234	Mtpap	mitochondrial poly(A) polymerase [Source:MGI Syn	0.041556
ENSMUSG00000055471	Alk	anaplastic lymphoma kinase [Source:MGI Symbol;A	0.041586
ENSMUSG00000006731	B4galnt1	beta-1,4-N-acetyl-galactosaminyl transferase 1 [Sou	0.041658
ENSMUSG00000043644	0610009L18Rik	RIKEN cDNA 0610009L18 gene [Source:MGI Symbo	0.041711
ENSMUSG00000026049	Tex30	testis expressed 30 [Source:MGI Symbol;Acc:MGI:1	0.041825
ENSMUSG00000035181	Heatr5a	HEAT repeat containing 5A [Source:MGI Symbol;Ac	0.041879
ENSMUSG00000065336	Snora34	small nucleolar RNA, H/ACA box 34 [Source:MGI Sy	0.041884
ENSMUSG00000051860	Samd7	sterile alpha motif domain containing 7 [Source:MG	0.041905
ENSMUSG00000033102	Cdc14b	CDC14 cell division cycle 14B [Source:MGI Symbol;A	0.041955
ENSMUSG00000091908	Gm17231	predicted gene 17231 [Source:MGI Symbol;Acc:MG	0.041963
ENSMUSG00000061650	Med9	mediator of RNA polymerase II transcription, subur	0.041967
ENSMUSG00000027804	Ppid	peptidylprolyl isomerase D (cyclophilin D) [Source:N	0.041999

ENSMUSG00000006423	C330007P06Rik	RIKEN cDNA C330007P06 gene [Source:MGI Symbo	0.042005
ENSMUSG00000085350	4930529I22Rik	RIKEN cDNA 4930529I22 gene [Source:MGI Symbol	0.04201
ENSMUSG00000019990	Pde7b	phosphodiesterase 7B [Source:MGI Symbol;Acc:MG	0.042095
ENSMUSG00000031783	Polr2c	polymerase (RNA) II (DNA directed) polypeptide C [	0.042102
ENSMUSG00000035851	Ythdc1	YTH domain containing 1 [Source:MGI Symbol;Acc:	0.042128
ENSMUSG00000020530	Ggnbp2	gametogenetin binding protein 2 [Source:MGI Sym	0.042138
ENSMUSG00000062202	Btbd9	BTB (POZ) domain containing 9 [Source:MGI Symbo	0.042144
ENSMUSG00000022494	Shisa9	shisa homolog 9 (Xenopus laevis) [Source:MGI Sym	0.042174
ENSMUSG00000030203	Dusp16	dual specificity phosphatase 16 [Source:MGI Symbo	0.042208
ENSMUSG00000062561	Gm10118	predicted gene 10118 [Source:MGI Symbol;Acc:MG	0.042226
ENSMUSG00000031198	Fundc2	FUN14 domain containing 2 [Source:MGI Symbol;A	0.04223
ENSMUSG00000085793	Lin52	lin-52 homolog (C. elegans) [Source:MGI Symbol;Ac	0.042346
ENSMUSG00000028185	Dnase2b	deoxyribonuclease II beta [Source:MGI Symbol;Acc	0.042397
ENSMUSG00000034336	Ina	internexin neuronal intermediate filament protein,	0.042421
ENSMUSG00000032021	Crtam	cytotoxic and regulatory T cell molecule [Source:M	0.042455
ENSMUSG00000003746	Man1a	mannosidase 1, alpha [Source:MGI Symbol;Acc:MG	0.042497
ENSMUSG00000024097	Srsf7	serine/arginine-rich splicing factor 7 [Source:MGI S	0.042501
ENSMUSG00000009246	Trpm5	transient receptor potential cation channel, subfam	0.042504
ENSMUSG00000025736	Jmjd8	jumonji domain containing 8 [Source:MGI Symbol;A	0.042546
ENSMUSG00000029375	Cxcl15	chemokine (C-X-C motif) ligand 15 [Source:MGI Syn	0.042558
ENSMUSG00000020000	Moxd1	monooxygenase, DBH-like 1 [Source:MGI Symbol;A	0.042561
ENSMUSG00000029130	Rnf32	ring finger protein 32 [Source:MGI Symbol;Acc:MGI	0.042566
ENSMUSG00000002108	Nr1h3	nuclear receptor subfamily 1, group H, member 3 [	0.042574
ENSMUSG00000043079	Synpo	synaptopodin [Source:MGI Symbol;Acc:MGI:10994	0.042574
ENSMUSG00000033862	Cdk10	cyclin-dependent kinase 10 [Source:MGI Symbol;Ac	0.042621
ENSMUSG00000043702	Pde12	phosphodiesterase 12 [Source:MGI Symbol;Acc:MG	0.042629
ENSMUSG00000027581	Stmn3	stathmin-like 3 [Source:MGI Symbol;Acc:MGI:1277	0.042642
ENSMUSG00000030084	Plxna1	plexin A1 [Source:MGI Symbol;Acc:MGI:107685]	0.042686
ENSMUSG00000071649	B3gat3	beta-1,3-glucuronyltransferase 3 (glucuronosyltran	0.042744
ENSMUSG00000097792	Gm27042	predicted gene, 27042 [Source:MGI Symbol;Acc:M	0.042746
ENSMUSG00000060166	Zdhhc8	zinc finger, DHHC domain containing 8 [Source:MG	0.042748
ENSMUSG00000048078	Tenm4	teneurin transmembrane protein 4 [Source:MGI Sy	0.042774
ENSMUSG00000031847	1700030J22Rik	RIKEN cDNA 1700030J22 gene [Source:MGI Symbol	0.042838
ENSMUSG00000035681	Kcnc2	potassium voltage gated channel, Shaw-related sub	0.042853
ENSMUSG00000032194	Kank2	KN motif and ankyrin repeat domains 2 [Source:MG	0.043041
ENSMUSG00000020241	Col6a2	collagen, type VI, alpha 2 [Source:MGI Symbol;Acc:	0.043101
ENSMUSG00000025804	Ccr1	chemokine (C-C motif) receptor 1 [Source:MGI Sym	0.043187
ENSMUSG00000020669	Sh3yl1	Sh3 domain YSC-like 1 [Source:MGI Symbol;Acc:MG	0.043227
ENSMUSG00000085290	Gm16129	predicted gene 16129 [Source:MGI Symbol;Acc:MG	0.043257
ENSMUSG00000096001	2610528A11Rik	RIKEN cDNA 2610528A11 gene [Source:MGI Symbo	0.043265
ENSMUSG00000066607	6030419C18Rik	RIKEN cDNA 6030419C18 gene [Source:MGI Symbo	0.043283
ENSMUSG00000091985	Gm17354	predicted gene, 17354 [Source:MGI Symbol;Acc:M	0.043312
ENSMUSG00000052911	Lamb2	laminin, beta 2 [Source:MGI Symbol;Acc:MGI:9991	0.04336
ENSMUSG00000028049	Scamp3	secretory carrier membrane protein 3 [Source:MGI	0.043361
ENSMUSG00000058301	Upf1	UPF1 regulator of nonsense transcripts homolog (y	0.043368
ENSMUSG00000064280	Ccdc146	coiled-coil domain containing 146 [Source:MGI Sym	0.04337
ENSMUSG00000062980	Cped1	cadherin-like and PC-esterase domain containing 1	0.043377

ENSMUSG00000027204	Fbn1	fibrillin 1 [Source:MGI Symbol;Acc:MGI:95489]	0.043383
ENSMUSG00000086226	Gm12660	predicted gene 12660 [Source:MGI Symbol;Acc:MG	0.043385
ENSMUSG00000002846	Timmdc1	translocase of inner mitochondrial membrane dom	0.043394
ENSMUSG00000079138	Gm8818	predicted pseudogene 8818 [Source:MGI Symbol;A	0.043422
ENSMUSG00000050357	Rltpr	RGD motif, leucine rich repeats, tropomodulin dom	0.043436
ENSMUSG00000048856	Slc25a47	solute carrier family 25, member 47 [Source:MGI Sy	0.043557
ENSMUSG00000079588	Tmem182	transmembrane protein 182 [Source:MGI Symbol;A	0.043557
ENSMUSG00000053182	Gm609	predicted gene 609 [Source:MGI Symbol;Acc:MGI:2	0.043561
ENSMUSG00000035941	Ibtk	inhibitor of Bruton agammaglobulinemia tyrosine k	0.043568
ENSMUSG00000030339	Ltbr	lymphotoxin B receptor [Source:MGI Symbol;Acc:M	0.04359
ENSMUSG00000063531	Sema3e	sema domain, immunoglobulin domain (Ig), short b	0.043629
ENSMUSG00000021996	Esd	esterase D/formylglutathione hydrolase [Source:M	0.043637
ENSMUSG00000026648	Dclre1c	DNA cross-link repair 1C, PSO2 homolog (S. cerevisi	0.04368
ENSMUSG00000035441	Myo1d	myosin ID [Source:MGI Symbol;Acc:MGI:107728]	0.043702
ENSMUSG00000034390	Cmip	c-Maf inducing protein [Source:MGI Symbol;Acc:M	0.04373
ENSMUSG00000017831	Rab5a	RAB5A, member RAS oncogene family [Source:MGI	0.043746
ENSMUSG00000027412	Lpin3	lipin 3 [Source:MGI Symbol;Acc:MGI:1891342]	0.04378
ENSMUSG00000090039	Gm15894	predicted gene 15894 [Source:MGI Symbol;Acc:MG	0.043793
ENSMUSG00000036907	C1ql2	complement component 1, q subcomponent-like 2	0.043809
ENSMUSG00000085867	Gm5834	predicted pseudogene 5834 [Source:MGI Symbol;A	0.043931
ENSMUSG00000025658	Cnksr2	connector enhancer of kinase suppressor of Ras 2 [	0.043954
ENSMUSG00000012777	Acpt	acid phosphatase, testicular [Source:MGI Symbol;A	0.043966
ENSMUSG00000019868	Vta1	Vps20-associated 1 homolog (S. cerevisiae) [Source	0.044109
ENSMUSG00000022754	Tmem45a	transmembrane protein 45a [Source:MGI Symbol;A	0.044136
ENSMUSG00000021687	Scamp1	secretory carrier membrane protein 1 [Source:MGI	0.044154
ENSMUSG00000058297	Spock2	sparc/osteonectin, cwcv and kazal-like domains pro	0.044162
ENSMUSG00000046432	Ngfrap1	nerve growth factor receptor (TNFRSF16) associate	0.04419
ENSMUSG00000053886	Sh2d4a	SH2 domain containing 4A [Source:MGI Symbol;Acc	0.044285
ENSMUSG00000040599	Mis12	MIS12 homolog (yeast) [Source:MGI Symbol;Acc:M	0.044439
ENSMUSG00000033039	Micall1	microtubule associated monooxygenase, calponin a	0.044443
ENSMUSG00000000776	Polr3d	polymerase (RNA) III (DNA directed) polypeptide D	0.044507
ENSMUSG00000025386	Pde6g	phosphodiesterase 6G, cGMP-specific, rod, gamma	0.044516
ENSMUSG00000044337	Ackr3	atypical chemokine receptor 3 [Source:MGI Symbol	0.044577
ENSMUSG00000043535	Setx	senataxin [Source:MGI Symbol;Acc:MGI:2443480]	0.044578
ENSMUSG00000034145	Tmem63c	transmembrane protein 63c [Source:MGI Symbol;A	0.044611
ENSMUSG00000026308	Klhl30	kelch-like 30 [Source:MGI Symbol;Acc:MGI:191803	0.04468
ENSMUSG00000020691	Mettl2	methyltransferase like 2 [Source:MGI Symbol;Acc:M	0.044689
ENSMUSG00000019122	Ccl9	chemokine (C-C motif) ligand 9 [Source:MGI Symbo	0.044812
ENSMUSG00000092094	Zfp804b	zinc finger protein 804B [Source:MGI Symbol;Acc:M	0.044865
ENSMUSG00000026199	Ankzf1	ankyrin repeat and zinc finger domain containing 1	0.044875
ENSMUSG00000040761	Spen	SPEN homolog, transcriptional regulator (Drosophil	0.04491
ENSMUSG00000027357	Crls1	cardiolipin synthase 1 [Source:MGI Symbol;Acc:MG	0.044923
ENSMUSG00000083093	Gm15385	predicted gene 15385 [Source:MGI Symbol;Acc:MG	0.044975
ENSMUSG00000032097	Ddx6	DEAD (Asp-Glu-Ala-Asp) box polypeptide 6 [Source:	0.044979
ENSMUSG00000021714	Cenpk	centromere protein K [Source:MGI Symbol;Acc:MG	0.044989
ENSMUSG00000061111	Fam195b	family with sequence similarity 195, member B [Sou	0.04501
ENSMUSG00000006412	Pfdn2	prefoldin 2 [Source:MGI Symbol;Acc:MGI:1276111]	0.045085

ENSMUSG00000036686	Cc2d1a	coiled-coil and C2 domain containing 1A [Source:M	0.045249
ENSMUSG00000020108	Ddit4	DNA-damage-inducible transcript 4 [Source:MGI Sy	0.045256
ENSMUSG00000027457	Snph	syntaphilin [Source:MGI Symbol;Acc:MGI:2139270]	0.045305
ENSMUSG00000019188	H13	histocompatibility 13 [Source:MGI Symbol;Acc:MGI	0.045316
ENSMUSG00000063388	BC023105	cDNA sequence BC023105 [Source:MGI Symbol;Acc	0.045332
ENSMUSG00000078905	Gm14393	predicted gene 14393 [Source:MGI Symbol;Acc:MG	0.045348
ENSMUSG00000019539	Rcn3	reticulocalbin 3, EF-hand calcium binding domain [S	0.045434
ENSMUSG00000020895	Tmem107	transmembrane protein 107 [Source:MGI Symbol;A	0.045446
ENSMUSG00000042834	Nrep	neuronal regeneration related protein [Source:MGI	0.045485
ENSMUSG00000027864	Ptgfrn	prostaglandin F2 receptor negative regulator [Sour	0.045494
ENSMUSG00000030662	Ipo5	importin 5 [Source:MGI Symbol;Acc:MGI:1917822]	0.045516
ENSMUSG00000039543	Ttc18	tetratricopeptide repeat domain 18 [Source:MGI Sy	0.045538
ENSMUSG00000072295	Als2cr11	amyotrophic lateral sclerosis 2 (juvenile) chromoso	0.04555
ENSMUSG00000002250	Ppard	peroxisome proliferator activator receptor delta [So	0.045615
ENSMUSG00000002980	Bcam	basal cell adhesion molecule [Source:MGI Symbol;A	0.045622
ENSMUSG00000052726	Kcnt2	potassium channel, subfamily T, member 2 [Source	0.045637
ENSMUSG00000029608	Rph3a	rabphilin 3A [Source:MGI Symbol;Acc:MGI:102788]	0.045679
ENSMUSG00000005397	Nid1	nidogen 1 [Source:MGI Symbol;Acc:MGI:97342]	0.045703
ENSMUSG00000087082	Gm15423	predicted gene 15423 [Source:MGI Symbol;Acc:MG	0.045786
ENSMUSG00000092397	C130080G10Rik	RIKEN cDNA C130080G10 gene [Source:MGI Symbo	0.0458
ENSMUSG00000097194	9330175E14Rik	RIKEN cDNA 9330175E14 gene [Source:MGI Symbo	0.045827
ENSMUSG00000090394	4930523C07Rik	RIKEN cDNA 4930523C07 gene [Source:MGI Symbo	0.045846
ENSMUSG00000063884	Ptcd3	pentatricopeptide repeat domain 3 [Source:MGI Sy	0.045883
ENSMUSG00000003031	Cdkn1b	cyclin-dependent kinase inhibitor 1B [Source:MGI S	0.045893
ENSMUSG00000021281	Tnfaip2	tumor necrosis factor, alpha-induced protein 2 [Sou	0.045913
ENSMUSG00000075538	Gm10855	predicted gene 10855 [Source:MGI Symbol;Acc:MG	0.045926
ENSMUSG00000090582	Gm17024	predicted gene 17024 [Source:MGI Symbol;Acc:MG	0.045979
ENSMUSG00000020476	Dbnl	drebrin-like [Source:MGI Symbol;Acc:MGI:700006]	0.04599
ENSMUSG00000031987	Egln1	EGL nine homolog 1 (C. elegans) [Source:MGI Symb	0.046012
ENSMUSG00000082377	Gm14415	predicted gene 14415 [Source:MGI Symbol;Acc:MG	0.04607
ENSMUSG00000029282	Amtn	amelotin [Source:MGI Symbol;Acc:MGI:1918671]	0.046077
ENSMUSG00000015599	Ttbk1	tau tubulin kinase 1 [Source:MGI Symbol;Acc:MGI:2	0.046107
ENSMUSG00000030816	Rnf40	ring finger protein 40 [Source:MGI Symbol;Acc:MGI	0.046144
ENSMUSG00000067199	Frat1	frequently rearranged in advanced T cell lymphoma	0.046149
ENSMUSG00000018442	Derl2	Der1-like domain family, member 2 [Source:MGI Sy	0.04615
ENSMUSG00000025888	Casp1	caspase 1 [Source:MGI Symbol;Acc:MGI:96544]	0.046185
ENSMUSG00000034341	Wbp2	WW domain binding protein 2 [Source:MGI Symbol	0.046206
ENSMUSG00000034799	Unc13a	unc-13 homolog A (C. elegans) [Source:MGI Symbo	0.046228
ENSMUSG00000079457	Gm7609	predicted pseudogene 7609 [Source:MGI Symbol;A	0.046229
ENSMUSG00000027314	Dll4	delta-like 4 (Drosophila) [Source:MGI Symbol;Acc:M	0.046234
ENSMUSG00000027305	Ndufaf1	NADH dehydrogenase (ubiquinone) 1 alpha subcom	0.046276
ENSMUSG00000004937	Sgta	small glutamine-rich tetratricopeptide repeat (TPR)	0.046329
ENSMUSG00000006307	Kmt2b	lysine (K)-specific methyltransferase 2B [Source:MG	0.046348
ENSMUSG00000094286	Gm12424	predicted gene 12424 [Source:MGI Symbol;Acc:MG	0.046355
ENSMUSG00000089716	Gm6264	predicted gene 6264 [Source:MGI Symbol;Acc:MGI:	0.046358
ENSMUSG00000037622	Wdtc1	WD and tetratricopeptide repeats 1 [Source:MGI Sy	0.046382
ENSMUSG00000028195	Cyr61	cysteine rich protein 61 [Source:MGI Symbol;Acc:M	0.046394

ENSMUSG00000024527	Afg3l2	AFG3(ATPase family gene 3)-like 2 (yeast) [Source:N	0.046498
ENSMUSG00000015476	Prrt1	proline-rich transmembrane protein 1 [Source:MGI	0.046508
ENSMUSG00000064634	Gm22620	predicted gene, 22620 [Source:MGI Symbol;Acc:MG	0.046519
ENSMUSG00000020397	Med7	mediator complex subunit 7 [Source:MGI Symbol;A	0.046578
ENSMUSG00000037974	Muc5ac	mucin 5, subtypes A and C, tracheobronchial/gastri	0.046587
ENSMUSG00000018293	Pfn1	profilin 1 [Source:MGI Symbol;Acc:MGI:97549]	0.046602
ENSMUSG00000058454	Dhcr7	7-dehydrocholesterol reductase [Source:MGI Symb	0.046616
ENSMUSG00000015214	Mtmr1	myotubularin related protein 1 [Source:MGI Symbo	0.046639
ENSMUSG00000065611	Mir23a	microRNA 23a [Source:MGI Symbol;Acc:MGI:26768	0.046732
ENSMUSG00000052912	Smarca5-ps	SWI/SNF related, matrix associated, actin depenent	0.046773
ENSMUSG00000040565	Btaf1	BTAF1 RNA polymerase II, B-TFIID transcription fact	0.046921
ENSMUSG00000021707	Dhfr	dihydrofolate reductase [Source:MGI Symbol;Acc:N	0.046959
ENSMUSG00000028641	Lepre1	leprecan 1 [Source:MGI Symbol;Acc:MGI:1888921]	0.046976
ENSMUSG00000060636	Rpl35a	ribosomal protein L35A [Source:MGI Symbol;Acc:M	0.047034
ENSMUSG00000024219	Anks1	ankyrin repeat and SAM domain containing 1 [Sour	0.047077
ENSMUSG00000034653	Ythdc2	YTH domain containing 2 [Source:MGI Symbol;Acc:	0.047117
ENSMUSG00000081683	Fzd10	frizzled homolog 10 (Drosophila) [Source:MGI Symb	0.047194
ENSMUSG00000042268	Slc26a9	solute carrier family 26, member 9 [Source:MGI Syr	0.047219
ENSMUSG00000040302	Rbm48	RNA binding motif protein 48 [Source:MGI Symbol;	0.047234
ENSMUSG00000040363	Bcor	BCL6 interacting corepressor [Source:MGI Symbol;A	0.047261
ENSMUSG00000097145	9230114K14Rik	RIKEN cDNA 9230114K14 gene [Source:MGI Symbo	0.047335
ENSMUSG00000030701	Plekhb1	pleckstrin homology domain containing, family B (e	0.047397
ENSMUSG00000019139	Isyna1	myo-inositol 1-phosphate synthase A1 [Source:MG	0.04741
ENSMUSG00000091149	Gm17020	predicted gene 17020 [Source:MGI Symbol;Acc:MG	0.047413
ENSMUSG00000021590	Spata9	spermatogenesis associated 9 [Source:MGI Symbol	0.047415
ENSMUSG00000026701	Prdx6	peroxiredoxin 6 [Source:MGI Symbol;Acc:MGI:8943	0.04742
ENSMUSG00000033819	Ppp1r16a	protein phosphatase 1, regulatory (inhibitor) subun	0.047425
ENSMUSG00000063626	Unc5d	unc-5 homolog D (C. elegans) [Source:MGI Symbol;	0.047425
ENSMUSG00000028766	Alpl	alkaline phosphatase, liver/bone/kidney [Source:M	0.047466
ENSMUSG00000020841	Cpd	carboxypeptidase D [Source:MGI Symbol;Acc:MGI:1	0.047472
ENSMUSG00000047193	Dync2h1	dynein cytoplasmic 2 heavy chain 1 [Source:MGI Sy	0.047504
ENSMUSG00000088634	Gm24958	predicted gene, 24958 [Source:MGI Symbol;Acc:MG	0.047534
ENSMUSG00000034795	Ccdc122	coiled-coil domain containing 122 [Source:MGI Sym	0.047543
ENSMUSG00000022089	Bin3	bridging integrator 3 [Source:MGI Symbol;Acc:MGI:	0.047562
ENSMUSG00000042406	Atf4	activating transcription factor 4 [Source:MGI Symbo	0.047632
ENSMUSG00000012483	Rpa3	replication protein A3 [Source:MGI Symbol;Acc:MG	0.047634
ENSMUSG00000097592	4930500F10Rik	RIKEN cDNA 4930500F10 gene [Source:MGI Symbo	0.047712
ENSMUSG00000077557	Gm23706	predicted gene, 23706 [Source:MGI Symbol;Acc:MG	0.047729
ENSMUSG00000073399	Trim40	tripartite motif-containing 40 [Source:MGI Symbol;	0.047746
ENSMUSG00000080906	Gm12095	predicted gene 12095 [Source:MGI Symbol;Acc:MG	0.047754
ENSMUSG00000071005	Ccl19	chemokine (C-C motif) ligand 19 [Source:MGI Symb	0.047896
ENSMUSG00000021263	Degs2	degenerative spermatocyte homolog 2 (Drosophila	0.047912
ENSMUSG00000021265	Slc25a29	solute carrier family 25 (mitochondrial carrier, palm	0.047928
ENSMUSG00000034902	Pip5k1c	phosphatidylinositol-4-phosphate 5-kinase, type 1 g	0.047972
ENSMUSG00000043943	Naalad2	N-acetylated alpha-linked acidic dipeptidase 2 [Sou	0.047983
ENSMUSG00000090925	1810064F22Rik	RIKEN cDNA 1810064F22 gene [Source:MGI Symbo	0.047992
ENSMUSG00000067873	Htatsf1	HIV TAT specific factor 1 [Source:MGI Symbol;Acc:N	0.048015

ENSMUSG00000033282	Rpgrip1l	Rpgrip1-like [Source:MGI Symbol;Acc:MGI:1920563	0.048031
ENSMUSG00000082141	Gm11212	predicted gene 11212 [Source:MGI Symbol;Acc:MG	0.048066
ENSMUSG00000000420	Galnt1	UDP-N-acetyl-alpha-D-galactosamine:polypeptide N	0.048116
ENSMUSG00000076269	Mir374	microRNA 374 [Source:MGI Symbol;Acc:MGI:36299	0.048116
ENSMUSG00000021171	Esyt2	extended synaptotagmin-like protein 2 [Source:MG	0.048129
ENSMUSG00000023830	Igf2r	insulin-like growth factor 2 receptor [Source:MGI Sy	0.048196
ENSMUSG00000028273	Pdlim5	PDZ and LIM domain 5 [Source:MGI Symbol;Acc:MG	0.048205
ENSMUSG00000031938	4931406C07Rik	RIKEN cDNA 4931406C07 gene [Source:MGI Symbo	0.048233
ENSMUSG00000060012	Kif13b	kinesin family member 13B [Source:MGI Symbol;Ac	0.04825
ENSMUSG00000032281	Acsbg1	acyl-CoA synthetase bubblegum family member 1 [	0.048277
ENSMUSG00000038517	Tbkbp1	TBK1 binding protein 1 [Source:MGI Symbol;Acc:MG	0.048301
ENSMUSG00000008690	Ncaph2	non-SMC condensin II complex, subunit H2 [Source	0.048328
ENSMUSG00000055447	Cd47	CD47 antigen (Rh-related antigen, integrin-associat	0.048439
ENSMUSG00000043263	Pyhin1	pyrin and HIN domain family, member 1 [Source:M	0.048472
ENSMUSG00000073910	Mob3b	MOB kinase activator 3B [Source:MGI Symbol;Acc:M	0.048499
ENSMUSG00000084739	Gm22067	predicted gene, 22067 [Source:MGI Symbol;Acc:MG	0.048522
ENSMUSG00000064210	Ano6	anoctamin 6 [Source:MGI Symbol;Acc:MGI:214589(	0.048613
ENSMUSG00000034875	Nudt19	nudix (nucleoside diphosphate linked moiety X)-typ	0.048626
ENSMUSG00000020074	Ccar1	cell division cycle and apoptosis regulator 1 [Source	0.048631
ENSMUSG00000073421	H2-Ab1	histocompatibility 2, class II antigen A, beta 1 [Sour	0.048648
ENSMUSG00000022141	Nipbl	Nipped-B homolog (Drosophila) [Source:MGI Symb	0.048836
ENSMUSG00000074303	Gm10664	predicted gene 10664 [Source:MGI Symbol;Acc:MG	0.048844
ENSMUSG00000060470	Gpr97	G protein-coupled receptor 97 [Source:MGI Symbo	0.048845
ENSMUSG00000035279	Ssc5d	scavenger receptor cysteine rich domain containing	0.048899
ENSMUSG00000028976	Slc2a5	solute carrier family 2 (facilitated glucose transport	0.048963
ENSMUSG00000014602	Kif1a	kinesin family member 1A [Source:MGI Symbol;Acc	0.048975
ENSMUSG00000001986	Gria3	glutamate receptor, ionotropic, AMPA3 (alpha 3) [S	0.048985
ENSMUSG00000039087	Rreb1	ras responsive element binding protein 1 [Source:M	0.048994
ENSMUSG00000031906	Smpd3	sphingomyelin phosphodiesterase 3, neutral [Sourc	0.049053
ENSMUSG00000020109	Dnajb12	DnaJ (Hsp40) homolog, subfamily B, member 12 [So	0.049135
ENSMUSG00000031077	Fadd	Fas (TNFRSF6)-associated via death domain [Source	0.049153
ENSMUSG00000018672	Copz2	coatomer protein complex, subunit zeta 2 [Source:I	0.04916
ENSMUSG00000039005	Tlr4	toll-like receptor 4 [Source:MGI Symbol;Acc:MGI:96	0.049186
ENSMUSG00000031382	Asb11	ankyrin repeat and SOCS box-containing 11 [Source	0.049213
ENSMUSG00000002028	Kmt2a	lysine (K)-specific methyltransferase 2A [Source:MG	0.04924
ENSMUSG00000024844	Banf1	barrier to autointegration factor 1 [Source:MGI Syn	0.049261
ENSMUSG00000062198	2700097O09Rik	RIKEN cDNA 2700097O09 gene [Source:MGI Symbo	0.049282
ENSMUSG00000028042	Zbtb7b	zinc finger and BTB domain containing 7B [Source:M	0.049301
ENSMUSG00000055865	Fam19a3	family with sequence similarity 19, member A3 [Sou	0.049322
ENSMUSG00000097236	4831440E17Rik	RIKEN cDNA 4831440E17 gene [Source:MGI Symbo	0.049411
ENSMUSG00000029777	Gars	glycyl-tRNA synthetase [Source:MGI Symbol;Acc:M	0.049454
ENSMUSG00000030786	Itgam	integrin alpha M [Source:MGI Symbol;Acc:MGI:966	0.049454
ENSMUSG00000030614	Tmem126b	transmembrane protein 126B [Source:MGI Symbol;	0.049528
ENSMUSG00000021904	Sema3g	sema domain, immunoglobulin domain (Ig), short b	0.049561
ENSMUSG00000042104	Uggt2	UDP-glucose glycoprotein glucosyltransferase 2 [So	0.049564
ENSMUSG00000027201	Myef2	myelin basic protein expression factor 2, repressor	0.049567
ENSMUSG00000034837	Gnat1	guanine nucleotide binding protein, alpha transduc	0.049586

ENSMUSG00000001700	Gramd3	GRAM domain containing 3 [Source:MGI Symbol;Ac	0.049608
ENSMUSG00000020124	Usp15	ubiquitin specific peptidase 15 [Source:MGI Symbo	0.049666
ENSMUSG00000047237	Fbxw21	F-box and WD-40 domain protein 21 [Source:MGI S	0.049698
ENSMUSG00000033323	Ctdp1	CTD (carboxy-terminal domain, RNA polymerase II,	0.049718
ENSMUSG00000084587	n-R5s174	nuclear encoded rRNA 5S 174 [Source:MGI Symbol;	0.049728
ENSMUSG00000061524	Zic2	zinc finger protein of the cerebellum 2 [Source:MG	0.049729
ENSMUSG00000041231	Ublcp1	ubiquitin-like domain containing CTD phosphatase	0.049748
ENSMUSG00000033191	Tie1	tyrosine kinase with immunoglobulin-like and EGF-l	0.049754
ENSMUSG00000086095	Gm15328	predicted gene 15328 [Source:MGI Symbol;Acc:MG	0.049767
ENSMUSG00000027959	Sass6	spindle assembly 6 homolog (C. elegans) [Source:M	0.049809
ENSMUSG00000032316	Clk3	CDC-like kinase 3 [Source:MGI Symbol;Acc:MGI:109	0.049841
ENSMUSG00000041498	Kif14	kinesin family member 14 [Source:MGI Symbol;Acc	0.049847
ENSMUSG00000058966	Fam57b	family with sequence similarity 57, member B [Sou	0.049849
ENSMUSG00000006519	Cyba	cytochrome b-245, alpha polypeptide [Source:MGI	0.049875
ENSMUSG00000026100	Mstn	myostatin [Source:MGI Symbol;Acc:MGI:95691]	0.049915
ENSMUSG00000072582	Ptrh2	peptidyl-tRNA hydrolase 2 [Source:MGI Symbol;Acc	0.049927
ENSMUSG00000004980	Hnrnpa2b1	heterogeneous nuclear ribonucleoprotein A2/B1 [S	0.04993
ENSMUSG00000029687	Ezh2	enhancer of zeste homolog 2 (Drosophila) [Source:I	0.049973
ENSMUSG00000097364	Gm26719	predicted gene, 26719 [Source:MGI Symbol;Acc:MG	0.049975
ENSMUSG00000094326	Gm10045	predicted pseudogene 10045 [Source:MGI Symbol;	0.049977
ENSMUSG00000055044	Pdlim1	PDZ and LIM domain 1 (elfin) [Source:MGI Symbol;A	0.049998
ENSMUSG00000061311	Rag1	recombination activating gene 1 [Source:MGI Symb	0.050027
ENSMUSG00000038071	Npy6r	neuropeptide Y receptor Y6 [Source:MGI Symbol;Ac	0.050121
ENSMUSG00000024858	Adrbk1	adrenergic receptor kinase, beta 1 [Source:MGI Syr	0.050123
ENSMUSG00000010290	AI597479	expressed sequence AI597479 [Source:MGI Symbol	0.050222
ENSMUSG00000042477	Tfap2e	transcription factor AP-2, epsilon [Source:MGI Sym	0.050248
ENSMUSG00000021262	Evl	Ena-vasodilator stimulated phosphoprotein [Source	0.050321
ENSMUSG00000086918	4930429F24Rik	RIKEN cDNA 4930429F24 gene [Source:MGI Symbo	0.050334
ENSMUSG00000029270	Fam69a	family with sequence similarity 69, member A [Sou	0.050372
ENSMUSG00000083421	Gm14776	predicted gene 14776 [Source:MGI Symbol;Acc:MG	0.050497
ENSMUSG00000019132	BC005537	cDNA sequence BC005537 [Source:MGI Symbol;Acc	0.050559
ENSMUSG00000035125	Gcfc2	GC-rich sequence DNA binding factor 2 [Source:MG	0.05061
ENSMUSG00000026188	Tmem169	transmembrane protein 169 [Source:MGI Symbol;A	0.050642
ENSMUSG00000058392	Rrp1b	ribosomal RNA processing 1 homolog B (S. cerevisia	0.05065
ENSMUSG00000027452	Acss1	acyl-CoA synthetase short-chain family member 1 [	0.050692
ENSMUSG00000042453	Reln	reelin [Source:MGI Symbol;Acc:MGI:103022]	0.050706
ENSMUSG00000026153	Fam135a	family with sequence similarity 135, member A [Sou	0.050789
ENSMUSG00000049670	Morn4	MORN repeat containing 4 [Source:MGI Symbol;Ac	0.0508
ENSMUSG00000003345	Csnk1g2	casein kinase 1, gamma 2 [Source:MGI Symbol;Acc:	0.050867
ENSMUSG00000032562	Gnai2	guanine nucleotide binding protein (G protein), alp	0.050889
ENSMUSG00000082383	Gm9670	predicted gene 9670 [Source:MGI Symbol;Acc:MGI:	0.050923
ENSMUSG00000023186	Vwa5a	von Willebrand factor A domain containing 5A [Sou	0.050966
ENSMUSG00000015340	Cybb	cytochrome b-245, beta polypeptide [Source:MGI S	0.051086
ENSMUSG00000042793	Lgr6	leucine-rich repeat-containing G protein-coupled re	0.0511
ENSMUSG00000030683	Sez6l2	seizure related 6 homolog like 2 [Source:MGI Symb	0.051129
ENSMUSG00000082896	Gm5844	predicted gene 5844 [Source:MGI Symbol;Acc:MGI:	0.051154
ENSMUSG00000020325	Fstl3	follistatin-like 3 [Source:MGI Symbol;Acc:MGI:1890	0.051178

ENSMUSG00000062353	Gm15772	predicted gene 15772 [Source:MGI Symbol;Acc:MG	0.051265
ENSMUSG00000087620	5330434G04Rik	RIKEN cDNA 5330434G04 gene [Source:MGI Symbo	0.051317
ENSMUSG00000073295	Nudt11	nudix (nucleoside diphosphate linked moiety X)-typ	0.051348
ENSMUSG00000000385	Tmprss2	transmembrane protease, serine 2 [Source:MGI Sy	0.051374
ENSMUSG00000041115	Iqsec2	IQ motif and Sec7 domain 2 [Source:MGI Symbol;A	0.051392
ENSMUSG00000050671	Ism2	isthmin 2 homolog (zebrafish) [Source:MGI Symbol	0.051421
ENSMUSG00000044681	Cnpy1	canopy 1 homolog (zebrafish) [Source:MGI Symbol;	0.051424
ENSMUSG00000022635	Zcrb1	zinc finger CCHC-type and RNA binding motif 1 [Sou	0.051434
ENSMUSG00000097393	D030068K23Rik	RIKEN cDNA D030068K23 gene [Source:MGI Symbo	0.051441
ENSMUSG00000044548	Dact1	dapper homolog 1, antagonist of beta-catenin (xen	0.0515
ENSMUSG00000024784	Gpha2	glycoprotein hormone alpha 2 [Source:MGI Symbol	0.051544
ENSMUSG00000024713	Pcsk5	proprotein convertase subtilisin/kexin type 5 [Sour	0.051655
ENSMUSG00000020658	Efr3b	EFR3 homolog B (S. cerevisiae) [Source:MGI Symbo	0.051696
ENSMUSG00000032741	Tpcn1	two pore channel 1 [Source:MGI Symbol;Acc:MGI:2	0.051699
ENSMUSG00000033088	Triobp	TRIO and F-actin binding protein [Source:MGI Symb	0.051707
ENSMUSG00000030002	Dusp11	dual specificity phosphatase 11 (RNA/RNP complex	0.051757
ENSMUSG00000027811	4930579G24Rik	RIKEN cDNA 4930579G24 gene [Source:MGI Symbo	0.051824
ENSMUSG00000040688	Tbl3	transducin (beta)-like 3 [Source:MGI Symbol;Acc:M	0.051825
ENSMUSG00000039623	Ap5z1	adaptor-related protein complex 5, zeta 1 subunit [	0.051845
ENSMUSG00000043773	1700048O20Rik	RIKEN cDNA 1700048O20 gene [Source:MGI Symbo	0.05186
ENSMUSG00000035045	Zc3h12b	zinc finger CCCH-type containing 12B [Source:MGI S	0.051879
ENSMUSG00000037259	Dzank1	double zinc ribbon and ankyrin repeat domains 1 [S	0.05191
ENSMUSG00000030753	Prkrir	protein-kinase, interferon-inducible double strande	0.051926
ENSMUSG00000086470	Gm12580	predicted gene 12580 [Source:MGI Symbol;Acc:MG	0.051934
ENSMUSG00000058835	Abi1	abl-interactor 1 [Source:MGI Symbol;Acc:MGI:1049	0.051946
ENSMUSG00000026176	Ctdsp1	CTD (carboxy-terminal domain, RNA polymerase II,	0.05195
ENSMUSG00000033423	Eri3	exoribonuclease 3 [Source:MGI Symbol;Acc:MGI:21	0.052
ENSMUSG00000026096	Osgepl1	O-sialoglycoprotein endopeptidase-like 1 [Source:M	0.052033
ENSMUSG00000028221	Tmem55a	transmembrane protein 55A [Source:MGI Symbol;A	0.052038
ENSMUSG00000021258	Ccnk	cyclin K [Source:MGI Symbol;Acc:MGI:1276106]	0.052068
ENSMUSG00000001056	Nhp2	NHP2 ribonucleoprotein [Source:MGI Symbol;Acc:M	0.052081
ENSMUSG00000045691	Thtpa	thiamine triphosphatase [Source:MGI Symbol;Acc:M	0.052141
ENSMUSG00000022442	Ttll1	tubulin tyrosine ligase-like 1 [Source:MGI Symbol;A	0.052163
ENSMUSG00000031386	Hcfc1	host cell factor C1 [Source:MGI Symbol;Acc:MGI:10	0.052172
ENSMUSG00000054737	Zfp182	zinc finger protein 182 [Source:MGI Symbol;Acc:MG	0.052264
ENSMUSG00000020936	Nmt1	N-myristoyltransferase 1 [Source:MGI Symbol;Acc:I	0.05227
ENSMUSG00000037513	Samd4b	sterile alpha motif domain containing 4B [Source:M	0.052303
ENSMUSG00000040681	Hmgn1	high mobility group nucleosomal binding domain 1	0.052318
ENSMUSG00000047246	Hist1h2be	histone cluster 1, H2be [Source:MGI Symbol;Acc:M	0.052324
ENSMUSG00000021974	Fgf9	fibroblast growth factor 9 [Source:MGI Symbol;Acc	0.052381
ENSMUSG00000049649	Gpr3	G-protein coupled receptor 3 [Source:MGI Symbol;	0.052436
ENSMUSG00000024247	Pkdcc	protein kinase domain containing, cytoplasmic [Sou	0.052441
ENSMUSG00000084808	9430091E24Rik	RIKEN cDNA 9430091E24 gene [Source:MGI Symbo	0.052478
ENSMUSG00000060601	Nr1h2	nuclear receptor subfamily 1, group H, member 2 [S	0.052513
ENSMUSG00000056071	S100a9	S100 calcium binding protein A9 (calgranulin B) [So	0.052543
ENSMUSG00000022512	Cldn1	claudin 1 [Source:MGI Symbol;Acc:MGI:1276109]	0.052584
ENSMUSG00000033220	Rac2	RAS-related C3 botulinum substrate 2 [Source:MGI	0.0526

ENSMUSG00000060703	Cd302	CD302 antigen [Source:MGI Symbol;Acc:MGI:1913	0.05262
ENSMUSG00000041144	Dnah7b	dynein, axonemal, heavy chain 7B [Source:MGI Sym	0.052621
ENSMUSG00000040957	Cables1	CDK5 and Abl enzyme substrate 1 [Source:MGI Sym	0.052637
ENSMUSG00000036199	Ndufa13	NADH dehydrogenase (ubiquinone) 1 alpha subcom	0.052703
ENSMUSG00000021760	Gpx8	glutathione peroxidase 8 (putative) [Source:MGI Sy	0.05273
ENSMUSG00000027195	Hsd17b12	hydroxysteroid (17-beta) dehydrogenase 12 [Sourc	0.052786
ENSMUSG00000029700	Slc13a1	solute carrier family 13 (sodium/sulfate symporters	0.052801
ENSMUSG00000030123	Plxnd1	plexin D1 [Source:MGI Symbol;Acc:MGI:2154244]	0.05284
ENSMUSG00000019261	Map1s	microtubule-associated protein 1S [Source:MGI Syr	0.052853
ENSMUSG00000045287	Rtn4rl1	reticulon 4 receptor-like 1 [Source:MGI Symbol;Acc	0.052856
ENSMUSG00000052613	Pcdh15	protocadherin 15 [Source:MGI Symbol;Acc:MGI:18	0.052858
ENSMUSG00000037593	BC030499	cDNA sequence BC030499 [Source:MGI Symbol;Acc	0.052887
ENSMUSG00000016637	Ift27	intraflagellar transport 27 [Source:MGI Symbol;Acc	0.052891
ENSMUSG00000030432	Rpl28	ribosomal protein L28 [Source:MGI Symbol;Acc:MG	0.052973
ENSMUSG00000042256	Ptchd4	patched domain containing 4 [Source:MGI Symbol;	0.052973
ENSMUSG00000067780	Pi15	peptidase inhibitor 15 [Source:MGI Symbol;Acc:MG	0.053031
ENSMUSG00000002580	Mien1	migration and invasion enhancer 1 [Source:MGI Syr	0.053047
ENSMUSG00000090397	Gm17096	predicted gene 17096 [Source:MGI Symbol;Acc:MG	0.053076
ENSMUSG00000093904	Tomm20	translocase of outer mitochondrial membrane 20 h	0.05309
ENSMUSG00000089715	Cbx6	chromobox 6 [Source:MGI Symbol;Acc:MGI:351262	0.053099
ENSMUSG00000032671	A930018P22Rik	RIKEN cDNA A930018P22 gene [Source:MGI Symbo	0.053247
ENSMUSG00000063904	Dpp3	dipeptidylpeptidase 3 [Source:MGI Symbol;Acc:MG	0.053276
ENSMUSG00000086506	Gm15911	predicted gene 15911 [Source:MGI Symbol;Acc:MG	0.053278
ENSMUSG00000020368	Canx	calnexin [Source:MGI Symbol;Acc:MGI:88261]	0.053295
ENSMUSG00000001910	Nacc1	nucleus accumbens associated 1, BEN and BTB (PO	0.053303
ENSMUSG00000027637	1110008F13Rik	RIKEN cDNA 1110008F13 gene [Source:MGI Symbo	0.053344
ENSMUSG00000025933	Tmem14a	transmembrane protein 14A [Source:MGI Symbol;A	0.053378
ENSMUSG00000021236	Entpd5	ectonucleoside triphosphate diphosphohydrolase 5	0.053401
ENSMUSG00000032402	Smad3	SMAD family member 3 [Source:MGI Symbol;Acc:M	0.053414
ENSMUSG00000028618	Tmem59	transmembrane protein 59 [Source:MGI Symbol;Ac	0.05344
ENSMUSG00000086342	Gm12932	predicted gene 12932 [Source:MGI Symbol;Acc:MG	0.053492
ENSMUSG00000063689	Hist2h2ab	histone cluster 2, H2ab [Source:MGI Symbol;Acc:M	0.053522
ENSMUSG00000051177	Plcb1	phospholipase C, beta 1 [Source:MGI Symbol;Acc:M	0.053596
ENSMUSG00000028010	Gar1	GAR1 ribonucleoprotein homolog (yeast) [Source:M	0.053613
ENSMUSG00000025817	Nudt5	nudix (nucleoside diphosphate linked moiety X)-typ	0.053718
ENSMUSG00000078496	Gm13152	predicted gene 13152 [Source:MGI Symbol;Acc:MG	0.053778
ENSMUSG00000019873	Reep3	receptor accessory protein 3 [Source:MGI Symbol;A	0.053822
ENSMUSG00000024145	Pigf	phosphatidylinositol glycan anchor biosynthesis, cla	0.053865
ENSMUSG00000038384	Setd1b	SET domain containing 1B [Source:MGI Symbol;Acc	0.053875
ENSMUSG00000042202	Slc35e2	solute carrier family 35, member E2 [Source:MGI Sy	0.05388
ENSMUSG00000039601	Rcan2	regulator of calcineurin 2 [Source:MGI Symbol;Acc:	0.053894
ENSMUSG00000062488	I830012O16Rik	RIKEN cDNA I830012O16 gene [Source:MGI Symbo	0.053898
ENSMUSG00000022972	1110004E09Rik	RIKEN cDNA 1110004E09 gene [Source:MGI Symbo	0.054047
ENSMUSG00000032067	Pts	6-pyruvoyl-tetrahydropterin synthase [Source:MGI	0.054078
ENSMUSG00000086414	Gm15645	predicted gene 15645 [Source:MGI Symbol;Acc:MG	0.0541
ENSMUSG00000024077	Strn	striatin, calmodulin binding protein [Source:MGI Sy	0.054135
ENSMUSG00000039531	Zufsp	zinc finger with UFM1-specific peptidase domain [S	0.054137

ENSMUSG00000032263	Bckdhb	branched chain ketoacid dehydrogenase E1, beta p	0.054166
ENSMUSG00000018481	Appbp2	amyloid beta precursor protein (cytoplasmic tail) bi	0.054199
ENSMUSG00000006526	Tmem110	transmembrane protein 110 [Source:MGI Symbol;A	0.054205
ENSMUSG00000030376	Slc8a2	solute carrier family 8 (sodium/calcium exchanger),	0.054257
ENSMUSG00000063632	Sox11	SRY-box containing gene 11 [Source:MGI Symbol;Ac	0.054278
ENSMUSG00000058006	Mdn1	midasin homolog (yeast) [Source:MGI Symbol;Acc:M	0.054393
ENSMUSG00000030059	Tmf1	TATA element modulatory factor 1 [Source:MGI Sy	0.054455
ENSMUSG00000086632	1700112J05Rik	RIKEN cDNA 1700112J05 gene [Source:MGI Symbol	0.054498
ENSMUSG00000049553	Polr1a	polymerase (RNA) I polypeptide A [Source:MGI Sym	0.054508
ENSMUSG00000001300	Efnb2	ephrin B2 [Source:MGI Symbol;Acc:MGI:105097]	0.054598
ENSMUSG00000036678	Aaas	achalasia, adrenocortical insufficiency, alacrimia [So	0.054626
ENSMUSG00000021557	Agtpbp1	ATP/GTP binding protein 1 [Source:MGI Symbol;Acc	0.054638
ENSMUSG00000027488	Snta1	syntrophin, acidic 1 [Source:MGI Symbol;Acc:MGI:1	0.054646
ENSMUSG00000000934	Top1mt	DNA topoisomerase 1, mitochondrial [Source:MGI S	0.05468
ENSMUSG00000022744	Cldn25	claudin 25 [Source:MGI Symbol;Acc:MGI:2447860]	0.054693
ENSMUSG00000068039	Tcp1	t-complex protein 1 [Source:MGI Symbol;Acc:MGI:9	0.054795
ENSMUSG00000055632	Hmcn2	hemicentin 2 [Source:MGI Symbol;Acc:MGI:267783	0.054884
ENSMUSG00000035472	Slc25a21	solute carrier family 25 (mitochondrial oxodicarbox	0.054888
ENSMUSG00000049076	Acap2	ArfGAP with coiled-coil, ankyrin repeat and PH dom	0.05491
ENSMUSG00000057335	Cep170	centrosomal protein 170 [Source:MGI Symbol;Acc:M	0.054939
ENSMUSG00000040506	Ambra1	autophagy/beclin 1 regulator 1 [Source:MGI Symbo	0.05494
ENSMUSG00000022421	Nptxr	neuronal pentraxin receptor [Source:MGI Symbol;A	0.054962
ENSMUSG00000025395	Prim1	DNA primase, p49 subunit [Source:MGI Symbol;Acc	0.054992
ENSMUSG00000088323	Gm25663	predicted gene, 25663 [Source:MGI Symbol;Acc:MG	0.055019
ENSMUSG00000070493	Chchd2	coiled-coil-helix-coiled-coil-helix domain containing	0.055032
ENSMUSG00000098025	Gm27013	predicted gene, 27013 [Source:MGI Symbol;Acc:MG	0.055049
ENSMUSG00000003680	Taf6l	TAF6-like RNA polymerase II, p300/CBP-associated	0.055054
ENSMUSG00000022429	Dmc1	DMC1 dosage suppressor of mck1 homolog, meiosi	0.055131
ENSMUSG00000066551	Hmgb1	high mobility group box 1 [Source:MGI Symbol;Acc:	0.055218
ENSMUSG00000034926	Dhcr24	24-dehydrocholesterol reductase [Source:MGI Sym	0.055239
ENSMUSG00000030188	Magohb	mago-nashi homolog B (Drosophila) [Source:MGI Sy	0.055245
ENSMUSG00000035399	Oser1	oxidative stress responsive serine rich 1 [Source:MG	0.055363
ENSMUSG00000027598	Itch	itchy, E3 ubiquitin protein ligase [Source:MGI Symb	0.055374
ENSMUSG00000057143	Trim12c	tripartite motif-containing 12C [Source:MGI Symbo	0.05538
ENSMUSG00000035969	Rusc2	RUN and SH3 domain containing 2 [Source:MGI Syn	0.055391
ENSMUSG00000026851	BC005624	cDNA sequence BC005624 [Source:MGI Symbol;Acc	0.055425
ENSMUSG00000027424	Mgme1	mitochondrial genome maintainance exonuclease 1	0.055466
ENSMUSG00000020549	Elac2	elaC homolog 2 (E. coli) [Source:MGI Symbol;Acc:M	0.055473
ENSMUSG00000097504	4930516B21Rik	RIKEN cDNA 4930516B21 gene [Source:MGI Symbo	0.055497
ENSMUSG00000003053	Cyp2c29	cytochrome P450, family 2, subfamily c, polypeptid	0.055502
ENSMUSG00000063897	CAAA01118383.1	dehydrogenase/reductase (SDR family) X chromoso	0.055502
ENSMUSG00000089827	1700023H06Rik	RIKEN cDNA 1700023H06 gene [Source:MGI Symbo	0.055519
ENSMUSG00000072852	2310040G07Rik	RIKEN cDNA 2310040G07 gene [Source:MGI Symbo	0.055551
ENSMUSG00000059842	Zfp341	zinc finger protein 341 [Source:MGI Symbol;Acc:MG	0.055572
ENSMUSG00000003033	Ap1m1	adaptor-related protein complex AP-1, mu subunit	0.055607
ENSMUSG00000040928	S100pbp	S100P binding protein [Source:MGI Symbol;Acc:MG	0.055686
ENSMUSG00000052117	D630039A03Rik	RIKEN cDNA D630039A03 gene [Source:MGI Symbo	0.055706

ENSMUSG00000085151	1110018N20Rik	RIKEN cDNA 1110018N20 gene [Source:MGI Symbo	0.055738
ENSMUSG00000033060	Lmo7	LIM domain only 7 [Source:MGI Symbol;Acc:MGI:1	0.055745
ENSMUSG00000039199	Zdhhc1	zinc finger, DHHC domain containing 1 [Source:MG	0.055765
ENSMUSG00000028691	Prdx1	peroxiredoxin 1 [Source:MGI Symbol;Acc:MGI:9952	0.055768
ENSMUSG00000015401	Tmem27	transmembrane protein 27 [Source:MGI Symbol;Ac	0.055801
ENSMUSG00000084440	Gm26254	predicted gene, 26254 [Source:MGI Symbol;Acc:M	0.055826
ENSMUSG00000088549	Gm24959	predicted gene, 24959 [Source:MGI Symbol;Acc:M	0.055839
ENSMUSG00000040121	Rep15	RAB15 effector protein [Source:MGI Symbol;Acc:M	0.055881
ENSMUSG00000072875	Gpr27	G protein-coupled receptor 27 [Source:MGI Symbo	0.05589
ENSMUSG00000058070	Eml1	echinoderm microtubule associated protein like 1 [	0.055898
ENSMUSG00000034958	Atcay	ataxia, cerebellar, Cayman type homolog (human) [	0.055977
ENSMUSG00000038538	Ubn2	ubinuclein 2 [Source:MGI Symbol;Acc:MGI:244423	0.055983
ENSMUSG00000004054	Map3k11	mitogen-activated protein kinase kinase kinase 11 [	0.056007
ENSMUSG00000040549	Ckap5	cytoskeleton associated protein 5 [Source:MGI Sym	0.056068
ENSMUSG00000001229	Dpp9	dipeptidylpeptidase 9 [Source:MGI Symbol;Acc:MG	0.056101
ENSMUSG00000027667	Zfp639	zinc finger protein 639 [Source:MGI Symbol;Acc:M	0.056106
ENSMUSG00000032637	Atxn2l	ataxin 2-like [Source:MGI Symbol;Acc:MGI:244624	0.056125
ENSMUSG00000078185	Chml	choroideremia-like [Source:MGI Symbol;Acc:MGI:1	0.056134
ENSMUSG00000085589	A430078I02Rik	RIKEN cDNA A430078I02 gene [Source:MGI Symbol	0.056135
ENSMUSG00000037996	Slc24a2	solute carrier family 24 (sodium/potassium/calcium	0.056149
ENSMUSG00000046519	Golph3l	golgi phosphoprotein 3-like [Source:MGI Symbol;A	0.05615
ENSMUSG00000086184	Gm12764	predicted gene 12764 [Source:MGI Symbol;Acc:MG	0.056205
ENSMUSG00000002014	Ssr4	signal sequence receptor, delta [Source:MGI Symbo	0.056213
ENSMUSG00000020646	Mboat2	membrane bound O-acyltransferase domain contai	0.056219
ENSMUSG00000002957	Ap2a2	adaptor-related protein complex 2, alpha 2 subunit	0.056246
ENSMUSG00000024782	Ak3	adenylate kinase 3 [Source:MGI Symbol;Acc:MGI:1	0.056307
ENSMUSG00000030751	Psma1	proteasome (prosome, macropain) subunit, alpha t	0.056309
ENSMUSG00000024778	Fas	Fas (TNF receptor superfamily member 6) [Source:	0.056356
ENSMUSG00000083104	Gm12917	predicted gene 12917 [Source:MGI Symbol;Acc:MG	0.056397
ENSMUSG00000037221	Mospd3	motile sperm domain containing 3 [Source:MGI Sym	0.056404
ENSMUSG00000037434	Slc30a1	solute carrier family 30 (zinc transporter), member	0.056414
ENSMUSG00000008140	Emc10	ER membrane protein complex subunit 10 [Source:	0.056424
ENSMUSG00000000561	Wdr77	WD repeat domain 77 [Source:MGI Symbol;Acc:MG	0.056493
ENSMUSG00000039176	Polg	polymerase (DNA directed), gamma [Source:MGI Sy	0.056557
ENSMUSG00000050822	Slc29a4	solute carrier family 29 (nucleoside transporters), n	0.056568
ENSMUSG00000084843	B230312C02Rik	RIKEN cDNA B230312C02 gene [Source:MGI Symbo	0.056608
ENSMUSG00000046828	Mettl21e	methyltransferase like 21E [Source:MGI Symbol;Ac	0.05661
ENSMUSG00000043613	Mmp3	matrix metallopeptidase 3 [Source:MGI Symbol;Ac	0.056641
ENSMUSG00000051043	Gprc5c	G protein-coupled receptor, family C, group 5, mem	0.056653
ENSMUSG00000045671	Spred2	sprouty-related, EVH1 domain containing 2 [Source	0.056887
ENSMUSG00000040249	Lrp1	low density lipoprotein receptor-related protein 1 [	0.056906
ENSMUSG00000022340	Sybu	syntabulin (syntaxin-interacting) [Source:MGI Symb	0.056962
ENSMUSG00000033768	Nrxn2	neurexin II [Source:MGI Symbol;Acc:MGI:1096362]	0.056991
ENSMUSG00000022552	Sharpin	SHANK-associated RH domain interacting protein [S	0.057035
ENSMUSG00000085397	Gm13524	predicted gene 13524 [Source:MGI Symbol;Acc:MG	0.057097
ENSMUSG00000090877	Hspa1b	heat shock protein 1B [Source:MGI Symbol;Acc:MG	0.057163
ENSMUSG00000025738	Fbxl16	F-box and leucine-rich repeat protein 16 [Source:M	0.057214

ENSMUSG00000075502	Zbtbd6	kelch repeat and BTB (POZ) domain containing 6 [S	0.057219
ENSMUSG00000020834	Dhrs13	dehydrogenase/reductase (SDR family) member 13	0.057242
ENSMUSG00000079048	4933413L06Rik	RIKEN cDNA 4933413L06 gene [Source:MGI Symbo	0.057269
ENSMUSG00000025359	Pmel	premelanosome protein [Source:MGI Symbol;Acc:M	0.057288
ENSMUSG00000063015	Ccni	cyclin I [Source:MGI Symbol;Acc:MGI:1341077]	0.057292
ENSMUSG00000039262	Prrc2b	proline-rich coiled-coil 2B [Source:MGI Symbol;Acc	0.057335
ENSMUSG00000037902	Sirpa	signal-regulatory protein alpha [Source:MGI Symbo	0.05736
ENSMUSG00000026646	Suv39h2	suppressor of variegation 3-9 homolog 2 (Drosophi	0.057378
ENSMUSG00000036391	Sec24a	Sec24 related gene family, member A (S. cerevisiae	0.057431
ENSMUSG00000025995	Wdr75	WD repeat domain 75 [Source:MGI Symbol;Acc:MG	0.057452
ENSMUSG00000071860	2900055J20Rik	RIKEN cDNA 2900055J20 gene [Source:MGI Symbol	0.05748
ENSMUSG00000059003	Grin2a	glutamate receptor, ionotropic, NMDA2A (epsilon 1	0.057495
ENSMUSG00000005983	1700037C18Rik	RIKEN cDNA 1700037C18 gene [Source:MGI Symbo	0.057497
ENSMUSG00000022280	Rnf19a	ring finger protein 19A [Source:MGI Symbol;Acc:M	0.05765
ENSMUSG00000030000	Add2	adducin 2 (beta) [Source:MGI Symbol;Acc:MGI:879	0.057686
ENSMUSG00000022037	Clu	clusterin [Source:MGI Symbol;Acc:MGI:88423]	0.05769
ENSMUSG00000032096	Arcn1	archain 1 [Source:MGI Symbol;Acc:MGI:2387591]	0.057706
ENSMUSG00000035621	Midn	midnolin [Source:MGI Symbol;Acc:MGI:1890222]	0.057749
ENSMUSG00000018678	Sp2	Sp2 transcription factor [Source:MGI Symbol;Acc:M	0.057777
ENSMUSG00000085311	Gm15445	predicted gene 15445 [Source:MGI Symbol;Acc:MG	0.057777
ENSMUSG00000027602	Map1lc3a	microtubule-associated protein 1 light chain 3 alph	0.057801
ENSMUSG00000086015	4833417C18Rik	RIKEN cDNA 4833417C18 gene [Source:MGI Symbo	0.057805
ENSMUSG00000051243	Islr2	immunoglobulin superfamily containing leucine-ric	0.057813
ENSMUSG00000024807	Syvn1	synovial apoptosis inhibitor 1, synoviolin [Source:M	0.057994
ENSMUSG00000056851	Pcbp2	poly(rC) binding protein 2 [Source:MGI Symbol;Acc	0.058094
ENSMUSG00000035725	Prkx	protein kinase, X-linked [Source:MGI Symbol;Acc:M	0.058137
ENSMUSG00000031372	Trex2	three prime repair exonuclease 2 [Source:MGI Sym	0.058194
ENSMUSG00000073436	Eme2	essential meiotic endonuclease 1 homolog 2 (S. por	0.058196
ENSMUSG00000033276	Stk36	serine/threonine kinase 36 [Source:MGI Symbol;Ac	0.058249
ENSMUSG00000079002	C030006K11Rik	RIKEN cDNA C030006K11 gene [Source:MGI Symbo	0.058347
ENSMUSG00000044763	Trmt10c	tRNA methyltransferase 10C [Source:MGI Symbol;A	0.058351
ENSMUSG00000026585	Kifap3	kinesin-associated protein 3 [Source:MGI Symbol;A	0.058355
ENSMUSG00000024581	Napg	N-ethylmaleimide sensitive fusion protein attachm	0.058375
ENSMUSG00000033453	Adamts15	a disintegrin-like and metallopeptidase (reprolysin	0.058388
ENSMUSG00000006276	Eps15l1	epidermal growth factor receptor pathway substra	0.058408
ENSMUSG00000044700	Tmem201	transmembrane protein 201 [Source:MGI Symbol;A	0.058421
ENSMUSG00000005142	Man2b1	mannosidase 2, alpha B1 [Source:MGI Symbol;Acc:	0.058452
ENSMUSG00000049690	Nckap5	NCK-associated protein 5 [Source:MGI Symbol;Acc:	0.058486
ENSMUSG00000036598	Ccdc113	coiled-coil domain containing 113 [Source:MGI Sym	0.058492
ENSMUSG00000050708	Ftl1	ferritin light chain 1 [Source:MGI Symbol;Acc:MGI:9	0.058516
ENSMUSG00000071719	Tmem28	transmembrane protein 28 [Source:MGI Symbol;Ac	0.058517
ENSMUSG00000021708	Rasgrf2	RAS protein-specific guanine nucleotide-releasing f	0.058565
ENSMUSG00000061479	Snrpa	small nuclear ribonucleoprotein polypeptide A [Sou	0.058566
ENSMUSG00000030876	Mettl9	methyltransferase like 9 [Source:MGI Symbol;Acc:M	0.058603
ENSMUSG00000039157	Fam102a	family with sequence similarity 102, member A [Sou	0.05865
ENSMUSG00000028559	Osbpl9	oxysterol binding protein-like 9 [Source:MGI Symbo	0.058658
ENSMUSG00000036565	Ttyh3	tweety homolog 3 (Drosophila) [Source:MGI Symbo	0.058736

ENSMUSG00000000628	Hk2	hexokinase 2 [Source:MGI Symbol;Acc:MGI:131519	0.058743
ENSMUSG00000026610	Esrrg	estrogen-related receptor gamma [Source:MGI Sym	0.058751
ENSMUSG00000037553	Zdhhc18	zinc finger, DHHC domain containing 18 [Source:MG	0.058764
ENSMUSG00000024081	Cebpz	CCAAT/enhancer binding protein zeta [Source:MGI	0.058784
ENSMUSG00000048970	C1galt1c1	C1GALT1-specific chaperone 1 [Source:MGI Symbol	0.058887
ENSMUSG00000041193	Pla2g5	phospholipase A2, group V [Source:MGI Symbol;Ac	0.058923
ENSMUSG00000083274	Gm14080	predicted gene 14080 [Source:MGI Symbol;Acc:MG	0.058931
ENSMUSG00000043670	Diras1	DIRAS family, GTP-binding RAS-like 1 [Source:MGI S	0.058935
ENSMUSG00000020211	Sf3a2	splicing factor 3a, subunit 2 [Source:MGI Symbol;Ac	0.058962
ENSMUSG00000040220	Gas8	growth arrest specific 8 [Source:MGI Symbol;Acc:M	0.05899
ENSMUSG00000028495	Rps6	ribosomal protein S6 [Source:MGI Symbol;Acc:MGI	0.059042
ENSMUSG00000023087	Ccrn4l	CCR4 carbon catabolite repression 4-like (S. cerevis	0.059058
ENSMUSG00000083107	Gm5303	predicted gene 5303 [Source:MGI Symbol;Acc:MGI:	0.059063
ENSMUSG00000040105	Ppapdc2	phosphatidic acid phosphatase type 2 domain cont	0.059131
ENSMUSG00000063047	Zfp780b	zinc finger protein 780B [Source:MGI Symbol;Acc:M	0.059161
ENSMUSG00000041426	Hibch	3-hydroxyisobutyryl-Coenzyme A hydrolase [Source	0.059193
ENSMUSG00000032834	Pwp2	PWP2 periodic tryptophan protein homolog (yeast)	0.059262
ENSMUSG00000038047	Haus6	HAUS augmin-like complex, subunit 6 [Source:MGI	0.059274
ENSMUSG00000027582	Zgpat	zinc finger, CCCH-type with G patch domain [Source	0.059289
ENSMUSG00000020571	Pdia6	protein disulfide isomerase associated 6 [Source:M	0.05933
ENSMUSG00000097213	Gm26745	predicted gene, 26745 [Source:MGI Symbol;Acc:MG	0.05939
ENSMUSG00000056952	Tatdn2	TatD DNase domain containing 2 [Source:MGI Symb	0.05944
ENSMUSG00000085828	Gm15612	predicted gene 15612 [Source:MGI Symbol;Acc:MG	0.059484
ENSMUSG00000042302	Ehbp1	EH domain binding protein 1 [Source:MGI Symbol;A	0.059486
ENSMUSG00000082852	Gm12021	predicted gene 12021 [Source:MGI Symbol;Acc:MG	0.059514
ENSMUSG00000039768	Dnajc11	DnaJ (Hsp40) homolog, subfamily C, member 11 [So	0.059691
ENSMUSG00000085218	BB218582	expressed sequence BB218582 [Source:MGI Symbo	0.059724
ENSMUSG00000050002	Idnk	idnK gluconokinase homolog (E. coli) [Source:MGI S	0.059769
ENSMUSG00000095470	Gm21863	predicted gene, 21863 [Source:MGI Symbol;Acc:MG	0.059878
ENSMUSG00000039683	Sdk1	sidekick homolog 1 (chicken) [Source:MGI Symbol;A	0.059886
ENSMUSG00000082209	Eif4a-ps4	eukaryotic translation initiation factor 4A, pseudog	0.059896
ENSMUSG00000086075	Gm15728	predicted gene 15728 [Source:MGI Symbol;Acc:MG	0.059898
ENSMUSG00000036112	Metap2	methionine aminopeptidase 2 [Source:MGI Symbol	0.06002
ENSMUSG00000085229	Gm11672	predicted gene 11672 [Source:MGI Symbol;Acc:MG	0.060042
ENSMUSG00000041390	Mdfic	MyoD family inhibitor domain containing [Source:M	0.060064
ENSMUSG00000021696	Elovl7	ELOVL family member 7, elongation of long chain fa	0.060153
ENSMUSG00000033174	Mgll	monoglyceride lipase [Source:MGI Symbol;Acc:MGI	0.060157
ENSMUSG00000038481	Cdk19	cyclin-dependent kinase 19 [Source:MGI Symbol;Ac	0.060204
ENSMUSG00000055782	Abcd2	ATP-binding cassette, sub-family D (ALD), member	0.060266
ENSMUSG00000030329	Pianp	PILR alpha associated neural protein [Source:MGI S	0.060279
ENSMUSG00000034912	Mdga2	MAM domain containing glycosylphosphatidylinosi	0.060331
ENSMUSG00000025199	Chuk	conserved helix-loop-helix ubiquitous kinase [Sourc	0.06044
ENSMUSG00000022817	Itgb5	integrin beta 5 [Source:MGI Symbol;Acc:MGI:96614	0.060449
ENSMUSG00000020918	Kat2a	K(lysine) acetyltransferase 2A [Source:MGI Symbol;	0.060457
ENSMUSG00000018707	Dync1h1	dynein cytoplasmic 1 heavy chain 1 [Source:MGI Sy	0.060474
ENSMUSG00000031231	Cox7b	cytochrome c oxidase subunit VIIb [Source:MGI Syr	0.060513
ENSMUSG00000024037	Wdr4	WD repeat domain 4 [Source:MGI Symbol;Acc:MGI	0.060563

ENSMUSG00000020219	Timm13	translocase of inner mitochondrial membrane 13 [S	0.060582
ENSMUSG00000025656	Arhgef9	CDC42 guanine nucleotide exchange factor (GEF) 9	0.060606
ENSMUSG00000034327	Kctd9	potassium channel tetramerisation domain contain	0.06065
ENSMUSG00000056880	Gadl1	glutamate decarboxylase-like 1 [Source:MGI Symbo	0.060662
ENSMUSG00000022518	4930562C15Rik	RIKEN cDNA 4930562C15 gene [Source:MGI Symbo	0.060675
ENSMUSG00000066800	Rnasel	ribonuclease L (2', 5'-oligoisoadenylate synthetase-	0.060691
ENSMUSG00000056493	Foxk1	forkhead box K1 [Source:MGI Symbol;Acc:MGI:134	0.060763
ENSMUSG00000035021	Baz1a	bromodomain adjacent to zinc finger domain 1A [S	0.060837
ENSMUSG00000094090	Gm8494	predicted gene 8494 [Source:MGI Symbol;Acc:MGI:	0.060863
ENSMUSG00000009097	Tbx1	T-box 1 [Source:MGI Symbol;Acc:MGI:98493]	0.060877
ENSMUSG00000062234	Gak	cyclin G associated kinase [Source:MGI Symbol;Acc	0.06091
ENSMUSG00000020974	Pole2	polymerase (DNA directed), epsilon 2 (p59 subunit)	0.060913
ENSMUSG00000018286	Psmb6	proteasome (prosome, macropain) subunit, beta ty	0.060938
ENSMUSG00000022978	Mis18a	MIS18 kinetochore protein homolog A (S. pombe) [	0.061022
ENSMUSG00000005442	Cic	capicua homolog (Drosophila) [Source:MGI Symbol	0.06106
ENSMUSG00000038045	Sult6b1	sulfotransferase family, cytosolic, 6B, member 1 [S	0.061158
ENSMUSG00000058812	0610039K10Rik	RIKEN cDNA 0610039K10 gene [Source:MGI Symbo	0.061158
ENSMUSG00000022602	Arc	activity regulated cytoskeletal-associated protein [S	0.061307
ENSMUSG00000030806	Stx1b	syntaxin 1B [Source:MGI Symbol;Acc:MGI:1930705	0.061353
ENSMUSG00000086625	Gm11831	predicted gene 11831 [Source:MGI Symbol;Acc:MG	0.061355
ENSMUSG00000024679	Ms4a6d	membrane-spanning 4-domains, subfamily A, mem	0.061376
ENSMUSG00000029822	Osbpl3	oxysterol binding protein-like 3 [Source:MGI Symbo	0.061391
ENSMUSG00000030689	Ino80e	INO80 complex subunit E [Source:MGI Symbol;Acc:	0.061446
ENSMUSG00000024208	Uqcc2	ubiquinol-cytochrome c reductase complex assemb	0.061476
ENSMUSG00000032456	Nmnat3	nicotinamide nucleotide adenylyltransferase 3 [Sou	0.061506
ENSMUSG00000003038	Hmgn2	high mobility group nucleosomal binding domain 2	0.061531
ENSMUSG00000040040	Ift88	intraflagellar transport 88 [Source:MGI Symbol;Acc	0.061648
ENSMUSG00000042699	Dhx9	DEAH (Asp-Glu-Ala-His) box polypeptide 9 [Source:	0.061725
ENSMUSG00000078502	Gm13212	predicted gene 13212 [Source:MGI Symbol;Acc:MG	0.061809
ENSMUSG00000083964	Gm14122	predicted gene 14122 [Source:MGI Symbol;Acc:MG	0.061829
ENSMUSG00000034928	Rnf44	ring finger protein 44 [Source:MGI Symbol;Acc:MGI	0.061854
ENSMUSG00000080365	Gm25776	predicted gene, 25776 [Source:MGI Symbol;Acc:M	0.061871
ENSMUSG00000087373	Gm15892	predicted gene 15892 [Source:MGI Symbol;Acc:MG	0.061898
ENSMUSG00000095087	Gm25328	predicted gene, 25328 [Source:MGI Symbol;Acc:M	0.061934
ENSMUSG00000031314	Taf1	TAF1 RNA polymerase II, TATA box binding protein	0.062012
ENSMUSG00000022240	Ctnnd2	catenin (cadherin associated protein), delta 2 [Sour	0.062019
ENSMUSG00000016503	Gtf3a	general transcription factor III A [Source:MGI Symb	0.062021
ENSMUSG00000048897	Zfp710	zinc finger protein 710 [Source:MGI Symbol;Acc:M	0.06203
ENSMUSG00000048832	Vps37c	vacuolar protein sorting 37C (yeast) [Source:MGI Sy	0.062115
ENSMUSG00000078235	Fam43b	family with sequence similarity 43, member B [Sour	0.062186
ENSMUSG00000038453	Srcin1	SRC kinase signaling inhibitor 1 [Source:MGI Symbo	0.062236
ENSMUSG00000097825	9630001P10Rik	RIKEN cDNA 9630001P10 gene [Source:MGI Symbo	0.062247
ENSMUSG00000036298	Slc2a13	solute carrier family 2 (facilitated glucose transport	0.062283
ENSMUSG00000066129	Kndc1	kinase non-catalytic C-lobe domain (KIND) containi	0.062296
ENSMUSG00000061353	Cxcl12	chemokine (C-X-C motif) ligand 12 [Source:MGI Syn	0.062315
ENSMUSG00000039456	Morc3	microrchidia 3 [Source:MGI Symbol;Acc:MGI:21368	0.062329
ENSMUSG00000074627	Mroh8	maestro heat-like repeat family member 8 [Source:	0.06237

ENSMUSG00000085250	Gm15234	predicted gene 15234 [Source:MGI Symbol;Acc:MG	0.062373
ENSMUSG00000004895	Prcc	papillary renal cell carcinoma (translocation-associa	0.062378
ENSMUSG00000049799	Lrrc19	leucine rich repeat containing 19 [Source:MGI Syml	0.062399
ENSMUSG00000012076	Brms1l	breast cancer metastasis-suppressor 1-like [Source:	0.062417
ENSMUSG00000030654	Arl6ip1	ADP-ribosylation factor-like 6 interacting protein 1	0.062433
ENSMUSG00000001150	Mcm3ap	minichromosome maintenance deficient 3 (S. cerev	0.062438
ENSMUSG00000054312	Mrps21	mitochondrial ribosomal protein S21 [Source:MGI S	0.06244
ENSMUSG00000036944	Tmem71	transmembrane protein 71 [Source:MGI Symbol;Ac	0.062492
ENSMUSG00000051768	Xrcc1	X-ray repair complementing defective repair in Chi	0.062516
ENSMUSG00000056692	D17Wsu92e	DNA segment, Chr 17, Wayne State University 92, e	0.062519
ENSMUSG00000034714	Ttyh2	tweety homolog 2 (Drosophila) [Source:MGI Symbo	0.062552
ENSMUSG00000026315	Serpinb8	serine (or cysteine) peptidase inhibitor, clade B, me	0.062593
ENSMUSG00000054556	Gm4876	predicted gene 4876 [Source:MGI Symbol;Acc:MGI:	0.062608
ENSMUSG00000000538	Tom1l2	target of myb1-like 2 (chicken) [Source:MGI Symbo	0.062659
ENSMUSG00000026484	Rnf2	ring finger protein 2 [Source:MGI Symbol;Acc:MGI:	0.062663
ENSMUSG00000056290	Ms4a4b	membrane-spanning 4-domains, subfamily A, mem	0.062757
ENSMUSG00000098008	A930001A20Rik	RIKEN cDNA A930001A20 gene [Source:MGI Symbo	0.062761
ENSMUSG00000035637	Grhpr	glyoxylate reductase/hydroxypyruvate reductase [S	0.062787
ENSMUSG00000061589	Dot1l	DOT1-like, histone H3 methyltransferase (S. cerevis	0.062837
ENSMUSG00000027951	Adar	adenosine deaminase, RNA-specific [Source:MGI Sy	0.062855
ENSMUSG00000053062	Jam2	junction adhesion molecule 2 [Source:MGI Symbol;	0.062861
ENSMUSG00000020024	Ccdc41	coiled-coil domain containing 41 [Source:MGI Symb	0.062878
ENSMUSG00000020605	Hs1bp3	HCLS1 binding protein 3 [Source:MGI Symbol;Acc:M	0.062974
ENSMUSG00000043051	Disc1	disrupted in schizophrenia 1 [Source:MGI Symbol;A	0.062985
ENSMUSG00000022508	Bcl6	B cell leukemia/lymphoma 6 [Source:MGI Symbol;A	0.063102
ENSMUSG00000074178	Gm10638	predicted gene 10638 [Source:MGI Symbol;Acc:MG	0.063142
ENSMUSG00000028849	Map7d1	MAP7 domain containing 1 [Source:MGI Symbol;Ac	0.063145
ENSMUSG00000028677	Rnf220	ring finger protein 220 [Source:MGI Symbol;Acc:M	0.063198
ENSMUSG00000050967	Creg2	cellular repressor of E1A-stimulated genes 2 [Sourc	0.063222
ENSMUSG00000029681	Bcl7b	B cell CLL/lymphoma 7B [Source:MGI Symbol;Acc:M	0.063249
ENSMUSG00000057615	Ldoc1	leucine zipper, down-regulated in cancer 1 [Source:	0.06325
ENSMUSG00000027905	Ddx20	DEAD (Asp-Glu-Ala-Asp) box polypeptide 20 [Sourc	0.063277
ENSMUSG00000022964	Tmem50b	transmembrane protein 50B [Source:MGI Symbol;A	0.063286
ENSMUSG00000041566	Tssk1	testis-specific serine kinase 1 [Source:MGI Symbol;	0.063346
ENSMUSG00000029007	Agtrap	angiotensin II, type I receptor-associated protein [S	0.063356
ENSMUSG00000078117	Gm16485	predicted gene 16485 [Source:MGI Symbol;Acc:MG	0.063363
ENSMUSG00000091089	A930018M24Rik	RIKEN cDNA A930018M24 gene [Source:MGI Symb	0.063367
ENSMUSG00000001157	Gmcl1	germ cell-less homolog 1 (Drosophila) [Source:MGI	0.063389
ENSMUSG00000013419	Zfp651	zinc finger protein 651 [Source:MGI Symbol;Acc:M	0.063439
ENSMUSG00000071984	Fndc1	fibronectin type III domain containing 1 [Source:M	0.063466
ENSMUSG00000052955	Cpvl	carboxypeptidase, vitellogenic-like [Source:MGI Sy	0.063594
ENSMUSG00000075010	AW112010	expressed sequence AW112010 [Source:MGI Symb	0.063686
ENSMUSG00000046380	Jrk	jerky [Source:MGI Symbol;Acc:MGI:106214]	0.0637
ENSMUSG00000070687	Htr1d	5-hydroxytryptamine (serotonin) receptor 1D [Sour	0.063732
ENSMUSG00000040415	Dtx3	deltex 3 homolog (Drosophila) [Source:MGI Symbo	0.063745
ENSMUSG00000021624	Cd180	CD180 antigen [Source:MGI Symbol;Acc:MGI:1194	0.063772
ENSMUSG00000020882	Cacnb1	calcium channel, voltage-dependent, beta 1 subuni	0.063816

ENSMUSG00000097385	Gm26814	predicted gene, 26814 [Source:MGI Symbol;Acc:MG	0.063848
ENSMUSG00000049470	Aff4	AF4/FMR2 family, member 4 [Source:MGI Symbol;A	0.063849
ENSMUSG00000040712	Camta2	calmodulin binding transcription activator 2 [Source	0.063931
ENSMUSG00000032913	Lrig2	leucine-rich repeats and immunoglobulin-like doma	0.063963
ENSMUSG00000027104	Atf2	activating transcription factor 2 [Source:MGI Symbo	0.064003
ENSMUSG00000021940	Ptpn20	protein tyrosine phosphatase, non-receptor type 2(	0.064014
ENSMUSG00000058163	Gm5431	predicted gene 5431 [Source:MGI Symbol;Acc:MGI:	0.064081
ENSMUSG00000088108	Snora47	small nucleolar RNA, H/ACA box 47 [Source:MGI Sy	0.064103
ENSMUSG00000031872	Bean1	brain expressed, associated with Nedd4, 1 [Source:	0.064118
ENSMUSG00000086164	Gm13029	predicted gene 13029 [Source:MGI Symbol;Acc:MG	0.064124
ENSMUSG00000015341	Golga7	golgi autoantigen, golgin subfamily a, 7 [Source:MG	0.064125
ENSMUSG00000062098	Btbd3	BTB (POZ) domain containing 3 [Source:MGI Symbo	0.064143
ENSMUSG00000023795	Pisd-ps2	phosphatidylserine decarboxylase, pseudogene 2 [S	0.064161
ENSMUSG00000047730	Fcgbp	Fc fragment of IgG binding protein [Source:MGI Syr	0.064161
ENSMUSG00000007880	Arid1a	AT rich interactive domain 1A (SWI-like) [Source:M(	0.064174
ENSMUSG00000052934	Fbxo31	F-box protein 31 [Source:MGI Symbol;Acc:MGI:135	0.064217
ENSMUSG00000022091	Sorbs3	sorbin and SH3 domain containing 3 [Source:MGI S	0.064348
ENSMUSG00000072844	G530011O06Rik	RIKEN cDNA G530011O06 gene [Source:MGI Symbo	0.064358
ENSMUSG00000074661	Pde4d	phosphodiesterase 4D, cAMP specific [Source:MGI	0.064395
ENSMUSG00000028430	Nol6	nucleolar protein family 6 (RNA-associated) [Source	0.064448
ENSMUSG00000045268	Zfp691	zinc finger protein 691 [Source:MGI Symbol;Acc:MG	0.064483
ENSMUSG00000074467	Gm10702	predicted gene 10702 [Source:MGI Symbol;Acc:MG	0.064489
ENSMUSG00000024962	Vegfb	vascular endothelial growth factor B [Source:MGI S	0.064499
ENSMUSG00000021635	Rad17	RAD17 homolog (S. pombe) [Source:MGI Symbol;A(	0.064519
ENSMUSG00000033379	Atp6v0b	ATPase, H+ transporting, lysosomal V0 subunit B [S	0.064575
ENSMUSG00000085824	2410114N07Rik	RIKEN cDNA 2410114N07 gene [Source:MGI Symbo	0.064626
ENSMUSG00000019278	Dpep1	dipeptidase 1 (renal) [Source:MGI Symbol;Acc:MGI	0.064643
ENSMUSG00000020232	Hmg20b	high mobility group 20B [Source:MGI Symbol;Acc:M	0.064669
ENSMUSG00000039678	Tbc1d13	TBC1 domain family, member 13 [Source:MGI Symb	0.06467
ENSMUSG00000031232	Magt1	magnesium transporter 1 [Source:MGI Symbol;Acc:	0.064718
ENSMUSG00000009681	Bcr	breakpoint cluster region [Source:MGI Symbol;Acc:	0.064729
ENSMUSG00000029672	Fam3c	family with sequence similarity 3, member C [Sourc	0.064736
ENSMUSG00000041164	Zmiz2	zinc finger, MIZ-type containing 2 [Source:MGI Sym	0.064782
ENSMUSG00000025583	Rptor	regulatory associated protein of MTOR, complex 1	0.064839
ENSMUSG00000021665	Hexb	hexosaminidase B [Source:MGI Symbol;Acc:MGI:96	0.064892
ENSMUSG00000021379	Id4	inhibitor of DNA binding 4 [Source:MGI Symbol;Acc	0.064897
ENSMUSG00000023467	Tulp2	tubby-like protein 2 [Source:MGI Symbol;Acc:MGI:1	0.064913
ENSMUSG00000021938	Pspc1	paraspeckle protein 1 [Source:MGI Symbol;Acc:MG	0.064946
ENSMUSG00000010045	Tmem115	transmembrane protein 115 [Source:MGI Symbol;A	0.064958
ENSMUSG00000063364	3300002I08Rik	RIKEN cDNA 3300002I08 gene [Source:MGI Symbol	0.06496
ENSMUSG00000026650	Meig1	meiosis expressed gene 1 [Source:MGI Symbol;Acc:	0.064996
ENSMUSG00000034762	Glis1	GLIS family zinc finger 1 [Source:MGI Symbol;Acc:M	0.065013
ENSMUSG00000039838	Slc45a1	solute carrier family 45, member 1 [Source:MGI Syr	0.065066
ENSMUSG00000059921	Unc5c	unc-5 homolog C (C. elegans) [Source:MGI Symbol;A	0.065118
ENSMUSG00000022983	Scaf4	SR-related CTD-associated factor 4 [Source:MGI Syr	0.065139
ENSMUSG00000031214	Ophn1	oligophrenin 1 [Source:MGI Symbol;Acc:MGI:21510	0.065154
ENSMUSG00000061654	Spry3	sprouty homolog 3 (Drosophila) [Source:MGI Symb	0.06517

ENSMUSG00000072964	Bhlhb9	basic helix-loop-helix domain containing, class B9 [S	0.065189
ENSMUSG00000029020	Mfn2	mitofusin 2 [Source:MGI Symbol;Acc:MGI:2442230]	0.065205
ENSMUSG00000031134	Rbmx	RNA binding motif protein, X chromosome [Source:	0.065223
ENSMUSG00000081264	Gm14021	predicted gene 14021 [Source:MGI Symbol;Acc:MG	0.065312
ENSMUSG00000017713	Tha1	threonine aldolase 1 [Source:MGI Symbol;Acc:MGI:	0.065322
ENSMUSG00000026821	Ralgds	ral guanine nucleotide dissociation stimulator [Sour	0.065326
ENSMUSG00000022292	Rrm2b	ribonucleotide reductase M2 B (TP53 inducible) [So	0.065464
ENSMUSG00000023022	Lima1	LIM domain and actin binding 1 [Source:MGI Symbo	0.065477
ENSMUSG00000030256	Bhlhe41	basic helix-loop-helix family, member e41 [Source:M	0.065493
ENSMUSG00000049832	Gm9840	predicted gene 9840 [Source:MGI Symbol;Acc:MGI:	0.06558
ENSMUSG00000097725	4930593A02Rik	RIKEN cDNA 4930593A02 gene [Source:MGI Symbo	0.065603
ENSMUSG00000030322	Mbd4	methyl-CpG binding domain protein 4 [Source:MGI	0.065642
ENSMUSG00000055319	Sec23ip	Sec23 interacting protein [Source:MGI Symbol;Acc:	0.065684
ENSMUSG00000028277	Ube2j1	ubiquitin-conjugating enzyme E2J 1 [Source:MGI Sy	0.065769
ENSMUSG00000022556	Hsf1	heat shock factor 1 [Source:MGI Symbol;Acc:MGI:9	0.065802
ENSMUSG00000038212	Hiatl1	hippocampus abundant transcript-like 1 [Source:M	0.065851
ENSMUSG00000023484	Prph	peripherin [Source:MGI Symbol;Acc:MGI:97774]	0.065854
ENSMUSG00000020076	Ddx50	DEAD (Asp-Glu-Ala-Asp) box polypeptide 50 [Source	0.065886
ENSMUSG00000025586	Cpeb1	cytoplasmic polyadenylation element binding prote	0.065904
ENSMUSG00000029108	Pcdh7	protocadherin 7 [Source:MGI Symbol;Acc:MGI:186	0.065982
ENSMUSG00000022965	Ifngr2	interferon gamma receptor 2 [Source:MGI Symbol;	0.065986
ENSMUSG00000086943	4732414G09Rik	RIKEN cDNA 4732414G09 gene [Source:MGI Symbo	0.066092
ENSMUSG00000027416	Otor	otoraplin [Source:MGI Symbol;Acc:MGI:1888678]	0.066108
ENSMUSG00000022177	Haus4	HAUS augmin-like complex, subunit 4 [Source:MGI	0.066172
ENSMUSG00000031532	Tmem66	transmembrane protein 66 [Source:MGI Symbol;Ac	0.06623
ENSMUSG00000036356	Csgalnact1	chondroitin sulfate N-acetylgalactosaminyltransfera	0.066255
ENSMUSG00000089855	Gm15662	predicted gene 15662 [Source:MGI Symbol;Acc:MG	0.066265
ENSMUSG00000032303	Chrna3	cholinergic receptor, nicotinic, alpha polypeptide 3	0.066287
ENSMUSG00000035171	1110059E24Rik	RIKEN cDNA 1110059E24 gene [Source:MGI Symbo	0.066332
ENSMUSG00000036966	Spryd3	SPRY domain containing 3 [Source:MGI Symbol;Acc	0.066396
ENSMUSG00000043421	Hilpda	hypoxia inducible lipid droplet associated [Source:N	0.06643
ENSMUSG00000004451	Ralb	v-ral simian leukemia viral oncogene homolog B (ra	0.066439
ENSMUSG00000029012	Orc5	origin recognition complex, subunit 5 [Source:MGI	0.066484
ENSMUSG00000073154	9330158H04Rik	RIKEN cDNA 9330158H04 gene [Source:MGI Symbo	0.066509
ENSMUSG00000022220	Adcy4	adenylate cyclase 4 [Source:MGI Symbol;Acc:MGI:9	0.066519
ENSMUSG00000033712	Ccar2	cell cycle activator and apoptosis regulator 2 [Sourc	0.06653
ENSMUSG00000032186	Tmod2	tropomodulin 2 [Source:MGI Symbol;Acc:MGI:1355	0.066579
ENSMUSG00000024180	Tmem8	transmembrane protein 8 (five membrane-spannin	0.06658
ENSMUSG00000090861	4930570N19Rik	RIKEN cDNA 4930570N19 gene [Source:MGI Symbo	0.066639
ENSMUSG00000019173	Rab5c	RAB5C, member RAS oncogene family [Source:MGI	0.066727
ENSMUSG00000068663	Clec16a	C-type lectin domain family 16, member A [Source:	0.066776
ENSMUSG00000026965	Anapc2	anaphase promoting complex subunit 2 [Source:M	0.066807
ENSMUSG00000056569	Mpz	myelin protein zero [Source:MGI Symbol;Acc:MGI:1	0.066919
ENSMUSG00000026005	Rpe	ribulose-5-phosphate-3-epimerase [Source:MGI Sy	0.066944
ENSMUSG00000052446	Zfp961	zinc finger protein 961 [Source:MGI Symbol;Acc:M	0.06695
ENSMUSG00000021279	Cdc42bpb	CDC42 binding protein kinase beta [Source:MGI Sy	0.066957
ENSMUSG00000097616	1110019D14Rik	RIKEN cDNA 1110019D14 gene [Source:MGI Symbo	0.06704

ENSMUSG00000035268	Pkig	protein kinase inhibitor, gamma [Source:MGI Symb	0.067087
ENSMUSG00000015468	Notch4	notch 4 [Source:MGI Symbol;Acc:MGI:107471]	0.067129
ENSMUSG00000020116	Pno1	partner of NOB1 homolog (S. cerevisiae) [Source:M	0.067152
ENSMUSG00000038564	Ift172	intraflagellar transport 172 [Source:MGI Symbol;Ac	0.067248
ENSMUSG00000038370	Pcp4l1	Purkinje cell protein 4-like 1 [Source:MGI Symbol;A	0.067259
ENSMUSG00000060791	Gmfg	glia maturation factor, gamma [Source:MGI Symbo	0.067274
ENSMUSG00000022403	St13	suppression of tumorigenicity 13 [Source:MGI Syml	0.067304
ENSMUSG00000021786	Oxsm	3-oxoacyl-ACP synthase, mitochondrial [Source:MG	0.06731
ENSMUSG00000051184	Zfp524	zinc finger protein 524 [Source:MGI Symbol;Acc:M(	0.067486
ENSMUSG00000097914	Gm26838	predicted gene, 26838 [Source:MGI Symbol;Acc:M(	0.067507
ENSMUSG00000049191	Rgag4	retrotransposon gag domain containing 4 [Source:N	0.067617
ENSMUSG00000003184	Irf3	interferon regulatory factor 3 [Source:MGI Symbol;	0.06771
ENSMUSG00000087331	1810021B22Rik	RIKEN cDNA 1810021B22 gene [Source:MGI Symbo	0.067719
ENSMUSG00000093628	Gm20616	predicted gene 20616 [Source:MGI Symbol;Acc:MG	0.067724
ENSMUSG00000048387	Osr1	odd-skipped related 1 (Drosophila) [Source:MGI Sy	0.067732
ENSMUSG00000048668	Rhno1	RAD9-HUS1-RAD1 interacting nuclear orphan 1 [So	0.067746
ENSMUSG00000052981	Ube2ql1	ubiquitin-conjugating enzyme E2Q family-like 1 [So	0.067747
ENSMUSG00000035413	Tmem98	transmembrane protein 98 [Source:MGI Symbol;Ac	0.067769
ENSMUSG00000047824	Pygo2	pygopus 2 [Source:MGI Symbol;Acc:MGI:1916161]	0.067809
ENSMUSG00000022070	Bora	bora, aurora kinase A activator [Source:MGI Symbo	0.067826
ENSMUSG00000090638	Gm17028	predicted gene 17028 [Source:MGI Symbol;Acc:MG	0.067841
ENSMUSG00000030055	Rab43	RAB43, member RAS oncogene family [Source:MGI	0.067954
ENSMUSG00000036578	Fxyd7	FXYD domain-containing ion transport regulator 7 [	0.067972
ENSMUSG00000079334	Nat6	N-acetyltransferase 6 [Source:MGI Symbol;Acc:MG	0.068013
ENSMUSG00000024940	Ltbp3	latent transforming growth factor beta binding pro	0.068112
ENSMUSG00000086407	Gm14123	predicted gene 14123 [Source:MGI Symbol;Acc:MG	0.068119
ENSMUSG00000084063	Gm5934	predicted gene 5934 [Source:MGI Symbol;Acc:MGI:	0.068226
ENSMUSG00000024610	Cd74	CD74 antigen (invariant polypeptide of major histo	0.068302
ENSMUSG00000026896	Ifih1	interferon induced with helicase C domain 1 [Sourc	0.068305
ENSMUSG00000045827	Serpinb9	serine (or cysteine) peptidase inhibitor, clade B, me	0.068307
ENSMUSG00000024120	Lrpprc	leucine-rich PPR-motif containing [Source:MGI Sym	0.068308
ENSMUSG00000072872	Rybp	RING1 and YY1 binding protein [Source:MGI Symbo	0.068313
ENSMUSG00000033569	Bai3	brain-specific angiogenesis inhibitor 3 [Source:MGI	0.068393
ENSMUSG00000091003	Gm7244	predicted gene 7244 [Source:MGI Symbol;Acc:MGI:	0.068405
ENSMUSG00000038976	Ppp1r9b	protein phosphatase 1, regulatory subunit 9B [Sour	0.068407
ENSMUSG00000096970	2210017G18Rik	RIKEN cDNA 2210017G18 gene [Source:MGI Symbo	0.068409
ENSMUSG00000002565	Scin	scinderin [Source:MGI Symbol;Acc:MGI:1306794]	0.068571
ENSMUSG00000088506	Gm24409	predicted gene, 24409 [Source:MGI Symbol;Acc:M(	0.068591
ENSMUSG00000037578	Pkd2l1	polycystic kidney disease 2-like 1 [Source:MGI Syml	0.068605
ENSMUSG00000005981	Trap1	TNF receptor-associated protein 1 [Source:MGI Syn	0.06864
ENSMUSG00000050097	Ces2b	carboxyesterase 2B [Source:MGI Symbol;Acc:MGI:2	0.068696
ENSMUSG00000033960	9430020K01Rik	RIKEN cDNA 9430020K01 gene [Source:MGI Symbo	0.068704
ENSMUSG00000026205	Slc23a3	solute carrier family 23 (nucleobase transporters),	0.068764
ENSMUSG00000027071	P2rx3	purinergic receptor P2X, ligand-gated ion channel,	0.068784
ENSMUSG00000042487	Leo1	Leo1, Paf1/RNA polymerase II complex component	0.068794
ENSMUSG00000042293	Gm5617	predicted gene 5617 [Source:MGI Symbol;Acc:MGI:	0.068825
ENSMUSG00000025468	Caly	calcyon neuron-specific vesicular protein [Source:M	0.068843

ENSMUSG00000032940	Rbm11	RNA binding motif protein 11 [Source:MGI Symbol;	0.068854
ENSMUSG00000029663	Gngt1	guanine nucleotide binding protein (G protein), gar	0.068941
ENSMUSG00000022270	Fam134b	family with sequence similarity 134, member B [Sou	0.069104
ENSMUSG00000027259	Adal	adenosine deaminase-like [Source:MGI Symbol;Acc	0.069134
ENSMUSG00000026674	Ddr2	discoidin domain receptor family, member 2 [Sourc	0.0692
ENSMUSG00000024660	Incenp	inner centromere protein [Source:MGI Symbol;Acc:	0.06922
ENSMUSG00000063681	Crb1	crumbs homolog 1 (Drosophila) [Source:MGI Symb	0.06922
ENSMUSG00000055313	Pgbd1	piggyBac transposable element derived 1 [Source:N	0.069221
ENSMUSG00000028672	Hmgcl	3-hydroxy-3-methylglutaryl-Coenzyme A lyase [Sou	0.069237
ENSMUSG00000068240	Gm11808	predicted gene 11808 [Source:MGI Symbol;Acc:MG	0.069264
ENSMUSG00000081739	Mdm4-ps	transformed mouse 3T3 cell double minute 4, pseu	0.069296
ENSMUSG00000041078	Grid1	glutamate receptor, ionotropic, delta 1 [Source:MG	0.069364
ENSMUSG00000036106	Prr5	proline rich 5 (renal) [Source:MGI Symbol;Acc:MGI:	0.069383
ENSMUSG00000060802	B2m	beta-2 microglobulin [Source:MGI Symbol;Acc:MGI	0.069469
ENSMUSG00000018398	8-Sep	septin 8 [Source:MGI Symbol;Acc:MGI:894310]	0.069512
ENSMUSG00000029291	Rufy3	RUN and FYVE domain containing 3 [Source:MGI Sy	0.069531
ENSMUSG00000002496	Tsc2	tuberous sclerosis 2 [Source:MGI Symbol;Acc:MGI:1	0.069603
ENSMUSG00000070319	Eif3g	eukaryotic translation initiation factor 3, subunit G	0.06967
ENSMUSG00000058558	Rpl5	ribosomal protein L5 [Source:MGI Symbol;Acc:MGI	0.069719
ENSMUSG00000047904	Sstr2	somatostatin receptor 2 [Source:MGI Symbol;Acc:N	0.069751
ENSMUSG00000030062	Rpn1	ribophorin I [Source:MGI Symbol;Acc:MGI:98084]	0.069773
ENSMUSG00000047797	Gjb1	gap junction protein, beta 1 [Source:MGI Symbol;A	0.069831
ENSMUSG00000035885	Cox8a	cytochrome c oxidase subunit VIIIa [Source:MGI Syr	0.069858
ENSMUSG00000029005	Draxin	dorsal inhibitory axon guidance protein [Source:MG	0.069871
ENSMUSG00000019362	D8Ertd738e	DNA segment, Chr 8, ERATO Doi 738, expressed [So	0.069876
ENSMUSG00000024887	Asah2	N-acylsphingosine amidohydrolase 2 [Source:MGI S	0.069877
ENSMUSG00000039706	Ldb2	LIM domain binding 2 [Source:MGI Symbol;Acc:MG	0.069883
ENSMUSG00000001415	Smg5	Smg-5 homolog, nonsense mediated mRNA decay f	0.069889
ENSMUSG00000000374	Trappc10	trafficking protein particle complex 10 [Source:MGI	0.069891
ENSMUSG00000042901	Aida	axin interactor, dorsalization associated [Source:M	0.069929
ENSMUSG00000032386	Trip4	thyroid hormone receptor interactor 4 [Source:MG	0.06994
ENSMUSG00000025354	Dnajc14	DnaJ (Hsp40) homolog, subfamily C, member 14 [So	0.070032
ENSMUSG00000044857	Lemd2	LEM domain containing 2 [Source:MGI Symbol;Acc:	0.070106
ENSMUSG00000002768	Mea1	male enhanced antigen 1 [Source:MGI Symbol;Acc:	0.070112
ENSMUSG00000025050	Pcgf6	polycomb group ring finger 6 [Source:MGI Symbol;A	0.070133
ENSMUSG00000085204	Gm15327	predicted gene 15327 [Source:MGI Symbol;Acc:MG	0.070162
ENSMUSG00000037993	Dhx38	DEAH (Asp-Glu-Ala-His) box polypeptide 38 [Source	0.07034
ENSMUSG00000058076	Sdhc	succinate dehydrogenase complex, subunit C, integ	0.070396
ENSMUSG00000042677	Zc3h12a	zinc finger CCCH type containing 12A [Source:MGI S	0.070423
ENSMUSG00000021868	Ppif	peptidylprolyl isomerase F (cyclophilin F) [Source:M	0.070429
ENSMUSG00000020897	Aurkb	aurora kinase B [Source:MGI Symbol;Acc:MGI:1071	0.070457
ENSMUSG00000028645	Slc2a1	solute carrier family 2 (facilitated glucose transport	0.070461
ENSMUSG00000081798	Gm8152	predicted gene 8152 [Source:MGI Symbol;Acc:MGI:	0.070489
ENSMUSG00000097761	4930534D22Rik	RIKEN cDNA 4930534D22 gene [Source:MGI Symbo	0.070495
ENSMUSG00000031748	Gnao1	guanine nucleotide binding protein, alpha O [Sourc	0.07051
ENSMUSG00000027712	Anxa5	annexin A5 [Source:MGI Symbol;Acc:MGI:106008]	0.070531
ENSMUSG00000090399	Nav1	neuron navigator 1 [Source:MGI Symbol;Acc:MGI:2	0.070538

ENSMUSG00000038279	Nop2	NOP2 nucleolar protein [Source:MGI Symbol;Acc:M	0.070555
ENSMUSG00000028214	Gem	GTP binding protein (gene overexpressed in skeleta	0.070565
ENSMUSG00000023961	Enpp4	ectonucleotide pyrophosphatase/phosphodiesteras	0.070566
ENSMUSG00000025157	Zdhhc16	zinc finger, DHHC domain containing 16 [Source:M(	0.070572
ENSMUSG00000048216	Gpr85	G protein-coupled receptor 85 [Source:MGI Symbo	0.070629
ENSMUSG00000062661	Ncs1	neuronal calcium sensor 1 [Source:MGI Symbol;Acc	0.070635
ENSMUSG00000015880	Ncapg	non-SMC condensin I complex, subunit G [Source:M	0.070652
ENSMUSG00000030693	Klk10	kallikrein related-peptidase 10 [Source:MGI Symbo	0.070691
ENSMUSG00000021071	Trim9	tripartite motif-containing 9 [Source:MGI Symbol;A	0.070715
ENSMUSG00000028243	Ubxn2b	UBX domain protein 2B [Source:MGI Symbol;Acc:M	0.070747
ENSMUSG00000046879	Irgm1	immunity-related GTPase family M member 1 [Sou	0.070919
ENSMUSG00000002210	Smg9	smg-9 homolog, nonsense mediated mRNA decay f	0.07092
ENSMUSG00000057421	Las1l	LAS1-like (S. cerevisiae) [Source:MGI Symbol;Acc:M	0.070938
ENSMUSG00000053664	Uck2	uridine-cytidine kinase 2 [Source:MGI Symbol;Acc:M	0.071086
ENSMUSG00000072915	Gm12258	predicted gene 12258 [Source:MGI Symbol;Acc:MG	0.071192
ENSMUSG00000074623	Gm826	predicted gene 826 [Source:MGI Symbol;Acc:MGI:2	0.0712
ENSMUSG00000078840	Gm10999	predicted gene 10999 [Source:MGI Symbol;Acc:MG	0.071238
ENSMUSG00000014859	E2f4	E2F transcription factor 4 [Source:MGI Symbol;Acc:	0.071242
ENSMUSG00000073412	Lst1	leukocyte specific transcript 1 [Source:MGI Symbol	0.071312
ENSMUSG00000009378	Slc16a12	solute carrier family 16 (monocarboxylic acid trans	0.071337
ENSMUSG00000060204	Gm5868	predicted gene 5868 [Source:MGI Symbol;Acc:MGI:	0.071349
ENSMUSG00000038244	Mical2	microtubule associated monooxygenase, calponin a	0.071356
ENSMUSG00000025597	Klhl4	kelch-like 4 [Source:MGI Symbol;Acc:MGI:2442829]	0.071379
ENSMUSG00000046345	Smco1	single-pass membrane protein with coiled-coil dom	0.071392
ENSMUSG00000000915	Hip1r	huntingtin interacting protein 1 related [Source:M(	0.071401
ENSMUSG00000037035	Inhbb	inhibin beta-B [Source:MGI Symbol;Acc:MGI:96571	0.071401
ENSMUSG00000039270	Megf9	multiple EGF-like-domains 9 [Source:MGI Symbol;A	0.071423
ENSMUSG00000046711	Hmga1	high mobility group AT-hook 1 [Source:MGI Symbol	0.071474
ENSMUSG00000097324	Mir143hg	Mir143 and Mir145 host gene (non-coding RNA) [So	0.071531
ENSMUSG00000040236	Trappc5	trafficking protein particle complex 5 [Source:MGI S	0.071611
ENSMUSG00000026134	Prim2	DNA primase, p58 subunit [Source:MGI Symbol;Acc	0.071739
ENSMUSG00000030789	Itgax	integrin alpha X [Source:MGI Symbol;Acc:MGI:9660	0.071816
ENSMUSG00000070287	Slc35g2	solute carrier family 35, member G2 [Source:MGI S	0.071853
ENSMUSG00000029119	Man2b2	mannosidase 2, alpha B2 [Source:MGI Symbol;Acc:I	0.071874
ENSMUSG00000027272	Ubr1	ubiquitin protein ligase E3 component n-recognin 1	0.071879
ENSMUSG00000037843	Vstm2l	V-set and transmembrane domain containing 2-like	0.071897
ENSMUSG00000097731	2700016F22Rik	RIKEN cDNA 2700016F22 gene [Source:MGI Symbo	0.071915
ENSMUSG00000073792	Alg6	asparagine-linked glycosylation 6 (alpha-1,3,-glucos	0.071948
ENSMUSG00000087131	Gm16152	predicted gene 16152 [Source:MGI Symbol;Acc:MG	0.07199
ENSMUSG00000067356	B430203G13Rik	RIKEN cDNA B430203G13 gene [Source:MGI Symbo	0.072024
ENSMUSG00000044017	Gpr133	G protein-coupled receptor 133 [Source:MGI Symb	0.07211
ENSMUSG00000044018	Mrpl50	mitochondrial ribosomal protein L50 [Source:MGI S	0.072134
ENSMUSG00000037649	H2-DMa	histocompatibility 2, class II, locus DMa [Source:MG	0.072136
ENSMUSG00000022160	Mettl3	methyltransferase like 3 [Source:MGI Symbol;Acc:M	0.072148
ENSMUSG00000084416	Rpl10a-ps1	ribosomal protein L10A, pseudogene 1 [Source:MG	0.072159
ENSMUSG00000021474	Sfxn1	sideroflexin 1 [Source:MGI Symbol;Acc:MGI:213767	0.072203
ENSMUSG00000097309	Gm17249	predicted gene, 17249 [Source:MGI Symbol;Acc:M(	0.072227

ENSMUSG00000068696	Gpr88	G-protein coupled receptor 88 [Source:MGI Symbo	0.072263
ENSMUSG00000050640	Tmem150c	transmembrane protein 150C [Source:MGI Symbol;	0.072277
ENSMUSG00000028226	Mmp16	matrix metallopeptidase 16 [Source:MGI Symbol;A	0.072371
ENSMUSG00000003810	Mast2	microtubule associated serine/threonine kinase 2 [	0.072378
ENSMUSG00000041670	Rims1	regulating synaptic membrane exocytosis 1 [Source	0.07242
ENSMUSG00000032103	Pus3	pseudouridine synthase 3 [Source:MGI Symbol;Acc	0.072445
ENSMUSG00000062345	Serpinb2	serine (or cysteine) peptidase inhibitor, clade B, me	0.072488
ENSMUSG00000055818	A230083G16Rik	RIKEN cDNA A230083G16 gene [Source:MGI Symbo	0.07251
ENSMUSG00000086825	Gm15675	predicted gene 15675 [Source:MGI Symbol;Acc:MG	0.07257
ENSMUSG00000020548	1700086D15Rik	RIKEN cDNA 1700086D15 gene [Source:MGI Symbo	0.072639
ENSMUSG00000025575	Cant1	calcium activated nucleotidase 1 [Source:MGI Symb	0.072657
ENSMUSG00000029439	Sfswap	splicing factor, suppressor of white-apricot homolo	0.072666
ENSMUSG00000035042	Ccl5	chemokine (C-C motif) ligand 5 [Source:MGI Symbo	0.072674
ENSMUSG00000041837	Pdcd7	programmed cell death 7 [Source:MGI Symbol;Acc:	0.072691
ENSMUSG00000068735	Trp53i11	transformation related protein 53 inducible protein	0.072707
ENSMUSG00000083728	Gm13854	predicted gene 13854 [Source:MGI Symbol;Acc:MG	0.072713
ENSMUSG00000052759	Gpr25	G protein-coupled receptor 25 [Source:MGI Symbo	0.072746
ENSMUSG00000021395	Spin1	spindlin 1 [Source:MGI Symbol;Acc:MGI:109242]	0.072748
ENSMUSG00000037253	Mex3c	mex3 homolog C (C. elegans) [Source:MGI Symbol;	0.072784
ENSMUSG00000002459	Rgs20	regulator of G-protein signaling 20 [Source:MGI Syr	0.072801
ENSMUSG00000030470	Csrp3	cysteine and glycine-rich protein 3 [Source:MGI Syr	0.072862
ENSMUSG00000083877	Gm14740	predicted gene 14740 [Source:MGI Symbol;Acc:MG	0.072904
ENSMUSG00000026568	Mpc2	mitochondrial pyruvate carrier 2 [Source:MGI Symb	0.072908
ENSMUSG00000076613	Ighg2b	immunoglobulin heavy constant gamma 2B [Source	0.072937
ENSMUSG00000029767	Calu	calumenin [Source:MGI Symbol;Acc:MGI:1097158]	0.07295
ENSMUSG00000023852	Chd1	chromodomain helicase DNA binding protein 1 [Sou	0.07297
ENSMUSG00000022420	Dnal4	dynein, axonemal, light chain 4 [Source:MGI Symbo	0.072976
ENSMUSG00000000197	Nalcn	sodium leak channel, non-selective [Source:MGI Sy	0.073018
ENSMUSG00000045284	Dcaf12l1	DDB1 and CUL4 associated factor 12-like 1 [Source:	0.073055
ENSMUSG00000080950	Gm7278	predicted gene 7278 [Source:MGI Symbol;Acc:MGI:	0.073122
ENSMUSG00000034755	Pcdh11x	protocadherin 11 X-linked [Source:MGI Symbol;Acc	0.073163
ENSMUSG00000087286	Gm9013	predicted gene 9013 [Source:MGI Symbol;Acc:MGI:	0.07318
ENSMUSG00000054951	9130008F23Rik	RIKEN cDNA 9130008F23 gene [Source:MGI Symbo	0.073186
ENSMUSG00000060187	Lrrc10	leucine rich repeat containing 10 [Source:MGI Syml	0.073282
ENSMUSG00000031822	Gse1	genetic suppressor element 1 [Source:MGI Symbol;	0.073318
ENSMUSG00000021244	Ylpm1	YLP motif containing 1 [Source:MGI Symbol;Acc:MG	0.07332
ENSMUSG00000022864	D16Ertd472e	DNA segment, Chr 16, ERATO Doi 472, expressed [S	0.073366
ENSMUSG00000042523	Dnal1	dynein, axonemal, light chain 1 [Source:MGI Symbo	0.073398
ENSMUSG00000030207	8430419L09Rik	RIKEN cDNA 8430419L09 gene [Source:MGI Symbo	0.073405
ENSMUSG00000035960	Apex1	apurinic/apyrimidinic endonuclease 1 [Source:MGI	0.073409
ENSMUSG00000024242	Map4k3	mitogen-activated protein kinase kinase kinase kina	0.073436
ENSMUSG00000029246	Ppat	phosphoribosyl pyrophosphate amidotransferase [S	0.073446
ENSMUSG00000002319	Ipo4	importin 4 [Source:MGI Symbol;Acc:MGI:1923001]	0.073473
ENSMUSG00000035202	Lars2	leucyl-tRNA synthetase, mitochondrial [Source:MG	0.0735
ENSMUSG00000024817	Uhrf2	ubiquitin-like, containing PHD and RING finger dom	0.073578
ENSMUSG00000036834	Plch1	phospholipase C, eta 1 [Source:MGI Symbol;Acc:MG	0.07359
ENSMUSG00000050910	Cdr2l	cerebellar degeneration-related protein 2-like [Sou	0.073714

ENSMUSG00000022191	Drosha	drosha, ribonuclease type III [Source:MGI Symbol;A	0.07375
ENSMUSG00000007987	Rabl5	RAB, member of RAS oncogene family-like 5 [Sourc	0.073761
ENSMUSG00000039648	Ccbl1	cysteine conjugate-beta lyase 1 [Source:MGI Symbo	0.073769
ENSMUSG00000044062	0610009B14Rik	RIKEN cDNA 0610009B14 gene [Source:MGI Symbo	0.073769
ENSMUSG00000032235	Narg2	NMDA receptor-regulated gene 2 [Source:MGI Sym	0.073844
ENSMUSG00000007815	Rhoa	ras homolog gene family, member A [Source:MGI S	0.073858
ENSMUSG00000003518	Dusp3	dual specificity phosphatase 3 (vaccinia virus phosp	0.073885
ENSMUSG00000090663	Srcap	Snf2-related CREBBP activator protein [Source:MGI	0.073965
ENSMUSG00000041638	Gcn1l1	GCN1 general control of amino-acid synthesis 1-like	0.074019
ENSMUSG00000053198	Prx	periaxin [Source:MGI Symbol;Acc:MGI:108176]	0.074039
ENSMUSG00000079808	AC168977.1		0.074069
ENSMUSG00000008496	Pou2f2	POU domain, class 2, transcription factor 2 [Source	0.074091
ENSMUSG00000024079	Eif2ak2	eukaryotic translation initiation factor 2-alpha kinas	0.074094
ENSMUSG00000020121	Srgap1	SLIT-ROBO Rho GTPase activating protein 1 [Source	0.074102
ENSMUSG00000089253	Gm25716	predicted gene, 25716 [Source:MGI Symbol;Acc:MG	0.074133
ENSMUSG00000044469	Tnfaip8l1	tumor necrosis factor, alpha-induced protein 8-like	0.074217
ENSMUSG00000022525	Hrasls	HRAS-like suppressor [Source:MGI Symbol;Acc:MGI	0.074228
ENSMUSG00000030120	Mlf2	myeloid leukemia factor 2 [Source:MGI Symbol;Acc	0.074232
ENSMUSG00000066944	4921513D11Rik	RIKEN cDNA 4921513D11 gene [Source:MGI Symbo	0.074341
ENSMUSG00000003226	Ranbp2	RAN binding protein 2 [Source:MGI Symbol;Acc:MG	0.074396
ENSMUSG00000019359	Gdpd2	glycerophosphodiester phosphodiesterase domain	0.074485
ENSMUSG00000043190	Rfesd	Rieske (Fe-S) domain containing [Source:MGI Symb	0.074513
ENSMUSG00000049097	Ankrd34a	ankyrin repeat domain 34A [Source:MGI Symbol;Ac	0.074528
ENSMUSG00000049723	Mmp12	matrix metallopeptidase 12 [Source:MGI Symbol;Ac	0.074531
ENSMUSG00000071660	Ttc9c	tetratricopeptide repeat domain 9C [Source:MGI Sy	0.074558
ENSMUSG00000066553	Gm6969	predicted pseudogene 6969 [Source:MGI Symbol;A	0.074562
ENSMUSG00000000301	Pemt	phosphatidylethanolamine N-methyltransferase [So	0.074578
ENSMUSG00000027852	Nras	neuroblastoma ras oncogene [Source:MGI Symbol;	0.074597
ENSMUSG00000064030	Wibg	within bgcn homolog (Drosophila) [Source:MGI Sym	0.074612
ENSMUSG00000020140	Lgr5	leucine rich repeat containing G protein coupled re	0.074677
ENSMUSG00000027433	Xrn2	5'-3' exoribonuclease 2 [Source:MGI Symbol;Acc:M	0.074823
ENSMUSG00000063236	1110038F14Rik	RIKEN cDNA 1110038F14 gene [Source:MGI Symbo	0.074851
ENSMUSG00000072962	Gm16401	predicted gene 16401 [Source:MGI Symbol;Acc:MG	0.07496
ENSMUSG00000081086	Gm9506	predicted gene 9506 [Source:MGI Symbol;Acc:MGI	0.074979
ENSMUSG00000020814	Mxra7	matrix-remodelling associated 7 [Source:MGI Symb	0.075056
ENSMUSG00000031993	Snx19	sorting nexin 19 [Source:MGI Symbol;Acc:MGI:192	0.075122
ENSMUSG00000024392	Bag6	BCL2-associated athanogene 6 [Source:MGI Symbo	0.075213
ENSMUSG00000044433	Camsap3	calmodulin regulated spectrin-associated protein fa	0.075216
ENSMUSG00000021180	Rps6ka5	ribosomal protein S6 kinase, polypeptide 5 [Source	0.075235
ENSMUSG00000069227	Gprin1	G protein-regulated inducer of neurite outgrowth 1	0.075235
ENSMUSG00000042286	Stab1	stabilin 1 [Source:MGI Symbol;Acc:MGI:2178742]	0.075284
ENSMUSG00000022008	Gpalpp1	GPALPP motifs containing 1 [Source:MGI Symbol;Ac	0.075442
ENSMUSG00000089640	Gm6397	predicted gene 6397 [Source:MGI Symbol;Acc:MGI	0.075453
ENSMUSG00000092364	Gm20476	predicted gene 20476 [Source:MGI Symbol;Acc:MG	0.075485
ENSMUSG00000045730	Adrb2	adrenergic receptor, beta 2 [Source:MGI Symbol;Ac	0.075495
ENSMUSG00000021994	Wnt5a	wingless-related MMTV integration site 5A [Source	0.075557
ENSMUSG00000092781	Mir3057	microRNA 3057 [Source:MGI Symbol;Acc:MGI:4834	0.075577

ENSMUSG00000018931	Gm16515	predicted gene, Gm16515 [Source:MGI Symbol;Acc	0.075612
ENSMUSG00000068196	Col8a1	collagen, type VIII, alpha 1 [Source:MGI Symbol;Acc	0.075618
ENSMUSG00000026289	Atg16l1	autophagy related 16-like 1 (S. cerevisiae) [Source:I	0.075624
ENSMUSG00000038034	Igsf8	immunoglobulin superfamily, member 8 [Source:M	0.07571
ENSMUSG00000008683	Rps15a	ribosomal protein S15A [Source:MGI Symbol;Acc:M	0.07573
ENSMUSG00000011877	Git1	G protein-coupled receptor kinase-interactor 1 [Sou	0.075771
ENSMUSG00000002997	Prkar2b	protein kinase, cAMP dependent regulatory, type II	0.075781
ENSMUSG00000081585	Gm12926	predicted gene 12926 [Source:MGI Symbol;Acc:MG	0.075792
ENSMUSG00000034789	Rab24	RAB24, member RAS oncogene family [Source:MGI	0.075843
ENSMUSG00000024773	Atg2a	autophagy related 2A [Source:MGI Symbol;Acc:MG	0.075862
ENSMUSG00000062753	AI413582	expressed sequence AI413582 [Source:MGI Symbol	0.075868
ENSMUSG00000055745	Ldoc1l	leucine zipper, down-regulated in cancer 1-like [Sou	0.07587
ENSMUSG00000029253	Cenpc1	centromere protein C1 [Source:MGI Symbol;Acc:M	0.075916
ENSMUSG00000094002	Gm9866	predicted gene 9866 [Source:MGI Symbol;Acc:MGI:	0.075954
ENSMUSG00000034730	Bai1	brain-specific angiogenesis inhibitor 1 [Source:MGI	0.075973
ENSMUSG00000032773	Chrm1	cholinergic receptor, muscarinic 1, CNS [Source:MG	0.075992
ENSMUSG00000042462	Dctpp1	dCTP pyrophosphatase 1 [Source:MGI Symbol;Acc:I	0.075999
ENSMUSG00000022092	Ppp3cc	protein phosphatase 3, catalytic subunit, gamma iso	0.07603
ENSMUSG00000026198	Abcb6	ATP-binding cassette, sub-family B (MDR/TAP), mei	0.076128
ENSMUSG00000042029	Ncapg2	non-SMC condensin II complex, subunit G2 [Source	0.076139
ENSMUSG00000003344	Btbd2	BTB (POZ) domain containing 2 [Source:MGI Symbo	0.076171
ENSMUSG00000038816	Ctnnal1	catenin (cadherin associated protein), alpha-like 1 [	0.076264
ENSMUSG00000025868	Higd2a	HIG1 domain family, member 2A [Source:MGI Symb	0.076466
ENSMUSG00000000957	Mmp14	matrix metallopeptidase 14 (membrane-inserted) [	0.076472
ENSMUSG00000075330	A930003A15Rik	RIKEN cDNA A930003A15 gene [Source:MGI Symbo	0.076523
ENSMUSG00000039953	Clstn1	calsyntenin 1 [Source:MGI Symbol;Acc:MGI:192989	0.076534
ENSMUSG00000078350	Smim1	small integral membrane protein 1 [Source:MGI Syi	0.076664
ENSMUSG00000022428	Cby1	chibby homolog 1 (Drosophila) [Source:MGI Symbo	0.076673
ENSMUSG00000020990	Cdkl1	cyclin-dependent kinase-like 1 (CDC2-related kinase	0.0767
ENSMUSG00000020886	Dlg4	discs, large homolog 4 (Drosophila) [Source:MGI Sy	0.076721
ENSMUSG00000026644	Acbd7	acyl-Coenzyme A binding domain containing 7 [Sou	0.07673
ENSMUSG00000075553	Gm5464	predicted gene 5464 [Source:MGI Symbol;Acc:MGI:	0.076785
ENSMUSG00000070392	Gm20634	predicted gene 20634 [Source:MGI Symbol;Acc:MG	0.076831
ENSMUSG00000032553	Srprb	signal recognition particle receptor, B subunit [Sour	0.076895
ENSMUSG00000048003	Catsper4	cation channel, sperm associated 4 [Source:MGI Sy	0.076907
ENSMUSG00000054874	Pcnxl3	pecanex-like 3 (Drosophila) [Source:MGI Symbol;Ac	0.076938
ENSMUSG00000030720	Cln3	ceroid lipofuscinosis, neuronal 3, juvenile (Batten, S	0.076974
ENSMUSG00000031820	Babam1	BRISC and BRCA1 A complex member 1 [Source:MG	0.077016
ENSMUSG00000053615	Gm9913	predicted gene 9913 [Source:MGI Symbol;Acc:MGI:	0.077049
ENSMUSG00000005469	Prkaca	protein kinase, cAMP dependent, catalytic, alpha [S	0.077053
ENSMUSG00000050299	Gm9843	predicted gene 9843 [Source:MGI Symbol;Acc:MGI:	0.07706
ENSMUSG00000071568	Gm5874	predicted gene 5874 [Source:MGI Symbol;Acc:MGI:	0.077064
ENSMUSG00000070729	Gm12966	predicted gene 12966 [Source:MGI Symbol;Acc:MG	0.077065
ENSMUSG00000041035	Dpcd	deleted in primary ciliary dyskinesia [Source:MGI Sy	0.077118
ENSMUSG00000085553	Gm14808	predicted gene 14808 [Source:MGI Symbol;Acc:MG	0.077148
ENSMUSG00000098234	Snhg6	small nucleolar RNA host gene (non-protein coding	0.077163
ENSMUSG00000027215	Cd82	CD82 antigen [Source:MGI Symbol;Acc:MGI:104651	0.077193

ENSMUSG00000085447	9530026F06Rik	RIKEN cDNA 9530026F06 gene [Source:MGI Symbo	0.077207
ENSMUSG00000040490	Lrfn2	leucine rich repeat and fibronectin type III domain	0.077224
ENSMUSG00000050266	Olfr690	olfactory receptor 690 [Source:MGI Symbol;Acc:M	0.077272
ENSMUSG00000036596	Cpz	carboxypeptidase Z [Source:MGI Symbol;Acc:MGI:8	0.077281
ENSMUSG00000021911	Parg	poly (ADP-ribose) glycohydrolase [Source:MGI Sym	0.077302
ENSMUSG00000042772	Smg7	Smg-7 homolog, nonsense mediated mRNA decay f	0.077323
ENSMUSG00000082683	Gm12697	predicted gene 12697 [Source:MGI Symbol;Acc:MG	0.077423
ENSMUSG00000064731	Snord45b	small nucleolar RNA, C/D box 45B [Source:MGI Sym	0.07743
ENSMUSG00000095947	Gm25195	predicted gene, 25195 [Source:MGI Symbol;Acc:M	0.077431
ENSMUSG00000019772	Vip	vasoactive intestinal polypeptide [Source:MGI Sym	0.077482
ENSMUSG00000023104	Rfc2	replication factor C (activator 1) 2 [Source:MGI Sym	0.077525
ENSMUSG00000034224	Slc38a8	solute carrier family 38, member 8 [Source:MGI Sy	0.077526
ENSMUSG00000001445	Mrpl10	mitochondrial ribosomal protein L10 [Source:MGI S	0.077571
ENSMUSG00000032393	Dpp8	dipeptidylpeptidase 8 [Source:MGI Symbol;Acc:MG	0.077618
ENSMUSG00000093190	Gm22875	predicted gene, 22875 [Source:MGI Symbol;Acc:M	0.07771
ENSMUSG00000098148	Gm20204	predicted gene, 20204 [Source:MGI Symbol;Acc:M	0.07774
ENSMUSG00000041842	Fhdc1	FH2 domain containing 1 [Source:MGI Symbol;Acc:	0.077744
ENSMUSG00000032446	Eomes	eomesodermin homolog (Xenopus laevis) [Source:N	0.07775
ENSMUSG00000018841	Rad51d	RAD51 homolog D [Source:MGI Symbol;Acc:MGI:12	0.077848
ENSMUSG00000044060	A830010M20Rik	RIKEN cDNA A830010M20 gene [Source:MGI Symb	0.077852
ENSMUSG00000094410	Zbed6	zinc finger, BED domain containing 6 [Source:MGI S	0.077921
ENSMUSG00000013275	Slc41a1	solute carrier family 41, member 1 [Source:MGI Sy	0.077964
ENSMUSG00000086756	Gm14051	predicted gene 14051 [Source:MGI Symbol;Acc:MG	0.077977
ENSMUSG00000063268	Parp10	poly (ADP-ribose) polymerase family, member 10 [S	0.077998
ENSMUSG00000045216	Hs6st1	heparan sulfate 6-O-sulfotransferase 1 [Source:MG	0.07806
ENSMUSG00000045980	Tmem104	transmembrane protein 104 [Source:MGI Symbol;A	0.078126
ENSMUSG00000029819	Npy	neuropeptide Y [Source:MGI Symbol;Acc:MGI:9737	0.078164
ENSMUSG00000022565	Plec	plectin [Source:MGI Symbol;Acc:MGI:1277961]	0.07817
ENSMUSG00000074649	BC029722	cDNA sequence BC029722 [Source:MGI Symbol;Acc	0.07827
ENSMUSG00000030157	Clec2d	C-type lectin domain family 2, member d [Source:M	0.078278
ENSMUSG00000088583	Gm22965	predicted gene, 22965 [Source:MGI Symbol;Acc:M	0.078289
ENSMUSG00000037525	Bcdin3d	BCDIN3 domain containing [Source:MGI Symbol;Ac	0.0783
ENSMUSG00000083806	Gm12131	predicted gene 12131 [Source:MGI Symbol;Acc:MG	0.078339
ENSMUSG00000032410	Xrn1	5'-3' exoribonuclease 1 [Source:MGI Symbol;Acc:M	0.078368
ENSMUSG00000022285	Ywhaz	tyrosine 3-monooxygenase/tryptophan 5-monooxy	0.078379
ENSMUSG00000038369	Ncoa6	nuclear receptor coactivator 6 [Source:MGI Symbol	0.078386
ENSMUSG00000029359	Tesc	tescalcin [Source:MGI Symbol;Acc:MGI:1930803]	0.078411
ENSMUSG00000062937	Mtap	methylthioadenosine phosphorylase [Source:MGI S	0.078492
ENSMUSG00000039523	Cep104	centrosomal protein 104 [Source:MGI Symbol;Acc:I	0.078546
ENSMUSG00000068859	Sp9	trans-acting transcription factor 9 [Source:MGI Sym	0.078556
ENSMUSG00000043241	Upf2	UPF2 regulator of nonsense transcripts homolog (y	0.078557
ENSMUSG00000027907	S100a11	S100 calcium binding protein A11 (calgizzarin) [Sou	0.078576
ENSMUSG00000083883	Gm14941	predicted gene 14941 [Source:MGI Symbol;Acc:MG	0.078608
ENSMUSG00000050215	Olfr70	olfactory receptor 70 [Source:MGI Symbol;Acc:MGI	0.078628
ENSMUSG00000083241	Gm13259	predicted gene 13259 [Source:MGI Symbol;Acc:MG	0.078629
ENSMUSG00000044556	Tex38	testis expressed 38 [Source:MGI Symbol;Acc:MGI:1	0.078649
ENSMUSG00000024867	Pip5k1b	phosphatidylinositol-4-phosphate 5-kinase, type 1	0.078674

ENSMUSG00000086578	Gm13583	predicted gene 13583 [Source:MGI Symbol;Acc:MG	0.078692
ENSMUSG00000061451	Tmem151a	transmembrane protein 151A [Source:MGI Symbol;	0.078694
ENSMUSG00000023918	Gpr115	G protein-coupled receptor 115 [Source:MGI Symb	0.078695
ENSMUSG00000062054	Iah1	isoamyl acetate-hydrolyzing esterase 1 homolog (S.	0.078727
ENSMUSG00000034430	Zxdc	ZXD family zinc finger C [Source:MGI Symbol;Acc:M	0.078742
ENSMUSG00000029831	Npvf	neuropeptide VF precursor [Source:MGI Symbol;Ac	0.078744
ENSMUSG00000091968	Gm17115	predicted gene 17115 [Source:MGI Symbol;Acc:MG	0.078748
ENSMUSG00000028955	Vamp3	vesicle-associated membrane protein 3 [Source:MG	0.078783
ENSMUSG00000078838	Gm17382	predicted gene, 17382 [Source:MGI Symbol;Acc:MG	0.078793
ENSMUSG00000047515	BC049715	cDNA sequence BC049715 [Source:MGI Symbol;Acc	0.078859
ENSMUSG00000036777	Anln	anillin, actin binding protein [Source:MGI Symbol;A	0.0789
ENSMUSG00000038115	Ano2	anoctamin 2 [Source:MGI Symbol;Acc:MGI:238721	0.079105
ENSMUSG00000024292	Cyp4f14	cytochrome P450, family 4, subfamily f, polypeptide	0.079139
ENSMUSG00000053046	Brsk2	BR serine/threonine kinase 2 [Source:MGI Symbol;A	0.079171
ENSMUSG00000041180	Hectd2	HECT domain containing 2 [Source:MGI Symbol;Acc	0.07929
ENSMUSG00000053977	Cd8a	CD8 antigen, alpha chain [Source:MGI Symbol;Acc:I	0.079375
ENSMUSG00000090084	Srpx	sushi-repeat-containing protein [Source:MGI Symbo	0.079396
ENSMUSG00000064325	Hhip	Hedgehog-interacting protein [Source:MGI Symbol;	0.07945
ENSMUSG00000038537	Mc3r	melanocortin 3 receptor [Source:MGI Symbol;Acc:M	0.079479
ENSMUSG00000083458	Gm5510	predicted pseudogene 5510 [Source:MGI Symbol;A	0.079508
ENSMUSG00000023050	Map3k12	mitogen-activated protein kinase kinase kinase 12 [	0.079561
ENSMUSG00000022521	Crebbp	CREB binding protein [Source:MGI Symbol;Acc:MGI	0.079565
ENSMUSG00000036555	Iqce	IQ motif containing E [Source:MGI Symbol;Acc:MGI	0.079602
ENSMUSG00000031833	Mast3	microtubule associated serine/threonine kinase 3 [	0.079612
ENSMUSG00000040464	Gtpbp10	GTP-binding protein 10 (putative) [Source:MGI Sym	0.079655
ENSMUSG00000022659	Gcsam	germinal center associated, signaling and motility [	0.079704
ENSMUSG00000067872	Ccdc87	coiled-coil domain containing 87 [Source:MGI Symb	0.079766
ENSMUSG00000032285	Dnaja4	DnaJ (Hsp40) homolog, subfamily A, member 4 [Sou	0.079787
ENSMUSG00000030483	Cyp2b10	cytochrome P450, family 2, subfamily b, polypeptid	0.079816
ENSMUSG00000015312	Gadd45b	growth arrest and DNA-damage-inducible 45 beta [	0.079912
ENSMUSG00000062169	Cnih4	cornichon homolog 4 (Drosophila) [Source:MGI Syn	0.079936
ENSMUSG00000030443	Zfp583	zinc finger protein 583 [Source:MGI Symbol;Acc:MG	0.079979
ENSMUSG00000033880	Lgals3bp	lectin, galactoside-binding, soluble, 3 binding prote	0.080023
ENSMUSG00000029470	P2rx4	purinergic receptor P2X, ligand-gated ion channel 4	0.08003
ENSMUSG00000085239	Gm12150	predicted gene 12150 [Source:MGI Symbol;Acc:MG	0.080044
ENSMUSG00000091096	Gm17304	predicted gene, 17304 [Source:MGI Symbol;Acc:MG	0.080053
ENSMUSG00000056257	Gm5447	predicted gene 5447 [Source:MGI Symbol;Acc:MGI:	0.080055
ENSMUSG00000020132	Rab21	RAB21, member RAS oncogene family [Source:MGI	0.080084
ENSMUSG00000022672	Prkdc	protein kinase, DNA activated, catalytic polypeptide	0.080124
ENSMUSG00000097040	2610316D01Rik	RIKEN cDNA 2610316D01 gene [Source:MGI Symbo	0.080236
ENSMUSG00000000617	Grm6	glutamate receptor, metabotropic 6 [Source:MGI S	0.080259
ENSMUSG00000050989	Sepn1	selenoprotein N, 1 [Source:MGI Symbol;Acc:MGI:21	0.080267
ENSMUSG00000048402	Gli2	GLI-Kruppel family member GLI2 [Source:MGI Symb	0.080273
ENSMUSG00000020876	Snx11	sorting nexin 11 [Source:MGI Symbol;Acc:MGI:1921	0.080275
ENSMUSG00000025103	Btbd1	BTB (POZ) domain containing 1 [Source:MGI Symbo	0.080301
ENSMUSG00000007891	Ctsd	cathepsin D [Source:MGI Symbol;Acc:MGI:88562]	0.080335
ENSMUSG00000085826	Gm15638	predicted gene 15638 [Source:MGI Symbol;Acc:MG	0.080539

ENSMUSG00000039298	Cdk5rap2	CDK5 regulatory subunit associated protein 2 [Sour	0.080552
ENSMUSG00000037060	Prkcdbp	protein kinase C, delta binding protein [Source:MGI	0.080564
ENSMUSG00000097489	Gm26808	predicted gene, 26808 [Source:MGI Symbol;Acc:MG	0.08059
ENSMUSG00000024735	Prpf19	PRP19/PSO4 pre-mRNA processing factor 19 homo	0.080656
ENSMUSG00000030282	Cmas	cytidine monophospho-N-acetylneuraminic acid syn	0.080667
ENSMUSG00000056553	Ptprn2	protein tyrosine phosphatase, receptor type, N poly	0.08067
ENSMUSG00000031807	Pgls	6-phosphogluconolactonase [Source:MGI Symbol;A	0.080679
ENSMUSG00000010376	Nedd8	neural precursor cell expressed, developmentally d	0.080689
ENSMUSG00000055762	Eef1d	eukaryotic translation elongation factor 1 delta (gu	0.080741
ENSMUSG00000045009	Prrt3	proline-rich transmembrane protein 3 [Source:MGI	0.080799
ENSMUSG00000040029	Ipo8	importin 8 [Source:MGI Symbol;Acc:MGI:2444611]	0.080802
ENSMUSG00000029335	Bmp3	bone morphogenetic protein 3 [Source:MGI Symbo	0.080871
ENSMUSG00000021048	Mthfd1	methylenetetrahydrofolate dehydrogenase (NADP+	0.080889
ENSMUSG00000002660	Clpp	ClpP caseinolytic peptidase, ATP-dependent, protec	0.080915
ENSMUSG00000055805	Fmnl1	formin-like 1 [Source:MGI Symbol;Acc:MGI:188899	0.080961
ENSMUSG00000083783	Gm12739	predicted gene 12739 [Source:MGI Symbol;Acc:MG	0.080997
ENSMUSG00000043388	Tmem130	transmembrane protein 130 [Source:MGI Symbol;A	0.081023
ENSMUSG00000040725	Hnrnpul1	heterogeneous nuclear ribonucleoprotein U-like 1 [	0.081066
ENSMUSG00000081974	Gm11960	predicted gene 11960 [Source:MGI Symbol;Acc:MG	0.08127
ENSMUSG00000020435	Osbp2	oxysterol binding protein 2 [Source:MGI Symbol;Ac	0.08128
ENSMUSG00000094649	Gm7102	predicted gene 7102 [Source:MGI Symbol;Acc:MGI:	0.081307
ENSMUSG00000030613	Ccdc90b	coiled-coil domain containing 90B [Source:MGI Sym	0.08132
ENSMUSG00000003575	Crtc1	CREB regulated transcription coactivator 1 [Source:	0.081327
ENSMUSG00000022881	Rfc4	replication factor C (activator 1) 4 [Source:MGI Sym	0.08134
ENSMUSG00000031561	Tenm3	teneurin transmembrane protein 3 [Source:MGI Sy	0.08134
ENSMUSG00000008892	Vdac3	voltage-dependent anion channel 3 [Source:MGI Sy	0.081352
ENSMUSG00000062444	Ap3b2	adaptor-related protein complex 3, beta 2 subunit [	0.0814
ENSMUSG00000024109	Nrxn1	neurexin I [Source:MGI Symbol;Acc:MGI:1096391]	0.081404
ENSMUSG00000020694	Tlk2	tousled-like kinase 2 (Arabidopsis) [Source:MGI Syn	0.081427
ENSMUSG00000013858	Tmem259	transmembrane protein 259 [Source:MGI Symbol;A	0.081522
ENSMUSG00000037243	Zfp692	zinc finger protein 692 [Source:MGI Symbol;Acc:MG	0.081526
ENSMUSG00000012123	Aim1l	absent in melanoma 1-like [Source:MGI Symbol;Acc	0.081537
ENSMUSG00000019892	Lrriq1	leucine-rich repeats and IQ motif containing 1 [Sou	0.081604
ENSMUSG00000031274	Col4a5	collagen, type IV, alpha 5 [Source:MGI Symbol;Acc:	0.081615
ENSMUSG00000028339	Col15a1	collagen, type XV, alpha 1 [Source:MGI Symbol;Acc	0.081701
ENSMUSG00000020400	Tnip1	TNFAIP3 interacting protein 1 [Source:MGI Symbol;	0.08172
ENSMUSG00000022243	Slc45a2	solute carrier family 45, member 2 [Source:MGI Syn	0.081735
ENSMUSG00000035764	Fbxo45	F-box protein 45 [Source:MGI Symbol;Acc:MGI:244	0.081746
ENSMUSG00000086837	Gm16618	predicted gene, 16618 [Source:MGI Symbol;Acc:MG	0.081758
ENSMUSG00000039391	Ccdc81	coiled-coil domain containing 81 [Source:MGI Symb	0.081761
ENSMUSG00000019470	Xab2	XPA binding protein 2 [Source:MGI Symbol;Acc:MG	0.0818
ENSMUSG00000047617	BC029214	cDNA sequence BC029214 [Source:MGI Symbol;Acc	0.081828
ENSMUSG00000030220	Arhgdib	Rho, GDP dissociation inhibitor (GDI) beta [Source:I	0.081891
ENSMUSG00000037470	Uggt1	UDP-glucose glycoprotein glucosyltransferase 1 [So	0.081901
ENSMUSG00000032198	Dock6	dedicator of cytokinesis 6 [Source:MGI Symbol;Acc:	0.081961
ENSMUSG00000054618	Gm9951	predicted gene 9951 [Source:MGI Symbol;Acc:MGI:	0.081981
ENSMUSG00000043168	4930426D05Rik	RIKEN cDNA 4930426D05 gene [Source:MGI Symbo	0.082042

ENSMUSG00000024958	Gpr137	G protein-coupled receptor 137 [Source:MGI Symb	0.082157
ENSMUSG00000066440	Zfyve26	zinc finger, FYVE domain containing 26 [Source:MG	0.082177
ENSMUSG00000032966	Fkbp1a	FK506 binding protein 1a [Source:MGI Symbol;Acc:	0.082219
ENSMUSG00000087264	1500002O10Rik	RIKEN cDNA 1500002O10 gene [Source:MGI Symbo	0.082268
ENSMUSG00000089917	Uckl1	uridine-cytidine kinase 1-like 1 [Source:MGI Symbo	0.082312
ENSMUSG00000061286	Exosc5	exosome component 5 [Source:MGI Symbol;Acc:M	0.08234
ENSMUSG00000070348	Ccnd1	cyclin D1 [Source:MGI Symbol;Acc:MGI:88313]	0.082387
ENSMUSG00000049482	Ctu2	cytosolic thiouridylase subunit 2 homolog (S. pomb	0.082474
ENSMUSG00000038400	Pmepa1	prostate transmembrane protein, androgen induce	0.082537
ENSMUSG00000043866	Taf10	TAF10 RNA polymerase II, TATA box binding protei	0.082572
ENSMUSG00000033128	Gga1	golgi associated, gamma adaptin ear containing, AR	0.082671
ENSMUSG00000039481	Nrtn	neurturin [Source:MGI Symbol;Acc:MGI:108417]	0.082675
ENSMUSG00000022673	Mcm4	minichromosome maintenance deficient 4 homolog	0.082701
ENSMUSG00000001707	Eef1e1	eukaryotic translation elongation factor 1 epsilon 1	0.082704
ENSMUSG00000071540	3425401B19Rik	RIKEN cDNA 3425401B19 gene [Source:MGI Symbo	0.082729
ENSMUSG00000080237	Gm14239	predicted gene 14239 [Source:MGI Symbol;Acc:MG	0.082773
ENSMUSG00000034761	Map4k5	mitogen-activated protein kinase kinase kinase kina	0.082819
ENSMUSG00000037007	Zfp113	zinc finger protein 113 [Source:MGI Symbol;Acc:MG	0.082882
ENSMUSG00000017307	Acot8	acyl-CoA thioesterase 8 [Source:MGI Symbol;Acc:M	0.083021
ENSMUSG00000060459	Kng2	kininogen 2 [Source:MGI Symbol;Acc:MGI:3027157	0.083031
ENSMUSG00000081276	Gm13132	predicted gene 13132 [Source:MGI Symbol;Acc:MG	0.083041
ENSMUSG00000030699	Tbx6	T-box 6 [Source:MGI Symbol;Acc:MGI:102539]	0.083052
ENSMUSG00000035158	Mitf	microphthalmia-associated transcription factor [So	0.083058
ENSMUSG00000032261	Sh3bgrl2	SH3 domain binding glutamic acid-rich protein like	0.083083
ENSMUSG00000027466	Rbck1	RanBP-type and C3HC4-type zinc finger containing	0.083097
ENSMUSG00000061540	Orm2	orosomucoid 2 [Source:MGI Symbol;Acc:MGI:9744	0.083191
ENSMUSG00000029718	Pcolce	procollagen C-endopeptidase enhancer protein [So	0.083203
ENSMUSG00000020716	Nf1	neurofibromatosis 1 [Source:MGI Symbol;Acc:MGI:	0.083205
ENSMUSG00000070639	Lrrc8b	leucine rich repeat containing 8 family, member B [	0.083215
ENSMUSG00000032624	Eml4	echinoderm microtubule associated protein like 4 [	0.083297
ENSMUSG00000061028	Clasrp	CLK4-associating serine/arginine rich protein [Sour	0.083322
ENSMUSG00000037499	Nenf	neuron derived neurotrophic factor [Source:MGI Sy	0.083337
ENSMUSG00000022425	Enpp2	ectonucleotide pyrophosphatase/phosphodiesteras	0.0834
ENSMUSG00000026247	Ecel1	endothelin converting enzyme-like 1 [Source:MGI S	0.083459
ENSMUSG00000057531	Dtnbp1	dystrobrevin binding protein 1 [Source:MGI Symbo	0.083459
ENSMUSG00000055069	Rab39	RAB39, member RAS oncogene family [Source:MGI	0.083475
ENSMUSG00000045128	Rpl18a	ribosomal protein L18A [Source:MGI Symbol;Acc:M	0.083573
ENSMUSG00000031461	Myom2	myomesin 2 [Source:MGI Symbol;Acc:MGI:132835	0.083587
ENSMUSG00000020248	Nfyb	nuclear transcription factor-Y beta [Source:MGI Sy	0.083591
ENSMUSG00000038352	Arl5c	ADP-ribosylation factor-like 5C [Source:MGI Symbo	0.083609
ENSMUSG00000027273	Snap25	synaptosomal-associated protein 25 [Source:MGI S	0.083625
ENSMUSG00000039824	Myl6b	myosin, light polypeptide 6B [Source:MGI Symbol;A	0.083632
ENSMUSG00000028164	Manba	mannosidase, beta A, lysosomal [Source:MGI Symb	0.083691
ENSMUSG00000043541	Casc1	cancer susceptibility candidate 1 [Source:MGI Symb	0.083783
ENSMUSG00000039860	Srrm3	serine/arginine repetitive matrix 3 [Source:MGI Sy	0.083817
ENSMUSG00000022756	Slc7a4	solute carrier family 7 (cationic amino acid transpo	0.083829
ENSMUSG00000031672	Got2	glutamate oxaloacetate transaminase 2, mitochond	0.083894

ENSMUSG00000086186	1700024P03Rik	RIKEN cDNA 1700024P03 gene [Source:MGI Symbo	0.083927
ENSMUSG00000063200	Nol7	nucleolar protein 7 [Source:MGI Symbol;Acc:MGI:1	0.083948
ENSMUSG00000042032	Mat2b	methionine adenosyltransferase II, beta [Source:M	0.083962
ENSMUSG00000026527	Rgs7	regulator of G protein signaling 7 [Source:MGI Sym	0.084001
ENSMUSG00000097702	Gm26739	predicted gene, 26739 [Source:MGI Symbol;Acc:M	0.084022
ENSMUSG00000084328	Gm14046	predicted gene 14046 [Source:MGI Symbol;Acc:MG	0.084038
ENSMUSG00000029601	Iqcd	IQ motif containing D [Source:MGI Symbol;Acc:MG	0.08405
ENSMUSG00000089935	Gm16343	predicted gene 16343 [Source:MGI Symbol;Acc:MG	0.084051
ENSMUSG00000022759	4930451C15Rik	RIKEN cDNA 4930451C15 gene [Source:MGI Symbo	0.084059
ENSMUSG00000063245	Gm13248	predicted gene 13248 [Source:MGI Symbol;Acc:MG	0.084217
ENSMUSG00000031697	Orc6	origin recognition complex, subunit 6 [Source:MGI	0.084225
ENSMUSG00000027423	Snx5	sorting nexin 5 [Source:MGI Symbol;Acc:MGI:1916	0.084236
ENSMUSG00000059498	Fcgr3	Fc receptor, IgG, low affinity III [Source:MGI Symbo	0.084276
ENSMUSG00000085261	Gm13814	predicted gene 13814 [Source:MGI Symbol;Acc:MG	0.084369
ENSMUSG00000074166	AW146154	expressed sequence AW146154 [Source:MGI Symb	0.084379
ENSMUSG00000085991	Gm11479	predicted gene 11479 [Source:MGI Symbol;Acc:MG	0.084383
ENSMUSG00000042447	Mios	missing oocyte, meiosis regulator, homolog (Droso	0.084404
ENSMUSG00000045551	Fpr1	formyl peptide receptor 1 [Source:MGI Symbol;Acc	0.084406
ENSMUSG00000039533	Mmd2	monocyte to macrophage differentiation-associate	0.084411
ENSMUSG00000083306	Gm13868	predicted gene 13868 [Source:MGI Symbol;Acc:MG	0.084514
ENSMUSG00000048581	E130311K13Rik	RIKEN cDNA E130311K13 gene [Source:MGI Symbo	0.084587
ENSMUSG00000023026	Dip2b	DIP2 disco-interacting protein 2 homolog B (Drosop	0.08475
ENSMUSG00000061474	Mrps36	mitochondrial ribosomal protein S36 [Source:MGI S	0.084796
ENSMUSG00000079654	Prrt4	proline-rich transmembrane protein 4 [Source:MGI	0.084831
ENSMUSG00000002129	Sf3a1	splicing factor 3a, subunit 1 [Source:MGI Symbol;Ac	0.084892
ENSMUSG00000000411	Tssk3	testis-specific serine kinase 3 [Source:MGI Symbol;	0.084914
ENSMUSG00000080527	Gm23880	predicted gene, 23880 [Source:MGI Symbol;Acc:M	0.084961
ENSMUSG00000034931	Dhx8	DEAH (Asp-Glu-Ala-His) box polypeptide 8 [Source:	0.084996
ENSMUSG00000001441	Npepps	aminopeptidase puromycin sensitive [Source:MGI S	0.085033
ENSMUSG00000027859	Ngf	nerve growth factor [Source:MGI Symbol;Acc:MGI:	0.085046
ENSMUSG00000022175	Lrp10	low-density lipoprotein receptor-related protein 10	0.085062
ENSMUSG00000039347	Atp6v0e2	ATPase, H+ transporting, lysosomal V0 subunit E2 [	0.085073
ENSMUSG00000030779	Rbbp6	retinoblastoma binding protein 6 [Source:MGI Sym	0.085082
ENSMUSG00000020459	Mtif2	mitochondrial translational initiation factor 2 [Sour	0.085135
ENSMUSG00000072889	Nfxl1	nuclear transcription factor, X-box binding-like 1 [S	0.085189
ENSMUSG00000026312	Cdh7	cadherin 7, type 2 [Source:MGI Symbol;Acc:MGI:24	0.085212
ENSMUSG00000036098	Myrf	myelin regulatory factor [Source:MGI Symbol;Acc:M	0.085248
ENSMUSG00000092178	Gm3912	predicted gene 3912 [Source:MGI Symbol;Acc:MGI	0.085253
ENSMUSG00000005823	Gpr108	G protein-coupled receptor 108 [Source:MGI Symb	0.085267
ENSMUSG00000051403	Ppp1r37	protein phosphatase 1, regulatory subunit 37 [Sour	0.08533
ENSMUSG00000052504	Epha3	Eph receptor A3 [Source:MGI Symbol;Acc:MGI:996	0.085347
ENSMUSG00000029832	Nfe2l3	nuclear factor, erythroid derived 2, like 3 [Source:M	0.08537
ENSMUSG00000031849	Comp	cartilage oligomeric matrix protein [Source:MGI Sy	0.085388
ENSMUSG00000034659	Tmem109	transmembrane protein 109 [Source:MGI Symbol;A	0.085406
ENSMUSG00000062397	Zfp706	zinc finger protein 706 [Source:MGI Symbol;Acc:M	0.085413
ENSMUSG00000040978	Gm11992	predicted gene 11992 [Source:MGI Symbol;Acc:MG	0.08549
ENSMUSG00000021795	Sftpd	surfactant associated protein D [Source:MGI Symbo	0.085496

ENSMUSG00000048546	Tob2	transducer of ERBB2, 2 [Source:MGI Symbol;Acc:M	0.085574
ENSMUSG00000089158	Gm23583	predicted gene, 23583 [Source:MGI Symbol;Acc:M	0.085597
ENSMUSG00000028076	Cd1d1	CD1d1 antigen [Source:MGI Symbol;Acc:MGI:1076	0.085642
ENSMUSG00000096655	1700065D16Rik	RIKEN cDNA 1700065D16 gene [Source:MGI Symbo	0.085666
ENSMUSG00000063239	Grm4	glutamate receptor, metabotropic 4 [Source:MGI S	0.085749
ENSMUSG00000020810	Cygb	cytoglobin [Source:MGI Symbol;Acc:MGI:2149481]	0.085758
ENSMUSG00000087674	4930447M23Rik	RIKEN cDNA 4930447M23 gene [Source:MGI Symb	0.085786
ENSMUSG00000049148	Plcxd3	phosphatidylinositol-specific phospholipase C, X do	0.085824
ENSMUSG00000022391	Rangap1	RAN GTPase activating protein 1 [Source:MGI Symb	0.085939
ENSMUSG00000029458	Brap	BRCA1 associated protein [Source:MGI Symbol;Acc	0.085959
ENSMUSG00000029673	Auts2	autism susceptibility candidate 2 [Source:MGI Symb	0.085961
ENSMUSG00000017677	Wsb1	WD repeat and SOCS box-containing 1 [Source:MGI	0.085989
ENSMUSG00000026491	Ahctf1	AT hook containing transcription factor 1 [Source:M	0.086034
ENSMUSG00000024937	Ehbp1l1	EH domain binding protein 1-like 1 [Source:MGI Sy	0.086169
ENSMUSG00000042066	Tmcc2	transmembrane and coiled-coil domains 2 [Source:	0.086253
ENSMUSG00000097904	Gm26711	predicted gene, 26711 [Source:MGI Symbol;Acc:M	0.086293
ENSMUSG00000069372	Ctxn3	cortexin 3 [Source:MGI Symbol;Acc:MGI:3642816]	0.086299
ENSMUSG00000009566	Fpgs	folylpolyglutamyl synthetase [Source:MGI Symbol;	0.08633
ENSMUSG00000086877	A230072C01Rik	RIKEN cDNA A230072C01 gene [Source:MGI Symbo	0.086341
ENSMUSG00000034891	Sncb	synuclein, beta [Source:MGI Symbol;Acc:MGI:1889	0.086394
ENSMUSG00000030495	Slc7a10	solute carrier family 7 (cationic amino acid transpo	0.08641
ENSMUSG00000097167	Gm16740	predicted gene, 16740 [Source:MGI Symbol;Acc:M	0.086432
ENSMUSG00000020451	Limk2	LIM motif-containing protein kinase 2 [Source:MGI	0.086491
ENSMUSG00000038457	Tmem255b	transmembrane protein 255B [Source:MGI Symbol;	0.086559
ENSMUSG00000002603	Tgfb1	transforming growth factor, beta 1 [Source:MGI Sy	0.08656
ENSMUSG00000084907	Gm14505	predicted gene 14505 [Source:MGI Symbol;Acc:MG	0.086573
ENSMUSG00000021963	Sap18	Sin3-associated polypeptide 18 [Source:MGI Symbo	0.086635
ENSMUSG00000030805	Stx4a	syntaxin 4A (placental) [Source:MGI Symbol;Acc:M	0.086684
ENSMUSG00000063590	Slc22a28	solute carrier family 22, member 28 [Source:MGI S	0.086734
ENSMUSG00000054894	Atp5s	ATP synthase, H+ transporting, mitochondrial F0 co	0.086794
ENSMUSG00000073052	D130052B06Rik	RIKEN cDNA D130052B06 gene [Source:MGI Symbo	0.086798
ENSMUSG00000014158	Trpv4	transient receptor potential cation channel, subfam	0.086818
ENSMUSG00000042498	D330045A20Rik	RIKEN cDNA D330045A20 gene [Source:MGI Symbo	0.086824
ENSMUSG00000081866	Gm12401	predicted gene 12401 [Source:MGI Symbol;Acc:MG	0.086835
ENSMUSG00000097149	G630030J09Rik	RIKEN cDNA G630030J09 gene [Source:MGI Symbo	0.086863
ENSMUSG00000028854	Slc9a1	solute carrier family 9 (sodium/hydrogen exchange	0.086865
ENSMUSG00000031738	Irx6	Iroquois related homeobox 6 (Drosophila) [Source:	0.086873
ENSMUSG00000021118	Plek2	pleckstrin 2 [Source:MGI Symbol;Acc:MGI:1351466	0.086942
ENSMUSG00000028646	Rragc	Ras-related GTP binding C [Source:MGI Symbol;Acc	0.086951
ENSMUSG00000033615	Cplx1	complexin 1 [Source:MGI Symbol;Acc:MGI:104727]	0.086966
ENSMUSG00000002052	Supt6	suppressor of Ty 6 [Source:MGI Symbol;Acc:MGI:1	0.087046
ENSMUSG00000087413	Gm11266	predicted gene 11266 [Source:MGI Symbol;Acc:MG	0.087075
ENSMUSG00000002058	Unc119	unc-119 homolog (C. elegans) [Source:MGI Symbol;	0.087111
ENSMUSG00000098021	Gm9522	predicted gene 9522 [Source:MGI Symbol;Acc:MGI	0.087121
ENSMUSG00000001305	Rrp15	ribosomal RNA processing 15 homolog (S. cerevisia	0.087289
ENSMUSG00000025257	Ribc1	RIB43A domain with coiled-coils 1 [Source:MGI Sym	0.087325
ENSMUSG00000040697	Dnajc16	DnaJ (Hsp40) homolog, subfamily C, member 16 [S	0.087354

ENSMUSG00000063060	Sox7	SRY-box containing gene 7 [Source:MGI Symbol;Acc	0.087387
ENSMUSG00000051980	Casr	calcium-sensing receptor [Source:MGI Symbol;Acc:	0.087441
ENSMUSG00000048600	Gm5763	predicted pseudogene 5763 [Source:MGI Symbol;A	0.087475
ENSMUSG00000028458	Tesk1	testis specific protein kinase 1 [Source:MGI Symbol	0.087476
ENSMUSG00000020268	Lyrm7	LYR motif containing 7 [Source:MGI Symbol;Acc:MG	0.087511
ENSMUSG00000033577	Myo6	myosin VI [Source:MGI Symbol;Acc:MGI:104785]	0.08753
ENSMUSG00000028978	Nos3	nitric oxide synthase 3, endothelial cell [Source:MG	0.08755
ENSMUSG00000055194	Actbl2	actin, beta-like 2 [Source:MGI Symbol;Acc:MGI:244	0.087593
ENSMUSG00000025902	Sox17	SRY-box containing gene 17 [Source:MGI Symbol;A	0.08764
ENSMUSG00000022360	Atad2	ATPase family, AAA domain containing 2 [Source:M	0.087688
ENSMUSG00000029686	Cul1	cullin 1 [Source:MGI Symbol;Acc:MGI:1349658]	0.087733
ENSMUSG00000027188	Pamr1	peptidase domain containing associated with musc	0.087736
ENSMUSG00000029710	Ephb4	Eph receptor B4 [Source:MGI Symbol;Acc:MGI:104	0.087768
ENSMUSG00000087222	E030042O20Rik	RIKEN cDNA E030042O20 gene [Source:MGI Symbo	0.087835
ENSMUSG00000028869	Gnl2	guanine nucleotide binding protein-like 2 (nucleola	0.087852
ENSMUSG00000031887	Tradd	TNFRSF1A-associated via death domain [Source:MG	0.087877
ENSMUSG00000028062	Lamtor2	late endosomal/lysosomal adaptor, MAPK and MTC	0.087885
ENSMUSG00000010136	Pifo	primary cilia formation [Source:MGI Symbol;Acc:M	0.087893
ENSMUSG00000046836	Brox	BRO1 domain and CAAX motif containing [Source:N	0.087953
ENSMUSG00000075391	Gm13443	predicted gene 13443 [Source:MGI Symbol;Acc:MG	0.087989
ENSMUSG00000035325	Sec31a	Sec31 homolog A (S. cerevisiae) [Source:MGI Symb	0.088064
ENSMUSG00000052951	C130021I20Rik	Riken cDNA C130021I20 gene [Source:MGI Symbol	0.088174
ENSMUSG00000055629	B4galnt4	beta-1,4-N-acetyl-galactosaminyl transferase 4 [Sou	0.088176
ENSMUSG00000067578	Cbln4	cerebellin 4 precursor protein [Source:MGI Symbol	0.088185
ENSMUSG00000031371	Haus7	HAUS augmin-like complex, subunit 7 [Source:MGI	0.088198
ENSMUSG00000022179	4931414P19Rik	RIKEN cDNA 4931414P19 gene [Source:MGI Symbo	0.088281
ENSMUSG00000028145	Them4	thioesterase superfamily member 4 [Source:MGI Sy	0.088302
ENSMUSG00000001098	Kctd10	potassium channel tetramerisation domain contain	0.088308
ENSMUSG00000042729	Wdr74	WD repeat domain 74 [Source:MGI Symbol;Acc:MG	0.088313
ENSMUSG00000025578	Cbx8	chromobox 8 [Source:MGI Symbol;Acc:MGI:135358	0.088334
ENSMUSG00000024539	Ptpn2	protein tyrosine phosphatase, non-receptor type 2	0.088337
ENSMUSG00000025035	Arl3	ADP-ribosylation factor-like 3 [Source:MGI Symbol;	0.088338
ENSMUSG00000021713	Ppwd1	peptidylprolyl isomerase domain and WD repeat co	0.088341
ENSMUSG00000038793	Lefty1	left right determination factor 1 [Source:MGI Symb	0.088418
ENSMUSG00000034121	Mks1	Meckel syndrome, type 1 [Source:MGI Symbol;Acc:	0.0885
ENSMUSG00000062825	Actg1	actin, gamma, cytoplasmic 1 [Source:MGI Symbol;A	0.088507
ENSMUSG00000053846	Lipg	lipase, endothelial [Source:MGI Symbol;Acc:MGI:13	0.08852
ENSMUSG00000028710	Atpaf1	ATP synthase mitochondrial F1 complex assembly f	0.088583
ENSMUSG00000015942	Gtf2ird2	GTF2I repeat domain containing 2 [Source:MGI Sym	0.08862
ENSMUSG00000000263	Glra1	glycine receptor, alpha 1 subunit [Source:MGI Symb	0.088735
ENSMUSG00000022235	Cmbl	carboxymethylenebutenolidase-like (Pseudomonas	0.088766
ENSMUSG00000066036	Ubr4	ubiquitin protein ligase E3 component n-recognin 4	0.088799
ENSMUSG00000020774	Aspa	aspartoacylase [Source:MGI Symbol;Acc:MGI:8791	0.08883
ENSMUSG00000050730	Arhgap42	Rho GTPase activating protein 42 [Source:MGI Sym	0.088838
ENSMUSG00000033545	Znrf1	zinc and ring finger 1 [Source:MGI Symbol;Acc:MGI	0.088858
ENSMUSG00000058620	Adra2b	adrenergic receptor, alpha 2b [Source:MGI Symbol;	0.088904
ENSMUSG00000028550	Atg4c	autophagy related 4C, cysteine peptidase [Source:N	0.088907

ENSMUSG00000030587	2200002D01Rik	RIKEN cDNA 2200002D01 gene [Source:MGI Symbo	0.088993
ENSMUSG00000090002	Gm16006	predicted gene 16006 [Source:MGI Symbol;Acc:MG	0.088998
ENSMUSG00000025727	A930017K11Rik	RIKEN cDNA A930017K11 gene [Source:MGI Symbo	0.089026
ENSMUSG00000035392	Dennd1a	DENN/MADD domain containing 1A [Source:MGI Sy	0.089071
ENSMUSG00000073375	Lrrc30	leucine rich repeat containing 30 [Source:MGI Syml	0.089132
ENSMUSG00000019897	Ccdc59	coiled-coil domain containing 59 [Source:MGI Symb	0.089295
ENSMUSG00000053414	Hunk	hormonally upregulated Neu-associated kinase [So	0.08935
ENSMUSG00000015745	Plekho1	pleckstrin homology domain containing, family O m	0.089434
ENSMUSG00000038658	C030046E11Rik	RIKEN cDNA C030046E11 gene [Source:MGI Symbo	0.089475
ENSMUSG00000036980	Taf6	TAF6 RNA polymerase II, TATA box binding protein	0.089552
ENSMUSG00000072294	Klf12	Kruppel-like factor 12 [Source:MGI Symbol;Acc:MG	0.089605
ENSMUSG00000054893	Zfp667	zinc finger protein 667 [Source:MGI Symbol;Acc:M(	0.089649
ENSMUSG00000042323	Pbrm1	polybromo 1 [Source:MGI Symbol;Acc:MGI:192399	0.089685
ENSMUSG00000057816	1700007G11Rik	RIKEN cDNA 1700007G11 gene [Source:MGI Symbo	0.089702
ENSMUSG00000024137	E4f1	E4F transcription factor 1 [Source:MGI Symbol;Acc:	0.089708
ENSMUSG00000079462	Gm15737	predicted gene 15737 [Source:MGI Symbol;Acc:MG	0.089711
ENSMUSG00000001802	Lrp3	low density lipoprotein receptor-related protein 3 [	0.089789
ENSMUSG00000035726	Supt16	suppressor of Ty 16 [Source:MGI Symbol;Acc:MGI:1	0.089793
ENSMUSG00000081291	Gm14738	predicted gene 14738 [Source:MGI Symbol;Acc:MG	0.089855
ENSMUSG00000025920	Stau2	staufen (RNA binding protein) homolog 2 (Drosoph	0.089886
ENSMUSG00000040918	Slc19a2	solute carrier family 19 (thiamine transporter), mer	0.089934
ENSMUSG00000034007	Scaper	S phase cyclin A-associated protein in the ER [Sourc	0.089936
ENSMUSG00000005198	Polr2a	polymerase (RNA) II (DNA directed) polypeptide A [	0.09005
ENSMUSG00000086288	Gm15265	predicted gene 15265 [Source:MGI Symbol;Acc:MG	0.090059
ENSMUSG00000045377	Tmem88	transmembrane protein 88 [Source:MGI Symbol;Ac	0.090102
ENSMUSG00000028048	Gba	glucosidase, beta, acid [Source:MGI Symbol;Acc:M(	0.09015
ENSMUSG00000009621	Vav2	vav 2 oncogene [Source:MGI Symbol;Acc:MGI:1027	0.090168
ENSMUSG00000028289	Epha7	Eph receptor A7 [Source:MGI Symbol;Acc:MGI:952	0.090175
ENSMUSG00000001120	Pcbp3	poly(rC) binding protein 3 [Source:MGI Symbol;Acc	0.090229
ENSMUSG00000050132	Sarm1	sterile alpha and HEAT/Armadillo motif containing	0.090229
ENSMUSG00000038046	Rnmtl1	RNA methyltransferase like 1 [Source:MGI Symbol;	0.090236
ENSMUSG00000027067	Ssrp1	structure specific recognition protein 1 [Source:MG	0.090297
ENSMUSG00000037992	Rara	retinoic acid receptor, alpha [Source:MGI Symbol;A	0.090353
ENSMUSG00000070372	Capza1	capping protein (actin filament) muscle Z-line, alph	0.09041
ENSMUSG00000055202	Zfp811	zinc finger protein 811 [Source:MGI Symbol;Acc:M(	0.090438
ENSMUSG00000026914	Psmd14	proteasome (prosome, macropain) 26S subunit, no	0.090473
ENSMUSG00000022906	Parp9	poly (ADP-ribose) polymerase family, member 9 [S	0.090484
ENSMUSG00000025652	Tmem89	transmembrane protein 89 [Source:MGI Symbol;Ac	0.090548
ENSMUSG00000024084	Qpct	glutaminyl-peptide cyclotransferase (glutaminyl cy	0.090645
ENSMUSG00000020261	Slc36a1	solute carrier family 36 (proton/amino acid sympor	0.090718
ENSMUSG00000024908	Ppp6r3	protein phosphatase 6, regulatory subunit 3 [Sourc	0.090736
ENSMUSG00000030523	Trpm1	transient receptor potential cation channel, subfam	0.090739
ENSMUSG00000004934	Pias4	protein inhibitor of activated STAT 4 [Source:MGI S	0.090744
ENSMUSG00000092174	Gm7029	predicted gene 7029 [Source:MGI Symbol;Acc:MGI:	0.090746
ENSMUSG00000084933	Gm13962	predicted gene 13962 [Source:MGI Symbol;Acc:MG	0.090791
ENSMUSG00000010064	Slc38a3	solute carrier family 38, member 3 [Source:MGI Syr	0.090819
ENSMUSG00000047021	Ccdc108	coiled-coil domain containing 108 [Source:MGI Sym	0.090824

ENSMUSG00000049038	Mterfd3	MTERF domain containing 3 [Source:MGI Symbol;A	0.090836
ENSMUSG00000028949	Smarcd3	SWI/SNF related, matrix associated, actin depende	0.09084
ENSMUSG00000021188	Trip11	thyroid hormone receptor interactor 11 [Source:M	0.090859
ENSMUSG00000027502	Rtfdc1	replication termination factor 2 domain containing	0.090868
ENSMUSG00000042961	Egflam	EGF-like, fibronectin type III and laminin G domains	0.091003
ENSMUSG00000036057	Ptpn23	protein tyrosine phosphatase, non-receptor type 2	0.091028
ENSMUSG00000021747	4930452B06Rik	RIKEN cDNA 4930452B06 gene [Source:MGI Symbo	0.091099
ENSMUSG00000049420	Tmem200a	transmembrane protein 200A [Source:MGI Symbol	0.09111
ENSMUSG00000017550	Atad5	ATPase family, AAA domain containing 5 [Source:M	0.091164
ENSMUSG00000069094	Pde7a	phosphodiesterase 7A [Source:MGI Symbol;Acc:M	0.091291
ENSMUSG00000028567	Txndc12	thioredoxin domain containing 12 (endoplasmic ret	0.09135
ENSMUSG00000033066	Gas7	growth arrest specific 7 [Source:MGI Symbol;Acc:M	0.091396
ENSMUSG00000029729	Zkscan1	zinc finger with KRAB and SCAN domains 1 [Source:	0.091444
ENSMUSG00000087351	Gm11464	predicted gene 11464 [Source:MGI Symbol;Acc:MG	0.091473
ENSMUSG00000064065	Ipcef1	interaction protein for cytohesin exchange factors	0.091482
ENSMUSG00000040390	Map3k10	mitogen-activated protein kinase kinase kinase 10	0.091488
ENSMUSG00000038135	Crygn	crystallin, gamma N [Source:MGI Symbol;Acc:MGI:	0.09151
ENSMUSG00000017897	Eya2	eyes absent 2 homolog (Drosophila) [Source:MGI S	0.091521
ENSMUSG00000019370	Calm3	calmodulin 3 [Source:MGI Symbol;Acc:MGI:103249	0.091538
ENSMUSG00000032554	Trf	transferrin [Source:MGI Symbol;Acc:MGI:98821]	0.091544
ENSMUSG00000056856	Jakmip3	janus kinase and microtubule interacting protein 3	0.091566
ENSMUSG00000020905	Usp43	ubiquitin specific peptidase 43 [Source:MGI Symbo	0.091686
ENSMUSG00000063235	Ptpmt1	protein tyrosine phosphatase, mitochondrial 1 [Sou	0.091701
ENSMUSG00000028107	Tars2	threonyl-tRNA synthetase 2, mitochondrial (putativ	0.091864
ENSMUSG00000029328	Hnrnpdl	heterogeneous nuclear ribonucleoprotein D-like [S	0.091884
ENSMUSG00000001418	0610031J06Rik	RIKEN cDNA 0610031J06 gene [Source:MGI Symbol	0.091904
ENSMUSG00000043262	Uevld	UEV and lactate/malate dehyrogenase domains [So	0.091909
ENSMUSG00000028890	Mtf1	metal response element binding transcription facto	0.091943
ENSMUSG00000027642	Rpn2	ribophorin II [Source:MGI Symbol;Acc:MGI:98085]	0.091944
ENSMUSG00000049516	Spty2d1	SPT2, Suppressor of Ty, domain containing 1 (S. cer	0.091974
ENSMUSG00000041801	Phlda3	pleckstrin homology-like domain, family A, membe	0.092068
ENSMUSG00000043251	Exoc3l	exocyst complex component 3-like [Source:MGI Sy	0.092112
ENSMUSG00000024053	Emilin2	elastin microfibril interfacer 2 [Source:MGI Symbol	0.092125
ENSMUSG00000036644	Tbc1d9b	TBC1 domain family, member 9B [Source:MGI Sym	0.09213
ENSMUSG00000070570	Slc17a7	solute carrier family 17 (sodium-dependent inorgar	0.092142
ENSMUSG00000023036	Pcdhga9	protocadherin gamma subfamily A, 9 [Source:MGI	0.092185
ENSMUSG00000002221	Paxip1	PAX interacting (with transcription-activation doma	0.092223
ENSMUSG00000025964	Adam23	a disintegrin and metallopeptidase domain 23 [Sou	0.092253
ENSMUSG00000070995	Gm6313	predicted gene 6313 [Source:MGI Symbol;Acc:MGI	0.092281
ENSMUSG00000027778	Ift80	intraflagellar transport 80 [Source:MGI Symbol;Acc	0.092286
ENSMUSG00000011257	Pabpc4	poly(A) binding protein, cytoplasmic 4 [Source:MGI	0.092309
ENSMUSG00000020817	Rabep1	rabaptin, RAB GTPase binding effector protein 1 [S	0.092408
ENSMUSG00000025917	Cops5	COP9 (constitutive photomorphogenic) homolog, s	0.092426
ENSMUSG00000000365	Rnf17	ring finger protein 17 [Source:MGI Symbol;Acc:MGI	0.09246
ENSMUSG00000059203	Il1rapl2	interleukin 1 receptor accessory protein-like 2 [Sou	0.092473
ENSMUSG00000019861	Gopc	golgi associated PDZ and coiled-coil motif containin	0.092486
ENSMUSG00000006732	Mettl1	methyltransferase like 1 [Source:MGI Symbol;Acc:N	0.092555

ENSMUSG00000024172	St6gal2	beta galactoside alpha 2,6 sialyltransferase 2 [Sourc	0.092586
ENSMUSG00000029386	Tctn2	tectonic family member 2 [Source:MGI Symbol;Acc	0.092611
ENSMUSG00000037119	D15Ertd621e	DNA segment, Chr 15, ERATO Doi 621, expressed [S	0.092636
ENSMUSG00000084141	Olfr1372-ps1	olfactory receptor 1372, pseudogene 1 [Source:MG	0.092711
ENSMUSG00000017264	Exosc10	exosome component 10 [Source:MGI Symbol;Acc:M	0.092718
ENSMUSG00000021840	Mapk1ip1l	mitogen-activated protein kinase 1 interacting prot	0.092723
ENSMUSG00000040167	Ikzf5	IKAROS family zinc finger 5 [Source:MGI Symbol;Ac	0.092759
ENSMUSG00000031622	Sin3b	transcriptional regulator, SIN3B (yeast) [Source:MG	0.092812
ENSMUSG00000032050	Rdx	radixin [Source:MGI Symbol;Acc:MGI:97887]	0.092846
ENSMUSG00000001995	Sipa1l2	signal-induced proliferation-associated 1 like 2 [Sou	0.092931
ENSMUSG00000029068	Ccnl2	cyclin L2 [Source:MGI Symbol;Acc:MGI:1927119]	0.092973
ENSMUSG00000079499	6530402F18Rik	RIKEN cDNA 6530402F18 gene [Source:MGI Symbo	0.093062
ENSMUSG00000025722	Wdr73	WD repeat domain 73 [Source:MGI Symbol;Acc:MG	0.093104
ENSMUSG00000003208	Ccdc94	coiled-coil domain containing 94 [Source:MGI Symb	0.093142
ENSMUSG00000022503	Nubp1	nucleotide binding protein 1 [Source:MGI Symbol;A	0.093174
ENSMUSG00000068452	Duox2	dual oxidase 2 [Source:MGI Symbol;Acc:MGI:30362	0.093197
ENSMUSG00000047897	Ripply2	ripply2 homolog (zebrafish) [Source:MGI Symbol;Ac	0.093237
ENSMUSG00000065694	Gm25411	predicted gene, 25411 [Source:MGI Symbol;Acc:MG	0.093281
ENSMUSG00000032313	AI118078	expressed sequence AI118078 [Source:MGI Symbol	0.09329
ENSMUSG00000020159	Gabrp	gamma-aminobutyric acid (GABA) A receptor, pi [So	0.093292
ENSMUSG00000025644	Gm7628	predicted gene 7628 [Source:MGI Symbol;Acc:MGI:	0.093486
ENSMUSG00000020135	Apc2	adenomatosis polyposis coli 2 [Source:MGI Symbol	0.093489
ENSMUSG00000020424	Gatsl3	GATS protein-like 3 [Source:MGI Symbol;Acc:MGI:1	0.093492
ENSMUSG00000043290	Zfp784	zinc finger protein 784 [Source:MGI Symbol;Acc:MG	0.093499
ENSMUSG00000035469	Rcbtb1	regulator of chromosome condensation (RCC1) and	0.093548
ENSMUSG00000055302	Mrfap1	Morf4 family associated protein 1 [Source:MGI Sym	0.093566
ENSMUSG00000006589	Aprt	adenine phosphoribosyl transferase [Source:MGI Sy	0.093592
ENSMUSG00000003847	Nfat5	nuclear factor of activated T cells 5 [Source:MGI Sy	0.093656
ENSMUSG00000027896	Slc16a4	solute carrier family 16 (monocarboxylic acid transp	0.093751
ENSMUSG00000042520	Ubap2l	ubiquitin associated protein 2-like [Source:MGI Sym	0.093774
ENSMUSG00000061410	Zcchc14	zinc finger, CCHC domain containing 14 [Source:MG	0.093803
ENSMUSG00000073405	C920025E04Rik	RIKEN cDNA C920025E04 gene [Source:MGI Symbo	0.093861
ENSMUSG00000022452	Smdt1	single-pass membrane protein with aspartate rich t	0.093865
ENSMUSG00000051457	Spn	sialophorin [Source:MGI Symbol;Acc:MGI:98384]	0.093865
ENSMUSG00000033161	Atp1a1	ATPase, Na+/K+ transporting, alpha 1 polypeptide [	0.093866
ENSMUSG00000052949	Rnf157	ring finger protein 157 [Source:MGI Symbol;Acc:MG	0.093917
ENSMUSG00000033579	Fa2h	fatty acid 2-hydroxylase [Source:MGI Symbol;Acc:M	0.093919
ENSMUSG00000026203	Dnajb2	DnaJ (Hsp40) homolog, subfamily B, member 2 [Sou	0.094039
ENSMUSG00000055923	Aasdh	aminoadipate-semialdehyde dehydrogenase [Sourc	0.094069
ENSMUSG00000033187	BC016579	cDNA sequence, BC016579 [Source:MGI Symbol;Ac	0.094155
ENSMUSG00000027220	Syt13	synaptotagmin XIII [Source:MGI Symbol;Acc:MGI:1	0.094221
ENSMUSG00000040649	Rimklb	ribosomal modification protein rimK-like family me	0.094289
ENSMUSG00000021253	Tgfb3	transforming growth factor, beta 3 [Source:MGI Sy	0.094294
ENSMUSG00000068348	Gm10238	predicted pseudogene 10238 [Source:MGI Symbol;	0.094366
ENSMUSG00000025776	Crispld1	cysteine-rich secretory protein LCCL domain contai	0.094451
ENSMUSG00000074923	Pak6	p21 protein (Cdc42/Rac)-activated kinase 6 [Source	0.09452
ENSMUSG00000053483	Usp21	ubiquitin specific peptidase 21 [Source:MGI Symbo	0.094606

ENSMUSG00000098168	D630036G22Rik	RIKEN cDNA D630036G22 gene [Source:MGI Symbo	0.094636
ENSMUSG00000020460	Rps27a	ribosomal protein S27A [Source:MGI Symbol;Acc:M	0.094648
ENSMUSG00000027165	B230118H07Rik	RIKEN cDNA B230118H07 gene [Source:MGI Symbo	0.094665
ENSMUSG00000045349	Sh2d5	SH2 domain containing 5 [Source:MGI Symbol;Acc:	0.094688
ENSMUSG00000052293	Taf9	TAF9 RNA polymerase II, TATA box binding protein	0.094713
ENSMUSG00000048483	Zdhhc22	zinc finger, DHHC-type containing 22 [Source:MGI S	0.094747
ENSMUSG00000030213	Atf7ip	activating transcription factor 7 interacting protein	0.094783
ENSMUSG00000026307	Scly	selenocysteine lyase [Source:MGI Symbol;Acc:MGI:	0.094811
ENSMUSG00000032527	Pccb	propionyl Coenzyme A carboxylase, beta polypeptid	0.094816
ENSMUSG00000027224	Duoxa1	dual oxidase maturation factor 1 [Source:MGI Symb	0.094831
ENSMUSG00000018761	Mpdu1	mannose-P-dolichol utilization defect 1 [Source:MG	0.094869
ENSMUSG00000048764	Tmprss11f	transmembrane protease, serine 11f [Source:MGI S	0.095021
ENSMUSG00000025082	Vwa2	von Willebrand factor A domain containing 2 [Sourc	0.095039
ENSMUSG00000013833	Med16	mediator complex subunit 16 [Source:MGI Symbol;	0.095079
ENSMUSG00000020868	Xylt2	xylosyltransferase II [Source:MGI Symbol;Acc:MGI:2	0.095206
ENSMUSG00000048747	E130114P18Rik	RIKEN cDNA E130114P18 gene [Source:MGI Symbo	0.095221
ENSMUSG00000021647	Cartpt	CART prepropeptide [Source:MGI Symbol;Acc:MGI:	0.095235
ENSMUSG00000027665	Pik3ca	phosphatidylinositol 3-kinase, catalytic, alpha polyp	0.095296
ENSMUSG00000021643	Serf1	small EDRK-rich factor 1 [Source:MGI Symbol;Acc:M	0.095403
ENSMUSG00000010095	Slc3a2	solute carrier family 3 (activators of dibasic and neu	0.095409
ENSMUSG00000085851	4921518K17Rik	RIKEN cDNA 4921518K17 gene [Source:MGI Symbo	0.095429
ENSMUSG00000022721	Trmt2a	TRM2 tRNA methyltransferase 2A [Source:MGI Sym	0.095456
ENSMUSG00000045625	Pigz	phosphatidylinositol glycan anchor biosynthesis, cla	0.095462
ENSMUSG00000085444	Gm13936	predicted gene 13936 [Source:MGI Symbol;Acc:MG	0.095468
ENSMUSG00000028785	Hpca	hippocalcin [Source:MGI Symbol;Acc:MGI:1336200	0.095473
ENSMUSG00000028364	Tnc	tenascin C [Source:MGI Symbol;Acc:MGI:101922]	0.095491
ENSMUSG00000041380	Htr2c	5-hydroxytryptamine (serotonin) receptor 2C [Sour	0.095538
ENSMUSG00000029632	Ndufa4	NADH dehydrogenase (ubiquinone) 1 alpha subcom	0.095576
ENSMUSG00000096841	Gm25596	predicted gene, 25596 [Source:MGI Symbol;Acc:MG	0.095606
ENSMUSG00000053768	Chchd3	coiled-coil-helix-coiled-coil-helix domain containing	0.095626
ENSMUSG00000081128	Gm13328	predicted gene 13328 [Source:MGI Symbol;Acc:MG	0.095673
ENSMUSG00000005899	Smpd4	sphingomyelin phosphodiesterase 4 [Source:MGI Sy	0.095748
ENSMUSG00000046909	Tefm	transcription elongation factor, mitochondrial [Sou	0.095761
ENSMUSG00000055407	Map6	microtubule-associated protein 6 [Source:MGI Sym	0.095834
ENSMUSG00000053907	Mat2a	methionine adenosyltransferase II, alpha [Source:M	0.09587
ENSMUSG00000089026	Gm25014	predicted gene, 25014 [Source:MGI Symbol;Acc:MG	0.095899
ENSMUSG00000021506	Pitx1	paired-like homeodomain transcription factor 1 [So	0.095938
ENSMUSG00000010721	Lmbr1	limb region 1 [Source:MGI Symbol;Acc:MGI:186174	0.095967
ENSMUSG00000066839	Ecsit	ECSIT homolog (Drosophila) [Source:MGI Symbol;A	0.096012
ENSMUSG00000018765	Fxr2	fragile X mental retardation, autosomal homolog 2	0.09605
ENSMUSG00000067438	Hmx1	H6 homeobox 1 [Source:MGI Symbol;Acc:MGI:1071	0.096087
ENSMUSG00000030539	Sema4b	sema domain, immunoglobulin domain (Ig), transm	0.096257
ENSMUSG00000042541	Shfm1	split hand/foot malformation (ectrodactyly) type 1	0.096266
ENSMUSG00000056076	Eif3b	eukaryotic translation initiation factor 3, subunit B	0.096392
ENSMUSG00000024853	Sf3b2	splicing factor 3b, subunit 2 [Source:MGI Symbol;Ac	0.096452
ENSMUSG00000022914	Brwd1	bromodomain and WD repeat domain containing 1	0.096493
ENSMUSG00000023963	Cyp39a1	cytochrome P450, family 39, subfamily a, polypepti	0.096519

ENSMUSG00000031022	BC051019	cDNA sequence BC051019 [Source:MGI Symbol;Acc	0.096522
ENSMUSG00000031070	Mrgprf	MAS-related GPR, member F [Source:MGI Symbol;A	0.096586
ENSMUSG00000002458	Rgs19	regulator of G-protein signaling 19 [Source:MGI Sym	0.096591
ENSMUSG00000041762	Gpr155	G protein-coupled receptor 155 [Source:MGI Symb	0.096605
ENSMUSG00000035754	Wdr18	WD repeat domain 18 [Source:MGI Symbol;Acc:MG	0.096654
ENSMUSG00000046160	Olig1	oligodendrocyte transcription factor 1 [Source:MGI	0.096668
ENSMUSG00000027399	Il1a	interleukin 1 alpha [Source:MGI Symbol;Acc:MGI:9	0.09669
ENSMUSG00000058587	Tmod3	tropomodulin 3 [Source:MGI Symbol;Acc:MGI:1355	0.096708
ENSMUSG00000080718	Gm8019	predicted gene 8019 [Source:MGI Symbol;Acc:MGI:	0.096781
ENSMUSG00000030842	Lamtor1	late endosomal/lysosomal adaptor, MAPK and MTC	0.096786
ENSMUSG00000061013	Mkx	mohawk homeobox [Source:MGI Symbol;Acc:MGI:	0.096822
ENSMUSG00000037771	Slc32a1	solute carrier family 32 (GABA vesicular transporte	0.096828
ENSMUSG00000019256	Ahr	aryl-hydrocarbon receptor [Source:MGI Symbol;Acc	0.096839
ENSMUSG00000028964	Park7	Parkinson disease (autosomal recessive, early onse	0.096839
ENSMUSG00000045078	Rnf216	ring finger protein 216 [Source:MGI Symbol;Acc:MG	0.096852
ENSMUSG00000020034	Tcp11l2	t-complex 11 (mouse) like 2 [Source:MGI Symbol;A	0.096904
ENSMUSG00000046541	Zfp526	zinc finger protein 526 [Source:MGI Symbol;Acc:MG	0.096957
ENSMUSG00000066152	Slc31a2	solute carrier family 31, member 2 [Source:MGI Sym	0.09729
ENSMUSG00000053158	Fes	feline sarcoma oncogene [Source:MGI Symbol;Acc:	0.097355
ENSMUSG00000032115	Hyou1	hypoxia up-regulated 1 [Source:MGI Symbol;Acc:M	0.097417
ENSMUSG00000010803	Gabra1	gamma-aminobutyric acid (GABA) A receptor, subu	0.097435
ENSMUSG00000035278	Plekhj1	pleckstrin homology domain containing, family J m	0.097452
ENSMUSG00000041703	Zic5	zinc finger protein of the cerebellum 5 [Source:MG	0.09747
ENSMUSG00000049305	Ccdc71	coiled-coil domain containing 71 [Source:MGI Symb	0.097489
ENSMUSG00000025453	Nnt	nicotinamide nucleotide transhydrogenase [Source	0.097535
ENSMUSG00000056752	Dnah9	dynein, axonemal, heavy chain 9 [Source:MGI Symb	0.097549
ENSMUSG00000044528	Tram1l1	translocation associated membrane protein 1-like 1	0.097571
ENSMUSG00000038264	Sema7a	sema domain, immunoglobulin domain (Ig), and GP	0.09758
ENSMUSG00000042558	Adprhl2	ADP-ribosylhydrolase like 2 [Source:MGI Symbol;Ac	0.097649
ENSMUSG00000021285	Ppp1r13b	protein phosphatase 1, regulatory (inhibitor) subun	0.097777
ENSMUSG00000049281	Scn3b	sodium channel, voltage-gated, type III, beta [Sourc	0.097808
ENSMUSG00000035048	Anapc13	anaphase promoting complex subunit 13 [Source:M	0.097886
ENSMUSG00000051648	Kctd19	potassium channel tetramerisation domain contain	0.097902
ENSMUSG00000071679	Zcchc16	zinc finger, CCHC domain containing 16 [Source:MG	0.097906
ENSMUSG00000085465	Gm15347	predicted gene 15347 [Source:MGI Symbol;Acc:MG	0.097956
ENSMUSG00000039427	Alg1	asparagine-linked glycosylation 1 (beta-1,4-mannos	0.097998
ENSMUSG00000034850	Tmem127	transmembrane protein 127 [Source:MGI Symbol;A	0.09801
ENSMUSG00000075467	Dnlz	DNL-type zinc finger [Source:MGI Symbol;Acc:MGI:	0.098109
ENSMUSG00000082487	Gm11628	predicted gene 11628 [Source:MGI Symbol;Acc:MG	0.098144
ENSMUSG00000030111	A2m	alpha-2-macroglobulin [Source:MGI Symbol;Acc:MG	0.098167
ENSMUSG00000028115	Bnipl	BCL2/adenovirus E1B 19kD interacting protein like	0.098259
ENSMUSG00000022876	Samsn1	SAM domain, SH3 domain and nuclear localization	0.098262
ENSMUSG00000085955	Gm12356	predicted gene 12356 [Source:MGI Symbol;Acc:MG	0.098328
ENSMUSG00000043067	Dpy19l1	dpy-19-like 1 (C. elegans) [Source:MGI Symbol;Acc:	0.09834
ENSMUSG00000043068	Fam89a	family with sequence similarity 89, member A [Sou	0.098613
ENSMUSG00000007659	Bcl2l1	BCL2-like 1 [Source:MGI Symbol;Acc:MGI:88139]	0.098667
ENSMUSG00000059401	Mamld1	mastermind-like domain containing 1 [Source:MGI	0.098667

ENSMUSG00000038542	Pcid2	PCI domain containing 2 [Source:MGI Symbol;Acc:M	0.098697
ENSMUSG00000004661	Arid3b	AT rich interactive domain 3B (BRIGHT-like) [Source	0.098708
ENSMUSG00000071657	Bscl2	Bernardinelli-Seip congenital lipodystrophy 2 homo	0.09876
ENSMUSG00000026718	Stam	signal transducing adaptor molecule (SH3 domain a	0.098781
ENSMUSG00000036960	Clca5	chloride channel calcium activated 5 [Source:MGI S	0.09881
ENSMUSG00000036957	Lrfn3	leucine rich repeat and fibronectin type III domain	0.098818
ENSMUSG00000044165	Bcl2l15	BCLl2-like 15 [Source:MGI Symbol;Acc:MGI:268541	0.09885
ENSMUSG00000005625	Psmd4	proteasome (prosome, macropain) 26S subunit, no	0.099024
ENSMUSG00000071035	Gm5499	predicted pseudogene 5499 [Source:MGI Symbol;A	0.099048
ENSMUSG00000089028	Gm25010	predicted gene, 25010 [Source:MGI Symbol;Acc:M	0.099104
ENSMUSG00000039556	Ppp1r3f	protein phosphatase 1, regulatory (inhibitor) subun	0.099114
ENSMUSG00000062203	Gspt1	G1 to S phase transition 1 [Source:MGI Symbol;Acc	0.099122
ENSMUSG00000090048	Gm16317	predicted gene 16317 [Source:MGI Symbol;Acc:MG	0.099122
ENSMUSG00000021144	Mta1	metastasis associated 1 [Source:MGI Symbol;Acc:M	0.099129
ENSMUSG00000087184	Gm11650	predicted gene 11650 [Source:MGI Symbol;Acc:MG	0.099135
ENSMUSG00000081471	Gm14735	predicted gene 14735 [Source:MGI Symbol;Acc:MG	0.099145
ENSMUSG00000032120	C2cd2l	C2 calcium-dependent domain containing 2-like [So	0.099234
ENSMUSG00000017478	Zc3h18	zinc finger CCCH-type containing 18 [Source:MGI Sy	0.099235
ENSMUSG00000079418	Atg4a	autophagy related 4A, cysteine peptidase [Source:M	0.099273
ENSMUSG00000039463	Slc9a8	solute carrier family 9 (sodium/hydrogen exchange	0.099283
ENSMUSG00000038156	Spon1	spondin 1, (f-spondin) extracellular matrix protein [	0.099292
ENSMUSG00000036707	Cab39	calcium binding protein 39 [Source:MGI Symbol;Ac	0.099299
ENSMUSG00000085341	Gm15863	predicted gene 15863 [Source:MGI Symbol;Acc:MG	0.099347
ENSMUSG00000029618	Ocm	oncomodulin [Source:MGI Symbol;Acc:MGI:97401]	0.099455
ENSMUSG00000087606	Gm16121	predicted gene 16121 [Source:MGI Symbol;Acc:MG	0.099484
ENSMUSG00000046961	Gpr156	G protein-coupled receptor 156 [Source:MGI Symb	0.099513
ENSMUSG00000074934	Grem1	gremlin 1 [Source:MGI Symbol;Acc:MGI:1344337]	0.099524
ENSMUSG00000038180	Spag4	sperm associated antigen 4 [Source:MGI Symbol;Ac	0.09953
ENSMUSG00000038500	Prr3	proline-rich polypeptide 3 [Source:MGI Symbol;Acc	0.099584
ENSMUSG00000072214	5-Sep	septin 5 [Source:MGI Symbol;Acc:MGI:1195461]	0.099594
ENSMUSG00000018995	Nars2	asparaginyl-tRNA synthetase 2 (mitochondrial)(puta	0.099625
ENSMUSG00000042097	Zfp239	zinc finger protein 239 [Source:MGI Symbol;Acc:M	0.099654
ENSMUSG00000024211	Grm8	glutamate receptor, metabotropic 8 [Source:MGI S	0.099661
ENSMUSG00000071478	Hist1h2ad	histone cluster 1, H2ad [Source:MGI Symbol;Acc:M	0.099663
ENSMUSG00000020866	Cacna1g	calcium channel, voltage-dependent, T type, alpha	0.099718
ENSMUSG00000038685	Rtel1	regulator of telomere elongation helicase 1 [Source	0.099755
ENSMUSG00000001576	Ergic1	endoplasmic reticulum-golgi intermediate compart	0.099773
ENSMUSG00000054469	Lclat1	lysocardiolipin acyltransferase 1 [Source:MGI Symb	0.099787
ENSMUSG00000031843	Mphosph6	M phase phosphoprotein 6 [Source:MGI Symbol;Ac	0.099793
ENSMUSG00000024045	Akap8	A kinase (PRKA) anchor protein 8 [Source:MGI Sym	0.099795
ENSMUSG00000019810	Fuca2	fucosidase, alpha-L- 2, plasma [Source:MGI Symbol	0.09981
ENSMUSG00000022136	Dnajc3	DnaJ (Hsp40) homolog, subfamily C, member 3 [Sou	0.099823
ENSMUSG00000055235	Wdr86	WD repeat domain 86 [Source:MGI Symbol;Acc:MG	0.09985
ENSMUSG00000030780	BC017158	cDNA sequence BC017158 [Source:MGI Symbol;Acc	0.099888
ENSMUSG00000038332	Sesn1	sestrin 1 [Source:MGI Symbol;Acc:MGI:2155278]	0.099903
ENSMUSG00000030802	Bckdk	branched chain ketoacid dehydrogenase kinase [So	0.09996
ENSMUSG00000031631	4933411K20Rik	RIKEN cDNA 4933411K20 gene [Source:MGI Symbo	0.099984

ENSMUSG00000026842	Abl1	c-abl oncogene 1, non-receptor tyrosine kinase [So	0.10003
ENSMUSG00000036894	Rap2b	RAP2B, member of RAS oncogene family [Source:M	0.10021
ENSMUSG00000096528	G430049J08Rik	RIKEN cDNA G430049J08 gene [Source:MGI Symbo	0.10024
ENSMUSG00000026774	4931423N10Rik	RIKEN cDNA 4931423N10 gene [Source:MGI Symbo	0.10031
ENSMUSG00000041658	Rragb	Ras-related GTP binding B [Source:MGI Symbol;Acc	0.10036
ENSMUSG00000022974	Paxbp1	PAX3 and PAX7 binding protein 1 [Source:MGI Sym	0.1005
ENSMUSG00000032583	Mon1a	MON1 homolog A (yeast) [Source:MGI Symbol;Acc:	0.1006
ENSMUSG00000075271	Ttc30a1	tetratricopeptide repeat domain 30A1 [Source:MGI	0.1006
ENSMUSG00000086200	Gm13659	predicted gene 13659 [Source:MGI Symbol;Acc:MG	0.10061
ENSMUSG00000068290	Ddrgk1	DDRGK domain containing 1 [Source:MGI Symbol;A	0.10062
ENSMUSG00000039069	Mtg2	mitochondrial ribosome associated GTPase 2 [Sour	0.10063
ENSMUSG00000058240	Cryzl1	crystallin, zeta (quinone reductase)-like 1 [Source:N	0.10065
ENSMUSG00000021768	Dusp13	dual specificity phosphatase 13 [Source:MGI Symbo	0.10071
ENSMUSG00000051455	Gm1564	predicted gene 1564 [Source:MGI Symbol;Acc:MGI:	0.10072
ENSMUSG00000081284	Gm13673	predicted gene 13673 [Source:MGI Symbol;Acc:MG	0.10074
ENSMUSG00000063480	Nhp2l1	NHP2 non-histone chromosome protein 2-like 1 (S.	0.10075
ENSMUSG00000095990	Zfp97	zinc finger protein 97 [Source:MGI Symbol;Acc:MGI	0.10076
ENSMUSG00000020260	Pofut2	protein O-fucosyltransferase 2 [Source:MGI Symbo	0.10078
ENSMUSG00000085417	Gm13919	predicted gene 13919 [Source:MGI Symbol;Acc:MG	0.10084
ENSMUSG00000016256	Ctsz	cathepsin Z [Source:MGI Symbol;Acc:MGI:1891190	0.10086
ENSMUSG00000015759	Cnih1	cornichon homolog 1 (Drosophila) [Source:MGI Syn	0.10087
ENSMUSG00000001017	Chtop	chromatin target of PRMT1 [Source:MGI Symbol;Ac	0.1009
ENSMUSG00000000295	Hddc2	HD domain containing 2 [Source:MGI Symbol;Acc:N	0.10096
ENSMUSG00000034445	Cyb561a3	cytochrome b561 family, member A3 [Source:MGI	0.10096
ENSMUSG00000028634	Hivep3	human immunodeficiency virus type I enhancer bin	0.10101
ENSMUSG00000085845	Gm13944	predicted gene 13944 [Source:MGI Symbol;Acc:MG	0.10101
ENSMUSG00000026679	Enkur	enkurin, TRPC channel interacting protein [Source:I	0.10107
ENSMUSG00000003346	Abhd17a	abhydrolase domain containing 17A [Source:MGI S	0.10118
ENSMUSG00000031789	Cngb1	cyclic nucleotide gated channel beta 1 [Source:MGI	0.10132
ENSMUSG00000020627	Klhl29	kelch-like 29 [Source:MGI Symbol;Acc:MGI:268385	0.10135
ENSMUSG00000095654	Plscr5	phospholipid scramblase family, member 5 [Source	0.10143
ENSMUSG00000039741	Bahcc1	BAH domain and coiled-coil containing 1 [Source:M	0.10144
ENSMUSG00000030560	Ctsc	cathepsin C [Source:MGI Symbol;Acc:MGI:109553]	0.10148
ENSMUSG00000038917	3930402G23Rik	RIKEN cDNA 3930402G23 gene [Source:MGI Symbo	0.10148
ENSMUSG00000092086	Gm6793	predicted gene 6793 [Source:MGI Symbol;Acc:MGI:	0.10148
ENSMUSG00000032590	Apeh	acylpeptide hydrolase [Source:MGI Symbol;Acc:MG	0.10153
ENSMUSG00000004071	Cdip1	cell death inducing Trp53 target 1 [Source:MGI Sym	0.10155
ENSMUSG00000014426	Map3k4	mitogen-activated protein kinase kinase kinase 4 [S	0.1016
ENSMUSG00000033152	Podxl2	podocalyxin-like 2 [Source:MGI Symbol;Acc:MGI:24	0.1017
ENSMUSG00000000126	Wnt9a	wingless-type MMTV integration site 9A [Source:M	0.10186
ENSMUSG00000001436	Slc19a1	solute carrier family 19 (folate transporter), membe	0.10195
ENSMUSG00000038208	Pgap3	post-GPI attachment to proteins 3 [Source:MGI Syn	0.10195
ENSMUSG00000041889	Shisa4	shisa homolog 4 (Xenopus laevis) [Source:MGI Sym	0.10197
ENSMUSG00000036636	Clcn7	chloride channel 7 [Source:MGI Symbol;Acc:MGI:13	0.102
ENSMUSG00000034384	Barhl2	BarH-like 2 (Drosophila) [Source:MGI Symbol;Acc:M	0.10203
ENSMUSG00000003190	Bcl2l12	BCL2-like 12 (proline rich) [Source:MGI Symbol;Acc	0.10204
ENSMUSG00000018648	Dusp14	dual specificity phosphatase 14 [Source:MGI Symbo	0.10207

ENSMUSG00000094777	Hist1h2ap	histone cluster 1, H2ap [Source:MGI Symbol;Acc:M	0.10208
ENSMUSG00000042628	Zfyve1	zinc finger, FYVE domain containing 1 [Source:MGI	0.1021
ENSMUSG00000052563	D930048N14Rik	RIKEN cDNA D930048N14 gene [Source:MGI Symbo	0.10213
ENSMUSG00000060678	Hist1h4c	histone cluster 1, H4c [Source:MGI Symbol;Acc:MG	0.10229
ENSMUSG00000009035	Tmem184b	transmembrane protein 184b [Source:MGI Symbol;	0.10232
ENSMUSG00000027612	Mmp24	matrix metallopeptidase 24 [Source:MGI Symbol;Ac	0.10234
ENSMUSG00000082587	Gm12129	predicted gene 12129 [Source:MGI Symbol;Acc:MG	0.10235
ENSMUSG00000003039	Fam32a	family with sequence similarity 32, member A [Sou	0.10247
ENSMUSG00000081557	Gm5697	predicted gene 5697 [Source:MGI Symbol;Acc:MGI:	0.10247
ENSMUSG00000070866	Zfp804a	zinc finger protein 804A [Source:MGI Symbol;Acc:M	0.10248
ENSMUSG00000044348	Slc25a53	solute carrier family 25, member 53 [Source:MGI Sy	0.10249
ENSMUSG00000098240	Gm4575	predicted gene 4575 [Source:MGI Symbol;Acc:MGI:	0.10253
ENSMUSG00000038304	Cd160	CD160 antigen [Source:MGI Symbol;Acc:MGI:18603	0.1026
ENSMUSG00000095047	Gm22852	predicted gene, 22852 [Source:MGI Symbol;Acc:MG	0.10268
ENSMUSG00000020441	2310033P09Rik	RIKEN cDNA 2310033P09 gene [Source:MGI Symbo	0.10269
ENSMUSG00000090262	Mpv17	MpV17 mitochondrial inner membrane protein [So	0.10269
ENSMUSG00000072845	Tmprss11a	transmembrane protease, serine 11a [Source:MGI S	0.1027
ENSMUSG00000025898	Cwf19l2	CWF19-like 2, cell cycle control (S. pombe) [Source:	0.10272
ENSMUSG00000005514	Por	P450 (cytochrome) oxidoreductase [Source:MGI Sy	0.10273
ENSMUSG00000032513	Gorasp1	golgi reassembly stacking protein 1 [Source:MGI Sy	0.10276
ENSMUSG00000086655	C330018A13Rik	RIKEN cDNA C330018A13 gene [Source:MGI Symbo	0.10279
ENSMUSG00000040612	Ildr2	immunoglobulin-like domain containing receptor 2	0.10284
ENSMUSG00000074359	Psg23	pregnancy-specific glycoprotein 23 [Source:MGI Sy	0.10287
ENSMUSG00000087434	Gm11202	predicted gene 11202 [Source:MGI Symbol;Acc:MG	0.10292
ENSMUSG00000029198	Grpel1	GrpE-like 1, mitochondrial [Source:MGI Symbol;Acc	0.10295
ENSMUSG00000071359	Tbpl1	TATA box binding protein-like 1 [Source:MGI Symbo	0.10295
ENSMUSG00000057649	Brd9	bromodomain containing 9 [Source:MGI Symbol;Ac	0.103
ENSMUSG00000020676	Ccl11	chemokine (C-C motif) ligand 11 [Source:MGI Symb	0.10311
ENSMUSG00000029610	Aimp2	aminoacyl tRNA synthetase complex-interacting mu	0.10314
ENSMUSG00000097158	Gm26516	predicted gene, 26516 [Source:MGI Symbol;Acc:MG	0.10317
ENSMUSG00000091938	Gm2564	predicted gene 2564 [Source:MGI Symbol;Acc:MGI:	0.10331
ENSMUSG00000078128	Gm2178	predicted gene 2178 [Source:MGI Symbol;Acc:MGI:	0.10332
ENSMUSG00000087545	Gm13966	predicted gene 13966 [Source:MGI Symbol;Acc:MG	0.10339
ENSMUSG00000083420	Gm15665	predicted gene 15665 [Source:MGI Symbol;Acc:MG	0.10344
ENSMUSG00000087525	Gm13062	predicted gene 13062 [Source:MGI Symbol;Acc:MG	0.10344
ENSMUSG00000039164	Naif1	nuclear apoptosis inducing factor 1 [Source:MGI Sy	0.10347
ENSMUSG00000027597	Ahcy	S-adenosylhomocysteine hydrolase [Source:MGI Sy	0.1035
ENSMUSG00000030272	Camk1	calcium/calmodulin-dependent protein kinase I [So	0.10364
ENSMUSG00000003469	Phyhip	phytanoyl-CoA hydroxylase interacting protein [Sou	0.10371
ENSMUSG00000030087	Klf15	Kruppel-like factor 15 [Source:MGI Symbol;Acc:MG	0.10374
ENSMUSG00000047044	D030056L22Rik	RIKEN cDNA D030056L22 gene [Source:MGI Symbo	0.10384
ENSMUSG00000053063	Clec12a	C-type lectin domain family 12, member a [Source:	0.1039
ENSMUSG00000021583	Erap1	endoplasmic reticulum aminopeptidase 1 [Source:M	0.10392
ENSMUSG00000096549	Prickle4	prickle homolog 4 (Drosophila) [Source:MGI Symbo	0.10392
ENSMUSG00000050550	Gm11868	predicted gene 11868 [Source:MGI Symbol;Acc:MG	0.10403
ENSMUSG00000029843	Slc13a4	solute carrier family 13 (sodium/sulfate symporters	0.10408
ENSMUSG00000066721	Zfp575	zinc finger protein 575 [Source:MGI Symbol;Acc:MG	0.10408

ENSMUSG00000033006	Sox10	SRY-box containing gene 10 [Source:MGI Symbol;A	0.10422
ENSMUSG00000060121	Gemin2	gem (nuclear organelle) associated protein 2 [Sourc	0.10422
ENSMUSG00000001542	Ell2	elongation factor RNA polymerase II 2 [Source:MGI	0.10439
ENSMUSG00000050503	Fbxl22	F-box and leucine-rich repeat protein 22 [Source:M	0.10442
ENSMUSG00000037692	Ahdc1	AT hook, DNA binding motif, containing 1 [Source:N	0.10452
ENSMUSG00000039985	Fam60a	family with sequence similarity 60, member A [Sour	0.10456
ENSMUSG00000037315	Phf16	PHD finger protein 16 [Source:MGI Symbol;Acc:MG	0.10459
ENSMUSG00000065097	Snora16a	small nucleolar RNA, H/ACA box 16A [Source:MGI S	0.1046
ENSMUSG00000025218	Poll	polymerase (DNA directed), lambda [Source:MGI Sy	0.10463
ENSMUSG00000028833	Ncdn	neurochondrin [Source:MGI Symbol;Acc:MGI:1347	0.10463
ENSMUSG00000097536	2610037D02Rik	RIKEN cDNA 2610037D02 gene [Source:MGI Symbo	0.10477
ENSMUSG00000054008	Ndst1	N-deacetylase/N-sulfotransferase (heparan glucosa	0.10478
ENSMUSG00000052468	Pmp2	peripheral myelin protein 2 [Source:MGI Symbol;A	0.10497
ENSMUSG00000069792	Wfdc17	WAP four-disulfide core domain 17 [Source:MGI Sy	0.10499
ENSMUSG00000003309	Ap1m2	adaptor protein complex AP-1, mu 2 subunit [Sourc	0.10513
ENSMUSG00000025423	Pias2	protein inhibitor of activated STAT 2 [Source:MGI S	0.10517
ENSMUSG00000031385	Plxnb3	plexin B3 [Source:MGI Symbol;Acc:MGI:2154240]	0.10519
ENSMUSG00000006498	Ptbp1	polypyrimidine tract binding protein 1 [Source:MGI	0.10536
ENSMUSG00000025956	Mettl21a	methyltransferase like 21A [Source:MGI Symbol;Ac	0.10536
ENSMUSG00000005124	Wisp1	WNT1 inducible signaling pathway protein 1 [Sourc	0.10537
ENSMUSG00000048647	Exd1	exonuclease 3'-5' domain containing 1 [Source:MG	0.10541
ENSMUSG00000010154	Spire2	spire homolog 2 (Drosophila) [Source:MGI Symbol;	0.10542
ENSMUSG00000052712	BC004004	cDNA sequence BC004004 [Source:MGI Symbol;Ac	0.10551
ENSMUSG00000087086	Gm13269	predicted gene 13269 [Source:MGI Symbol;Acc:MG	0.10552
ENSMUSG00000039217	Il18	interleukin 18 [Source:MGI Symbol;Acc:MGI:10793	0.10557
ENSMUSG00000041112	Elmo1	engulfment and cell motility 1 [Source:MGI Symbol	0.10571
ENSMUSG00000046110	Serinc4	serine incorporator 4 [Source:MGI Symbol;Acc:MGI	0.10574
ENSMUSG00000028066	Pmf1	polyamine-modulated factor 1 [Source:MGI Symbo	0.10575
ENSMUSG00000044813	Shb	src homology 2 domain-containing transforming pr	0.10577
ENSMUSG00000025480	Syce1	synaptonemal complex central element protein 1 [	0.10579
ENSMUSG00000032867	Fbxw8	F-box and WD-40 domain protein 8 [Source:MGI Sy	0.10581
ENSMUSG00000057342	Sphk2	sphingosine kinase 2 [Source:MGI Symbol;Acc:MGI	0.10581
ENSMUSG00000063713	Klk1b24	kallikrein 1-related peptidase b24 [Source:MGI Sym	0.10581
ENSMUSG00000056268	Dennd1b	DENN/MADD domain containing 1B [Source:MGI Sy	0.10589
ENSMUSG00000064563	Snora7a	small nucleolar RNA, H/ACA box 7A [Source:MGI Sy	0.10599
ENSMUSG00000040446	Rprd1a	regulation of nuclear pre-mRNA domain containing	0.106
ENSMUSG00000026201	Stk16	serine/threonine kinase 16 [Source:MGI Symbol;Ac	0.10604
ENSMUSG00000085539	Gm15561	predicted gene 15561 [Source:MGI Symbol;Acc:MG	0.10606
ENSMUSG00000081719	Gm13726	predicted gene 13726 [Source:MGI Symbol;Acc:MG	0.10607
ENSMUSG00000024459	H2-M5	histocompatibility 2, M region locus 5 [Source:MGI	0.10611
ENSMUSG00000026073	Il1r2	interleukin 1 receptor, type II [Source:MGI Symbol;	0.10619
ENSMUSG00000050628	Ubald2	UBA-like domain containing 2 [Source:MGI Symbol;	0.10626
ENSMUSG00000029086	Prom1	prominin 1 [Source:MGI Symbol;Acc:MGI:1100886]	0.10643
ENSMUSG00000032254	Kif23	kinesin family member 23 [Source:MGI Symbol;Acc	0.10647
ENSMUSG00000058444	Map2k5	mitogen-activated protein kinase kinase 5 [Source:	0.10648
ENSMUSG00000071234	Syndig1l	synapse differentiation inducing 1 like [Source:MGI	0.10654
ENSMUSG00000043872	Zmym1	zinc finger, MYM domain containing 1 [Source:MGI	0.1067

ENSMUSG00000018040	Rrp7a	ribosomal RNA processing 7 homolog A (S. cerevisia	0.10677
ENSMUSG00000028958	Tmub1	transmembrane and ubiquitin-like domain containi	0.10687
ENSMUSG00000040591	1110051M20Rik	RIKEN cDNA 1110051M20 gene [Source:MGI Symb	0.1069
ENSMUSG00000082183	Gm15994	predicted gene 15994 [Source:MGI Symbol;Acc:MG	0.10694
ENSMUSG00000084860	2610204G07Rik	RIKEN cDNA 2610204G07 gene [Source:MGI Symbo	0.107
ENSMUSG00000002981	Clptm1	cleft lip and palate associated transmembrane prot	0.10702
ENSMUSG00000045752	Tssc4	tumor-suppressing subchromosomal transferable f	0.10712
ENSMUSG00000079645	Gm17193	predicted gene 17193 [Source:MGI Symbol;Acc:MG	0.10714
ENSMUSG00000057406	Whsc1	Wolf-Hirschhorn syndrome candidate 1 (human) [S	0.10715
ENSMUSG00000040420	Cdh18	cadherin 18 [Source:MGI Symbol;Acc:MGI:1344366	0.1072
ENSMUSG00000097470	Gm26587	predicted gene, 26587 [Source:MGI Symbol;Acc:M	0.1072
ENSMUSG00000030706	Mrpl48	mitochondrial ribosomal protein L48 [Source:MGI S	0.10723
ENSMUSG00000044080	S100a1	S100 calcium binding protein A1 [Source:MGI Symb	0.10728
ENSMUSG00000044320	1700001O22Rik	RIKEN cDNA 1700001O22 gene [Source:MGI Symbo	0.1073
ENSMUSG00000097759	Gm26753	predicted gene, 26753 [Source:MGI Symbol;Acc:M	0.10735
ENSMUSG00000043782	Ccdc64b	coiled-coil domain containing 64B [Source:MGI Sym	0.10738
ENSMUSG00000020246	Hcfc2	host cell factor C2 [Source:MGI Symbol;Acc:MGI:19	0.1074
ENSMUSG00000035397	Klf16	Kruppel-like factor 16 [Source:MGI Symbol;Acc:MG	0.10743
ENSMUSG00000072812	Ahnak2	AHNAK nucleoprotein 2 [Source:MGI Symbol;Acc:M	0.10743
ENSMUSG00000036599	Chst12	carbohydrate sulfotransferase 12 [Source:MGI Sym	0.1075
ENSMUSG00000040006	Ginm1	glycoprotein integral membrane 1 [Source:MGI Sym	0.10751
ENSMUSG00000000976	Heatr6	HEAT repeat containing 6 [Source:MGI Symbol;Acc:	0.10752
ENSMUSG00000039501	Znfx1	zinc finger, NFX1-type containing 1 [Source:MGI Sy	0.10752
ENSMUSG00000022416	Cacna1i	calcium channel, voltage-dependent, alpha 1I subu	0.10756
ENSMUSG00000029151	Slc30a3	solute carrier family 30 (zinc transporter), member	0.10759
ENSMUSG00000010825	Grid2ip	glutamate receptor, ionotropic, delta 2 (Grid2) inte	0.10765
ENSMUSG00000022935	Grik1	glutamate receptor, ionotropic, kainate 1 [Source:N	0.1077
ENSMUSG00000009073	Nf2	neurofibromatosis 2 [Source:MGI Symbol;Acc:MGI:	0.10773
ENSMUSG00000033182	Kbtbd12	kelch repeat and BTB (POZ) domain containing 12 [	0.10775
ENSMUSG00000041355	Ssr2	signal sequence receptor, beta [Source:MGI Symbo	0.10775
ENSMUSG00000037791	Phf12	PHD finger protein 12 [Source:MGI Symbol;Acc:MG	0.10776
ENSMUSG00000029499	Pxmp2	peroxisomal membrane protein 2 [Source:MGI Sym	0.10781
ENSMUSG00000048138	Dmrt2	doublesex and mab-3 related transcription factor 2	0.10783
ENSMUSG00000040283	Btnl9	butyrophilin-like 9 [Source:MGI Symbol;Acc:MGI:24	0.10794
ENSMUSG00000048721	Fndc9	fibronectin type III domain containing 9 [Source:M	0.10797
ENSMUSG00000021939	Ctsb	cathepsin B [Source:MGI Symbol;Acc:MGI:88561]	0.10799
ENSMUSG00000045318	Adra2c	adrenergic receptor, alpha 2c [Source:MGI Symbol;	0.10805
ENSMUSG00000020070	Rufy2	RUN and FYVE domain-containing 2 [Source:MGI Sy	0.10813
ENSMUSG00000093514	Gm9320	predicted gene 9320 [Source:MGI Symbol;Acc:MGI:	0.10813
ENSMUSG00000026889	Rbm18	RNA binding motif protein 18 [Source:MGI Symbol;	0.10826
ENSMUSG00000058400	Qrfpr	pyroglutamylated RFamide peptide receptor [Sourc	0.10827
ENSMUSG00000085134	Gm11659	predicted gene 11659 [Source:MGI Symbol;Acc:MG	0.10836
ENSMUSG00000086715	B230112J18Rik	RIKEN cDNA B230112J18 gene [Source:MGI Symbol	0.10836
ENSMUSG00000028843	Sh3bgrl3	SH3 domain binding glutamic acid-rich protein-like	0.10838
ENSMUSG00000053199	Arhgap20	Rho GTPase activating protein 20 [Source:MGI Sym	0.10845
ENSMUSG00000078302	Foxd1	forkhead box D1 [Source:MGI Symbol;Acc:MGI:134	0.10848
ENSMUSG00000038387	Rras	Harvey rat sarcoma oncogene, subgroup R [Source:	0.10849

ENSMUSG00000097025	Gm26558	predicted gene, 26558 [Source:MGI Symbol;Acc:M(	0.10849
ENSMUSG00000056763	Cspp1	centrosome and spindle pole associated protein 1 [	0.10853
ENSMUSG00000036820	Amdhd2	amidohydrolase domain containing 2 [Source:MGI S	0.10854
ENSMUSG00000033882	Rbm46	RNA binding motif protein 46 [Source:MGI Symbol;	0.10856
ENSMUSG00000071604	Fam189a2	family with sequence similarity 189, member A2 [S(	0.10857
ENSMUSG00000083826	Gm13039	predicted gene 13039 [Source:MGI Symbol;Acc:MG	0.10864
ENSMUSG00000027950	Chrnb2	cholinergic receptor, nicotinic, beta polypeptide 2 (	0.1087
ENSMUSG00000002679	Med6	mediator of RNA polymerase II transcription, subur	0.10875
ENSMUSG00000026179	Pnkd	paroxysmal nonkinesiogenic dyskinesia [Source:MG	0.10881
ENSMUSG00000020601	Trib2	tribbles homolog 2 (Drosophila) [Source:MGI Symb	0.10893
ENSMUSG00000017221	Psmd3	proteasome (prosome, macropain) 26S subunit, no	0.10898
ENSMUSG00000028070	Apoa1bp	apolipoprotein A-I binding protein [Source:MGI Syn	0.10898
ENSMUSG00000093226	Gm25749	predicted gene, 25749 [Source:MGI Symbol;Acc:M(	0.10902
ENSMUSG00000059040	Gm5506	predicted gene 5506 [Source:MGI Symbol;Acc:MGI:	0.10903
ENSMUSG00000029438	Bcl7a	B cell CLL/lymphoma 7A [Source:MGI Symbol;Acc:M	0.10909
ENSMUSG00000037876	Jmjd1c	jumonji domain containing 1C [Source:MGI Symbol	0.10923
ENSMUSG00000097099	Gm9917	predicted gene 9917 [Source:MGI Symbol;Acc:MGI:	0.10926
ENSMUSG00000016319	Slc25a5	solute carrier family 25 (mitochondrial carrier, aden	0.1094
ENSMUSG00000077416	Gm23747	predicted gene, 23747 [Source:MGI Symbol;Acc:M(	0.1094
ENSMUSG00000047678	Gpr82	G protein-coupled receptor 82 [Source:MGI Symbo	0.10943
ENSMUSG00000021999	Cpb2	carboxypeptidase B2 (plasma) [Source:MGI Symbol	0.10951
ENSMUSG00000094935	Gm9726	predicted gene 9726 [Source:MGI Symbol;Acc:MGI:	0.10965
ENSMUSG00000025260	Hsd17b10	hydroxysteroid (17-beta) dehydrogenase 10 [Sourc	0.10966
ENSMUSG00000029298	Gbp9	guanylate-binding protein 9 [Source:MGI Symbol;A	0.10983
ENSMUSG00000096948	Gm4221	predicted gene 4221 [Source:MGI Symbol;Acc:MGI:	0.10983
ENSMUSG00000033904	Ccp110	centriolar coiled coil protein 110 [Source:MGI Symb	0.10984
ENSMUSG00000091844	Gm8251	predicted gene 8251 [Source:MGI Symbol;Acc:MGI:	0.10988
ENSMUSG00000044997	E130304I02Rik	RIKEN cDNA E130304I02 gene [Source:MGI Symbol	0.10989
ENSMUSG00000034867	Ankrd27	ankyrin repeat domain 27 (VPS9 domain) [Source:M	0.10991
ENSMUSG00000012609	Ttll5	tubulin tyrosine ligase-like family, member 5 [Sourc	0.10992
ENSMUSG00000022860	Chodl	chondrolectin [Source:MGI Symbol;Acc:MGI:21790	0.10995
ENSMUSG00000031021	Tmem9b	TMEM9 domain family, member B [Source:MGI Syn	0.10998
ENSMUSG00000050244	Heatr1	HEAT repeat containing 1 [Source:MGI Symbol;Acc:	0.10999
ENSMUSG00000004933	Matk	megakaryocyte-associated tyrosine kinase [Source:	0.11
ENSMUSG00000024335	Brd2	bromodomain containing 2 [Source:MGI Symbol;Ac	0.11014
ENSMUSG00000008307	1700109H08Rik	RIKEN cDNA 1700109H08 gene [Source:MGI Symbo	0.11015
ENSMUSG00000029920	Smarcad1	SWI/SNF-related, matrix-associated actin-depender	0.11026
ENSMUSG00000029782	Tmem209	transmembrane protein 209 [Source:MGI Symbol;A	0.11029
ENSMUSG00000029505	Ep400	E1A binding protein p400 [Source:MGI Symbol;Acc:	0.11038
ENSMUSG00000036867	Smad6	SMAD family member 6 [Source:MGI Symbol;Acc:M	0.11066
ENSMUSG00000024353	Mzb1	marginal zone B and B1 cell-specific protein 1 [Sour	0.11074
ENSMUSG00000020982	Nemf	nuclear export mediator factor [Source:MGI Symbo	0.1108
ENSMUSG00000007594	Hapln4	hyaluronan and proteoglycan link protein 4 [Source	0.11085
ENSMUSG00000095687	Rnaset2a	ribonuclease T2A [Source:MGI Symbol;Acc:MGI:191	0.11089
ENSMUSG00000020017	Hal	histidine ammonia lyase [Source:MGI Symbol;Acc:M	0.1109
ENSMUSG00000047126	Cltc	clathrin, heavy polypeptide (Hc) [Source:MGI Symb	0.11113
ENSMUSG00000071089	Trim75	tripartite motif-containing 75 [Source:MGI Symbol;	0.11117

ENSMUSG00000028979	Masp2	mannan-binding lectin serine peptidase 2 [Source:N	0.11121
ENSMUSG00000022762	Ncam2	neural cell adhesion molecule 2 [Source:MGI Symb	0.11123
ENSMUSG00000037326	Capn15	calpain 15 [Source:MGI Symbol;Acc:MGI:1355075]	0.11127
ENSMUSG00000097029	Gm26560	predicted gene, 26560 [Source:MGI Symbol;Acc:MC	0.11129
ENSMUSG00000071256	Zfp213	zinc finger protein 213 [Source:MGI Symbol;Acc:MC	0.11134
ENSMUSG00000030591	Psmd8	proteasome (prosome, macropain) 26S subunit, no	0.11136
ENSMUSG00000090243	Gm16103	predicted gene 16103 [Source:MGI Symbol;Acc:MG	0.11136
ENSMUSG00000013236	Ptprs	protein tyrosine phosphatase, receptor type, S [Sou	0.11138
ENSMUSG00000036040	Adamtsl2	ADAMTS-like 2 [Source:MGI Symbol;Acc:MGI:1925(	0.11144
ENSMUSG00000030717	Nupr1	nuclear protein transcription regulator 1 [Source:M	0.11146
ENSMUSG00000032185	Carm1	coactivator-associated arginine methyltransferase 1	0.11146
ENSMUSG00000028713	Cyp4b1	cytochrome P450, family 4, subfamily b, polypeptid	0.11147
ENSMUSG00000067889	Sptbn2	spectrin beta, non-erythrocytic 2 [Source:MGI Sym	0.11149
ENSMUSG00000047264	Zfp358	zinc finger protein 358 [Source:MGI Symbol;Acc:MC	0.11156
ENSMUSG00000074277	Phldb3	pleckstrin homology-like domain, family B, membe	0.11161
ENSMUSG00000098201	Gm26983	predicted gene, 26983 [Source:MGI Symbol;Acc:MC	0.11164
ENSMUSG00000021252	0610007P14Rik	RIKEN cDNA 0610007P14 gene [Source:MGI Symbo	0.11167
ENSMUSG00000027015	Cybrd1	cytochrome b reductase 1 [Source:MGI Symbol;Acc	0.11172
ENSMUSG00000019302	Atp6v0a1	ATPase, H+ transporting, lysosomal V0 subunit A1 [	0.11179
ENSMUSG00000029202	Pds5a	PDS5, regulator of cohesion maintenance, homolog	0.1118
ENSMUSG00000007107	Atp1a4	ATPase, Na+/K+ transporting, alpha 4 polypeptide [	0.11182
ENSMUSG00000026819	Slc25a25	solute carrier family 25 (mitochondrial carrier, phos	0.11193
ENSMUSG00000042737	Dpm3	dolichyl-phosphate mannosyltransferase polypepti	0.11195
ENSMUSG00000023328	Ache	acetylcholinesterase [Source:MGI Symbol;Acc:MGI:	0.11201
ENSMUSG00000054414	Slc30a7	solute carrier family 30 (zinc transporter), member	0.11203
ENSMUSG00000052160	Pld4	phospholipase D family, member 4 [Source:MGI Sy	0.11207
ENSMUSG00000043162	Pyurf	Pigy upstream reading frame [Source:MGI Symbol;/	0.11212
ENSMUSG00000078952	Gm2373	predicted gene 2373 [Source:MGI Symbol;Acc:MGI:	0.11214
ENSMUSG00000014075	Tctex1d2	Tctex1 domain containing 2 [Source:MGI Symbol;A	0.11217
ENSMUSG00000007817	Zmiz1	zinc finger, MIZ-type containing 1 [Source:MGI Sym	0.11221
ENSMUSG00000032744	Heyl	hairy/enhancer-of-split related with YRPW motif-lik	0.11229
ENSMUSG00000021645	Smn1	survival motor neuron 1 [Source:MGI Symbol;Acc:N	0.11236
ENSMUSG00000051373	Ppapdc3	phosphatidic acid phosphatase type 2 domain cont	0.11236
ENSMUSG00000043623	4933412A08Rik	RIKEN cDNA 4933412A08 gene [Source:MGI Symbo	0.11245
ENSMUSG00000056812	St8sia3	ST8 alpha-N-acetyl-neuraminide alpha-2,8-sialyltra	0.11259
ENSMUSG00000094832	Gm21846	predicted gene, 21846 [Source:MGI Symbol;Acc:MC	0.11267
ENSMUSG00000053716	Dusp7	dual specificity phosphatase 7 [Source:MGI Symbol	0.11282
ENSMUSG00000031652	N4bp1	NEDD4 binding protein 1 [Source:MGI Symbol;Acc:	0.11284
ENSMUSG00000038497	Tmco3	transmembrane and coiled-coil domains 3 [Source:	0.11286
ENSMUSG00000032850	Rnft2	ring finger protein, transmembrane 2 [Source:MGI	0.11291
ENSMUSG00000045039	Megf8	multiple EGF-like-domains 8 [Source:MGI Symbol;A	0.11291
ENSMUSG00000056222	Spock1	sparc/osteonectin, cwcv and kazal-like domains pro	0.11291
ENSMUSG00000068040	Tm9sf4	transmembrane 9 superfamily protein member 4 [S	0.11303
ENSMUSG00000030557	Mef2a	myocyte enhancer factor 2A [Source:MGI Symbol;A	0.11309
ENSMUSG00000070547	Mrgprb1	MAS-related GPR, member B1 [Source:MGI Symbol	0.11311
ENSMUSG00000041528	Rnf123	ring finger protein 123 [Source:MGI Symbol;Acc:MC	0.11316
ENSMUSG00000042747	Krtcap2	keratinocyte associated protein 2 [Source:MGI Sym	0.11316

ENSMUSG00000037124	Trim58	tripartite motif-containing 58 [Source:MGI Symbol;	0.11329
ENSMUSG00000027455	Nsfl1c	NSFL1 (p97) cofactor (p47) [Source:MGI Symbol;Ac	0.11333
ENSMUSG00000045302	Preb	prolactin regulatory element binding [Source:MGI S	0.11337
ENSMUSG00000025132	Arhgdia	Rho GDP dissociation inhibitor (GDI) alpha [Source:	0.11341
ENSMUSG00000025612	Bach1	BTB and CNC homology 1 [Source:MGI Symbol;Acc:	0.11342
ENSMUSG00000031729	Ist1	increased sodium tolerance 1 homolog (yeast) [Sou	0.11343
ENSMUSG00000085160	Gm13617	predicted gene 13617 [Source:MGI Symbol;Acc:MG	0.11343
ENSMUSG00000085653	Gm15179	predicted gene 15179 [Source:MGI Symbol;Acc:MG	0.11345
ENSMUSG00000095556	AC163993.1		0.11353
ENSMUSG00000015790	Surf1	surfeit gene 1 [Source:MGI Symbol;Acc:MGI:98443]	0.11356
ENSMUSG00000067220	Cnga1	cyclic nucleotide gated channel alpha 1 [Source:MG	0.11356
ENSMUSG00000049625	Tifab	TRAF-interacting protein with forkhead-associated	0.11357
ENSMUSG00000036822	Topors	topoisomerase I binding, arginine/serine-rich [Sour	0.11362
ENSMUSG00000040258	Nxph4	neurexophilin 4 [Source:MGI Symbol;Acc:MGI:1336	0.11363
ENSMUSG00000053641	Dennd4a	DENN/MADD domain containing 4A [Source:MGI Sy	0.11365
ENSMUSG00000004849	Ap1s1	adaptor protein complex AP-1, sigma 1 [Source:MG	0.11367
ENSMUSG00000097027	Gm26559	predicted gene, 26559 [Source:MGI Symbol;Acc:MG	0.11376
ENSMUSG00000053553	3110082I17Rik	RIKEN cDNA 3110082I17 gene [Source:MGI Symbol	0.11377
ENSMUSG00000013338	Fer1l4	fer-1-like 4 (C. elegans) [Source:MGI Symbol;Acc:M	0.11379
ENSMUSG00000055172	C1ra	complement component 1, r subcomponent A [Sou	0.1138
ENSMUSG00000013736	Trnt1	tRNA nucleotidyl transferase, CCA-adding, 1 [Sourc	0.11387
ENSMUSG00000042492	Tbc1d10b	TBC1 domain family, member 10b [Source:MGI Sym	0.1139
ENSMUSG00000045010	Gm4779	predicted gene 4779 [Source:MGI Symbol;Acc:MGI:	0.1139
ENSMUSG00000049109	Themis	thymocyte selection associated [Source:MGI Symbo	0.1139
ENSMUSG00000077737	Snord72	small nucleolar RNA, C/D box 72 [Source:MGI Symb	0.1139
ENSMUSG00000029120	Ppp2r2c	protein phosphatase 2 (formerly 2A), regulatory su	0.11394
ENSMUSG00000098062	Gm26931	predicted gene, 26931 [Source:MGI Symbol;Acc:MG	0.11402
ENSMUSG00000092675	Gm25262	predicted gene, 25262 [Source:MGI Symbol;Acc:MG	0.11404
ENSMUSG00000073179	Gm10478	predicted gene 10478 [Source:MGI Symbol;Acc:MG	0.11405
ENSMUSG00000067629	Syngap1	synaptic Ras GTPase activating protein 1 homolog (	0.11408
ENSMUSG00000020482	Ccdc117	coiled-coil domain containing 117 [Source:MGI Sym	0.11416
ENSMUSG00000002233	Rhoc	ras homolog gene family, member C [Source:MGI S	0.11418
ENSMUSG00000087635	Gm13414	predicted gene 13414 [Source:MGI Symbol;Acc:MG	0.11424
ENSMUSG00000034135	Sik3	SIK family kinase 3 [Source:MGI Symbol;Acc:MGI:24	0.11439
ENSMUSG00000019715	Gle1	GLE1 RNA export mediator (yeast) [Source:MGI Syn	0.1144
ENSMUSG00000020821	Kif1c	kinesin family member 1C [Source:MGI Symbol;Acc	0.11448
ENSMUSG00000032782	Cntrob	centrobin, centrosomal BRCA2 interacting protein [	0.11454
ENSMUSG00000085046	Gm11467	predicted gene 11467 [Source:MGI Symbol;Acc:MG	0.11455
ENSMUSG00000026841	Fibcd1	fibrinogen C domain containing 1 [Source:MGI Sym	0.1146
ENSMUSG00000013611	Snx31	sorting nexin 31 [Source:MGI Symbol;Acc:MGI:1913	0.11464
ENSMUSG00000026991	Pkp4	plakophilin 4 [Source:MGI Symbol;Acc:MGI:109281	0.11464
ENSMUSG00000022022	Mtrf1	mitochondrial translational release factor 1 [Source	0.11466
ENSMUSG00000026963	Tmem210	transmembrane protein 210 [Source:MGI Symbol;A	0.11472
ENSMUSG00000087203	Gm13986	predicted gene 13986 [Source:MGI Symbol;Acc:MG	0.11482
ENSMUSG00000090071	Cdk5r2	cyclin-dependent kinase 5, regulatory subunit 2 (p3	0.11491
ENSMUSG00000039254	Pomt1	protein-O-mannosyltransferase 1 [Source:MGI Sym	0.11492
ENSMUSG00000068522	Aard	alanine and arginine rich domain containing protei	0.11492

ENSMUSG00000055322	Tns1	tensin 1 [Source:MGI Symbol;Acc:MGI:104552]	0.11495
ENSMUSG00000088170	Gm25219	predicted gene, 25219 [Source:MGI Symbol;Acc:MG	0.11499
ENSMUSG00000017299	Dnttip1	deoxynucleotidyltransferase, terminal, interacting	0.11505
ENSMUSG00000039206	Daglb	diacylglycerol lipase, beta [Source:MGI Symbol;Acc	0.11511
ENSMUSG00000049086	Bmyc	brain expressed myelocytomatosis oncogene [Sour	0.11517
ENSMUSG00000031432	Prps1	phosphoribosyl pyrophosphate synthetase 1 [Sourc	0.11525
ENSMUSG00000098243	Gm4258	predicted gene 4258 [Source:MGI Symbol;Acc:MGI:	0.11532
ENSMUSG00000039691	Tspan10	tetraspanin 10 [Source:MGI Symbol;Acc:MGI:23847	0.11535
ENSMUSG00000029697	Fezf1	Fez family zinc finger 1 [Source:MGI Symbol;Acc:MG	0.11542
ENSMUSG00000093674	Rpl41	ribosomal protein L41 [Source:MGI Symbol;Acc:MG	0.11542
ENSMUSG00000046841	Ckap4	cytoskeleton-associated protein 4 [Source:MGI Sym	0.11545
ENSMUSG00000025602	Zfp202	zinc finger protein 202 [Source:MGI Symbol;Acc:MG	0.11555
ENSMUSG00000063568	Jazf1	JAZF zinc finger 1 [Source:MGI Symbol;Acc:MGI:214	0.11562
ENSMUSG00000021958	Pinx1	PIN2/TERF1 interacting, telomerase inhibitor 1 [Sou	0.11577
ENSMUSG00000083201	Gm12080	predicted gene 12080 [Source:MGI Symbol;Acc:MG	0.11578
ENSMUSG00000038578	Susd1	sushi domain containing 1 [Source:MGI Symbol;Acc	0.11581
ENSMUSG00000087220	Gm11377	predicted gene 11377 [Source:MGI Symbol;Acc:MG	0.11581
ENSMUSG00000082536	Gm13456	predicted gene 13456 [Source:MGI Symbol;Acc:MG	0.1159
ENSMUSG00000019432	Ddx39b	DEAD (Asp-Glu-Ala-Asp) box polypeptide 39B [Sour	0.11596
ENSMUSG00000042225	Ammecr1	Alport syndrome, mental retardation, midface hypo	0.11599
ENSMUSG00000063296	Tmem117	transmembrane protein 117 [Source:MGI Symbol;A	0.116
ENSMUSG00000035614	Fam179b	family with sequence similarity 179, member B [Sou	0.11601
ENSMUSG00000038729	Akap2	A kinase (PRKA) anchor protein 2 [Source:MGI Symb	0.11611
ENSMUSG00000056220	Pla2g4a	phospholipase A2, group IVA (cytosolic, calcium-de	0.11611
ENSMUSG00000025616	Usp16	ubiquitin specific peptidase 16 [Source:MGI Symbo	0.11624
ENSMUSG00000059981	Taok2	TAO kinase 2 [Source:MGI Symbol;Acc:MGI:191591	0.11632
ENSMUSG00000033022	Cdo1	cysteine dioxygenase 1, cytosolic [Source:MGI Symb	0.11633
ENSMUSG00000097300	Gm26835	predicted gene, 26835 [Source:MGI Symbol;Acc:MG	0.11637
ENSMUSG00000090137	Uba52	ubiquitin A-52 residue ribosomal protein fusion pro	0.11638
ENSMUSG00000035164	Zc3h12c	zinc finger CCCH type containing 12C [Source:MGI S	0.11653
ENSMUSG00000036352	Ubac1	ubiquitin associated domain containing 1 [Source:M	0.11653
ENSMUSG00000007476	Lrrc8a	leucine rich repeat containing 8A [Source:MGI Sym	0.11655
ENSMUSG00000021319	Sfrp4	secreted frizzled-related protein 4 [Source:MGI Syn	0.11658
ENSMUSG00000039717	Ralyl	RALY RNA binding protein-like [Source:MGI Symbol	0.11664
ENSMUSG00000042380	Smim12	small integral membrane protein 12 [Source:MGI Sy	0.11667
ENSMUSG00000051344	Plekhm3	pleckstrin homology domain containing, family M,	0.1168
ENSMUSG00000038879	Nipal2	NIPA-like domain containing 2 [Source:MGI Symbol	0.11685
ENSMUSG00000089682	Bcl2l2	BCL2-like 2 [Source:MGI Symbol;Acc:MGI:108052]	0.11687
ENSMUSG00000095909	Gm10324	predicted gene 10324 [Source:MGI Symbol;Acc:MG	0.11687
ENSMUSG00000025887	Casp12	caspase 12 [Source:MGI Symbol;Acc:MGI:1312922]	0.11691
ENSMUSG00000007721	Ccdc124	coiled-coil domain containing 124 [Source:MGI Sym	0.11692
ENSMUSG00000018697	Aatf	apoptosis antagonizing transcription factor [Source	0.11694
ENSMUSG00000074637	Sox2	SRY-box containing gene 2 [Source:MGI Symbol;Acc	0.11694
ENSMUSG00000025737	Wdr24	WD repeat domain 24 [Source:MGI Symbol;Acc:MG	0.1171
ENSMUSG00000036977	Anapc10	anaphase promoting complex subunit 10 [Source:M	0.11716
ENSMUSG00000040843	Tiprl	TIP41, TOR signalling pathway regulator-like (S. cere	0.11716
ENSMUSG00000073725	Lmbrd1	LMBR1 domain containing 1 [Source:MGI Symbol;A	0.11717

ENSMUSG00000080268	Brms1	breast cancer metastasis-suppressor 1 [Source:MG	0.11718
ENSMUSG00000041453	Rpl21	ribosomal protein L21 [Source:MGI Symbol;Acc:MG	0.11719
ENSMUSG00000040183	Ankrd6	ankyrin repeat domain 6 [Source:MGI Symbol;Acc:M	0.11723
ENSMUSG00000034275	Igsf9b	immunoglobulin superfamily, member 9B [Source:M	0.11724
ENSMUSG00000032387	Rbpms2	RNA binding protein with multiple splicing 2 [Sourc	0.11726
ENSMUSG00000025135	Anapc11	anaphase promoting complex subunit 11 [Source:M	0.11729
ENSMUSG00000022887	Masp1	mannan-binding lectin serine peptidase 1 [Source:M	0.11731
ENSMUSG00000059555	Tor4a	torsin family 4, member A [Source:MGI Symbol;Acc	0.11735
ENSMUSG00000032571	Pik3r4	phosphatidylinositol 3 kinase, regulatory subunit, p	0.11736
ENSMUSG00000085581	Gm12786	predicted gene 12786 [Source:MGI Symbol;Acc:MG	0.11739
ENSMUSG00000035027	Map2k2	mitogen-activated protein kinase kinase 2 [Source:I	0.11749
ENSMUSG00000000384	Tbrg4	transforming growth factor beta regulated gene 4 [	0.1175
ENSMUSG00000022419	Deptor	DEP domain containing MTOR-interacting protein [	0.11754
ENSMUSG00000033763	Mtss1l	metastasis suppressor 1-like [Source:MGI Symbol;A	0.11765
ENSMUSG00000037936	Scarb1	scavenger receptor class B, member 1 [Source:MGI	0.11772
ENSMUSG00000002486	Tchp	trichoplein, keratin filament binding [Source:MGI S	0.11774
ENSMUSG00000047789	Slc38a9	solute carrier family 38, member 9 [Source:MGI Syr	0.11775
ENSMUSG00000041966	Dcaf17	DDB1 and CUL4 associated factor 17 [Source:MGI S	0.11776
ENSMUSG00000068856	Sf3b4	splicing factor 3b, subunit 4 [Source:MGI Symbol;Ac	0.11777
ENSMUSG00000030216	Wbp11	WW domain binding protein 11 [Source:MGI Symbo	0.11787
ENSMUSG00000038893	Fam117a	family with sequence similarity 117, member A [Sou	0.11791
ENSMUSG00000032437	Stt3b	STT3, subunit of the oligosaccharyltransferase com	0.11793
ENSMUSG00000060550	H2-Q7	histocompatibility 2, Q region locus 7 [Source:MGI S	0.11796
ENSMUSG00000060860	Ube2s	ubiquitin-conjugating enzyme E2S [Source:MGI Sym	0.11798
ENSMUSG00000023755	Rhebl1	Ras homolog enriched in brain like 1 [Source:MGI S	0.11799
ENSMUSG00000010054	Tusc2	tumor suppressor candidate 2 [Source:MGI Symbol	0.11817
ENSMUSG00000013846	St3gal1	ST3 beta-galactoside alpha-2,3-sialyltransferase 1 [	0.11823
ENSMUSG00000033538	Casp4	caspase 4, apoptosis-related cysteine peptidase [So	0.1184
ENSMUSG00000045983	Eif4g1	eukaryotic translation initiation factor 4, gamma 1	0.11844
ENSMUSG00000025876	Unc5a	unc-5 homolog A (C. elegans) [Source:MGI Symbol;	0.11845
ENSMUSG00000048603	Gm9828	predicted gene 9828 [Source:MGI Symbol;Acc:MGI	0.11845
ENSMUSG00000049904	Tmem17	transmembrane protein 17 [Source:MGI Symbol;Ac	0.11845
ENSMUSG00000025508	Rplp2	ribosomal protein, large P2 [Source:MGI Symbol;Ac	0.11852
ENSMUSG00000030609	Aen	apoptosis enhancing nuclease [Source:MGI Symbol	0.11853
ENSMUSG00000041642	Kif21b	kinesin family member 21B [Source:MGI Symbol;Ac	0.11861
ENSMUSG00000017686	Rhot1	ras homolog gene family, member T1 [Source:MGI	0.11864
ENSMUSG00000020198	Ap3d1	adaptor-related protein complex 3, delta 1 subunit	0.11864
ENSMUSG00000024911	Fibp	fibroblast growth factor (acidic) intracellular bindin	0.11869
ENSMUSG00000024947	Men1	multiple endocrine neoplasia 1 [Source:MGI Symbo	0.11884
ENSMUSG00000044250	Pced1b	PC-esterase domain containing 1B [Source:MGI Syn	0.1189
ENSMUSG00000066361	Serpina3c	serine (or cysteine) peptidase inhibitor, clade A, me	0.1189
ENSMUSG00000036712	Cyld	cylindromatosis (turban tumor syndrome) [Source:	0.11897
ENSMUSG00000041534	Rbp3	retinol binding protein 3, interstitial [Source:MGI Sy	0.11897
ENSMUSG00000024992	Pde6c	phosphodiesterase 6C, cGMP specific, cone, alpha p	0.11899
ENSMUSG00000020354	Sgcd	sarcoglycan, delta (dystrophin-associated glycoprot	0.119
ENSMUSG00000026090	2010300C02Rik	RIKEN cDNA 2010300C02 gene [Source:MGI Symbo	0.11906
ENSMUSG00000053291	Rab4b	RAB4B, member RAS oncogene family [Source:MGI	0.11909

ENSMUSG00000003721	Insig2	insulin induced gene 2 [Source:MGI Symbol;Acc:MG	0.1192
ENSMUSG00000031584	Gsr	glutathione reductase [Source:MGI Symbol;Acc:MG	0.1193
ENSMUSG00000027636	Sla2	Src-like-adaptor 2 [Source:MGI Symbol;Acc:MGI:19	0.11932
ENSMUSG00000038060	Dlec1	deleted in lung and esophageal cancer 1 [Source:M	0.11938
ENSMUSG00000034041	Lyl1	lymphoblastomic leukemia 1 [Source:MGI Symbol;A	0.11941
ENSMUSG00000015944	Gatsl2	GATS protein-like 2 [Source:MGI Symbol;Acc:MGI:1	0.11951
ENSMUSG00000030678	Maz	MYC-associated zinc finger protein (purine-binding	0.11953
ENSMUSG00000024966	Stip1	stress-induced phosphoprotein 1 [Source:MGI Sym	0.11963
ENSMUSG00000086533	Gm16266	predicted gene 16266 [Source:MGI Symbol;Acc:MG	0.11972
ENSMUSG00000027465	Tbc1d20	TBC1 domain family, member 20 [Source:MGI Symb	0.11973
ENSMUSG00000073096	Lrrc61	leucine rich repeat containing 61 [Source:MGI Symb	0.11974
ENSMUSG00000084496	Gm22514	predicted gene, 22514 [Source:MGI Symbol;Acc:MG	0.11975
ENSMUSG00000030284	Creld1	cysteine-rich with EGF-like domains 1 [Source:MGI	0.11985
ENSMUSG00000022614	Lmf2	lipase maturation factor 2 [Source:MGI Symbol;Acc	0.11987
ENSMUSG00000028758	Kif17	kinesin family member 17 [Source:MGI Symbol;Acc	0.11988
ENSMUSG00000037957	Wdr20a	WD repeat domain 20A [Source:MGI Symbol;Acc:M	0.1199
ENSMUSG00000084325	Gm15550	predicted gene 15550 [Source:MGI Symbol;Acc:MG	0.11992
ENSMUSG00000032338	Hcn4	hyperpolarization-activated, cyclic nucleotide-gated	0.11994
ENSMUSG00000022432	Smc1b	structural maintenance of chromosomes 1B [Source	0.11996
ENSMUSG00000041653	Pnpla3	patatin-like phospholipase domain containing 3 [So	0.12003
ENSMUSG00000070056	Mfhas1	malignant fibrous histiocytoma amplified sequence	0.1201
ENSMUSG00000019054	Fis1	fission 1 (mitochondrial outer membrane) homolog	0.1202
ENSMUSG00000031355	Arhgap6	Rho GTPase activating protein 6 [Source:MGI Symb	0.12028
ENSMUSG00000021057	Akap5	A kinase (PRKA) anchor protein 5 [Source:MGI Symb	0.12032
ENSMUSG00000027381	Bcl2l11	BCL2-like 11 (apoptosis facilitator) [Source:MGI Syn	0.12033
ENSMUSG00000074813	Gm14005	predicted gene 14005 [Source:MGI Symbol;Acc:MG	0.12036
ENSMUSG00000089695	Gm15946	predicted gene 15946 [Source:MGI Symbol;Acc:MG	0.12042
ENSMUSG00000038598	AI481877	expressed sequence AI481877 [Source:MGI Symbol	0.12043
ENSMUSG00000078498	Gm13151	predicted gene 13151 [Source:MGI Symbol;Acc:MG	0.12043
ENSMUSG00000086156	Gm15354	predicted gene 15354 [Source:MGI Symbol;Acc:MG	0.12044
ENSMUSG00000032818	Loxhd1	lipoxygenase homology domains 1 [Source:MGI Syn	0.12045
ENSMUSG00000035521	Gnptg	N-acetylglucosamine-1-phosphotransferase, gamm	0.12045
ENSMUSG00000050423	Ppp1r3g	protein phosphatase 1, regulatory (inhibitor) subun	0.12061
ENSMUSG00000069516	Lyz2	lysozyme 2 [Source:MGI Symbol;Acc:MGI:96897]	0.12063
ENSMUSG00000034621	Gpatch8	G patch domain containing 8 [Source:MGI Symbol;A	0.12077
ENSMUSG00000042308	Setd1a	SET domain containing 1A [Source:MGI Symbol;Acc	0.12086
ENSMUSG00000089876	Tmem102	transmembrane protein 102 [Source:MGI Symbol;A	0.12087
ENSMUSG00000000340	Dbt	dihydrolipoamide branched chain transacylase E2 [	0.12097
ENSMUSG00000029156	Sgcb	sarcoglycan, beta (dystrophin-associated glycoprote	0.12104
ENSMUSG00000090936	Gm17705	predicted gene, 17705 [Source:MGI Symbol;Acc:MG	0.12111
ENSMUSG00000057789	Bak1	BCL2-antagonist/killer 1 [Source:MGI Symbol;Acc:M	0.12116
ENSMUSG00000020929	Eftud2	elongation factor Tu GTP binding domain containin	0.12117
ENSMUSG00000063796	Slc22a8	solute carrier family 22 (organic anion transporter),	0.12117
ENSMUSG00000038342	Mlxip	MLX interacting protein [Source:MGI Symbol;Acc:M	0.12121
ENSMUSG00000036323	Srp72	signal recognition particle 72 [Source:MGI Symbol;A	0.12122
ENSMUSG00000033460	Armcx1	armadillo repeat containing, X-linked 1 [Source:MG	0.12123
ENSMUSG00000041303	Gtf3c3	general transcription factor IIIC, polypeptide 3 [Sou	0.12124

ENSMUSG00000086596	Susd5	sushi domain containing 5 [Source:MGI Symbol;Acc	0.12124
ENSMUSG00000021771	Vdac2	voltage-dependent anion channel 2 [Source:MGI Sy	0.12128
ENSMUSG00000029817	Tra2a	transformer 2 alpha homolog (Drosophila) [Source:	0.12137
ENSMUSG00000040009	Gnaz	guanine nucleotide binding protein, alpha z subunit	0.12137
ENSMUSG00000048100	Taf13	TAF13 RNA polymerase II, TATA box binding protein	0.12139
ENSMUSG00000062070	Pgk1	phosphoglycerate kinase 1 [Source:MGI Symbol;Acc	0.12139
ENSMUSG00000026792	Lrsam1	leucine rich repeat and sterile alpha motif containin	0.12142
ENSMUSG00000021731	Mrps30	mitochondrial ribosomal protein S30 [Source:MGI S	0.12143
ENSMUSG00000018770	Atp5g3	ATP synthase, H+ transporting, mitochondrial F0 co	0.12151
ENSMUSG00000071042	Rasgrp3	RAS, guanyl releasing protein 3 [Source:MGI Symbo	0.12153
ENSMUSG00000021200	Asb2	ankyrin repeat and SOCS box-containing 2 [Source:	0.12156
ENSMUSG00000038412	Higd1a	HIG1 domain family, member 1A [Source:MGI Symb	0.12158
ENSMUSG00000038555	Reep2	receptor accessory protein 2 [Source:MGI Symbol;A	0.12158
ENSMUSG00000042333	Tnfrsf14	tumor necrosis factor receptor superfamily, membe	0.1216
ENSMUSG00000053644	Aldh7a1	aldehyde dehydrogenase family 7, member A1 [Sou	0.12163
ENSMUSG00000025231	Sufu	suppressor of fused homolog (Drosophila) [Source:	0.12166
ENSMUSG00000091542	Gm17167	predicted gene 17167 [Source:MGI Symbol;Acc:MG	0.12168
ENSMUSG00000034254	Agpat1	1-acylglycerol-3-phosphate O-acyltransferase 1 (lys	0.12178
ENSMUSG00000071054	Safb	scaffold attachment factor B [Source:MGI Symbol;A	0.12182
ENSMUSG00000097911	Gm26691	predicted gene, 26691 [Source:MGI Symbol;Acc:MG	0.12182
ENSMUSG00000091511	Vmn2r87	vomeronasal 2, receptor 87 [Source:MGI Symbol;Ac	0.12183
ENSMUSG00000057363	Uxs1	UDP-glucuronate decarboxylase 1 [Source:MGI Sym	0.12184
ENSMUSG00000054074	Skida1	SKI/DACH domain containing 1 [Source:MGI Symbo	0.12187
ENSMUSG00000024897	Apba1	amyloid beta (A4) precursor protein binding, family	0.12192
ENSMUSG00000083789	Gm6092	predicted gene 6092 [Source:MGI Symbol;Acc:MGI:	0.12206
ENSMUSG00000021190	Lgmn	legumain [Source:MGI Symbol;Acc:MGI:1330838]	0.1222
ENSMUSG00000028741	Mrto4	MRT4, mRNA turnover 4, homolog (S. cerevisiae) [S	0.12231
ENSMUSG00000030518	Fam189a1	family with sequence similarity 189, member A1 [So	0.12233
ENSMUSG00000080810	Gm11737	predicted gene 11737 [Source:MGI Symbol;Acc:MG	0.12233
ENSMUSG00000034617	Mtrr	5-methyltetrahydrofolate-homocysteine methyltra	0.12241
ENSMUSG00000035674	Ndufa3	NADH dehydrogenase (ubiquinone) 1 alpha subcom	0.12241
ENSMUSG00000043740	B430306N03Rik	RIKEN cDNA B430306N03 gene [Source:MGI Symbo	0.12241
ENSMUSG00000002812	Flii	flightless I homolog (Drosophila) [Source:MGI Symb	0.12244
ENSMUSG00000027474	Ccm2l	cerebral cavernous malformation 2-like [Source:MG	0.12244
ENSMUSG00000026960	Arl6ip6	ADP-ribosylation factor-like 6 interacting protein 6	0.1225
ENSMUSG00000031510	1700128E19Rik	RIKEN cDNA 1700128E19 gene [Source:MGI Symbo	0.1225
ENSMUSG00000047910	Pcdhb16	protocadherin beta 16 [Source:MGI Symbol;Acc:MG	0.12251
ENSMUSG00000024222	Fkbp5	FK506 binding protein 5 [Source:MGI Symbol;Acc:M	0.12253
ENSMUSG00000025220	Mgea5	meningioma expressed antigen 5 (hyaluronidase) [S	0.12253
ENSMUSG00000051235	Gen1	Gen homolog 1, endonuclease (Drosophila) [Source	0.12255
ENSMUSG00000042292	Mkl1	MKL (megakaryoblastic leukemia)/myocardin-like 1	0.12258
ENSMUSG00000025007	Aldh18a1	aldehyde dehydrogenase 18 family, member A1 [So	0.12262
ENSMUSG00000015478	Rnf5	ring finger protein 5 [Source:MGI Symbol;Acc:MGI:	0.12269
ENSMUSG00000097636	5830416P10Rik	RIKEN cDNA 5830416P10 gene [Source:MGI Symbo	0.12274
ENSMUSG00000097174	Gm4890	predicted gene 4890 [Source:MGI Symbol;Acc:MGI:	0.12282
ENSMUSG00000088493	Gm24401	predicted gene, 24401 [Source:MGI Symbol;Acc:MG	0.12288
ENSMUSG00000021607	Mrpl36	mitochondrial ribosomal protein L36 [Source:MGI S	0.12289

ENSMUSG00000004500	Zfp324	zinc finger protein 324 [Source:MGI Symbol;Acc:MG	0.12299
ENSMUSG00000056128	Gm9991	predicted gene 9991 [Source:MGI Symbol;Acc:MGI:	0.12299
ENSMUSG00000028601	Echdc2	enoyl Coenzyme A hydratase domain containing 2 [	0.12301
ENSMUSG00000012211	Tex22	testis expressed gene 22 [Source:MGI Symbol;Acc:M	0.12302
ENSMUSG00000026678	Rgs5	regulator of G-protein signaling 5 [Source:MGI Sym	0.12307
ENSMUSG00000021483	Cdk20	cyclin-dependent kinase 20 [Source:MGI Symbol;Ac	0.1231
ENSMUSG00000071317	Bves	blood vessel epicardial substance [Source:MGI Sym	0.12314
ENSMUSG00000018569	Cldn7	claudin 7 [Source:MGI Symbol;Acc:MGI:1859285]	0.12315
ENSMUSG00000031358	Msl3	male-specific lethal 3 homolog (Drosophila) [Source	0.12316
ENSMUSG00000022111	Uchl3	ubiquitin carboxyl-terminal esterase L3 (ubiquitin th	0.12325
ENSMUSG00000028555	Ttc39a	tetratricopeptide repeat domain 39A [Source:MGI S	0.12327
ENSMUSG00000023781	Hes7	hairy and enhancer of split 7 (Drosophila) [Source:M	0.12328
ENSMUSG00000036749	Pramel5	preferentially expressed antigen in melanoma like 5	0.1233
ENSMUSG00000021427	Ssr1	signal sequence receptor, alpha [Source:MGI Symb	0.12334
ENSMUSG00000020649	Rrm2	ribonucleotide reductase M2 [Source:MGI Symbol;A	0.12337
ENSMUSG00000024402	Lta	lymphotoxin A [Source:MGI Symbol;Acc:MGI:10479	0.12338
ENSMUSG00000052997	Uba2	ubiquitin-like modifier activating enzyme 2 [Source	0.12347
ENSMUSG00000045659	Plekha7	pleckstrin homology domain containing, family A m	0.12362
ENSMUSG00000076609	Igkc	immunoglobulin kappa constant [Source:MGI Symb	0.12372
ENSMUSG00000001794	Capns1	calpain, small subunit 1 [Source:MGI Symbol;Acc:M	0.12373
ENSMUSG00000041697	Cox6a1	cytochrome c oxidase subunit VIa polypeptide 1 [So	0.12376
ENSMUSG00000028419	Chmp5	charged multivesicular body protein 5 [Source:MGI	0.1238
ENSMUSG00000085772	D630024D03Rik	RIKEN cDNA D630024D03 gene [Source:MGI Symbo	0.1238
ENSMUSG00000033487	Fndc3a	fibronectin type III domain containing 3A [Source:M	0.12386
ENSMUSG00000037989	Wnk2	WNK lysine deficient protein kinase 2 [Source:MGI	0.12391
ENSMUSG00000086283	2810433D01Rik	RIKEN cDNA 2810433D01 gene [Source:MGI Symbo	0.12394
ENSMUSG00000001783	Rtcb	RNA 2',3'-cyclic phosphate and 5'-OH ligase [Source	0.12402
ENSMUSG00000087153	Gm6483	predicted gene 6483 [Source:MGI Symbol;Acc:MGI:	0.12402
ENSMUSG00000029629	Phf14	PHD finger protein 14 [Source:MGI Symbol;Acc:MG	0.12407
ENSMUSG00000022812	Gsk3b	glycogen synthase kinase 3 beta [Source:MGI Symb	0.12421
ENSMUSG00000039987	Phtf2	putative homeodomain transcription factor 2 [Sour	0.12423
ENSMUSG00000037466	4930427A07Rik	RIKEN cDNA 4930427A07 gene [Source:MGI Symbo	0.12425
ENSMUSG00000037552	Plekhg2	pleckstrin homology domain containing, family G (w	0.12434
ENSMUSG00000022075	Rhobtb2	Rho-related BTB domain containing 2 [Source:MGI	0.12441
ENSMUSG00000095845	Gm5741	predicted gene 5741 [Source:MGI Symbol;Acc:MGI:	0.12443
ENSMUSG00000020704	Asic2	acid-sensing (proton-gated) ion channel 2 [Source:M	0.12451
ENSMUSG00000063787	Chchd1	coiled-coil-helix-coiled-coil-helix domain containing	0.12452
ENSMUSG00000020527	Myo19	myosin XIX [Source:MGI Symbol;Acc:MGI:1913446]	0.12454
ENSMUSG00000071655	Ubxn1	UBX domain protein 1 [Source:MGI Symbol;Acc:MG	0.12465
ENSMUSG00000025370	Cdh9	cadherin 9 [Source:MGI Symbol;Acc:MGI:107433]	0.12467
ENSMUSG00000042439	Zfp532	zinc finger protein 532 [Source:MGI Symbol;Acc:MG	0.12468
ENSMUSG00000078875	Gm14419	predicted gene 14419 [Source:MGI Symbol;Acc:MG	0.12471
ENSMUSG00000032548	Slco2a1	solute carrier organic anion transporter family, mer	0.12477
ENSMUSG00000030682	Cdipt	CDP-diacylglycerol--inositol 3-phosphatidyltransfera	0.1248
ENSMUSG00000005089	Slc1a2	solute carrier family 1 (glial high affinity glutamate	0.12483
ENSMUSG00000084930	Gm11706	predicted gene 11706 [Source:MGI Symbol;Acc:MG	0.12483
ENSMUSG00000022055	Nefl	neurofilament, light polypeptide [Source:MGI Symb	0.12488

ENSMUSG00000097835	Gm26910	predicted gene, 26910 [Source:MGI Symbol;Acc:M(	0.12491
ENSMUSG00000063524	Eno1	enolase 1, alpha non-neuron [Source:MGI Symbol;/	0.12507
ENSMUSG00000004665	Cnn2	calponin 2 [Source:MGI Symbol;Acc:MGI:105093]	0.12509
ENSMUSG00000054580	Pla2r1	phospholipase A2 receptor 1 [Source:MGI Symbol;/	0.1251
ENSMUSG00000029407	Uso1	USO1 vesicle docking factor [Source:MGI Symbol;A(	0.12513
ENSMUSG00000040387	Klhl32	kelch-like 32 [Source:MGI Symbol;Acc:MGI:361279(	0.12527
ENSMUSG00000049600	Zbtb45	zinc finger and BTB domain containing 45 [Source:N	0.12547
ENSMUSG00000052229	Gpr17	G protein-coupled receptor 17 [Source:MGI Symbo	0.1255
ENSMUSG00000030960	Mettl10	methyltransferase like 10 [Source:MGI Symbol;Acc:	0.12561
ENSMUSG00000036882	Arhgap33	Rho GTPase activating protein 33 [Source:MGI Sym	0.12562
ENSMUSG00000086040	Wipf3	WAS/WASL interacting protein family, member 3 [S	0.12567
ENSMUSG00000086165	Gm15690	predicted gene 15690 [Source:MGI Symbol;Acc:MG	0.12567
ENSMUSG00000050965	Prkca	protein kinase C, alpha [Source:MGI Symbol;Acc:M(	0.12568
ENSMUSG00000030554	Synm	synemin, intermediate filament protein [Source:M(	0.12569
ENSMUSG00000020703	5530401A14Rik	RIKEN cDNA 5530401A14 gene [Source:MGI Symbo	0.12572
ENSMUSG00000048720	Tbc1d12	TBC1D12: TBC1 domain family, member 12 [Source	0.12577
ENSMUSG00000091383	Hist1h2al	histone cluster 1, H2al [Source:MGI Symbol;Acc:M(	0.12577
ENSMUSG00000082404	Gm14799	predicted gene 14799 [Source:MGI Symbol;Acc:MG	0.12578
ENSMUSG00000000791	Il12rb1	interleukin 12 receptor, beta 1 [Source:MGI Symbo	0.12581
ENSMUSG00000020111	Micu1	mitochondrial calcium uptake 1 [Source:MGI Symb(	0.1259
ENSMUSG00000055926	Gm14137	predicted gene 14137 [Source:MGI Symbol;Acc:MG	0.1259
ENSMUSG00000073058	Gm10463	predicted gene 10463 [Source:MGI Symbol;Acc:MG	0.12593
ENSMUSG00000032714	Syde1	synapse defective 1, Rho GTPase, homolog 1 (C. ele	0.12599
ENSMUSG00000017493	Igfbp4	insulin-like growth factor binding protein 4 [Source	0.12603
ENSMUSG00000059343	Aldoart1	aldolase 1 A, retrogene 1 [Source:MGI Symbol;Acc:	0.12608
ENSMUSG00000022311	Csmd3	CUB and Sushi multiple domains 3 [Source:MGI Syn	0.12609
ENSMUSG00000049176	Frmpd4	FERM and PDZ domain containing 4 [Source:MGI Sy	0.12617
ENSMUSG00000042604	Kcna4	potassium voltage-gated channel, shaker-related su	0.12619
ENSMUSG00000098051	Gm27032	predicted gene, 27032 [Source:MGI Symbol;Acc:M(	0.12625
ENSMUSG00000038274	Fau	Finkel-Biskis-Reilly murine sarcoma virus (FBR-MuS\	0.12626
ENSMUSG00000028248	Sfrs18	serine/arginine-rich splicing factor 18 [Source:MGI	0.12633
ENSMUSG00000034371	Dak	dihydroxyacetone kinase 2 homolog (yeast) [Source	0.1264
ENSMUSG00000034165	Ccnd3	cyclin D3 [Source:MGI Symbol;Acc:MGI:88315]	0.12645
ENSMUSG00000083359	Gm12656	predicted gene 12656 [Source:MGI Symbol;Acc:MG	0.12646
ENSMUSG00000030127	Cops7a	COP9 (constitutive photomorphogenic) homolog, s	0.12655
ENSMUSG00000031165	Was	Wiskott-Aldrich syndrome homolog (human) [Sour(	0.12656
ENSMUSG00000005299	Letm1	leucine zipper-EF-hand containing transmembrane	0.12658
ENSMUSG00000005533	Igf1r	insulin-like growth factor I receptor [Source:MGI Sy	0.12658
ENSMUSG00000044502	Bod1	biorientation of chromosomes in cell division 1 [Sou	0.12658
ENSMUSG00000027944	Hax1	HCLS1 associated X-1 [Source:MGI Symbol;Acc:MGI	0.12667
ENSMUSG00000082558	Snrp1c-ps2	U1 small nuclear ribonucleoprotein 1C, pseudogen(	0.12684
ENSMUSG00000073802	Cdkn2b	cyclin-dependent kinase inhibitor 2B (p15, inhibits (	0.12698
ENSMUSG00000085298	F730035M05Rik	RIKEN cDNA F730035M05 gene [Source:MGI Symb(	0.12701
ENSMUSG00000024424	Ttc39c	tetratricopeptide repeat domain 39C [Source:MGI S	0.12703
ENSMUSG00000044254	Pcsk9	proprotein convertase subtilisin/kexin type 9 [Sour(	0.12703
ENSMUSG00000087625	4930419G24Rik	RIKEN cDNA 4930419G24 gene [Source:MGI Symbo	0.1271
ENSMUSG00000026641	Usf1	upstream transcription factor 1 [Source:MGI Symb(	0.12715

ENSMUSG00000024286	Ccny	cyclin Y [Source:MGI Symbol;Acc:MGI:1915224]	0.12718
ENSMUSG00000038250	Usp38	ubiquitin specific peptidase 38 [Source:MGI Symbo	0.1272
ENSMUSG00000090070	Gm16577	predicted gene 16577 [Source:MGI Symbol;Acc:MG	0.12724
ENSMUSG00000059278	Lsmd1	LSM domain containing 1 [Source:MGI Symbol;Acc:	0.12727
ENSMUSG00000030235	Slco1c1	solute carrier organic anion transporter family, mer	0.12735
ENSMUSG00000019951	Uhrf1bp1l	UHRF1 (ICBP90) binding protein 1-like [Source:MGI	0.12746
ENSMUSG00000074406	Zfp628	zinc finger protein 628 [Source:MGI Symbol;Acc:M(	0.12756
ENSMUSG00000072898	Gm10434	predicted gene 10434 [Source:MGI Symbol;Acc:MG	0.12763
ENSMUSG00000035828	Pim3	proviral integration site 3 [Source:MGI Symbol;Acc:	0.12774
ENSMUSG00000025527	Satl1	spermidine/spermine N1-acetyl transferase-like 1 [	0.1278
ENSMUSG00000020067	Mypn	myopalladin [Source:MGI Symbol;Acc:MGI:191605	0.12787
ENSMUSG00000084828	Gm12367	predicted gene 12367 [Source:MGI Symbol;Acc:MG	0.12788
ENSMUSG00000033200	Tpsg1	tryptase gamma 1 [Source:MGI Symbol;Acc:MGI:13	0.12792
ENSMUSG00000097960	A330074K22Rik	RIKEN cDNA A330074K22 gene [Source:MGI Symbo	0.12792
ENSMUSG00000019791	Hint3	histidine triad nucleotide binding protein 3 [Source	0.12793
ENSMUSG00000026319	2310035C23Rik	RIKEN cDNA 2310035C23 gene [Source:MGI Symbo	0.12794
ENSMUSG00000030306	Tmtc1	transmembrane and tetratricopeptide repeat conta	0.12794
ENSMUSG00000089714	Gm16092	predicted gene 16092 [Source:MGI Symbol;Acc:MG	0.12795
ENSMUSG00000064359	mt-Tg	mitochondrially encoded tRNA glycine [Source:MGI	0.12804
ENSMUSG00000025931	Paqr8	progestin and adipoQ receptor family member VIII	0.12805
ENSMUSG00000092090	Gm3294	predicted gene 3294 [Source:MGI Symbol;Acc:MGI:	0.12813
ENSMUSG00000042178	Armc5	armadillo repeat containing 5 [Source:MGI Symbol;	0.12833
ENSMUSG00000024518	Rax	retina and anterior neural fold homeobox [Source:I	0.12845
ENSMUSG00000030793	Pycard	PYD and CARD domain containing [Source:MGI Sym	0.12847
ENSMUSG00000042535	Gtpbp1	GTP binding protein 1 [Source:MGI Symbol;Acc:MG	0.12847
ENSMUSG00000034751	Mast4	microtubule associated serine/threonine kinase far	0.12848
ENSMUSG00000033430	Terf2ip	telomeric repeat binding factor 2, interacting prote	0.12854
ENSMUSG00000034206	Polq	polymerase (DNA directed), theta [Source:MGI Sym	0.12856
ENSMUSG00000001482	Def8	differentially expressed in FDCP 8 [Source:MGI Sym	0.12861
ENSMUSG00000005575	Ube2m	ubiquitin-conjugating enzyme E2M [Source:MGI Sy	0.12865
ENSMUSG00000032289	Thsd4	thrombospondin, type I, domain containing 4 [Sour	0.12866
ENSMUSG00000034295	Fhod3	formin homology 2 domain containing 3 [Source:M	0.1287
ENSMUSG00000025532	Crcp	calcitonin gene-related peptide-receptor componer	0.12874
ENSMUSG00000090659	Zfp493	zinc finger protein 493 [Source:MGI Symbol;Acc:M(	0.12879
ENSMUSG00000040752	Myh6	myosin, heavy polypeptide 6, cardiac muscle, alpha	0.12895
ENSMUSG00000048582	Gja3	gap junction protein, alpha 3 [Source:MGI Symbol;A	0.12898
ENSMUSG00000058230	Grlf1	glucocorticoid receptor DNA binding factor 1 [Sourc	0.12899
ENSMUSG00000064080	Fbln2	fibulin 2 [Source:MGI Symbol;Acc:MGI:95488]	0.12901
ENSMUSG00000006675	P4htm	prolyl 4-hydroxylase, transmembrane (endoplasmic	0.12903
ENSMUSG00000039041	Adrm1	adhesion regulating molecule 1 [Source:MGI Symbo	0.12904
ENSMUSG00000034333	Zbed4	zinc finger, BED domain containing 4 [Source:MGI S	0.12905
ENSMUSG00000032766	Gng11	guanine nucleotide binding protein (G protein), gar	0.12908
ENSMUSG00000022125	Cln5	ceroid-lipofuscinosis, neuronal 5 [Source:MGI Symb	0.12911
ENSMUSG00000082888	Gm12934	predicted gene 12934 [Source:MGI Symbol;Acc:MG	0.12916
ENSMUSG00000033720	Sfxn5	sideroflexin 5 [Source:MGI Symbol;Acc:MGI:213768	0.12918
ENSMUSG00000020134	Peli1	pellino 1 [Source:MGI Symbol;Acc:MGI:1914495]	0.1292
ENSMUSG00000078812	Eif5a	eukaryotic translation initiation factor 5A [Source:N	0.1293

ENSMUSG00000002791	Pdxk-ps	pyridoxal (pyridoxine, vitamin B6) kinase, pseudoge	0.12933
ENSMUSG00000097156	Gm3764	predicted gene 3764 [Source:MGI Symbol;Acc:MGI:	0.12933
ENSMUSG00000085776	5430402O13Rik	RIKEN cDNA 5430402O13 gene [Source:MGI Symbo	0.12937
ENSMUSG00000047143	Dmrta2	doublesex and mab-3 related transcription factor li	0.1294
ENSMUSG00000049336	Tenm2	teneurin transmembrane protein 2 [Source:MGI Sy	0.12942
ENSMUSG00000019790	Stxbp5	syntaxin binding protein 5 (tomosyn) [Source:MGI S	0.12947
ENSMUSG00000075316	Scn9a	sodium channel, voltage-gated, type IX, alpha [Sour	0.1295
ENSMUSG00000040181	Fmo1	flavin containing monooxygenase 1 [Source:MGI Sy	0.12952
ENSMUSG00000031157	Pqbp1	polyglutamine binding protein 1 [Source:MGI Symb	0.12969
ENSMUSG00000015932	Dstn	destrin [Source:MGI Symbol;Acc:MGI:1929270]	0.12975
ENSMUSG00000033031	C330027C09Rik	RIKEN cDNA C330027C09 gene [Source:MGI Symbo	0.12976
ENSMUSG00000031523	Dlc1	deleted in liver cancer 1 [Source:MGI Symbol;Acc:M	0.12985
ENSMUSG00000033629	Ptplad1	protein tyrosine phosphatase-like A domain contai	0.12985
ENSMUSG00000097057	Gm17638	predicted gene, 17638 [Source:MGI Symbol;Acc:MG	0.12985
ENSMUSG00000029625	Cpsf4	cleavage and polyadenylation specific factor 4 [Sou	0.12987
ENSMUSG00000078908	Mon1b	MON1 homolog b (yeast) [Source:MGI Symbol;Acc:	0.12988
ENSMUSG00000051413	Plagl2	pleiomorphic adenoma gene-like 2 [Source:MGI Syn	0.12991
ENSMUSG00000020389	Cdkl3	cyclin-dependent kinase-like 3 [Source:MGI Symbol	0.12993
ENSMUSG00000039542	Ncam1	neural cell adhesion molecule 1 [Source:MGI Symbo	0.12994
ENSMUSG00000028757	Ddost	dolichyl-di-phosphooligosaccharide-protein glycotr	0.12996
ENSMUSG00000084917	Gm17477	predicted gene, 17477 [Source:MGI Symbol;Acc:MG	0.12999
ENSMUSG00000023909	Paqr4	progestin and adipoQ receptor family member IV [S	0.13034
ENSMUSG00000074782	4833422C13Rik	RIKEN cDNA 4833422C13 gene [Source:MGI Symbo	0.13034
ENSMUSG00000027714	Exosc9	exosome component 9 [Source:MGI Symbol;Acc:M	0.1304
ENSMUSG00000025227	Tmem180	transmembrane protein 180 [Source:MGI Symbol;A	0.13048
ENSMUSG00000070939	Tgfbrap1	transforming growth factor, beta receptor associat	0.13048
ENSMUSG00000048655	Ccdc169	coiled-coil domain containing 169 [Source:MGI Sym	0.1305
ENSMUSG00000078994	Zfp429	zinc finger protein 429 [Source:MGI Symbol;Acc:MG	0.13064
ENSMUSG00000066151	Fkbp15	FK506 binding protein 15 [Source:MGI Symbol;Acc:	0.13068
ENSMUSG00000067653	Ankrd23	ankyrin repeat domain 23 [Source:MGI Symbol;Acc	0.13072
ENSMUSG00000085148	Mir22hg	Mir22 host gene (non-protein coding) [Source:MGI	0.13073
ENSMUSG00000071342	Lsmem1	leucine-rich single-pass membrane protein 1 [Sourc	0.13074
ENSMUSG00000022099	Dmtn	dematin actin binding protein [Source:MGI Symbol;	0.13075
ENSMUSG00000041236	Vps41	vacuolar protein sorting 41 (yeast) [Source:MGI Syn	0.13081
ENSMUSG00000031930	Wwp2	WW domain containing E3 ubiquitin protein ligase	0.13083
ENSMUSG00000035773	Kiss1r	KISS1 receptor [Source:MGI Symbol;Acc:MGI:21487	0.13083
ENSMUSG00000025212	Sfxn3	sideroflexin 3 [Source:MGI Symbol;Acc:MGI:213767	0.13085
ENSMUSG00000053049	Gm15413	predicted gene 15413 [Source:MGI Symbol;Acc:MG	0.13087
ENSMUSG00000052102	Gnpda1	glucosamine-6-phosphate deaminase 1 [Source:MG	0.13089
ENSMUSG00000027935	Rab13	RAB13, member RAS oncogene family [Source:MGI	0.131
ENSMUSG00000077391	Gm24336	predicted gene, 24336 [Source:MGI Symbol;Acc:MG	0.13101
ENSMUSG00000088037	Gm24992	predicted gene, 24992 [Source:MGI Symbol;Acc:MG	0.13103
ENSMUSG00000035235	Trim13	tripartite motif-containing 13 [Source:MGI Symbol;	0.13104
ENSMUSG00000081696	Gm12717	predicted gene 12717 [Source:MGI Symbol;Acc:MG	0.13108
ENSMUSG00000089800	Rps19-ps10	ribosomal protein S19, pseudogene 10 [Source:MG	0.13114
ENSMUSG00000097419	Gm26666	predicted gene, 26666 [Source:MGI Symbol;Acc:MG	0.13115
ENSMUSG00000094199	Gm25450	predicted gene, 25450 [Source:MGI Symbol;Acc:MG	0.13118

ENSMUSG00000097859	Gm26601	predicted gene, 26601 [Source:MGI Symbol;Acc:M(	0.13122
ENSMUSG00000057672	Pkn1	protein kinase N1 [Source:MGI Symbol;Acc:MGI:10	0.13131
ENSMUSG00000015149	Sirt2	sirtuin 2 [Source:MGI Symbol;Acc:MGI:1927664]	0.13133
ENSMUSG00000098111	Gm4654	predicted gene 4654 [Source:MGI Symbol;Acc:MGI:	0.13133
ENSMUSG00000018559	Ctdnep1	CTD nuclear envelope phosphatase 1 [Source:MGI S	0.13136
ENSMUSG00000031026	Trim66	tripartite motif-containing 66 [Source:MGI Symbol;	0.13137
ENSMUSG00000008976	Gabpa	GA repeat binding protein, alpha [Source:MGI Symb	0.13154
ENSMUSG00000022656	Pvrl3	poliovirus receptor-related 3 [Source:MGI Symbol;A	0.13161
ENSMUSG00000007777	0610009B22Rik	RIKEN cDNA 0610009B22 gene [Source:MGI Symbo	0.13164
ENSMUSG00000072825	Cep170b	centrosomal protein 170B [Source:MGI Symbol;Acc	0.13171
ENSMUSG00000018042	Cyb5r3	cytochrome b5 reductase 3 [Source:MGI Symbol;Ac	0.13178
ENSMUSG00000011306	Sugp1	SURP and G patch domain containing 1 [Source:MG	0.13192
ENSMUSG00000074182	Znhit6	zinc finger, HIT type 6 [Source:MGI Symbol;Acc:MG	0.13209
ENSMUSG00000019194	Scn1b	sodium channel, voltage-gated, type I, beta [Source	0.1321
ENSMUSG00000034889	Cactin	cactin, spliceosome C complex subunit [Source:MG	0.13216
ENSMUSG00000061838	Suclg2	succinate-Coenzyme A ligase, GDP-forming, beta su	0.13216
ENSMUSG00000018822	Sfrp5	secreted frizzled-related sequence protein 5 [Sourc	0.13217
ENSMUSG00000055553	Kxd1	KxDL motif containing 1 [Source:MGI Symbol;Acc:M	0.13222
ENSMUSG00000014773	Dll1	delta-like 1 (Drosophila) [Source:MGI Symbol;Acc:M	0.13224
ENSMUSG00000024210	Ip6k3	inositol hexaphosphate kinase 3 [Source:MGI Symb	0.13224
ENSMUSG00000037033	Clca4	chloride channel calcium activated 4 [Source:MGI S	0.13224
ENSMUSG00000026155	Smap1	small ArfGAP 1 [Source:MGI Symbol;Acc:MGI:2138	0.13225
ENSMUSG00000090273	Prr22	proline rich 22 [Source:MGI Symbol;Acc:MGI:26853	0.13228
ENSMUSG00000064877	Gm23377	predicted gene, 23377 [Source:MGI Symbol;Acc:M(	0.13243
ENSMUSG00000022938	Fam3b	family with sequence similarity 3, member B [Sourc	0.13245
ENSMUSG00000044734	Serpinb1a	serine (or cysteine) peptidase inhibitor, clade B, me	0.13246
ENSMUSG00000022404	Slc25a17	solute carrier family 25 (mitochondrial carrier, perc	0.13255
ENSMUSG00000092131	BC050972	cDNA sequence BC050972 [Source:MGI Symbol;Acc	0.13256
ENSMUSG00000084904	Gm14827	predicted gene 14827 [Source:MGI Symbol;Acc:MG	0.13257
ENSMUSG00000047819	Tigd4	tigger transposable element derived 4 [Source:MGI	0.13258
ENSMUSG00000085807	Gm13069	predicted gene 13069 [Source:MGI Symbol;Acc:MG	0.13258
ENSMUSG00000084231	Gm11975	predicted gene 11975 [Source:MGI Symbol;Acc:MG	0.13259
ENSMUSG00000092274	Neat1	nuclear paraspeckle assembly transcript 1 (non-pro	0.13262
ENSMUSG00000032827	Ppp1r9a	protein phosphatase 1, regulatory (inhibitor) subur	0.13282
ENSMUSG00000091390	Gm17168	predicted gene 17168 [Source:MGI Symbol;Acc:MG	0.13288
ENSMUSG00000054426	A930005H10Rik	RIKEN cDNA A930005H10 gene [Source:MGI Symbo	0.1329
ENSMUSG00000031960	Aars	alanyl-tRNA synthetase [Source:MGI Symbol;Acc:M	0.13291
ENSMUSG00000045031	Cetn4	centrin 4 [Source:MGI Symbol;Acc:MGI:2677454]	0.13299
ENSMUSG00000044005	Gls2	glutaminase 2 (liver, mitochondrial) [Source:MGI Sy	0.13303
ENSMUSG00000026207	Speg	SPEG complex locus [Source:MGI Symbol;Acc:MGI:1	0.13305
ENSMUSG00000038963	Slco4a1	solute carrier organic anion transporter family, mer	0.13311
ENSMUSG00000084987	Gm13134	predicted gene 13134 [Source:MGI Symbol;Acc:MG	0.13313
ENSMUSG00000021661	Ankra2	ankyrin repeat, family A (RFXANK-like), 2 [Source:M	0.13317
ENSMUSG00000057181	5730455P16Rik	RIKEN cDNA 5730455P16 gene [Source:MGI Symbo	0.13318
ENSMUSG00000093187	Mir344-2	microRNA 344-2 [Source:MGI Symbol;Acc:MGI:371	0.13319
ENSMUSG00000036304	Zdhhc23	zinc finger, DHHC domain containing 23 [Source:M(	0.13322
ENSMUSG00000089735	Gm8428	predicted gene 8428 [Source:MGI Symbol;Acc:MGI:	0.13322

ENSMUSG00000032688	Malt1	mucosa associated lymphoid tissue lymphoma tran	0.13326
ENSMUSG00000027217	Tspan18	tetraspanin 18 [Source:MGI Symbol;Acc:MGI:19171	0.13327
ENSMUSG00000003662	Ciao1	cytosolic iron-sulfur protein assembly 1 [Source:MG	0.13336
ENSMUSG00000019039	Dalrd3	DALR anticodon binding domain containing 3 [Sour	0.13342
ENSMUSG00000016024	Lbp	lipopolysaccharide binding protein [Source:MGI Syr	0.13346
ENSMUSG00000089662	Gm14057	predicted gene 14057 [Source:MGI Symbol;Acc:MG	0.13357
ENSMUSG00000018548	Trim37	tripartite motif-containing 37 [Source:MGI Symbol;	0.13359
ENSMUSG00000037086	1110059M19Rik	RIKEN cDNA 1110059M19 gene [Source:MGI Symb	0.13361
ENSMUSG00000022871	Fetub	fetuin beta [Source:MGI Symbol;Acc:MGI:1890221]	0.13366
ENSMUSG00000002409	Dyrk1b	dual-specificity tyrosine-(Y)-phosphorylation regula	0.13374
ENSMUSG00000006784	Ttc25	tetratricopeptide repeat domain 25 [Source:MGI Sy	0.13375
ENSMUSG00000097882	Gm16529	predicted gene, 16529 [Source:MGI Symbol;Acc:MG	0.13375
ENSMUSG00000048106	4632415L05Rik	RIKEN cDNA 4632415L05 gene [Source:MGI Symbo	0.13378
ENSMUSG00000031153	Gripap1	GRIP1 associated protein 1 [Source:MGI Symbol;Ac	0.13383
ENSMUSG00000015222	Map2	microtubule-associated protein 2 [Source:MGI Sym	0.13384
ENSMUSG00000010517	Faf1	Fas-associated factor 1 [Source:MGI Symbol;Acc:M	0.13388
ENSMUSG00000078681	Tm2d3	TM2 domain containing 3 [Source:MGI Symbol;Acc:	0.13388
ENSMUSG00000046806	3110062M04Rik	RIKEN cDNA 3110062M04 gene [Source:MGI Symb	0.13394
ENSMUSG00000064289	Tank	TRAF family member-associated Nf-kappa B activat	0.13397
ENSMUSG00000053801	Grwd1	glutamate-rich WD repeat containing 1 [Source:MG	0.13404
ENSMUSG00000085408	C530005A16Rik	RIKEN cDNA C530005A16 gene [Source:MGI Symbo	0.13413
ENSMUSG00000034738	Nostrin	nitric oxide synthase trafficker [Source:MGI Symbol	0.13417
ENSMUSG00000049658	Bdp1	B double prime 1, subunit of RNA polymerase III tra	0.13417
ENSMUSG00000098154	Gm5787	predicted gene 5787 [Source:MGI Symbol;Acc:MGI:	0.13417
ENSMUSG00000022142	Nup155	nucleoporin 155 [Source:MGI Symbol;Acc:MGI:218	0.13432
ENSMUSG00000019978	Epb4.1l2	erythrocyte protein band 4.1-like 2 [Source:MGI Sy	0.13434
ENSMUSG00000055026	Gabrg3	gamma-aminobutyric acid (GABA) A receptor, subu	0.13448
ENSMUSG00000035620	Ric8b	resistance to inhibitors of cholinesterase 8 homolog	0.13453
ENSMUSG00000022112	Gpc5	glypican 5 [Source:MGI Symbol;Acc:MGI:1194894]	0.13455
ENSMUSG00000041548	Hspb8	heat shock protein 8 [Source:MGI Symbol;Acc:MGI:	0.13457
ENSMUSG00000044229	Nxpe4	neurexophilin and PC-esterase domain family, mem	0.13458
ENSMUSG00000033061	Resp18	regulated endocrine-specific protein 18 [Source:MG	0.13466
ENSMUSG00000087334	AW495222	expressed sequence AW495222 [Source:MGI Symb	0.13466
ENSMUSG00000074247	Dda1	DET1 and DDB1 associated 1 [Source:MGI Symbol;A	0.1347
ENSMUSG00000039158	Akna	AT-hook transcription factor [Source:MGI Symbol;A	0.13476
ENSMUSG00000027016	Zfp385b	zinc finger protein 385B [Source:MGI Symbol;Acc:M	0.13479
ENSMUSG00000038811	Gngt2	guanine nucleotide binding protein (G protein), gan	0.13485
ENSMUSG00000051074	4930579K19Rik	RIKEN cDNA 4930579K19 gene [Source:MGI Symbo	0.13496
ENSMUSG00000057637	Prdm2	PR domain containing 2, with ZNF domain [Source:I	0.13503
ENSMUSG00000038366	Lasp1	LIM and SH3 protein 1 [Source:MGI Symbol;Acc:MG	0.13504
ENSMUSG00000029658	Wdr95	WD40 repeat domain 95 [Source:MGI Symbol;Acc:M	0.13508
ENSMUSG00000068638	4930403O15Rik	RIKEN cDNA 4930403O15 gene [Source:MGI Symbo	0.13508
ENSMUSG00000029145	Eif2b4	eukaryotic translation initiation factor 2B, subunit 4	0.13515
ENSMUSG00000092842	Gm24333	predicted gene, 24333 [Source:MGI Symbol;Acc:MG	0.13517
ENSMUSG00000030219	Erp27	endoplasmic reticulum protein 27 [Source:MGI Sym	0.1352
ENSMUSG00000065265	Gm23455	predicted gene, 23455 [Source:MGI Symbol;Acc:MG	0.13528
ENSMUSG00000041506	Rrp9	RRP9, small subunit (SSU) processome component,	0.13535

ENSMUSG00000094790	Gm6625	predicted gene 6625 [Source:MGI Symbol;Acc:MGI:	0.13542
ENSMUSG00000024365	Cyp21a1	cytochrome P450, family 21, subfamily a, polypepti	0.13543
ENSMUSG00000034435	Tmem30b	transmembrane protein 30B [Source:MGI Symbol;A	0.13548
ENSMUSG00000066149	Cdc26	cell division cycle 26 [Source:MGI Symbol;Acc:MGI:	0.13548
ENSMUSG00000081651	Gm15530	predicted gene 15530 [Source:MGI Symbol;Acc:MG	0.13551
ENSMUSG00000027580	Helz2	helicase with zinc finger 2, transcriptional coactivat	0.13554
ENSMUSG00000009079	Ewsr1	Ewing sarcoma breakpoint region 1 [Source:MGI Sy	0.13561
ENSMUSG00000029385	Ccng2	cyclin G2 [Source:MGI Symbol;Acc:MGI:1095734]	0.13565
ENSMUSG00000058192	Zfp846	zinc finger protein 846 [Source:MGI Symbol;Acc:MG	0.13578
ENSMUSG00000025162	Csnk1d	casein kinase 1, delta [Source:MGI Symbol;Acc:MGI	0.13587
ENSMUSG00000021794	Glud1	glutamate dehydrogenase 1 [Source:MGI Symbol;A	0.1359
ENSMUSG00000031819	Emc8	ER membrane protein complex subunit 8 [Source:M	0.13592
ENSMUSG00000027746	Ufm1	ubiquitin-fold modifier 1 [Source:MGI Symbol;Acc:M	0.13595
ENSMUSG00000033938	Ndufb7	NADH dehydrogenase (ubiquinone) 1 beta subcom	0.13606
ENSMUSG00000038690	Atp5j2	ATP synthase, H+ transporting, mitochondrial F0 co	0.13608
ENSMUSG00000036172	Cd200r3	CD200 receptor 3 [Source:MGI Symbol;Acc:MGI:19	0.13609
ENSMUSG00000031204	Asb12	ankyrin repeat and SOCS box-containing 12 [Source	0.13614
ENSMUSG00000050335	Lgals3	lectin, galactose binding, soluble 3 [Source:MGI Syr	0.13616
ENSMUSG00000041878	8430432A02Rik	RIKEN cDNA 8430432A02 gene [Source:MGI Symbo	0.13618
ENSMUSG00000084378	Gm15238	predicted gene 15238 [Source:MGI Symbol;Acc:MG	0.13622
ENSMUSG00000030551	Nr2f2	nuclear receptor subfamily 2, group F, member 2 [S	0.13624
ENSMUSG00000066592	Gm7589	predicted gene 7589 [Source:MGI Symbol;Acc:MGI:	0.13626
ENSMUSG00000027350	Chgb	chromogranin B [Source:MGI Symbol;Acc:MGI:8839	0.13632
ENSMUSG00000086513	9130208D14Rik	RIKEN cDNA 9130208D14 gene [Source:MGI Symbo	0.13633
ENSMUSG00000024969	Mark2	MAP/microtubule affinity-regulating kinase 2 [Sour	0.13636
ENSMUSG00000036782	Klhl13	kelch-like 13 [Source:MGI Symbol;Acc:MGI:191470	0.1364
ENSMUSG00000045854	Lyrm2	LYR motif containing 2 [Source:MGI Symbol;Acc:MG	0.13645
ENSMUSG00000057388	Mrpl18	mitochondrial ribosomal protein L18 [Source:MGI S	0.13646
ENSMUSG00000026694	Mettl13	methyltransferase like 13 [Source:MGI Symbol;Acc:	0.13649
ENSMUSG00000083433	Gm15405	predicted gene 15405 [Source:MGI Symbol;Acc:MG	0.13649
ENSMUSG00000028373	Astn2	astrotactin 2 [Source:MGI Symbol;Acc:MGI:188927	0.13651
ENSMUSG00000024963	Dnajc4	DnaJ (Hsp40) homolog, subfamily C, member 4 [Sou	0.13659
ENSMUSG00000035686	Thrsp	thyroid hormone responsive [Source:MGI Symbol;A	0.13659
ENSMUSG00000085766	2810430I11Rik	RIKEN cDNA 2810430I11 gene [Source:MGI Symbol	0.13659
ENSMUSG00000050860	Phospho1	phosphatase, orphan 1 [Source:MGI Symbol;Acc:M	0.13666
ENSMUSG00000025161	Slc16a3	solute carrier family 16 (monocarboxylic acid trans	0.13672
ENSMUSG00000015474	Ppt2	palmitoyl-protein thioesterase 2 [Source:MGI Symb	0.13674
ENSMUSG00000025337	Sbds	Shwachman-Bodian-Diamond syndrome homolog (	0.13677
ENSMUSG00000086914	Gm16124	predicted gene 16124 [Source:MGI Symbol;Acc:MG	0.13677
ENSMUSG00000007850	Hnrnph1	heterogeneous nuclear ribonucleoprotein H1 [Sour	0.13684
ENSMUSG00000031907	Zfp90	zinc finger protein 90 [Source:MGI Symbol;Acc:MGI	0.13692
ENSMUSG00000028451	1700022I11Rik	RIKEN cDNA 1700022I11 gene [Source:MGI Symbol	0.13697
ENSMUSG00000097342	Gm26618	predicted gene, 26618 [Source:MGI Symbol;Acc:MG	0.137
ENSMUSG00000029671	Wnt16	wingless-related MMTV integration site 16 [Source	0.13701
ENSMUSG00000028149	Rap1gds1	RAP1, GTP-GDP dissociation stimulator 1 [Source:M	0.13712
ENSMUSG00000023051	Tarbp2	TAR (HIV) RNA binding protein 2 [Source:MGI Symb	0.13719
ENSMUSG00000062713	Sim2	single-minded homolog 2 (Drosophila) [Source:MGI	0.13721

ENSMUSG00000085238	4930479D17Rik	RIKEN cDNA 4930479D17 gene [Source:MGI Symbo	0.13721
ENSMUSG00000078427	Sarnp	SAP domain containing ribonucleoprotein [Source:I	0.13722
ENSMUSG00000085949	Gm14275	predicted gene 14275 [Source:MGI Symbol;Acc:MG	0.13726
ENSMUSG00000068959	Zfp619	zinc finger protein 619 [Source:MGI Symbol;Acc:M(	0.13741
ENSMUSG00000086904	Gm13404	predicted gene 13404 [Source:MGI Symbol;Acc:MG	0.13741
ENSMUSG00000078517	Emc1	ER membrane protein complex subunit 1 [Source:M	0.13752
ENSMUSG00000041608	Entpd3	ectonucleoside triphosphate diphosphohydrolase 3	0.13766
ENSMUSG00000021622	Ckmt2	creatine kinase, mitochondrial 2 [Source:MGI Symb	0.13768
ENSMUSG00000035596	Mboat7	membrane bound O-acyltransferase domain contai	0.13772
ENSMUSG00000039934	Gsap	gamma-secretase activating protein [Source:MGI S	0.13776
ENSMUSG00000047417	Rexo1	REX1, RNA exonuclease 1 homolog (S. cerevisiae) [	0.13779
ENSMUSG00000059149	Mfsd4	major facilitator superfamily domain containing 4 [	0.13784
ENSMUSG00000004642	Slbp	stem-loop binding protein [Source:MGI Symbol;Acc	0.13795
ENSMUSG00000038456	Dennd2a	DENN/MADD domain containing 2A [Source:MGI Sy	0.138
ENSMUSG00000042404	Dennd4b	DENN/MADD domain containing 4B [Source:MGI Sy	0.13801
ENSMUSG00000028088	Fmo5	flavin containing monooxygenase 5 [Source:MGI Sy	0.13806
ENSMUSG00000030752	Kdm8	lysine (K)-specific demethylase 8 [Source:MGI Symb	0.13816
ENSMUSG00000053390	Zfp952	zinc finger protein 952 [Source:MGI Symbol;Acc:M(	0.13821
ENSMUSG00000088789	Scarna13	small Cajal body-specific RNA 1 [Source:MGI Symbo	0.13826
ENSMUSG00000083978	Gm5060	predicted gene 5060 [Source:MGI Symbol;Acc:MGI:	0.1383
ENSMUSG00000073998	Olfr520	olfactory receptor 520 [Source:MGI Symbol;Acc:M(	0.13834
ENSMUSG00000090277	Gm8338	predicted gene 8338 [Source:MGI Symbol;Acc:MGI:	0.13835
ENSMUSG00000084350	Znf41-ps	ZNF41, pseudogene [Source:MGI Symbol;Acc:MGI:1	0.13837
ENSMUSG00000065904	Gm26109	predicted gene, 26109 [Source:MGI Symbol;Acc:M(	0.13839
ENSMUSG00000064353	mt-Td	mitochondrially encoded tRNA aspartic acid [Sourc	0.13841
ENSMUSG00000091305	Gm17100	predicted gene 17100 [Source:MGI Symbol;Acc:MG	0.13841
ENSMUSG00000056888	Glipr1	GLI pathogenesis-related 1 (glioma) [Source:MGI Sy	0.13842
ENSMUSG00000029209	Gnpda2	glucosamine-6-phosphate deaminase 2 [Source:MG	0.13843
ENSMUSG00000031060	Rbm10	RNA binding motif protein 10 [Source:MGI Symbol;	0.13843
ENSMUSG00000055214	Pld5	phospholipase D family, member 5 [Source:MGI Sy	0.13843
ENSMUSG00000039473	Ubn1	ubinuclein 1 [Source:MGI Symbol;Acc:MGI:1891307	0.13844
ENSMUSG00000037977	6430571L13Rik	RIKEN cDNA 6430571L13 gene [Source:MGI Symbo	0.13846
ENSMUSG00000085260	1700013G23Rik	RIKEN cDNA 1700013G23 gene [Source:MGI Symbo	0.13846
ENSMUSG00000053038	Gm6180	predicted pseudogene 6180 [Source:MGI Symbol;A	0.13847
ENSMUSG00000040548	Tex2	testis expressed gene 2 [Source:MGI Symbol;Acc:M	0.13859
ENSMUSG00000039021	Ttc16	tetratricopeptide repeat domain 16 [Source:MGI Sy	0.13864
ENSMUSG00000079497	Gm13420	predicted gene 13420 [Source:MGI Symbol;Acc:MG	0.13867
ENSMUSG00000057228	Aadat	aminoadipate aminotransferase [Source:MGI Symb	0.13868
ENSMUSG00000057605	Gm6807	predicted gene 6807 [Source:MGI Symbol;Acc:MGI:	0.1387
ENSMUSG00000006678	Pola1	polymerase (DNA directed), alpha 1 [Source:MGI Sy	0.13887
ENSMUSG00000027171	Prrg4	proline rich Gla (G-carboxyglutamic acid) 4 (transm	0.13898
ENSMUSG00000022827	Rabl3	RAB, member of RAS oncogene family-like 3 [Sourc	0.139
ENSMUSG00000011960	Ccnt1	cyclin T1 [Source:MGI Symbol;Acc:MGI:1328363]	0.13908
ENSMUSG00000021198	Unc79	unc-79 homolog (C. elegans) [Source:MGI Symbol;A	0.13909
ENSMUSG00000051864	Tbc1d22a	TBC1 domain family, member 22a [Source:MGI Syn	0.13911
ENSMUSG00000047459	Dynlrb1	dynein light chain roadblock-type 1 [Source:MGI Sy	0.13921
ENSMUSG00000046671	Mtfr1l	mitochondrial fission regulator 1-like [Source:MGI S	0.13924

ENSMUSG00000020986	Sec23a	SEC23A (S. cerevisiae) [Source:MGI Symbol;Acc:MG	0.13932
ENSMUSG00000028274	Rngtt	RNA guanylyltransferase and 5'-phosphatase [Sour	0.13934
ENSMUSG00000067367	Lyar	Ly1 antibody reactive clone [Source:MGI Symbol;Ac	0.13936
ENSMUSG00000000631	Myo18a	myosin XVIIIA [Source:MGI Symbol;Acc:MGI:266718	0.13937
ENSMUSG00000037995	Igsf9	immunoglobulin superfamily, member 9 [Source:M	0.13945
ENSMUSG00000027651	Rprd1b	regulation of nuclear pre-mRNA domain containing	0.13946
ENSMUSG00000087377	AV099323	expressed sequence AV099323 [Source:MGI Symbo	0.13946
ENSMUSG00000085568	Gm14858	predicted gene 14858 [Source:MGI Symbol;Acc:MG	0.13947
ENSMUSG00000071648	Rom1	rod outer segment membrane protein 1 [Source:M	0.13961
ENSMUSG00000031374	Zfp92	zinc finger protein 92 [Source:MGI Symbol;Acc:MGI	0.13963
ENSMUSG00000040661	Rad54l2	RAD54 like 2 (S. cerevisiae) [Source:MGI Symbol;Ac	0.13963
ENSMUSG00000024078	Ttc27	tetratricopeptide repeat domain 27 [Source:MGI Sy	0.13967
ENSMUSG00000074802	Gas2l3	growth arrest-specific 2 like 3 [Source:MGI Symbol;	0.13967
ENSMUSG00000025968	Ndufs1	NADH dehydrogenase (ubiquinone) Fe-S protein 1 [	0.13969
ENSMUSG00000092657	Gm25240	predicted gene, 25240 [Source:MGI Symbol;Acc:MG	0.13975
ENSMUSG00000023967	Mrps18a	mitochondrial ribosomal protein S18A [Source:MGI	0.13977
ENSMUSG00000030603	Psmc4	proteasome (prosome, macropain) 26S subunit, AT	0.13979
ENSMUSG00000093409	Gm25193	predicted gene, 25193 [Source:MGI Symbol;Acc:MG	0.1398
ENSMUSG00000003438	Timm50	translocase of inner mitochondrial membrane 50 [S	0.13982
ENSMUSG00000002546	Golga2	golgi autoantigen, golgin subfamily a, 2 [Source:MG	0.13989
ENSMUSG00000054537	Tmprss11e	transmembrane protease, serine 11e [Source:MGI S	0.13999
ENSMUSG00000086687	4930547E08Rik	RIKEN cDNA 4930547E08 gene [Source:MGI Symbo	0.14
ENSMUSG00000046637	Ttc34	tetratricopeptide repeat domain 34 [Source:MGI Sy	0.14001
ENSMUSG00000066224	Arid3c	AT rich interactive domain 3C (BRIGHT-like) [Source	0.14003
ENSMUSG00000019066	Rab3d	RAB3D, member RAS oncogene family [Source:MGI	0.14009
ENSMUSG00000084142	Gm9103	predicted gene 9103 [Source:MGI Symbol;Acc:MGI:	0.14016
ENSMUSG00000064622	Gm22304	predicted gene, 22304 [Source:MGI Symbol;Acc:MG	0.14017
ENSMUSG00000023921	Mut	methylmalonyl-Coenzyme A mutase [Source:MGI S	0.14018
ENSMUSG00000028707	Dmbx1	diencephalon/mesencephalon homeobox 1 [Source	0.14022
ENSMUSG00000021185	9030617O03Rik	RIKEN cDNA 9030617O03 gene [Source:MGI Symbo	0.14025
ENSMUSG00000044033	Ccdc141	coiled-coil domain containing 141 [Source:MGI Sym	0.14026
ENSMUSG00000082345	Gm13622	predicted gene 13622 [Source:MGI Symbol;Acc:MG	0.14028
ENSMUSG00000036422	Pcdh8	protocadherin 8 [Source:MGI Symbol;Acc:MGI:1306	0.14037
ENSMUSG00000039826	Trub2	TruB pseudouridine (psi) synthase homolog 2 (E. co	0.14042
ENSMUSG00000021782	Dlg5	discs, large homolog 5 (Drosophila) [Source:MGI Sy	0.14044
ENSMUSG00000020308	Tpgs1	tubulin polyglutamylase complex subunit 1 [Source	0.14045
ENSMUSG00000073489	Ifi204	interferon activated gene 204 [Source:MGI Symbol;	0.14045
ENSMUSG00000005045	Chd5	chromodomain helicase DNA binding protein 5 [Sou	0.14052
ENSMUSG00000038319	Kcnh2	potassium voltage-gated channel, subfamily H (eag	0.14058
ENSMUSG00000006764	Tph2	tryptophan hydroxylase 2 [Source:MGI Symbol;Acc	0.1406
ENSMUSG00000047248	C2cd3	C2 calcium-dependent domain containing 3 [Source	0.14063
ENSMUSG00000044573	Acp1	acid phosphatase 1, soluble [Source:MGI Symbol;Ac	0.1407
ENSMUSG00000095253	Zfp799	zinc finger protein 799 [Source:MGI Symbol;Acc:MG	0.1407
ENSMUSG00000052214	Opa3	optic atrophy 3 [Source:MGI Symbol;Acc:MGI:2686	0.14079
ENSMUSG00000085316	D330050G23Rik	RIKEN cDNA D330050G23 gene [Source:MGI Symbo	0.1408
ENSMUSG00000024646	Cyb5	cytochrome b-5 [Source:MGI Symbol;Acc:MGI:1926	0.1409
ENSMUSG00000032397	Tipin	timeless interacting protein [Source:MGI Symbol;Ac	0.14097

ENSMUSG00000026110	Mgat4a	mannoside acetylglucosaminyltransferase 4, isoenz	0.14099
ENSMUSG00000052861	Dnah6	dynein, axonemal, heavy chain 6 [Source:MGI Symb	0.14099
ENSMUSG00000031640	Klkb1	kallikrein B, plasma 1 [Source:MGI Symbol;Acc:MGI	0.14101
ENSMUSG00000023938	Aars2	alanyl-tRNA synthetase 2, mitochondrial (putative)	0.14102
ENSMUSG00000095762	Gm16378	predicted gene 16378 [Source:MGI Symbol;Acc:MG	0.14102
ENSMUSG00000024268	Celf4	CUGBP, Elav-like family member 4 [Source:MGI Syn	0.14104
ENSMUSG00000085622	3110056K07Rik	RIKEN cDNA 3110056K07 gene [Source:MGI Symbo	0.14114
ENSMUSG00000024112	Cacna1h	calcium channel, voltage-dependent, T type, alpha	0.14131
ENSMUSG00000046352	Gjb2	gap junction protein, beta 2 [Source:MGI Symbol;A	0.14131
ENSMUSG00000035569	Ankrd11	ankyrin repeat domain 11 [Source:MGI Symbol;Acc	0.14134
ENSMUSG00000031813	Mvb12a	multivesicular body subunit 12A [Source:MGI Symb	0.14137
ENSMUSG00000070509	Rgma	RGM domain family, member A [Source:MGI Symb	0.14142
ENSMUSG00000022824	Muc13	mucin 13, epithelial transmembrane [Source:MGI S	0.14158
ENSMUSG00000039345	Mettl22	methyltransferase like 22 [Source:MGI Symbol;Acc:	0.14158
ENSMUSG00000090108	Gm15717	predicted gene 15717 [Source:MGI Symbol;Acc:MG	0.14167
ENSMUSG00000096701	Gm9285	predicted gene 9285 [Source:MGI Symbol;Acc:MGI:	0.1417
ENSMUSG00000059201	Lep	leptin [Source:MGI Symbol;Acc:MGI:104663]	0.14174
ENSMUSG00000007892	Rplp1	ribosomal protein, large, P1 [Source:MGI Symbol;A	0.14181
ENSMUSG00000039367	Sec24c	Sec24 related gene family, member C (S. cerevisiae	0.14193
ENSMUSG00000033634	Cml2	camello-like 2 [Source:MGI Symbol;Acc:MGI:21364	0.14194
ENSMUSG00000025034	Trim8	tripartite motif-containing 8 [Source:MGI Symbol;A	0.14203
ENSMUSG00000042377	Fam83g	family with sequence similarity 83, member G [Sou	0.14203
ENSMUSG00000009185	Ccl8	chemokine (C-C motif) ligand 8 [Source:MGI Symbo	0.14207
ENSMUSG00000029772	Ahcyl2	S-adenosylhomocysteine hydrolase-like 2 [Source:M	0.14207
ENSMUSG00000027459	Fam110a	family with sequence similarity 110, member A [Sou	0.14208
ENSMUSG00000030559	Rab38	RAB38, member of RAS oncogene family [Source:M	0.14208
ENSMUSG00000093682	Gm20681	predicted gene 20681 [Source:MGI Symbol;Acc:MG	0.14212
ENSMUSG00000035847	Ids	iduronate 2-sulfatase [Source:MGI Symbol;Acc:MG	0.14215
ENSMUSG00000097323	4930426I24Rik	RIKEN cDNA 4930426I24 gene [Source:MGI Symbol	0.14217
ENSMUSG00000038095	Sbno1	sno, strawberry notch homolog 1 (Drosophila) [Sou	0.14219
ENSMUSG00000030987	Stim1	stromal interaction molecule 1 [Source:MGI Symbo	0.1423
ENSMUSG00000001604	Tcea3	transcription elongation factor A (SII), 3 [Source:M	0.14236
ENSMUSG00000035105	Egln3	EGL nine homolog 3 (C. elegans) [Source:MGI Symb	0.14236
ENSMUSG00000004994	Ccdc130	coiled-coil domain containing 130 [Source:MGI Sym	0.14248
ENSMUSG00000026917	Wdr5	WD repeat domain 5 [Source:MGI Symbol;Acc:MGI	0.14256
ENSMUSG00000024028	Tff2	trefoil factor 2 (spasmolytic protein 1) [Source:MGI	0.14257
ENSMUSG00000020732	Rab37	RAB37, member of RAS oncogene family [Source:M	0.1426
ENSMUSG00000040667	Nup88	nucleoporin 88 [Source:MGI Symbol;Acc:MGI:1049	0.1426
ENSMUSG00000092549	Gm20491	predicted gene 20491 [Source:MGI Symbol;Acc:MG	0.14264
ENSMUSG00000013033	Lphn1	latrophilin 1 [Source:MGI Symbol;Acc:MGI:1929461	0.14276
ENSMUSG00000005043	Sgsh	N-sulfoglucosamine sulfohydrolase (sulfamidase) [S	0.14278
ENSMUSG00000042305	Tmem183a	transmembrane protein 183A [Source:MGI Symbol;	0.14278
ENSMUSG00000033268	Duox1	dual oxidase 1 [Source:MGI Symbol;Acc:MGI:21394	0.1428
ENSMUSG00000031256	Cstf2	cleavage stimulation factor, 3' pre-RNA subunit 2 [S	0.14281
ENSMUSG00000033170	Card10	caspase recruitment domain family, member 10 [So	0.14286
ENSMUSG00000022247	Brix1	BRX1, biogenesis of ribosomes, homolog (S. cerevis	0.14291
ENSMUSG00000026082	Rev1	REV1 homolog (S. cerevisiae) [Source:MGI Symbol;	0.14293

ENSMUSG00000028778	Hcrtr1	hypocretin (orexin) receptor 1 [Source:MGI Symbol	0.14296
ENSMUSG00000071552	Tigit	T cell immunoreceptor with Ig and ITIM domains [S	0.14302
ENSMUSG00000002900	Lamb1	laminin B1 [Source:MGI Symbol;Acc:MGI:96743]	0.14304
ENSMUSG00000031169	Porcn	porcupine homolog (Drosophila) [Source:MGI Symb	0.14305
ENSMUSG00000080753	Gm15613	predicted gene 15613 [Source:MGI Symbol;Acc:MG	0.14311
ENSMUSG00000059775	Rps26-ps1	ribosomal protein S26, pseudogene 1 [Source:MGI	0.14312
ENSMUSG00000046934	Csl	citrate synthase like [Source:MGI Symbol;Acc:MGI:	0.14317
ENSMUSG00000055430	Nap1l5	nucleosome assembly protein 1-like 5 [Source:MGI	0.14326
ENSMUSG00000028648	Ndufs5	NADH dehydrogenase (ubiquinone) Fe-S protein 5 [	0.14328
ENSMUSG00000093780	Gm22395	predicted gene, 22395 [Source:MGI Symbol;Acc:MG	0.14328
ENSMUSG00000042616	Oscp1	organic solute carrier partner 1 [Source:MGI Symbo	0.14331
ENSMUSG00000020151	Ptprr	protein tyrosine phosphatase, receptor type, R [Sou	0.14333
ENSMUSG00000061607	Mdc1	mediator of DNA damage checkpoint 1 [Source:MG	0.14336
ENSMUSG00000097545	A930011O12Rik	RIKEN cDNA A930011O12 gene [Source:MGI Symbo	0.14336
ENSMUSG00000097596	Gm26673	predicted gene, 26673 [Source:MGI Symbol;Acc:MG	0.14337
ENSMUSG00000079143	Gm11052	predicted gene 11052 [Source:MGI Symbol;Acc:MG	0.14342
ENSMUSG00000042567	Nek10	NIMA (never in mitosis gene a)- related kinase 10 [S	0.14344
ENSMUSG00000022407	Adsl	adenylosuccinate lyase [Source:MGI Symbol;Acc:M	0.14345
ENSMUSG00000056738	A730036I17Rik	RIKEN cDNA A730036I17 gene [Source:MGI Symbol	0.14369
ENSMUSG00000071015	Gm136	predicted gene 136 [Source:MGI Symbol;Acc:MGI:2	0.1437
ENSMUSG00000001376	Ccdc132	coiled-coil domain containing 132 [Source:MGI Sym	0.14375
ENSMUSG00000003526	Prodh	proline dehydrogenase [Source:MGI Symbol;Acc:M	0.1438
ENSMUSG00000018509	Cenpv	centromere protein V [Source:MGI Symbol;Acc:MG	0.14386
ENSMUSG00000050105	Grrp1	glycine/arginine rich protein 1 [Source:MGI Symbol	0.14388
ENSMUSG00000037938	Chchd5	coiled-coil-helix-coiled-coil-helix domain containing	0.1439
ENSMUSG00000093378	Gm20663	predicted gene 20663 [Source:MGI Symbol;Acc:MG	0.14391
ENSMUSG00000068259	Olfr461	olfactory receptor 461 [Source:MGI Symbol;Acc:MG	0.14394
ENSMUSG00000042331	Specc1	sperm antigen with calponin homology and coiled-c	0.14395
ENSMUSG00000028096	Gpr89	G protein-coupled receptor 89 [Source:MGI Symbo	0.14396
ENSMUSG00000029641	Rasl11a	RAS-like, family 11, member A [Source:MGI Symbol	0.14404
ENSMUSG00000008373	Prpf31	PRP31 pre-mRNA processing factor 31 homolog (ye	0.14416
ENSMUSG00000097062	Gm17586	predicted gene, 17586 [Source:MGI Symbol;Acc:MG	0.14416
ENSMUSG00000052584	Serp2	stress-associated endoplasmic reticulum protein fa	0.14418
ENSMUSG00000084329	Gm6733	predicted gene 6733 [Source:MGI Symbol;Acc:MGI:	0.14424
ENSMUSG00000068302	Noto	notochord homolog (Xenopus laevis) [Source:MGI S	0.14431
ENSMUSG00000097418	Gm26667	predicted gene, 26667 [Source:MGI Symbol;Acc:MG	0.14436
ENSMUSG00000003868	Ruvbl2	RuvB-like protein 2 [Source:MGI Symbol;Acc:MGI:1	0.14441
ENSMUSG00000032244	Fem1b	feminization 1 homolog b (C. elegans) [Source:MGI	0.14447
ENSMUSG00000041544	Ptchd2	patched domain containing 2 [Source:MGI Symbol;	0.14448
ENSMUSG00000087396	4933407K13Rik	RIKEN cDNA 4933407K13 gene [Source:MGI Symbo	0.14451
ENSMUSG00000020901	Pik3r5	phosphoinositide-3-kinase, regulatory subunit 5, p1	0.14459
ENSMUSG00000064373	Sepp1	selenoprotein P, plasma, 1 [Source:MGI Symbol;Acc	0.1446
ENSMUSG00000028023	Pitx2	paired-like homeodomain transcription factor 2 [So	0.14464
ENSMUSG00000040097	Flywch1	FLYWCH-type zinc finger 1 [Source:MGI Symbol;Acc	0.14467
ENSMUSG00000020827	Mink1	misshapen-like kinase 1 (zebrafish) [Source:MGI Sy	0.14489
ENSMUSG00000079055	Slc8a3	solute carrier family 8 (sodium/calcium exchanger),	0.14494
ENSMUSG00000042744	Gm15800	predicted gene 15800 [Source:MGI Symbol;Acc:MG	0.14503

ENSMUSG00000047415	Gpr68	G protein-coupled receptor 68 [Source:MGI Symbo	0.14504
ENSMUSG00000030092	Cntn6	contactin 6 [Source:MGI Symbol;Acc:MGI:1858223]	0.14506
ENSMUSG00000024340	Btnl2	butyrophilin-like 2 [Source:MGI Symbol;Acc:MGI:18	0.1451
ENSMUSG00000021207	Akr1c21	aldo-keto reductase family 1, member C21 [Source:	0.14517
ENSMUSG00000031200	Mtcp1	mature T cell proliferation 1 [Source:MGI Symbol;A	0.14524
ENSMUSG00000047155	Cyp4x1	cytochrome P450, family 4, subfamily x, polypeptid	0.14525
ENSMUSG00000002524	Puf60	poly-U binding splicing factor 60 [Source:MGI Symb	0.14526
ENSMUSG00000004264	Phb2	prohibitin 2 [Source:MGI Symbol;Acc:MGI:102520]	0.14529
ENSMUSG00000032060	Cryab	crystallin, alpha B [Source:MGI Symbol;Acc:MGI:88	0.14529
ENSMUSG00000021592	Arsk	arylsulfatase K [Source:MGI Symbol;Acc:MGI:19242	0.14532
ENSMUSG00000021476	Habp4	hyaluronic acid binding protein 4 [Source:MGI Sym	0.14537
ENSMUSG00000055866	Per2	period circadian clock 2 [Source:MGI Symbol;Acc:M	0.14541
ENSMUSG00000024220	Zfp523	zinc finger protein 523 [Source:MGI Symbol;Acc:M	0.14552
ENSMUSG00000066677	Pydc3	pyrin domain containing 3 [Source:MGI Symbol;Acc	0.14563
ENSMUSG00000029828	4921507P07Rik	RIKEN cDNA 4921507P07 gene [Source:MGI Symbo	0.1458
ENSMUSG00000032193	Ldlr	low density lipoprotein receptor [Source:MGI Symb	0.1458
ENSMUSG00000033792	Atp7a	ATPase, Cu++ transporting, alpha polypeptide [Sou	0.14599
ENSMUSG00000071547	Nt5dc2	5'-nucleotidase domain containing 2 [Source:MGI S	0.14603
ENSMUSG00000036523	Greb1	gene regulated by estrogen in breast cancer protei	0.14608
ENSMUSG00000051910	Sox6	SRY-box containing gene 6 [Source:MGI Symbol;Acc	0.14614
ENSMUSG00000039263	Npepl1	aminopeptidase-like 1 [Source:MGI Symbol;Acc:MG	0.14619
ENSMUSG00000034818	Celf5	CUGBP, Elav-like family member 5 [Source:MGI Syn	0.14627
ENSMUSG00000023046	Igfbp6	insulin-like growth factor binding protein 6 [Source	0.14631
ENSMUSG00000050705	2310061I04Rik	RIKEN cDNA 2310061I04 gene [Source:MGI Symbol	0.14633
ENSMUSG00000021047	Nova1	neuro-oncological ventral antigen 1 [Source:MGI Sy	0.14638
ENSMUSG00000033909	Usp36	ubiquitin specific peptidase 36 [Source:MGI Symbo	0.14644
ENSMUSG00000036144	Meox2	mesenchyme homeobox 2 [Source:MGI Symbol;Acc	0.14656
ENSMUSG00000051499	Zfp786	zinc finger protein 786 [Source:MGI Symbol;Acc:M	0.14665
ENSMUSG00000039236	Isg20	interferon-stimulated protein [Source:MGI Symbol;	0.1467
ENSMUSG00000030929	Eri2	exoribonuclease 2 [Source:MGI Symbol;Acc:MGI:19	0.14688
ENSMUSG00000029326	Enoph1	enolase-phosphatase 1 [Source:MGI Symbol;Acc:M	0.14689
ENSMUSG00000046658	Zfp316	zinc finger protein 316 [Source:MGI Symbol;Acc:M	0.14691
ENSMUSG00000020914	Top2a	topoisomerase (DNA) II alpha [Source:MGI Symbol;	0.14696
ENSMUSG00000020097	Sgpl1	sphingosine phosphate lyase 1 [Source:MGI Symbo	0.147
ENSMUSG00000021957	Tkt	transketolase [Source:MGI Symbol;Acc:MGI:105992	0.14712
ENSMUSG00000062075	Lmnb2	lamin B2 [Source:MGI Symbol;Acc:MGI:96796]	0.14721
ENSMUSG00000003099	Ppp5c	protein phosphatase 5, catalytic subunit [Source:M	0.14727
ENSMUSG00000028931	Kcnab2	potassium voltage-gated channel, shaker-related su	0.14728
ENSMUSG00000054000	Tusc1	tumor suppressor candidate 1 [Source:MGI Symbol	0.14732
ENSMUSG00000082429	Gm13171	predicted gene 13171 [Source:MGI Symbol;Acc:MG	0.14735
ENSMUSG00000050931	Sgms2	sphingomyelin synthase 2 [Source:MGI Symbol;Acc	0.1474
ENSMUSG00000023971	Rrp36	ribosomal RNA processing 36 homolog (S. cerevisia	0.14744
ENSMUSG00000087943	Gm24245	predicted gene, 24245 [Source:MGI Symbol;Acc:M	0.14757
ENSMUSG00000003420	Fcgrt	Fc receptor, IgG, alpha chain transporter [Source:M	0.14762
ENSMUSG00000084949	Gm16249	predicted gene 16249 [Source:MGI Symbol;Acc:MG	0.14765
ENSMUSG00000031548	Sfrp1	secreted frizzled-related protein 1 [Source:MGI Syn	0.14771
ENSMUSG00000028556	Dock7	dedicator of cytokinesis 7 [Source:MGI Symbol;Acc:	0.14775

ENSMUSG00000037487	Ubr5	ubiquitin protein ligase E3 component n-recognin 5	0.1478
ENSMUSG00000037685	Atp8a1	ATPase, aminophospholipid transporter (APLT), clas	0.14785
ENSMUSG00000036989	Trim3	tripartite motif-containing 3 [Source:MGI Symbol;A	0.148
ENSMUSG00000097747	Gm26534	predicted gene, 26534 [Source:MGI Symbol;Acc:MG	0.14802
ENSMUSG00000045248	Med26	mediator complex subunit 26 [Source:MGI Symbol;	0.14808
ENSMUSG00000041731	Pgm5	phosphoglucomutase 5 [Source:MGI Symbol;Acc:M	0.14811
ENSMUSG00000047085	Lrrc4b	leucine rich repeat containing 4B [Source:MGI Syml	0.14813
ENSMUSG00000062960	Kdr	kinase insert domain protein receptor [Source:MGI	0.14815
ENSMUSG00000063457	Rps15	ribosomal protein S15 [Source:MGI Symbol;Acc:MG	0.14831
ENSMUSG00000098087	Gm17750	predicted gene, 17750 [Source:MGI Symbol;Acc:MG	0.14841
ENSMUSG00000006456	Rbm14	RNA binding motif protein 14 [Source:MGI Symbol;	0.14842
ENSMUSG00000040263	Klhdc4	kelch domain containing 4 [Source:MGI Symbol;Acc	0.14847
ENSMUSG00000067203	H2-K2	histocompatibility 2, K region locus 2 [Source:MGI S	0.14849
ENSMUSG00000004105	Angptl2	angiopoietin-like 2 [Source:MGI Symbol;Acc:MGI:1	0.14864
ENSMUSG00000060063	Alox5ap	arachidonate 5-lipoxygenase activating protein [So	0.14866
ENSMUSG00000056749	Nfil3	nuclear factor, interleukin 3, regulated [Source:MG	0.14891
ENSMUSG00000028655	Mfsd2a	major facilitator superfamily domain containing 2A	0.149
ENSMUSG00000079477	Rab7	RAB7, member RAS oncogene family [Source:MGI S	0.14902
ENSMUSG00000062380	Tubb3	tubulin, beta 3 class III [Source:MGI Symbol;Acc:MG	0.14903
ENSMUSG00000025409	Mbd6	methyl-CpG binding domain protein 6 [Source:MGI	0.14907
ENSMUSG00000024194	Cuta	cutA divalent cation tolerance homolog (E. coli) [So	0.14915
ENSMUSG00000025962	Fastkd2	FAST kinase domains 2 [Source:MGI Symbol;Acc:MG	0.14917
ENSMUSG00000025362	Rps26	ribosomal protein S26 [Source:MGI Symbol;Acc:MG	0.14919
ENSMUSG00000052302	Tbc1d30	TBC1 domain family, member 30 [Source:MGI Symb	0.14919
ENSMUSG00000022120	Rnf219	ring finger protein 219 [Source:MGI Symbol;Acc:MG	0.14926
ENSMUSG00000022758	P2rx6	purinergic receptor P2X, ligand-gated ion channel,	0.14927
ENSMUSG00000002289	Angptl4	angiopoietin-like 4 [Source:MGI Symbol;Acc:MGI:1	0.14929
ENSMUSG00000026825	Dnm1	dynamin 1 [Source:MGI Symbol;Acc:MGI:107384]	0.14931
ENSMUSG00000062012	Zfp13	zinc finger protein 13 [Source:MGI Symbol;Acc:MGI	0.14932
ENSMUSG00000082148	Gm12266	predicted gene 12266 [Source:MGI Symbol;Acc:MG	0.14934
ENSMUSG00000032081	Apoc3	apolipoprotein C-III [Source:MGI Symbol;Acc:MGI:8	0.14939
ENSMUSG00000024737	Slc15a3	solute carrier family 15, member 3 [Source:MGI Syr	0.14947
ENSMUSG00000023999	Kif6	kinesin family member 6 [Source:MGI Symbol;Acc:M	0.1495
ENSMUSG00000038188	Scarf1	scavenger receptor class F, member 1 [Source:MGI	0.14953
ENSMUSG00000029313	Aff1	AF4/FMR2 family, member 1 [Source:MGI Symbol;A	0.14954
ENSMUSG00000039634	Zfp189	zinc finger protein 189 [Source:MGI Symbol;Acc:MG	0.14956
ENSMUSG00000082500	Gm15195	predicted gene 15195 [Source:MGI Symbol;Acc:MG	0.1496
ENSMUSG00000004356	Utp20	UTP20, small subunit (SSU) processome componen	0.14962
ENSMUSG00000042087	4933440N22Rik	RIKEN cDNA 4933440N22 gene [Source:MGI Symbo	0.14962
ENSMUSG00000041748	Ackr4	atypical chemokine receptor 4 [Source:MGI Symbo	0.14963
ENSMUSG00000029147	Ppm1g	protein phosphatase 1G (formerly 2C), magnesium-	0.14964
ENSMUSG00000097039	Pvt1	plasmacytoma variant translocation 1 [Source:MGI	0.14965
ENSMUSG00000025857	Heatr2	HEAT repeat containing 2 [Source:MGI Symbol;Acc:	0.14976
ENSMUSG00000040154	Wfdc5	WAP four-disulfide core domain 5 [Source:MGI Sym	0.14977
ENSMUSG00000085329	2810404F17Rik	RIKEN cDNA 2810404F17 gene [Source:MGI Symbo	0.14978
ENSMUSG00000026827	Gpd2	glycerol phosphate dehydrogenase 2, mitochondria	0.14983
ENSMUSG00000056987	Fam71d	family with sequence similarity 71, member D [Sou	0.14983

ENSMUSG00000047804	Akap10	A kinase (PRKA) anchor protein 10 [Source:MGI Syn	0.14984
ENSMUSG00000036411	9530077C05Rik	RIKEN cDNA 9530077C05 gene [Source:MGI Symbo	0.14986
ENSMUSG00000084946	Dlx1os	distal-less homeobox 1, opposite strand [Source:M	0.14989
ENSMUSG00000002396	Ocel1	occludin/ELL domain containing 1 [Source:MGI Sym	0.14998
ENSMUSG00000028878	Fam76a	family with sequence similarity 76, member A [Sou	0.15004
ENSMUSG00000044968	Napepld	N-acyl phosphatidylethanolamine phospholipase D	0.15007
ENSMUSG00000072955	Tmsb15l	thymosin beta 15b like [Source:MGI Symbol;Acc:M	0.15008
ENSMUSG00000037490	Slc2a12	solute carrier family 2 (facilitated glucose transport	0.15024
ENSMUSG00000031994	Adamts8	a disintegrin-like and metallopeptidase (reprolysin	0.15026
ENSMUSG00000030519	Apba2	amyloid beta (A4) precursor protein-binding, family	0.15028
ENSMUSG00000021550	2210016F16Rik	RIKEN cDNA 2210016F16 gene [Source:MGI Symbo	0.15039
ENSMUSG00000037822	Smim14	small integral membrane protein 14 [Source:MGI S	0.15039
ENSMUSG00000054452	Aes	amino-terminal enhancer of split [Source:MGI Sym	0.15039
ENSMUSG00000086311	Gm12122	predicted gene 12122 [Source:MGI Symbol;Acc:MG	0.15051
ENSMUSG00000030431	Tmem238	transmembrane protein 238 [Source:MGI Symbol;A	0.15055
ENSMUSG00000000823	Znf512b	zinc finger protein 512B [Source:MGI Symbol;Acc:M	0.15057
ENSMUSG00000036718	Micall2	MICAL-like 2 [Source:MGI Symbol;Acc:MGI:244481	0.15073
ENSMUSG00000081143	Gm15823	predicted gene 15823 [Source:MGI Symbol;Acc:MG	0.15081
ENSMUSG00000084848	4933408N05Rik	RIKEN cDNA 4933408N05 gene [Source:MGI Symbo	0.15087
ENSMUSG00000024800	Rpp30	ribonuclease P/MRP 30 subunit [Source:MGI Symbo	0.15089
ENSMUSG00000085884	Gm15342	predicted gene 15342 [Source:MGI Symbol;Acc:MG	0.15089
ENSMUSG00000033055	Ankrd54	ankyrin repeat domain 54 [Source:MGI Symbol;Acc	0.151
ENSMUSG00000024269	Tpgs2	tubulin polyglutamylase complex subunit 2 [Source	0.15102
ENSMUSG00000039004	Bmp6	bone morphogenetic protein 6 [Source:MGI Symbo	0.15105
ENSMUSG00000043091	Tuba1c	tubulin, alpha 1C [Source:MGI Symbol;Acc:MGI:109	0.15105
ENSMUSG00000025885	Myo5b	myosin VB [Source:MGI Symbol;Acc:MGI:106598]	0.15113
ENSMUSG00000035594	Chrna5	cholinergic receptor, nicotinic, alpha polypeptide 5	0.15114
ENSMUSG00000095681	Gm8281	predicted gene, 8281 [Source:MGI Symbol;Acc:MG	0.15118
ENSMUSG00000060284	Sp7	Sp7 transcription factor 7 [Source:MGI Symbol;Acc:	0.15121
ENSMUSG00000032358	Fam83b	family with sequence similarity 83, member B [Sou	0.15124
ENSMUSG00000020072	Pbld1	phenazine biosynthesis-like protein domain contair	0.15126
ENSMUSG00000046822	Slc39a3	solute carrier family 39 (zinc transporter), member	0.15131
ENSMUSG00000032598	Nckipsd	NCK interacting protein with SH3 domain [Source:N	0.15135
ENSMUSG00000039891	Txlnb	taxilin beta [Source:MGI Symbol;Acc:MGI:2671945]	0.15135
ENSMUSG00000076612	Ighg2c	immunoglobulin heavy constant gamma 2C [Source	0.15138
ENSMUSG00000039952	Dag1	dystroglycan 1 [Source:MGI Symbol;Acc:MGI:10186	0.15143
ENSMUSG00000026135	Zfp142	zinc finger protein 142 [Source:MGI Symbol;Acc:M	0.15146
ENSMUSG00000041329	Atp1b2	ATPase, Na+/K+ transporting, beta 2 polypeptide [S	0.15153
ENSMUSG00000014503	Pkd2l2	polycystic kidney disease 2-like 2 [Source:MGI Sym	0.15155
ENSMUSG00000022353	Mtss1	metastasis suppressor 1 [Source:MGI Symbol;Acc:N	0.15156
ENSMUSG00000058831	Opn1sw	opsin 1 (cone pigments), short-wave-sensitive (colo	0.15168
ENSMUSG00000048186	Bend7	BEN domain containing 7 [Source:MGI Symbol;Acc:	0.15172
ENSMUSG00000038705	Gmeb2	glucocorticoid modulatory element binding protein	0.15173
ENSMUSG00000046691	Chtf8	CTF8, chromosome transmission fidelity factor 8 [S	0.15175
ENSMUSG00000032179	Bmp5	bone morphogenetic protein 5 [Source:MGI Symbo	0.15178
ENSMUSG00000004099	Dnmt1	DNA methyltransferase (cytosine-5) 1 [Source:MGI	0.15182
ENSMUSG00000020456	Ogdh	oxoglutarate (alpha-ketoglutarate) dehydrogenase	0.15191

ENSMUSG00000046854	Pip5kl1	phosphatidylinositol-4-phosphate 5-kinase-like 1 [S	0.15198
ENSMUSG00000097462	9530026P05Rik	RIKEN cDNA 9530026P05 gene [Source:MGI Symbo	0.15201
ENSMUSG00000093577	Gm20632	predicted gene 20632 [Source:MGI Symbol;Acc:MG	0.15203
ENSMUSG00000002297	Dbf4	DBF4 homolog (S. cerevisiae) [Source:MGI Symbol;A	0.1521
ENSMUSG00000020925	Ccdc43	coiled-coil domain containing 43 [Source:MGI Symb	0.1521
ENSMUSG00000092486	2610524H06Rik	RIKEN cDNA 2610524H06 gene [Source:MGI Symbo	0.1522
ENSMUSG00000003573	Homer3	homer homolog 3 (Drosophila) [Source:MGI Symbo	0.15224
ENSMUSG00000020059	Sycp3	synaptonemal complex protein 3 [Source:MGI Sym	0.15226
ENSMUSG00000059824	Dbp	D site albumin promoter binding protein [Source:M	0.15226
ENSMUSG00000022367	Has2	hyaluronan synthase 2 [Source:MGI Symbol;Acc:MG	0.15236
ENSMUSG00000033581	Igf2bp2	insulin-like growth factor 2 mRNA binding protein 2	0.15236
ENSMUSG00000021457	Syk	spleen tyrosine kinase [Source:MGI Symbol;Acc:MG	0.15245
ENSMUSG00000083268	Gm11511	predicted gene 11511 [Source:MGI Symbol;Acc:MG	0.15251
ENSMUSG00000031065	Cdk16	cyclin-dependent kinase 16 [Source:MGI Symbol;Ac	0.15257
ENSMUSG00000002205	Vrk3	vaccinia related kinase 3 [Source:MGI Symbol;Acc:M	0.1526
ENSMUSG00000087298	Gm9392	predicted pseudogene 9392 [Source:MGI Symbol;A	0.15262
ENSMUSG00000006169	Clint1	clathrin interactor 1 [Source:MGI Symbol;Acc:MGI:	0.15265
ENSMUSG00000020937	Plcd3	phospholipase C, delta 3 [Source:MGI Symbol;Acc:M	0.15283
ENSMUSG00000073569	Gm10540	predicted gene 10540 [Source:MGI Symbol;Acc:MG	0.15283
ENSMUSG00000056133	Gm9992	predicted gene 9992 [Source:MGI Symbol;Acc:MGI:	0.15286
ENSMUSG00000057177	Gsk3a	glycogen synthase kinase 3 alpha [Source:MGI Sym	0.15291
ENSMUSG00000001507	Itga3	integrin alpha 3 [Source:MGI Symbol;Acc:MGI:9660	0.15298
ENSMUSG00000021758	Ddx4	DEAD (Asp-Glu-Ala-Asp) box polypeptide 4 [Source:	0.15304
ENSMUSG00000094614	Gm21769	predicted gene, 21769 [Source:MGI Symbol;Acc:MG	0.15307
ENSMUSG00000027962	Vcam1	vascular cell adhesion molecule 1 [Source:MGI Sym	0.15308
ENSMUSG00000055917	Zfp277	zinc finger protein 277 [Source:MGI Symbol;Acc:MG	0.15314
ENSMUSG00000051067	Lingo3	leucine rich repeat and Ig domain containing 3 [Sou	0.15327
ENSMUSG00000061808	Ttr	transthyretin [Source:MGI Symbol;Acc:MGI:98865]	0.15328
ENSMUSG00000031725	Ces1f	carboxylesterase 1F [Source:MGI Symbol;Acc:MGI:2	0.15337
ENSMUSG00000093760	Gm20678	predicted gene 20678 [Source:MGI Symbol;Acc:MG	0.15345
ENSMUSG00000021143	Pacs2	phosphofurin acidic cluster sorting protein 2 [Sourc	0.15348
ENSMUSG00000041132	N4bp2l1	NEDD4 binding protein 2-like 1 [Source:MGI Symbo	0.15351
ENSMUSG00000046410	Kcnk6	potassium inwardly-rectifying channel, subfamily K,	0.15357
ENSMUSG00000038580	Sct	secretin [Source:MGI Symbol;Acc:MGI:99466]	0.1536
ENSMUSG00000029798	Herc6	hect domain and RLD 6 [Source:MGI Symbol;Acc:M	0.15364
ENSMUSG00000040372	Gpr63	G protein-coupled receptor 63 [Source:MGI Symbo	0.15367
ENSMUSG00000055831	Gm9982	predicted gene 9982 [Source:MGI Symbol;Acc:MGI:	0.15369
ENSMUSG00000091318	Gm5415	predicted gene 5415 [Source:MGI Symbol;Acc:MGI:	0.1537
ENSMUSG00000031351	Zfp185	zinc finger protein 185 [Source:MGI Symbol;Acc:MG	0.1538
ENSMUSG00000076039	Gm22137	predicted gene, 22137 [Source:MGI Symbol;Acc:MG	0.1538
ENSMUSG00000024805	Pcgf5	polycomb group ring finger 5 [Source:MGI Symbol;A	0.15383
ENSMUSG00000097609	Gm26659	predicted gene, 26659 [Source:MGI Symbol;Acc:MG	0.15384
ENSMUSG00000025013	Tll2	tolloid-like 2 [Source:MGI Symbol;Acc:MGI:134604	0.15389
ENSMUSG00000039202	Abhd2	abhydrolase domain containing 2 [Source:MGI Sym	0.15393
ENSMUSG00000032300	1700017B05Rik	RIKEN cDNA 1700017B05 gene [Source:MGI Symbo	0.15396
ENSMUSG00000028845	Tekt2	tektin 2 [Source:MGI Symbol;Acc:MGI:1346335]	0.15398
ENSMUSG00000000567	Sox9	SRY-box containing gene 9 [Source:MGI Symbol;Acc	0.154

ENSMUSG00000024491	Rbm27	RNA binding motif protein 27 [Source:MGI Symbol;	0.154
ENSMUSG00000028803	Nipal3	NIPA-like domain containing 3 [Source:MGI Symbol	0.15417
ENSMUSG00000042328	Hps4	Hermansky-Pudlak syndrome 4 homolog (human) [	0.15421
ENSMUSG00000052415	Tchh	trichohyalin [Source:MGI Symbol;Acc:MGI:2177944	0.15433
ENSMUSG00000029482	Aacs	acetoacetyl-CoA synthetase [Source:MGI Symbol;A	0.15443
ENSMUSG00000071644	Eef1g	eukaryotic translation elongation factor 1 gamma [	0.15444
ENSMUSG00000091665	Gm17101	predicted gene 17101 [Source:MGI Symbol;Acc:MG	0.15445
ENSMUSG00000030788	Rnf141	ring finger protein 141 [Source:MGI Symbol;Acc:M	0.15449
ENSMUSG00000091865	Gm17244	predicted gene, 17244 [Source:MGI Symbol;Acc:M	0.15449
ENSMUSG00000030604	Zfp626	zinc finger protein 626 [Source:MGI Symbol;Acc:M	0.1545
ENSMUSG00000093865	Lrit3	leucine-rich repeat, immunoglobulin-like and trans	0.15452
ENSMUSG00000030122	Ptms	parathymosin [Source:MGI Symbol;Acc:MGI:19164	0.15461
ENSMUSG00000094958	3110021N24Rik	RIKEN cDNA 3110021N24 gene [Source:MGI Symbo	0.15461
ENSMUSG00000037166	Ppp1r14a	protein phosphatase 1, regulatory (inhibitor) subun	0.15463
ENSMUSG00000041632	Mrps27	mitochondrial ribosomal protein S27 [Source:MGI S	0.15464
ENSMUSG00000022615	Tymp	thymidine phosphorylase [Source:MGI Symbol;Acc:	0.15471
ENSMUSG00000005417	Mprip	myosin phosphatase Rho interacting protein [Sourc	0.15489
ENSMUSG00000049922	Slc35c1	solute carrier family 35, member C1 [Source:MGI S	0.15492
ENSMUSG00000080850	Gm12439	predicted gene 12439 [Source:MGI Symbol;Acc:MG	0.15497
ENSMUSG00000028635	Edn2	endothelin 2 [Source:MGI Symbol;Acc:MGI:95284]	0.15498
ENSMUSG00000020808	Fam64a	family with sequence similarity 64, member A [Sou	0.15508
ENSMUSG00000028702	Rad54l	RAD54 like (S. cerevisiae) [Source:MGI Symbol;Acc:	0.1551
ENSMUSG00000057729	Prtn3	proteinase 3 [Source:MGI Symbol;Acc:MGI:893580	0.15518
ENSMUSG00000020930	Ccdc103	coiled-coil domain containing 103 [Source:MGI Sym	0.15526
ENSMUSG00000087355	Gm13187	predicted gene 13187 [Source:MGI Symbol;Acc:MG	0.15534
ENSMUSG00000038990	Cables2	CDK5 and Abl enzyme substrate 2 [Source:MGI Sym	0.15547
ENSMUSG00000046598	Bdh1	3-hydroxybutyrate dehydrogenase, type 1 [Source:	0.15552
ENSMUSG00000020098	Pcbd1	pterin 4 alpha carbinolamine dehydratase/dimeriza	0.15554
ENSMUSG00000025025	Mxi1	Max interacting protein 1 [Source:MGI Symbol;Acc:	0.15555
ENSMUSG00000043020	Wdr63	WD repeat domain 63 [Source:MGI Symbol;Acc:MG	0.15555
ENSMUSG00000075232	Amd1	S-adenosylmethionine decarboxylase 1 [Source:MG	0.15555
ENSMUSG00000081583	Gm14769	predicted gene 14769 [Source:MGI Symbol;Acc:MG	0.15555
ENSMUSG00000051853	Arf3	ADP-ribosylation factor 3 [Source:MGI Symbol;Acc:	0.15557
ENSMUSG00000058173	Smco4	single-pass membrane protein with coiled-coil dom	0.15561
ENSMUSG00000029238	Clock	circadian locomotor output cycles kaput [Source:M	0.15562
ENSMUSG00000079286	Gm11084	predicted gene 11084 [Source:MGI Symbol;Acc:MG	0.15572
ENSMUSG00000070304	Scn2b	sodium channel, voltage-gated, type II, beta [Sourc	0.15574
ENSMUSG00000046223	Plaur	plasminogen activator, urokinase receptor [Source:	0.15575
ENSMUSG00000026575	Nme7	NME/NM23 family member 7 [Source:MGI Symbol;	0.15576
ENSMUSG00000019975	Ikbip	IKBKB interacting protein [Source:MGI Symbol;Acc:	0.1558
ENSMUSG00000085118	Gm15774	predicted gene 15774 [Source:MGI Symbol;Acc:MG	0.15581
ENSMUSG00000046922	Gpr6	G protein-coupled receptor 6 [Source:MGI Symbol;	0.15587
ENSMUSG00000055944	E130018O15Rik	RIKEN cDNA E130018O15 gene [Source:MGI Symbo	0.15593
ENSMUSG00000021681	Aggf1	angiogenic factor with G patch and FHA domains 1	0.15598
ENSMUSG00000019986	Ahi1	Abelson helper integration site 1 [Source:MGI Symb	0.156
ENSMUSG00000024422	Dhx16	DEAH (Asp-Glu-Ala-His) box polypeptide 16 [Source	0.15605
ENSMUSG00000053093	Myh7	myosin, heavy polypeptide 7, cardiac muscle, beta	0.15605

ENSMUSG00000002985	Apoe	apolipoprotein E [Source:MGI Symbol;Acc:MGI:880	0.15607
ENSMUSG00000021000	Ctage5	CTAGE family, member 5 [Source:MGI Symbol;Acc:I	0.15614
ENSMUSG00000081534	Slc48a1	solute carrier family 48 (heme transporter), membe	0.15614
ENSMUSG00000036412	Arsi	arylsulfatase i [Source:MGI Symbol;Acc:MGI:26709	0.1562
ENSMUSG00000097035	Gm26692	predicted gene, 26692 [Source:MGI Symbol;Acc:M	0.15626
ENSMUSG00000020069	Hnrnph3	heterogeneous nuclear ribonucleoprotein H3 [Sour	0.15629
ENSMUSG00000029330	Cds1	CDP-diacylglycerol synthase 1 [Source:MGI Symbol;	0.1565
ENSMUSG00000070002	Ell	elongation factor RNA polymerase II [Source:MGI S	0.15654
ENSMUSG00000020412	Ascc2	activating signal cointegrator 1 complex subunit 2 [	0.15667
ENSMUSG00000083363	Gm12237	predicted gene 12237 [Source:MGI Symbol;Acc:MG	0.15675
ENSMUSG00000085389	1700003M07Rik	RIKEN cDNA 1700003M07 gene [Source:MGI Symbo	0.15683
ENSMUSG00000029416	Slc15a4	solute carrier family 15, member 4 [Source:MGI Sy	0.15686
ENSMUSG00000085395	Gm13056	predicted gene 13056 [Source:MGI Symbol;Acc:MG	0.1569
ENSMUSG00000086158	Gm5918	predicted gene 5918 [Source:MGI Symbol;Acc:MGI:	0.1569
ENSMUSG00000022131	Gpr180	G protein-coupled receptor 180 [Source:MGI Symb	0.15692
ENSMUSG00000024236	Svil	supervillin [Source:MGI Symbol;Acc:MGI:2147319]	0.15698
ENSMUSG00000024471	Myot	myotilin [Source:MGI Symbol;Acc:MGI:1889800]	0.15698
ENSMUSG00000038886	Man2a2	mannosidase 2, alpha 2 [Source:MGI Symbol;Acc:M	0.15705
ENSMUSG00000069565	Dazap1	DAZ associated protein 1 [Source:MGI Symbol;Acc:	0.15713
ENSMUSG00000031505	Carkd	carbohydrate kinase domain containing [Source:M	0.15714
ENSMUSG00000029345	Tfip11	tuftelin interacting protein 11 [Source:MGI Symbol;	0.15716
ENSMUSG00000031167	Rbm3	RNA binding motif protein 3 [Source:MGI Symbol;A	0.15721
ENSMUSG00000004460	Dnajb11	DnaJ (Hsp40) homolog, subfamily B, member 11 [S	0.15728
ENSMUSG00000032419	Tbx18	T-box18 [Source:MGI Symbol;Acc:MGI:1923615]	0.15732
ENSMUSG00000026773	Pfkfb3	6-phosphofructo-2-kinase/fructose-2,6-biphosphat	0.15736
ENSMUSG00000064141	Zfp69	zinc finger protein 69 [Source:MGI Symbol;Acc:MG	0.15736
ENSMUSG00000042709	Atpaf2	ATP synthase mitochondrial F1 complex assembly f	0.15738
ENSMUSG00000026637	Traf5	TNF receptor-associated factor 5 [Source:MGI Sym	0.15742
ENSMUSG00000041737	Tmem45b	transmembrane protein 45b [Source:MGI Symbol;A	0.15742
ENSMUSG00000087783	Gm23170	predicted gene, 23170 [Source:MGI Symbol;Acc:M	0.15746
ENSMUSG00000026640	Plxna2	plexin A2 [Source:MGI Symbol;Acc:MGI:107684]	0.15747
ENSMUSG00000049539	Hist1h1a	histone cluster 1, H1a [Source:MGI Symbol;Acc:MG	0.15748
ENSMUSG00000075122	Cd80	CD80 antigen [Source:MGI Symbol;Acc:MGI:10177	0.15748
ENSMUSG00000080440	Gm25848	predicted gene, 25848 [Source:MGI Symbol;Acc:M	0.15748
ENSMUSG00000003283	Hck	hemopoietic cell kinase [Source:MGI Symbol;Acc:M	0.15752
ENSMUSG00000035711	Dok3	docking protein 3 [Source:MGI Symbol;Acc:MGI:13	0.15755
ENSMUSG00000024924	Vldlr	very low density lipoprotein receptor [Source:MGI	0.15763
ENSMUSG00000028189	Ctbs	chitobiase, di-N-acetyl- [Source:MGI Symbol;Acc:M	0.15766
ENSMUSG00000097337	Gm26606	predicted gene, 26606 [Source:MGI Symbol;Acc:M	0.15779
ENSMUSG00000038515	Grtp1	GH regulated TBC protein 1 [Source:MGI Symbol;Ac	0.15788
ENSMUSG00000068860	Gm128	predicted gene 128 [Source:MGI Symbol;Acc:MGI:2	0.15796
ENSMUSG00000095891	Gm10717	predicted gene 10717 [Source:MGI Symbol;Acc:MG	0.15799
ENSMUSG00000015092	Edf1	endothelial differentiation-related factor 1 [Source	0.15806
ENSMUSG00000044026	Slc35g1	solute carrier family 35, member G1 [Source:MGI S	0.15808
ENSMUSG00000038708	Golga4	golgi autoantigen, golgin subfamily a, 4 [Source:MG	0.15812
ENSMUSG00000021670	Hmgcr	3-hydroxy-3-methylglutaryl-Coenzyme A reductase	0.15819
ENSMUSG00000000194	Gpr107	G protein-coupled receptor 107 [Source:MGI Symb	0.15825

ENSMUSG00000015484	Fam163a	family with sequence similarity 163, member A [Sou	0.15825
ENSMUSG00000026309	Ilkap	integrin-linked kinase-associated serine/threonine	0.15827
ENSMUSG00000085391	Gm16150	predicted gene 16150 [Source:MGI Symbol;Acc:MG	0.15829
ENSMUSG00000048878	Hexim1	hexamethylene bis-acetamide inducible 1 [Source:M	0.15835
ENSMUSG00000086775	5730588L14Rik	RIKEN cDNA 5730588L14 gene [Source:MGI Symbo	0.15839
ENSMUSG00000060314	Zfp941	zinc finger protein 941 [Source:MGI Symbol;Acc:MG	0.15842
ENSMUSG00000001420	Tmem79	transmembrane protein 79 [Source:MGI Symbol;Ac	0.15843
ENSMUSG00000067851	Arfgef1	ADP-ribosylation factor guanine nucleotide-exchan	0.15843
ENSMUSG00000037411	Serpine1	serine (or cysteine) peptidase inhibitor, clade E, me	0.15846
ENSMUSG00000015812	Gnrh1	gonadotropin releasing hormone 1 [Source:MGI Sy	0.15854
ENSMUSG00000022132	Cldn10	claudin 10 [Source:MGI Symbol;Acc:MGI:1913101]	0.15856
ENSMUSG00000033623	Pcgf3	polycomb group ring finger 3 [Source:MGI Symbol;	0.15862
ENSMUSG00000052760	A630001G21Rik	RIKEN cDNA A630001G21 gene [Source:MGI Symbo	0.15866
ENSMUSG00000032889	Gm6685	predicted pseudogene 6685 [Source:MGI Symbol;A	0.15869
ENSMUSG00000040785	Ttc3	tetratricopeptide repeat domain 3 [Source:MGI Sym	0.15877
ENSMUSG00000046798	Cldn12	claudin 12 [Source:MGI Symbol;Acc:MGI:1929288]	0.15879
ENSMUSG00000006356	Crip2	cysteine rich protein 2 [Source:MGI Symbol;Acc:MG	0.15881
ENSMUSG00000072972	Adam4	a disintegrin and metallopeptidase domain 4 [Sourc	0.15882
ENSMUSG00000002968	Med25	mediator of RNA polymerase II transcription, subur	0.15889
ENSMUSG00000031902	Nfatc3	nuclear factor of activated T cells, cytoplasmic, calc	0.159
ENSMUSG00000024006	Stk38	serine/threonine kinase 38 [Source:MGI Symbol;Ac	0.15901
ENSMUSG00000038005	2700029M09Rik	RIKEN cDNA 2700029M09 gene [Source:MGI Symbo	0.15908
ENSMUSG00000021149	Gtpbp4	GTP binding protein 4 [Source:MGI Symbol;Acc:MG	0.15911
ENSMUSG00000038648	Creb3l2	cAMP responsive element binding protein 3-like 2 [	0.15915
ENSMUSG00000002345	2310045N01Rik	RIKEN cDNA 2310045N01 gene [Source:MGI Symbo	0.15917
ENSMUSG00000039385	Cdh6	cadherin 6 [Source:MGI Symbol;Acc:MGI:107435]	0.15917
ENSMUSG00000063063	Ctnna2	catenin (cadherin associated protein), alpha 2 [Sou	0.15922
ENSMUSG00000053040	Aph1c	anterior pharynx defective 1c homolog (C. elegans)	0.15925
ENSMUSG00000026708	Cenpl	centromere protein L [Source:MGI Symbol;Acc:MG	0.15934
ENSMUSG00000005410	Mcm5	minichromosome maintenance deficient 5, cell divi	0.15935
ENSMUSG00000033805	Ephx4	epoxide hydrolase 4 [Source:MGI Symbol;Acc:MGI:	0.15935
ENSMUSG00000048230	Fbxo43	F-box protein 43 [Source:MGI Symbol;Acc:MGI:192	0.15938
ENSMUSG00000018171	Vmp1	vacuole membrane protein 1 [Source:MGI Symbol;	0.15941
ENSMUSG00000060279	Ap2a1	adaptor-related protein complex 2, alpha 1 subunit	0.15941
ENSMUSG00000061024	Rrs1	RRS1 ribosome biogenesis regulator homolog (S. ce	0.15941
ENSMUSG00000047182	Irs3	insulin receptor substrate 3 [Source:MGI Symbol;Ac	0.15949
ENSMUSG00000054702	Ap1s3	adaptor-related protein complex AP-1, sigma 3 [Sou	0.15957
ENSMUSG00000020692	Nle1	notchless homolog 1 (Drosophila) [Source:MGI Sym	0.15963
ENSMUSG00000051586	Mical3	microtubule associated monooxygenase, calponin a	0.15973
ENSMUSG00000019558	Slc6a8	solute carrier family 6 (neurotransmitter transporte	0.15975
ENSMUSG00000034042	Gpbp1l1	GC-rich promoter binding protein 1-like 1 [Source:M	0.15979
ENSMUSG00000032431	Crtap	cartilage associated protein [Source:MGI Symbol;Ac	0.15985
ENSMUSG00000020775	Mrpl38	mitochondrial ribosomal protein L38 [Source:MGI S	0.15991
ENSMUSG00000026623	Lpgat1	lysophosphatidylglycerol acyltransferase 1 [Source:	0.15992
ENSMUSG00000001039	B9d1	B9 protein domain 1 [Source:MGI Symbol;Acc:MGI:	0.15994
ENSMUSG00000031812	Map1lc3b	microtubule-associated protein 1 light chain 3 beta	0.16002
ENSMUSG00000086690	4933406J10Rik	RIKEN cDNA 4933406J10 gene [Source:MGI Symbol	0.16003

ENSMUSG00000031241	Tbx22	T-box 22 [Source:MGI Symbol;Acc:MGI:2389465]	0.16009
ENSMUSG00000023031	Cela1	chymotrypsin-like elastase family, member 1 [Sourc	0.16012
ENSMUSG00000032870	Smap2	small ArfGAP 2 [Source:MGI Symbol;Acc:MGI:1917(	0.16012
ENSMUSG00000054499	Dedd2	death effector domain-containing DNA binding prot	0.16031
ENSMUSG00000073856	Iqck	IQ motif containing K [Source:MGI Symbol;Acc:MGI	0.16032
ENSMUSG00000040435	Ppp1r15a	protein phosphatase 1, regulatory (inhibitor) subun	0.16038
ENSMUSG00000041084	Ostc	oligosaccharyltransferase complex subunit [Source:	0.16048
ENSMUSG00000085337	Gm15964	predicted gene 15964 [Source:MGI Symbol;Acc:MG	0.16052
ENSMUSG00000028927	Padi2	peptidyl arginine deiminase, type II [Source:MGI Sy	0.16054
ENSMUSG00000031489	Adrb3	adrenergic receptor, beta 3 [Source:MGI Symbol;Ac	0.16056
ENSMUSG00000003402	Prkcsh	protein kinase C substrate 80K-H [Source:MGI Symb	0.16061
ENSMUSG00000078960	Tob2	transducer of ERBB2, 2 [Source:MGI Symbol;Acc:M	0.16067
ENSMUSG00000096943	Gm26721	predicted gene, 26721 [Source:MGI Symbol;Acc:M(	0.16069
ENSMUSG00000037210	Fam193a	family with sequence similarity 193, member A [Sou	0.1607
ENSMUSG00000092607	Scnm1	sodium channel modifier 1 [Source:MGI Symbol;Ac	0.16073
ENSMUSG00000065431	Mir186	microRNA 186 [Source:MGI Symbol;Acc:MGI:26768	0.16076
ENSMUSG00000087482	Gm12720	predicted gene 12720 [Source:MGI Symbol;Acc:MG	0.16077
ENSMUSG00000037664	Cdkn1c	cyclin-dependent kinase inhibitor 1C (P57) [Source:	0.16088
ENSMUSG00000018599	Mief2	mitochondrial elongation factor 2 [Source:MGI Sym	0.16096
ENSMUSG00000028804	Csmd2	CUB and Sushi multiple domains 2 [Source:MGI Syn	0.16098
ENSMUSG00000051316	Taf7	TAF7 RNA polymerase II, TATA box binding protein	0.16104
ENSMUSG00000097383	1500026H17Rik	RIKEN cDNA 1500026H17 gene [Source:MGI Symbo	0.16106
ENSMUSG00000068740	Celsr2	cadherin, EGF LAG seven-pass G-type receptor 2 (fl	0.16109
ENSMUSG00000024357	Sil1	endoplasmic reticulum chaperone SIL1 homolog (S.	0.16112
ENSMUSG00000027082	Tfpi	tissue factor pathway inhibitor [Source:MGI Symbo	0.16119
ENSMUSG00000015829	Tnr	tenascin R [Source:MGI Symbol;Acc:MGI:99516]	0.16121
ENSMUSG00000027509	Rae1	RAE1 RNA export 1 homolog (S. pombe) [Source:M	0.16126
ENSMUSG00000069020	Urm1	ubiquitin related modifier 1 homolog (S. cerevisiae)	0.16136
ENSMUSG00000059430	Actg2	actin, gamma 2, smooth muscle, enteric [Source:M	0.1614
ENSMUSG00000034543	Morc2a	microrchidia 2A [Source:MGI Symbol;Acc:MGI:1921	0.16144
ENSMUSG00000029128	Rab28	RAB28, member RAS oncogene family [Source:MGI	0.16146
ENSMUSG00000060441	Trim5	tripartite motif-containing 5 [Source:MGI Symbol;A	0.16146
ENSMUSG00000050908	Tvp23a	trans-golgi network vesicle protein 23A [Source:MG	0.16151
ENSMUSG00000045598	Zfp553	zinc finger protein 553 [Source:MGI Symbol;Acc:M(	0.16152
ENSMUSG00000046623	Gjb4	gap junction protein, beta 4 [Source:MGI Symbol;A	0.16158
ENSMUSG00000024163	Mapk8ip3	mitogen-activated protein kinase 8 interacting prot	0.16163
ENSMUSG00000044288	Cnr1	cannabinoid receptor 1 (brain) [Source:MGI Symbo	0.16163
ENSMUSG00000048007	Timm8a1	translocase of inner mitochondrial membrane 8A1	0.16166
ENSMUSG00000027404	Snrpb	small nuclear ribonucleoprotein B [Source:MGI Sym	0.16168
ENSMUSG00000056174	Col8a2	collagen, type VIII, alpha 2 [Source:MGI Symbol;Acc	0.16168
ENSMUSG00000029094	Afap1	actin filament associated protein 1 [Source:MGI Syr	0.16174
ENSMUSG00000055817	Mta3	metastasis associated 3 [Source:MGI Symbol;Acc:M	0.16184
ENSMUSG00000086588	Gm11729	predicted gene 11729 [Source:MGI Symbol;Acc:MG	0.1619
ENSMUSG00000041617	Ccdc74a	coiled-coil domain containing 74A [Source:MGI Sym	0.16192
ENSMUSG00000051147	Nat2	N-acetyltransferase 2 (arylamine N-acetyltransferas	0.16193
ENSMUSG00000033857	Engase	endo-beta-N-acetylglucosaminidase [Source:MGI S	0.162
ENSMUSG00000034800	Zfp661	zinc finger protein 661 [Source:MGI Symbol;Acc:M(	0.16205

ENSMUSG00000097313	Gm26569	predicted gene, 26569 [Source:MGI Symbol;Acc:MG	0.16208
ENSMUSG00000031129	Slc9a9	solute carrier family 9 (sodium/hydrogen exchange	0.16214
ENSMUSG00000068328	Aup1	ancient ubiquitous protein 1 [Source:MGI Symbol;A	0.16223
ENSMUSG00000029581	Fscn1	fascin homolog 1, actin bundling protein (Strongylo	0.16226
ENSMUSG00000066900	Suds3	suppressor of defective silencing 3 homolog (S. cere	0.16229
ENSMUSG00000079070	Gm3985	predicted gene 3985 [Source:MGI Symbol;Acc:MGI:	0.16233
ENSMUSG00000055723	Rras2	related RAS viral (r-ras) oncogene homolog 2 [Sourc	0.16235
ENSMUSG00000044456	Rin3	Ras and Rab interactor 3 [Source:MGI Symbol;Acc:M	0.16236
ENSMUSG00000029660	Tex26	testis expressed 26 [Source:MGI Symbol;Acc:MGI:1	0.16237
ENSMUSG00000087672	Gm15122	predicted gene 15122 [Source:MGI Symbol;Acc:MG	0.16237
ENSMUSG00000028108	Ecm1	extracellular matrix protein 1 [Source:MGI Symbol;	0.16238
ENSMUSG00000024563	Smad2	SMAD family member 2 [Source:MGI Symbol;Acc:M	0.16239
ENSMUSG00000015970	Chdh	choline dehydrogenase [Source:MGI Symbol;Acc:M	0.16242
ENSMUSG00000025816	Sec61a2	Sec61, alpha subunit 2 (S. cerevisiae) [Source:MGI S	0.16244
ENSMUSG00000085730	Gm13307	predicted gene 13307 [Source:MGI Symbol;Acc:MG	0.16247
ENSMUSG00000087283	Gm13580	predicted gene 13580 [Source:MGI Symbol;Acc:MG	0.16247
ENSMUSG00000022443	Myh9	myosin, heavy polypeptide 9, non-muscle [Source:M	0.16249
ENSMUSG00000047129	1700113H08Rik	RIKEN cDNA 1700113H08 gene [Source:MGI Symbo	0.16249
ENSMUSG00000080974	Gm9009	predicted pseudogene 9009 [Source:MGI Symbol;A	0.16257
ENSMUSG00000087326	Gm12503	predicted gene 12503 [Source:MGI Symbol;Acc:MG	0.16258
ENSMUSG00000029546	Uncx	UNC homeobox [Source:MGI Symbol;Acc:MGI:108	0.16259
ENSMUSG00000078183	Gm15610	predicted gene 15610 [Source:MGI Symbol;Acc:MG	0.16274
ENSMUSG00000096952	Gm26572	predicted gene, 26572 [Source:MGI Symbol;Acc:MG	0.16277
ENSMUSG00000017404	Rpl19	ribosomal protein L19 [Source:MGI Symbol;Acc:MG	0.16279
ENSMUSG00000022679	Mpv17l	Mpv17 transgene, kidney disease mutant-like [Sour	0.16283
ENSMUSG00000040472	Rabggta	Rab geranylgeranyl transferase, a subunit [Source:M	0.16284
ENSMUSG00000097876	Gm16892	predicted gene, 16892 [Source:MGI Symbol;Acc:MG	0.16287
ENSMUSG00000048376	F2r	coagulation factor II (thrombin) receptor [Source:M	0.16289
ENSMUSG00000057969	Sema3b	sema domain, immunoglobulin domain (Ig), short b	0.16291
ENSMUSG00000085946	AA387200	expressed sequence AA387200 [Source:MGI Symbo	0.16298
ENSMUSG00000093650	Gm20631	predicted gene 20631 [Source:MGI Symbol;Acc:MG	0.16301
ENSMUSG00000000983	Wfdc18	WAP four-disulfide core domain 18 [Source:MGI Sy	0.16307
ENSMUSG00000022312	Eif3h	eukaryotic translation initiation factor 3, subunit H	0.16311
ENSMUSG00000013584	Aldh1a2	aldehyde dehydrogenase family 1, subfamily A2 [So	0.16314
ENSMUSG00000019528	Gyg	glycogenin [Source:MGI Symbol;Acc:MGI:1351614]	0.16314
ENSMUSG00000031409	Tceal6	transcription elongation factor A (SII)-like 6 [Source	0.16316
ENSMUSG00000025813	Homer2	homer homolog 2 (Drosophila) [Source:MGI Symbo	0.16317
ENSMUSG00000041552	Ptchd1	patched domain containing 1 [Source:MGI Symbol;	0.16318
ENSMUSG00000035104	Eva1a	eva-1 homolog A (C. elegans) [Source:MGI Symbol;	0.16327
ENSMUSG00000087180	Gm11924	predicted gene 11924 [Source:MGI Symbol;Acc:MG	0.16329
ENSMUSG00000026532	Spta1	spectrin alpha, erythrocytic 1 [Source:MGI Symbol;	0.16334
ENSMUSG00000087125	A230108P19Rik	RIKEN cDNA A230108P19 gene [Source:MGI Symbo	0.16335
ENSMUSG00000092625	Gm20404	predicted gene 20404 [Source:MGI Symbol;Acc:MG	0.16336
ENSMUSG00000020455	Trim11	tripartite motif-containing 11 [Source:MGI Symbol;	0.16338
ENSMUSG00000034744	Nagk	N-acetylglucosamine kinase [Source:MGI Symbol;A	0.1634
ENSMUSG00000016206	H2-M3	histocompatibility 2, M region locus 3 [Source:MGI	0.16344
ENSMUSG00000001497	Pax9	paired box gene 9 [Source:MGI Symbol;Acc:MGI:97	0.16351

ENSMUSG00000057093	C030039L03Rik	RIKEN cDNA C030039L03 gene [Source:MGI Symbo	0.16353
ENSMUSG00000025921	Rdh10	retinol dehydrogenase 10 (all-trans) [Source:MGI Sy	0.16355
ENSMUSG00000074876	Spata5l1	spermatogenesis associated 5-like 1 [Source:MGI Sy	0.16357
ENSMUSG00000043460	Elfn2	leucine rich repeat and fibronectin type III, extracel	0.16362
ENSMUSG00000016493	Cd46	CD46 antigen, complement regulatory protein [Sou	0.16366
ENSMUSG00000039713	Plekhg5	pleckstrin homology domain containing, family G (w	0.16368
ENSMUSG00000047419	Cmya5	cardiomyopathy associated 5 [Source:MGI Symbol;	0.16371
ENSMUSG00000056758	Hmga2	high mobility group AT-hook 2 [Source:MGI Symbol	0.16371
ENSMUSG00000080780	Gm11252	predicted gene 11252 [Source:MGI Symbol;Acc:MG	0.16371
ENSMUSG00000020585	Laptm4a	lysosomal-associated protein transmembrane 4A [S	0.16379
ENSMUSG00000019849	Prep	prolyl endopeptidase [Source:MGI Symbol;Acc:MGI	0.16393
ENSMUSG00000027824	Vmn2r1	vomeronasal 2, receptor 1 [Source:MGI Symbol;Acc	0.16404
ENSMUSG00000039813	Tbc1d2	TBC1 domain family, member 2 [Source:MGI Symbo	0.16407
ENSMUSG00000021067	Sav1	salvador homolog 1 (Drosophila) [Source:MGI Symb	0.16417
ENSMUSG00000025204	Ndufb8	NADH dehydrogenase (ubiquinone) 1 beta subcom	0.16418
ENSMUSG00000016528	Mapkapk2	MAP kinase-activated protein kinase 2 [Source:MG	0.16421
ENSMUSG00000026464	Zc3h11a	zinc finger CCCH type containing 11A [Source:MGI S	0.16427
ENSMUSG00000097406	Gm26833	predicted gene, 26833 [Source:MGI Symbol;Acc:MG	0.16431
ENSMUSG00000048915	Efna5	ephrin A5 [Source:MGI Symbol;Acc:MGI:107444]	0.16436
ENSMUSG00000043998	Mgat2	mannoside acetylglucosaminyltransferase 2 [Source	0.16437
ENSMUSG00000066652	Lefty2	left-right determination factor 2 [Source:MGI Symb	0.16437
ENSMUSG00000024299	Adamts10	a disintegrin-like and metallopeptidase (reprolysin t	0.16442
ENSMUSG00000019927	Ube2d1	ubiquitin-conjugating enzyme E2D 1 [Source:MGI S	0.16443
ENSMUSG00000021257	Angel1	angel homolog 1 (Drosophila) [Source:MGI Symbol;	0.16443
ENSMUSG00000015176	Nolc1	nucleolar and coiled-body phosphoprotein 1 [Sourc	0.16444
ENSMUSG00000038984	Tspyl5	testis-specific protein, Y-encoded-like 5 [Source:MG	0.16444
ENSMUSG00000096981	Gm16845	predicted gene, 16845 [Source:MGI Symbol;Acc:MG	0.16445
ENSMUSG00000015950	Ncf1	neutrophil cytosolic factor 1 [Source:MGI Symbol;A	0.16446
ENSMUSG00000009905	Kdsr	3-ketodihydrosphingosine reductase [Source:MGI S	0.16447
ENSMUSG00000027765	P2ry1	purinergic receptor P2Y, G-protein coupled 1 [Sourc	0.16447
ENSMUSG00000034258	Mfsd7c	major facilitator superfamily domain containing 7C	0.16453
ENSMUSG00000090213	Tmem189	transmembrane protein 189 [Source:MGI Symbol;A	0.16453
ENSMUSG00000022701	2610015P09Rik	RIKEN cDNA 2610015P09 gene [Source:MGI Symbo	0.16456
ENSMUSG00000021569	Trip13	thyroid hormone receptor interactor 13 [Source:MG	0.16457
ENSMUSG00000021660	Btf3	basic transcription factor 3 [Source:MGI Symbol;Ac	0.16471
ENSMUSG00000044583	Tlr7	toll-like receptor 7 [Source:MGI Symbol;Acc:MGI:21	0.16475
ENSMUSG00000031375	Bgn	biglycan [Source:MGI Symbol;Acc:MGI:88158]	0.16476
ENSMUSG00000031399	Fam3a	family with sequence similarity 3, member A [Sourc	0.16478
ENSMUSG00000081189	Gm12725	predicted gene 12725 [Source:MGI Symbol;Acc:MG	0.16478
ENSMUSG00000022002	4930564B18Rik	RIKEN cDNA 4930564B18 gene [Source:MGI Symbo	0.16481
ENSMUSG00000088950	Gm24939	predicted gene, 24939 [Source:MGI Symbol;Acc:MG	0.16487
ENSMUSG00000021606	Ndufs6	NADH dehydrogenase (ubiquinone) Fe-S protein 6 [	0.1649
ENSMUSG00000033020	Polr2f	polymerase (RNA) II (DNA directed) polypeptide F [	0.1649
ENSMUSG00000020706	Ftsj3	FtsJ homolog 3 (E. coli) [Source:MGI Symbol;Acc:M	0.16492
ENSMUSG00000024457	Trim26	tripartite motif-containing 26 [Source:MGI Symbol;	0.16495
ENSMUSG00000030319	Cand2	cullin-associated and neddylation-dissociated 2 (pu	0.165
ENSMUSG00000028644	Ermap	erythroblast membrane-associated protein [Source	0.16503

ENSMUSG00000029066	Mrpl20	mitochondrial ribosomal protein L20 [Source:MGI S	0.16506
ENSMUSG00000037706	Cd81	CD81 antigen [Source:MGI Symbol;Acc:MGI:109639	0.16511
ENSMUSG00000042985	Upk3b	uroplakin 3B [Source:MGI Symbol;Acc:MGI:214088	0.16518
ENSMUSG00000086118	Gm14169	predicted gene 14169 [Source:MGI Symbol;Acc:MG	0.16519
ENSMUSG00000019471	Cdc37	cell division cycle 37 [Source:MGI Symbol;Acc:MGI:	0.16525
ENSMUSG00000030435	U2af2	U2 small nuclear ribonucleoprotein auxiliary factor	0.16525
ENSMUSG00000014470	Rnf166	ring finger protein 166 [Source:MGI Symbol;Acc:MG	0.16526
ENSMUSG00000032262	Elovl4	elongation of very long chain fatty acids (FEN1/Elo2	0.16531
ENSMUSG00000022753	Tmem30c	transmembrane protein 30C [Source:MGI Symbol;A	0.16541
ENSMUSG00000046321	Hs3st2	heparan sulfate (glucosamine) 3-O-sulfotransferase	0.16546
ENSMUSG00000035215	Lsm7	LSM7 homolog, U6 small nuclear RNA associated (S	0.16558
ENSMUSG00000026303	Mlph	melanophilin [Source:MGI Symbol;Acc:MGI:217638	0.16562
ENSMUSG00000038388	Mpp6	membrane protein, palmitoylated 6 (MAGUK p55 s	0.16579
ENSMUSG00000046240	Hepacam	hepatocyte cell adhesion molecule [Source:MGI Syr	0.16585
ENSMUSG00000093239	Gm24106	predicted gene, 24106 [Source:MGI Symbol;Acc:MG	0.16592
ENSMUSG00000097773	Gm10614	predicted gene 10614 [Source:MGI Symbol;Acc:MG	0.16592
ENSMUSG00000092272	Gm19301	predicted gene, 19301 [Source:MGI Symbol;Acc:MG	0.16596
ENSMUSG00000028609	Magoh	mago-nashi homolog, proliferation-associated (Dro	0.16598
ENSMUSG00000060961	Slc4a4	solute carrier family 4 (anion exchanger), member 4	0.16598
ENSMUSG00000028793	Rnf19b	ring finger protein 19B [Source:MGI Symbol;Acc:MG	0.16609
ENSMUSG00000031871	Cdh5	cadherin 5 [Source:MGI Symbol;Acc:MGI:105057]	0.1661
ENSMUSG00000031832	Taf1c	TATA box binding protein (Tbp)-associated factor, R	0.16613
ENSMUSG00000046688	Tifa	TRAF-interacting protein with forkhead-associated	0.16619
ENSMUSG00000002658	Gtf2f1	general transcription factor IIF, polypeptide 1 [Sour	0.16623
ENSMUSG00000032673	Prorsd1	prolyl-tRNA synthetase domain containing 1 [Sourc	0.16623
ENSMUSG00000024743	Syt7	synaptotagmin VII [Source:MGI Symbol;Acc:MGI:18	0.16631
ENSMUSG00000022667	Cd200r1	CD200 receptor 1 [Source:MGI Symbol;Acc:MGI:18	0.16638
ENSMUSG00000024477	Pggt1b	protein geranylgeranyltransferase type I, beta subu	0.16639
ENSMUSG00000086843	E030013I19Rik	RIKEN cDNA E030013I19 gene [Source:MGI Symbol	0.16641
ENSMUSG00000031410	Nxf7	nuclear RNA export factor 7 [Source:MGI Symbol;A	0.16654
ENSMUSG00000026837	Col5a1	collagen, type V, alpha 1 [Source:MGI Symbol;Acc:M	0.16656
ENSMUSG00000097138	Gm26625	predicted gene, 26625 [Source:MGI Symbol;Acc:MG	0.16669
ENSMUSG00000043384	Gprasp1	G protein-coupled receptor associated sorting prot	0.1667
ENSMUSG00000042251	Pm20d1	peptidase M20 domain containing 1 [Source:MGI Sy	0.16672
ENSMUSG00000079260	Tmppe	transmembrane protein with metallophosphoester	0.16676
ENSMUSG00000061032	Rrp1	ribosomal RNA processing 1 homolog (S. cerevisiae	0.16679
ENSMUSG00000094500	Smim18	small integral membrane protein 18 [Source:MGI Sy	0.16679
ENSMUSG00000046516	Cox17	cytochrome c oxidase assembly protein 17 [Source:	0.16681
ENSMUSG00000020903	Stx8	syntaxin 8 [Source:MGI Symbol;Acc:MGI:1890156]	0.16688
ENSMUSG00000028782	Bai2	brain-specific angiogenesis inhibitor 2 [Source:MGI	0.1669
ENSMUSG00000029097	Trmt44	tRNA methyltransferase 44 [Source:MGI Symbol;Ac	0.16702
ENSMUSG00000037705	Tecta	tectorin alpha [Source:MGI Symbol;Acc:MGI:10957	0.16702
ENSMUSG00000039943	Plcb4	phospholipase C, beta 4 [Source:MGI Symbol;Acc:M	0.16703
ENSMUSG00000036327	Qsox2	quiescin Q6 sulfhydryl oxidase 2 [Source:MGI Symb	0.16709
ENSMUSG00000018363	Smurf2	SMAD specific E3 ubiquitin protein ligase 2 [Source	0.1671
ENSMUSG00000001103	Sebox	SEBOX homeobox [Source:MGI Symbol;Acc:MGI:10	0.16713
ENSMUSG00000012443	Kif11	kinesin family member 11 [Source:MGI Symbol;Acc	0.16714

ENSMUSG00000031360	Ctps2	cytidine 5'-triphosphate synthase 2 [Source:MGI Sy	0.16714
ENSMUSG00000054165	4922502B01Rik	RIKEN cDNA 4922502B01 gene [Source:MGI Symbo	0.16723
ENSMUSG00000032478	Nme6	NME/NM23 nucleoside diphosphate kinase 6 [Sour	0.16726
ENSMUSG00000020978	Klhdc2	kelch domain containing 2 [Source:MGI Symbol;Acc	0.16735
ENSMUSG00000021040	Slirp	SRA stem-loop interacting RNA binding protein [Sou	0.16737
ENSMUSG00000042686	Jph1	junctophilin 1 [Source:MGI Symbol;Acc:MGI:18914	0.16742
ENSMUSG00000047757	Fancb	Fanconi anemia, complementation group B [Source	0.16742
ENSMUSG00000079681	Zglp1	zinc finger, GATA-like protein 1 [Source:MGI Symbo	0.16744
ENSMUSG00000022295	Atp6v1c1	ATPase, H+ transporting, lysosomal V1 subunit C1 [	0.16745
ENSMUSG00000024381	Bin1	bridging integrator 1 [Source:MGI Symbol;Acc:MGI:	0.16748
ENSMUSG00000078879	Gm14306	predicted gene 14306 [Source:MGI Symbol;Acc:MG	0.16748
ENSMUSG00000003484	Cyp4f18	cytochrome P450, family 4, subfamily f, polypeptide	0.1675
ENSMUSG00000029053	Prkcz	protein kinase C, zeta [Source:MGI Symbol;Acc:MG	0.16755
ENSMUSG00000032092	Mpzl2	myelin protein zero-like 2 [Source:MGI Symbol;Acc	0.16756
ENSMUSG00000006673	Qrich1	glutamine-rich 1 [Source:MGI Symbol;Acc:MGI:191	0.1676
ENSMUSG00000053877	Srcap	Snf2-related CREBBP activator protein [Source:MGI	0.16771
ENSMUSG00000052942	Glis3	GLIS family zinc finger 3 [Source:MGI Symbol;Acc:M	0.16777
ENSMUSG00000039738	Slx4	SLX4 structure-specific endonuclease subunit homo	0.16778
ENSMUSG00000033272	Slc35a4	solute carrier family 35, member A4 [Source:MGI Sy	0.16785
ENSMUSG00000044626	Liph	lipase, member H [Source:MGI Symbol;Acc:MGI:23	0.16791
ENSMUSG00000074238	Ap1ar	adaptor-related protein complex 1 associated regul	0.16793
ENSMUSG00000001768	Rin2	Ras and Rab interactor 2 [Source:MGI Symbol;Acc:M	0.16803
ENSMUSG00000021023	1110008L16Rik	RIKEN cDNA 1110008L16 gene [Source:MGI Symbo	0.16805
ENSMUSG00000032332	Col12a1	collagen, type XII, alpha 1 [Source:MGI Symbol;Acc:	0.16811
ENSMUSG00000073805	Fam196a	family with sequence similarity 196, member A [Sou	0.16814
ENSMUSG00000071724	Smpd5	sphingomyelin phosphodiesterase 5 [Source:MGI Sy	0.1682
ENSMUSG00000064797	Gm24357	predicted gene, 24357 [Source:MGI Symbol;Acc:MG	0.16823
ENSMUSG00000027879	Sec22b	SEC22 vesicle trafficking protein homolog B (S. cere	0.16824
ENSMUSG00000082198	Gm14504	predicted gene 14504 [Source:MGI Symbol;Acc:MG	0.16824
ENSMUSG00000028857	Tmem222	transmembrane protein 222 [Source:MGI Symbol;A	0.16825
ENSMUSG00000085929	Gm13421	predicted gene 13421 [Source:MGI Symbol;Acc:MG	0.16832
ENSMUSG00000020114	Cand1	cullin associated and neddylation disassociated 1 [S	0.16835
ENSMUSG00000091811	Prr24	proline rich 24 [Source:MGI Symbol;Acc:MGI:19135	0.16839
ENSMUSG00000096727	Psmb9	proteasome (prosome, macropain) subunit, beta ty	0.16844
ENSMUSG00000027971	Ndst4	N-deacetylase/N-sulfotransferase (heparin glucosa	0.16845
ENSMUSG00000089922	3110039M20Rik	RIKEN cDNA 3110039M20 gene [Source:MGI Symbo	0.16848
ENSMUSG00000091251	Kiss1	KiSS-1 metastasis-suppressor [Source:MGI Symbol;A	0.16851
ENSMUSG00000086013	Gm15706	predicted gene 15706 [Source:MGI Symbol;Acc:MG	0.1686
ENSMUSG00000044429	Cryga	crystallin, gamma A [Source:MGI Symbol;Acc:MGI:8	0.16869
ENSMUSG00000057863	Rpl36	ribosomal protein L36 [Source:MGI Symbol;Acc:MG	0.16872
ENSMUSG00000049739	Zfp646	zinc finger protein 646 [Source:MGI Symbol;Acc:MG	0.16876
ENSMUSG00000006360	Crip1	cysteine-rich protein 1 (intestinal) [Source:MGI Sym	0.16886
ENSMUSG00000001930	Vwf	Von Willebrand factor homolog [Source:MGI Symbo	0.16899
ENSMUSG00000062961	Ccdc177	coiled-coil domain containing 177 [Source:MGI Sym	0.16902
ENSMUSG00000055900	Tmem69	transmembrane protein 69 [Source:MGI Symbol;Ac	0.16905
ENSMUSG00000091393	5330438I03Rik	RIKEN cDNA 5330438I03 gene [Source:MGI Symbol	0.16905
ENSMUSG00000031549	Ido2	indoleamine 2,3-dioxygenase 2 [Source:MGI Symbo	0.16907

ENSMUSG00000050222	Il17d	interleukin 17D [Source:MGI Symbol;Acc:MGI:2446	0.16917
ENSMUSG00000021703	Serinc5	serine incorporator 5 [Source:MGI Symbol;Acc:MGI	0.1692
ENSMUSG00000079003	Samd1	sterile alpha motif domain containing 1 [Source:MG	0.16921
ENSMUSG00000052316	Lrrc15	leucine rich repeat containing 15 [Source:MGI Syml	0.16923
ENSMUSG00000097625	Gm26561	predicted gene, 26561 [Source:MGI Symbol;Acc:MG	0.16926
ENSMUSG00000045350	Fam186a	family with sequence similarity 186, member A [Sou	0.16929
ENSMUSG00000098192	Gm3534	predicted pseudogene 3534 [Source:MGI Symbol;A	0.16934
ENSMUSG00000029563	Foxp2	forkhead box P2 [Source:MGI Symbol;Acc:MGI:214	0.16938
ENSMUSG00000097793	Gm17259	predicted gene, 17259 [Source:MGI Symbol;Acc:MG	0.16938
ENSMUSG00000058794	Nfe2	nuclear factor, erythroid derived 2 [Source:MGI Syr	0.16939
ENSMUSG00000003360	Ddx23	DEAD (Asp-Glu-Ala-Asp) box polypeptide 23 [Source	0.16944
ENSMUSG00000078768	Zfp566	zinc finger protein 566 [Source:MGI Symbol;Acc:MG	0.16946
ENSMUSG00000026193	Fn1	fibronectin 1 [Source:MGI Symbol;Acc:MGI:95566]	0.16958
ENSMUSG00000093561	Gm20699	predicted gene 20699 [Source:MGI Symbol;Acc:MG	0.16959
ENSMUSG00000030093	Wnt7a	wingless-related MMTV integration site 7A [Source	0.16969
ENSMUSG00000047443	Fam132b	family with sequence similarity 132, member B [Sou	0.16972
ENSMUSG00000028437	Ubap1	ubiquitin-associated protein 1 [Source:MGI Symbol	0.16979
ENSMUSG00000017778	Cox7c	cytochrome c oxidase subunit VIIc [Source:MGI Syn	0.16981
ENSMUSG00000022540	Rogdi	rogdi homolog (Drosophila) [Source:MGI Symbol;Ac	0.16989
ENSMUSG00000028182	Lrriq3	leucine-rich repeats and IQ motif containing 3 [Sou	0.16989
ENSMUSG00000096010	Hist4h4	histone cluster 4, H4 [Source:MGI Symbol;Acc:MGI:	0.1699
ENSMUSG00000027456	Sdcbp2	syndecan binding protein (syntenin) 2 [Source:MGI	0.16991
ENSMUSG00000071180	Smim15	small integral membrane protein 15 [Source:MGI S	0.16992
ENSMUSG00000038615	Nfe2l1	nuclear factor, erythroid derived 2,-like 1 [Source:N	0.16996
ENSMUSG00000036169	Sostdc1	sclerostin domain containing 1 [Source:MGI Symbo	0.16997
ENSMUSG00000092765	Gm23363	predicted gene, 23363 [Source:MGI Symbol;Acc:MG	0.17
ENSMUSG00000073045	Gm5570	predicted gene 5570 [Source:MGI Symbol;Acc:MGI:	0.17002
ENSMUSG00000086881	Gm13594	predicted gene 13594 [Source:MGI Symbol;Acc:MG	0.17004
ENSMUSG00000042846	Lrrtm3	leucine rich repeat transmembrane neuronal 3 [Sou	0.17008
ENSMUSG00000033228	Scaf11	SR-related CTD-associated factor 11 [Source:MGI Sy	0.1701
ENSMUSG00000006313	Upk1a	uroplakin 1A [Source:MGI Symbol;Acc:MGI:98911]	0.17014
ENSMUSG00000051029	Serpinb1b	serine (or cysteine) peptidase inhibitor, clade B, me	0.17021
ENSMUSG00000009292	Trpm2	transient receptor potential cation channel, subfam	0.17025
ENSMUSG00000050035	Fhl4	four and a half LIM domains 4 [Source:MGI Symbol	0.17026
ENSMUSG00000023439	Gnb3	guanine nucleotide binding protein (G protein), bet	0.17027
ENSMUSG00000028699	Tspan1	tetraspanin 1 [Source:MGI Symbol;Acc:MGI:191405	0.17031
ENSMUSG00000030641	4632434I11Rik	RIKEN cDNA 4632434I11 gene [Source:MGI Symbol	0.17033
ENSMUSG00000020205	Phlda1	pleckstrin homology-like domain, family A, membe	0.1704
ENSMUSG00000030103	Bhlhe40	basic helix-loop-helix family, member e40 [Source:I	0.17041
ENSMUSG00000086900	Gm16040	predicted gene 16040 [Source:MGI Symbol;Acc:MG	0.17049
ENSMUSG00000026723	Trdmt1	tRNA aspartic acid methyltransferase 1 [Source:MG	0.17057
ENSMUSG00000078670	Fam174b	family with sequence similarity 174, member B [Sou	0.17064
ENSMUSG00000068739	Sars	seryl-aminoacyl-tRNA synthetase [Source:MGI Sym	0.17074
ENSMUSG00000080364	Gm25777	predicted gene, 25777 [Source:MGI Symbol;Acc:MG	0.1708
ENSMUSG00000002055	Spag5	sperm associated antigen 5 [Source:MGI Symbol;Ac	0.17083
ENSMUSG00000046402	Rbp1	retinol binding protein 1, cellular [Source:MGI Syml	0.17083
ENSMUSG00000060240	Cend1	cell cycle exit and neuronal differentiation 1 [Sourc	0.17083

ENSMUSG00000024378	Stard4	StAR-related lipid transfer (START) domain containi	0.17085
ENSMUSG00000028600	Podn	podocan [Source:MGI Symbol;Acc:MGI:2674939]	0.17085
ENSMUSG00000046999	1110032F04Rik	RIKEN cDNA 1110032F04 gene [Source:MGI Symbo	0.1709
ENSMUSG00000039899	Fgl2	fibrinogen-like protein 2 [Source:MGI Symbol;Acc:N	0.17091
ENSMUSG00000077394	Gm24339	predicted gene, 24339 [Source:MGI Symbol;Acc:MG	0.17091
ENSMUSG00000047854	Stx19	syntaxin 19 [Source:MGI Symbol;Acc:MGI:1915409]	0.17092
ENSMUSG00000028329	Xpa	xeroderma pigmentosum, complementation group	0.1711
ENSMUSG00000022677	Fopnl	Fgfr1op N-terminal like [Source:MGI Symbol;Acc:M	0.17113
ENSMUSG00000052085	Dock8	dedicator of cytokinesis 8 [Source:MGI Symbol;Acc:	0.17115
ENSMUSG00000021692	Dimt1	DIM1 dimethyladenosine transferase 1-like (S. cere	0.17118
ENSMUSG00000021484	Lman2	lectin, mannose-binding 2 [Source:MGI Symbol;Acc	0.17121
ENSMUSG00000034783	Cd207	CD207 antigen [Source:MGI Symbol;Acc:MGI:2180(	0.17121
ENSMUSG00000022186	Oxct1	3-oxoacid CoA transferase 1 [Source:MGI Symbol;A	0.17122
ENSMUSG00000087090	Nctc1	non-coding transcript 1 [Source:MGI Symbol;Acc:M	0.17133
ENSMUSG00000086179	Gm14317	predicted gene 14317 [Source:MGI Symbol;Acc:MG	0.17137
ENSMUSG00000090284	Gm17613	predicted gene, 17613 [Source:MGI Symbol;Acc:MG	0.17141
ENSMUSG00000084213	Hspe1-ps5	heat shock protein 1 (chaperonin 10), pseudogene	0.17148
ENSMUSG00000090942	F830016B08Rik	RIKEN cDNA F830016B08 gene [Source:MGI Symbo	0.17155
ENSMUSG00000020307	Cdc34	cell division cycle 34 [Source:MGI Symbol;Acc:MGI:	0.17167
ENSMUSG00000003273	Car11	carbonic anhydrase 11 [Source:MGI Symbol;Acc:MG	0.17171
ENSMUSG00000020839	Tmigd1	transmembrane and immunoglobulin domain conta	0.17171
ENSMUSG00000030043	Tacr1	tachykinin receptor 1 [Source:MGI Symbol;Acc:MGI	0.17175
ENSMUSG00000093180	Gm25690	predicted gene, 25690 [Source:MGI Symbol;Acc:MG	0.1718
ENSMUSG00000056429	Tgoln1	trans-golgi network protein [Source:MGI Symbol;Ac	0.17182
ENSMUSG00000037661	Gpr160	G protein-coupled receptor 160 [Source:MGI Symb	0.17197
ENSMUSG00000022702	Hira	histone cell cycle regulation defective homolog A (S	0.172
ENSMUSG00000014074	Rnf168	ring finger protein 168 [Source:MGI Symbol;Acc:MG	0.17203
ENSMUSG00000062381	Vps28	vacuolar protein sorting 28 (yeast) [Source:MGI Syr	0.17207
ENSMUSG00000055862	Izumo4	IZUMO family member 4 [Source:MGI Symbol;Acc:N	0.17208
ENSMUSG00000022351	Sqle	squalene epoxidase [Source:MGI Symbol;Acc:MGI:1	0.17213
ENSMUSG00000032890	Rims3	regulating synaptic membrane exocytosis 3 [Source	0.17238
ENSMUSG00000093687	Gm20637	predicted gene 20637 [Source:MGI Symbol;Acc:MG	0.1724
ENSMUSG00000090019	Gimap1	GTPase, IMAP family member 1 [Source:MGI Symbo	0.17241
ENSMUSG00000000359	Rem1	rad and gem related GTP binding protein 1 [Source	0.17255
ENSMUSG00000049577	Zfpm1	zinc finger protein, multitype 1 [Source:MGI Symbo	0.17264
ENSMUSG00000096974	Gm26881	predicted gene, 26881 [Source:MGI Symbol;Acc:MG	0.17276
ENSMUSG00000023307	5-Mar	membrane-associated ring finger (C3HC4) 5 [Source	0.1728
ENSMUSG00000070305	Mpzl3	myelin protein zero-like 3 [Source:MGI Symbol;Acc	0.17281
ENSMUSG00000022471	Xrcc6	X-ray repair complementing defective repair in Chi	0.17283
ENSMUSG00000033326	Kdm4a	lysine (K)-specific demethylase 4A [Source:MGI Sym	0.17283
ENSMUSG00000020792	Exoc7	exocyst complex component 7 [Source:MGI Symbo	0.17286
ENSMUSG00000097072	Foxl2os	forkhead box L2 opposite strand transcript [Source	0.17293
ENSMUSG00000047712	Ust	uronyl-2-sulfotransferase [Source:MGI Symbol;Acc:	0.17297
ENSMUSG00000062554	Gm12751	predicted gene 12751 [Source:MGI Symbol;Acc:MG	0.173
ENSMUSG00000075229	Ccdc58	coiled-coil domain containing 58 [Source:MGI Symb	0.17303
ENSMUSG00000002550	Uck1	uridine-cytidine kinase 1 [Source:MGI Symbol;Acc:N	0.17308
ENSMUSG00000042082	Arsb	arylsulfatase B [Source:MGI Symbol;Acc:MGI:88075	0.17309

ENSMUSG00000060487	Samd5	sterile alpha motif domain containing 5 [Source:MG	0.1731
ENSMUSG00000086544	Gm13704	predicted gene 13704 [Source:MGI Symbol;Acc:MG	0.17317
ENSMUSG00000036534	Slc38a7	solute carrier family 38, member 7 [Source:MGI Sy	0.17328
ENSMUSG00000004568	Arhgef18	rho/rac guanine nucleotide exchange factor (GEF) 1	0.17341
ENSMUSG00000074582	Arfgef2	ADP-ribosylation factor guanine nucleotide-exchan	0.17345
ENSMUSG00000028743	Akr7a5	aldo-keto reductase family 7, member A5 (aflatoxir	0.17354
ENSMUSG00000068962	Zfp114	zinc finger protein 114 [Source:MGI Symbol;Acc:M	0.17362
ENSMUSG00000027577	Chrna4	cholinergic receptor, nicotinic, alpha polypeptide 4	0.17371
ENSMUSG00000033216	Eefsec	eukaryotic elongation factor, selenocysteine-tRNA-	0.17373
ENSMUSG00000037548	H2-DMb2	histocompatibility 2, class II, locus Mb2 [Source:MG	0.17373
ENSMUSG00000030619	Eed	embryonic ectoderm development [Source:MGI Sy	0.17382
ENSMUSG00000009927	Rps25	ribosomal protein S25 [Source:MGI Symbol;Acc:MG	0.17386
ENSMUSG00000015094	Npdc1	neural proliferation, differentiation and control 1 [S	0.1739
ENSMUSG00000053166	Cdh22	cadherin 22 [Source:MGI Symbol;Acc:MGI:1341843	0.17392
ENSMUSG00000082688	Gm12124	predicted gene 12124 [Source:MGI Symbol;Acc:MG	0.17394
ENSMUSG00000038415	Foxq1	forkhead box Q1 [Source:MGI Symbol;Acc:MGI:129	0.17404
ENSMUSG00000027242	Wdr76	WD repeat domain 76 [Source:MGI Symbol;Acc:MG	0.17406
ENSMUSG00000049303	Syt12	synaptotagmin XII [Source:MGI Symbol;Acc:MGI:21	0.17406
ENSMUSG00000038383	Pigu	phosphatidylinositol glycan anchor biosynthesis, cla	0.17419
ENSMUSG00000024999	Noc3l	nucleolar complex associated 3 homolog (S. cerevis	0.17421
ENSMUSG00000006333	Rps9	ribosomal protein S9 [Source:MGI Symbol;Acc:MGI	0.17424
ENSMUSG00000090840	1700092M07Rik	RIKEN cDNA 1700092M07 gene [Source:MGI Symb	0.17432
ENSMUSG00000034586	Hid1	HID1 domain containing [Source:MGI Symbol;Acc:M	0.17441
ENSMUSG00000039628	Hs3st6	heparan sulfate (glucosamine) 3-O-sulfotransferase	0.17447
ENSMUSG00000029471	Camkk2	calcium/calmodulin-dependent protein kinase kina	0.17453
ENSMUSG00000040524	Zfp609	zinc finger protein 609 [Source:MGI Symbol;Acc:M	0.17457
ENSMUSG00000031990	Jam3	junction adhesion molecule 3 [Source:MGI Symbol;	0.17465
ENSMUSG00000020612	Prkar1a	protein kinase, cAMP dependent regulatory, type I,	0.17468
ENSMUSG00000038300	Pth2	parathyroid hormone 2 [Source:MGI Symbol;Acc:M	0.1747
ENSMUSG00000029599	Ddx54	DEAD (Asp-Glu-Ala-Asp) box polypeptide 54 [Sourc	0.17471
ENSMUSG00000061259	Tmprss11d	transmembrane protease, serine 11d [Source:MGI	0.17481
ENSMUSG00000027510	Rbm38	RNA binding motif protein 38 [Source:MGI Symbol;	0.17496
ENSMUSG00000029992	Gfpt1	glutamine fructose-6-phosphate transaminase 1 [S	0.17499
ENSMUSG00000026686	Lmx1a	LIM homeobox transcription factor 1 alpha [Source	0.175
ENSMUSG00000097088	Gm26615	predicted gene, 26615 [Source:MGI Symbol;Acc:M	0.175
ENSMUSG00000020473	Aebp1	AE binding protein 1 [Source:MGI Symbol;Acc:MGI:	0.17504
ENSMUSG00000050271	D8Ertd82e	DNA segment, Chr 8, ERATO Doi 82, expressed [Sou	0.17507
ENSMUSG00000073758	Sh3d21	SH3 domain containing 21 [Source:MGI Symbol;Acc	0.1751
ENSMUSG00000077167	Gm24119	predicted gene, 24119 [Source:MGI Symbol;Acc:M	0.17518
ENSMUSG00000024327	Slc39a7	solute carrier family 39 (zinc transporter), member	0.17519
ENSMUSG00000030732	Chrdl2	chordin-like 2 [Source:MGI Symbol;Acc:MGI:191637	0.17521
ENSMUSG00000039148	Sart1	squamous cell carcinoma antigen recognized by T c	0.17523
ENSMUSG00000022604	Cep97	centrosomal protein 97 [Source:MGI Symbol;Acc:M	0.17528
ENSMUSG00000021384	Susd3	sushi domain containing 3 [Source:MGI Symbol;Acc	0.1753
ENSMUSG00000027540	Ptpn1	protein tyrosine phosphatase, non-receptor type 1	0.17534
ENSMUSG00000032525	Nktr	natural killer tumor recognition sequence [Source:M	0.17539
ENSMUSG00000097281	Gm26685	predicted gene, 26685 [Source:MGI Symbol;Acc:M	0.17545

ENSMUSG00000069769	Msi2	musashi RNA-binding protein 2 [Source:MGI Symbo	0.17546
ENSMUSG00000022106	Rcbtb2	regulator of chromosome condensation (RCC1) and	0.17549
ENSMUSG00000025467	Prap1	proline-rich acidic protein 1 [Source:MGI Symbol;A	0.17552
ENSMUSG00000097168	C230088H06Rik	RIKEN cDNA C230088H06 gene [Source:MGI Symbo	0.17552
ENSMUSG00000042472	Zfp410	zinc finger protein 410 [Source:MGI Symbol;Acc:M	0.17568
ENSMUSG00000008153	Clstn3	calsyntenin 3 [Source:MGI Symbol;Acc:MGI:217832	0.17569
ENSMUSG00000086638	4930405A21Rik	RIKEN cDNA 4930405A21 gene [Source:MGI Symbo	0.17577
ENSMUSG00000097737	Gm26530	predicted gene, 26530 [Source:MGI Symbol;Acc:M	0.17579
ENSMUSG00000022194	Pabpn1	poly(A) binding protein, nuclear 1 [Source:MGI Sym	0.17584
ENSMUSG00000069729	Arid1b	AT rich interactive domain 1B (SWI-like) [Source:M	0.17585
ENSMUSG00000039167	Eltd1	EGF, latrophilin seven transmembrane domain con	0.17591
ENSMUSG00000051537	Gm5124	predicted pseudogene 5124 [Source:MGI Symbol;A	0.17596
ENSMUSG00000039529	Atp8b1	ATPase, class I, type 8B, member 1 [Source:MGI Sy	0.17598
ENSMUSG00000071714	Csf2rb2	colony stimulating factor 2 receptor, beta 2, low-af	0.17601
ENSMUSG00000046330	Rpl37a	ribosomal protein L37a [Source:MGI Symbol;Acc:M	0.17603
ENSMUSG00000054434	Tmem120b	transmembrane protein 120B [Source:MGI Symbol;	0.17609
ENSMUSG00000086003	B230206L02Rik	RIKEN cDNA B230206L02 gene [Source:MGI Symbo	0.17616
ENSMUSG00000018921	Pelp1	proline, glutamic acid and leucine rich protein 1 [So	0.17622
ENSMUSG00000085633	4930580A09Rik	RIKEN cDNA 4930580A09 gene [Source:MGI Symbo	0.17627
ENSMUSG00000061118	Dnajc30	DnaJ (Hsp40) homolog, subfamily C, member 30 [So	0.17643
ENSMUSG00000064767	Snord35b	small nucleolar RNA, C/D box 35B [Source:MGI Sym	0.17652
ENSMUSG00000002111	Spi1	spleen focus forming virus (SFFV) proviral integratio	0.17658
ENSMUSG00000027528	Fabp9	fatty acid binding protein 9, testis [Source:MGI Sym	0.1766
ENSMUSG00000075470	Alg10b	asparagine-linked glycosylation 10B (alpha-1,2-gluc	0.1766
ENSMUSG00000064409	n-R5s40	nuclear encoded rRNA 5S 40 [Source:MGI Symbol;A	0.17678
ENSMUSG00000029452	Tmem116	transmembrane protein 116 [Source:MGI Symbol;A	0.17681
ENSMUSG00000026344	Lypd1	Ly6/Plaur domain containing 1 [Source:MGI Symbo	0.17685
ENSMUSG00000025492	Ifitm3	interferon induced transmembrane protein 3 [Sour	0.17688
ENSMUSG00000030871	Ears2	glutamyl-tRNA synthetase 2 (mitochondrial)(putati	0.17692
ENSMUSG00000007739	Cct4	chaperonin containing Tcp1, subunit 4 (delta) [Sour	0.17702
ENSMUSG00000089948	Gm16583	predicted gene 16583 [Source:MGI Symbol;Acc:MG	0.17723
ENSMUSG00000038608	Dock10	dedicator of cytokinesis 10 [Source:MGI Symbol;Ac	0.17735
ENSMUSG00000065047	Gm24045	predicted gene, 24045 [Source:MGI Symbol;Acc:M	0.17737
ENSMUSG00000026112	Coa5	cytochrome C oxidase assembly factor 5 [Source:M	0.17739
ENSMUSG00000070495	Ctcfl	CCCTC-binding factor (zinc finger protein)-like [Sou	0.17744
ENSMUSG00000032507	Fbxl2	F-box and leucine-rich repeat protein 2 [Source:MG	0.17752
ENSMUSG00000064514	Gm22455	predicted gene, 22455 [Source:MGI Symbol;Acc:M	0.17755
ENSMUSG00000034471	Caskin2	CASK-interacting protein 2 [Source:MGI Symbol;Ac	0.17766
ENSMUSG00000043153	Ispd	isoprenoid synthase domain containing [Source:MG	0.17768
ENSMUSG00000047146	Tet1	tet methylcytosine dioxygenase 1 [Source:MGI Sym	0.17769
ENSMUSG00000021493	Pdlim7	PDZ and LIM domain 7 [Source:MGI Symbol;Acc:M	0.17782
ENSMUSG00000049285	Mblac1	metallo-beta-lactamase domain containing 1 [Sour	0.17794
ENSMUSG00000024014	Pim1	proviral integration site 1 [Source:MGI Symbol;Acc:	0.17801
ENSMUSG00000086322	E130218I03Rik	RIKEN cDNA E130218I03 gene [Source:MGI Symbol	0.17814
ENSMUSG00000080895	Gm11645	predicted gene 11645 [Source:MGI Symbol;Acc:MG	0.17821
ENSMUSG00000072720	Myo18b	myosin XVIIIb [Source:MGI Symbol;Acc:MGI:19216	0.17822
ENSMUSG00000065715	Snord7	small nucleolar RNA, C/D box 7 [Source:MGI Symbo	0.1783

ENSMUSG00000059602	Syn3	synapsin III [Source:MGI Symbol;Acc:MGI:1351334]	0.17832
ENSMUSG00000025743	Sdc3	syndecan 3 [Source:MGI Symbol;Acc:MGI:1349163]	0.17833
ENSMUSG00000033762	Recql4	RecQ protein-like 4 [Source:MGI Symbol;Acc:MGI:1	0.17835
ENSMUSG00000026234	Ncl	nucleolin [Source:MGI Symbol;Acc:MGI:97286]	0.17836
ENSMUSG00000031518	Spata4	spermatogenesis associated 4 [Source:MGI Symbol	0.17861
ENSMUSG00000041789	2700046A07Rik	RIKEN cDNA 2700046A07 gene [Source:MGI Symbo	0.17868
ENSMUSG00000030527	Crtc3	CREB regulated transcription coactivator 3 [Source:	0.17869
ENSMUSG00000027395	Polr1b	polymerase (RNA) I polypeptide B [Source:MGI Sym	0.1787
ENSMUSG00000066306	Numa1	nuclear mitotic apparatus protein 1 [Source:MGI Sy	0.17879
ENSMUSG00000090043	Gm16167	predicted gene 16167 [Source:MGI Symbol;Acc:MG	0.17884
ENSMUSG00000041794	Myrip	myosin VIIA and Rab interacting protein [Source:M	0.17895
ENSMUSG00000037580	Gch1	GTP cyclohydrolase 1 [Source:MGI Symbol;Acc:MGI	0.179
ENSMUSG00000062210	Tnfaip8	tumor necrosis factor, alpha-induced protein 8 [Sou	0.17901
ENSMUSG00000082823	Gm15828	predicted gene 15828 [Source:MGI Symbol;Acc:MG	0.17908
ENSMUSG00000026971	Itgb6	integrin beta 6 [Source:MGI Symbol;Acc:MGI:96615	0.17911
ENSMUSG00000030766	Arhgap17	Rho GTPase activating protein 17 [Source:MGI Sym	0.17912
ENSMUSG00000059456	Ptk2b	PTK2 protein tyrosine kinase 2 beta [Source:MGI Sy	0.17913
ENSMUSG00000028150	Rorc	RAR-related orphan receptor gamma [Source:MGI S	0.1792
ENSMUSG00000006154	Eps8l1	EPS8-like 1 [Source:MGI Symbol;Acc:MGI:1914675]	0.17921
ENSMUSG00000017929	B4galt5	UDP-Gal:betaGlcNAc beta 1,4-galactosyltransferase	0.17927
ENSMUSG00000058921	Slc10a5	solute carrier family 10 (sodium/bile acid cotranspo	0.17928
ENSMUSG00000050199	Lgr4	leucine-rich repeat-containing G protein-coupled re	0.17931
ENSMUSG00000043850	Clrn1	clarin 1 [Source:MGI Symbol;Acc:MGI:2388124]	0.17936
ENSMUSG00000044442	N6amt1	N-6 adenine-specific DNA methyltransferase 1 (put	0.17942
ENSMUSG00000059248	9-Sep	septin 9 [Source:MGI Symbol;Acc:MGI:1858222]	0.17943
ENSMUSG00000030733	Sh2b1	SH2B adaptor protein 1 [Source:MGI Symbol;Acc:M	0.17945
ENSMUSG00000060586	H2-Eb1	histocompatibility 2, class II antigen E beta [Source:	0.17945
ENSMUSG00000059852	Kcng2	potassium voltage-gated channel, subfamily G, mer	0.17946
ENSMUSG00000024174	Pot1b	protection of telomeres 1B [Source:MGI Symbol;Ac	0.17948
ENSMUSG00000027070	Lrp2	low density lipoprotein receptor-related protein 2 [	0.17949
ENSMUSG00000026749	Nek6	NIMA (never in mitosis gene a)-related expressed k	0.17952
ENSMUSG00000039988	Ankrd13c	ankyrin repeat domain 13c [Source:MGI Symbol;Ac	0.17959
ENSMUSG00000024181	Mrpl28	mitochondrial ribosomal protein L28 [Source:MGI S	0.1798
ENSMUSG00000035834	Polr3g	polymerase (RNA) III (DNA directed) polypeptide G	0.17982
ENSMUSG00000051851	Cxx1c	CAAX box 1C [Source:MGI Symbol;Acc:MGI:192011	0.17983
ENSMUSG00000033730	Egr3	early growth response 3 [Source:MGI Symbol;Acc:N	0.17987
ENSMUSG00000026048	Ercc5	excision repair cross-complementing rodent repair	0.17996
ENSMUSG00000053825	Ppfia2	protein tyrosine phosphatase, receptor type, f poly	0.17998
ENSMUSG00000028945	Rheb	Ras homolog enriched in brain [Source:MGI Symbo	0.18001
ENSMUSG00000024382	Ercc3	excision repair cross-complementing rodent repair	0.18009
ENSMUSG00000097372	Gm26876	predicted gene, 26876 [Source:MGI Symbol;Acc:MG	0.1801
ENSMUSG00000003872	Lin7b	lin-7 homolog B (C. elegans) [Source:MGI Symbol;A	0.18013
ENSMUSG00000045896	Paip2b	poly(A) binding protein interacting protein 2B [Sour	0.18015
ENSMUSG00000085950	Gm13589	predicted gene 13589 [Source:MGI Symbol;Acc:MG	0.18015
ENSMUSG00000021892	Sh3bp5	SH3-domain binding protein 5 (BTK-associated) [So	0.18016
ENSMUSG00000041734	Kirrel	kin of IRRE like (Drosophila) [Source:MGI Symbol;A	0.1803
ENSMUSG00000043289	Mei4	meiosis-specific, MEI4 homolog (S. cerevisiae) [Sou	0.18034

ENSMUSG00000087199	Gm15818	predicted gene 15818 [Source:MGI Symbol;Acc:MG	0.18034
ENSMUSG00000048583	Igf2	insulin-like growth factor 2 [Source:MGI Symbol;Ac	0.1804
ENSMUSG00000021610	Clptm1l	CLPTM1-like [Source:MGI Symbol;Acc:MGI:244289	0.18045
ENSMUSG00000026458	Ppfia4	protein tyrosine phosphatase, receptor type, f poly	0.18051
ENSMUSG00000023473	Celsr3	cadherin, EGF LAG seven-pass G-type receptor 3 (fl	0.18057
ENSMUSG00000028278	Rragd	Ras-related GTP binding D [Source:MGI Symbol;Acc	0.18058
ENSMUSG00000029642	Polr1d	polymerase (RNA) I polypeptide D [Source:MGI Sym	0.18058
ENSMUSG00000040380	Cbln3	cerebellin 3 precursor protein [Source:MGI Symbol	0.18059
ENSMUSG00000027194	Ttc17	tetratricopeptide repeat domain 17 [Source:MGI Sy	0.18062
ENSMUSG00000009376	Met	met proto-oncogene [Source:MGI Symbol;Acc:MGI	0.18063
ENSMUSG00000028776	Tinagl1	tubulointerstitial nephritis antigen-like 1 [Source:M	0.18066
ENSMUSG00000023978	Prph2	peripherin 2 [Source:MGI Symbol;Acc:MGI:102791]	0.18072
ENSMUSG00000084823	Gm17216	predicted gene 17216 [Source:MGI Symbol;Acc:MG	0.18075
ENSMUSG00000084908	C79798	expressed sequence C79798 [Source:MGI Symbol;A	0.18076
ENSMUSG00000030664	1110006G14Rik	RIKEN cDNA 1110006G14 gene [Source:MGI Symbo	0.1808
ENSMUSG00000087703	Gm15650	predicted gene 15650 [Source:MGI Symbol;Acc:MG	0.18093
ENSMUSG00000002076	Hsf2bp	heat shock transcription factor 2 binding protein [S	0.181
ENSMUSG00000074619	1700034I23Rik	RIKEN cDNA 1700034I23 gene [Source:MGI Symbol	0.18108
ENSMUSG00000031862	Atp13a1	ATPase type 13A1 [Source:MGI Symbol;Acc:MGI:21	0.18109
ENSMUSG00000027547	Sall4	sal-like 4 (Drosophila) [Source:MGI Symbol;Acc:MG	0.18113
ENSMUSG00000031394	Opn1mw	opsin 1 (cone pigments), medium-wave-sensitive (c	0.18123
ENSMUSG00000038845	Phb	prohibitin [Source:MGI Symbol;Acc:MGI:97572]	0.18139
ENSMUSG00000022791	Tnk2	tyrosine kinase, non-receptor, 2 [Source:MGI Symb	0.18152
ENSMUSG00000093390	Gm20652	predicted gene 20652 [Source:MGI Symbol;Acc:MG	0.18153
ENSMUSG00000055027	Smyd1	SET and MYND domain containing 1 [Source:MGI Sy	0.18162
ENSMUSG00000035298	Klhl35	kelch-like 35 [Source:MGI Symbol;Acc:MGI:191943	0.18173
ENSMUSG00000028444	Cntfr	ciliary neurotrophic factor receptor [Source:MGI Sy	0.18177
ENSMUSG00000055216	9430025C20Rik	RIKEN cDNA 9430025C20 gene [Source:MGI Symbo	0.18179
ENSMUSG00000046179	E2f8	E2F transcription factor 8 [Source:MGI Symbol;Acc:	0.18183
ENSMUSG00000003814	Calr	calreticulin [Source:MGI Symbol;Acc:MGI:88252]	0.18184
ENSMUSG00000026938	Fcna	ficolin A [Source:MGI Symbol;Acc:MGI:1340905]	0.18186
ENSMUSG00000054428	Atpif1	ATPase inhibitory factor 1 [Source:MGI Symbol;Acc	0.18187
ENSMUSG00000031527	Eri1	exoribonuclease 1 [Source:MGI Symbol;Acc:MGI:19	0.18188
ENSMUSG00000055415	Atp10b	ATPase, class V, type 10B [Source:MGI Symbol;Acc:	0.18192
ENSMUSG00000035944	Ttc38	tetratricopeptide repeat domain 38 [Source:MGI Sy	0.18198
ENSMUSG00000026888	Grb14	growth factor receptor bound protein 14 [Source:M	0.18205
ENSMUSG00000052105	Soga2	SOGA family member 2 [Source:MGI Symbol;Acc:M	0.18207
ENSMUSG00000006310	Zbtb32	zinc finger and BTB domain containing 32 [Source:M	0.18217
ENSMUSG00000058441	Panx2	pannexin 2 [Source:MGI Symbol;Acc:MGI:1890615]	0.18221
ENSMUSG00000037012	Hk1	hexokinase 1 [Source:MGI Symbol;Acc:MGI:96103]	0.18222
ENSMUSG00000020747	2310067B10Rik	RIKEN cDNA 2310067B10 gene [Source:MGI Symbo	0.18223
ENSMUSG00000074892	B3galt5	UDP-Gal:betaGlcNAc beta 1,3-galactosyltransferase	0.18226
ENSMUSG00000059439	Bcas3	breast carcinoma amplified sequence 3 [Source:MG	0.1823
ENSMUSG00000026944	Abca2	ATP-binding cassette, sub-family A (ABC1), member	0.18242
ENSMUSG00000039747	Orai2	ORAI calcium release-activated calcium modulator	0.18242
ENSMUSG00000076456	Mir760	microRNA 760 [Source:MGI Symbol;Acc:MGI:36916	0.18243
ENSMUSG00000021876	Rnase4	ribonuclease, RNase A family 4 [Source:MGI Symbo	0.1827

ENSMUSG00000033306	Lpp	LIM domain containing preferred translocation part	0.18273
ENSMUSG00000035376	Ptplb	protein tyrosine phosphatase-like (proline instead c	0.1828
ENSMUSG00000058152	Chsy3	chondroitin sulfate synthase 3 [Source:MGI Symbol	0.18287
ENSMUSG00000022960	Donson	downstream neighbor of SON [Source:MGI Symbol;	0.18288
ENSMUSG00000027185	Nat10	N-acetyltransferase 10 [Source:MGI Symbol;Acc:MG	0.18289
ENSMUSG00000052364	B630019K06Rik	RIKEN cDNA B630019K06 gene [Source:MGI Symbo	0.18296
ENSMUSG00000010051	Hyal1	hyaluronoglucosaminidase 1 [Source:MGI Symbol;A	0.18305
ENSMUSG00000004885	Crabp2	cellular retinoic acid binding protein II [Source:MGI	0.1831
ENSMUSG00000020255	D10Wsu102e	DNA segment, Chr 10, Wayne State University 102,	0.18318
ENSMUSG00000031289	Il13ra2	interleukin 13 receptor, alpha 2 [Source:MGI Symb	0.18321
ENSMUSG00000084799	Gm11602	predicted gene 11602 [Source:MGI Symbol;Acc:MG	0.18323
ENSMUSG00000061397	Krt79	keratin 79 [Source:MGI Symbol;Acc:MGI:2385030]	0.18336
ENSMUSG00000002833	Hdgfrp2	hepatoma-derived growth factor, related protein 2	0.18342
ENSMUSG00000004939	Nmrk2	nicotinamide riboside kinase 2 [Source:MGI Symbo	0.18344
ENSMUSG00000002845	Tmem39a	transmembrane protein 39a [Source:MGI Symbol;A	0.18354
ENSMUSG00000006395	Hyi	hydroxypyruvate isomerase homolog (E. coli) [Sour	0.18356
ENSMUSG00000057738	Sptan1	spectrin alpha, non-erythrocytic 1 [Source:MGI Sym	0.18357
ENSMUSG00000032359	Ctsh	cathepsin H [Source:MGI Symbol;Acc:MGI:107285]	0.18364
ENSMUSG00000030748	Il4ra	interleukin 4 receptor, alpha [Source:MGI Symbol;A	0.18367
ENSMUSG00000027347	Rasgrp1	RAS guanyl releasing protein 1 [Source:MGI Symbo	0.18379
ENSMUSG00000095304	Plac9a	placenta specific 9a [Source:MGI Symbol;Acc:MGI:2	0.18381
ENSMUSG00000018565	Elp5	elongator acetyltransferase complex subunit 5 [Sou	0.18409
ENSMUSG00000097304	Rpl31-ps15	ribosomal protein L31, pseudogene 15 [Source:MG	0.18414
ENSMUSG00000020830	Vmo1	vitelline membrane outer layer 1 homolog (chicken	0.18415
ENSMUSG00000021062	Rab15	RAB15, member RAS oncogene family [Source:MGI	0.18418
ENSMUSG00000026787	Gad2	glutamic acid decarboxylase 2 [Source:MGI Symbol	0.18419
ENSMUSG00000046593	Tmem215	transmembrane protein 215 [Source:MGI Symbol;A	0.1842
ENSMUSG00000037570	Mcrs1	microspherule protein 1 [Source:MGI Symbol;Acc:N	0.18429
ENSMUSG00000006818	Sod2	superoxide dismutase 2, mitochondrial [Source:MG	0.18432
ENSMUSG00000031286	Glt28d2	glycosyltransferase 28 domain containing 2 [Source	0.18434
ENSMUSG00000058588	Vmn1r21	vomeronasal 1 receptor 21 [Source:MGI Symbol;Ac	0.18438
ENSMUSG00000027498	Cstf1	cleavage stimulation factor, 3' pre-RNA, subunit 1 [	0.18443
ENSMUSG00000042613	Pbxip1	pre B cell leukemia transcription factor interacting	0.18452
ENSMUSG00000050721	Plekho2	pleckstrin homology domain containing, family O m	0.18459
ENSMUSG00000071658	Gng3	guanine nucleotide binding protein (G protein), gar	0.18461
ENSMUSG00000023011	Faim2	Fas apoptotic inhibitory molecule 2 [Source:MGI Sy	0.18466
ENSMUSG00000017837	Nkiras2	NFKB inhibitor interacting Ras-like protein 2 [Sourc	0.18471
ENSMUSG00000050556	Kcnb1	potassium voltage gated channel, Shab-related sub	0.18478
ENSMUSG00000025786	Zdhhc3	zinc finger, DHHC domain containing 3 [Source:MG	0.18486
ENSMUSG00000075463	4930594M22Rik	RIKEN cDNA 4930594M22 gene [Source:MGI Symb	0.18487
ENSMUSG00000028597	Gpx7	glutathione peroxidase 7 [Source:MGI Symbol;Acc:	0.18504
ENSMUSG00000024949	Sf1	splicing factor 1 [Source:MGI Symbol;Acc:MGI:1095	0.18525
ENSMUSG00000038563	Eftud1	elongation factor Tu GTP binding domain containin	0.18532
ENSMUSG00000092344	Hspe1-ps3	heat shock protein 1 (chaperonin 10), pseudogene	0.18532
ENSMUSG00000085804	Gm12973	predicted gene 12973 [Source:MGI Symbol;Acc:MG	0.18534
ENSMUSG00000003949	Hlf	hepatic leukemia factor [Source:MGI Symbol;Acc:M	0.18537
ENSMUSG00000038473	Nos1ap	nitric oxide synthase 1 (neuronal) adaptor protein [	0.18541

ENSMUSG00000089651	Gm16353	predicted gene 16353 [Source:MGI Symbol;Acc:MG	0.18542
ENSMUSG00000049001	Ndnf	neuron-derived neurotrophic factor [Source:MGI Sy	0.18544
ENSMUSG00000038235	F11r	F11 receptor [Source:MGI Symbol;Acc:MGI:132139	0.18549
ENSMUSG00000030428	Ttyh1	tweety homolog 1 (Drosophila) [Source:MGI Symbo	0.18558
ENSMUSG00000039640	Mrpl12	mitochondrial ribosomal protein L12 [Source:MGI S	0.18562
ENSMUSG00000073988	Ttpa	tocopherol (alpha) transfer protein [Source:MGI Sy	0.18569
ENSMUSG00000034765	Dusp5	dual specificity phosphatase 5 [Source:MGI Symbol	0.1857
ENSMUSG00000041881	Ndufa7	NADH dehydrogenase (ubiquinone) 1 alpha subcom	0.18583
ENSMUSG00000014837	4931428F04Rik	RIKEN cDNA 4931428F04 gene [Source:MGI Symbo	0.18593
ENSMUSG00000018068	Ints2	integrator complex subunit 2 [Source:MGI Symbol;	0.18595
ENSMUSG00000002910	Arrdc2	arrestin domain containing 2 [Source:MGI Symbol;	0.18604
ENSMUSG00000020755	Sap30bp	SAP30 binding protein [Source:MGI Symbol;Acc:MG	0.18615
ENSMUSG00000075028	Prdm11	PR domain containing 11 [Source:MGI Symbol;Acc:	0.18625
ENSMUSG00000030110	Ret	ret proto-oncogene [Source:MGI Symbol;Acc:MGI:9	0.18626
ENSMUSG00000039787	Cercam	cerebral endothelial cell adhesion molecule [Source	0.18628
ENSMUSG00000019487	Trip10	thyroid hormone receptor interactor 10 [Source:M	0.18632
ENSMUSG00000030397	Mark4	MAP/microtubule affinity-regulating kinase 4 [Sour	0.18633
ENSMUSG00000044030	Irf2bp1	interferon regulatory factor 2 binding protein 1 [So	0.18636
ENSMUSG00000058975	Kcnc1	potassium voltage gated channel, Shaw-related sub	0.18638
ENSMUSG00000027485	Bpifb1	BPI fold containing family B, member 1 [Source:MG	0.18639
ENSMUSG00000034463	Scara3	scavenger receptor class A, member 3 [Source:MGI	0.18644
ENSMUSG00000073633	Fbxo36	F-box protein 36 [Source:MGI Symbol;Acc:MGI:128	0.18645
ENSMUSG00000024528	Srfbp1	serum response factor binding protein 1 [Source:M	0.18653
ENSMUSG00000035093	Secisbp2l	SECIS binding protein 2-like [Source:MGI Symbol;Ac	0.18654
ENSMUSG00000044221	Grsf1	G-rich RNA sequence binding factor 1 [Source:MGI	0.18655
ENSMUSG00000000531	Grasp	GRP1 (general receptor for phosphoinositides 1)-as	0.18661
ENSMUSG00000027784	Ppm1l	protein phosphatase 1 (formerly 2C)-like [Source:M	0.18661
ENSMUSG00000031879	Fam96b	family with sequence similarity 96, member B [Sour	0.18664
ENSMUSG00000002767	Mrpl2	mitochondrial ribosomal protein L2 [Source:MGI Sy	0.18665
ENSMUSG00000022056	Adam7	a disintegrin and metallopeptidase domain 7 [Sourc	0.18674
ENSMUSG00000049103	Ccr2	chemokine (C-C motif) receptor 2 [Source:MGI Sym	0.18675
ENSMUSG00000038545	Cul7	cullin 7 [Source:MGI Symbol;Acc:MGI:1913765]	0.18681
ENSMUSG00000027291	Vps39	vacuolar protein sorting 39 (yeast) [Source:MGI Syr	0.18686
ENSMUSG00000052135	Foxo6	forkhead box O6 [Source:MGI Symbol;Acc:MGI:267	0.18687
ENSMUSG00000024878	Cbwd1	COBW domain containing 1 [Source:MGI Symbol;Ac	0.18691
ENSMUSG00000022218	Tgm1	transglutaminase 1, K polypeptide [Source:MGI Syn	0.18695
ENSMUSG00000054690	Emcn	endomucin [Source:MGI Symbol;Acc:MGI:1891716]	0.18696
ENSMUSG00000002343	Armc6	armadillo repeat containing 6 [Source:MGI Symbol;	0.18707
ENSMUSG00000086603	Gm13620	predicted gene 13620 [Source:MGI Symbol;Acc:MG	0.18707
ENSMUSG00000063802	Hspbp1	HSPA (heat shock 70kDa) binding protein, cytoplasr	0.1871
ENSMUSG00000018543	1700001P01Rik	RIKEN cDNA 1700001P01 gene [Source:MGI Symbo	0.18713
ENSMUSG00000086392	A330050B17Rik	RIKEN cDNA A330050B17 gene [Source:MGI Symbo	0.18742
ENSMUSG00000020335	Zfp354b	zinc finger protein 354B [Source:MGI Symbol;Acc:M	0.18744
ENSMUSG00000039349	C130074G19Rik	RIKEN cDNA C130074G19 gene [Source:MGI Symbo	0.18748
ENSMUSG00000006344	Ggt5	gamma-glutamyltransferase 5 [Source:MGI Symbol	0.18749
ENSMUSG00000038903	Ccdc68	coiled-coil domain containing 68 [Source:MGI Symb	0.18757
ENSMUSG00000052544	St6galnac3	ST6 (alpha-N-acetyl-neuraminyl-2,3-beta-galactosy	0.18757

ENSMUSG00000026211	Obsl1	obscurin-like 1 [Source:MGI Symbol;Acc:MGI:2138	0.18764
ENSMUSG00000054509	Parp4	poly (ADP-ribose) polymerase family, member 4 [So	0.18771
ENSMUSG00000050473	Slc35d3	solute carrier family 35, member D3 [Source:MGI S	0.18773
ENSMUSG00000020520	Galnt10	UDP-N-acetyl-alpha-D-galactosamine:polypeptide N	0.18779
ENSMUSG00000003200	Sh3gl1	SH3-domain GRB2-like 1 [Source:MGI Symbol;Acc:M	0.18781
ENSMUSG00000097675	1700101I11Rik	RIKEN cDNA 1700101I11 gene [Source:MGI Symbol	0.18791
ENSMUSG00000028946	Hes3	hairy and enhancer of split 3 (Drosophila) [Source:M	0.18796
ENSMUSG00000028860	Sytl1	synaptotagmin-like 1 [Source:MGI Symbol;Acc:MGI	0.18797
ENSMUSG00000021578	Ccdc127	coiled-coil domain containing 127 [Source:MGI Sym	0.18799
ENSMUSG00000030004	Nat8	N-acetyltransferase 8 (GCN5-related, putative) [Sou	0.18802
ENSMUSG00000063730	Hsd3b2	hydroxy-delta-5-steroid dehydrogenase, 3 beta- an	0.18802
ENSMUSG00000068221	Pdxp	pyridoxal (pyridoxine, vitamin B6) phosphatase [So	0.18802
ENSMUSG00000087182	Gm13334	predicted gene 13334 [Source:MGI Symbol;Acc:MG	0.18803
ENSMUSG00000092500	Gm20400	predicted gene 20400 [Source:MGI Symbol;Acc:MG	0.18803
ENSMUSG00000096768	Erdr1	erythroid differentiation regulator 1 [Source:MGI S	0.18804
ENSMUSG00000097576	D930030I03Rik	RIKEN cDNA D930030I03 gene [Source:MGI Symbol	0.18807
ENSMUSG00000021898	Asb14	ankyrin repeat and SOCS box-containing 14 [Source	0.1881
ENSMUSG00000034211	Mrps17	mitochondrial ribosomal protein S17 [Source:MGI S	0.18828
ENSMUSG00000096883	AI848285	expressed sequence AI848285 [Source:MGI Symbol	0.18828
ENSMUSG00000022841	Ap2m1	adaptor-related protein complex 2, mu 1 subunit [S	0.18831
ENSMUSG00000034310	Tmem132d	transmembrane protein 132D [Source:MGI Symbol	0.18832
ENSMUSG00000020331	Hcn2	hyperpolarization-activated, cyclic nucleotide-gated	0.18833
ENSMUSG00000004394	Tmed4	transmembrane emp24 protein transport domain c	0.18839
ENSMUSG00000030934	Oat	ornithine aminotransferase [Source:MGI Symbol;Ac	0.18843
ENSMUSG00000037674	Rfx7	regulatory factor X, 7 [Source:MGI Symbol;Acc:MGI	0.18846
ENSMUSG00000059810	Rgs3	regulator of G-protein signaling 3 [Source:MGI Sym	0.18848
ENSMUSG00000040151	Hs2st1	heparan sulfate 2-O-sulfotransferase 1 [Source:MG	0.18856
ENSMUSG00000021975	Ints9	integrator complex subunit 9 [Source:MGI Symbol;A	0.18861
ENSMUSG00000024414	Mrpl27	mitochondrial ribosomal protein L27 [Source:MGI S	0.18886
ENSMUSG00000034245	Hdac11	histone deacetylase 11 [Source:MGI Symbol;Acc:M	0.18897
ENSMUSG00000083863	Gm13341	predicted gene 13341 [Source:MGI Symbol;Acc:MG	0.18898
ENSMUSG00000092626	9130230N09Rik	RIKEN cDNA 9130230N09 gene [Source:MGI Symbo	0.18898
ENSMUSG00000030543	Mesp2	mesoderm posterior 2 [Source:MGI Symbol;Acc:MC	0.18906
ENSMUSG00000003037	Rab8a	RAB8A, member RAS oncogene family [Source:MGI	0.18908
ENSMUSG00000079677	Fdx1l	ferredoxin 1-like [Source:MGI Symbol;Acc:MGI:191	0.18909
ENSMUSG00000031328	Flna	filamin, alpha [Source:MGI Symbol;Acc:MGI:95556]	0.18912
ENSMUSG00000036533	Cdc42ep3	CDC42 effector protein (Rho GTPase binding) 3 [Sou	0.18913
ENSMUSG00000042118	Bhmt2	betaine-homocysteine methyltransferase 2 [Source	0.18921
ENSMUSG00000050947	Amigo1	adhesion molecule with Ig like domain 1 [Source:M	0.18929
ENSMUSG00000035246	Pcyt1b	phosphate cytidylyltransferase 1, choline, beta isof	0.18935
ENSMUSG00000016346	Kcnq2	potassium voltage-gated channel, subfamily Q, mer	0.18938
ENSMUSG00000027520	Zdbf2	zinc finger, DBF-type containing 2 [Source:MGI Sym	0.18944
ENSMUSG00000060981	Hist1h4h	histone cluster 1, H4h [Source:MGI Symbol;Acc:MG	0.18954
ENSMUSG00000064264	Zfp428	zinc finger protein 428 [Source:MGI Symbol;Acc:MC	0.18956
ENSMUSG00000041625	Ggact	gamma-glutamylamine cyclotransferase [Source:M	0.18973
ENSMUSG00000020953	Coch	coagulation factor C homolog (Limulus polyphemus	0.18976
ENSMUSG00000085232	Gm11962	predicted gene 11962 [Source:MGI Symbol;Acc:MG	0.18977

ENSMUSG00000006335	Tfpt	TCF3 (E2A) fusion partner [Source:MGI Symbol;Acc	0.18991
ENSMUSG00000016349	Eef1a2	eukaryotic translation elongation factor 1 alpha 2 [S	0.19002
ENSMUSG00000039183	Nubp2	nucleotide binding protein 2 [Source:MGI Symbol;A	0.19006
ENSMUSG00000027160	Ccdc34	coiled-coil domain containing 34 [Source:MGI Symb	0.19007
ENSMUSG00000086264	Gm15850	predicted gene 15850 [Source:MGI Symbol;Acc:MG	0.19008
ENSMUSG00000031969	Acad8	acyl-Coenzyme A dehydrogenase family, member 8	0.19025
ENSMUSG00000096960	A230028O05Rik	RIKEN cDNA A230028O05 gene [Source:MGI Symbo	0.19029
ENSMUSG00000039428	Tmem135	transmembrane protein 135 [Source:MGI Symbol;A	0.19036
ENSMUSG00000045589	Frrs1l	ferric-chelate reductase 1 like [Source:MGI Symbol;	0.19036
ENSMUSG00000026470	Stx6	syntaxin 6 [Source:MGI Symbol;Acc:MGI:1926235]	0.19039
ENSMUSG00000054510	Gm14461	predicted gene 14461 [Source:MGI Symbol;Acc:MG	0.19042
ENSMUSG00000086111	Gm15326	predicted gene 15326 [Source:MGI Symbol;Acc:MG	0.19045
ENSMUSG00000042631	Xkr7	X Kell blood group precursor related family membe	0.19048
ENSMUSG00000098238	Gm8756	predicted gene 8756 [Source:MGI Symbol;Acc:MGI:	0.19049
ENSMUSG00000056004	9330182L06Rik	RIKEN cDNA 9330182L06 gene [Source:MGI Symbo	0.19052
ENSMUSG00000086907	Gm15298	predicted gene 15298 [Source:MGI Symbol;Acc:MG	0.19062
ENSMUSG00000097520	Gm26843	predicted gene, 26843 [Source:MGI Symbol;Acc:M	0.19062
ENSMUSG00000064068	Mtx1	metaxin 1 [Source:MGI Symbol;Acc:MGI:103025]	0.1907
ENSMUSG00000066178	6030445D17Rik	RIKEN cDNA 6030445D17 gene [Source:MGI Symbo	0.19075
ENSMUSG00000025060	Slk	STE20-like kinase [Source:MGI Symbol;Acc:MGI:103	0.1909
ENSMUSG00000087633	Gm14455	predicted gene 14455 [Source:MGI Symbol;Acc:MG	0.1909
ENSMUSG00000024732	Ccdc86	coiled-coil domain containing 86 [Source:MGI Symb	0.19104
ENSMUSG00000022519	Srl	sarcalumenin [Source:MGI Symbol;Acc:MGI:214662	0.19108
ENSMUSG00000086290	Snhg12	small nucleolar RNA host gene 12 [Source:MGI Sym	0.19108
ENSMUSG00000037519	Ppfia1	protein tyrosine phosphatase, receptor type, f poly	0.19111
ENSMUSG00000041203	2310036O22Rik	RIKEN cDNA 2310036O22 gene [Source:MGI Symbo	0.19111
ENSMUSG00000042662	Dusp15	dual specificity phosphatase-like 15 [Source:MGI Sy	0.19115
ENSMUSG00000039414	Heatr5b	HEAT repeat containing 5B [Source:MGI Symbol;Ac	0.19116
ENSMUSG00000028255	Clca3	chloride channel calcium activated 3 [Source:MGI S	0.19119
ENSMUSG00000081677	Mdk-ps1	midkine pseudogene 1 [Source:MGI Symbol;Acc:M	0.19127
ENSMUSG00000031262	Cenpi	centromere protein I [Source:MGI Symbol;Acc:MGI	0.19131
ENSMUSG00000010911	Apip	APAF1 interacting protein [Source:MGI Symbol;Acc	0.19134
ENSMUSG00000051503	Gm6583	predicted gene 6583 [Source:MGI Symbol;Acc:MGI:	0.19153
ENSMUSG00000029676	Pot1a	protection of telomeres 1A [Source:MGI Symbol;Ac	0.19154
ENSMUSG00000030800	Prss8	protease, serine, 8 (prostasin) [Source:MGI Symbol	0.19177
ENSMUSG00000024736	Tmem132a	transmembrane protein 132A [Source:MGI Symbol;	0.1918
ENSMUSG00000001663	Gstt1	glutathione S-transferase, theta 1 [Source:MGI Sym	0.19181
ENSMUSG00000097527	1700112J16Rik	RIKEN cDNA 1700112J16 gene [Source:MGI Symbol	0.19182
ENSMUSG00000081470	Gm14131	predicted gene 14131 [Source:MGI Symbol;Acc:MG	0.19184
ENSMUSG00000026074	Map4k4	mitogen-activated protein kinase kinase kinase kina	0.19189
ENSMUSG00000026123	Plekhb2	pleckstrin homology domain containing, family B (e	0.19189
ENSMUSG00000045725	Prr15	proline rich 15 [Source:MGI Symbol;Acc:MGI:19252	0.19189
ENSMUSG00000044701	Il27	interleukin 27 [Source:MGI Symbol;Acc:MGI:23844	0.19193
ENSMUSG00000059252	Tpt1-ps5	tumor protein, translationally-controlled, pseudoge	0.19194
ENSMUSG00000098116	Gm7191	predicted gene 7191 [Source:MGI Symbol;Acc:MGI:	0.19198
ENSMUSG00000028467	Gba2	glucosidase beta 2 [Source:MGI Symbol;Acc:MGI:26	0.19201
ENSMUSG00000093697	Gm20686	predicted gene 20686 [Source:MGI Symbol;Acc:MG	0.19201

ENSMUSG00000083909	Gm15842	predicted gene 15842 [Source:MGI Symbol;Acc:MG	0.19206
ENSMUSG00000070574	2310016G11Rik	RIKEN cDNA 2310016G11 gene [Source:MGI Symbo	0.19217
ENSMUSG00000029552	Tes	testis derived transcript [Source:MGI Symbol;Acc:M	0.1922
ENSMUSG00000085177	Gm11209	predicted gene 11209 [Source:MGI Symbol;Acc:MG	0.19223
ENSMUSG00000086777	B230104C08Rik	RIKEN cDNA B230104C08 gene [Source:MGI Symbo	0.19231
ENSMUSG00000085975	Gm13572	predicted gene 13572 [Source:MGI Symbol;Acc:MG	0.19234
ENSMUSG00000028780	Sema3c	sema domain, immunoglobulin domain (Ig), short b	0.19237
ENSMUSG00000022961	Son	Son DNA binding protein [Source:MGI Symbol;Acc:I	0.19252
ENSMUSG00000034813	Grip1	glutamate receptor interacting protein 1 [Source:M	0.19258
ENSMUSG00000028059	Arhgef2	rho/rac guanine nucleotide exchange factor (GEF) 2	0.19261
ENSMUSG00000028191	Bcl10	B cell leukemia/lymphoma 10 [Source:MGI Symbol;	0.19268
ENSMUSG00000086370	Ftx	Ftx transcript, Xist regulator (non-protein coding) [S	0.19269
ENSMUSG00000021916	Glt8d1	glycosyltransferase 8 domain containing 1 [Source:	0.1927
ENSMUSG00000032558	Nphp3	nephronophthisis 3 (adolescent) [Source:MGI Symb	0.1928
ENSMUSG00000092438	Gm18734	predicted gene, 18734 [Source:MGI Symbol;Acc:MG	0.19286
ENSMUSG00000031504	Rab20	RAB20, member RAS oncogene family [Source:MGI	0.19291
ENSMUSG00000052698	Tln2	talin 2 [Source:MGI Symbol;Acc:MGI:1917799]	0.19293
ENSMUSG00000046364	Rpl27a	ribosomal protein L27A [Source:MGI Symbol;Acc:M	0.19296
ENSMUSG00000002668	Dennd1c	DENN/MADD domain containing 1C [Source:MGI Sy	0.19297
ENSMUSG00000095567	Noc2l	nucleolar complex associated 2 homolog (S. cerevis	0.19297
ENSMUSG00000092225	Gm2381	predicted gene 2381 [Source:MGI Symbol;Acc:MGI:	0.19299
ENSMUSG00000040136	Abcc8	ATP-binding cassette, sub-family C (CFTR/MRP), me	0.19308
ENSMUSG00000000202	Btbd17	BTB (POZ) domain containing 17 [Source:MGI Symb	0.1931
ENSMUSG00000050234	Gja4	gap junction protein, alpha 4 [Source:MGI Symbol;A	0.19317
ENSMUSG00000019832	Rab32	RAB32, member RAS oncogene family [Source:MGI	0.19318
ENSMUSG00000003444	Med29	mediator complex subunit 29 [Source:MGI Symbol;	0.19323
ENSMUSG00000052609	Plekhg3	pleckstrin homology domain containing, family G (w	0.19327
ENSMUSG00000078716	Tmem8b	transmembrane protein 8B [Source:MGI Symbol;Ac	0.1933
ENSMUSG00000097819	Gm26813	predicted gene, 26813 [Source:MGI Symbol;Acc:MG	0.19333
ENSMUSG00000042650	Alkbh5	alkB, alkylation repair homolog 5 (E. coli) [Source:M	0.19334
ENSMUSG00000037148	Arhgap10	Rho GTPase activating protein 10 [Source:MGI Sym	0.19336
ENSMUSG00000047719	Ubiad1	UbiA prenyltransferase domain containing 1 [Sourc	0.19352
ENSMUSG00000087621	1700003G18Rik	RIKEN cDNA 1700003G18 gene [Source:MGI Symbo	0.19353
ENSMUSG00000013415	Igf2bp1	insulin-like growth factor 2 mRNA binding protein 1	0.19358
ENSMUSG00000089987	Gpx2-ps1	glutathione peroxidase 2, pseudogene 1 [Source:M	0.19361
ENSMUSG00000030374	Strn4	striatin, calmodulin binding protein 4 [Source:MGI S	0.1937
ENSMUSG00000016150	Tenm1	teneurin transmembrane protein 1 [Source:MGI Sy	0.19374
ENSMUSG00000020491	2810021J22Rik	RIKEN cDNA 2810021J22 gene [Source:MGI Symbol	0.19375
ENSMUSG00000027531	Impa1	inositol (myo)-1(or 4)-monophosphatase 1 [Source:	0.19381
ENSMUSG00000010538	Tsacc	TSSK6 activating co-chaperone [Source:MGI Symbo	0.19384
ENSMUSG00000039372	4-Mar	membrane-associated ring finger (C3HC4) 4 [Source	0.19392
ENSMUSG00000040373	Cacng5	calcium channel, voltage-dependent, gamma subur	0.19392
ENSMUSG00000005583	Mef2c	myocyte enhancer factor 2C [Source:MGI Symbol;A	0.194
ENSMUSG00000003435	Supt5	suppressor of Ty 5 [Source:MGI Symbol;Acc:MGI:12	0.19401
ENSMUSG00000024769	Cdc42bpg	CDC42 binding protein kinase gamma (DMPK-like) [	0.19403
ENSMUSG00000038079	Tmem237	transmembrane protein 237 [Source:MGI Symbol;A	0.19403
ENSMUSG00000030854	Ptpn5	protein tyrosine phosphatase, non-receptor type 5	0.19407

ENSMUSG00000057101	Zfp180	zinc finger protein 180 [Source:MGI Symbol;Acc:M(	0.19417
ENSMUSG00000017176	Nt5c3b	5'-nucleotidase, cytosolic IIIB [Source:MGI Symbol;/	0.1942
ENSMUSG00000032717	Mdfi	MyoD family inhibitor [Source:MGI Symbol;Acc:MG	0.19429
ENSMUSG00000027115	Kif18a	kinesin family member 18A [Source:MGI Symbol;Ac	0.19434
ENSMUSG00000026070	Il18r1	interleukin 18 receptor 1 [Source:MGI Symbol;Acc:I	0.19436
ENSMUSG00000005917	Otx1	orthodenticle homolog 1 (Drosophila) [Source:MGI	0.19437
ENSMUSG00000044407	Qk	quaking [Source:MGI Symbol;Acc:MGI:97837]	0.19441
ENSMUSG00000009281	Rarres2	retinoic acid receptor responder (tazarotene induce	0.19455
ENSMUSG00000047343	Mettl21c	methyltransferase like 21C [Source:MGI Symbol;Ac	0.19456
ENSMUSG00000031774	Fam192a	family with sequence similarity 192, member A [Sou	0.19457
ENSMUSG00000087616	Gm14257	predicted gene 14257 [Source:MGI Symbol;Acc:MG	0.19465
ENSMUSG00000033099	Nol12	nucleolar protein 12 [Source:MGI Symbol;Acc:MGI:	0.1947
ENSMUSG00000014351	Gip	gastric inhibitory polypeptide [Source:MGI Symbol;	0.19471
ENSMUSG00000085636	Gm11769	predicted gene 11769 [Source:MGI Symbol;Acc:MG	0.19478
ENSMUSG00000039552	Rsph4a	radial spoke head 4 homolog A (Chlamydomonas) [	0.19479
ENSMUSG00000085968	Gm13027	predicted gene 13027 [Source:MGI Symbol;Acc:MG	0.19486
ENSMUSG00000028197	Col24a1	collagen, type XXIV, alpha 1 [Source:MGI Symbol;A	0.19489
ENSMUSG00000031785	Gpr56	G protein-coupled receptor 56 [Source:MGI Symbo	0.19491
ENSMUSG00000097331	F420014N23Rik	RIKEN cDNA F420014N23 gene [Source:MGI Symbo	0.19495
ENSMUSG00000092417	Gpank1	G patch domain and ankyrin repeats 1 [Source:MGI	0.19501
ENSMUSG00000032182	Yipf2	Yip1 domain family, member 2 [Source:MGI Symbo	0.19515
ENSMUSG00000040481	Bptf	bromodomain PHD finger transcription factor [Sour	0.19515
ENSMUSG00000091845	Gm4604	predicted gene 4604 [Source:MGI Symbol;Acc:MGI:	0.19515
ENSMUSG00000043858	Nup62	nucleoporin 62 [Source:MGI Symbol;Acc:MGI:1351	0.19524
ENSMUSG00000037805	Rpl10a	ribosomal protein L10A [Source:MGI Symbol;Acc:M	0.19526
ENSMUSG00000009145	Dqx1	DEAQ RNA-dependent ATPase [Source:MGI Symbol	0.19529
ENSMUSG00000087328	Gm12120	predicted gene 12120 [Source:MGI Symbol;Acc:MG	0.19535
ENSMUSG00000017747	Ghdc	GH3 domain containing [Source:MGI Symbol;Acc:M	0.1954
ENSMUSG00000070934	Rraga	Ras-related GTP binding A [Source:MGI Symbol;Acc	0.19544
ENSMUSG00000045252	Zfp574	zinc finger protein 574 [Source:MGI Symbol;Acc:M(	0.19547
ENSMUSG00000033949	Trim36	tripartite motif-containing 36 [Source:MGI Symbol;	0.19549
ENSMUSG00000025147	Mob2	MOB kinase activator 2 [Source:MGI Symbol;Acc:M	0.19576
ENSMUSG00000091864	Gm17102	predicted gene 17102 [Source:MGI Symbol;Acc:MG	0.19578
ENSMUSG00000030314	Atg7	autophagy related 7 [Source:MGI Symbol;Acc:MGI:	0.19582
ENSMUSG00000010609	Psen2	presenilin 2 [Source:MGI Symbol;Acc:MGI:109284]	0.19592
ENSMUSG00000068245	Phf11d	PHD finger protein 11D [Source:MGI Symbol;Acc:M	0.19593
ENSMUSG00000029122	Evc	Ellis van Creveld gene syndrome [Source:MGI Symb	0.19596
ENSMUSG00000020083	2010107G23Rik	RIKEN cDNA 2010107G23 gene [Source:MGI Symbo	0.19599
ENSMUSG00000028917	Plekhm2	pleckstrin homology domain containing, family M (	0.19605
ENSMUSG00000031639	Tlr3	toll-like receptor 3 [Source:MGI Symbol;Acc:MGI:2	0.19613
ENSMUSG00000001827	Folr1	folate receptor 1 (adult) [Source:MGI Symbol;Acc:N	0.19615
ENSMUSG00000033671	Cep350	centrosomal protein 350 [Source:MGI Symbol;Acc:I	0.1964
ENSMUSG00000021248	Tmed10	transmembrane emp24-like trafficking protein 10 (	0.19642
ENSMUSG00000006345	Ggt1	gamma-glutamyltransferase 1 [Source:MGI Symbol	0.19645
ENSMUSG00000078550	Dear1	dual endothelin 1/angiotensin II receptor 1 [Source	0.19645
ENSMUSG00000086475	Gm12139	predicted gene 12139 [Source:MGI Symbol;Acc:MG	0.19652
ENSMUSG00000024959	Bad	BCL2-associated agonist of cell death [Source:MGI S	0.19653

ENSMUSG00000057132	Rpgrip1	retinitis pigmentosa GTPase regulator interacting p	0.19658
ENSMUSG00000062905	Vmn1r32	vomeronasal 1 receptor 32 [Source:MGI Symbol;Ac	0.19658
ENSMUSG00000060032	H2afj	H2A histone family, member J [Source:MGI Symbol	0.19659
ENSMUSG00000026269	Rnpepl1	arginyl aminopeptidase (aminopeptidase B)-like 1 [	0.19663
ENSMUSG00000068699	Flnc	filamin C, gamma [Source:MGI Symbol;Acc:MGI:95	0.19668
ENSMUSG00000031585	Gtf2e2	general transcription factor II E, polypeptide 2 (beta	0.19671
ENSMUSG00000026558	Uck2	uridine-cytidine kinase 2 [Source:MGI Symbol;Acc:M	0.19678
ENSMUSG00000033389	Arhgap44	Rho GTPase activating protein 44 [Source:MGI Sym	0.19679
ENSMUSG00000041765	Ubac2	ubiquitin associated domain containing 2 [Source:M	0.19681
ENSMUSG00000056124	B4galt6	UDP-Gal:betaGlcNAc beta 1,4-galactosyltransferase	0.19693
ENSMUSG00000022946	Dopey2	dopey family member 2 [Source:MGI Symbol;Acc:M	0.19699
ENSMUSG00000029442	Wdr66	WD repeat domain 66 [Source:MGI Symbol;Acc:MG	0.19711
ENSMUSG00000078880	Gm14308	predicted gene 14308 [Source:MGI Symbol;Acc:MG	0.19718
ENSMUSG00000086699	Gm16510	predicted pseudogene 16510 [Source:MGI Symbol;	0.19719
ENSMUSG00000061518	Cox5b	cytochrome c oxidase subunit Vb [Source:MGI Sym	0.19727
ENSMUSG00000030630	Fah	fumarylacetoacetate hydrolase [Source:MGI Symbo	0.19728
ENSMUSG00000006299	Aamp	angio-associated migratory protein [Source:MGI Sy	0.19729
ENSMUSG00000019082	Slc25a22	solute carrier family 25 (mitochondrial carrier, gluta	0.19731
ENSMUSG00000082082	Gm13230	predicted gene 13230 [Source:MGI Symbol;Acc:MG	0.19733
ENSMUSG00000030451	Herc2	hect (homologous to the E6-AP (UBE3A) carboxyl te	0.19737
ENSMUSG00000043831	Lysmd4	LysM, putative peptidoglycan-binding, domain cont	0.19739
ENSMUSG00000042105	Inpp5f	inositol polyphosphate-5-phosphatase F [Source:M	0.19744
ENSMUSG00000021477	Ctsl	cathepsin L [Source:MGI Symbol;Acc:MGI:88564]	0.19745
ENSMUSG00000056214	Pard6g	par-6 partitioning defective 6 homolog gamma (C. e	0.19745
ENSMUSG00000053588	A730061H03Rik	RIKEN cDNA A730061H03 gene [Source:MGI Symbo	0.19746
ENSMUSG00000001313	Rnd2	Rho family GTPase 2 [Source:MGI Symbol;Acc:MGI:	0.19747
ENSMUSG00000049300	Prmt6	protein arginine N-methyltransferase 6 [Source:MG	0.19747
ENSMUSG00000066697	Gm10172	predicted gene 10172 [Source:MGI Symbol;Acc:MG	0.19748
ENSMUSG00000029162	Khk	ketohexokinase [Source:MGI Symbol;Acc:MGI:1096	0.19751
ENSMUSG00000050397	Foxl2	forkhead box L2 [Source:MGI Symbol;Acc:MGI:1349	0.19761
ENSMUSG00000051747	Ttn	titin [Source:MGI Symbol;Acc:MGI:98864]	0.19763
ENSMUSG00000022515	Anks3	ankyrin repeat and sterile alpha motif domain cont	0.19777
ENSMUSG00000020401	Fam71b	family with sequence similarity 71, member B [Sour	0.19792
ENSMUSG00000008438	Adam21	a disintegrin and metallopeptidase domain 21 [Sou	0.19795
ENSMUSG00000024043	Arhgap28	Rho GTPase activating protein 28 [Source:MGI Sym	0.198
ENSMUSG00000005354	Txn2	thioredoxin 2 [Source:MGI Symbol;Acc:MGI:192946	0.19803
ENSMUSG00000084942	7330404K18Rik	RIKEN cDNA 7330404K18 gene [Source:MGI Symbo	0.19811
ENSMUSG00000070796	Psg21	pregnancy-specific glycoprotein 21 [Source:MGI Sy	0.19812
ENSMUSG00000088629	Gm23030	predicted gene, 23030 [Source:MGI Symbol;Acc:MG	0.19824
ENSMUSG00000025932	Eya1	eyes absent 1 homolog (Drosophila) [Source:MGI Sy	0.19839
ENSMUSG00000090329	Gm17160	predicted gene 17160 [Source:MGI Symbol;Acc:MG	0.1984
ENSMUSG00000044024	Rell2	RELT-like 2 [Source:MGI Symbol;Acc:MGI:1918044]	0.19843
ENSMUSG00000027799	Nbea	neurobeachin [Source:MGI Symbol;Acc:MGI:13470	0.19848
ENSMUSG00000097274	Gm26564	predicted gene, 26564 [Source:MGI Symbol;Acc:MG	0.19856
ENSMUSG00000043300	B3galnt1	UDP-GalNAc:betaGlcNAc beta 1,3-galactosaminyltr	0.19859
ENSMUSG00000097091	4930526L06Rik	RIKEN cDNA 4930526L06 gene [Source:MGI Symbo	0.19861
ENSMUSG00000038534	Osbpl7	oxysterol binding protein-like 7 [Source:MGI Symbo	0.19864

ENSMUSG00000050541	Adra1b	adrenergic receptor, alpha 1b [Source:MGI Symbol;	0.19864
ENSMUSG00000020721	Helz	helicase with zinc finger domain [Source:MGI Symb	0.19867
ENSMUSG00000031148	Gpkow	G patch domain and KOW motifs [Source:MGI Syml	0.19867
ENSMUSG00000053113	Socs3	suppressor of cytokine signaling 3 [Source:MGI Sym	0.19867
ENSMUSG00000010755	Cars	cysteinyl-tRNA synthetase [Source:MGI Symbol;Acc	0.19876
ENSMUSG00000001419	Mef2d	myocyte enhancer factor 2D [Source:MGI Symbol;A	0.19886
ENSMUSG00000064389	Gm26446	predicted gene, 26446 [Source:MGI Symbol;Acc:MG	0.19888
ENSMUSG00000063972	Nr6a1	nuclear receptor subfamily 6, group A, member 1 [S	0.19889
ENSMUSG00000026784	Pdss1	prenyl (solanesyl) diphosphate synthase, subunit 1	0.19902
ENSMUSG00000053007	Creb5	cAMP responsive element binding protein 5 [Sourc	0.19906
ENSMUSG00000026150	Mff	mitochondrial fission factor [Source:MGI Symbol;Ac	0.19907
ENSMUSG00000022035	Ccdc25	coiled-coil domain containing 25 [Source:MGI Symb	0.19909
ENSMUSG00000047649	Cd3eap	CD3E antigen, epsilon polypeptide associated prote	0.19912
ENSMUSG00000026349	Ccnt2	cyclin T2 [Source:MGI Symbol;Acc:MGI:1920199]	0.19914
ENSMUSG00000074676	Foxs1	forkhead box S1 [Source:MGI Symbol;Acc:MGI:9554	0.19917
ENSMUSG00000033502	Cdc14a	CDC14 cell division cycle 14A [Source:MGI Symbol;A	0.19924
ENSMUSG00000039879	Heca	headcase homolog (Drosophila) [Source:MGI Symb	0.19927
ENSMUSG00000036962	Gm101	predicted gene 101 [Source:MGI Symbol;Acc:MGI:2	0.19935
ENSMUSG00000020272	Stk10	serine/threonine kinase 10 [Source:MGI Symbol;Ac	0.1994
ENSMUSG00000043661	Gm9785	predicted pseudogene 9785 [Source:MGI Symbol;A	0.19949
ENSMUSG00000024665	Fads2	fatty acid desaturase 2 [Source:MGI Symbol;Acc:MG	0.1995
ENSMUSG00000029516	Cit	citron [Source:MGI Symbol;Acc:MGI:105313]	0.19966
ENSMUSG00000001751	Naglu	alpha-N-acetylglucosaminidase (Sanfilippo disease	0.19973
ENSMUSG00000056698	Elmod3	ELMO/CED-12 domain containing 3 [Source:MGI Sy	0.19974
ENSMUSG00000006705	Pknox1	Pbx/knotted 1 homeobox [Source:MGI Symbol;Acc:	0.19978
ENSMUSG00000023259	Slc26a6	solute carrier family 26, member 6 [Source:MGI Syr	0.19985
ENSMUSG00000085143	Gm11520	predicted gene 11520 [Source:MGI Symbol;Acc:MG	0.19993
ENSMUSG00000062866	Phactr2	phosphatase and actin regulator 2 [Source:MGI Syn	0.19994
ENSMUSG00000031904	Slc7a6	solute carrier family 7 (cationic amino acid transpo	0.19999
ENSMUSG00000091426	Gm17228	predicted gene 17228 [Source:MGI Symbol;Acc:MG	0.19999
ENSMUSG00000022658	Tagln3	transgelin 3 [Source:MGI Symbol;Acc:MGI:1926784	0.20001
ENSMUSG00000095724	Gm21319	predicted gene, 21319 [Source:MGI Symbol;Acc:MG	0.20005
ENSMUSG00000028312	Smc2	structural maintenance of chromosomes 2 [Source:	0.20006
ENSMUSG00000019659	Ccdc12	coiled-coil domain containing 12 [Source:MGI Symb	0.20019
ENSMUSG00000033107	Rnf125	ring finger protein 125 [Source:MGI Symbol;Acc:MG	0.2002
ENSMUSG00000029121	Crmp1	collapsin response mediator protein 1 [Source:MGI	0.20029
ENSMUSG00000038256	Bcl9	B cell CLL/lymphoma 9 [Source:MGI Symbol;Acc:MG	0.20035
ENSMUSG00000086741	Gm15816	predicted gene 15816 [Source:MGI Symbol;Acc:MG	0.20035
ENSMUSG00000018433	Nol11	nucleolar protein 11 [Source:MGI Symbol;Acc:MGI:	0.20041
ENSMUSG00000030232	Aebp2	AE binding protein 2 [Source:MGI Symbol;Acc:MGI:	0.20044
ENSMUSG00000024012	Mtch1	mitochondrial carrier homolog 1 (C. elegans) [Sourc	0.20046
ENSMUSG00000000325	Arvcf	armadillo repeat gene deleted in velo-cardio-facial	0.20047
ENSMUSG00000004567	Mcoln1	mucolipin 1 [Source:MGI Symbol;Acc:MGI:1890498	0.20072
ENSMUSG00000018651	Tada2a	transcriptional adaptor 2A [Source:MGI Symbol;Acc	0.20074
ENSMUSG00000041912	Tdrkh	tudor and KH domain containing protein [Source:M	0.20084
ENSMUSG00000073557	Ppp1r12b	protein phosphatase 1, regulatory (inhibitor) subur	0.20084
ENSMUSG00000097129	4930507D05Rik	RIKEN cDNA 4930507D05 gene [Source:MGI Symbo	0.20086

ENSMUSG00000097649	Gm10561	predicted gene 10561 [Source:MGI Symbol;Acc:MG	0.20091
ENSMUSG00000021327	Zkscan3	zinc finger with KRAB and SCAN domains 3 [Source:	0.20096
ENSMUSG00000036278	Macrod1	MACRO domain containing 1 [Source:MGI Symbol;A	0.20097
ENSMUSG00000044496	2510039O18Rik	RIKEN cDNA 2510039O18 gene [Source:MGI Symbo	0.20098
ENSMUSG00000030530	Furin	furin (paired basic amino acid cleaving enzyme) [So	0.20102
ENSMUSG00000087190	D430001F17Rik	RIKEN cDNA D430001F17 gene [Source:MGI Symbo	0.20107
ENSMUSG00000091952	Gm17709	predicted gene, 17709 [Source:MGI Symbol;Acc:MG	0.20108
ENSMUSG00000022453	Naga	N-acetyl galactosaminidase, alpha [Source:MGI Sym	0.20113
ENSMUSG00000005621	Zfp592	zinc finger protein 592 [Source:MGI Symbol;Acc:MG	0.20117
ENSMUSG00000010651	Acaa1b	acetyl-Coenzyme A acyltransferase 1B [Source:MGI	0.20117
ENSMUSG00000039661	Dusp26	dual specificity phosphatase 26 (putative) [Source:M	0.20121
ENSMUSG00000086191	1110035M17Rik	RIKEN cDNA 1110035M17 gene [Source:MGI Symbo	0.20122
ENSMUSG00000071074	Yipf3	Yip1 domain family, member 3 [Source:MGI Symbo	0.20126
ENSMUSG00000022390	Zc3h7b	zinc finger CCCH type containing 7B [Source:MGI Sy	0.20127
ENSMUSG00000037740	Mrps26	mitochondrial ribosomal protein S26 [Source:MGI S	0.20128
ENSMUSG00000055639	Dach1	dachshund 1 (Drosophila) [Source:MGI Symbol;Acc	0.20128
ENSMUSG00000000486	1-Sep	septin 1 [Source:MGI Symbol;Acc:MGI:1858916]	0.20129
ENSMUSG00000032470	Mras	muscle and microspikes RAS [Source:MGI Symbol;A	0.20129
ENSMUSG00000041605	5730559C18Rik	RIKEN cDNA 5730559C18 gene [Source:MGI Symbo	0.2013
ENSMUSG00000039714	Cplx3	complexin 3 [Source:MGI Symbol;Acc:MGI:238457	0.20132
ENSMUSG00000086849	Gm13840	predicted gene 13840 [Source:MGI Symbol;Acc:MG	0.20134
ENSMUSG00000002871	Tpra1	transmembrane protein, adipocyte asscociated 1 [S	0.20136
ENSMUSG00000031027	Stk33	serine/threonine kinase 33 [Source:MGI Symbol;Ac	0.20136
ENSMUSG00000058351	Smim4	small integral membrane protein 4 [Source:MGI Sym	0.20136
ENSMUSG00000054773	4930566N20Rik	RIKEN cDNA 4930566N20 gene [Source:MGI Symbo	0.2014
ENSMUSG00000022130	Tgds	TDP-glucose 4,6-dehydratase [Source:MGI Symbol;	0.20143
ENSMUSG00000041301	Cftr	cystic fibrosis transmembrane conductance regulat	0.20149
ENSMUSG00000029314	Agpat9	1-acylglycerol-3-phosphate O-acyltransferase 9 [So	0.2015
ENSMUSG00000020447	Npc1l1	NPC1-like 1 [Source:MGI Symbol;Acc:MGI:2685089	0.20151
ENSMUSG00000097008	Gm26860	predicted gene, 26860 [Source:MGI Symbol;Acc:MG	0.20151
ENSMUSG00000097667	Gm26764	predicted gene, 26764 [Source:MGI Symbol;Acc:MG	0.20156
ENSMUSG00000067714	Lpar5	lysophosphatidic acid receptor 5 [Source:MGI Symb	0.20162
ENSMUSG00000086930	Gm14873	predicted gene 14873 [Source:MGI Symbol;Acc:MG	0.20162
ENSMUSG00000020804	Aanat	arylalkylamine N-acetyltransferase [Source:MGI Sym	0.20168
ENSMUSG00000058690	Ccser2	coiled-coil serine rich 2 [Source:MGI Symbol;Acc:M	0.20169
ENSMUSG00000018474	Chd3	chromodomain helicase DNA binding protein 3 [Sou	0.20173
ENSMUSG00000020672	Sntg2	syntrophin, gamma 2 [Source:MGI Symbol;Acc:MGI	0.20173
ENSMUSG00000075297	H60b	histocompatibility 60b [Source:MGI Symbol;Acc:MG	0.20176
ENSMUSG00000080538	Gm25541	predicted gene, 25541 [Source:MGI Symbol;Acc:MG	0.20178
ENSMUSG00000074139	1700057G04Rik	RIKEN cDNA 1700057G04 gene [Source:MGI Symbo	0.20184
ENSMUSG00000021879	Dnah12	dynein, axonemal, heavy chain 12 [Source:MGI Sym	0.20191
ENSMUSG00000087719	Gm26464	predicted gene, 26464 [Source:MGI Symbol;Acc:MG	0.20195
ENSMUSG00000024401	Tnf	tumor necrosis factor [Source:MGI Symbol;Acc:MG	0.20196
ENSMUSG00000069808	Fam57a	family with sequence similarity 57, member A [Sour	0.20198
ENSMUSG00000036087	Slain2	SLAIN motif family, member 2 [Source:MGI Symbol	0.20212
ENSMUSG00000030357	Fkbp4	FK506 binding protein 4 [Source:MGI Symbol;Acc:M	0.20215
ENSMUSG00000079103	Tgm7	transglutaminase 7 [Source:MGI Symbol;Acc:MGI:2	0.20215

ENSMUSG00000050144	Slc25a44	solute carrier family 25, member 44 [Source:MGI Sy	0.2022
ENSMUSG00000039849	Pcif1	PDX1 C-terminal inhibiting factor 1 [Source:MGI Syr	0.20226
ENSMUSG00000034263	Vwa9	von Willebrand factor A domain containing 9 [Sourc	0.20227
ENSMUSG00000024403	Atp6v1g2	ATPase, H+ transporting, lysosomal V1 subunit G2 [	0.20233
ENSMUSG00000037563	Rps16	ribosomal protein S16 [Source:MGI Symbol;Acc:MG	0.20239
ENSMUSG00000097401	Gm26744	predicted gene, 26744 [Source:MGI Symbol;Acc:MG	0.20241
ENSMUSG00000026858	Fam73b	family with sequence similarity 73, member B [Sour	0.20246
ENSMUSG00000042978	Sbk1	SH3-binding kinase 1 [Source:MGI Symbol;Acc:MGI	0.20247
ENSMUSG00000074896	Ifit3	interferon-induced protein with tetratricopeptide r	0.20247
ENSMUSG00000004562	Arhgef40	Rho guanine nucleotide exchange factor (GEF) 40 [S	0.20249
ENSMUSG00000006269	Atp6v1b1	ATPase, H+ transporting, lysosomal V1 subunit B1 [	0.2025
ENSMUSG00000021983	Atp8a2	ATPase, aminophospholipid transporter-like, class I	0.2025
ENSMUSG00000074346	1700095B22Rik	RIKEN cDNA 1700095B22 gene [Source:MGI Symbo	0.20254
ENSMUSG00000047547	Cltb	clathrin, light polypeptide (Lcb) [Source:MGI Symbo	0.20258
ENSMUSG00000087336	Gm15860	predicted gene 15860 [Source:MGI Symbol;Acc:MG	0.20263
ENSMUSG00000022770	Dlg1	discs, large homolog 1 (Drosophila) [Source:MGI Sy	0.20267
ENSMUSG00000018750	Zbtb4	zinc finger and BTB domain containing 4 [Source:M	0.20268
ENSMUSG00000029580	Actb	actin, beta [Source:MGI Symbol;Acc:MGI:87904]	0.2027
ENSMUSG00000024855	Pacs1	phosphofurin acidic cluster sorting protein 1 [Sourc	0.20274
ENSMUSG00000055197	Fev	FEV (ETS oncogene family) [Source:MGI Symbol;Acc	0.20275
ENSMUSG00000024792	Zfpl1	zinc finger like protein 1 [Source:MGI Symbol;Acc:M	0.2028
ENSMUSG00000031891	Hsd11b2	hydroxysteroid 11-beta dehydrogenase 2 [Source:M	0.2028
ENSMUSG00000021608	Lpcat1	lysophosphatidylcholine acyltransferase 1 [Source:I	0.20283
ENSMUSG00000025450	Gm9752	predicted gene 9752 [Source:MGI Symbol;Acc:MGI:	0.20285
ENSMUSG00000021990	Spata13	spermatogenesis associated 13 [Source:MGI Symbo	0.20286
ENSMUSG00000011254	Thg1l	tRNA-histidine guanylyltransferase 1-like (S. cerevis	0.20295
ENSMUSG00000041025	Iffo2	intermediate filament family orphan 2 [Source:MGI	0.20296
ENSMUSG00000067081	Asb18	ankyrin repeat and SOCS box-containing 18 [Source	0.20298
ENSMUSG00000018849	Wwc1	WW, C2 and coiled-coil domain containing 1 [Sourc	0.203
ENSMUSG00000032640	Chsy1	chondroitin sulfate synthase 1 [Source:MGI Symbol	0.203
ENSMUSG00000053070	9230110C19Rik	RIKEN cDNA 9230110C19 gene [Source:MGI Symbo	0.203
ENSMUSG00000038722	Bud31	BUD31 homolog (yeast) [Source:MGI Symbol;Acc:M	0.20305
ENSMUSG00000097162	2310010J17Rik	RIKEN cDNA 2310010J17 gene [Source:MGI Symbol	0.20308
ENSMUSG00000039630	Hnrnpu	heterogeneous nuclear ribonucleoprotein U [Sourc	0.20311
ENSMUSG00000032803	Cdv3	carnitine deficiency-associated gene expressed in v	0.20316
ENSMUSG00000027472	Pdrg1	p53 and DNA damage regulated 1 [Source:MGI Sym	0.20317
ENSMUSG00000009588	St6galnac1	ST6 (alpha-N-acetyl-neuraminyl-2,3-beta-galactosyl	0.20326
ENSMUSG00000090122	Kcne1l	potassium voltage-gated channel, Isk-related family	0.20332
ENSMUSG00000073478	D730003I15Rik	RIKEN cDNA D730003I15 gene [Source:MGI Symbol	0.20335
ENSMUSG00000004508	Gab2	growth factor receptor bound protein 2-associated	0.20339
ENSMUSG00000029577	Ube3b	ubiquitin protein ligase E3B [Source:MGI Symbol;Ac	0.2034
ENSMUSG00000029551	Psmg3	proteasome (prosome, macropain) assembly chape	0.20345
ENSMUSG00000092827	Mir3091	microRNA 3091 [Source:MGI Symbol;Acc:MGI:4834	0.20346
ENSMUSG00000032126	Hmbs	hydroxymethylbilane synthase [Source:MGI Symbo	0.20349
ENSMUSG00000070802	Pnmal2	PNMA-like 2 [Source:MGI Symbol;Acc:MGI:364585	0.20351
ENSMUSG00000054958	Nt5c1a	5'-nucleotidase, cytosolic IA [Source:MGI Symbol;A	0.20354
ENSMUSG00000003299	Mrpl4	mitochondrial ribosomal protein L4 [Source:MGI Sy	0.20358

ENSMUSG00000083454	Gm13226	predicted gene 13226 [Source:MGI Symbol;Acc:MG	0.20361
ENSMUSG00000001173	Ocrl	oculocerebrorenal syndrome of Lowe [Source:MGI	0.20363
ENSMUSG00000024430	Cabyr	calcium-binding tyrosine-(Y)-phosphorylation regul	0.20364
ENSMUSG00000022820	Ndufb4	NADH dehydrogenase (ubiquinone) 1 beta subcom	0.20368
ENSMUSG00000025825	Iscu	IscU iron-sulfur cluster scaffold homolog (E. coli) [S	0.20371
ENSMUSG00000024901	Peli3	pellino 3 [Source:MGI Symbol;Acc:MGI:1924963]	0.20382
ENSMUSG00000039672	Kcne2	potassium voltage-gated channel, Isk-related subfa	0.20385
ENSMUSG00000055540	Epha6	Eph receptor A6 [Source:MGI Symbol;Acc:MGI:108	0.20385
ENSMUSG00000029507	Pus1	pseudouridine synthase 1 [Source:MGI Symbol;Acc	0.20388
ENSMUSG00000043794	D830025C05Rik	RIKEN cDNA D830025C05 gene [Source:MGI Symbo	0.20395
ENSMUSG00000019464	Ptger1	prostaglandin E receptor 1 (subtype EP1) [Source:M	0.20399
ENSMUSG00000029869	Ephb6	Eph receptor B6 [Source:MGI Symbol;Acc:MGI:109	0.204
ENSMUSG00000093502	Gm20700	predicted gene 20700 [Source:MGI Symbol;Acc:MG	0.20402
ENSMUSG00000027323	Rad51	RAD51 homolog [Source:MGI Symbol;Acc:MGI:978	0.20412
ENSMUSG00000036062	N28178	expressed sequence N28178 [Source:MGI Symbol;A	0.20412
ENSMUSG00000063576	Klhdc3	kelch domain containing 3 [Source:MGI Symbol;Acc	0.20412
ENSMUSG00000041135	Ripk2	receptor (TNFRSF)-interacting serine-threonine kin	0.20417
ENSMUSG00000024841	Eif1ad	eukaryotic translation initiation factor 1A domain c	0.20422
ENSMUSG00000037990	Sh3rf3	SH3 domain containing ring finger 3 [Source:MGI Sy	0.20424
ENSMUSG00000040711	Sh3pxd2b	SH3 and PX domains 2B [Source:MGI Symbol;Acc:M	0.20424
ENSMUSG00000053128	Rnf26	ring finger protein 26 [Source:MGI Symbol;Acc:MG	0.20426
ENSMUSG00000032353	Tmed3	transmembrane emp24 domain containing 3 [Sourc	0.20436
ENSMUSG00000048616	Nog	noggin [Source:MGI Symbol;Acc:MGI:104327]	0.20441
ENSMUSG00000080645	Mir1198	microRNA 1198 [Source:MGI Symbol;Acc:MGI:3783	0.20444
ENSMUSG00000046985	Tapt1	transmembrane anterior posterior transformation	0.20445
ENSMUSG00000027428	Rbbp9	retinoblastoma binding protein 9 [Source:MGI Sym	0.20456
ENSMUSG00000021613	Hapln1	hyaluronan and proteoglycan link protein 1 [Source	0.20462
ENSMUSG00000067149	Igj	immunoglobulin joining chain [Source:MGI Symbol;	0.20465
ENSMUSG00000020952	Scfd1	Sec1 family domain containing 1 [Source:MGI Symb	0.20466
ENSMUSG00000040113	Mettl11b	methyltransferase like 11B [Source:MGI Symbol;Ac	0.20467
ENSMUSG00000072423	Psmb11	proteasome (prosome, macropain) subunit, beta ty	0.20471
ENSMUSG00000035769	Xylb	xylulokinase homolog (H. influenzae) [Source:MGI S	0.20491
ENSMUSG00000038485	Socs7	suppressor of cytokine signaling 7 [Source:MGI Sym	0.20491
ENSMUSG00000050538	B230217C12Rik	RIKEN cDNA B230217C12 gene [Source:MGI Symbo	0.20491
ENSMUSG00000037628	Cdkn3	cyclin-dependent kinase inhibitor 3 [Source:MGI Sy	0.20492
ENSMUSG00000025283	Sat1	spermidine/spermine N1-acetyl transferase 1 [Sour	0.20495
ENSMUSG00000081824	BC002163	cDNA sequence BC002163 [Source:MGI Symbol;Acc	0.20502
ENSMUSG00000030499	Kctd15	potassium channel tetramerisation domain contain	0.20504
ENSMUSG00000023972	Ptk7	PTK7 protein tyrosine kinase 7 [Source:MGI Symbol	0.20507
ENSMUSG00000028264	Spaca1	sperm acrosome associated 1 [Source:MGI Symbol;	0.2051
ENSMUSG00000032582	Rbm6	RNA binding motif protein 6 [Source:MGI Symbol;A	0.20515
ENSMUSG00000085891	Gm14634	predicted gene 14634 [Source:MGI Symbol;Acc:MG	0.20536
ENSMUSG00000019734	Tmc4	transmembrane channel-like gene family 4 [Source	0.20538
ENSMUSG00000026812	Tsc1	tuberous sclerosis 1 [Source:MGI Symbol;Acc:MGI:	0.20538
ENSMUSG00000057836	Xlr3a	X-linked lymphocyte-regulated 3A [Source:MGI Sym	0.2054
ENSMUSG00000028932	Psmc2	proteasome (prosome, macropain) 26S subunit, AT	0.20555
ENSMUSG00000022206	Npr3	natriuretic peptide receptor 3 [Source:MGI Symbol	0.2056

ENSMUSG00000030036	Mogs	mannosyl-oligosaccharide glucosidase [Source:MGI	0.2056
ENSMUSG00000097109	Gm26580	predicted gene, 26580 [Source:MGI Symbol;Acc:MG	0.20564
ENSMUSG00000041119	Pde9a	phosphodiesterase 9A [Source:MGI Symbol;Acc:MG	0.20566
ENSMUSG00000005501	Usp40	ubiquitin specific peptidase 40 [Source:MGI Symbo	0.20577
ENSMUSG00000092593	Gm20492	predicted gene 20492 [Source:MGI Symbol;Acc:MG	0.20587
ENSMUSG00000022668	Gtpbp8	GTP-binding protein 8 (putative) [Source:MGI Symb	0.20593
ENSMUSG00000032125	Robo4	roundabout homolog 4 (Drosophila) [Source:MGI S	0.20593
ENSMUSG00000035431	Sstr1	somatostatin receptor 1 [Source:MGI Symbol;Acc:M	0.20595
ENSMUSG00000029249	Rest	RE1-silencing transcription factor [Source:MGI Sym	0.20604
ENSMUSG00000020153	Ndufs7	NADH dehydrogenase (ubiquinone) Fe-S protein 7 [	0.20623
ENSMUSG00000064262	Gimap8	GTPase, IMAP family member 8 [Source:MGI Symbo	0.20627
ENSMUSG00000033335	Dnm2	dynamin 2 [Source:MGI Symbol;Acc:MGI:109547]	0.20631
ENSMUSG00000019055	Plod1	procollagen-lysine, 2-oxoglutarate 5-dioxygenase 1	0.20639
ENSMUSG00000021242	Npc2	Niemann Pick type C2 [Source:MGI Symbol;Acc:MG	0.20641
ENSMUSG00000057716	Tmem178b	transmembrane protein 178B [Source:MGI Symbol;	0.20648
ENSMUSG00000024647	Cbln2	cerebellin 2 precursor protein [Source:MGI Symbol	0.2065
ENSMUSG00000050069	Grem2	gremlin 2 homolog, cysteine knot superfamily (Xen	0.2066
ENSMUSG00000012017	Scarf2	scavenger receptor class F, member 2 [Source:MGI	0.20668
ENSMUSG00000032344	Mb21d1	Mab-21 domain containing 1 [Source:MGI Symbol;A	0.20669
ENSMUSG00000010047	Hyal2	hyaluronoglucosaminidase 2 [Source:MGI Symbol;A	0.20679
ENSMUSG00000035372	1810055G02Rik	RIKEN cDNA 1810055G02 gene [Source:MGI Symbo	0.20704
ENSMUSG00000048756	Foxo3	forkhead box O3 [Source:MGI Symbol;Acc:MGI:189	0.20711
ENSMUSG00000000308	Ckmt1	creatine kinase, mitochondrial 1, ubiquitous [Sourc	0.20715
ENSMUSG00000050315	Synpo2	synaptopodin 2 [Source:MGI Symbol;Acc:MGI:2153	0.20725
ENSMUSG00000050332	Amer1	APC membrane recruitment 1 [Source:MGI Symbol	0.20753
ENSMUSG00000032735	Ablim3	actin binding LIM protein family, member 3 [Source	0.20754
ENSMUSG00000033526	Ppip5k1	diphosphoinositol pentakisphosphate kinase 1 [Sou	0.20754
ENSMUSG00000035790	Cep19	centrosomal protein 19 [Source:MGI Symbol;Acc:M	0.20756
ENSMUSG00000035863	Palm	paralemmin [Source:MGI Symbol;Acc:MGI:1261814	0.20763
ENSMUSG00000063611	Gm10134	predicted gene 10134 [Source:MGI Symbol;Acc:MG	0.20764
ENSMUSG00000097184	4632428C04Rik	RIKEN cDNA 4632428C04 gene [Source:MGI Symbo	0.20766
ENSMUSG00000008822	Acyp1	acylphosphatase 1, erythrocyte (common) type [So	0.20771
ENSMUSG00000004872	Pax3	paired box gene 3 [Source:MGI Symbol;Acc:MGI:97	0.20775
ENSMUSG00000030610	Det1	de-etiolated homolog 1 (Arabidopsis) [Source:MGI	0.20783
ENSMUSG00000096979	Gm26880	predicted gene, 26880 [Source:MGI Symbol;Acc:MG	0.20786
ENSMUSG00000075415	Fnbp1	formin binding protein 1 [Source:MGI Symbol;Acc:M	0.20792
ENSMUSG00000034863	Ano8	anoctamin 8 [Source:MGI Symbol;Acc:MGI:2687327	0.20795
ENSMUSG00000046743	Fat4	FAT tumor suppressor homolog 4 (Drosophila) [Sou	0.20801
ENSMUSG00000085629	Gm11697	predicted gene 11697 [Source:MGI Symbol;Acc:MG	0.20802
ENSMUSG00000019578	Ubxn6	UBX domain protein 6 [Source:MGI Symbol;Acc:MG	0.20807
ENSMUSG00000020893	Per1	period circadian clock 1 [Source:MGI Symbol;Acc:M	0.20814
ENSMUSG00000020492	Ska2	spindle and kinetochore associated complex subun	0.20815
ENSMUSG00000033596	Rfwd3	ring finger and WD repeat domain 3 [Source:MGI S	0.20817
ENSMUSG00000047181	Samd14	sterile alpha motif domain containing 14 [Source:M	0.20817
ENSMUSG00000015711	Prune	prune homolog (Drosophila) [Source:MGI Symbol;A	0.20823
ENSMUSG00000036030	Prtg	protogenin homolog (Gallus gallus) [Source:MGI Sy	0.20828
ENSMUSG00000035778	Ggta1	glycoprotein galactosyltransferase alpha 1, 3 [Sourc	0.20858

ENSMUSG00000031805	Jak3	Janus kinase 3 [Source:MGI Symbol;Acc:MGI:99928	0.20859
ENSMUSG00000028851	Nudc	nuclear distribution gene C homolog (Aspergillus) [S	0.20864
ENSMUSG00000022792	Yars2	tyrosyl-tRNA synthetase 2 (mitochondrial) [Source:	0.20868
ENSMUSG00000083554	Gm6645	predicted gene 6645 [Source:MGI Symbol;Acc:MGI:	0.20868
ENSMUSG00000086076	Ncr3-ps	natural cytotoxicity triggering receptor 3, pseudoge	0.20871
ENSMUSG00000005087	Cd44	CD44 antigen [Source:MGI Symbol;Acc:MGI:88338]	0.20876
ENSMUSG00000085448	Gm13963	predicted gene 13963 [Source:MGI Symbol;Acc:MG	0.20876
ENSMUSG00000015605	Srf	serum response factor [Source:MGI Symbol;Acc:MG	0.20884
ENSMUSG00000086949	Gm13066	predicted gene 13066 [Source:MGI Symbol;Acc:MG	0.20892
ENSMUSG00000086192	Gm13609	predicted gene 13609 [Source:MGI Symbol;Acc:MG	0.20897
ENSMUSG00000022517	Mgrn1	mahogunin, ring finger 1 [Source:MGI Symbol;Acc:M	0.20898
ENSMUSG00000001964	Emd	emerin [Source:MGI Symbol;Acc:MGI:108117]	0.20901
ENSMUSG00000039244	E130309D02Rik	RIKEN cDNA E130309D02 gene [Source:MGI Symbo	0.20908
ENSMUSG00000097146	Gm4211	predicted gene 4211 [Source:MGI Symbol;Acc:MGI:	0.20914
ENSMUSG00000097239	Gm27029	predicted gene, 27029 [Source:MGI Symbol;Acc:MG	0.20922
ENSMUSG00000057246	BC051142	cDNA sequence BC051142 [Source:MGI Symbol;Acc	0.20923
ENSMUSG00000030091	Nup210	nucleoporin 210 [Source:MGI Symbol;Acc:MGI:185	0.20928
ENSMUSG00000024266	Adad2	adenosine deaminase domain containing 2 [Source	0.20929
ENSMUSG00000095178	Gm22716	predicted gene, 22716 [Source:MGI Symbol;Acc:MG	0.20929
ENSMUSG00000006570	Defb2	defensin beta 2 [Source:MGI Symbol;Acc:MGI:1338	0.20933
ENSMUSG00000049252	Lrp1b	low density lipoprotein-related protein 1B (deleted	0.20944
ENSMUSG00000025377	Enthd2	ENTH domain containing 2 [Source:MGI Symbol;Acc	0.20945
ENSMUSG00000024914	Drap1	Dr1 associated protein 1 (negative cofactor 2 alpha	0.20953
ENSMUSG00000018858	Ict1	immature colon carcinoma transcript 1 [Source:MG	0.20955
ENSMUSG00000031059	Ndufb11	NADH dehydrogenase (ubiquinone) 1 beta subcom	0.20963
ENSMUSG00000023927	Satb1	special AT-rich sequence binding protein 1 [Source:	0.20965
ENSMUSG00000032012	Pvrl1	poliovirus receptor-related 1 [Source:MGI Symbol;A	0.20966
ENSMUSG00000036748	Cuedc2	CUE domain containing 2 [Source:MGI Symbol;Acc:	0.20975
ENSMUSG00000020496	Rnf187	ring finger protein 187 [Source:MGI Symbol;Acc:MG	0.20984
ENSMUSG00000030889	Vwa3a	von Willebrand factor A domain containing 3A [Sou	0.20992
ENSMUSG00000027544	Nfatc2	nuclear factor of activated T cells, cytoplasmic, calc	0.20995
ENSMUSG00000048572	Tmem252	transmembrane protein 252 [Source:MGI Symbol;A	0.21007
ENSMUSG00000064127	Med14	mediator complex subunit 14 [Source:MGI Symbol;	0.21014
ENSMUSG00000059866	Tnip2	TNFAIP3 interacting protein 2 [Source:MGI Symbol;	0.21024
ENSMUSG00000042386	Tex13	testis expressed gene 13 [Source:MGI Symbol;Acc:M	0.21029
ENSMUSG00000003068	Stk11	serine/threonine kinase 11 [Source:MGI Symbol;Ac	0.21035
ENSMUSG00000055102	Zfp819	zinc finger protein 819 [Source:MGI Symbol;Acc:MG	0.21035
ENSMUSG00000030670	Cyp2r1	cytochrome P450, family 2, subfamily r, polypeptid	0.21038
ENSMUSG00000022536	Glyr1	glyoxylate reductase 1 homolog (Arabidopsis) [Sour	0.21053
ENSMUSG00000091523	AU040972	expressed sequence AU040972 [Source:MGI Symbo	0.21083
ENSMUSG00000045482	Trrap	transformation/transcription domain-associated pr	0.21084
ENSMUSG00000039356	Exosc2	exosome component 2 [Source:MGI Symbol;Acc:M	0.21108
ENSMUSG00000028571	Cyp2j13	cytochrome P450, family 2, subfamily j, polypeptide	0.21119
ENSMUSG00000097973	Gm4929	predicted gene 4929 [Source:MGI Symbol;Acc:MGI:	0.21119
ENSMUSG00000085028	Slc2a4rg-ps	Slc2a4 regulator, pseudogene [Source:MGI Symbol;	0.21121
ENSMUSG00000074358	Ccdc61	coiled-coil domain containing 61 [Source:MGI Symb	0.21124
ENSMUSG00000027762	Sucnr1	succinate receptor 1 [Source:MGI Symbol;Acc:MGI:	0.2113

ENSMUSG00000086485	Gm15458	predicted gene 15458 [Source:MGI Symbol;Acc:MG	0.21134
ENSMUSG00000005980	Dnase1	deoxyribonuclease I [Source:MGI Symbol;Acc:MGI:	0.21143
ENSMUSG00000029104	Htt	huntingtin [Source:MGI Symbol;Acc:MGI:96067]	0.21145
ENSMUSG00000000743	Chmp1a	charged multivesicular body protein 1A [Source:M	0.21164
ENSMUSG00000083529	Gm15035	predicted gene 15035 [Source:MGI Symbol;Acc:MG	0.21164
ENSMUSG00000030264	Thumpd3	THUMP domain containing 3 [Source:MGI Symbol;	0.21167
ENSMUSG00000024481	4833403I15Rik	RIKEN cDNA 4833403I15 gene [Source:MGI Symbol	0.21176
ENSMUSG00000084974	Gm15567	predicted gene 15567 [Source:MGI Symbol;Acc:MG	0.21176
ENSMUSG00000031780	Ccl17	chemokine (C-C motif) ligand 17 [Source:MGI Symb	0.21182
ENSMUSG00000030281	Il17rc	interleukin 17 receptor C [Source:MGI Symbol;Acc:	0.2119
ENSMUSG00000050896	Rtn4rl2	reticulon 4 receptor-like 2 [Source:MGI Symbol;Acc	0.21197
ENSMUSG00000063065	Mapk3	mitogen-activated protein kinase 3 [Source:MGI Sy	0.21201
ENSMUSG00000020346	Mgat1	mannoside acetylglucosaminyltransferase 1 [Source	0.21207
ENSMUSG00000030798	Cd37	CD37 antigen [Source:MGI Symbol;Acc:MGI:88330]	0.21207
ENSMUSG00000040158	Tax1bp3	Tax1 (human T cell leukemia virus type I) binding pr	0.21208
ENSMUSG00000021396	Nxnl2	nucleoredoxin-like 2 [Source:MGI Symbol;Acc:MGI:	0.21209
ENSMUSG00000022561	Gpaa1	GPI anchor attachment protein 1 [Source:MGI Sym	0.21212
ENSMUSG00000093661	Eif4e3	eukaryotic translation initiation factor 4E member 3	0.21218
ENSMUSG00000031684	Slc10a7	solute carrier family 10 (sodium/bile acid cotranspo	0.21219
ENSMUSG00000031511	Arhgef7	Rho guanine nucleotide exchange factor (GEF7) [So	0.21224
ENSMUSG00000015943	Bola1	bolA-like 1 (E. coli) [Source:MGI Symbol;Acc:MGI:1	0.21228
ENSMUSG00000050702	Rfpl3s	RIKEN cDNA 4930563M21 gene [Source:MGI Symbo	0.21228
ENSMUSG00000064602	Snora41	small nucleolar RNA, H/ACA box 41 [Source:MGI Sy	0.21231
ENSMUSG00000031996	Aplp2	amyloid beta (A4) precursor-like protein 2 [Source:	0.21237
ENSMUSG00000022538	Lsg1	large subunit GTPase 1 homolog (S. cerevisiae) [Sou	0.21241
ENSMUSG00000029299	Abcg3	ATP-binding cassette, sub-family G (WHITE), memb	0.21244
ENSMUSG00000047880	Cxcr5	chemokine (C-X-C motif) receptor 5 [Source:MGI Sy	0.2125
ENSMUSG00000018372	Cep95	centrosomal protein 95 [Source:MGI Symbol;Acc:M	0.21252
ENSMUSG00000063727	Tnfrsf11b	tumor necrosis factor receptor superfamily, membe	0.21252
ENSMUSG00000084252	Gm12036	predicted gene 12036 [Source:MGI Symbol;Acc:MG	0.21254
ENSMUSG00000042425	Frmpd3	FERM and PDZ domain containing 3 [Source:MGI Sy	0.21267
ENSMUSG00000023968	Crip3	cysteine-rich protein 3 [Source:MGI Symbol;Acc:M	0.21268
ENSMUSG00000092229	Gm5977	predicted gene 5977 [Source:MGI Symbol;Acc:MGI:	0.21274
ENSMUSG00000017144	Rnd3	Rho family GTPase 3 [Source:MGI Symbol;Acc:MGI:	0.21276
ENSMUSG00000024780	Cdc37l1	cell division cycle 37-like 1 [Source:MGI Symbol;Acc	0.2128
ENSMUSG00000025417	Pip4k2c	phosphatidylinositol-5-phosphate 4-kinase, type II,	0.2128
ENSMUSG00000032485	Scap	SREBF chaperone [Source:MGI Symbol;Acc:MGI:21	0.21294
ENSMUSG00000030424	Zfp939	zinc finger protein 939 [Source:MGI Symbol;Acc:M	0.21296
ENSMUSG00000025993	Slc40a1	solute carrier family 40 (iron-regulated transporter	0.21297
ENSMUSG00000049916	2610318N02Rik	RIKEN cDNA 2610318N02 gene [Source:MGI Symbo	0.21298
ENSMUSG00000021496	Pcbd2	pterin 4 alpha carbinolamine dehydratase/dimeriza	0.21301
ENSMUSG00000041939	Mvk	mevalonate kinase [Source:MGI Symbol;Acc:MGI:1	0.21307
ENSMUSG00000021209	Ppp4r4	protein phosphatase 4, regulatory subunit 4 [Sourc	0.21309
ENSMUSG00000034974	Dapk3	death-associated protein kinase 3 [Source:MGI Sym	0.21311
ENSMUSG00000025499	Hras	Harvey rat sarcoma virus oncogene [Source:MGI Sy	0.21321
ENSMUSG00000011751	Sptbn4	spectrin beta, non-erythrocytic 4 [Source:MGI Sym	0.21322
ENSMUSG00000014782	Plekhg4	pleckstrin homology domain containing, family G (w	0.21326

ENSMUSG00000030731	Syt3	synaptotagmin III [Source:MGI Symbol;Acc:MGI:99	0.21327
ENSMUSG00000069520	Tmem19	transmembrane protein 19 [Source:MGI Symbol;Ac	0.21339
ENSMUSG00000034194	R3hcc1	R3H domain and coiled-coil containing 1 [Source:M	0.21342
ENSMUSG00000091636	A330050F15Rik	RIKEN cDNA A330050F15 gene [Source:MGI Symbo	0.21354
ENSMUSG00000022466	Rpap3	RNA polymerase II associated protein 3 [Source:MG	0.21355
ENSMUSG00000090017	Gm16213	predicted gene 16213 [Source:MGI Symbol;Acc:MG	0.21355
ENSMUSG00000020870	Cdc34-ps	cell division cycle 34 homolog, pseudogene (S. cere	0.21382
ENSMUSG00000038894	Irs2	insulin receptor substrate 2 [Source:MGI Symbol;A	0.21392
ENSMUSG00000047013	Fbxo41	F-box protein 41 [Source:MGI Symbol;Acc:MGI:126	0.21393
ENSMUSG00000028975	Pex14	peroxisomal biogenesis factor 14 [Source:MGI Sym	0.21404
ENSMUSG00000014599	Csf1	colony stimulating factor 1 (macrophage) [Source:N	0.21405
ENSMUSG00000074235	Gm10649	predicted gene 10649 [Source:MGI Symbol;Acc:MG	0.21439
ENSMUSG00000016087	Fli1	Friend leukemia integration 1 [Source:MGI Symbol;	0.21451
ENSMUSG00000020493	Prr11	proline rich 11 [Source:MGI Symbol;Acc:MGI:24444	0.21471
ENSMUSG00000026956	Uap1l1	UDP-N-acteylglucosamine pyrophosphorylase 1-like	0.21488
ENSMUSG00000013483	Card14	caspase recruitment domain family, member 14 [So	0.21493
ENSMUSG00000049494	Gm12669	predicted gene 12669 [Source:MGI Symbol;Acc:MG	0.21498
ENSMUSG00000022394	L3mbtl2	l(3)mbt-like 2 (Drosophila) [Source:MGI Symbol;Acc	0.215
ENSMUSG00000030410	Dmwd	dystrophia myotonica-containing WD repeat motif	0.2151
ENSMUSG00000021901	Bap1	Brca1 associated protein 1 [Source:MGI Symbol;Acc	0.21512
ENSMUSG00000038872	Zfhx3	zinc finger homeobox 3 [Source:MGI Symbol;Acc:M	0.21525
ENSMUSG00000048924	Ccdc125	coiled-coil domain containing 125 [Source:MGI Sym	0.21525
ENSMUSG00000029560	Snx8	sorting nexin 8 [Source:MGI Symbol;Acc:MGI:24438	0.21543
ENSMUSG00000024197	Plin3	perilipin 3 [Source:MGI Symbol;Acc:MGI:1914155]	0.21555
ENSMUSG00000086154	Gm16196	predicted gene 16196 [Source:MGI Symbol;Acc:MG	0.21555
ENSMUSG00000048215	A630023P12Rik	RIKEN cDNA A630023P12 gene [Source:MGI Symbo	0.21568
ENSMUSG00000026969	Fam166a	family with sequence similarity 166, member A [Sou	0.21576
ENSMUSG00000086187	Gm12860	predicted gene 12860 [Source:MGI Symbol;Acc:MG	0.2158
ENSMUSG00000024870	Rab1b	RAB1B, member RAS oncogene family [Source:MGI	0.21581
ENSMUSG00000036872	Abcc12	ATP-binding cassette, sub-family C (CFTR/MRP), me	0.21584
ENSMUSG00000022474	Pmm1	phosphomannomutase 1 [Source:MGI Symbol;Acc:	0.21586
ENSMUSG00000085744	Gm12259	predicted gene 12259 [Source:MGI Symbol;Acc:MG	0.21586
ENSMUSG00000087925	Gm23232	predicted gene, 23232 [Source:MGI Symbol;Acc:M	0.21587
ENSMUSG00000064355	mt-Tk	mitochondrially encoded tRNA lysine [Source:MGI S	0.21588
ENSMUSG00000024829	Mrpl21	mitochondrial ribosomal protein L21 [Source:MGI S	0.2159
ENSMUSG00000093779	Gm20648	predicted gene 20648 [Source:MGI Symbol;Acc:MG	0.21598
ENSMUSG00000034758	Tle6	transducin-like enhancer of split 6, homolog of Dro	0.216
ENSMUSG00000001127	Araf	v-raf murine sarcoma 3611 viral oncogene homolog	0.21607
ENSMUSG00000030739	Myh14	myosin, heavy polypeptide 14 [Source:MGI Symbol	0.21611
ENSMUSG00000028565	Nfia	nuclear factor I/A [Source:MGI Symbol;Acc:MGI:10	0.21619
ENSMUSG00000039419	Cntnap2	contactin associated protein-like 2 [Source:MGI Sy	0.21631
ENSMUSG00000025481	Urah	urate (5-hydroxyiso-) hydrolase [Source:MGI Symbo	0.21633
ENSMUSG00000084826	AI847159	expressed sequence AI847159 [Source:MGI Symbol	0.21634
ENSMUSG00000026228	Htr2b	5-hydroxytryptamine (serotonin) receptor 2B [Sour	0.21638
ENSMUSG00000026388	3110009E18Rik	RIKEN cDNA 3110009E18 gene [Source:MGI Symbo	0.21643
ENSMUSG00000005951	Shpk	sedoheptulokinase [Source:MGI Symbol;Acc:MGI:1	0.21648
ENSMUSG00000043419	A030009H04Rik	RIKEN cDNA A030009H04 gene [Source:MGI Symbo	0.21654

ENSMUSG00000084970	1700060J05Rik	RIKEN cDNA 1700060J05 gene [Source:MGI Symbol	0.21664
ENSMUSG00000030990	Pgap2	post-GPI attachment to proteins 2 [Source:MGI Syn	0.2167
ENSMUSG00000022711	Pmm2	phosphomannomutase 2 [Source:MGI Symbol;Acc:	0.21672
ENSMUSG00000036620	Mgat4b	mannoside acetylglucosaminyltransferase 4, isoenz	0.21679
ENSMUSG00000087308	Gm16195	predicted gene 16195 [Source:MGI Symbol;Acc:MG	0.21695
ENSMUSG00000025019	Lcor	ligand dependent nuclear receptor corepressor [So	0.21708
ENSMUSG00000079020	Slc45a4	solute carrier family 45, member 4 [Source:MGI Syr	0.21711
ENSMUSG00000004730	Emr1	EGF-like module containing, mucin-like, hormone r	0.21712
ENSMUSG00000073684	2610002J02Rik	RIKEN cDNA 2610002J02 gene [Source:MGI Symbol	0.21723
ENSMUSG00000048485	Zbtb8b	zinc finger and BTB domain containing 8b [Source:N	0.21727
ENSMUSG00000016427	Ndufa1	NADH dehydrogenase (ubiquinone) 1 alpha subcom	0.21728
ENSMUSG00000057110	Cep110	centrosomal protein 110 [Source:MGI Symbol;Acc:I	0.21728
ENSMUSG00000058019	Ces5a	carboxylesterase 5A [Source:MGI Symbol;Acc:MGI:	0.21741
ENSMUSG00000021676	Iqgap2	IQ motif containing GTPase activating protein 2 [So	0.21762
ENSMUSG00000028874	Fgr	Gardner-Rasheed feline sarcoma viral (Fgr) oncoger	0.21765
ENSMUSG00000097662	Gm2093	predicted gene 2093 [Source:MGI Symbol;Acc:MGI:	0.21772
ENSMUSG00000023033	Scn8a	sodium channel, voltage-gated, type VIII, alpha [Sou	0.21774
ENSMUSG00000006777	Krt23	keratin 23 [Source:MGI Symbol;Acc:MGI:2148866]	0.21779
ENSMUSG00000039615	Stub1	STIP1 homology and U-Box containing protein 1 [Sc	0.21789
ENSMUSG00000026634	Angel2	angel homolog 2 (Drosophila) [Source:MGI Symbol;	0.21796
ENSMUSG00000090303	Vmn2r-ps3	vomeronasal 2, receptor, pseudogene 3 [Source:M	0.21798
ENSMUSG00000035342	Lzts2	leucine zipper, putative tumor suppressor 2 [Source	0.21802
ENSMUSG00000042700	Sipa1l1	signal-induced proliferation-associated 1 like 1 [Sou	0.21811
ENSMUSG00000097193	Gm26664	predicted gene, 26664 [Source:MGI Symbol;Acc:M	0.21813
ENSMUSG00000025495	Ptdss2	phosphatidylserine synthase 2 [Source:MGI Symbo	0.21825
ENSMUSG00000025464	Paox	polyamine oxidase (exo-N4-amino) [Source:MGI Sy	0.21826
ENSMUSG00000025156	Gps1	G protein pathway suppressor 1 [Source:MGI Symb	0.21828
ENSMUSG00000004267	Eno2	enolase 2, gamma neuronal [Source:MGI Symbol;A	0.21832
ENSMUSG00000043716	Rpl7	ribosomal protein L7 [Source:MGI Symbol;Acc:MGI	0.21832
ENSMUSG00000034708	Grn	granulin [Source:MGI Symbol;Acc:MGI:95832]	0.21834
ENSMUSG00000026548	Slamf9	SLAM family member 9 [Source:MGI Symbol;Acc:M	0.21838
ENSMUSG00000042608	Stk40	serine/threonine kinase 40 [Source:MGI Symbol;Ac	0.21838
ENSMUSG00000047591	Mafa	v-maf musculoaponeurotic fibrosarcoma oncogene	0.21841
ENSMUSG00000030098	Grip2	glutamate receptor interacting protein 2 [Source:M	0.21842
ENSMUSG00000053603	4930442H23Rik	RIKEN cDNA 4930442H23 gene [Source:MGI Symbo	0.21847
ENSMUSG00000031976	Urb2	URB2 ribosome biogenesis 2 homolog (S. cerevisiae	0.21854
ENSMUSG00000015342	Xk	Kell blood group precursor (McLeod phenotype) ho	0.21855
ENSMUSG00000051502	Ufsp1	UFM1-specific peptidase 1 [Source:MGI Symbol;Acc	0.21858
ENSMUSG00000051107	Gm15440	predicted gene 15440 [Source:MGI Symbol;Acc:MG	0.21863
ENSMUSG00000039275	Foxk2	forkhead box K2 [Source:MGI Symbol;Acc:MGI:191	0.21867
ENSMUSG00000052031	Tagap1	T cell activation GTPase activating protein 1 [Source	0.21868
ENSMUSG00000046500	Fam19a4	family with sequence similarity 19, member A4 [Sou	0.21869
ENSMUSG00000036160	Surf6	surfeit gene 6 [Source:MGI Symbol;Acc:MGI:98447]	0.21873
ENSMUSG00000036832	Lpar3	lysophosphatidic acid receptor 3 [Source:MGI Symb	0.21873
ENSMUSG00000057134	Ado	2-aminoethanethiol (cysteamine) dioxygenase [Sou	0.21877
ENSMUSG00000063849	Ppcdc	phosphopantothenoylcysteine decarboxylase [Sour	0.21878
ENSMUSG00000069601	Ank3	ankyrin 3, epithelial [Source:MGI Symbol;Acc:MGI:8	0.21889

ENSMUSG00000031144	Syp	synaptophysin [Source:MGI Symbol;Acc:MGI:98467	0.21903
ENSMUSG00000041920	Slc16a6	solute carrier family 16 (monocarboxylic acid transp	0.21909
ENSMUSG00000049932	H2afx	H2A histone family, member X [Source:MGI Symbo	0.21917
ENSMUSG00000086987	Gm12484	predicted gene 12484 [Source:MGI Symbol;Acc:MG	0.21919
ENSMUSG00000084859	1700080N15Rik	RIKEN cDNA 1700080N15 gene [Source:MGI Symbo	0.21921
ENSMUSG00000024570	Rbfa	ribosome binding factor A [Source:MGI Symbol;Acc	0.21926
ENSMUSG00000050855	Zfp940	zinc finger protein 940 [Source:MGI Symbol;Acc:M	0.21927
ENSMUSG00000027671	Actl6a	actin-like 6A [Source:MGI Symbol;Acc:MGI:186145	0.21929
ENSMUSG00000035437	Rabgap1	RAB GTPase activating protein 1 [Source:MGI Symb	0.21931
ENSMUSG00000078747	Gm20878	predicted gene, 20878 [Source:MGI Symbol;Acc:M	0.21945
ENSMUSG00000083500	Gm15470	predicted gene 15470 [Source:MGI Symbol;Acc:MG	0.21947
ENSMUSG00000085242	Gm11537	predicted gene 11537 [Source:MGI Symbol;Acc:MG	0.21952
ENSMUSG00000022500	Litaf	LPS-induced TN factor [Source:MGI Symbol;Acc:MG	0.21954
ENSMUSG00000071226	Cecr2	cat eye syndrome chromosome region, candidate 2	0.21954
ENSMUSG00000027661	Slc2a10	solute carrier family 2 (facilitated glucose transport	0.21961
ENSMUSG00000015467	Egfl8	EGF-like domain 8 [Source:MGI Symbol;Acc:MGI:19	0.21962
ENSMUSG00000006019	Dhx34	DEAH (Asp-Glu-Ala-His) box polypeptide 34 [Source	0.21964
ENSMUSG00000082004	Gm13519	predicted gene 13519 [Source:MGI Symbol;Acc:MG	0.21968
ENSMUSG00000024620	Pdgfrb	platelet derived growth factor receptor, beta polyp	0.21969
ENSMUSG00000078494	Gm13201	predicted gene 13201 [Source:MGI Symbol;Acc:MG	0.2197
ENSMUSG00000097316	Gm10516	predicted gene 10516 [Source:MGI Symbol;Acc:MG	0.21971
ENSMUSG00000024173	Tpsab1	tryptase alpha/beta 1 [Source:MGI Symbol;Acc:MG	0.21974
ENSMUSG00000037894	H2afz	H2A histone family, member Z [Source:MGI Symbol	0.21977
ENSMUSG00000036860	Mrpl55	mitochondrial ribosomal protein L55 [Source:MGI S	0.2198
ENSMUSG00000007570	Fance	Fanconi anemia, complementation group E [Source	0.21988
ENSMUSG00000048949	Gm6206	predicted pseudogene 6206 [Source:MGI Symbol;A	0.22001
ENSMUSG00000002908	Kcnn1	potassium intermediate/small conductance calcium	0.22004
ENSMUSG00000045440	Insm2	insulinoma-associated 2 [Source:MGI Symbol;Acc:N	0.22004
ENSMUSG00000050737	Ptges	prostaglandin E synthase [Source:MGI Symbol;Acc:	0.22011
ENSMUSG00000043029	Trpv3	transient receptor potential cation channel, subfam	0.22016
ENSMUSG00000038663	Fsd2	fibronectin type III and SPRY domain containing 2 [S	0.2202
ENSMUSG00000014778	Fhod1	formin homology 2 domain containing 1 [Source:M	0.22021
ENSMUSG00000052291	5330438D12Rik	RIKEN cDNA 5330438D12 gene [Source:MGI Symbo	0.22023
ENSMUSG00000085207	Gm11767	predicted gene 11767 [Source:MGI Symbol;Acc:MG	0.22034
ENSMUSG00000029623	Pdap1	PDGFA associated protein 1 [Source:MGI Symbol;A	0.22038
ENSMUSG00000032299	Commd4	COMM domain containing 4 [Source:MGI Symbol;A	0.22045
ENSMUSG00000077797	Snord19	small nucleolar RNA, C/D box 19 [Source:MGI Symb	0.22049
ENSMUSG00000074365	Crxos1	Crx opposite strand transcript 1 [Source:MGI Symb	0.22052
ENSMUSG00000028064	Sema4a	sema domain, immunoglobulin domain (Ig), transm	0.22054
ENSMUSG00000015087	Rabl6	RAB, member of RAS oncogene family-like 6 [Sourc	0.22055
ENSMUSG00000056144	Trim34a	tripartite motif-containing 34A [Source:MGI Symbo	0.22056
ENSMUSG00000082812	Gm14860	predicted gene 14860 [Source:MGI Symbol;Acc:MG	0.22056
ENSMUSG00000018893	Mb	myoglobin [Source:MGI Symbol;Acc:MGI:96922]	0.22057
ENSMUSG00000021947	Cryl1	crystallin, lambda 1 [Source:MGI Symbol;Acc:MGI:1	0.22057
ENSMUSG00000074516	Gm10709	predicted gene 10709 [Source:MGI Symbol;Acc:MG	0.22063
ENSMUSG00000060212	Pcnxl2	pecanex-like 2 (Drosophila) [Source:MGI Symbol;Ac	0.22069
ENSMUSG00000081423	Gm12882	predicted gene 12882 [Source:MGI Symbol;Acc:MG	0.2207

ENSMUSG00000042675	Ypel3	yippee-like 3 (Drosophila) [Source:MGI Symbol;Acc	0.22077
ENSMUSG00000071253	Slc25a16	solute carrier family 25 (mitochondrial carrier, Grav	0.22095
ENSMUSG00000017405	Nek8	NIMA (never in mitosis gene a)-related expressed k	0.22104
ENSMUSG00000001143	Lman2l	lectin, mannose-binding 2-like [Source:MGI Symbol	0.22105
ENSMUSG00000003549	Ercc1	excision repair cross-complementing rodent repair	0.22107
ENSMUSG00000032171	Pin1	protein (peptidyl-prolyl cis/trans isomerase) NIMA-	0.22107
ENSMUSG00000031657	Heatr3	HEAT repeat containing 3 [Source:MGI Symbol;Acc:	0.22121
ENSMUSG00000020890	Gucy2e	guanylate cyclase 2e [Source:MGI Symbol;Acc:MGI:	0.22124
ENSMUSG00000027316	Gfra4	glial cell line derived neurotrophic factor family rec	0.22125
ENSMUSG00000022000	Zc3h13	zinc finger CCCH type containing 13 [Source:MGI Sy	0.22141
ENSMUSG00000009418	Nav1	neuron navigator 1 [Source:MGI Symbol;Acc:MGI:2	0.22143
ENSMUSG00000052914	Cyp2j6	cytochrome P450, family 2, subfamily j, polypeptide	0.22143
ENSMUSG00000037905	Bri3bp	Bri3 binding protein [Source:MGI Symbol;Acc:MGI:	0.22152
ENSMUSG00000057098	Ebf1	early B cell factor 1 [Source:MGI Symbol;Acc:MGI:9	0.22152
ENSMUSG00000003072	Atp5d	ATP synthase, H+ transporting, mitochondrial F1 co	0.22154
ENSMUSG00000020387	Phf15	PHD finger protein 15 [Source:MGI Symbol;Acc:MG	0.22154
ENSMUSG00000034462	Pkd2	polycystic kidney disease 2 [Source:MGI Symbol;Ac	0.22155
ENSMUSG00000040570	Rundc3b	RUN domain containing 3B [Source:MGI Symbol;Ac	0.22156
ENSMUSG00000097908	4933404O12Rik	RIKEN cDNA 4933404O12 gene [Source:MGI Symbo	0.22156
ENSMUSG00000089764	Gm16580	predicted gene 16580 [Source:MGI Symbol;Acc:MG	0.22157
ENSMUSG00000092300	Cdk3-ps	cyclin-dependent kinase 3, pseudogene [Source:MG	0.22165
ENSMUSG00000003352	Cacnb3	calcium channel, voltage-dependent, beta 3 subuni	0.22173
ENSMUSG00000078599	Skint8	selection and upkeep of intraepithelial T cells 8 [So	0.22176
ENSMUSG00000039737	Prkrip1	Prkr interacting protein 1 (IL11 inducible) [Source:M	0.22179
ENSMUSG00000028367	Txn1	thioredoxin 1 [Source:MGI Symbol;Acc:MGI:98874]	0.22188
ENSMUSG00000042564	Fam227a	family with sequence similarity 227, member A [Sou	0.2219
ENSMUSG00000030865	Chp2	calcineurin-like EF hand protein 2 [Source:MGI Sym	0.22192
ENSMUSG00000074227	Spint2	serine protease inhibitor, Kunitz type 2 [Source:MG	0.22193
ENSMUSG00000025869	Nop16	NOP16 nucleolar protein [Source:MGI Symbol;Acc:I	0.22197
ENSMUSG00000042389	Tsen2	tRNA splicing endonuclease 2 homolog (S. cerevisia	0.22202
ENSMUSG00000046062	Ppp1r15b	protein phosphatase 1, regulatory (inhibitor) subur	0.22203
ENSMUSG00000093803	Ppp2r3d	protein phosphatase 2 (formerly 2A), regulatory su	0.22205
ENSMUSG00000081884	Gm11821	predicted gene 11821 [Source:MGI Symbol;Acc:MG	0.22226
ENSMUSG00000006288	Ttc5	tetratricopeptide repeat domain 5 [Source:MGI Syr	0.22232
ENSMUSG00000060639	Hist1h4i	histone cluster 1, H4i [Source:MGI Symbol;Acc:MGI	0.22232
ENSMUSG00000033773	Rpap2	RNA polymerase II associated protein 2 [Source:MG	0.22247
ENSMUSG00000034781	Gna11	guanine nucleotide binding protein, alpha 11 [Sourc	0.22278
ENSMUSG00000035770	Dync1li2	dynein, cytoplasmic 1 light intermediate chain 2 [So	0.2229
ENSMUSG00000022973	Synj1	synaptojanin 1 [Source:MGI Symbol;Acc:MGI:13549	0.22291
ENSMUSG00000048481	Mypop	Myb-related transcription factor, partner of profilin	0.22301
ENSMUSG00000002831	Plin4	perilipin 4 [Source:MGI Symbol;Acc:MGI:1929709]	0.22303
ENSMUSG00000040016	Ptger3	prostaglandin E receptor 3 (subtype EP3) [Source:M	0.22318
ENSMUSG00000027247	Arhgap1	Rho GTPase activating protein 1 [Source:MGI Symb	0.22328
ENSMUSG00000021928	Ebpl	emopamil binding protein-like [Source:MGI Symbol	0.22329
ENSMUSG00000025272	Tro	trophinin [Source:MGI Symbol;Acc:MGI:1928994]	0.22332
ENSMUSG00000051615	Rap2a	RAS related protein 2a [Source:MGI Symbol;Acc:MG	0.22338
ENSMUSG00000043648	Pld6	phospholipase D family, member 6 [Source:MGI Sy	0.22355

ENSMUSG00000020537	Drg2	developmentally regulated GTP binding protein 2 [S	0.22372
ENSMUSG00000041238	Rbbp8	retinoblastoma binding protein 8 [Source:MGI Sym	0.22374
ENSMUSG00000048967	Yjefn3	YjeF N-terminal domain containing 3 [Source:MGI S	0.22377
ENSMUSG00000021193	Pitrm1	pitrilysin metallepetidase 1 [Source:MGI Symbol;Ac	0.22392
ENSMUSG00000042116	Vwa1	von Willebrand factor A domain containing 1 [Sourc	0.22395
ENSMUSG00000037887	Dusp8	dual specificity phosphatase 8 [Source:MGI Symbol	0.22396
ENSMUSG00000058486	Wdr91	WD repeat domain 91 [Source:MGI Symbol;Acc:MG	0.22398
ENSMUSG00000018340	Anxa6	annexin A6 [Source:MGI Symbol;Acc:MGI:88255]	0.22406
ENSMUSG00000065746	Gm25005	predicted gene, 25005 [Source:MGI Symbol;Acc:MG	0.22406
ENSMUSG00000024560	Cxxc1	CXXC finger 1 (PHD domain) [Source:MGI Symbol;A	0.22409
ENSMUSG00000044375	BC027072	cDNA sequence BC027072 [Source:MGI Symbol;Acc	0.2242
ENSMUSG00000033707	Lrrc24	leucine rich repeat containing 24 [Source:MGI Syml	0.2243
ENSMUSG00000034968	Lbx2	ladybird homeobox homolog 2 (Drosophila) [Source	0.22431
ENSMUSG00000057359	Gm17494	predicted gene, 17494 [Source:MGI Symbol;Acc:MG	0.22436
ENSMUSG00000035632	Cnot3	CCR4-NOT transcription complex, subunit 3 [Source	0.22439
ENSMUSG00000082922	Gm6939	predicted gene 6939 [Source:MGI Symbol;Acc:MGI:	0.22441
ENSMUSG00000090370	Gm21989	predicted gene 21989 [Source:MGI Symbol;Acc:MG	0.22446
ENSMUSG00000097430	Gm10544	predicted gene 10544 [Source:MGI Symbol;Acc:MG	0.22449
ENSMUSG00000037151	Lrrc20	leucine rich repeat containing 20 [Source:MGI Syml	0.22451
ENSMUSG00000031893	Tsnaxip1	translin-associated factor X (Tsnax) interacting prot	0.22456
ENSMUSG00000064647	Gm23301	predicted gene, 23301 [Source:MGI Symbol;Acc:MG	0.22457
ENSMUSG00000081340	Gm12275	predicted gene 12275 [Source:MGI Symbol;Acc:MG	0.22462
ENSMUSG00000014529	4930511M11Rik	RIKEN cDNA 4930511M11 gene [Source:MGI Symbo	0.22468
ENSMUSG00000052456	Asna1	arsA arsenite transporter, ATP-binding, homolog 1	0.22469
ENSMUSG00000085322	Gm14261	predicted gene 14261 [Source:MGI Symbol;Acc:MG	0.2247
ENSMUSG00000018405	Mrm1	mitochondrial rRNA methyltransferase 1 homolog (	0.22471
ENSMUSG00000022847	Thpo	thrombopoietin [Source:MGI Symbol;Acc:MGI:1018	0.22474
ENSMUSG00000085022	Gm5860	predicted gene 5860 [Source:MGI Symbol;Acc:MGI:	0.22474
ENSMUSG00000066456	Hmgn3	high mobility group nucleosomal binding domain 3	0.22481
ENSMUSG00000040441	Slc26a10	solute carrier family 26, member 10 [Source:MGI Sy	0.22506
ENSMUSG00000028540	Dph2	DPH2 homolog (S. cerevisiae) [Source:MGI Symbol;	0.22507
ENSMUSG00000046186	Cd109	CD109 antigen [Source:MGI Symbol;Acc:MGI:24452	0.2251
ENSMUSG00000021917	Spcs1	signal peptidase complex subunit 1 homolog (S. cer	0.22512
ENSMUSG00000031948	Kars	lysyl-tRNA synthetase [Source:MGI Symbol;Acc:MG	0.22518
ENSMUSG00000074805	Gm14047	predicted gene 14047 [Source:MGI Symbol;Acc:MG	0.22519
ENSMUSG00000082357	Gm13542	predicted gene 13542 [Source:MGI Symbol;Acc:MG	0.22519
ENSMUSG00000027255	Arfgap2	ADP-ribosylation factor GTPase activating protein 2	0.22528
ENSMUSG00000073007	Fam46d	family with sequence similarity 46, member D [Sou	0.22528
ENSMUSG00000067341	H2-Eb2	histocompatibility 2, class II antigen E beta2 [Source	0.22531
ENSMUSG00000090955	Gm17097	predicted gene 17097 [Source:MGI Symbol;Acc:MG	0.22532
ENSMUSG00000075581	Gm16409	predicted gene 16409 [Source:MGI Symbol;Acc:MG	0.22533
ENSMUSG00000090522	Gm5919	predicted gene 5919 [Source:MGI Symbol;Acc:MGI:	0.22535
ENSMUSG00000070690	5830473C10Rik	RIKEN cDNA 5830473C10 gene [Source:MGI Symbo	0.22539
ENSMUSG00000085468	Gm15343	predicted gene 15343 [Source:MGI Symbol;Acc:MG	0.22539
ENSMUSG00000088371	Gm25180	predicted gene, 25180 [Source:MGI Symbol;Acc:MG	0.2254
ENSMUSG00000021273	Fdft1	farnesyl diphosphate farnesyl transferase 1 [Source	0.22541
ENSMUSG00000065463	Snord45c	small nucleolar RNA, C/D box 45C [Source:MGI Sym	0.22542

ENSMUSG00000000326	Comt	catechol-O-methyltransferase [Source:MGI Symbol	0.22543
ENSMUSG00000064796	Terc	telomerase RNA component [Source:MGI Symbol;A	0.22545
ENSMUSG00000021791	Dydc2	DPY30 domain containing 2 [Source:MGI Symbol;Ac	0.22549
ENSMUSG00000047613	A430005L14Rik	RIKEN cDNA A430005L14 gene [Source:MGI Symbo	0.22553
ENSMUSG00000065409	Mir30e	microRNA 30e [Source:MGI Symbol;Acc:MGI:36193	0.22556
ENSMUSG00000031876	Cmtm1	CKLF-like MARVEL transmembrane domain contain	0.22559
ENSMUSG00000065870	Rnu3a	U3A small nuclear RNA [Source:MGI Symbol;Acc:M	0.22561
ENSMUSG00000084674	Gm26027	predicted gene, 26027 [Source:MGI Symbol;Acc:MG	0.22561
ENSMUSG00000074677	Sirpb1c	signal-regulatory protein beta 1C [Source:MGI Sym	0.22562
ENSMUSG00000082872	Gm15773	predicted gene 15773 [Source:MGI Symbol;Acc:MG	0.22562
ENSMUSG00000052146	Rps10	ribosomal protein S10 [Source:MGI Symbol;Acc:MG	0.22563
ENSMUSG00000071052	Rpl7a-ps5	ribosomal protein L7A, pseudogene 5 [Source:MGI	0.22563
ENSMUSG00000079391	Gm2974	predicted gene 2974 [Source:MGI Symbol;Acc:MGI:	0.22567
ENSMUSG00000051124	Gimap9	GTPase, IMAP family member 9 [Source:MGI Symbo	0.2257
ENSMUSG00000077925	Mir453	microRNA 453 [Source:MGI Symbol;Acc:MGI:37185	0.2257
ENSMUSG00000078604	Gm10961	predicted gene 10961 [Source:MGI Symbol;Acc:MG	0.2257
ENSMUSG00000094628	Gm3252	predicted gene 3252 [Source:MGI Symbol;Acc:MGI:	0.22571
ENSMUSG00000080922	Gm2214	predicted gene 2214 [Source:MGI Symbol;Acc:MGI:	0.22572
ENSMUSG00000089585	Gm23338	predicted gene, 23338 [Source:MGI Symbol;Acc:MG	0.22572
ENSMUSG00000073094	Smim9	small integral membrane protein 9 [Source:MGI Sy	0.22573
ENSMUSG00000053054	Adh6a	alcohol dehydrogenase 6A (class V) [Source:MGI Sy	0.22574
ENSMUSG00000096385	Gm11168	predicted gene 11168 [Source:MGI Symbol;Acc:MG	0.22575
ENSMUSG00000088359	Gm24009	predicted gene, 24009 [Source:MGI Symbol;Acc:MG	0.22577
ENSMUSG00000080748	Gm15661	predicted gene 15661 [Source:MGI Symbol;Acc:MG	0.22579
ENSMUSG00000025364	Pa2g4	proliferation-associated 2G4 [Source:MGI Symbol;A	0.22583
ENSMUSG00000077611	Gm23946	predicted gene, 23946 [Source:MGI Symbol;Acc:MG	0.22587
ENSMUSG00000029236	Nmu	neuromedin U [Source:MGI Symbol;Acc:MGI:18604	0.22588
ENSMUSG00000046006	Gapt	Grb2-binding adaptor, transmembrane [Source:MG	0.22588
ENSMUSG00000046242	1600029I14Rik	RIKEN cDNA 1600029I14 gene [Source:MGI Symbol	0.22588
ENSMUSG00000054116	E130116L18Rik	RIKEN cDNA E130116L18 gene [Source:MGI Symbo	0.22588
ENSMUSG00000036570	Fxyd1	FXYD domain-containing ion transport regulator 1 [	0.22589
ENSMUSG00000000278	Scpep1	serine carboxypeptidase 1 [Source:MGI Symbol;Acc	0.2259
ENSMUSG00000009093	Gstt4	glutathione S-transferase, theta 4 [Source:MGI Sym	0.22591
ENSMUSG00000092719	Gm22859	predicted gene, 22859 [Source:MGI Symbol;Acc:MG	0.22592
ENSMUSG00000091419	Gm17450	predicted gene, 17450 [Source:MGI Symbol;Acc:MG	0.22594
ENSMUSG00000087240	Gm15976	predicted gene 15976 [Source:MGI Symbol;Acc:MG	0.22595
ENSMUSG00000092819	Gm23639	predicted gene, 23639 [Source:MGI Symbol;Acc:MG	0.22605
ENSMUSG00000095868	Gm23136	predicted gene, 23136 [Source:MGI Symbol;Acc:MG	0.22607
ENSMUSG00000047777	Phf13	PHD finger protein 13 [Source:MGI Symbol;Acc:MG	0.22611
ENSMUSG00000038335	Tsr1	TSR1 20S rRNA accumulation [Source:MGI Symbol;A	0.22616
ENSMUSG00000021823	Vcl	vinculin [Source:MGI Symbol;Acc:MGI:98927]	0.22619
ENSMUSG00000028882	Ppp1r8	protein phosphatase 1, regulatory (inhibitor) subun	0.2262
ENSMUSG00000034919	Ttc22	tetratricopeptide repeat domain 22 [Source:MGI Sy	0.22629
ENSMUSG00000087192	4930412L05Rik	RIKEN cDNA 4930412L05 gene [Source:MGI Symbo	0.22629
ENSMUSG00000037461	Ints7	integrator complex subunit 7 [Source:MGI Symbol;A	0.22637
ENSMUSG00000026939	Tmem141	transmembrane protein 141 [Source:MGI Symbol;A	0.22639
ENSMUSG00000021203	Otub2	OTU domain, ubiquitin aldehyde binding 2 [Source:	0.2264

ENSMUSG00000063446	E130309F12Rik	RIKEN cDNA E130309F12 gene [Source:MGI Symbo	0.22643
ENSMUSG00000041057	Wdr43	WD repeat domain 43 [Source:MGI Symbol;Acc:MG	0.22648
ENSMUSG00000040025	Ythdf2	YTH domain family 2 [Source:MGI Symbol;Acc:MGI:	0.22657
ENSMUSG00000031865	Dctn1	dynactin 1 [Source:MGI Symbol;Acc:MGI:107745]	0.22658
ENSMUSG00000031827	Cotl1	coactosin-like 1 (Dictyostelium) [Source:MGI Symbo	0.2267
ENSMUSG00000003813	Rad23a	RAD23a homolog (S. cerevisiae) [Source:MGI Symb	0.22681
ENSMUSG00000066235	Pomgnt2	protein O-linked mannose beta 1,4-N-acetylglucosa	0.22681
ENSMUSG00000097502	4930528D03Rik	RIKEN cDNA 4930528D03 gene [Source:MGI Symbo	0.22683
ENSMUSG00000039703	Nploc4	nuclear protein localization 4 homolog (S. cerevisia	0.22688
ENSMUSG00000025647	Shisa5	shisa homolog 5 (Xenopus laevis) [Source:MGI Sym	0.22689
ENSMUSG00000020544	Cox11	cytochrome c oxidase assembly protein 11 [Source:	0.22693
ENSMUSG00000029911	Ssbp1	single-stranded DNA binding protein 1 [Source:MG	0.22694
ENSMUSG00000040225	Prrc2c	proline-rich coiled-coil 2C [Source:MGI Symbol;Acc:	0.22695
ENSMUSG00000052560	Cpne8	copine VIII [Source:MGI Symbol;Acc:MGI:1914121]	0.22695
ENSMUSG00000059900	Tmem40	transmembrane protein 40 [Source:MGI Symbol;Ac	0.227
ENSMUSG00000097820	E530011L22Rik	RIKEN cDNA E530011L22 gene [Source:MGI Symbo	0.22704
ENSMUSG00000031875	Cmtm3	CKLF-like MARVEL transmembrane domain contain	0.22726
ENSMUSG00000001632	Brpf1	bromodomain and PHD finger containing, 1 [Source	0.22742
ENSMUSG00000022304	Dpys	dihydropyrimidinase [Source:MGI Symbol;Acc:MGI:	0.22746
ENSMUSG00000024973	Hrasls5	HRAS-like suppressor family, member 5 [Source:MG	0.22746
ENSMUSG00000056978	Hamp2	hepcidin antimicrobial peptide 2 [Source:MGI Symb	0.22754
ENSMUSG00000083316	Gm15185	predicted gene 15185 [Source:MGI Symbol;Acc:MG	0.22759
ENSMUSG00000070844	Vmn2r42	vomeronasal 2, receptor 42 [Source:MGI Symbol;A	0.22765
ENSMUSG00000086885	Gm13846	predicted gene 13846 [Source:MGI Symbol;Acc:MG	0.22766
ENSMUSG00000094732	1500015L24Rik	RIKEN cDNA 1500015L24 gene [Source:MGI Symbo	0.22767
ENSMUSG00000097173	Gm26829	predicted gene, 26829 [Source:MGI Symbol;Acc:MG	0.22767
ENSMUSG00000098194	Gm7286	predicted gene 7286 [Source:MGI Symbol;Acc:MGI:	0.22767
ENSMUSG00000087333	Gm13652	predicted gene 13652 [Source:MGI Symbol;Acc:MG	0.22768
ENSMUSG00000066647	Gm5113	predicted gene 5113 [Source:MGI Symbol;Acc:MGI:	0.22772
ENSMUSG00000033256	Shf	Src homology 2 domain containing F [Source:MGI S	0.22773
ENSMUSG00000034424	Gcsh	glycine cleavage system protein H (aminomethyl ca	0.22779
ENSMUSG00000078919	Dpm1	dolichol-phosphate (beta-D) mannosyltransferase 1	0.22783
ENSMUSG00000082079	Gm14490	predicted gene 14490 [Source:MGI Symbol;Acc:MG	0.22786
ENSMUSG00000020864	Ankrd40	ankyrin repeat domain 40 [Source:MGI Symbol;Acc	0.22804
ENSMUSG00000038760	Trhr	thyrotropin releasing hormone receptor [Source:M	0.22804
ENSMUSG00000039853	Trim14	tripartite motif-containing 14 [Source:MGI Symbol;	0.22816
ENSMUSG00000084597	Gm22343	predicted gene, 22343 [Source:MGI Symbol;Acc:MG	0.22816
ENSMUSG00000030411	Nova2	neuro-oncological ventral antigen 2 [Source:MGI Sy	0.22817
ENSMUSG00000029815	Malsu1	mitochondrial assembly of ribosomal large subunit	0.2283
ENSMUSG00000028582	Cc2d1b	coiled-coil and C2 domain containing 1B [Source:M	0.22839
ENSMUSG00000033392	Clasp2	CLIP associating protein 2 [Source:MGI Symbol;Acc:	0.22839
ENSMUSG00000055013	Agap1	ArfGAP with GTPase domain, ankyrin repeat and PH	0.22839
ENSMUSG00000029166	Mapre3	microtubule-associated protein, RP/EB family, mem	0.22841
ENSMUSG00000031604	Sc4mol	sterol-C4-methyl oxidase-like [Source:MGI Symbol;	0.22846
ENSMUSG00000024251	Thada	thyroid adenoma associated [Source:MGI Symbol;A	0.22887
ENSMUSG00000087211	1500016L03Rik	RIKEN cDNA 1500016L03 gene [Source:MGI Symbo	0.22891
ENSMUSG00000036529	Sbf1	SET binding factor 1 [Source:MGI Symbol;Acc:MGI:	0.22899

ENSMUSG00000049672	Zbtb14	zinc finger and BTB domain containing 14 [Source:M	0.229
ENSMUSG00000021952	Xpo4	exportin 4 [Source:MGI Symbol;Acc:MGI:1888526]	0.22903
ENSMUSG00000000392	Fap	fibroblast activation protein [Source:MGI Symbol;A	0.22909
ENSMUSG00000027939	Nup210l	nucleoporin 210-like [Source:MGI Symbol;Acc:MGI:	0.22909
ENSMUSG00000003848	Nob1	NIN1/RPN12 binding protein 1 homolog (S. cerevisi	0.2291
ENSMUSG00000041729	Coro2b	coronin, actin binding protein, 2B [Source:MGI Sym	0.2291
ENSMUSG00000033931	Rbm34	RNA binding motif protein 34 [Source:MGI Symbol;	0.22912
ENSMUSG00000094686	Ccl21a	chemokine (C-C motif) ligand 21A (serine) [Source:I	0.22917
ENSMUSG00000082187	Gm13670	predicted gene 13670 [Source:MGI Symbol;Acc:MG	0.22927
ENSMUSG00000073147	5031425E22Rik	RIKEN cDNA 5031425E22 gene [Source:MGI Symbo	0.22935
ENSMUSG00000029319	Coq2	coenzyme Q2 homolog, prenyltransferase (yeast) [S	0.22937
ENSMUSG00000090706	Gm17233	predicted gene 17233 [Source:MGI Symbol;Acc:MG	0.22943
ENSMUSG00000001844	Zdhhc4	zinc finger, DHHC domain containing 4 [Source:MG	0.22945
ENSMUSG00000083623	Gm7224	predicted gene 7224 [Source:MGI Symbol;Acc:MGI:	0.22947
ENSMUSG00000022094	Slc39a14	solute carrier family 39 (zinc transporter), member	0.22962
ENSMUSG00000044149	Nkrf	NF-kappaB repressing factor [Source:MGI Symbol;A	0.22962
ENSMUSG00000092444	Gm18733	predicted gene, 18733 [Source:MGI Symbol;Acc:M(	0.22963
ENSMUSG00000029095	Ablim2	actin-binding LIM protein 2 [Source:MGI Symbol;Ac	0.22969
ENSMUSG00000060402	Chst8	carbohydrate (N-acetylgalactosamine 4-0) sulfotrar	0.22969
ENSMUSG00000040699	Limd2	LIM domain containing 2 [Source:MGI Symbol;Acc:I	0.22974
ENSMUSG00000001943	Vsig2	V-set and immunoglobulin domain containing 2 [So	0.22983
ENSMUSG00000033510	Otud7a	OTU domain containing 7A [Source:MGI Symbol;Ac	0.22984
ENSMUSG00000056201	Cfl1	cofilin 1, non-muscle [Source:MGI Symbol;Acc:MGI	0.22987
ENSMUSG00000020829	Slc46a1	solute carrier family 46, member 1 [Source:MGI Syr	0.2299
ENSMUSG00000097864	Gm26856	predicted gene, 26856 [Source:MGI Symbol;Acc:M(	0.22998
ENSMUSG00000097809	Gm26554	predicted gene, 26554 [Source:MGI Symbol;Acc:M(	0.23001
ENSMUSG00000051480	A130050O07Rik	RIKEN cDNA A130050O07 gene [Source:MGI Symbo	0.23011
ENSMUSG00000030245	Golt1b	golgi transport 1 homolog B (S. cerevisiae) [Source:	0.23014
ENSMUSG00000026770	Il2ra	interleukin 2 receptor, alpha chain [Source:MGI Syr	0.23036
ENSMUSG00000031788	Kifc3	kinesin family member C3 [Source:MGI Symbol;Acc	0.2304
ENSMUSG00000074576	Mocs3	molybdenum cofactor synthesis 3 [Source:MGI Sym	0.23047
ENSMUSG00000066571	4931406P16Rik	RIKEN cDNA 4931406P16 gene [Source:MGI Symbo	0.23054
ENSMUSG00000072960	BC065397	cDNA sequence BC065397 [Source:MGI Symbol;Acc	0.23055
ENSMUSG00000083907	Plk-ps1	polo-like kinase, pseudogene 1 [Source:MGI Symbo	0.23068
ENSMUSG00000028284	Map3k7	mitogen-activated protein kinase kinase kinase 7 [S	0.23079
ENSMUSG00000045876	Pcdhb8	protocadherin beta 8 [Source:MGI Symbol;Acc:MGI	0.2308
ENSMUSG00000055725	Paqr3	progestin and adipoQ receptor family member III [S	0.23085
ENSMUSG00000074030	Exoc8	exocyst complex component 8 [Source:MGI Symbo	0.23107
ENSMUSG00000092390	Gm20541	predicted gene 20541 [Source:MGI Symbol;Acc:MG	0.23117
ENSMUSG00000028003	Lrat	lecithin-retinol acyltransferase (phosphatidylcholin	0.2312
ENSMUSG00000061633	1700029P11Rik	RIKEN cDNA 1700029P11 gene [Source:MGI Symbo	0.23121
ENSMUSG00000026626	Ppp2r5a	protein phosphatase 2, regulatory subunit B (B56),	0.23122
ENSMUSG00000000134	Tfe3	transcription factor E3 [Source:MGI Symbol;Acc:M(	0.23124
ENSMUSG00000068262	Gm5879	predicted gene 5879 [Source:MGI Symbol;Acc:MGI:	0.23128
ENSMUSG00000022773	Ypel1	yippee-like 1 (Drosophila) [Source:MGI Symbol;Acc	0.23135
ENSMUSG00000021451	Sema4d	sema domain, immunoglobulin domain (Ig), transm	0.23138
ENSMUSG00000036251	Trpm8	transient receptor potential cation channel, subfam	0.23139

ENSMUSG00000029038	Ssu72	Ssu72 RNA polymerase II CTD phosphatase homolo	0.2314
ENSMUSG00000094969	Gm22420	predicted gene, 22420 [Source:MGI Symbol;Acc:M	0.23142
ENSMUSG00000065999	Gm13154	predicted gene 13154 [Source:MGI Symbol;Acc:MG	0.23143
ENSMUSG00000025961	4933402D24Rik	RIKEN cDNA 4933402D24 gene [Source:MGI Symbo	0.23148
ENSMUSG00000029570	Lfng	LFNG O-fucosylpeptide 3-beta-N-acetylglucosamin	0.23156
ENSMUSG00000030048	Gkn3	gastrokine 3 [Source:MGI Symbol;Acc:MGI:191613	0.23157
ENSMUSG00000020869	Lrrc59	leucine rich repeat containing 59 [Source:MGI Sym	0.23162
ENSMUSG00000027655	Dhx35	DEAH (Asp-Glu-Ala-His) box polypeptide 35 [Source	0.23164
ENSMUSG00000067028	Cntnap5b	contactin associated protein-like 5B [Source:MGI S	0.23166
ENSMUSG00000087630	Gm11513	predicted gene 11513 [Source:MGI Symbol;Acc:MG	0.2317
ENSMUSG00000032763	Ilvbl	ilvB (bacterial acetolactate synthase)-like [Source:	0.23175
ENSMUSG00000086123	Gm16060	predicted pseudogene 16060 [Source:MGI Symbol;	0.23177
ENSMUSG00000086316	2210013O21Rik	RIKEN cDNA 2210013O21 gene [Source:MGI Symbo	0.23181
ENSMUSG00000082208	Gm15379	predicted gene 15379 [Source:MGI Symbol;Acc:MG	0.2319
ENSMUSG00000018446	C1qbp	complement component 1, q subcomponent bindir	0.23192
ENSMUSG00000070605	Gm13251	predicted gene 13251 [Source:MGI Symbol;Acc:MG	0.23218
ENSMUSG00000018899	Irf1	interferon regulatory factor 1 [Source:MGI Symbol;	0.23219
ENSMUSG00000097411	B430218F22Rik	RIKEN cDNA B430218F22 gene [Source:MGI Symbo	0.23223
ENSMUSG00000052533	Nup188	nucleoporin 188 [Source:MGI Symbol;Acc:MGI:244	0.23225
ENSMUSG00000090199	Gm16029	predicted gene 16029 [Source:MGI Symbol;Acc:MG	0.23227
ENSMUSG00000070867	Trabd2b	TraB domain containing 2B [Source:MGI Symbol;Ac	0.23236
ENSMUSG00000084968	Gm12743	predicted gene 12743 [Source:MGI Symbol;Acc:MG	0.23236
ENSMUSG00000085637	Gm15980	predicted gene 15980 [Source:MGI Symbol;Acc:MG	0.2324
ENSMUSG00000045008	9030612E09Rik	RIKEN cDNA 9030612E09 gene [Source:MGI Symbo	0.23252
ENSMUSG00000039656	Rxrb	retinoid X receptor beta [Source:MGI Symbol;Acc:M	0.23255
ENSMUSG00000030761	Myo7a	myosin VIIA [Source:MGI Symbol;Acc:MGI:104510]	0.23258
ENSMUSG00000044477	Zfand3	zinc finger, AN1-type domain 3 [Source:MGI Symbo	0.23261
ENSMUSG00000095375	Gm21955	predicted gene, 21955 [Source:MGI Symbol;Acc:M	0.23281
ENSMUSG00000031378	Abcd1	ATP-binding cassette, sub-family D (ALD), member	0.23283
ENSMUSG00000037446	Tulp1	tubby like protein 1 [Source:MGI Symbol;Acc:MGI:1	0.23289
ENSMUSG00000060733	Ipmk	inositol polyphosphate multikinase [Source:MGI Sy	0.23296
ENSMUSG00000026185	Igfbp5	insulin-like growth factor binding protein 5 [Source	0.23307
ENSMUSG00000097602	4930519P11Rik	RIKEN cDNA 4930519P11 gene [Source:MGI Symbo	0.23316
ENSMUSG00000030814	Bcl7c	B cell CLL/lymphoma 7C [Source:MGI Symbol;Acc:M	0.2332
ENSMUSG00000031790	Mmp15	matrix metallopeptidase 15 [Source:MGI Symbol;A	0.23324
ENSMUSG00000078348	Sf3b5	splicing factor 3b, subunit 5 [Source:MGI Symbol;A	0.23329
ENSMUSG00000040488	Ltbp4	latent transforming growth factor beta binding pro	0.2334
ENSMUSG00000055943	Emc7	ER membrane protein complex subunit 7 [Source:M	0.23352
ENSMUSG00000086554	9530034E10Rik	RIKEN cDNA 9530034E10 gene [Source:MGI Symbo	0.23353
ENSMUSG00000027858	Tspan2	tetraspanin 2 [Source:MGI Symbol;Acc:MGI:191799	0.2336
ENSMUSG00000074822	Gm10766	predicted gene 10766 [Source:MGI Symbol;Acc:MG	0.23361
ENSMUSG00000070462	Mesdc1	mesoderm development candidate 1 [Source:MGI S	0.23372
ENSMUSG00000026411	Tmem9	transmembrane protein 9 [Source:MGI Symbol;Acc	0.23374
ENSMUSG00000034486	Gbx2	gastrulation brain homeobox 2 [Source:MGI Symbo	0.23376
ENSMUSG00000024669	Cd5	CD5 antigen [Source:MGI Symbol;Acc:MGI:88340]	0.23385
ENSMUSG00000017764	Zswim1	zinc finger SWIM-type containing 1 [Source:MGI Sy	0.23396
ENSMUSG00000079235	Ccdc13	coiled-coil domain containing 13 [Source:MGI Symb	0.23399

ENSMUSG00000030674	Qprt	quinolinate phosphoribosyltransferase [Source:MG	0.23402
ENSMUSG00000037735	2810032G03Rik	RIKEN cDNA 2810032G03 gene [Source:MGI Symbo	0.23404
ENSMUSG00000039771	Polr2j	polymerase (RNA) II (DNA directed) polypeptide J [S	0.23405
ENSMUSG00000051786	Tubgcp6	tubulin, gamma complex associated protein 6 [Sour	0.23411
ENSMUSG00000024295	Cyp4f41-ps	cytochrome P450, family 4, subfamily f, polypeptide	0.23413
ENSMUSG00000021012	Zc3h14	zinc finger CCCH type containing 14 [Source:MGI Sy	0.23414
ENSMUSG00000049928	Glp2r	glucagon-like peptide 2 receptor [Source:MGI Symb	0.23415
ENSMUSG00000074259	Gramd2	GRAM domain containing 2 [Source:MGI Symbol;Ac	0.23416
ENSMUSG00000012429	Mplkip	M-phase specific PLK1 intereacting protein [Source	0.23438
ENSMUSG00000038080	Kdm1b	lysine (K)-specific demethylase 1B [Source:MGI Sym	0.23441
ENSMUSG00000040562	Gstm2	glutathione S-transferase, mu 2 [Source:MGI Symbo	0.23454
ENSMUSG00000029276	Glmn	glomulin, FKBP associated protein [Source:MGI Sym	0.23463
ENSMUSG00000041205	Map6d1	MAP6 domain containing 1 [Source:MGI Symbol;Ac	0.23465
ENSMUSG00000029111	Nelfa	negative elongation factor complex member A, Wh	0.23467
ENSMUSG00000056418	BC043934	cDNA sequence BC043934 [Source:MGI Symbol;Acc	0.23467
ENSMUSG00000024965	Fermt3	fermitin family homolog 3 (Drosophila) [Source:MG	0.23468
ENSMUSG00000071653	1810009A15Rik	RIKEN cDNA 1810009A15 gene [Source:MGI Symbo	0.23468
ENSMUSG00000047746	Fbxo40	F-box protein 40 [Source:MGI Symbol;Acc:MGI:244	0.23474
ENSMUSG00000015533	Itga2	integrin alpha 2 [Source:MGI Symbol;Acc:MGI:9660	0.23475
ENSMUSG00000036019	Tmtc2	transmembrane and tetratricopeptide repeat conta	0.23493
ENSMUSG00000038902	Pogz	pogo transposable element with ZNF domain [Sour	0.23493
ENSMUSG00000026571	Dcaf6	DDB1 and CUL4 associated factor 6 [Source:MGI Sy	0.23509
ENSMUSG00000021704	Mtx3	metaxin 3 [Source:MGI Symbol;Acc:MGI:2686040]	0.23524
ENSMUSG00000029036	Atad3a	ATPase family, AAA domain containing 3A [Source:I	0.2353
ENSMUSG00000085189	Gm11963	predicted gene 11963 [Source:MGI Symbol;Acc:MG	0.23536
ENSMUSG00000081099	Gm5270	predicted gene 5270 [Source:MGI Symbol;Acc:MGI:	0.23537
ENSMUSG00000005364	Il5ra	interleukin 5 receptor, alpha [Source:MGI Symbol;A	0.23542
ENSMUSG00000034681	Rnps1	ribonucleic acid binding protein S1 [Source:MGI Syr	0.23556
ENSMUSG00000097354	2310001H17Rik	RIKEN cDNA 2310001H17 gene [Source:MGI Symbo	0.23561
ENSMUSG00000028447	Dctn3	dynactin 3 [Source:MGI Symbol;Acc:MGI:1859251]	0.23564
ENSMUSG00000038173	Enpp6	ectonucleotide pyrophosphatase/phosphodiesteras	0.23566
ENSMUSG00000087173	Gm11337	predicted gene 11337 [Source:MGI Symbol;Acc:MG	0.2357
ENSMUSG00000028772	Zcchc17	zinc finger, CCHC domain containing 17 [Source:MG	0.23574
ENSMUSG00000029093	Sorcs2	sortilin-related VPS10 domain containing receptor 2	0.23575
ENSMUSG00000016933	Plcg1	phospholipase C, gamma 1 [Source:MGI Symbol;Ac	0.23589
ENSMUSG00000020432	Tcn2	transcobalamin 2 [Source:MGI Symbol;Acc:MGI:985	0.23594
ENSMUSG00000029062	Cdk11b	cyclin-dependent kinase 11B [Source:MGI Symbol;A	0.23602
ENSMUSG00000024672	Ms4a7	membrane-spanning 4-domains, subfamily A, mem	0.23604
ENSMUSG00000000751	Rpa1	replication protein A1 [Source:MGI Symbol;Acc:MG	0.23609
ENSMUSG00000024944	Arl2	ADP-ribosylation factor-like 2 [Source:MGI Symbol;	0.2361
ENSMUSG00000063808	Gpatch1	G patch domain containing 1 [Source:MGI Symbol;A	0.23618
ENSMUSG00000027639	Samhd1	SAM domain and HD domain, 1 [Source:MGI Symbo	0.2363
ENSMUSG00000001496	Nkx2-1	NK2 homeobox 1 [Source:MGI Symbol;Acc:MGI:108	0.23634
ENSMUSG00000020003	Pex7	peroxisomal biogenesis factor 7 [Source:MGI Symb	0.23635
ENSMUSG00000055108	Phxr2	per-hexamer repeat gene 2 [Source:MGI Symbol;Ac	0.23635
ENSMUSG00000026012	Cd28	CD28 antigen [Source:MGI Symbol;Acc:MGI:88327]	0.23643
ENSMUSG00000090080	Gm15872	predicted gene 15872 [Source:MGI Symbol;Acc:MG	0.23659

ENSMUSG00000019761	Krt10	keratin 10 [Source:MGI Symbol;Acc:MGI:96685]	0.2366
ENSMUSG00000040231	Syngr4	synaptogyrin 4 [Source:MGI Symbol;Acc:MGI:1928	0.23663
ENSMUSG00000032220	Myo1e	myosin IE [Source:MGI Symbol;Acc:MGI:106621]	0.23666
ENSMUSG00000030655	Smg1	SMG1 homolog, phosphatidylinositol 3-kinase-relat	0.23667
ENSMUSG00000084128	Esrp2	epithelial splicing regulatory protein 2 [Source:MGI	0.2367
ENSMUSG00000048796	Cyb561d1	cytochrome b-561 domain containing 1 [Source:MG	0.23672
ENSMUSG00000086330	1700007J10Rik	RIKEN cDNA 1700007J10 gene [Source:MGI Symbol	0.23673
ENSMUSG00000036009	Mettl25	methyltransferase like 25 [Source:MGI Symbol;Acc:	0.2368
ENSMUSG00000051335	Gfod1	glucose-fructose oxidoreductase domain containing	0.2368
ENSMUSG00000074398	Gm15441	predicted gene 15441 [Source:MGI Symbol;Acc:MG	0.2368
ENSMUSG00000049339	Fam134a	family with sequence similarity 134, member A [So	0.23685
ENSMUSG00000090041	Gm16027	predicted gene 16027 [Source:MGI Symbol;Acc:MG	0.23686
ENSMUSG00000064437	Snord49b	small nucleolar RNA, C/D box 49B [Source:MGI Sym	0.23688
ENSMUSG00000027329	Spef1	sperm flagellar 1 [Source:MGI Symbol;Acc:MGI:351	0.23689
ENSMUSG00000027809	Etfdh	electron transferring flavoprotein, dehydrogenase	0.2369
ENSMUSG00000064612	Gm26457	predicted gene, 26457 [Source:MGI Symbol;Acc:MG	0.23695
ENSMUSG00000025134	Alyref	Aly/REF export factor [Source:MGI Symbol;Acc:MG	0.23702
ENSMUSG00000020850	Prpf8	pre-mRNA processing factor 8 [Source:MGI Symbol	0.23703
ENSMUSG00000011096	Akt1s1	AKT1 substrate 1 (proline-rich) [Source:MGI Symbo	0.23708
ENSMUSG00000051695	Pcbp1	poly(rC) binding protein 1 [Source:MGI Symbol;Acc	0.23709
ENSMUSG00000021737	Psmd6	proteasome (prosome, macropain) 26S subunit, no	0.23713
ENSMUSG00000031373	Car5b	carbonic anhydrase 5b, mitochondrial [Source:MGI	0.23715
ENSMUSG00000050211	Pla2g4e	phospholipase A2, group IVE [Source:MGI Symbol;A	0.23715
ENSMUSG00000014980	Tsen15	tRNA splicing endonuclease 15 homolog (S. cerevisi	0.23736
ENSMUSG00000087400	Gm15270	predicted gene 15270 [Source:MGI Symbol;Acc:MG	0.23742
ENSMUSG00000036246	Gmip	Gem-interacting protein [Source:MGI Symbol;Acc:M	0.23757
ENSMUSG00000029596	Sdsl	serine dehydratase-like [Source:MGI Symbol;Acc:M	0.23762
ENSMUSG00000025787	Tgm4	transglutaminase 4 (prostate) [Source:MGI Symbol;	0.23764
ENSMUSG00000003527	Dgcr14	DiGeorge syndrome critical region gene 14 [Source	0.2377
ENSMUSG00000094439	Gm21969	predicted gene 21969 [Source:MGI Symbol;Acc:MG	0.2377
ENSMUSG00000058818	Pirb	paired Ig-like receptor B [Source:MGI Symbol;Acc:M	0.23773
ENSMUSG00000082374	Gm12741	predicted gene 12741 [Source:MGI Symbol;Acc:MG	0.23774
ENSMUSG00000060534	Dcc	deleted in colorectal carcinoma [Source:MGI Symbo	0.23784
ENSMUSG00000030595	Nfkbib	nuclear factor of kappa light polypeptide gene enha	0.23808
ENSMUSG00000086320	Gm12840	predicted gene 12840 [Source:MGI Symbol;Acc:MG	0.23814
ENSMUSG00000027400	Pdyn	prodynorphin [Source:MGI Symbol;Acc:MGI:97535]	0.23815
ENSMUSG00000042734	Ttc9	tetratricopeptide repeat domain 9 [Source:MGI Syn	0.23822
ENSMUSG00000043102	Qrfp	pyroglutamylated RFamide peptide [Source:MGI Sy	0.23825
ENSMUSG00000045932	Ifit2	interferon-induced protein with tetratricopeptide r	0.23827
ENSMUSG00000025155	Dus1l	dihydrouridine synthase 1-like (S. cerevisiae) [Sourc	0.23834
ENSMUSG00000035984	Nme5	NME/NM23 family member 5 [Source:MGI Symbol;	0.23843
ENSMUSG00000085635	Gm14565	predicted gene 14565 [Source:MGI Symbol;Acc:MG	0.23848
ENSMUSG00000057160	Gm16372	predicted pseudogene 16372 [Source:MGI Symbol;	0.23853
ENSMUSG00000032425	Zfp949	zinc finger protein 949 [Source:MGI Symbol;Acc:MG	0.23857
ENSMUSG00000092164	Rergl	RERG/RAS-like [Source:MGI Symbol;Acc:MGI:36429	0.23857
ENSMUSG00000058897	Col25a1	collagen, type XXV, alpha 1 [Source:MGI Symbol;Ac	0.23858
ENSMUSG00000089794	Gm16337	predicted gene 16337 [Source:MGI Symbol;Acc:MG	0.23859

ENSMUSG00000030243	Recql	RecQ protein-like [Source:MGI Symbol;Acc:MGI:10	0.23861
ENSMUSG00000079554	Aox2	aldehyde oxidase 2 [Source:MGI Symbol;Acc:MGI:3	0.23862
ENSMUSG00000083734	Gm16209	predicted gene 16209 [Source:MGI Symbol;Acc:MG	0.23864
ENSMUSG00000079243	Xirp1	xin actin-binding repeat containing 1 [Source:MGI S	0.23866
ENSMUSG00000073774	Kncn	kinocilin [Source:MGI Symbol;Acc:MGI:3614952]	0.23867
ENSMUSG00000081308	Gm14480	predicted gene 14480 [Source:MGI Symbol;Acc:MG	0.23867
ENSMUSG00000086935	Gm15956	predicted gene 15956 [Source:MGI Symbol;Acc:MG	0.23869
ENSMUSG00000024228	Nudt12	nudix (nucleoside diphosphate linked moiety X)-typ	0.23872
ENSMUSG00000034450	Gulo	gulonolactone (L-) oxidase [Source:MGI Symbol;Acc	0.23872
ENSMUSG00000026799	Med27	mediator complex subunit 27 [Source:MGI Symbol;	0.23873
ENSMUSG00000036052	Dnajb5	DnaJ (Hsp40) homolog, subfamily B, member 5 [Sou	0.23875
ENSMUSG00000034685	Fam171a2	family with sequence similarity 171, member A2 [S	0.23878
ENSMUSG00000052234	Epx	eosinophil peroxidase [Source:MGI Symbol;Acc:MG	0.23881
ENSMUSG00000055835	Zfp1	zinc finger protein 1 [Source:MGI Symbol;Acc:MGI:	0.23881
ENSMUSG00000037797	Adh4	alcohol dehydrogenase 4 (class II), pi polypeptide [	0.23883
ENSMUSG00000038014	Fam120a	family with sequence similarity 120, member A [Sou	0.23892
ENSMUSG00000028959	Fastk	Fas-activated serine/threonine kinase [Source:MGI	0.23896
ENSMUSG00000022584	Ly6c2	lymphocyte antigen 6 complex, locus C2 [Source:M	0.23902
ENSMUSG00000047878	A4galt	alpha 1,4-galactosyltransferase [Source:MGI Symbo	0.23912
ENSMUSG00000020892	Aloxe3	arachidonate lipoxygenase 3 [Source:MGI Symbol;A	0.23917
ENSMUSG00000051225	Fam83a	family with sequence similarity 83, member A [Sou	0.23917
ENSMUSG00000056370	Sftpb	surfactant associated protein B [Source:MGI Symbo	0.23918
ENSMUSG00000038077	Kcna6	potassium voltage-gated channel, shaker-related, s	0.23923
ENSMUSG00000012296	Tjap1	tight junction associated protein 1 [Source:MGI Syn	0.23926
ENSMUSG00000086695	Gm15247	predicted gene 15247 [Source:MGI Symbol;Acc:MG	0.23926
ENSMUSG00000025371	Chmp6	charged multivesicular body protein 6 [Source:MGI	0.23938
ENSMUSG00000028397	Kdm4c	lysine (K)-specific demethylase 4C [Source:MGI Sym	0.23946
ENSMUSG00000036211	Hist1h1t	histone cluster 1, H1t [Source:MGI Symbol;Acc:MG	0.23946
ENSMUSG00000009863	Sdhb	succinate dehydrogenase complex, subunit B, iron	0.23951
ENSMUSG00000022454	Nell2	NEL-like 2 [Source:MGI Symbol;Acc:MGI:1858510]	0.23951
ENSMUSG00000097368	Gm10390	predicted gene 10390 [Source:MGI Symbol;Acc:MG	0.23951
ENSMUSG00000085443	Gm15416	predicted gene 15416 [Source:MGI Symbol;Acc:MG	0.23956
ENSMUSG00000059772	Slx1b	SLX1 structure-specific endonuclease subunit homo	0.2397
ENSMUSG00000020591	Ntsr2	neurotensin receptor 2 [Source:MGI Symbol;Acc:M	0.23983
ENSMUSG00000071078	Nr2c2ap	nuclear receptor 2C2-associated protein [Source:M	0.23983
ENSMUSG00000016526	Dyrk3	dual-specificity tyrosine-(Y)-phosphorylation regula	0.23984
ENSMUSG00000090369	4933411K16Rik	RIKEN cDNA 4933411K16 gene [Source:MGI Symbo	0.23987
ENSMUSG00000000148	Brat1	BRCA1-associated ATM activator 1 [Source:MGI Syn	0.23996
ENSMUSG00000066704	Cyp2b19	cytochrome P450, family 2, subfamily b, polypeptid	0.23996
ENSMUSG00000038002	Cramp1l	Crm, cramped-like (Drosophila) [Source:MGI Symbo	0.24
ENSMUSG00000046324	Ermp1	endoplasmic reticulum metallopeptidase 1 [Source	0.24
ENSMUSG00000092187	Gm20457	predicted gene 20457 [Source:MGI Symbol;Acc:MG	0.24008
ENSMUSG00000022176	Rem2	rad and gem related GTP binding protein 2 [Source	0.24011
ENSMUSG00000031431	Tsc22d3	TSC22 domain family, member 3 [Source:MGI Symb	0.24015
ENSMUSG00000097466	D430036J16Rik	RIKEN cDNA D430036J16 gene [Source:MGI Symbo	0.24019
ENSMUSG00000049659	Aftph	aftiphilin [Source:MGI Symbol;Acc:MGI:1923012]	0.24033
ENSMUSG00000079364	Gm3558	predicted gene 3558 [Source:MGI Symbol;Acc:MGI:	0.24034

ENSMUSG00000042810	Krba1	KRAB-A domain containing 1 [Source:MGI Symbol;A	0.24038
ENSMUSG00000032320	Rcn2	reticulocalbin 2 [Source:MGI Symbol;Acc:MGI:1349	0.24046
ENSMUSG00000019726	Lyst	lysosomal trafficking regulator [Source:MGI Symbo	0.24052
ENSMUSG00000048911	Rnf24	ring finger protein 24 [Source:MGI Symbol;Acc:MGI	0.24055
ENSMUSG00000040852	Plekhh2	pleckstrin homology domain containing, family H (v	0.2406
ENSMUSG00000025195	Dnmbp	dynamin binding protein [Source:MGI Symbol;Acc:M	0.24063
ENSMUSG00000054079	Utp18	UTP18, small subunit (SSU) processome componen	0.24066
ENSMUSG00000029722	Agfg2	ArfGAP with FG repeats 2 [Source:MGI Symbol;Acc:	0.24067
ENSMUSG00000001131	Timp1	tissue inhibitor of metalloproteinase 1 [Source:MGI	0.24074
ENSMUSG00000093617	4933438K21Rik	RIKEN cDNA 4933438K21 gene [Source:MGI Symbo	0.24076
ENSMUSG00000085788	4930470G03Rik	RIKEN cDNA 4930470G03 gene [Source:MGI Symbo	0.24077
ENSMUSG00000052616	Ccdc79	coiled-coil domain containing 79 [Source:MGI Symb	0.24082
ENSMUSG00000047992	Fam69c	family with sequence similarity 69, member C [Sour	0.24085
ENSMUSG00000082107	Gm16216	predicted gene 16216 [Source:MGI Symbol;Acc:MG	0.241
ENSMUSG00000091705	H2-Q2	histocompatibility 2, Q region locus 2 [Source:MGI S	0.24109
ENSMUSG00000024410	3110002H16Rik	RIKEN cDNA 3110002H16 gene [Source:MGI Symbo	0.24119
ENSMUSG00000028889	Yrdc	yrdC domain containing (E.coli) [Source:MGI Symbo	0.24122
ENSMUSG00000040842	Szrd1	SUZ RNA binding domain containing 1 [Source:MGI	0.24123
ENSMUSG00000025271	Pfkfb1	6-phosphofructo-2-kinase/fructose-2,6-biphosphat	0.24128
ENSMUSG00000004630	Pcp2	Purkinje cell protein 2 (L7) [Source:MGI Symbol;Acc	0.24132
ENSMUSG00000080775	Gm6368	predicted gene 6368 [Source:MGI Symbol;Acc:MGI:	0.24135
ENSMUSG00000035478	Mbd3	methyl-CpG binding domain protein 3 [Source:MGI	0.24136
ENSMUSG00000024330	Col11a2	collagen, type XI, alpha 2 [Source:MGI Symbol;Acc:I	0.24138
ENSMUSG00000029714	Gigyf1	GRB10 interacting GYF protein 1 [Source:MGI Symb	0.2414
ENSMUSG00000050288	Fzd2	frizzled homolog 2 (Drosophila) [Source:MGI Symbo	0.24149
ENSMUSG00000030847	Bag3	BCL2-associated athanogene 3 [Source:MGI Symbo	0.24152
ENSMUSG00000044352	Sowaha	sosondowah ankyrin repeat domain family membe	0.2417
ENSMUSG00000044164	Rnf182	ring finger protein 182 [Source:MGI Symbol;Acc:MG	0.24176
ENSMUSG00000047733	BC048609	cDNA sequence BC048609 [Source:MGI Symbol;Acc	0.24176
ENSMUSG00000074991	Gabrr3	gamma-aminobutyric acid (GABA) receptor, rho 3 [S	0.24179
ENSMUSG00000029286	Enam	enamelin [Source:MGI Symbol;Acc:MGI:1333772]	0.24201
ENSMUSG00000050912	Tmem123	transmembrane protein 123 [Source:MGI Symbol;A	0.24209
ENSMUSG00000097624	Gm5091	predicted gene 5091 [Source:MGI Symbol;Acc:MGI:	0.2421
ENSMUSG00000090408	Gm17402	predicted gene, 17402 [Source:MGI Symbol;Acc:MG	0.24211
ENSMUSG00000056091	St3gal5	ST3 beta-galactoside alpha-2,3-sialyltransferase 5 [S	0.24214
ENSMUSG00000064696	Gm24148	predicted gene, 24148 [Source:MGI Symbol;Acc:MG	0.24214
ENSMUSG00000087121	Gm7847	predicted gene 7847 [Source:MGI Symbol;Acc:MGI:	0.24215
ENSMUSG00000029455	Aldh2	aldehyde dehydrogenase 2, mitochondrial [Source:	0.24216
ENSMUSG00000074830	Zfp640	zinc finger protein 640 [Source:MGI Symbol;Acc:MG	0.24219
ENSMUSG00000073018	Gm1070	predicted gene 1070 [Source:MGI Symbol;Acc:MGI:	0.24222
ENSMUSG00000020057	Dram1	DNA-damage regulated autophagy modulator 1 [So	0.24223
ENSMUSG00000027966	Col11a1	collagen, type XI, alpha 1 [Source:MGI Symbol;Acc:	0.24223
ENSMUSG00000086390	1810019D21Rik	RIKEN cDNA 1810019D21 gene [Source:MGI Symbo	0.24224
ENSMUSG00000013646	Sh3bp5l	SH3 binding domain protein 5 like [Source:MGI Sym	0.24232
ENSMUSG00000028413	B4galt1	UDP-Gal:betaGlcNAc beta 1,4- galactosyltransferas	0.24235
ENSMUSG00000085267	Gm14235	predicted gene 14235 [Source:MGI Symbol;Acc:MG	0.24236
ENSMUSG00000042364	Snx18	sorting nexin 18 [Source:MGI Symbol;Acc:MGI:2137	0.2424

ENSMUSG00000057123	Gja5	gap junction protein, alpha 5 [Source:MGI Symbol;A	0.24247
ENSMUSG00000019997	Ctgf	connective tissue growth factor [Source:MGI Symb	0.2425
ENSMUSG00000049160	4930469G21Rik	RIKEN cDNA 4930469G21 gene [Source:MGI Symbo	0.24253
ENSMUSG00000051969	Tlr11	toll-like receptor 11 [Source:MGI Symbol;Acc:MGI:3	0.24253
ENSMUSG00000073781	Gm6471	predicted gene 6471 [Source:MGI Symbol;Acc:MGI:	0.24253
ENSMUSG00000065440	Mirlet7g	microRNA let7g [Source:MGI Symbol;Acc:MGI:2676	0.24254
ENSMUSG00000093774	Vmn1r-ps151	vomeronasal 1 receptor, pseudogene 151 [Source:I	0.24255
ENSMUSG00000026253	Chrng	cholinergic receptor, nicotinic, gamma polypeptide	0.24256
ENSMUSG00000030945	Acsm2	acyl-CoA synthetase medium-chain family member	0.24256
ENSMUSG00000011154	1700026D08Rik	RIKEN cDNA 1700026D08 gene [Source:MGI Symbo	0.2426
ENSMUSG00000092278	Gm8752	predicted pseudogene 8752 [Source:MGI Symbol;A	0.2426
ENSMUSG00000086846	Gm13642	predicted gene 13642 [Source:MGI Symbol;Acc:MG	0.24262
ENSMUSG00000024891	Slc29a2	solute carrier family 29 (nucleoside transporters), n	0.24263
ENSMUSG00000085631	9630028H03Rik	RIKEN cDNA 9630028H03 gene [Source:MGI Symbo	0.24264
ENSMUSG00000053465	Hs6st3	heparan sulfate 6-O-sulfotransferase 3 [Source:MG	0.24267
ENSMUSG00000086967	4930566F21Rik	RIKEN cDNA 4930566F21 gene [Source:MGI Symbo	0.24269
ENSMUSG00000030685	Kctd13	potassium channel tetramerisation domain contain	0.24272
ENSMUSG00000023045	Soat2	sterol O-acyltransferase 2 [Source:MGI Symbol;Acc	0.24277
ENSMUSG00000083027	Gm13140	predicted gene 13140 [Source:MGI Symbol;Acc:MG	0.24284
ENSMUSG00000029811	Aoc1	amine oxidase, copper-containing 1 [Source:MGI Sy	0.24285
ENSMUSG00000034911	Ushbp1	Usher syndrome 1C binding protein 1 [Source:MGI	0.24288
ENSMUSG00000050217	Lgsn	lengsin, lens protein with glutamine synthetase dor	0.24292
ENSMUSG00000024142	Mlst8	MTOR associated protein, LST8 homolog (S. cerevis	0.24299
ENSMUSG00000028396	2310002L09Rik	RIKEN cDNA 2310002L09 gene [Source:MGI Symbo	0.243
ENSMUSG00000028995	Fam126a	family with sequence similarity 126, member A [Sou	0.24307
ENSMUSG00000031441	Atp11a	ATPase, class VI, type 11A [Source:MGI Symbol;Acc	0.24319
ENSMUSG00000073293	Nudt10	nudix (nucleoside diphosphate linked moiety X)-typ	0.24321
ENSMUSG00000050087	Cby3	chibby homolog 3 (Drosophila) [Source:MGI Symbo	0.24322
ENSMUSG00000083149	Gm11380	predicted gene 11380 [Source:MGI Symbol;Acc:MG	0.24323
ENSMUSG00000040432	Ltb4r2	leukotriene B4 receptor 2 [Source:MGI Symbol;Acc	0.24331
ENSMUSG00000001518	Itfg2	integrin alpha FG-GAP repeat containing 2 [Source:	0.24343
ENSMUSG00000035047	Kri1	KRI1 homolog (S. cerevisiae) [Source:MGI Symbol;A	0.24347
ENSMUSG00000089125	Gm26403	predicted gene, 26403 [Source:MGI Symbol;Acc:M	0.24348
ENSMUSG00000057370	Rpl21-ps12	ribosomal protein L21, pseudogene 12 [Source:MG	0.24349
ENSMUSG00000048782	Insc	inscuteable homolog (Drosophila) [Source:MGI Sym	0.24359
ENSMUSG00000007038	Neu1	neuraminidase 1 [Source:MGI Symbol;Acc:MGI:973	0.24361
ENSMUSG00000074448	Gm5615	predicted gene 5615 [Source:MGI Symbol;Acc:MGI:	0.24375
ENSMUSG00000050201	Otop2	otopetrin 2 [Source:MGI Symbol;Acc:MGI:2388365	0.24378
ENSMUSG00000031816	Mthfsd	methenyltetrahydrofolate synthetase domain conta	0.2439
ENSMUSG00000039474	Wfs1	Wolfram syndrome 1 homolog (human) [Source:M	0.24397
ENSMUSG00000037089	Slc35b2	solute carrier family 35, member B2 [Source:MGI S	0.2441
ENSMUSG00000025877	Hk3	hexokinase 3 [Source:MGI Symbol;Acc:MGI:267096	0.24412
ENSMUSG00000092483	Gm20421	predicted gene 20421 [Source:MGI Symbol;Acc:MG	0.24415
ENSMUSG00000004207	Psap	prosaposin [Source:MGI Symbol;Acc:MGI:97783]	0.24417
ENSMUSG00000038271	Iffo1	intermediate filament family orphan 1 [Source:MGI	0.24417
ENSMUSG00000052033	Pfdn4	prefoldin 4 [Source:MGI Symbol;Acc:MGI:1923512]	0.24419
ENSMUSG00000079733	Tmem181b-ps	transmembrane protein 181B, pseudogene [Source	0.24419

ENSMUSG00000020340	Cyfip2	cytoplasmic FMR1 interacting protein 2 [Source:MG	0.24423
ENSMUSG00000036553	Sh3tc1	SH3 domain and tetratricopeptide repeats 1 [Sourc	0.24434
ENSMUSG00000030612	Mrpl46	mitochondrial ribosomal protein L46 [Source:MGI S	0.24448
ENSMUSG00000031166	Wdr13	WD repeat domain 13 [Source:MGI Symbol;Acc:MG	0.2445
ENSMUSG00000053522	Lgals7	lectin, galactose binding, soluble 7 [Source:MGI Syr	0.24454
ENSMUSG00000024906	Mus81	MUS81 endonuclease homolog (yeast) [Source:MG	0.24464
ENSMUSG00000097310	A930038B10Rik	RIKEN cDNA A930038B10 gene [Source:MGI Symbo	0.24465
ENSMUSG00000018819	Lsp1	lymphocyte specific 1 [Source:MGI Symbol;Acc:MG	0.24479
ENSMUSG00000062157	Ifnlr1	interferon lambda receptor 1 [Source:MGI Symbol;	0.24495
ENSMUSG00000030822	Prr14	proline rich 14 [Source:MGI Symbol;Acc:MGI:23845	0.24501
ENSMUSG00000001020	S100a4	S100 calcium binding protein A4 [Source:MGI Symb	0.24505
ENSMUSG00000020652	Cenpo	centromere protein O [Source:MGI Symbol;Acc:MG	0.24507
ENSMUSG00000035275	Raver2	ribonucleoprotein, PTB-binding 2 [Source:MGI Sym	0.24523
ENSMUSG00000036109	Mbnl3	muscleblind-like 3 (Drosophila) [Source:MGI Symbo	0.24525
ENSMUSG00000089139	Mir1964	microRNA 1964 [Source:MGI Symbol;Acc:MGI:3837	0.24529
ENSMUSG00000027419	Pcsk2	proprotein convertase subtilisin/kexin type 2 [Sour	0.24533
ENSMUSG00000085526	Gm16083	predicted gene 16083 [Source:MGI Symbol;Acc:MG	0.24537
ENSMUSG00000009555	Cdk9	cyclin-dependent kinase 9 (CDC2-related kinase) [S	0.2454
ENSMUSG00000064267	Hvcn1	hydrogen voltage-gated channel 1 [Source:MGI Syn	0.2454
ENSMUSG00000018102	Hist1h2bc	histone cluster 1, H2bc [Source:MGI Symbol;Acc:M	0.24542
ENSMUSG00000093483	AA465934	expressed sequence AA465934 [Source:MGI Symbo	0.24544
ENSMUSG00000026709	Dars2	aspartyl-tRNA synthetase 2 (mitochondrial) [Source	0.24548
ENSMUSG00000030019	Fbxl14	F-box and leucine-rich repeat protein 14 [Source:M	0.24549
ENSMUSG00000020457	Drg1	developmentally regulated GTP binding protein 1 [S	0.24555
ENSMUSG00000026342	Slc35f5	solute carrier family 35, member F5 [Source:MGI Sy	0.24557
ENSMUSG00000087424	5730405O15Rik	RIKEN cDNA 5730405O15 gene [Source:MGI Symbo	0.24559
ENSMUSG00000026494	Kif26b	kinesin family member 26B [Source:MGI Symbol;Ac	0.24561
ENSMUSG00000031492	Chrnb3	cholinergic receptor, nicotinic, beta polypeptide 3 [	0.24564
ENSMUSG00000097507	Gm26538	predicted gene, 26538 [Source:MGI Symbol;Acc:MG	0.24565
ENSMUSG00000045532	C1ql1	complement component 1, q subcomponent-like 1	0.24593
ENSMUSG00000030313	Dennd5b	DENN/MADD domain containing 5B [Source:MGI Sy	0.24594
ENSMUSG00000022386	Trmu	tRNA 5-methylaminomethyl-2-thiouridylate methyl	0.24604
ENSMUSG00000089807	Gm16298	predicted gene 16298 [Source:MGI Symbol;Acc:MG	0.2461
ENSMUSG00000055795	Gm5160	predicted gene 5160 [Source:MGI Symbol;Acc:MGI:	0.24612
ENSMUSG00000085464	Gm16208	predicted gene 16208 [Source:MGI Symbol;Acc:MG	0.24613
ENSMUSG00000048495	Tyw5	tRNA-yW synthesizing protein 5 [Source:MGI Symb	0.24614
ENSMUSG00000000792	Slc5a5	solute carrier family 5 (sodium iodide symporter), n	0.24626
ENSMUSG00000054302	Eapp	E2F-associated phosphoprotein [Source:MGI Symb	0.2464
ENSMUSG00000032667	Pon2	paraoxonase 2 [Source:MGI Symbol;Acc:MGI:10668	0.24641
ENSMUSG00000045107	Saysd1	SAYSVFN motif domain containing 1 [Source:MGI Sy	0.24642
ENSMUSG00000089588	Gm23548	predicted gene, 23548 [Source:MGI Symbol;Acc:MG	0.24644
ENSMUSG00000070806	Zmynd12	zinc finger, MYND domain containing 12 [Source:M	0.24652
ENSMUSG00000025512	Chid1	chitinase domain containing 1 [Source:MGI Symbol	0.24656
ENSMUSG00000051682	Treml4	triggering receptor expressed on myeloid cells-like	0.2466
ENSMUSG00000040350	Trim7	tripartite motif-containing 7 [Source:MGI Symbol;A	0.24666
ENSMUSG00000026989	Dapl1	death associated protein-like 1 [Source:MGI Symbo	0.24672
ENSMUSG00000064145	Arih2	ariadne homolog 2 (Drosophila) [Source:MGI Symb	0.24673

ENSMUSG00000035095	Fam167a	family with sequence similarity 167, member A [Sou	0.24684
ENSMUSG00000038637	Lrrc56	leucine rich repeat containing 56 [Source:MGI Syml	0.24684
ENSMUSG00000035673	Sbno2	strawberry notch homolog 2 (Drosophila) [Source:M	0.24689
ENSMUSG00000097755	2010110K18Rik	RIKEN cDNA 2010110K18 gene [Source:MGI Symbo	0.24705
ENSMUSG00000086725	A630052C17Rik	RIKEN cDNA A630052C17 gene [Source:MGI Symbo	0.24707
ENSMUSG00000030180	Kdm5a	lysine (K)-specific demethylase 5A [Source:MGI Sym	0.24709
ENSMUSG00000031903	Pla2g15	phospholipase A2, group XV [Source:MGI Symbol;A	0.24709
ENSMUSG00000097259	Gm26766	predicted gene, 26766 [Source:MGI Symbol;Acc:M(	0.24721
ENSMUSG00000092035	Peg10	paternally expressed 10 [Source:MGI Symbol;Acc:M	0.24725
ENSMUSG00000030528	Blm	Bloom syndrome, RecQ helicase-like [Source:MGI S	0.24726
ENSMUSG00000097191	5730422E09Rik	RIKEN cDNA 5730422E09 gene [Source:MGI Symbo	0.24737
ENSMUSG00000091109	Gm17622	predicted gene, 17622 [Source:MGI Symbol;Acc:M(	0.24738
ENSMUSG00000065590	Mir212	microRNA 212 [Source:MGI Symbol;Acc:MGI:26768	0.24739
ENSMUSG00000027209	Fam227b	family with sequence similarity 227, member B [Sou	0.24743
ENSMUSG00000092985	Mir378b	microRNA 378b [Source:MGI Symbol;Acc:MGI:4950	0.24743
ENSMUSG00000038298	Pdzk1	PDZ domain containing 1 [Source:MGI Symbol;Acc:	0.24744
ENSMUSG00000030869	Ndufab1	NADH dehydrogenase (ubiquinone) 1, alpha/beta s	0.24748
ENSMUSG00000067882	Casp16	caspase 16, apoptosis-related cysteine peptidase [S	0.24755
ENSMUSG00000025728	Pigq	phosphatidylinositol glycan anchor biosynthesis, cla	0.24774
ENSMUSG00000084771	A230072E10Rik	RIKEN cDNA A230072E10 gene [Source:MGI Symbo	0.24776
ENSMUSG00000090358	Gm2822	predicted gene 2822 [Source:MGI Symbol;Acc:MGI:	0.24782
ENSMUSG00000086507	Gm11206	predicted gene 11206 [Source:MGI Symbol;Acc:MG	0.2479
ENSMUSG00000042459	Bpifa2	BPI fold containing family A, member 2 [Source:MG	0.24798
ENSMUSG00000085042	2010001M06Rik	RIKEN cDNA 2010001M06 gene [Source:MGI Symb	0.24798
ENSMUSG00000080180	Gm17235	predicted gene 17235 [Source:MGI Symbol;Acc:MG	0.24799
ENSMUSG00000038181	Chpf2	chondroitin polymerizing factor 2 [Source:MGI Sym	0.24803
ENSMUSG00000071645	Tut1	terminal uridylyl transferase 1, U6 snRNA-specific [	0.24805
ENSMUSG00000020736	Nt5c	5',3'-nucleotidase, cytosolic [Source:MGI Symbol;A	0.24807
ENSMUSG00000039485	Tspyl4	TSPY-like 4 [Source:MGI Symbol;Acc:MGI:106393]	0.24808
ENSMUSG00000020180	Snrpd3	small nuclear ribonucleoprotein D3 [Source:MGI Sy	0.2481
ENSMUSG00000050424	Pnma5	paraneoplastic antigen family 5 [Source:MGI Symb	0.24827
ENSMUSG00000077382	n-R5s200	nuclear encoded rRNA 5S 200 [Source:MGI Symbol;	0.24831
ENSMUSG00000051729	Gm5087	predicted gene 5087 [Source:MGI Symbol;Acc:MGI:	0.24832
ENSMUSG00000069495	Epc2	enhancer of polycomb homolog 2 (Drosophila) [Sou	0.24832
ENSMUSG00000036606	Plxnb2	plexin B2 [Source:MGI Symbol;Acc:MGI:2154239]	0.24834
ENSMUSG00000027878	Notch2	notch 2 [Source:MGI Symbol;Acc:MGI:97364]	0.24836
ENSMUSG00000084159	Gm12696	predicted gene 12696 [Source:MGI Symbol;Acc:MG	0.24846
ENSMUSG00000031799	Tpm4	tropomyosin 4 [Source:MGI Symbol;Acc:MGI:24492	0.24848
ENSMUSG00000075570	Krt26	keratin 26 [Source:MGI Symbol;Acc:MGI:2444913]	0.24852
ENSMUSG00000085135	Gm13713	predicted gene 13713 [Source:MGI Symbol;Acc:MG	0.24854
ENSMUSG00000034858	Fam214a	family with sequence similarity 214, member A [Sou	0.24856
ENSMUSG00000039959	Hip1	huntingtin interacting protein 1 [Source:MGI Symb	0.24858
ENSMUSG00000022878	Adipoq	adiponectin, C1Q and collagen domain containing [	0.24874
ENSMUSG00000029909	Prss37	protease, serine, 37 [Source:MGI Symbol;Acc:MGI:	0.24891
ENSMUSG00000087067	Gm11532	predicted gene 11532 [Source:MGI Symbol;Acc:MG	0.24892
ENSMUSG00000041488	Stx3	syntaxin 3 [Source:MGI Symbol;Acc:MGI:103077]	0.24893
ENSMUSG00000055707	Klhl26	kelch-like 26 [Source:MGI Symbol;Acc:MGI:244307	0.24894

ENSMUSG00000028982	Slc25a33	solute carrier family 25, member 33 [Source:MGI Sy	0.24897
ENSMUSG00000027931	Npr1	natriuretic peptide receptor 1 [Source:MGI Symbol	0.24899
ENSMUSG00000046947	Adck2	aarF domain containing kinase 2 [Source:MGI Symb	0.24899
ENSMUSG00000014301	Pam16	presequence translocase-asssociated motor 16 hor	0.24907
ENSMUSG00000003970	Rpl8	ribosomal protein L8 [Source:MGI Symbol;Acc:MGI	0.24909
ENSMUSG00000053081	1700069B07Rik	RIKEN cDNA 1700069B07 gene [Source:MGI Symbo	0.24916
ENSMUSG00000097124	A530020G20Rik	RIKEN cDNA A530020G20 gene [Source:MGI Symbo	0.24918
ENSMUSG00000027164	Traf6	TNF receptor-associated factor 6 [Source:MGI Symb	0.24919
ENSMUSG00000027339	Rassf2	Ras association (RalGDS/AF-6) domain family memb	0.24926
ENSMUSG00000023272	Creld2	cysteine-rich with EGF-like domains 2 [Source:MGI	0.24927
ENSMUSG00000029923	Rab19	RAB19, member RAS oncogene family [Source:MGI	0.24935
ENSMUSG00000091402	BC048943	cDNA sequence BC048943 [Source:MGI Symbol;Acc	0.24936
ENSMUSG00000097390	Gm26785	predicted gene, 26785 [Source:MGI Symbol;Acc:MG	0.24942
ENSMUSG00000071037	Camkmt	calmodulin-lysine N-methyltransferase [Source:MG	0.24967
ENSMUSG00000094344	Gm11942	predicted gene 11942 [Source:MGI Symbol;Acc:MG	0.24969
ENSMUSG00000027163	Commd9	COMM domain containing 9 [Source:MGI Symbol;A	0.24975
ENSMUSG00000025867	Cplx2	complexin 2 [Source:MGI Symbol;Acc:MGI:104726]	0.24979
ENSMUSG00000058388	Phtf1	putative homeodomain transcription factor 1 [Sour	0.24982
ENSMUSG00000091957	Rps2-ps10	ribosomal protein S2, pseudogene 10 [Source:MGI	0.2501
ENSMUSG00000040250	Asun	asunder, spermatogenesis regulator [Source:MGI S	0.25011
ENSMUSG00000052688	5430435G22Rik	RIKEN cDNA 5430435G22 gene [Source:MGI Symbo	0.25018
ENSMUSG00000062797	l7Rn6	lethal, Chr 7, Rinchik 6 [Source:MGI Symbol;Acc:MG	0.25019
ENSMUSG00000036641	Ccdc148	coiled-coil domain containing 148 [Source:MGI Sym	0.2502
ENSMUSG00000031155	Pim2	proviral integration site 2 [Source:MGI Symbol;Acc:	0.25045
ENSMUSG00000025572	Tmc6	transmembrane channel-like gene family 6 [Source	0.25053
ENSMUSG00000026304	Rab17	RAB17, member RAS oncogene family [Source:MGI	0.25058
ENSMUSG00000023151	Lrrc69	leucine rich repeat containing 69 [Source:MGI Symb	0.25061
ENSMUSG00000086766	Gm15969	predicted gene 15969 [Source:MGI Symbol;Acc:MG	0.25061
ENSMUSG00000032382	Snx1	sorting nexin 1 [Source:MGI Symbol;Acc:MGI:19283	0.25063
ENSMUSG00000097953	Gm26952	predicted gene, 26952 [Source:MGI Symbol;Acc:MG	0.25066
ENSMUSG00000057060	Slc35f3	solute carrier family 35, member F3 [Source:MGI Sy	0.25071
ENSMUSG00000059244	Gm10062	predicted gene 10062 [Source:MGI Symbol;Acc:MG	0.25076
ENSMUSG00000039048	Foxred1	FAD-dependent oxidoreductase domain containing	0.2509
ENSMUSG00000038840	Birc7	baculoviral IAP repeat-containing 7 (livin) [Source:M	0.25093
ENSMUSG00000026725	Tnn	tenascin N [Source:MGI Symbol;Acc:MGI:2665790]	0.25095
ENSMUSG00000020740	Gga3	golgi associated, gamma adaptin ear containing, AR	0.25101
ENSMUSG00000025209	Peo1	progressive external ophthalmoplegia 1 (human) [S	0.25103
ENSMUSG00000096296	Snord88c	small nucleolar RNA, C/D box 88C [Source:MGI Sym	0.25106
ENSMUSG00000081887	Gm12097	predicted gene 12097 [Source:MGI Symbol;Acc:MG	0.25111
ENSMUSG00000034801	Sos2	son of sevenless homolog 2 (Drosophila) [Source:M	0.25125
ENSMUSG00000002010	Idh3g	isocitrate dehydrogenase 3 (NAD+), gamma [Source	0.25132
ENSMUSG00000062480	Acat3	acetyl-Coenzyme A acetyltransferase 3 [Source:MG	0.25137
ENSMUSG00000071690	Gm10344	predicted gene 10344 [Source:MGI Symbol;Acc:MG	0.25141
ENSMUSG00000057895	Zfp105	zinc finger protein 105 [Source:MGI Symbol;Acc:MG	0.25162
ENSMUSG00000036880	Acaa2	acetyl-Coenzyme A acyltransferase 2 (mitochondria	0.25176
ENSMUSG00000032175	Tyk2	tyrosine kinase 2 [Source:MGI Symbol;Acc:MGI:192	0.25183
ENSMUSG00000034006	Pqlc1	PQ loop repeat containing 1 [Source:MGI Symbol;A	0.25204

ENSMUSG00000086196	Gm13571	predicted gene 13571 [Source:MGI Symbol;Acc:MG	0.25207
ENSMUSG00000054770	Kctd18	potassium channel tetramerisation domain contain	0.25208
ENSMUSG00000032609	Klhdc8b	kelch domain containing 8B [Source:MGI Symbol;Ac	0.25213
ENSMUSG00000063931	Pepd	peptidase D [Source:MGI Symbol;Acc:MGI:97542]	0.25214
ENSMUSG00000031570	Ppapdc1b	phosphatidic acid phosphatase type 2 domain cont	0.25225
ENSMUSG00000027401	Tgm3	transglutaminase 3, E polypeptide [Source:MGI Syn	0.25239
ENSMUSG00000029575	Mmab	methylmalonic aciduria (cobalamin deficiency) type	0.25245
ENSMUSG00000021007	Spata7	spermatogenesis associated 7 [Source:MGI Symbol	0.25247
ENSMUSG00000031220	Awat2	acyl-CoA wax alcohol acyltransferase 2 [Source:MG	0.25248
ENSMUSG00000058099	Nfam1	Nfat activating molecule with ITAM motif 1 [Source	0.25248
ENSMUSG00000097402	Gm17399	predicted gene, 17399 [Source:MGI Symbol;Acc:MG	0.2525
ENSMUSG00000022621	Rabl2	RAB, member of RAS oncogene family-like 2 [Sourc	0.25285
ENSMUSG00000063935	Zar1	zygote arrest 1 [Source:MGI Symbol;Acc:MGI:2180	0.25287
ENSMUSG00000010453	Kansl3	KAT8 regulatory NSL complex subunit 3 [Source:MG	0.25288
ENSMUSG00000061780	Cfd	complement factor D (adipsin) [Source:MGI Symbo	0.25291
ENSMUSG00000026222	Sp100	nuclear antigen Sp100 [Source:MGI Symbol;Acc:MG	0.25295
ENSMUSG00000029627	Zkscan14	zinc finger with KRAB and SCAN domains 14 [Sourc	0.25299
ENSMUSG00000078519	2310026L22Rik	RIKEN cDNA 2310026L22 gene [Source:MGI Symbo	0.25301
ENSMUSG00000083819	Gm11977	predicted gene 11977 [Source:MGI Symbol;Acc:MG	0.25308
ENSMUSG00000057554	Lgals8	lectin, galactose binding, soluble 8 [Source:MGI Syn	0.25311
ENSMUSG00000031984	2810004N23Rik	RIKEN cDNA 2810004N23 gene [Source:MGI Symbo	0.25316
ENSMUSG00000053581	Zfand2a	zinc finger, AN1-type domain 2A [Source:MGI Symb	0.25316
ENSMUSG00000037251	Pomk	protein-O-mannose kinase [Source:MGI Symbol;Acc	0.25325
ENSMUSG00000097758	Dio3os	deiodinase, iodothyronine type III, opposite strand	0.25325
ENSMUSG00000016757	Ttll12	tubulin tyrosine ligase-like family, member 12 [Sou	0.25326
ENSMUSG00000021604	Irx4	Iroquois related homeobox 4 (Drosophila) [Source:	0.25342
ENSMUSG00000029434	Vps33a	vacuolar protein sorting 33A (yeast) [Source:MGI Sy	0.25344
ENSMUSG00000089911	Hiat1	hippocampus abundant gene transcript 1 [Source:N	0.25348
ENSMUSG00000027238	Frmd5	FERM domain containing 5 [Source:MGI Symbol;Ac	0.25352
ENSMUSG00000014867	Surf4	surfeit gene 4 [Source:MGI Symbol;Acc:MGI:98445	0.25353
ENSMUSG00000024767	Otub1	OTU domain, ubiquitin aldehyde binding 1 [Source:	0.25354
ENSMUSG00000025871	4833439L19Rik	RIKEN cDNA 4833439L19 gene [Source:MGI Symbo	0.25358
ENSMUSG00000065563	Mir103-2	microRNA 103-2 [Source:MGI Symbol;Acc:MGI:361	0.25366
ENSMUSG00000085008	Gm13399	predicted gene 13399 [Source:MGI Symbol;Acc:MG	0.25368
ENSMUSG00000002741	Ykt6	YKT6 homolog (S. Cerevisiae) [Source:MGI Symbol;	0.25373
ENSMUSG00000001630	Stk38l	serine/threonine kinase 38 like [Source:MGI Symbo	0.25379
ENSMUSG00000029528	Pxn	paxillin [Source:MGI Symbol;Acc:MGI:108295]	0.25382
ENSMUSG00000091079	Gm17105	predicted gene 17105 [Source:MGI Symbol;Acc:MG	0.25382
ENSMUSG00000039903	Eva1c	eva-1 homolog C (C. elegans) [Source:MGI Symbol;	0.25397
ENSMUSG00000036466	Megf11	multiple EGF-like-domains 11 [Source:MGI Symbol;	0.25401
ENSMUSG00000086344	Gm11345	predicted gene 11345 [Source:MGI Symbol;Acc:MG	0.25413
ENSMUSG00000004069	Dnaja3	DnaJ (Hsp40) homolog, subfamily A, member 3 [Sou	0.25417
ENSMUSG00000096956	0610007N19Rik	RIKEN cDNA 0610007N19 [Source:MGI Symbol;Acc	0.25421
ENSMUSG00000024519	Cplx4	complexin 4 [Source:MGI Symbol;Acc:MGI:2685803	0.25426
ENSMUSG00000037235	Mxd4	Max dimerization protein 4 [Source:MGI Symbol;Ac	0.25426
ENSMUSG00000085794	Vax2os	Vax2 opposite strand transcript [Source:MGI Symbo	0.25426
ENSMUSG00000089647	Gm2245	predicted gene 2245 [Source:MGI Symbol;Acc:MGI	0.2543

ENSMUSG00000020994	Pnn	pinin [Source:MGI Symbol;Acc:MGI:1100514]	0.25434
ENSMUSG00000097166	9330179D12Rik	RIKEN cDNA 9330179D12 gene [Source:MGI Symbo	0.25442
ENSMUSG00000094671	Gm20382	predicted gene, 20382 [Source:MGI Symbol;Acc:M	0.25443
ENSMUSG00000005804	Bloc1s6	biogenesis of organelles complex-1, subunit 6, palli	0.25454
ENSMUSG00000097290	1300002E11Rik	RIKEN cDNA 1300002E11 gene [Source:MGI Symbo	0.25457
ENSMUSG00000079262	Slco1a6	solute carrier organic anion transporter family, mer	0.25459
ENSMUSG00000030532	Hddc3	HD domain containing 3 [Source:MGI Symbol;Acc:M	0.25461
ENSMUSG00000019731	Slc35e1	solute carrier family 35, member E1 [Source:MGI Sy	0.25474
ENSMUSG00000003228	Grk5	G protein-coupled receptor kinase 5 [Source:MGI S	0.25479
ENSMUSG00000031934	Panx1	pannexin 1 [Source:MGI Symbol;Acc:MGI:1860055]	0.25489
ENSMUSG00000075555	Gm10863	predicted gene 10863 [Source:MGI Symbol;Acc:MG	0.25489
ENSMUSG00000087271	2900008C10Rik	RIKEN cDNA 2900008C10 gene [Source:MGI Symbo	0.25489
ENSMUSG00000030887	Pdzd9	PDZ domain containing 9 [Source:MGI Symbol;Acc:	0.25493
ENSMUSG00000042216	Sgsm1	small G protein signaling modulator 1 [Source:MGI	0.255
ENSMUSG00000048406	B330016D10Rik	RIKEN cDNA B330016D10 gene [Source:MGI Symbo	0.25502
ENSMUSG00000032000	Birc3	baculoviral IAP repeat-containing 3 [Source:MGI Sy	0.25506
ENSMUSG00000019899	Lama2	laminin, alpha 2 [Source:MGI Symbol;Acc:MGI:9991	0.25537
ENSMUSG00000033713	Foxn3	forkhead box N3 [Source:MGI Symbol;Acc:MGI:191	0.25537
ENSMUSG00000024019	Cmtr1	cap methyltransferase 1 [Source:MGI Symbol;Acc:M	0.25543
ENSMUSG00000029759	Pon3	paraoxonase 3 [Source:MGI Symbol;Acc:MGI:10668	0.25548
ENSMUSG00000045506	A930002H24Rik	RIKEN cDNA A930002H24 gene [Source:MGI Symbo	0.25549
ENSMUSG00000024427	Spry4	sprouty homolog 4 (Drosophila) [Source:MGI Symb	0.2555
ENSMUSG00000042579	4632404H12Rik	RIKEN cDNA 4632404H12 gene [Source:MGI Symbo	0.25565
ENSMUSG00000086238	Gm14258	predicted gene 14258 [Source:MGI Symbol;Acc:MG	0.25566
ENSMUSG00000060019	Gm10073	predicted pseudogene 10073 [Source:MGI Symbol;	0.25599
ENSMUSG00000042524	Sun2	Sad1 and UNC84 domain containing 2 [Source:MGI	0.2561
ENSMUSG00000020288	Ahsa2	AHA1, activator of heat shock protein ATPase 2 [So	0.25614
ENSMUSG00000024986	Hhex	hematopoietically expressed homeobox [Source:M	0.25617
ENSMUSG00000058267	Mrps14	mitochondrial ribosomal protein S14 [Source:MGI S	0.25617
ENSMUSG00000062044	Lmtk3	lemur tyrosine kinase 3 [Source:MGI Symbol;Acc:M	0.25648
ENSMUSG00000074282	Zfp94	zinc finger protein 94 [Source:MGI Symbol;Acc:MGI	0.25649
ENSMUSG00000035678	Tnfsf9	tumor necrosis factor (ligand) superfamily, membe	0.25651
ENSMUSG00000039774	Galnt12	UDP-N-acetyl-alpha-D-galactosamine:polypeptide N	0.25654
ENSMUSG00000015461	Atf6b	activating transcription factor 6 beta [Source:MGI S	0.25655
ENSMUSG00000058056	Palld	palladin, cytoskeletal associated protein [Source:M	0.25658
ENSMUSG00000029223	Uchl1	ubiquitin carboxy-terminal hydrolase L1 [Source:M	0.2567
ENSMUSG00000074405	Zfp865	zinc finger protein 865 [Source:MGI Symbol;Acc:M	0.25673
ENSMUSG00000091238	Gm17103	predicted gene 17103 [Source:MGI Symbol;Acc:MG	0.25676
ENSMUSG00000002332	Dhrs1	dehydrogenase/reductase (SDR family) member 1 [	0.25684
ENSMUSG00000085083	Gm11615	predicted gene 11615 [Source:MGI Symbol;Acc:MG	0.25687
ENSMUSG00000045275	Lca5l	Leber congenital amaurosis 5-like [Source:MGI Sym	0.25691
ENSMUSG00000053550	Shisa7	shisa homolog 7 (Xenopus laevis) [Source:MGI Sym	0.25693
ENSMUSG00000072662	Mansc4	MANSC domain containing 4 [Source:MGI Symbol;A	0.25702
ENSMUSG00000025237	Parp6	poly (ADP-ribose) polymerase family, member 6 [So	0.25703
ENSMUSG00000064347	mt-Ta	mitochondrially encoded tRNA alanine [Source:MG	0.25706
ENSMUSG00000033377	Palmd	palmdelphin [Source:MGI Symbol;Acc:MGI:214889	0.25712
ENSMUSG00000029047	Pex10	peroxisomal biogenesis factor 10 [Source:MGI Sym	0.25724

ENSMUSG00000029559	2210016L21Rik	RIKEN cDNA 2210016L21 gene [Source:MGI Symbo	0.25725
ENSMUSG00000000037	Scml2	sex comb on midleg-like 2 (Drosophila) [Source:MG	0.25726
ENSMUSG00000038644	Pold1	polymerase (DNA directed), delta 1, catalytic subur	0.25735
ENSMUSG00000086629	2810403D21Rik	RIKEN cDNA 2810403D21 gene [Source:MGI Symbo	0.25735
ENSMUSG00000086584	Gm12002	predicted gene 12002 [Source:MGI Symbol;Acc:MG	0.25741
ENSMUSG00000024339	Tap2	transporter 2, ATP-binding cassette, sub-family B (M	0.25751
ENSMUSG00000049517	Rps23	ribosomal protein S23 [Source:MGI Symbol;Acc:MG	0.25766
ENSMUSG00000027953	Slc50a1	solute carrier family 50 (sugar transporter), membe	0.25769
ENSMUSG00000066170	E230001N04Rik	RIKEN cDNA E230001N04 gene [Source:MGI Symbo	0.2577
ENSMUSG00000036968	Cnpy4	canopy 4 homolog (zebrafish) [Source:MGI Symbol;	0.25773
ENSMUSG00000025235	Bbs4	Bardet-Biedl syndrome 4 (human) [Source:MGI Sym	0.25778
ENSMUSG00000082283	Gm14472	predicted gene 14472 [Source:MGI Symbol;Acc:MG	0.25778
ENSMUSG00000020776	Fbf1	Fas (TNFRSF6) binding factor 1 [Source:MGI Symbo	0.25785
ENSMUSG00000001870	Ltbp1	latent transforming growth factor beta binding prot	0.25787
ENSMUSG00000024140	Epas1	endothelial PAS domain protein 1 [Source:MGI Sym	0.25787
ENSMUSG00000016496	Cd274	CD274 antigen [Source:MGI Symbol;Acc:MGI:19264	0.25793
ENSMUSG00000091154	1700024N20Rik	RIKEN cDNA 1700024N20Rik gene [Source:MGI Sym	0.25794
ENSMUSG00000023072	Cep89	centrosomal protein 89 [Source:MGI Symbol;Acc:M	0.25796
ENSMUSG00000025024	Smndc1	survival motor neuron domain containing 1 [Source	0.25814
ENSMUSG00000082051	Gm16072	predicted gene 16072 [Source:MGI Symbol;Acc:MG	0.25815
ENSMUSG00000040365	Trim41	tripartite motif-containing 41 [Source:MGI Symbol;	0.2583
ENSMUSG00000042870	Tom1	target of myb1 homolog (chicken) [Source:MGI Sym	0.2584
ENSMUSG00000021096	Ppm1a	protein phosphatase 1A, magnesium dependent, al	0.25843
ENSMUSG00000048696	Mex3d	mex3 homolog D (C. elegans) [Source:MGI Symbol;	0.25847
ENSMUSG00000046709	Mapk10	mitogen-activated protein kinase 10 [Source:MGI S	0.25864
ENSMUSG00000074199	Krtdap	keratinocyte differentiation associated protein [Sou	0.25873
ENSMUSG00000021779	Thrb	thyroid hormone receptor beta [Source:MGI Symbo	0.25878
ENSMUSG00000072109	A530040E14Rik	RIKEN cDNA A530040E14 gene [Source:MGI Symbo	0.25891
ENSMUSG00000039438	Ttc36	tetratricopeptide repeat domain 36 [Source:MGI Sy	0.25898
ENSMUSG00000070371	Prss36	protease, serine, 36 [Source:MGI Symbol;Acc:MGI:1	0.25912
ENSMUSG00000038069	Cdkn2aip	CDKN2A interacting protein [Source:MGI Symbol;Ac	0.25921
ENSMUSG00000020628	Trappc12	trafficking protein particle complex 12 [Source:MGI	0.25924
ENSMUSG00000073591	Pcdhb22	protocadherin beta 22 [Source:MGI Symbol;Acc:MG	0.25927
ENSMUSG00000082499	Gm13615	predicted gene 13615 [Source:MGI Symbol;Acc:MG	0.2593
ENSMUSG00000029034	Cpsf3l	cleavage and polyadenylation specific factor 3-like	0.25936
ENSMUSG00000095663	Gm2310	predicted gene 2310 [Source:MGI Symbol;Acc:MGI:	0.25939
ENSMUSG00000030155	Clec2e	C-type lectin domain family 2, member e [Source:M	0.2594
ENSMUSG00000097740	E030044B06Rik	RIKEN cDNA E030044B06 gene [Source:MGI Symbo	0.25954
ENSMUSG00000038704	Aspdh	aspartate dehydrogenase domain containing [Sourc	0.25969
ENSMUSG00000073680	Tmem88b	transmembrane protein 88B [Source:MGI Symbol;A	0.25969
ENSMUSG00000027091	Zc3h15	zinc finger CCCH-type containing 15 [Source:MGI Sy	0.25973
ENSMUSG00000028158	Mttp	microsomal triglyceride transfer protein [Source:M	0.25974
ENSMUSG00000022199	Slc22a17	solute carrier family 22 (organic cation transporter)	0.25976
ENSMUSG00000026883	Dab2ip	disabled 2 interacting protein [Source:MGI Symbol;	0.25981
ENSMUSG00000025613	Cct8	chaperonin containing Tcp1, subunit 8 (theta) [Sour	0.25983
ENSMUSG00000087064	Gm11721	predicted gene 11721 [Source:MGI Symbol;Acc:MG	0.25996
ENSMUSG00000073158	9030624G23Rik	RIKEN cDNA 9030624G23 gene [Source:MGI Symbo	0.25998

ENSMUSG00000009569	Mkl2	MKL/myocardin-like 2 [Source:MGI Symbol;Acc:MG	0.26016
ENSMUSG00000084157	Gm15576	predicted gene 15576 [Source:MGI Symbol;Acc:MG	0.26024
ENSMUSG00000028631	Kcnq4	potassium voltage-gated channel, subfamily Q, mer	0.26036
ENSMUSG00000060985	Tdrd5	tudor domain containing 5 [Source:MGI Symbol;Acc	0.26046
ENSMUSG00000001924	Uba1	ubiquitin-like modifier activating enzyme 1 [Source	0.26048
ENSMUSG00000038526	Car14	carbonic anhydrase 14 [Source:MGI Symbol;Acc:MG	0.26048
ENSMUSG00000029033	Acap3	ArfGAP with coiled-coil, ankyrin repeat and PH dom	0.2605
ENSMUSG00000041674	BC006965	cDNA sequence BC006965 [Source:MGI Symbol;Acc	0.26051
ENSMUSG00000029310	Nudt9	nudix (nucleoside diphosphate linked moiety X)-typ	0.26058
ENSMUSG00000015850	Adamtsl4	ADAMTS-like 4 [Source:MGI Symbol;Acc:MGI:2389(	0.26065
ENSMUSG00000015597	Zfp318	zinc finger protein 318 [Source:MGI Symbol;Acc:MG	0.2607
ENSMUSG00000047497	Adamts12	a disintegrin-like and metallopeptidase (reprolysin	0.26073
ENSMUSG00000075024	Gm10803	predicted gene 10803 [Source:MGI Symbol;Acc:MG	0.26079
ENSMUSG00000095098	Ccdc85b	coiled-coil domain containing 85B [Source:MGI Sym	0.26086
ENSMUSG00000032788	Pdxk	pyridoxal (pyridoxine, vitamin B6) kinase [Source:M	0.26089
ENSMUSG00000087512	Gm11985	predicted gene 11985 [Source:MGI Symbol;Acc:MG	0.26091
ENSMUSG00000029089	Pacrgl	PARK2 co-regulated-like [Source:MGI Symbol;Acc:N	0.26104
ENSMUSG00000023873	1700010I14Rik	RIKEN cDNA 1700010I14 gene [Source:MGI Symbol	0.26109
ENSMUSG00000033777	Tlr13	toll-like receptor 13 [Source:MGI Symbol;Acc:MGI:3	0.26109
ENSMUSG00000090206	Tepp	testis, prostate and placenta expressed [Source:MG	0.26113
ENSMUSG00000043885	Slc36a4	solute carrier family 36 (proton/amino acid sympor	0.26121
ENSMUSG00000065578	Mir181b-2	microRNA 181b-2 [Source:MGI Symbol;Acc:MGI:36	0.26124
ENSMUSG00000097333	Zfp87	zinc finger protein 87 [Source:MGI Symbol;Acc:MGI	0.26125
ENSMUSG00000027107	Chrna1	cholinergic receptor, nicotinic, alpha polypeptide 1	0.2614
ENSMUSG00000030600	Lrfn1	leucine rich repeat and fibronectin type III domain	0.26146
ENSMUSG00000023010	Tmbim6	transmembrane BAX inhibitor motif containing 6 [S	0.26152
ENSMUSG00000097709	2810429I04Rik	RIKEN cDNA 2810429I04 gene [Source:MGI Symbol	0.26155
ENSMUSG00000048332	Lhfp	lipoma HMGIC fusion partner [Source:MGI Symbol;	0.2616
ENSMUSG00000024283	Wac	WW domain containing adaptor with coiled-coil [So	0.26161
ENSMUSG00000031979	Cog2	component of oligomeric golgi complex 2 [Source:N	0.26182
ENSMUSG00000037482	Erv3	endogenous retroviral sequence 3 [Source:MGI Sym	0.26193
ENSMUSG00000004446	Bid	BH3 interacting domain death agonist [Source:MGI	0.262
ENSMUSG00000063434	Sorcs3	sortilin-related VPS10 domain containing receptor 3	0.262
ENSMUSG00000022708	Zbtb20	zinc finger and BTB domain containing 20 [Source:N	0.26202
ENSMUSG00000024565	Sall3	sal-like 3 (Drosophila) [Source:MGI Symbol;Acc:MG	0.26203
ENSMUSG00000037275	Gemin5	gem (nuclear organelle) associated protein 5 [Sourc	0.26218
ENSMUSG00000083718	Ccnb2-ps	cyclin B2, pseudogene [Source:MGI Symbol;Acc:MG	0.26222
ENSMUSG00000051989	Smim11	small integral membrane protein 11 [Source:MGI Sy	0.26227
ENSMUSG00000082491	Gm5909	predicted gene 5909 [Source:MGI Symbol;Acc:MGI:	0.26234
ENSMUSG00000019461	Plscr3	phospholipid scramblase 3 [Source:MGI Symbol;Acc	0.26235
ENSMUSG00000040296	Ddx58	DEAD (Asp-Glu-Ala-Asp) box polypeptide 58 [Sourc	0.26235
ENSMUSG00000049957	Ccdc137	coiled-coil domain containing 137 [Source:MGI Sym	0.26238
ENSMUSG00000050552	Lamtor4	late endosomal/lysosomal adaptor, MAPK and MTC	0.26247
ENSMUSG00000088453	Gm25706	predicted gene, 25706 [Source:MGI Symbol;Acc:MG	0.26251
ENSMUSG00000028362	Tnfsf8	tumor necrosis factor (ligand) superfamily, membe	0.26253
ENSMUSG00000005696	Sh2d1a	SH2 domain protein 1A [Source:MGI Symbol;Acc:M	0.26282
ENSMUSG00000001767	Crnkl1	Crn, crooked neck-like 1 (Drosophila) [Source:MGI S	0.26286

ENSMUSG00000038622	Med30	mediator complex subunit 30 [Source:MGI Symbol;	0.26287
ENSMUSG00000029096	Htra3	HtrA serine peptidase 3 [Source:MGI Symbol;Acc:M	0.26288
ENSMUSG00000034880	Mrpl34	mitochondrial ribosomal protein L34 [Source:MGI S	0.26291
ENSMUSG00000020709	Adap2	ArfGAP with dual PH domains 2 [Source:MGI Symbo	0.26305
ENSMUSG00000024253	Dync2li1	dynein cytoplasmic 2 light intermediate chain 1 [So	0.26317
ENSMUSG00000055456	Gm9972	predicted gene 9972 [Source:MGI Symbol;Acc:MGI:	0.26323
ENSMUSG00000022231	Sema5a	sema domain, seven thrombospondin repeats (type	0.26324
ENSMUSG00000054942	Fam73a	family with sequence similarity 73, member A [Sour	0.26326
ENSMUSG00000063281	Zfp35	zinc finger protein 35 [Source:MGI Symbol;Acc:MGI	0.2633
ENSMUSG00000056106	Gm9990	predicted gene 9990 [Source:MGI Symbol;Acc:MGI:	0.26345
ENSMUSG00000004768	Rab23	RAB23, member RAS oncogene family [Source:MGI	0.26363
ENSMUSG00000029616	Erp29	endoplasmic reticulum protein 29 [Source:MGI Sym	0.26363
ENSMUSG00000049872	Fam26e	family with sequence similarity 26, member E [Sour	0.26372
ENSMUSG00000041351	Rap1gap	Rap1 GTPase-activating protein [Source:MGI Symbo	0.26375
ENSMUSG00000031519	Asb5	ankyrin repeat and SOCs box-containing 5 [Source:I	0.26395
ENSMUSG00000059495	Arhgef12	Rho guanine nucleotide exchange factor (GEF) 12 [S	0.26396
ENSMUSG00000073002	Vamp5	vesicle-associated membrane protein 5 [Source:MG	0.26397
ENSMUSG00000019960	Dusp6	dual specificity phosphatase 6 [Source:MGI Symbol	0.26404
ENSMUSG00000050840	Cdh20	cadherin 20 [Source:MGI Symbol;Acc:MGI:1346069	0.26404
ENSMUSG00000063953	Amd2	S-adenosylmethionine decarboxylase 2 [Source:MG	0.26414
ENSMUSG00000028136	Snx27	sorting nexin family member 27 [Source:MGI Symb	0.26429
ENSMUSG00000021131	Erh	enhancer of rudimentary homolog (Drosophila) [So	0.26453
ENSMUSG00000024803	Ankrd1	ankyrin repeat domain 1 (cardiac muscle) [Source:N	0.26454
ENSMUSG00000049357	4933408B17Rik	RIKEN cDNA 4933408B17 gene [Source:MGI Symbo	0.26461
ENSMUSG00000086355	4933428C19Rik	RIKEN cDNA 4933428C19 gene [Source:MGI Symbo	0.26461
ENSMUSG00000024558	Mapk4	mitogen-activated protein kinase 4 [Source:MGI Sy	0.26473
ENSMUSG00000034109	Golim4	golgi integral membrane protein 4 [Source:MGI Syn	0.26474
ENSMUSG00000027257	Pacsin3	protein kinase C and casein kinase substrate in neu	0.2648
ENSMUSG00000028542	Slc6a9	solute carrier family 6 (neurotransmitter transporte	0.2648
ENSMUSG00000027887	Sypl2	synaptophysin-like 2 [Source:MGI Symbol;Acc:MGI:	0.26484
ENSMUSG00000003528	Slc25a1	solute carrier family 25 (mitochondrial carrier, citra	0.26486
ENSMUSG00000037577	Ephx3	epoxide hydrolase 3 [Source:MGI Symbol;Acc:MGI:	0.26488
ENSMUSG00000054766	Set	SET nuclear oncogene [Source:MGI Symbol;Acc:MG	0.26491
ENSMUSG00000083906	Gm15186	predicted gene 15186 [Source:MGI Symbol;Acc:MG	0.26497
ENSMUSG00000026018	Ica1l	islet cell autoantigen 1-like [Source:MGI Symbol;Ac	0.26498
ENSMUSG00000026525	Opn3	opsin 3 [Source:MGI Symbol;Acc:MGI:1338022]	0.26498
ENSMUSG00000032492	Pth1r	parathyroid hormone 1 receptor [Source:MGI Symb	0.26498
ENSMUSG00000033053	1700028P14Rik	RIKEN cDNA 1700028P14 gene [Source:MGI Symbo	0.26505
ENSMUSG00000051242	Pcdhb9	protocadherin beta 9 [Source:MGI Symbol;Acc:MGI	0.26508
ENSMUSG00000027646	Src	Rous sarcoma oncogene [Source:MGI Symbol;Acc:M	0.26517
ENSMUSG00000071350	Setdb2	SET domain, bifurcated 2 [Source:MGI Symbol;Acc:	0.26517
ENSMUSG00000028573	Fggy	FGGY carbohydrate kinase domain containing [Sour	0.2652
ENSMUSG00000047658	Gal3st3	galactose-3-O-sulfotransferase 3 [Source:MGI Symb	0.2652
ENSMUSG00000041986	Elmod1	ELMO/CED-12 domain containing 1 [Source:MGI Sy	0.26526
ENSMUSG00000052296	Ppp6r1	protein phosphatase 6, regulatory subunit 1 [Sourc	0.26529
ENSMUSG00000027131	Emc4	ER membrane protein complex subunit 4 [Source:M	0.26535
ENSMUSG00000038406	Scaf1	SR-related CTD-associated factor 1 [Source:MGI Syr	0.26536

ENSMUSG00000093559	Gm20705	predicted gene 20705 [Source:MGI Symbol;Acc:MG	0.26538
ENSMUSG00000090217	Gm16116	predicted gene 16116 [Source:MGI Symbol;Acc:MG	0.26539
ENSMUSG00000086065	Gm16180	predicted gene 16180 [Source:MGI Symbol;Acc:MG	0.26541
ENSMUSG00000068580	Zfyve19	zinc finger, FYVE domain containing 19 [Source:MG	0.26547
ENSMUSG00000073676	Hspe1	heat shock protein 1 (chaperonin 10) [Source:MGI S	0.26547
ENSMUSG00000059713	Rcan3	regulator of calcineurin 3 [Source:MGI Symbol;Acc:	0.26563
ENSMUSG00000028840	Zfp593	zinc finger protein 593 [Source:MGI Symbol;Acc:MG	0.26565
ENSMUSG00000066406	Akap13	A kinase (PRKA) anchor protein 13 [Source:MGI Syn	0.26565
ENSMUSG00000085741	5430405H02Rik	RIKEN cDNA 5430405H02 gene [Source:MGI Symbo	0.26572
ENSMUSG00000050677	Ccdc96	coiled-coil domain containing 96 [Source:MGI Symb	0.26578
ENSMUSG00000062997	Rpl35	ribosomal protein L35 [Source:MGI Symbol;Acc:MG	0.26578
ENSMUSG00000011837	Snapc2	small nuclear RNA activating complex, polypeptide	0.26605
ENSMUSG00000032396	Dis3l	DIS3 mitotic control homolog (S. cerevisiae)-like [So	0.26613
ENSMUSG00000046818	Ddit4l	DNA-damage-inducible transcript 4-like [Source:MG	0.26616
ENSMUSG00000021038	Vipas39	VPS33B interacting protein, apical-basolateral polar	0.26617
ENSMUSG00000021469	Msx2	msh homeobox 2 [Source:MGI Symbol;Acc:MGI:971	0.26625
ENSMUSG00000024150	Mcfd2	multiple coagulation factor deficiency 2 [Source:MG	0.2663
ENSMUSG00000030636	1700010L04Rik	RIKEN cDNA 1700010L04 gene [Source:MGI Symbo	0.26632
ENSMUSG00000085549	1700047F07Rik	RIKEN cDNA 1700047F07 gene [Source:MGI Symbo	0.26634
ENSMUSG00000026941	Mamdc4	MAM domain containing 4 [Source:MGI Symbol;Acc	0.26637
ENSMUSG00000035206	Sppl2b	signal peptide peptidase like 2B [Source:MGI Symbo	0.2664
ENSMUSG00000023030	Slc11a2	solute carrier family 11 (proton-coupled divalent m	0.26651
ENSMUSG00000035572	Dcaf10	DDB1 and CUL4 associated factor 10 [Source:MGI S	0.26661
ENSMUSG00000024213	Nudt3	nudix (nucleotide diphosphate linked moiety X)-typ	0.26672
ENSMUSG00000029003	Mad2l2	MAD2 mitotic arrest deficient-like 2 [Source:MGI Sy	0.26673
ENSMUSG00000089685	Gm9017	predicted gene 9017 [Source:MGI Symbol;Acc:MGI:	0.2669
ENSMUSG00000085774	Gm13055	predicted gene 13055 [Source:MGI Symbol;Acc:MG	0.26706
ENSMUSG00000084519	Gm22478	predicted gene, 22478 [Source:MGI Symbol;Acc:MG	0.26709
ENSMUSG00000060397	Zfp128	zinc finger protein 128 [Source:MGI Symbol;Acc:MG	0.26713
ENSMUSG00000063882	Uqcrh	ubiquinol-cytochrome c reductase hinge protein [So	0.26713
ENSMUSG00000027875	Hmgcs2	3-hydroxy-3-methylglutaryl-Coenzyme A synthase 2	0.26722
ENSMUSG00000034973	Dopey1	dopey family member 1 [Source:MGI Symbol;Acc:M	0.26724
ENSMUSG00000021228	Acot3	acyl-CoA thioesterase 3 [Source:MGI Symbol;Acc:M	0.26738
ENSMUSG00000029712	Actl6b	actin-like 6B [Source:MGI Symbol;Acc:MGI:1933548	0.26741
ENSMUSG00000003062	Stard3nl	STARD3 N-terminal like [Source:MGI Symbol;Acc:M	0.26744
ENSMUSG00000027439	Gzf1	GDNF-inducible zinc finger protein 1 [Source:MGI S	0.26749
ENSMUSG00000052384	Nrros	negative regulator of reactive oxygen species [Sour	0.26752
ENSMUSG00000089736	Gm14378	predicted gene 14378 [Source:MGI Symbol;Acc:MG	0.26753
ENSMUSG00000024170	Telo2	TEL2, telomere maintenance 2, homolog (S. cerevis	0.26786
ENSMUSG00000006221	Hspb7	heat shock protein family, member 7 (cardiovascula	0.2679
ENSMUSG00000040495	Chrm4	cholinergic receptor, muscarinic 4 [Source:MGI Sym	0.26791
ENSMUSG00000035953	Tmem55b	transmembrane protein 55b [Source:MGI Symbol;A	0.26797
ENSMUSG00000021364	Elovl2	elongation of very long chain fatty acids (FEN1/Elo2	0.26799
ENSMUSG00000037447	Arid5a	AT rich interactive domain 5A (MRF1-like) [Source:M	0.26803
ENSMUSG00000040690	Col16a1	collagen, type XVI, alpha 1 [Source:MGI Symbol;Acc	0.26814
ENSMUSG00000002731	Prkra	protein kinase, interferon inducible double strande	0.26815
ENSMUSG00000029670	Ing3	inhibitor of growth family, member 3 [Source:MGI S	0.26827

ENSMUSG00000014856	Tmem208	transmembrane protein 208 [Source:MGI Symbol;A	0.2683
ENSMUSG00000048142	Nat8l	N-acetyltransferase 8-like [Source:MGI Symbol;Acc	0.26832
ENSMUSG00000039616	Mocos	molybdenum cofactor sulfurase [Source:MGI Symb	0.26846
ENSMUSG00000087053	Gm15082	predicted gene 15082 [Source:MGI Symbol;Acc:MG	0.26846
ENSMUSG00000024826	Dpf2	D4, zinc and double PHD fingers family 2 [Source:M	0.26847
ENSMUSG00000042446	Zmym4	zinc finger, MYM-type 4 [Source:MGI Symbol;Acc:M	0.26847
ENSMUSG00000057506	Bloc1s2	biogenesis of lysosome-related organelles complex	0.26849
ENSMUSG00000041000	Trim62	tripartite motif-containing 62 [Source:MGI Symbol;	0.26853
ENSMUSG00000037296	Lsm1	LSM1 homolog, U6 small nuclear RNA associated (S	0.26856
ENSMUSG00000020513	Tubd1	tubulin, delta 1 [Source:MGI Symbol;Acc:MGI:1891	0.26857
ENSMUSG00000097847	4930478K11Rik	RIKEN cDNA 4930478K11 gene [Source:MGI Symbo	0.26861
ENSMUSG00000047518	Slfnl1	schlafen like 1 [Source:MGI Symbol;Acc:MGI:30453	0.26864
ENSMUSG00000024248	Cox7a2l	cytochrome c oxidase subunit VIIa polypeptide 2-lik	0.26866
ENSMUSG00000028039	Efna3	ephrin A3 [Source:MGI Symbol;Acc:MGI:106644]	0.26868
ENSMUSG00000027895	Kcnc4	potassium voltage gated channel, Shaw-related sub	0.2688
ENSMUSG00000097684	Gm26645	predicted gene, 26645 [Source:MGI Symbol;Acc:MG	0.26896
ENSMUSG00000025792	Slc25a10	solute carrier family 25 (mitochondrial carrier, dica	0.26901
ENSMUSG00000019935	Slc17a8	solute carrier family 17 (sodium-dependent inorgar	0.26902
ENSMUSG00000028673	Fuca1	fucosidase, alpha-L- 1, tissue [Source:MGI Symbol;A	0.26904
ENSMUSG00000027715	Ccna2	cyclin A2 [Source:MGI Symbol;Acc:MGI:108069]	0.26906
ENSMUSG00000052310	Slc39a1	solute carrier family 39 (zinc transporter), member	0.26911
ENSMUSG00000086924	Gm11766	predicted gene 11766 [Source:MGI Symbol;Acc:MG	0.2692
ENSMUSG00000011008	Mcoln2	mucolipin 2 [Source:MGI Symbol;Acc:MGI:1915529	0.26924
ENSMUSG00000028176	Lrrc7	leucine rich repeat containing 7 [Source:MGI Symbo	0.2693
ENSMUSG00000040648	Ppip5k2	diphosphoinositol pentakisphosphate kinase 2 [Sou	0.26934
ENSMUSG00000086986	Gm12103	predicted gene 12103 [Source:MGI Symbol;Acc:MG	0.26949
ENSMUSG00000002372	Ranbp3	RAN binding protein 3 [Source:MGI Symbol;Acc:MG	0.26955
ENSMUSG00000037260	Hgsnat	heparan-alpha-glucosaminide N-acetyltransferase [	0.26959
ENSMUSG00000026520	Pycr2	pyrroline-5-carboxylate reductase family, member	0.26972
ENSMUSG00000085072	C920011F04Rik	RIKEN cDNA C920011F04 gene [Source:MGI Symbo	0.26977
ENSMUSG00000031756	Cenpn	centromere protein N [Source:MGI Symbol;Acc:MG	0.26982
ENSMUSG00000071202	Ccdc78	coiled-coil domain containing 78 [Source:MGI Symb	0.26982
ENSMUSG00000034083	Ccdc174	coiled-coil domain containing 174 [Source:MGI Sym	0.26984
ENSMUSG00000019146	Cacng2	calcium channel, voltage-dependent, gamma subur	0.26999
ENSMUSG00000046793	Gpr61	G protein-coupled receptor 61 [Source:MGI Symbo	0.27004
ENSMUSG00000035211	Xrra1	X-ray radiation resistance associated 1 [Source:MG	0.27026
ENSMUSG00000086211	Gm12462	predicted gene 12462 [Source:MGI Symbol;Acc:MG	0.27028
ENSMUSG00000023118	Sympk	symplekin [Source:MGI Symbol;Acc:MGI:1915438]	0.27036
ENSMUSG00000042079	Hnrnpf	heterogeneous nuclear ribonucleoprotein F [Source	0.27038
ENSMUSG00000034487	Kdelc2	KDEL (Lys-Asp-Glu-Leu) containing 2 [Source:MGI S	0.27042
ENSMUSG00000022537	Tmem44	transmembrane protein 44 [Source:MGI Symbol;Ac	0.27047
ENSMUSG00000026492	Tfb2m	transcription factor B2, mitochondrial [Source:MGI	0.2705
ENSMUSG00000081058	Hist2h3c2	histone cluster 2, H3c2 [Source:MGI Symbol;Acc:M	0.27055
ENSMUSG00000059146	Ntrk3	neurotrophic tyrosine kinase, receptor, type 3 [Sou	0.2706
ENSMUSG00000021803	Cdhr1	cadherin-related family member 1 [Source:MGI Syr	0.27061
ENSMUSG00000028036	Ptgfr	prostaglandin F receptor [Source:MGI Symbol;Acc:I	0.27064
ENSMUSG00000029501	Ankle2	ankyrin repeat and LEM domain containing 2 [Sourc	0.27076

ENSMUSG00000027559	Car3	carbonic anhydrase 3 [Source:MGI Symbol;Acc:MG	0.27082
ENSMUSG00000059475	Zfp426	zinc finger protein 426 [Source:MGI Symbol;Acc:MG	0.27087
ENSMUSG00000022426	Josd1	Josephin domain containing 1 [Source:MGI Symbol;	0.271
ENSMUSG00000038803	Ost4	oligosaccharyltransferase 4 homolog (S. cerevisiae)	0.271
ENSMUSG00000082342	Gm5395	predicted gene 5395 [Source:MGI Symbol;Acc:MGI:	0.27103
ENSMUSG00000032436	Cmtm7	CKLF-like MARVEL transmembrane domain contain	0.27106
ENSMUSG00000027454	Gins1	GINS complex subunit 1 (Psf1 homolog) [Source:MG	0.2711
ENSMUSG00000079343	Gm5077	predicted gene 5077 [Source:MGI Symbol;Acc:MGI:	0.27111
ENSMUSG00000039354	Smarcal1	SWI/SNF related matrix associated, actin dependen	0.27115
ENSMUSG00000081236	Gm13574	predicted gene 13574 [Source:MGI Symbol;Acc:MG	0.27116
ENSMUSG00000022369	Mtbp	Mdm2, transformed 3T3 cell double minute p53 bir	0.27119
ENSMUSG00000057173	Rfx8	regulatory factor X 8 [Source:MGI Symbol;Acc:MGI:	0.27125
ENSMUSG00000091243	Vgll3	vestigial like 3 (Drosophila) [Source:MGI Symbol;Ac	0.27141
ENSMUSG00000027035	Cers6	ceramide synthase 6 [Source:MGI Symbol;Acc:MGI:	0.27142
ENSMUSG00000034883	Lrr1	leucine rich repeat protein 1 [Source:MGI Symbol;A	0.27142
ENSMUSG00000097384	Gm26815	predicted gene, 26815 [Source:MGI Symbol;Acc:MG	0.27155
ENSMUSG00000035385	Ccl2	chemokine (C-C motif) ligand 2 [Source:MGI Symbo	0.27158
ENSMUSG00000060275	Nrg2	neuregulin 2 [Source:MGI Symbol;Acc:MGI:109824	0.27161
ENSMUSG00000062687	Gm6531	predicted gene 6531 [Source:MGI Symbol;Acc:MGI:	0.27165
ENSMUSG00000092641	Gm23563	predicted gene, 23563 [Source:MGI Symbol;Acc:MG	0.27181
ENSMUSG00000026421	Csrp1	cysteine and glycine-rich protein 1 [Source:MGI Syr	0.27184
ENSMUSG00000067224	Gm3695	predicted gene 3695 [Source:MGI Symbol;Acc:MGI:	0.27185
ENSMUSG00000038349	Plcl1	phospholipase C-like 1 [Source:MGI Symbol;Acc:MG	0.27191
ENSMUSG00000047502	Mroh7	maestro heat-like repeat family member 7 [Source:	0.27195
ENSMUSG00000053773	Rdh8	retinol dehydrogenase 8 [Source:MGI Symbol;Acc:M	0.27195
ENSMUSG00000027849	Syt6	synaptotagmin VI [Source:MGI Symbol;Acc:MGI:18	0.27208
ENSMUSG00000062822	4833420G17Rik	RIKEN cDNA 4833420G17 gene [Source:MGI Symbo	0.27211
ENSMUSG00000097318	1700007L15Rik	RIKEN cDNA 1700007L15 gene [Source:MGI Symbo	0.27218
ENSMUSG00000002083	Bbc3	BCL2 binding component 3 [Source:MGI Symbol;Ac	0.27219
ENSMUSG00000020086	H2afy2	H2A histone family, member Y2 [Source:MGI Symb	0.27232
ENSMUSG00000001847	Rac1	RAS-related C3 botulinum substrate 1 [Source:MGI	0.27234
ENSMUSG00000035653	Lrfn5	leucine rich repeat and fibronectin type III domain	0.27254
ENSMUSG00000079293	Clec7a	C-type lectin domain family 7, member a [Source:M	0.27255
ENSMUSG00000043257	Pigv	phosphatidylinositol glycan anchor biosynthesis, cla	0.27277
ENSMUSG00000070717	Gm10300	predicted gene 10300 [Source:MGI Symbol;Acc:MG	0.2728
ENSMUSG00000029060	Mib2	mindbomb homolog 2 (Drosophila) [Source:MGI Sy	0.27287
ENSMUSG00000046747	Gm9812	predicted gene 9812 [Source:MGI Symbol;Acc:MGI:	0.27287
ENSMUSG00000028266	Lmo4	LIM domain only 4 [Source:MGI Symbol;Acc:MGI:1	0.27302
ENSMUSG00000020707	Rnf135	ring finger protein 135 [Source:MGI Symbol;Acc:MG	0.27307
ENSMUSG00000024300	Myo1f	myosin IF [Source:MGI Symbol;Acc:MGI:107711]	0.27307
ENSMUSG00000025404	R3hdm2	R3H domain containing 2 [Source:MGI Symbol;Acc:	0.27312
ENSMUSG00000046959	Slc26a1	solute carrier family 26 (sulfate transporter), memb	0.27314
ENSMUSG00000071757	Zhx2	zinc fingers and homeoboxes 2 [Source:MGI Symbo	0.2732
ENSMUSG00000001082	Mfsd10	major facilitator superfamily domain containing 10	0.27329
ENSMUSG00000039179	Tekt5	tektin 5 [Source:MGI Symbol;Acc:MGI:1917676]	0.27329
ENSMUSG00000073144	4930599N23Rik	RIKEN cDNA 4930599N23 gene [Source:MGI Symbo	0.2733
ENSMUSG00000084755	Gm16276	predicted gene 16276 [Source:MGI Symbol;Acc:MG	0.27332

ENSMUSG00000027223	Mapk8ip1	mitogen-activated protein kinase 8 interacting prot	0.27333
ENSMUSG00000067924	Cxx1b	CAAX box 1B [Source:MGI Symbol;Acc:MGI:357650	0.27334
ENSMUSG00000055022	Cntn1	contactin 1 [Source:MGI Symbol;Acc:MGI:105980]	0.27337
ENSMUSG00000069223	4930451E10Rik	RIKEN cDNA 4930451E10 gene [Source:MGI Symbo	0.27337
ENSMUSG00000023224	Serping1	serine (or cysteine) peptidase inhibitor, clade G, me	0.2734
ENSMUSG00000022100	Xpo7	exportin 7 [Source:MGI Symbol;Acc:MGI:1929705]	0.27354
ENSMUSG00000032679	Cd59a	CD59a antigen [Source:MGI Symbol;Acc:MGI:10917	0.27361
ENSMUSG00000028465	Tln1	talin 1 [Source:MGI Symbol;Acc:MGI:1099832]	0.27382
ENSMUSG00000089636	1700058P15Rik	RIKEN cDNA 1700058P15 gene [Source:MGI Symbo	0.27398
ENSMUSG00000059409	Ppp2r5d	protein phosphatase 2, regulatory subunit B (B56),	0.27405
ENSMUSG00000079428	Tceal7	transcription elongation factor A (SII)-like 7 [Source	0.27406
ENSMUSG00000022999	Lmbr1l	limb region 1 like [Source:MGI Symbol;Acc:MGI:128	0.27408
ENSMUSG00000097428	AW047730	expressed sequence AW047730 [Source:MGI Symb	0.27411
ENSMUSG00000096330	Gm21976	predicted gene 21976 [Source:MGI Symbol;Acc:MG	0.27423
ENSMUSG00000030134	Rasgef1a	RasGEF domain family, member 1A [Source:MGI Sy	0.27426
ENSMUSG00000008028	1700008O03Rik	RIKEN cDNA 1700008O03 gene [Source:MGI Symbo	0.27429
ENSMUSG00000024212	Mllt1	myeloid/lymphoid or mixed-lineage leukemia (trith	0.27436
ENSMUSG00000019769	Syne1	spectrin repeat containing, nuclear envelope 1 [Sou	0.27443
ENSMUSG00000086103	Gm11832	predicted gene 11832 [Source:MGI Symbol;Acc:MG	0.27453
ENSMUSG00000078091	Gm10912	predicted gene 10912 [Source:MGI Symbol;Acc:MG	0.27454
ENSMUSG00000025905	Oprk1	opioid receptor, kappa 1 [Source:MGI Symbol;Acc:M	0.27463
ENSMUSG00000048022	Tmem229a	transmembrane protein 229A [Source:MGI Symbol;	0.27463
ENSMUSG00000021811	Dnajc9	DnaJ (Hsp40) homolog, subfamily C, member 9 [Sou	0.27473
ENSMUSG00000003534	Ddr1	discoidin domain receptor family, member 1 [Sourc	0.27476
ENSMUSG00000049661	Mtag2	metastasis associated gene 2 [Source:MGI Symbol;	0.27477
ENSMUSG00000051355	Commd1	COMM domain containing 1 [Source:MGI Symbol;A	0.27494
ENSMUSG00000030718	Ppme1	protein phosphatase methylesterase 1 [Source:MG	0.27495
ENSMUSG00000048222	Mfap1b	microfibrillar-associated protein 1B [Source:MGI Sy	0.275
ENSMUSG00000003154	Foxj2	forkhead box J2 [Source:MGI Symbol;Acc:MGI:1926	0.27508
ENSMUSG00000087435	Gm16323	predicted gene 16323 [Source:MGI Symbol;Acc:MG	0.27509
ENSMUSG00000053119	Chmp3	charged multivesicular body protein 3 [Source:MGI	0.27514
ENSMUSG00000027427	Polr3f	polymerase (RNA) III (DNA directed) polypeptide F	0.27516
ENSMUSG00000020262	Adarb1	adenosine deaminase, RNA-specific, B1 [Source:MG	0.27517
ENSMUSG00000033365	Ipo13	importin 13 [Source:MGI Symbol;Acc:MGI:2385205	0.27521
ENSMUSG00000024968	Rcor2	REST corepressor 2 [Source:MGI Symbol;Acc:MGI:1	0.27541
ENSMUSG00000026976	Pax8	paired box gene 8 [Source:MGI Symbol;Acc:MGI:97	0.27543
ENSMUSG00000087885	Gm23600	predicted gene, 23600 [Source:MGI Symbol;Acc:MG	0.27543
ENSMUSG00000022892	App	amyloid beta (A4) precursor protein [Source:MGI Sy	0.27544
ENSMUSG00000028898	Trnau1ap	tRNA selenocysteine 1 associated protein 1 [Source	0.27545
ENSMUSG00000078864	Gm14322	predicted gene 14322 [Source:MGI Symbol;Acc:MG	0.27551
ENSMUSG00000028736	Pax7	paired box gene 7 [Source:MGI Symbol;Acc:MGI:97	0.27553
ENSMUSG00000030588	Yif1b	Yip1 interacting factor homolog B (S. cerevisiae) [So	0.27562
ENSMUSG00000022751	Nit2	nitrilase family, member 2 [Source:MGI Symbol;Acc	0.27567
ENSMUSG00000027317	Ppp1r14d	protein phosphatase 1, regulatory (inhibitor) subun	0.27568
ENSMUSG00000096255	Dynlt1b	dynein light chain Tctex-type 1B [Source:MGI Symb	0.27569
ENSMUSG00000040771	Oard1	O-acyl-ADP-ribose deacylase 1 [Source:MGI Symbo	0.27571
ENSMUSG00000028656	Cap1	CAP, adenylate cyclase-associated protein 1 (yeast)	0.2758

ENSMUSG00000063511	Snrnp70	small nuclear ribonucleoprotein 70 (U1) [Source:M	0.27587
ENSMUSG00000050604	3110021A11Rik	RIKEN cDNA 3110021A11 gene [Source:MGI Symbo	0.27593
ENSMUSG00000039646	Vasn	vasorin [Source:MGI Symbol;Acc:MGI:2177651]	0.27596
ENSMUSG00000035629	1700021K19Rik	RIKEN cDNA 1700021K19 gene [Source:MGI Symbo	0.27597
ENSMUSG00000024521	Pmaip1	phorbol-12-myristate-13-acetate-induced protein 1	0.27601
ENSMUSG00000029547	Ints1	integrator complex subunit 1 [Source:MGI Symbol;	0.27603
ENSMUSG00000021852	Slc35f4	solute carrier family 35, member F4 [Source:MGI Sy	0.27614
ENSMUSG00000003316	Glg1	golgi apparatus protein 1 [Source:MGI Symbol;Acc:	0.27617
ENSMUSG00000027641	Rbl1	retinoblastoma-like 1 (p107) [Source:MGI Symbol;A	0.27622
ENSMUSG00000034022	Cpsf1	cleavage and polyadenylation specific factor 1 [Sou	0.27623
ENSMUSG00000039483	Asb6	ankyrin repeat and SOCS box-containing 6 [Source:	0.27624
ENSMUSG00000097686	Gm26647	predicted gene, 26647 [Source:MGI Symbol;Acc:M	0.27637
ENSMUSG00000078771	Evi2a	ecotropic viral integration site 2a [Source:MGI Sym	0.27646
ENSMUSG00000046994	Mars2	methionine-tRNA synthetase 2 (mitochondrial) [Sou	0.27651
ENSMUSG00000038291	Snx25	sorting nexin 25 [Source:MGI Symbol;Acc:MGI:214	0.27655
ENSMUSG00000062127	Cttnbp2nl	CTTNBP2 N-terminal like [Source:MGI Symbol;Acc:	0.27661
ENSMUSG00000028914	Casp9	caspase 9 [Source:MGI Symbol;Acc:MGI:1277950]	0.27673
ENSMUSG00000093080	Mir3060	microRNA 3060 [Source:MGI Symbol;Acc:MGI:4834	0.27679
ENSMUSG00000057003	Myh4	myosin, heavy polypeptide 4, skeletal muscle [Sour	0.27684
ENSMUSG00000037214	Thap1	THAP domain containing, apoptosis associated prot	0.2769
ENSMUSG00000035201	B020004J07Rik	RIKEN cDNA B020004J07 gene [Source:MGI Symbol	0.27712
ENSMUSG00000079511	Ccdc142	coiled-coil domain containing 142 [Source:MGI Sym	0.27724
ENSMUSG00000031834	Pik3r2	phosphatidylinositol 3-kinase, regulatory subunit, p	0.27726
ENSMUSG00000033352	Map2k4	mitogen-activated protein kinase kinase 4 [Source:	0.27731
ENSMUSG00000030545	Pex11a	peroxisomal biogenesis factor 11 alpha [Source:MG	0.27733
ENSMUSG00000022354	Ndufb9	NADH dehydrogenase (ubiquinone) 1 beta subcom	0.27739
ENSMUSG00000059213	Ddn	dendrin [Source:MGI Symbol;Acc:MGI:108101]	0.27743
ENSMUSG00000020191	4930415F15Rik	RIKEN cDNA 4930415F15 gene [Source:MGI Symbo	0.2776
ENSMUSG00000084244	Gm14020	predicted gene 14020 [Source:MGI Symbol;Acc:MG	0.2776
ENSMUSG00000017561	Crlf3	cytokine receptor-like factor 3 [Source:MGI Symbol	0.2777
ENSMUSG00000091476	Gm16432	predicted gene 16432 [Source:MGI Symbol;Acc:MG	0.27775
ENSMUSG00000028937	Acot7	acyl-CoA thioesterase 7 [Source:MGI Symbol;Acc:M	0.27783
ENSMUSG00000024376	Epb4.1l4a	erythrocyte protein band 4.1-like 4a [Source:MGI S	0.27785
ENSMUSG00000003153	Slc2a3	solute carrier family 2 (facilitated glucose transport	0.27789
ENSMUSG00000053141	Ptprt	protein tyrosine phosphatase, receptor type, T [Sou	0.27791
ENSMUSG00000027568	Ntsr1	neurotensin receptor 1 [Source:MGI Symbol;Acc:M	0.27798
ENSMUSG00000039637	Coro7	coronin 7 [Source:MGI Symbol;Acc:MGI:1926135]	0.27801
ENSMUSG00000020182	Ddc	dopa decarboxylase [Source:MGI Symbol;Acc:MGI:	0.27804
ENSMUSG00000030849	Fgfr2	fibroblast growth factor receptor 2 [Source:MGI Sy	0.27814
ENSMUSG00000023007	Prpf40b	PRP40 pre-mRNA processing factor 40 homolog B (	0.27815
ENSMUSG00000026425	Srgap2	SLIT-ROBO Rho GTPase activating protein 2 [Source	0.27817
ENSMUSG00000029500	Pgam5	phosphoglycerate mutase family member 5 [Source	0.27817
ENSMUSG00000047766	Lrrc49	leucine rich repeat containing 49 [Source:MGI Sym	0.27823
ENSMUSG00000029392	Rilpl1	Rab interacting lysosomal protein-like 1 [Source:M	0.27827
ENSMUSG00000031158	Timm17b	translocase of inner mitochondrial membrane 17b	0.27828
ENSMUSG00000019368	Sec14l4	SEC14-like 4 (S. cerevisiae) [Source:MGI Symbol;Ac	0.2783
ENSMUSG00000078234	Klhdc7a	kelch domain containing 7A [Source:MGI Symbol;A	0.27848

ENSMUSG00000051351	Zfp46	zinc finger protein 46 [Source:MGI Symbol;Acc:MGI	0.2785
ENSMUSG00000069303	Hist1h2br	histone cluster 1 H2br [Source:MGI Symbol;Acc:MG	0.27857
ENSMUSG00000082292	Gm12250	predicted gene 12250 [Source:MGI Symbol;Acc:MG	0.27866
ENSMUSG00000047284	Neurl4	neuralized homolog 4 (Drosophila) [Source:MGI Syr	0.27873
ENSMUSG00000066705	Fxyd6	FXYD domain-containing ion transport regulator 6 [	0.27876
ENSMUSG00000081265	Gm11282	predicted gene 11282 [Source:MGI Symbol;Acc:MG	0.27879
ENSMUSG00000037907	Ankrd13b	ankyrin repeat domain 13b [Source:MGI Symbol;Ac	0.27888
ENSMUSG00000079598	Clec2l	C-type lectin domain family 2, member L [Source:M	0.27889
ENSMUSG00000004100	Ppan	peter pan homolog (Drosophila) [Source:MGI Symb	0.27892
ENSMUSG00000052752	Traf7	TNF receptor-associated factor 7 [Source:MGI Symb	0.27893
ENSMUSG00000034190	Chmp7	charged multivesicular body protein 7 [Source:MGI	0.27896
ENSMUSG00000073184	Gm10479	predicted gene 10479 [Source:MGI Symbol;Acc:MG	0.27905
ENSMUSG00000038224	Serpinf2	serine (or cysteine) peptidase inhibitor, clade F, me	0.2791
ENSMUSG00000028354	Fmn2	formin 2 [Source:MGI Symbol;Acc:MGI:1859252]	0.27919
ENSMUSG00000027367	Stard7	START domain containing 7 [Source:MGI Symbol;Ac	0.27925
ENSMUSG00000030032	Wdr54	WD repeat domain 54 [Source:MGI Symbol;Acc:MG	0.27929
ENSMUSG00000039509	Nup133	nucleoporin 133 [Source:MGI Symbol;Acc:MGI:244	0.27929
ENSMUSG00000033253	Szt2	seizure threshold 2 [Source:MGI Symbol;Acc:MGI:3	0.27934
ENSMUSG00000072324	Gm8420	predicted gene 8420 [Source:MGI Symbol;Acc:MGI:	0.27935
ENSMUSG00000023353	Agap3	ArfGAP with GTPase domain, ankyrin repeat and PH	0.27954
ENSMUSG00000067150	Xpo5	exportin 5 [Source:MGI Symbol;Acc:MGI:1913789]	0.27955
ENSMUSG00000072624	Gm5460	predicted gene 5460 [Source:MGI Symbol;Acc:MGI:	0.27956
ENSMUSG00000067995	Gtf2f2	general transcription factor IIF, polypeptide 2 [Sour	0.2797
ENSMUSG00000087242	C78197	expressed sequence C78197 [Source:MGI Symbol;A	0.27972
ENSMUSG00000071715	Ncf4	neutrophil cytosolic factor 4 [Source:MGI Symbol;A	0.27973
ENSMUSG00000022765	Snap29	synaptosomal-associated protein 29 [Source:MGI S	0.27974
ENSMUSG00000095325	Zfp870	zinc finger protein 870 [Source:MGI Symbol;Acc:MG	0.27984
ENSMUSG00000096257	Gm6537	predicted gene 6537 [Source:MGI Symbol;Acc:MGI:	0.27985
ENSMUSG00000045348	Nyap1	neuronal tyrosine-phosphorylated phosphoinositid	0.27988
ENSMUSG00000086137	Gm16248	predicted gene 16248 [Source:MGI Symbol;Acc:MG	0.28002
ENSMUSG00000049871	Nlrc3	NLR family, CARD domain containing 3 [Source:MG	0.28007
ENSMUSG00000038354	Ankrd35	ankyrin repeat domain 35 [Source:MGI Symbol;Acc	0.28016
ENSMUSG00000070953	Rabepk	Rab9 effector protein with kelch motifs [Source:MG	0.2802
ENSMUSG00000074748	Atxn7l3b	ataxin 7-like 3B [Source:MGI Symbol;Acc:MGI:1914	0.28021
ENSMUSG00000034773	BC030867	cDNA sequence BC030867 [Source:MGI Symbol;Acc	0.28024
ENSMUSG00000030008	Pradc1	protease-associated domain containing 1 [Source:M	0.28026
ENSMUSG00000007033	Hspa1l	heat shock protein 1-like [Source:MGI Symbol;Acc:I	0.28045
ENSMUSG00000021240	Abcd4	ATP-binding cassette, sub-family D (ALD), member	0.28046
ENSMUSG00000088712	Gm22728	predicted gene, 22728 [Source:MGI Symbol;Acc:MG	0.28051
ENSMUSG00000093413	Snora15	small nucleolar RNA, H/ACA box 15 [Source:MGI Sy	0.28055
ENSMUSG00000032018	Sc5d	sterol-C5-desaturase (fungal ERG3, delta-5-desatur	0.28059
ENSMUSG00000006270	Vax1	ventral anterior homeobox containing gene 1 [Sour	0.2806
ENSMUSG00000026510	Trp53bp2	transformation related protein 53 binding protein 2	0.28066
ENSMUSG00000020712	Tcam1	testicular cell adhesion molecule 1 [Source:MGI Syr	0.28073
ENSMUSG00000009090	Ap1b1	adaptor protein complex AP-1, beta 1 subunit [Sour	0.28085
ENSMUSG00000010044	Zmynd10	zinc finger, MYND domain containing 10 [Source:M	0.28088
ENSMUSG00000078779	Zfp59	zinc finger protein 59 [Source:MGI Symbol;Acc:MGI	0.28096

ENSMUSG00000049287	Iba57	IBA57, iron-sulfur cluster assembly homolog (S. cer	0.28141
ENSMUSG00000077637	Gm22771	predicted gene, 22771 [Source:MGI Symbol;Acc:M(	0.28148
ENSMUSG00000091166	Tstd1	thiosulfate sulfurtransferase (rhodanese)-like doma	0.28156
ENSMUSG00000097626	4921504A21Rik	RIKEN cDNA 4921504A21 gene [Source:MGI Symbo	0.28161
ENSMUSG00000069300	Hist1h2bj	histone cluster 1, H2bj [Source:MGI Symbol;Acc:M(	0.28164
ENSMUSG00000032184	Lysmd2	LysM, putative peptidoglycan-binding, domain cont	0.28173
ENSMUSG00000026727	Rsu1	Ras suppressor protein 1 [Source:MGI Symbol;Acc:I	0.28176
ENSMUSG00000038583	Pln	phospholamban [Source:MGI Symbol;Acc:MGI:976	0.28178
ENSMUSG00000039620	6430573F11Rik	RIKEN cDNA 6430573F11 gene [Source:MGI Symbo	0.28179
ENSMUSG00000036403	Cep135	centrosomal protein 135 [Source:MGI Symbol;Acc:I	0.28181
ENSMUSG00000022272	Myo10	myosin X [Source:MGI Symbol;Acc:MGI:107716]	0.28184
ENSMUSG00000090599	Gm20731	predicted gene, 20731 [Source:MGI Symbol;Acc:M(	0.28188
ENSMUSG00000038143	Stox2	storkhead box 2 [Source:MGI Symbol;Acc:MGI:1918	0.28197
ENSMUSG00000085984	1700001G11Rik	RIKEN cDNA 1700001G11 gene [Source:MGI Symbo	0.28197
ENSMUSG00000029344	Tpst2	protein-tyrosine sulfotransferase 2 [Source:MGI Sy	0.28198
ENSMUSG00000006024	Napa	N-ethylmaleimide sensitive fusion protein attachm	0.28205
ENSMUSG00000087542	Gm15878	predicted gene 15878 [Source:MGI Symbol;Acc:MG	0.28207
ENSMUSG00000022678	Nde1	nuclear distribution gene E homolog 1 (A nidulans)	0.28208
ENSMUSG00000058239	Usf2	upstream transcription factor 2 [Source:MGI Symbo	0.28212
ENSMUSG00000032434	Cmtm6	CKLF-like MARVEL transmembrane domain contain	0.28216
ENSMUSG00000078612	1700024P16Rik	RIKEN cDNA 1700024P16 gene [Source:MGI Symbo	0.28219
ENSMUSG00000044550	Tceal3	transcription elongation factor A (SII)-like 3 [Source	0.28224
ENSMUSG00000042745	Id1	inhibitor of DNA binding 1 [Source:MGI Symbol;Acc	0.28227
ENSMUSG00000041229	Phf8	PHD finger protein 8 [Source:MGI Symbol;Acc:MGI:	0.28235
ENSMUSG00000088947	Gm23257	predicted gene, 23257 [Source:MGI Symbol;Acc:M(	0.28235
ENSMUSG00000057058	Skap1	src family associated phosphoprotein 1 [Source:MG	0.28236
ENSMUSG00000018326	Ywhab	tyrosine 3-monooxygenase/tryptophan 5-monooxy	0.28237
ENSMUSG00000030298	Sec13	SEC13 homolog (S. cerevisiae) [Source:MGI Symbol	0.28241
ENSMUSG00000077440	Gm23130	predicted gene, 23130 [Source:MGI Symbol;Acc:M(	0.28247
ENSMUSG00000035403	Crb2	crumbs homolog 2 (Drosophila) [Source:MGI Symb	0.28255
ENSMUSG00000097690	4930505N22Rik	RIKEN cDNA 4930505N22 gene [Source:MGI Symbo	0.2826
ENSMUSG00000087141	Plcxd2	phosphatidylinositol-specific phospholipase C, X do	0.28263
ENSMUSG00000030226	Lmo3	LIM domain only 3 [Source:MGI Symbol;Acc:MGI:1(	0.28265
ENSMUSG00000030138	Bms1	BMS1 homolog, ribosome assembly protein (yeast)	0.28271
ENSMUSG00000051736	Fam229b	family with sequence similarity 229, member B [Sou	0.28273
ENSMUSG00000030862	Cpxm2	carboxypeptidase X 2 (M14 family) [Source:MGI Sy	0.28277
ENSMUSG00000032217	Rnf111	ring finger 111 [Source:MGI Symbol;Acc:MGI:19349	0.28285
ENSMUSG00000024026	Glo1	glyoxalase 1 [Source:MGI Symbol;Acc:MGI:95742]	0.28286
ENSMUSG00000004366	Sst	somatostatin [Source:MGI Symbol;Acc:MGI:98326]	0.28292
ENSMUSG00000044938	Klhl31	kelch-like 31 [Source:MGI Symbol;Acc:MGI:304530	0.28292
ENSMUSG00000005483	Dnajb1	DnaJ (Hsp40) homolog, subfamily B, member 1 [Sou	0.28298
ENSMUSG00000089672	Gp49a	glycoprotein 49 A [Source:MGI Symbol;Acc:MGI:10	0.28298
ENSMUSG00000097587	4930578M01Rik	RIKEN cDNA 4930578M01 gene [Source:MGI Symb	0.28302
ENSMUSG00000018378	Cuedc1	CUE domain containing 1 [Source:MGI Symbol;Acc:	0.28303
ENSMUSG00000047030	Spata2	spermatogenesis associated 2 [Source:MGI Symbol	0.28308
ENSMUSG00000032368	Zic1	zinc finger protein of the cerebellum 1 [Source:MG	0.28317
ENSMUSG00000074925	Ptar1	protein prenyltransferase alpha subunit repeat con	0.28331

ENSMUSG00000086838	Gm16212	predicted gene 16212 [Source:MGI Symbol;Acc:MG	0.28338
ENSMUSG00000086953	4933437N03Rik	RIKEN cDNA 4933437N03 gene [Source:MGI Symbo	0.2834
ENSMUSG00000037106	Fer1l6	fer-1-like 6 (C. elegans) [Source:MGI Symbol;Acc:M	0.28358
ENSMUSG00000071262	Zfp957	zinc finger protein 957 [Source:MGI Symbol;Acc:M	0.28376
ENSMUSG00000015980	Lrrc27	leucine rich repeat containing 27 [Source:MGI Sym	0.28386
ENSMUSG00000042485	Mustn1	musculoskeletal, embryonic nuclear protein 1 [Sou	0.28387
ENSMUSG00000037761	Actr5	ARP5 actin-related protein 5 [Source:MGI Symbol;A	0.28395
ENSMUSG00000058925	1700011I03Rik	RIKEN cDNA 1700011I03 gene [Source:MGI Symbol	0.28406
ENSMUSG00000003812	Dnase2a	deoxyribonuclease II alpha [Source:MGI Symbol;Ac	0.28422
ENSMUSG00000032807	Alox12b	arachidonate 12-lipoxygenase, 12R type [Source:M	0.28425
ENSMUSG00000052353	9930013L23Rik	RIKEN cDNA 9930013L23 gene [Source:MGI Symbo	0.28447
ENSMUSG00000036442	Thap11	THAP domain containing 11 [Source:MGI Symbol;A	0.28449
ENSMUSG00000030037	Mrpl53	mitochondrial ribosomal protein L53 [Source:MGI S	0.28452
ENSMUSG00000097732	D930048G16Rik	RIKEN cDNA D930048G16 gene [Source:MGI Symbo	0.28452
ENSMUSG00000014850	Msh3	mutS homolog 3 (E. coli) [Source:MGI Symbol;Acc:M	0.28454
ENSMUSG00000046722	Cdc42se1	CDC42 small effector 1 [Source:MGI Symbol;Acc:M	0.28457
ENSMUSG00000055024	Ep300	E1A binding protein p300 [Source:MGI Symbol;Acc:	0.28458
ENSMUSG00000074489	Bglap3	bone gamma-carboxyglutamate protein 3 [Source:M	0.28459
ENSMUSG00000037351	Actr1b	ARP1 actin-related protein 1B, centractin beta [Sou	0.28463
ENSMUSG00000031864	Ints10	integrator complex subunit 10 [Source:MGI Symbol	0.28465
ENSMUSG00000022263	Trio	triple functional domain (PTPRF interacting) [Sourc	0.28478
ENSMUSG00000063146	Clip2	CAP-GLY domain containing linker protein 2 [Sourc	0.2848
ENSMUSG00000020454	Eif4enif1	eukaryotic translation initiation factor 4E nuclear in	0.28485
ENSMUSG00000010406	Mrpl52	mitochondrial ribosomal protein L52 [Source:MGI S	0.28499
ENSMUSG00000029502	Golga3	golgi autoantigen, golgin subfamily a, 3 [Source:MG	0.28502
ENSMUSG00000034993	Vat1	vesicle amine transport protein 1 homolog (T califo	0.28511
ENSMUSG00000075528	Aarsd1	alanyl-tRNA synthetase domain containing 1 [Sourc	0.28517
ENSMUSG00000090305	Gm5459	predicted gene 5459 [Source:MGI Symbol;Acc:MGI:	0.28528
ENSMUSG00000038520	Tbc1d17	TBC1 domain family, member 17 [Source:MGI Symb	0.28531
ENSMUSG00000031931	Ankrd49	ankyrin repeat domain 49 [Source:MGI Symbol;Acc	0.28533
ENSMUSG00000073867	AA474408	expressed sequence AA474408 [Source:MGI Symbo	0.28535
ENSMUSG00000032549	Rab6b	RAB6B, member RAS oncogene family [Source:MGI	0.28536
ENSMUSG00000006585	Cdt1	chromatin licensing and DNA replication factor 1 [S	0.28537
ENSMUSG00000034917	Tjp3	tight junction protein 3 [Source:MGI Symbol;Acc:M	0.28552
ENSMUSG00000058290	Espl1	extra spindle poles-like 1 (S. cerevisiae) [Source:MG	0.28562
ENSMUSG00000081494	Gm14130	predicted gene 14130 [Source:MGI Symbol;Acc:MG	0.28562
ENSMUSG00000074366	Obox5	oocyte specific homeobox 5 [Source:MGI Symbol;A	0.28567
ENSMUSG00000066621	Tecpr1	tectonin beta-propeller repeat containing 1 [Source	0.28571
ENSMUSG00000021871	Pnp	purine-nucleoside phosphorylase [Source:MGI Sym	0.28573
ENSMUSG00000028811	Yars	tyrosyl-tRNA synthetase [Source:MGI Symbol;Acc:N	0.28588
ENSMUSG00000035429	Ptprh	protein tyrosine phosphatase, receptor type, H [Sou	0.28595
ENSMUSG00000078160	Gm16503	predicted gene 16503 [Source:MGI Symbol;Acc:MG	0.28599
ENSMUSG00000025794	Rpl14	ribosomal protein L14 [Source:MGI Symbol;Acc:MG	0.28602
ENSMUSG00000093806	Asmt	acetylserotonin O-methyltransferase [Source:MGI S	0.28605
ENSMUSG00000083689	Gm14094	predicted gene 14094 [Source:MGI Symbol;Acc:MG	0.28607
ENSMUSG00000095061	E030018B13Rik	RIKEN cDNA E030018B13 gene [Source:MGI Symbo	0.28607
ENSMUSG00000072621	Slfn10-ps	schlafen 10, pseudogene [Source:MGI Symbol;Acc:I	0.28609

ENSMUSG00000078061	Gm22824	predicted gene, 22824 [Source:MGI Symbol;Acc:MG	0.28609
ENSMUSG00000085838	Gm13706	predicted gene 13706 [Source:MGI Symbol;Acc:MG	0.28611
ENSMUSG00000057534	Gm15698	predicted gene 15698 [Source:MGI Symbol;Acc:MG	0.28612
ENSMUSG00000047257	Prss45	protease, serine, 45 [Source:MGI Symbol;Acc:MGI:3	0.28613
ENSMUSG00000020780	Srp68	signal recognition particle 68 [Source:MGI Symbol;A	0.28615
ENSMUSG00000024639	Gnaq	guanine nucleotide binding protein, alpha q polype	0.28619
ENSMUSG00000085498	Gm14023	predicted gene 14023 [Source:MGI Symbol;Acc:MG	0.28621
ENSMUSG00000036986	Pml	promyelocytic leukemia [Source:MGI Symbol;Acc:M	0.28639
ENSMUSG00000097347	Gm17275	predicted gene, 17275 [Source:MGI Symbol;Acc:MG	0.28645
ENSMUSG00000062808	Hist1h3d	histone cluster 1, H3d [Source:MGI Symbol;Acc:MG	0.28646
ENSMUSG00000039461	Tcta	T cell leukemia translocation altered gene [Source:I	0.28649
ENSMUSG00000020068	Pbld2	phenazine biosynthesis-like protein domain contair	0.2865
ENSMUSG00000045875	Adra1a	adrenergic receptor, alpha 1a [Source:MGI Symbol;	0.28654
ENSMUSG00000051236	Msrb3	methionine sulfoxide reductase B3 [Source:MGI Sy	0.28667
ENSMUSG00000033991	Ttc37	tetratricopeptide repeat domain 37 [Source:MGI Sy	0.28668
ENSMUSG00000015843	Rxrg	retinoid X receptor gamma [Source:MGI Symbol;Ac	0.28674
ENSMUSG00000001785	Pwp1	PWP1 homolog (S. cerevisiae) [Source:MGI Symbol;	0.28675
ENSMUSG00000031538	Plat	plasminogen activator, tissue [Source:MGI Symbol;	0.28695
ENSMUSG00000049687	Fam109b	family with sequence similarity 109, member B [Sou	0.2871
ENSMUSG00000036893	Ehmt1	euchromatic histone methyltransferase 1 [Source:M	0.28713
ENSMUSG00000093074	Gm25949	predicted gene, 25949 [Source:MGI Symbol;Acc:MG	0.2872
ENSMUSG00000054728	Phactr1	phosphatase and actin regulator 1 [Source:MGI Syn	0.28725
ENSMUSG00000003660	Snrnp200	small nuclear ribonucleoprotein 200 (U5) [Source:M	0.28731
ENSMUSG00000085315	A430018G15Rik	RIKEN cDNA A430018G15 gene [Source:MGI Symbo	0.2874
ENSMUSG00000007944	Ttc9b	tetratricopeptide repeat domain 9B [Source:MGI Sy	0.28741
ENSMUSG00000022816	Fstl1	follistatin-like 1 [Source:MGI Symbol;Acc:MGI:1027	0.28762
ENSMUSG00000002835	Chaf1a	chromatin assembly factor 1, subunit A (p150) [Sou	0.28764
ENSMUSG00000036817	Sun1	Sad1 and UNC84 domain containing 1 [Source:MGI	0.28768
ENSMUSG00000031966	Glb1l3	galactosidase, beta 1 like 3 [Source:MGI Symbol;Ac	0.28771
ENSMUSG00000049036	Tmem121	transmembrane protein 121 [Source:MGI Symbol;A	0.28775
ENSMUSG00000088025	Rprl3	ribonuclease P RNA-like 3 [Source:MGI Symbol;Acc	0.28777
ENSMUSG00000032305	Fam219b	family with sequence similarity 219, member B [Sou	0.28778
ENSMUSG00000042156	Dzip1	DAZ interacting protein 1 [Source:MGI Symbol;Acc:	0.28779
ENSMUSG00000046056	Sbsn	suprabasin [Source:MGI Symbol;Acc:MGI:2446326]	0.28783
ENSMUSG00000097195	Snhg5	small nucleolar RNA host gene 5 [Source:MGI Symb	0.28794
ENSMUSG00000022779	Top3b	topoisomerase (DNA) III beta [Source:MGI Symbol;A	0.28805
ENSMUSG00000007216	Zfp775	zinc finger protein 775 [Source:MGI Symbol;Acc:MG	0.28818
ENSMUSG00000027827	Kcnab1	potassium voltage-gated channel, shaker-related su	0.28824
ENSMUSG00000036779	Papd5	PAP associated domain containing 5 [Source:MGI Sy	0.28837
ENSMUSG00000093749	4921523L03Rik	RIKEN cDNA 4921523L03 gene [Source:MGI Symbo	0.28842
ENSMUSG00000034329	Brip1	BRCA1 interacting protein C-terminal helicase 1 [So	0.28851
ENSMUSG00000057103	Cml1	camello-like 1 [Source:MGI Symbol;Acc:MGI:19133	0.28851
ENSMUSG00000007610	Gtpbp3	GTP binding protein 3 [Source:MGI Symbol;Acc:MG	0.2886
ENSMUSG00000024816	Frmd8	FERM domain containing 8 [Source:MGI Symbol;Ac	0.28867
ENSMUSG00000040616	Tmem51	transmembrane protein 51 [Source:MGI Symbol;Ac	0.28869
ENSMUSG00000020894	Vamp2	vesicle-associated membrane protein 2 [Source:MG	0.28881
ENSMUSG00000018909	Arrb1	arrestin, beta 1 [Source:MGI Symbol;Acc:MGI:9947	0.28885

ENSMUSG00000037348	Paqr7	progestin and adipoQ receptor family member VII [	0.28886
ENSMUSG00000031967	Afg3l1	AFG3(ATPase family gene 3)-like 1 (yeast) [Source:N	0.28893
ENSMUSG00000030838	Ush1c	Usher syndrome 1C [Source:MGI Symbol;Acc:MGI:1	0.28898
ENSMUSG00000093073	Mir124a-2	microRNA 124a-2 [Source:MGI Symbol;Acc:MGI:36	0.28898
ENSMUSG00000058761	Rnf169	ring finger protein 169 [Source:MGI Symbol;Acc:MG	0.28902
ENSMUSG00000030726	Pold3	polymerase (DNA-directed), delta 3, accessory subu	0.28904
ENSMUSG00000047905	Gm8566	predicted pseudogene 8566 [Source:MGI Symbol;A	0.28908
ENSMUSG00000027187	Cat	catalase [Source:MGI Symbol;Acc:MGI:88271]	0.28919
ENSMUSG00000071073	Lrrc73	leucine rich repeat containing 73 [Source:MGI Syml	0.28921
ENSMUSG00000061062	Gm10093	predicted pseudogene 10093 [Source:MGI Symbol;	0.28923
ENSMUSG00000038765	Lmx1b	LIM homeobox transcription factor 1 beta [Source:	0.28924
ENSMUSG00000033955	Tnks1bp1	tankyrase 1 binding protein 1 [Source:MGI Symbol;	0.28929
ENSMUSG00000037169	Mycn	v-myc myelocytomatosis viral related oncogene, ne	0.2893
ENSMUSG00000046093	Hpcal4	hippocalcin-like 4 [Source:MGI Symbol;Acc:MGI:21	0.28935
ENSMUSG00000057667	Bloc1s3	biogenesis of lysosome-related organelles complex	0.28942
ENSMUSG00000038508	Gdf15	growth differentiation factor 15 [Source:MGI Symb	0.28944
ENSMUSG00000097205	Gm17540	predicted gene, 17540 [Source:MGI Symbol;Acc:MG	0.28947
ENSMUSG00000047094	Ofcc1	orofacial cleft 1 candidate 1 [Source:MGI Symbol;A	0.28952
ENSMUSG00000001285	Myg1	melanocyte proliferating gene 1 [Source:MGI Symb	0.28955
ENSMUSG00000032482	Cspg5	chondroitin sulfate proteoglycan 5 [Source:MGI Syr	0.28955
ENSMUSG00000031209	Heph	hephaestin [Source:MGI Symbol;Acc:MGI:1332240]	0.28969
ENSMUSG00000006315	Tmem147	transmembrane protein 147 [Source:MGI Symbol;A	0.28973
ENSMUSG00000022557	Bop1	block of proliferation 1 [Source:MGI Symbol;Acc:M	0.28975
ENSMUSG00000035529	Prdm4	PR domain containing 4 [Source:MGI Symbol;Acc:M	0.2898
ENSMUSG00000001065	Zfp276	zinc finger protein (C2H2 type) 276 [Source:MGI Sy	0.28984
ENSMUSG00000091510	Mtag2	metastasis associated gene 2 [Source:MGI Symbol;	0.28989
ENSMUSG00000097967	Gm27002	predicted gene, 27002 [Source:MGI Symbol;Acc:MG	0.28992
ENSMUSG00000069273	Hist1h3e	histone cluster 1, H3e [Source:MGI Symbol;Acc:MG	0.28995
ENSMUSG00000026357	Rgs18	regulator of G-protein signaling 18 [Source:MGI Syr	0.29
ENSMUSG00000051721	BC068281	cDNA sequence BC068281 [Source:MGI Symbol;Acc	0.29009
ENSMUSG00000078619	Smarcd2	SWI/SNF related, matrix associated, actin depende	0.29014
ENSMUSG00000003411	Rab3b	RAB3B, member RAS oncogene family [Source:MGI	0.29022
ENSMUSG00000023994	Nfya	nuclear transcription factor-Y alpha [Source:MGI Sy	0.29034
ENSMUSG00000039431	Mtmr7	myotubularin related protein 7 [Source:MGI Symbo	0.29043
ENSMUSG00000095288	Gm7618	predicted pseudogene 7618 [Source:MGI Symbol;A	0.29045
ENSMUSG00000088153	Gm26348	predicted gene, 26348 [Source:MGI Symbol;Acc:MG	0.29049
ENSMUSG00000045391	1700120B22Rik	RIKEN cDNA 1700120B22 gene [Source:MGI Symbo	0.29051
ENSMUSG00000037279	Ovol2	ovo-like 2 (Drosophila) [Source:MGI Symbol;Acc:MG	0.29055
ENSMUSG00000084140	Gm14000	predicted gene 14000 [Source:MGI Symbol;Acc:MG	0.29068
ENSMUSG00000022957	Itsn1	intersectin 1 (SH3 domain protein 1A) [Source:MGI	0.29077
ENSMUSG00000085124	Gm12766	predicted gene 12766 [Source:MGI Symbol;Acc:MG	0.29078
ENSMUSG00000038172	Ttc39b	tetratricopeptide repeat domain 39B [Source:MGI S	0.2908
ENSMUSG00000062564	Tex28	testis expressed 28 [Source:MGI Symbol;Acc:MGI:2	0.2909
ENSMUSG00000035606	Ky	kyphoscoliosis peptidase [Source:MGI Symbol;Acc:I	0.29095
ENSMUSG00000038116	Phf20	PHD finger protein 20 [Source:MGI Symbol;Acc:MG	0.29101
ENSMUSG00000035232	Pdk3	pyruvate dehydrogenase kinase, isoenzyme 3 [Sour	0.29102
ENSMUSG00000097391	Mirg	miRNA containing gene [Source:MGI Symbol;Acc:M	0.29102

ENSMUSG00000037455	Slc18b1	solute carrier family 18, subfamily B, member 1 [So	0.29113
ENSMUSG00000037971	1110032A03Rik	RIKEN cDNA 1110032A03 gene [Source:MGI Symbo	0.29115
ENSMUSG00000034111	Tmed8	transmembrane emp24 domain containing 8 [Sourc	0.29118
ENSMUSG00000052572	Dlg2	discs, large homolog 2 (Drosophila) [Source:MGI Sy	0.29122
ENSMUSG00000040264	Gbp5	guanylate binding protein 5 [Source:MGI Symbol;Ac	0.29135
ENSMUSG00000024566	Atp9b	ATPase, class II, type 9B [Source:MGI Symbol;Acc:M	0.29152
ENSMUSG00000033735	Spr	sepiapterin reductase [Source:MGI Symbol;Acc:MG	0.29152
ENSMUSG00000033793	Atp6v1h	ATPase, H+ transporting, lysosomal V1 subunit H [S	0.29156
ENSMUSG00000044708	Kcnj10	potassium inwardly-rectifying channel, subfamily J,	0.29163
ENSMUSG00000080832	M6pr-ps	mannose-6-phosphate receptor, pseudogene [Sour	0.29173
ENSMUSG00000027652	Ralgapb	Ral GTPase activating protein, beta subunit (non-ca	0.29174
ENSMUSG00000033732	Sf3b3	splicing factor 3b, subunit 3 [Source:MGI Symbol;Ac	0.29176
ENSMUSG00000035305	Ror1	receptor tyrosine kinase-like orphan receptor 1 [So	0.29176
ENSMUSG00000020363	Gfpt2	glutamine fructose-6-phosphate transaminase 2 [So	0.29182
ENSMUSG00000079227	Ccr5	chemokine (C-C motif) receptor 5 [Source:MGI Sym	0.29184
ENSMUSG00000084925	1810062O18Rik	RIKEN cDNA 1810062O18 gene [Source:MGI Symbo	0.29186
ENSMUSG00000026147	Col9a1	collagen, type IX, alpha 1 [Source:MGI Symbol;Acc:I	0.29188
ENSMUSG00000055489	Ano5	anoctamin 5 [Source:MGI Symbol;Acc:MGI:357665	0.29197
ENSMUSG00000047822	Gm6484	predicted gene 6484 [Source:MGI Symbol;Acc:MGI:	0.29222
ENSMUSG00000031838	Ifi30	interferon gamma inducible protein 30 [Source:MG	0.29224
ENSMUSG00000078648	Gm17546	predicted gene, 17546 [Source:MGI Symbol;Acc:MG	0.29224
ENSMUSG00000025494	Sigirr	single immunoglobulin and toll-interleukin 1 recept	0.29235
ENSMUSG00000036972	Zic4	zinc finger protein of the cerebellum 4 [Source:MG	0.29235
ENSMUSG00000071867	Gm6304	predicted gene 6304 [Source:MGI Symbol;Acc:MGI:	0.29237
ENSMUSG00000024661	Fth1	ferritin heavy chain 1 [Source:MGI Symbol;Acc:MGI	0.29238
ENSMUSG00000079641	Rpl39	ribosomal protein L39 [Source:MGI Symbol;Acc:MG	0.29243
ENSMUSG00000070436	Serpinh1	serine (or cysteine) peptidase inhibitor, clade H, me	0.29247
ENSMUSG00000096146	Kcnj11	potassium inwardly rectifying channel, subfamily J,	0.29255
ENSMUSG00000028657	Ppt1	palmitoyl-protein thioesterase 1 [Source:MGI Symb	0.29261
ENSMUSG00000051950	B3galtl	beta 1,3-galactosyltransferase-like [Source:MGI Syr	0.29269
ENSMUSG00000025280	Polr3a	polymerase (RNA) III (DNA directed) polypeptide A	0.29275
ENSMUSG00000048277	Syngr2	synaptogyrin 2 [Source:MGI Symbol;Acc:MGI:1328	0.29275
ENSMUSG00000019254	Ppp1r12c	protein phosphatase 1, regulatory (inhibitor) subun	0.29279
ENSMUSG00000079604	Gm13219	predicted gene 13219 [Source:MGI Symbol;Acc:MG	0.29286
ENSMUSG00000081663	Gm11207	predicted gene 11207 [Source:MGI Symbol;Acc:MG	0.29286
ENSMUSG00000025223	Ldb1	LIM domain binding 1 [Source:MGI Symbol;Acc:MG	0.29287
ENSMUSG00000026697	Myoc	myocilin [Source:MGI Symbol;Acc:MGI:1202864]	0.29288
ENSMUSG00000038754	Elovl3	elongation of very long chain fatty acids (FEN1/Elo2	0.2931
ENSMUSG00000086942	Gm15489	predicted gene 15489 [Source:MGI Symbol;Acc:MG	0.29311
ENSMUSG00000021910	Nisch	nischarin [Source:MGI Symbol;Acc:MGI:1928323]	0.29313
ENSMUSG00000031555	Adam9	a disintegrin and metallopeptidase domain 9 (meltr	0.29316
ENSMUSG00000061887	Ssbp3	single-stranded DNA binding protein 3 [Source:MGI	0.29332
ENSMUSG00000082894	Gm6480	predicted pseudogene 6480 [Source:MGI Symbol;A	0.29338
ENSMUSG00000033739	Fkbpl	FK506 binding protein-like [Source:MGI Symbol;Acc	0.29339
ENSMUSG00000088856	Gm24727	predicted gene, 24727 [Source:MGI Symbol;Acc:MG	0.29339
ENSMUSG00000028125	Abca4	ATP-binding cassette, sub-family A (ABC1), member	0.29342
ENSMUSG00000050505	Pcdh20	protocadherin 20 [Source:MGI Symbol;Acc:MGI:24	0.29351

ENSMUSG00000033966	Cdkl4	cyclin-dependent kinase-like 4 [Source:MGI Symbol	0.29363
ENSMUSG00000097935	Gm3809	predicted gene 3809 [Source:MGI Symbol;Acc:MGI:	0.29364
ENSMUSG00000018974	Sart3	squamous cell carcinoma antigen recognized by T c	0.29368
ENSMUSG00000074075	Gm10621	predicted gene 10621 [Source:MGI Symbol;Acc:MG	0.29373
ENSMUSG00000022150	Dab2	disabled 2, mitogen-responsive phosphoprotein [So	0.29382
ENSMUSG00000030224	Strap	serine/threonine kinase receptor associated protei	0.29394
ENSMUSG00000026854	Usp20	ubiquitin specific peptidase 20 [Source:MGI Symbo	0.29396
ENSMUSG00000027523	Gnas	GNAS (guanine nucleotide binding protein, alpha st	0.29398
ENSMUSG00000089871	Speer7-ps1	spermatogenesis associated glutamate (E)-rich prot	0.29407
ENSMUSG00000000441	Raf1	v-raf-leukemia viral oncogene 1 [Source:MGI Symb	0.29409
ENSMUSG00000041375	Ccdc9	coiled-coil domain containing 9 [Source:MGI Symbo	0.29412
ENSMUSG00000022023	Wbp4	WW domain binding protein 4 [Source:MGI Symbol	0.29422
ENSMUSG00000028280	Gabrr1	gamma-aminobutyric acid (GABA) C receptor, subu	0.29429
ENSMUSG00000013465	Nelfb	negative elongation factor complex member B, Cob	0.29431
ENSMUSG00000046133	C130073F10Rik	RIKEN cDNA C130073F10 gene [Source:MGI Symbo	0.29434
ENSMUSG00000082274	Gm14026	predicted gene 14026 [Source:MGI Symbol;Acc:MG	0.29436
ENSMUSG00000038812	Trmt112	tRNA methyltransferase 11-2 [Source:MGI Symbol;	0.29445
ENSMUSG00000037921	Ddx60	DEAD (Asp-Glu-Ala-Asp) box polypeptide 60 [Source	0.2945
ENSMUSG00000005982	Naa60	N(alpha)-acetyltransferase 60, NatF catalytic subun	0.29457
ENSMUSG00000028434	Epb4.1l4b	erythrocyte protein band 4.1-like 4b [Source:MGI S	0.29462
ENSMUSG00000031442	Mcf2l	mcf.2 transforming sequence-like [Source:MGI Sym	0.29468
ENSMUSG00000086224	2700069I18Rik	RIKEN cDNA 2700069I18 gene [Source:MGI Symbol	0.29469
ENSMUSG00000063804	Lin28b	lin-28 homolog B (C. elegans) [Source:MGI Symbol;	0.29472
ENSMUSG00000027088	Phospho2	phosphatase, orphan 2 [Source:MGI Symbol;Acc:M	0.29483
ENSMUSG00000037541	Shank2	SH3/ankyrin domain gene 2 [Source:MGI Symbol;A	0.29488
ENSMUSG00000020524	Gria1	glutamate receptor, ionotropic, AMPA1 (alpha 1) [S	0.2949
ENSMUSG00000096377	Mir5123	microRNA 5123 [Source:MGI Symbol;Acc:MGI:4950	0.29492
ENSMUSG00000084892	Gm14471	predicted gene 14471 [Source:MGI Symbol;Acc:MG	0.29497
ENSMUSG00000029705	Cux1	cut-like homeobox 1 [Source:MGI Symbol;Acc:MGI:	0.29536
ENSMUSG00000037149	Ddx1	DEAD (Asp-Glu-Ala-Asp) box polypeptide 1 [Source:	0.29536
ENSMUSG00000062270	Morf4l1	mortality factor 4 like 1 [Source:MGI Symbol;Acc:M	0.29541
ENSMUSG00000037946	Fgd3	FYVE, RhoGEF and PH domain containing 3 [Source	0.29542
ENSMUSG00000024387	Csnk2b	casein kinase 2, beta polypeptide [Source:MGI Sym	0.29548
ENSMUSG00000087624	9230111E07Rik	RIKEN cDNA 9230111E07 gene [Source:MGI Symbo	0.29562
ENSMUSG00000030246	Ldhb	lactate dehydrogenase B [Source:MGI Symbol;Acc:I	0.29566
ENSMUSG00000058600	Rpl30	ribosomal protein L30 [Source:MGI Symbol;Acc:MG	0.29567
ENSMUSG00000021466	Ptch1	patched homolog 1 [Source:MGI Symbol;Acc:MGI:1	0.29576
ENSMUSG00000019883	Echdc1	enoyl Coenzyme A hydratase domain containing 1 [	0.29586
ENSMUSG00000031965	Tbx20	T-box 20 [Source:MGI Symbol;Acc:MGI:1888496]	0.29593
ENSMUSG00000042790	Rnf214	ring finger protein 214 [Source:MGI Symbol;Acc:MG	0.29602
ENSMUSG00000025789	St8sia2	ST8 alpha-N-acetyl-neuraminide alpha-2,8-sialyltra	0.2961
ENSMUSG00000081118	Gm6758	predicted gene 6758 [Source:MGI Symbol;Acc:MGI:	0.2961
ENSMUSG00000057262	Gm10020	predicted pseudogene 10020 [Source:MGI Symbol;	0.29616
ENSMUSG00000026104	Stat1	signal transducer and activator of transcription 1 [S	0.29621
ENSMUSG00000031494	Cd209a	CD209a antigen [Source:MGI Symbol;Acc:MGI:2157	0.29623
ENSMUSG00000030474	Siglece	sialic acid binding Ig-like lectin E [Source:MGI Symb	0.29637
ENSMUSG00000021759	Ppap2a	phosphatidic acid phosphatase type 2A [Source:MG	0.29642

ENSMUSG00000049504	Proser1	proline and serine rich 1 [Source:MGI Symbol;Acc:M	0.29642
ENSMUSG00000022479	Vdr	vitamin D receptor [Source:MGI Symbol;Acc:MGI:1(	0.29645
ENSMUSG00000081700	Gm14337	predicted gene 14337 [Source:MGI Symbol;Acc:MG	0.2966
ENSMUSG00000023169	Slc38a1	solute carrier family 38, member 1 [Source:MGI Syr	0.29661
ENSMUSG00000084081	Gm12057	predicted gene 12057 [Source:MGI Symbol;Acc:MG	0.29665
ENSMUSG00000039539	Sgcz	sarcoglycan zeta [Source:MGI Symbol;Acc:MGI:238	0.29666
ENSMUSG00000061928	Dsg1b	desmoglein 1 beta [Source:MGI Symbol;Acc:MGI:26	0.29682
ENSMUSG00000036054	Sugp2	SURP and G patch domain containing 2 [Source:MG	0.29687
ENSMUSG00000036306	Lzts1	leucine zipper, putative tumor suppressor 1 [Source	0.2969
ENSMUSG00000006476	Nsmf	NMDA receptor synaptonuclear signaling and neur	0.29699
ENSMUSG00000020228	Helb	helicase (DNA) B [Source:MGI Symbol;Acc:MGI:215	0.29704
ENSMUSG00000034032	Rpap1	RNA polymerase II associated protein 1 [Source:MG	0.2971
ENSMUSG00000005150	Wdr83	WD repeat domain containing 83 [Source:MGI Sym	0.29721
ENSMUSG00000028790	Khdrbs1	KH domain containing, RNA binding, signal transdu	0.29723
ENSMUSG00000028960	Ube4b	ubiquitination factor E4B, UFD2 homolog (S. cerevis	0.29724
ENSMUSG00000027546	Atp9a	ATPase, class II, type 9A [Source:MGI Symbol;Acc:M	0.29734
ENSMUSG00000036357	Gpr101	G protein-coupled receptor 101 [Source:MGI Symb	0.29751
ENSMUSG00000025137	Pcyt2	phosphate cytidylyltransferase 2, ethanolamine [So	0.29758
ENSMUSG00000039257	Vstm2b	V-set and transmembrane domain containing 2B [S	0.29761
ENSMUSG00000022009	Nufip1	nuclear fragile X mental retardation protein interac	0.29765
ENSMUSG00000032625	Thsd7a	thrombospondin, type I, domain containing 7A [Sou	0.29767
ENSMUSG00000062248	Cks2	CDC28 protein kinase regulatory subunit 2 [Source:	0.29767
ENSMUSG00000042606	Hirip3	HIRA interacting protein 3 [Source:MGI Symbol;Acc	0.29779
ENSMUSG00000043284	Tmem11	transmembrane protein 11 [Source:MGI Symbol;Ac	0.29783
ENSMUSG00000028034	Fubp1	far upstream element (FUSE) binding protein 1 [Sou	0.29784
ENSMUSG00000000876	Pxmp4	peroxisomal membrane protein 4 [Source:MGI Sym	0.29802
ENSMUSG00000050619	Zscan29	zinc finger SCAN domains 29 [Source:MGI Symbol;A	0.29808
ENSMUSG00000087687	Pet100	PET100 homolog (S. cerevisiae) [Source:MGI Symbo	0.29811
ENSMUSG00000048371	Pdp2	pyruvate dehyrogenase phosphatase catalytic subu	0.29816
ENSMUSG00000022306	Zfpm2	zinc finger protein, multitype 2 [Source:MGI Symbo	0.29821
ENSMUSG00000026630	Batf3	basic leucine zipper transcription factor, ATF-like 3	0.29825
ENSMUSG00000042078	Svop	SV2 related protein [Source:MGI Symbol;Acc:MGI:1	0.29831
ENSMUSG00000034189	Hsdl1	hydroxysteroid dehydrogenase like 1 [Source:MGI S	0.29834
ENSMUSG00000021903	Galnt15	UDP-N-acetyl-alpha-D-galactosamine:polypeptide N	0.29836
ENSMUSG00000097768	2310043M15Rik	RIKEN cDNA 2310043M15 gene [Source:MGI Symbo	0.29838
ENSMUSG00000009545	Kcnq1	potassium voltage-gated channel, subfamily Q, mer	0.29842
ENSMUSG00000004285	Atp6v1f	ATPase, H+ transporting, lysosomal V1 subunit F [So	0.29849
ENSMUSG00000028750	Pla2g2c	phospholipase A2, group IIC [Source:MGI Symbol;A	0.2985
ENSMUSG00000044709	Gemin7	gem (nuclear organelle) associated protein 7 [Sourc	0.2985
ENSMUSG00000084373	Rps2-ps4	ribosomal protein S2, pseudogene 4 [Source:MGI S	0.29864
ENSMUSG00000042207	Kdm5b	lysine (K)-specific demethylase 5B [Source:MGI Sym	0.29865
ENSMUSG00000086561	Gm15540	predicted gene 15540 [Source:MGI Symbol;Acc:MG	0.29866
ENSMUSG00000083327	Vcp-rs	valosin containing protein, related sequence [Sourc	0.29867
ENSMUSG00000081943	Gm9078	predicted gene 9078 [Source:MGI Symbol;Acc:MGI:	0.2987
ENSMUSG00000059878	Zfp422	zinc finger protein 422 [Source:MGI Symbol;Acc:MG	0.29872
ENSMUSG00000039210	Gpatch2	G patch domain containing 2 [Source:MGI Symbol;A	0.29874
ENSMUSG00000080870	Gm14111	predicted gene 14111 [Source:MGI Symbol;Acc:MG	0.29874

ENSMUSG00000030680	Pagr1a	PAXIP1 associated glutamate rich protein 1A [Sourc	0.29875
ENSMUSG00000063830	Gm6065	predicted gene 6065 [Source:MGI Symbol;Acc:MGI:	0.29877
ENSMUSG00000046756	Mrps7	mitchondrial ribosomal protein S7 [Source:MGI Syn	0.29882
ENSMUSG00000020176	Grb10	growth factor receptor bound protein 10 [Source:M	0.29884
ENSMUSG00000035177	Nlrp2	NLR family, pyrin domain containing 2 [Source:MGI	0.29884
ENSMUSG00000030035	Wbp1	WW domain binding protein 1 [Source:MGI Symbol	0.29886
ENSMUSG00000029432	Gbas	glioblastoma amplified sequence [Source:MGI Sym	0.29894
ENSMUSG00000027514	Zbp1	Z-DNA binding protein 1 [Source:MGI Symbol;Acc:M	0.29912
ENSMUSG00000026270	Capn10	calpain 10 [Source:MGI Symbol;Acc:MGI:1344392]	0.29923
ENSMUSG00000021453	Gadd45g	growth arrest and DNA-damage-inducible 45 gamm	0.29925
ENSMUSG00000079564	Gm11149	predicted gene 11149 [Source:MGI Symbol;Acc:MG	0.29931
ENSMUSG00000052222	1700084E18Rik	RIKEN cDNA 1700084E18 gene [Source:MGI Symbo	0.29934
ENSMUSG00000025403	Shmt2	serine hydroxymethyltransferase 2 (mitochondrial)	0.29941
ENSMUSG00000045052	Prlhr	prolactin releasing hormone receptor [Source:MGI	0.29943
ENSMUSG00000026880	Stom	stomatin [Source:MGI Symbol;Acc:MGI:95403]	0.29953
ENSMUSG00000021044	Adck1	aarF domain containing kinase 1 [Source:MGI Symb	0.29957
ENSMUSG00000021843	Ktn1	kinectin 1 [Source:MGI Symbol;Acc:MGI:109153]	0.2996
ENSMUSG00000098221	Gm27030	predicted gene, 27030 [Source:MGI Symbol;Acc:M(	0.29961
ENSMUSG00000032288	Imp3	IMP3, U3 small nucleolar ribonucleoprotein, homol	0.29971
ENSMUSG00000037533	Rapgef6	Rap guanine nucleotide exchange factor (GEF) 6 [Sc	0.29973
ENSMUSG00000020105	Lrig3	leucine-rich repeats and immunoglobulin-like doma	0.29976
ENSMUSG00000092617	Gm8412	predicted gene 8412 [Source:MGI Symbol;Acc:MGI:	0.29976
ENSMUSG00000071661	Zbtb3	zinc finger and BTB domain containing 3 [Source:M	0.29997
ENSMUSG00000087279	4930544D05Rik	RIKEN cDNA 4930544D05 gene [Source:MGI Symbo	0.30001
ENSMUSG00000086549	Gm13648	predicted gene 13648 [Source:MGI Symbol;Acc:MG	0.30002
ENSMUSG00000083553	Gm11989	predicted gene 11989 [Source:MGI Symbol;Acc:MG	0.30005
ENSMUSG00000087388	Gm12128	predicted gene 12128 [Source:MGI Symbol;Acc:MG	0.30007
ENSMUSG00000077265	Gm24430	predicted gene, 24430 [Source:MGI Symbol;Acc:M(	0.30008
ENSMUSG00000047514	Tspyl1	testis-specific protein, Y-encoded-like 1 [Source:M(	0.3001
ENSMUSG00000058287	Gm12253	predicted gene 12253 [Source:MGI Symbol;Acc:MG	0.3001
ENSMUSG00000005202	Shbg	sex hormone binding globulin [Source:MGI Symbol;	0.30013
ENSMUSG00000000439	Mkrn2	makorin, ring finger protein, 2 [Source:MGI Symbol	0.30019
ENSMUSG00000032691	Nlrp3	NLR family, pyrin domain containing 3 [Source:MGI	0.30019
ENSMUSG00000047454	Gphn	gephyrin [Source:MGI Symbol;Acc:MGI:109602]	0.30021
ENSMUSG00000023943	Sult1c1	sulfotransferase family, cytosolic, 1C, member 1 [Sc	0.30026
ENSMUSG00000022456	3-Sep	septin 3 [Source:MGI Symbol;Acc:MGI:1345148]	0.30027
ENSMUSG00000023935	Spats1	spermatogenesis associated, serine-rich 1 [Source:I	0.30032
ENSMUSG00000032038	St3gal4	ST3 beta-galactoside alpha-2,3-sialyltransferase 4 [S	0.30038
ENSMUSG00000064057	Scgb3a1	secretoglobin, family 3A, member 1 [Source:MGI Sy	0.30045
ENSMUSG00000017754	Pltp	phospholipid transfer protein [Source:MGI Symbol;	0.30047
ENSMUSG00000051329	Nup160	nucleoporin 160 [Source:MGI Symbol;Acc:MGI:192	0.30059
ENSMUSG00000062510	Nsl1	NSL1, MIND kinetochore complex component, hom	0.30069
ENSMUSG00000023048	Prr13	proline rich 13 [Source:MGI Symbol;Acc:MGI:19134	0.3007
ENSMUSG00000021848	Otx2	orthodenticle homolog 2 (Drosophila) [Source:MGI	0.30075
ENSMUSG00000025870	Arl10	ADP-ribosylation factor-like 10 [Source:MGI Symbo	0.30079
ENSMUSG00000039304	Tnfsf10	tumor necrosis factor (ligand) superfamily, membe	0.30081
ENSMUSG00000036896	C1qc	complement component 1, q subcomponent, C cha	0.30085

ENSMUSG00000032047	Acat1	acetyl-Coenzyme A acetyltransferase 1 [Source:MG	0.30088
ENSMUSG00000074657	Kif5a	kinesin family member 5A [Source:MGI Symbol;Acc	0.30092
ENSMUSG00000030486	Zfp108	zinc finger protein 108 [Source:MGI Symbol;Acc:MG	0.30098
ENSMUSG00000073705	Apitd1	apoptosis-inducing, TAF9-like domain 1 [Source:MG	0.301
ENSMUSG00000033684	Qsox1	quiescin Q6 sulfhydryl oxidase 1 [Source:MGI Symb	0.30105
ENSMUSG00000014504	Srp19	signal recognition particle 19 [Source:MGI Symbol;A	0.30108
ENSMUSG00000097204	Gm17690	predicted gene, 17690 [Source:MGI Symbol;Acc:MG	0.30119
ENSMUSG00000045314	Sowahb	sosondowah ankyrin repeat domain family membe	0.3012
ENSMUSG00000097453	Gm26894	predicted gene, 26894 [Source:MGI Symbol;Acc:MG	0.30129
ENSMUSG00000058440	Nrf1	nuclear respiratory factor 1 [Source:MGI Symbol;Ac	0.30148
ENSMUSG00000063870	Chd4	chromodomain helicase DNA binding protein 4 [Sou	0.30149
ENSMUSG00000078588	Ccdc24	coiled-coil domain containing 24 [Source:MGI Symb	0.30155
ENSMUSG00000031669	Gins3	GINS complex subunit 3 (Psf3 homolog) [Source:MG	0.30156
ENSMUSG00000004791	Pgf	placental growth factor [Source:MGI Symbol;Acc:M	0.30158
ENSMUSG00000030744	Rps3	ribosomal protein S3 [Source:MGI Symbol;Acc:MGI	0.3017
ENSMUSG00000090357	Gm17077	predicted gene 17077 [Source:MGI Symbol;Acc:MG	0.30177
ENSMUSG00000021615	Xrcc4	X-ray repair complementing defective repair in Chi	0.30183
ENSMUSG00000007379	Dennd2c	DENN/MADD domain containing 2C [Source:MGI Sy	0.30185
ENSMUSG00000034115	Scn11a	sodium channel, voltage-gated, type XI, alpha [Sou	0.3019
ENSMUSG00000070814	6330408A02Rik	RIKEN cDNA 6330408A02 gene [Source:MGI Symbo	0.30199
ENSMUSG00000022748	Cmss1	cms small ribosomal subunit 1 [Source:MGI Symbol	0.30202
ENSMUSG00000024902	Mrpl11	mitochondrial ribosomal protein L11 [Source:MGI S	0.30207
ENSMUSG00000029071	Dvl1	dishevelled, dsh homolog 1 (Drosophila) [Source:M	0.30218
ENSMUSG00000030930	Chst15	carbohydrate (N-acetylgalactosamine 4-sulfate 6-O	0.30227
ENSMUSG00000024953	Prdx5	peroxiredoxin 5 [Source:MGI Symbol;Acc:MGI:1859	0.30228
ENSMUSG00000086219	2410137F16Rik	RIKEN cDNA 2410137F16 gene [Source:MGI Symbo	0.3023
ENSMUSG00000024238	Zeb1	zinc finger E-box binding homeobox 1 [Source:MGI	0.30235
ENSMUSG00000046442	Ppm1e	protein phosphatase 1E (PP2C domain containing) [	0.30246
ENSMUSG00000027865	Gdap2	ganglioside-induced differentiation-associated-prot	0.30247
ENSMUSG00000033916	Chmp2a	charged multivesicular body protein 2A [Source:MG	0.30247
ENSMUSG00000026442	Nfasc	neurofascin [Source:MGI Symbol;Acc:MGI:104753]	0.30248
ENSMUSG00000031226	Pbdc1	polysaccharide biosynthesis domain containing 1 [S	0.30248
ENSMUSG00000047945	Marcksl1	MARCKS-like 1 [Source:MGI Symbol;Acc:MGI:97143	0.3025
ENSMUSG00000075054	Yae1d1	Yae1 domain containing 1 [Source:MGI Symbol;Acc	0.3025
ENSMUSG00000029173	Sepsecs	Sep (O-phosphoserine) tRNA:Sec (selenocysteine) t	0.30251
ENSMUSG00000074300	BC030870	cDNA sequence BC030870 [Source:MGI Symbol;Acc	0.30253
ENSMUSG00000065934	Gm25630	predicted gene, 25630 [Source:MGI Symbol;Acc:MG	0.30267
ENSMUSG00000032897	Nfyc	nuclear transcription factor-Y gamma [Source:MGI	0.30271
ENSMUSG00000084738	Gm25809	predicted gene, 25809 [Source:MGI Symbol;Acc:MG	0.30274
ENSMUSG00000097189	Gm26594	predicted gene, 26594 [Source:MGI Symbol;Acc:MG	0.30276
ENSMUSG00000052373	Mpp3	membrane protein, palmitoylated 3 (MAGUK p55 s	0.30283
ENSMUSG00000021116	Eif2s1	eukaryotic translation initiation factor 2, subunit 1	0.30284
ENSMUSG00000021773	Comtd1	catechol-O-methyltransferase domain containing 1	0.30285
ENSMUSG00000021991	Cacna2d3	calcium channel, voltage-dependent, alpha2/delta	0.30287
ENSMUSG00000019806	Aig1	androgen-induced 1 [Source:MGI Symbol;Acc:MGI:	0.30292
ENSMUSG00000046196	Ttc39d	tetratricopeptide repeat domain 39D [Source:MGI	0.30293
ENSMUSG00000097929	2810011L19Rik	RIKEN cDNA 2810011L19 gene [Source:MGI Symbo	0.30295

ENSMUSG00000077505	Gm24233	predicted gene, 24233 [Source:MGI Symbol;Acc:M(	0.30298
ENSMUSG00000073599	Ecscr	endothelial cell surface expressed chemotaxis and a	0.30307
ENSMUSG00000025510	Cd151	CD151 antigen [Source:MGI Symbol;Acc:MGI:10963	0.30314
ENSMUSG00000044694	2010007H06Rik	RIKEN cDNA 2010007H06 gene [Source:MGI Symbo	0.30321
ENSMUSG00000081492	Gm9311	predicted gene 9311 [Source:MGI Symbol;Acc:MGI:	0.30322
ENSMUSG00000027932	Slc27a3	solute carrier family 27 (fatty acid transporter), me	0.30323
ENSMUSG00000081460	Gm13168	predicted gene 13168 [Source:MGI Symbol;Acc:MG	0.30323
ENSMUSG00000086058	Gm12575	predicted gene 12575 [Source:MGI Symbol;Acc:MG	0.30324
ENSMUSG00000024516	Sec11c	SEC11 homolog C (S. cerevisiae) [Source:MGI Symb	0.30325
ENSMUSG00000018661	Cog1	component of oligomeric golgi complex 1 [Source:N	0.30336
ENSMUSG00000041460	Cacna2d4	calcium channel, voltage-dependent, alpha 2/delta	0.30339
ENSMUSG00000097705	Gm26740	predicted gene, 26740 [Source:MGI Symbol;Acc:M(	0.30339
ENSMUSG00000035390	Brsk1	BR serine/threonine kinase 1 [Source:MGI Symbol;/	0.30342
ENSMUSG00000097676	Gm26612	predicted gene, 26612 [Source:MGI Symbol;Acc:M(	0.30347
ENSMUSG00000084789	Gm12974	predicted gene 12974 [Source:MGI Symbol;Acc:MG	0.3035
ENSMUSG00000087478	4930506C21Rik	RIKEN cDNA 4930506C21 gene [Source:MGI Symbo	0.3035
ENSMUSG00000015766	Eps8	epidermal growth factor receptor pathway substrat	0.30352
ENSMUSG00000066688	Gm13083	predicted gene 13083 [Source:MGI Symbol;Acc:MG	0.30353
ENSMUSG00000075012	Fjx1	four jointed box 1 (Drosophila) [Source:MGI Symbo	0.30364
ENSMUSG00000037188	Grhl3	grainyhead-like 3 (Drosophila) [Source:MGI Symbol	0.30367
ENSMUSG00000038895	Zfp653	zinc finger protein 653 [Source:MGI Symbol;Acc:M(	0.30368
ENSMUSG00000086890	1700021J08Rik	RIKEN cDNA 1700021J08 gene [Source:MGI Symbol	0.3037
ENSMUSG00000020961	Ston2	stonin 2 [Source:MGI Symbol;Acc:MGI:1918272]	0.30377
ENSMUSG00000068141	Gm10232	predicted pseudogene 10232 [Source:MGI Symbol;	0.30379
ENSMUSG00000050212	Eva1b	eva-1 homolog B (C. elegans) [Source:MGI Symbol;/	0.30381
ENSMUSG00000042106	Fam212a	family with sequence similarity 212, member A [Sou	0.30383
ENSMUSG00000067321	Gm7931	predicted pseudogene 7931 [Source:MGI Symbol;A	0.30383
ENSMUSG00000038296	Galnt18	UDP-N-acetyl-alpha-D-galactosamine:polypeptide N	0.30386
ENSMUSG00000046215	Rprml	reprimo-like [Source:MGI Symbol;Acc:MGI:2144486	0.30391
ENSMUSG00000053541	Gm4759	predicted gene 4759 [Source:MGI Symbol;Acc:MGI:	0.30392
ENSMUSG00000027001	Dusp19	dual specificity phosphatase 19 [Source:MGI Symbo	0.30393
ENSMUSG00000020684	Rasl10b	RAS-like, family 10, member B [Source:MGI Symbol	0.30405
ENSMUSG00000031066	Usp11	ubiquitin specific peptidase 11 [Source:MGI Symbo	0.30408
ENSMUSG00000038451	Spsb2	splA/ryanodine receptor domain and SOCS box con	0.30424
ENSMUSG00000020220	Vps13d	vacuolar protein sorting 13 D (yeast) [Source:MGI S	0.30425
ENSMUSG00000053510	Nrd1	nardilysin, N-arginine dibasic convertase, NRD conv	0.30438
ENSMUSG00000033488	BC026585	cDNA sequence BC026585 [Source:MGI Symbol;Acc	0.30456
ENSMUSG00000032754	Slc8b1	solute carrier family 8 (sodium/lithium/calcium exc	0.3046
ENSMUSG00000008393	Carhsp1	calcium regulated heat stable protein 1 [Source:MC	0.30468
ENSMUSG00000091172	Gm17120	predicted gene 17120 [Source:MGI Symbol;Acc:MG	0.30472
ENSMUSG00000063305	Psg20	pregnancy-specific glycoprotein 20 [Source:MGI Syr	0.30476
ENSMUSG00000092906	Gm25736	predicted gene, 25736 [Source:MGI Symbol;Acc:M(	0.30484
ENSMUSG00000086041	Gm12869	predicted gene 12869 [Source:MGI Symbol;Acc:MG	0.30487
ENSMUSG00000074753	Gm14092	predicted gene 14092 [Source:MGI Symbol;Acc:MG	0.30488
ENSMUSG00000031447	Lamp1	lysosomal-associated membrane protein 1 [Source:	0.3049
ENSMUSG00000083587	Gm14741	predicted gene 14741 [Source:MGI Symbol;Acc:MG	0.3049
ENSMUSG00000002984	Tomm40	translocase of outer mitochondrial membrane 40 h	0.30504

ENSMUSG00000024590	Lmnb1	lamin B1 [Source:MGI Symbol;Acc:MGI:96795]	0.30513
ENSMUSG00000082851	Gm11931	predicted gene 11931 [Source:MGI Symbol;Acc:MG	0.30514
ENSMUSG00000036136	Fam110c	family with sequence similarity 110, member C [Sou	0.30517
ENSMUSG00000017943	Gdap1l1	ganglioside-induced differentiation-associated prot	0.30521
ENSMUSG00000022710	Usp7	ubiquitin specific peptidase 7 [Source:MGI Symbol;	0.30521
ENSMUSG00000086916	Gm15903	predicted gene 15903 [Source:MGI Symbol;Acc:MG	0.30524
ENSMUSG00000088670	Gm23663	predicted gene, 23663 [Source:MGI Symbol;Acc:MG	0.30539
ENSMUSG00000085078	C030013C21Rik	RIKEN cDNA C030013C21 gene [Source:MGI Symbo	0.3054
ENSMUSG00000091080	Prr23a	proline rich 23A [Source:MGI Symbol;Acc:MGI:3645	0.30543
ENSMUSG00000092455	Gm20456	predicted gene 20456 [Source:MGI Symbol;Acc:MG	0.30547
ENSMUSG00000096837	Gm12651	predicted gene 12651 [Source:MGI Symbol;Acc:MG	0.30551
ENSMUSG00000040896	Kcnd3	potassium voltage-gated channel, Shal-related fam	0.30562
ENSMUSG00000056014	A430033K04Rik	RIKEN cDNA A430033K04 gene [Source:MGI Symbo	0.3058
ENSMUSG00000041241	Mul1	mitochondrial ubiquitin ligase activator of NFKB 1 [	0.30589
ENSMUSG00000063362	Alg11	asparagine-linked glycosylation 11 (alpha-1,2-mann	0.3059
ENSMUSG00000038838	Vars2	valyl-tRNA synthetase 2, mitochondrial (putative) [	0.30591
ENSMUSG00000032491	Nradd	neurotrophin receptor associated death domain [So	0.306
ENSMUSG00000074355	Gm10676	predicted gene 10676 [Source:MGI Symbol;Acc:MG	0.3062
ENSMUSG00000022385	Gtse1	G two S phase expressed protein 1 [Source:MGI Syr	0.30623
ENSMUSG00000029775	Klhdc10	kelch domain containing 10 [Source:MGI Symbol;Ac	0.30626
ENSMUSG00000050213	Snip1	Smad nuclear interacting protein 1 [Source:MGI Syr	0.30632
ENSMUSG00000042997	Nhlrc3	NHL repeat containing 3 [Source:MGI Symbol;Acc:M	0.30639
ENSMUSG00000041890	Git2	G protein-coupled receptor kinase-interactor 2 [Sou	0.30641
ENSMUSG00000053963	6330403A02Rik	RIKEN cDNA 6330403A02 gene [Source:MGI Symbo	0.30651
ENSMUSG00000026029	Casp8	caspase 8 [Source:MGI Symbol;Acc:MGI:1261423]	0.30654
ENSMUSG00000022763	Aifm3	apoptosis-inducing factor, mitochondrion-associate	0.30655
ENSMUSG00000087784	Gm23171	predicted gene, 23171 [Source:MGI Symbol;Acc:MG	0.30655
ENSMUSG00000040205	Cuzd1	CUB and zona pellucida-like domains 1 [Source:MG	0.30656
ENSMUSG00000022097	Sftpc	surfactant associated protein C [Source:MGI Symbo	0.30657
ENSMUSG00000041578	Crx	cone-rod homeobox containing gene [Source:MGI S	0.30658
ENSMUSG00000082063	Gm12993	predicted gene 12993 [Source:MGI Symbol;Acc:MG	0.30659
ENSMUSG00000022441	Efcab6	EF-hand calcium binding domain 6 [Source:MGI Syr	0.30671
ENSMUSG00000087938	Gm26049	predicted gene, 26049 [Source:MGI Symbol;Acc:MG	0.30675
ENSMUSG00000092889	Gm22121	predicted gene, 22121 [Source:MGI Symbol;Acc:MG	0.30682
ENSMUSG00000029836	Cbx3	chromobox 3 [Source:MGI Symbol;Acc:MGI:108515	0.30687
ENSMUSG00000039395	Mreg	melanoregulin [Source:MGI Symbol;Acc:MGI:21518	0.30692
ENSMUSG00000037679	Inf2	inverted formin, FH2 and WH2 domain containing [	0.3071
ENSMUSG00000038593	Tctn1	tectonic family member 1 [Source:MGI Symbol;Acc	0.3071
ENSMUSG00000027078	Ube2l6	ubiquitin-conjugating enzyme E2L 6 [Source:MGI Sy	0.30712
ENSMUSG00000074673	Ttll9	tubulin tyrosine ligase-like family, member 9 [Sourc	0.30716
ENSMUSG00000026994	Galnt3	UDP-N-acetyl-alpha-D-galactosamine:polypeptide N	0.30729
ENSMUSG00000084998	Gm16279	predicted gene 16279 [Source:MGI Symbol;Acc:MG	0.30739
ENSMUSG00000017756	Slc12a7	solute carrier family 12, member 7 [Source:MGI Syr	0.3074
ENSMUSG00000004936	Map2k1	mitogen-activated protein kinase kinase 1 [Source:I	0.30746
ENSMUSG00000089762	Ier5l	immediate early response 5-like [Source:MGI Symb	0.30755
ENSMUSG00000006517	Mvd	mevalonate (diphospho) decarboxylase [Source:MG	0.30767
ENSMUSG00000041797	Abca9	ATP-binding cassette, sub-family A (ABC1), member	0.30771

ENSMUSG00000025369	Smarcc2	SWI/SNF related, matrix associated, actin depender	0.30774
ENSMUSG00000086867	Gm4577	predicted gene 4577 [Source:MGI Symbol;Acc:MGI:	0.30777
ENSMUSG00000098020	Gm27012	predicted gene, 27012 [Source:MGI Symbol;Acc:MG	0.30785
ENSMUSG00000073787	Gm10575	predicted gene 10575 [Source:MGI Symbol;Acc:MG	0.30791
ENSMUSG00000027342	Pcna	proliferating cell nuclear antigen [Source:MGI Symb	0.30798
ENSMUSG00000085665	Gm12059	predicted gene 12059 [Source:MGI Symbol;Acc:MG	0.308
ENSMUSG00000025279	Dnase1l3	deoxyribonuclease 1-like 3 [Source:MGI Symbol;Acc	0.30811
ENSMUSG00000004837	Grap	GRB2-related adaptor protein [Source:MGI Symbol;	0.30824
ENSMUSG00000046178	Nxph1	neurexophilin 1 [Source:MGI Symbol;Acc:MGI:1074	0.30828
ENSMUSG00000051331	Cacna1c	calcium channel, voltage-dependent, L type, alpha	0.30856
ENSMUSG00000092286	Gm20448	predicted gene 20448 [Source:MGI Symbol;Acc:MG	0.30868
ENSMUSG00000000884	Gnb1l	guanine nucleotide binding protein (G protein), bet	0.30876
ENSMUSG00000040627	Aicda	activation-induced cytidine deaminase [Source:MG	0.30882
ENSMUSG00000090624	Gm17060	predicted gene 17060 [Source:MGI Symbol;Acc:MG	0.30882
ENSMUSG00000032612	Usp4	ubiquitin specific peptidase 4 (proto-oncogene) [So	0.30892
ENSMUSG00000051486	Pcdhb11	protocadherin beta 11 [Source:MGI Symbol;Acc:MG	0.309
ENSMUSG00000046180	4930550L24Rik	RIKEN cDNA 4930550L24 gene [Source:MGI Symbo	0.30905
ENSMUSG00000040434	Gyltl1b	glycosyltransferase-like 1B [Source:MGI Symbol;Acc	0.30908
ENSMUSG00000070323	Mmp27	matrix metallopeptidase 27 [Source:MGI Symbol;Ac	0.3091
ENSMUSG00000085274	BC046401	cDNA sequence BC046401 [Source:MGI Symbol;Acc	0.3091
ENSMUSG00000011256	Adam19	a disintegrin and metallopeptidase domain 19 (mel	0.30914
ENSMUSG00000038324	Trpc4ap	transient receptor potential cation channel, subfam	0.30923
ENSMUSG00000068601	Gm10244	predicted gene 10244 [Source:MGI Symbol;Acc:MG	0.30926
ENSMUSG00000097102	2310069G16Rik	RIKEN cDNA 2310069G16 gene [Source:MGI Symbo	0.30936
ENSMUSG00000034898	Filip1	filamin A interacting protein 1 [Source:MGI Symbol	0.30955
ENSMUSG00000025130	P4hb	prolyl 4-hydroxylase, beta polypeptide [Source:MG	0.30959
ENSMUSG00000064326	Siva1	SIVA1, apoptosis-inducing factor [Source:MGI Symb	0.3096
ENSMUSG00000021585	Cast	calpastatin [Source:MGI Symbol;Acc:MGI:1098236]	0.30963
ENSMUSG00000019232	Etnppl	ethanolamine phosphate phospholyase [Source:MG	0.30965
ENSMUSG00000025648	Pfkfb4	6-phosphofructo-2-kinase/fructose-2,6-biphosphat	0.30967
ENSMUSG00000038371	Sbf2	SET binding factor 2 [Source:MGI Symbol;Acc:MGI:	0.3097
ENSMUSG00000042826	Fgf11	fibroblast growth factor 11 [Source:MGI Symbol;Ac	0.3097
ENSMUSG00000050390	C77080	expressed sequence C77080 [Source:MGI Symbol;A	0.30975
ENSMUSG00000031691	Tnpo2	transportin 2 (importin 3, karyopherin beta 2b) [So	0.3099
ENSMUSG00000032116	Stt3a	STT3, subunit of the oligosaccharyltransferase com	0.30992
ENSMUSG00000029394	Cdk2ap1	CDK2 (cyclin-dependent kinase 2)-associated prote	0.30993
ENSMUSG00000020739	Nup85	nucleoporin 85 [Source:MGI Symbol;Acc:MGI:3046	0.31007
ENSMUSG00000030895	Hpx	hemopexin [Source:MGI Symbol;Acc:MGI:105112]	0.31014
ENSMUSG00000026500	Cox20	COX20 Cox2 chaperone [Source:MGI Symbol;Acc:M	0.31023
ENSMUSG00000026456	Cyb5r1	cytochrome b5 reductase 1 [Source:MGI Symbol;Ac	0.31025
ENSMUSG00000028678	Kif2c	kinesin family member 2C [Source:MGI Symbol;Acc	0.31031
ENSMUSG00000036218	Pdzrn4	PDZ domain containing RING finger 4 [Source:MGI S	0.31033
ENSMUSG00000047116	9430041J12Rik	RIKEN cDNA 9430041J12 gene [Source:MGI Symbol	0.31034
ENSMUSG00000041046	Ramp3	receptor (calcitonin) activity modifying protein 3 [S	0.31037
ENSMUSG00000024442	0610009O20Rik	RIKEN cDNA 0610009O20 gene [Source:MGI Symbo	0.31038
ENSMUSG00000037653	Kctd8	potassium channel tetramerisation domain contain	0.31042
ENSMUSG00000027615	Hps3	Hermansky-Pudlak syndrome 3 homolog (human) [	0.31047

ENSMUSG00000033790	Tubgcp5	tubulin, gamma complex associated protein 5 [Sour	0.31047
ENSMUSG00000028452	Vcp	valosin containing protein [Source:MGI Symbol;Acc	0.31053
ENSMUSG00000079042	Trim61	tripartite motif-containing 61 [Source:MGI Symbol;	0.3106
ENSMUSG00000096403	Gm9825	predicted gene 9825 [Source:MGI Symbol;Acc:MGI:	0.31062
ENSMUSG00000064299	4921528I07Rik	RIKEN cDNA 4921528I07 gene [Source:MGI Symbol	0.31074
ENSMUSG00000087405	Gm14232	predicted gene 14232 [Source:MGI Symbol;Acc:MG	0.31078
ENSMUSG00000026003	Acadl	acyl-Coenzyme A dehydrogenase, long-chain [Sour	0.31087
ENSMUSG00000086298	Gm11716	predicted gene 11716 [Source:MGI Symbol;Acc:MG	0.31089
ENSMUSG00000042211	Fbxo38	F-box protein 38 [Source:MGI Symbol;Acc:MGI:244	0.3109
ENSMUSG00000020674	Pxdn	peroxidasin homolog (Drosophila) [Source:MGI Sym	0.31095
ENSMUSG00000031835	Mbtps1	membrane-bound transcription factor peptidase, si	0.31102
ENSMUSG00000021366	Hivep1	human immunodeficiency virus type I enhancer bin	0.3111
ENSMUSG00000023991	Foxp4	forkhead box P4 [Source:MGI Symbol;Acc:MGI:192	0.31112
ENSMUSG00000029703	Lrwd1	leucine-rich repeats and WD repeat domain contai	0.31117
ENSMUSG00000092009	Myh15	myosin, heavy chain 15 [Source:MGI Symbol;Acc:M	0.31122
ENSMUSG00000022324	Matn2	matrilin 2 [Source:MGI Symbol;Acc:MGI:109613]	0.3114
ENSMUSG00000026601	Axdnd1	axonemal dynein light chain domain containing 1 [S	0.31155
ENSMUSG00000005763	Cd247	CD247 antigen [Source:MGI Symbol;Acc:MGI:8833	0.31167
ENSMUSG00000019489	Cd70	CD70 antigen [Source:MGI Symbol;Acc:MGI:119527	0.31177
ENSMUSG00000052798	Nup107	nucleoporin 107 [Source:MGI Symbol;Acc:MGI:214	0.31179
ENSMUSG00000061848	Gm5805	predicted gene 5805 [Source:MGI Symbol;Acc:MGI:	0.31182
ENSMUSG00000042474	Faim3	Fas apoptotic inhibitory molecule 3 [Source:MGI Sy	0.31189
ENSMUSG00000055368	Slc6a2	solute carrier family 6 (neurotransmitter transporte	0.31189
ENSMUSG00000051166	Eml5	echinoderm microtubule associated protein like 5 [	0.3119
ENSMUSG00000032315	Cyp1a1	cytochrome P450, family 1, subfamily a, polypeptid	0.31191
ENSMUSG00000017707	Serinc3	serine incorporator 3 [Source:MGI Symbol;Acc:MGI	0.31199
ENSMUSG00000068114	Ccdc134	coiled-coil domain containing 134 [Source:MGI Sym	0.31204
ENSMUSG00000017631	Abr	active BCR-related gene [Source:MGI Symbol;Acc:M	0.31209
ENSMUSG00000061039	Olfr920	olfactory receptor 920 [Source:MGI Symbol;Acc:MG	0.31211
ENSMUSG00000033961	Zfp446	zinc finger protein 446 [Source:MGI Symbol;Acc:MG	0.31213
ENSMUSG00000040943	Tet2	tet methylcytosine dioxygenase 2 [Source:MGI Sym	0.31215
ENSMUSG00000080885	Rpl10-ps6	ribosomal protein L10, pseudogene 6 [Source:MGI	0.31222
ENSMUSG00000037202	Prf1	perforin 1 (pore forming protein) [Source:MGI Sym	0.31224
ENSMUSG00000038372	Gmds	GDP-mannose 4, 6-dehydratase [Source:MGI Symb	0.31224
ENSMUSG00000015363	Trabd	TraB domain containing [Source:MGI Symbol;Acc:M	0.31229
ENSMUSG00000028011	Tdo2	tryptophan 2,3-dioxygenase [Source:MGI Symbol;A	0.31229
ENSMUSG00000030909	Anks4b	ankyrin repeat and sterile alpha motif domain cont	0.31229
ENSMUSG00000085289	Gm15337	predicted gene 15337 [Source:MGI Symbol;Acc:MG	0.31229
ENSMUSG00000083752	Gm12014	predicted gene 12014 [Source:MGI Symbol;Acc:MG	0.3123
ENSMUSG00000088575	Gm24537	predicted gene, 24537 [Source:MGI Symbol;Acc:MG	0.31232
ENSMUSG00000026017	Carf	calcium response factor [Source:MGI Symbol;Acc:M	0.31237
ENSMUSG00000028749	Pla2g2f	phospholipase A2, group IIF [Source:MGI Symbol;A	0.31239
ENSMUSG00000037196	Pacrg	PARK2 co-regulated [Source:MGI Symbol;Acc:MGI:1	0.31239
ENSMUSG00000097543	Gm805	predicted gene 805 [Source:MGI Symbol;Acc:MGI:2	0.31243
ENSMUSG00000089498	Gm26359	predicted gene, 26359 [Source:MGI Symbol;Acc:MG	0.31247
ENSMUSG00000059939	9430015G10Rik	RIKEN cDNA 9430015G10 gene [Source:MGI Symbo	0.31262
ENSMUSG00000003135	Cnot11	CCR4-NOT transcription complex, subunit 11 [Sour	0.31271

ENSMUSG00000090001	Gm9719	predicted gene 9719 [Source:MGI Symbol;Acc:MGI:	0.31275
ENSMUSG00000024677	Ms4a6b	membrane-spanning 4-domains, subfamily A, mem	0.3128
ENSMUSG00000050890	Pdik1l	PDLIM1 interacting kinase 1 like [Source:MGI Symb	0.31288
ENSMUSG00000028019	Pdgfc	platelet-derived growth factor, C polypeptide [Sour	0.31289
ENSMUSG00000029765	Plxna4	plexin A4 [Source:MGI Symbol;Acc:MGI:2179061]	0.31297
ENSMUSG00000037750	Fam222b	family with sequence similarity 222, member B [Sou	0.31297
ENSMUSG00000053565	Eif3k	eukaryotic translation initiation factor 3, subunit K	0.31298
ENSMUSG00000000826	Dnajc5	DnaJ (Hsp40) homolog, subfamily C, member 5 [Sou	0.313
ENSMUSG00000086937	Gm15063	predicted gene 15063 [Source:MGI Symbol;Acc:MG	0.31301
ENSMUSG00000087552	Gm15819	predicted gene 15819 [Source:MGI Symbol;Acc:MG	0.31304
ENSMUSG00000020525	Ppm1d	protein phosphatase 1D magnesium-dependent, de	0.31307
ENSMUSG00000097016	Gm26708	predicted gene, 26708 [Source:MGI Symbol;Acc:M(	0.31307
ENSMUSG00000029657	Hsph1	heat shock 105kDa/110kDa protein 1 [Source:MGI !	0.31314
ENSMUSG00000021504	B4galt7	xylosylprotein beta1,4-galactosyltransferase, polyp	0.31316
ENSMUSG00000020888	Dvl2	dishevelled 2, dsh homolog (Drosophila) [Source:M	0.31321
ENSMUSG00000029074	Ttll10	tubulin tyrosine ligase-like family, member 10 [Sou	0.31322
ENSMUSG00000079008	Gm14124	predicted gene 14124 [Source:MGI Symbol;Acc:MG	0.31324
ENSMUSG00000005813	Metap1	methionyl aminopeptidase 1 [Source:MGI Symbol;A	0.31327
ENSMUSG00000067919	Rex2	reduced expression 2 [Source:MGI Symbol;Acc:MG	0.31328
ENSMUSG00000021094	Dhrs7	dehydrogenase/reductase (SDR family) member 7 [	0.31333
ENSMUSG00000021266	Wars	tryptophanyl-tRNA synthetase [Source:MGI Symbo	0.3134
ENSMUSG00000051256	Jagn1	jagunal homolog 1 (Drosophila) [Source:MGI Symbo	0.31347
ENSMUSG00000048304	Slitrk3	SLIT and NTRK-like family, member 3 [Source:MGI S	0.3135
ENSMUSG00000070583	Fv1	Friend virus susceptibility 1 [Source:MGI Symbol;Ac	0.3135
ENSMUSG00000032014	Oaf	OAF homolog (Drosophila) [Source:MGI Symbol;Acc	0.31352
ENSMUSG00000074824	Rslcan18	regulator of sex-limitation candidate 18 [Source:M(	0.31361
ENSMUSG00000021130	Galnt16	UDP-N-acetyl-alpha-D-galactosamine:polypeptide N	0.31377
ENSMUSG00000053749	Gm9920	predicted gene 9920 [Source:MGI Symbol;Acc:MGI:	0.31398
ENSMUSG00000025381	Cnpy2	canopy 2 homolog (zebrafish) [Source:MGI Symbol;	0.31399
ENSMUSG00000052026	Slc6a7	solute carrier family 6 (neurotransmitter transporte	0.31407
ENSMUSG00000039701	Usp53	ubiquitin specific peptidase 53 [Source:MGI Symbo	0.31408
ENSMUSG00000086245	Gm16170	predicted gene 16170 [Source:MGI Symbol;Acc:MG	0.31411
ENSMUSG00000083594	Gm13722	predicted gene 13722 [Source:MGI Symbol;Acc:MG	0.31414
ENSMUSG00000067455	Hist1h4j	histone cluster 1, H4j [Source:MGI Symbol;Acc:MGI	0.31431
ENSMUSG00000050732	Vamp8	vesicle-associated membrane protein 8 [Source:M(	0.31433
ENSMUSG00000029578	Wipi2	WD repeat domain, phosphoinositide interacting 2	0.31434
ENSMUSG00000042367	Gjb3	gap junction protein, beta 3 [Source:MGI Symbol;A	0.31442
ENSMUSG00000087231	E230016M11Rik	RIKEN cDNA E230016M11 gene [Source:MGI Symbo	0.31446
ENSMUSG00000028962	Slc4a2	solute carrier family 4 (anion exchanger), member :	0.31452
ENSMUSG00000085847	Gm14540	predicted gene 14540 [Source:MGI Symbol;Acc:MG	0.31453
ENSMUSG00000040204	2810417H13Rik	RIKEN cDNA 2810417H13 gene [Source:MGI Symbo	0.31455
ENSMUSG00000094910	D430019H16Rik	RIKEN cDNA D430019H16 gene [Source:MGI Symbo	0.31458
ENSMUSG00000050447	Lypd6	LY6/PLAUR domain containing 6 [Source:MGI Symb	0.3146
ENSMUSG00000022382	Wnt7b	wingless-related MMTV integration site 7B [Source	0.31469
ENSMUSG00000046719	Nxph3	neurexophilin 3 [Source:MGI Symbol;Acc:MGI:1336	0.31473
ENSMUSG00000048776	Pthlh	parathyroid hormone-like peptide [Source:MGI Syn	0.31475
ENSMUSG00000021785	Ngly1	N-glycanase 1 [Source:MGI Symbol;Acc:MGI:19132	0.31479

ENSMUSG00000053536	Cstf2t	cleavage stimulation factor, 3' pre-RNA subunit 2, t	0.315
ENSMUSG00000040124	Gorab	golgin, RAB6-interacting [Source:MGI Symbol;Acc:N	0.31506
ENSMUSG00000024939	Fam89b	family with sequence similarity 89, member B [Sou	0.31517
ENSMUSG00000027087	Itgav	integrin alpha V [Source:MGI Symbol;Acc:MGI:9660	0.31527
ENSMUSG00000025474	Tubgcp2	tubulin, gamma complex associated protein 2 [Sou	0.31535
ENSMUSG00000029090	Gpr125	G protein-coupled receptor 125 [Source:MGI Symb	0.31535
ENSMUSG00000074575	Kcng1	potassium voltage-gated channel, subfamily G, mer	0.31549
ENSMUSG00000006599	Gtf2h1	general transcription factor II H, polypeptide 1 [Sou	0.31555
ENSMUSG00000034544	Rsrc1	arginine/serine-rich coiled-coil 1 [Source:MGI Symb	0.31555
ENSMUSG00000056427	Slit3	slit homolog 3 (Drosophila) [Source:MGI Symbol;Ac	0.31577
ENSMUSG00000053411	Cbx7	chromobox 7 [Source:MGI Symbol;Acc:MGI:119643	0.31579
ENSMUSG00000033177	Tmprss7	transmembrane serine protease 7 [Source:MGI Syn	0.3158
ENSMUSG00000041431	Ccnb1	cyclin B1 [Source:MGI Symbol;Acc:MGI:88302]	0.3158
ENSMUSG00000040414	Slc25a28	solute carrier family 25, member 28 [Source:MGI Sy	0.31589
ENSMUSG00000033826	Dnah8	dynein, axonemal, heavy chain 8 [Source:MGI Symb	0.3159
ENSMUSG00000022415	Syngr1	synaptogyrin 1 [Source:MGI Symbol;Acc:MGI:13283	0.31601
ENSMUSG00000022769	Sdf2l1	stromal cell-derived factor 2-like 1 [Source:MGI Syn	0.31613
ENSMUSG00000036632	Alg5	asparagine-linked glycosylation 5 (dolichyl-phospha	0.31623
ENSMUSG00000027408	Cpxm1	carboxypeptidase X 1 (M14 family) [Source:MGI Syn	0.31624
ENSMUSG00000047749	Zc3hav1l	zinc finger CCCH-type, antiviral 1-like [Source:MGI S	0.31626
ENSMUSG00000038375	Trp53inp2	transformation related protein 53 inducible nuclear	0.31633
ENSMUSG00000094719	Gm5108	predicted gene 5108 [Source:MGI Symbol;Acc:MGI:	0.31635
ENSMUSG00000042502	Cd2bp2	CD2 antigen (cytoplasmic tail) binding protein 2 [So	0.31636
ENSMUSG00000026843	Fubp3	far upstream element (FUSE) binding protein 3 [Sou	0.31652
ENSMUSG00000085784	Gm12158	predicted gene 12158 [Source:MGI Symbol;Acc:MG	0.31653
ENSMUSG00000082420	Gm6517	predicted gene 6517 [Source:MGI Symbol;Acc:MGI:	0.31664
ENSMUSG00000030007	Cct7	chaperonin containing Tcp1, subunit 7 (eta) [Source	0.31666
ENSMUSG00000030866	Ern2	endoplasmic reticulum (ER) to nucleus signalling 2 [	0.31668
ENSMUSG00000037225	Fgf2	fibroblast growth factor 2 [Source:MGI Symbol;Acc	0.31682
ENSMUSG00000037681	Esyt3	extended synaptotagmin-like protein 3 [Source:MG	0.31692
ENSMUSG00000003458	Ncstn	nicastrin [Source:MGI Symbol;Acc:MGI:1891700]	0.31704
ENSMUSG00000026594	Ralgps2	Ral GEF with PH domain and SH3 binding motif 2 [S	0.31711
ENSMUSG00000004651	Tyr	tyrosinase [Source:MGI Symbol;Acc:MGI:98880]	0.31724
ENSMUSG00000094296	Gm21798	predicted gene, 21798 [Source:MGI Symbol;Acc:MG	0.31749
ENSMUSG00000093405	Gm20684	predicted gene 20684 [Source:MGI Symbol;Acc:MG	0.31752
ENSMUSG00000010592	Dazl	deleted in azoospermia-like [Source:MGI Symbol;Ac	0.31754
ENSMUSG00000081657	Gm15466	predicted gene 15466 [Source:MGI Symbol;Acc:MG	0.3176
ENSMUSG00000045075	Gm9796	predicted gene 9796 [Source:MGI Symbol;Acc:MGI:	0.31765
ENSMUSG00000089129	Gm26407	predicted gene, 26407 [Source:MGI Symbol;Acc:MG	0.31766
ENSMUSG00000079108	Srp54b	signal recognition particle 54B [Source:MGI Symbol	0.31768
ENSMUSG00000031997	Trpc6	transient receptor potential cation channel, subfam	0.31769
ENSMUSG00000024474	Ik	IK cytokine [Source:MGI Symbol;Acc:MGI:1345142]	0.31774
ENSMUSG00000010277	2610507B11Rik	RIKEN cDNA 2610507B11 gene [Source:MGI Symbo	0.31786
ENSMUSG00000051238	Swsap1	SWIM type zinc finger 7 associated protein 1 [Sourc	0.31787
ENSMUSG00000075256	Cerkl	ceramide kinase-like [Source:MGI Symbol;Acc:MGI:	0.31793
ENSMUSG00000028414	Fktn	fukutin [Source:MGI Symbol;Acc:MGI:2179507]	0.31794
ENSMUSG00000074771	Ankef1	ankyrin repeat and EF-hand domain containing 1 [S	0.31796

ENSMUSG00000028333	Anp32b	acidic (leucine-rich) nuclear phosphoprotein 32 fam	0.31802
ENSMUSG00000024308	Tapbp	TAP binding protein [Source:MGI Symbol;Acc:MGI:1	0.31815
ENSMUSG00000023984	Gm20517	predicted gene 20517 [Source:MGI Symbol;Acc:MG	0.31821
ENSMUSG00000057897	Camk2b	calcium/calmodulin-dependent protein kinase II, be	0.31834
ENSMUSG00000047228	BC048546	cDNA sequence BC048546 [Source:MGI Symbol;Acc	0.31836
ENSMUSG00000022462	Slc38a2	solute carrier family 38, member 2 [Source:MGI Syr	0.31845
ENSMUSG00000020089	Ppa1	pyrophosphatase (inorganic) 1 [Source:MGI Symbo	0.31846
ENSMUSG00000031910	Has3	hyaluronan synthase 3 [Source:MGI Symbol;Acc:MG	0.31852
ENSMUSG00000039607	Rbms3	RNA binding motif, single stranded interacting prot	0.31855
ENSMUSG00000025066	Sfr1	SWI5 dependent recombination repair 1 [Source:M	0.31856
ENSMUSG00000017950	Hnf4a	hepatic nuclear factor 4, alpha [Source:MGI Symbo	0.31857
ENSMUSG00000027522	Stx16	syntaxin 16 [Source:MGI Symbol;Acc:MGI:1923396]	0.31863
ENSMUSG00000035131	Brinp3	bone morphogenetic protein/retinoic acid inducible	0.31863
ENSMUSG00000064090	Vrk2	vaccinia related kinase 2 [Source:MGI Symbol;Acc:M	0.31903
ENSMUSG00000031012	Cask	calcium/calmodulin-dependent serine protein kinas	0.3191
ENSMUSG00000043510	Hscb	HscB iron-sulfur cluster co-chaperone homolog (E. c	0.31911
ENSMUSG00000028128	F3	coagulation factor III [Source:MGI Symbol;Acc:MGI:	0.31914
ENSMUSG00000074622	Mafb	v-maf musculoaponeurotic fibrosarcoma oncogene	0.31926
ENSMUSG00000059479	B3gnt8	UDP-GlcNAc:betaGal beta-1,3-N-acetylglucosaminy	0.31933
ENSMUSG00000034998	Foxn2	forkhead box N2 [Source:MGI Symbol;Acc:MGI:134	0.31934
ENSMUSG00000092465	Gm20472	predicted gene 20472 [Source:MGI Symbol;Acc:MG	0.31936
ENSMUSG00000052631	Sh2d6	SH2 domain containing 6 [Source:MGI Symbol;Acc:	0.31941
ENSMUSG00000014498	Ankrd52	ankyrin repeat domain 52 [Source:MGI Symbol;Acc	0.31945
ENSMUSG00000042423	Fbrs	fibrosin [Source:MGI Symbol;Acc:MGI:104648]	0.31947
ENSMUSG00000033808	Tmem87a	transmembrane protein 87A [Source:MGI Symbol;A	0.31948
ENSMUSG00000075706	Gpx4	glutathione peroxidase 4 [Source:MGI Symbol;Acc:	0.31955
ENSMUSG00000029028	Lrrc47	leucine rich repeat containing 47 [Source:MGI Syml	0.3196
ENSMUSG00000019320	Noxo1	NADPH oxidase organizer 1 [Source:MGI Symbol;Ac	0.31972
ENSMUSG00000083400	Gm11900	predicted gene 11900 [Source:MGI Symbol;Acc:MG	0.31976
ENSMUSG00000032291	Crabp1	cellular retinoic acid binding protein I [Source:MGI	0.31982
ENSMUSG00000027074	Slc43a3	solute carrier family 43, member 3 [Source:MGI Syr	0.3199
ENSMUSG00000034156	Bzrap1	benzodiazepine receptor associated protein 1 [Sour	0.31991
ENSMUSG00000038398	Upf3a	UPF3 regulator of nonsense transcripts homolog A	0.31996
ENSMUSG00000020361	Hspa4	heat shock protein 4 [Source:MGI Symbol;Acc:MGI:	0.32011
ENSMUSG00000097183	Gm17501	predicted gene, 17501 [Source:MGI Symbol;Acc:MG	0.32015
ENSMUSG00000034055	Phka1	phosphorylase kinase alpha 1 [Source:MGI Symbol;	0.32022
ENSMUSG00000082245	Gm6292	predicted gene 6292 [Source:MGI Symbol;Acc:MGI:	0.32033
ENSMUSG00000079415	Cntf	ciliary neurotrophic factor [Source:MGI Symbol;Acc	0.32035
ENSMUSG00000045374	Wdr81	WD repeat domain 81 [Source:MGI Symbol;Acc:MG	0.32043
ENSMUSG00000027698	Nceh1	neutral cholesterol ester hydrolase 1 [Source:MGI S	0.32055
ENSMUSG00000073371	Gm6594	predicted pseudogene 6594 [Source:MGI Symbol;A	0.32063
ENSMUSG00000050549	5730508B09Rik	RIKEN cDNA 5730508B09 gene [Source:MGI Symbo	0.32065
ENSMUSG00000036078	Sigmar1	sigma non-opioid intracellular receptor 1 [Source:M	0.3207
ENSMUSG00000039130	Zc3hc1	zinc finger, C3HC type 1 [Source:MGI Symbol;Acc:M	0.32078
ENSMUSG00000017132	Cyth1	cytohesin 1 [Source:MGI Symbol;Acc:MGI:1334257	0.3209
ENSMUSG00000093772	4931403E22Rik	RIKEN cDNA 4931403E22 gene [Source:MGI Symbo	0.3209
ENSMUSG00000033554	Dph5	DPH5 homolog (S. cerevisiae) [Source:MGI Symbol;	0.32098

ENSMUSG00000018820	Zfyve27	zinc finger, FYVE domain containing 27 [Source:MG	0.32108
ENSMUSG00000082330	Ldha-ps	lactate dehydrogenase A, pseudogene [Source:MGI	0.32111
ENSMUSG00000093606	B130034C11Rik	RIKEN cDNA B130034C11 gene [Source:MGI Symbo	0.32111
ENSMUSG00000091586	Cyp4f17	cytochrome P450, family 4, subfamily f, polypeptid	0.32116
ENSMUSG00000025938	Slco5a1	solute carrier organic anion transporter family, me	0.32122
ENSMUSG00000073481	2-Mar	mitochondrial amidoxime reducing component 2 [S	0.32122
ENSMUSG00000086328	2700033N17Rik	RIKEN cDNA 2700033N17 gene [Source:MGI Symbo	0.32122
ENSMUSG00000068917	Clk2	CDC-like kinase 2 [Source:MGI Symbol;Acc:MGI:10	0.32124
ENSMUSG00000079407	1700110I01Rik	RIKEN cDNA 1700110I01 gene [Source:MGI Symbol	0.3213
ENSMUSG00000033285	Wdr3	WD repeat domain 3 [Source:MGI Symbol;Acc:MGI	0.32133
ENSMUSG00000022370	Mrpl13	mitochondrial ribosomal protein L13 [Source:MGI S	0.32138
ENSMUSG00000047242	Taf9b	TAF9B RNA polymerase II, TATA box binding protei	0.32145
ENSMUSG00000006057	Atp5g1	ATP synthase, H+ transporting, mitochondrial F0 co	0.3216
ENSMUSG00000022012	Enox1	ecto-NOX disulfide-thiol exchanger 1 [Source:MGI S	0.3216
ENSMUSG00000090637	Gm6189	predicted gene 6189 [Source:MGI Symbol;Acc:MGI	0.32161
ENSMUSG00000000409	Lck	lymphocyte protein tyrosine kinase [Source:MGI Sy	0.32163
ENSMUSG00000043811	Rtn4r	reticulon 4 receptor [Source:MGI Symbol;Acc:MGI:	0.32163
ENSMUSG00000082593	Gm11331	predicted gene 11331 [Source:MGI Symbol;Acc:MG	0.32165
ENSMUSG00000039059	Hrh3	histamine receptor H3 [Source:MGI Symbol;Acc:M	0.32169
ENSMUSG00000022031	Elp3	elongator acetyltransferase complex subunit 3 [Sou	0.32183
ENSMUSG00000028980	H6pd	hexose-6-phosphate dehydrogenase (glucose 1-deh	0.32192
ENSMUSG00000046532	Ar	androgen receptor [Source:MGI Symbol;Acc:MGI:8	0.32192
ENSMUSG00000028479	Gne	glucosamine (UDP-N-acetyl)-2-epimerase/N-acetyl	0.32197
ENSMUSG00000028469	Npr2	natriuretic peptide receptor 2 [Source:MGI Symbol	0.32209
ENSMUSG00000042548	Asxl1	additional sex combs like 1 [Source:MGI Symbol;Ac	0.3221
ENSMUSG00000054418	2900041M22Rik	RIKEN cDNA 2900041M22 gene [Source:MGI Symb	0.32226
ENSMUSG00000036091	Hyal3	hyaluronoglucosaminidase 3 [Source:MGI Symbol;A	0.32228
ENSMUSG00000074164	Vmn2r-ps54	vomeronasal 2, receptor, pseudogene 54 [Source:M	0.32233
ENSMUSG00000030101	Sumf1	sulfatase modifying factor 1 [Source:MGI Symbol;A	0.32243
ENSMUSG00000063600	Egfem1	EGF-like and EMI domain containing 1 [Source:MGI	0.32249
ENSMUSG00000027222	Pex16	peroxisomal biogenesis factor 16 [Source:MGI Sym	0.32251
ENSMUSG00000024579	Pcyox1l	prenylcysteine oxidase 1 like [Source:MGI Symbol;A	0.32252
ENSMUSG00000042797	Aqp11	aquaporin 11 [Source:MGI Symbol;Acc:MGI:191358	0.32255
ENSMUSG00000046079	Lrrc8d	leucine rich repeat containing 8D [Source:MGI Sym	0.32256
ENSMUSG00000049202	4930515G13Rik	RIKEN cDNA 4930515G13 gene [Source:MGI Symbo	0.32257
ENSMUSG00000074129	Rpl13a	ribosomal protein L13A [Source:MGI Symbol;Acc:M	0.3226
ENSMUSG00000042035	Igsf3	immunoglobulin superfamily, member 3 [Source:M	0.32261
ENSMUSG00000050783	Htr1f	5-hydroxytryptamine (serotonin) receptor 1F [Sour	0.32265
ENSMUSG00000097142	Gm26778	predicted gene, 26778 [Source:MGI Symbol;Acc:M	0.32265
ENSMUSG00000021494	Ddx41	DEAD (Asp-Glu-Ala-Asp) box polypeptide 41 [Sourc	0.32268
ENSMUSG00000047166	Tigd5	tigger transposable element derived 5 [Source:MGI	0.32268
ENSMUSG00000048307	Ankrd46	ankyrin repeat domain 46 [Source:MGI Symbol;Acc	0.3227
ENSMUSG00000034171	Faah	fatty acid amide hydrolase [Source:MGI Symbol;Ac	0.32276
ENSMUSG00000066107	Gm12666	predicted gene 12666 [Source:MGI Symbol;Acc:MG	0.32282
ENSMUSG00000040624	Plekhg1	pleckstrin homology domain containing, family G (v	0.32286
ENSMUSG00000001588	Acap1	ArfGAP with coiled-coil, ankyrin repeat and PH dom	0.32297
ENSMUSG00000073164	2410018L13Rik	RIKEN cDNA 2410018L13 gene [Source:MGI Symbo	0.32297

ENSMUSG00000067049	Unc93a	unc-93 homolog A (C. elegans) [Source:MGI Symbo	0.32298
ENSMUSG00000035949	Fbxw2	F-box and WD-40 domain protein 2 [Source:MGI Sy	0.32299
ENSMUSG00000029371	Cxcl5	chemokine (C-X-C motif) ligand 5 [Source:MGI Syml	0.32301
ENSMUSG00000039783	Kmo	kynurenine 3-monooxygenase (kynurenine 3-hydro	0.32303
ENSMUSG00000041708	Mpped1	metallophosphoesterase domain containing 1 [Sou	0.32303
ENSMUSG00000026582	Sele	selectin, endothelial cell [Source:MGI Symbol;Acc:N	0.32304
ENSMUSG00000052013	Btla	B and T lymphocyte associated [Source:MGI Symbo	0.32309
ENSMUSG00000044390	Tigd3	tigger transposable element derived 3 [Source:MGI	0.3231
ENSMUSG00000044881	Coa4	cytochrome c oxidase assembly factor 4 [Source:M(	0.3232
ENSMUSG00000024007	Ppil1	peptidylprolyl isomerase (cyclophilin)-like 1 [Source	0.32322
ENSMUSG00000035247	Hectd1	HECT domain containing 1 [Source:MGI Symbol;Acc	0.32324
ENSMUSG00000034387	Ssu2	ssu-2 homolog (C. elegans) [Source:MGI Symbol;Ac	0.32336
ENSMUSG00000032356	Rasgrf1	RAS protein-specific guanine nucleotide-releasing f	0.32357
ENSMUSG00000050373	Snx21	sorting nexin family member 21 [Source:MGI Symb	0.3236
ENSMUSG00000042451	Mybph	myosin binding protein H [Source:MGI Symbol;Acc:	0.32365
ENSMUSG00000015806	Qdpr	quinoid dihydropteridine reductase [Source:MGI Sy	0.32367
ENSMUSG00000097197	9530027J09Rik	RIKEN cDNA 9530027J09 gene [Source:MGI Symbol	0.32369
ENSMUSG00000029026	Trp73	transformation related protein 73 [Source:MGI Sym	0.32374
ENSMUSG00000015127	Unkl	unkempt-like (Drosophila) [Source:MGI Symbol;Acc	0.32385
ENSMUSG00000074480	Mex3a	mex3 homolog A (C. elegans) [Source:MGI Symbol;/	0.32393
ENSMUSG00000058736	Gm10047	predicted gene 10047 [Source:MGI Symbol;Acc:MG	0.32399
ENSMUSG00000056872	Gm10011	predicted gene 10011 [Source:MGI Symbol;Acc:MG	0.32401
ENSMUSG00000042751	Nmnat2	nicotinamide nucleotide adenylyltransferase 2 [Sou	0.32414
ENSMUSG00000025784	Clec3b	C-type lectin domain family 3, member b [Source:M	0.32421
ENSMUSG00000024347	Psd2	pleckstrin and Sec7 domain containing 2 [Source:M	0.32439
ENSMUSG00000029656	C8b	complement component 8, beta polypeptide [Sourc	0.32442
ENSMUSG00000060147	Serpinb6a	serine (or cysteine) peptidase inhibitor, clade B, me	0.32442
ENSMUSG00000038751	Ptk6	PTK6 protein tyrosine kinase 6 [Source:MGI Symbol	0.32443
ENSMUSG00000030811	Fbxl19	F-box and leucine-rich repeat protein 19 [Source:M	0.32445
ENSMUSG00000032181	Scg3	secretogranin III [Source:MGI Symbol;Acc:MGI:103(	0.32451
ENSMUSG00000032112	Trappc4	trafficking protein particle complex 4 [Source:MGI S	0.32454
ENSMUSG00000041014	Nrg3	neuregulin 3 [Source:MGI Symbol;Acc:MGI:109716]	0.3246
ENSMUSG00000072809	9330160F10Rik	RIKEN cDNA 9330160F10 gene [Source:MGI Symbo	0.32478
ENSMUSG00000014232	Cluap1	clusterin associated protein 1 [Source:MGI Symbol;	0.32479
ENSMUSG00000028729	Ebna1bp2	EBNA1 binding protein 2 [Source:MGI Symbol;Acc:N	0.32489
ENSMUSG00000028068	Iqgap3	IQ motif containing GTPase activating protein 3 [So	0.32493
ENSMUSG00000030725	Lipt2	lipoyl(octanoyl) transferase 2 (putative) [Source:M(	0.32493
ENSMUSG00000030850	Ate1	arginyltransferase 1 [Source:MGI Symbol;Acc:MGI:1	0.32494
ENSMUSG00000073455	9030025P20Rik	RIKEN cDNA 9030025P20 gene [Source:MGI Symbo	0.32501
ENSMUSG00000097397	Gm16861	predicted gene, 16861 [Source:MGI Symbol;Acc:M(	0.32501
ENSMUSG00000030303	Far2	fatty acyl CoA reductase 2 [Source:MGI Symbol;Acc	0.32503
ENSMUSG00000032381	Fam96a	family with sequence similarity 96, member A [Sou	0.32503
ENSMUSG00000039084	Chad	chondroadherin [Source:MGI Symbol;Acc:MGI:109(	0.32507
ENSMUSG00000039364	Sectm1b	secreted and transmembrane 1B [Source:MGI Syml	0.32507
ENSMUSG00000051185	Fam174a	family with sequence similarity 174, member A [Sou	0.32509
ENSMUSG00000035873	Pawr	PRKC, apoptosis, WT1, regulator [Source:MGI Symb	0.32515
ENSMUSG00000051483	Cbr1	carbonyl reductase 1 [Source:MGI Symbol;Acc:MGI	0.32546

ENSMUSG00000040111	Gramd1b	GRAM domain containing 1B [Source:MGI Symbol;A	0.32549
ENSMUSG00000063001	Rps23-ps	ribosomal protein S23, pseudogene [Source:MGI Sy	0.3255
ENSMUSG00000058773	Hist1h1b	histone cluster 1, H1b [Source:MGI Symbol;Acc:MG	0.32552
ENSMUSG00000061242	Gm9104	predicted gene 9104 [Source:MGI Symbol;Acc:MGI:	0.32553
ENSMUSG00000027200	Sema6d	sema domain, transmembrane domain (TM), and c	0.32559
ENSMUSG00000047428	Dlk2	delta-like 2 homolog (Drosophila) [Source:MGI Sym	0.32563
ENSMUSG00000057982	Zfp809	zinc finger protein 809 [Source:MGI Symbol;Acc:MG	0.32563
ENSMUSG00000021710	Nln	neurolysin (metallopeptidase M3 family) [Source:M	0.3257
ENSMUSG00000001211	Agpat3	1-acylglycerol-3-phosphate O-acyltransferase 3 [So	0.3258
ENSMUSG00000009640	Dmap1	DNA methyltransferase 1-associated protein 1 [Sou	0.32582
ENSMUSG00000024740	Ddb1	damage specific DNA binding protein 1 [Source:MG	0.32582
ENSMUSG00000081056	Gm13998	predicted gene 13998 [Source:MGI Symbol;Acc:MG	0.32583
ENSMUSG00000097435	Gm26783	predicted gene, 26783 [Source:MGI Symbol;Acc:MG	0.32583
ENSMUSG00000033429	Mcee	methylmalonyl CoA epimerase [Source:MGI Symbo	0.32584
ENSMUSG00000090211	Gm16050	predicted gene 16050 [Source:MGI Symbol;Acc:MG	0.32593
ENSMUSG00000037486	Asxl2	additional sex combs like 2 (Drosophila) [Source:MG	0.32594
ENSMUSG00000030401	Rtn2	reticulon 2 (Z-band associated protein) [Source:MG	0.32611
ENSMUSG00000040055	Gjb6	gap junction protein, beta 6 [Source:MGI Symbol;A	0.32613
ENSMUSG00000074825	Itpripl1	inositol 1,4,5-triphosphate receptor interacting pro	0.32616
ENSMUSG00000030380	Mzf1	myeloid zinc finger 1 [Source:MGI Symbol;Acc:MGI	0.32634
ENSMUSG00000048174	Tmem81	transmembrane protein 81 [Source:MGI Symbol;Ac	0.32643
ENSMUSG00000063698	Sfxn4	sideroflexin 4 [Source:MGI Symbol;Acc:MGI:213768	0.32647
ENSMUSG00000091014	Gm5244	predicted pseudogene 5244 [Source:MGI Symbol;A	0.32647
ENSMUSG00000011658	Fuz	fuzzy homolog (Drosophila) [Source:MGI Symbol;Ac	0.32658
ENSMUSG00000025880	Smad7	SMAD family member 7 [Source:MGI Symbol;Acc:M	0.32663
ENSMUSG00000086782	E130102H24Rik	RIKEN cDNA E130102H24 gene [Source:MGI Symbo	0.32671
ENSMUSG00000039684	Gm5422	predicted pseudogene 5422 [Source:MGI Symbol;A	0.32675
ENSMUSG00000032040	Dcps	decapping enzyme, scavenger [Source:MGI Symbol	0.32678
ENSMUSG00000047989	Ino80c	INO80 complex subunit C [Source:MGI Symbol;Acc:	0.32743
ENSMUSG00000029287	Tgfbr3	transforming growth factor, beta receptor III [Sourc	0.32746
ENSMUSG00000097280	AI849053	expressed sequence AI849053 [Source:MGI Symbol	0.32758
ENSMUSG00000037752	Xkr8	X Kell blood group precursor related family membe	0.32764
ENSMUSG00000032777	Gtf3c1	general transcription factor III C 1 [Source:MGI Sym	0.32766
ENSMUSG00000039081	Zfp503	zinc finger protein 503 [Source:MGI Symbol;Acc:MG	0.3277
ENSMUSG00000057577	Gm10025	predicted gene 10025 [Source:MGI Symbol;Acc:MG	0.32782
ENSMUSG00000020844	Nxn	nucleoredoxin [Source:MGI Symbol;Acc:MGI:10933	0.32785
ENSMUSG00000037020	Wdr62	WD repeat domain 62 [Source:MGI Symbol;Acc:MG	0.32799
ENSMUSG00000064566	Gm24144	predicted gene, 24144 [Source:MGI Symbol;Acc:MG	0.32801
ENSMUSG00000085194	Gm16012	predicted gene 16012 [Source:MGI Symbol;Acc:MG	0.32802
ENSMUSG00000023931	Efhb	EF hand domain family, member B [Source:MGI Syr	0.32803
ENSMUSG00000072419	Dppa2	developmental pluripotency associated 2 [Source:N	0.32803
ENSMUSG00000024232	Bambi	BMP and activin membrane-bound inhibitor [Sourc	0.32804
ENSMUSG00000084800	Gm12205	predicted gene 12205 [Source:MGI Symbol;Acc:MG	0.32807
ENSMUSG00000020611	Gna13	guanine nucleotide binding protein, alpha 13 [Sour	0.32815
ENSMUSG00000037418	Best1	bestrophin 1 [Source:MGI Symbol;Acc:MGI:134633	0.32815
ENSMUSG00000015957	Wnt11	wingless-related MMTV integration site 11 [Source	0.32818
ENSMUSG00000015971	Actr8	ARP8 actin-related protein 8 [Source:MGI Symbol;A	0.32823

ENSMUSG00000032468	Armc8	armadillo repeat containing 8 [Source:MGI Symbol;	0.32823
ENSMUSG00000025317	Car5a	carbonic anhydrase 5a, mitochondrial [Source:MGI	0.32829
ENSMUSG00000017639	Rab11fip4	RAB11 family interacting protein 4 (class II) [Source	0.32833
ENSMUSG00000091082	Gm17130	predicted gene 17130 [Source:MGI Symbol;Acc:MG	0.32842
ENSMUSG00000028642	4930538K18Rik	RIKEN cDNA 4930538K18 gene [Source:MGI Symbo	0.32866
ENSMUSG00000031533	Mrps31	mitochondrial ribosomal protein S31 [Source:MGI S	0.3287
ENSMUSG00000038013	Wipf2	WAS/WASL interacting protein family, member 2 [S	0.32878
ENSMUSG00000089571	Gm23008	predicted gene, 23008 [Source:MGI Symbol;Acc:MG	0.32878
ENSMUSG00000033597	Caskin1	CASK interacting protein 1 [Source:MGI Symbol;Acc	0.3289
ENSMUSG00000034127	Tspan8	tetraspanin 8 [Source:MGI Symbol;Acc:MGI:238491	0.32904
ENSMUSG00000002748	Baz1b	bromodomain adjacent to zinc finger domain, 1B [S	0.32915
ENSMUSG00000027007	Ssfa2	sperm specific antigen 2 [Source:MGI Symbol;Acc:M	0.32924
ENSMUSG00000083396	Gm15542	predicted gene 15542 [Source:MGI Symbol;Acc:MG	0.32929
ENSMUSG00000057963	Itpk1	inositol 1,3,4-triphosphate 5/6 kinase [Source:MGI	0.3293
ENSMUSG00000085269	Gm15777	predicted gene 15777 [Source:MGI Symbol;Acc:MG	0.3293
ENSMUSG00000030042	Pole4	polymerase (DNA-directed), epsilon 4 (p12 subunit)	0.32932
ENSMUSG00000039197	Adk	adenosine kinase [Source:MGI Symbol;Acc:MGI:879	0.32941
ENSMUSG00000060206	Zfp462	zinc finger protein 462 [Source:MGI Symbol;Acc:MG	0.32941
ENSMUSG00000042804	Gpr153	G protein-coupled receptor 153 [Source:MGI Symb	0.32957
ENSMUSG00000074121	Ntf5	neurotrophin 5 [Source:MGI Symbol;Acc:MGI:9738	0.32962
ENSMUSG00000097800	B230344G16Rik	RIKEN cDNA B230344G16 gene [Source:MGI Symbo	0.32963
ENSMUSG00000009030	Pdcl	phosducin-like [Source:MGI Symbol;Acc:MGI:19147	0.32965
ENSMUSG00000049124	Gm8186	predicted gene 8186 [Source:MGI Symbol;Acc:MGI:	0.3297
ENSMUSG00000047371	Zfp768	zinc finger protein 768 [Source:MGI Symbol;Acc:MG	0.32971
ENSMUSG00000027189	Trim44	tripartite motif-containing 44 [Source:MGI Symbol;	0.32975
ENSMUSG00000005674	Tomm40l	translocase of outer mitochondrial membrane 40 h	0.32981
ENSMUSG00000042808	Gpx2	glutathione peroxidase 2 [Source:MGI Symbol;Acc:M	0.32984
ENSMUSG00000053024	Cntn2	contactin 2 [Source:MGI Symbol;Acc:MGI:104518]	0.32985
ENSMUSG00000024429	Gnl1	guanine nucleotide binding protein-like 1 [Source:M	0.32992
ENSMUSG00000033530	Ttc7b	tetratricopeptide repeat domain 7B [Source:MGI Sy	0.33005
ENSMUSG00000048707	Tprn	taperin [Source:MGI Symbol;Acc:MGI:2139535]	0.33006
ENSMUSG00000045996	Polr2k	polymerase (RNA) II (DNA directed) polypeptide K [	0.33011
ENSMUSG00000020411	Nipal4	NIPA-like domain containing 4 [Source:MGI Symbol	0.33016
ENSMUSG00000062438	Adam1b	a disintegrin and metallopeptidase domain 1b [Sou	0.33017
ENSMUSG00000015305	Sash1	SAM and SH3 domain containing 1 [Source:MGI Syr	0.33021
ENSMUSG00000053929	Cyhr1	cysteine and histidine rich 1 [Source:MGI Symbol;A	0.33021
ENSMUSG00000079010	Gm11032	predicted gene 11032 [Source:MGI Symbol;Acc:MG	0.33022
ENSMUSG00000027322	Siglec1	sialic acid binding Ig-like lectin 1, sialoadhesin [Sour	0.33027
ENSMUSG00000083630	Gm15602	predicted gene 15602 [Source:MGI Symbol;Acc:MG	0.33029
ENSMUSG00000004788	Eif2b2	eukaryotic translation initiation factor 2B, subunit 2	0.33041
ENSMUSG00000026768	Itga8	integrin alpha 8 [Source:MGI Symbol;Acc:MGI:1094	0.33041
ENSMUSG00000078493	A930039A15Rik	RIKEN cDNA A930039A15 gene [Source:MGI Symbo	0.33049
ENSMUSG00000035506	Slc12a8	solute carrier family 12 (potassium/chloride transpo	0.33051
ENSMUSG00000001467	Cyp51	cytochrome P450, family 51 [Source:MGI Symbol;A	0.33057
ENSMUSG00000006281	Tep1	telomerase associated protein 1 [Source:MGI Symb	0.33066
ENSMUSG00000028383	Hsdl2	hydroxysteroid dehydrogenase like 2 [Source:MGI S	0.33079
ENSMUSG00000080957	Gm15739	predicted gene 15739 [Source:MGI Symbol;Acc:MG	0.33085

ENSMUSG00000040913	Fbxw4	F-box and WD-40 domain protein 4 [Source:MGI Sy	0.3309
ENSMUSG00000020020	Usp44	ubiquitin specific peptidase 44 [Source:MGI Symbo	0.33093
ENSMUSG00000021222	Dcaf4	DDB1 and CUL4 associated factor 4 [Source:MGI Sy	0.33107
ENSMUSG00000040327	Cul9	cullin 9 [Source:MGI Symbol;Acc:MGI:1925559]	0.33112
ENSMUSG00000031749	St3gal2	ST3 beta-galactoside alpha-2,3-sialyltransferase 2 [	0.33113
ENSMUSG00000020653	Klf11	Kruppel-like factor 11 [Source:MGI Symbol;Acc:MG	0.33114
ENSMUSG00000079550	Mpp4	membrane protein, palmitoylated 4 (MAGUK p55 s	0.33131
ENSMUSG00000090247	Bloc1s1	biogenesis of lysosome-related organelles complex	0.33137
ENSMUSG00000085174	Gm16206	predicted gene 16206 [Source:MGI Symbol;Acc:MG	0.33157
ENSMUSG00000023156	Rpp14	ribonuclease P 14 subunit [Source:MGI Symbol;Acc	0.3316
ENSMUSG00000020576	Nbas	neuroblastoma amplified sequence [Source:MGI Sy	0.33169
ENSMUSG00000090004	Gm16125	predicted gene 16125 [Source:MGI Symbol;Acc:MG	0.3317
ENSMUSG00000002266	Zim1	zinc finger, imprinted 1 [Source:MGI Symbol;Acc:M	0.33174
ENSMUSG00000001021	S100a3	S100 calcium binding protein A3 [Source:MGI Symb	0.33179
ENSMUSG00000000215	Ins2	insulin II [Source:MGI Symbol;Acc:MGI:96573]	0.33186
ENSMUSG00000041791	Capza3	capping protein (actin filament) muscle Z-line, alph	0.33195
ENSMUSG00000025330	Padi4	peptidyl arginine deiminase, type IV [Source:MGI Sy	0.33196
ENSMUSG00000047996	Prrg1	proline rich Gla (G-carboxyglutamic acid) 1 [Source:	0.33199
ENSMUSG00000032265	Fam46a	family with sequence similarity 46, member A [Sou	0.33201
ENSMUSG00000062580	Timm17a	translocase of inner mitochondrial membrane 17a	0.33204
ENSMUSG00000011832	Evi5l	ecotropic viral integration site 5 like [Source:MGI Sy	0.33219
ENSMUSG00000020225	Tmbim4	transmembrane BAX inhibitor motif containing 4 [S	0.3322
ENSMUSG00000085178	1700064E03Rik	RIKEN cDNA 1700064E03 gene [Source:MGI Symbo	0.33221
ENSMUSG00000078907	Fam186b	family with sequence similarity 186, member B [Sou	0.33226
ENSMUSG00000015702	Anxa9	annexin A9 [Source:MGI Symbol;Acc:MGI:1923711]	0.33239
ENSMUSG00000031821	Gins2	GINS complex subunit 2 (Psf2 homolog) [Source:MG	0.33245
ENSMUSG00000089844	A530032D15Rik	RIKEN cDNA A530032D15Rik gene [Source:MGI Syn	0.33247
ENSMUSG00000025277	Abhd6	abhydrolase domain containing 6 [Source:MGI Sym	0.33257
ENSMUSG00000034311	Kif4	kinesin family member 4 [Source:MGI Symbol;Acc:M	0.33258
ENSMUSG00000035314	Gdpd5	glycerophosphodiester phosphodiesterase domain	0.33259
ENSMUSG00000061904	Slc25a3	solute carrier family 25 (mitochondrial carrier, phos	0.33261
ENSMUSG00000046562	Unc119b	unc-119 homolog B (C. elegans) [Source:MGI Symb	0.33274
ENSMUSG00000026278	Bok	BCL2-related ovarian killer protein [Source:MGI Syn	0.33279
ENSMUSG00000087138	Gm15545	predicted gene 15545 [Source:MGI Symbol;Acc:MG	0.33279
ENSMUSG00000033439	Trmt13	tRNA methyltransferase 13 [Source:MGI Symbol;Ac	0.33282
ENSMUSG00000060969	Irx1	Iroquois related homeobox 1 (Drosophila) [Source:	0.33287
ENSMUSG00000015337	Endog	endonuclease G [Source:MGI Symbol;Acc:MGI:126	0.33291
ENSMUSG00000093656	Gm20628	predicted gene 20628 [Source:MGI Symbol;Acc:MG	0.33293
ENSMUSG00000067365	Tmem128	transmembrane protein 128 [Source:MGI Symbol;A	0.33311
ENSMUSG00000037960	1110007C09Rik	RIKEN cDNA 1110007C09 gene [Source:MGI Symbo	0.33315
ENSMUSG00000036908	Unc93b1	unc-93 homolog B1 (C. elegans) [Source:MGI Symb	0.33318
ENSMUSG00000032806	Slc10a3	solute carrier family 10 (sodium/bile acid cotransp	0.33336
ENSMUSG00000070337	Gpr179	G protein-coupled receptor 179 [Source:MGI Symb	0.33339
ENSMUSG00000050836	Ntng1	netrin G1 [Source:MGI Symbol;Acc:MGI:1934028]	0.33341
ENSMUSG00000097455	Gm26891	predicted gene, 26891 [Source:MGI Symbol;Acc:M	0.33344
ENSMUSG00000052557	Gan	giant axonal neuropathy [Source:MGI Symbol;Acc:M	0.33349
ENSMUSG00000017376	Nlk	nemo like kinase [Source:MGI Symbol;Acc:MGI:120	0.33355

ENSMUSG00000025056	Nr0b1	nuclear receptor subfamily 0, group B, member 1 [S	0.33357
ENSMUSG00000063254	B230325K18Rik	RIKEN cDNA B230325K18 gene [Source:MGI Symbo	0.33365
ENSMUSG00000021243	Fcf1	FCF1 small subunit (SSU) processome component h	0.33372
ENSMUSG00000073753	Gm10571	predicted gene 10571 [Source:MGI Symbol;Acc:MG	0.33376
ENSMUSG00000075224	Lrrc55	leucine rich repeat containing 55 [Source:MGI Symb	0.33377
ENSMUSG00000059291	Rpl11	ribosomal protein L11 [Source:MGI Symbol;Acc:MG	0.33382
ENSMUSG00000081930	Gm12345	predicted gene 12345 [Source:MGI Symbol;Acc:MG	0.33384
ENSMUSG00000029283	Cdc7	cell division cycle 7 (S. cerevisiae) [Source:MGI Sym	0.33388
ENSMUSG00000097584	1700125G22Rik	RIKEN cDNA 1700125G22 gene [Source:MGI Symbo	0.33389
ENSMUSG00000049742	C330006D17Rik	RIKEN cDNA C330006D17 gene [Source:MGI Symbo	0.33391
ENSMUSG00000045257	Morn2	MORN repeat containing 2 [Source:MGI Symbol;Ac	0.33394
ENSMUSG00000005374	Tbl2	transducin (beta)-like 2 [Source:MGI Symbol;Acc:M	0.33399
ENSMUSG00000097588	Gm17388	predicted gene, 17388 [Source:MGI Symbol;Acc:M	0.33405
ENSMUSG00000038807	Rap1gap2	RAP1 GTPase activating protein 2 [Source:MGI Sym	0.33411
ENSMUSG00000097006	9530082P21Rik	RIKEN cDNA 9530082P21 gene [Source:MGI Symbo	0.33416
ENSMUSG00000014791	Elmo3	engulfment and cell motility 3 [Source:MGI Symbol	0.33419
ENSMUSG00000001166	Oas1c	2'-5' oligoadenylate synthetase 1C [Source:MGI Syr	0.33424
ENSMUSG00000086822	5330413P13Rik	RIKEN cDNA 5330413P13 gene [Source:MGI Symbo	0.33426
ENSMUSG00000041733	Coq5	coenzyme Q5 homolog, methyltransferase (yeast) [	0.33427
ENSMUSG00000048661	Lemd3	LEM domain containing 3 [Source:MGI Symbol;Acc:	0.33428
ENSMUSG00000049598	Vsig8	V-set and immunoglobulin domain containing 8 [So	0.33438
ENSMUSG00000046834	Krt1	keratin 1 [Source:MGI Symbol;Acc:MGI:96698]	0.33443
ENSMUSG00000026895	Ndufa8	NADH dehydrogenase (ubiquinone) 1 alpha subcom	0.33459
ENSMUSG00000056328	Myh1	myosin, heavy polypeptide 1, skeletal muscle, adult	0.33461
ENSMUSG00000020037	Rfx4	regulatory factor X, 4 (influences HLA class II expres	0.33467
ENSMUSG00000061232	H2-K1	histocompatibility 2, K1, K region [Source:MGI Sym	0.33469
ENSMUSG00000032480	Dhx30	DEAH (Asp-Glu-Ala-His) box polypeptide 30 [Source	0.33473
ENSMUSG00000078521	Aunip	aurora kinase A and ninein interacting protein [Sou	0.33478
ENSMUSG00000035699	Slc51a	solute carrier family 51, alpha subunit [Source:MGI	0.3348
ENSMUSG00000061482	Hist1h4d	histone cluster 1, H4d [Source:MGI Symbol;Acc:MG	0.33481
ENSMUSG00000080242	Gm15487	predicted gene 15487 [Source:MGI Symbol;Acc:MG	0.33484
ENSMUSG00000024118	1600002H07Rik	RIKEN cDNA 1600002H07 gene [Source:MGI Symbo	0.33494
ENSMUSG00000018479	1700125H20Rik	RIKEN cDNA 1700125H20 gene [Source:MGI Symbo	0.33497
ENSMUSG00000015452	Ager	advanced glycosylation end product-specific recept	0.33499
ENSMUSG00000053684	BC048403	cDNA sequence BC048403 [Source:MGI Symbol;Acc	0.33499
ENSMUSG00000081793	5730416F02Rik	RIKEN cDNA 5730416F02 gene [Source:MGI Symbo	0.33506
ENSMUSG00000029228	Lnx1	ligand of numb-protein X 1 [Source:MGI Symbol;Ac	0.3351
ENSMUSG00000074006	Omp	olfactory marker protein [Source:MGI Symbol;Acc:M	0.33511
ENSMUSG00000042115	Klhdc8a	kelch domain containing 8A [Source:MGI Symbol;A	0.33512
ENSMUSG00000085705	Gm16046	predicted gene 16046 [Source:MGI Symbol;Acc:MG	0.33512
ENSMUSG00000097482	Gm17634	predicted gene, 17634 [Source:MGI Symbol;Acc:M	0.33513
ENSMUSG00000002825	Qtrt1	queuine tRNA-ribosyltransferase 1 [Source:MGI Syr	0.33514
ENSMUSG00000051518	Rps19bp1	ribosomal protein S19 binding protein 1 [Source:M	0.33515
ENSMUSG00000085910	Gm12395	predicted gene 12395 [Source:MGI Symbol;Acc:MG	0.3352
ENSMUSG00000043760	Pkhd1	polycystic kidney and hepatic disease 1 [Source:MG	0.33556
ENSMUSG00000036197	Gxylt1	glucoside xylosyltransferase 1 [Source:MGI Symbol	0.33558
ENSMUSG00000021294	Kif26a	kinesin family member 26A [Source:MGI Symbol;Ac	0.33563

ENSMUSG00000086188	Gm15169	predicted gene 15169 [Source:MGI Symbol;Acc:MG	0.33564
ENSMUSG00000025934	Gsta3	glutathione S-transferase, alpha 3 [Source:MGI Sym	0.33566
ENSMUSG00000049401	Ogfr	opioid growth factor receptor [Source:MGI Symbol;	0.33583
ENSMUSG00000097834	Gm26911	predicted gene, 26911 [Source:MGI Symbol;Acc:MG	0.33585
ENSMUSG00000076964	Gm23252	predicted gene, 23252 [Source:MGI Symbol;Acc:MG	0.33589
ENSMUSG00000019933	2310015B20Rik	RIKEN cDNA 2310015B20 gene [Source:MGI Symbo	0.33593
ENSMUSG00000009115	Spatc1l	spermatogenesis and centriole associated 1 like [So	0.33595
ENSMUSG00000063714	1700011J10Rik	RIKEN cDNA 1700011J10 gene [Source:MGI Symbol	0.33596
ENSMUSG00000040703	Cyp2s1	cytochrome P450, family 2, subfamily s, polypeptid	0.33602
ENSMUSG00000090778	Gm3235	predicted gene 3235 [Source:MGI Symbol;Acc:MGI:	0.33638
ENSMUSG00000024580	Grpel2	GrpE-like 2, mitochondrial [Source:MGI Symbol;Acc	0.33639
ENSMUSG00000028999	Rint1	RAD50 interactor 1 [Source:MGI Symbol;Acc:MGI:1	0.33639
ENSMUSG00000021609	Slc6a3	solute carrier family 6 (neurotransmitter transporte	0.33644
ENSMUSG00000030310	Slc6a1	solute carrier family 6 (neurotransmitter transporte	0.33645
ENSMUSG00000033940	Brk1	BRICK1, SCAR/WAVE actin-nucleating complex subu	0.33661
ENSMUSG00000065530	Mir99a	microRNA 99a [Source:MGI Symbol;Acc:MGI:26769	0.33661
ENSMUSG00000006576	Slc4a3	solute carrier family 4 (anion exchanger), member 3	0.33679
ENSMUSG00000005628	Tmod4	tropomodulin 4 [Source:MGI Symbol;Acc:MGI:1355	0.33697
ENSMUSG00000085586	Gm11613	predicted gene 11613 [Source:MGI Symbol;Acc:MG	0.33715
ENSMUSG00000032648	Pygm	muscle glycogen phosphorylase [Source:MGI Symbo	0.33734
ENSMUSG00000097254	C430042M11Rik	RIKEN cDNA C430042M11 gene [Source:MGI Symbo	0.33735
ENSMUSG00000008668	Rps18	ribosomal protein S18 [Source:MGI Symbol;Acc:MG	0.3374
ENSMUSG00000035051	Dhx57	DEAH (Asp-Glu-Ala-Asp/His) box polypeptide 57 [So	0.3374
ENSMUSG00000086206	Gm13783	predicted gene 13783 [Source:MGI Symbol;Acc:MG	0.3374
ENSMUSG00000083182	Gm13828	predicted gene 13828 [Source:MGI Symbol;Acc:MG	0.33751
ENSMUSG00000029364	Wsb2	WD repeat and SOCS box-containing 2 [Source:MGI	0.33755
ENSMUSG00000062168	Ppef1	protein phosphatase with EF hand calcium-binding	0.33755
ENSMUSG00000083176	D11Bhm181e	DNA segment, Chr 11, Boehm 181, expressed [Sour	0.33762
ENSMUSG00000020715	Ern1	endoplasmic reticulum (ER) to nucleus signalling 1 [	0.33775
ENSMUSG00000021239	Vsx2	visual system homeobox 2 [Source:MGI Symbol;Acc	0.33775
ENSMUSG00000084711	Gm24629	predicted gene, 24629 [Source:MGI Symbol;Acc:MG	0.33775
ENSMUSG00000074206	Adh6b	alcohol dehydrogenase 6B (class V) [Source:MGI Sy	0.3378
ENSMUSG00000021288	Klc1	kinesin light chain 1 [Source:MGI Symbol;Acc:MGI:1	0.33781
ENSMUSG00000023995	Tspo2	translocator protein 2 [Source:MGI Symbol;Acc:MG	0.33786
ENSMUSG00000032119	Hinfp	histone H4 transcription factor [Source:MGI Symbo	0.33789
ENSMUSG00000024742	Fen1	flap structure specific endonuclease 1 [Source:MGI	0.33791
ENSMUSG00000001249	Hpn	hepsin [Source:MGI Symbol;Acc:MGI:1196620]	0.33794
ENSMUSG00000055067	Smyd3	SET and MYND domain containing 3 [Source:MGI Sy	0.33803
ENSMUSG00000087425	Gm15404	predicted gene 15404 [Source:MGI Symbol;Acc:MG	0.33808
ENSMUSG00000044726	BC030476	cDNA sequence BC030476 [Source:MGI Symbol;Acc	0.33811
ENSMUSG00000022553	Maf1	MAF1 homolog (S. cerevisiae) [Source:MGI Symbol;	0.33812
ENSMUSG00000014313	Cox6c	cytochrome c oxidase subunit VIc [Source:MGI Sym	0.33813
ENSMUSG00000030691	Fchsd2	FCH and double SH3 domains 2 [Source:MGI Symbo	0.33814
ENSMUSG00000027298	Tyro3	TYRO3 protein tyrosine kinase 3 [Source:MGI Symb	0.33819
ENSMUSG00000022610	Mapk12	mitogen-activated protein kinase 12 [Source:MGI S	0.33823
ENSMUSG00000027996	Sfrp2	secreted frizzled-related protein 2 [Source:MGI Sym	0.33823
ENSMUSG00000084051	Gm15146	predicted gene 15146 [Source:MGI Symbol;Acc:MG	0.33823

ENSMUSG00000085256	3010003L10Rik	RIKEN cDNA 3010003L10 gene [Source:MGI Symbo	0.33823
ENSMUSG00000078570	1110065P20Rik	RIKEN cDNA 1110065P20 gene [Source:MGI Symbo	0.33824
ENSMUSG00000021767	Kat6b	K(lysine) acetyltransferase 6B [Source:MGI Symbol;	0.33826
ENSMUSG00000074591	Gm410	predicted gene 410 [Source:MGI Symbol;Acc:MGI:2	0.33834
ENSMUSG00000034432	Cops8	COP9 (constitutive photomorphogenic) homolog, s	0.33835
ENSMUSG00000036362	P2ry13	purinergic receptor P2Y, G-protein coupled 13 [Sou	0.33838
ENSMUSG00000080396	Snord111	small nucleolar RNA, C/D box 111 [Source:MGI Sym	0.33842
ENSMUSG00000003863	Ppfia3	protein tyrosine phosphatase, receptor type, f poly	0.33843
ENSMUSG00000063317	Usp31	ubiquitin specific peptidase 31 [Source:MGI Symbo	0.33848
ENSMUSG00000028944	Prkag2	protein kinase, AMP-activated, gamma 2 non-catal	0.33854
ENSMUSG00000059316	Slc27a4	solute carrier family 27 (fatty acid transporter), me	0.3386
ENSMUSG00000049800	Sertad2	SERTA domain containing 2 [Source:MGI Symbol;Ac	0.33877
ENSMUSG00000098039	Gm26925	predicted gene, 26925 [Source:MGI Symbol;Acc:M	0.3388
ENSMUSG00000097713	Gm7741	predicted gene 7741 [Source:MGI Symbol;Acc:MGI:	0.33881
ENSMUSG00000020673	Tpo	thyroid peroxidase [Source:MGI Symbol;Acc:MGI:9	0.33889
ENSMUSG00000043795	Gm14492	predicted gene 14492 [Source:MGI Symbol;Acc:MG	0.33893
ENSMUSG00000021874	4933413J09Rik	RIKEN cDNA 4933413J09 gene [Source:MGI Symbol	0.33897
ENSMUSG00000015023	Ddx19a	DEAD (Asp-Glu-Ala-Asp) box polypeptide 19a [Sour	0.33905
ENSMUSG00000006215	Zbtb17	zinc finger and BTB domain containing 17 [Source:M	0.33925
ENSMUSG00000082057	Gm15789	predicted gene 15789 [Source:MGI Symbol;Acc:MG	0.33929
ENSMUSG00000083044	Gm12416	predicted gene 12416 [Source:MGI Symbol;Acc:MG	0.33937
ENSMUSG00000031171	Ftsj1	FtsJ homolog 1 (E. coli) [Source:MGI Symbol;Acc:M	0.33938
ENSMUSG00000044986	Tst	thiosulfate sulfurtransferase, mitochondrial [Source	0.33941
ENSMUSG00000033007	Asic4	acid-sensing (proton-gated) ion channel family mer	0.33942
ENSMUSG00000065211	Gm26265	predicted gene, 26265 [Source:MGI Symbol;Acc:M	0.33952
ENSMUSG00000001525	Tubb5	tubulin, beta 5 class I [Source:MGI Symbol;Acc:MGI	0.33953
ENSMUSG00000031641	Cbr4	carbonyl reductase 4 [Source:MGI Symbol;Acc:MGI	0.33956
ENSMUSG00000030697	Ppp4c	protein phosphatase 4, catalytic subunit [Source:M	0.33961
ENSMUSG00000096977	Gm10827	predicted gene 10827 [Source:MGI Symbol;Acc:MG	0.33962
ENSMUSG00000070283	Ndufaf3	NADH dehydrogenase (ubiquinone) 1 alpha subcom	0.33963
ENSMUSG00000084960	B430010I23Rik	RIKEN cDNA B430010I23 gene [Source:MGI Symbol	0.33963
ENSMUSG00000091415	Ak9	adenylate kinase 9 [Source:MGI Symbol;Acc:MGI:2	0.33964
ENSMUSG00000083064	Gm7785	predicted gene 7785 [Source:MGI Symbol;Acc:MGI:	0.33965
ENSMUSG00000027076	Timm10	translocase of inner mitochondrial membrane 10 [S	0.33972
ENSMUSG00000042320	Prox2	prospero homeobox 2 [Source:MGI Symbol;Acc:MG	0.33977
ENSMUSG00000034990	Otoa	otoancorin [Source:MGI Symbol;Acc:MGI:2149209]	0.33978
ENSMUSG00000022683	Pla2g10	phospholipase A2, group X [Source:MGI Symbol;Ac	0.3398
ENSMUSG00000014603	Alx3	aristaless-like homeobox 3 [Source:MGI Symbol;Ac	0.33984
ENSMUSG00000097386	9130213A22Rik	RIKEN cDNA 9130213A22 gene [Source:MGI Symbo	0.33989
ENSMUSG00000036915	Kirrel2	kin of IRRE like 2 (Drosophila) [Source:MGI Symbol;	0.34003
ENSMUSG00000055494	Gm14168	predicted gene 14168 [Source:MGI Symbol;Acc:MG	0.34003
ENSMUSG00000073888	Ccl27a	chemokine (C-C motif) ligand 27A [Source:MGI Sym	0.34008
ENSMUSG00000045664	Cdc42ep2	CDC42 effector protein (Rho GTPase binding) 2 [Sou	0.34009
ENSMUSG00000081238	Gm15836	predicted gene 15836 [Source:MGI Symbol;Acc:MG	0.34012
ENSMUSG00000087496	Gm13523	predicted gene 13523 [Source:MGI Symbol;Acc:MG	0.34021
ENSMUSG00000096938	9530052E02Rik	RIKEN cDNA 9530052E02 gene [Source:MGI Symbo	0.34028
ENSMUSG00000022840	Adcy5	adenylate cyclase 5 [Source:MGI Symbol;Acc:MGI:9	0.34038

ENSMUSG00000034732	Pabpc5	poly(A) binding protein, cytoplasmic 5 [Source:MGI	0.34055
ENSMUSG00000045062	Pcdhb7	protocadherin beta 7 [Source:MGI Symbol;Acc:MGI	0.34079
ENSMUSG00000086416	Gm14002	predicted gene 14002 [Source:MGI Symbol;Acc:MG	0.34081
ENSMUSG00000085102	1700010K24Rik	RIKEN cDNA 1700010K24 gene [Source:MGI Symbo	0.34087
ENSMUSG00000052151	Ppap2c	phosphatidic acid phosphatase type 2C [Source:MG	0.34088
ENSMUSG00000036912	Piwil4	piwi-like RNA-mediated gene silencing 4 [Source:M	0.34089
ENSMUSG00000038425	Poli	polymerase (DNA directed), iota [Source:MGI Symb	0.34101
ENSMUSG00000074736	Syndig1	synapse differentiation inducing 1 [Source:MGI Sym	0.34102
ENSMUSG00000061048	Cdh3	cadherin 3 [Source:MGI Symbol;Acc:MGI:88356]	0.34108
ENSMUSG00000077309	n-R5s8	nuclear encoded rRNA 5S 8 [Source:MGI Symbol;Ac	0.34124
ENSMUSG00000001864	Aif1l	allograft inflammatory factor 1-like [Source:MGI Sy	0.34143
ENSMUSG00000051550	Zfp579	zinc finger protein 579 [Source:MGI Symbol;Acc:MG	0.34153
ENSMUSG00000084146	Gm12104	predicted gene 12104 [Source:MGI Symbol;Acc:MG	0.34156
ENSMUSG00000051705	Senp8	SUMO/sentrin specific peptidase 8 [Source:MGI Syn	0.34162
ENSMUSG00000054640	Slc8a1	solute carrier family 8 (sodium/calcium exchanger),	0.34163
ENSMUSG00000048281	Dleu7	deleted in lymphocytic leukemia, 7 [Source:MGI Sy	0.34171
ENSMUSG00000022681	Ntan1	N-terminal Asn amidase [Source:MGI Symbol;Acc:M	0.34178
ENSMUSG00000026463	Atp2b4	ATPase, Ca++ transporting, plasma membrane 4 [So	0.34199
ENSMUSG00000043924	Ncmap	noncompact myelin associated protein [Source:MG	0.34199
ENSMUSG00000006720	Zfp184	zinc finger protein 184 (Kruppel-like) [Source:MGI S	0.34211
ENSMUSG00000039810	Zc3h10	zinc finger CCCH type containing 10 [Source:MGI Sy	0.34211
ENSMUSG00000090099	Gm8837	predicted gene 8837 [Source:MGI Symbol;Acc:MGI:	0.34214
ENSMUSG00000022562	Oplah	5-oxoprolinase (ATP-hydrolysing) [Source:MGI Sym	0.34217
ENSMUSG00000030030	1700003E16Rik	RIKEN cDNA 1700003E16 gene [Source:MGI Symbo	0.34225
ENSMUSG00000032690	Oas2	2'-5' oligoadenylate synthetase 2 [Source:MGI Sym	0.34229
ENSMUSG00000020701	Tmem132e	transmembrane protein 132E [Source:MGI Symbol;	0.34249
ENSMUSG00000097563	Gm26638	predicted gene, 26638 [Source:MGI Symbol;Acc:MG	0.3425
ENSMUSG00000026495	Efcab2	EF-hand calcium binding domain 2 [Source:MGI Syn	0.34266
ENSMUSG00000000320	Alox12	arachidonate 12-lipoxygenase [Source:MGI Symbol	0.34274
ENSMUSG00000021371	Mcur1	mitochondrial calcium uniporter regulator 1 [Sourc	0.34289
ENSMUSG00000020799	Tekt1	tektin 1 [Source:MGI Symbol;Acc:MGI:1333819]	0.34293
ENSMUSG00000057411	Fam173a	family with sequence similarity 173, member A [Sou	0.34294
ENSMUSG00000032259	Drd2	dopamine receptor D2 [Source:MGI Symbol;Acc:MG	0.34305
ENSMUSG00000094613	A630076J17Rik	RIKEN cDNA A630076J17 gene [Source:MGI Symbo	0.3431
ENSMUSG00000020695	Mrc2	mannose receptor, C type 2 [Source:MGI Symbol;Ac	0.34311
ENSMUSG00000086451	4933431K23Rik	RIKEN cDNA 4933431K23 gene [Source:MGI Symbo	0.34326
ENSMUSG00000054032	Gm9932	predicted gene 9932 [Source:MGI Symbol;Acc:MGI:	0.34328
ENSMUSG00000032268	Tmprss5	transmembrane protease, serine 5 (spinesin) [Sour	0.34331
ENSMUSG00000030790	Adm	adrenomedullin [Source:MGI Symbol;Acc:MGI:1080	0.34339
ENSMUSG00000061578	Ksr2	kinase suppressor of ras 2 [Source:MGI Symbol;Acc	0.34339
ENSMUSG00000021373	Cap2	CAP, adenylate cyclase-associated protein, 2 (yeast	0.34352
ENSMUSG00000022995	Enah	enabled homolog (Drosophila) [Source:MGI Symbo	0.34352
ENSMUSG00000047807	9330154K18Rik	RIKEN cDNA 9330154K18 gene [Source:MGI Symbo	0.34353
ENSMUSG00000072235	Tuba1a	tubulin, alpha 1A [Source:MGI Symbol;Acc:MGI:988	0.34357
ENSMUSG00000032783	Troap	trophinin associated protein [Source:MGI Symbol;A	0.34361
ENSMUSG00000081269	Gm14659	predicted gene 14659 [Source:MGI Symbol;Acc:MG	0.34364
ENSMUSG00000041538	H2-Ob	histocompatibility 2, O region beta locus [Source:M	0.34366

ENSMUSG00000037833	Sh2d4b	SH2 domain containing 4B [Source:MGI Symbol;Acc	0.34367
ENSMUSG00000038467	Chmp4b	charged multivesicular body protein 4B [Source:MG	0.34367
ENSMUSG00000028494	Plin2	perilipin 2 [Source:MGI Symbol;Acc:MGI:87920]	0.34369
ENSMUSG00000029454	Mapkapk5	MAP kinase-activated protein kinase 5 [Source:MG	0.34372
ENSMUSG00000042350	Arel1	apoptosis resistant E3 ubiquitin protein ligase 1 [So	0.34374
ENSMUSG00000049588	Ccdc69	coiled-coil domain containing 69 [Source:MGI Symb	0.34398
ENSMUSG00000029032	Arhgef16	Rho guanine nucleotide exchange factor (GEF) 16 [S	0.344
ENSMUSG00000073155	1810058I24Rik	RIKEN cDNA 1810058I24 gene [Source:MGI Symbol	0.34401
ENSMUSG00000041479	Syt15	synaptotagmin XV [Source:MGI Symbol;Acc:MGI:24	0.3441
ENSMUSG00000023826	Park2	Parkinson disease (autosomal recessive, juvenile) 2	0.34446
ENSMUSG00000078249	Hmga1-rs1	high mobility group AT-hook I, related sequence 1 [	0.34448
ENSMUSG00000032534	Cep63	centrosomal protein 63 [Source:MGI Symbol;Acc:M	0.34466
ENSMUSG00000092595	Gm20427	predicted gene 20427 [Source:MGI Symbol;Acc:MG	0.34466
ENSMUSG00000085319	Gm13330	predicted gene 13330 [Source:MGI Symbol;Acc:MG	0.3447
ENSMUSG00000032203	Pigb	phosphatidylinositol glycan anchor biosynthesis, cla	0.34476
ENSMUSG00000025651	Uqcrc1	ubiquinol-cytochrome c reductase core protein 1 [S	0.34484
ENSMUSG00000021086	Ccdc175	coiled-coil domain containing 175 [Source:MGI Sym	0.34495
ENSMUSG00000026078	Pdcl3	phosducin-like 3 [Source:MGI Symbol;Acc:MGI:191	0.34513
ENSMUSG00000026209	Dnpep	aspartyl aminopeptidase [Source:MGI Symbol;Acc:I	0.34514
ENSMUSG00000050150	Slc9b1	solute carrier family 9, subfamily B (NHA1, cation p	0.3452
ENSMUSG00000028910	Mecr	mitochondrial trans-2-enoyl-CoA reductase [Source	0.34523
ENSMUSG00000097989	Gm4335	predicted gene 4335 [Source:MGI Symbol;Acc:MGI:	0.34523
ENSMUSG00000038843	Gcnt1	glucosaminyl (N-acetyl) transferase 1, core 2 [Sourc	0.34528
ENSMUSG00000021973	Micu2	mitochondrial calcium uptake 2 [Source:MGI Symb	0.34535
ENSMUSG00000081944	Gm12276	predicted gene 12276 [Source:MGI Symbol;Acc:MG	0.34537
ENSMUSG00000003378	Grik5	glutamate receptor, ionotropic, kainate 5 (gamma 2	0.34548
ENSMUSG00000019889	Ptprk	protein tyrosine phosphatase, receptor type, K [Sou	0.34551
ENSMUSG00000037049	Smpd1	sphingomyelin phosphodiesterase 1, acid lysosoma	0.34555
ENSMUSG00000061360	Phf5a	PHD finger protein 5A [Source:MGI Symbol;Acc:MG	0.34571
ENSMUSG00000081528	Gm12924	predicted gene 12924 [Source:MGI Symbol;Acc:MG	0.34571
ENSMUSG00000051262	Cml3	camello-like 3 [Source:MGI Symbol;Acc:MGI:21364	0.34573
ENSMUSG00000097997	Gm27017	predicted gene, 27017 [Source:MGI Symbol;Acc:M	0.34579
ENSMUSG00000048521	Cxcr6	chemokine (C-X-C motif) receptor 6 [Source:MGI Sy	0.3459
ENSMUSG00000032405	Pias1	protein inhibitor of activated STAT 1 [Source:MGI S	0.34599
ENSMUSG00000043496	Tril	TLR4 interactor with leucine-rich repeats [Source:M	0.34599
ENSMUSG00000015839	Nfe2l2	nuclear factor, erythroid derived 2, like 2 [Source:M	0.34601
ENSMUSG00000020156	Mum1	melanoma associated antigen (mutated) 1 [Source:	0.34614
ENSMUSG00000093921	Gm23864	predicted gene, 23864 [Source:MGI Symbol;Acc:M	0.34614
ENSMUSG00000035187	Nkx6-1	NK6 homeobox 1 [Source:MGI Symbol;Acc:MGI:12	0.34615
ENSMUSG00000096926	Gm26823	predicted gene, 26823 [Source:MGI Symbol;Acc:M	0.34615
ENSMUSG00000026687	Aldh9a1	aldehyde dehydrogenase 9, subfamily A1 [Source:M	0.34621
ENSMUSG00000083700	Gm2986	predicted gene 2986 [Source:MGI Symbol;Acc:MGI:	0.34624
ENSMUSG00000005610	Eif4g2	eukaryotic translation initiation factor 4, gamma 2	0.34626
ENSMUSG00000036850	Mrpl41	mitochondrial ribosomal protein L41 [Source:MGI S	0.34626
ENSMUSG00000059565	Gm5292	predicted gene 5292 [Source:MGI Symbol;Acc:MGI:	0.34637
ENSMUSG00000086181	C230034O21Rik	RIKEN cDNA C230034O21 gene [Source:MGI Symbo	0.34641
ENSMUSG00000020899	Pfas	phosphoribosylformylglycinamidine synthase (FGAI	0.34644

ENSMUSG00000031921	Terf2	telomeric repeat binding factor 2 [Source:MGI Sym	0.34648
ENSMUSG00000005103	Wdr1	WD repeat domain 1 [Source:MGI Symbol;Acc:MGI	0.34649
ENSMUSG00000005078	Jkamp	JNK1/MAPK8-associated membrane protein [Sourc	0.34652
ENSMUSG00000034855	Cxcl10	chemokine (C-X-C motif) ligand 10 [Source:MGI Syn	0.34668
ENSMUSG00000041707	1810011H11Rik	RIKEN cDNA 1810011H11 gene [Source:MGI Symbo	0.34689
ENSMUSG00000042750	Bex2	brain expressed X-linked 2 [Source:MGI Symbol;Acc	0.34689
ENSMUSG00000026887	Mrrf	mitochondrial ribosome recycling factor [Source:M	0.34709
ENSMUSG00000000594	Gm2a	GM2 ganglioside activator protein [Source:MGI Syn	0.34714
ENSMUSG00000054423	Cadps	Ca2+-dependent secretion activator [Source:MGI S	0.34715
ENSMUSG00000005580	Adcy9	adenylate cyclase 9 [Source:MGI Symbol;Acc:MGI:1	0.3472
ENSMUSG00000020785	Camkk1	calcium/calmodulin-dependent protein kinase kina	0.34734
ENSMUSG00000048027	Rgmb	RGM domain family, member B [Source:MGI Symbo	0.34735
ENSMUSG00000021224	Numb	numb gene homolog (Drosophila) [Source:MGI Sym	0.34737
ENSMUSG00000029635	Cdk8	cyclin-dependent kinase 8 [Source:MGI Symbol;Acc	0.34739
ENSMUSG00000072249	Gm5635	predicted gene 5635 [Source:MGI Symbol;Acc:MGI:	0.34741
ENSMUSG00000030060	Hmces	5-hydroxymethylcytosine (hmC) binding, ES cell spe	0.34744
ENSMUSG00000097286	Gm26684	predicted gene, 26684 [Source:MGI Symbol;Acc:MG	0.34748
ENSMUSG00000065396	Mir99b	microRNA 99b [Source:MGI Symbol;Acc:MGI:26769	0.34752
ENSMUSG00000025422	Agap2	ArfGAP with GTPase domain, ankyrin repeat and PH	0.34753
ENSMUSG00000021541	Trpc7	transient receptor potential cation channel, subfam	0.34754
ENSMUSG00000097308	Gm6410	predicted gene 6410 [Source:MGI Symbol;Acc:MGI:	0.34759
ENSMUSG00000020160	Meis1	Meis homeobox 1 [Source:MGI Symbol;Acc:MGI:10	0.34777
ENSMUSG00000031831	Dnaaf1	dynein, axonemal assembly factor 1 [Source:MGI Sy	0.34784
ENSMUSG00000032577	Mapkapk3	mitogen-activated protein kinase-activated protein	0.34784
ENSMUSG00000045410	Akr1e1	aldo-keto reductase family 1, member E1 [Source:M	0.34792
ENSMUSG00000038239	Hrc	histidine rich calcium binding protein [Source:MGI S	0.34797
ENSMUSG00000097748	Gm26533	predicted gene, 26533 [Source:MGI Symbol;Acc:MG	0.34799
ENSMUSG00000025952	Crygc	crystallin, gamma C [Source:MGI Symbol;Acc:MGI:8	0.34805
ENSMUSG00000097930	C330002G04Rik	RIKEN cDNA C330002G04 gene [Source:MGI Symbo	0.34812
ENSMUSG00000023411	Nfatc4	nuclear factor of activated T cells, cytoplasmic, calc	0.34816
ENSMUSG00000046269	Usp27x	ubiquitin specific peptidase 27, X chromosome [Sou	0.34817
ENSMUSG00000025780	Itih5	inter-alpha (globulin) inhibitor H5 [Source:MGI Sym	0.34828
ENSMUSG00000038523	1700003F12Rik	RIKEN cDNA 1700003F12 gene [Source:MGI Symbo	0.34844
ENSMUSG00000022863	Btg3	B cell translocation gene 3 [Source:MGI Symbol;Acc	0.34847
ENSMUSG00000043336	Filip1l	filamin A interacting protein 1-like [Source:MGI Syn	0.34848
ENSMUSG00000004393	Ddx56	DEAD (Asp-Glu-Ala-Asp) box polypeptide 56 [Source	0.34881
ENSMUSG00000021275	Tecpr2	tectonin beta-propeller repeat containing 2 [Source	0.34887
ENSMUSG00000005950	P2rx5	purinergic receptor P2X, ligand-gated ion channel, 5	0.34897
ENSMUSG00000055137	5033411D12Rik	RIKEN cDNA 5033411D12 gene [Source:MGI Symbo	0.34906
ENSMUSG00000062352	Itgb1bp1	integrin beta 1 binding protein 1 [Source:MGI Symb	0.3492
ENSMUSG00000082157	Gm12038	predicted gene 12038 [Source:MGI Symbol;Acc:MG	0.34926
ENSMUSG00000020430	Pes1	pescadillo homolog 1, containing BRCT domain (zeb	0.34928
ENSMUSG00000022248	Rad1	RAD1 homolog (S. pombe) [Source:MGI Symbol;Acc	0.34935
ENSMUSG00000000560	Gabra2	gamma-aminobutyric acid (GABA) A receptor, subu	0.34937
ENSMUSG00000046607	Hrk	harakiri, BCL2 interacting protein (contains only BH	0.34945
ENSMUSG00000026740	Dnajc1	DnaJ (Hsp40) homolog, subfamily C, member 1 [Sou	0.34947
ENSMUSG00000021764	Ndufs4	NADH dehydrogenase (ubiquinone) Fe-S protein 4 [	0.34955

ENSMUSG00000087120	Gm12279	predicted gene 12279 [Source:MGI Symbol;Acc:MG	0.34962
ENSMUSG00000074763	Gm14105	predicted gene 14105 [Source:MGI Symbol;Acc:MG	0.3498
ENSMUSG00000019214	Chtf18	CTF18, chromosome transmission fidelity factor 18	0.34981
ENSMUSG00000040112	Mrps35	mitochondrial ribosomal protein S35 [Source:MGI S	0.34993
ENSMUSG00000097749	Gm16938	predicted gene, 16938 [Source:MGI Symbol;Acc:MG	0.34994
ENSMUSG00000031661	Nkd1	naked cuticle 1 homolog (Drosophila) [Source:MGI	0.35003
ENSMUSG00000040606	Kazn	kazrin, periplakin interacting protein [Source:MGI S	0.3505
ENSMUSG00000097890	4930547M16Rik	RIKEN cDNA 4930547M16 gene [Source:MGI Symbo	0.35054
ENSMUSG00000038855	Itpkb	inositol 1,4,5-trisphosphate 3-kinase B [Source:MG	0.35063
ENSMUSG00000025980	Hspd1	heat shock protein 1 (chaperonin) [Source:MGI Sym	0.35065
ENSMUSG00000056411	Gm12500	predicted gene 12500 [Source:MGI Symbol;Acc:MG	0.35084
ENSMUSG00000087721	Gm26280	predicted gene, 26280 [Source:MGI Symbol;Acc:MG	0.35088
ENSMUSG00000091507	Gm17194	predicted gene 17194 [Source:MGI Symbol;Acc:MG	0.35091
ENSMUSG00000086962	Gm12248	predicted gene 12248 [Source:MGI Symbol;Acc:MG	0.35092
ENSMUSG00000028998	Tomm7	translocase of outer mitochondrial membrane 7 ho	0.35095
ENSMUSG00000037814	Larp1b	La ribonucleoprotein domain family, member 1B [S	0.35095
ENSMUSG00000059013	Sh2d3c	SH2 domain containing 3C [Source:MGI Symbol;Acc	0.35097
ENSMUSG00000004031	Brinp2	bone morphogenic protein/retinoic acid inducible n	0.35118
ENSMUSG00000028953	Abcf2	ATP-binding cassette, sub-family F (GCN20), memb	0.35125
ENSMUSG00000040972	Igsf21	immunoglobulin superfamily, member 21 [Source:M	0.35132
ENSMUSG00000071047	Ces1a	carboxylesterase 1A [Source:MGI Symbol;Acc:MGI:	0.35139
ENSMUSG00000090121	Abhd12b	abhydrolase domain containing 12B [Source:MGI Sy	0.35141
ENSMUSG00000025758	Plk4	polo-like kinase 4 [Source:MGI Symbol;Acc:MGI:10	0.3515
ENSMUSG00000056258	Kcnq3	potassium voltage-gated channel, subfamily Q, mer	0.35153
ENSMUSG00000024533	Spire1	spire homolog 1 (Drosophila) [Source:MGI Symbol;	0.35158
ENSMUSG00000094595	Fsbp	fibrinogen silencer binding protein [Source:MGI Syr	0.35171
ENSMUSG00000020216	Jsrp1	junctional sarcoplasmic reticulum protein 1 [Source	0.35184
ENSMUSG00000015804	Med28	mediator of RNA polymerase II transcription, subur	0.35188
ENSMUSG00000066026	Dhrs3	dehydrogenase/reductase (SDR family) member 3 [	0.3519
ENSMUSG00000094247	Gm23968	predicted gene, 23968 [Source:MGI Symbol;Acc:MG	0.35203
ENSMUSG00000019338	Zfp687	zinc finger protein 687 [Source:MGI Symbol;Acc:MG	0.35204
ENSMUSG00000028837	Psmb2	proteasome (prosome, macropain) subunit, beta ty	0.35216
ENSMUSG00000098232	Gm3671	predicted gene 3671 [Source:MGI Symbol;Acc:MGI:	0.35217
ENSMUSG00000074521	Gm14327	predicted gene 14327 [Source:MGI Symbol;Acc:MG	0.3522
ENSMUSG00000020900	Myh10	myosin, heavy polypeptide 10, non-muscle [Source	0.35221
ENSMUSG00000026220	Slc16a14	solute carrier family 16 (monocarboxylic acid transp	0.35229
ENSMUSG00000044783	Hjurp	Holliday junction recognition protein [Source:MGI S	0.35232
ENSMUSG00000031928	Mre11a	meiotic recombination 11 homolog A (S. cerevisiae)	0.35237
ENSMUSG00000040189	Ccdc114	coiled-coil domain containing 114 [Source:MGI Sym	0.35242
ENSMUSG00000036292	Gramd1c	GRAM domain containing 1C [Source:MGI Symbol;A	0.35243
ENSMUSG00000028756	Pink1	PTEN induced putative kinase 1 [Source:MGI Symbo	0.3527
ENSMUSG00000050295	Foxc1	forkhead box C1 [Source:MGI Symbol;Acc:MGI:134	0.35273
ENSMUSG00000025372	Baiap2	brain-specific angiogenesis inhibitor 1-associated p	0.35284
ENSMUSG00000070870	Cryge	crystallin, gamma E [Source:MGI Symbol;Acc:MGI:8	0.35285
ENSMUSG00000087107	AI662270	expressed sequence AI662270 [Source:MGI Symbol	0.35286
ENSMUSG00000096983	2010015M23Rik	RIKEN cDNA 2010015M23 gene [Source:MGI Symbo	0.35291
ENSMUSG00000004347	Pde1c	phosphodiesterase 1C [Source:MGI Symbol;Acc:MG	0.35293

ENSMUSG00000050567	Maml1	mastermind like 1 (Drosophila) [Source:MGI Symbo	0.35296
ENSMUSG00000060743	H3f3a	H3 histone, family 3A [Source:MGI Symbol;Acc:MG	0.35297
ENSMUSG00000023009	Nckap5l	NCK-associated protein 5-like [Source:MGI Symbol;	0.353
ENSMUSG00000029376	Mthfd2l	methylenetetrahydrofolate dehydrogenase (NADP-	0.353
ENSMUSG00000022225	Cma1	chymase 1, mast cell [Source:MGI Symbol;Acc:MGI:	0.3533
ENSMUSG00000097781	9330136K24Rik	RIKEN cDNA 9330136K24 gene [Source:MGI Symbo	0.35333
ENSMUSG00000075419	Dolk	dolichol kinase [Source:MGI Symbol;Acc:MGI:2677	0.35336
ENSMUSG00000033544	Angptl1	angiopoietin-like 1 [Source:MGI Symbol;Acc:MGI:1	0.35351
ENSMUSG00000026544	Dusp23	dual specificity phosphatase 23 [Source:MGI Symbo	0.35357
ENSMUSG00000089999	Gm6485	predicted gene 6485 [Source:MGI Symbol;Acc:MGI:	0.35357
ENSMUSG00000088249	Gm25908	predicted gene, 25908 [Source:MGI Symbol;Acc:M	0.35358
ENSMUSG00000087289	4933424M12Rik	RIKEN cDNA 4933424M12 gene [Source:MGI Symb	0.35363
ENSMUSG00000032796	Lama1	laminin, alpha 1 [Source:MGI Symbol;Acc:MGI:998	0.35365
ENSMUSG00000057116	AF366264	cDNA sequence AF366264 [Source:MGI Symbol;Ac	0.35366
ENSMUSG00000029152	Ociad1	OCIA domain containing 1 [Source:MGI Symbol;Ac	0.35368
ENSMUSG00000037095	Lrg1	leucine-rich alpha-2-glycoprotein 1 [Source:MGI Sy	0.35371
ENSMUSG00000001473	Tubb6	tubulin, beta 6 class V [Source:MGI Symbol;Acc:MG	0.35374
ENSMUSG00000007867	Ift43	intraflagellar transport 43 homolog (Chlamydomon	0.35376
ENSMUSG00000014077	Chp1	calcineurin-like EF hand protein 1 [Source:MGI Sym	0.35377
ENSMUSG00000048154	Kmt2d	lysine (K)-specific methyltransferase 2D [Source:M	0.35384
ENSMUSG00000063838	Cdc42ep5	CDC42 effector protein (Rho GTPase binding) 5 [So	0.35385
ENSMUSG00000009092	Derl3	Der1-like domain family, member 3 [Source:MGI Sy	0.35389
ENSMUSG00000029169	Dhx15	DEAH (Asp-Glu-Ala-His) box polypeptide 15 [Source	0.35394
ENSMUSG00000097829	MYADM	myeloid-associated differentiation marker (Myadm	0.35411
ENSMUSG00000041911	Dlx1	distal-less homeobox 1 [Source:MGI Symbol;Acc:M	0.35413
ENSMUSG00000037926	Ssh2	slingshot homolog 2 (Drosophila) [Source:MGI Sym	0.35418
ENSMUSG00000000085	Scmh1	sex comb on midleg homolog 1 [Source:MGI Symbo	0.35419
ENSMUSG00000047881	Rell1	RELT-like 1 [Source:MGI Symbol;Acc:MGI:2140767]	0.35429
ENSMUSG00000037159	Wee2	WEE1 homolog 2 (S. pombe) [Source:MGI Symbol;A	0.35443
ENSMUSG00000031751	Amfr	autocrine motility factor receptor [Source:MGI Sym	0.35448
ENSMUSG00000063049	Ing2	inhibitor of growth family, member 2 [Source:MGI S	0.35454
ENSMUSG00000003779	Kif20a	kinesin family member 20A [Source:MGI Symbol;Ac	0.35455
ENSMUSG00000022833	Ccdc14	coiled-coil domain containing 14 [Source:MGI Symb	0.35457
ENSMUSG00000028992	Nmnat1	nicotinamide nucleotide adenylyltransferase 1 [Sou	0.35468
ENSMUSG00000029372	Ppbp	pro-platelet basic protein [Source:MGI Symbol;Acc:	0.3547
ENSMUSG00000031918	Mtmr2	myotubularin related protein 2 [Source:MGI Symbo	0.35471
ENSMUSG00000021363	Mak	male germ cell-associated kinase [Source:MGI Syml	0.35489
ENSMUSG00000083284	Gm13688	predicted gene 13688 [Source:MGI Symbol;Acc:MG	0.35508
ENSMUSG00000056089	Gm5468	predicted gene 5468 [Source:MGI Symbol;Acc:MGI:	0.3551
ENSMUSG00000041202	Pla2g2d	phospholipase A2, group IID [Source:MGI Symbol;A	0.35512
ENSMUSG00000027710	Acad9	acyl-Coenzyme A dehydrogenase family, member 9	0.35518
ENSMUSG00000029076	Sdf4	stromal cell derived factor 4 [Source:MGI Symbol;A	0.35519
ENSMUSG00000052516	Robo2	roundabout homolog 2 (Drosophila) [Source:MGI S	0.35533
ENSMUSG00000026213	Stk11ip	serine/threonine kinase 11 interacting protein [Sou	0.35541
ENSMUSG00000059991	Nptx2	neuronal pentraxin 2 [Source:MGI Symbol;Acc:MGI	0.35541
ENSMUSG00000018927	Ccl6	chemokine (C-C motif) ligand 6 [Source:MGI Symbo	0.35543
ENSMUSG00000060371	Caln1	calneuron 1 [Source:MGI Symbol;Acc:MGI:2155987	0.35547

ENSMUSG00000062647	Rpl7a	ribosomal protein L7A [Source:MGI Symbol;Acc:MG	0.35555
ENSMUSG00000021335	Slc17a1	solute carrier family 17 (sodium phosphate), memb	0.35559
ENSMUSG00000002948	Map2k7	mitogen-activated protein kinase kinase 7 [Source:I	0.35562
ENSMUSG00000093846	Gm4425	predicted gene 4425 [Source:MGI Symbol;Acc:MGI:	0.35564
ENSMUSG00000035696	Rnf38	ring finger protein 38 [Source:MGI Symbol;Acc:MG	0.35568
ENSMUSG00000032633	Flcn	folliculin [Source:MGI Symbol;Acc:MGI:2442184]	0.3558
ENSMUSG00000011752	Pgam1	phosphoglycerate mutase 1 [Source:MGI Symbol;A	0.35581
ENSMUSG00000020708	Psmc5	protease (prosome, macropain) 26S subunit, ATPas	0.35582
ENSMUSG00000079469	Pigb	phosphatidylinositol glycan anchor biosynthesis, cla	0.35583
ENSMUSG00000028996	Rbp7	retinol binding protein 7, cellular [Source:MGI Sym	0.35587
ENSMUSG00000091519	Skor2	SKI family transcriptional corepressor 2 [Source:MG	0.35591
ENSMUSG00000040659	Efhd2	EF hand domain containing 2 [Source:MGI Symbol;	0.35596
ENSMUSG00000026839	Upp2	uridine phosphorylase 2 [Source:MGI Symbol;Acc:N	0.35604
ENSMUSG00000024099	Ndufv2	NADH dehydrogenase (ubiquinone) flavoprotein 2	0.3562
ENSMUSG00000038970	Lmtk2	lemur tyrosine kinase 2 [Source:MGI Symbol;Acc:M	0.35625
ENSMUSG00000046603	Tcaim	T cell activation inhibitor, mitochondrial [Source:M	0.35636
ENSMUSG00000087301	Gm13629	predicted gene 13629 [Source:MGI Symbol;Acc:MG	0.3564
ENSMUSG00000049929	Lpar4	lysophosphatidic acid receptor 4 [Source:MGI Symb	0.35642
ENSMUSG00000028420	Tmem38b	transmembrane protein 38B [Source:MGI Symbol;A	0.35647
ENSMUSG00000050074	Spink8	serine peptidase inhibitor, Kazal type 8 [Source:MG	0.35653
ENSMUSG00000059734	Ndufs8	NADH dehydrogenase (ubiquinone) Fe-S protein 8	0.35655
ENSMUSG00000086845	Gm13010	predicted gene 13010 [Source:MGI Symbol;Acc:MG	0.35659
ENSMUSG00000030774	Pak1	p21 protein (Cdc42/Rac)-activated kinase 1 [Source	0.3566
ENSMUSG00000031449	Atp4b	ATPase, H+/K+ exchanging, beta polypeptide [Sour	0.35661
ENSMUSG00000024696	Lpxn	leupaxin [Source:MGI Symbol;Acc:MGI:2147677]	0.35663
ENSMUSG00000020075	Ddx21	DEAD (Asp-Glu-Ala-Asp) box polypeptide 21 [Sourc	0.35669
ENSMUSG00000053111	Fank1	fibronectin type 3 and ankyrin repeat domains 1 [S	0.35672
ENSMUSG00000040794	C1qtnf4	C1q and tumor necrosis factor related protein 4 [So	0.35678
ENSMUSG00000021024	Psma6	proteasome (prosome, macropain) subunit, alpha t	0.3568
ENSMUSG00000088708	Gm25558	predicted gene, 25558 [Source:MGI Symbol;Acc:M	0.35683
ENSMUSG00000000805	Car4	carbonic anhydrase 4 [Source:MGI Symbol;Acc:MG	0.35702
ENSMUSG00000020224	Llph	LLP homolog, long-term synaptic facilitation (Aplysi	0.35702
ENSMUSG00000093085	Gm24325	predicted gene, 24325 [Source:MGI Symbol;Acc:M	0.35714
ENSMUSG00000018470	Kcnab3	potassium voltage-gated channel, shaker-related su	0.35721
ENSMUSG00000071893	Vmn1r4	vomeronasal 1 receptor 4 [Source:MGI Symbol;Acc	0.35723
ENSMUSG00000029254	Stap1	signal transducing adaptor family member 1 [Sourc	0.35725
ENSMUSG00000037933	Bicd2	bicaudal D homolog 2 (Drosophila) [Source:MGI Sy	0.35725
ENSMUSG00000086268	Gm11670	predicted gene 11670 [Source:MGI Symbol;Acc:MG	0.35725
ENSMUSG00000032900	1700065I17Rik	RIKEN cDNA 1700065I17 gene [Source:MGI Symbol	0.35731
ENSMUSG00000086340	1810059C17Rik	RIKEN cDNA 1810059C17 gene [Source:MGI Symbo	0.35736
ENSMUSG00000071192	Wfikkn1	WAP, FS, Ig, KU, and NTR-containing protein 1 [Sou	0.35737
ENSMUSG00000093383	Gm20642	predicted gene 20642 [Source:MGI Symbol;Acc:MG	0.35746
ENSMUSG00000027134	Lpcat4	lysophosphatidylcholine acyltransferase 4 [Source:I	0.35747
ENSMUSG00000061959	Ces1e	carboxylesterase 1E [Source:MGI Symbol;Acc:MGI:	0.35749
ENSMUSG00000058806	Col13a1	collagen, type XIII, alpha 1 [Source:MGI Symbol;Acc	0.35768
ENSMUSG00000049538	Adamts16	a disintegrin-like and metallopeptidase (reprolysin	0.35775
ENSMUSG00000079362	Gbp6	guanylate binding protein 6 [Source:MGI Symbol;A	0.35778

ENSMUSG00000053399	Adamts18	a disintegrin-like and metallopeptidase (reprolysin	0.35792
ENSMUSG00000031264	Btk	Bruton agammaglobulinemia tyrosine kinase [Sourc	0.35796
ENSMUSG00000039958	Mettl20	methyltransferase like 20 [Source:MGI Symbol;Acc:	0.35797
ENSMUSG00000020235	Fzr1	fizzy/cell division cycle 20 related 1 (Drosophila) [So	0.35801
ENSMUSG00000001909	Trmt1	tRNA methyltransferase 1 [Source:MGI Symbol;Acc	0.35803
ENSMUSG00000045467	Ttll13	tubulin tyrosine ligase-like family, member 13 [Sou	0.35806
ENSMUSG00000031781	Ciapin1	cytokine induced apoptosis inhibitor 1 [Source:MGI	0.35808
ENSMUSG00000003809	Gcdh	glutaryl-Coenzyme A dehydrogenase [Source:MGI S	0.3581
ENSMUSG00000026043	Col3a1	collagen, type III, alpha 1 [Source:MGI Symbol;Acc:	0.35812
ENSMUSG00000023147	Wrb	tryptophan rich basic protein [Source:MGI Symbol;	0.35823
ENSMUSG00000028445	Enho	energy homeostasis associated [Source:MGI Symbo	0.35823
ENSMUSG00000000903	Vpreb3	pre-B lymphocyte gene 3 [Source:MGI Symbol;Acc:	0.35826
ENSMUSG00000041757	Plekha6	pleckstrin homology domain containing, family A m	0.35832
ENSMUSG00000028973	Abcb8	ATP-binding cassette, sub-family B (MDR/TAP), mer	0.35838
ENSMUSG00000091997	Gm6611	predicted gene 6611 [Source:MGI Symbol;Acc:MGI:	0.35845
ENSMUSG00000082809	Gm14150	predicted gene 14150 [Source:MGI Symbol;Acc:MG	0.3585
ENSMUSG00000022570	Tsta3	tissue specific transplantation antigen P35B [Source	0.35852
ENSMUSG00000039193	Nlrc4	NLR family, CARD domain containing 4 [Source:MG	0.35853
ENSMUSG00000093535	4930533I22Rik	RIKEN cDNA 4930533I22 gene [Source:MGI Symbol	0.35856
ENSMUSG00000038085	Cnbd2	cyclic nucleotide binding domain containing 2 [Sou	0.35862
ENSMUSG00000074283	Zfp109	zinc finger protein 109 [Source:MGI Symbol;Acc:MG	0.35863
ENSMUSG00000054717	Hmgb2	high mobility group box 2 [Source:MGI Symbol;Acc:	0.35866
ENSMUSG00000032232	Cgnl1	cingulin-like 1 [Source:MGI Symbol;Acc:MGI:19154	0.35868
ENSMUSG00000094152	Slc6a16	solute carrier family 6, member 16 [Source:MGI Syr	0.3587
ENSMUSG00000003233	Dvl3	dishevelled 3, dsh homolog (Drosophila) [Source:M	0.35879
ENSMUSG00000042903	Foxo4	forkhead box O4 [Source:MGI Symbol;Acc:MGI:189	0.35879
ENSMUSG00000026051	1500015O10Rik	RIKEN cDNA 1500015O10 gene [Source:MGI Symbo	0.35883
ENSMUSG00000024777	Ppp2r5b	protein phosphatase 2, regulatory subunit B (B56),	0.35899
ENSMUSG00000086498	Gm12681	predicted gene 12681 [Source:MGI Symbol;Acc:MG	0.35903
ENSMUSG00000087070	Gm12505	predicted gene 12505 [Source:MGI Symbol;Acc:MG	0.35911
ENSMUSG00000049643	2310022A10Rik	RIKEN cDNA 2310022A10 gene [Source:MGI Symbo	0.35922
ENSMUSG00000086727	4931428L18Rik	RIKEN cDNA 4931428L18 gene [Source:MGI Symbo	0.35924
ENSMUSG00000027679	Dnajc19	DnaJ (Hsp40) homolog, subfamily C, member 19 [So	0.35927
ENSMUSG00000026816	Gtf3c5	general transcription factor IIIC, polypeptide 5 [Sou	0.3593
ENSMUSG00000022139	Mbnl2	muscleblind-like 2 [Source:MGI Symbol;Acc:MGI:21	0.35936
ENSMUSG00000021540	Smad5	SMAD family member 5 [Source:MGI Symbol;Acc:M	0.35938
ENSMUSG00000025375	Aatk	apoptosis-associated tyrosine kinase [Source:MGI S	0.35946
ENSMUSG00000060675	Pla2g16	phospholipase A2, group XVI [Source:MGI Symbol;A	0.35947
ENSMUSG00000006464	Bbs1	Bardet-Biedl syndrome 1 (human) [Source:MGI Sym	0.35953
ENSMUSG00000087601	Uchl1os	Uchl1 opposite strand transcript (head to head) [So	0.3596
ENSMUSG00000085771	C230066G23Rik	RIKEN cDNA C230066G23 gene [Source:MGI Symbo	0.35962
ENSMUSG00000038057	Dbil5	diazepam binding inhibitor-like 5 [Source:MGI Sym	0.35963
ENSMUSG00000024277	Mapre2	microtubule-associated protein, RP/EB family, mem	0.35969
ENSMUSG00000020474	Polm	polymerase (DNA directed), mu [Source:MGI Symbo	0.35983
ENSMUSG00000034210	Efcab14	EF-hand calcium binding domain 14 [Source:MGI Sy	0.35985
ENSMUSG00000000632	Sez6	seizure related gene 6 [Source:MGI Symbol;Acc:MG	0.35987
ENSMUSG00000090244	Gm16572	predicted gene 16572 [Source:MGI Symbol;Acc:MG	0.35998

ENSMUSG00000077639	Gm22772	predicted gene, 22772 [Source:MGI Symbol;Acc:MG	0.35999
ENSMUSG00000029727	Cyp3a13	cytochrome P450, family 3, subfamily a, polypeptid	0.36002
ENSMUSG00000023333	Gcm1	glial cells missing homolog 1 (Drosophila) [Source:M	0.36005
ENSMUSG00000032602	Slc25a20	solute carrier family 25 (mitochondrial carnitine/ac	0.36012
ENSMUSG00000037966	Ninj1	ninjurin 1 [Source:MGI Symbol;Acc:MGI:1196617]	0.36016
ENSMUSG00000044081	4930441O14Rik	RIKEN cDNA 4930441O14 gene [Source:MGI Symbo	0.36019
ENSMUSG00000090671	Gm5067	predicted gene 5067 [Source:MGI Symbol;Acc:MGI:	0.36019
ENSMUSG00000053398	Phgdh	3-phosphoglycerate dehydrogenase [Source:MGI Sy	0.3602
ENSMUSG00000074852	Hpse2	heparanase 2 [Source:MGI Symbol;Acc:MGI:268581	0.36022
ENSMUSG00000094257	Ap3s1-ps2	adaptor-related protein complex 3, sigma 1 subunit	0.36026
ENSMUSG00000027699	Ect2	ect2 oncogene [Source:MGI Symbol;Acc:MGI:95281	0.36028
ENSMUSG00000030708	Dnajb13	DnaJ (Hsp40) related, subfamily B, member 13 [Sou	0.36028
ENSMUSG00000025172	Ankrd2	ankyrin repeat domain 2 (stretch responsive muscle	0.36033
ENSMUSG00000019590	Cyb561	cytochrome b-561 [Source:MGI Symbol;Acc:MGI:10	0.36036
ENSMUSG00000042417	Ccno	cyclin O [Source:MGI Symbol;Acc:MGI:2145534]	0.36037
ENSMUSG00000034826	Nup54	nucleoporin 54 [Source:MGI Symbol;Acc:MGI:1920	0.36038
ENSMUSG00000002635	Pdcd2l	programmed cell death 2-like [Source:MGI Symbol;	0.36045
ENSMUSG00000029875	AI836003	expressed sequence AI836003 [Source:MGI Symbol	0.36045
ENSMUSG00000048731	Ggnbp1	gametogenetin binding protein 1 [Source:MGI Sym	0.36048
ENSMUSG00000006005	Tpr	translocated promoter region [Source:MGI Symbol;	0.36052
ENSMUSG00000029135	Fosl2	fos-like antigen 2 [Source:MGI Symbol;Acc:MGI:102	0.36055
ENSMUSG00000033396	Spg11	spastic paraplegia 11 [Source:MGI Symbol;Acc:MGI	0.36062
ENSMUSG00000046761	Fam83h	family with sequence similarity 83, member H [Sou	0.36071
ENSMUSG00000046591	Ticrr	TOPBP1-interacting checkpoint and replication regu	0.36072
ENSMUSG00000062190	Lancl2	LanC (bacterial lantibiotic synthetase component C	0.36082
ENSMUSG00000001521	Tulp3	tubby-like protein 3 [Source:MGI Symbol;Acc:MGI:1	0.36089
ENSMUSG00000019814	Ltv1	LTV1 homolog (S. cerevisiae) [Source:MGI Symbol;A	0.36093
ENSMUSG00000031618	Nr3c2	nuclear receptor subfamily 3, group C, member 2 [S	0.36094
ENSMUSG00000025937	Lactb2	lactamase, beta 2 [Source:MGI Symbol;Acc:MGI:24	0.36096
ENSMUSG00000045521	Tssk2	testis-specific serine kinase 2 [Source:MGI Symbol;A	0.36096
ENSMUSG00000073528	Gm10530	predicted gene 10530 [Source:MGI Symbol;Acc:MG	0.361
ENSMUSG00000024124	Prss30	protease, serine, 30 [Source:MGI Symbol;Acc:MGI:1	0.36101
ENSMUSG00000078967	Gm3272	predicted pseudogene 3272 [Source:MGI Symbol;A	0.36101
ENSMUSG00000026083	Eif5b	eukaryotic translation initiation factor 5B [Source:M	0.36105
ENSMUSG00000050973	Gdpgp1	GDP-D-glucose phosphorylase 1 [Source:MGI Symb	0.36105
ENSMUSG00000041849	Card6	caspase recruitment domain family, member 6 [Sou	0.36107
ENSMUSG00000088982	Gm25600	predicted gene, 25600 [Source:MGI Symbol;Acc:MG	0.36108
ENSMUSG00000029048	Rer1	RER1 retention in endoplasmic reticulum 1 homolo	0.36119
ENSMUSG00000035004	Igsf6	immunoglobulin superfamily, member 6 [Source:M	0.36123
ENSMUSG00000086006	Gm13293	predicted gene 13293 [Source:MGI Symbol;Acc:MG	0.36125
ENSMUSG00000020267	Hint1	histidine triad nucleotide binding protein 1 [Source	0.36133
ENSMUSG00000047423	AI837181	expressed sequence AI837181 [Source:MGI Symbol	0.36138
ENSMUSG00000021499	Catsper3	cation channel, sperm associated 3 [Source:MGI Sy	0.36141
ENSMUSG00000021675	F2rl2	coagulation factor II (thrombin) receptor-like 2 [Sou	0.36145
ENSMUSG00000064063	BC048507	cDNA sequence BC048507 [Source:MGI Symbol;Acc	0.36149
ENSMUSG00000086017	Gm11872	predicted gene 11872 [Source:MGI Symbol;Acc:MG	0.36149
ENSMUSG00000028478	Clta	clathrin, light polypeptide (Lca) [Source:MGI Symbo	0.36151

ENSMUSG00000081745	Gm15549	predicted gene 15549 [Source:MGI Symbol;Acc:MG	0.36153
ENSMUSG00000021420	Fars2	phenylalanine-tRNA synthetase 2 (mitochondrial) [S	0.36154
ENSMUSG00000048703	Speer4b	spermatogenesis associated glutamate (E)-rich prot	0.36155
ENSMUSG00000021428	Riok1	RIO kinase 1 (yeast) [Source:MGI Symbol;Acc:MGI:1	0.36158
ENSMUSG00000061816	Myl1	myosin, light polypeptide 1 [Source:MGI Symbol;Ac	0.36158
ENSMUSG00000018507	Trpv2	transient receptor potential cation channel, subfam	0.36169
ENSMUSG00000059741	Myl3	myosin, light polypeptide 3 [Source:MGI Symbol;Ac	0.36176
ENSMUSG00000004056	Akt2	thymoma viral proto-oncogene 2 [Source:MGI Sym	0.36188
ENSMUSG00000015776	Med22	mediator complex subunit 22 [Source:MGI Symbol;	0.36193
ENSMUSG00000002393	Nr2f6	nuclear receptor subfamily 2, group F, member 6 [S	0.36202
ENSMUSG00000018287	Spag7	sperm associated antigen 7 [Source:MGI Symbol;Ac	0.36222
ENSMUSG00000046908	Ltb4r1	leukotriene B4 receptor 1 [Source:MGI Symbol;Acc	0.36233
ENSMUSG00000084761	Gm12406	predicted gene 12406 [Source:MGI Symbol;Acc:MG	0.36252
ENSMUSG00000001289	Pfdn5	prefoldin 5 [Source:MGI Symbol;Acc:MGI:1928753]	0.36261
ENSMUSG00000022151	Ttc33	tetratricopeptide repeat domain 33 [Source:MGI Sy	0.36263
ENSMUSG00000078917	Gm11281	predicted gene 11281 [Source:MGI Symbol;Acc:MG	0.36264
ENSMUSG00000060419	Rps16-ps2	ribosomal protein S16, pseudogene 2 [Source:MGI	0.36267
ENSMUSG00000043311	D17H6S53E	DNA segment, Chr 17, human D6S53E [Source:MGI	0.36269
ENSMUSG00000097413	A830052D11Rik	RIKEN cDNA A830052D11 gene [Source:MGI Symbo	0.36273
ENSMUSG00000040632	Nrl	neural retina leucine zipper gene [Source:MGI Sym	0.36275
ENSMUSG00000033105	Lss	lanosterol synthase [Source:MGI Symbol;Acc:MGI:1	0.36281
ENSMUSG00000031826	Usp10	ubiquitin specific peptidase 10 [Source:MGI Symbo	0.36301
ENSMUSG00000022181	C6	complement component 6 [Source:MGI Symbol;Acc	0.36303
ENSMUSG00000028539	Artn	artemin [Source:MGI Symbol;Acc:MGI:1333791]	0.36308
ENSMUSG00000002799	Jag2	jagged 2 [Source:MGI Symbol;Acc:MGI:1098270]	0.3632
ENSMUSG00000047935	Gm5607	predicted gene 5607 [Source:MGI Symbol;Acc:MGI:	0.36352
ENSMUSG00000049744	Arhgap15	Rho GTPase activating protein 15 [Source:MGI Sym	0.36353
ENSMUSG00000097799	Gm26899	predicted gene, 26899 [Source:MGI Symbol;Acc:MG	0.36357
ENSMUSG00000006127	Inpp5k	inositol polyphosphate 5-phosphatase K [Source:M	0.3636
ENSMUSG00000009772	Nuak2	NUAK family, SNF1-like kinase, 2 [Source:MGI Symb	0.36367
ENSMUSG00000034707	Gns	glucosamine (N-acetyl)-6-sulfatase [Source:MGI Sym	0.36376
ENSMUSG00000022548	Apod	apolipoprotein D [Source:MGI Symbol;Acc:MGI:880	0.36399
ENSMUSG00000040824	Snrpd2	small nuclear ribonucleoprotein D2 [Source:MGI Sy	0.36407
ENSMUSG00000071669	Snx29	sorting nexin 29 [Source:MGI Symbol;Acc:MGI:192	0.36416
ENSMUSG00000030022	Adamts9	a disintegrin-like and metallopeptidase (reprolysin	0.3642
ENSMUSG00000042918	Mamstr	MEF2 activating motif and SAP domain containing t	0.3642
ENSMUSG00000049288	Lix1l	Lix1-like [Source:MGI Symbol;Acc:MGI:3036267]	0.3642
ENSMUSG00000019297	Nop9	NOP9 nucleolar protein [Source:MGI Symbol;Acc:M	0.36425
ENSMUSG00000025151	Maged1	melanoma antigen, family D, 1 [Source:MGI Symbo	0.36427
ENSMUSG00000020166	Cnot2	CCR4-NOT transcription complex, subunit 2 [Source	0.36428
ENSMUSG00000021414	Fam217a	family with sequence similarity 217, member A [Sou	0.36437
ENSMUSG00000044055	Otos	otospiralin [Source:MGI Symbol;Acc:MGI:2672814]	0.36439
ENSMUSG00000018659	Pnpo	pyridoxine 5'-phosphate oxidase [Source:MGI Symb	0.36442
ENSMUSG00000070424	Art5	ADP-ribosyltransferase 5 [Source:MGI Symbol;Acc:I	0.36446
ENSMUSG00000086292	Gm16052	predicted gene 16052 [Source:MGI Symbol;Acc:MG	0.36453
ENSMUSG00000097247	1500012K07Rik	RIKEN cDNA 1500012K07 gene [Source:MGI Symbo	0.36454
ENSMUSG00000098028	Gm8824	predicted gene 8824 [Source:MGI Symbol;Acc:MGI:	0.36469

ENSMUSG00000038612	Mcl1	myeloid cell leukemia sequence 1 [Source:MGI Sym	0.3647
ENSMUSG00000020752	Recql5	RecQ protein-like 5 [Source:MGI Symbol;Acc:MGI:2	0.36472
ENSMUSG00000032251	Irak1bp1	interleukin-1 receptor-associated kinase 1 binding	0.36474
ENSMUSG00000042688	Mapk6	mitogen-activated protein kinase 6 [Source:MGI Sy	0.36478
ENSMUSG00000006058	Snf8	SNF8, ESCRT-II complex subunit, homolog (S. cerevi	0.36484
ENSMUSG00000025889	Snca	synuclein, alpha [Source:MGI Symbol;Acc:MGI:127	0.36497
ENSMUSG00000025557	Slc15a1	solute carrier family 15 (oligopeptide transporter),	0.36501
ENSMUSG00000045503	Sys1	SYS1 Golgi-localized integral membrane protein ho	0.36508
ENSMUSG00000058254	Tspan7	tetraspanin 7 [Source:MGI Symbol;Acc:MGI:129840	0.36508
ENSMUSG00000074818	Pdzd7	PDZ domain containing 7 [Source:MGI Symbol;Acc:	0.36517
ENSMUSG00000055312	0610012H03Rik	RIKEN cDNA 0610012H03 gene [Source:MGI Symbo	0.36523
ENSMUSG00000015994	Fnta	farnesyltransferase, CAAX box, alpha [Source:MGI S	0.36527
ENSMUSG00000029146	Snx17	sorting nexin 17 [Source:MGI Symbol;Acc:MGI:238	0.36529
ENSMUSG00000015846	Rxra	retinoid X receptor alpha [Source:MGI Symbol;Acc:	0.36531
ENSMUSG00000021037	Ahsa1	AHA1, activator of heat shock protein ATPase 1 [So	0.36543
ENSMUSG00000022747	St3gal6	ST3 beta-galactoside alpha-2,3-sialyltransferase 6 [	0.36546
ENSMUSG00000027353	Mcm8	minichromosome maintenance deficient 8 (S. cerev	0.36547
ENSMUSG00000069441	Dsg1a	desmoglein 1 alpha [Source:MGI Symbol;Acc:MGI:9	0.36547
ENSMUSG00000058488	Kl	klotho [Source:MGI Symbol;Acc:MGI:1101771]	0.36548
ENSMUSG00000007035	Msh5	mutS homolog 5 (E. coli) [Source:MGI Symbol;Acc:M	0.3655
ENSMUSG00000000740	Rpl13	ribosomal protein L13 [Source:MGI Symbol;Acc:MG	0.36552
ENSMUSG00000097855	A930007I19Rik	RIKEN cDNA A930007I19 gene [Source:MGI Symbol	0.36561
ENSMUSG00000013091	Tmem190	transmembrane protein 190 [Source:MGI Symbol;A	0.3657
ENSMUSG00000056271	Lman1l	lectin, mannose-binding 1 like [Source:MGI Symbol	0.3657
ENSMUSG00000013160	Atp6v0d1	ATPase, H+ transporting, lysosomal V0 subunit D1 [	0.36571
ENSMUSG00000062040	Zfp27	zinc finger protein 27 [Source:MGI Symbol;Acc:MG	0.36571
ENSMUSG00000048218	Amigo2	adhesion molecule with Ig like domain 2 [Source:M	0.36574
ENSMUSG00000035139	Secisbp2	SECIS binding protein 2 [Source:MGI Symbol;Acc:M	0.36575
ENSMUSG00000021668	Polk	polymerase (DNA directed), kappa [Source:MGI Sym	0.36593
ENSMUSG00000085584	Rgag1	retrotransposon gag domain containing 1 [Source:M	0.36618
ENSMUSG00000068267	Cenpb	centromere protein B [Source:MGI Symbol;Acc:MG	0.3662
ENSMUSG00000032017	Grik4	glutamate receptor, ionotropic, kainate 4 [Source:M	0.3663
ENSMUSG00000036504	Phpt1	phosphohistidine phosphatase 1 [Source:MGI Symb	0.36642
ENSMUSG00000029762	Akr1b8	aldo-keto reductase family 1, member B8 [Source:M	0.36658
ENSMUSG00000029587	Zfp12	zinc finger protein 12 [Source:MGI Symbol;Acc:MG	0.3667
ENSMUSG00000037364	Srrt	serrate RNA effector molecule homolog (Arabidops	0.36682
ENSMUSG00000035245	Eogt	EGF domain-specific O-linked N-acetylglucosamine	0.36685
ENSMUSG00000079022	Col22a1	collagen, type XXII, alpha 1 [Source:MGI Symbol;Ac	0.36697
ENSMUSG00000033985	Tesk2	testis-specific kinase 2 [Source:MGI Symbol;Acc:MG	0.36719
ENSMUSG00000028541	B4galt2	UDP-Gal:betaGlcNAc beta 1,4- galactosyltransfera	0.36736
ENSMUSG00000018776	Slc35g3	solute carrier family 35, member G3 [Source:MGI S	0.36742
ENSMUSG00000031400	G6pdx	glucose-6-phosphate dehydrogenase X-linked [Sou	0.36743
ENSMUSG00000030148	Clec4a2	C-type lectin domain family 4, member a2 [Source:	0.36748
ENSMUSG00000020439	Smtn	smoothelin [Source:MGI Symbol;Acc:MGI:1354727	0.36749
ENSMUSG00000079553	Kifc1	kinesin family member C1 [Source:MGI Symbol;Acc	0.36755
ENSMUSG00000038486	Sv2a	synaptic vesicle glycoprotein 2 a [Source:MGI Symb	0.36765
ENSMUSG00000028599	Tnfrsf1b	tumor necrosis factor receptor superfamily, memb	0.36766

ENSMUSG00000044966	Fbxo48	F-box protein 48 [Source:MGI Symbol;Acc:MGI:244	0.36768
ENSMUSG00000032394	Igdcc3	immunoglobulin superfamily, DCC subclass, membe	0.36771
ENSMUSG00000016487	Ppfibp1	PTPRF interacting protein, binding protein 1 (liprin	0.36773
ENSMUSG00000049493	Pls1	plastin 1 (I-isoform) [Source:MGI Symbol;Acc:MGI:1	0.36774
ENSMUSG00000092680	Snord55	small nucleolar RNA, C/D box 55 [Source:MGI Symb	0.3678
ENSMUSG00000049295	Zfp219	zinc finger protein 219 [Source:MGI Symbol;Acc:MG	0.36781
ENSMUSG00000024927	Rela	v-rel reticuloendotheliosis viral oncogene homolog	0.36792
ENSMUSG00000052419	2610001J05Rik	RIKEN cDNA 2610001J05 gene [Source:MGI Symbol	0.36797
ENSMUSG00000098123	Gm4518	predicted gene 4518 [Source:MGI Symbol;Acc:MGI:	0.36801
ENSMUSG00000050248	Evc2	Ellis van Creveld syndrome 2 [Source:MGI Symbol;A	0.36809
ENSMUSG00000086464	Gm15835	predicted gene 15835 [Source:MGI Symbol;Acc:MG	0.36815
ENSMUSG00000007888	Crlf1	cytokine receptor-like factor 1 [Source:MGI Symbol	0.3682
ENSMUSG00000086078	Gm11657	predicted gene 11657 [Source:MGI Symbol;Acc:MG	0.36822
ENSMUSG00000014294	Ndufa2	NADH dehydrogenase (ubiquinone) 1 alpha subcom	0.36824
ENSMUSG00000036502	Tmem255a	transmembrane protein 255A [Source:MGI Symbol;	0.36831
ENSMUSG00000086352	4930401G09Rik	RIKEN cDNA 4930401G09 gene [Source:MGI Symbo	0.36832
ENSMUSG00000018919	Tm4sf5	transmembrane 4 superfamily member 5 [Source:M	0.36836
ENSMUSG00000082320	Gm11877	predicted gene 11877 [Source:MGI Symbol;Acc:MG	0.36836
ENSMUSG00000086375	Gm12127	predicted gene 12127 [Source:MGI Symbol;Acc:MG	0.36838
ENSMUSG00000095780	Gm24674	predicted gene, 24674 [Source:MGI Symbol;Acc:MG	0.36838
ENSMUSG00000021102	Glrx5	glutaredoxin 5 homolog (S. cerevisiae) [Source:MGI	0.36849
ENSMUSG00000070342	Gm10287	predicted gene 10287 [Source:MGI Symbol;Acc:MG	0.36855
ENSMUSG00000060548	Tnfrsf19	tumor necrosis factor receptor superfamily, membe	0.36857
ENSMUSG00000049755	Zfp672	zinc finger protein 672 [Source:MGI Symbol;Acc:MG	0.36859
ENSMUSG00000031951	Tmem231	transmembrane protein 231 [Source:MGI Symbol;A	0.36875
ENSMUSG00000024423	Impact	imprinted and ancient [Source:MGI Symbol;Acc:MG	0.36878
ENSMUSG00000030924	2610020H08Rik	RIKEN cDNA 2610020H08 gene [Source:MGI Symbo	0.36879
ENSMUSG00000076315	Mir343	microRNA 343 [Source:MGI Symbol;Acc:MGI:37185	0.36882
ENSMUSG00000098105	Gm8825	predicted gene 8825 [Source:MGI Symbol;Acc:MGI:	0.36882
ENSMUSG00000037278	Tmem97	transmembrane protein 97 [Source:MGI Symbol;Ac	0.36887
ENSMUSG00000032239	Rp9	retinitis pigmentosa 9 (human) [Source:MGI Symbo	0.36895
ENSMUSG00000032500	Dclk3	doublecortin-like kinase 3 [Source:MGI Symbol;Acc	0.36895
ENSMUSG00000081565	Rps12-ps5	ribosomal protein S12, pseudogene 5 [Source:MGI	0.36912
ENSMUSG00000028415	Spink4	serine peptidase inhibitor, Kazal type 4 [Source:MG	0.36915
ENSMUSG00000063430	Wscd2	WSC domain containing 2 [Source:MGI Symbol;Acc	0.36915
ENSMUSG00000019179	Mdh2	malate dehydrogenase 2, NAD (mitochondrial) [Sou	0.36921
ENSMUSG00000044627	Swi5	SWI5 recombination repair homolog (yeast) [Source	0.36929
ENSMUSG00000018900	Slc22a5	solute carrier family 22 (organic cation transporter)	0.36933
ENSMUSG00000022676	Snai2	snail homolog 2 (Drosophila) [Source:MGI Symbol;A	0.36944
ENSMUSG00000001552	Jup	junction plakoglobin [Source:MGI Symbol;Acc:MGI:	0.36947
ENSMUSG00000039956	Mrap	melanocortin 2 receptor accessory protein [Source:	0.3695
ENSMUSG00000025128	Bhlhe22	basic helix-loop-helix family, member e22 [Source:I	0.36952
ENSMUSG00000085956	4930481B07Rik	RIKEN cDNA 4930481B07 gene [Source:MGI Symbo	0.36961
ENSMUSG00000040867	Begain	brain-enriched guanylate kinase-associated [Source	0.36966
ENSMUSG00000074607	Tox2	TOX high mobility group box family member 2 [Sou	0.3698
ENSMUSG00000049044	Rapgef4	Rap guanine nucleotide exchange factor (GEF) 4 [So	0.36987
ENSMUSG00000049090	Zadh2	zinc binding alcohol dehydrogenase, domain contai	0.36989

ENSMUSG00000045288	Ush1g	Usher syndrome 1G [Source:MGI Symbol;Acc:MGI:2	0.36995
ENSMUSG00000083897	Gm14843	predicted gene 14843 [Source:MGI Symbol;Acc:MG	0.37019
ENSMUSG00000093040	Gm26141	predicted gene, 26141 [Source:MGI Symbol;Acc:MC	0.37022
ENSMUSG00000027102	Hoxd8	homeobox D8 [Source:MGI Symbol;Acc:MGI:96209	0.37024
ENSMUSG00000083122	Gm15578	predicted gene 15578 [Source:MGI Symbol;Acc:MG	0.37024
ENSMUSG00000033967	2310014L17Rik	RIKEN cDNA 2310014L17 gene [Source:MGI Symbo	0.37027
ENSMUSG00000013150	Gfod2	glucose-fructose oxidoreductase domain containing	0.37032
ENSMUSG00000085573	Gm15418	predicted gene 15418 [Source:MGI Symbol;Acc:MG	0.37035
ENSMUSG00000048070	Pirt	phosphoinositide-interacting regulator of transient	0.37051
ENSMUSG00000062417	Hist1h3g	histone cluster 1, H3g [Source:MGI Symbol;Acc:MG	0.37052
ENSMUSG00000054626	Xlr	X-linked lymphocyte-regulated complex [Source:MC	0.37055
ENSMUSG00000040720	1110037F02Rik	RIKEN cDNA 1110037F02 gene [Source:MGI Symbo	0.37067
ENSMUSG00000020843	Timm22	translocase of inner mitochondrial membrane 22 [S	0.37068
ENSMUSG00000022209	Dhrs2	dehydrogenase/reductase member 2 [Source:MGI S	0.37071
ENSMUSG00000036613	Tssc1	tumor suppressing subtransferable candidate 1 [So	0.37073
ENSMUSG00000075569	Rsph10b	radial spoke head 10 homolog B (Chlamydomonas)	0.37097
ENSMUSG00000028246	Faxc	failed axon connections homolog (Drosophila) [Sou	0.37103
ENSMUSG00000035112	Wnk4	WNK lysine deficient protein kinase 4 [Source:MGI	0.37112
ENSMUSG00000034412	Tbc1d10a	TBC1 domain family, member 10a [Source:MGI Syn	0.37113
ENSMUSG00000070733	Fryl	furry homolog-like (Drosophila) [Source:MGI Symbo	0.37114
ENSMUSG00000040270	Bach2	BTB and CNC homology 2 [Source:MGI Symbol;Acc:	0.37122
ENSMUSG00000063316	Rpl27	ribosomal protein L27 [Source:MGI Symbol;Acc:MG	0.37128
ENSMUSG00000004609	Cd33	CD33 antigen [Source:MGI Symbol;Acc:MGI:99440]	0.37137
ENSMUSG00000095306	Gm21807	predicted gene, 21807 [Source:MGI Symbol;Acc:MC	0.37137
ENSMUSG00000034201	Gas2l1	growth arrest-specific 2 like 1 [Source:MGI Symbol;	0.37139
ENSMUSG00000059325	Hopx	HOP homeobox [Source:MGI Symbol;Acc:MGI:1916	0.37145
ENSMUSG00000031344	Gabrq	gamma-aminobutyric acid (GABA) A receptor, subu	0.37147
ENSMUSG00000052397	Ezr	ezrin [Source:MGI Symbol;Acc:MGI:98931]	0.37148
ENSMUSG00000020183	Cpm	carboxypeptidase M [Source:MGI Symbol;Acc:MGI:	0.3716
ENSMUSG00000021792	Fam213a	family with sequence similarity 213, member A [So	0.3716
ENSMUSG00000045969	Ing1	inhibitor of growth family, member 1 [Source:MGI S	0.37163
ENSMUSG00000004085	Zak	sterile alpha motif and leucine zipper containing kir	0.37181
ENSMUSG00000033192	Lpcat2	lysophosphatidylcholine acyltransferase 2 [Source:I	0.37181
ENSMUSG00000097059	Fam120aos	family with sequence similarity 120A opposite strar	0.37183
ENSMUSG00000022505	Emp2	epithelial membrane protein 2 [Source:MGI Symbo	0.37198
ENSMUSG00000097451	Rian	RNA imprinted and accumulated in nucleus [Source	0.3721
ENSMUSG00000027411	Vps16	vacuolar protein sorting 16 (yeast) [Source:MGI Syr	0.37214
ENSMUSG00000039146	Ifi44l	interferon-induced protein 44 like [Source:MGI Sym	0.37217
ENSMUSG00000068466	Gm5518	predicted gene 5518 [Source:MGI Symbol;Acc:MGI:	0.37218
ENSMUSG00000082436	Gm11688	predicted gene 11688 [Source:MGI Symbol;Acc:MG	0.37227
ENSMUSG00000018537	Pcgf2	polycomb group ring finger 2 [Source:MGI Symbol;A	0.37246
ENSMUSG00000054589	Gm9949	predicted gene 9949 [Source:MGI Symbol;Acc:MGI:	0.37248
ENSMUSG00000021036	Sptlc2	serine palmitoyltransferase, long chain base subuni	0.37251
ENSMUSG00000085380	E130120K24Rik	RIKEN cDNA E130120K24 gene [Source:MGI Symbo	0.37251
ENSMUSG00000084215	Gm11814	predicted gene 11814 [Source:MGI Symbol;Acc:MG	0.37254
ENSMUSG00000097840	Gm26756	predicted gene, 26756 [Source:MGI Symbol;Acc:MC	0.37255
ENSMUSG00000014039	Prdm15	PR domain containing 15 [Source:MGI Symbol;Acc:I	0.37258

ENSMUSG00000056167	Cnot10	CCR4-NOT transcription complex, subunit 10 [Sourc	0.3726
ENSMUSG00000008475	Arpc5	actin related protein 2/3 complex, subunit 5 [Sourc	0.37261
ENSMUSG00000012889	Podnl1	podocan-like 1 [Source:MGI Symbol;Acc:MGI:26853	0.37265
ENSMUSG00000019718	L3hypdh	L-3-hydroxyproline dehydratase (trans-) [Source:M	0.37266
ENSMUSG00000084390	Gm15425	predicted gene 15425 [Source:MGI Symbol;Acc:MG	0.37279
ENSMUSG00000052429	Prmt1	protein arginine N-methyltransferase 1 [Source:MG	0.37283
ENSMUSG00000025782	Taf3	TAF3 RNA polymerase II, TATA box binding protein	0.373
ENSMUSG00000038290	Smg6	Smg-6 homolog, nonsense mediated mRNA decay f	0.37301
ENSMUSG00000060128	Gm10075	predicted gene 10075 [Source:MGI Symbol;Acc:MG	0.37304
ENSMUSG00000071753	C230004F18Rik	RIKEN cDNA C230004F18 gene [Source:MGI Symbo	0.37304
ENSMUSG00000022718	Dgcr8	DiGeorge syndrome critical region gene 8 [Source:M	0.37308
ENSMUSG00000026563	Tada1	transcriptional adaptor 1 [Source:MGI Symbol;Acc:	0.37309
ENSMUSG00000041423	Paqr6	progestin and adipoQ receptor family member VI [S	0.37319
ENSMUSG00000092361	Gm20433	predicted gene 20433 [Source:MGI Symbol;Acc:MG	0.37323
ENSMUSG00000043972	Opn5	opsin 5 [Source:MGI Symbol;Acc:MGI:2662912]	0.37324
ENSMUSG00000080478	Snord23	small nucleolar RNA, C/D box 23 [Source:MGI Symb	0.37325
ENSMUSG00000087526	Gm15738	predicted gene 15738 [Source:MGI Symbol;Acc:MG	0.37325
ENSMUSG00000074758	Gm5535	predicted gene 5535 [Source:MGI Symbol;Acc:MGI	0.37329
ENSMUSG00000038828	Tmem214	transmembrane protein 214 [Source:MGI Symbol;A	0.37331
ENSMUSG00000034227	Foxj1	forkhead box J1 [Source:MGI Symbol;Acc:MGI:1347	0.37336
ENSMUSG00000020357	Flt4	FMS-like tyrosine kinase 4 [Source:MGI Symbol;Acc	0.3734
ENSMUSG00000005881	Ergic3	ERGIC and golgi 3 [Source:MGI Symbol;Acc:MGI:19	0.37341
ENSMUSG00000085639	Gm15410	predicted gene 15410 [Source:MGI Symbol;Acc:MG	0.37361
ENSMUSG00000027227	Sord	sorbitol dehydrogenase [Source:MGI Symbol;Acc:M	0.37369
ENSMUSG00000078591	Hs3st4	heparan sulfate (glucosamine) 3-O-sulfotransferase	0.37373
ENSMUSG00000096269	Rpl31-ps21	ribosomal protein L31, pseudogene 21 [Source:MG	0.37396
ENSMUSG00000021485	Mxd3	Max dimerization protein 3 [Source:MGI Symbol;Ac	0.37401
ENSMUSG00000041891	Lman1	lectin, mannose-binding, 1 [Source:MGI Symbol;Acc	0.37407
ENSMUSG00000002006	Pdzd4	PDZ domain containing 4 [Source:MGI Symbol;Acc:	0.3741
ENSMUSG00000058833	2810428I15Rik	RIKEN cDNA 2810428I15 gene [Source:MGI Symbol	0.37416
ENSMUSG00000078453	Abracl	ABRA C-terminal like [Source:MGI Symbol;Acc:MGI	0.37416
ENSMUSG00000043013	Onecut1	one cut domain, family member 1 [Source:MGI Sym	0.37419
ENSMUSG00000054885	4930578G10Rik	RIKEN cDNA 4930578G10 gene [Source:MGI Symbo	0.37429
ENSMUSG00000003227	Edar	ectodysplasin-A receptor [Source:MGI Symbol;Acc:	0.3743
ENSMUSG00000022568	Scrib	scribbled homolog (Drosophila) [Source:MGI Symbo	0.37436
ENSMUSG00000093599	Gm23572	predicted gene, 23572 [Source:MGI Symbol;Acc:M	0.37436
ENSMUSG00000015721	Nlrp5	NLR family, pyrin domain containing 5 [Source:MGI	0.37441
ENSMUSG00000041720	Pi4ka	phosphatidylinositol 4-kinase, catalytic, alpha polyp	0.37452
ENSMUSG00000068747	Sort1	sortilin 1 [Source:MGI Symbol;Acc:MGI:1338015]	0.37454
ENSMUSG00000044103	Il1f9	interleukin 1 family, member 9 [Source:MGI Symbo	0.3746
ENSMUSG00000097338	Gm2464	predicted gene 2464 [Source:MGI Symbol;Acc:MGI	0.37463
ENSMUSG00000073231	Gm10483	predicted gene 10483 [Source:MGI Symbol;Acc:MG	0.37465
ENSMUSG00000032519	Slc25a38	solute carrier family 25, member 38 [Source:MGI Sy	0.37477
ENSMUSG00000041079	Rwdd2b	RWD domain containing 2B [Source:MGI Symbol;Ac	0.37479
ENSMUSG00000028224	Nbn	nibrin [Source:MGI Symbol;Acc:MGI:1351625]	0.37489
ENSMUSG00000090608	Gm17200	predicted gene 17200 [Source:MGI Symbol;Acc:MG	0.37507
ENSMUSG00000015937	H2afy	H2A histone family, member Y [Source:MGI Symbo	0.37515

ENSMUSG00000030602	Pak4	p21 protein (Cdc42/Rac)-activated kinase 4 [Source	0.37518
ENSMUSG00000072963	Gm10447	predicted gene 10447 [Source:MGI Symbol;Acc:MG	0.37531
ENSMUSG00000035697	Hmha1	histocompatibility (minor) HA-1 [Source:MGI Symb	0.37532
ENSMUSG00000037376	Trmt6	tRNA methyltransferase 6 [Source:MGI Symbol;Acc	0.3754
ENSMUSG00000031530	Dusp4	dual specificity phosphatase 4 [Source:MGI Symbol	0.37541
ENSMUSG00000029179	Zcchc4	zinc finger, CCHC domain containing 4 [Source:MGI	0.37547
ENSMUSG00000016918	Sulf1	sulfatase 1 [Source:MGI Symbol;Acc:MGI:2138563]	0.37548
ENSMUSG00000036452	Arhgap26	Rho GTPase activating protein 26 [Source:MGI Sym	0.37552
ENSMUSG00000006998	Psmd2	proteasome (prosome, macropain) 26S subunit, no	0.37554
ENSMUSG00000039043	2610034B18Rik	RIKEN cDNA 2610034B18 gene [Source:MGI Symbo	0.37567
ENSMUSG00000021365	Nedd9	neural precursor cell expressed, developmentally d	0.37569
ENSMUSG00000026248	Mrpl44	mitochondrial ribosomal protein L44 [Source:MGI S	0.37582
ENSMUSG00000022619	Mapk8ip2	mitogen-activated protein kinase 8 interacting prot	0.37586
ENSMUSG00000097493	9930014A18Rik	RIKEN cDNA 9930014A18 gene [Source:MGI Symbo	0.37603
ENSMUSG00000026223	Itm2c	integral membrane protein 2C [Source:MGI Symbol	0.37605
ENSMUSG00000036281	Snapc4	small nuclear RNA activating complex, polypeptide	0.37611
ENSMUSG00000055826	Tescl	tescalcin-like [Source:MGI Symbol;Acc:MGI:191655	0.37616
ENSMUSG00000086420	Gm8865	predicted gene 8865 [Source:MGI Symbol;Acc:MGI:	0.37619
ENSMUSG00000029524	Sirt4	sirtuin 4 [Source:MGI Symbol;Acc:MGI:1922637]	0.37623
ENSMUSG00000079492	Gm11127	predicted gene 11127 [Source:MGI Symbol;Acc:MG	0.37633
ENSMUSG00000015165	Hnrnpl	heterogeneous nuclear ribonucleoprotein L [Source	0.3764
ENSMUSG00000018677	Slc25a39	solute carrier family 25, member 39 [Source:MGI Sy	0.37651
ENSMUSG00000020485	Supt4a	suppressor of Ty 4A [Source:MGI Symbol;Acc:MGI:1	0.37665
ENSMUSG00000026820	Ptges2	prostaglandin E synthase 2 [Source:MGI Symbol;Ac	0.37667
ENSMUSG00000044197	Gpr146	G protein-coupled receptor 146 [Source:MGI Symb	0.37667
ENSMUSG00000028970	Abcb1b	ATP-binding cassette, sub-family B (MDR/TAP), me	0.37668
ENSMUSG00000023079	Gtf2ird1	general transcription factor II I repeat domain-cont	0.37669
ENSMUSG00000060181	Slc35e3	solute carrier family 35, member E3 [Source:MGI Sy	0.37683
ENSMUSG00000013766	Ly6g6e	lymphocyte antigen 6 complex, locus G6E [Source:M	0.37704
ENSMUSG00000032875	Arhgef17	Rho guanine nucleotide exchange factor (GEF) 17 [S	0.37706
ENSMUSG00000025731	0610011F06Rik	RIKEN cDNA 0610011F06 gene [Source:MGI Symbo	0.37707
ENSMUSG00000058706	0610030E20Rik	RIKEN cDNA 0610030E20 gene [Source:MGI Symbo	0.37708
ENSMUSG00000098076	Gm6981	predicted pseudogene 6981 [Source:MGI Symbol;A	0.37711
ENSMUSG00000006941	Eif1b	eukaryotic translation initiation factor 1B [Source:M	0.37714
ENSMUSG00000065036	Gm22362	predicted gene, 22362 [Source:MGI Symbol;Acc:M	0.37716
ENSMUSG00000083745	Gm13543	predicted gene 13543 [Source:MGI Symbol;Acc:MG	0.37719
ENSMUSG00000048065	Cyb5r2	cytochrome b5 reductase 2 [Source:MGI Symbol;Ac	0.3772
ENSMUSG00000085667	Gm12992	predicted gene 12992 [Source:MGI Symbol;Acc:MG	0.37737
ENSMUSG00000031605	Klhl2	kelch-like 2, Mayven [Source:MGI Symbol;Acc:MGI:	0.37746
ENSMUSG00000086602	Gm15609	predicted gene 15609 [Source:MGI Symbol;Acc:MG	0.3777
ENSMUSG00000026277	Stk25	serine/threonine kinase 25 (yeast) [Source:MGI Sym	0.37782
ENSMUSG00000030556	Lrrc28	leucine rich repeat containing 28 [Source:MGI Symb	0.37799
ENSMUSG00000030785	Cox6a2	cytochrome c oxidase subunit VIa polypeptide 2 [So	0.37799
ENSMUSG00000037373	Ctbp1	C-terminal binding protein 1 [Source:MGI Symbol;A	0.37801
ENSMUSG00000031482	Slc25a15	solute carrier family 25 (mitochondrial carrier ornit	0.37804
ENSMUSG00000042589	Cux2	cut-like homeobox 2 [Source:MGI Symbol;Acc:MGI:	0.37817
ENSMUSG00000097903	Gm26679	predicted gene, 26679 [Source:MGI Symbol;Acc:M	0.37821

ENSMUSG00000055401	Fbxo6	F-box protein 6 [Source:MGI Symbol;Acc:MGI:1354	0.37837
ENSMUSG00000031913	Vps4a	vacuolar protein sorting 4a (yeast) [Source:MGI Syr	0.37839
ENSMUSG00000032959	Pebp1	phosphatidylethanolamine binding protein 1 [Sourc	0.37851
ENSMUSG00000066196	Spag8	sperm associated antigen 8 [Source:MGI Symbol;Ac	0.37864
ENSMUSG00000084315	Vmn1r-ps128	vomeronasal 1 receptor, pseudogene 128 [Source:I	0.37869
ENSMUSG00000038764	Ptpn3	protein tyrosine phosphatase, non-receptor type 3	0.37874
ENSMUSG00000026519	Tmem63a	transmembrane protein 63a [Source:MGI Symbol;A	0.37875
ENSMUSG00000081998	Gm13245	predicted gene 13245 [Source:MGI Symbol;Acc:MG	0.37875
ENSMUSG00000048520	Fbxl13	F-box and leucine-rich repeat protein 13 [Source:M	0.37877
ENSMUSG00000040260	Daam2	dishevelled associated activator of morphogenesis	0.37879
ENSMUSG00000042532	Golga7b	golgi autoantigen, golgin subfamily a, 7B [Source:M	0.37879
ENSMUSG00000006575	Rundc3a	RUN domain containing 3A [Source:MGI Symbol;Ac	0.37894
ENSMUSG00000062758	Gm16477	predicted pseudogene 16477 [Source:MGI Symbol;	0.37896
ENSMUSG00000075032	Hist1h3i	histone cluster 1, H3i [Source:MGI Symbol;Acc:MGI	0.37898
ENSMUSG00000027446	9230104L09Rik	RIKEN cDNA 9230104L09 gene [Source:MGI Symbo	0.37899
ENSMUSG00000083436	Gm11471	predicted gene 11471 [Source:MGI Symbol;Acc:MG	0.37899
ENSMUSG00000097049	6530411M01Rik	RIKEN cDNA 6530411M01 gene [Source:MGI Symb	0.37899
ENSMUSG00000026301	Iqca	IQ motif containing with AAA domain [Source:MGI	0.379
ENSMUSG00000091941	Gm7399	predicted gene 7399 [Source:MGI Symbol;Acc:MGI:	0.37902
ENSMUSG00000093006	Gm24157	predicted gene, 24157 [Source:MGI Symbol;Acc:MC	0.37902
ENSMUSG00000093919	Gm20760	predicted gene, 20760 [Source:MGI Symbol;Acc:MC	0.37902
ENSMUSG00000093485	Gm20708	predicted gene 20708 [Source:MGI Symbol;Acc:MG	0.37904
ENSMUSG00000087500	Gm12426	predicted gene 12426 [Source:MGI Symbol;Acc:MG	0.37905
ENSMUSG00000086883	Gm15992	predicted gene 15992 [Source:MGI Symbol;Acc:MG	0.37907
ENSMUSG00000086763	B230378P21Rik	RIKEN cDNA B230378P21 gene [Source:MGI Symbo	0.37908
ENSMUSG00000087014	Gm16364	predicted gene 16364 [Source:MGI Symbol;Acc:MG	0.37911
ENSMUSG00000085932	Gm15556	predicted gene 15556 [Source:MGI Symbol;Acc:MG	0.37912
ENSMUSG00000034330	Plcg2	phospholipase C, gamma 2 [Source:MGI Symbol;Ac	0.37913
ENSMUSG00000090053	Palm2	paralemmin 2 [Source:MGI Symbol;Acc:MGI:19346	0.37915
ENSMUSG00000031142	Cacna1f	calcium channel, voltage-dependent, alpha 1F subu	0.3792
ENSMUSG00000097405	D630044L22Rik	RIKEN cDNA gene D630044L22 gene [Source:MGI S	0.37929
ENSMUSG00000089336	Gm24347	predicted gene, 24347 [Source:MGI Symbol;Acc:MC	0.37938
ENSMUSG00000087306	A230004M16Rik	RIKEN cDNA A230004M16 gene [Source:MGI Symb	0.37939
ENSMUSG00000086348	1700017J07Rik	RIKEN cDNA 1700017J07 gene [Source:MGI Symbol	0.3794
ENSMUSG00000089432	Mir1966	microRNA 1966 [Source:MGI Symbol;Acc:MGI:3837	0.3794
ENSMUSG00000089188	Gm22942	predicted gene, 22942 [Source:MGI Symbol;Acc:MC	0.37941
ENSMUSG00000024909	Efemp2	epidermal growth factor-containing fibulin-like extr	0.37942
ENSMUSG00000082988	Gm5903	predicted gene 5903 [Source:MGI Symbol;Acc:MGI:	0.37944
ENSMUSG00000056900	Usp13	ubiquitin specific peptidase 13 (isopeptidase T-3) [S	0.37945
ENSMUSG00000076376	Mir674	microRNA 674 [Source:MGI Symbol;Acc:MGI:36296	0.37945
ENSMUSG00000083811	Gm13071	predicted gene 13071 [Source:MGI Symbol;Acc:MG	0.37945
ENSMUSG00000097664	Gm26765	predicted gene, 26765 [Source:MGI Symbol;Acc:MC	0.37945
ENSMUSG00000065513	Mir26a-1	microRNA 26a-1 [Source:MGI Symbol;Acc:MGI:267	0.37947
ENSMUSG00000095281	Gm22549	predicted gene, 22549 [Source:MGI Symbol;Acc:MC	0.37949
ENSMUSG00000054044	Gm9933	predicted gene 9933 [Source:MGI Symbol;Acc:MGI:	0.3795
ENSMUSG00000097060	Gm26759	predicted gene, 26759 [Source:MGI Symbol;Acc:MC	0.3795
ENSMUSG00000030228	Pik3c2g	phosphatidylinositol 3-kinase, C2 domain containin	0.37954

ENSMUSG00000098073	Gm8048	predicted gene 8048 [Source:MGI Symbol;Acc:MGI:	0.37955
ENSMUSG00000032363	Adamts7	a disintegrin-like and metallopeptidase (reprolysin t	0.37956
ENSMUSG00000091668	Gm8358	predicted gene 8358 [Source:MGI Symbol;Acc:MGI:	0.37956
ENSMUSG00000094018	S100a2	S100 calcium binding protein A2 [Source:MGI Symb	0.37957
ENSMUSG00000047230	Cldn2	claudin 2 [Source:MGI Symbol;Acc:MGI:1276110]	0.37959
ENSMUSG00000016356	Col20a1	collagen, type XX, alpha 1 [Source:MGI Symbol;Acc:	0.3796
ENSMUSG00000022622	Acr	acrosin prepropeptide [Source:MGI Symbol;Acc:MG	0.3796
ENSMUSG00000078821	Gm17414	predicted gene, 17414 [Source:MGI Symbol;Acc:MG	0.3796
ENSMUSG00000087239	Gm7613	predicted gene 7613 [Source:MGI Symbol;Acc:MGI:	0.3796
ENSMUSG00000029730	Mcm7	minichromosome maintenance deficient 7 (S. cerev	0.37962
ENSMUSG00000081999	Gm13461	predicted gene 13461 [Source:MGI Symbol;Acc:MG	0.37963
ENSMUSG00000081572	Gm14138	predicted gene 14138 [Source:MGI Symbol;Acc:MG	0.37966
ENSMUSG00000076424	Gm25986	predicted gene, 25986 [Source:MGI Symbol;Acc:MG	0.37969
ENSMUSG00000046032	Snx12	sorting nexin 12 [Source:MGI Symbol;Acc:MGI:191	0.37973
ENSMUSG00000074236	Gm9387	predicted pseudogene 9387 [Source:MGI Symbol;A	0.37974
ENSMUSG00000040935	Padi6	peptidyl arginine deiminase, type VI [Source:MGI Sy	0.37977
ENSMUSG00000027793	Ccna1	cyclin A1 [Source:MGI Symbol;Acc:MGI:108042]	0.37978
ENSMUSG00000071193	Gm4938	predicted pseudogene 4938 [Source:MGI Symbol;A	0.37978
ENSMUSG00000072894	Ppp1r2-ps2	protein phosphatase 1, regulatory (inhibitor) subun	0.37979
ENSMUSG00000010760	Phlda2	pleckstrin homology-like domain, family A, membe	0.37982
ENSMUSG00000062651	H2afb1	H2A histone family, member B1 [Source:MGI Symb	0.37982
ENSMUSG00000041841	Rpl37	ribosomal protein L37 [Source:MGI Symbol;Acc:MG	0.37983
ENSMUSG00000097785	B230217O12Rik	RIKEN cDNA B230217O12 gene [Source:MGI Symbo	0.37985
ENSMUSG00000041872	Il17f	interleukin 17F [Source:MGI Symbol;Acc:MGI:2676	0.3799
ENSMUSG00000040133	Gpr176	G protein-coupled receptor 176 [Source:MGI Symb	0.37993
ENSMUSG00000026834	Acvr1c	activin A receptor, type IC [Source:MGI Symbol;Acc	0.37996
ENSMUSG00000024462	Gabbr1	gamma-aminobutyric acid (GABA) B receptor, 1 [So	0.37997
ENSMUSG00000026715	Serpinc1	serine (or cysteine) peptidase inhibitor, clade C (an	0.38005
ENSMUSG00000087267	4933427J07Rik	RIKEN cDNA 4933427J07 gene [Source:MGI Symbol	0.38007
ENSMUSG00000064609	Gm25635	predicted gene, 25635 [Source:MGI Symbol;Acc:MG	0.38009
ENSMUSG00000085377	4930551L18Rik	RIKEN cDNA 4930551L18 gene [Source:MGI Symbo	0.38014
ENSMUSG00000085682	Gm14267	predicted gene 14267 [Source:MGI Symbol;Acc:MG	0.38016
ENSMUSG00000030020	Prickle2	prickle homolog 2 (Drosophila) [Source:MGI Symbo	0.3802
ENSMUSG00000013076	Amotl1	angiomotin-like 1 [Source:MGI Symbol;Acc:MGI:19	0.38025
ENSMUSG00000024241	Sos1	son of sevenless homolog 1 (Drosophila) [Source:M	0.3803
ENSMUSG00000030447	Cyfip1	cytoplasmic FMR1 interacting protein 1 [Source:MG	0.3803
ENSMUSG00000087057	Gm11730	predicted gene 11730 [Source:MGI Symbol;Acc:MG	0.38033
ENSMUSG00000056162	Cndp1	carnosine dipeptidase 1 (metallopeptidase M20 far	0.38039
ENSMUSG00000021182	Ccdc88c	coiled-coil domain containing 88C [Source:MGI Sym	0.3805
ENSMUSG00000079593	Gm14597	predicted gene 14597 [Source:MGI Symbol;Acc:MG	0.38053
ENSMUSG00000096422	Igkv12-44	immunoglobulin kappa variable 12-44 [Source:MGI	0.38056
ENSMUSG00000088604	Gm25820	predicted gene, 25820 [Source:MGI Symbol;Acc:MG	0.38057
ENSMUSG00000090509	Sfta2	surfactant associated 2 [Source:MGI Symbol;Acc:M	0.3806
ENSMUSG00000092109	Gm9376	predicted gene 9376 [Source:MGI Symbol;Acc:MGI:	0.3806
ENSMUSG00000096279	Gm9312	predicted gene 9312 [Source:MGI Symbol;Acc:MGI:	0.38061
ENSMUSG00000040706	Agmat	agmatine ureohydrolase (agmatinase) [Source:MGI	0.38064
ENSMUSG00000097729	2310015A10Rik	RIKEN cDNA 2310015A10 gene [Source:MGI Symbo	0.38071

ENSMUSG00000036949	Slc39a12	solute carrier family 39 (zinc transporter), member	0.38073
ENSMUSG00000052926	Rnaseh2a	ribonuclease H2, large subunit [Source:MGI Symbo	0.38075
ENSMUSG00000090272	Mndal	myeloid nuclear differentiation antigen like [Source	0.38079
ENSMUSG00000039831	Arhgap29	Rho GTPase activating protein 29 [Source:MGI Sym	0.38081
ENSMUSG00000035407	Kank4	KN motif and ankyrin repeat domains 4 [Source:MG	0.3809
ENSMUSG00000090779	Gm17110	predicted gene 17110 [Source:MGI Symbol;Acc:MG	0.3809
ENSMUSG00000030898	Cckbr	cholecystokinin B receptor [Source:MGI Symbol;Acc	0.38104
ENSMUSG00000028051	Hcn3	hyperpolarization-activated, cyclic nucleotide-gated	0.38105
ENSMUSG00000061536	Sec22c	SEC22 vesicle trafficking protein homolog C (S. cere	0.38107
ENSMUSG00000022867	Usp25	ubiquitin specific peptidase 25 [Source:MGI Symbo	0.38108
ENSMUSG00000068226	Gm6723	predicted pseudogene 6723 [Source:MGI Symbol;A	0.38116
ENSMUSG00000097839	Gm26909	predicted gene, 26909 [Source:MGI Symbol;Acc:MG	0.38121
ENSMUSG00000031392	Irak1	interleukin-1 receptor-associated kinase 1 [Source:	0.38125
ENSMUSG00000004988	Fxyd4	FXYD domain-containing ion transport regulator 4 [	0.38128
ENSMUSG00000010505	Myt1	myelin transcription factor 1 [Source:MGI Symbol;A	0.38137
ENSMUSG00000069515	Lyz1	lysozyme 1 [Source:MGI Symbol;Acc:MGI:96902]	0.38138
ENSMUSG00000034652	Cd300a	CD300A antigen [Source:MGI Symbol;Acc:MGI:244	0.38139
ENSMUSG00000026735	Ptf1a	pancreas specific transcription factor, 1a [Source:M	0.3814
ENSMUSG00000005949	Ctns	cystinosis, nephropathic [Source:MGI Symbol;Acc:M	0.38142
ENSMUSG00000033208	S100b	S100 protein, beta polypeptide, neural [Source:MG	0.38142
ENSMUSG00000040550	Otud6b	OTU domain containing 6B [Source:MGI Symbol;Ac	0.38146
ENSMUSG00000064841	Gm26205	predicted gene, 26205 [Source:MGI Symbol;Acc:MG	0.38146
ENSMUSG00000070420	Zscan25	zinc finger and SCAN domain containing 25 [Source	0.3815
ENSMUSG00000046287	Pnma3	paraneoplastic antigen MA3 [Source:MGI Symbol;A	0.38154
ENSMUSG00000001029	Icam2	intercellular adhesion molecule 2 [Source:MGI Sym	0.38155
ENSMUSG00000021196	Pfkp	phosphofructokinase, platelet [Source:MGI Symbol	0.38158
ENSMUSG00000043659	Npsr1	neuropeptide S receptor 1 [Source:MGI Symbol;Acc	0.38162
ENSMUSG00000039515	Ppp2r4	protein phosphatase 2A, regulatory subunit B (PR 5	0.38171
ENSMUSG00000082684	Gm12774	predicted gene 12774 [Source:MGI Symbol;Acc:MG	0.38173
ENSMUSG00000054855	Rnd1	Rho family GTPase 1 [Source:MGI Symbol;Acc:MGI:	0.38175
ENSMUSG00000046556	Zfp319	zinc finger protein 319 [Source:MGI Symbol;Acc:MG	0.38176
ENSMUSG00000032898	Fbxo21	F-box protein 21 [Source:MGI Symbol;Acc:MGI:192	0.38177
ENSMUSG00000022941	Ripply3	ripply3 homolog (zebrafish) [Source:MGI Symbol;Ac	0.38188
ENSMUSG00000084403	Rps15a-ps8	ribosomal protein S15A, pseudogene 8 [Source:MG	0.38191
ENSMUSG00000020096	Tbata	thymus, brain and testes associated [Source:MGI Sy	0.38196
ENSMUSG00000097344	Gm26732	predicted gene, 26732 [Source:MGI Symbol;Acc:MG	0.38197
ENSMUSG00000018143	Mafk	v-maf musculoaponeurotic fibrosarcoma oncogene	0.38213
ENSMUSG00000019848	Popdc3	popeye domain containing 3 [Source:MGI Symbol;A	0.38213
ENSMUSG00000034613	Ppm1h	protein phosphatase 1H (PP2C domain containing)	0.38216
ENSMUSG00000045246	Kcng4	potassium voltage-gated channel, subfamily G, mer	0.38228
ENSMUSG00000018417	Myo1b	myosin IB [Source:MGI Symbol;Acc:MGI:107752]	0.38238
ENSMUSG00000047098	Rnf31	ring finger protein 31 [Source:MGI Symbol;Acc:MGI	0.38238
ENSMUSG00000068220	Lgals1	lectin, galactose binding, soluble 1 [Source:MGI Syr	0.38252
ENSMUSG00000022208	Jph4	junctophilin 4 [Source:MGI Symbol;Acc:MGI:244311	0.38254
ENSMUSG00000058589	Anks1b	ankyrin repeat and sterile alpha motif domain cont	0.38262
ENSMUSG00000025261	Huwe1	HECT, UBA and WWE domain containing 1 [Source:	0.38265
ENSMUSG00000082529	Gm11199	predicted gene 11199 [Source:MGI Symbol;Acc:MG	0.38283

ENSMUSG00000032024	Clmp	CXADR-like membrane protein [Source:MGI Symbo	0.38285
ENSMUSG00000040195	Tmem194	transmembrane protein 194 [Source:MGI Symbol;A	0.38292
ENSMUSG00000048425	Olfr686	olfactory receptor 686 [Source:MGI Symbol;Acc:M(	0.38294
ENSMUSG00000021497	Txndc15	thioredoxin domain containing 15 [Source:MGI Sym	0.38296
ENSMUSG00000028622	Mrpl37	mitochondrial ribosomal protein L37 [Source:MGI S	0.38301
ENSMUSG00000001962	Fam50a	family with sequence similarity 50, member A [Sou	0.38308
ENSMUSG00000038248	Sobp	sine oculis-binding protein homolog (Drosophila) [S	0.38309
ENSMUSG00000025142	Aspscr1	alveolar soft part sarcoma chromosome region, car	0.38317
ENSMUSG00000038576	Susd4	sushi domain containing 4 [Source:MGI Symbol;Acc	0.38318
ENSMUSG00000041168	Lonp1	lon peptidase 1, mitochondrial [Source:MGI Symbo	0.3832
ENSMUSG00000097787	2700046G09Rik	RIKEN cDNA 2700046G09 gene [Source:MGI Symbo	0.38334
ENSMUSG00000025127	Gcgr	glucagon receptor [Source:MGI Symbol;Acc:MGI:99	0.38338
ENSMUSG00000042185	Nfrkb	nuclear factor related to kappa B binding protein [S	0.38338
ENSMUSG00000020286	1700093K21Rik	RIKEN cDNA 1700093K21 gene [Source:MGI Symbo	0.38349
ENSMUSG00000036781	Rps27l	ribosomal protein S27-like [Source:MGI Symbol;Acc	0.3837
ENSMUSG00000003541	Ier3	immediate early response 3 [Source:MGI Symbol;A	0.38373
ENSMUSG00000026831	1700007K13Rik	RIKEN cDNA 1700007K13 gene [Source:MGI Symbo	0.38383
ENSMUSG00000080937	Gm13510	predicted gene 13510 [Source:MGI Symbol;Acc:MG	0.38385
ENSMUSG00000020427	Igfbp3	insulin-like growth factor binding protein 3 [Source	0.38391
ENSMUSG00000047342	Zfp286	zinc finger protein 286 [Source:MGI Symbol;Acc:M(	0.3841
ENSMUSG00000045795	Whamm	WAS protein homolog associated with actin, golgi n	0.38414
ENSMUSG00000038910	Plcl2	phospholipase C-like 2 [Source:MGI Symbol;Acc:M(	0.38415
ENSMUSG00000065987	Cd209b	CD209b antigen [Source:MGI Symbol;Acc:MGI:1916	0.38418
ENSMUSG00000019689	1110001J03Rik	RIKEN cDNA 1110001J03 gene [Source:MGI Symbol	0.38426
ENSMUSG00000057691	Zfp746	zinc finger protein 746 [Source:MGI Symbol;Acc:M(	0.38436
ENSMUSG00000036672	Cenpt	centromere protein T [Source:MGI Symbol;Acc:MG	0.38444
ENSMUSG00000031216	Stard8	START domain containing 8 [Source:MGI Symbol;Ac	0.38449
ENSMUSG00000040502	9-Mar	membrane-associated ring finger (C3HC4) 9 [Source	0.38449
ENSMUSG00000079440	Alpi	alkaline phosphatase, intestinal [Source:MGI Symbo	0.38456
ENSMUSG00000097754	Gm26687	predicted gene, 26687 [Source:MGI Symbol;Acc:M(	0.38456
ENSMUSG00000052368	Gm9873	predicted gene 9873 [Source:MGI Symbol;Acc:MGI:	0.38458
ENSMUSG00000077004	Gm23876	predicted gene, 23876 [Source:MGI Symbol;Acc:M(	0.38458
ENSMUSG00000093087	Mir3473d	microRNA 3473d [Source:MGI Symbol;Acc:MGI:495	0.38458
ENSMUSG00000066537	Vmn2r57	vomeronasal 2, receptor 57 [Source:MGI Symbol;A	0.38459
ENSMUSG00000071735	Gm16462	predicted gene 16462 [Source:MGI Symbol;Acc:MG	0.3846
ENSMUSG00000078487	Ankrd65	ankyrin repeat domain 65 [Source:MGI Symbol;Acc	0.3846
ENSMUSG00000085368	Gm16188	predicted gene 16188 [Source:MGI Symbol;Acc:MG	0.3846
ENSMUSG00000093052	Gm24816	predicted gene, 24816 [Source:MGI Symbol;Acc:M(	0.3846
ENSMUSG00000027955	Fam198b	family with sequence similarity 198, member B [Sou	0.38461
ENSMUSG00000044453	Ffar1	free fatty acid receptor 1 [Source:MGI Symbol;Acc:	0.38461
ENSMUSG00000008035	Mid1ip1	Mid1 interacting protein 1 (gastrulation specific G1	0.38463
ENSMUSG00000042371	Slc5a10	solute carrier family 5 (sodium/glucose cotransport	0.38463
ENSMUSG00000086146	Gm15729	predicted gene 15729 [Source:MGI Symbol;Acc:MG	0.38463
ENSMUSG00000090129	Olfr287	olfactory receptor 287 [Source:MGI Symbol;Acc:M(	0.38463
ENSMUSG00000082262	Gm14730	predicted gene 14730 [Source:MGI Symbol;Acc:MG	0.38464
ENSMUSG00000078925	D030018L15Rik	RIKEN cDNA D030018L15 gene [Source:MGI Symbo	0.38467
ENSMUSG00000044689	Gm13749	predicted gene 13749 [Source:MGI Symbol;Acc:MG	0.3847

ENSMUSG00000022577	Ly6h	lymphocyte antigen 6 complex, locus H [Source:MG	0.38476
ENSMUSG00000097541	Gm26752	predicted gene, 26752 [Source:MGI Symbol;Acc:M(	0.38484
ENSMUSG00000008730	Hipk1	homeodomain interacting protein kinase 1 [Source	0.38485
ENSMUSG00000081372	Gm16171	predicted gene 16171 [Source:MGI Symbol;Acc:MG	0.38491
ENSMUSG00000083736	Gm6039	predicted gene 6039 [Source:MGI Symbol;Acc:MGI:	0.38495
ENSMUSG00000030978	Rrm1	ribonucleotide reductase M1 [Source:MGI Symbol;/	0.38504
ENSMUSG00000036104	Rab3gap1	RAB3 GTPase activating protein subunit 1 [Source:N	0.3851
ENSMUSG00000080866	Gm5687	predicted gene 5687 [Source:MGI Symbol;Acc:MGI:	0.38519
ENSMUSG00000072688	Gm10399	predicted gene 10399 [Source:MGI Symbol;Acc:MG	0.3852
ENSMUSG00000021559	Dapk1	death associated protein kinase 1 [Source:MGI Sym	0.38521
ENSMUSG00000046010	Zfp830	zinc finger protein 830 [Source:MGI Symbol;Acc:M(	0.38521
ENSMUSG00000087819	Gm25117	predicted gene, 25117 [Source:MGI Symbol;Acc:M(	0.38526
ENSMUSG00000033294	Noc4l	nucleolar complex associated 4 homolog (S. cerevis	0.38527
ENSMUSG00000042045	Sln	sarcolipin [Source:MGI Symbol;Acc:MGI:1913652]	0.38541
ENSMUSG00000021598	Med10	mediator of RNA polymerase II transcription, subur	0.38548
ENSMUSG00000045193	Cirbp	cold inducible RNA binding protein [Source:MGI Sy	0.38548
ENSMUSG00000074939	Chrm5	cholinergic receptor, muscarinic 5 [Source:MGI Sym	0.38554
ENSMUSG00000097408	Gm26831	predicted gene, 26831 [Source:MGI Symbol;Acc:M(	0.38557
ENSMUSG00000089447	Gm22532	predicted gene, 22532 [Source:MGI Symbol;Acc:M(	0.38561
ENSMUSG00000000682	Cd52	CD52 antigen [Source:MGI Symbol;Acc:MGI:134608	0.38564
ENSMUSG00000083922	Gm3835	predicted pseudogene 3835 [Source:MGI Symbol;A	0.38564
ENSMUSG00000074146	4930579C12Rik	RIKEN cDNA 4930579C12 gene [Source:MGI Symbo	0.38565
ENSMUSG00000052014	A330070K13Rik	RIKEN cDNA A330070K13 gene [Source:MGI Symbo	0.3857
ENSMUSG00000019890	Nts	neurotensin [Source:MGI Symbol;Acc:MGI:1328351	0.38571
ENSMUSG00000005892	Trh	thyrotropin releasing hormone [Source:MGI Symbo	0.38573
ENSMUSG00000041096	Tspyl2	TSPY-like 2 [Source:MGI Symbol;Acc:MGI:106244]	0.38576
ENSMUSG00000005232	G6pc2	glucose-6-phosphatase, catalytic, 2 [Source:MGI Sy	0.38577
ENSMUSG00000036180	Gatad2a	GATA zinc finger domain containing 2A [Source:MG	0.38577
ENSMUSG00000047238	Mageh1	melanoma antigen, family H, 1 [Source:MGI Symbo	0.38584
ENSMUSG00000086739	Gm4409	predicted gene 4409 [Source:MGI Symbol;Acc:MGI:	0.38584
ENSMUSG00000086711	Gm15482	predicted gene 15482 [Source:MGI Symbol;Acc:MG	0.38588
ENSMUSG00000022477	Aco2	aconitase 2, mitochondrial [Source:MGI Symbol;Ac	0.38591
ENSMUSG00000085106	Gm16000	predicted gene 16000 [Source:MGI Symbol;Acc:MG	0.38591
ENSMUSG00000092563	Gm3617	predicted gene 3617 [Source:MGI Symbol;Acc:MGI:	0.38592
ENSMUSG00000027690	Slc2a2	solute carrier family 2 (facilitated glucose transport	0.38593
ENSMUSG00000071229	Timm8a2	translocase of inner mitochondrial membrane 8A2	0.38594
ENSMUSG00000083485	Gm12308	predicted gene 12308 [Source:MGI Symbol;Acc:MG	0.38594
ENSMUSG00000081933	Gm14767	predicted gene 14767 [Source:MGI Symbol;Acc:MG	0.38598
ENSMUSG00000064232	Gm5414	predicted gene 5414 [Source:MGI Symbol;Acc:MGI:	0.38599
ENSMUSG00000039546	Ajap1	adherens junction associated protein 1 [Source:MG	0.38601
ENSMUSG00000030236	Slco1b2	solute carrier organic anion transporter family, mer	0.38602
ENSMUSG00000055150	Zfp78	zinc finger protein 78 [Source:MGI Symbol;Acc:MG	0.38602
ENSMUSG00000081376	Gm11694	predicted gene 11694 [Source:MGI Symbol;Acc:MG	0.38602
ENSMUSG00000044664	Prss42	protease, serine, 42 [Source:MGI Symbol;Acc:MGI:	0.38604
ENSMUSG00000049013	Prss48	protease, serine, 48 [Source:MGI Symbol;Acc:MGI:	0.38604
ENSMUSG00000027908	Tchhl1	trichohyalin-like 1 [Source:MGI Symbol;Acc:MGI:19	0.38605
ENSMUSG00000082978	Rpsa-ps11	ribosomal protein SA, pseudogene 11 [Source:MGI	0.38605

ENSMUSG00000076009	Mir688	microRNA 688 [Source:MGI Symbol;Acc:MGI:36299	0.38607
ENSMUSG00000065537	Mir132	microRNA 132 [Source:MGI Symbol;Acc:MGI:26768	0.38608
ENSMUSG00000083943	Gm11483	predicted gene 11483 [Source:MGI Symbol;Acc:MG	0.38608
ENSMUSG00000082254	Gm11922	predicted gene 11922 [Source:MGI Symbol;Acc:MG	0.3861
ENSMUSG00000083973	Gm13459	predicted gene 13459 [Source:MGI Symbol;Acc:MG	0.3861
ENSMUSG00000027551	Zfp64	zinc finger protein 64 [Source:MGI Symbol;Acc:MGI	0.38612
ENSMUSG00000036492	Rnf39	ring finger protein 39 [Source:MGI Symbol;Acc:MGI	0.38613
ENSMUSG00000085045	4921522H14Rik	RIKEN cDNA 4921522H14 gene [Source:MGI Symbo	0.38614
ENSMUSG00000086585	Gm16126	predicted gene 16126 [Source:MGI Symbol;Acc:MG	0.38614
ENSMUSG00000084201	Gm11926	predicted gene 11926 [Source:MGI Symbol;Acc:MG	0.38617
ENSMUSG00000029465	Arpc3	actin related protein 2/3 complex, subunit 3 [Sourc	0.38622
ENSMUSG00000091742	Gm5093	predicted gene 5093 [Source:MGI Symbol;Acc:MGI:	0.38622
ENSMUSG00000070498	Tmem132b	transmembrane protein 132B [Source:MGI Symbol;	0.38624
ENSMUSG00000045777	Ifitm10	interferon induced transmembrane protein 10 [Sou	0.38651
ENSMUSG00000047832	Cdca4	cell division cycle associated 4 [Source:MGI Symbol	0.38659
ENSMUSG00000044468	Fam46c	family with sequence similarity 46, member C [Sour	0.38661
ENSMUSG00000089820	Gm15775	predicted gene 15775 [Source:MGI Symbol;Acc:MG	0.3867
ENSMUSG00000062470	5730577I03Rik	RIKEN cDNA 5730577I03 gene [Source:MGI Symbol	0.38677
ENSMUSG00000036561	Ppp6r2	protein phosphatase 6, regulatory subunit 2 [Sourc	0.38678
ENSMUSG00000091785	Rpl29-ps5	ribosomal protein L29, pseudogene 5 [Source:MGI	0.38691
ENSMUSG00000032459	Mrps22	mitochondrial ribosomal protein S22 [Source:MGI S	0.38694
ENSMUSG00000087666	B230359F08Rik	RIKEN cDNA B230359F08 gene [Source:MGI Symbo	0.38696
ENSMUSG00000061979	Wbscr16	Williams-Beuren syndrome chromosome region 16	0.38697
ENSMUSG00000091078	Gm17218	predicted gene 17218 [Source:MGI Symbol;Acc:MG	0.38697
ENSMUSG00000085230	Gm16013	predicted gene 16013 [Source:MGI Symbol;Acc:MG	0.38699
ENSMUSG00000084688	n-R5s43	nuclear encoded rRNA 5S 43 [Source:MGI Symbol;A	0.38704
ENSMUSG00000039110	Mycbpap	MYCBP associated protein [Source:MGI Symbol;Acc	0.38706
ENSMUSG00000006538	Ihh	Indian hedgehog [Source:MGI Symbol;Acc:MGI:965	0.38707
ENSMUSG00000087513	Gm13236	predicted gene 13236 [Source:MGI Symbol;Acc:MG	0.38707
ENSMUSG00000074653	Lrrc31	leucine rich repeat containing 31 [Source:MGI Syml	0.38708
ENSMUSG00000082535	Gm7860	predicted gene 7860 [Source:MGI Symbol;Acc:MGI:	0.38708
ENSMUSG00000028081	Rps3a1	ribosomal protein S3A1 [Source:MGI Symbol;Acc:M	0.38712
ENSMUSG00000057223	Gm6578	predicted gene 6578 [Source:MGI Symbol;Acc:MGI:	0.38713
ENSMUSG00000043385	Olfr267	olfactory receptor 267 [Source:MGI Symbol;Acc:MG	0.38714
ENSMUSG00000005338	Cadm3	cell adhesion molecule 3 [Source:MGI Symbol;Acc:M	0.38715
ENSMUSG00000029054	Gabrd	gamma-aminobutyric acid (GABA) A receptor, subu	0.38718
ENSMUSG00000025839	Pramel7	preferentially expressed antigen in melanoma like 7	0.3872
ENSMUSG00000020883	Fbxl20	F-box and leucine-rich repeat protein 20 [Source:M	0.38721
ENSMUSG00000087292	Gm13832	predicted gene 13832 [Source:MGI Symbol;Acc:MG	0.38721
ENSMUSG00000074785	Plxnc1	plexin C1 [Source:MGI Symbol;Acc:MGI:1890127]	0.38729
ENSMUSG00000026664	Phyh	phytanoyl-CoA hydroxylase [Source:MGI Symbol;Ac	0.3873
ENSMUSG00000086749	Gm12037	predicted gene 12037 [Source:MGI Symbol;Acc:MG	0.38731
ENSMUSG00000031775	Pllp	plasma membrane proteolipid [Source:MGI Symbo	0.38745
ENSMUSG00000082469	Gm13079	predicted gene 13079 [Source:MGI Symbol;Acc:MG	0.38767
ENSMUSG00000097607	AC245272.1		0.38769
ENSMUSG00000026811	St6galnac6	ST6 (alpha-N-acetyl-neuraminyl-2,3-beta-galactosyl	0.3877
ENSMUSG00000038205	Prkab2	protein kinase, AMP-activated, beta 2 non-catalytic	0.3877

ENSMUSG00000074704	Rad21l	RAD21-like (S. pombe) [Source:MGI Symbol;Acc:MG	0.38771
ENSMUSG00000082517	Gm5757	predicted gene 5757 [Source:MGI Symbol;Acc:MGI:	0.38771
ENSMUSG00000074564	Gm10720	predicted gene 10720 [Source:MGI Symbol;Acc:MG	0.38773
ENSMUSG00000054905	Stfa3	stefin A3 [Source:MGI Symbol;Acc:MGI:106196]	0.38776
ENSMUSG00000056600	Olfr90	olfactory receptor 90 [Source:MGI Symbol;Acc:MGI	0.38776
ENSMUSG00000046652	Tas2r143	taste receptor, type 2, member 143 [Source:MGI Sy	0.38778
ENSMUSG00000022269	11-Mar	membrane-associated ring finger (C3HC4) 11 [Sour	0.3878
ENSMUSG00000046782	Ttc6	tetratricopeptide repeat domain 6 [Source:MGI Syr	0.38786
ENSMUSG00000043549	4932442L08Rik	RIKEN cDNA 4932442L08 gene [Source:MGI Symbo	0.38787
ENSMUSG00000082855	Gm14537	predicted gene 14537 [Source:MGI Symbol;Acc:MG	0.38793
ENSMUSG00000087201	Gm15261	predicted gene 15261 [Source:MGI Symbol;Acc:MG	0.38793
ENSMUSG00000022982	Sod1	superoxide dismutase 1, soluble [Source:MGI Symb	0.38795
ENSMUSG00000028683	Eif2b3	eukaryotic translation initiation factor 2B, subunit 3	0.38797
ENSMUSG00000082512	Gm13937	predicted gene 13937 [Source:MGI Symbol;Acc:MG	0.38798
ENSMUSG00000081496	Gm13428	predicted gene 13428 [Source:MGI Symbol;Acc:MG	0.38801
ENSMUSG00000052099	Prss51	protease, serine, 51 [Source:MGI Symbol;Acc:MGI:1	0.38802
ENSMUSG00000065701	Rny1	RNA, Y1 small cytoplasmic, Ro-associated [Source:M	0.38805
ENSMUSG00000077476	Gm24794	predicted gene, 24794 [Source:MGI Symbol;Acc:MG	0.38805
ENSMUSG00000030168	Adipor2	adiponectin receptor 2 [Source:MGI Symbol;Acc:MG	0.38807
ENSMUSG00000097229	Gm26598	predicted gene, 26598 [Source:MGI Symbol;Acc:MG	0.38809
ENSMUSG00000097642	Gm26866	predicted gene, 26866 [Source:MGI Symbol;Acc:MG	0.38809
ENSMUSG00000073236	2500004C02Rik	RIKEN cDNA 2500004C02 gene [Source:MGI Symbo	0.3881
ENSMUSG00000089995	Gm15716	predicted gene 15716 [Source:MGI Symbol;Acc:MG	0.3881
ENSMUSG00000097681	Gm26643	predicted gene, 26643 [Source:MGI Symbol;Acc:MG	0.38812
ENSMUSG00000020902	Ntn1	netrin 1 [Source:MGI Symbol;Acc:MGI:105088]	0.38822
ENSMUSG00000026126	Ptpn18	protein tyrosine phosphatase, non-receptor type 18	0.38822
ENSMUSG00000003657	Calb2	calbindin 2 [Source:MGI Symbol;Acc:MGI:101914]	0.38826
ENSMUSG00000077839	Gm26053	predicted gene, 26053 [Source:MGI Symbol;Acc:MG	0.38831
ENSMUSG00000026271	Gpr35	G protein-coupled receptor 35 [Source:MGI Symbo	0.38832
ENSMUSG00000028018	Gstcd	glutathione S-transferase, C-terminal domain conta	0.38834
ENSMUSG00000030352	Tspan9	tetraspanin 9 [Source:MGI Symbol;Acc:MGI:192455	0.38853
ENSMUSG00000057604	Lmcd1	LIM and cysteine-rich domains 1 [Source:MGI Symb	0.38855
ENSMUSG00000025873	Faf2	Fas associated factor family member 2 [Source:MG	0.38856
ENSMUSG00000012117	Dhdds	dehydrodolichyl diphosphate synthase [Source:MG	0.38862
ENSMUSG00000092323	BB365896	expressed sequence BB365896 [Source:MGI Symbo	0.38876
ENSMUSG00000041037	Irgq	immunity-related GTPase family, Q [Source:MGI Sy	0.38892
ENSMUSG00000032531	Amotl2	angiomotin-like 2 [Source:MGI Symbol;Acc:MGI:19	0.38903
ENSMUSG00000069844	Sco1	SCO cytochrome oxidase deficient homolog 1 (yeas	0.38904
ENSMUSG00000078813	Leng1	leukocyte receptor cluster (LRC) member 1 [Source	0.38909
ENSMUSG00000060260	Pwwp2b	PWWP domain containing 2B [Source:MGI Symbol;	0.38912
ENSMUSG00000097601	Gm26660	predicted gene, 26660 [Source:MGI Symbol;Acc:MG	0.38921
ENSMUSG00000032077	Bud13	BUD13 homolog (yeast) [Source:MGI Symbol;Acc:M	0.38923
ENSMUSG00000087260	Lamtor5	late endosomal/lysosomal adaptor, MAPK and MTO	0.38923
ENSMUSG00000030402	Ppm1n	protein phosphatase, Mg2+/Mn2+ dependent, 1N (	0.38925
ENSMUSG00000028024	Enpep	glutamyl aminopeptidase [Source:MGI Symbol;Acc:	0.38929
ENSMUSG00000043015	Tmem194b	transmembrane protein 194B [Source:MGI Symbol;	0.38931
ENSMUSG00000041598	Cdc42ep4	CDC42 effector protein (Rho GTPase binding) 4 [Sou	0.38935

ENSMUSG00000097685	Gm26646	predicted gene, 26646 [Source:MGI Symbol;Acc:MG	0.3894
ENSMUSG00000020536	Llgl1	lethal giant larvae homolog 1 (Drosophila) [Source:	0.38944
ENSMUSG00000059714	Flot1	flotillin 1 [Source:MGI Symbol;Acc:MGI:1100500]	0.38951
ENSMUSG00000086043	Gm12473	predicted gene 12473 [Source:MGI Symbol;Acc:MG	0.38956
ENSMUSG00000054836	Elp6	elongator acetyltransferase complex subunit 6 [Sou	0.38959
ENSMUSG00000040312	Cchcr1	coiled-coil alpha-helical rod protein 1 [Source:MGI	0.3897
ENSMUSG00000061731	Ext1	exostoses (multiple) 1 [Source:MGI Symbol;Acc:MG	0.38978
ENSMUSG00000044791	Setd2	SET domain containing 2 [Source:MGI Symbol;Acc:M	0.38992
ENSMUSG00000040003	Magi2	membrane associated guanylate kinase, WW and P	0.38996
ENSMUSG00000055872	Gm9985	predicted gene 9985 [Source:MGI Symbol;Acc:MGI:	0.39006
ENSMUSG00000032295	Man2c1	mannosidase, alpha, class 2C, member 1 [Source:M	0.39007
ENSMUSG00000096995	2810029C07Rik	RIKEN cDNA 2810029C07 gene [Source:MGI Symbo	0.39016
ENSMUSG00000030946	Lhpp	phospholysine phosphohistidine inorganic pyropho	0.39017
ENSMUSG00000079215	Zfp664	zinc finger protein 664 [Source:MGI Symbol;Acc:MG	0.39019
ENSMUSG00000006342	Susd2	sushi domain containing 2 [Source:MGI Symbol;Acc	0.39026
ENSMUSG00000035580	Kcnh8	potassium voltage-gated channel, subfamily H (eag	0.39031
ENSMUSG00000093758	Gm20701	predicted gene 20701 [Source:MGI Symbol;Acc:MG	0.39051
ENSMUSG00000036885	Arhgef26	Rho guanine nucleotide exchange factor (GEF) 26 [S	0.39065
ENSMUSG00000004655	Aqp1	aquaporin 1 [Source:MGI Symbol;Acc:MGI:103201]	0.39074
ENSMUSG00000028152	Tspan5	tetraspanin 5 [Source:MGI Symbol;Acc:MGI:192809	0.39077
ENSMUSG00000046897	Zfp740	zinc finger protein 740 [Source:MGI Symbol;Acc:MG	0.39087
ENSMUSG00000024176	Sox8	SRY-box containing gene 8 [Source:MGI Symbol;Acc	0.39091
ENSMUSG00000061981	Flot2	flotillin 2 [Source:MGI Symbol;Acc:MGI:103309]	0.39091
ENSMUSG00000046109	AB041806	hypothetical protein, MNCb-2457 [Source:MGI Sym	0.39112
ENSMUSG00000046387	Pcdhb17	protocadherin beta 17 [Source:MGI Symbol;Acc:MG	0.39121
ENSMUSG00000025133	Ints4	integrator complex subunit 4 [Source:MGI Symbol;A	0.39127
ENSMUSG00000026142	Rhbdd1	rhomboid domain containing 1 [Source:MGI Symbo	0.39131
ENSMUSG00000081404	Gm15694	predicted gene 15694 [Source:MGI Symbol;Acc:MG	0.39133
ENSMUSG00000020178	Adora2a	adenosine A2a receptor [Source:MGI Symbol;Acc:M	0.39134
ENSMUSG00000036959	Bcorl1	BCL6 co-repressor-like 1 [Source:MGI Symbol;Acc:M	0.39152
ENSMUSG00000048865	Arhgap30	Rho GTPase activating protein 30 [Source:MGI Sym	0.39161
ENSMUSG00000026180	Cxcr2	chemokine (C-X-C motif) receptor 2 [Source:MGI Sy	0.39162
ENSMUSG00000097566	A930024N18Rik	RIKEN cDNA A930024N18 gene [Source:MGI Symbo	0.39167
ENSMUSG00000028868	Wasf2	WAS protein family, member 2 [Source:MGI Symbo	0.39174
ENSMUSG00000038146	Notch3	notch 3 [Source:MGI Symbol;Acc:MGI:99460]	0.39179
ENSMUSG00000029456	Acad10	acyl-Coenzyme A dehydrogenase family, member 1	0.39184
ENSMUSG00000020483	Dynll2	dynein light chain LC8-type 2 [Source:MGI Symbol;A	0.39191
ENSMUSG00000023764	Sfi1	Sfi1 homolog, spindle assembly associated (yeast) [	0.39196
ENSMUSG00000055538	Zcchc24	zinc finger, CCHC domain containing 24 [Source:MG	0.39196
ENSMUSG00000021595	Nsun2	NOL1/NOP2/Sun domain family member 2 [Source:	0.39222
ENSMUSG00000073139	BC023829	cDNA sequence BC023829 [Source:MGI Symbol;Acc	0.39229
ENSMUSG00000034748	Sirt6	sirtuin 6 [Source:MGI Symbol;Acc:MGI:1354161]	0.3923
ENSMUSG00000041852	Tcf20	transcription factor 20 [Source:MGI Symbol;Acc:MG	0.39233
ENSMUSG00000039756	Dnttip2	deoxynucleotidyltransferase, terminal, interacting p	0.39239
ENSMUSG00000097042	Gm17491	predicted gene, 17491 [Source:MGI Symbol;Acc:MG	0.39242
ENSMUSG00000044244	Il20rb	interleukin 20 receptor beta [Source:MGI Symbol;A	0.3925
ENSMUSG00000025217	Btrc	beta-transducin repeat containing protein [Source:	0.39256

ENSMUSG00000039316	Rftn1	raftlin lipid raft linker 1 [Source:MGI Symbol;Acc:M	0.39264
ENSMUSG00000038763	Alpk3	alpha-kinase 3 [Source:MGI Symbol;Acc:MGI:21512	0.39265
ENSMUSG00000035179	Ppp1r32	protein phosphatase 1, regulatory subunit 32 [Sour	0.39269
ENSMUSG00000087410	2310065F04Rik	RIKEN cDNA 2310065F04 gene [Source:MGI Symbo	0.3928
ENSMUSG00000049960	Mrps16	mitochondrial ribosomal protein S16 [Source:MGI S	0.39282
ENSMUSG00000061576	Dpp6	dipeptidylpeptidase 6 [Source:MGI Symbol;Acc:MG	0.39293
ENSMUSG00000030170	Wnt5b	wingless-related MMTV integration site 5B [Source	0.39295
ENSMUSG00000021495	Fam193b	family with sequence similarity 193, member B [Sou	0.39297
ENSMUSG00000029913	Prdm5	PR domain containing 5 [Source:MGI Symbol;Acc:M	0.39298
ENSMUSG00000023495	Pcbp4	poly(rC) binding protein 4 [Source:MGI Symbol;Acc	0.39302
ENSMUSG00000051295	9630028B13Rik	RIKEN cDNA 9630028B13 gene [Source:MGI Symbo	0.39307
ENSMUSG00000026958	Dpp7	dipeptidylpeptidase 7 [Source:MGI Symbol;Acc:MG	0.39317
ENSMUSG00000029161	Cgref1	cell growth regulator with EF hand domain 1 [Sourc	0.39323
ENSMUSG00000020381	3010026O09Rik	RIKEN cDNA 3010026O09 gene [Source:MGI Symbo	0.39324
ENSMUSG00000038594	Cep85l	centrosomal protein 85-like [Source:MGI Symbol;A	0.39324
ENSMUSG00000049555	Tmie	transmembrane inner ear [Source:MGI Symbol;Acc	0.39328
ENSMUSG00000033458	Fan1	FANCD2/FANCI-associated nuclease 1 [Source:MGI	0.39331
ENSMUSG00000035011	Zbtb7a	zinc finger and BTB domain containing 7a [Source:N	0.39333
ENSMUSG00000071104	Ccdc110	coiled-coil domain containing 110 [Source:MGI Sym	0.39336
ENSMUSG00000001052	Sec24b	Sec24 related gene family, member B (S. cerevisiae	0.39337
ENSMUSG00000041992	Rapgef5	Rap guanine nucleotide exchange factor (GEF) 5 [So	0.39352
ENSMUSG00000033400	Agl	amylo-1,6-glucosidase, 4-alpha-glucanotransferase	0.3936
ENSMUSG00000027998	Plrg1	pleiotropic regulator 1, PRL1 homolog (Arabidopsis	0.39364
ENSMUSG00000084897	Gm14226	predicted gene 14226 [Source:MGI Symbol;Acc:MG	0.39365
ENSMUSG00000041147	Brca2	breast cancer 2 [Source:MGI Symbol;Acc:MGI:1093	0.39368
ENSMUSG00000087341	0610040F04Rik	RIKEN cDNA 0610040F04 gene [Source:MGI Symbo	0.39371
ENSMUSG00000024871	Doc2g	double C2, gamma [Source:MGI Symbol;Acc:MGI:1	0.39373
ENSMUSG00000026611	Spata17	spermatogenesis associated 17 [Source:MGI Symbo	0.39374
ENSMUSG00000070421	Olfr658	olfactory receptor 658 [Source:MGI Symbol;Acc:MG	0.39374
ENSMUSG00000042632	Pla2g6	phospholipase A2, group VI [Source:MGI Symbol;Ac	0.39391
ENSMUSG00000018774	Cd68	CD68 antigen [Source:MGI Symbol;Acc:MGI:88342]	0.39393
ENSMUSG00000091095	Gm17305	predicted gene, 17305 [Source:MGI Symbol;Acc:MG	0.39403
ENSMUSG00000097858	9530052C20Rik	RIKEN cDNA 9530052C20 gene [Source:MGI Symbo	0.39411
ENSMUSG00000021177	Tdp1	tyrosyl-DNA phosphodiesterase 1 [Source:MGI Sym	0.39415
ENSMUSG00000031796	Gtl3	gene trap locus 3 [Source:MGI Symbol;Acc:MGI:107	0.39415
ENSMUSG00000030109	Slc6a12	solute carrier family 6 (neurotransmitter transporte	0.39421
ENSMUSG00000039578	Ccser1	coiled-coil serine rich 1 [Source:MGI Symbol;Acc:M	0.39424
ENSMUSG00000051232	Tmem199	transmembrane protein 199 [Source:MGI Symbol;A	0.39436
ENSMUSG00000047921	Trappc9	trafficking protein particle complex 9 [Source:MGI S	0.39439
ENSMUSG00000066554	Gm10167	predicted pseudogene 10167 [Source:MGI Symbol;	0.39439
ENSMUSG00000040731	Eif4h	eukaryotic translation initiation factor 4H [Source:N	0.39444
ENSMUSG00000044167	Foxo1	forkhead box O1 [Source:MGI Symbol;Acc:MGI:189	0.39456
ENSMUSG00000019853	Hebp2	heme binding protein 2 [Source:MGI Symbol;Acc:M	0.3946
ENSMUSG00000027420	Bfsp1	beaded filament structural protein 1, in lens-CP94 [	0.39464
ENSMUSG00000054383	Pnma1	paraneoplastic antigen MA1 [Source:MGI Symbol;A	0.39473
ENSMUSG00000081892	Gm8864	predicted gene 8864 [Source:MGI Symbol;Acc:MGI:	0.39484
ENSMUSG00000045994	B3gat1	beta-1,3-glucuronyltransferase 1 (glucuronosyltran	0.39496

ENSMUSG00000038642	Ctss	cathepsin S [Source:MGI Symbol;Acc:MGI:107341]	0.39505
ENSMUSG00000080533	Mir1191	microRNA 1191 [Source:MGI Symbol;Acc:MGI:3783	0.39513
ENSMUSG00000021136	Smoc1	SPARC related modular calcium binding 1 [Source:I	0.39516
ENSMUSG00000065637	Gm26397	predicted gene, 26397 [Source:MGI Symbol;Acc:MG	0.39516
ENSMUSG00000097023	AI854517	expressed sequence AI854517 [Source:MGI Symbol	0.39538
ENSMUSG00000071647	Eml3	echinoderm microtubule associated protein like 3 [	0.39542
ENSMUSG00000031250	Tnmd	tenomodulin [Source:MGI Symbol;Acc:MGI:192988	0.39549
ENSMUSG00000082210	Gm14382	predicted gene 14382 [Source:MGI Symbol;Acc:MG	0.39554
ENSMUSG00000034066	Farp2	FERM, RhoGEF and pleckstrin domain protein 2 [So	0.3956
ENSMUSG00000025537	Phkg1	phosphorylase kinase gamma 1 [Source:MGI Symbo	0.39563
ENSMUSG00000027460	Angpt4	angiopoietin 4 [Source:MGI Symbol;Acc:MGI:13368	0.39575
ENSMUSG00000028717	Tal1	T cell acute lymphocytic leukemia 1 [Source:MGI Sy	0.39577
ENSMUSG00000023336	Wfdc1	WAP four-disulfide core domain 1 [Source:MGI Sym	0.39584
ENSMUSG00000044950	Pwwp2a	PWWP domain containing 2A [Source:MGI Symbol;	0.39605
ENSMUSG00000079495	Gm11128	predicted gene 11128 [Source:MGI Symbol;Acc:MG	0.39626
ENSMUSG00000026347	Tmem163	transmembrane protein 163 [Source:MGI Symbol;A	0.39628
ENSMUSG00000030515	Tarsl2	threonyl-tRNA synthetase-like 2 [Source:MGI Symb	0.39631
ENSMUSG00000085732	Gm4279	predicted gene 4279 [Source:MGI Symbol;Acc:MGI:	0.39633
ENSMUSG00000038801	Scgb1c1	secretoglobin, family 1C, member 1 [Source:MGI Sy	0.39641
ENSMUSG00000018217	Pmp22	peripheral myelin protein 22 [Source:MGI Symbol;A	0.39651
ENSMUSG00000005803	Sqrdl	sulfide quinone reductase-like (yeast) [Source:MGI	0.39659
ENSMUSG00000033983	Coil	coilin [Source:MGI Symbol;Acc:MGI:104842]	0.39662
ENSMUSG00000067722	BC003965	cDNA sequence BC003965 [Source:MGI Symbol;Acc	0.39673
ENSMUSG00000034265	Zdhhc14	zinc finger, DHHC domain containing 14 [Source:MG	0.39679
ENSMUSG00000085689	Gm16838	predicted gene, 16838 [Source:MGI Symbol;Acc:MG	0.39683
ENSMUSG00000041189	Chrnb1	cholinergic receptor, nicotinic, beta polypeptide 1 (	0.39686
ENSMUSG00000026475	Rgs16	regulator of G-protein signaling 16 [Source:MGI Syr	0.39702
ENSMUSG00000059229	Gm6802	predicted gene 6802 [Source:MGI Symbol;Acc:MGI:	0.39714
ENSMUSG00000072640	Lyrm9	LYR motif containing 9 [Source:MGI Symbol;Acc:MG	0.39718
ENSMUSG00000022414	Tab1	TGF-beta activated kinase 1/MAP3K7 binding prote	0.39722
ENSMUSG00000030917	Tmem159	transmembrane protein 159 [Source:MGI Symbol;A	0.39737
ENSMUSG00000070471	Fam194a	family with sequence similarity 194, member A [Sou	0.39738
ENSMUSG00000024764	Naa40	N(alpha)-acetyltransferase 40, NatD catalytic subur	0.39756
ENSMUSG00000028031	Dkk2	dickkopf homolog 2 (Xenopus laevis) [Source:MGI S	0.39756
ENSMUSG00000086155	9430041J12Rik	RIKEN cDNA 9430041J12 gene [Source:MGI Symbol	0.39759
ENSMUSG00000049985	Ankrd55	ankyrin repeat domain 55 [Source:MGI Symbol;Acc	0.3976
ENSMUSG00000024990	Rbp4	retinol binding protein 4, plasma [Source:MGI Symb	0.39764
ENSMUSG00000059534	Uqcr10	ubiquinol-cytochrome c reductase, complex III subu	0.39766
ENSMUSG00000046295	Ankle1	ankyrin repeat and LEM domain containing 1 [Sourc	0.39771
ENSMUSG00000044350	Lacc1	laccase (multicopper oxidoreductase) domain conta	0.39778
ENSMUSG00000012428	Steap4	STEAP family member 4 [Source:MGI Symbol;Acc:M	0.39786
ENSMUSG00000041624	Gucy1a2	guanylate cyclase 1, soluble, alpha 2 [Source:MGI S	0.39792
ENSMUSG00000037032	Apbb1	amyloid beta (A4) precursor protein-binding, family	0.39811
ENSMUSG00000062963	Ufc1	ubiquitin-fold modifier conjugating enzyme 1 [Sour	0.39811
ENSMUSG00000028753	Vwa5b1	von Willebrand factor A domain containing 5B1 [So	0.3982
ENSMUSG00000039153	Runx2	runt related transcription factor 2 [Source:MGI Sym	0.39823
ENSMUSG00000090666	Gm17567	predicted gene, 17567 [Source:MGI Symbol;Acc:MG	0.39836

ENSMUSG00000090697	Nsun4	NOL1/NOP2/Sun domain family, member 4 [Source	0.39842
ENSMUSG00000049624	Slc17a5	solute carrier family 17 (anion/sugar transporter), r	0.39845
ENSMUSG00000026810	Dpm2	dolichol-phosphate (beta-D) mannosyltransferase 2	0.3985
ENSMUSG00000035576	L3mbtl1	l(3)mbt-like (Drosophila) [Source:MGI Symbol;Acc:M	0.39851
ENSMUSG00000043467	Zbtb37	zinc finger and BTB domain containing 37 [Source:M	0.39855
ENSMUSG00000003665	Has1	hyaluronan synthase1 [Source:MGI Symbol;Acc:MG	0.39857
ENSMUSG00000067279	Ppp1r3c	protein phosphatase 1, regulatory (inhibitor) subun	0.39867
ENSMUSG00000097535	Gm26592	predicted gene, 26592 [Source:MGI Symbol;Acc:MG	0.39867
ENSMUSG00000001100	Poldip2	polymerase (DNA-directed), delta interacting prote	0.39872
ENSMUSG00000044145	1810024B03Rik	RIKEN cDNA 1810024B03 gene [Source:MGI Symbo	0.39873
ENSMUSG00000081004	Gm9157	predicted gene 9157 [Source:MGI Symbol;Acc:MGI:	0.39878
ENSMUSG00000050050	Ccdc158	coiled-coil domain containing 158 [Source:MGI Sym	0.39887
ENSMUSG00000024678	Ms4a4d	membrane-spanning 4-domains, subfamily A, mem	0.39897
ENSMUSG00000057147	Dph6	diphthamine biosynthesis 6 [Source:MGI Symbol;Ac	0.39901
ENSMUSG00000040412	5330417C22Rik	RIKEN cDNA 5330417C22 gene [Source:MGI Symbo	0.39905
ENSMUSG00000039536	Stau1	staufen (RNA binding protein) homolog 1 (Drosoph	0.39906
ENSMUSG00000021486	Prelid1	PRELI domain containing 1 [Source:MGI Symbol;Acc	0.39908
ENSMUSG00000028463	Car9	carbonic anhydrase 9 [Source:MGI Symbol;Acc:MG	0.39908
ENSMUSG00000040174	Alkbh3	alkB, alkylation repair homolog 3 (E. coli) [Source:M	0.39916
ENSMUSG00000019312	Grb7	growth factor receptor bound protein 7 [Source:MG	0.39918
ENSMUSG00000087322	Gm16075	predicted gene 16075 [Source:MGI Symbol;Acc:MG	0.39918
ENSMUSG00000023393	Slc17a9	solute carrier family 17, member 9 [Source:MGI Syr	0.39919
ENSMUSG00000029922	Mkrn1	makorin, ring finger protein, 1 [Source:MGI Symbol	0.39921
ENSMUSG00000015027	Galns	galactosamine (N-acetyl)-6-sulfate sulfatase [Sourc	0.39924
ENSMUSG00000048330	Ric3	resistance to inhibitors of cholinesterase 3 homolog	0.39924
ENSMUSG00000036196	Slc26a8	solute carrier family 26, member 8 [Source:MGI Syr	0.39925
ENSMUSG00000090173	Fbxw10	F-box and WD-40 domain protein 10 [Source:MGI S	0.39928
ENSMUSG00000052593	Adam17	a disintegrin and metallopeptidase domain 17 [Sou	0.39939
ENSMUSG00000022440	C1qtnf6	C1q and tumor necrosis factor related protein 6 [So	0.39952
ENSMUSG00000025487	Psmd13	proteasome (prosome, macropain) 26S subunit, no	0.39952
ENSMUSG00000024369	Nelfe	negative elongation factor complex member E, Rdb	0.39958
ENSMUSG00000074345	Tnfaip8l3	tumor necrosis factor, alpha-induced protein 8-like	0.39961
ENSMUSG00000020798	Spns3	spinster homolog 3 [Source:MGI Symbol;Acc:MGI:1	0.39973
ENSMUSG00000038695	Josd2	Josephin domain containing 2 [Source:MGI Symbol;	0.39993
ENSMUSG00000045053	Kcng3	potassium voltage-gated channel, subfamily G, mer	0.39995
ENSMUSG00000002379	Ndufa11	NADH dehydrogenase (ubiquinone) 1 alpha subcom	0.39996
ENSMUSG00000087668	Gm11186	predicted gene 11186 [Source:MGI Symbol;Acc:MG	0.39996
ENSMUSG00000082996	Gm6238	predicted pseudogene 6238 [Source:MGI Symbol;A	0.39998
ENSMUSG00000058357	Gm6612	predicted gene 6612 [Source:MGI Symbol;Acc:MGI:	0.40002
ENSMUSG00000046367	4931440L10Rik	RIKEN cDNA 4931440L10 gene [Source:MGI Symbo	0.40003
ENSMUSG00000055670	Zzef1	zinc finger, ZZ-type with EF hand domain 1 [Source:	0.40007
ENSMUSG00000079871	Gm4984	predicted pseudogene 4984 [Source:MGI Symbol;A	0.40017
ENSMUSG00000044566	Cage1	cancer antigen 1 [Source:MGI Symbol;Acc:MGI:191	0.40023
ENSMUSG00000042213	Zfand4	zinc finger, AN1-type domain 4 [Source:MGI Symbo	0.40031
ENSMUSG00000026933	Camsap1	calmodulin regulated spectrin-associated protein 1	0.40041
ENSMUSG00000027297	Ltk	leukocyte tyrosine kinase [Source:MGI Symbol;Acc:	0.40049
ENSMUSG00000002257	Def6	differentially expressed in FDCP 6 [Source:MGI Sym	0.40056

ENSMUSG00000018451	6330403K07Rik	RIKEN cDNA 6330403K07 gene [Source:MGI Symbo	0.40065
ENSMUSG00000064225	Paqr9	progestin and adipoQ receptor family member IX [S	0.40076
ENSMUSG00000090625	Gm20721	predicted gene, 20721 [Source:MGI Symbol;Acc:MG	0.40079
ENSMUSG00000035983	Gm7008	predicted gene 7008 [Source:MGI Symbol;Acc:MGI:	0.4008
ENSMUSG00000029009	Mthfr	5,10-methylenetetrahydrofolate reductase [Source	0.40098
ENSMUSG00000042873	Lhfpl4	lipoma HMGIC fusion partner-like protein 4 [Source	0.40102
ENSMUSG00000081851	Gm13323	predicted gene 13323 [Source:MGI Symbol;Acc:MG	0.40106
ENSMUSG00000074738	B930041F14Rik	RIKEN cDNA B930041F14 gene [Source:MGI Symbo	0.40113
ENSMUSG00000064778	Gm25206	predicted gene, 25206 [Source:MGI Symbol;Acc:MG	0.40115
ENSMUSG00000032036	Kirrel3	kin of IRRE like 3 (Drosophila) [Source:MGI Symbol;	0.40116
ENSMUSG00000014763	Fam120b	family with sequence similarity 120, member B [Sou	0.4012
ENSMUSG00000030427	Lilra6	leukocyte immunoglobulin-like receptor, subfamily	0.40126
ENSMUSG00000070331	Qrich2	glutamine rich 2 [Source:MGI Symbol;Acc:MGI:268	0.40126
ENSMUSG00000081069	Gm13928	predicted gene 13928 [Source:MGI Symbol;Acc:MG	0.40127
ENSMUSG00000019970	Sgk1	serum/glucocorticoid regulated kinase 1 [Source:M	0.40132
ENSMUSG00000030324	Rho	rhodopsin [Source:MGI Symbol;Acc:MGI:97914]	0.40134
ENSMUSG00000081822	Gm15626	predicted gene 15626 [Source:MGI Symbol;Acc:MG	0.40155
ENSMUSG00000020790	Ankfy1	ankyrin repeat and FYVE domain containing 1 [Sour	0.40167
ENSMUSG00000009214	Tmem8c	transmembrane protein 8C [Source:MGI Symbol;Ac	0.40173
ENSMUSG00000051817	Sox12	SRY-box containing gene 12 [Source:MGI Symbol;Ac	0.40184
ENSMUSG00000029207	Apbb2	amyloid beta (A4) precursor protein-binding, family	0.40187
ENSMUSG00000022579	Gpihbp1	GPI-anchored HDL-binding protein 1 [Source:MGI S	0.40188
ENSMUSG00000052142	Rasal3	RAS protein activator like 3 [Source:MGI Symbol;Ac	0.40198
ENSMUSG00000040269	Mrps28	mitochondrial ribosomal protein S28 [Source:MGI S	0.40199
ENSMUSG00000048175	Asb8	ankyrin repeat and SOCS box-containing 8 [Source:	0.40201
ENSMUSG00000028838	Extl1	exostoses (multiple)-like 1 [Source:MGI Symbol;Acc	0.40222
ENSMUSG00000094086	Gm23992	predicted gene, 23992 [Source:MGI Symbol;Acc:MG	0.40224
ENSMUSG00000030830	Itgal	integrin alpha L [Source:MGI Symbol;Acc:MGI:9660	0.40233
ENSMUSG00000042167	Papd4	PAP associated domain containing 4 [Source:MGI Sy	0.40234
ENSMUSG00000086997	Gm12981	predicted gene 12981 [Source:MGI Symbol;Acc:MG	0.40259
ENSMUSG00000034932	Mrpl54	mitochondrial ribosomal protein L54 [Source:MGI S	0.4026
ENSMUSG00000036376	Abt1	activator of basal transcription 1 [Source:MGI Symb	0.40268
ENSMUSG00000085715	Tsix	X (inactive)-specific transcript, opposite strand [Sou	0.40284
ENSMUSG00000030259	Rassf8	Ras association (RalGDS/AF-6) domain family (N-ter	0.40285
ENSMUSG00000029084	Cd38	CD38 antigen [Source:MGI Symbol;Acc:MGI:107474	0.40292
ENSMUSG00000033948	Zswim5	zinc finger SWIM-type containing 5 [Source:MGI Sy	0.40298
ENSMUSG00000021913	Ogdhl	oxoglutarate dehydrogenase-like [Source:MGI Sym	0.40306
ENSMUSG00000029638	Glcci1	glucocorticoid induced transcript 1 [Source:MGI Sy	0.40306
ENSMUSG00000037343	Taf2	TAF2 RNA polymerase II, TATA box binding protein	0.40306
ENSMUSG00000038630	Zkscan16	zinc finger with KRAB and SCAN domains 16 [Source	0.40313
ENSMUSG00000079330	Lemd1	LEM domain containing 1 [Source:MGI Symbol;Acc:	0.40319
ENSMUSG00000028587	Orc1	origin recognition complex, subunit 1 [Source:MGI	0.4032
ENSMUSG00000022971	Ifnar2	interferon (alpha and beta) receptor 2 [Source:MGI	0.40324
ENSMUSG00000020717	Pecam1	platelet/endothelial cell adhesion molecule 1 [Sour	0.40326
ENSMUSG00000014361	Mertk	c-mer proto-oncogene tyrosine kinase [Source:MGI	0.40336
ENSMUSG00000086129	Gm13558	predicted gene 13558 [Source:MGI Symbol;Acc:MG	0.40357
ENSMUSG00000092623	Gm20413	predicted gene 20413 [Source:MGI Symbol;Acc:MG	0.40373

ENSMUSG00000025348	Itga7	integrin alpha 7 [Source:MGI Symbol;Acc:MGI:1027	0.40378
ENSMUSG00000061313	Ddhd2	DDHD domain containing 2 [Source:MGI Symbol;Ac	0.4038
ENSMUSG00000020654	Adcy3	adenylate cyclase 3 [Source:MGI Symbol;Acc:MGI:9	0.40382
ENSMUSG00000035283	Adrb1	adrenergic receptor, beta 1 [Source:MGI Symbol;Ac	0.40401
ENSMUSG00000021408	Ripk1	receptor (TNFRSF)-interacting serine-threonine kin	0.4041
ENSMUSG00000082860	Gm13204	predicted gene 13204 [Source:MGI Symbol;Acc:MG	0.40414
ENSMUSG00000070637	Gm694	predicted gene 694 [Source:MGI Symbol;Acc:MGI:2	0.40419
ENSMUSG00000020375	Rufy1	RUN and FYVE domain containing 1 [Source:MGI Sy	0.40423
ENSMUSG00000033788	Dysf	dysferlin [Source:MGI Symbol;Acc:MGI:1349385]	0.40428
ENSMUSG00000064120	Mocs1	molybdenum cofactor synthesis 1 [Source:MGI Sym	0.40433
ENSMUSG00000026171	Rnf25	ring finger protein 25 [Source:MGI Symbol;Acc:MG	0.40436
ENSMUSG00000048001	Hes5	hairy and enhancer of split 5 (Drosophila) [Source:M	0.40449
ENSMUSG00000093575	Gm20695	predicted gene 20695 [Source:MGI Symbol;Acc:MG	0.40454
ENSMUSG00000098048	Gm8330	predicted gene 8330 [Source:MGI Symbol;Acc:MGI:	0.40454
ENSMUSG00000020545	Lrrc72	leucine rich repeat containing 72 [Source:MGI Syml	0.40457
ENSMUSG00000085735	4930550G17Rik	RIKEN cDNA 4930550G17 gene [Source:MGI Symbo	0.405
ENSMUSG00000026662	Sephs1	selenophosphate synthetase 1 [Source:MGI Symbo	0.40508
ENSMUSG00000025289	Prdx4	peroxiredoxin 4 [Source:MGI Symbol;Acc:MGI:1859	0.4051
ENSMUSG00000039410	Prdm16	PR domain containing 16 [Source:MGI Symbol;Acc:	0.40513
ENSMUSG00000039798	2600006K01Rik	RIKEN cDNA 2600006K01 gene [Source:MGI Symbo	0.40531
ENSMUSG00000024505	Dtwd2	DTW domain containing 2 [Source:MGI Symbol;Acc	0.4054
ENSMUSG00000026116	Tmem131	transmembrane protein 131 [Source:MGI Symbol;A	0.40545
ENSMUSG00000002778	Kdelr1	KDEL (Lys-Asp-Glu-Leu) endoplasmic reticulum prot	0.40551
ENSMUSG00000020048	Hsp90b1	heat shock protein 90, beta (Grp94), member 1 [So	0.40555
ENSMUSG00000010342	Tex14	testis expressed gene 14 [Source:MGI Symbol;Acc:M	0.40565
ENSMUSG00000026596	Abl2	v-abl Abelson murine leukemia viral oncogene 2 (ar	0.40579
ENSMUSG00000039452	Snx22	sorting nexin 22 [Source:MGI Symbol;Acc:MGI:2685	0.40584
ENSMUSG00000029466	Anapc7	anaphase promoting complex subunit 7 [Source:MG	0.40586
ENSMUSG00000089235	Gm23119	predicted gene, 23119 [Source:MGI Symbol;Acc:MG	0.40587
ENSMUSG00000019428	Fkbp8	FK506 binding protein 8 [Source:MGI Symbol;Acc:M	0.40597
ENSMUSG00000036437	Npy1r	neuropeptide Y receptor Y1 [Source:MGI Symbol;Ac	0.40597
ENSMUSG00000056999	Ide	insulin degrading enzyme [Source:MGI Symbol;Acc:	0.40608
ENSMUSG00000029826	Zc3hav1	zinc finger CCCH type, antiviral 1 [Source:MGI Symb	0.40611
ENSMUSG00000053289	Ddx10	DEAD (Asp-Glu-Ala-Asp) box polypeptide 10 [Source	0.40624
ENSMUSG00000013701	Timm23	translocase of inner mitochondrial membrane 23 [S	0.4063
ENSMUSG00000029370	Rassf6	Ras association (RalGDS/AF-6) domain family meml	0.40635
ENSMUSG00000003308	Keap1	kelch-like ECH-associated protein 1 [Source:MGI Sy	0.4064
ENSMUSG00000033855	Ston1	stonin 1 [Source:MGI Symbol;Acc:MGI:1924307]	0.40643
ENSMUSG00000047388	Atmin	ATM interactor [Source:MGI Symbol;Acc:MGI:2682	0.40649
ENSMUSG00000089982	Gm7568	predicted gene 7568 [Source:MGI Symbol;Acc:MGI:	0.4065
ENSMUSG00000093314	Mir5136	microRNA 5136 [Source:MGI Symbol;Acc:MGI:4950	0.40651
ENSMUSG00000027562	Car2	carbonic anhydrase 2 [Source:MGI Symbol;Acc:MG	0.40658
ENSMUSG00000062859	Tcp11	t-complex protein 11 [Source:MGI Symbol;Acc:MGI	0.40683
ENSMUSG00000042099	Kank3	KN motif and ankyrin repeat domains 3 [Source:MG	0.40684
ENSMUSG00000039563	2210406O10Rik	RIKEN cDNA 2210406O10 gene [Source:MGI Symbo	0.40685
ENSMUSG00000027933	Ints3	integrator complex subunit 3 [Source:MGI Symbol;A	0.40689
ENSMUSG00000022375	Lrrc6	leucine rich repeat containing 6 (testis) [Source:MG	0.40697

ENSMUSG00000027668	Mfn1	mitofusin 1 [Source:MGI Symbol;Acc:MGI:1914664]	0.40716
ENSMUSG00000009248	Ascl2	achaete-scute complex homolog 2 (Drosophila) [So	0.40721
ENSMUSG00000032737	Inppl1	inositol polyphosphate phosphatase-like 1 [Source:	0.40726
ENSMUSG00000032786	Alas1	aminolevulinic acid synthase 1 [Source:MGI Symbo	0.40727
ENSMUSG00000020359	Phykpl	5-phosphohydroxy-L-lysine phospholyase [Source:M	0.4073
ENSMUSG00000045498	Pcdhb3	protocadherin beta 3 [Source:MGI Symbol;Acc:MGI	0.40752
ENSMUSG00000009114	2610028H24Rik	RIKEN cDNA 2610028H24 gene [Source:MGI Symbo	0.40784
ENSMUSG00000025489	Ifitm5	interferon induced transmembrane protein 5 [Sour	0.40785
ENSMUSG00000027186	Elf5	E74-like factor 5 [Source:MGI Symbol;Acc:MGI:133	0.40792
ENSMUSG00000046546	Fam43a	family with sequence similarity 43, member A [Sour	0.40804
ENSMUSG00000032323	Cyp11a1	cytochrome P450, family 11, subfamily a, polypepti	0.40805
ENSMUSG00000055882	Abhd16b	abhydrolase domain containing 16B [Source:MGI Sy	0.40806
ENSMUSG00000014601	Strip1	striatin interacting protein 1 [Source:MGI Symbol;A	0.40818
ENSMUSG00000016624	Phf21b	PHD finger protein 21B [Source:MGI Symbol;Acc:M	0.40819
ENSMUSG00000097546	Gm26749	predicted gene, 26749 [Source:MGI Symbol;Acc:M	0.40831
ENSMUSG00000040998	Npnt	nephronectin [Source:MGI Symbol;Acc:MGI:214881	0.40838
ENSMUSG00000067616	Klk11	kallikrein related-peptidase 11 [Source:MGI Symbo	0.40843
ENSMUSG00000028121	Bcar3	breast cancer anti-estrogen resistance 3 [Source:M	0.40844
ENSMUSG00000025466	Fuom	fucose mutarotase [Source:MGI Symbol;Acc:MGI:1	0.40859
ENSMUSG00000046794	Ppp1r3b	protein phosphatase 1, regulatory (inhibitor) subun	0.40859
ENSMUSG00000025037	Maoa	monoamine oxidase A [Source:MGI Symbol;Acc:MG	0.40872
ENSMUSG00000002227	Mov10	Moloney leukemia virus 10 [Source:MGI Symbol;Ac	0.40877
ENSMUSG00000031133	Arhgef6	Rac/Cdc42 guanine nucleotide exchange factor (GE	0.40886
ENSMUSG00000030935	Acsm3	acyl-CoA synthetase medium-chain family member	0.40887
ENSMUSG00000026348	Acmsd	amino carboxymuconate semialdehyde decarboxyla	0.40898
ENSMUSG00000089679	Gm16299	predicted gene 16299 [Source:MGI Symbol;Acc:MG	0.40901
ENSMUSG00000013974	1810033B17Rik	RIKEN cDNA 1810033B17 gene [Source:MGI Symbo	0.40902
ENSMUSG00000048478	Spata33	spermatogenesis associated 33 [Source:MGI Symbo	0.40903
ENSMUSG00000078572	1810043H04Rik	RIKEN cDNA 1810043H04 gene [Source:MGI Symbo	0.40903
ENSMUSG00000034936	Arl4d	ADP-ribosylation factor-like 4D [Source:MGI Symbo	0.40913
ENSMUSG00000032802	Srxn1	sulfiredoxin 1 homolog (S. cerevisiae) [Source:MGI	0.40914
ENSMUSG00000014873	Surf2	surfeit gene 2 [Source:MGI Symbol;Acc:MGI:98444]	0.40917
ENSMUSG00000024440	Pcdh12	protocadherin 12 [Source:MGI Symbol;Acc:MGI:185	0.40926
ENSMUSG00000042943	4922501L14Rik	RIKEN cDNA 4922501L14 gene [Source:MGI Symbo	0.4093
ENSMUSG00000097143	Gm17608	predicted gene, 17608 [Source:MGI Symbol;Acc:M	0.4094
ENSMUSG00000031982	Arv1	ARV1 homolog (yeast) [Source:MGI Symbol;Acc:MG	0.40947
ENSMUSG00000083695	Rnf138rt1	ring finger protein 138, retrogene 1 [Source:MGI Sy	0.40951
ENSMUSG00000042671	Rgs8	regulator of G-protein signaling 8 [Source:MGI Sym	0.40958
ENSMUSG00000087384	Gm15558	predicted gene 15558 [Source:MGI Symbol;Acc:MG	0.40958
ENSMUSG00000026707	Nsun6	NOL1/NOP2/Sun domain family member 6 [Source	0.40959
ENSMUSG00000090323	Gm5263	predicted gene 5263 [Source:MGI Symbol;Acc:MGI:	0.4096
ENSMUSG00000004945	Tmem242	transmembrane protein 242 [Source:MGI Symbol;A	0.40962
ENSMUSG00000075023	Accsl	1-aminocyclopropane-1-carboxylate synthase (non-	0.40969
ENSMUSG00000023052	Npff	neuropeptide FF-amide peptide precursor [Source:	0.40984
ENSMUSG00000097801	Gm26777	predicted gene, 26777 [Source:MGI Symbol;Acc:M	0.40986
ENSMUSG00000049489	Fam58b	family with sequence similarity 58, member B [Sou	0.40992
ENSMUSG00000091898	Tnnc1	troponin C, cardiac/slow skeletal [Source:MGI Symb	0.40992

ENSMUSG00000020330	Hmmr	hyaluronan mediated motility receptor (RHAMM) [S	0.40998
ENSMUSG00000014496	Ankrd28	ankyrin repeat domain 28 [Source:MGI Symbol;Acc	0.41001
ENSMUSG00000034757	Tmub2	transmembrane and ubiquitin-like domain containi	0.41007
ENSMUSG00000001016	Ilf2	interleukin enhancer binding factor 2 [Source:MGI S	0.4101
ENSMUSG00000041064	Pif1	PIF1 5'-to-3' DNA helicase homolog (S. cerevisiae) [S	0.4101
ENSMUSG00000054135	A430110L20Rik	RIKEN cDNA A430110L20 gene [Source:MGI Symbo	0.41012
ENSMUSG00000094634	Gm3468	predicted gene 3468 [Source:MGI Symbol;Acc:MGI:	0.41012
ENSMUSG00000053613	4732444A12Rik	RIKEN cDNA 4732444A12 gene [Source:MGI Symbo	0.41013
ENSMUSG00000031483	Erlin2	ER lipid raft associated 2 [Source:MGI Symbol;Acc:N	0.41015
ENSMUSG00000029420	Rimbp2	RIMS binding protein 2 [Source:MGI Symbol;Acc:M	0.41019
ENSMUSG00000021716	Srek1ip1	splicing regulatory glutamine/lysine-rich protein 1ir	0.41024
ENSMUSG00000095105	Edaradd	EDAR (ectodysplasin-A receptor)-associated death	0.41036
ENSMUSG00000040848	Sft2d2	SFT2 domain containing 2 [Source:MGI Symbol;Acc	0.41037
ENSMUSG00000071537	Klrg2	killer cell lectin-like receptor subfamily G, member	0.41039
ENSMUSG00000048058	Ldlrad3	low density lipoprotein receptor class A domain cor	0.41042
ENSMUSG00000067299	Crygd	crystallin, gamma D [Source:MGI Symbol;Acc:MGI:8	0.41047
ENSMUSG00000031681	Smad1	SMAD family member 1 [Source:MGI Symbol;Acc:M	0.41049
ENSMUSG00000079993	Gm11799	predicted gene 11799 [Source:MGI Symbol;Acc:MG	0.41058
ENSMUSG00000052496	Pkdrej	polycystic kidney disease (polycystin) and REJ (sper	0.41059
ENSMUSG00000054091	1810037I17Rik	RIKEN cDNA 1810037I17 gene [Source:MGI Symbol	0.41066
ENSMUSG00000027952	Pmvk	phosphomevalonate kinase [Source:MGI Symbol;Ac	0.41074
ENSMUSG00000041361	Myzap	myocardial zonula adherens protein [Source:MGI S	0.41074
ENSMUSG00000043017	Ptgir	prostaglandin I receptor (IP) [Source:MGI Symbol;A	0.41076
ENSMUSG00000037419	Endod1	endonuclease domain containing 1 [Source:MGI Sy	0.41084
ENSMUSG00000085433	Gm16001	predicted gene 16001 [Source:MGI Symbol;Acc:MG	0.41084
ENSMUSG00000036510	Cdh8	cadherin 8 [Source:MGI Symbol;Acc:MGI:107434]	0.41089
ENSMUSG00000020490	Btnl10	butyrophilin-like 10 [Source:MGI Symbol;Acc:MGI:2	0.41091
ENSMUSG00000023274	Cd4	CD4 antigen [Source:MGI Symbol;Acc:MGI:88335]	0.41099
ENSMUSG00000029797	Sspo	SCO-spondin [Source:MGI Symbol;Acc:MGI:267431	0.41102
ENSMUSG00000024538	Ppic	peptidylprolyl isomerase C [Source:MGI Symbol;Acc	0.41115
ENSMUSG00000020265	Sumo3	SMT3 suppressor of mif two 3 homolog 3 (yeast) [S	0.41122
ENSMUSG00000023143	Nagpa	N-acetylglucosamine-1-phosphodiester alpha-N-ace	0.41125
ENSMUSG00000026238	Ptma	prothymosin alpha [Source:MGI Symbol;Acc:MGI:9	0.41129
ENSMUSG00000056300	Gm13247	predicted gene 13247 [Source:MGI Symbol;Acc:MG	0.41133
ENSMUSG00000092932	Gm22906	predicted gene, 22906 [Source:MGI Symbol;Acc:MG	0.41147
ENSMUSG00000085565	Gm15721	predicted gene 15721 [Source:MGI Symbol;Acc:MG	0.41149
ENSMUSG00000025410	Dctn2	dynactin 2 [Source:MGI Symbol;Acc:MGI:107733]	0.41151
ENSMUSG00000019872	Smpdl3a	sphingomyelin phosphodiesterase, acid-like 3A [Sou	0.41159
ENSMUSG00000085521	4930526A20Rik	RIKEN cDNA 4930526A20 gene [Source:MGI Symbo	0.41166
ENSMUSG00000024041	Cryaa	crystallin, alpha A [Source:MGI Symbol;Acc:MGI:88	0.41167
ENSMUSG00000024843	Chka	choline kinase alpha [Source:MGI Symbol;Acc:MGI:	0.41167
ENSMUSG00000022738	Gsc2	goosecoid homebox 2 [Source:MGI Symbol;Acc:MG	0.41178
ENSMUSG00000039155	Cdh26	cadherin-like 26 [Source:MGI Symbol;Acc:MGI:2685	0.41181
ENSMUSG00000053332	Gas5	growth arrest specific 5 [Source:MGI Symbol;Acc:M	0.41185
ENSMUSG00000097108	Gm26581	predicted gene, 26581 [Source:MGI Symbol;Acc:MG	0.41198
ENSMUSG00000085818	Gm13267	predicted gene 13267 [Source:MGI Symbol;Acc:MG	0.41199
ENSMUSG00000028779	Pef1	penta-EF hand domain containing 1 [Source:MGI Sy	0.41209

ENSMUSG00000039781	Azi1	5-azacytidine induced gene 1 [Source:MGI Symbol;A	0.41214
ENSMUSG00000033405	Nudt15	nudix (nucleoside diphosphate linked moiety X)-typ	0.41228
ENSMUSG00000041130	Zfp598	zinc finger protein 598 [Source:MGI Symbol;Acc:M(	0.41236
ENSMUSG00000051896	Tex37	testis expressed 37 [Source:MGI Symbol;Acc:MGI:1	0.41238
ENSMUSG00000029125	Stx18	syntaxin 18 [Source:MGI Symbol;Acc:MGI:1918366]	0.41244
ENSMUSG00000046168	Kcnrg	potassium channel regulator [Source:MGI Symbol;A	0.41245
ENSMUSG00000083902	Gm15975	predicted gene 15975 [Source:MGI Symbol;Acc:MG	0.41246
ENSMUSG00000019865	Nmbr	neuromedin B receptor [Source:MGI Symbol;Acc:M	0.41265
ENSMUSG00000040936	Ulk4	unc-51-like kinase 4 [Source:MGI Symbol;Acc:MGI:1	0.41265
ENSMUSG00000025940	Tmem70	transmembrane protein 70 [Source:MGI Symbol;Ac	0.41267
ENSMUSG00000084992	Gm13842	predicted gene 13842 [Source:MGI Symbol;Acc:MG	0.41282
ENSMUSG00000094158	Gm23264	predicted gene, 23264 [Source:MGI Symbol;Acc:M(	0.41301
ENSMUSG00000019969	Psen1	presenilin 1 [Source:MGI Symbol;Acc:MGI:1202717	0.41303
ENSMUSG00000040007	Bahd1	bromo adjacent homology domain containing 1 [So	0.41306
ENSMUSG00000052675	Zfp112	zinc finger protein 112 [Source:MGI Symbol;Acc:M(	0.41314
ENSMUSG00000030468	Siglecg	sialic acid binding Ig-like lectin G [Source:MGI Symb	0.41315
ENSMUSG00000091648	C2cd4d	C2 calcium-dependent domain containing 4D [Sour	0.41339
ENSMUSG00000044037	Als2cl	ALS2 C-terminal like [Source:MGI Symbol;Acc:MGI:1	0.41352
ENSMUSG00000087268	Gm14486	predicted gene 14486 [Source:MGI Symbol;Acc:MG	0.41353
ENSMUSG00000073490	AI607873	expressed sequence AI607873 [Source:MGI Symbol	0.41361
ENSMUSG00000039653	Baat	bile acid-Coenzyme A: amino acid N-acyltransferase	0.41362
ENSMUSG00000022288	4930447A16Rik	RIKEN cDNA 4930447A16 gene [Source:MGI Symbo	0.41368
ENSMUSG00000002204	Napsa	napsin A aspartic peptidase [Source:MGI Symbol;Ac	0.41372
ENSMUSG00000078365	Mos	Moloney sarcoma oncogene [Source:MGI Symbol;A	0.41377
ENSMUSG00000061535	C1qtnf7	C1q and tumor necrosis factor related protein 7 [So	0.41382
ENSMUSG00000068206	Pick1	protein interacting with C kinase 1 [Source:MGI Syn	0.41391
ENSMUSG00000006850	Tmco6	transmembrane and coiled-coil domains 6 [Source:	0.41393
ENSMUSG00000038408	1700018A04Rik	RIKEN cDNA 1700018A04 gene [Source:MGI Symbo	0.41394
ENSMUSG00000067860	Zic3	zinc finger protein of the cerebellum 3 [Source:MG	0.41413
ENSMUSG00000030970	Ctbp2	C-terminal binding protein 2 [Source:MGI Symbol;A	0.41423
ENSMUSG00000098158	Gm4804	predicted gene 4804 [Source:MGI Symbol;Acc:MGI:	0.41434
ENSMUSG00000096971	4930556M19Rik	RIKEN cDNA 4930556M19 gene [Source:MGI Symb	0.41437
ENSMUSG00000027438	Napb	N-ethylmaleimide sensitive fusion protein attachm	0.41447
ENSMUSG00000008036	Ap2s1	adaptor-related protein complex 2, sigma 1 subunit	0.41451
ENSMUSG00000087347	1700047K16Rik	RIKEN cDNA 1700047K16 gene [Source:MGI Symbo	0.4146
ENSMUSG00000087995	Gm25054	predicted gene, 25054 [Source:MGI Symbol;Acc:M(	0.41462
ENSMUSG00000089959	Gm16268	predicted gene 16268 [Source:MGI Symbol;Acc:MG	0.41463
ENSMUSG00000061207	Stk19	serine/threonine kinase 19 [Source:MGI Symbol;Ac	0.41464
ENSMUSG00000087458	Gm13999	predicted gene 13999 [Source:MGI Symbol;Acc:MG	0.41464
ENSMUSG00000037514	Pank2	pantothenate kinase 2 [Source:MGI Symbol;Acc:M(	0.41482
ENSMUSG00000097117	Gm26730	predicted gene, 26730 [Source:MGI Symbol;Acc:M(	0.41496
ENSMUSG00000021982	Cdadc1	cytidine and dCMP deaminase domain containing 1	0.415
ENSMUSG00000035517	Tdrd7	tudor domain containing 7 [Source:MGI Symbol;Acc	0.41504
ENSMUSG00000090406	Gm17058	predicted gene 17058 [Source:MGI Symbol;Acc:MG	0.41504
ENSMUSG00000020461	Clhc1	clathrin heavy chain linker domain containing 1 [So	0.41508
ENSMUSG00000073838	Tufm	Tu translation elongation factor, mitochondrial [Sou	0.41512
ENSMUSG00000077711	AF357399	snoRNA AF357399 [Source:MGI Symbol;Acc:MGI:54	0.41516

ENSMUSG00000083076	Gm11408	predicted gene 11408 [Source:MGI Symbol;Acc:MG	0.41516
ENSMUSG00000020770	Unk	unkempt homolog (Drosophila) [Source:MGI Symbo	0.41541
ENSMUSG00000029915	Clec5a	C-type lectin domain family 5, member a [Source:M	0.41541
ENSMUSG00000062300	Pvrl2	poliovirus receptor-related 2 [Source:MGI Symbol;A	0.4155
ENSMUSG00000037197	Rbm17	RNA binding motif protein 17 [Source:MGI Symbol;	0.41552
ENSMUSG00000097706	E030037K01Rik	RIKEN cDNA E030037K01 gene [Source:MGI Symbo	0.41554
ENSMUSG00000085501	Gm11772	predicted gene 11772 [Source:MGI Symbol;Acc:MG	0.41571
ENSMUSG00000028700	Pomgnt1	protein O-linked mannose beta 1,2-N-acetylglucosa	0.41578
ENSMUSG00000022969	Il10rb	interleukin 10 receptor, beta [Source:MGI Symbol;A	0.41579
ENSMUSG00000006676	Usp19	ubiquitin specific peptidase 19 [Source:MGI Symbo	0.41581
ENSMUSG00000036622	Atp13a2	ATPase type 13A2 [Source:MGI Symbol;Acc:MGI:19	0.41587
ENSMUSG00000046338	Gpat2	glycerol-3-phosphate acyltransferase 2, mitochond	0.41587
ENSMUSG00000041570	Camsap2	calmodulin regulated spectrin-associated protein fa	0.41591
ENSMUSG00000031540	Kat6a	K(lysine) acetyltransferase 6A [Source:MGI Symbol;	0.41599
ENSMUSG00000040723	Rcsd1	RCSD domain containing 1 [Source:MGI Symbol;Acc	0.41605
ENSMUSG00000025959	Klf7	Kruppel-like factor 7 (ubiquitous) [Source:MGI Sym	0.41619
ENSMUSG00000047675	Rps8	ribosomal protein S8 [Source:MGI Symbol;Acc:MGI	0.41622
ENSMUSG00000030861	Acadsb	acyl-Coenzyme A dehydrogenase, short/branched c	0.41631
ENSMUSG00000022201	Zfr	zinc finger RNA binding protein [Source:MGI Symbo	0.41651
ENSMUSG00000022371	Col14a1	collagen, type XIV, alpha 1 [Source:MGI Symbol;Acc	0.41655
ENSMUSG00000014769	Psmb1	proteasome (prosome, macropain) subunit, beta ty	0.4166
ENSMUSG00000075425	Gm13547	predicted gene 13547 [Source:MGI Symbol;Acc:MG	0.41667
ENSMUSG00000033075	Senp1	SUMO1/sentrin specific peptidase 1 [Source:MGI Sy	0.41671
ENSMUSG00000092085	Gm17224	predicted gene 17224 [Source:MGI Symbol;Acc:MG	0.41673
ENSMUSG00000057722	Lepr	leptin receptor [Source:MGI Symbol;Acc:MGI:1049	0.41686
ENSMUSG00000090031	4732440D04Rik	RIKEN cDNA 4732440D04 gene [Source:MGI Symbo	0.41701
ENSMUSG00000097467	Gm26737	predicted gene, 26737 [Source:MGI Symbol;Acc:M	0.41704
ENSMUSG00000040298	Btbd16	BTB (POZ) domain containing 16 [Source:MGI Symb	0.41705
ENSMUSG00000034908	Sidt2	SID1 transmembrane family, member 2 [Source:M	0.41711
ENSMUSG00000021799	Opn4	opsin 4 (melanopsin) [Source:MGI Symbol;Acc:MGI	0.41715
ENSMUSG00000024033	Rsph1	radial spoke head 1 homolog (Chlamydomonas) [So	0.4172
ENSMUSG00000034403	Pja1	praja1, RING-H2 motif containing [Source:MGI Sym	0.41728
ENSMUSG00000029126	Nsg1	neuron specific gene family member 1 [Source:MG	0.41738
ENSMUSG00000000154	Slc22a18	solute carrier family 22 (organic cation transporter)	0.41742
ENSMUSG00000053675	Tgm5	transglutaminase 5 [Source:MGI Symbol;Acc:MGI:1	0.41746
ENSMUSG00000030047	Arhgap25	Rho GTPase activating protein 25 [Source:MGI Sym	0.41762
ENSMUSG00000040374	Pex2	peroxisomal biogenesis factor 2 [Source:MGI Symb	0.41768
ENSMUSG00000084866	A930006K02Rik	RIKEN cDNA A930006K02 gene [Source:MGI Symbo	0.41779
ENSMUSG00000041360	D19Bwg1357e	DNA segment, Chr 19, Brigham & Women's Genetic	0.41781
ENSMUSG00000090157	Gm16534	predicted gene 16534 [Source:MGI Symbol;Acc:MG	0.41784
ENSMUSG00000051504	Siglech	sialic acid binding Ig-like lectin H [Source:MGI Symb	0.41785
ENSMUSG00000009731	Kcnd1	potassium voltage-gated channel, Shal-related fam	0.41789
ENSMUSG00000028344	Invs	inversin [Source:MGI Symbol;Acc:MGI:1335082]	0.41796
ENSMUSG00000057497	Fam136a	family with sequence similarity 136, member A [Sou	0.41796
ENSMUSG00000027603	Ggt7	gamma-glutamyltransferase 7 [Source:MGI Symbol	0.41803
ENSMUSG00000056602	Fry	furry homolog (Drosophila) [Source:MGI Symbol;Ac	0.41809
ENSMUSG00000097284	4930480K23Rik	RIKEN cDNA 4930480K23 gene [Source:MGI Symbo	0.41815

ENSMUSG00000088378	Gm25184	predicted gene, 25184 [Source:MGI Symbol;Acc:MG	0.41816
ENSMUSG00000083910	Gm13604	predicted gene 13604 [Source:MGI Symbol;Acc:MG	0.41817
ENSMUSG00000057778	Cyb5d2	cytochrome b5 domain containing 2 [Source:MGI Sy	0.41837
ENSMUSG00000085119	Gm13644	predicted gene 13644 [Source:MGI Symbol;Acc:MG	0.41837
ENSMUSG00000034116	Vav1	vav 1 oncogene [Source:MGI Symbol;Acc:MGI:9892	0.41848
ENSMUSG00000038084	Opa1	optic atrophy 1 [Source:MGI Symbol;Acc:MGI:1921	0.41852
ENSMUSG00000026127	Imp4	IMP4, U3 small nucleolar ribonucleoprotein, homol	0.41855
ENSMUSG00000098208	Gm8349	predicted gene 8349 [Source:MGI Symbol;Acc:MGI:	0.41863
ENSMUSG00000028393	Alad	aminolevulinate, delta-, dehydratase [Source:MGI S	0.41865
ENSMUSG00000029409	U90926	cDNA sequence U90926 [Source:MGI Symbol;Acc:M	0.4187
ENSMUSG00000031545	Agpat6	1-acylglycerol-3-phosphate O-acyltransferase 6 (lys	0.41874
ENSMUSG00000097410	Gm26668	predicted gene, 26668 [Source:MGI Symbol;Acc:MG	0.41887
ENSMUSG00000074228	Gm10645	predicted gene 10645 [Source:MGI Symbol;Acc:MG	0.4189
ENSMUSG00000048939	Atp13a5	ATPase type 13A5 [Source:MGI Symbol;Acc:MGI:24	0.41893
ENSMUSG00000052369	Tmem106c	transmembrane protein 106C [Source:MGI Symbol;	0.41893
ENSMUSG00000018334	Ksr1	kinase suppressor of ras 1 [Source:MGI Symbol;Acc	0.41898
ENSMUSG00000026600	Soat1	sterol O-acyltransferase 1 [Source:MGI Symbol;Acc	0.41898
ENSMUSG00000035551	Igfbpl1	insulin-like growth factor binding protein-like 1 [Sou	0.41898
ENSMUSG00000090693	Trim43a	tripartite motif-containing 43A [Source:MGI Symbo	0.41903
ENSMUSG00000027890	Gstm4	glutathione S-transferase, mu 4 [Source:MGI Symbo	0.41907
ENSMUSG00000024087	Cyp1b1	cytochrome P450, family 1, subfamily b, polypeptid	0.41909
ENSMUSG00000024989	Cep55	centrosomal protein 55 [Source:MGI Symbol;Acc:M	0.4191
ENSMUSG00000022159	Rab2b	RAB2B, member RAS oncogene family [Source:MGI	0.41913
ENSMUSG00000037415	Ranbp10	RAN binding protein 10 [Source:MGI Symbol;Acc:M	0.41916
ENSMUSG00000002781	Tmem143	transmembrane protein 143 [Source:MGI Symbol;A	0.41933
ENSMUSG00000074215	Gm10643	predicted gene 10643 [Source:MGI Symbol;Acc:MG	0.41936
ENSMUSG00000002769	Gnmt	glycine N-methyltransferase [Source:MGI Symbol;A	0.41941
ENSMUSG00000073738	Gm10567	predicted gene 10567 [Source:MGI Symbol;Acc:MG	0.41943
ENSMUSG00000033373	Fntb	farnesyltransferase, CAAX box, beta [Source:MGI Sy	0.41949
ENSMUSG00000097879	Gm26869	predicted gene, 26869 [Source:MGI Symbol;Acc:MG	0.41951
ENSMUSG00000068120	Gm10231	predicted pseudogene 10231 [Source:MGI Symbol;	0.41958
ENSMUSG00000090812	Samd15	sterile alpha motif domain containing 15 [Source:M	0.41969
ENSMUSG00000097093	C330013E15Rik	RIKEN cDNA C330013E15 gene [Source:MGI Symbo	0.41972
ENSMUSG00000097854	Gm26602	predicted gene, 26602 [Source:MGI Symbol;Acc:MG	0.41977
ENSMUSG00000017715	Pgs1	phosphatidylglycerophosphate synthase 1 [Source:	0.41995
ENSMUSG00000029155	Spata18	spermatogenesis associated 18 [Source:MGI Symbo	0.41999
ENSMUSG00000057069	Ero1lb	ERO1-like beta (S. cerevisiae) [Source:MGI Symbol;A	0.42003
ENSMUSG00000031257	Nox1	NADPH oxidase 1 [Source:MGI Symbol;Acc:MGI:245	0.42004
ENSMUSG00000007589	Tinf2	Terf1 (TRF1)-interacting nuclear factor 2 [Source:M	0.42017
ENSMUSG00000009112	Bcl2l13	BCL2-like 13 (apoptosis facilitator) [Source:MGI Sym	0.4202
ENSMUSG00000035198	Tubg1	tubulin, gamma 1 [Source:MGI Symbol;Acc:MGI:10	0.42029
ENSMUSG00000057236	Rbbp4	retinoblastoma binding protein 4 [Source:MGI Sym	0.42033
ENSMUSG00000090891	D6Ertd527e	DNA segment, Chr 6, ERATO Doi 527, expressed [So	0.42041
ENSMUSG00000036285	Noa1	nitric oxide associated 1 [Source:MGI Symbol;Acc:M	0.42043
ENSMUSG00000093800	Gm20796	predicted gene, 20796 [Source:MGI Symbol;Acc:MG	0.42048
ENSMUSG00000029201	Ugdh	UDP-glucose dehydrogenase [Source:MGI Symbol;A	0.42058
ENSMUSG00000031445	Proz	protein Z, vitamin K-dependent plasma glycoprotei	0.42061

ENSMUSG00000034429	Zfp707	zinc finger protein 707 [Source:MGI Symbol;Acc:MG	0.42078
ENSMUSG00000097718	Gm26896	predicted gene, 26896 [Source:MGI Symbol;Acc:MG	0.4208
ENSMUSG00000027203	Dut	deoxyuridine triphosphatase [Source:MGI Symbol;A	0.42084
ENSMUSG00000034947	Tmem106a	transmembrane protein 106A [Source:MGI Symbol;	0.42087
ENSMUSG00000089542	Gm25835	predicted gene, 25835 [Source:MGI Symbol;Acc:MG	0.42087
ENSMUSG00000015016	Acsf3	acyl-CoA synthetase family member 3 [Source:MGI	0.42088
ENSMUSG00000004846	Plod3	procollagen-lysine, 2-oxoglutarate 5-dioxygenase 3	0.42093
ENSMUSG00000059864	Olfr1393	olfactory receptor 1393 [Source:MGI Symbol;Acc:M	0.42096
ENSMUSG00000035274	Tpbg	trophoblast glycoprotein [Source:MGI Symbol;Acc:I	0.42098
ENSMUSG00000038697	Taf5l	TAF5-like RNA polymerase II, p300/CBP-associated	0.421
ENSMUSG00000085399	9130206I24Rik	RIKEN cDNA 9130206I24 gene [Source:MGI Symbol	0.42112
ENSMUSG00000086662	BC046251	cDNA sequence BC046251 [Source:MGI Symbol;Acc	0.42119
ENSMUSG00000074793	Hspa12b	heat shock protein 12B [Source:MGI Symbol;Acc:M	0.42121
ENSMUSG00000080454	Gm24195	predicted gene, 24195 [Source:MGI Symbol;Acc:MG	0.42134
ENSMUSG00000025243	Slc6a20b	solute carrier family 6 (neurotransmitter transporte	0.42138
ENSMUSG00000087151	Gm14022	predicted gene 14022 [Source:MGI Symbol;Acc:MG	0.42139
ENSMUSG00000097744	D030040B21Rik	RIKEN cDNA D030040B21 gene [Source:MGI Symbo	0.42141
ENSMUSG00000023980	Taf8	TAF8 RNA polymerase II, TATA box binding protein	0.42142
ENSMUSG00000075042	4930431P03Rik	RIKEN cDNA 4930431P03 gene [Source:MGI Symbo	0.42162
ENSMUSG00000049692	Tmem239	transmembrane 239 [Source:MGI Symbol;Acc:MGI:	0.42168
ENSMUSG00000083015	Gm16518	predicted gene, 16518 [Source:MGI Symbol;Acc:MG	0.42169
ENSMUSG00000004187	Kifc2	kinesin family member C2 [Source:MGI Symbol;Acc	0.42185
ENSMUSG00000041297	Cdk13	cyclin-dependent kinase 13 [Source:MGI Symbol;Ac	0.42194
ENSMUSG00000027575	Arfgap1	ADP-ribosylation factor GTPase activating protein 1	0.42203
ENSMUSG00000089757	Gm16334	predicted gene 16334 [Source:MGI Symbol;Acc:MG	0.42209
ENSMUSG00000052534	Pbx1	pre B cell leukemia homeobox 1 [Source:MGI Symb	0.42218
ENSMUSG00000087174	5530601H04Rik	RIKEN cDNA 5530601H04 gene [Source:MGI Symbo	0.42228
ENSMUSG00000062867	Impdh2	inosine 5'-phosphate dehydrogenase 2 [Source:MG	0.42247
ENSMUSG00000087578	Gm15605	predicted gene 15605 [Source:MGI Symbol;Acc:MG	0.42256
ENSMUSG00000026245	Farsb	phenylalanyl-tRNA synthetase, beta subunit [Sourc	0.42261
ENSMUSG00000023159	Psg29	pregnancy-specific glycoprotein 29 [Source:MGI Sy	0.42264
ENSMUSG00000030741	Spns1	spinster homolog 1 [Source:MGI Symbol;Acc:MGI:1	0.42265
ENSMUSG00000027702	Lrrc34	leucine rich repeat containing 34 [Source:MGI Sym	0.42272
ENSMUSG00000022893	Adamts1	a disintegrin-like and metallopeptidase (reprolysin	0.4229
ENSMUSG00000003437	Paf1	Paf1, RNA polymerase II associated factor, homolog	0.42296
ENSMUSG00000061864	Galntl6	UDP-N-acetyl-alpha-D-galactosamine:polypeptide N	0.423
ENSMUSG00000049184	Purg	purine-rich element binding protein G [Source:MGI	0.42301
ENSMUSG00000030541	Idh2	isocitrate dehydrogenase 2 (NADP+), mitochondria	0.42318
ENSMUSG00000049866	Arl4c	ADP-ribosylation factor-like 4C [Source:MGI Symbo	0.4232
ENSMUSG00000063952	Brpf3	bromodomain and PHD finger containing, 3 [Source	0.4233
ENSMUSG00000034833	Tespa1	thymocyte expressed, positive selection associated	0.42339
ENSMUSG00000044258	Ctla2a	cytotoxic T lymphocyte-associated protein 2 alpha	0.42341
ENSMUSG00000042705	Commd10	COMM domain containing 10 [Source:MGI Symbol;	0.42345
ENSMUSG00000052414	Atf7	activating transcription factor 7 [Source:MGI Symbo	0.42345
ENSMUSG00000056973	Ces1d	carboxylesterase 1D [Source:MGI Symbol;Acc:MGI:	0.42345
ENSMUSG00000029380	Cxcl1	chemokine (C-X-C motif) ligand 1 [Source:MGI Sym	0.42351
ENSMUSG00000021490	Slc34a1	solute carrier family 34 (sodium phosphate), memb	0.42356

ENSMUSG00000025091	Pnliprp2	pancreatic lipase-related protein 2 [Source:MGI Syr	0.42359
ENSMUSG00000015143	Actn1	actinin, alpha 1 [Source:MGI Symbol;Acc:MGI:2137	0.42361
ENSMUSG00000064105	Cnnm2	cyclin M2 [Source:MGI Symbol;Acc:MGI:2151054]	0.42364
ENSMUSG00000097794	Gm2990	predicted gene 2990 [Source:MGI Symbol;Acc:MGI:	0.42365
ENSMUSG00000051735	Rinl	Ras and Rab interactor-like [Source:MGI Symbol;Ac	0.42375
ENSMUSG00000010663	Fads1	fatty acid desaturase 1 [Source:MGI Symbol;Acc:M	0.42377
ENSMUSG00000004371	Il11	interleukin 11 [Source:MGI Symbol;Acc:MGI:10761	0.42385
ENSMUSG00000019852	D10Bwg1379e	DNA segment, Chr 10, Brigham & Women's Genetic	0.42392
ENSMUSG00000062861	Zfp28	zinc finger protein 28 [Source:MGI Symbol;Acc:MGI	0.42397
ENSMUSG00000027983	Cyp2u1	cytochrome P450, family 2, subfamily u, polypeptid	0.42405
ENSMUSG00000032649	Colgalt2	collagen beta(1-O)galactosyltransferase 2 [Source:N	0.42419
ENSMUSG00000005986	Ankrd13d	ankyrin repeat domain 13 family, member D [Sourc	0.42425
ENSMUSG00000038776	Ephx1	epoxide hydrolase 1, microsomal [Source:MGI Sym	0.4243
ENSMUSG00000030494	Rhpn2	rhophilin, Rho GTPase binding protein 2 [Source:M	0.42439
ENSMUSG00000028795	Ccdc28b	coiled coil domain containing 28B [Source:MGI Sym	0.42441
ENSMUSG00000047259	Mc4r	melanocortin 4 receptor [Source:MGI Symbol;Acc:N	0.4245
ENSMUSG00000032911	Cspg4	chondroitin sulfate proteoglycan 4 [Source:MGI Syr	0.42453
ENSMUSG00000043432	Leng9	leukocyte receptor cluster (LRC) member 9 [Source	0.4248
ENSMUSG00000097068	Gm26760	predicted gene, 26760 [Source:MGI Symbol;Acc:M	0.42491
ENSMUSG00000037341	Slc9a7	solute carrier family 9 (sodium/hydrogen exchange	0.42498
ENSMUSG00000036948	BC037034	cDNA sequence BC037034 [Source:MGI Symbol;Acc	0.42512
ENSMUSG00000049382	Krt8	keratin 8 [Source:MGI Symbol;Acc:MGI:96705]	0.42517
ENSMUSG00000081605	Gm15953	predicted gene 15953 [Source:MGI Symbol;Acc:MG	0.42518
ENSMUSG00000031075	Ano1	anoctamin 1, calcium activated chloride channel [S	0.42522
ENSMUSG00000020009	Ifngr1	interferon gamma receptor 1 [Source:MGI Symbol;	0.42539
ENSMUSG00000044982	Sft2d3	SFT2 domain containing 3 [Source:MGI Symbol;Acc	0.42551
ENSMUSG00000085958	A930019D19Rik	RIKEN cDNA A930019D19 gene [Source:MGI Symbo	0.42567
ENSMUSG00000057719	Sh3rf2	SH3 domain containing ring finger 2 [Source:MGI Sy	0.42568
ENSMUSG00000085188	Gm11368	predicted gene 11368 [Source:MGI Symbol;Acc:MG	0.42571
ENSMUSG00000084391	Gm13967	predicted gene 13967 [Source:MGI Symbol;Acc:MG	0.42573
ENSMUSG00000081153	Gm11401	predicted gene 11401 [Source:MGI Symbol;Acc:MG	0.42578
ENSMUSG00000028797	Tmem234	transmembrane protein 234 [Source:MGI Symbol;A	0.4258
ENSMUSG00000037754	Ppp1r16b	protein phosphatase 1, regulatory (inhibitor) subun	0.42585
ENSMUSG00000041957	Pkp2	plakophilin 2 [Source:MGI Symbol;Acc:MGI:191470	0.42585
ENSMUSG00000096370	Gm21992	predicted gene 21992 [Source:MGI Symbol;Acc:MG	0.42588
ENSMUSG00000022504	Ciita	class II transactivator [Source:MGI Symbol;Acc:MGI	0.42591
ENSMUSG00000025373	Rnf41	ring finger protein 41 [Source:MGI Symbol;Acc:MGI	0.42592
ENSMUSG00000095407	Tmem200c	transmembrane protein 200C [Source:MGI Symbol;	0.42592
ENSMUSG00000038518	Jarid2	jumonji, AT rich interactive domain 2 [Source:MGI S	0.42598
ENSMUSG00000087590	2410004N09Rik	RIKEN cDNA 2410004N09 gene [Source:MGI Symbo	0.42613
ENSMUSG00000073788	Gm10576	predicted gene 10576 [Source:MGI Symbol;Acc:MG	0.42615
ENSMUSG00000085931	Gm12648	predicted gene 12648 [Source:MGI Symbol;Acc:MG	0.42619
ENSMUSG00000026576	Atp1b1	ATPase, Na+/K+ transporting, beta 1 polypeptide [S	0.42622
ENSMUSG00000020484	Xbp1	X-box binding protein 1 [Source:MGI Symbol;Acc:M	0.42624
ENSMUSG00000085181	Gm12709	predicted gene 12709 [Source:MGI Symbol;Acc:MG	0.42629
ENSMUSG00000086842	Gm15681	predicted gene 15681 [Source:MGI Symbol;Acc:MG	0.42631
ENSMUSG00000024218	Taf11	TAF11 RNA polymerase II, TATA box binding protein	0.42637

ENSMUSG00000002190	Clgn	calmegin [Source:MGI Symbol;Acc:MGI:107472]	0.42644
ENSMUSG00000097814	Gm5101	predicted gene 5101 [Source:MGI Symbol;Acc:MGI:	0.42649
ENSMUSG00000032380	Dapk2	death-associated protein kinase 2 [Source:MGI Sym	0.42652
ENSMUSG00000027593	Raly	hnRNP-associated with lethal yellow [Source:MGI S	0.42659
ENSMUSG00000091587	Gm17191	predicted gene 17191 [Source:MGI Symbol;Acc:MG	0.4266
ENSMUSG00000087047	1700110K17Rik	RIKEN cDNA 1700110K17 gene [Source:MGI Symbo	0.42665
ENSMUSG00000020689	Itgb3	integrin beta 3 [Source:MGI Symbol;Acc:MGI:96612	0.42667
ENSMUSG00000049680	Urgcp	upregulator of cell proliferation [Source:MGI Symb	0.42673
ENSMUSG00000026875	Traf1	TNF receptor-associated factor 1 [Source:MGI Sym	0.42674
ENSMUSG00000035852	Misp	mitotic spindle positioning [Source:MGI Symbol;Ac	0.42674
ENSMUSG00000035916	Ptprq	protein tyrosine phosphatase, receptor type, Q [So	0.42688
ENSMUSG00000067344	Rps25-ps1	ribosomal protein S25, pseudogene 1 [Source:MGI	0.42695
ENSMUSG00000020919	Stat5b	signal transducer and activator of transcription 5B	0.42699
ENSMUSG00000022204	Ngdn	neuroguidin, EIF4E binding protein [Source:MGI Sy	0.42701
ENSMUSG00000093816	Gm26445	predicted gene, 26445 [Source:MGI Symbol;Acc:M	0.42706
ENSMUSG00000073876	Gm13305	predicted gene 13305 [Source:MGI Symbol;Acc:MG	0.4271
ENSMUSG00000037531	Mrpl47	mitochondrial ribosomal protein L47 [Source:MGI S	0.42714
ENSMUSG00000044177	Wfikkn2	WAP, follistatin/kazal, immunoglobulin, kunitz and	0.42728
ENSMUSG00000079481	Nhsl2	NHS-like 2 [Source:MGI Symbol;Acc:MGI:3645090]	0.42735
ENSMUSG00000028245	Nsmaf	neutral sphingomyelinase (N-SMase) activation ass	0.42743
ENSMUSG00000014846	Tppp3	tubulin polymerization-promoting protein family m	0.42746
ENSMUSG00000024008	Cpne5	copine V [Source:MGI Symbol;Acc:MGI:2385908]	0.42749
ENSMUSG00000025486	Sirt3	sirtuin 3 [Source:MGI Symbol;Acc:MGI:1927665]	0.42755
ENSMUSG00000025785	Exosc7	exosome component 7 [Source:MGI Symbol;Acc:M	0.42768
ENSMUSG00000032041	Tirap	toll-interleukin 1 receptor (TIR) domain-containing	0.42769
ENSMUSG00000039994	Timeless	timeless circadian clock 1 [Source:MGI Symbol;Acc:	0.42773
ENSMUSG00000043602	Zfp3	zinc finger protein 3 [Source:MGI Symbol;Acc:MGI:	0.42774
ENSMUSG00000038762	Abcf1	ATP-binding cassette, sub-family F (GCN20), memb	0.42779
ENSMUSG00000092905	Gm25735	predicted gene, 25735 [Source:MGI Symbol;Acc:M	0.42781
ENSMUSG00000086851	A430108G06Rik	RIKEN cDNA A430108G06 gene [Source:MGI Symbo	0.42789
ENSMUSG00000002365	Snx9	sorting nexin 9 [Source:MGI Symbol;Acc:MGI:1913	0.42792
ENSMUSG00000026418	Tnni1	troponin I, skeletal, slow 1 [Source:MGI Symbol;Ac	0.42792
ENSMUSG00000023572	Ccndbp1	cyclin D-type binding-protein 1 [Source:MGI Symbo	0.42794
ENSMUSG00000039231	Suv39h1	suppressor of variegation 3-9 homolog 1 (Drosophi	0.42799
ENSMUSG00000032258	Lca5	Leber congenital amaurosis 5 (human) [Source:MG	0.42808
ENSMUSG00000045867	Cradd	CASP2 and RIPK1 domain containing adaptor with d	0.42816
ENSMUSG00000081402	Gm15455	predicted gene 15455 [Source:MGI Symbol;Acc:MG	0.42822
ENSMUSG00000073682	Gm10563	predicted gene 10563 [Source:MGI Symbol;Acc:MG	0.42824
ENSMUSG00000025935	Tram1	translocating chain-associating membrane protein	0.42836
ENSMUSG00000035199	Arl6ip5	ADP-ribosylation factor-like 6 interacting protein 5	0.42845
ENSMUSG00000094627	Gm10182	predicted pseudogene 10182 [Source:MGI Symbol;	0.42845
ENSMUSG00000044576	Gareml	GRB2 associated, regulator of MAPK1-like [Source:I	0.42846
ENSMUSG00000040034	Nup43	nucleoporin 43 [Source:MGI Symbol;Acc:MGI:1917	0.42856
ENSMUSG00000032271	Nnmt	nicotinamide N-methyltransferase [Source:MGI Syr	0.42858
ENSMUSG00000036526	Card11	caspase recruitment domain family, member 11 [So	0.42863
ENSMUSG00000050071	Bex1	brain expressed gene 1 [Source:MGI Symbol;Acc:M	0.42874
ENSMUSG00000098004	Gm27027	predicted gene, 27027 [Source:MGI Symbol;Acc:M	0.42877

ENSMUSG00000021097	Clmn	calmin [Source:MGI Symbol;Acc:MGI:2136957]	0.42883
ENSMUSG00000026553	Copa	coatomer protein complex subunit alpha [Source:M	0.42889
ENSMUSG00000045636	Mtus1	mitochondrial tumor suppressor 1 [Source:MGI Syr	0.42892
ENSMUSG00000097285	Gm26683	predicted gene, 26683 [Source:MGI Symbol;Acc:MG	0.42896
ENSMUSG00000088219	Gm22077	predicted gene, 22077 [Source:MGI Symbol;Acc:MG	0.429
ENSMUSG00000072722	Gm6588	predicted gene 6588 [Source:MGI Symbol;Acc:MGI:	0.42904
ENSMUSG00000022221	Ripk3	receptor-interacting serine-threonine kinase 3 [Sou	0.42911
ENSMUSG00000035595	1600002K03Rik	RIKEN cDNA 1600002K03 gene [Source:MGI Symbo	0.42911
ENSMUSG00000020388	Pdlim4	PDZ and LIM domain 4 [Source:MGI Symbol;Acc:MG	0.42919
ENSMUSG00000010080	Epn3	epsin 3 [Source:MGI Symbol;Acc:MGI:1919139]	0.42931
ENSMUSG00000035184	Fam124a	family with sequence similarity 124, member A [Sou	0.42935
ENSMUSG00000039095	En2	engrailed 2 [Source:MGI Symbol;Acc:MGI:95390]	0.42937
ENSMUSG00000055809	Dnaaf3	dynein, axonemal assembly factor 3 [Source:MGI Sy	0.42941
ENSMUSG00000015095	Fbxw5	F-box and WD-40 domain protein 5 [Source:MGI Sy	0.42948
ENSMUSG00000021234	Fam161b	family with sequence similarity 161, member B [Sou	0.42954
ENSMUSG00000024274	Zscan30	zinc finger and SCAN domain containing 30 [Source	0.42958
ENSMUSG00000082090	Gm16481	predicted gene 16481 [Source:MGI Symbol;Acc:MG	0.42963
ENSMUSG00000036067	Slc2a6	solute carrier family 2 (facilitated glucose transport	0.42964
ENSMUSG00000067847	Romo1	reactive oxygen species modulator 1 [Source:MGI S	0.42967
ENSMUSG00000021103	Mnat1	menage a trois 1 [Source:MGI Symbol;Acc:MGI:106	0.42968
ENSMUSG00000074221	Zfp568	zinc finger protein 568 [Source:MGI Symbol;Acc:MG	0.42973
ENSMUSG00000057230	Aak1	AP2 associated kinase 1 [Source:MGI Symbol;Acc:M	0.42979
ENSMUSG00000021314	Amph	amphiphysin [Source:MGI Symbol;Acc:MGI:103574	0.42981
ENSMUSG00000097917	Gm26839	predicted gene, 26839 [Source:MGI Symbol;Acc:MG	0.42986
ENSMUSG00000015672	Mrpl32	mitochondrial ribosomal protein L32 [Source:MGI S	0.42992
ENSMUSG00000034509	Mad2l1bp	MAD2L1 binding protein [Source:MGI Symbol;Acc:M	0.42997
ENSMUSG00000068745	Mybphl	myosin binding protein H-like [Source:MGI Symbol;	0.43001
ENSMUSG00000085145	Gm11614	predicted gene 11614 [Source:MGI Symbol;Acc:MG	0.43019
ENSMUSG00000082653	Gm15897	predicted gene 15897 [Source:MGI Symbol;Acc:MG	0.43026
ENSMUSG00000031072	Oraov1	oral cancer overexpressed 1 [Source:MGI Symbol;A	0.43038
ENSMUSG00000022475	Hdac7	histone deacetylase 7 [Source:MGI Symbol;Acc:MG	0.4304
ENSMUSG00000053552	Ebf4	early B cell factor 4 [Source:MGI Symbol;Acc:MGI:2	0.43053
ENSMUSG00000019768	Esr1	estrogen receptor 1 (alpha) [Source:MGI Symbol;Ac	0.43057
ENSMUSG00000025791	Pgm2	phosphoglucomutase 2 [Source:MGI Symbol;Acc:M	0.43057
ENSMUSG00000029436	Mmp17	matrix metallopeptidase 17 [Source:MGI Symbol;Ac	0.43057
ENSMUSG00000064246	Chi3l1	chitinase 3-like 1 [Source:MGI Symbol;Acc:MGI:134	0.43058
ENSMUSG00000036646	Man1b1	mannosidase, alpha, class 1B, member 1 [Source:M	0.43068
ENSMUSG00000024617	Camk2a	calcium/calmodulin-dependent protein kinase II alp	0.4307
ENSMUSG00000085376	Gm14508	predicted gene 14508 [Source:MGI Symbol;Acc:MG	0.43073
ENSMUSG00000075558	Gm17151	predicted gene 17151 [Source:MGI Symbol;Acc:MG	0.4308
ENSMUSG00000035356	Nfkbiz	nuclear factor of kappa light polypeptide gene enha	0.43082
ENSMUSG00000074461	Gm10699	predicted gene 10699 [Source:MGI Symbol;Acc:MG	0.43083
ENSMUSG00000044155	Naa38	N(alpha)-acetyltransferase 38, NatC auxiliary subun	0.4309
ENSMUSG00000015093	Clic3	chloride intracellular channel 3 [Source:MGI Symbo	0.43092
ENSMUSG00000039450	Dcxr	dicarbonyl L-xylulose reductase [Source:MGI Symbo	0.43095
ENSMUSG00000067825	Pex26	peroxisomal biogenesis factor 26 [Source:MGI Sym	0.43095
ENSMUSG00000033196	Myh2	myosin, heavy polypeptide 2, skeletal muscle, adult	0.43102

ENSMUSG00000037852	Cpe	carboxypeptidase E [Source:MGI Symbol;Acc:MGI:1	0.43102
ENSMUSG00000040820	Hlcs	holocarboxylase synthetase (biotin- [propriony-Coe	0.43121
ENSMUSG00000034057	Myrfl	myelin regulatory factor-like [Source:MGI Symbol;A	0.43123
ENSMUSG00000055839	Tceb2	transcription elongation factor B (SIII), polypeptide	0.43123
ENSMUSG00000025278	Flnb	filamin, beta [Source:MGI Symbol;Acc:MGI:244608	0.43126
ENSMUSG00000079614	Seh1l	SEH1-like (S. cerevisiae [Source:MGI Symbol;Acc:M	0.43128
ENSMUSG00000084616	Gm25047	predicted gene, 25047 [Source:MGI Symbol;Acc:M	0.43136
ENSMUSG00000041341	Atg2b	autophagy related 2B [Source:MGI Symbol;Acc:MG	0.43139
ENSMUSG00000021575	Ahrr	aryl-hydrocarbon receptor repressor [Source:MGI S	0.43141
ENSMUSG00000040028	Elavl1	ELAV (embryonic lethal, abnormal vision)-like 1 (Hu	0.43149
ENSMUSG00000032915	Emr4	EGF-like module containing, mucin-like, hormone r	0.43159
ENSMUSG00000043298	Smco3	single-pass membrane protein with coiled-coil dom	0.43159
ENSMUSG00000053441	Adamts19	a disintegrin-like and metallopeptidase (reprolysin	0.4316
ENSMUSG00000022427	Tomm22	translocase of outer mitochondrial membrane 22 h	0.43163
ENSMUSG00000048486	Fitm2	fat storage-inducing transmembrane protein 2 [Sou	0.43165
ENSMUSG00000069911	Fam196b	family with sequence similarity 196, member B [Sou	0.43171
ENSMUSG00000027173	Depdc7	DEP domain containing 7 [Source:MGI Symbol;Acc:	0.43184
ENSMUSG00000053797	Krt16	keratin 16 [Source:MGI Symbol;Acc:MGI:96690]	0.43186
ENSMUSG00000044629	Cnrip1	cannabinoid receptor interacting protein 1 [Source:	0.43187
ENSMUSG00000020328	Nudcd2	NudC domain containing 2 [Source:MGI Symbol;Acc	0.43188
ENSMUSG00000023330	Dtwd1	DTW domain containing 1 [Source:MGI Symbol;Acc	0.43194
ENSMUSG00000026260	Ndufa10	NADH dehydrogenase (ubiquinone) 1 alpha subcom	0.43202
ENSMUSG00000097188	Snhg1	small nucleolar RNA host gene (non-protein coding	0.43206
ENSMUSG00000039662	Icmt	isoprenylcysteine carboxyl methyltransferase [Sour	0.43212
ENSMUSG00000068922	Msto1	misato homolog 1 (Drosophila) [Source:MGI Symbo	0.43216
ENSMUSG00000026355	Mcm6	minichromosome maintenance deficient 6 (MIS5 h	0.43225
ENSMUSG00000032518	Rpsa	ribosomal protein SA [Source:MGI Symbol;Acc:MGI	0.43236
ENSMUSG00000086236	5830418P13Rik	RIKEN cDNA 5830418P13 gene [Source:MGI Symbo	0.43236
ENSMUSG00000095352	Gm20783	predicted gene, 20783 [Source:MGI Symbol;Acc:M	0.43236
ENSMUSG00000040714	Klc3	kinesin light chain 3 [Source:MGI Symbol;Acc:MGI:1	0.4324
ENSMUSG00000031029	Eif3f	eukaryotic translation initiation factor 3, subunit F	0.43257
ENSMUSG00000062031	Athl1	ATH1, acid trehalase-like 1 (yeast) [Source:MGI Sym	0.43257
ENSMUSG00000097013	Gm26710	predicted gene, 26710 [Source:MGI Symbol;Acc:M	0.43258
ENSMUSG00000097456	Gm16958	predicted gene, 16958 [Source:MGI Symbol;Acc:M	0.43261
ENSMUSG00000018865	Sult4a1	sulfotransferase family 4A, member 1 [Source:MGI	0.43274
ENSMUSG00000008958	Vps72	vacuolar protein sorting 72 (yeast) [Source:MGI Syr	0.43288
ENSMUSG00000022772	Senp5	SUMO/sentrin specific peptidase 5 [Source:MGI Syr	0.43291
ENSMUSG00000058626	Capn11	calpain 11 [Source:MGI Symbol;Acc:MGI:1352490]	0.43293
ENSMUSG00000032698	Lmo2	LIM domain only 2 [Source:MGI Symbol;Acc:MGI:1	0.43295
ENSMUSG00000021838	Samd4	sterile alpha motif domain containing 4 [Source:M	0.43297
ENSMUSG00000067235	H2-Q10	histocompatibility 2, Q region locus 10 [Source:MG	0.43314
ENSMUSG00000086746	Gm15222	predicted gene 15222 [Source:MGI Symbol;Acc:MG	0.4332
ENSMUSG00000033998	Kcnk1	potassium channel, subfamily K, member 1 [Source	0.43337
ENSMUSG00000040447	Spns2	spinster homolog 2 [Source:MGI Symbol;Acc:MGI:2	0.43343
ENSMUSG00000029348	Asphd2	aspartate beta-hydroxylase domain containing 2 [S	0.43363
ENSMUSG00000018595	Glra4	glycine receptor, alpha 4 subunit [Source:MGI Sym	0.4337
ENSMUSG00000026602	Nphs2	nephrosis 2, podocin [Source:MGI Symbol;Acc:MGI	0.43373

ENSMUSG00000020744	Slc25a19	solute carrier family 25 (mitochondrial thiamine py	0.43387
ENSMUSG00000034910	Pygo1	pygopus 1 [Source:MGI Symbol;Acc:MGI:1919385]	0.43392
ENSMUSG00000029387	Gtf2h3	general transcription factor IIH, polypeptide 3 [Sou	0.43393
ENSMUSG00000025169	Ogfod3	2-oxoglutarate and iron-dependent oxygenase dom	0.43395
ENSMUSG00000094936	Rbm4	RNA binding motif protein 4 [Source:MGI Symbol;A	0.43428
ENSMUSG00000006378	Gcat	glycine C-acetyltransferase (2-amino-3-ketobutyrat	0.43431
ENSMUSG00000038831	Ralgps1	Ral GEF with PH domain and SH3 binding motif 1 [S	0.43431
ENSMUSG00000073471	Rsph3a	radial spoke 3A homolog (Chlamydomonas) [Source	0.43432
ENSMUSG00000042641	Rgsl1	regulator of G-protein signaling like 1 [Source:MGI	0.43437
ENSMUSG00000020462	Ccdc104	coiled-coil domain containing 104 [Source:MGI Sym	0.43443
ENSMUSG00000080666	Gm25000	predicted gene, 25000 [Source:MGI Symbol;Acc:MG	0.43444
ENSMUSG00000097322	A530083I20Rik	RIKEN cDNA A530083I20 gene [Source:MGI Symbol	0.43463
ENSMUSG00000032228	Tcf12	transcription factor 12 [Source:MGI Symbol;Acc:MG	0.43466
ENSMUSG00000020477	Mrps24	mitochondrial ribosomal protein S24 [Source:MGI S	0.43467
ENSMUSG00000074743	Thbd	thrombomodulin [Source:MGI Symbol;Acc:MGI:987	0.43469
ENSMUSG00000001062	Vps9d1	VPS9 domain containing 1 [Source:MGI Symbol;Acc	0.43482
ENSMUSG00000017774	Myo1c	myosin IC [Source:MGI Symbol;Acc:MGI:106612]	0.43484
ENSMUSG00000030905	Crym	crystallin, mu [Source:MGI Symbol;Acc:MGI:102675	0.43491
ENSMUSG00000097150	Gm26513	predicted gene, 26513 [Source:MGI Symbol;Acc:MG	0.43504
ENSMUSG00000027422	Rrbp1	ribosome binding protein 1 [Source:MGI Symbol;Ac	0.43509
ENSMUSG00000021417	Eci2	enoyl-Coenzyme A delta isomerase 2 [Source:MGI S	0.4351
ENSMUSG00000026283	Ing5	inhibitor of growth family, member 5 [Source:MGI S	0.43511
ENSMUSG00000018476	Kdm6b	KDM1 lysine (K)-specific demethylase 6B [Source:M	0.43513
ENSMUSG00000043207	Zmpste24	zinc metallopeptidase, STE24 [Source:MGI Symbol;	0.43515
ENSMUSG00000082515	Gm12902	predicted gene 12902 [Source:MGI Symbol;Acc:MG	0.43516
ENSMUSG00000022780	Mfi2	antigen p97 (melanoma associated) identified by m	0.43519
ENSMUSG00000026131	Dst	dystonin [Source:MGI Symbol;Acc:MGI:104627]	0.43519
ENSMUSG00000082651	Gm14001	predicted gene 14001 [Source:MGI Symbol;Acc:MG	0.43523
ENSMUSG00000097832	Gm26912	predicted gene, 26912 [Source:MGI Symbol;Acc:MG	0.43533
ENSMUSG00000092718	Gm22860	predicted gene, 22860 [Source:MGI Symbol;Acc:MG	0.43549
ENSMUSG00000039982	Dtx4	deltex 4 homolog (Drosophila) [Source:MGI Symbol	0.43564
ENSMUSG00000089759	3632454L22Rik	RIKEN cDNA 3632454L22 gene [Source:MGI Symbo	0.43566
ENSMUSG00000002475	Abhd3	abhydrolase domain containing 3 [Source:MGI Sym	0.4357
ENSMUSG00000022967	Ifnar1	interferon (alpha and beta) receptor 1 [Source:MGI	0.4357
ENSMUSG00000028475	5430416O09Rik	RIKEN cDNA 5430416O09 gene [Source:MGI Symbo	0.43576
ENSMUSG00000024683	Mrpl16	mitochondrial ribosomal protein L16 [Source:MGI S	0.4358
ENSMUSG00000046791	2410016O06Rik	RIKEN cDNA 2410016O06 gene [Source:MGI Symbo	0.43593
ENSMUSG00000073982	Rhog	ras homolog gene family, member G [Source:MGI S	0.436
ENSMUSG00000006527	Sfmbt1	Scm-like with four mbt domains 1 [Source:MGI Sym	0.43607
ENSMUSG00000027999	Pla2g12a	phospholipase A2, group XIIA [Source:MGI Symbol;	0.43613
ENSMUSG00000020921	Tmem101	transmembrane protein 101 [Source:MGI Symbol;A	0.43621
ENSMUSG00000090066	1110002E22Rik	RIKEN cDNA 1110002E22 gene [Source:MGI Symbo	0.43628
ENSMUSG00000030882	Dnhd1	dynein heavy chain domain 1 [Source:MGI Symbol;	0.4363
ENSMUSG00000018844	Fndc8	fibronectin type III domain containing 8 [Source:MG	0.43634
ENSMUSG00000051669	AU021092	expressed sequence AU021092 [Source:MGI Symbo	0.43636
ENSMUSG00000085760	Gm14302	predicted gene 14302 [Source:MGI Symbol;Acc:MG	0.43645
ENSMUSG00000025858	Get4	golgi to ER traffic protein 4 homolog (S. cerevisiae)	0.4365

ENSMUSG00000026975	Dph7	diphthamine biosynethesis 7 [Source:MGI Symbol;A	0.43658
ENSMUSG00000037280	Galnt6	UDP-N-acetyl-alpha-D-galactosamine:polypeptide N	0.43659
ENSMUSG00000033249	Hsf4	heat shock transcription factor 4 [Source:MGI Symb	0.43663
ENSMUSG00000002109	Ddb2	damage specific DNA binding protein 2 [Source:MG	0.4367
ENSMUSG00000021391	Cenpp	centromere protein P [Source:MGI Symbol;Acc:MG	0.4367
ENSMUSG00000049823	Zbtb12	zinc finger and BTB domain containing 12 [Source:M	0.43699
ENSMUSG00000075592	Nynrin	NYN domain and retroviral integrase containing [So	0.43702
ENSMUSG00000026208	Des	desmin [Source:MGI Symbol;Acc:MGI:94885]	0.43707
ENSMUSG00000053263	Gm12592	predicted gene 12592 [Source:MGI Symbol;Acc:MG	0.43713
ENSMUSG00000030145	Zfp248	zinc finger protein 248 [Source:MGI Symbol;Acc:MG	0.43717
ENSMUSG00000007279	Scube2	signal peptide, CUB domain, EGF-like 2 [Source:MG	0.43719
ENSMUSG00000023089	Ndufa5	NADH dehydrogenase (ubiquinone) 1 alpha subcom	0.43738
ENSMUSG00000074136	4930513N10Rik	RIKEN cDNA 4930513N10 gene [Source:MGI Symbo	0.43745
ENSMUSG00000024193	Phf1	PHD finger protein 1 [Source:MGI Symbol;Acc:MGI:	0.43746
ENSMUSG00000070520	Ndnl2	necdin-like 2 [Source:MGI Symbol;Acc:MGI:191389	0.43752
ENSMUSG00000017446	C1qtnf1	C1q and tumor necrosis factor related protein 1 [So	0.43753
ENSMUSG00000025475	Gpr123	G protein-coupled receptor 123 [Source:MGI Symb	0.43759
ENSMUSG00000048826	Dact2	dapper homolog 2, antagonist of beta-catenin (xen	0.43764
ENSMUSG00000022476	Polr3h	polymerase (RNA) III (DNA directed) polypeptide H	0.43776
ENSMUSG00000037366	Pafah2	platelet-activating factor acetylhydrolase 2 [Source	0.43782
ENSMUSG00000020802	Ube2o	ubiquitin-conjugating enzyme E2O [Source:MGI Syr	0.43788
ENSMUSG00000032403	2300009A05Rik	RIKEN cDNA 2300009A05 gene [Source:MGI Symbo	0.43794
ENSMUSG00000014177	Tvp23b	trans-golgi network vesicle protein 23B [Source:MG	0.43808
ENSMUSG00000024925	Rnaseh2c	ribonuclease H2, subunit C [Source:MGI Symbol;Ac	0.4381
ENSMUSG00000003500	Impdh1	inosine 5'-phosphate dehydrogenase 1 [Source:MG	0.43823
ENSMUSG00000043671	Dpy19l3	dpy-19-like 3 (C. elegans) [Source:MGI Symbol;Acc:	0.43828
ENSMUSG00000029716	Tfr2	transferrin receptor 2 [Source:MGI Symbol;Acc:MG	0.43832
ENSMUSG00000061322	Dnaic1	dynein, axonemal, intermediate chain 1 [Source:MG	0.43838
ENSMUSG00000097860	Gm26857	predicted gene, 26857 [Source:MGI Symbol;Acc:MG	0.43843
ENSMUSG00000048644	Ctxn1	cortexin 1 [Source:MGI Symbol;Acc:MGI:88566]	0.43845
ENSMUSG00000022469	Rapgef3	Rap guanine nucleotide exchange factor (GEF) 3 [So	0.43847
ENSMUSG00000089669	Tnfsf13	tumor necrosis factor (ligand) superfamily, membe	0.43847
ENSMUSG00000045038	Prkce	protein kinase C, epsilon [Source:MGI Symbol;Acc:M	0.43854
ENSMUSG00000045672	Col27a1	collagen, type XXVII, alpha 1 [Source:MGI Symbol;A	0.43861
ENSMUSG00000026154	1110058L19Rik	RIKEN cDNA 1110058L19 gene [Source:MGI Symbo	0.43862
ENSMUSG00000034040	Wbscr17	Williams-Beuren syndrome chromosome region 17	0.43864
ENSMUSG00000043122	A530016L24Rik	RIKEN cDNA A530016L24 gene [Source:MGI Symbo	0.43865
ENSMUSG00000026860	Sh3glb2	SH3-domain GRB2-like endophilin B2 [Source:MGI S	0.43867
ENSMUSG00000050164	Mchr1	melanin-concentrating hormone receptor 1 [Source	0.43875
ENSMUSG00000027552	E2f5	E2F transcription factor 5 [Source:MGI Symbol;Acc:	0.43889
ENSMUSG00000052062	Pard3b	par-3 partitioning defective 3 homolog B (C. elegan	0.4389
ENSMUSG00000097330	Gm26672	predicted gene, 26672 [Source:MGI Symbol;Acc:MG	0.4389
ENSMUSG00000005893	Nr2c2	nuclear receptor subfamily 2, group C, member 2 [S	0.43893
ENSMUSG00000079608	Stard6	StAR-related lipid transfer (START) domain containi	0.43897
ENSMUSG00000061288	Taok3	TAO kinase 3 [Source:MGI Symbol;Acc:MGI:304117	0.439
ENSMUSG00000022723	Crybg3	beta-gamma crystallin domain containing 3 [Source	0.43901
ENSMUSG00000026728	Vim	vimentin [Source:MGI Symbol;Acc:MGI:98932]	0.43903

ENSMUSG00000074039	4930520O04Rik	RIKEN cDNA 4930520O04 gene [Source:MGI Symbo	0.43908
ENSMUSG00000052403	Gm13520	predicted gene 13520 [Source:MGI Symbol;Acc:MG	0.43924
ENSMUSG00000092988	Mir3093	microRNA 3093 [Source:MGI Symbol;Acc:MGI:4834	0.43932
ENSMUSG00000056708	Ier5	immediate early response 5 [Source:MGI Symbol;A	0.43936
ENSMUSG00000031844	Hsd17b2	hydroxysteroid (17-beta) dehydrogenase 2 [Source	0.43946
ENSMUSG00000031068	Glrx3	glutaredoxin 3 [Source:MGI Symbol;Acc:MGI:13536	0.43948
ENSMUSG00000048232	Fbxo10	F-box protein 10 [Source:MGI Symbol;Acc:MGI:268	0.43948
ENSMUSG00000032536	Trak1	trafficking protein, kinesin binding 1 [Source:MGI S	0.43958
ENSMUSG00000022265	Ank	progressive ankylosis [Source:MGI Symbol;Acc:MGI	0.43961
ENSMUSG00000050370	Ch25h	cholesterol 25-hydroxylase [Source:MGI Symbol;Ac	0.43961
ENSMUSG00000069270	Hist1h2ac	histone cluster 1, H2ac [Source:MGI Symbol;Acc:M	0.43975
ENSMUSG00000078853	Igtp	interferon gamma induced GTPase [Source:MGI Sy	0.43994
ENSMUSG00000070697	Utp3	UTP3, small subunit (SSU) processome component,	0.43996
ENSMUSG00000041298	Katnal1	katanin p60 subunit A-like 1 [Source:MGI Symbol;A	0.43997
ENSMUSG00000085830	E130003G02Rik	RIKEN cDNA E130003G02 gene [Source:MGI Symbo	0.43999
ENSMUSG00000046753	Ccdc66	coiled-coil domain containing 66 [Source:MGI Symb	0.44011
ENSMUSG00000063760	Rnf217	ring finger protein 217 [Source:MGI Symbol;Acc:M	0.44015
ENSMUSG00000031896	Ctrl	chymotrypsin-like [Source:MGI Symbol;Acc:MGI:88	0.44017
ENSMUSG00000039055	Eme1	essential meiotic endonuclease 1 homolog 1 (S. po	0.44032
ENSMUSG00000015542	Nat9	N-acetyltransferase 9 (GCN5-related, putative) [Sou	0.44038
ENSMUSG00000045822	Zswim3	zinc finger SWIM-type containing 3 [Source:MGI Sy	0.44038
ENSMUSG00000026574	Dpt	dermatopontin [Source:MGI Symbol;Acc:MGI:1928	0.4405
ENSMUSG00000004892	Bcan	brevican [Source:MGI Symbol;Acc:MGI:1096385]	0.44058
ENSMUSG00000032864	Rag2	recombination activating gene 2 [Source:MGI Symb	0.4406
ENSMUSG00000041895	Wipi1	WD repeat domain, phosphoinositide interacting 1	0.44074
ENSMUSG00000054400	Cklf	chemokine-like factor [Source:MGI Symbol;Acc:MG	0.4408
ENSMUSG00000076431	Sox4	SRY-box containing gene 4 [Source:MGI Symbol;Ac	0.44112
ENSMUSG00000085067	Gm15631	predicted gene 15631 [Source:MGI Symbol;Acc:MG	0.44112
ENSMUSG00000090733	Rps27	ribosomal protein S27 [Source:MGI Symbol;Acc:MG	0.44114
ENSMUSG00000041471	Fam35a	family with sequence similarity 35, member A [Sou	0.44158
ENSMUSG00000025878	Uimc1	ubiquitin interaction motif containing 1 [Source:M	0.4416
ENSMUSG00000031583	Wrn	Werner syndrome homolog (human) [Source:MGI S	0.44161
ENSMUSG00000078240	Gm3550	predicted gene 3550 [Source:MGI Symbol;Acc:MGI	0.44162
ENSMUSG00000068335	Dok1	docking protein 1 [Source:MGI Symbol;Acc:MGI:89	0.44171
ENSMUSG00000021125	Arg2	arginase type II [Source:MGI Symbol;Acc:MGI:1330	0.44191
ENSMUSG00000041362	4930506M07Rik	RIKEN cDNA 4930506M07 gene [Source:MGI Symb	0.44196
ENSMUSG00000041073	Nacad	NAC alpha domain containing [Source:MGI Symbol;	0.44209
ENSMUSG00000028961	Pgd	phosphogluconate dehydrogenase [Source:MGI Sy	0.44212
ENSMUSG00000086004	Gm7805	predicted gene 7805 [Source:MGI Symbol;Acc:MGI	0.44219
ENSMUSG00000018239	Zcchc10	zinc finger, CCHC domain containing 10 [Source:M	0.44221
ENSMUSG00000030611	Mrps11	mitochondrial ribosomal protein S11 [Source:MGI S	0.4423
ENSMUSG00000097119	B230354K17Rik	RIKEN cDNA B230354K17 gene [Source:MGI Symbo	0.4423
ENSMUSG00000079168	Cd209g	CD209g antigen [Source:MGI Symbol;Acc:MGI:1917	0.44232
ENSMUSG00000087029	Gm14133	predicted gene 14133 [Source:MGI Symbol;Acc:MG	0.44233
ENSMUSG00000031365	Zfp275	zinc finger protein 275 [Source:MGI Symbol;Acc:M	0.44236
ENSMUSG00000079434	Neu2	neuraminidase 2 [Source:MGI Symbol;Acc:MGI:134	0.44238
ENSMUSG00000027776	Il12a	interleukin 12a [Source:MGI Symbol;Acc:MGI:9653	0.4425

ENSMUSG00000019823	Mical1	microtubule associated monooxygenase, calponin a	0.44281
ENSMUSG00000005161	Prdx2	peroxiredoxin 2 [Source:MGI Symbol;Acc:MGI:1094	0.44288
ENSMUSG00000085182	Gm13596	predicted gene 13596 [Source:MGI Symbol;Acc:MG	0.44296
ENSMUSG00000028461	Ccdc107	coiled-coil domain containing 107 [Source:MGI Sym	0.44301
ENSMUSG00000033147	Slc22a15	solute carrier family 22 (organic anion/cation trans	0.44303
ENSMUSG00000038150	Ormdl3	ORM1-like 3 (S. cerevisiae) [Source:MGI Symbol;Ac	0.44315
ENSMUSG00000000782	Tcf7	transcription factor 7, T cell specific [Source:MGI Sy	0.44317
ENSMUSG00000034453	Polr3b	polymerase (RNA) III (DNA directed) polypeptide B	0.4432
ENSMUSG00000022516	Nudt16l1	nudix (nucleoside diphosphate linked moiety X)-typ	0.44333
ENSMUSG00000087381	Gm16008	predicted gene 16008 [Source:MGI Symbol;Acc:MG	0.44333
ENSMUSG00000073568	Arl14epl	ADP-ribosylation factor-like 14 effector protein-like	0.44338
ENSMUSG00000069041	Slc25a31	solute carrier family 25 (mitochondrial carrier; aden	0.4434
ENSMUSG00000049939	Lrrc4	leucine rich repeat containing 4 [Source:MGI Symb	0.44341
ENSMUSG00000085763	2700081L22Rik	RIKEN cDNA 2700081L22 gene [Source:MGI Symbo	0.44341
ENSMUSG00000015357	Clpx	caseinolytic peptidase X (E.coli) [Source:MGI Symbo	0.44343
ENSMUSG00000080823	Gm11477	predicted gene 11477 [Source:MGI Symbol;Acc:MG	0.44344
ENSMUSG00000042726	Trafd1	TRAF type zinc finger domain containing 1 [Source:	0.44347
ENSMUSG00000079709	Gm3435	predicted gene 3435 [Source:MGI Symbol;Acc:MGI:	0.44347
ENSMUSG00000074527	Gm14296	predicted gene 14296 [Source:MGI Symbol;Acc:MG	0.44354
ENSMUSG00000087382	1300015D01Rik	RIKEN cDNA 1300015D01 gene [Source:MGI Symbo	0.44362
ENSMUSG00000028309	Rnf20	ring finger protein 20 [Source:MGI Symbol;Acc:MGI	0.4437
ENSMUSG00000023915	Tnfrsf21	tumor necrosis factor receptor superfamily, membe	0.44372
ENSMUSG00000085762	4930443B20Rik	RIKEN cDNA 4930443B20 gene [Source:MGI Symbo	0.44376
ENSMUSG00000082455	Gm13665	predicted gene 13665 [Source:MGI Symbol;Acc:MG	0.44378
ENSMUSG00000094690	1600014C23Rik	RIKEN cDNA 1600014C23 gene [Source:MGI Symbo	0.44379
ENSMUSG00000085528	4930401O10Rik	RIKEN cDNA 4930401O10 gene [Source:MGI Symbo	0.4438
ENSMUSG00000028696	Ipp	IAP promoted placental gene [Source:MGI Symbol;	0.44382
ENSMUSG00000018362	Kpna2	karyopherin (importin) alpha 2 [Source:MGI Symbo	0.44384
ENSMUSG00000069895	Atxn1l	ataxin 1-like [Source:MGI Symbol;Acc:MGI:3694797	0.44396
ENSMUSG00000053218	Gm9900	predicted gene 9900 [Source:MGI Symbol;Acc:MGI:	0.44402
ENSMUSG00000083899	Gm12346	predicted gene 12346 [Source:MGI Symbol;Acc:MG	0.44406
ENSMUSG00000088290	Gm26413	predicted gene, 26413 [Source:MGI Symbol;Acc:MG	0.44409
ENSMUSG00000025357	Dgka	diacylglycerol kinase, alpha [Source:MGI Symbol;Ac	0.44413
ENSMUSG00000021880	Rnase6	ribonuclease, RNase A family, 6 [Source:MGI Symbo	0.44425
ENSMUSG00000085881	Gm15912	predicted gene 15912 [Source:MGI Symbol;Acc:MG	0.4443
ENSMUSG00000020250	Txnrd1	thioredoxin reductase 1 [Source:MGI Symbol;Acc:M	0.44432
ENSMUSG00000033159	Cnppd1	cyclin Pas1/PHO80 domain containing 1 [Source:MG	0.44443
ENSMUSG00000028751	Pla2g2e	phospholipase A2, group IIE [Source:MGI Symbol;A	0.44447
ENSMUSG00000020758	Itgb4	integrin beta 4 [Source:MGI Symbol;Acc:MGI:96613	0.44448
ENSMUSG00000070871	Ccnyl1	cyclin Y-like 1 [Source:MGI Symbol;Acc:MGI:213861	0.44455
ENSMUSG00000038552	Fndc4	fibronectin type III domain containing 4 [Source:MG	0.44462
ENSMUSG00000048148	Nwd1	NACHT and WD repeat domain containing 1 [Source	0.44464
ENSMUSG00000001227	Sema6b	sema domain, transmembrane domain (TM), and cy	0.44467
ENSMUSG00000059309	Hist1h3f	histone cluster 1, H3f [Source:MGI Symbol;Acc:MG	0.44468
ENSMUSG00000026942	Traf2	TNF receptor-associated factor 2 [Source:MGI Symb	0.44476
ENSMUSG00000028786	Tmem54	transmembrane protein 54 [Source:MGI Symbol;Ac	0.4448
ENSMUSG00000043621	Ubxn10	UBX domain protein 10 [Source:MGI Symbol;Acc:M	0.44486

ENSMUSG00000094959	Gm24112	predicted gene, 24112 [Source:MGI Symbol;Acc:MG	0.44497
ENSMUSG00000093164	Gm22993	predicted gene, 22993 [Source:MGI Symbol;Acc:MG	0.44498
ENSMUSG00000071671	Gm10343	predicted gene 10343 [Source:MGI Symbol;Acc:MG	0.445
ENSMUSG00000022686	B3gnt5	UDP-GlcNAc:betaGal beta-1,3-N-acetylglucosaminy	0.44501
ENSMUSG00000042800	1700015E13Rik	RIKEN cDNA 1700015E13 gene [Source:MGI Symbo	0.44501
ENSMUSG00000051251	Nhlh1	nescient helix loop helix 1 [Source:MGI Symbol;Acc	0.44515
ENSMUSG00000037493	Cib2	calcium and integrin binding family member 2 [Sou	0.44534
ENSMUSG00000019737	Syne4	spectrin repeat containing, nuclear envelope family	0.4454
ENSMUSG00000038119	Cdon	cell adhesion molecule-related/down-regulated by	0.44546
ENSMUSG00000031889	D230025D16Rik	RIKEN cDNA D230025D16 gene [Source:MGI Symbo	0.44571
ENSMUSG00000066842	Hmcn1	hemicentin 1 [Source:MGI Symbol;Acc:MGI:268504	0.4458
ENSMUSG00000070461	9230112E08Rik	RIKEN cDNA 9230112E08 gene [Source:MGI Symbo	0.44581
ENSMUSG00000025420	Katnal2	katanin p60 subunit A-like 2 [Source:MGI Symbol;A	0.44586
ENSMUSG00000037656	Slc20a2	solute carrier family 20, member 2 [Source:MGI Syr	0.4459
ENSMUSG00000045174	Amer3	APC membrane recruitment 3 [Source:MGI Symbol	0.44593
ENSMUSG00000034842	Art3	ADP-ribosyltransferase 3 [Source:MGI Symbol;Acc:I	0.44605
ENSMUSG00000034532	Fbxo16	F-box protein 16 [Source:MGI Symbol;Acc:MGI:135	0.4461
ENSMUSG00000044134	Fam109a	family with sequence similarity 109, member A [Sou	0.44619
ENSMUSG00000097345	AA543186	expressed sequence AA543186 [Source:MGI Symbo	0.44622
ENSMUSG00000035919	Bbs9	Bardet-Biedl syndrome 9 (human) [Source:MGI Sym	0.44623
ENSMUSG00000044876	Zfp444	zinc finger protein 444 [Source:MGI Symbol;Acc:MG	0.44623
ENSMUSG00000010122	Slc47a1	solute carrier family 47, member 1 [Source:MGI Syr	0.44628
ENSMUSG00000053730	Tmem39b	transmembrane protein 39b [Source:MGI Symbol;A	0.44628
ENSMUSG00000001524	Gtf2h4	general transcription factor II H, polypeptide 4 [Sou	0.44646
ENSMUSG00000025779	Ly96	lymphocyte antigen 96 [Source:MGI Symbol;Acc:M	0.44651
ENSMUSG00000093908	Gm5784	predicted gene 5784 [Source:MGI Symbol;Acc:MGI	0.44651
ENSMUSG00000024302	Dtna	dystrobrevin alpha [Source:MGI Symbol;Acc:MGI:1	0.44652
ENSMUSG00000067795	4930444P10Rik	RIKEN cDNA 4930444P10 gene [Source:MGI Symbo	0.44675
ENSMUSG00000026227	2810459M11Rik	RIKEN cDNA 2810459M11 gene [Source:MGI Symbo	0.44705
ENSMUSG00000026705	Klhl20	kelch-like 20 [Source:MGI Symbol;Acc:MGI:244485	0.44725
ENSMUSG00000074886	Grk6	G protein-coupled receptor kinase 6 [Source:MGI S	0.44726
ENSMUSG00000093345	Gm26112	predicted gene, 26112 [Source:MGI Symbol;Acc:MG	0.44734
ENSMUSG00000085240	Gm12119	predicted gene 12119 [Source:MGI Symbol;Acc:MG	0.44735
ENSMUSG00000033767	D930015E06Rik	RIKEN cDNA D930015E06 gene [Source:MGI Symbo	0.44736
ENSMUSG00000014767	Tbp	TATA box binding protein [Source:MGI Symbol;Acc:	0.44754
ENSMUSG00000076617	Ighm	immunoglobulin heavy constant mu [Source:MGI S	0.44757
ENSMUSG00000053835	H2-T24	histocompatibility 2, T region locus 24 [Source:MGI	0.4476
ENSMUSG00000056919	4922501C03Rik	RIKEN cDNA 4922501C03 gene [Source:MGI Symbo	0.4476
ENSMUSG00000018401	Mtmr4	myotubularin related protein 4 [Source:MGI Symbo	0.44766
ENSMUSG00000064177	Ghrl	ghrelin [Source:MGI Symbol;Acc:MGI:1930008]	0.44773
ENSMUSG00000034216	Vps18	vacuolar protein sorting 18 (yeast) [Source:MGI Syr	0.44784
ENSMUSG00000023279	Bmp15	bone morphogenetic protein 15 [Source:MGI Symb	0.44786
ENSMUSG00000041143	Tmco4	transmembrane and coiled-coil domains 4 [Source:	0.44792
ENSMUSG00000089706	B230216N24Rik	RIKEN cDNA B230216N24 gene [Source:MGI Symbo	0.44792
ENSMUSG00000032815	Fanca	Fanconi anemia, complementation group A [Source	0.44796
ENSMUSG00000063751	Gm13939	predicted gene 13939 [Source:MGI Symbol;Acc:MG	0.44805
ENSMUSG00000042821	Snai1	snail homolog 1 (Drosophila) [Source:MGI Symbol;A	0.44811

ENSMUSG00000043259	Fam13c	family with sequence similarity 13, member C [Sour	0.44811
ENSMUSG00000056209	Npm3	nucleoplasmin 3 [Source:MGI Symbol;Acc:MGI:894	0.44811
ENSMUSG00000050936	Hist2h2bb	histone cluster 2, H2bb [Source:MGI Symbol;Acc:M	0.4482
ENSMUSG00000030217	Art4	ADP-ribosyltransferase 4 [Source:MGI Symbol;Acc:I	0.44827
ENSMUSG00000020657	Dnajc27	DnaJ (Hsp40) homolog, subfamily C, member 27 [So	0.44836
ENSMUSG00000075478	Slitrk1	SLIT and NTRK-like family, member 1 [Source:MGI S	0.4484
ENSMUSG00000028834	Trim63	tripartite motif-containing 63 [Source:MGI Symbol;	0.44861
ENSMUSG00000048170	Mcmbp	MCM (minichromosome maintenance deficient) bi	0.44865
ENSMUSG00000003429	Rps11	ribosomal protein S11 [Source:MGI Symbol;Acc:MG	0.44882
ENSMUSG00000082746	Rps12-ps1	ribosomal protein S12, pseudogene 1 [Source:MGI	0.44886
ENSMUSG00000062545	Tlr12	toll-like receptor 12 [Source:MGI Symbol;Acc:MGI:3	0.44888
ENSMUSG00000091754	Gm3636	predicted gene 3636 [Source:MGI Symbol;Acc:MGI:	0.44888
ENSMUSG00000020042	Btbd11	BTB (POZ) domain containing 11 [Source:MGI Symb	0.4489
ENSMUSG00000022185	Acin1	apoptotic chromatin condensation inducer 1 [Sourc	0.4489
ENSMUSG00000030074	Gxylt2	glucoside xylosyltransferase 2 [Source:MGI Symbol;	0.4489
ENSMUSG00000097080	1700086O06Rik	RIKEN cDNA 1700086O06 gene [Source:MGI Symbo	0.44892
ENSMUSG00000008435	Rdh13	retinol dehydrogenase 13 (all-trans and 9-cis) [Sour	0.44896
ENSMUSG00000031671	Setd6	SET domain containing 6 [Source:MGI Symbol;Acc:M	0.44907
ENSMUSG00000044022	Pcdhb21	protocadherin beta 21 [Source:MGI Symbol;Acc:MG	0.44907
ENSMUSG00000065979	Cpped1	calcineurin-like phosphoesterase domain containin	0.4492
ENSMUSG00000021743	Fezf2	Fez family zinc finger 2 [Source:MGI Symbol;Acc:MG	0.44924
ENSMUSG00000092097	Gm5819	predicted gene 5819 [Source:MGI Symbol;Acc:MGI:	0.44932
ENSMUSG00000017119	Nbr1	neighbor of Brca1 gene 1 [Source:MGI Symbol;Acc:	0.44948
ENSMUSG00000067879	3110035E14Rik	RIKEN cDNA 3110035E14 gene [Source:MGI Symbo	0.4495
ENSMUSG00000005125	Ndrg1	N-myc downstream regulated gene 1 [Source:MGI S	0.44951
ENSMUSG00000026918	Brd3	bromodomain containing 3 [Source:MGI Symbol;Ac	0.44955
ENSMUSG00000097728	Gm26840	predicted gene, 26840 [Source:MGI Symbol;Acc:MG	0.44957
ENSMUSG00000092470	Gm20518	predicted gene 20518 [Source:MGI Symbol;Acc:MG	0.44961
ENSMUSG00000004018	Fancl	Fanconi anemia, complementation group L [Source	0.44971
ENSMUSG00000097048	1600020E01Rik	RIKEN cDNA 1600020E01 gene [Source:MGI Symbo	0.44974
ENSMUSG00000039804	Ncoa5	nuclear receptor coactivator 5 [Source:MGI Symbol	0.44981
ENSMUSG00000033068	Entpd6	ectonucleoside triphosphate diphosphohydrolase 6	0.44982
ENSMUSG00000097711	Gm5523	predicted pseudogene 5523 [Source:MGI Symbol;A	0.44982
ENSMUSG00000022595	Lypd2	Ly6/Plaur domain containing 2 [Source:MGI Symbo	0.4499
ENSMUSG00000097019	Gm26663	predicted gene, 26663 [Source:MGI Symbol;Acc:MG	0.44992
ENSMUSG00000014418	Hps5	Hermansky-Pudlak syndrome 5 homolog (human) [	0.44997
ENSMUSG00000002064	Sdf2	stromal cell derived factor 2 [Source:MGI Symbol;A	0.45002
ENSMUSG00000040945	Rcc2	regulator of chromosome condensation 2 [Source:M	0.45003
ENSMUSG00000029106	Add1	adducin 1 (alpha) [Source:MGI Symbol;Acc:MGI:879	0.45005
ENSMUSG00000074140	Zfp319	zinc finger protein 319 [Source:MGI Symbol;Acc:MG	0.45039
ENSMUSG00000031302	Nlgn3	neuroligin 3 [Source:MGI Symbol;Acc:MGI:2444609	0.4504
ENSMUSG00000088069	Gm23250	predicted gene, 23250 [Source:MGI Symbol;Acc:MG	0.45045
ENSMUSG00000025176	Hoga1	4-hydroxy-2-oxoglutarate aldolase 1 [Source:MGI S	0.45051
ENSMUSG00000032178	Ilf3	interleukin enhancer binding factor 3 [Source:MGI S	0.45052
ENSMUSG00000044359	P2ry4	pyrimidinergic receptor P2Y, G-protein coupled, 4 [	0.45054
ENSMUSG00000030681	Mvp	major vault protein [Source:MGI Symbol;Acc:MGI:1	0.45055
ENSMUSG00000026790	Odf2	outer dense fiber of sperm tails 2 [Source:MGI Sym	0.45057

ENSMUSG00000051065	Mb21d2	Mab-21 domain containing 2 [Source:MGI Symbol;A	0.45062
ENSMUSG00000054277	Arfgap3	ADP-ribosylation factor GTPase activating protein 3	0.45073
ENSMUSG00000075272	Ttc30a2	tetratricopeptide repeat domain 30A2 [Source:MGI	0.45083
ENSMUSG00000046811	Gltpd2	glycolipid transfer protein domain containing 2 [Sou	0.45093
ENSMUSG00000082424	Gm13292	predicted gene 13292 [Source:MGI Symbol;Acc:MG	0.45095
ENSMUSG00000052087	Rgs14	regulator of G-protein signaling 14 [Source:MGI Syr	0.45097
ENSMUSG00000054708	Ankrd24	ankyrin repeat domain 24 [Source:MGI Symbol;Acc	0.45099
ENSMUSG00000020781	Tsen54	tRNA splicing endonuclease 54 homolog (S. cerevisi	0.45104
ENSMUSG00000025265	Fgd1	FYVE, RhoGEF and PH domain containing 1 [Source	0.45105
ENSMUSG00000019932	Kera	keratocan [Source:MGI Symbol;Acc:MGI:1202398]	0.45108
ENSMUSG00000050608	Minos1	mitochondrial inner membrane organizing system 1	0.4511
ENSMUSG00000028706	Nsun4	NOL1/NOP2/Sun domain family, member 4 [Source	0.45113
ENSMUSG00000039154	Shd	src homology 2 domain-containing transforming pr	0.45114
ENSMUSG00000034918	Cdhr2	cadherin-related family member 2 [Source:MGI Syr	0.45115
ENSMUSG00000085786	Gm15987	predicted gene 15987 [Source:MGI Symbol;Acc:MG	0.45116
ENSMUSG00000026280	Atg4b	autophagy related 4B, cysteine peptidase [Source:M	0.45117
ENSMUSG00000034401	Spata6	spermatogenesis associated 6 [Source:MGI Symbol	0.45119
ENSMUSG00000032579	Hemk1	HemK methyltransferase family member 1 [Source:	0.45125
ENSMUSG00000021418	Rpp40	ribonuclease P 40 subunit [Source:MGI Symbol;Acc	0.45141
ENSMUSG00000035824	Tk2	thymidine kinase 2, mitochondrial [Source:MGI Syn	0.45155
ENSMUSG00000011632	Pinlyp	phospholipase A2 inhibitor and LY6/PLAUR domain	0.45156
ENSMUSG00000040188	Scamp2	secretory carrier membrane protein 2 [Source:MGI	0.45158
ENSMUSG00000024485	Slc4a9	solute carrier family 4, sodium bicarbonate cotrans	0.45162
ENSMUSG00000035262	Amh	anti-Mullerian hormone [Source:MGI Symbol;Acc:M	0.45162
ENSMUSG00000021763	BC067074	cDNA sequence BC067074 [Source:MGI Symbol;Acc	0.45168
ENSMUSG00000001998	Ap4e1	adaptor-related protein complex AP-4, epsilon 1 [Sc	0.45172
ENSMUSG00000041220	Elovl6	ELOVL family member 6, elongation of long chain fa	0.45174
ENSMUSG00000026047	Kdelc1	KDEL (Lys-Asp-Glu-Leu) containing 1 [Source:MGI S	0.45175
ENSMUSG00000097015	Gm26705	predicted gene, 26705 [Source:MGI Symbol;Acc:MC	0.45175
ENSMUSG00000085088	4931413K12Rik	RIKEN cDNA 4931413K12 gene [Source:MGI Symbo	0.45207
ENSMUSG00000066797	Zfp648	zinc finger protein 648 [Source:MGI Symbol;Acc:MC	0.4521
ENSMUSG00000029713	Gnb2	guanine nucleotide binding protein (G protein), bet	0.45214
ENSMUSG00000093460	Six3os1	Six3 opposite strand transcript 1 [Source:MGI Symb	0.45215
ENSMUSG00000028771	Ptpn12	protein tyrosine phosphatase, non-receptor type 12	0.45217
ENSMUSG00000036770	4933433C11Rik	RIKEN cDNA 4933433C11 gene [Source:MGI Symbo	0.45217
ENSMUSG00000024978	Gpam	glycerol-3-phosphate acyltransferase, mitochondria	0.45225
ENSMUSG00000072258	Taf1a	TATA box binding protein (Tbp)-associated factor, R	0.45227
ENSMUSG00000052301	Doc2a	double C2, alpha [Source:MGI Symbol;Acc:MGI:109	0.45231
ENSMUSG00000001642	Akr1b3	aldo-keto reductase family 1, member B3 (aldose re	0.45232
ENSMUSG00000097722	Gm26841	predicted gene, 26841 [Source:MGI Symbol;Acc:MC	0.45242
ENSMUSG00000047787	Flrt1	fibronectin leucine rich transmembrane protein 1 [S	0.45263
ENSMUSG00000037600	1810019J16Rik	RIKEN cDNA 1810019J16 gene [Source:MGI Symbol	0.45269
ENSMUSG00000096968	Gm26620	predicted gene, 26620 [Source:MGI Symbol;Acc:MC	0.45286
ENSMUSG00000034452	Slc24a1	solute carrier family 24 (sodium/potassium/calcium	0.45287
ENSMUSG00000006715	Gmnn	geminin [Source:MGI Symbol;Acc:MGI:1927344]	0.45302
ENSMUSG00000015671	Psma2	proteasome (prosome, macropain) subunit, alpha t	0.4531
ENSMUSG00000085936	2610307P16Rik	RIKEN cDNA 2610307P16 gene [Source:MGI Symbo	0.4531

ENSMUSG00000028331	5830415F09Rik	RIKEN cDNA 5830415F09 gene [Source:MGI Symbo	0.45316
ENSMUSG00000041477	Dcp1b	DCP1 decapping enzyme homolog B (S. cerevisiae)	0.45327
ENSMUSG00000081248	Gm13787	predicted gene 13787 [Source:MGI Symbol;Acc:MG	0.45329
ENSMUSG00000000223	Drp2	dystrophin related protein 2 [Source:MGI Symbol;A	0.45349
ENSMUSG00000029419	Gm996	predicted gene 996 [Source:MGI Symbol;Acc:MGI:2	0.45349
ENSMUSG00000035936	Aldh5a1	aldhehyde dehydrogenase family 5, subfamily A1 [S	0.45354
ENSMUSG00000084785	Gm13972	predicted gene 13972 [Source:MGI Symbol;Acc:MG	0.45356
ENSMUSG00000058571	Gpc6	glypican 6 [Source:MGI Symbol;Acc:MGI:1346322]	0.45358
ENSMUSG00000087077	Gm12480	predicted gene 12480 [Source:MGI Symbol;Acc:MG	0.45405
ENSMUSG00000041199	Rpusd1	RNA pseudouridylate synthase domain containing 1	0.45421
ENSMUSG00000050821	Fam131a	family with sequence similarity 131, member A [Sou	0.45421
ENSMUSG00000097018	Gm26872	predicted gene, 26872 [Source:MGI Symbol;Acc:MG	0.45432
ENSMUSG00000086563	Gm14136	predicted gene 14136 [Source:MGI Symbol;Acc:MG	0.45434
ENSMUSG00000086855	Gm13844	predicted gene 13844 [Source:MGI Symbol;Acc:MG	0.45435
ENSMUSG00000032504	Pdcd6ip	programmed cell death 6 interacting protein [Sourc	0.45438
ENSMUSG00000085318	Gm12524	predicted gene 12524 [Source:MGI Symbol;Acc:MG	0.45445
ENSMUSG00000042228	Lyn	Yamaguchi sarcoma viral (v-yes-1) oncogene homol	0.45447
ENSMUSG00000067642	Gpr113	G protein-coupled receptor 113 [Source:MGI Symb	0.4545
ENSMUSG00000044788	Fads6	fatty acid desaturase domain family, member 6 [So	0.45456
ENSMUSG00000056306	Sertm1	serine rich and transmembrane domain containing	0.45473
ENSMUSG00000027177	Hipk3	homeodomain interacting protein kinase 3 [Source	0.45483
ENSMUSG00000043461	Sptssb	serine palmitoyltransferase, small subunit B [Sourc	0.45484
ENSMUSG00000032750	Gab3	growth factor receptor bound protein 2-associated	0.45493
ENSMUSG00000020471	Pold2	polymerase (DNA directed), delta 2, regulatory sub	0.45494
ENSMUSG00000032311	Nrg4	neuregulin 4 [Source:MGI Symbol;Acc:MGI:193383	0.45506
ENSMUSG00000030161	Gabarapl1	gamma-aminobutyric acid (GABA) A receptor-assoc	0.45507
ENSMUSG00000026672	Optn	optineurin [Source:MGI Symbol;Acc:MGI:1918898]	0.45519
ENSMUSG00000054455	Vapb	vesicle-associated membrane protein, associated p	0.45521
ENSMUSG00000028755	Cda	cytidine deaminase [Source:MGI Symbol;Acc:MGI:1	0.4553
ENSMUSG00000035354	Uvrag	UV radiation resistance associated gene [Source:M	0.45535
ENSMUSG00000091021	Gm17300	predicted gene, 17300 [Source:MGI Symbol;Acc:MG	0.45535
ENSMUSG00000062372	Otof	otoferlin [Source:MGI Symbol;Acc:MGI:1891247]	0.45537
ENSMUSG00000098142	Gm7507	predicted gene 7507 [Source:MGI Symbol;Acc:MGI:	0.45538
ENSMUSG00000074651	Mcidas	multiciliate differentiation and DNA synthesis assoc	0.4554
ENSMUSG00000024776	Stambpl1	STAM binding protein like 1 [Source:MGI Symbol;A	0.45542
ENSMUSG00000038982	Bloc1s5	biogenesis of organelles complex-1, subunit 5, mut	0.45544
ENSMUSG00000027099	Mtx2	metaxin 2 [Source:MGI Symbol;Acc:MGI:1859652]	0.45547
ENSMUSG00000027840	Wnt2b	wingless related MMTV integration site 2b [Source:	0.45549
ENSMUSG00000002346	Slc25a42	solute carrier family 25, member 42 [Source:MGI Sy	0.45553
ENSMUSG00000005447	Pafah1b3	platelet-activating factor acetylhydrolase, isoform 1	0.45555
ENSMUSG00000024063	Lbh	limb-bud and heart [Source:MGI Symbol;Acc:MGI:1	0.45557
ENSMUSG00000051727	Kctd14	potassium channel tetramerisation domain contain	0.45559
ENSMUSG00000003410	Elavl3	ELAV (embryonic lethal, abnormal vision, Drosophil	0.45569
ENSMUSG00000097525	Gm26845	predicted gene, 26845 [Source:MGI Symbol;Acc:MG	0.45577
ENSMUSG00000024354	Slc23a1	solute carrier family 23 (nucleobase transporters), r	0.45581
ENSMUSG00000031189	Aff2	AF4/FMR2 family, member 2 [Source:MGI Symbol;A	0.45589
ENSMUSG00000060708	Bloc1s4	biogenesis of organelles complex-1, subunit 4, capp	0.45601

ENSMUSG00000032298	Neil1	nei endonuclease VIII-like 1 (E. coli) [Source:MGI Sy	0.45602
ENSMUSG00000034881	Tbxa2r	thromboxane A2 receptor [Source:MGI Symbol;Acc	0.45619
ENSMUSG00000035266	Helq	helicase, POLQ-like [Source:MGI Symbol;Acc:MGI:2	0.45619
ENSMUSG00000040383	Aqr	aquarius [Source:MGI Symbol;Acc:MGI:1276102]	0.45621
ENSMUSG00000023944	Hsp90ab1	heat shock protein 90 alpha (cytosolic), class B mem	0.45624
ENSMUSG00000094900	Chdc2	calponin homology domain containing 2 [Source:M	0.45625
ENSMUSG00000058057	Mettl7a3	methyltransferase like 7A3 [Source:MGI Symbol;Ac	0.45628
ENSMUSG00000005413	Hmox1	heme oxygenase (decycling) 1 [Source:MGI Symbol	0.45632
ENSMUSG00000087348	Gm15582	predicted gene 15582 [Source:MGI Symbol;Acc:MG	0.45633
ENSMUSG00000036661	Dennd3	DENN/MADD domain containing 3 [Source:MGI Syr	0.45648
ENSMUSG00000013622	Atraid	all-trans retinoic acid induced differentiation factor	0.45653
ENSMUSG00000085546	Gm14252	predicted gene 14252 [Source:MGI Symbol;Acc:MG	0.45659
ENSMUSG00000051832	E230016K23Rik	RIKEN cDNA E230016K23 gene [Source:MGI Symbo	0.45691
ENSMUSG00000023017	Asic1	acid-sensing (proton-gated) ion channel 1 [Source:I	0.45693
ENSMUSG00000033703	Fuk	fucokinase [Source:MGI Symbol;Acc:MGI:1916071]	0.45695
ENSMUSG00000089909	Gm16164	predicted gene 16164 [Source:MGI Symbol;Acc:MG	0.45701
ENSMUSG00000091900	Gm4353	predicted gene 4353 [Source:MGI Symbol;Acc:MGI:	0.45702
ENSMUSG00000049612	Omg	oligodendrocyte myelin glycoprotein [Source:MGI S	0.4571
ENSMUSG00000022913	Psmg1	proteasome (prosome, macropain) assembly chape	0.45716
ENSMUSG00000075702	Selm	selenoprotein M [Source:MGI Symbol;Acc:MGI:214	0.45738
ENSMUSG00000042757	Tmem108	transmembrane protein 108 [Source:MGI Symbol;A	0.45741
ENSMUSG00000073482	Gm10517	predicted gene 10517 [Source:MGI Symbol;Acc:MG	0.45746
ENSMUSG00000004880	Lbr	lamin B receptor [Source:MGI Symbol;Acc:MGI:213	0.45757
ENSMUSG00000005716	Pvalb	parvalbumin [Source:MGI Symbol;Acc:MGI:97821]	0.45765
ENSMUSG00000026930	Gpsm1	G-protein signalling modulator 1 (AGS3-like, C. eleg	0.4577
ENSMUSG00000089818	Gm15950	predicted gene 15950 [Source:MGI Symbol;Acc:MG	0.45773
ENSMUSG00000086152	Gm11454	predicted gene 11454 [Source:MGI Symbol;Acc:MG	0.45776
ENSMUSG00000025580	Eif4a3	eukaryotic translation initiation factor 4A3 [Source:	0.45784
ENSMUSG00000000811	Txnrd3	thioredoxin reductase 3 [Source:MGI Symbol;Acc:M	0.45793
ENSMUSG00000063160	Numbl	numb-like [Source:MGI Symbol;Acc:MGI:894702]	0.45798
ENSMUSG00000036273	Lrrk2	leucine-rich repeat kinase 2 [Source:MGI Symbol;A	0.45799
ENSMUSG00000043664	Tmem221	transmembrane protein 221 [Source:MGI Symbol;A	0.45801
ENSMUSG00000089769	Gm16574	predicted gene 16574 [Source:MGI Symbol;Acc:MG	0.45806
ENSMUSG00000024038	Ndufv3	NADH dehydrogenase (ubiquinone) flavoprotein 3	0.45819
ENSMUSG00000048706	Lurap1l	leucine rich adaptor protein 1-like [Source:MGI Sym	0.45824
ENSMUSG00000027014	Cwc22	CWC22 spliceosome-associated protein homolog (S	0.45831
ENSMUSG00000035248	Zcchc6	zinc finger, CCHC domain containing 6 [Source:MGI	0.45838
ENSMUSG00000072884	Gm10433	predicted gene 10433 [Source:MGI Symbol;Acc:MG	0.45838
ENSMUSG00000048728	Zfp454	zinc finger protein 454 [Source:MGI Symbol;Acc:M	0.45841
ENSMUSG00000075043	Tmem223	transmembrane protein 223 [Source:MGI Symbol;A	0.45841
ENSMUSG00000087429	Gm16235	predicted gene 16235 [Source:MGI Symbol;Acc:MG	0.45846
ENSMUSG00000028327	1300002K09Rik	RIKEN cDNA 1300002K09 gene [Source:MGI Symbo	0.45856
ENSMUSG00000041730	Prrxl1	paired related homeobox protein-like 1 [Source:MG	0.45857
ENSMUSG00000023277	Twf2	twinfilin, actin-binding protein, homolog 2 (Drosop	0.45868
ENSMUSG00000036188	Ankmy2	ankyrin repeat and MYND domain containing 2 [So	0.45876
ENSMUSG00000051790	Nlgn2	neuroligin 2 [Source:MGI Symbol;Acc:MGI:2681835	0.45881
ENSMUSG00000031329	Tsx	testis specific X-linked gene [Source:MGI Symbol;A	0.45883

ENSMUSG00000084839	Gm14097	predicted gene 14097 [Source:MGI Symbol;Acc:MG	0.45894
ENSMUSG00000019923	Zwint	ZW10 interactor [Source:MGI Symbol;Acc:MGI:128	0.45895
ENSMUSG00000035382	Pcsk7	proprotein convertase subtilisin/kexin type 7 [Sour	0.45908
ENSMUSG00000063415	Cyp26b1	cytochrome P450, family 26, subfamily b, polypepti	0.4591
ENSMUSG00000097439	Gm16754	predicted gene, 16754 [Source:MGI Symbol;Acc:M	0.4591
ENSMUSG00000061758	Akr1b10	aldo-keto reductase family 1, member B10 (aldose	0.45921
ENSMUSG00000087387	Gm15420	predicted gene 15420 [Source:MGI Symbol;Acc:MG	0.4593
ENSMUSG00000096996	Gm26792	predicted gene, 26792 [Source:MGI Symbol;Acc:M	0.4593
ENSMUSG00000092124	B930094E09Rik	RIKEN cDNA B930094E09 gene [Source:MGI Symbo	0.45934
ENSMUSG00000029649	Pomp	proteasome maturation protein [Source:MGI Symb	0.45939
ENSMUSG00000073658	Crygb	crystallin, gamma B [Source:MGI Symbol;Acc:MGI:8	0.45941
ENSMUSG00000050530	Fam171a1	family with sequence similarity 171, member A1 [S	0.45945
ENSMUSG00000086714	0610009E02Rik	RIKEN cDNA 0610009E02 gene [Source:MGI Symbo	0.45949
ENSMUSG00000054435	Gimap4	GTPase, IMAP family member 4 [Source:MGI Symb	0.45964
ENSMUSG00000024790	Sac3d1	SAC3 domain containing 1 [Source:MGI Symbol;Acc	0.45974
ENSMUSG00000097996	Gm5313	predicted gene 5313 [Source:MGI Symbol;Acc:MGI:	0.45977
ENSMUSG00000037477	Tbx10	T-box 10 [Source:MGI Symbol;Acc:MGI:1261436]	0.4598
ENSMUSG00000096039	D830030K20Rik	RIKEN cDNA D830030K20 gene [Source:MGI Symbo	0.45981
ENSMUSG00000081678	Gm12959	predicted gene 12959 [Source:MGI Symbol;Acc:MG	0.45982
ENSMUSG00000092867	Mir3058	microRNA 3058 [Source:MGI Symbol;Acc:MGI:4834	0.45982
ENSMUSG00000082246	Gm13047	predicted gene 13047 [Source:MGI Symbol;Acc:MG	0.45984
ENSMUSG00000078186	Gm13821	predicted gene 13821 [Source:MGI Symbol;Acc:MG	0.45986
ENSMUSG00000096364	Gpr45	G protein-coupled receptor 45 [Source:MGI Symbo	0.45986
ENSMUSG00000097293	D630002J18Rik	RIKEN cDNA D630002J18 gene [Source:MGI Symbo	0.4599
ENSMUSG00000025577	Cbx2	chromobox 2 [Source:MGI Symbol;Acc:MGI:88289]	0.45991
ENSMUSG00000027628	Aar2	AAR2 splicing factor homolog (S. cerevisiae) [Sourc	0.45998
ENSMUSG00000093858	Gm19967	predicted gene, 19967 [Source:MGI Symbol;Acc:M	0.46001
ENSMUSG00000080776	Gm12174	predicted gene 12174 [Source:MGI Symbol;Acc:MG	0.46005
ENSMUSG00000009733	Tfcp2	transcription factor CP2 [Source:MGI Symbol;Acc:M	0.46006
ENSMUSG00000020542	Myocd	myocardin [Source:MGI Symbol;Acc:MGI:2137495]	0.46007
ENSMUSG00000031535	Dkk4	dickkopf homolog 4 (Xenopus laevis) [Source:MGI S	0.4601
ENSMUSG00000052137	Rbm12b2	RNA binding motif protein 12 B2 [Source:MGI Symb	0.46011
ENSMUSG00000081311	Gm12197	predicted gene 12197 [Source:MGI Symbol;Acc:MG	0.46018
ENSMUSG00000080902	Gm11226	predicted gene 11226 [Source:MGI Symbol;Acc:MG	0.4602
ENSMUSG00000089622	Gm25447	predicted gene, 25447 [Source:MGI Symbol;Acc:M	0.46023
ENSMUSG00000094852	Gm9034	predicted pseudogene 9034 [Source:MGI Symbol;A	0.46025
ENSMUSG00000086677	Gm12284	predicted gene 12284 [Source:MGI Symbol;Acc:MG	0.46027
ENSMUSG00000091230	Gm6970	predicted gene 6970 [Source:MGI Symbol;Acc:MGI:	0.46039
ENSMUSG00000089983	2010320O07Rik	RIKEN cDNA 2010320O07 gene [Source:MGI Symbo	0.4604
ENSMUSG00000026235	Epha4	Eph receptor A4 [Source:MGI Symbol;Acc:MGI:982	0.46041
ENSMUSG00000003865	Gys1	glycogen synthase 1, muscle [Source:MGI Symbol;A	0.46065
ENSMUSG00000075703	Ept1	ethanolaminephosphotransferase 1 (CDP-ethanola	0.46068
ENSMUSG00000043683	Fem1a	feminization 1 homolog a (C. elegans) [Source:MGI	0.46075
ENSMUSG00000028307	Aldob	aldolase B, fructose-bisphosphate [Source:MGI Sym	0.46079
ENSMUSG00000079644	Gm1110	predicted gene 1110 [Source:MGI Symbol;Acc:MGI:	0.46083
ENSMUSG00000025436	Xrcc6bp1	XRCC6 binding protein 1 [Source:MGI Symbol;Acc:M	0.46089
ENSMUSG00000094824	Gm23803	predicted gene, 23803 [Source:MGI Symbol;Acc:M	0.46108

ENSMUSG00000090179	Gm16307	predicted gene 16307 [Source:MGI Symbol;Acc:MG	0.46111
ENSMUSG00000094334	Fabp5l2	fatty acid binding protein 5-like 2 [Source:MGI Sym	0.46111
ENSMUSG00000032044	Rpusd4	RNA pseudouridylate synthase domain containing 4	0.46112
ENSMUSG00000020027	Socs2	suppressor of cytokine signaling 2 [Source:MGI Sym	0.46113
ENSMUSG00000084945	C030037F17Rik	RIKEN cDNA C030037F17 gene [Source:MGI Symbo	0.46114
ENSMUSG00000048498	Cd300e	CD300e antigen [Source:MGI Symbol;Acc:MGI:2387	0.46116
ENSMUSG00000081635	Gm15071	predicted gene 15071 [Source:MGI Symbol;Acc:MG	0.46117
ENSMUSG00000031230	Fgf16	fibroblast growth factor 16 [Source:MGI Symbol;Ac	0.46118
ENSMUSG00000007805	Twist2	twist basic helix-loop-helix transcription factor 2 [S	0.46123
ENSMUSG00000087114	Gm16099	predicted gene 16099 [Source:MGI Symbol;Acc:MG	0.46125
ENSMUSG00000040596	Pogk	pogo transposable element with KRAB domain [Sou	0.4613
ENSMUSG00000023805	Synj2	synaptojanin 2 [Source:MGI Symbol;Acc:MGI:1201	0.46133
ENSMUSG00000038805	Six3	sine oculis-related homeobox 3 [Source:MGI Symbo	0.46144
ENSMUSG00000019738	Polr2i	polymerase (RNA) II (DNA directed) polypeptide I [S	0.46145
ENSMUSG00000032216	Nedd4	neural precursor cell expressed, developmentally d	0.46145
ENSMUSG00000046679	C87436	expressed sequence C87436 [Source:MGI Symbol;A	0.46152
ENSMUSG00000051495	Irf2bp2	interferon regulatory factor 2 binding protein 2 [So	0.46182
ENSMUSG00000048440	Cyp4f16	cytochrome P450, family 4, subfamily f, polypeptide	0.46183
ENSMUSG00000061143	Maml3	mastermind like 3 (Drosophila) [Source:MGI Symbo	0.46185
ENSMUSG00000001138	Cnnm3	cyclin M3 [Source:MGI Symbol;Acc:MGI:2151055]	0.46196
ENSMUSG00000033114	Slc35d2	solute carrier family 35, member D2 [Source:MGI S	0.46196
ENSMUSG00000028427	Aqp7	aquaporin 7 [Source:MGI Symbol;Acc:MGI:1314647	0.46197
ENSMUSG00000045211	Nudt18	nudix (nucleoside diphosphate linked moiety X)-typ	0.46221
ENSMUSG00000081894	Gm14212	predicted gene 14212 [Source:MGI Symbol;Acc:MG	0.46232
ENSMUSG00000001829	Clpb	ClpB caseinolytic peptidase B [Source:MGI Symbol;	0.46241
ENSMUSG00000034037	Fgd5	FYVE, RhoGEF and PH domain containing 5 [Source	0.46244
ENSMUSG00000052821	Cysltr1	cysteinyl leukotriene receptor 1 [Source:MGI Symb	0.4625
ENSMUSG00000097207	6030443J06Rik	RIKEN cDNA 6030443J06 gene [Source:MGI Symbol	0.46254
ENSMUSG00000078958	Atp6ap1l	ATPase, H+ transporting, lysosomal accessory prote	0.4626
ENSMUSG00000046949	Nqo2	NAD(P)H dehydrogenase, quinone 2 [Source:MGI S	0.46277
ENSMUSG00000003731	Kpna6	karyopherin (importin) alpha 6 [Source:MGI Symbo	0.46281
ENSMUSG00000095041	AC149090.1		0.46281
ENSMUSG00000097459	Gm26895	predicted gene, 26895 [Source:MGI Symbol;Acc:M	0.46291
ENSMUSG00000089407	Gm24878	predicted gene, 24878 [Source:MGI Symbol;Acc:M	0.46293
ENSMUSG00000092644	Gm23564	predicted gene, 23564 [Source:MGI Symbol;Acc:M	0.46293
ENSMUSG00000077816	Gm22735	predicted gene, 22735 [Source:MGI Symbol;Acc:M	0.46294
ENSMUSG00000074224	4932431P20Rik	RIKEN cDNA 4932431P20 gene [Source:MGI Symbo	0.46296
ENSMUSG00000045915	Ccdc42	coiled-coil domain containing 42 [Source:MGI Symb	0.46297
ENSMUSG00000070713	Gm10282	predicted pseudogene 10282 [Source:MGI Symbol;	0.46297
ENSMUSG00000062083	Rpl13a-ps1	ribosomal protein 13A, pseudogene 1 [Source:MGI	0.46298
ENSMUSG00000051638	Gm9857	predicted gene 9857 [Source:MGI Symbol;Acc:MGI:	0.46301
ENSMUSG00000020402	Vdac1	voltage-dependent anion channel 1 [Source:MGI Sy	0.46305
ENSMUSG00000074570	Cass4	Cas scaffolding protein family member 4 [Source:M	0.4631
ENSMUSG00000087293	Gm14341	predicted gene 14341 [Source:MGI Symbol;Acc:MG	0.46318
ENSMUSG00000054293	A630033H20Rik	RIKEN cDNA A630033H20 gene [Source:MGI Symbo	0.46331
ENSMUSG00000097223	Gm2449	predicted gene 2449 [Source:MGI Symbol;Acc:MGI:	0.46334
ENSMUSG00000041530	Ago1	argonaute RISC catalytic subunit 1 [Source:MGI Syn	0.46339

ENSMUSG00000014349	Ube2z	ubiquitin-conjugating enzyme E2Z (putative) [Sourc	0.46343
ENSMUSG00000054944	5330416C01Rik	RIKEN cDNA 5330416C01 gene [Source:MGI Symbo	0.46343
ENSMUSG00000097314	Gm26567	predicted gene, 26567 [Source:MGI Symbol;Acc:MC	0.46343
ENSMUSG00000083033	Gm9083	predicted gene 9083 [Source:MGI Symbol;Acc:MGI:	0.46344
ENSMUSG00000030382	Slc27a5	solute carrier family 27 (fatty acid transporter), me	0.46357
ENSMUSG00000033444	Specc1l	sperm antigen with calponin homology and coiled-c	0.4636
ENSMUSG00000070577	Gm572	predicted gene 572 [Source:MGI Symbol;Acc:MGI:2	0.46369
ENSMUSG00000032965	Ift57	intraflagellar transport 57 [Source:MGI Symbol;Acc	0.46371
ENSMUSG00000035284	Vps13c	vacuolar protein sorting 13C (yeast) [Source:MGI Sy	0.46371
ENSMUSG00000097003	D930007P13Rik	Riken cDNA D930007P13 gene [Source:MGI Symbol	0.46375
ENSMUSG00000032743	D430042O09Rik	RIKEN cDNA D430042O09 gene [Source:MGI Symbo	0.46384
ENSMUSG00000029190	D5Ertd579e	DNA segment, Chr 5, ERATO Doi 579, expressed [So	0.46388
ENSMUSG00000044991	1110034G24Rik	RIKEN cDNA 1110034G24 gene [Source:MGI Symbo	0.46394
ENSMUSG00000051278	4930422G04Rik	RIKEN cDNA 4930422G04 gene [Source:MGI Symbo	0.46396
ENSMUSG00000092586	Ly6g6c	lymphocyte antigen 6 complex, locus G6C [Source:M	0.46406
ENSMUSG00000089789	Rdh1	retinol dehydrogenase 1 (all trans) [Source:MGI Syn	0.46418
ENSMUSG00000040433	Zbtb38	zinc finger and BTB domain containing 38 [Source:M	0.46422
ENSMUSG00000094603	Gm25872	predicted gene, 25872 [Source:MGI Symbol;Acc:MC	0.46431
ENSMUSG00000038067	Csf3	colony stimulating factor 3 (granulocyte) [Source:M	0.46438
ENSMUSG00000067369	Trmt2b	TRM2 tRNA methyltransferase 2B [Source:MGI Sym	0.46439
ENSMUSG00000089365	Gm23823	predicted gene, 23823 [Source:MGI Symbol;Acc:MC	0.46439
ENSMUSG00000038975	Rabggtb	RAB geranylgeranyl transferase, b subunit [Source:I	0.46459
ENSMUSG00000069814	E130309D14Rik	RIKEN cDNA E130309D14 gene [Source:MGI Symbo	0.46459
ENSMUSG00000097875	Gm26704	predicted gene, 26704 [Source:MGI Symbol;Acc:MC	0.46459
ENSMUSG00000024556	Me2	malic enzyme 2, NAD(+)-dependent, mitochondrial	0.4646
ENSMUSG00000097843	Gm26755	predicted gene, 26755 [Source:MGI Symbol;Acc:MC	0.46466
ENSMUSG00000032557	Uba5	ubiquitin-like modifier activating enzyme 5 [Source	0.46473
ENSMUSG00000038025	Phf2	PHD finger protein 2 [Source:MGI Symbol;Acc:MGI:	0.46475
ENSMUSG00000052921	Arhgef15	Rho guanine nucleotide exchange factor (GEF) 15 [S	0.46483
ENSMUSG00000036560	Lgi4	leucine-rich repeat LGI family, member 4 [Source:M	0.46486
ENSMUSG00000045954	Sdpr	serum deprivation response [Source:MGI Symbol;A	0.46488
ENSMUSG00000074502	Ubtfl1	upstream binding transcription factor, RNA polyme	0.46493
ENSMUSG00000025810	Nrp1	neuropilin 1 [Source:MGI Symbol;Acc:MGI:106206]	0.46511
ENSMUSG00000086296	D030055H07Rik	RIKEN cDNA D030055H07 gene [Source:MGI Symbo	0.46527
ENSMUSG00000038930	Rccd1	RCC1 domain containing 1 [Source:MGI Symbol;Acc	0.46534
ENSMUSG00000035242	Oaz1	ornithine decarboxylase antizyme 1 [Source:MGI Sy	0.46535
ENSMUSG00000044948	Wdr96	WD repeat domain 96 [Source:MGI Symbol;Acc:MG	0.46551
ENSMUSG00000048458	Fam212b	family with sequence similarity 212, member B [Sou	0.46552
ENSMUSG00000030166	Rad52	RAD52 homolog (S. cerevisiae) [Source:MGI Symbo	0.46556
ENSMUSG00000090258	Churc1	churchill domain containing 1 [Source:MGI Symbol;	0.46559
ENSMUSG00000030966	Trim21	tripartite motif-containing 21 [Source:MGI Symbol;	0.4656
ENSMUSG00000062995	Ica1	islet cell autoantigen 1 [Source:MGI Symbol;Acc:MC	0.46563
ENSMUSG00000097095	Gm26547	predicted gene, 26547 [Source:MGI Symbol;Acc:MC	0.46569
ENSMUSG00000082027	Gm11416	predicted gene 11416 [Source:MGI Symbol;Acc:MG	0.4657
ENSMUSG00000055694	Gdf1	growth differentiation factor 1 [Source:MGI Symbo	0.46571
ENSMUSG00000097371	Gm26875	predicted gene, 26875 [Source:MGI Symbol;Acc:MC	0.46571
ENSMUSG00000031370	Zrsr2	zinc finger (CCCH type), RNA binding motif and seri	0.46573

ENSMUSG00000086569	4930542H20Rik	RIKEN cDNA 4930542H20 gene [Source:MGI Symbo	0.46574
ENSMUSG00000026285	Pdcd1	programmed cell death 1 [Source:MGI Symbol;Acc:	0.46575
ENSMUSG00000098156	Gm27023	predicted gene, 27023 [Source:MGI Symbol;Acc:M(	0.46577
ENSMUSG00000066724	Gm10175	predicted gene 10175 [Source:MGI Symbol;Acc:MG	0.4658
ENSMUSG00000073902	Gm1966	predicted gene 1966 [Source:MGI Symbol;Acc:MGI:	0.4658
ENSMUSG00000081121	Gm12791	predicted gene 12791 [Source:MGI Symbol;Acc:MG	0.46581
ENSMUSG00000055110	A630012P03Rik	RIKEN cDNA A630012P03 gene [Source:MGI Symbo	0.46583
ENSMUSG00000074342	I830077J02Rik	RIKEN cDNA I830077J02 gene [Source:MGI Symbol;	0.46584
ENSMUSG00000039579	Grin3a	glutamate receptor ionotropic, NMDA3A [Source:M	0.46585
ENSMUSG00000039058	Ak5	adenylate kinase 5 [Source:MGI Symbol;Acc:MGI:2(	0.46593
ENSMUSG00000025922	Gm5045	predicted gene 5045 [Source:MGI Symbol;Acc:MGI:	0.46595
ENSMUSG00000080798	Gm5135	predicted pseudogene 5135 [Source:MGI Symbol;A	0.46598
ENSMUSG00000085035	Gm12031	predicted gene 12031 [Source:MGI Symbol;Acc:MG	0.466
ENSMUSG00000096942	Rps19-ps6	ribosomal protein S19, pseudogene 6 [Source:MGI	0.466
ENSMUSG00000073051	Gm14812	predicted gene 14812 [Source:MGI Symbol;Acc:MG	0.46601
ENSMUSG00000056031	9330154J02Rik	RIKEN cDNA 9330154J02 gene [Source:MGI Symbol	0.4661
ENSMUSG00000086310	Gm12278	predicted gene 12278 [Source:MGI Symbol;Acc:MG	0.4661
ENSMUSG00000073407	Gm6034	predicted gene 6034 [Source:MGI Symbol;Acc:MGI:	0.46614
ENSMUSG00000045201	Lrrc3b	leucine rich repeat containing 3B [Source:MGI Sym	0.46616
ENSMUSG00000024785	Rcl1	RNA terminal phosphate cyclase-like 1 [Source:MG	0.46621
ENSMUSG00000065282	Gm24455	predicted gene, 24455 [Source:MGI Symbol;Acc:M(	0.46629
ENSMUSG00000028952	Zbtb48	zinc finger and BTB domain containing 48 [Source:N	0.46631
ENSMUSG00000018572	Phf23	PHD finger protein 23 [Source:MGI Symbol;Acc:MG	0.46646
ENSMUSG00000064168	Hist1h2bh	histone cluster 1, H2bh [Source:MGI Symbol;Acc:M	0.46649
ENSMUSG00000035765	Dym	dymeclin [Source:MGI Symbol;Acc:MGI:1918480]	0.46654
ENSMUSG00000029103	Lrpap1	low density lipoprotein receptor-related protein as	0.46655
ENSMUSG00000053646	Plxnb1	plexin B1 [Source:MGI Symbol;Acc:MGI:2154238]	0.46669
ENSMUSG00000097177	9330159M07Rik	RIKEN cDNA 9330159M07 gene [Source:MGI Symbo	0.46669
ENSMUSG00000082108	Gm13777	predicted gene 13777 [Source:MGI Symbol;Acc:MG	0.46679
ENSMUSG00000082332	Gm13384	predicted gene 13384 [Source:MGI Symbol;Acc:MG	0.46682
ENSMUSG00000082791	Gm4875	predicted gene 4875 [Source:MGI Symbol;Acc:MGI:	0.46682
ENSMUSG00000057193	Slc44a2	solute carrier family 44, member 2 [Source:MGI Syr	0.46684
ENSMUSG00000081929	Rps11-ps2	ribosomal protein S11, pseudogene 2 [Source:MGI	0.46691
ENSMUSG00000082981	Gm11526	predicted gene 11526 [Source:MGI Symbol;Acc:MG	0.46693
ENSMUSG00000002771	Grin2d	glutamate receptor, ionotropic, NMDA2D (epsilon 4	0.46697
ENSMUSG00000089785	Gm8429	predicted gene 8429 [Source:MGI Symbol;Acc:MGI:	0.46699
ENSMUSG00000089048	Gm26167	predicted gene, 26167 [Source:MGI Symbol;Acc:M(	0.46701
ENSMUSG00000040459	Arglu1	arginine and glutamate rich 1 [Source:MGI Symbol;	0.46713
ENSMUSG00000043059	Zfp513	zinc finger protein 513 [Source:MGI Symbol;Acc:M(	0.46727
ENSMUSG00000067212	H2-T23	histocompatibility 2, T region locus 23 [Source:MGI	0.46729
ENSMUSG00000010914	Pdhx	pyruvate dehydrogenase complex, component X [S	0.46732
ENSMUSG00000081670	Gm15697	predicted gene 15697 [Source:MGI Symbol;Acc:MG	0.46742
ENSMUSG00000048484	Gm7461	predicted gene 7461 [Source:MGI Symbol;Acc:MGI:	0.46746
ENSMUSG00000028842	Ago3	argonaute RISC catalytic subunit 3 [Source:MGI Syn	0.46748
ENSMUSG00000063172	Hspb11	heat shock protein family B (small), member 11 [So	0.46752
ENSMUSG00000042129	Rassf4	Ras association (RalGDS/AF-6) domain family meml	0.46764
ENSMUSG00000085938	Gm14371	predicted gene 14371 [Source:MGI Symbol;Acc:MG	0.46772

ENSMUSG00000080845	Gm9115	predicted gene 9115 [Source:MGI Symbol;Acc:MGI:	0.46776
ENSMUSG00000025955	Akr1cl	aldo-keto reductase family 1, member C-like [Sourc	0.46779
ENSMUSG00000092394	Gm20445	predicted gene 20445 [Source:MGI Symbol;Acc:MG	0.46782
ENSMUSG00000039990	2700050L05Rik	RIKEN cDNA 2700050L05 gene [Source:MGI Symbo	0.46785
ENSMUSG00000089753	Gm11709	predicted gene 11709 [Source:MGI Symbol;Acc:MG	0.46786
ENSMUSG00000090079	Gm15849	predicted gene 15849 [Source:MGI Symbol;Acc:MG	0.46786
ENSMUSG00000077271	Gm22781	predicted gene, 22781 [Source:MGI Symbol;Acc:M(	0.46787
ENSMUSG00000082350	Gm14008	predicted gene 14008 [Source:MGI Symbol;Acc:MG	0.4679
ENSMUSG00000031727	Pmfbp1	polyamine modulated factor 1 binding protein 1 [S(	0.46791
ENSMUSG00000061991	Hist1h2af	histone cluster 1, H2af [Source:MGI Symbol;Acc:M(	0.46792
ENSMUSG00000078922	Tgtp1	T cell specific GTPase 1 [Source:MGI Symbol;Acc:M	0.46793
ENSMUSG00000024955	Esrra	estrogen related receptor, alpha [Source:MGI Symb	0.46795
ENSMUSG00000064452	Gm24564	predicted gene, 24564 [Source:MGI Symbol;Acc:M(	0.46796
ENSMUSG00000081520	Gm16200	predicted gene 16200 [Source:MGI Symbol;Acc:MG	0.46796
ENSMUSG00000088942	Gm23260	predicted gene, 23260 [Source:MGI Symbol;Acc:M(	0.468
ENSMUSG00000059816	Fam159a	family with sequence similarity 159, member A [Sou	0.46801
ENSMUSG00000088822	Gm25225	predicted gene, 25225 [Source:MGI Symbol;Acc:M(	0.46801
ENSMUSG00000088010	Gm23740	predicted gene, 23740 [Source:MGI Symbol;Acc:M(	0.46802
ENSMUSG00000087459	4930465A12Rik	RIKEN cDNA 4930465A12 gene [Source:MGI Symbo	0.46803
ENSMUSG00000092354	Gm20548	predicted gene 20548 [Source:MGI Symbol;Acc:MG	0.46807
ENSMUSG00000083834	Gm12577	predicted gene 12577 [Source:MGI Symbol;Acc:MG	0.4681
ENSMUSG00000017670	Elmo2	engulfment and cell motility 2 [Source:MGI Symbol	0.46814
ENSMUSG00000086645	Gm15743	predicted gene 15743 [Source:MGI Symbol;Acc:MG	0.46814
ENSMUSG00000029651	Mtus2	microtubule associated tumor suppressor candidat	0.46816
ENSMUSG00000053420	Gm4792	predicted gene 4792 [Source:MGI Symbol;Acc:MGI:	0.46823
ENSMUSG00000007036	Abhd16a	abhydrolase domain containing 16A [Source:MGI S	0.46833
ENSMUSG00000025241	Fyco1	FYVE and coiled-coil domain containing 1 [Source:N	0.46834
ENSMUSG00000026499	Acbd3	acyl-Coenzyme A binding domain containing 3 [Sou	0.46862
ENSMUSG00000020838	Slc6a4	solute carrier family 6 (neurotransmitter transporte	0.46863
ENSMUSG00000026374	Tsn	translin [Source:MGI Symbol;Acc:MGI:109263]	0.46879
ENSMUSG00000036862	Dchs1	dachsous 1 (Drosophila) [Source:MGI Symbol;Acc:N	0.46882
ENSMUSG00000085396	6720401G13Rik	RIKEN cDNA 6720401G13 gene [Source:MGI Symbo	0.46882
ENSMUSG00000032246	Calml4	calmodulin-like 4 [Source:MGI Symbol;Acc:MGI:192	0.46883
ENSMUSG00000016179	Camk1g	calcium/calmodulin-dependent protein kinase I gar	0.46889
ENSMUSG00000033295	Ptprf	protein tyrosine phosphatase, receptor type, F [Sou	0.46891
ENSMUSG00000058672	Tubb2a	tubulin, beta 2A class IIA [Source:MGI Symbol;Acc:N	0.46897
ENSMUSG00000087159	Gm15246	predicted gene 15246 [Source:MGI Symbol;Acc:MG	0.46908
ENSMUSG00000031258	Xkrx	X Kell blood group precursor related X linked [Sourc	0.46909
ENSMUSG00000042428	Mgat3	mannoside acetylglucosaminyltransferase 3 [Source	0.46915
ENSMUSG00000042770	Hebp1	heme binding protein 1 [Source:MGI Symbol;Acc:M	0.46921
ENSMUSG00000046727	Cystm1	cysteine-rich transmembrane module containing 1	0.46922
ENSMUSG00000024922	Ovol1	OVO homolog-like 1 (Drosophila) [Source:MGI Sym	0.4693
ENSMUSG00000031983	2310022B05Rik	RIKEN cDNA 2310022B05 gene [Source:MGI Symbo	0.46933
ENSMUSG00000086681	Gm16178	predicted gene 16178 [Source:MGI Symbol;Acc:MG	0.46937
ENSMUSG00000010307	Tmem86a	transmembrane protein 86A [Source:MGI Symbol;A	0.4695
ENSMUSG00000026806	Ddx31	DEAD/H (Asp-Glu-Ala-Asp/His) box polypeptide 31	0.46957
ENSMUSG00000053134	Supt7l	suppressor of Ty 7-like [Source:MGI Symbol;Acc:M(	0.46961

ENSMUSG00000015852	Fcrls	Fc receptor-like S, scavenger receptor [Source:MGI	0.46975
ENSMUSG00000027524	Edn3	endothelin 3 [Source:MGI Symbol;Acc:MGI:95285]	0.46981
ENSMUSG00000060938	Rpl26	ribosomal protein L26 [Source:MGI Symbol;Acc:MG	0.46982
ENSMUSG00000025159	Mms19	MMS19 (MET18 S. cerevisiae) [Source:MGI Symbol;	0.46995
ENSMUSG00000079557	2-Mar	membrane-associated ring finger (C3HC4) 2 [Source	0.47018
ENSMUSG00000059791	Nrm	nurim (nuclear envelope membrane protein) [Sourc	0.47019
ENSMUSG00000026950	Neb	nebulin [Source:MGI Symbol;Acc:MGI:97292]	0.47023
ENSMUSG00000052713	Zfp608	zinc finger protein 608 [Source:MGI Symbol;Acc:M	0.47026
ENSMUSG00000042605	Atxn2	ataxin 2 [Source:MGI Symbol;Acc:MGI:1277223]	0.47031
ENSMUSG00000088373	Mir1956	microRNA 1956 [Source:MGI Symbol;Acc:MGI:3837	0.47034
ENSMUSG00000015981	Stk32c	serine/threonine kinase 32C [Source:MGI Symbol;A	0.47036
ENSMUSG00000086912	4930505O20Rik	RIKEN cDNA 4930505O20 gene [Source:MGI Symbo	0.47038
ENSMUSG00000027326	Casc5	cancer susceptibility candidate 5 [Source:MGI Symb	0.4704
ENSMUSG00000083993	Gm14398	predicted gene 14398 [Source:MGI Symbol;Acc:MG	0.4704
ENSMUSG00000069554	I830134H01Rik	RIKEN cDNA I830134H01 gene [Source:MGI Symbol	0.47042
ENSMUSG00000095472	Snord88a	small nucleolar RNA, C/D box 88A [Source:MGI Sym	0.47042
ENSMUSG00000098157	Gm9022	predicted gene 9022 [Source:MGI Symbol;Acc:MGI:	0.47044
ENSMUSG00000085382	Gm13861	predicted gene 13861 [Source:MGI Symbol;Acc:MG	0.47045
ENSMUSG00000027940	Tpm3	tropomyosin 3, gamma [Source:MGI Symbol;Acc:M	0.47046
ENSMUSG00000086826	Gm11739	predicted gene 11739 [Source:MGI Symbol;Acc:MG	0.47073
ENSMUSG00000002803	Btbd6	BTB (POZ) domain containing 6 [Source:MGI Symbo	0.47091
ENSMUSG00000096992	Gm26788	predicted gene, 26788 [Source:MGI Symbol;Acc:M	0.47098
ENSMUSG00000050751	Pgbd5	piggyBac transposable element derived 5 [Source:M	0.47106
ENSMUSG00000023032	Slc4a8	solute carrier family 4 (anion exchanger), member 8	0.47109
ENSMUSG00000033863	Klf9	Kruppel-like factor 9 [Source:MGI Symbol;Acc:MGI:	0.47109
ENSMUSG00000035900	Gramd4	GRAM domain containing 4 [Source:MGI Symbol;Ac	0.47109
ENSMUSG00000027569	Mrgbp	MRG/MORF4L binding protein [Source:MGI Symbo	0.47111
ENSMUSG00000032411	Tfdp2	transcription factor Dp 2 [Source:MGI Symbol;Acc:M	0.47122
ENSMUSG00000042496	Prdm10	PR domain containing 10 [Source:MGI Symbol;Acc:	0.47122
ENSMUSG00000075705	Msrb1	methionine sulfoxide reductase B1 [Source:MGI Sy	0.47126
ENSMUSG00000086556	Gm15444	predicted gene 15444 [Source:MGI Symbol;Acc:MG	0.47138
ENSMUSG00000024074	Crim1	cysteine rich transmembrane BMP regulator 1 (cho	0.4714
ENSMUSG00000074571	1700101G07Rik	RIKEN cDNA 1700101G07 gene [Source:MGI Symbo	0.47143
ENSMUSG00000000739	Sult5a1	sulfotransferase family 5A, member 1 [Source:MGI	0.47145
ENSMUSG00000033475	Tomm6	translocase of outer mitochondrial membrane 6 ho	0.47153
ENSMUSG00000026649	1700009P17Rik	RIKEN cDNA 1700009P17 gene [Source:MGI Symbo	0.47154
ENSMUSG00000030400	Ercc2	excision repair cross-complementing rodent repair	0.47157
ENSMUSG00000030500	Slc17a6	solute carrier family 17 (sodium-dependent inorgan	0.47165
ENSMUSG00000070476	Fam217b	family with sequence similarity 217, member B [Sou	0.47172
ENSMUSG00000050043	Tmx2	thioredoxin-related transmembrane protein 2 [Sou	0.47173
ENSMUSG00000020280	Pus10	pseudouridylate synthase 10 [Source:MGI Symbol;A	0.47182
ENSMUSG00000046152	Fut10	fucosyltransferase 10 [Source:MGI Symbol;Acc:MG	0.47204
ENSMUSG00000097509	B230322F03Rik	RIKEN cDNA B230322F03 gene [Source:MGI Symbo	0.47205
ENSMUSG00000025196	Cpn1	carboxypeptidase N, polypeptide 1 [Source:MGI Sy	0.47218
ENSMUSG00000078161	BC007180	cDNA sequence BC007180 [Source:MGI Symbol;Acc	0.47218
ENSMUSG00000055116	Arntl	aryl hydrocarbon receptor nuclear translocator-like	0.47219
ENSMUSG00000028602	Tnfrsf8	tumor necrosis factor receptor superfamily, membe	0.4722

ENSMUSG00000040997	Abhd4	abhydrolase domain containing 4 [Source:MGI Sym	0.4722
ENSMUSG00000028798	Eif3i	eukaryotic translation initiation factor 3, subunit I [	0.47225
ENSMUSG00000070837	Aurkc	aurora kinase C [Source:MGI Symbol;Acc:MGI:1321	0.47225
ENSMUSG00000080626	Mir1192	microRNA 1192 [Source:MGI Symbol;Acc:MGI:3783	0.47228
ENSMUSG00000098144	1700029N11Rik	RIKEN cDNA 1700029N11 gene [Source:MGI Symbo	0.4723
ENSMUSG00000097918	Ascl5	achaete-scute complex homolog 5 (Drosophila) [So	0.47232
ENSMUSG00000006611	Hfe	hemochromatosis [Source:MGI Symbol;Acc:MGI:10	0.47235
ENSMUSG00000087613	Gm13855	predicted gene 13855 [Source:MGI Symbol;Acc:MG	0.47235
ENSMUSG00000028689	Ccdc163	coiled-coil domain containing 163 [Source:MGI Sym	0.47252
ENSMUSG00000037984	Neurod6	neurogenic differentiation 6 [Source:MGI Symbol;A	0.4726
ENSMUSG00000030421	Uri1	URI1, prefoldin-like chaperone [Source:MGI Symbo	0.47264
ENSMUSG00000037503	Fam168b	family with sequence similarity 168, member B [Sou	0.47273
ENSMUSG00000024065	Ehd3	EH-domain containing 3 [Source:MGI Symbol;Acc:M	0.47274
ENSMUSG00000038587	Akap12	A kinase (PRKA) anchor protein (gravin) 12 [Source:	0.47277
ENSMUSG00000026339	Ccdc93	coiled-coil domain containing 93 [Source:MGI Symb	0.47304
ENSMUSG00000052920	Prkg1	protein kinase, cGMP-dependent, type I [Source:M	0.47307
ENSMUSG00000047965	Rpl9-ps7	ribosomal protein L9, pseudogene 7 [Source:MGI S	0.4732
ENSMUSG00000040467	4921522P10Rik	RIKEN cDNA 4921522P10 gene [Source:MGI Symbo	0.47322
ENSMUSG00000093489	Gm20625	predicted gene 20625 [Source:MGI Symbol;Acc:MG	0.47329
ENSMUSG00000052962	Mrpl35	mitochondrial ribosomal protein L35 [Source:MGI S	0.47333
ENSMUSG00000039145	Camk1d	calcium/calmodulin-dependent protein kinase ID [S	0.47341
ENSMUSG00000036026	Tmem63b	transmembrane protein 63b [Source:MGI Symbol;A	0.47353
ENSMUSG00000043913	Ccdc60	coiled-coil domain containing 60 [Source:MGI Symb	0.47353
ENSMUSG00000068794	Col28a1	collagen, type XXVIII, alpha 1 [Source:MGI Symbol;A	0.47358
ENSMUSG00000086740	Gm17029	predicted gene 17029 [Source:MGI Symbol;Acc:MG	0.47365
ENSMUSG00000020698	Cct6b	chaperonin containing Tcp1, subunit 6b (zeta) [Sou	0.47367
ENSMUSG00000024604	Rbm22	RNA binding motif protein 22 [Source:MGI Symbol;	0.47374
ENSMUSG00000030880	Polr3e	polymerase (RNA) III (DNA directed) polypeptide E	0.47384
ENSMUSG00000062991	Nrg1	neuregulin 1 [Source:MGI Symbol;Acc:MGI:96083]	0.47388
ENSMUSG00000015869	Prpsap1	phosphoribosyl pyrophosphate synthetase-associat	0.47393
ENSMUSG00000008384	Sertad1	SERTA domain containing 1 [Source:MGI Symbol;Ac	0.474
ENSMUSG00000023175	Bsg	basigin [Source:MGI Symbol;Acc:MGI:88208]	0.47406
ENSMUSG00000039375	Wdr17	WD repeat domain 17 [Source:MGI Symbol;Acc:MG	0.47406
ENSMUSG00000024121	Atp6v0c	ATPase, H+ transporting, lysosomal V0 subunit C [S	0.47417
ENSMUSG00000000562	Adora3	adenosine A3 receptor [Source:MGI Symbol;Acc:M	0.47427
ENSMUSG00000044854	1700056E22Rik	RIKEN cDNA 1700056E22 gene [Source:MGI Symbo	0.47428
ENSMUSG00000087365	C430049B03Rik	RIKEN cDNA C430049B03 gene [Source:MGI Symbo	0.47432
ENSMUSG00000031221	Igbp1	immunoglobulin (CD79A) binding protein 1 [Source	0.47433
ENSMUSG00000068037	Mas1	MAS1 oncogene [Source:MGI Symbol;Acc:MGI:969	0.47435
ENSMUSG00000085434	Gm11725	predicted gene 11725 [Source:MGI Symbol;Acc:MG	0.47435
ENSMUSG00000020913	Krt24	keratin 24 [Source:MGI Symbol;Acc:MGI:1922956]	0.47442
ENSMUSG00000040325	Vprbp	Vpr (HIV-1) binding protein [Source:MGI Symbol;Ac	0.47442
ENSMUSG00000087639	Gm15512	predicted gene 15512 [Source:MGI Symbol;Acc:MG	0.47444
ENSMUSG00000086061	Gm13075	predicted gene 13075 [Source:MGI Symbol;Acc:MG	0.47448
ENSMUSG00000066510	Ankdd1a	ankyrin repeat and death domain containing 1A [So	0.47456
ENSMUSG00000089646	Gm15796	predicted gene 15796 [Source:MGI Symbol;Acc:MG	0.47456
ENSMUSG00000006463	Zdhhc24	zinc finger, DHHC domain containing 24 [Source:M	0.47468

ENSMUSG00000073242	Gm10485	predicted gene 10485 [Source:MGI Symbol;Acc:MG	0.47469
ENSMUSG00000079562	Maea	macrophage erythroblast attacher [Source:MGI Syr	0.47479
ENSMUSG00000035401	2210018M11Rik	RIKEN cDNA 2210018M11 gene [Source:MGI Symb	0.4748
ENSMUSG00000038827	BC026590	cDNA sequence BC026590 [Source:MGI Symbol;Acc	0.47481
ENSMUSG00000030353	Tead4	TEA domain family member 4 [Source:MGI Symbol;	0.47484
ENSMUSG00000088763	Gm24391	predicted gene, 24391 [Source:MGI Symbol;Acc:M	0.47488
ENSMUSG00000027797	Dclk1	doublecortin-like kinase 1 [Source:MGI Symbol;Acc	0.47503
ENSMUSG00000023992	Trem2	triggering receptor expressed on myeloid cells 2 [S	0.47504
ENSMUSG00000033446	Lpar6	lysophosphatidic acid receptor 6 [Source:MGI Symb	0.47526
ENSMUSG00000025875	Tspan17	tetraspanin 17 [Source:MGI Symbol;Acc:MGI:19215	0.4753
ENSMUSG00000085812	Gm15843	predicted gene 15843 [Source:MGI Symbol;Acc:MG	0.47533
ENSMUSG00000047250	Ptgs1	prostaglandin-endoperoxide synthase 1 [Source:M	0.47544
ENSMUSG00000087416	Gm15906	predicted gene 15906 [Source:MGI Symbol;Acc:MG	0.47548
ENSMUSG00000096751	Gm21971	predicted gene 21971 [Source:MGI Symbol;Acc:MG	0.47555
ENSMUSG00000033021	Gmppa	GDP-mannose pyrophosphorylase A [Source:MGI S	0.47559
ENSMUSG00000097457	2310031A07Rik	RIKEN cDNA 2310031A07 gene [Source:MGI Symbo	0.47559
ENSMUSG00000041958	Pigs	phosphatidylinositol glycan anchor biosynthesis, cl	0.47565
ENSMUSG00000038072	Galnt11	UDP-N-acetyl-alpha-D-galactosamine:polypeptide N	0.47566
ENSMUSG00000035877	Zhx3	zinc fingers and homeoboxes 3 [Source:MGI Symbo	0.4757
ENSMUSG00000093726	Gm20667	predicted gene 20667 [Source:MGI Symbol;Acc:MG	0.47582
ENSMUSG00000064590	Gm22301	predicted gene, 22301 [Source:MGI Symbol;Acc:M	0.47585
ENSMUSG00000087505	Gm15241	predicted gene 15241 [Source:MGI Symbol;Acc:MG	0.47586
ENSMUSG00000090655	Vmn2r120	vomeronasal 2, receptor 120 [Source:MGI Symbol;	0.47586
ENSMUSG00000087411	Gm16048	predicted gene 16048 [Source:MGI Symbol;Acc:MG	0.47588
ENSMUSG00000080701	Gm12340	predicted gene 12340 [Source:MGI Symbol;Acc:MG	0.4759
ENSMUSG00000082204	Gm7722	predicted gene 7722 [Source:MGI Symbol;Acc:MGI	0.4759
ENSMUSG00000086552	A730090H04Rik	RIKEN cDNA A730090H04 gene [Source:MGI Symbo	0.47591
ENSMUSG00000059108	Ifitm6	interferon induced transmembrane protein 6 [Sour	0.47592
ENSMUSG00000092268	Gm20540	predicted gene 20540 [Source:MGI Symbol;Acc:MG	0.47612
ENSMUSG00000033965	Slc16a2	solute carrier family 16 (monocarboxylic acid trans	0.47618
ENSMUSG00000044296	Zfp879	zinc finger protein 879 [Source:MGI Symbol;Acc:M	0.47624
ENSMUSG00000061718	Ppp1r1b	protein phosphatase 1, regulatory (inhibitor) subur	0.47637
ENSMUSG00000026035	Ppil3	peptidylprolyl isomerase (cyclophilin)-like 3 [Source	0.47639
ENSMUSG00000028060	2810403A07Rik	RIKEN cDNA 2810403A07 gene [Source:MGI Symbo	0.4764
ENSMUSG00000031255	Sytl4	synaptotagmin-like 4 [Source:MGI Symbol;Acc:MGI	0.4764
ENSMUSG00000023949	Tcte1	t-complex-associated testis expressed 1 [Source:M	0.47649
ENSMUSG00000035129	Gm6781	predicted gene 6781 [Source:MGI Symbol;Acc:MGI	0.47652
ENSMUSG00000037916	Ndufv1	NADH dehydrogenase (ubiquinone) flavoprotein 1	0.47667
ENSMUSG00000068062	Gm14164	predicted gene 14164 [Source:MGI Symbol;Acc:MG	0.47676
ENSMUSG00000041815	Poldip3	polymerase (DNA-directed), delta interacting prote	0.47678
ENSMUSG00000071343	Gm10327	predicted pseudogene 10327 [Source:MGI Symbol;	0.4768
ENSMUSG00000021368	Tbc1d7	TBC1 domain family, member 7 [Source:MGI Symb	0.47682
ENSMUSG00000060429	Sntb1	syntrophin, basic 1 [Source:MGI Symbol;Acc:MGI:1	0.47692
ENSMUSG00000081553	Gm12699	predicted gene 12699 [Source:MGI Symbol;Acc:MG	0.47693
ENSMUSG00000079165	Sap25	sin3 associated polypeptide [Source:MGI Symbol;A	0.47695
ENSMUSG00000081570	Gm12192	predicted gene 12192 [Source:MGI Symbol;Acc:MG	0.47695
ENSMUSG00000001761	Smo	smoothened homolog (Drosophila) [Source:MGI Sy	0.47696

ENSMUSG00000045273	Cenph	centromere protein H [Source:MGI Symbol;Acc:MG	0.47702
ENSMUSG00000046490	Rnf222	ring finger protein 222 [Source:MGI Symbol;Acc:M(	0.47704
ENSMUSG00000083465	Gm11652	predicted gene 11652 [Source:MGI Symbol;Acc:MG	0.47704
ENSMUSG00000022596	Slurp1	secreted Ly6/Plaur domain containing 1 [Source:M(	0.47707
ENSMUSG00000031596	Slc7a2	solute carrier family 7 (cationic amino acid transpo	0.47711
ENSMUSG00000050138	Kcnk12	potassium channel, subfamily K, member 12 [Sourc	0.47716
ENSMUSG00000085578	Gm11527	predicted gene 11527 [Source:MGI Symbol;Acc:MG	0.47722
ENSMUSG00000036006	Fam65b	family with sequence similarity 65, member B [Sou	0.47723
ENSMUSG00000074195	AI747448	expressed sequence AI747448 [Source:MGI Symbol	0.47723
ENSMUSG00000050978	Gm5215	predicted gene 5215 [Source:MGI Symbol;Acc:MGI:	0.47725
ENSMUSG00000054083	Capn12	calpain 12 [Source:MGI Symbol;Acc:MGI:1891369]	0.47726
ENSMUSG00000024771	Lipk	lipase, family member K [Source:MGI Symbol;Acc:M	0.47728
ENSMUSG00000033520	Idi2	isopentenyl-diphosphate delta isomerase 2 [Source	0.47729
ENSMUSG00000066838	Zfp772	zinc finger protein 772 [Source:MGI Symbol;Acc:M(	0.47731
ENSMUSG00000086159	Gm13025	predicted gene 13025 [Source:MGI Symbol;Acc:MG	0.47731
ENSMUSG00000022315	Slc30a8	solute carrier family 30 (zinc transporter), member	0.47732
ENSMUSG00000085091	E230015J15Rik	RIKEN cDNA E230015J15 gene [Source:MGI Symbol	0.47733
ENSMUSG00000078921	Tgtp2	T cell specific GTPase 2 [Source:MGI Symbol;Acc:M	0.47749
ENSMUSG00000084850	Gm12092	predicted gene 12092 [Source:MGI Symbol;Acc:MG	0.4775
ENSMUSG00000000711	Rab5b	RAB5B, member RAS oncogene family [Source:MGI	0.47751
ENSMUSG00000027994	Ccdc109b	coiled-coil domain containing 109B [Source:MGI Sy	0.47753
ENSMUSG00000033355	Rtp4	receptor transporter protein 4 [Source:MGI Symbo	0.47759
ENSMUSG00000001583	Tnk1	tyrosine kinase, non-receptor, 1 [Source:MGI Symb	0.47768
ENSMUSG00000028126	Pip5k1a	phosphatidylinositol-4-phosphate 5-kinase, type 1 a	0.47769
ENSMUSG00000090003	Gm15882	predicted gene 15882 [Source:MGI Symbol;Acc:MG	0.47771
ENSMUSG00000042369	Rbm45	RNA binding motif protein 45 [Source:MGI Symbol;	0.47772
ENSMUSG00000024032	Tff1	trefoil factor 1 [Source:MGI Symbol;Acc:MGI:88135	0.47785
ENSMUSG00000074625	Arhgap40	Rho GTPase activating protein 40 [Source:MGI Sym	0.47795
ENSMUSG00000013495	Tmem175	transmembrane protein 175 [Source:MGI Symbol;A	0.478
ENSMUSG00000040471	Ggt6	gamma-glutamyltransferase 6 [Source:MGI Symbol	0.47801
ENSMUSG00000095564	Gm20474	predicted gene 20474 [Source:MGI Symbol;Acc:MG	0.47801
ENSMUSG00000082036	Gm13724	predicted gene 13724 [Source:MGI Symbol;Acc:MG	0.47805
ENSMUSG00000020697	Lig3	ligase III, DNA, ATP-dependent [Source:MGI Symbo	0.47808
ENSMUSG00000079685	Ulbp1	UL16 binding protein 1 [Source:MGI Symbol;Acc:M(	0.47809
ENSMUSG00000056917	Sipa1	signal-induced proliferation associated gene 1 [Sou	0.47814
ENSMUSG00000094626	Cecr6	cat eye syndrome chromosome region, candidate 6	0.47825
ENSMUSG00000023235	Ccl25	chemokine (C-C motif) ligand 25 [Source:MGI Symb	0.47826
ENSMUSG00000081487	Gm13689	predicted gene 13689 [Source:MGI Symbol;Acc:MG	0.47827
ENSMUSG00000096145	Vkorc1	vitamin K epoxide reductase complex, subunit 1 [So	0.47834
ENSMUSG00000024399	Ltb	lymphotoxin B [Source:MGI Symbol;Acc:MGI:10479	0.47838
ENSMUSG00000086368	Gm13830	predicted gene 13830 [Source:MGI Symbol;Acc:MG	0.47848
ENSMUSG00000035311	Gnptab	N-acetylglucosamine-1-phosphate transferase, alph	0.47853
ENSMUSG00000034645	Zyg11a	zyg-11 family member A, cell cycle regulator [Sourc	0.47865
ENSMUSG00000086326	Gm13200	predicted gene 13200 [Source:MGI Symbol;Acc:MG	0.47867
ENSMUSG00000059288	Cdyl	chromodomain protein, Y chromosome-like [Source	0.47868
ENSMUSG00000097571	Jpx	Jpx transcript, Xist activator (non-protein coding) [S	0.47891
ENSMUSG00000026970	Rbms1	RNA binding motif, single stranded interacting prot	0.47894

ENSMUSG00000041012	Cmtm8	CKLF-like MARVEL transmembrane domain contain	0.47897
ENSMUSG00000079259	Trim71	tripartite motif-containing 71 [Source:MGI Symbol;	0.47901
ENSMUSG00000005566	Trim28	tripartite motif-containing 28 [Source:MGI Symbol;	0.4791
ENSMUSG00000029167	Ppargc1a	peroxisome proliferative activated receptor, gamm	0.47911
ENSMUSG00000003269	Cyth2	cytohesin 2 [Source:MGI Symbol;Acc:MGI:1334255	0.47913
ENSMUSG00000021678	F2rl1	coagulation factor II (thrombin) receptor-like 1 [Sou	0.47917
ENSMUSG00000011158	Brf1	BRF1 homolog, subunit of RNA polymerase III trans	0.47921
ENSMUSG00000053117	E330013P04Rik	RIKEN cDNA E330013P04 gene [Source:MGI Symbo	0.47922
ENSMUSG00000047189	Gm9818	predicted gene 9818 [Source:MGI Symbol;Acc:MGI:	0.47927
ENSMUSG00000046636	Gm7729	predicted gene 7729 [Source:MGI Symbol;Acc:MGI:	0.4793
ENSMUSG00000071646	Mta2	metastasis-associated gene family, member 2 [Sou	0.47935
ENSMUSG00000021895	Arhgef3	Rho guanine nucleotide exchange factor (GEF) 3 [S	0.47936
ENSMUSG00000032333	Stoml1	stomatin-like 1 [Source:MGI Symbol;Acc:MGI:1916	0.47936
ENSMUSG00000019433	Gipc1	GIPC PDZ domain containing family, member 1 [Sou	0.47951
ENSMUSG00000021460	Auh	AU RNA binding protein/enoyl-coenzyme A hydrata	0.4797
ENSMUSG00000054676	1600014C10Rik	RIKEN cDNA 1600014C10 gene [Source:MGI Symbo	0.47974
ENSMUSG00000014959	Gorasp2	golgi reassembly stacking protein 2 [Source:MGI Sy	0.47977
ENSMUSG00000039759	Thap3	THAP domain containing, apoptosis associated prot	0.47985
ENSMUSG00000009471	Myod1	myogenic differentiation 1 [Source:MGI Symbol;Ac	0.47987
ENSMUSG00000054675	Tmem119	transmembrane protein 119 [Source:MGI Symbol;A	0.47991
ENSMUSG00000082826	Gm6097	predicted pseudogene 6097 [Source:MGI Symbol;A	0.47994
ENSMUSG00000040860	Crocc	ciliary rootlet coiled-coil, rootletin [Source:MGI Syn	0.47998
ENSMUSG00000081221	Gm14760	predicted gene 14760 [Source:MGI Symbol;Acc:MG	0.48
ENSMUSG00000023940	Sgol1	shugoshin-like 1 (S. pombe) [Source:MGI Symbol;A	0.48049
ENSMUSG00000084184	Gm13474	predicted gene 13474 [Source:MGI Symbol;Acc:MG	0.4805
ENSMUSG00000000183	Fgf6	fibroblast growth factor 6 [Source:MGI Symbol;Acc	0.48055
ENSMUSG00000097216	4932441J04Rik	RIKEN cDNA 4932441J04 gene [Source:MGI Symbol	0.48057
ENSMUSG00000032652	Crebl2	cAMP responsive element binding protein-like 2 [S	0.48061
ENSMUSG00000097796	Gm16702	predicted gene, 16702 [Source:MGI Symbol;Acc:M	0.48061
ENSMUSG00000018733	Pex12	peroxisomal biogenesis factor 12 [Source:MGI Sym	0.48063
ENSMUSG00000020018	Snrpf	small nuclear ribonucleoprotein polypeptide F [Sou	0.48068
ENSMUSG00000039512	Uhrf1bp1	UHRF1 (ICBP90) binding protein 1 [Source:MGI Sym	0.48069
ENSMUSG00000029602	Rasal1	RAS protein activator like 1 (GAP1 like) [Source:MG	0.48074
ENSMUSG00000040746	Rnf167	ring finger protein 167 [Source:MGI Symbol;Acc:M	0.48081
ENSMUSG00000088059	Mir1949	microRNA 1949 [Source:MGI Symbol;Acc:MGI:3837	0.48081
ENSMUSG00000087563	Gm14205	predicted gene 14205 [Source:MGI Symbol;Acc:MG	0.48084
ENSMUSG00000015501	Hivep2	human immunodeficiency virus type I enhancer bin	0.48091
ENSMUSG00000086568	9130020K20Rik	RIKEN cDNA 9130020K20 gene [Source:MGI Symbo	0.481
ENSMUSG00000001672	Marveld3	MARVEL (membrane-associating) domain containir	0.48103
ENSMUSG00000085913	Gm15601	predicted gene 15601 [Source:MGI Symbol;Acc:MG	0.48103
ENSMUSG00000052730	Gm5111	predicted gene 5111 [Source:MGI Symbol;Acc:MGI:	0.48105
ENSMUSG00000032388	Spg21	spastic paraplegia 21 homolog (human) [Source:M	0.48114
ENSMUSG00000014748	Tex261	testis expressed gene 261 [Source:MGI Symbol;Acc	0.48118
ENSMUSG00000039007	Cpq	carboxypeptidase Q [Source:MGI Symbol;Acc:MGI:	0.48122
ENSMUSG00000018547	Pip4k2b	phosphatidylinositol-5-phosphate 4-kinase, type II,	0.48132
ENSMUSG00000038742	Angptl6	angiopoietin-like 6 [Source:MGI Symbol;Acc:MGI:1	0.48137
ENSMUSG00000069136	A130006I12Rik	RIKEN cDNA A130006I12 gene [Source:MGI Symbol	0.48139

ENSMUSG00000024960	Plcb3	phospholipase C, beta 3 [Source:MGI Symbol;Acc:M	0.48142
ENSMUSG00000039133	9330171B17Rik	RIKEN cDNA 9330171B17 gene [Source:MGI Symbo	0.48143
ENSMUSG00000037140	Tas2r108	taste receptor, type 2, member 108 [Source:MGI Sy	0.48148
ENSMUSG00000051650	B3gnt2	UDP-GlcNAc:betaGal beta-1,3-N-acetylglucosaminy	0.48151
ENSMUSG00000021919	Chat	choline acetyltransferase [Source:MGI Symbol;Acc:	0.48155
ENSMUSG00000030351	Tspan11	tetraspanin 11 [Source:MGI Symbol;Acc:MGI:19157	0.48157
ENSMUSG00000028358	Zfp618	zinc finger protein 618 [Source:MGI Symbol;Acc:MG	0.48158
ENSMUSG00000036002	Fam214b	family with sequence similarity 214, member B [Sou	0.48159
ENSMUSG00000028223	Decr1	2,4-dienoyl CoA reductase 1, mitochondrial [Source	0.48171
ENSMUSG00000074001	Klhl40	kelch-like 40 [Source:MGI Symbol;Acc:MGI:191958	0.48178
ENSMUSG00000022949	Clic6	chloride intracellular channel 6 [Source:MGI Symbo	0.48181
ENSMUSG00000077879	Gm23912	predicted gene, 23912 [Source:MGI Symbol;Acc:MG	0.48182
ENSMUSG00000083125	Gm16203	predicted gene 16203 [Source:MGI Symbol;Acc:MG	0.48182
ENSMUSG00000048686	Hmgb4	high-mobility group box 4 [Source:MGI Symbol;Acc	0.48184
ENSMUSG00000089536	Scarna3a	small Cajal body-specific RNA 3A [Source:MGI Symb	0.48187
ENSMUSG00000020321	Mdh1	malate dehydrogenase 1, NAD (soluble) [Source:MG	0.48189
ENSMUSG00000025538	Sumf2	sulfatase modifying factor 2 [Source:MGI Symbol;A	0.48189
ENSMUSG00000050786	Ccdc126	coiled-coil domain containing 126 [Source:MGI Sym	0.48193
ENSMUSG00000085802	Gm16059	predicted gene 16059 [Source:MGI Symbol;Acc:MG	0.48197
ENSMUSG00000031837	Necab2	N-terminal EF-hand calcium binding protein 2 [Sour	0.4821
ENSMUSG00000026430	Rassf5	Ras association (RalGDS/AF-6) domain family memb	0.48233
ENSMUSG00000083827	Gm15712	predicted gene 15712 [Source:MGI Symbol;Acc:MG	0.48239
ENSMUSG00000051596	Otop1	otopetrin 1 [Source:MGI Symbol;Acc:MGI:2388363]	0.48252
ENSMUSG00000022305	Lrp12	low density lipoprotein-related protein 12 [Source:	0.48284
ENSMUSG00000043008	Klhl6	kelch-like 6 [Source:MGI Symbol;Acc:MGI:2686922]	0.48285
ENSMUSG00000033576	Apol6	apolipoprotein L 6 [Source:MGI Symbol;Acc:MGI:19	0.48288
ENSMUSG00000029001	Fbxo44	F-box protein 44 [Source:MGI Symbol;Acc:MGI:135	0.48299
ENSMUSG00000083422	Gm15604	predicted gene 15604 [Source:MGI Symbol;Acc:MG	0.48301
ENSMUSG00000081257	Gm13607	predicted gene 13607 [Source:MGI Symbol;Acc:MG	0.48303
ENSMUSG00000019943	Atp2b1	ATPase, Ca++ transporting, plasma membrane 1 [So	0.48316
ENSMUSG00000029447	Cct6a	chaperonin containing Tcp1, subunit 6a (zeta) [Sour	0.4832
ENSMUSG00000084399	Gm15666	predicted gene 15666 [Source:MGI Symbol;Acc:MG	0.4832
ENSMUSG00000064844	Gm26202	predicted gene, 26202 [Source:MGI Symbol;Acc:MG	0.48322
ENSMUSG00000045095	Magi1	membrane associated guanylate kinase, WW and P	0.48328
ENSMUSG00000002699	Lcp2	lymphocyte cytosolic protein 2 [Source:MGI Symbo	0.48332
ENSMUSG00000060992	Copz1	coatomer protein complex, subunit zeta 1 [Source:	0.48332
ENSMUSG00000035183	Slc24a5	solute carrier family 24, member 5 [Source:MGI Syr	0.48338
ENSMUSG00000031917	Nip7	nuclear import 7 homolog (S. cerevisiae) [Source:M	0.48351
ENSMUSG00000091472	Gm3739	predicted gene 3739 [Source:MGI Symbol;Acc:MGI:	0.48355
ENSMUSG00000061086	Myl4	myosin, light polypeptide 4 [Source:MGI Symbol;Ac	0.48359
ENSMUSG00000076430	Hus1b	Hus1 homolog b (S. pombe) [Source:MGI Symbol;A	0.4836
ENSMUSG00000098178	Gm26924	predicted gene, 26924 [Source:MGI Symbol;Acc:MG	0.48365
ENSMUSG00000018604	Tbx3	T-box 3 [Source:MGI Symbol;Acc:MGI:98495]	0.48377
ENSMUSG00000004462	Tbccd1	TBCC domain containing 1 [Source:MGI Symbol;Acc	0.48383
ENSMUSG00000085523	Gm15945	predicted gene 15945 [Source:MGI Symbol;Acc:MG	0.48393
ENSMUSG00000007480	Mc5r	melanocortin 5 receptor [Source:MGI Symbol;Acc:M	0.48406
ENSMUSG00000009941	Nxf2	nuclear RNA export factor 2 [Source:MGI Symbol;A	0.48407

ENSMUSG00000044034	Npb	neuropeptide B [Source:MGI Symbol;Acc:MGI:2387	0.48409
ENSMUSG00000037443	Cep85	centrosomal protein 85 [Source:MGI Symbol;Acc:M	0.48416
ENSMUSG00000072946	Ptgr2	prostaglandin reductase 2 [Source:MGI Symbol;Acc	0.48417
ENSMUSG00000031576	Kcnu1	potassium channel, subfamily U, member 1 [Source	0.48423
ENSMUSG00000024997	Prdx3	peroxiredoxin 3 [Source:MGI Symbol;Acc:MGI:8803	0.48428
ENSMUSG00000030218	Mgp	matrix Gla protein [Source:MGI Symbol;Acc:MGI:96	0.48432
ENSMUSG00000074113	Gm10629	predicted gene 10629 [Source:MGI Symbol;Acc:MG	0.48437
ENSMUSG00000003234	Abcf3	ATP-binding cassette, sub-family F (GCN20), memb	0.48438
ENSMUSG00000029535	Triap1	TP53 regulated inhibitor of apoptosis 1 [Source:MG	0.48439
ENSMUSG00000020287	Mpg	N-methylpurine-DNA glycosylase [Source:MGI Sym	0.48443
ENSMUSG00000042570	Mier2	mesoderm induction early response 1, family mem	0.48443
ENSMUSG00000044505	Lingo4	leucine rich repeat and Ig domain containing 4 [Sou	0.48444
ENSMUSG00000013155	Enkd1	enkurin domain containing 1 [Source:MGI Symbol;A	0.48448
ENSMUSG00000032869	Psmf1	proteasome (prosome, macropain) inhibitor subuni	0.4845
ENSMUSG00000048087	Gm4737	predicted gene 4737 [Source:MGI Symbol;Acc:MGI:	0.48471
ENSMUSG00000023349	Clec4n	C-type lectin domain family 4, member n [Source:M	0.4848
ENSMUSG00000024379	Tslp	thymic stromal lymphopoietin [Source:MGI Symbol	0.48481
ENSMUSG00000062611	Rps3a2	ribosomal protein S3A2 [Source:MGI Symbol;Acc:M	0.48484
ENSMUSG00000039462	Col10a1	collagen, type X, alpha 1 [Source:MGI Symbol;Acc:M	0.48485
ENSMUSG00000038916	Soga3	SOGA family member 3 [Source:MGI Symbol;Acc:M	0.48499
ENSMUSG00000076435	Acsf2	acyl-CoA synthetase family member 2 [Source:MGI	0.48507
ENSMUSG00000037172	E330009J07Rik	RIKEN cDNA E330009J07 gene [Source:MGI Symbol	0.48508
ENSMUSG00000026032	Ndufb3	NADH dehydrogenase (ubiquinone) 1 beta subcom	0.48513
ENSMUSG00000034108	Ccs	copper chaperone for superoxide dismutase [Sourc	0.48513
ENSMUSG00000077380	Gm22661	predicted gene, 22661 [Source:MGI Symbol;Acc:MG	0.48514
ENSMUSG00000025329	Padi1	peptidyl arginine deiminase, type I [Source:MGI Sym	0.48516
ENSMUSG00000030983	Bccip	BRCA2 and CDKN1A interacting protein [Source:MG	0.48516
ENSMUSG00000086108	Gm5602	predicted gene 5602 [Source:MGI Symbol;Acc:MGI:	0.48545
ENSMUSG00000084980	4921508A21Rik	RIKEN cDNA 4921508A21 gene [Source:MGI Symbo	0.48585
ENSMUSG00000031513	Leprotl1	leptin receptor overlapping transcript-like 1 [Source	0.4859
ENSMUSG00000034525	BC018507	cDNA sequence BC018507 [Source:MGI Symbol;Acc	0.48606
ENSMUSG00000098138	Gm9081	predicted gene 9081 [Source:MGI Symbol;Acc:MGI:	0.48617
ENSMUSG00000052406	Rexo4	REX4, RNA exonuclease 4 homolog (S. cerevisiae) [S	0.4864
ENSMUSG00000022297	Fzd6	frizzled homolog 6 (Drosophila) [Source:MGI Symbo	0.48643
ENSMUSG00000082279	Gm12870	predicted gene 12870 [Source:MGI Symbol;Acc:MG	0.48653
ENSMUSG00000050668	Gpatch11	G patch domain containing 11 [Source:MGI Symbol	0.4866
ENSMUSG00000034593	Myo5a	myosin VA [Source:MGI Symbol;Acc:MGI:105976]	0.48665
ENSMUSG00000086096	Gm12688	predicted gene 12688 [Source:MGI Symbol;Acc:MG	0.48666
ENSMUSG00000071652	Ints5	integrator complex subunit 5 [Source:MGI Symbol;A	0.48667
ENSMUSG00000038217	Tlcd2	TLC domain containing 2 [Source:MGI Symbol;Acc:M	0.48671
ENSMUSG00000037868	Egr2	early growth response 2 [Source:MGI Symbol;Acc:M	0.48673
ENSMUSG00000026849	Tor1a	torsin family 1, member A (torsin A) [Source:MGI Sy	0.4868
ENSMUSG00000076438	Oxct2b	3-oxoacid CoA transferase 2B [Source:MGI Symbol;	0.48685
ENSMUSG00000055416	Fam136b-ps	family with sequence similarity 136, member B, pse	0.48716
ENSMUSG00000062456	Rpl9-ps6	ribosomal protein L9, pseudogene 6 [Source:MGI Sy	0.48716
ENSMUSG00000031520	Vegfc	vascular endothelial growth factor C [Source:MGI S	0.4872
ENSMUSG00000035455	Fignl1	fidgetin-like 1 [Source:MGI Symbol;Acc:MGI:18906	0.48722

ENSMUSG00000034903	Cobll1	Cobl-like 1 [Source:MGI Symbol;Acc:MGI:2442894]	0.48727
ENSMUSG00000072492	Gm10364	predicted pseudogene 10364 [Source:MGI Symbol;]	0.48727
ENSMUSG00000031626	Sorbs2	sorbin and SH3 domain containing 2 [Source:MGI S	0.48736
ENSMUSG00000036185	Sapcd1	suppressor APC domain containing 1 [Source:MGI S	0.48736
ENSMUSG00000086484	Nron	non-protein coding RNA, repressor of NFAT [Source	0.48742
ENSMUSG00000040964	Arhgef10l	Rho guanine nucleotide exchange factor (GEF) 10-li	0.48743
ENSMUSG00000075599	Smok3a	sperm motility kinase 3A [Source:MGI Symbol;Acc:I	0.48763
ENSMUSG00000028016	Ints12	integrator complex subunit 12 [Source:MGI Symbol	0.48775
ENSMUSG00000035954	Dock4	dedicator of cytokinesis 4 [Source:MGI Symbol;Acc:	0.48775
ENSMUSG00000028100	Nudt17	nudix (nucleoside diphosphate linked moiety X)-typ	0.48777
ENSMUSG00000038299	Wdr36	WD repeat domain 36 [Source:MGI Symbol;Acc:MG	0.4878
ENSMUSG00000027605	Acss2	acyl-CoA synthetase short-chain family member 2 [	0.48795
ENSMUSG00000056158	Car10	carbonic anhydrase 10 [Source:MGI Symbol;Acc:M	0.48808
ENSMUSG00000085941	Gm11201	predicted gene 11201 [Source:MGI Symbol;Acc:MG	0.48809
ENSMUSG00000065509	Mir421	microRNA 421 [Source:MGI Symbol;Acc:MGI:37185	0.48811
ENSMUSG00000004633	Chn2	chimerin (chimaerin) 2 [Source:MGI Symbol;Acc:M	0.4882
ENSMUSG00000083391	Gm14148	predicted gene 14148 [Source:MGI Symbol;Acc:MG	0.48821
ENSMUSG00000096975	Gm16386	predicted gene 16386 [Source:MGI Symbol;Acc:MG	0.48824
ENSMUSG00000021306	Gpr137b	G protein-coupled receptor 137B [Source:MGI Sym	0.48827
ENSMUSG00000047434	Xxylt1	xyloside xylosyltransferase 1 [Source:MGI Symbol;A	0.48827
ENSMUSG00000022706	Mrpl40	mitochondrial ribosomal protein L40 [Source:MGI S	0.48831
ENSMUSG00000030621	Me3	malic enzyme 3, NADP(+)-dependent, mitochondria	0.48832
ENSMUSG00000026360	Rgs2	regulator of G-protein signaling 2 [Source:MGI Sym	0.48833
ENSMUSG00000002588	Pon1	paraoxonase 1 [Source:MGI Symbol;Acc:MGI:10329	0.48844
ENSMUSG00000022545	Ercc4	excision repair cross-complementing rodent repair	0.48871
ENSMUSG00000086134	Gm16159	predicted gene 16159 [Source:MGI Symbol;Acc:MG	0.48877
ENSMUSG00000081992	Gm13408	predicted gene 13408 [Source:MGI Symbol;Acc:MG	0.48879
ENSMUSG00000025608	Podxl	podocalyxin-like [Source:MGI Symbol;Acc:MGI:135	0.48884
ENSMUSG00000034639	Setmar	SET domain without mariner transposase fusion [So	0.48887
ENSMUSG00000037143	4930529M08Rik	RIKEN cDNA 4930529M08 gene [Source:MGI Symb	0.48888
ENSMUSG00000050148	Ubqln2	ubiquilin 2 [Source:MGI Symbol;Acc:MGI:1860283]	0.48901
ENSMUSG00000052837	Junb	Jun-B oncogene [Source:MGI Symbol;Acc:MGI:9664	0.48905
ENSMUSG00000041809	Efhc1	EF-hand domain (C-terminal) containing 1 [Source:I	0.48906
ENSMUSG00000046280	She	src homology 2 domain-containing transforming pr	0.48906
ENSMUSG00000087277	2010013B24Rik	RIKEN cDNA 2010013B24 gene [Source:MGI Symbo	0.4891
ENSMUSG00000059058	Tma7-ps	translational machinery associated 7 homolog (S. c	0.48925
ENSMUSG00000040618	Pck2	phosphoenolpyruvate carboxykinase 2 (mitochond	0.4893
ENSMUSG00000048191	Muc6	mucin 6, gastric [Source:MGI Symbol;Acc:MGI:2663	0.4893
ENSMUSG00000097619	4833422M21Rik	RIKEN cDNA 4833422M21 gene [Source:MGI Symb	0.48934
ENSMUSG00000025533	Asl	argininosuccinate lyase [Source:MGI Symbol;Acc:M	0.48938
ENSMUSG00000097658	Gm16755	predicted gene, 16755 [Source:MGI Symbol;Acc:M	0.48943
ENSMUSG00000031452	1700029H14Rik	RIKEN cDNA 1700029H14 gene [Source:MGI Symbo	0.48954
ENSMUSG00000022554	Fam203a	family with sequence similarity 203, member A [So	0.48961
ENSMUSG00000028969	Cdk5	cyclin-dependent kinase 5 [Source:MGI Symbol;Acc	0.48967
ENSMUSG00000043518	Rai2	retinoic acid induced 2 [Source:MGI Symbol;Acc:M	0.48978
ENSMUSG00000034391	Fbxo15	F-box protein 15 [Source:MGI Symbol;Acc:MGI:135	0.48989
ENSMUSG00000097707	Gm26742	predicted gene, 26742 [Source:MGI Symbol;Acc:M	0.48994

ENSMUSG00000097862	Gm26859	predicted gene, 26859 [Source:MGI Symbol;Acc:MG	0.49002
ENSMUSG00000066682	Pilrb2	paired immunoglobin-like type 2 receptor beta 2 [S	0.49004
ENSMUSG00000047466	8030462N17Rik	RIKEN cDNA 8030462N17 gene [Source:MGI Symbo	0.49015
ENSMUSG00000043126	D830039M14Rik	RIKEN cDNA D830039M14 gene [Source:MGI Symb	0.49016
ENSMUSG00000004233	Wars2	tryptophanyl tRNA synthetase 2 (mitochondrial) [So	0.49018
ENSMUSG00000097305	Gm17276	predicted gene, 17276 [Source:MGI Symbol;Acc:MG	0.49018
ENSMUSG00000009739	Pou6f1	POU domain, class 6, transcription factor 1 [Source	0.49019
ENSMUSG00000028640	Tfap2c	transcription factor AP-2, gamma [Source:MGI Sym	0.49024
ENSMUSG00000061119	Prcp	prolylcarboxypeptidase (angiotensinase C) [Source:	0.49028
ENSMUSG00000004952	Rasa4	RAS p21 protein activator 4 [Source:MGI Symbol;Ac	0.49034
ENSMUSG00000030878	Cdr2	cerebellar degeneration-related 2 [Source:MGI Sym	0.49035
ENSMUSG00000084497	Gm22107	predicted gene, 22107 [Source:MGI Symbol;Acc:MG	0.49036
ENSMUSG00000004044	Ptrf	polymerase I and transcript release factor [Source:I	0.49044
ENSMUSG00000037922	Bank1	B cell scaffold protein with ankyrin repeats 1 [Sourc	0.49047
ENSMUSG00000085457	1110046J04Rik	RIKEN cDNA 1110046J04 gene [Source:MGI Symbol	0.49049
ENSMUSG00000021276	Cinp	cyclin-dependent kinase 2 interacting protein [Sour	0.49059
ENSMUSG00000061878	Sphk1	sphingosine kinase 1 [Source:MGI Symbol;Acc:MGI:	0.49061
ENSMUSG00000030877	4933427G17Rik	RIKEN cDNA 4933427G17 gene [Source:MGI Symbo	0.49071
ENSMUSG00000086671	Gm13618	predicted gene 13618 [Source:MGI Symbol;Acc:MG	0.49078
ENSMUSG00000033434	Gtpbp6	GTP binding protein 6 (putative) [Source:MGI Symb	0.49081
ENSMUSG00000041346	Wrap53	WD repeat containing, antisense to Trp53 [Source:I	0.49092
ENSMUSG00000061477	Rps7	ribosomal protein S7 [Source:MGI Symbol;Acc:MGI	0.49095
ENSMUSG00000085043	Gm13184	predicted gene 13184 [Source:MGI Symbol;Acc:MG	0.49095
ENSMUSG00000034987	Hrh2	histamine receptor H2 [Source:MGI Symbol;Acc:MG	0.49097
ENSMUSG00000020564	Atxn7l1	ataxin 7-like 1 [Source:MGI Symbol;Acc:MGI:35844	0.49101
ENSMUSG00000000794	Kcnn3	potassium intermediate/small conductance calcium	0.4911
ENSMUSG00000057522	Spop	speckle-type POZ protein [Source:MGI Symbol;Acc:	0.49124
ENSMUSG00000071567	A830039N20Rik	RIKEN cDNA A830039N20 gene [Source:MGI Symbo	0.49126
ENSMUSG00000018377	Vezf1	vascular endothelial zinc finger 1 [Source:MGI Symb	0.49147
ENSMUSG00000070000	Fcho1	FCH domain only 1 [Source:MGI Symbol;Acc:MGI:1	0.49147
ENSMUSG00000098188	Sowahc	sosondowah ankyrin repeat domain family membe	0.49151
ENSMUSG00000067292	Gm10203	predicted gene 10203 [Source:MGI Symbol;Acc:MG	0.49152
ENSMUSG00000054733	Msra	methionine sulfoxide reductase A [Source:MGI Sym	0.49163
ENSMUSG00000031935	Med17	mediator complex subunit 17 [Source:MGI Symbol;	0.49175
ENSMUSG00000005873	Reep5	receptor accessory protein 5 [Source:MGI Symbol;A	0.49183
ENSMUSG00000001027	Scn4a	sodium channel, voltage-gated, type IV, alpha [Sour	0.49185
ENSMUSG00000020395	Itk	IL2 inducible T cell kinase [Source:MGI Symbol;Acc:	0.49197
ENSMUSG00000023966	Rsph9	radial spoke head 9 homolog (Chlamydomonas) [So	0.49203
ENSMUSG00000030276	Ttll3	tubulin tyrosine ligase-like family, member 3 [Sourc	0.49216
ENSMUSG00000079429	Mroh2a	maestro heat-like repeat family member 2A [Sourc	0.49217
ENSMUSG00000052298	Cdc42se2	CDC42 small effector 2 [Source:MGI Symbol;Acc:MG	0.49235
ENSMUSG00000026436	Elk4	ELK4, member of ETS oncogene family [Source:MGI	0.49242
ENSMUSG00000026335	Pam	peptidylglycine alpha-amidating monooxygenase [S	0.49244
ENSMUSG00000029868	Trpv6	transient receptor potential cation channel, subfam	0.49255
ENSMUSG00000034620	Tmem5	transmembrane protein 5 [Source:MGI Symbol;Acc	0.49259
ENSMUSG00000086189	Gm15462	predicted gene 15462 [Source:MGI Symbol;Acc:MG	0.49266
ENSMUSG00000019916	P4ha1	procollagen-proline, 2-oxoglutarate 4-dioxygenase	0.49284

ENSMUSG00000026387	Sctr	secretin receptor [Source:MGI Symbol;Acc:MGI:244	0.49289
ENSMUSG00000033498	Strc	stereocilin [Source:MGI Symbol;Acc:MGI:2153816]	0.4929
ENSMUSG00000020898	Ctc1	CTS telomere maintenance complex component 1 [	0.49292
ENSMUSG00000045019	Acer1	alkaline ceramidase 1 [Source:MGI Symbol;Acc:MG	0.49293
ENSMUSG00000045667	Smtnl2	smoothelin-like 2 [Source:MGI Symbol;Acc:MGI:244	0.493
ENSMUSG00000086964	Gm15948	predicted gene 15948 [Source:MGI Symbol;Acc:MG	0.49303
ENSMUSG00000093157	Mir3098	microRNA 3098 [Source:MGI Symbol;Acc:MGI:4834	0.49304
ENSMUSG00000080791	Gm11442	predicted gene 11442 [Source:MGI Symbol;Acc:MG	0.49307
ENSMUSG00000033420	Antxr1	anthrax toxin receptor 1 [Source:MGI Symbol;Acc:M	0.4931
ENSMUSG00000021090	Lrrc9	leucine rich repeat containing 9 [Source:MGI Symb	0.49311
ENSMUSG00000086796	4932702P03Rik	RIKEN cDNA 4932702P03 gene [Source:MGI Symbo	0.4932
ENSMUSG00000098012	Gm8907	predicted gene 8907 [Source:MGI Symbol;Acc:MGI:	0.4932
ENSMUSG00000020922	Lsm12	LSM12 homolog (S. cerevisiae) [Source:MGI Symbo	0.49322
ENSMUSG00000086266	Igf2os	insulin-like growth factor 2, opposite strand [Sourc	0.49327
ENSMUSG00000074650	Gm10735	predicted gene 10735 [Source:MGI Symbol;Acc:MG	0.49344
ENSMUSG00000031845	Bcmo1	beta-carotene 15,15'-monooxygenase [Source:MGI	0.49367
ENSMUSG00000032489	Kif9	kinesin family member 9 [Source:MGI Symbol;Acc:M	0.49369
ENSMUSG00000051359	Ncald	neurocalcin delta [Source:MGI Symbol;Acc:MGI:119	0.49371
ENSMUSG00000032346	Ooep	oocyte expressed protein [Source:MGI Symbol;Acc:	0.49373
ENSMUSG00000020289	Nprl3	nitrogen permease regulator-like 3 [Source:MGI Sy	0.49378
ENSMUSG00000062432	Cyp26c1	cytochrome P450, family 26, subfamily c, polypepti	0.49386
ENSMUSG00000047867	Gimap6	GTPase, IMAP family member 6 [Source:MGI Symb	0.49395
ENSMUSG00000022828	Gtf2e1	general transcription factor II E, polypeptide 1 (alph	0.49407
ENSMUSG00000048351	Selrc1	Sel1 repeat containing 1 [Source:MGI Symbol;Acc:M	0.49414
ENSMUSG00000021867	Tmem254b	transmembrane protein 254b [Source:MGI Symbol;	0.49416
ENSMUSG00000060377	Rpl36a-ps1	ribosomal protein L36A, pseudogene 1 [Source:MG	0.49417
ENSMUSG00000024621	Csf1r	colony stimulating factor 1 receptor [Source:MGI S	0.49426
ENSMUSG00000023861	Mpc1	mitochondrial pyruvate carrier 1 [Source:MGI Symb	0.49448
ENSMUSG00000038797	Zscan2	zinc finger and SCAN domain containing 2 [Source:M	0.49451
ENSMUSG00000089746	Gm3513	predicted gene 3513 [Source:MGI Symbol;Acc:MGI:	0.49459
ENSMUSG00000003581	Rnf215	ring finger protein 215 [Source:MGI Symbol;Acc:MG	0.49465
ENSMUSG00000053004	Hrh1	histamine receptor H1 [Source:MGI Symbol;Acc:MG	0.49471
ENSMUSG00000029027	Dffb	DNA fragmentation factor, beta subunit [Source:MG	0.49481
ENSMUSG00000079711	Smok4a	sperm motility kinase 4A [Source:MGI Symbol;Acc:M	0.49484
ENSMUSG00000031434	Morc4	microrchidia 4 [Source:MGI Symbol;Acc:MGI:19229	0.49485
ENSMUSG00000034683	Ppp1r1c	protein phosphatase 1, regulatory (inhibitor) subun	0.49497
ENSMUSG00000059422	Gm8116	predicted gene 8116 [Source:MGI Symbol;Acc:MGI:	0.49516
ENSMUSG00000001569	Nom1	nucleolar protein with MIF4G domain 1 [Source:MG	0.49528
ENSMUSG00000032350	Gclc	glutamate-cysteine ligase, catalytic subunit [Source	0.49529
ENSMUSG00000049950	Rpp38	ribonuclease P/MRP 38 subunit [Source:MGI Symb	0.49535
ENSMUSG00000036752	Tubb4b	tubulin, beta 4B class IVB [Source:MGI Symbol;Acc:	0.49537
ENSMUSG00000038495	Otud7b	OTU domain containing 7B [Source:MGI Symbol;Ac	0.4954
ENSMUSG00000017767	Spata25	spermatogenesis associated 25 [Source:MGI Symb	0.49545
ENSMUSG00000023912	Slc25a27	solute carrier family 25, member 27 [Source:MGI S	0.49546
ENSMUSG00000020173	Cobl	cordon-bleu WH2 repeat [Source:MGI Symbol;Acc:	0.49551
ENSMUSG00000097334	2700012I20Rik	RIKEN cDNA 2700012I20 gene [Source:MGI Symbol	0.49553
ENSMUSG00000090799	Klhl33	kelch-like 33 [Source:MGI Symbol;Acc:MGI:364459	0.49554

ENSMUSG00000042138	Msantd2	Myb/SANT-like DNA-binding domain containing 2 [S	0.49568
ENSMUSG00000027669	Gnb4	guanine nucleotide binding protein (G protein), bet	0.49576
ENSMUSG00000039545	4930524L23Rik	RIKEN cDNA 4930524L23 gene [Source:MGI Symbo	0.4958
ENSMUSG00000022665	Ccdc80	coiled-coil domain containing 80 [Source:MGI Symb	0.49582
ENSMUSG00000005251	Ripk4	receptor-interacting serine-threonine kinase 4 [Sou	0.49585
ENSMUSG00000013698	Pea15a	phosphoprotein enriched in astrocytes 15A [Source	0.49624
ENSMUSG00000097261	4930532G15Rik	RIKEN cDNA 4930532G15 gene [Source:MGI Symbo	0.4963
ENSMUSG00000022347	A1bg	alpha-1-B glycoprotein [Source:MGI Symbol;Acc:M	0.49635
ENSMUSG00000035951	Ascl3	achaete-scute complex homolog 3 (Drosophila) [So	0.49645
ENSMUSG00000097559	D430018E03Rik	RIKEN cDNA D430018E03 gene [Source:MGI Symbo	0.49645
ENSMUSG00000053094	Tmem248	transmembrane protein 248 [Source:MGI Symbol;A	0.49647
ENSMUSG00000094650	Gm25361	predicted gene, 25361 [Source:MGI Symbol;Acc:M	0.49658
ENSMUSG00000024926	Kat5	K(lysine) acetyltransferase 5 [Source:MGI Symbol;A	0.4967
ENSMUSG00000035601	Trmt10b	tRNA methyltransferase 10B [Source:MGI Symbol;A	0.4967
ENSMUSG00000093525	Gm8974	predicted gene 8974 [Source:MGI Symbol;Acc:MGI	0.49673
ENSMUSG00000035270	Impg2	interphotoreceptor matrix proteoglycan 2 [Source:	0.49676
ENSMUSG00000071064	Zfp827	zinc finger protein 827 [Source:MGI Symbol;Acc:M	0.4968
ENSMUSG00000048827	Pkd1l3	polycystic kidney disease 1 like 3 [Source:MGI Symb	0.49691
ENSMUSG00000054252	Fgfr3	fibroblast growth factor receptor 3 [Source:MGI Sy	0.49692
ENSMUSG00000019866	Aim1	absent in melanoma 1 [Source:MGI Symbol;Acc:M	0.49698
ENSMUSG00000016327	Atp1b4	ATPase, (Na+)/K+ transporting, beta 4 polypeptide	0.49711
ENSMUSG00000032174	Icam5	intercellular adhesion molecule 5, telencephalin [S	0.49714
ENSMUSG00000079575	Rbpsuh-rs3	recombining binding protein suppressor of hairless	0.49716
ENSMUSG00000047959	Kcna3	potassium voltage-gated channel, shaker-related su	0.49717
ENSMUSG00000034126	Pomt2	protein-O-mannosyltransferase 2 [Source:MGI Sym	0.49721
ENSMUSG00000040048	Ndufb10	NADH dehydrogenase (ubiquinone) 1 beta subcom	0.49738
ENSMUSG00000056492	Gpr116	G protein-coupled receptor 116 [Source:MGI Symb	0.49742
ENSMUSG00000048400	Prss54	protease, serine, 54 [Source:MGI Symbol;Acc:MGI:	0.49755
ENSMUSG00000073532	Gm7276	predicted gene 7276 [Source:MGI Symbol;Acc:MGI	0.49762
ENSMUSG00000034575	Papd7	PAP associated domain containing 7 [Source:MGI S	0.49764
ENSMUSG00000051054	1700014D04Rik	RIKEN cDNA 1700014D04 gene [Source:MGI Symbo	0.49764
ENSMUSG00000062906	Hdac10	histone deacetylase 10 [Source:MGI Symbol;Acc:M	0.49766
ENSMUSG00000044276	4933427E11Rik	RIKEN cDNA 4933427E11 gene [Source:MGI Symbo	0.49768
ENSMUSG00000028639	Ybx1	Y box protein 1 [Source:MGI Symbol;Acc:MGI:9914	0.49789
ENSMUSG00000085336	Gm11732	predicted gene 11732 [Source:MGI Symbol;Acc:MG	0.49796
ENSMUSG00000072591	5930412G12Rik	RIKEN cDNA 5930412G12 gene [Source:MGI Symbo	0.49797
ENSMUSG00000022996	Wnt10b	wingless related MMTV integration site 10b [Source	0.49798
ENSMUSG00000028086	Fbxw7	F-box and WD-40 domain protein 7 [Source:MGI Sy	0.49802
ENSMUSG00000057137	Tmem140	transmembrane protein 140 [Source:MGI Symbol;A	0.49805
ENSMUSG00000005871	Apc	adenomatosis polyposis coli [Source:MGI Symbol;A	0.49812
ENSMUSG00000019782	Rwdd1	RWD domain containing 1 [Source:MGI Symbol;Acc	0.4982
ENSMUSG00000011267	Zfp296	zinc finger protein 296 [Source:MGI Symbol;Acc:M	0.49829
ENSMUSG00000022559	Fbxl6	F-box and leucine-rich repeat protein 6 [Source:MG	0.49833
ENSMUSG00000087617	Gm14268	predicted gene 14268 [Source:MGI Symbol;Acc:MG	0.49841
ENSMUSG00000040594	Ranbp17	RAN binding protein 17 [Source:MGI Symbol;Acc:M	0.49846
ENSMUSG00000015001	Oc90	otoconin 90 [Source:MGI Symbol;Acc:MGI:1313269	0.49852
ENSMUSG00000022359	Wdyhv1	WDYHV motif containing 1 [Source:MGI Symbol;Ac	0.49853

ENSMUSG00000045518	Onecut3	one cut domain, family member 3 [Source:MGI Sym	0.49855
ENSMUSG00000049796	Crh	corticotropin releasing hormone [Source:MGI Symb	0.49864
ENSMUSG00000079657	Rab26	RAB26, member RAS oncogene family [Source:MGI	0.49864
ENSMUSG00000093452	Gm20687	predicted gene 20687 [Source:MGI Symbol;Acc:MG	0.49865
ENSMUSG00000034105	Tldc1	TBC/LysM associated domain containing 1 [Source:	0.49879
ENSMUSG00000086870	Gm15288	predicted gene 15288 [Source:MGI Symbol;Acc:MG	0.49885
ENSMUSG00000087327	Gm15884	predicted gene 15884 [Source:MGI Symbol;Acc:MG	0.49885
ENSMUSG00000008734	Gprc5b	G protein-coupled receptor, family C, group 5, mem	0.49901
ENSMUSG00000009614	Sardh	sarcosine dehydrogenase [Source:MGI Symbol;Acc:	0.49909
ENSMUSG00000035505	Cox18	cytochrome c oxidase assembly protein 18 [Source:	0.49909
ENSMUSG00000040859	Bsdc1	BSD domain containing 1 [Source:MGI Symbol;Acc:	0.49918
ENSMUSG00000005057	Sh2b2	SH2B adaptor protein 2 [Source:MGI Symbol;Acc:M	0.49923
ENSMUSG00000020315	Sptbn1	spectrin beta, non-erythrocytic 1 [Source:MGI Syml	0.4994
ENSMUSG00000032177	Pde4a	phosphodiesterase 4A, cAMP specific [Source:MGI	0.4994
ENSMUSG00000043895	S1pr2	sphingosine-1-phosphate receptor 2 [Source:MGI S	0.4994
ENSMUSG00000045312	Lhfpl2	lipoma HMGIC fusion partner-like 2 [Source:MGI Sy	0.49942
ENSMUSG00000002103	Acp2	acid phosphatase 2, lysosomal [Source:MGI Symbol	0.49984
ENSMUSG00000026659	Dusp12	dual specificity phosphatase 12 [Source:MGI Symbo	0.4999
ENSMUSG00000031210	Gpr165	G protein-coupled receptor 165 [Source:MGI Symb	0.49993
ENSMUSG00000024571	Gm16286	predicted gene 16286 [Source:MGI Symbol;Acc:MG	0.49998
ENSMUSG00000031554	Adam5	a disintegrin and metallopeptidase domain 5 [Sourc	0.5
ENSMUSG00000045538	Ddx28	DEAD (Asp-Glu-Ala-Asp) box polypeptide 28 [Sourc	0.50003
ENSMUSG00000022098	Bmp1	bone morphogenetic protein 1 [Source:MGI Symbo	0.50005
ENSMUSG00000026473	Glul	glutamate-ammonia ligase (glutamine synthetase)	0.50009
ENSMUSG00000038446	Cdc40	cell division cycle 40 [Source:MGI Symbol;Acc:MGI:	0.50023
ENSMUSG00000028551	Cdkn2c	cyclin-dependent kinase inhibitor 2C (p18, inhibits	0.50029
ENSMUSG00000086449	9030204H09Rik	RIKEN cDNA 9030204H09 gene [Source:MGI Symbo	0.50039
ENSMUSG00000097595	2510016G02Rik	RIKEN cDNA 2510016G02 gene [Source:MGI Symbo	0.50045
ENSMUSG00000085779	2310050B05Rik	RIKEN cDNA 2310050B05 gene [Source:MGI Symbo	0.50047
ENSMUSG00000061666	Gdpd1	glycerophosphodiester phosphodiesterase domain	0.5007
ENSMUSG00000069272	Hist1h2ae	histone cluster 1, H2ae [Source:MGI Symbol;Acc:M	0.50077
ENSMUSG00000054072	Iigp1	interferon inducible GTPase 1 [Source:MGI Symbol;	0.50094
ENSMUSG00000020783	1200014J11Rik	RIKEN cDNA 1200014J11 gene [Source:MGI Symbol	0.50103
ENSMUSG00000035585	Tsen34	tRNA splicing endonuclease 34 homolog (S. cerevisi	0.50106
ENSMUSG00000032537	Ephb1	Eph receptor B1 [Source:MGI Symbol;Acc:MGI:109	0.50138
ENSMUSG00000020593	Lpin1	lipin 1 [Source:MGI Symbol;Acc:MGI:1891340]	0.50162
ENSMUSG00000086794	Gm11642	predicted gene 11642 [Source:MGI Symbol;Acc:MG	0.50164
ENSMUSG00000055932	Fto	fat mass and obesity associated [Source:MGI Symb	0.50169
ENSMUSG00000007415	Gatad1	GATA zinc finger domain containing 1 [Source:MGI	0.50177
ENSMUSG00000092368	A930015D03Rik	RIKEN cDNA A930015D03 gene [Source:MGI Symbo	0.50181
ENSMUSG00000019804	Snx3	sorting nexin 3 [Source:MGI Symbol;Acc:MGI:1860	0.50185
ENSMUSG00000038550	Gm129	predicted gene 129 [Source:MGI Symbol;Acc:MGI:2	0.50187
ENSMUSG00000036698	Ago2	argonaute RISC catalytic subunit 2 [Source:MGI Syn	0.50189
ENSMUSG00000017817	Jph2	junctophilin 2 [Source:MGI Symbol;Acc:MGI:18914	0.50192
ENSMUSG00000097257	Gm26768	predicted gene, 26768 [Source:MGI Symbol;Acc:M	0.50201
ENSMUSG00000085837	4933400F03Rik	RIKEN cDNA 4933400F03 gene [Source:MGI Symbo	0.50206
ENSMUSG00000027286	Lrrc57	leucine rich repeat containing 57 [Source:MGI Syml	0.50218

ENSMUSG00000028525	Pde4b	phosphodiesterase 4B, cAMP specific [Source:MGI	0.50219
ENSMUSG00000049811	Fam161a	family with sequence similarity 161, member A [Sou	0.50222
ENSMUSG00000035172	Plekhh3	pleckstrin homology domain containing, family H (w	0.50225
ENSMUSG00000060780	Lrrtm1	leucine rich repeat transmembrane neuronal 1 [Sou	0.50227
ENSMUSG00000079435	Rpl36a	ribosomal protein L36A [Source:MGI Symbol;Acc:M	0.50227
ENSMUSG00000049946	BC030500	cDNA sequence BC030500 [Source:MGI Symbol;Acc	0.50231
ENSMUSG00000086742	Gm16201	predicted gene 16201 [Source:MGI Symbol;Acc:MG	0.50233
ENSMUSG00000097537	2610020C07Rik	RIKEN cDNA 2610020C07 gene [Source:MGI Symbo	0.50238
ENSMUSG00000031916	Cog8	component of oligomeric golgi complex 8 [Source:M	0.50241
ENSMUSG00000073985	Gm10602	predicted gene 10602 [Source:MGI Symbol;Acc:MG	0.50242
ENSMUSG00000023965	Fbxl17	F-box and leucine-rich repeat protein 17 [Source:M	0.50261
ENSMUSG00000007458	M6pr	mannose-6-phosphate receptor, cation dependent	0.50277
ENSMUSG00000039233	Tbce	tubulin-specific chaperone E [Source:MGI Symbol;A	0.50278
ENSMUSG00000038048	Cntnap5c	contactin associated protein-like 5C [Source:MGI Sy	0.50285
ENSMUSG00000004268	Emg1	EMG1 nucleolar protein homolog (S. cerevisiae) [So	0.50294
ENSMUSG00000022193	Psmb5	proteasome (prosome, macropain) subunit, beta ty	0.50301
ENSMUSG00000087155	Gm15008	predicted gene 15008 [Source:MGI Symbol;Acc:MG	0.50302
ENSMUSG00000097275	Gm26648	predicted gene, 26648 [Source:MGI Symbol;Acc:MG	0.50302
ENSMUSG00000001786	Fbxo7	F-box protein 7 [Source:MGI Symbol;Acc:MGI:1917	0.50304
ENSMUSG00000084306	Gm11743	predicted gene 11743 [Source:MGI Symbol;Acc:MG	0.50304
ENSMUSG00000035495	Tstd2	thiosulfate sulfurtransferase (rhodanese)-like doma	0.50308
ENSMUSG00000086784	Isoc2a	isochorismatase domain containing 2a [Source:MGI	0.50322
ENSMUSG00000033222	Ttf2	transcription termination factor, RNA polymerase II	0.50324
ENSMUSG00000037542	Aldh8a1	aldehyde dehydrogenase 8 family, member A1 [Sou	0.50329
ENSMUSG00000026798	Coq4	coenzyme Q4 homolog (yeast) [Source:MGI Symbo	0.50333
ENSMUSG00000023191	Leprel2	leprecan-like 2 [Source:MGI Symbol;Acc:MGI:13152	0.50335
ENSMUSG00000025083	Afap1l2	actin filament associated protein 1-like 2 [Source:M	0.50337
ENSMUSG00000085887	Gm11648	predicted gene 11648 [Source:MGI Symbol;Acc:MG	0.5034
ENSMUSG00000020372	Gnb2l1	guanine nucleotide binding protein (G protein), bet	0.50349
ENSMUSG00000031714	Gab1	growth factor receptor bound protein 2-associated	0.5035
ENSMUSG00000040813	Tex264	testis expressed gene 264 [Source:MGI Symbol;Acc	0.5035
ENSMUSG00000017861	Mybl2	myeloblastosis oncogene-like 2 [Source:MGI Symbo	0.50361
ENSMUSG00000072878	1700123L14Rik	RIKEN cDNA 1700123L14 gene [Source:MGI Symbo	0.50367
ENSMUSG00000077448	Gm23126	predicted gene, 23126 [Source:MGI Symbol;Acc:MG	0.50369
ENSMUSG00000070084	Mir486	microRNA 486 [Source:MGI Symbol;Acc:MGI:36194	0.50371
ENSMUSG00000098057	Gm6283	predicted gene 6283 [Source:MGI Symbol;Acc:MGI:	0.50376
ENSMUSG00000042581	Thsd7b	thrombospondin, type I, domain containing 7B [Sou	0.50387
ENSMUSG00000019178	Styxl1	serine/threonine/tyrosine interacting-like 1 [Source	0.50388
ENSMUSG00000026333	Gin1	gypsy retrotransposon integrase 1 [Source:MGI Sym	0.50412
ENSMUSG00000055188	2900002K06Rik	RIKEN cDNA 2900002K06 gene [Source:MGI Symbo	0.50416
ENSMUSG00000097074	4833428L15Rik	RIKEN cDNA 4833428L15 gene [Source:MGI Symbo	0.50417
ENSMUSG00000019979	Apaf1	apoptotic peptidase activating factor 1 [Source:MG	0.50427
ENSMUSG00000035772	Mrps2	mitochondrial ribosomal protein S2 [Source:MGI Sy	0.50433
ENSMUSG00000037708	BC061194	cDNA sequence BC061194 [Source:MGI Symbol;Acc	0.50436
ENSMUSG00000051977	Prdm9	PR domain containing 9 [Source:MGI Symbol;Acc:M	0.50436
ENSMUSG00000092931	Gm22904	predicted gene, 22904 [Source:MGI Symbol;Acc:MG	0.50438
ENSMUSG00000071856	Mcc	mutated in colorectal cancers [Source:MGI Symbol;	0.50446

ENSMUSG00000000948	Snrpn	small nuclear ribonucleoprotein N [Source:MGI Syn	0.50448
ENSMUSG00000025269	Apex2	apurinic/apyrimidinic endonuclease 2 [Source:MGI	0.50449
ENSMUSG00000058022	Adtrp	androgen dependent TFPI regulating protein [Sourc	0.50451
ENSMUSG00000034839	Larp6	La ribonucleoprotein domain family, member 6 [So	0.50462
ENSMUSG00000042719	Naa25	N(alpha)-acetyltransferase 25, NatB auxiliary subur	0.50462
ENSMUSG00000050777	Tmem37	transmembrane protein 37 [Source:MGI Symbol;Ac	0.50475
ENSMUSG00000082203	Gm15636	predicted gene 15636 [Source:MGI Symbol;Acc:MG	0.50505
ENSMUSG00000033416	Gucd1	guanylyl cyclase domain containing 1 [Source:MGI S	0.50528
ENSMUSG00000041420	Meis3	Meis homeobox 3 [Source:MGI Symbol;Acc:MGI:10	0.50528
ENSMUSG00000017466	Timp2	tissue inhibitor of metalloproteinase 2 [Source:MGI	0.50538
ENSMUSG00000030124	Lag3	lymphocyte-activation gene 3 [Source:MGI Symbol;	0.50559
ENSMUSG00000066191	Anks6	ankyrin repeat and sterile alpha motif domain cont	0.50563
ENSMUSG00000024206	Rfx2	regulatory factor X, 2 (influences HLA class II expres	0.50568
ENSMUSG00000002102	Psmc3	proteasome (prosome, macropain) 26S subunit, AT	0.50576
ENSMUSG00000070407	Hs3st3b1	heparan sulfate (glucosamine) 3-O-sulfotransferase	0.50581
ENSMUSG00000073775	Kti12	KTI12 homolog, chromatin associated (S. cerevisiae	0.506
ENSMUSG00000089776	Gm15684	predicted gene 15684 [Source:MGI Symbol;Acc:MG	0.506
ENSMUSG00000027318	Adam33	a disintegrin and metallopeptidase domain 33 [Sou	0.50601
ENSMUSG00000084085	Gm16140	predicted gene 16140 [Source:MGI Symbol;Acc:MG	0.50603
ENSMUSG00000014198	Zfp385c	zinc finger protein 385C [Source:MGI Symbol;Acc:M	0.50615
ENSMUSG00000038028	9630033F20Rik	RIKEN cDNA 9630033F20 gene [Source:MGI Symbo	0.50642
ENSMUSG00000085455	1810059H22Rik	RIKEN cDNA 1810059H22 gene [Source:MGI Symbo	0.50644
ENSMUSG00000015365	Mov10l1	Moloney leukemia virus 10-like 1 [Source:MGI Sym	0.50648
ENSMUSG00000097791	Gm17315	predicted gene, 17315 [Source:MGI Symbol;Acc:M(	0.50656
ENSMUSG00000038650	Rnh1	ribonuclease/angiogenin inhibitor 1 [Source:MGI Sy	0.50661
ENSMUSG00000040274	Cdk6	cyclin-dependent kinase 6 [Source:MGI Symbol;Acc	0.50663
ENSMUSG00000002279	Lmf1	lipase maturation factor 1 [Source:MGI Symbol;Acc	0.50667
ENSMUSG00000007670	Khsrp	KH-type splicing regulatory protein [Source:MGI Sy	0.50668
ENSMUSG00000084694	Gm24893	predicted gene, 24893 [Source:MGI Symbol;Acc:M(	0.5067
ENSMUSG00000090215	Trim34b	tripartite motif-containing 34B [Source:MGI Symbo	0.50673
ENSMUSG00000080877	Rpl22-ps1	ribosomal protein L22, pseudogene 1 [Source:MGI	0.50679
ENSMUSG00000074210	E130208F15Rik	RIKEN cDNA E130208F15 gene [Source:MGI Symbo	0.50682
ENSMUSG00000020598	Nrcam	neuron-glia-CAM-related cell adhesion molecule [S	0.50684
ENSMUSG00000020573	Pik3cg	phosphoinositide-3-kinase, catalytic, gamma polyp	0.50712
ENSMUSG00000074364	Ehd2	EH-domain containing 2 [Source:MGI Symbol;Acc:M	0.50712
ENSMUSG00000034573	Ptpn13	protein tyrosine phosphatase, non-receptor type 1	0.50713
ENSMUSG00000086214	Gm14062	predicted gene 14062 [Source:MGI Symbol;Acc:MG	0.50729
ENSMUSG00000037904	Ankrd9	ankyrin repeat domain 9 [Source:MGI Symbol;Acc:M	0.50731
ENSMUSG00000028668	Tceb3	transcription elongation factor B (SIII), polypeptide	0.50748
ENSMUSG00000026785	Pkn3	protein kinase N3 [Source:MGI Symbol;Acc:MGI:23	0.50764
ENSMUSG00000052180	Serpinb6c	serine (or cysteine) peptidase inhibitor, clade B, me	0.5077
ENSMUSG00000095315	Gm10130	predicted gene 10130 [Source:MGI Symbol;Acc:MG	0.5077
ENSMUSG00000042514	Klhl14	kelch-like 14 [Source:MGI Symbol;Acc:MGI:192124	0.50781
ENSMUSG00000040021	Lats1	large tumor suppressor [Source:MGI Symbol;Acc:M	0.50782
ENSMUSG00000018740	Slc25a35	solute carrier family 25, member 35 [Source:MGI Sy	0.50788
ENSMUSG00000003955	Fam162a	family with sequence similarity 162, member A [Sou	0.50796
ENSMUSG00000021400	Wrnip1	Werner helicase interacting protein 1 [Source:MGI	0.5081

ENSMUSG00000002870	Mcm2	minichromosome maintenance deficient 2 mitotin	0.50811
ENSMUSG00000027207	Galk2	galactokinase 2 [Source:MGI Symbol;Acc:MGI:1917	0.50817
ENSMUSG00000003546	Klc4	kinesin light chain 4 [Source:MGI Symbol;Acc:MGI:1	0.50819
ENSMUSG00000034278	Dnajc17	DnaJ (Hsp40) homolog, subfamily C, member 17 [So	0.5082
ENSMUSG00000039232	Stx11	syntaxin 11 [Source:MGI Symbol;Acc:MGI:1921982]	0.50843
ENSMUSG00000041323	Ak7	adenylate kinase 7 [Source:MGI Symbol;Acc:MGI:19	0.50846
ENSMUSG00000023000	Dhh	desert hedgehog [Source:MGI Symbol;Acc:MGI:948	0.50847
ENSMUSG00000037313	Tacc3	transforming, acidic coiled-coil containing protein 3	0.50851
ENSMUSG00000023021	Cers5	ceramide synthase 5 [Source:MGI Symbol;Acc:MGI:	0.50857
ENSMUSG00000069089	Cdk7	cyclin-dependent kinase 7 [Source:MGI Symbol;Acc	0.5087
ENSMUSG00000022223	Sdr39u1	short chain dehydrogenase/reductase family 39U, r	0.50873
ENSMUSG00000027613	Eif6	eukaryotic translation initiation factor 6 [Source:MG	0.50877
ENSMUSG00000026668	Ucma	upper zone of growth plate and cartilage matrix ass	0.50886
ENSMUSG00000039033	Tasp1	taspase, threonine aspartase 1 [Source:MGI Symbo	0.50886
ENSMUSG00000032033	Barx2	BarH-like homeobox 2 [Source:MGI Symbol;Acc:MG	0.50887
ENSMUSG00000078653	Cntd1	cyclin N-terminal domain containing 1 [Source:MGI	0.50889
ENSMUSG00000034663	Bmp2k	BMP2 inducible kinase [Source:MGI Symbol;Acc:MG	0.50894
ENSMUSG00000088106	Gm22378	predicted gene, 22378 [Source:MGI Symbol;Acc:MG	0.50902
ENSMUSG00000029022	Miip	migration and invasion inhibitory protein [Source:M	0.50913
ENSMUSG00000097591	A330032B11Rik	RIKEN cDNA A330032B11 gene [Source:MGI Symbo	0.50917
ENSMUSG00000078667	1700094D03Rik	RIKEN cDNA 1700094D03 gene [Source:MGI Symbo	0.50918
ENSMUSG00000063889	Crem	cAMP responsive element modulator [Source:MGI S	0.50934
ENSMUSG00000074529	C330013J21Rik	RIKEN cDNA C330013J21 gene [Source:MGI Symbol	0.50935
ENSMUSG00000081104	Gm15989	predicted gene 15989 [Source:MGI Symbol;Acc:MG	0.50938
ENSMUSG00000031253	Srpx2	sushi-repeat-containing protein, X-linked 2 [Source	0.50942
ENSMUSG00000034220	Gpc1	glypican 1 [Source:MGI Symbol;Acc:MGI:1194891]	0.50948
ENSMUSG00000033860	Fgg	fibrinogen gamma chain [Source:MGI Symbol;Acc:M	0.50952
ENSMUSG00000030399	Ckm	creatine kinase, muscle [Source:MGI Symbol;Acc:M	0.50953
ENSMUSG00000042607	Asb4	ankyrin repeat and SOCS box-containing 4 [Source:	0.50954
ENSMUSG00000042510	AA986860	expressed sequence AA986860 [Source:MGI Symbo	0.5096
ENSMUSG00000027684	Mecom	MDS1 and EVI1 complex locus [Source:MGI Symbol	0.50965
ENSMUSG00000027300	Ubox5	U box domain containing 5 [Source:MGI Symbol;Ac	0.50975
ENSMUSG00000055980	Irs1	insulin receptor substrate 1 [Source:MGI Symbol;Ac	0.50975
ENSMUSG00000055396	D830050J10Rik	RIKEN cDNA D830050J10 gene [Source:MGI Symbo	0.50977
ENSMUSG00000083555	Gm12035	predicted gene 12035 [Source:MGI Symbol;Acc:MG	0.50985
ENSMUSG00000020413	Hus1	Hus1 homolog (S. pombe) [Source:MGI Symbol;Acc	0.50986
ENSMUSG00000002733	Plekha3	pleckstrin homology domain-containing, family A (p	0.50993
ENSMUSG00000084033	Gm7645	predicted gene 7645 [Source:MGI Symbol;Acc:MGI:	0.50996
ENSMUSG00000024517	Grp	gastrin releasing peptide [Source:MGI Symbol;Acc:M	0.51
ENSMUSG00000018381	Abi3	ABI gene family, member 3 [Source:MGI Symbol;Ac	0.51006
ENSMUSG00000026765	Lypd6b	LY6/PLAUR domain containing 6B [Source:MGI Sym	0.5101
ENSMUSG00000066861	Oas1g	2'-5' oligoadenylate synthetase 1G [Source:MGI Sym	0.51015
ENSMUSG00000061992	Gm4953	predicted pseudogene 4953 [Source:MGI Symbol;A	0.51019
ENSMUSG00000022783	Spag6	sperm associated antigen 6 [Source:MGI Symbol;Ac	0.5102
ENSMUSG00000041309	Nkx6-2	NK6 homeobox 2 [Source:MGI Symbol;Acc:MGI:135	0.51022
ENSMUSG00000001674	Ddx18	DEAD (Asp-Glu-Ala-Asp) box polypeptide 18 [Source	0.51024
ENSMUSG00000015659	Serac1	serine active site containing 1 [Source:MGI Symbol;	0.51027

ENSMUSG00000095440	Fignl2	fidgetin-like 2 [Source:MGI Symbol;Acc:MGI:36469	0.51041
ENSMUSG00000020143	Dock2	dedicator of cyto-kinesis 2 [Source:MGI Symbol;Acc	0.51044
ENSMUSG00000027168	Pax6	paired box gene 6 [Source:MGI Symbol;Acc:MGI:97	0.51046
ENSMUSG00000040705	A930016O22Rik	RIKEN cDNA A930016O22 gene [Source:MGI Symbo	0.51046
ENSMUSG00000048534	Amica1	adhesion molecule, interacts with CXADR antigen 1	0.5105
ENSMUSG00000039599	Fam149b	family with sequence similarity 149, member B [Sou	0.51058
ENSMUSG00000048200	Efcab4a	EF-hand calcium binding domain 4A [Source:MGI Sy	0.51066
ENSMUSG00000020635	Fkbp1b	FK506 binding protein 1b [Source:MGI Symbol;Acc:	0.51067
ENSMUSG00000044148	1810030O07Rik	RIKEN cDNA 1810030O07 gene [Source:MGI Symbo	0.51068
ENSMUSG00000028269	Gbp2b	guanylate binding protein 2b [Source:MGI Symbol;A	0.5107
ENSMUSG00000002015	Bcap31	B cell receptor associated protein 31 [Source:MGI S	0.51077
ENSMUSG00000049686	Orai1	ORAI calcium release-activated calcium modulator	0.5108
ENSMUSG00000070527	Mkrn3	makorin, ring finger protein, 3 [Source:MGI Symbol	0.51081
ENSMUSG00000047953	Gp5	glycoprotein 5 (platelet) [Source:MGI Symbol;Acc:M	0.51087
ENSMUSG00000070425	Trpc2	transient receptor potential cation channel, subfam	0.51087
ENSMUSG00000032739	Pram1	PML-RAR alpha-regulated adaptor molecule 1 [Sou	0.51104
ENSMUSG00000059182	Skap2	src family associated phosphoprotein 2 [Source:MG	0.51104
ENSMUSG00000055323	Gm9967	predicted gene 9967 [Source:MGI Symbol;Acc:MGI:	0.51121
ENSMUSG00000068686	Cd59b	CD59b antigen [Source:MGI Symbol;Acc:MGI:1888	0.51127
ENSMUSG00000017309	Cd300lg	CD300 antigen like family member G [Source:MGI S	0.51137
ENSMUSG00000030189	Ybx3	Y box protein 3 [Source:MGI Symbol;Acc:MGI:2137	0.51137
ENSMUSG00000021701	Plk2	polo-like kinase 2 [Source:MGI Symbol;Acc:MGI:10	0.51149
ENSMUSG00000040061	Plcb2	phospholipase C, beta 2 [Source:MGI Symbol;Acc:M	0.51162
ENSMUSG00000050343	Olfr1378	olfactory receptor 1378 [Source:MGI Symbol;Acc:M	0.51164
ENSMUSG00000036186	Fam69b	family with sequence similarity 69, member B [Sou	0.51165
ENSMUSG00000036315	Znrd1	zinc ribbon domain containing, 1 [Source:MGI Symb	0.51186
ENSMUSG00000065591	Gm22753	predicted gene, 22753 [Source:MGI Symbol;Acc:MG	0.51191
ENSMUSG00000048445	Ccdc57	coiled-coil domain containing 57 [Source:MGI Symb	0.51194
ENSMUSG00000055148	Klf2	Kruppel-like factor 2 (lung) [Source:MGI Symbol;Ac	0.51199
ENSMUSG00000030659	Nucb2	nucleobindin 2 [Source:MGI Symbol;Acc:MGI:1858	0.51205
ENSMUSG00000086349	Gm13497	predicted gene 13497 [Source:MGI Symbol;Acc:MG	0.51215
ENSMUSG00000026317	Cln8	ceroid-lipofuscinosis, neuronal 8 [Source:MGI Symb	0.51219
ENSMUSG00000080796	Gm9159	predicted gene 9159 [Source:MGI Symbol;Acc:MGI:	0.51219
ENSMUSG00000009585	Apobec3	apolipoprotein B mRNA editing enzyme, catalytic p	0.51222
ENSMUSG00000085459	Gm12272	predicted gene 12272 [Source:MGI Symbol;Acc:MG	0.51229
ENSMUSG00000083840	Gm15459	predicted gene 15459 [Source:MGI Symbol;Acc:MG	0.51232
ENSMUSG00000090881	Gm6904	predicted gene 6904 [Source:MGI Symbol;Acc:MGI:	0.51234
ENSMUSG00000087165	2010001A14Rik	RIKEN cDNA 2010001A14 gene [Source:MGI Symbo	0.51238
ENSMUSG00000043282	Gm5531	predicted gene 5531 [Source:MGI Symbol;Acc:MGI:	0.51241
ENSMUSG00000064043	Trerf1	transcriptional regulating factor 1 [Source:MGI Sym	0.51244
ENSMUSG00000027080	Med19	mediator of RNA polymerase II transcription, subur	0.51251
ENSMUSG00000020647	Ncoa1	nuclear receptor coactivator 1 [Source:MGI Symbol	0.51256
ENSMUSG00000031860	Pbx4	pre B cell leukemia homeobox 4 [Source:MGI Symb	0.51273
ENSMUSG00000050296	Abca12	ATP-binding cassette, sub-family A (ABC1), membe	0.51274
ENSMUSG00000038255	Neurod2	neurogenic differentiation 2 [Source:MGI Symbol;A	0.51282
ENSMUSG00000035357	Pdzrn3	PDZ domain containing RING finger 3 [Source:MGI S	0.51283
ENSMUSG00000098108	Gm27008	predicted gene, 27008 [Source:MGI Symbol;Acc:MG	0.51287

ENSMUSG00000073647	Gm10557	predicted gene 10557 [Source:MGI Symbol;Acc:MG	0.51296
ENSMUSG00000078787	Cyp2t4	cytochrome P450, family 2, subfamily t, polypeptid	0.51299
ENSMUSG00000098093	Gm8707	predicted gene 8707 [Source:MGI Symbol;Acc:MGI:	0.51333
ENSMUSG00000032905	Atg12	autophagy related 12 [Source:MGI Symbol;Acc:MG	0.51342
ENSMUSG00000072115	Ang	angiogenin, ribonuclease, RNase A family, 5 [Source	0.51343
ENSMUSG00000025318	Jph3	junctophilin 3 [Source:MGI Symbol;Acc:MGI:189149	0.51346
ENSMUSG00000062619	2310039H08Rik	RIKEN cDNA 2310039H08 gene [Source:MGI Symbo	0.51367
ENSMUSG00000014329	Bicc1	bicaudal C homolog 1 (Drosophila) [Source:MGI Syn	0.51391
ENSMUSG00000022300	Dcaf13	DDB1 and CUL4 associated factor 13 [Source:MGI S	0.51396
ENSMUSG00000024600	Slc27a6	solute carrier family 27 (fatty acid transporter), me	0.51403
ENSMUSG00000032952	Ap4b1	adaptor-related protein complex AP-4, beta 1 [Sour	0.51403
ENSMUSG00000036155	Mgat5	mannoside acetylglucosaminyltransferase 5 [Source	0.51406
ENSMUSG00000070330	Tmem235	transmembrane protein 235 [Source:MGI Symbol;A	0.51416
ENSMUSG00000049728	Zfp668	zinc finger protein 668 [Source:MGI Symbol;Acc:MG	0.51417
ENSMUSG00000035285	Nat14	N-acetyltransferase 14 [Source:MGI Symbol;Acc:MG	0.51438
ENSMUSG00000086232	Gm7546	predicted gene 7546 [Source:MGI Symbol;Acc:MGI:	0.5144
ENSMUSG00000030058	Copg1	coatomer protein complex, subunit gamma 1 [Sour	0.5145
ENSMUSG00000079311	Gm3222	predicted pseudogene 3222 [Source:MGI Symbol;A	0.51457
ENSMUSG00000003436	Dll3	delta-like 3 (Drosophila) [Source:MGI Symbol;Acc:M	0.51462
ENSMUSG00000097572	Gm26797	predicted gene, 26797 [Source:MGI Symbol;Acc:MG	0.51462
ENSMUSG00000028651	Ppie	peptidylprolyl isomerase E (cyclophilin E) [Source:M	0.51463
ENSMUSG00000097434	Gm16630	predicted gene, 16630 [Source:MGI Symbol;Acc:MG	0.51464
ENSMUSG00000025762	Larp1b	La ribonucleoprotein domain family, member 1B [S	0.51465
ENSMUSG00000097699	5430400D12Rik	RIKEN cDNA 5430400D12 gene [Source:MGI Symbo	0.51474
ENSMUSG00000002147	Stat6	signal transducer and activator of transcription 6 [S	0.51483
ENSMUSG00000080727	C920021L13Rik	RIKEN cDNA C920021L13 gene [Source:MGI Symbo	0.51483
ENSMUSG00000040528	Milr1	mast cell immunoglobulin like receptor 1 [Source:M	0.51492
ENSMUSG00000073423	Zfp414	zinc finger protein 414 [Source:MGI Symbol;Acc:MG	0.51496
ENSMUSG00000060735	Rxfp3	relaxin family peptide receptor 3 [Source:MGI Symb	0.5152
ENSMUSG00000061898	Rbak	RB-associated KRAB repressor [Source:MGI Symbol	0.51531
ENSMUSG00000074783	AU019990	expressed sequence AU019990 [Source:MGI Symbo	0.5156
ENSMUSG00000044231	Nhlrc1	NHL repeat containing 1 [Source:MGI Symbol;Acc:N	0.51561
ENSMUSG00000000958	Slc7a7	solute carrier family 7 (cationic amino acid transpo	0.51564
ENSMUSG00000053617	Sh3pxd2a	SH3 and PX domains 2A [Source:MGI Symbol;Acc:M	0.5157
ENSMUSG00000025854	Fam20c	family with sequence similarity 20, member C [Sour	0.51575
ENSMUSG00000032076	Cadm1	cell adhesion molecule 1 [Source:MGI Symbol;Acc:M	0.51578
ENSMUSG00000032988	Slc16a8	solute carrier family 16 (monocarboxylic acid transp	0.51579
ENSMUSG00000046567	4930430F08Rik	RIKEN cDNA 4930430F08 gene [Source:MGI Symbo	0.51598
ENSMUSG00000056050	Mia3	melanoma inhibitory activity 3 [Source:MGI Symbo	0.51601
ENSMUSG00000025823	Pdia4	protein disulfide isomerase associated 4 [Source:M	0.51606
ENSMUSG00000088008	Gm25492	predicted gene, 25492 [Source:MGI Symbol;Acc:MG	0.51606
ENSMUSG00000039725	2810408M09Rik	RIKEN cDNA 2810408M09 gene [Source:MGI Symbo	0.51611
ENSMUSG00000079671	2610203C22Rik	RIKEN cDNA 2610203C22 gene [Source:MGI Symbo	0.51612
ENSMUSG00000034321	Exosc1	exosome component 1 [Source:MGI Symbol;Acc:M	0.51614
ENSMUSG00000017737	Mmp9	matrix metallopeptidase 9 [Source:MGI Symbol;Acc	0.51623
ENSMUSG00000031823	Zdhhc7	zinc finger, DHHC domain containing 7 [Source:MG	0.51623
ENSMUSG00000038677	Scube3	signal peptide, CUB domain, EGF-like 3 [Source:MG	0.51642

ENSMUSG00000039801	2410089E03Rik	RIKEN cDNA 2410089E03 gene [Source:MGI Symbo	0.51644
ENSMUSG00000020185	E2f7	E2F transcription factor 7 [Source:MGI Symbol;Acc:	0.51651
ENSMUSG00000045975	C2cd2	C2 calcium-dependent domain containing 2 [Source	0.51655
ENSMUSG00000065952	C330021F23Rik	RIKEN cDNA C330021F23 gene [Source:MGI Symbo	0.51659
ENSMUSG00000048490	Nrip1	nuclear receptor interacting protein 1 [Source:MGI	0.51665
ENSMUSG00000029433	Diablo	diablo homolog (Drosophila) [Source:MGI Symbol;A	0.5167
ENSMUSG00000055045	Gm11190	predicted gene 11190 [Source:MGI Symbol;Acc:MG	0.51673
ENSMUSG00000018999	Slc35b4	solute carrier family 35, member B4 [Source:MGI Sy	0.51677
ENSMUSG00000036863	Syde2	synapse defective 1, Rho GTPase, homolog 2 (C. ele	0.51692
ENSMUSG00000034285	Nipsnap1	4-nitrophenylphosphatase domain and non-neuron	0.51693
ENSMUSG00000054598	9130230L23Rik	RIKEN cDNA 9130230L23 gene [Source:MGI Symbo	0.51698
ENSMUSG00000077250	Gm26075	predicted gene, 26075 [Source:MGI Symbol;Acc:M	0.51699
ENSMUSG00000071636	Rimbp3	RIMS binding protein 3 [Source:MGI Symbol;Acc:M	0.51701
ENSMUSG00000054309	Cpsf3	cleavage and polyadenylation specificity factor 3 [S	0.51702
ENSMUSG00000071415	Rpl23	ribosomal protein L23 [Source:MGI Symbol;Acc:MG	0.51711
ENSMUSG00000043398	Gpr135	G protein-coupled receptor 135 [Source:MGI Symb	0.51741
ENSMUSG00000045709	1700023L04Rik	RIKEN cDNA 1700023L04 gene [Source:MGI Symbo	0.51751
ENSMUSG00000020963	Tshr	thyroid stimulating hormone receptor [Source:MGI	0.5176
ENSMUSG00000030688	Stard10	START domain containing 10 [Source:MGI Symbol;A	0.51779
ENSMUSG00000097079	Gm26604	predicted gene, 26604 [Source:MGI Symbol;Acc:M	0.51785
ENSMUSG00000033737	Fndc3c1	fibronectin type III domain containing 3C1 [Source:	0.51802
ENSMUSG00000039013	Siglec5	sialic acid binding Ig-like lectin 5 [Source:MGI Symb	0.51812
ENSMUSG00000019795	Pcmt1	protein-L-isoaspartate (D-aspartate) O-methyltrans	0.51826
ENSMUSG00000020718	Polg2	polymerase (DNA directed), gamma 2, accessory su	0.51837
ENSMUSG00000027695	Pld1	phospholipase D1 [Source:MGI Symbol;Acc:MGI:10	0.5184
ENSMUSG00000032369	Plscr1	phospholipid scramblase 1 [Source:MGI Symbol;Acc	0.51847
ENSMUSG00000022033	Pbk	PDZ binding kinase [Source:MGI Symbol;Acc:MGI:1	0.51854
ENSMUSG00000029477	Morn3	MORN repeat containing 3 [Source:MGI Symbol;Acc	0.51861
ENSMUSG00000038240	Pdss2	prenyl (solanesyl) diphosphate synthase, subunit 2	0.51861
ENSMUSG00000021357	Exoc2	exocyst complex component 2 [Source:MGI Symbo	0.51881
ENSMUSG00000056666	Retsat	retinol saturase (all trans retinol 13,14 reductase) [	0.51881
ENSMUSG00000086706	Gm15848	predicted gene 15848 [Source:MGI Symbol;Acc:MG	0.51883
ENSMUSG00000002395	Use1	unconventional SNARE in the ER 1 homolog (S. cere	0.51887
ENSMUSG00000071531	Gprin2	G protein regulated inducer of neurite outgrowth 2	0.51888
ENSMUSG00000022376	Adcy8	adenylate cyclase 8 [Source:MGI Symbol;Acc:MGI:1	0.51898
ENSMUSG00000002280	Narfl	nuclear prelamin A recognition factor-like [Source:I	0.51901
ENSMUSG00000081208	Gm11400	predicted gene 11400 [Source:MGI Symbol;Acc:MG	0.51901
ENSMUSG00000053754	Chd8	chromodomain helicase DNA binding protein 8 [Sou	0.51903
ENSMUSG00000074604	Mgst2	microsomal glutathione S-transferase 2 [Source:M	0.51905
ENSMUSG00000039682	Lap3	leucine aminopeptidase 3 [Source:MGI Symbol;Acc	0.51909
ENSMUSG00000028133	Rwdd3	RWD domain containing 3 [Source:MGI Symbol;Acc	0.51931
ENSMUSG00000055305	Zfp93	zinc finger protein 93 [Source:MGI Symbol;Acc:MG	0.51934
ENSMUSG00000033478	Fam160b1	family with sequence similarity 160, member B1 [So	0.51939
ENSMUSG00000044345	Marveld1	MARVEL (membrane-associating) domain containin	0.51941
ENSMUSG00000085719	4933424L21Rik	RIKEN cDNA 4933424L21 gene [Source:MGI Symbo	0.51942
ENSMUSG00000074141	Il4i1	interleukin 4 induced 1 [Source:MGI Symbol;Acc:M	0.51947
ENSMUSG00000022799	Arhgap31	Rho GTPase activating protein 31 [Source:MGI Sym	0.51948

ENSMUSG00000007564	Ppp2r1a	protein phosphatase 2 (formerly 2A), regulatory su	0.51952
ENSMUSG00000097243	Gm26908	predicted gene, 26908 [Source:MGI Symbol;Acc:M(	0.51963
ENSMUSG00000020171	Yeats4	YEATS domain containing 4 [Source:MGI Symbol;Ac	0.5197
ENSMUSG00000024446	Rpp21	ribonuclease P 21 subunit [Source:MGI Symbol;Acc	0.5197
ENSMUSG00000041907	Gpr45	G protein-coupled receptor 45 [Source:MGI Symbo	0.51971
ENSMUSG00000034247	Plekhm1	pleckstrin homology domain containing, family M ('	0.51972
ENSMUSG00000022637	Cblb	Casitas B-lineage lymphoma b [Source:MGI Symbol	0.51997
ENSMUSG00000064585	Gm25129	predicted gene, 25129 [Source:MGI Symbol;Acc:M(	0.51997
ENSMUSG00000074826	Gm10767	predicted gene 10767 [Source:MGI Symbol;Acc:MG	0.52007
ENSMUSG00000022074	Tnfrsf10b	tumor necrosis factor receptor superfamily, membe	0.52017
ENSMUSG00000087436	Gm16156	predicted gene 16156 [Source:MGI Symbol;Acc:MG	0.52018
ENSMUSG00000084940	Gm15935	predicted gene 15935 [Source:MGI Symbol;Acc:MG	0.52019
ENSMUSG00000096999	Gm26793	predicted gene, 26793 [Source:MGI Symbol;Acc:M(	0.52026
ENSMUSG00000038195	Rilp	Rab interacting lysosomal protein [Source:MGI Sym	0.52032
ENSMUSG00000091272	Gm17641	predicted gene, 17641 [Source:MGI Symbol;Acc:M(	0.52032
ENSMUSG00000077633	Gm22148	predicted gene, 22148 [Source:MGI Symbol;Acc:M(	0.52033
ENSMUSG00000088485	Gm23640	predicted gene, 23640 [Source:MGI Symbol;Acc:M(	0.52034
ENSMUSG00000052848	C130026L21Rik	RIKEN cDNA C130026L21 gene [Source:MGI Symbo	0.52035
ENSMUSG00000062704	9430002A10Rik	RIKEN cDNA 9430002A10 gene [Source:MGI Symbo	0.52035
ENSMUSG00000084132	Gm15982	predicted gene 15982 [Source:MGI Symbol;Acc:MG	0.52036
ENSMUSG00000087618	Gm14114	predicted gene 14114 [Source:MGI Symbol;Acc:MG	0.52036
ENSMUSG00000090226	Gm16110	predicted gene 16110 [Source:MGI Symbol;Acc:MG	0.52036
ENSMUSG00000094557	Rpl35a-ps7	ribosomal protein L35A, pseudogene 7 [Source:MG	0.52036
ENSMUSG00000089607	Gm22500	predicted gene, 22500 [Source:MGI Symbol;Acc:M(	0.52037
ENSMUSG00000025216	Lbx1	ladybird homeobox homolog 1 (Drosophila) [Sourc	0.52038
ENSMUSG00000050241	Klre1	killer cell lectin-like receptor family E member 1 [Sc	0.52038
ENSMUSG00000065439	Mir140	microRNA 140 [Source:MGI Symbol;Acc:MGI:26768	0.52038
ENSMUSG00000086335	Gm12107	predicted gene 12107 [Source:MGI Symbol;Acc:MG	0.52038
ENSMUSG00000085748	Gm12280	predicted gene 12280 [Source:MGI Symbol;Acc:MG	0.52039
ENSMUSG00000048377	Foxi2	forkhead box I2 [Source:MGI Symbol;Acc:MGI:3028	0.5204
ENSMUSG00000055675	Kbtbd11	kelch repeat and BTB (POZ) domain containing 11 [	0.52046
ENSMUSG00000043857	Mgat5b	mannoside acetylglucosaminyltransferase 5, isoenz	0.52047
ENSMUSG00000050854	Tmem125	transmembrane protein 125 [Source:MGI Symbol;A	0.52052
ENSMUSG00000082585	Gm15387	predicted gene 15387 [Source:MGI Symbol;Acc:MG	0.5207
ENSMUSG00000039963	Ccdc40	coiled-coil domain containing 40 [Source:MGI Symb	0.52084
ENSMUSG00000010067	Rassf1	Ras association (RalGDS/AF-6) domain family meml	0.52096
ENSMUSG00000082917	Gm11440	predicted gene 11440 [Source:MGI Symbol;Acc:MG	0.52097
ENSMUSG00000004709	Cd244	CD244 natural killer cell receptor 2B4 [Source:MGI	0.52106
ENSMUSG00000082762	Gm12366	predicted gene 12366 [Source:MGI Symbol;Acc:MG	0.52109
ENSMUSG00000082861	Gm11362	predicted gene 11362 [Source:MGI Symbol;Acc:MG	0.52109
ENSMUSG00000073386	9830107B12Rik	RIKEN cDNA 9830107B12 gene [Source:MGI Symbo	0.5211
ENSMUSG00000077148	Gm22935	predicted gene, 22935 [Source:MGI Symbol;Acc:M(	0.52111
ENSMUSG00000060556	Olfr1418	olfactory receptor 1418 [Source:MGI Symbol;Acc:M	0.52112
ENSMUSG00000026249	Serpine2	serine (or cysteine) peptidase inhibitor, clade E, me	0.52117
ENSMUSG00000020374	Rasgef1c	RasGEF domain family, member 1C [Source:MGI Sy	0.52119
ENSMUSG00000032718	Mansc1	MANSC domain containing 1 [Source:MGI Symbol;A	0.52119
ENSMUSG00000031161	Hdac6	histone deacetylase 6 [Source:MGI Symbol;Acc:MG	0.52122

ENSMUSG00000058046	4933430I17Rik	RIKEN cDNA 4933430I17 gene [Source:MGI Symbol	0.52124
ENSMUSG00000054450	Gm9945	predicted gene 9945 [Source:MGI Symbol;Acc:MGI:	0.52125
ENSMUSG00000051703	Tmem198	transmembrane protein 198 [Source:MGI Symbol;A	0.52126
ENSMUSG00000078789	Dph1	DPH1 homolog (S. cerevisiae) [Source:MGI Symbol;	0.52135
ENSMUSG00000086773	Gm16192	predicted gene 16192 [Source:MGI Symbol;Acc:MG	0.5214
ENSMUSG00000047115	Fam221a	family with sequence similarity 221, member A [Sou	0.52143
ENSMUSG00000020910	Adprm	ADP-ribose/CDP-alcohol diphosphatase, manganese	0.52171
ENSMUSG00000097788	Gm16596	predicted gene, 16596 [Source:MGI Symbol;Acc:MG	0.52175
ENSMUSG00000050545	Fam228b	family with sequence similarity 228, member B [Sou	0.52176
ENSMUSG00000035258	Abi3bp	ABI gene family, member 3 (NESH) binding protein	0.52181
ENSMUSG00000084862	Gm16278	predicted gene 16278 [Source:MGI Symbol;Acc:MG	0.52193
ENSMUSG00000052673	Gm9887	predicted gene 9887 [Source:MGI Symbol;Acc:MGI:	0.52198
ENSMUSG00000006235	Epor	erythropoietin receptor [Source:MGI Symbol;Acc:M	0.52199
ENSMUSG00000071014	Ndufb6	NADH dehydrogenase (ubiquinone) 1 beta subcom	0.52199
ENSMUSG00000041954	Tnfrsf18	tumor necrosis factor receptor superfamily, membe	0.522
ENSMUSG00000092986	Gm25574	predicted gene, 25574 [Source:MGI Symbol;Acc:MG	0.52205
ENSMUSG00000031486	Gpr124	G protein-coupled receptor 124 [Source:MGI Symb	0.52208
ENSMUSG00000058900	Rsl1	regulator of sex limited protein 1 [Source:MGI Sym	0.52209
ENSMUSG00000003644	Rps6ka1	ribosomal protein S6 kinase polypeptide 1 [Source:	0.5221
ENSMUSG00000045100	Slc25a26	solute carrier family 25 (mitochondrial carrier, phos	0.52214
ENSMUSG00000097065	A630075F10Rik	RIKEN cDNA A630075F10 gene [Source:MGI Symbo	0.52225
ENSMUSG00000061458	Nol10	nucleolar protein 10 [Source:MGI Symbol;Acc:MGI:	0.52226
ENSMUSG00000021646	Mccc2	methylcrotonoyl-Coenzyme A carboxylase 2 (beta)	0.52227
ENSMUSG00000061755	Bod1l	biorientation of chromosomes in cell division 1-like	0.52227
ENSMUSG00000037104	Socs5	suppressor of cytokine signaling 5 [Source:MGI Sym	0.52229
ENSMUSG00000032657	Fam189b	family with sequence similarity 189, member B [Sou	0.52235
ENSMUSG00000025607	Copg2	coatomer protein complex, subunit gamma 2 [Sour	0.52251
ENSMUSG00000085150	Gm16346	predicted gene 16346 [Source:MGI Symbol;Acc:MG	0.52263
ENSMUSG00000001281	Itgb7	integrin beta 7 [Source:MGI Symbol;Acc:MGI:9661	0.52264
ENSMUSG00000058569	Tmed9	transmembrane emp24 protein transport domain c	0.52266
ENSMUSG00000042064	Myo3b	myosin IIIB [Source:MGI Symbol;Acc:MGI:2448580]	0.52269
ENSMUSG00000026578	Ccdc181	coiled-coil domain containing 181 [Source:MGI Sym	0.52287
ENSMUSG00000028730	Wdr65	WD repeat domain 65 [Source:MGI Symbol;Acc:MG	0.5229
ENSMUSG00000078531	Gm831	predicted gene 831 [Source:MGI Symbol;Acc:MGI:2	0.52291
ENSMUSG00000090026	Gm15996	predicted gene 15996 [Source:MGI Symbol;Acc:MG	0.52293
ENSMUSG00000040592	Cd79b	CD79B antigen [Source:MGI Symbol;Acc:MGI:96431	0.52294
ENSMUSG00000048899	Rimkla	ribosomal modification protein rimK-like family me	0.52299
ENSMUSG00000068924	Gm10253	predicted gene 10253 [Source:MGI Symbol;Acc:MG	0.52304
ENSMUSG00000022507	1810013L24Rik	RIKEN cDNA 1810013L24 gene [Source:MGI Symbo	0.52306
ENSMUSG00000084203	Gm15593	predicted gene 15593 [Source:MGI Symbol;Acc:MG	0.52307
ENSMUSG00000024245	Tmem178	transmembrane protein 178 [Source:MGI Symbol;A	0.52308
ENSMUSG00000062296	Trank1	tetratricopeptide repeat and ankyrin repeat contai	0.52309
ENSMUSG00000085146	Eif2c5	eukaryotic translation initiation factor 2C, 5 [Source	0.52309
ENSMUSG00000060030	Olfr317	olfactory receptor 317 [Source:MGI Symbol;Acc:MG	0.5231
ENSMUSG00000044424	Gm9493	predicted gene 9493 [Source:MGI Symbol;Acc:MGI:	0.52311
ENSMUSG00000083410	Gm14300	predicted gene 14300 [Source:MGI Symbol;Acc:MG	0.52312
ENSMUSG00000038179	Slamf7	SLAM family member 7 [Source:MGI Symbol;Acc:M	0.52314

ENSMUSG00000032586	Traip	TRAF-interacting protein [Source:MGI Symbol;Acc:M	0.52322
ENSMUSG00000003382	Etv3	ets variant gene 3 [Source:MGI Symbol;Acc:MGI:13	0.52336
ENSMUSG00000054405	Dnajc8	DnaJ (Hsp40) homolog, subfamily C, member 8 [Sou	0.52336
ENSMUSG00000087380	2210408F21Rik	RIKEN cDNA 2210408F21 gene [Source:MGI Symbo	0.52337
ENSMUSG00000066113	Adamtsl1	ADAMTS-like 1 [Source:MGI Symbol;Acc:MGI:1924	0.52339
ENSMUSG00000052794	1700030K09Rik	RIKEN cDNA 1700030K09 gene [Source:MGI Symbo	0.52346
ENSMUSG00000093673	Gm20644	predicted gene 20644 [Source:MGI Symbol;Acc:MG	0.52353
ENSMUSG00000044562	Rasip1	Ras interacting protein 1 [Source:MGI Symbol;Acc:M	0.52357
ENSMUSG00000022558	Mroh1	maestro heat-like repeat family member 1 [Source:	0.52368
ENSMUSG00000003992	Ssbp2	single-stranded DNA binding protein 2 [Source:MGI	0.5238
ENSMUSG00000027371	Fahd2a	fumarylacetoacetate hydrolase domain containing	0.52385
ENSMUSG00000087088	Gm16638	predicted gene, 16638 [Source:MGI Symbol;Acc:M	0.52388
ENSMUSG00000038225	Ccdc111	coiled-coil domain containing 111 [Source:MGI Sym	0.52389
ENSMUSG00000021577	Sdha	succinate dehydrogenase complex, subunit A, flavo	0.5239
ENSMUSG00000097017	Gm26707	predicted gene, 26707 [Source:MGI Symbol;Acc:M	0.52391
ENSMUSG00000073729	Gm6420	predicted gene 6420 [Source:MGI Symbol;Acc:MGI	0.52393
ENSMUSG00000086560	Gm13372	predicted gene 13372 [Source:MGI Symbol;Acc:MG	0.52393
ENSMUSG00000002307	Daxx	Fas death domain-associated protein [Source:MGI	0.52395
ENSMUSG00000032202	Rab27a	RAB27A, member RAS oncogene family [Source:M	0.52401
ENSMUSG00000098092	Gm2076	predicted gene 2076 [Source:MGI Symbol;Acc:MGI	0.52403
ENSMUSG00000088605	Gm24188	predicted gene, 24188 [Source:MGI Symbol;Acc:M	0.52406
ENSMUSG00000064916	Gm22573	predicted gene, 22573 [Source:MGI Symbol;Acc:M	0.52412
ENSMUSG00000024246	Thumpd2	THUMP domain containing 2 [Source:MGI Symbol;	0.52415
ENSMUSG00000039318	Rab3gap2	RAB3 GTPase activating protein subunit 2 [Source:	0.52418
ENSMUSG00000094091	Gm21885	predicted gene, 21885 [Source:MGI Symbol;Acc:M	0.5242
ENSMUSG00000044471	AB041803	cDNA sequence AB041803 [Source:MGI Symbol;Ac	0.52423
ENSMUSG00000087442	Gm14453	predicted gene 14453 [Source:MGI Symbol;Acc:MG	0.52425
ENSMUSG00000083921	Gm15750	predicted gene 15750 [Source:MGI Symbol;Acc:MG	0.52426
ENSMUSG00000080736	Gm15617	predicted gene 15617 [Source:MGI Symbol;Acc:MG	0.52428
ENSMUSG00000084870	Gm13954	predicted gene 13954 [Source:MGI Symbol;Acc:MG	0.52428
ENSMUSG00000064982	Gm25259	predicted gene, 25259 [Source:MGI Symbol;Acc:M	0.52432
ENSMUSG00000032065	Tex12	testis expressed gene 12 [Source:MGI Symbol;Acc:	0.52435
ENSMUSG00000062593	Lilrb4	leukocyte immunoglobulin-like receptor, subfamily	0.52438
ENSMUSG00000050312	Nsun3	NOL1/NOP2/Sun domain family member 3 [Source	0.52439
ENSMUSG00000029634	Rnf6	ring finger protein (C3H2C3 type) 6 [Source:MGI Sy	0.52448
ENSMUSG00000053472	Gm13534	predicted gene 13534 [Source:MGI Symbol;Acc:MG	0.52448
ENSMUSG00000032615	Nt5m	5',3'-nucleotidase, mitochondrial [Source:MGI Sym	0.5246
ENSMUSG00000000693	Loxl3	lysyl oxidase-like 3 [Source:MGI Symbol;Acc:MGI:1	0.52465
ENSMUSG00000040734	Ppp1r13l	protein phosphatase 1, regulatory (inhibitor) subu	0.52466
ENSMUSG00000077787	Gm23908	predicted gene, 23908 [Source:MGI Symbol;Acc:M	0.52468
ENSMUSG00000023800	Tiam2	T cell lymphoma invasion and metastasis 2 [Source	0.52473
ENSMUSG00000024067	Dpy30	dpy-30 homolog (C. elegans) [Source:MGI Symbol;	0.52475
ENSMUSG00000025090	Ccdc172	coiled-coil domain containing 172 [Source:MGI Sym	0.5248
ENSMUSG00000025333	Gpr143	G protein-coupled receptor 143 [Source:MGI Symb	0.52489
ENSMUSG00000044306	4930500M09Rik	RIKEN cDNA 4930500M09 gene [Source:MGI Symb	0.52496
ENSMUSG00000069540	Gm4925	predicted gene 4925 [Source:MGI Symbol;Acc:MGI	0.52498
ENSMUSG00000005378	Wbscr22	Williams Beuren syndrome chromosome region 22	0.52502

ENSMUSG00000080542	Gm22710	predicted gene, 22710 [Source:MGI Symbol;Acc:M(	0.52506
ENSMUSG00000085048	Gm15537	predicted gene 15537 [Source:MGI Symbol;Acc:MG	0.52506
ENSMUSG00000060380	C030014I23Rik	RIKEN cDNA C030014I23 gene [Source:MGI Symbol	0.52508
ENSMUSG00000037703	Lzts3	leucine zipper, putative tumor suppressor family m	0.5252
ENSMUSG00000028669	Pithd1	PITH (C-terminal proteasome-interacting domain of	0.52523
ENSMUSG00000026869	Psmd5	proteasome (prosome, macropain) 26S subunit, no	0.52528
ENSMUSG00000025484	Bet1l	blocked early in transport 1 homolog (S. cerevisiae)	0.5253
ENSMUSG00000067586	S1pr3	sphingosine-1-phosphate receptor 3 [Source:MGI S	0.5253
ENSMUSG00000089839	Rps19-ps11	ribosomal protein S19, pseudogene 11 [Source:MG	0.52535
ENSMUSG00000092371	Gm20511	predicted gene 20511 [Source:MGI Symbol;Acc:MG	0.52536
ENSMUSG00000097573	G730003C15Rik	RIKEN cDNA G730003C15 gene [Source:MGI Symbo	0.52557
ENSMUSG00000050377	Il31ra	interleukin 31 receptor A [Source:MGI Symbol;Acc:	0.52566
ENSMUSG00000030082	Sec61a1	Sec61 alpha 1 subunit (S. cerevisiae) [Source:MGI S	0.52571
ENSMUSG00000097610	A930012L18Rik	RIKEN cDNA A930012L18 gene [Source:MGI Symbo	0.52572
ENSMUSG00000040018	Cox15	cytochrome c oxidase assembly protein 15 [Source:	0.52575
ENSMUSG00000034868	Myl12b	myosin, light chain 12B, regulatory [Source:MGI Syr	0.52576
ENSMUSG00000026546	Ccdc19	coiled-coil domain containing 19 [Source:MGI Symb	0.5258
ENSMUSG00000001095	Slc13a2	solute carrier family 13 (sodium-dependent dicarbo	0.52583
ENSMUSG00000073616	Myeov2	myeloma overexpressed 2 [Source:MGI Symbol;Acc	0.52583
ENSMUSG00000040367	Lrrd1	leucine rich repeats and death domain containing 1	0.52593
ENSMUSG00000026443	Lrrn2	leucine rich repeat protein 2, neuronal [Source:MG	0.52596
ENSMUSG00000042590	Ipo11	importin 11 [Source:MGI Symbol;Acc:MGI:2442377	0.52597
ENSMUSG00000087503	Gm11459	predicted gene 11459 [Source:MGI Symbol;Acc:MG	0.52613
ENSMUSG00000042500	Ago4	argonaute RISC catalytic subunit 4 [Source:MGI Syn	0.52615
ENSMUSG00000081094	Rpl19-ps11	ribosomal protein L19, pseudogene 11 [Source:MG	0.52621
ENSMUSG00000054999	Naaladl1	N-acetylated alpha-linked acidic dipeptidase-like 1	0.52623
ENSMUSG00000055061	4931431C16Rik	RIKEN cDNA 4931431C16 gene [Source:MGI Symbo	0.52623
ENSMUSG00000034308	Sdr42e1	short chain dehydrogenase/reductase family 42E, n	0.52625
ENSMUSG00000098190	Gm5210	predicted gene 5210 [Source:MGI Symbol;Acc:MGI:	0.52631
ENSMUSG00000089429	Gm26004	predicted gene, 26004 [Source:MGI Symbol;Acc:M(	0.52634
ENSMUSG00000097998	Gm27019	predicted gene, 27019 [Source:MGI Symbol;Acc:M(	0.52634
ENSMUSG00000088169	Gm22059	predicted gene, 22059 [Source:MGI Symbol;Acc:M(	0.52636
ENSMUSG00000085624	Gm14573	predicted gene 14573 [Source:MGI Symbol;Acc:MG	0.52638
ENSMUSG00000035186	Ubd	ubiquitin D [Source:MGI Symbol;Acc:MGI:1344410]	0.52639
ENSMUSG00000024207	Acsbg2	acyl-CoA synthetase bubblegum family member 2 [	0.5264
ENSMUSG00000083852	Gm13007	predicted gene 13007 [Source:MGI Symbol;Acc:MG	0.52641
ENSMUSG00000031293	Rs1	retinoschisis (X-linked, juvenile) 1 (human) [Source:	0.52653
ENSMUSG00000047180	Neurl3	neuralized homolog 3 homolog (Drosophila) [Sourc	0.52655
ENSMUSG00000032267	Usp28	ubiquitin specific peptidase 28 [Source:MGI Symbo	0.52662
ENSMUSG00000039954	Stk32a	serine/threonine kinase 32A [Source:MGI Symbol;A	0.52662
ENSMUSG00000005312	Ubqln1	ubiquilin 1 [Source:MGI Symbol;Acc:MGI:1860276]	0.52663
ENSMUSG00000082941	Gm12850	predicted gene 12850 [Source:MGI Symbol;Acc:MG	0.52665
ENSMUSG00000034107	Ano7	anoctamin 7 [Source:MGI Symbol;Acc:MGI:305271	0.52675
ENSMUSG00000030884	Uqcrc2	ubiquinol cytochrome c reductase core protein 2 [S	0.52688
ENSMUSG00000024998	Plce1	phospholipase C, epsilon 1 [Source:MGI Symbol;Ac	0.52694
ENSMUSG00000000530	Acvrl1	activin A receptor, type II-like 1 [Source:MGI Symbo	0.527
ENSMUSG00000030315	Vgll4	vestigial like 4 (Drosophila) [Source:MGI Symbol;Ac	0.527

ENSMUSG00000097811	2810425M01Rik	RIKEN cDNA 2810425M01 gene [Source:MGI Symb	0.52719
ENSMUSG00000043323	Fbrsl1	fibrosin-like 1 [Source:MGI Symbol;Acc:MGI:19209	0.5272
ENSMUSG00000024350	Dnajc18	DnaJ (Hsp40) homolog, subfamily C, member 18 [S	0.52724
ENSMUSG00000090101	Snhg9	small nucleolar RNA host gene (non-protein coding	0.5273
ENSMUSG00000074281	Gm12522	predicted gene 12522 [Source:MGI Symbol;Acc:MG	0.52731
ENSMUSG00000060029	4930473A02Rik	RIKEN cDNA 4930473A02 gene [Source:MGI Symbo	0.52739
ENSMUSG00000042529	Kcnj12	potassium inwardly-rectifying channel, subfamily J,	0.52756
ENSMUSG00000054931	Zkscan4	zinc finger with KRAB and SCAN domains 4 [Source:	0.52758
ENSMUSG00000054659	Pm20d2	peptidase M20 domain containing 2 [Source:MGI S	0.52771
ENSMUSG00000073771	Btbd19	BTB (POZ) domain containing 19 [Source:MGI Symb	0.5279
ENSMUSG00000056515	Rab31	RAB31, member RAS oncogene family [Source:MGI	0.52797
ENSMUSG00000037363	Letm2	leucine zipper-EF-hand containing transmembrane	0.52802
ENSMUSG00000097887	Gm26542	predicted gene, 26542 [Source:MGI Symbol;Acc:M	0.52813
ENSMUSG00000009216	Fam163b	family with sequence similarity 163, member B [So	0.52816
ENSMUSG00000091133	Gm17108	predicted gene 17108 [Source:MGI Symbol;Acc:MG	0.52816
ENSMUSG00000091177	Gm15494	predicted gene 15494 [Source:MGI Symbol;Acc:MG	0.52824
ENSMUSG00000028476	Reck	reversion-inducing-cysteine-rich protein with kazal	0.52826
ENSMUSG00000025745	Hadha	hydroxyacyl-Coenzyme A dehydrogenase/3-ketoac	0.5283
ENSMUSG00000072596	Ear2	eosinophil-associated, ribonuclease A family, mem	0.52836
ENSMUSG00000086284	2900093L17Rik	RIKEN cDNA 2900093L17 gene [Source:MGI Symbo	0.52841
ENSMUSG00000049299	Trappc1	trafficking protein particle complex 1 [Source:MGI	0.52842
ENSMUSG00000051246	Msantd1	Myb/SANT-like DNA-binding domain containing 1 [	0.5286
ENSMUSG00000087475	4933406I18Rik	RIKEN cDNA 4933406I18 gene [Source:MGI Symbol	0.52861
ENSMUSG00000016200	Syt14	synaptotagmin XIV [Source:MGI Symbol;Acc:MGI:2	0.52872
ENSMUSG00000034300	Fam53c	family with sequence similarity 53, member C [Sou	0.52875
ENSMUSG00000029512	Ulk1	unc-51 like kinase 1 [Source:MGI Symbol;Acc:MGI:	0.52877
ENSMUSG00000097857	Gm26603	predicted gene, 26603 [Source:MGI Symbol;Acc:M	0.52877
ENSMUSG00000060177	Klk1b22	kallikrein 1-related peptidase b22 [Source:MGI Sym	0.52878
ENSMUSG00000078444	Gm10941	predicted gene 10941 [Source:MGI Symbol;Acc:MG	0.52878
ENSMUSG00000021556	Golm1	golgi membrane protein 1 [Source:MGI Symbol;Acc	0.52879
ENSMUSG00000026311	Asb1	ankyrin repeat and SOCS box-containing 1 [Source:	0.52891
ENSMUSG00000029847	2010107G12Rik	RIKEN cDNA 2010107G12 gene [Source:MGI Symbo	0.52891
ENSMUSG00000027243	Harbi1	harbinger transposase derived 1 [Source:MGI Symb	0.52897
ENSMUSG00000029413	Naaa	N-acylethanolamine acid amidase [Source:MGI Sym	0.52911
ENSMUSG00000035493	Tgfbi	transforming growth factor, beta induced [Source:	0.52912
ENSMUSG00000062073	Gm10109	predicted gene 10109 [Source:MGI Symbol;Acc:MG	0.52917
ENSMUSG00000076258	Gm23935	predicted gene, 23935 [Source:MGI Symbol;Acc:M	0.52919
ENSMUSG00000041187	Prkd2	protein kinase D2 [Source:MGI Symbol;Acc:MGI:21	0.52925
ENSMUSG00000009894	Snap47	synaptosomal-associated protein, 47 [Source:MGI	0.52927
ENSMUSG00000025983	Ccdc150	coiled-coil domain containing 150 [Source:MGI Sym	0.5293
ENSMUSG00000037103	Dcaf15	DDB1 and CUL4 associated factor 15 [Source:MGI S	0.52934
ENSMUSG00000094153	Gm8807	predicted gene 8807 [Source:MGI Symbol;Acc:MGI	0.5294
ENSMUSG00000045259	Klhdc9	kelch domain containing 9 [Source:MGI Symbol;Acc	0.52946
ENSMUSG00000066108	Muc5b	mucin 5, subtype B, tracheobronchial [Source:MGI	0.52946
ENSMUSG00000090293	Gm17034	predicted gene 17034 [Source:MGI Symbol;Acc:MG	0.52956
ENSMUSG00000019295	Tmem129	transmembrane protein 129 [Source:MGI Symbol;A	0.52959
ENSMUSG00000084349	Rpl3-ps1	ribosomal protein L3, pseudogene 1 [Source:MGI S	0.52972

ENSMUSG00000020175	Rab36	RAB36, member RAS oncogene family [Source:MGI	0.52973
ENSMUSG00000037660	Gdf7	growth differentiation factor 7 [Source:MGI Symbo	0.52984
ENSMUSG00000044224	Dnajc21	DnaJ (Hsp40) homolog, subfamily C, member 21 [So	0.53013
ENSMUSG00000032968	Inha	inhibin alpha [Source:MGI Symbol;Acc:MGI:96569]	0.53016
ENSMUSG00000029206	Nsun7	NOL1/NOP2/Sun domain family, member 7 [Source	0.53017
ENSMUSG00000093319	Gm25467	predicted gene, 25467 [Source:MGI Symbol;Acc:MG	0.53048
ENSMUSG00000090007	Rpl30-ps2	ribosomal protein L30, pseudogene 2 [Source:MGI	0.53051
ENSMUSG00000029275	Gfi1	growth factor independent 1 [Source:MGI Symbol;A	0.53052
ENSMUSG00000040147	Maob	monoamine oxidase B [Source:MGI Symbol;Acc:MG	0.53052
ENSMUSG00000066442	Mthfs	5, 10-methenyltetrahydrofolate synthetase [Source	0.53052
ENSMUSG00000037325	Bbs7	Bardet-Biedl syndrome 7 (human) [Source:MGI Sym	0.53054
ENSMUSG00000020907	Rcvrn	recoverin [Source:MGI Symbol;Acc:MGI:97883]	0.53056
ENSMUSG00000036461	Elf1	E74-like factor 1 [Source:MGI Symbol;Acc:MGI:107	0.53059
ENSMUSG00000040990	Sh3kbp1	SH3-domain kinase binding protein 1 [Source:MGI S	0.5306
ENSMUSG00000037395	Rcor3	REST corepressor 3 [Source:MGI Symbol;Acc:MGI:2	0.5308
ENSMUSG00000017679	Ttpal	tocopherol (alpha) transfer protein-like [Source:MG	0.53082
ENSMUSG00000025389	Mip	major intrinsic protein of eye lens fiber [Source:MG	0.53085
ENSMUSG00000047108	Dnajb7	DnaJ (Hsp40) homolog, subfamily B, member 7 [Sou	0.53086
ENSMUSG00000091306	Gm17606	predicted gene, 17606 [Source:MGI Symbol;Acc:MG	0.53098
ENSMUSG00000052508	Olfr536	olfactory receptor 536 [Source:MGI Symbol;Acc:MG	0.53099
ENSMUSG00000081731	Gm14078	predicted gene 14078 [Source:MGI Symbol;Acc:MG	0.531
ENSMUSG00000002228	Ppm1j	protein phosphatase 1J [Source:MGI Symbol;Acc:M	0.53104
ENSMUSG00000045237	1110012L19Rik	RIKEN cDNA 1110012L19 gene [Source:MGI Symbo	0.53105
ENSMUSG00000029861	Fam131b	family with sequence similarity 131, member B [Sou	0.53117
ENSMUSG00000031666	Rbl2	retinoblastoma-like 2 [Source:MGI Symbol;Acc:MG	0.53126
ENSMUSG00000087011	Gm12930	predicted gene 12930 [Source:MGI Symbol;Acc:MG	0.53128
ENSMUSG00000050157	Gm867	predicted gene 867 [Source:MGI Symbol;Acc:MGI:2	0.53134
ENSMUSG00000022855	Senp2	SUMO/sentrin specific peptidase 2 [Source:MGI Syr	0.53136
ENSMUSG00000024201	Kdm4b	lysine (K)-specific demethylase 4B [Source:MGI Sym	0.53137
ENSMUSG00000002068	Ccne1	cyclin E1 [Source:MGI Symbol;Acc:MGI:88316]	0.53138
ENSMUSG00000028580	Pum1	pumilio 1 (Drosophila) [Source:MGI Symbol;Acc:MG	0.53138
ENSMUSG00000034269	Setd5	SET domain containing 5 [Source:MGI Symbol;Acc:M	0.53149
ENSMUSG00000063334	Krr1	KRR1, small subunit (SSU) processome component,	0.53149
ENSMUSG00000032508	Myd88	myeloid differentiation primary response gene 88 [	0.53153
ENSMUSG00000089862	Gm16039	predicted gene 16039 [Source:MGI Symbol;Acc:MG	0.53155
ENSMUSG00000022283	Pabpc1	poly(A) binding protein, cytoplasmic 1 [Source:MGI	0.53156
ENSMUSG00000098220	Gm27031	predicted gene, 27031 [Source:MGI Symbol;Acc:MG	0.53157
ENSMUSG00000028521	Slc35d1	solute carrier family 35 (UDP-glucuronic acid/UDP-I	0.53159
ENSMUSG00000052271	Bhlha15	basic helix-loop-helix family, member a15 [Source:N	0.5316
ENSMUSG00000041438	Cirh1a	cirrhosis, autosomal recessive 1A (human) [Source:	0.53162
ENSMUSG00000030407	Qpctl	glutaminyl-peptide cyclotransferase-like [Source:M	0.53172
ENSMUSG00000028974	Dffa	DNA fragmentation factor, alpha subunit [Source:M	0.53184
ENSMUSG00000027374	Mrps5	mitochondrial ribosomal protein S5 [Source:MGI Sy	0.53187
ENSMUSG00000032064	Dixdc1	DIX domain containing 1 [Source:MGI Symbol;Acc:M	0.5319
ENSMUSG00000048108	Tmem72	transmembrane protein 72 [Source:MGI Symbol;Ac	0.53216
ENSMUSG00000034071	Zfp551	zinc fingr protein 551 [Source:MGI Symbol;Acc:MGI	0.5322
ENSMUSG00000031609	Sap30	sin3 associated polypeptide [Source:MGI Symbol;Ac	0.53222

ENSMUSG00000023236	Scg5	secretogranin V [Source:MGI Symbol;Acc:MGI:9828	0.53233
ENSMUSG00000030616	Sytl2	synaptotagmin-like 2 [Source:MGI Symbol;Acc:MGI	0.53239
ENSMUSG00000050188	Lsm10	U7 snRNP-specific Sm-like protein LSM10 [Source:N	0.53246
ENSMUSG00000022529	Zfp263	zinc finger protein 263 [Source:MGI Symbol;Acc:MG	0.53249
ENSMUSG00000083830	Gm8839	predicted gene 8839 [Source:MGI Symbol;Acc:MGI:	0.53249
ENSMUSG00000032575	Manf	mesencephalic astrocyte-derived neurotrophic fact	0.53254
ENSMUSG00000041235	Chd7	chromodomain helicase DNA binding protein 7 [Sou	0.53259
ENSMUSG00000086430	4930551O13Rik	RIKEN cDNA 4930551O13 gene [Source:MGI Symbo	0.5327
ENSMUSG00000070794	BC016548	cDNA sequence BC016548 [Source:MGI Symbol;Acc	0.53294
ENSMUSG00000068391	Chrac1	chromatin accessibility complex 1 [Source:MGI Sym	0.53306
ENSMUSG00000029149	Krtcap3	keratinocyte associated protein 3 [Source:MGI Sym	0.53309
ENSMUSG00000001504	Irx2	Iroquois related homeobox 2 (Drosophila) [Source:I	0.53311
ENSMUSG00000097920	Gm7784	predicted gene 7784 [Source:MGI Symbol;Acc:MGI:	0.53315
ENSMUSG00000083139	Gm12418	predicted gene 12418 [Source:MGI Symbol;Acc:MG	0.53319
ENSMUSG00000082509	Gm15673	predicted gene 15673 [Source:MGI Symbol;Acc:MG	0.53322
ENSMUSG00000025328	Padi3	peptidyl arginine deiminase, type III [Source:MGI Sy	0.53325
ENSMUSG00000031491	Chrna6	cholinergic receptor, nicotinic, alpha polypeptide 6	0.53328
ENSMUSG00000025534	Gusb	glucuronidase, beta [Source:MGI Symbol;Acc:MGI:9	0.53335
ENSMUSG00000021336	Slc17a4	solute carrier family 17 (sodium phosphate), memb	0.53337
ENSMUSG00000030881	Arfip2	ADP-ribosylation factor interacting protein 2 [Sourc	0.53337
ENSMUSG00000085623	Gm16041	predicted gene 16041 [Source:MGI Symbol;Acc:MG	0.53363
ENSMUSG00000097288	Gm2155	predicted gene 2155 [Source:MGI Symbol;Acc:MGI:	0.53363
ENSMUSG00000022613	Miox	myo-inositol oxygenase [Source:MGI Symbol;Acc:M	0.53377
ENSMUSG00000044021	Muc19	mucin 19 [Source:MGI Symbol;Acc:MGI:2676278]	0.53415
ENSMUSG00000046207	Pik3r6	phosphoinositide-3-kinase, regulatory subunit 6 [Sc	0.53423
ENSMUSG00000021235	Coq6	coenzyme Q6 homolog (yeast) [Source:MGI Symbo	0.53433
ENSMUSG00000030695	Aldoa	aldolase A, fructose-bisphosphate [Source:MGI Sym	0.53434
ENSMUSG00000068115	Ninl	ninein-like [Source:MGI Symbol;Acc:MGI:1925427]	0.53447
ENSMUSG00000018500	Adora2b	adenosine A2b receptor [Source:MGI Symbol;Acc:M	0.53457
ENSMUSG00000098176	Ccdc166	coiled-coil domain containing 166 [Source:MGI Sym	0.53459
ENSMUSG00000038026	Kcnj9	potassium inwardly-rectifying channel, subfamily J,	0.5346
ENSMUSG00000021271	Zfp839	zinc finger protein 839 [Source:MGI Symbol;Acc:MG	0.53462
ENSMUSG00000087263	Gm15726	predicted gene 15726 [Source:MGI Symbol;Acc:MG	0.53464
ENSMUSG00000090260	2810442N19Rik	RIKEN cDNA 2810442N19 gene [Source:MGI Symbo	0.53468
ENSMUSG00000039911	Spsb1	splA/ryanodine receptor domain and SOCS box con	0.53469
ENSMUSG00000026162	Nhej1	nonhomologous end-joining factor 1 [Source:MGI S	0.53477
ENSMUSG00000071454	Dtnb	dystrobrevin, beta [Source:MGI Symbol;Acc:MGI:12	0.53484
ENSMUSG00000033852	Pla2g4b	phospholipase A2, group IVB (cytosolic) [Source:MG	0.53485
ENSMUSG00000078308	Gm7293	predicted gene 7293 [Source:MGI Symbol;Acc:MGI:	0.53489
ENSMUSG00000032840	2410131K14Rik	RIKEN cDNA 2410131K14 gene [Source:MGI Symbo	0.53499
ENSMUSG00000053656	Dancr	differentiation antagonizing non-protein coding RN	0.53507
ENSMUSG00000087291	Gm11946	predicted gene 11946 [Source:MGI Symbol;Acc:MG	0.53511
ENSMUSG00000073877	Gm13306	predicted gene 13306 [Source:MGI Symbol;Acc:MG	0.53515
ENSMUSG00000047554	Tmem41b	transmembrane protein 41B [Source:MGI Symbol;A	0.53521
ENSMUSG00000016942	Tmprss6	transmembrane serine protease 6 [Source:MGI Syn	0.53549
ENSMUSG00000022575	Gsdmd	gasdermin D [Source:MGI Symbol;Acc:MGI:191639	0.53557
ENSMUSG00000070997	1700055D18Rik	RIKEN cDNA 1700055D18 gene [Source:MGI Symbo	0.53586

ENSMUSG00000006651	Aplp1	amyloid beta (A4) precursor-like protein 1 [Source:	0.53588
ENSMUSG00000021039	Snw1	SNW domain containing 1 [Source:MGI Symbol;Acc	0.53597
ENSMUSG00000078247	Airn	antisense Igf2r RNA [Source:MGI Symbol;Acc:MGI:1	0.53601
ENSMUSG00000022488	Nckap1l	NCK associated protein 1 like [Source:MGI Symbol;A	0.53602
ENSMUSG00000032580	Rbm5	RNA binding motif protein 5 [Source:MGI Symbol;A	0.53605
ENSMUSG00000081965	Gm11620	predicted gene 11620 [Source:MGI Symbol;Acc:MG	0.5361
ENSMUSG00000022793	B4galt4	UDP-Gal:betaGlcNAc beta 1,4-galactosyltransferase	0.53612
ENSMUSG00000090256	Alms1-ps1	Alstrom syndrome 1, pseudogene 1 [Source:MGI Sy	0.53613
ENSMUSG00000030787	Lyve1	lymphatic vessel endothelial hyaluronan receptor 1	0.5363
ENSMUSG00000052769	Gm9889	predicted gene 9889 [Source:MGI Symbol;Acc:MGI:	0.5365
ENSMUSG00000021114	Atp6v1d	ATPase, H+ transporting, lysosomal V1 subunit D [S	0.53652
ENSMUSG00000001763	Tspan33	tetraspanin 33 [Source:MGI Symbol;Acc:MGI:19190	0.53664
ENSMUSG00000088177	Gm23098	predicted gene, 23098 [Source:MGI Symbol;Acc:MG	0.53665
ENSMUSG00000038055	Dexi	dexamethasone-induced transcript [Source:MGI Sy	0.53667
ENSMUSG00000027378	Nphp1	nephronophthisis 1 (juvenile) homolog (human) [So	0.53668
ENSMUSG00000026121	Sema4c	sema domain, immunoglobulin domain (Ig), transm	0.5368
ENSMUSG00000041831	Sytl3	synaptotagmin-like 3 [Source:MGI Symbol;Acc:MGI	0.53684
ENSMUSG00000048787	Dcun1d3	DCN1, defective in cullin neddylation 1, domain cor	0.53685
ENSMUSG00000030247	Kcnj8	potassium inwardly-rectifying channel, subfamily J,	0.53693
ENSMUSG00000087579	1500017E21Rik	RIKEN cDNA 1500017E21 gene [Source:MGI Symbo	0.53701
ENSMUSG00000033124	Atg9a	autophagy related 9A [Source:MGI Symbol;Acc:MG	0.53714
ENSMUSG00000073492	Gm10521	predicted gene 10521 [Source:MGI Symbol;Acc:MG	0.53728
ENSMUSG00000086472	Gm16172	predicted gene 16172 [Source:MGI Symbol;Acc:MG	0.53728
ENSMUSG00000086772	Gm11705	predicted gene 11705 [Source:MGI Symbol;Acc:MG	0.53728
ENSMUSG00000037846	Rtkn2	rhotekin 2 [Source:MGI Symbol;Acc:MGI:2158417]	0.53729
ENSMUSG00000034271	Jdp2	Jun dimerization protein 2 [Source:MGI Symbol;Acc	0.53745
ENSMUSG00000038696	Mapkap1	mitogen-activated protein kinase associated protei	0.53754
ENSMUSG00000036687	Tmem184a	transmembrane protein 184a [Source:MGI Symbol;	0.53755
ENSMUSG00000035666	Gtf3c4	general transcription factor IIIC, polypeptide 4 [Sou	0.53756
ENSMUSG00000080573	Mir467f	microRNA 467f [Source:MGI Symbol;Acc:MGI:3783	0.53758
ENSMUSG00000045098	Suv420h1	suppressor of variegation 4-20 homolog 1 (Drosoph	0.5376
ENSMUSG00000085896	5330429C05Rik	RIKEN cDNA 5330429C05 gene [Source:MGI Symbo	0.53763
ENSMUSG00000081113	Gm7308	predicted pseudogene 7308 [Source:MGI Symbol;A	0.53765
ENSMUSG00000054759	Krtap5-2	keratin associated protein 5-2 [Source:MGI Symbol	0.53766
ENSMUSG00000082277	Gm15807	predicted gene 15807 [Source:MGI Symbol;Acc:MG	0.53767
ENSMUSG00000029368	Alb	albumin [Source:MGI Symbol;Acc:MGI:87991]	0.53768
ENSMUSG00000078202	Nrarp	Notch-regulated ankyrin repeat protein [Source:MG	0.53768
ENSMUSG00000022496	Tnfrsf17	tumor necrosis factor receptor superfamily, membe	0.53769
ENSMUSG00000009633	G0s2	G0/G1 switch gene 2 [Source:MGI Symbol;Acc:MGI	0.53776
ENSMUSG00000049100	Pcdh10	protocadherin 10 [Source:MGI Symbol;Acc:MGI:133	0.53777
ENSMUSG00000000731	Aire	autoimmune regulator (autoimmune polyendocrine	0.53785
ENSMUSG00000025393	Atp5b	ATP synthase, H+ transporting mitochondrial F1 com	0.53785
ENSMUSG00000005882	Uqcc1	ubiquinol-cytochrome c reductase complex assemb	0.53792
ENSMUSG00000023262	Acy1	aminoacylase 1 [Source:MGI Symbol;Acc:MGI:8791	0.53792
ENSMUSG00000097446	Gm26629	predicted gene, 26629 [Source:MGI Symbol;Acc:MG	0.53799
ENSMUSG00000038712	Fam63a	family with sequence similarity 63, member A [Sour	0.53807
ENSMUSG00000036333	Kidins220	kinase D-interacting substrate 220 [Source:MGI Syr	0.53809

ENSMUSG00000015112	Slc25a13	solute carrier family 25 (mitochondrial carrier, ader	0.5381
ENSMUSG00000040340	1700019B03Rik	RIKEN cDNA 1700019B03 gene [Source:MGI Symbo	0.53814
ENSMUSG00000026867	Gapvd1	GTPase activating protein and VPS9 domains 1 [Sou	0.53823
ENSMUSG00000030096	Slc6a6	solute carrier family 6 (neurotransmitter transporte	0.53825
ENSMUSG00000028809	Srrm1	serine/arginine repetitive matrix 1 [Source:MGI Syr	0.53832
ENSMUSG00000034930	Rtkn	rhotekin [Source:MGI Symbol;Acc:MGI:107371]	0.53857
ENSMUSG00000088014	Gm23743	predicted gene, 23743 [Source:MGI Symbol;Acc:M(	0.53859
ENSMUSG00000082744	Gm15625	predicted gene 15625 [Source:MGI Symbol;Acc:MG	0.53861
ENSMUSG00000036244	Tbc1d21	TBC1 domain family, member 21 [Source:MGI Syml	0.53864
ENSMUSG00000037523	Mavs	mitochondrial antiviral signaling protein [Source:M	0.53865
ENSMUSG00000036615	Rfxap	regulatory factor X-associated protein [Source:MGI	0.53867
ENSMUSG00000025645	Ccdc51	coiled-coil domain containing 51 [Source:MGI Symb	0.53877
ENSMUSG00000032109	Nlrx1	NLR family member X1 [Source:MGI Symbol;Acc:M(	0.5389
ENSMUSG00000032977	Fam207a	family with sequence similarity 207, member A [Sou	0.53896
ENSMUSG00000087603	Gm11339	predicted gene 11339 [Source:MGI Symbol;Acc:MG	0.53899
ENSMUSG00000036853	Mcoln3	mucolipin 3 [Source:MGI Symbol;Acc:MGI:1890500	0.539
ENSMUSG00000042476	Abcb4	ATP-binding cassette, sub-family B (MDR/TAP), mer	0.53903
ENSMUSG00000087408	Cers1	ceramide synthase 1 [Source:MGI Symbol;Acc:MGI:	0.53911
ENSMUSG00000033752	Mnd1	meiotic nuclear divisions 1 homolog (S. cerevisiae)	0.53916
ENSMUSG00000031642	Sh3rf1	SH3 domain containing ring finger 1 [Source:MGI Sy	0.53918
ENSMUSG00000095454	Gm11273	predicted gene 11273 [Source:MGI Symbol;Acc:MG	0.53918
ENSMUSG00000085001	BB114351	expressed sequence BB114351 [Source:MGI Symbo	0.53928
ENSMUSG00000031508	Ankrd10	ankyrin repeat domain 10 [Source:MGI Symbol;Acc	0.5393
ENSMUSG00000050052	Tdrp	testis development related protein [Source:MGI Sy	0.53944
ENSMUSG00000033751	Gadd45gip1	growth arrest and DNA-damage-inducible, gamma	0.53947
ENSMUSG00000070111	Gm10286	predicted gene 10286 [Source:MGI Symbol;Acc:MG	0.53952
ENSMUSG00000086545	Gm12	predicted gene 12 [Source:MGI Symbol;Acc:MGI:26	0.53978
ENSMUSG00000040086	Tnni3k	TNNI3 interacting kinase [Source:MGI Symbol;Acc:N	0.53981
ENSMUSG00000066894	Vsig10	V-set and immunoglobulin domain containing 10 [S	0.53987
ENSMUSG00000078903	Gm14391	predicted gene 14391 [Source:MGI Symbol;Acc:MG	0.53987
ENSMUSG00000015697	Setdb1	SET domain, bifurcated 1 [Source:MGI Symbol;Acc:	0.53998
ENSMUSG00000086480	Gm15287	predicted gene 15287 [Source:MGI Symbol;Acc:MG	0.54001
ENSMUSG00000079494	Cml5	camello-like 5 [Source:MGI Symbol;Acc:MGI:19162	0.54008
ENSMUSG00000021298	Gpr132	G protein-coupled receptor 132 [Source:MGI Symb	0.5401
ENSMUSG00000032123	Dpagt1	dolichyl-phosphate (UDP-N-acetylglucosamine) ace	0.54032
ENSMUSG00000024349	Tmem173	transmembrane protein 173 [Source:MGI Symbol;A	0.54035
ENSMUSG00000028488	Sh3gl2	SH3-domain GRB2-like 2 [Source:MGI Symbol;Acc:N	0.54039
ENSMUSG00000024603	Dctn4	dynactin 4 [Source:MGI Symbol;Acc:MGI:1914915]	0.54047
ENSMUSG00000079906	Gm15846	predicted gene 15846 [Source:MGI Symbol;Acc:MG	0.54058
ENSMUSG00000085761	4930455G09Rik	RIKEN cDNA 4930455G09 gene [Source:MGI Symbo	0.54059
ENSMUSG00000082829	Gm15780	predicted gene 15780 [Source:MGI Symbol;Acc:MG	0.54061
ENSMUSG00000036168	Ccdc38	coiled-coil domain containing 38 [Source:MGI Symb	0.54068
ENSMUSG00000082967	Gm16082	predicted gene 16082 [Source:MGI Symbol;Acc:MG	0.54073
ENSMUSG00000009076	Zmat5	zinc finger, matrin type 5 [Source:MGI Symbol;Acc:	0.54075
ENSMUSG00000032093	Cd3e	CD3 antigen, epsilon polypeptide [Source:MGI Sym	0.54075
ENSMUSG00000074274	D930028M14Rik	RIKEN cDNA D930028M14 gene [Source:MGI Symb	0.5408
ENSMUSG00000008206	Cers4	ceramide synthase 4 [Source:MGI Symbol;Acc:MGI:	0.54094

ENSMUSG00000059327	Eda	ectodysplasin-A [Source:MGI Symbol;Acc:MGI:1195	0.54095
ENSMUSG00000052658	5830454E08Rik	RIKEN cDNA 5830454E08 gene [Source:MGI Symbo	0.541
ENSMUSG00000097010	Gm26709	predicted gene, 26709 [Source:MGI Symbol;Acc:M	0.54102
ENSMUSG00000022146	Osmr	oncostatin M receptor [Source:MGI Symbol;Acc:M	0.54114
ENSMUSG00000038909	Kat7	K(lysine) acetyltransferase 7 [Source:MGI Symbol;A	0.54114
ENSMUSG00000022095	Fam160b2	family with sequence similarity 160, member B2 [S	0.54127
ENSMUSG00000037001	Zfp39	zinc finger protein 39 [Source:MGI Symbol;Acc:MGI	0.54132
ENSMUSG00000081984	Dnajb3	DnaJ (Hsp40) homolog, subfamily B, member 3 [Sou	0.54148
ENSMUSG00000086929	Gm11788	predicted gene 11788 [Source:MGI Symbol;Acc:MG	0.54151
ENSMUSG00000097416	Gm26670	predicted gene, 26670 [Source:MGI Symbol;Acc:M	0.54151
ENSMUSG00000026791	Slc2a8	solute carrier family 2, (facilitated glucose transpor	0.54157
ENSMUSG00000033009	Ogfod1	2-oxoglutarate and iron-dependent oxygenase dom	0.54179
ENSMUSG00000005677	Nr1i3	nuclear receptor subfamily 1, group I, member 3 [S	0.54199
ENSMUSG00000089829	Gm16565	predicted gene 16565 [Source:MGI Symbol;Acc:MG	0.5421
ENSMUSG00000021518	Ptdss1	phosphatidylserine synthase 1 [Source:MGI Symbol	0.54231
ENSMUSG00000054779	Gm12541	predicted gene 12541 [Source:MGI Symbol;Acc:MG	0.54233
ENSMUSG00000072980	Oip5	Opa interacting protein 5 [Source:MGI Symbol;Acc:	0.54237
ENSMUSG00000054488	Gm9946	predicted gene 9946 [Source:MGI Symbol;Acc:MGI	0.54241
ENSMUSG00000021461	Fancc	Fanconi anemia, complementation group C [Source	0.54242
ENSMUSG00000068227	Il2rb	interleukin 2 receptor, beta chain [Source:MGI Sym	0.54243
ENSMUSG00000031516	Dctn6	dynactin 6 [Source:MGI Symbol;Acc:MGI:1343154]	0.5427
ENSMUSG00000015748	Prpf3	PRP3 pre-mRNA processing factor 3 homolog (yeas	0.54271
ENSMUSG00000040511	Pvr	poliovirus receptor [Source:MGI Symbol;Acc:MGI:1	0.54272
ENSMUSG00000026608	Kctd3	potassium channel tetramerisation domain contain	0.54273
ENSMUSG00000056679	Gpr173	G-protein coupled receptor 173 [Source:MGI Symb	0.54278
ENSMUSG00000087575	Gm12976	predicted gene 12976 [Source:MGI Symbol;Acc:MG	0.54288
ENSMUSG00000008305	Tle1	transducin-like enhancer of split 1, homolog of Dro	0.5429
ENSMUSG00000023216	Epb4.2	erythrocyte protein band 4.2 [Source:MGI Symbol;	0.5429
ENSMUSG00000027500	Stmn2	stathmin-like 2 [Source:MGI Symbol;Acc:MGI:9824	0.54294
ENSMUSG00000054939	Zfp174	zinc finger protein 174 [Source:MGI Symbol;Acc:M	0.54294
ENSMUSG00000015749	Anp32e	acidic (leucine-rich) nuclear phosphoprotein 32 fam	0.54313
ENSMUSG00000030513	Pcsk6	proprotein convertase subtilisin/kexin type 6 [Sour	0.54315
ENSMUSG00000050394	Armcx6	armadillo repeat containing, X-linked 6 [Source:MG	0.54316
ENSMUSG00000003531	Dgcr6	DiGeorge syndrome critical region gene 6 [Source:M	0.54321
ENSMUSG00000027611	Procr	protein C receptor, endothelial [Source:MGI Symbo	0.54322
ENSMUSG00000062981	Mrpl42	mitochondrial ribosomal protein L42 [Source:MGI S	0.54323
ENSMUSG00000086921	Gm13189	predicted gene 13189 [Source:MGI Symbol;Acc:MG	0.54329
ENSMUSG00000040219	Ttc12	tetratricopeptide repeat domain 12 [Source:MGI Sy	0.5433
ENSMUSG00000091457	Gm17171	predicted gene 17171 [Source:MGI Symbol;Acc:MG	0.5433
ENSMUSG00000086286	Gm15138	predicted gene 15138 [Source:MGI Symbol;Acc:MG	0.54333
ENSMUSG00000092230	Gm18889	predicted gene, 18889 [Source:MGI Symbol;Acc:M	0.54334
ENSMUSG00000024391	Apom	apolipoprotein M [Source:MGI Symbol;Acc:MGI:19	0.54335
ENSMUSG00000061414	Efcab4b	EF-hand calcium binding domain 4B [Source:MGI Sy	0.54335
ENSMUSG00000065444	Mir27a	microRNA 27a [Source:MGI Symbol;Acc:MGI:26769	0.54335
ENSMUSG00000086675	9430029A11Rik	RIKEN cDNA 9430029A11 gene [Source:MGI Symbo	0.54338
ENSMUSG00000060068	Gm12222	predicted gene 12222 [Source:MGI Symbol;Acc:MG	0.5434
ENSMUSG00000064918	Gm22571	predicted gene, 22571 [Source:MGI Symbol;Acc:M	0.54341

ENSMUSG00000032191	Bcl2l10	Bcl2-like 10 [Source:MGI Symbol;Acc:MGI:1330841	0.54352
ENSMUSG00000011589	Fsd1	fibronectin type 3 and SPRY domain-containing pro	0.54356
ENSMUSG00000090105	Gm15890	predicted gene 15890 [Source:MGI Symbol;Acc:MG	0.54366
ENSMUSG00000020538	Srebf1	sterol regulatory element binding transcription fact	0.54368
ENSMUSG00000023249	Parp3	poly (ADP-ribose) polymerase family, member 3 [So	0.5438
ENSMUSG00000029468	P2rx7	purinergic receptor P2X, ligand-gated ion channel,	0.54401
ENSMUSG00000003808	Farsa	phenylalanyl-tRNA synthetase, alpha subunit [Sourc	0.54407
ENSMUSG00000043441	Gpr149	G protein-coupled receptor 149 [Source:MGI Symb	0.54408
ENSMUSG00000038302	Lace1	lactation elevated 1 [Source:MGI Symbol;Acc:MGI::	0.54426
ENSMUSG00000041959	S100a10	S100 calcium binding protein A10 (calpactin) [Sourc	0.54427
ENSMUSG00000079024	Gm21961	predicted gene, 21961 [Source:MGI Symbol;Acc:MG	0.54428
ENSMUSG00000083469	Gm14281	predicted gene 14281 [Source:MGI Symbol;Acc:MG	0.54433
ENSMUSG00000097723	Gm26848	predicted gene, 26848 [Source:MGI Symbol;Acc:MG	0.5444
ENSMUSG00000083610	Oaz2-ps	ornithine decarboxylase antizyme 2, pseudogene [S	0.54475
ENSMUSG00000028294	1700003M02Rik	RIKEN cDNA 1700003M02 gene [Source:MGI Symbo	0.54488
ENSMUSG00000020733	Slc9a3r1	solute carrier family 9 (sodium/hydrogen exchange	0.54495
ENSMUSG00000027075	Slc43a1	solute carrier family 43, member 1 [Source:MGI Sy	0.54497
ENSMUSG00000016028	Celsr1	cadherin, EGF LAG seven-pass G-type receptor 1 (fl	0.54505
ENSMUSG00000070643	Sox13	SRY-box containing gene 13 [Source:MGI Symbol;A	0.54507
ENSMUSG00000032131	Abcg4	ATP-binding cassette, sub-family G (WHITE), memb	0.54516
ENSMUSG00000045775	Slc16a5	solute carrier family 16 (monocarboxylic acid transp	0.54518
ENSMUSG00000035896	Rnase1	ribonuclease, RNase A family, 1 (pancreatic) [Sourc	0.54523
ENSMUSG00000036932	Aifm1	apoptosis-inducing factor, mitochondrion-associate	0.54527
ENSMUSG00000028664	Ephb2	Eph receptor B2 [Source:MGI Symbol;Acc:MGI:9961	0.54528
ENSMUSG00000000532	Acvr1b	activin A receptor, type 1B [Source:MGI Symbol;Ac	0.54549
ENSMUSG00000037652	Phc3	polyhomeotic-like 3 (Drosophila) [Source:MGI Symb	0.54549
ENSMUSG00000006931	Leprel4	leprecan-like 4 [Source:MGI Symbol;Acc:MGI:19134	0.54564
ENSMUSG00000086710	Gm13465	predicted gene 13465 [Source:MGI Symbol;Acc:MG	0.54577
ENSMUSG00000035085	1700020L24Rik	RIKEN cDNA 1700020L24 gene [Source:MGI Symbo	0.54585
ENSMUSG00000086405	9330198N18Rik	RIKEN cDNA 9330198N18 gene [Source:MGI Symbo	0.54585
ENSMUSG00000039252	Lgi2	leucine-rich repeat LGI family, member 2 [Source:M	0.54587
ENSMUSG00000030521	Mphosph10	M-phase phosphoprotein 10 (U3 small nucleolar rib	0.54596
ENSMUSG00000011382	Dhdh	dihydrodiol dehydrogenase (dimeric) [Source:MGI S	0.54602
ENSMUSG00000050379	6-Sep	septin 6 [Source:MGI Symbol;Acc:MGI:1888939]	0.54604
ENSMUSG00000008429	Herpud2	HERPUD family member 2 [Source:MGI Symbol;Acc	0.54606
ENSMUSG00000074384	AI429214	expressed sequence AI429214 [Source:MGI Symbol	0.5461
ENSMUSG00000042155	Klhl23	kelch-like 23 [Source:MGI Symbol;Acc:MGI:268353	0.54615
ENSMUSG00000005994	Tyrp1	tyrosinase-related protein 1 [Source:MGI Symbol;A	0.54638
ENSMUSG00000025026	Add3	adducin 3 (gamma) [Source:MGI Symbol;Acc:MGI:1	0.54638
ENSMUSG00000021587	Pcsk1	proprotein convertase subtilisin/kexin type 1 [Sourc	0.54639
ENSMUSG00000021671	Poc5	POC5 centriolar protein homolog (Chlamydomonas	0.54652
ENSMUSG00000022357	Klhl38	kelch-like 38 [Source:MGI Symbol;Acc:MGI:304531	0.54652
ENSMUSG00000020648	Dus4l	dihydrouridine synthase 4-like (S. cerevisiae) [Sourc	0.54653
ENSMUSG00000005667	Mthfd2	methylenetetrahydrofolate dehydrogenase (NAD+	0.54654
ENSMUSG00000030409	Dmpk	dystrophia myotonica-protein kinase [Source:MGI S	0.54654
ENSMUSG00000030980	Knop1	lysine rich nucleolar protein 1 [Source:MGI Symbol;	0.54655
ENSMUSG00000026547	Tagln2	transgelin 2 [Source:MGI Symbol;Acc:MGI:1312985	0.54673

ENSMUSG00000044092	C130050O18Rik	RIKEN cDNA C130050O18 gene [Source:MGI Symbo	0.5469
ENSMUSG00000031485	Prosc	proline synthetase co-transcribed [Source:MGI Sym	0.54691
ENSMUSG00000026829	Gbgt1	globoside alpha-1,3-N-acetylgalactosaminyltransfe	0.54702
ENSMUSG00000005481	Ddx39	DEAD (Asp-Glu-Ala-Asp) box polypeptide 39 [Sourc	0.54704
ENSMUSG00000049897	Gm711	predicted gene 711 [Source:MGI Symbol;Acc:MGI:2	0.54726
ENSMUSG00000047963	Stbd1	starch binding domain 1 [Source:MGI Symbol;Acc:M	0.54736
ENSMUSG00000027954	Efna1	ephrin A1 [Source:MGI Symbol;Acc:MGI:103236]	0.54768
ENSMUSG00000031078	Cttn	cortactin [Source:MGI Symbol;Acc:MGI:99695]	0.54768
ENSMUSG00000035109	Shc4	SHC (Src homology 2 domain containing) family, me	0.54776
ENSMUSG00000045594	Glb1	galactosidase, beta 1 [Source:MGI Symbol;Acc:MGI	0.54781
ENSMUSG00000034614	Pik3ip1	phosphoinositide-3-kinase interacting protein 1 [So	0.54792
ENSMUSG00000097613	Gm17597	predicted gene, 17597 [Source:MGI Symbol;Acc:MG	0.54804
ENSMUSG00000039117	Taf4a	TAF4A RNA polymerase II, TATA box binding protei	0.54808
ENSMUSG00000030255	Sspn	sarcospan [Source:MGI Symbol;Acc:MGI:1353511]	0.54815
ENSMUSG00000032086	Bace1	beta-site APP cleaving enzyme 1 [Source:MGI Symb	0.54818
ENSMUSG00000034024	Cct2	chaperonin containing Tcp1, subunit 2 (beta) [Sour	0.54819
ENSMUSG00000096959	4930509G22Rik	RIKEN cDNA 4930509G22 gene [Source:MGI Symbo	0.54833
ENSMUSG00000025352	Gdf11	growth differentiation factor 11 [Source:MGI Symb	0.54843
ENSMUSG00000063522	2010109I03Rik	RIKEN cDNA 2010109I03 gene [Source:MGI Symbol	0.5485
ENSMUSG00000091378	Gm4219	predicted gene 4219 [Source:MGI Symbol;Acc:MGI:	0.5485
ENSMUSG00000017802	Fam134c	family with sequence similarity 134, member C [Sou	0.54853
ENSMUSG00000028351	Brinp1	bone morphogenic protein/retinoic acid inducible n	0.54866
ENSMUSG00000063757	4632433K11Rik	RIKEN cDNA 4632433K11 gene [Source:MGI Symbo	0.5487
ENSMUSG00000090266	Mettl23	methyltransferase like 23 [Source:MGI Symbol;Acc:	0.54871
ENSMUSG00000040138	Ndp	Norrie disease (pseudoglioma) (human) [Source:MG	0.54881
ENSMUSG00000098202	B830012L14Rik	RIKEN cDNA B830012L14 gene [Source:MGI Symbo	0.54882
ENSMUSG00000048118	Arid4a	AT rich interactive domain 4A (RBP1-like) [Source:M	0.54895
ENSMUSG00000080021	Gm5915	predicted pseudogene 5915 [Source:MGI Symbol;A	0.54898
ENSMUSG00000063450	Syne2	spectrin repeat containing, nuclear envelope 2 [Sou	0.54916
ENSMUSG00000093424	6330562C20Rik	RIKEN cDNA 6330562C20 gene [Source:MGI Symbo	0.54917
ENSMUSG00000074472	Zfp872	zinc finger protein 872 [Source:MGI Symbol;Acc:MG	0.54918
ENSMUSG00000061455	Stx17	syntaxin 17 [Source:MGI Symbol;Acc:MGI:1914977]	0.5492
ENSMUSG00000046634	Pkd1l1	polycystic kidney disease 1 like 1 [Source:MGI Symb	0.54921
ENSMUSG00000084941	Gm11944	predicted gene 11944 [Source:MGI Symbol;Acc:MG	0.54931
ENSMUSG00000045319	Proser2	proline and serine rich 2 [Source:MGI Symbol;Acc:M	0.54933
ENSMUSG00000034164	Emid1	EMI domain containing 1 [Source:MGI Symbol;Acc:	0.54935
ENSMUSG00000055560	Zfp459	zinc finger protein 459 [Source:MGI Symbol;Acc:MG	0.54939
ENSMUSG00000073414	AU023871	expressed sequence AU023871 [Source:MGI Symbo	0.54941
ENSMUSG00000027379	Bub1	budding uninhibited by benzimidazoles 1 homolog	0.54943
ENSMUSG00000026587	Astn1	astrotactin 1 [Source:MGI Symbol;Acc:MGI:109856	0.5495
ENSMUSG00000028199	Cryz	crystallin, zeta [Source:MGI Symbol;Acc:MGI:88527	0.54958
ENSMUSG00000023192	Grm2	glutamate receptor, metabotropic 2 [Source:MGI Sy	0.54961
ENSMUSG00000078193	Gm2000	predicted gene 2000 [Source:MGI Symbol;Acc:MGI:	0.54967
ENSMUSG00000090516	Rps11-ps1	ribosomal protein S11, pseudogene 1 [Source:MGI	0.54969
ENSMUSG00000039983	Ccdc32	coiled-coil domain containing 32 [Source:MGI Symb	0.54975
ENSMUSG00000029550	Sppl3	signal peptide peptidase 3 [Source:MGI Symbol;Acc	0.54983
ENSMUSG00000052273	Dnah3	dynein, axonemal, heavy chain 3 [Source:MGI Symb	0.55012

ENSMUSG00000059571	Gm16218	predicted gene 16218 [Source:MGI Symbol;Acc:MG	0.55021
ENSMUSG00000097369	Gm26545	predicted gene, 26545 [Source:MGI Symbol;Acc:M(	0.55021
ENSMUSG00000079502	1700101E01Rik	RIKEN cDNA 1700101E01 gene [Source:MGI Symbo	0.55023
ENSMUSG00000063820	Arl9	ADP-ribosylation factor-like 9 [Source:MGI Symbol;	0.55024
ENSMUSG00000003070	Efna2	ephrin A2 [Source:MGI Symbol;Acc:MGI:102707]	0.55031
ENSMUSG00000095555	Gm26266	predicted gene, 26266 [Source:MGI Symbol;Acc:M(	0.55037
ENSMUSG00000027508	Pag1	phosphoprotein associated with glycosphingolipid r	0.55047
ENSMUSG00000081805	Gm14335	predicted gene 14335 [Source:MGI Symbol;Acc:MG	0.55076
ENSMUSG00000030888	Rrp8	ribosomal RNA processing 8, methyltransferase, ho	0.55077
ENSMUSG00000014303	Glis2	GLIS family zinc finger 2 [Source:MGI Symbol;Acc:M	0.55087
ENSMUSG00000000149	Gna12	guanine nucleotide binding protein, alpha 12 [Sour	0.55097
ENSMUSG00000028104	Polr3gl	polymerase (RNA) III (DNA directed) polypeptide G	0.55102
ENSMUSG00000046337	Fam178b	family with sequence similarity 178, member B [Sou	0.55104
ENSMUSG00000019794	Katna1	katanin p60 (ATPase-containing) subunit A1 [Source	0.55116
ENSMUSG00000058709	Egln2	EGL nine homolog 2 (C. elegans) [Source:MGI Symb	0.55127
ENSMUSG00000097487	Ptges3l	prostaglandin E synthase 3 (cytosolic)-like [Source:I	0.55127
ENSMUSG00000033166	Dis3	DIS3 mitotic control homolog (S. cerevisiae) [Source	0.55138
ENSMUSG00000036110	Slc17a2	solute carrier family 17 (sodium phosphate), memb	0.55148
ENSMUSG00000032023	4931429I11Rik	RIKEN cDNA 4931429I11 gene [Source:MGI Symbol	0.5515
ENSMUSG00000002763	Pex6	peroxisomal biogenesis factor 6 [Source:MGI Symb	0.55158
ENSMUSG00000051497	Kcnj16	potassium inwardly-rectifying channel, subfamily J,	0.55158
ENSMUSG00000074603	Gm10729	predicted gene 10729 [Source:MGI Symbol;Acc:MG	0.55168
ENSMUSG00000087072	Recql5os1	RecQ-like 5, opposite strand 1 [Source:MGI Symbol	0.55176
ENSMUSG00000022339	Ebag9	estrogen receptor-binding fragment-associated ger	0.55179
ENSMUSG00000052253	Zfp622	zinc finger protein 622 [Source:MGI Symbol;Acc:M(	0.55181
ENSMUSG00000095123	Gm21781	predicted gene, 21781 [Source:MGI Symbol;Acc:M(	0.55182
ENSMUSG00000025175	Fn3k	fructosamine 3 kinase [Source:MGI Symbol;Acc:MG	0.55183
ENSMUSG00000002797	Ggct	gamma-glutamyl cyclotransferase [Source:MGI Sym	0.55205
ENSMUSG00000068184	Ndufaf2	NADH dehydrogenase (ubiquinone) 1 alpha subcom	0.55221
ENSMUSG00000044043	Pcdhb14	protocadherin beta 14 [Source:MGI Symbol;Acc:M(	0.55233
ENSMUSG00000079426	Arpc4	actin related protein 2/3 complex, subunit 4 [Sourc	0.55233
ENSMUSG00000040410	Fbxl4	F-box and leucine-rich repeat protein 4 [Source:MG	0.55239
ENSMUSG00000043614	Vps37d	vacuolar protein sorting 37D (yeast) [Source:MGI S	0.55245
ENSMUSG00000065259	Snora30	small nucleolar RNA, H/ACA box 30 [Source:MGI Sy	0.55245
ENSMUSG00000026796	Fam129b	family with sequence similarity 129, member B [Sou	0.55269
ENSMUSG00000091745	Gm17098	predicted gene 17098 [Source:MGI Symbol;Acc:MG	0.5527
ENSMUSG00000029518	Rab35	RAB35, member RAS oncogene family [Source:MGI	0.55274
ENSMUSG00000029055	Plch2	phospholipase C, eta 2 [Source:MGI Symbol;Acc:M(	0.55282
ENSMUSG00000039196	Orm1	orosomucoid 1 [Source:MGI Symbol;Acc:MGI:9744:	0.55286
ENSMUSG00000026170	Cyp27a1	cytochrome P450, family 27, subfamily a, polypepti	0.55289
ENSMUSG00000057933	Gsta2	glutathione S-transferase, alpha 2 (Yc2) [Source:M(	0.55306
ENSMUSG00000083780	Gm12728	predicted gene 12728 [Source:MGI Symbol;Acc:MG	0.55306
ENSMUSG00000031595	Pdgfrl	platelet-derived growth factor receptor-like [Source	0.55307
ENSMUSG00000092571	Gm4134	predicted gene 4134 [Source:MGI Symbol;Acc:MGI:	0.55307
ENSMUSG00000083613	Pou2f3-rs1	POU domain, class 2, transcription factor 3, related	0.55309
ENSMUSG00000075701	Vimp	VCP-interacting membrane protein [Source:MGI Sy	0.55315
ENSMUSG00000066175	2510046G10Rik	RIKEN cDNA 2510046G10 gene [Source:MGI Symbo	0.55326

ENSMUSG00000032078	Zfp259	zinc finger protein 259 [Source:MGI Symbol;Acc:MG	0.5533
ENSMUSG00000042678	Myo15	myosin XV [Source:MGI Symbol;Acc:MGI:1261811]	0.55338
ENSMUSG00000080316	4930546H06Rik	RIKEN cDNA 4930546H06 gene [Source:MGI Symbo	0.55342
ENSMUSG00000027942	4933434E20Rik	RIKEN cDNA 4933434E20 gene [Source:MGI Symbo	0.55346
ENSMUSG00000040489	Sox30	SRY-box containing gene 30 [Source:MGI Symbol;A	0.55348
ENSMUSG00000028080	Lrba	LPS-responsive beige-like anchor [Source:MGI Sym	0.55349
ENSMUSG00000032458	Copb2	coatomer protein complex, subunit beta 2 (beta pri	0.55356
ENSMUSG00000051319	1500011K16Rik	RIKEN cDNA 1500011K16 gene [Source:MGI Symbo	0.55376
ENSMUSG00000024169	Ift140	intraflagellar transport 140 [Source:MGI Symbol;Ac	0.55388
ENSMUSG00000063672	Nkx6-3	NK6 homeobox 3 [Source:MGI Symbol;Acc:MGI:192	0.5539
ENSMUSG00000022197	Pdzd2	PDZ domain containing 2 [Source:MGI Symbol;Acc:	0.55422
ENSMUSG00000001105	Ift20	intraflagellar transport 20 [Source:MGI Symbol;Acc	0.55423
ENSMUSG00000090086	AI480526	expressed sequence AI480526 [Source:MGI Symbol	0.55434
ENSMUSG00000036699	Zcchc12	zinc finger, CCHC domain containing 12 [Source:MG	0.55445
ENSMUSG00000086573	Gm15384	predicted gene 15384 [Source:MGI Symbol;Acc:MG	0.5545
ENSMUSG00000073016	Uprt	uracil phosphoribosyltransferase (FUR1) homolog (	0.55455
ENSMUSG00000029480	Dhx37	DEAH (Asp-Glu-Ala-His) box polypeptide 37 [Source	0.55459
ENSMUSG00000084939	Gm830	predicted gene 830 [Source:MGI Symbol;Acc:MGI:2	0.55462
ENSMUSG00000018882	Mrpl45	mitochondrial ribosomal protein L45 [Source:MGI S	0.55463
ENSMUSG00000090429	Gm17271	predicted gene, 17271 [Source:MGI Symbol;Acc:MG	0.5548
ENSMUSG00000030317	Timp4	tissue inhibitor of metalloproteinase 4 [Source:MGI	0.55485
ENSMUSG00000053687	Dpep2	dipeptidase 2 [Source:MGI Symbol;Acc:MGI:244204	0.55488
ENSMUSG00000062284	Gm6030	predicted gene 6030 [Source:MGI Symbol;Acc:MGI:	0.55489
ENSMUSG00000088357	Gm24011	predicted gene, 24011 [Source:MGI Symbol;Acc:MG	0.55492
ENSMUSG00000036083	Slc17a3	solute carrier family 17 (sodium phosphate), memb	0.55494
ENSMUSG00000034345	Gtf2h5	general transcription factor IIH, polypeptide 5 [Sou	0.55496
ENSMUSG00000085105	Gm12758	predicted gene 12758 [Source:MGI Symbol;Acc:MG	0.55511
ENSMUSG00000036167	Pphln1	periphilin 1 [Source:MGI Symbol;Acc:MGI:1917029]	0.55515
ENSMUSG00000030711	Sult1a1	sulfotransferase family 1A, phenol-preferring, mem	0.55524
ENSMUSG00000020705	Ddx42	DEAD (Asp-Glu-Ala-Asp) box polypeptide 42 [Source	0.55529
ENSMUSG00000025610	Map3k7cl	Map3k7 C-terminal like [Source:MGI Symbol;Acc:M	0.5554
ENSMUSG00000086920	Gm12207	predicted gene 12207 [Source:MGI Symbol;Acc:MG	0.55552
ENSMUSG00000073514	Dok6	docking protein 6 [Source:MGI Symbol;Acc:MGI:363	0.55554
ENSMUSG00000028089	Chd1l	chromodomain helicase DNA binding protein 1-like	0.55566
ENSMUSG00000057215	Gm15514	predicted gene 15514 [Source:MGI Symbol;Acc:MG	0.55566
ENSMUSG00000031723	Txnl4b	thioredoxin-like 4B [Source:MGI Symbol;Acc:MGI:2	0.55577
ENSMUSG00000013973	Dedd	death effector domain-containing [Source:MGI Sym	0.55592
ENSMUSG00000038954	Supt3	suppressor of Ty 3 [Source:MGI Symbol;Acc:MGI:19	0.55592
ENSMUSG00000045665	Mfsd5	major facilitator superfamily domain containing 5 [S	0.55592
ENSMUSG00000065564	Mirlet7b	microRNA let7b [Source:MGI Symbol;Acc:MGI:2676	0.55593
ENSMUSG00000039611	Tmem246	transmembrane protein 246 [Source:MGI Symbol;A	0.55608
ENSMUSG00000090067	Gm2976	predicted gene 2976 [Source:MGI Symbol;Acc:MGI:	0.55622
ENSMUSG00000082806	Rpl13-ps1	ribosomal protein L13, pseudogene 1 [Source:MGI	0.55624
ENSMUSG00000064147	Rab44	RAB44, member RAS oncogene family [Source:MGI	0.55638
ENSMUSG00000047040	Prr15l	proline rich 15-like [Source:MGI Symbol;Acc:MGI:2	0.55641
ENSMUSG00000004947	Dtx2	deltex 2 homolog (Drosophila) [Source:MGI Symbol	0.55644
ENSMUSG00000014907	Naf1	nuclear assembly factor 1 homolog (S. cerevisiae) [S	0.55645

ENSMUSG00000047696	Ccdc144b	coiled-coil domain containing 144B [Source:MGI Sy	0.55645
ENSMUSG00000002043	Trappc6a	trafficking protein particle complex 6A [Source:MG	0.55646
ENSMUSG00000034731	Dgkh	diacylglycerol kinase, eta [Source:MGI Symbol;Acc:	0.55647
ENSMUSG00000038126	Mphosph9	M-phase phosphoprotein 9 [Source:MGI Symbol;Ac	0.55658
ENSMUSG00000086272	BC039966	cDNA sequence BC039966 [Source:MGI Symbol;Acc	0.55659
ENSMUSG00000027340	Slc23a2	solute carrier family 23 (nucleobase transporters),	0.55672
ENSMUSG00000029360	Gm9754	predicted gene 9754 [Source:MGI Symbol;Acc:MGI:	0.55672
ENSMUSG00000085295	4930430E12Rik	RIKEN cDNA 4930430E12 gene [Source:MGI Symbo	0.55679
ENSMUSG00000028885	Smpdl3b	sphingomyelin phosphodiesterase, acid-like 3B [Sou	0.55683
ENSMUSG00000089861	Gm16139	predicted gene 16139 [Source:MGI Symbol;Acc:MG	0.55694
ENSMUSG00000026511	Srp9	signal recognition particle 9 [Source:MGI Symbol;A	0.55695
ENSMUSG00000019087	Atp6ap1	ATPase, H+ transporting, lysosomal accessory prote	0.55702
ENSMUSG00000039521	Foxp3	forkhead box P3 [Source:MGI Symbol;Acc:MGI:189	0.55703
ENSMUSG00000015709	Arnt2	aryl hydrocarbon receptor nuclear translocator 2 [S	0.55717
ENSMUSG00000027947	Il6ra	interleukin 6 receptor, alpha [Source:MGI Symbol;A	0.55731
ENSMUSG00000029075	Tnfrsf4	tumor necrosis factor receptor superfamily, membe	0.55741
ENSMUSG00000024899	Papss2	3'-phosphoadenosine 5'-phosphosulfate synthase 2	0.55748
ENSMUSG00000051506	Wdfy4	WD repeat and FYVE domain containing 4 [Source:I	0.55758
ENSMUSG00000013822	Elof1	elongation factor 1 homolog (ELF1, S. cerevisiae) [S	0.55785
ENSMUSG00000020623	Map2k6	mitogen-activated protein kinase kinase 6 [Source:	0.55785
ENSMUSG00000029561	Oasl2	2'-5' oligoadenylate synthetase-like 2 [Source:MGI	0.55787
ENSMUSG00000034205	Loxl2	lysyl oxidase-like 2 [Source:MGI Symbol;Acc:MGI:2	0.55788
ENSMUSG00000083281	Gm13332	predicted gene 13332 [Source:MGI Symbol;Acc:MG	0.55796
ENSMUSG00000092597	Gm20486	predicted gene 20486 [Source:MGI Symbol;Acc:MG	0.55812
ENSMUSG00000061751	Kalrn	kalirin, RhoGEF kinase [Source:MGI Symbol;Acc:MG	0.55816
ENSMUSG00000025202	Scd3	stearoyl-coenzyme A desaturase 3 [Source:MGI Syr	0.5584
ENSMUSG00000024593	Megf10	multiple EGF-like-domains 10 [Source:MGI Symbol;	0.55854
ENSMUSG00000091041	Gm17720	predicted gene, 17720 [Source:MGI Symbol;Acc:M	0.55855
ENSMUSG00000038524	Fchsd1	FCH and double SH3 domains 1 [Source:MGI Symbo	0.55856
ENSMUSG00000080834	Gm15216	predicted gene 15216 [Source:MGI Symbol;Acc:MG	0.55864
ENSMUSG00000031825	Crispld2	cysteine-rich secretory protein LCCL domain contai	0.55868
ENSMUSG00000033033	Calhm2	calcium homeostasis modulator 2 [Source:MGI Sym	0.55871
ENSMUSG00000001036	Epn2	epsin 2 [Source:MGI Symbol;Acc:MGI:1333766]	0.55877
ENSMUSG00000061510	Gm10101	predicted gene 10101 [Source:MGI Symbol;Acc:MG	0.55884
ENSMUSG00000086873	Gm15672	predicted gene 15672 [Source:MGI Symbol;Acc:MG	0.55897
ENSMUSG00000037134	Prmt10	protein arginine methyltransferase 10 (putative) [S	0.55906
ENSMUSG00000094191	Rpl31-ps14	ribosomal protein L31, pseudogene 14 [Source:MG	0.55907
ENSMUSG00000007603	Dus3l	dihydrouridine synthase 3-like (S. cerevisiae) [Sourc	0.55911
ENSMUSG00000020514	Mrpl22	mitochondrial ribosomal protein L22 [Source:MGI S	0.55911
ENSMUSG00000040146	Rgl3	ral guanine nucleotide dissociation stimulator-like 3	0.55928
ENSMUSG00000087128	Gm12655	predicted gene 12655 [Source:MGI Symbol;Acc:MG	0.55928
ENSMUSG00000079444	Gm21981	predicted gene 21981 [Source:MGI Symbol;Acc:MG	0.55946
ENSMUSG00000021932	Rnaseh2b	ribonuclease H2, subunit B [Source:MGI Symbol;Ac	0.55949
ENSMUSG00000033257	Ttll4	tubulin tyrosine ligase-like family, member 4 [Sourc	0.55953
ENSMUSG00000039405	Prss23	protease, serine, 23 [Source:MGI Symbol;Acc:MGI:	0.55954
ENSMUSG00000084947	Gm15594	predicted gene 15594 [Source:MGI Symbol;Acc:MG	0.55955
ENSMUSG00000095395	Gm25617	predicted gene, 25617 [Source:MGI Symbol;Acc:M	0.55961

ENSMUSG00000056032	BC018473	cDNA sequence BC018473 [Source:MGI Symbol;Acc	0.55962
ENSMUSG00000003199	Mpnd	MPN domain containing [Source:MGI Symbol;Acc:M	0.55965
ENSMUSG00000047654	Tssk6	testis-specific serine kinase 6 [Source:MGI Symbol;A	0.55965
ENSMUSG00000038780	Smurf1	SMAD specific E3 ubiquitin protein ligase 1 [Source	0.55971
ENSMUSG00000082349	Gm15064	predicted gene 15064 [Source:MGI Symbol;Acc:MG	0.55974
ENSMUSG00000036941	Elac1	elaC homolog 1 (E. coli) [Source:MGI Symbol;Acc:M	0.5598
ENSMUSG00000058248	Kcnh1	potassium voltage-gated channel, subfamily H (eag	0.55982
ENSMUSG00000015488	Cacfd1	calcium channel flower domain containing 1 [Sourc	0.55983
ENSMUSG00000031808	Slc27a1	solute carrier family 27 (fatty acid transporter), me	0.55988
ENSMUSG00000071237	Gm6253	predicted pseudogene 6253 [Source:MGI Symbol;A	0.56006
ENSMUSG00000086205	Gm12679	predicted gene 12679 [Source:MGI Symbol;Acc:MG	0.56006
ENSMUSG00000071335	G630090E17Rik	RIKEN cDNA G630090E17 gene [Source:MGI Symbo	0.5601
ENSMUSG00000058905	Gm10051	predicted pseudogene 10051 [Source:MGI Symbol;	0.56011
ENSMUSG00000070524	Fcrlb	Fc receptor-like B [Source:MGI Symbol;Acc:MGI:35	0.56011
ENSMUSG00000024127	Prepl	prolyl endopeptidase-like [Source:MGI Symbol;Acc:	0.56012
ENSMUSG00000070886	Gm10304	predicted gene 10304 [Source:MGI Symbol;Acc:MG	0.56013
ENSMUSG00000091144	Phf11c	PHD finger protein 11C [Source:MGI Symbol;Acc:M	0.5602
ENSMUSG00000029994	Anxa4	annexin A4 [Source:MGI Symbol;Acc:MGI:88030]	0.56034
ENSMUSG00000039745	Htatip2	HIV-1 tat interactive protein 2, homolog (human) [S	0.56035
ENSMUSG00000086149	Gm15598	predicted gene 15598 [Source:MGI Symbol;Acc:MG	0.56036
ENSMUSG00000081355	Gm15264	predicted gene 15264 [Source:MGI Symbol;Acc:MG	0.56069
ENSMUSG00000021254	Gpatch2l	G patch domain containing 2 like [Source:MGI Syml	0.56076
ENSMUSG00000072692	Gm13826	predicted gene 13826 [Source:MGI Symbol;Acc:MG	0.5608
ENSMUSG00000046204	Pnma2	paraneoplastic antigen MA2 [Source:MGI Symbol;A	0.56099
ENSMUSG00000078923	Ube2v1	ubiquitin-conjugating enzyme E2 variant 1 [Source:	0.56099
ENSMUSG00000089812	Gm15867	predicted gene 15867 [Source:MGI Symbol;Acc:MG	0.56099
ENSMUSG00000054679	Srsf12	serine/arginine-rich splicing factor 12 [Source:MGI	0.56114
ENSMUSG00000097810	G730046D07Rik	RIKEN cDNA G730046D07 gene [Source:MGI Symbo	0.56125
ENSMUSG00000027490	E2f1	E2F transcription factor 1 [Source:MGI Symbol;Acc:	0.56128
ENSMUSG00000024972	Lgals12	lectin, galactose binding, soluble 12 [Source:MGI Sy	0.56129
ENSMUSG00000021339	Mrs2	MRS2 magnesium homeostasis factor homolog (S. c	0.56132
ENSMUSG00000004631	Sgce	sarcoglycan, epsilon [Source:MGI Symbol;Acc:MGI:	0.56135
ENSMUSG00000074194	Zfp791	zinc finger protein 791 [Source:MGI Symbol;Acc:MG	0.56151
ENSMUSG00000004798	Ulk2	unc-51 like kinase 2 [Source:MGI Symbol;Acc:MGI:1	0.56152
ENSMUSG00000040213	Ccbl2	cysteine conjugate-beta lyase 2 [Source:MGI Symbo	0.56156
ENSMUSG00000085595	Gm16090	predicted gene 16090 [Source:MGI Symbol;Acc:MG	0.56159
ENSMUSG00000026926	Pmpca	peptidase (mitochondrial processing) alpha [Source	0.56161
ENSMUSG00000026197	Zfand2b	zinc finger, AN1 type domain 2B [Source:MGI Symb	0.56163
ENSMUSG00000027360	Hdc	histidine decarboxylase [Source:MGI Symbol;Acc:M	0.56163
ENSMUSG00000038619	Ensa	endosulfine alpha [Source:MGI Symbol;Acc:MGI:18	0.56174
ENSMUSG00000029910	Mad2l1	MAD2 mitotic arrest deficient-like 1 [Source:MGI Sy	0.56179
ENSMUSG00000085214	0610005C13Rik	RIKEN cDNA 0610005C13 gene [Source:MGI Symbo	0.56182
ENSMUSG00000085702	Gm16722	predicted gene, 16722 [Source:MGI Symbol;Acc:MG	0.56184
ENSMUSG00000043639	Rbm20	RNA binding motif protein 20 [Source:MGI Symbol;	0.56207
ENSMUSG00000024436	Mrps18b	mitochondrial ribosomal protein S18B [Source:MGI	0.56209
ENSMUSG00000001741	Il16	interleukin 16 [Source:MGI Symbol;Acc:MGI:12708	0.56217
ENSMUSG00000020904	Wdr16	WD repeat domain 16 [Source:MGI Symbol;Acc:MG	0.56217

ENSMUSG00000015962	1700016C15Rik	RIKEN cDNA 1700016C15 gene [Source:MGI Symbo	0.56219
ENSMUSG00000080783	Gm8250	predicted gene 8250 [Source:MGI Symbol;Acc:MGI:	0.56232
ENSMUSG00000022412	Mief1	mitochondrial elongation factor 1 [Source:MGI Sym	0.56236
ENSMUSG00000043572	Pars2	prolyl-tRNA synthetase (mitochondrial)(putative) [S	0.56246
ENSMUSG00000036816	Atoh7	atonal homolog 7 (Drosophila) [Source:MGI Symbo	0.56254
ENSMUSG00000097693	4930471M09Rik	RIKEN cDNA 4930471M09 gene [Source:MGI Symbo	0.56254
ENSMUSG00000022387	Brd1	bromodomain containing 1 [Source:MGI Symbol;Ac	0.56256
ENSMUSG00000059658	Gm16379	predicted pseudogene 16379 [Source:MGI Symbol;	0.56256
ENSMUSG00000035829	Ppp1r26	protein phosphatase 1, regulatory subunit 26 [Sour	0.56259
ENSMUSG00000097648	9330185C12Rik	RIKEN cDNA 9330185C12 gene [Source:MGI Symbo	0.56261
ENSMUSG00000040969	Arhgef38	Rho guanine nucleotide exchange factor (GEF) 38 [S	0.56264
ENSMUSG00000044674	Fzd1	frizzled homolog 1 (Drosophila) [Source:MGI Symbo	0.56272
ENSMUSG00000052565	Hist1h1d	histone cluster 1, H1d [Source:MGI Symbol;Acc:MG	0.5628
ENSMUSG00000092696	Mir5133	microRNA 5133 [Source:MGI Symbol;Acc:MGI:4950	0.56283
ENSMUSG00000087652	Gm15918	predicted gene 15918 [Source:MGI Symbol;Acc:MG	0.56294
ENSMUSG00000088226	Gm22596	predicted gene, 22596 [Source:MGI Symbol;Acc:M	0.56297
ENSMUSG00000035273	Hpse	heparanase [Source:MGI Symbol;Acc:MGI:1343124	0.563
ENSMUSG00000073779	B230314M03Rik	RIKEN cDNA B230314M03 gene [Source:MGI Symbo	0.56304
ENSMUSG00000022571	Pycrl	pyrroline-5-carboxylate reductase-like [Source:MGI	0.5631
ENSMUSG00000064330	Pde6h	phosphodiesterase 6H, cGMP-specific, cone, gamm	0.56331
ENSMUSG00000089072	Gm24500	predicted gene, 24500 [Source:MGI Symbol;Acc:M	0.56337
ENSMUSG00000077450	Rab11b	RAB11B, member RAS oncogene family [Source:MG	0.56341
ENSMUSG00000001558	Klhl10	kelch-like 10 [Source:MGI Symbol;Acc:MGI:218106	0.5635
ENSMUSG00000053460	Ggcx	gamma-glutamyl carboxylase [Source:MGI Symbol;	0.56354
ENSMUSG00000016181	Diexf	digestive organ expansion factor homolog (zebrafis	0.56368
ENSMUSG00000079480	Pin4	protein (peptidyl-prolyl cis/trans isomerase) NIMA-	0.56368
ENSMUSG00000047710	Champ1	chromosome alignment maintaining phosphoprote	0.56371
ENSMUSG00000073130	Gm1141	predicted gene 1141 [Source:MGI Symbol;Acc:MGI:	0.56374
ENSMUSG00000085681	Gm13982	predicted gene 13982 [Source:MGI Symbol;Acc:MG	0.56378
ENSMUSG00000021360	Gcnt2	glucosaminyl (N-acetyl) transferase 2, I-branching e	0.5638
ENSMUSG00000039018	Mtg1	mitochondrial GTPase 1 homolog (S. cerevisiae) [So	0.56382
ENSMUSG00000022560	Slc52a2	solute carrier protein 52, member 2 [Source:MGI Sy	0.56395
ENSMUSG00000028184	Lphn2	latrophilin 2 [Source:MGI Symbol;Acc:MGI:2139714	0.56409
ENSMUSG00000018412	Kansl1	KAT8 regulatory NSL complex subunit 1 [Source:MG	0.56415
ENSMUSG00000089941	Gm16168	predicted gene 16168 [Source:MGI Symbol;Acc:MG	0.56422
ENSMUSG00000047473	Zfp30	zinc finger protein 30 [Source:MGI Symbol;Acc:MGI	0.56425
ENSMUSG00000029776	Hibadh	3-hydroxyisobutyrate dehydrogenase [Source:MGI	0.56435
ENSMUSG00000056116	H2-T22	histocompatibility 2, T region locus 22 [Source:MGI	0.56435
ENSMUSG00000024165	Hn1l	hematological and neurological expressed 1-like [So	0.56436
ENSMUSG00000048497	Mmgt2	membrane magnesium transporter 2 [Source:MGI S	0.56444
ENSMUSG00000044976	Wdr72	WD repeat domain 72 [Source:MGI Symbol;Acc:MG	0.56445
ENSMUSG00000032852	Rspo4	R-spondin family, member 4 [Source:MGI Symbol;A	0.56456
ENSMUSG00000032733	Snx33	sorting nexin 33 [Source:MGI Symbol;Acc:MGI:2443	0.5647
ENSMUSG00000027478	Dnmt3b	DNA methyltransferase 3B [Source:MGI Symbol;Acc	0.56473
ENSMUSG00000041974	Spidr	scaffolding protein involved i DNA repair [Source:M	0.56477
ENSMUSG00000040840	4930579G18Rik	RIKEN cDNA 4930579G18 gene [Source:MGI Symbo	0.56478
ENSMUSG00000031556	Tm2d2	TM2 domain containing 2 [Source:MGI Symbol;Acc	0.5649

ENSMUSG00000020546	Stxbp4	syntaxin binding protein 4 [Source:MGI Symbol;Acc	0.56492
ENSMUSG00000095930	Nim1	serine/threonine-protein kinase NIM1 [Source:MGI	0.56492
ENSMUSG00000027650	Tti1	TELO2 interacting protein 1 [Source:MGI Symbol;Ac	0.56506
ENSMUSG00000031787	Katnb1	katanin p80 (WD40-containing) subunit B 1 [Source	0.56506
ENSMUSG00000086770	Gm15351	predicted gene 15351 [Source:MGI Symbol;Acc:MG	0.56518
ENSMUSG00000024608	Rps14	ribosomal protein S14 [Source:MGI Symbol;Acc:MG	0.56519
ENSMUSG00000091890	A830073O21Rik	RIKEN cDNA A830073O21 gene [Source:MGI Symbo	0.56524
ENSMUSG00000005102	Eif2ak4	eukaryotic translation initiation factor 2 alpha kinas	0.56532
ENSMUSG00000031840	Rab3a	RAB3A, member RAS oncogene family [Source:MGI	0.56532
ENSMUSG00000041444	Arhgap32	Rho GTPase activating protein 32 [Source:MGI Sym	0.5655
ENSMUSG00000084144	Gm12516	predicted gene 12516 [Source:MGI Symbol;Acc:MG	0.56553
ENSMUSG00000043889	Gm8399	predicted gene 8399 [Source:MGI Symbol;Acc:MGI:	0.56557
ENSMUSG00000088478	Mir1946b	microRNA 1946b [Source:MGI Symbol;Acc:MGI:383	0.56561
ENSMUSG00000029522	Pla2g1b	phospholipase A2, group IB, pancreas [Source:MGI	0.56581
ENSMUSG00000040428	Plekha4	pleckstrin homology domain containing, family A (p	0.56585
ENSMUSG00000030512	Snrpa1	small nuclear ribonucleoprotein polypeptide A' [So	0.56592
ENSMUSG00000008129	4930432K21Rik	RIKEN cDNA 4930432K21 gene [Source:MGI Symbo	0.56597
ENSMUSG00000030403	Vasp	vasodilator-stimulated phosphoprotein [Source:MG	0.56597
ENSMUSG00000053178	Gm9897	predicted gene 9897 [Source:MGI Symbol;Acc:MGI:	0.56608
ENSMUSG00000041859	Mcm3	minichromosome maintenance deficient 3 (S. cerev	0.5662
ENSMUSG00000088632	Gm22314	predicted gene, 22314 [Source:MGI Symbol;Acc:M	0.56624
ENSMUSG00000045790	Ccdc149	coiled-coil domain containing 149 [Source:MGI Sym	0.56631
ENSMUSG00000085286	1700121F15Rik	RIKEN cDNA 1700121F15 gene [Source:MGI Symbo	0.56635
ENSMUSG00000020644	Id2	inhibitor of DNA binding 2 [Source:MGI Symbol;Acc	0.56637
ENSMUSG00000097326	A330048O09Rik	RIKEN cDNA A330048O09 gene [Source:MGI Symbo	0.56646
ENSMUSG00000050957	Insl6	insulin-like 6 [Source:MGI Symbol;Acc:MGI:135159	0.56649
ENSMUSG00000028826	Tmem57	transmembrane protein 57 [Source:MGI Symbol;Ac	0.56654
ENSMUSG00000025701	Alox5	arachidonate 5-lipoxygenase [Source:MGI Symbol;	0.56658
ENSMUSG00000090033	Gm16243	predicted gene 16243 [Source:MGI Symbol;Acc:MG	0.56658
ENSMUSG00000028847	Trappc3	trafficking protein particle complex 3 [Source:MGI	0.56659
ENSMUSG00000085211	B430219N15Rik	RIKEN cDNA B430219N15 gene [Source:MGI Symbo	0.56668
ENSMUSG00000001472	Tcf25	transcription factor 25 (basic helix-loop-helix) [Sour	0.56671
ENSMUSG00000083503	Gm13407	predicted gene 13407 [Source:MGI Symbol;Acc:MG	0.56673
ENSMUSG00000034786	Gpsm3	G-protein signalling modulator 3 (AGS3-like, C. eleg	0.56676
ENSMUSG00000081951	Gm15352	predicted gene 15352 [Source:MGI Symbol;Acc:MG	0.56676
ENSMUSG00000025058	5430427O19Rik	RIKEN cDNA 5430427O19 gene [Source:MGI Symbo	0.56679
ENSMUSG00000044641	Pard6b	par-6 (partitioning defective 6) homolog beta (C. el	0.56682
ENSMUSG00000031734	Irx3	Iroquois related homeobox 3 (Drosophila) [Source:	0.56686
ENSMUSG00000084959	4933407I08Rik	RIKEN cDNA 4933407I08 gene [Source:MGI Symbol	0.56687
ENSMUSG00000082310	Gm12904	predicted gene 12904 [Source:MGI Symbol;Acc:MG	0.56689
ENSMUSG00000034427	Myo15b	myosin XVB [Source:MGI Symbol;Acc:MGI:2685534	0.56691
ENSMUSG00000034520	Gjc1	gap junction protein, gamma 1 [Source:MGI Symbo	0.56717
ENSMUSG00000071265	1700086L19Rik	RIKEN cDNA 1700086L19 gene [Source:MGI Symbo	0.56717
ENSMUSG00000020847	Rph3al	rabphilin 3A-like (without C2 domains) [Source:MG	0.5672
ENSMUSG00000024594	Prrc1	proline-rich coiled-coil 1 [Source:MGI Symbol;Acc:N	0.56724
ENSMUSG00000036181	Hist1h1c	histone cluster 1, H1c [Source:MGI Symbol;Acc:MG	0.56725
ENSMUSG00000008398	Elk3	ELK3, member of ETS oncogene family [Source:MGI	0.56727

ENSMUSG00000063801	Ap3s2	adaptor-related protein complex 3, sigma 2 subunit	0.56736
ENSMUSG00000038861	Pi4kb	phosphatidylinositol 4-kinase, catalytic, beta polype	0.56762
ENSMUSG00000095836	Gm21960	predicted gene, 21960 [Source:MGI Symbol;Acc:MG	0.56769
ENSMUSG00000090194	Gm16161	predicted gene 16161 [Source:MGI Symbol;Acc:MG	0.56775
ENSMUSG00000081451	Gm12734	predicted gene 12734 [Source:MGI Symbol;Acc:MG	0.5678
ENSMUSG00000017314	Mpp2	membrane protein, palmitoylated 2 (MAGUK p55 s	0.56787
ENSMUSG00000062116	Zfp954	zinc finger protein 954 [Source:MGI Symbol;Acc:MG	0.56793
ENSMUSG00000042942	Greb1l	growth regulation by estrogen in breast cancer-like	0.56794
ENSMUSG00000022358	Fbxo32	F-box protein 32 [Source:MGI Symbol;Acc:MGI:191	0.56796
ENSMUSG00000080198	Gm13573	predicted gene 13573 [Source:MGI Symbol;Acc:MG	0.56799
ENSMUSG00000076498	Trbc2	T cell receptor beta, constant 2 [Source:MGI Symbo	0.568
ENSMUSG00000079261	Gm15217	predicted gene 15217 [Source:MGI Symbol;Acc:MG	0.56806
ENSMUSG00000001739	Cldn15	claudin 15 [Source:MGI Symbol;Acc:MGI:1913103]	0.56809
ENSMUSG00000037820	Tgm2	transglutaminase 2, C polypeptide [Source:MGI Syn	0.56829
ENSMUSG00000029545	Acads	acyl-Coenzyme A dehydrogenase, short chain [Sour	0.5683
ENSMUSG00000074971	Fibin	fin bud initiation factor homolog (zebrafish) [Source	0.56837
ENSMUSG00000062110	Scfd2	Sec1 family domain containing 2 [Source:MGI Symb	0.5685
ENSMUSG00000056211	R3hdm1	R3H domain containing 1 [Source:MGI Symbol;Acc:	0.56858
ENSMUSG00000097077	Gm16712	predicted gene, 16712 [Source:MGI Symbol;Acc:MG	0.56858
ENSMUSG00000027495	Fam210b	family with sequence similarity 210, member B [Sou	0.5687
ENSMUSG00000020264	Slc36a2	solute carrier family 36 (proton/amino acid sympor	0.56871
ENSMUSG00000010529	Gm266	predicted gene 266 [Source:MGI Symbol;Acc:MGI:2	0.56888
ENSMUSG00000034570	Inpp5j	inositol polyphosphate 5-phosphatase J [Source:MG	0.569
ENSMUSG00000031562	Dctd	dCMP deaminase [Source:MGI Symbol;Acc:MGI:244	0.56906
ENSMUSG00000097640	Gm20033	predicted gene, 20033 [Source:MGI Symbol;Acc:MG	0.56906
ENSMUSG00000089900	0610038L08Rik	RIKEN cDNA 0610038L08 gene [Source:MGI Symbo	0.56927
ENSMUSG00000026322	Htr4	5 hydroxytryptamine (serotonin) receptor 4 [Source	0.56933
ENSMUSG00000044864	Ankrd50	ankyrin repeat domain 50 [Source:MGI Symbol;Acc	0.56945
ENSMUSG00000087594	BC039771	cDNA sequence BC039771 [Source:MGI Symbol;Acc	0.56948
ENSMUSG00000092482	Gm20531	predicted gene 20531 [Source:MGI Symbol;Acc:MG	0.5696
ENSMUSG00000080790	Gm7638	predicted gene 7638 [Source:MGI Symbol;Acc:MGI:	0.56964
ENSMUSG00000029664	Tfpi2	tissue factor pathway inhibitor 2 [Source:MGI Symb	0.56967
ENSMUSG00000042831	Alkbh6	alkB, alkylation repair homolog 6 (E. coli) [Source:M	0.56969
ENSMUSG00000024359	Hspa9	heat shock protein 9 [Source:MGI Symbol;Acc:MGI:	0.5697
ENSMUSG00000035692	Isg15	ISG15 ubiquitin-like modifier [Source:MGI Symbol;A	0.5699
ENSMUSG00000041556	Fbxo2	F-box protein 2 [Source:MGI Symbol;Acc:MGI:2446	0.56993
ENSMUSG00000091686	4930471C04Rik	RIKEN cDNA 4930471C04 gene [Source:MGI Symbo	0.56995
ENSMUSG00000092807	Mir199b	microRNA 199b [Source:MGI Symbol;Acc:MGI:2676	0.56998
ENSMUSG00000085840	Gm11261	predicted gene 11261 [Source:MGI Symbol;Acc:MG	0.57005
ENSMUSG00000026565	Pou2f1	POU domain, class 2, transcription factor 1 [Source	0.57017
ENSMUSG00000056228	Cars2	cysteinyl-tRNA synthetase 2 (mitochondrial)(putativ	0.57018
ENSMUSG00000074862	BC025920	cDNA sequence BC025920 [Source:MGI Symbol;Acc	0.5703
ENSMUSG00000073409	H2-Q6	histocompatibility 2, Q region locus 6 [Source:MGI S	0.57032
ENSMUSG00000071102	Gm10313	predicted pseudogene 10313 [Source:MGI Symbol;	0.57034
ENSMUSG00000021326	Trim27	tripartite motif-containing 27 [Source:MGI Symbol;	0.5704
ENSMUSG00000021922	Itih4	inter alpha-trypsin inhibitor, heavy chain 4 [Source:	0.57041
ENSMUSG00000030643	Rab30	RAB30, member RAS oncogene family [Source:MGI	0.57042

ENSMUSG00000086682	Gm16023	predicted gene 16023 [Source:MGI Symbol;Acc:MG	0.57047
ENSMUSG00000051728	4930563D23Rik	RIKEN cDNA 4930563D23 gene [Source:MGI Symbo	0.57051
ENSMUSG00000033063	Cntnap3	contactin associated protein-like 3 [Source:MGI Sy	0.57058
ENSMUSG00000073077	Gm7173	predicted gene 7173 [Source:MGI Symbol;Acc:MGI	0.57058
ENSMUSG00000017697	Ada	adenosine deaminase [Source:MGI Symbol;Acc:MG	0.57065
ENSMUSG00000024798	Htr7	5-hydroxytryptamine (serotonin) receptor 7 [Sourc	0.57065
ENSMUSG00000037773	Pced1a	PC-esterase domain containing 1A [Source:MGI Sym	0.57066
ENSMUSG00000055435	Maf	avian musculoaponeurotic fibrosarcoma (v-maf) AS	0.57087
ENSMUSG00000029864	Gstk1	glutathione S-transferase kappa 1 [Source:MGI Sym	0.57094
ENSMUSG00000065147	Snora31	small nucleolar RNA, H/ACA box 31 [Source:MGI Sy	0.57095
ENSMUSG00000063320	1190007I07Rik	RIKEN cDNA 1190007I07 gene [Source:MGI Symbol	0.57103
ENSMUSG00000042096	Dao	D-amino acid oxidase [Source:MGI Symbol;Acc:MG	0.57104
ENSMUSG00000029521	Chek2	checkpoint kinase 2 [Source:MGI Symbol;Acc:MGI:	0.57105
ENSMUSG00000044006	Cilp2	cartilage intermediate layer protein 2 [Source:MGI	0.57124
ENSMUSG00000026181	Ppm1f	protein phosphatase 1F (PP2C domain containing)	0.57144
ENSMUSG00000036992	Nxt1	NTF2-related export protein 1 [Source:MGI Symbol	0.57147
ENSMUSG00000090777	Gm7873	predicted gene 7873 [Source:MGI Symbol;Acc:MGI	0.57148
ENSMUSG00000059995	Atxn7l3	ataxin 7-like 3 [Source:MGI Symbol;Acc:MGI:30362	0.57151
ENSMUSG00000063193	Cd300lb	CD300 antigen like family member B [Source:MGI S	0.57152
ENSMUSG00000074500	Zfp558	zinc finger protein 558 [Source:MGI Symbol;Acc:M	0.57184
ENSMUSG00000039976	Tbc1d16	TBC1 domain family, member 16 [Source:MGI Sym	0.57206
ENSMUSG00000080811	Gm14513	predicted gene 14513 [Source:MGI Symbol;Acc:MG	0.57207
ENSMUSG00000053470	Kdm3a	lysine (K)-specific demethylase 3A [Source:MGI Sym	0.57213
ENSMUSG00000041482	Piezo2	piezo-type mechanosensitive ion channel compone	0.57217
ENSMUSG00000029123	Stk32b	serine/threonine kinase 32B [Source:MGI Symbol;A	0.57219
ENSMUSG00000079639	Rhox4a	reproductive homeobox 4A [Source:MGI Symbol;A	0.57219
ENSMUSG00000026439	Rbbp5	retinoblastoma binding protein 5 [Source:MGI Sym	0.5722
ENSMUSG00000078926	Cdc20b	cell division cycle 20B [Source:MGI Symbol;Acc:MG	0.57221
ENSMUSG00000000142	Axin2	axin2 [Source:MGI Symbol;Acc:MGI:1270862]	0.57222
ENSMUSG00000024663	Rab3il1	RAB3A interacting protein (rabin3)-like 1 [Source:M	0.57223
ENSMUSG00000024791	Cdca5	cell division cycle associated 5 [Source:MGI Symbol	0.57224
ENSMUSG00000009734	Pou6f2	POU domain, class 6, transcription factor 2 [Source	0.57225
ENSMUSG00000023078	Cxcl13	chemokine (C-X-C motif) ligand 13 [Source:MGI Sym	0.57226
ENSMUSG00000044730	9930104L06Rik	RIKEN cDNA 9930104L06 gene [Source:MGI Symbo	0.57231
ENSMUSG00000083774	Gm7180	predicted pseudogene 7180 [Source:MGI Symbol;A	0.5724
ENSMUSG00000072918	1700022K14Rik	RIKEN cDNA 1700022K14 gene [Source:MGI Symbo	0.57246
ENSMUSG00000084865	Gm15723	predicted gene 15723 [Source:MGI Symbol;Acc:MG	0.57246
ENSMUSG00000005952	Trpv1	transient receptor potential cation channel, subfam	0.5725
ENSMUSG00000024483	Ankhd1	ankyrin repeat and KH domain containing 1 [Source	0.57251
ENSMUSG00000088071	Gm22818	predicted gene, 22818 [Source:MGI Symbol;Acc:M	0.57252
ENSMUSG00000091363	Gm7701	predicted gene 7701 [Source:MGI Symbol;Acc:MGI	0.57278
ENSMUSG00000029219	Slc10a4	solute carrier family 10 (sodium/bile acid cotransp	0.5728
ENSMUSG00000031654	Cbln1	cerebellin 1 precursor protein [Source:MGI Symbol	0.57281
ENSMUSG00000050625	Ccdc121	coiled-coil domain containing 121 [Source:MGI Sym	0.57281
ENSMUSG00000083579	Gm15538	predicted gene 15538 [Source:MGI Symbol;Acc:MG	0.57301
ENSMUSG00000054612	Mgmt	O-6-methylguanine-DNA methyltransferase [Sourc	0.57302
ENSMUSG00000078652	Psme3	proteasome (prosome, macropain) activator subu	0.57303

ENSMUSG00000060152	Pop5	processing of precursor 5, ribonuclease P/MRP fam	0.57315
ENSMUSG00000022216	Psme1	proteasome (prosome, macropain) activator subun	0.57326
ENSMUSG00000072969	Armcx5	armadillo repeat containing, X-linked 5 [Source:MG	0.57333
ENSMUSG00000024501	Dpysl3	dihydropyrimidinase-like 3 [Source:MGI Symbol;Acc	0.57335
ENSMUSG00000060882	Kcnd2	potassium voltage-gated channel, Shal-related fam	0.57336
ENSMUSG00000052387	Trpm3	transient receptor potential cation channel, subfam	0.57339
ENSMUSG00000028862	Map3k6	mitogen-activated protein kinase kinase kinase 6 [S	0.57343
ENSMUSG00000048067	Olfr1349	olfactory receptor 1349 [Source:MGI Symbol;Acc:M	0.57355
ENSMUSG00000031962	Cdh15	cadherin 15 [Source:MGI Symbol;Acc:MGI:106672]	0.57359
ENSMUSG00000060216	Arrb2	arrestin, beta 2 [Source:MGI Symbol;Acc:MGI:9947	0.57361
ENSMUSG00000004070	Hmox2	heme oxygenase (decycling) 2 [Source:MGI Symbol	0.57375
ENSMUSG00000086779	Gm13562	predicted gene 13562 [Source:MGI Symbol;Acc:MG	0.57385
ENSMUSG00000026536	Mnda	myeloid cell nuclear differentiation antigen [Source	0.57386
ENSMUSG00000028967	Errfi1	ERBB receptor feedback inhibitor 1 [Source:MGI Sy	0.57407
ENSMUSG00000097564	C430014B12Rik	RIKEN cDNA C430014B12 gene [Source:MGI Symbo	0.57407
ENSMUSG00000031985	Gnpat	glyceronephosphate O-acyltransferase [Source:MG	0.57417
ENSMUSG00000034579	Pla2g3	phospholipase A2, group III [Source:MGI Symbol;Ac	0.57419
ENSMUSG00000058754	Gm10048	predicted gene 10048 [Source:MGI Symbol;Acc:MG	0.57419
ENSMUSG00000082322	Gm9164	predicted gene 9164 [Source:MGI Symbol;Acc:MGI:	0.57437
ENSMUSG00000097170	Gm16982	predicted gene, 16982 [Source:MGI Symbol;Acc:M	0.57439
ENSMUSG00000037594	BC022687	cDNA sequence BC022687 [Source:MGI Symbol;Acc	0.57445
ENSMUSG00000002342	Tmem161a	transmembrane protein 161A [Source:MGI Symbol;	0.57449
ENSMUSG00000084910	C630043F03Rik	RIKEN cDNA C630043F03 gene [Source:MGI Symbo	0.57453
ENSMUSG00000087507	Gm16141	predicted gene 16141 [Source:MGI Symbol;Acc:MG	0.57455
ENSMUSG00000032199	Polr2m	polymerase (RNA) II (DNA directed) polypeptide M	0.57456
ENSMUSG00000085167	Gm12063	predicted gene 12063 [Source:MGI Symbol;Acc:MG	0.57456
ENSMUSG00000005474	Myl10	myosin, light chain 10, regulatory [Source:MGI Sym	0.57462
ENSMUSG00000074505	Fat3	FAT tumor suppressor homolog 3 (Drosophila) [Sou	0.57466
ENSMUSG00000090236	Car15	carbonic anhydrase 15 [Source:MGI Symbol;Acc:M	0.57467
ENSMUSG00000030086	Chchd6	coiled-coil-helix-coiled-coil-helix domain containing	0.5748
ENSMUSG00000007836	Hnrnpa0	heterogeneous nuclear ribonucleoprotein A0 [Sour	0.57487
ENSMUSG00000001440	Kpnb1	karyopherin (importin) beta 1 [Source:MGI Symbol;	0.57494
ENSMUSG00000027077	Smtnl1	smoothelin-like 1 [Source:MGI Symbol;Acc:MGI:19	0.575
ENSMUSG00000026389	Steap3	STEAP family member 3 [Source:MGI Symbol;Acc:M	0.57511
ENSMUSG00000040043	Rbms2	RNA binding motif, single stranded interacting prot	0.57513
ENSMUSG00000026927	Sdccag3	serologically defined colon cancer antigen 3 [Sourc	0.57519
ENSMUSG00000030714	Ccdc101	coiled-coil domain containing 101 [Source:MGI Sym	0.57521
ENSMUSG00000045071	E130308A19Rik	RIKEN cDNA E130308A19 gene [Source:MGI Symbo	0.57526
ENSMUSG00000086465	Gm16049	predicted gene 16049 [Source:MGI Symbol;Acc:MG	0.57528
ENSMUSG00000073794	I0C0044D17Rik	RIKEN cDNA I0C0044D17 gene [Source:MGI Symbo	0.57531
ENSMUSG00000030206	Gsg1	germ cell-specific gene 1 [Source:MGI Symbol;Acc:I	0.57534
ENSMUSG00000087595	1810012K08Rik	RIKEN cDNA 1810012K08 gene [Source:MGI Symbo	0.57539
ENSMUSG00000059669	Taf1b	TATA box binding protein (Tbp)-associated factor, R	0.57544
ENSMUSG00000040836	Gpr161	G protein-coupled receptor 161 [Source:MGI Symb	0.57546
ENSMUSG00000085601	Gm4969	predicted gene 4969 [Source:MGI Symbol;Acc:MGI:	0.57551
ENSMUSG00000024400	Wdr33	WD repeat domain 33 [Source:MGI Symbol;Acc:MG	0.5756
ENSMUSG00000083314	Gm11742	predicted gene 11742 [Source:MGI Symbol;Acc:MG	0.57562

ENSMUSG00000022313	Utp23	UTP23, small subunit (SSU) processome componen	0.57567
ENSMUSG00000029373	Pf4	platelet factor 4 [Source:MGI Symbol;Acc:MGI:188	0.57578
ENSMUSG00000002249	Tead3	TEA domain family member 3 [Source:MGI Symbol;	0.57592
ENSMUSG00000082600	Gm9673	predicted gene 9673 [Source:MGI Symbol;Acc:MGI:	0.57595
ENSMUSG00000088176	Gm23094	predicted gene, 23094 [Source:MGI Symbol;Acc:M	0.57605
ENSMUSG00000097673	Gm26608	predicted gene, 26608 [Source:MGI Symbol;Acc:M	0.57605
ENSMUSG00000089090	Gm25655	predicted gene, 25655 [Source:MGI Symbol;Acc:M	0.57607
ENSMUSG00000081889	Gm13188	predicted gene 13188 [Source:MGI Symbol;Acc:MG	0.57609
ENSMUSG00000020639	Pfn4	profilin family, member 4 [Source:MGI Symbol;Acc:	0.57614
ENSMUSG00000031297	Slc7a3	solute carrier family 7 (cationic amino acid transpo	0.57626
ENSMUSG00000020926	Adam11	a disintegrin and metallopeptidase domain 11 [Sou	0.57628
ENSMUSG00000032127	Vps11	vacuolar protein sorting 11 (yeast) [Source:MGI Sy	0.57628
ENSMUSG00000048109	Rbm15	RNA binding motif protein 15 [Source:MGI Symbol;	0.5763
ENSMUSG00000021820	Camk2g	calcium/calmodulin-dependent protein kinase II ga	0.57633
ENSMUSG00000093276	Gm26237	predicted gene, 26237 [Source:MGI Symbol;Acc:M	0.57636
ENSMUSG00000054863	Fam19a5	family with sequence similarity 19, member A5 [So	0.57639
ENSMUSG00000084792	1700056N10Rik	RIKEN cDNA 1700056N10 gene [Source:MGI Symbo	0.57646
ENSMUSG00000041040	Fam117b	family with sequence similarity 117, member B [So	0.57647
ENSMUSG00000087648	E130018N17Rik	RIKEN cDNA E130018N17 gene [Source:MGI Symbo	0.57651
ENSMUSG00000006724	Cyp27b1	cytochrome P450, family 27, subfamily b, polypepti	0.57652
ENSMUSG00000073555	Gm4951	predicted gene 4951 [Source:MGI Symbol;Acc:MGI:	0.57656
ENSMUSG00000091561	Gm6665	predicted gene 6665 [Source:MGI Symbol;Acc:MGI:	0.57656
ENSMUSG00000078490	C030017K20Rik	RIKEN cDNA C030017K20 gene [Source:MGI Symbo	0.57658
ENSMUSG00000064853	Gm23442	predicted gene, 23442 [Source:MGI Symbol;Acc:M	0.57659
ENSMUSG00000027893	Ahcyl1	S-adenosylhomocysteine hydrolase-like 1 [Source:M	0.5766
ENSMUSG00000087115	Gm12360	predicted gene 12360 [Source:MGI Symbol;Acc:MG	0.57662
ENSMUSG00000029235	Pdcl2	phosducin-like 2 [Source:MGI Symbol;Acc:MGI:189	0.57663
ENSMUSG00000033644	Piwil2	piwi-like RNA-mediated gene silencing 2 [Source:M	0.57665
ENSMUSG00000083760	Gm14524	predicted gene 14524 [Source:MGI Symbol;Acc:MG	0.57665
ENSMUSG00000078154	Gm12184	predicted gene 12184 [Source:MGI Symbol;Acc:MG	0.57667
ENSMUSG00000030327	Necap1	NECAP endocytosis associated 1 [Source:MGI Symb	0.57668
ENSMUSG00000060715	1700019A02Rik	RIKEN cDNA 1700019A02 gene [Source:MGI Symbo	0.5767
ENSMUSG00000073491	Pydc4	pyrin domain containing 4 [Source:MGI Symbol;Acc	0.5767
ENSMUSG00000083679	Gm12892	predicted gene 12892 [Source:MGI Symbol;Acc:MG	0.57684
ENSMUSG00000028821	Syf2	SYF2 homolog, RNA splicing factor (S. cerevisiae) [S	0.57692
ENSMUSG00000029110	Rnf4	ring finger protein 4 [Source:MGI Symbol;Acc:MGI:	0.57692
ENSMUSG00000087519	AV039307	expressed sequence AV039307 [Source:MGI Symbo	0.57694
ENSMUSG00000070063	Snora33	small nucleolar RNA, H/ACA box 33 [Source:MGI Sy	0.57713
ENSMUSG00000052133	Sema5b	sema domain, seven thrombospondin repeats (type	0.57724
ENSMUSG00000070738	Dgkd	diacylglycerol kinase, delta [Source:MGI Symbol;Ac	0.57732
ENSMUSG00000073600	Prob1	proline rich basic protein 1 [Source:MGI Symbol;Ac	0.57733
ENSMUSG00000089719	Gm15758	predicted gene 15758 [Source:MGI Symbol;Acc:MG	0.57741
ENSMUSG00000067261	Foxd3	forkhead box D3 [Source:MGI Symbol;Acc:MGI:134	0.57746
ENSMUSG00000038042	Ptpdc1	protein tyrosine phosphatase domain containing 1	0.57752
ENSMUSG00000090210	Itga10	integrin, alpha 10 [Source:MGI Symbol;Acc:MGI:21	0.57755
ENSMUSG00000021264	Yy1	YY1 transcription factor [Source:MGI Symbol;Acc:M	0.57766
ENSMUSG00000091649	Phf11b	PHD finger protein 11B [Source:MGI Symbol;Acc:M	0.57803

ENSMUSG00000030592	Ryr1	ryanodine receptor 1, skeletal muscle [Source:MGI	0.57805
ENSMUSG00000022437	Samm50	sorting and assembly machinery component 50 hor	0.57814
ENSMUSG00000085236	2610206C17Rik	RIKEN cDNA 2610206C17 gene [Source:MGI Symbo	0.57818
ENSMUSG00000045092	S1pr1	sphingosine-1-phosphate receptor 1 [Source:MGI S	0.57836
ENSMUSG00000040287	Stac3	SH3 and cysteine rich domain 3 [Source:MGI Symbo	0.57837
ENSMUSG00000025092	Hspa12a	heat shock protein 12A [Source:MGI Symbol;Acc:M	0.57838
ENSMUSG00000024772	Ehd1	EH-domain containing 1 [Source:MGI Symbol;Acc:M	0.57842
ENSMUSG00000048327	Ckap2l	cytoskeleton associated protein 2-like [Source:MGI	0.57855
ENSMUSG00000032028	Nxpe2	neurexophilin and PC-esterase domain family, mem	0.57871
ENSMUSG00000029163	Emilin1	elastin microfibril interfacer 1 [Source:MGI Symbol;	0.57877
ENSMUSG00000031753	Cog4	component of oligomeric golgi complex 4 [Source:M	0.57893
ENSMUSG00000073538	E330020D12Rik	Riken cDNA E330020D12 gene [Source:MGI Symbo	0.57896
ENSMUSG00000041126	H2afv	H2A histone family, member V [Source:MGI Symbo	0.579
ENSMUSG00000083473	Gm13207	predicted gene 13207 [Source:MGI Symbol;Acc:MG	0.57903
ENSMUSG00000091955	Gm9844	predicted pseudogene 9844 [Source:MGI Symbol;A	0.57906
ENSMUSG00000080544	Gm22714	predicted gene, 22714 [Source:MGI Symbol;Acc:MG	0.57909
ENSMUSG00000042594	Sh2b3	SH2B adaptor protein 3 [Source:MGI Symbol;Acc:M	0.57912
ENSMUSG00000086643	Gm15687	predicted gene 15687 [Source:MGI Symbol;Acc:MG	0.57914
ENSMUSG00000087178	A230056P14Rik	RIKEN cDNA A230056P14 gene [Source:MGI Symbo	0.57918
ENSMUSG00000009291	Pttg1ip	pituitary tumor-transforming 1 interacting protein	0.57924
ENSMUSG00000048636	A730049H05Rik	RIKEN cDNA A730049H05 gene [Source:MGI Symbo	0.57934
ENSMUSG00000038346	Zfp384	zinc finger protein 384 [Source:MGI Symbol;Acc:MG	0.57936
ENSMUSG00000029171	Pgm1	phosphoglucomutase 1 [Source:MGI Symbol;Acc:M	0.57945
ENSMUSG00000030433	Sbk2	SH3-binding domain kinase family, member 2 [Sour	0.57945
ENSMUSG00000071654	AI462493	expressed sequence AI462493 [Source:MGI Symbol	0.57953
ENSMUSG00000036918	Ttc7	tetratricopeptide repeat domain 7 [Source:MGI Syn	0.57968
ENSMUSG00000095134	Gm21857	predicted gene, 21857 [Source:MGI Symbol;Acc:MG	0.57968
ENSMUSG00000048874	Phf3	PHD finger protein 3 [Source:MGI Symbol;Acc:MGI:	0.5797
ENSMUSG00000082816	Gm11953	predicted gene 11953 [Source:MGI Symbol;Acc:MG	0.57971
ENSMUSG00000097772	5430416N02Rik	RIKEN cDNA 5430416N02 gene [Source:MGI Symbo	0.57974
ENSMUSG00000046792	Zfp787	zinc finger protein 787 [Source:MGI Symbol;Acc:MG	0.57976
ENSMUSG00000043673	Kcns3	potassium voltage-gated channel, delayed-rectifier	0.57978
ENSMUSG00000033308	Dpyd	dihydropyrimidine dehydrogenase [Source:MGI Syn	0.57979
ENSMUSG00000034701	Neurod1	neurogenic differentiation 1 [Source:MGI Symbol;A	0.57986
ENSMUSG00000052632	Asap2	ArfGAP with SH3 domain, ankyrin repeat and PH do	0.58021
ENSMUSG00000039886	Tmem120a	transmembrane protein 120A [Source:MGI Symbol	0.58027
ENSMUSG00000042672	Dcst1	DC-STAMP domain containing 1 [Source:MGI Symb	0.58027
ENSMUSG00000045327	6330549D23Rik	RIKEN cDNA 6330549D23 gene [Source:MGI Symbo	0.58033
ENSMUSG00000051008	4930412M03Rik	RIKEN cDNA 4930412M03 gene [Source:MGI Symbo	0.58033
ENSMUSG00000033585	Ndn	necdin [Source:MGI Symbol;Acc:MGI:97290]	0.58046
ENSMUSG00000026788	Zbtb43	zinc finger and BTB domain containing 43 [Source:M	0.58061
ENSMUSG00000041141	Pnmal1	PNMA-like 1 [Source:MGI Symbol;Acc:MGI:191894	0.58061
ENSMUSG00000028747	Htr6	5-hydroxytryptamine (serotonin) receptor 6 [Sourc	0.58062
ENSMUSG00000028436	Dcaf12	DDB1 and CUL4 associated factor 12 [Source:MGI S	0.58066
ENSMUSG00000037085	Trmt12	tRNA methyltranferase 12 [Source:MGI Symbol;Acc	0.58066
ENSMUSG00000087456	Gm16322	predicted gene 16322 [Source:MGI Symbol;Acc:MG	0.58074
ENSMUSG00000008318	Relt	RELT tumor necrosis factor receptor [Source:MGI Sy	0.58078

ENSMUSG00000020946	Gosr2	golgi SNAP receptor complex member 2 [Source:M	0.58084
ENSMUSG00000074056	Gm10615	predicted gene 10615 [Source:MGI Symbol;Acc:MG	0.5809
ENSMUSG00000024059	Clip4	CAP-GLY domain containing linker protein family, m	0.58095
ENSMUSG00000039686	Zer1	zyg-11 related, cell cycle regulator [Source:MGI Syn	0.58097
ENSMUSG00000039840	Epg5	ectopic P-granules autophagy protein 5 homolog (C	0.58102
ENSMUSG00000022853	Ehhadh	enoyl-Coenzyme A, hydratase/3-hydroxyacyl Coenz	0.58108
ENSMUSG00000021033	Gstz1	glutathione transferase zeta 1 (maleylacetoacetate	0.58119
ENSMUSG00000025504	Eps8l2	EPS8-like 2 [Source:MGI Symbol;Acc:MGI:2138828]	0.58119
ENSMUSG00000027800	Tm4sf1	transmembrane 4 superfamily member 1 [Source:M	0.58126
ENSMUSG00000028820	Sfpq	splicing factor proline/glutamine rich (polypyrimidi	0.58128
ENSMUSG00000047307	Pcdhb13	protocadherin beta 13 [Source:MGI Symbol;Acc:MG	0.58136
ENSMUSG00000037224	Zfyve28	zinc finger, FYVE domain containing 28 [Source:MG	0.58141
ENSMUSG00000053297	AI854703	expressed sequence AI854703 [Source:MGI Symbol	0.58143
ENSMUSG00000091515	Zfp422-rs1	zinc finger protein 422, related sequence 1 [Source	0.58147
ENSMUSG00000097746	Gm6225	predicted gene 6225 [Source:MGI Symbol;Acc:MGI:	0.58148
ENSMUSG00000081034	Gm5944	predicted gene 5944 [Source:MGI Symbol;Acc:MGI:	0.58161
ENSMUSG00000011349	Dmrtc2	doublesex and mab-3 related transcription factor li	0.58164
ENSMUSG00000085545	Gm13553	predicted gene 13553 [Source:MGI Symbol;Acc:MG	0.58174
ENSMUSG00000035337	Uchl4	ubiquitin carboxyl-terminal esterase L4 [Source:MG	0.58191
ENSMUSG00000027475	Kif3b	kinesin family member 3B [Source:MGI Symbol;Acc	0.58214
ENSMUSG00000097113	Gm19705	predicted gene, 19705 [Source:MGI Symbol;Acc:M	0.58225
ENSMUSG00000039246	Lyplal1	lysophospholipase-like 1 [Source:MGI Symbol;Acc:M	0.58229
ENSMUSG00000056025	Clca1	chloride channel calcium activated 1 [Source:MGI S	0.58229
ENSMUSG00000078949	R3hdml	R3H domain containing-like [Source:MGI Symbol;Ac	0.58233
ENSMUSG00000044461	Shisa2	shisa homolog 2 (Xenopus laevis) [Source:MGI Sym	0.58241
ENSMUSG00000060261	Gtf2i	general transcription factor II I [Source:MGI Symbo	0.58247
ENSMUSG00000022019	Tdrd3	tudor domain containing 3 [Source:MGI Symbol;Acc	0.58259
ENSMUSG00000028398	Tmem261	transmembrane protein 261 [Source:MGI Symbol;A	0.58259
ENSMUSG00000039209	Rpl39l	ribosomal protein L39-like [Source:MGI Symbol;Acc	0.58265
ENSMUSG00000037463	Fbxo27	F-box protein 27 [Source:MGI Symbol;Acc:MGI:268	0.58284
ENSMUSG00000042073	Abhd14b	abhydrolase domain containing 14b [Source:MGI Sy	0.58295
ENSMUSG00000028643	Ccdc23	coiled-coil domain containing 23 [Source:MGI Symb	0.58296
ENSMUSG00000082396	Zfp389	zinc finger protein 389 [Source:MGI Symbol;Acc:M	0.58298
ENSMUSG00000047986	Palm3	paralemmin 3 [Source:MGI Symbol;Acc:MGI:19215	0.5831
ENSMUSG00000065434	Mir7-1	microRNA 7-1 [Source:MGI Symbol;Acc:MGI:36194	0.58332
ENSMUSG00000030986	Dhx32	DEAH (Asp-Glu-Ala-His) box polypeptide 32 [Source	0.58333
ENSMUSG00000055760	Gemin6	gem (nuclear organelle) associated protein 6 [Sour	0.58334
ENSMUSG00000085603	Gm11346	predicted gene 11346 [Source:MGI Symbol;Acc:MG	0.58353
ENSMUSG00000023243	Kcnk5	potassium channel, subfamily K, member 5 [Source	0.58358
ENSMUSG00000016128	Stard13	StAR-related lipid transfer (START) domain containi	0.58368
ENSMUSG00000016619	Nup50	nucleoporin 50 [Source:MGI Symbol;Acc:MGI:1351	0.58369
ENSMUSG00000021367	Edn1	endothelin 1 [Source:MGI Symbol;Acc:MGI:95283]	0.58376
ENSMUSG00000097665	Gm16853	predicted gene, 16853 [Source:MGI Symbol;Acc:M	0.58376
ENSMUSG00000065089	Gm26493	predicted gene, 26493 [Source:MGI Symbol;Acc:M	0.58395
ENSMUSG00000072829	Gm7292	predicted gene 7292 [Source:MGI Symbol;Acc:MGI:	0.58395
ENSMUSG00000019478	Rab4a	RAB4A, member RAS oncogene family [Source:MGI	0.58396
ENSMUSG00000089255	Snora78	small nucleolar RNA, H/ACA box 7 [Source:MGI Sym	0.58396

ENSMUSG00000031858	Mau2	MAU2 chromatid cohesion factor homolog (C. eleg	0.58405
ENSMUSG00000081249	Gm11517	predicted gene 11517 [Source:MGI Symbol;Acc:MG	0.5841
ENSMUSG00000026404	Ddx59	DEAD (Asp-Glu-Ala-Asp) box polypeptide 59 [Sourc	0.58417
ENSMUSG00000035473	Galm	galactose mutarotase [Source:MGI Symbol;Acc:MG	0.58425
ENSMUSG00000021175	Cdca7l	cell division cycle associated 7 like [Source:MGI Syn	0.5843
ENSMUSG00000035355	Kcnh4	potassium voltage-gated channel, subfamily H (eag	0.58445
ENSMUSG00000048251	Bcl11b	B cell leukemia/lymphoma 11B [Source:MGI Symbo	0.58445
ENSMUSG00000093495	Gm23368	predicted gene, 23368 [Source:MGI Symbol;Acc:M	0.58453
ENSMUSG00000027425	Csrp2bp	cysteine and glycine-rich protein 2 binding protein	0.58454
ENSMUSG00000040265	Dnm3	dynamin 3 [Source:MGI Symbol;Acc:MGI:1341299]	0.58456
ENSMUSG00000085262	Gm11574	predicted gene 11574 [Source:MGI Symbol;Acc:MG	0.58459
ENSMUSG00000010110	Stx5a	syntaxin 5A [Source:MGI Symbol;Acc:MGI:1928483	0.58481
ENSMUSG00000040865	Ino80d	INO80 complex subunit D [Source:MGI Symbol;Acc:	0.58483
ENSMUSG00000025969	Nrp2	neuropilin 2 [Source:MGI Symbol;Acc:MGI:1100492	0.58495
ENSMUSG00000091412	Gm2895	predicted gene 2895 [Source:MGI Symbol;Acc:MGI:	0.58497
ENSMUSG00000079173	Zan	zonadhesin [Source:MGI Symbol;Acc:MGI:106656]	0.58499
ENSMUSG00000031755	Bbs2	Bardet-Biedl syndrome 2 (human) [Source:MGI Sym	0.58502
ENSMUSG00000039913	Pak7	p21 protein (Cdc42/Rac)-activated kinase 7 [Source	0.58515
ENSMUSG00000058741	Prr19	proline rich 19 [Source:MGI Symbol;Acc:MGI:36485	0.58516
ENSMUSG00000086376	1700071G01Rik	RIKEN cDNA 1700071G01 gene [Source:MGI Symbo	0.58533
ENSMUSG00000029715	Pop7	processing of precursor 7, ribonuclease P family, (S	0.58536
ENSMUSG00000048814	Lonrf2	LON peptidase N-terminal domain and ring finger 2	0.58536
ENSMUSG00000048039	Isg20l2	interferon stimulated exonuclease gene 20-like 2 [S	0.58546
ENSMUSG00000026028	Trak2	trafficking protein, kinesin binding 2 [Source:MGI S	0.58547
ENSMUSG00000078429	Ctdsp2	CTD (carboxy-terminal domain, RNA polymerase II,	0.58547
ENSMUSG00000078651	Aoc2	amine oxidase, copper containing 2 (retina-specific	0.58551
ENSMUSG00000030942	Thumpd1	THUMP domain containing 1 [Source:MGI Symbol;A	0.58558
ENSMUSG00000040479	Dgkz	diacylglycerol kinase zeta [Source:MGI Symbol;Acc:	0.58563
ENSMUSG00000002274	Metrn	meteorin, glial cell differentiation regulator [Source	0.5858
ENSMUSG00000067925	Cxx1a	CAAX box 1A [Source:MGI Symbol;Acc:MGI:191340	0.58582
ENSMUSG00000053769	Lysmd1	LysM, putative peptidoglycan-binding, domain cont	0.58591
ENSMUSG00000087469	Gm16142	predicted gene 16142 [Source:MGI Symbol;Acc:MG	0.58595
ENSMUSG00000044551	9930012K11Rik	RIKEN cDNA 9930012K11 gene [Source:MGI Symbo	0.58604
ENSMUSG00000029814	Igf2bp3	insulin-like growth factor 2 mRNA binding protein 3	0.58605
ENSMUSG00000043140	Tmem186	transmembrane protein 186 [Source:MGI Symbol;A	0.5861
ENSMUSG00000026764	Kif5c	kinesin family member 5C [Source:MGI Symbol;Acc	0.58617
ENSMUSG00000062329	Cytl1	cytokine-like 1 [Source:MGI Symbol;Acc:MGI:26849	0.58618
ENSMUSG00000027589	Pcmtd2	protein-L-isoaspartate (D-aspartate) O-methyltrans	0.58619
ENSMUSG00000028792	Ak2	adenylate kinase 2 [Source:MGI Symbol;Acc:MGI:8	0.58623
ENSMUSG00000038605	Samd10	sterile alpha motif domain containing 10 [Source:M	0.58624
ENSMUSG00000000184	Ccnd2	cyclin D2 [Source:MGI Symbol;Acc:MGI:88314]	0.5863
ENSMUSG00000073207	Ccdc160	coiled-coil domain containing 160 [Source:MGI Sym	0.58633
ENSMUSG00000028392	Bspry	B-box and SPRY domain containing [Source:MGI Sy	0.58645
ENSMUSG00000001270	Ckb	creatine kinase, brain [Source:MGI Symbol;Acc:MG	0.58653
ENSMUSG00000084544	Gm23183	predicted gene, 23183 [Source:MGI Symbol;Acc:M	0.58654
ENSMUSG00000087395	Gm12705	predicted gene 12705 [Source:MGI Symbol;Acc:MG	0.58654
ENSMUSG00000028292	Rars2	arginyl-tRNA synthetase 2, mitochondrial [Source:N	0.58657

ENSMUSG00000079140	Gm6374	predicted gene 6374 [Source:MGI Symbol;Acc:MGI:	0.58658
ENSMUSG00000089687	Rab42	RAB42, member RAS oncogene family [Source:MGI	0.58665
ENSMUSG00000066158	AY512931	cDNA sequence AY512931 [Source:MGI Symbol;Acc	0.58674
ENSMUSG00000028057	Rit1	Ras-like without CAAX 1 [Source:MGI Symbol;Acc:M	0.58695
ENSMUSG00000073380	Arrdc5	arrestin domain containing 5 [Source:MGI Symbol;A	0.58718
ENSMUSG00000051730	Mettl5	methyltransferase like 5 [Source:MGI Symbol;Acc:M	0.58722
ENSMUSG00000090863	A530084C06Rik	RIKEN cDNA A530084C06 gene [Source:MGI Symbo	0.58722
ENSMUSG00000068373	D430041D05Rik	RIKEN cDNA D430041D05 gene [Source:MGI Symbo	0.58724
ENSMUSG00000087233	4930594C11Rik	RIKEN cDNA 4930594C11 gene [Source:MGI Symbo	0.58724
ENSMUSG00000086241	4930483K19Rik	RIKEN cDNA 4930483K19 gene [Source:MGI Symbo	0.58726
ENSMUSG00000029833	Trim24	tripartite motif-containing 24 [Source:MGI Symbol;	0.58742
ENSMUSG00000051022	Hs3st1	heparan sulfate (glucosamine) 3-O-sulfotransferase	0.58744
ENSMUSG00000043633	Fam221b	family with sequence similarity 221, member B [Sou	0.58752
ENSMUSG00000031453	Rasa3	RAS p21 protein activator 3 [Source:MGI Symbol;Ac	0.58758
ENSMUSG00000029726	Mepce	methylphosphate capping enzyme [Source:MGI Sym	0.58772
ENSMUSG00000088552	Mir1957	microRNA 1957 [Source:MGI Symbol;Acc:MGI:3837	0.58778
ENSMUSG00000024936	Kcnk7	potassium channel, subfamily K, member 7 [Source	0.5878
ENSMUSG00000029474	Rnf34	ring finger protein 34 [Source:MGI Symbol;Acc:MGI	0.58786
ENSMUSG00000071470	Ccnb1ip1	cyclin B1 interacting protein 1 [Source:MGI Symbol	0.58786
ENSMUSG00000054871	Tmem158	transmembrane protein 158 [Source:MGI Symbol;A	0.58801
ENSMUSG00000074064	Mlycd	malonyl-CoA decarboxylase [Source:MGI Symbol;Ac	0.5881
ENSMUSG00000074890	Lcmt2	leucine carboxyl methyltransferase 2 [Source:MGI S	0.5881
ENSMUSG00000041592	Sdk2	sidekick homolog 2 (chicken) [Source:MGI Symbol;A	0.58812
ENSMUSG00000042357	Gjb5	gap junction protein, beta 5 [Source:MGI Symbol;A	0.58813
ENSMUSG00000087449	Gm12971	predicted gene 12971 [Source:MGI Symbol;Acc:MG	0.5882
ENSMUSG00000079276	Gm4736	predicted gene 4736 [Source:MGI Symbol;Acc:MGI:	0.58822
ENSMUSG00000091498	Mpc1-ps	mitochondrial pyruvate carrier 1, pseudogene [Sou	0.58831
ENSMUSG00000047866	Lonp2	lon peptidase 2, peroxisomal [Source:MGI Symbol;A	0.58834
ENSMUSG00000046191	Pcdhb20	protocadherin beta 20 [Source:MGI Symbol;Acc:MG	0.58836
ENSMUSG00000089475	Gm25363	predicted gene, 25363 [Source:MGI Symbol;Acc:MG	0.58847
ENSMUSG00000085782	Gm15624	predicted gene 15624 [Source:MGI Symbol;Acc:MG	0.58853
ENSMUSG00000038570	Fam154b	family with sequence similarity 154, member B [Sou	0.58858
ENSMUSG00000085403	Gm13068	predicted gene 13068 [Source:MGI Symbol;Acc:MG	0.58861
ENSMUSG00000037410	Tbc1d2b	TBC1 domain family, member 2B [Source:MGI Syml	0.5887
ENSMUSG00000006418	Rnf114	ring finger protein 114 [Source:MGI Symbol;Acc:MG	0.58884
ENSMUSG00000048450	Msx1	msh homeobox 1 [Source:MGI Symbol;Acc:MGI:971	0.58893
ENSMUSG00000000753	Serpinf1	serine (or cysteine) peptidase inhibitor, clade F, me	0.58896
ENSMUSG00000071181	3830408C21Rik	RIKEN cDNA 3830408C21 gene [Source:MGI Symbo	0.58904
ENSMUSG00000045140	Pigw	phosphatidylinositol glycan anchor biosynthesis, cla	0.58912
ENSMUSG00000097317	Gm17281	predicted gene, 17281 [Source:MGI Symbol;Acc:MG	0.58914
ENSMUSG00000052812	Atad2b	ATPase family, AAA domain containing 2B [Source:I	0.58917
ENSMUSG00000028416	Bag1	BCL2-associated athanogene 1 [Source:MGI Symbo	0.58922
ENSMUSG00000092536	Gm20501	predicted gene 20501 [Source:MGI Symbol;Acc:MG	0.58933
ENSMUSG00000025027	Xpnpep1	X-prolyl aminopeptidase (aminopeptidase P) 1, solu	0.58936
ENSMUSG00000097321	1700028E10Rik	RIKEN cDNA 1700028E10 gene [Source:MGI Symbo	0.58954
ENSMUSG00000072855	Gm10430	predicted gene 10430 [Source:MGI Symbol;Acc:MG	0.58964
ENSMUSG00000066637	Ttc32	tetratricopeptide repeat domain 32 [Source:MGI Sy	0.5897

ENSMUSG00000097246	Gm26890	predicted gene, 26890 [Source:MGI Symbol;Acc:MG	0.58975
ENSMUSG00000082186	Gm12403	predicted gene 12403 [Source:MGI Symbol;Acc:MG	0.58988
ENSMUSG00000087050	A030003K02Rik	RIKEN cDNA A030003K02 gene [Source:MGI Symbo	0.58996
ENSMUSG00000032641	Gpr19	G protein-coupled receptor 19 [Source:MGI Symbo	0.58997
ENSMUSG00000030201	Lrp6	low density lipoprotein receptor-related protein 6 [	0.59009
ENSMUSG00000020100	Slc29a3	solute carrier family 29 (nucleoside transporters), n	0.59022
ENSMUSG00000059975	Zfp74	zinc finger protein 74 [Source:MGI Symbol;Acc:MGI	0.59025
ENSMUSG00000020423	Btg2	B cell translocation gene 2, anti-proliferative [Sourc	0.59027
ENSMUSG00000038357	Camp	cathelicidin antimicrobial peptide [Source:MGI Sym	0.59032
ENSMUSG00000094487	Gm21958	predicted gene, 21958 [Source:MGI Symbol;Acc:MG	0.59038
ENSMUSG00000044667	D3Bwg0562e	DNA segment, Chr 3, Brigham & Women's Genetics	0.5904
ENSMUSG00000078370	Gm6316	predicted pseudogene 6316 [Source:MGI Symbol;A	0.59041
ENSMUSG00000029701	Rbm28	RNA binding motif protein 28 [Source:MGI Symbol;	0.59044
ENSMUSG00000043411	Usp48	ubiquitin specific peptidase 48 [Source:MGI Symbo	0.5906
ENSMUSG00000070044	Fam149a	family with sequence similarity 149, member A [Sou	0.59089
ENSMUSG00000090743	Gm17213	predicted gene 17213 [Source:MGI Symbol;Acc:MG	0.5909
ENSMUSG00000048701	Ccdc6	coiled-coil domain containing 6 [Source:MGI Symbo	0.59091
ENSMUSG00000031732	Phlpp2	PH domain and leucine rich repeat protein phospha	0.59107
ENSMUSG00000092577	Gm7393	predicted gene 7393 [Source:MGI Symbol;Acc:MGI:	0.59121
ENSMUSG00000030606	Hapln3	hyaluronan and proteoglycan link protein 3 [Source	0.5913
ENSMUSG00000097736	9530059O14Rik	RIKEN cDNA 9530059O14 gene [Source:MGI Symbo	0.59135
ENSMUSG00000079157	Fam155a	family with sequence similarity 155, member A [Sou	0.59153
ENSMUSG00000034994	Eef2	eukaryotic translation elongation factor 2 [Source:M	0.59158
ENSMUSG00000053574	4930563E22Rik	RIKEN cDNA 4930563E22 gene [Source:MGI Symbo	0.59159
ENSMUSG00000039910	Cited2	Cbp/p300-interacting transactivator, with Glu/Asp-	0.59167
ENSMUSG00000041696	Rasl12	RAS-like, family 12 [Source:MGI Symbol;Acc:MGI:19	0.59174
ENSMUSG00000021478	Drd1a	dopamine receptor D1A [Source:MGI Symbol;Acc:M	0.59185
ENSMUSG00000039989	Cbx4	chromobox 4 [Source:MGI Symbol;Acc:MGI:119598	0.592
ENSMUSG00000003178	Mical3	microtubule associated monooxygenase, calponin a	0.59213
ENSMUSG00000005705	Agrp	agouti related protein [Source:MGI Symbol;Acc:MG	0.59214
ENSMUSG00000097356	Gm26774	predicted gene, 26774 [Source:MGI Symbol;Acc:MG	0.59221
ENSMUSG00000084593	Gm22345	predicted gene, 22345 [Source:MGI Symbol;Acc:MG	0.59244
ENSMUSG00000088623	Gm23031	predicted gene, 23031 [Source:MGI Symbol;Acc:MG	0.59249
ENSMUSG00000086995	Gm13544	predicted gene 13544 [Source:MGI Symbol;Acc:MG	0.59251
ENSMUSG00000026577	Blzf1	basic leucine zipper nuclear factor 1 [Source:MGI Sy	0.59256
ENSMUSG00000006398	Cdc20	cell division cycle 20 [Source:MGI Symbol;Acc:MGI:	0.59259
ENSMUSG00000000627	Sema4f	sema domain, immunoglobulin domain (Ig), TM dom	0.5926
ENSMUSG00000090135	Gm15809	predicted gene 15809 [Source:MGI Symbol;Acc:MG	0.59264
ENSMUSG00000039096	Rsad1	radical S-adenosyl methionine domain containing 1	0.59266
ENSMUSG00000066687	Zbtb16	zinc finger and BTB domain containing 16 [Source:M	0.5928
ENSMUSG00000032212	Sltm	SAFB-like, transcription modulator [Source:MGI Sym	0.59283
ENSMUSG00000026688	Mgst3	microsomal glutathione S-transferase 3 [Source:MG	0.59284
ENSMUSG00000066197	Gpr139	G protein-coupled receptor 139 [Source:MGI Symb	0.59291
ENSMUSG00000016554	Eif3d	eukaryotic translation initiation factor 3, subunit D	0.59292
ENSMUSG00000048652	Samd13	sterile alpha motif domain containing 13 [Source:M	0.59296
ENSMUSG00000024530	Slmo1	slowmo homolog 1 (Drosophila) [Source:MGI Symb	0.59299
ENSMUSG00000029333	Rasgef1b	RasGEF domain family, member 1B [Source:MGI Sy	0.59303

ENSMUSG00000020867	Spata20	spermatogenesis associated 20 [Source:MGI Symbo	0.5931
ENSMUSG00000037991	Rmi2	RMI2, RecQ mediated genome instability 2, homolo	0.59311
ENSMUSG00000026836	Acvr1	activin A receptor, type 1 [Source:MGI Symbol;Acc:	0.59325
ENSMUSG00000048988	Elfn1	leucine rich repeat and fibronectin type III, extracel	0.59328
ENSMUSG00000061601	Pclo	piccolo (presynaptic cytomatrix protein) [Source:M	0.59335
ENSMUSG00000026241	Nppc	natriuretic peptide type C [Source:MGI Symbol;Acc	0.5936
ENSMUSG00000044206	Vsig4	V-set and immunoglobulin domain containing 4 [So	0.59364
ENSMUSG00000055717	Slain1	SLAIN motif family, member 1 [Source:MGI Symbol	0.59371
ENSMUSG00000082078	Gm12098	predicted gene 12098 [Source:MGI Symbol;Acc:MG	0.59371
ENSMUSG00000028394	Pole3	polymerase (DNA directed), epsilon 3 (p17 subunit)	0.59382
ENSMUSG00000044405	Adig	adipogenin [Source:MGI Symbol;Acc:MGI:2675492]	0.59396
ENSMUSG00000083307	AA414768	expressed sequence AA414768 [Source:MGI Symbo	0.59405
ENSMUSG00000087440	4930577N17Rik	RIKEN cDNA 4930577N17 gene [Source:MGI Symbo	0.59421
ENSMUSG00000086389	Gm15998	predicted gene 15998 [Source:MGI Symbol;Acc:MG	0.59423
ENSMUSG00000095385	D630033O11Rik	RIKEN cDNA D630033O11 gene [Source:MGI Symbo	0.59423
ENSMUSG00000040966	Slc22a2	solute carrier family 22 (organic cation transporter)	0.59426
ENSMUSG00000048012	Zfp473	zinc finger protein 473 [Source:MGI Symbol;Acc:M	0.59434
ENSMUSG00000025978	Rftn2	raftlin family member 2 [Source:MGI Symbol;Acc:M	0.59442
ENSMUSG00000047215	Rpl9	ribosomal protein L9 [Source:MGI Symbol;Acc:MGI	0.59448
ENSMUSG00000061130	Ppm1b	protein phosphatase 1B, magnesium dependent, be	0.59451
ENSMUSG00000027387	Zc3h8	zinc finger CCCH type containing 8 [Source:MGI Syn	0.59465
ENSMUSG00000081607	Gm15294	predicted gene 15294 [Source:MGI Symbol;Acc:MG	0.59471
ENSMUSG00000024036	Slc37a1	solute carrier family 37 (glycerol-3-phosphate trans	0.59487
ENSMUSG00000068923	Syt11	synaptotagmin XI [Source:MGI Symbol;Acc:MGI:185	0.59489
ENSMUSG00000000959	Oxa1l	oxidase assembly 1-like [Source:MGI Symbol;Acc:M	0.59496
ENSMUSG00000025927	Tfap2b	transcription factor AP-2 beta [Source:MGI Symbol;	0.59496
ENSMUSG00000058756	Thra	thyroid hormone receptor alpha [Source:MGI Symb	0.59502
ENSMUSG00000053310	Nrgn	neurogranin [Source:MGI Symbol;Acc:MGI:1927184	0.59503
ENSMUSG00000093637	Gm20636	predicted gene 20636 [Source:MGI Symbol;Acc:MG	0.59503
ENSMUSG00000058317	Ube2e2	ubiquitin-conjugating enzyme E2E 2 [Source:MGI Sy	0.59504
ENSMUSG00000097141	Gm10524	predicted gene 10524 [Source:MGI Symbol;Acc:MG	0.59517
ENSMUSG00000010048	Ifrd2	interferon-related developmental regulator 2 [Sour	0.59522
ENSMUSG00000096934	Gm26658	predicted gene, 26658 [Source:MGI Symbol;Acc:M	0.59525
ENSMUSG00000041654	Slc39a11	solute carrier family 39 (metal ion transporter), me	0.59526
ENSMUSG00000026455	Klhl12	kelch-like 12 [Source:MGI Symbol;Acc:MGI:238561	0.59527
ENSMUSG00000037347	Chst7	carbohydrate (N-acetylglucosamino) sulfotransfera	0.59527
ENSMUSG00000026019	Wdr12	WD repeat domain 12 [Source:MGI Symbol;Acc:MG	0.59537
ENSMUSG00000092802	Gm26460	predicted gene, 26460 [Source:MGI Symbol;Acc:M	0.59543
ENSMUSG00000034832	Tet3	tet methylcytosine dioxygenase 3 [Source:MGI Sym	0.59544
ENSMUSG00000035227	Spcs2	signal peptidase complex subunit 2 homolog (S. cer	0.59546
ENSMUSG00000089711	Gm16240	predicted gene 16240 [Source:MGI Symbol;Acc:MG	0.59546
ENSMUSG00000083552	Gm14814	predicted gene 14814 [Source:MGI Symbol;Acc:MG	0.59549
ENSMUSG00000029250	Polr2b	polymerase (RNA) II (DNA directed) polypeptide B [	0.59555
ENSMUSG00000098090	2700099C18Rik	RIKEN cDNA 2700099C18 gene [Source:MGI Symbo	0.59555
ENSMUSG00000082352	Gm13228	predicted gene 13228 [Source:MGI Symbol;Acc:MG	0.59572
ENSMUSG00000040828	Catsperd	catsper channel auxiliary subunit delta [Source:MG	0.59583
ENSMUSG00000080997	Gm11885	predicted gene 11885 [Source:MGI Symbol;Acc:MG	0.59595

ENSMUSG00000015405	Ace2	angiotensin I converting enzyme (peptidyl-dipeptid	0.59602
ENSMUSG00000050963	Kcns2	K+ voltage-gated channel, subfamily S, 2 [Source:M	0.59602
ENSMUSG00000053469	Tg	thyroglobulin [Source:MGI Symbol;Acc:MGI:98733]	0.5961
ENSMUSG00000078317	F8a	factor 8-associated gene A [Source:MGI Symbol;Acc	0.5961
ENSMUSG00000026098	Pms1	postmeiotic segregation increased 1 (S. cerevisiae)	0.59615
ENSMUSG00000054226	Tprkb	Tp53rk binding protein [Source:MGI Symbol;Acc:M	0.59622
ENSMUSG00000083412	Gm12268	predicted gene 12268 [Source:MGI Symbol;Acc:MG	0.59637
ENSMUSG00000055424	Gm9970	predicted gene 9970 [Source:MGI Symbol;Acc:MGI:	0.59638
ENSMUSG00000042279	H1foo	H1 histone family, member O, oocyte-specific [Sour	0.59641
ENSMUSG00000050428	Fbxo46	F-box protein 46 [Source:MGI Symbol;Acc:MGI:244	0.59648
ENSMUSG00000046080	Clec9a	C-type lectin domain family 9, member a [Source:M	0.59657
ENSMUSG00000019988	Nedd1	neural precursor cell expressed, developmentally d	0.59658
ENSMUSG00000037013	Ss18	synovial sarcoma translocation, Chromosome 18 [S	0.59658
ENSMUSG00000085693	Gm13371	predicted gene 13371 [Source:MGI Symbol;Acc:MG	0.5966
ENSMUSG00000039321	Uts2r	urotensin 2 receptor [Source:MGI Symbol;Acc:MGI:	0.59663
ENSMUSG00000028988	Ctnnbip1	catenin beta interacting protein 1 [Source:MGI Sym	0.59667
ENSMUSG00000092915	Gm24083	predicted gene, 24083 [Source:MGI Symbol;Acc:M(	0.59673
ENSMUSG00000087248	Gm16120	predicted gene 16120 [Source:MGI Symbol;Acc:MG	0.59675
ENSMUSG00000030257	Srgap3	SLIT-ROBO Rho GTPase activating protein 3 [Source	0.59677
ENSMUSG00000087623	Gm12404	predicted gene 12404 [Source:MGI Symbol;Acc:MG	0.59685
ENSMUSG00000041353	Tmem29	transmembrane protein 29 [Source:MGI Symbol;Ac	0.59702
ENSMUSG00000030796	Tead2	TEA domain family member 2 [Source:MGI Symbol;	0.59705
ENSMUSG00000022332	Khdrbs3	KH domain containing, RNA binding, signal transdu	0.59715
ENSMUSG00000027380	Acoxl	acyl-Coenzyme A oxidase-like [Source:MGI Symbol;	0.59717
ENSMUSG00000027570	Col9a3	collagen, type IX, alpha 3 [Source:MGI Symbol;Acc:	0.59728
ENSMUSG00000030102	Itpr1	inositol 1,4,5-trisphosphate receptor 1 [Source:MG	0.59737
ENSMUSG00000023791	Pigx	phosphatidylinositol glycan anchor biosynthesis, cla	0.59738
ENSMUSG00000006191	Cdkal1	CDK5 regulatory subunit associated protein 1-like 1	0.59739
ENSMUSG00000034528	Hsd17b13	hydroxysteroid (17-beta) dehydrogenase 13 [Sourc	0.59741
ENSMUSG00000049532	Sall2	sal-like 2 (Drosophila) [Source:MGI Symbol;Acc:MG	0.59756
ENSMUSG00000017740	Slc12a5	solute carrier family 12, member 5 [Source:MGI Syr	0.59758
ENSMUSG00000084413	Gm12017	predicted gene 12017 [Source:MGI Symbol;Acc:MG	0.59763
ENSMUSG00000058186	Gm13242	predicted gene 13242 [Source:MGI Symbol;Acc:MG	0.59766
ENSMUSG00000069712	4930444G20Rik	RIKEN cDNA 4930444G20 gene [Source:MGI Symbo	0.59766
ENSMUSG00000024011	Pi16	peptidase inhibitor 16 [Source:MGI Symbol;Acc:MG	0.59769
ENSMUSG00000032661	Oas3	2'-5' oligoadenylate synthetase 3 [Source:MGI Sym	0.59781
ENSMUSG00000087484	2900089D17Rik	RIKEN cDNA 2900089D17 gene [Source:MGI Symbo	0.59787
ENSMUSG00000032812	Arap1	ArfGAP with RhoGAP domain, ankyrin repeat and P	0.59789
ENSMUSG00000053799	Exoc6	exocyst complex component 6 [Source:MGI Symbo	0.5979
ENSMUSG00000073535	Gm5532	predicted gene 5532 [Source:MGI Symbol;Acc:MGI:	0.59791
ENSMUSG00000055775	Myh8	myosin, heavy polypeptide 8, skeletal muscle, perin	0.59797
ENSMUSG00000091993	B930036N10Rik	RIKEN cDNA B930036N10 gene [Source:MGI Symbo	0.59803
ENSMUSG00000059586	Nsmce2	non-SMC element 2 homolog (MMS21, S. cerevisia	0.59807
ENSMUSG00000042303	Sgsm3	small G protein signaling modulator 3 [Source:MGI	0.59823
ENSMUSG00000003604	Aven	apoptosis, caspase activation inhibitor [Source:MGI	0.59825
ENSMUSG00000028864	Hgf	hepatocyte growth factor [Source:MGI Symbol;Acc:	0.59835
ENSMUSG00000084106	Gm6136	predicted pseudogene 6136 [Source:MGI Symbol;A	0.59843

ENSMUSG00000024254	Abcg8	ATP-binding cassette, sub-family G (WHITE), memb	0.59844
ENSMUSG00000033047	Eif3l	eukaryotic translation initiation factor 3, subunit L	0.59856
ENSMUSG00000058420	Syt17	synaptotagmin XVII [Source:MGI Symbol;Acc:MGI:1	0.59868
ENSMUSG00000090674	Gm17082	predicted gene 17082 [Source:MGI Symbol;Acc:MG	0.59876
ENSMUSG00000073700	Klhl21	kelch-like 21 [Source:MGI Symbol;Acc:MGI:191928	0.59884
ENSMUSG00000071550	Wdr52	WD repeat domain 52 [Source:MGI Symbol;Acc:MG	0.59906
ENSMUSG00000083332	Gm7599	predicted gene 7599 [Source:MGI Symbol;Acc:MGI	0.59916
ENSMUSG00000052551	Adarb2	adenosine deaminase, RNA-specific, B2 [Source:MG	0.59919
ENSMUSG00000026809	1700026L06Rik	RIKEN cDNA 1700026L06 gene [Source:MGI Symbo	0.5992
ENSMUSG00000022303	Dcstamp	dentrocyte expressed seven transmembrane protei	0.59923
ENSMUSG00000001622	Csn3	casein kappa [Source:MGI Symbol;Acc:MGI:107461	0.59925
ENSMUSG00000038668	Lpar1	lysophosphatidic acid receptor 1 [Source:MGI Symb	0.59932
ENSMUSG00000041718	Alg13	asparagine-linked glycosylation 13 [Source:MGI Syr	0.59937
ENSMUSG00000067285	Gm16223	predicted gene 16223 [Source:MGI Symbol;Acc:MG	0.59944
ENSMUSG00000037697	Ddhd1	DDHD domain containing 1 [Source:MGI Symbol;Ac	0.5995
ENSMUSG00000065818	Snord35a	small nucleolar RNA, C/D box 35A [Source:MGI Sym	0.59953
ENSMUSG00000029526	1700123K08Rik	RIKEN cDNA 1700123K08 gene [Source:MGI Symbo	0.59956
ENSMUSG00000015013	Trappc2l	trafficking protein particle complex 2-like [Source:N	0.59957
ENSMUSG00000019846	Lama4	laminin, alpha 4 [Source:MGI Symbol;Acc:MGI:109	0.59957
ENSMUSG00000059890	Ube4a	ubiquitination factor E4A, UFD2 homolog (S. cerevis	0.59967
ENSMUSG00000022528	Hes1	hairy and enhancer of split 1 (Drosophila) [Source:N	0.59968
ENSMUSG00000040795	Iqcc	IQ motif containing C [Source:MGI Symbol;Acc:MGI	0.59984
ENSMUSG00000097404	Gm10814	predicted gene 10814 [Source:MGI Symbol;Acc:MG	0.59992
ENSMUSG00000085304	Gm12802	predicted gene 12802 [Source:MGI Symbol;Acc:MG	0.59997
ENSMUSG00000022372	Sla	src-like adaptor [Source:MGI Symbol;Acc:MGI:1042	0.60011
ENSMUSG00000087288	Gm13061	predicted gene 13061 [Source:MGI Symbol;Acc:MG	0.60016
ENSMUSG00000052850	Tas2r137	taste receptor, type 2, member 137 [Source:MGI Sy	0.60019
ENSMUSG00000085021	Gm12051	predicted gene 12051 [Source:MGI Symbol;Acc:MG	0.60021
ENSMUSG00000031154	Otud5	OTU domain containing 5 [Source:MGI Symbol;Acc:	0.60026
ENSMUSG00000091530	Cldn20	claudin 20 [Source:MGI Symbol;Acc:MGI:3646757]	0.60034
ENSMUSG00000037784	Dzip1l	DAZ interacting protein 1-like [Source:MGI Symbol;	0.60035
ENSMUSG00000091423	Gm17509	predicted gene, 17509 [Source:MGI Symbol;Acc:M	0.60048
ENSMUSG00000090083	Rnf8	ring finger protein 8 [Source:MGI Symbol;Acc:MGI:	0.6005
ENSMUSG00000022013	Dnajc15	DnaJ (Hsp40) homolog, subfamily C, member 15 [S	0.60051
ENSMUSG00000091577	Gm6211	predicted gene 6211 [Source:MGI Symbol;Acc:MGI	0.60054
ENSMUSG00000035086	Becn1	beclin 1, autophagy related [Source:MGI Symbol;Ac	0.60055
ENSMUSG00000087063	Gm15857	predicted gene 15857 [Source:MGI Symbol;Acc:MG	0.60074
ENSMUSG00000074671	Tspyl3	TSPY-like 3 [Source:MGI Symbol;Acc:MGI:2139328]	0.60081
ENSMUSG00000037605	Lphn3	latrophilin 3 [Source:MGI Symbol;Acc:MGI:244195	0.6009
ENSMUSG00000048292	Olfr1417	olfactory receptor 1417 [Source:MGI Symbol;Acc:M	0.601
ENSMUSG00000049571	9330101J02Rik	RIKEN cDNA 9330101J02 gene [Source:MGI Symbol	0.60123
ENSMUSG00000038665	Dgki	diacylglycerol kinase, iota [Source:MGI Symbol;Acc:	0.60138
ENSMUSG00000037884	1700017G19Rik	RIKEN cDNA 1700017G19 gene [Source:MGI Symbo	0.60144
ENSMUSG00000039632	Ccdc151	coiled-coil domain containing 151 [Source:MGI Sym	0.60145
ENSMUSG00000035934	Pknox2	Pbx/knotted 1 homeobox 2 [Source:MGI Symbol;Ac	0.60146
ENSMUSG00000050555	Hyls1	hydrolethalus syndrome 1 [Source:MGI Symbol;Acc	0.60146
ENSMUSG00000091562	Gm9581	predicted gene 9581 [Source:MGI Symbol;Acc:MGI	0.60148

ENSMUSG00000032725	Folr2	folate receptor 2 (fetal) [Source:MGI Symbol;Acc:M	0.60155
ENSMUSG00000045294	Insig1	insulin induced gene 1 [Source:MGI Symbol;Acc:MG	0.60158
ENSMUSG00000056753	C330011M18Rik	RIKEN cDNA C330011M18 gene [Source:MGI Symbo	0.60167
ENSMUSG00000056121	Fez2	fasciculation and elongation protein zeta 2 (zygin II	0.60173
ENSMUSG00000020472	Zkscan17	zinc finger with KRAB and SCAN domains 17 [Source	0.60174
ENSMUSG00000060559	Gm10083	predicted gene 10083 [Source:MGI Symbol;Acc:MG	0.60176
ENSMUSG00000061099	Gapdhs	glyceraldehyde-3-phosphate dehydrogenase, sperm	0.60183
ENSMUSG00000014245	Pigl	phosphatidylinositol glycan anchor biosynthesis, cla	0.60187
ENSMUSG00000046008	Pnlip	pancreatic lipase [Source:MGI Symbol;Acc:MGI:977	0.6019
ENSMUSG00000087037	4930432L08Rik	RIKEN cDNA 4930432L08 gene [Source:MGI Symbol	0.60193
ENSMUSG00000039680	Mrps6	mitochondrial ribosomal protein S6 [Source:MGI Sy	0.60199
ENSMUSG00000044042	Fmn1	formin 1 [Source:MGI Symbol;Acc:MGI:101815]	0.60219
ENSMUSG00000022490	Ppp1r1a	protein phosphatase 1, regulatory (inhibitor) subun	0.60222
ENSMUSG00000043286	Pnpla1	patatin-like phospholipase domain containing 1 [So	0.60235
ENSMUSG00000046808	Atp10d	ATPase, class V, type 10D [Source:MGI Symbol;Acc:	0.60251
ENSMUSG00000039201	Tbc1d25	TBC1 domain family, member 25 [Source:MGI Symb	0.60254
ENSMUSG00000033610	Pank1	pantothenate kinase 1 [Source:MGI Symbol;Acc:MG	0.60257
ENSMUSG00000020312	Shc2	SHC (Src homology 2 domain containing) transform	0.60264
ENSMUSG00000054321	Taf4b	TAF4B RNA polymerase II, TATA box binding protein	0.60264
ENSMUSG00000038888	Ctu1	cytosolic thiouridylase subunit 1 homolog (S. pomb	0.60267
ENSMUSG00000086308	G630016G05Rik	RIKEN cDNA G630016G05 gene [Source:MGI Symbo	0.60273
ENSMUSG00000045662	Henmt1	HEN1 methyltransferase homolog 1 (Arabidopsis) [S	0.60284
ENSMUSG00000042043	Tbca	tubulin cofactor A [Source:MGI Symbol;Acc:MGI:10	0.60285
ENSMUSG00000047363	Cstad	CSA-conditional, T cell activation-dependent protei	0.60286
ENSMUSG00000039901	9130011E15Rik	RIKEN cDNA 9130011E15 gene [Source:MGI Symbo	0.60292
ENSMUSG00000031393	Mecp2	methyl CpG binding protein 2 [Source:MGI Symbol;	0.60294
ENSMUSG00000028690	Mmachc	methylmalonic aciduria cblC type, with homocystin	0.60295
ENSMUSG00000008090	Fgfrl1	fibroblast growth factor receptor-like 1 [Source:MG	0.60304
ENSMUSG00000029754	Dlx6	distal-less homeobox 6 [Source:MGI Symbol;Acc:M	0.60312
ENSMUSG00000039530	Tusc3	tumor suppressor candidate 3 [Source:MGI Symbol	0.60314
ENSMUSG00000020334	Slc22a4	solute carrier family 22 (organic cation transporter)	0.60326
ENSMUSG00000024561	Mbd1	methyl-CpG binding domain protein 1 [Source:MGI	0.60327
ENSMUSG00000070891	Gm12689	predicted gene 12689 [Source:MGI Symbol;Acc:MG	0.60327
ENSMUSG00000087550	Gm14343	predicted gene 14343 [Source:MGI Symbol;Acc:MG	0.60329
ENSMUSG00000030244	Gys2	glycogen synthase 2 [Source:MGI Symbol;Acc:MGI:	0.60376
ENSMUSG00000097245	Gm5421	predicted gene 5421 [Source:MGI Symbol;Acc:MGI:	0.60377
ENSMUSG00000049699	Ucn2	urocortin 2 [Source:MGI Symbol;Acc:MGI:2176375]	0.60378
ENSMUSG00000015619	Gata3	GATA binding protein 3 [Source:MGI Symbol;Acc:M	0.60385
ENSMUSG00000046388	Gm9806	predicted gene 9806 [Source:MGI Symbol;Acc:MGI:	0.60407
ENSMUSG00000073607	Gm10548	predicted gene 10548 [Source:MGI Symbol;Acc:MG	0.6041
ENSMUSG00000030772	Dkk3	dickkopf homolog 3 (Xenopus laevis) [Source:MGI S	0.60413
ENSMUSG00000046085	4931422A03Rik	RIKEN cDNA 4931422A03 gene [Source:MGI Symbo	0.60415
ENSMUSG00000087549	Gm13427	predicted gene 13427 [Source:MGI Symbol;Acc:MG	0.60422
ENSMUSG00000024151	Msh2	mutS homolog 2 (E. coli) [Source:MGI Symbol;Acc:M	0.60427
ENSMUSG00000062472	Gm4945	predicted gene 4945 [Source:MGI Symbol;Acc:MGI:	0.60433
ENSMUSG00000085588	3110004A20Rik	RIKEN cDNA 3110004A20 gene [Source:MGI Symbo	0.60437
ENSMUSG00000070880	Gad1	glutamate decarboxylase 1 [Source:MGI Symbol;Ac	0.60451

ENSMUSG00000015396	Cd83	CD83 antigen [Source:MGI Symbol;Acc:MGI:132831	0.60474
ENSMUSG00000054391	4930517O19Rik	RIKEN cDNA 4930517O19 gene [Source:MGI Symbo	0.60475
ENSMUSG00000024164	C3	complement component 3 [Source:MGI Symbol;Ac	0.6048
ENSMUSG00000017716	Birc5	baculoviral IAP repeat-containing 5 [Source:MGI Sy	0.60484
ENSMUSG00000018126	Baiap2l2	BAI1-associated protein 2-like 2 [Source:MGI Symb	0.60487
ENSMUSG00000002625	Akap8l	A kinase (PRKA) anchor protein 8-like [Source:MGI S	0.60493
ENSMUSG00000046667	Rbm12b1	RNA binding motif protein 12 B1 [Source:MGI Symb	0.60497
ENSMUSG00000083788	Gm11499	predicted gene 11499 [Source:MGI Symbol;Acc:MG	0.60497
ENSMUSG00000028581	Laptm5	lysosomal-associated protein transmembrane 5 [So	0.60504
ENSMUSG00000046463	5930403N24Rik	RIKEN cDNA 5930403N24 gene [Source:MGI Symbo	0.60505
ENSMUSG00000052423	B4galt3	UDP-Gal:betaGlcNAc beta 1,4-galactosyltransferase	0.60506
ENSMUSG00000024456	Diap1	diaphanous homolog 1 (Drosophila) [Source:MGI Sy	0.60513
ENSMUSG00000097575	Gm26796	predicted gene, 26796 [Source:MGI Symbol;Acc:MG	0.60516
ENSMUSG00000008301	Phax	phosphorylated adaptor for RNA export [Source:MG	0.60527
ENSMUSG00000048758	Rpl29	ribosomal protein L29 [Source:MGI Symbol;Acc:MG	0.60527
ENSMUSG00000022184	Fbxo4	F-box protein 4 [Source:MGI Symbol;Acc:MGI:2146	0.60533
ENSMUSG00000002031	Ift46	intraflagellar transport 46 [Source:MGI Symbol;Acc	0.60536
ENSMUSG00000058298	Mcm9	minichromosome maintenance complex componen	0.60561
ENSMUSG00000090449	Gm17076	predicted gene 17076 [Source:MGI Symbol;Acc:MG	0.60563
ENSMUSG00000027533	Fabp5	fatty acid binding protein 5, epidermal [Source:MG	0.60564
ENSMUSG00000061411	8430427H17Rik	RIKEN cDNA 8430427H17 gene [Source:MGI Symbo	0.6057
ENSMUSG00000000028	Cdc45	cell division cycle 45 [Source:MGI Symbol;Acc:MGI:	0.60586
ENSMUSG00000010025	Aldh3a2	aldehyde dehydrogenase family 3, subfamily A2 [So	0.60586
ENSMUSG00000039458	Mtmr12	myotubularin related protein 12 [Source:MGI Symb	0.60591
ENSMUSG00000032892	Rangrf	RAN guanine nucleotide release factor [Source:MG	0.60593
ENSMUSG00000075595	Zfp652	zinc finger protein 652 [Source:MGI Symbol;Acc:MG	0.60597
ENSMUSG00000016541	Atxn10	ataxin 10 [Source:MGI Symbol;Acc:MGI:1859293]	0.60598
ENSMUSG00000079109	Pms2	postmeiotic segregation increased 2 (S. cerevisiae)	0.60604
ENSMUSG00000030897	Cnga4	cyclic nucleotide gated channel alpha 4 [Source:MG	0.60605
ENSMUSG00000029556	Hnf1a	HNF1 homeobox A [Source:MGI Symbol;Acc:MGI:9	0.60626
ENSMUSG00000010358	Ifi35	interferon-induced protein 35 [Source:MGI Symbol	0.60635
ENSMUSG00000031227	Magee1	melanoma antigen, family E, 1 [Source:MGI Symbol	0.60635
ENSMUSG00000034422	Parp14	poly (ADP-ribose) polymerase family, member 14 [S	0.60637
ENSMUSG00000021337	Scgn	secretagogin, EF-hand calcium binding protein [Sou	0.60639
ENSMUSG00000049550	Clip1	CAP-GLY domain containing linker protein 1 [Source	0.60651
ENSMUSG00000020680	Taf15	TAF15 RNA polymerase II, TATA box binding protein	0.60657
ENSMUSG00000023055	Calcoco1	calcium binding and coiled coil domain 1 [Source:M	0.60658
ENSMUSG00000015714	Cers2	ceramide synthase 2 [Source:MGI Symbol;Acc:MGI:	0.60665
ENSMUSG00000051844	B230319C09Rik	RIKEN cDNA B230319C09 gene [Source:MGI Symbo	0.60669
ENSMUSG00000064648	Gm23303	predicted gene, 23303 [Source:MGI Symbol;Acc:MG	0.60675
ENSMUSG00000085655	Gm15952	predicted gene 15952 [Source:MGI Symbol;Acc:MG	0.60679
ENSMUSG00000090257	Gm4524	predicted gene 4524 [Source:MGI Symbol;Acc:MGI:	0.60684
ENSMUSG00000028649	Macf1	microtubule-actin crosslinking factor 1 [Source:MG	0.60695
ENSMUSG00000022901	Cd86	CD86 antigen [Source:MGI Symbol;Acc:MGI:101773	0.60698
ENSMUSG00000089989	Flt3l	FMS-like tyrosine kinase 3 ligand [Source:MGI Symb	0.607
ENSMUSG00000040483	Xaf1	XIAP associated factor 1 [Source:MGI Symbol;Acc:M	0.60704
ENSMUSG00000025505	Tmem80	transmembrane protein 80 [Source:MGI Symbol;Ac	0.60709

ENSMUSG00000038618	Rassf7	Ras association (RalGDS/AF-6) domain family (N-ter	0.60709
ENSMUSG00000047686	Zcchc5	zinc finger, CCHC domain containing 5 [Source:MGI	0.60712
ENSMUSG00000086587	Gm11837	predicted gene 11837 [Source:MGI Symbol;Acc:MG	0.60721
ENSMUSG00000040430	Pitpnc1	phosphatidylinositol transfer protein, cytoplasmic 1	0.60724
ENSMUSG00000023266	Frs3	fibroblast growth factor receptor substrate 3 [Sourc	0.60732
ENSMUSG00000030870	Ubfd1	ubiquitin family domain containing 1 [Source:MGI S	0.60744
ENSMUSG00000031450	Grk1	G protein-coupled receptor kinase 1 [Source:MGI S	0.60763
ENSMUSG00000075324	Fign	fidgetin [Source:MGI Symbol;Acc:MGI:1890647]	0.60767
ENSMUSG00000043279	Trim56	tripartite motif-containing 56 [Source:MGI Symbol;	0.60769
ENSMUSG00000049307	Fut4	fucosyltransferase 4 [Source:MGI Symbol;Acc:MGI:	0.60771
ENSMUSG00000086360	Gm16214	predicted gene 16214 [Source:MGI Symbol;Acc:MG	0.60771
ENSMUSG00000040652	Oaz2	ornithine decarboxylase antizyme 2 [Source:MGI Sy	0.60775
ENSMUSG00000062074	Wisp3	WNT1 inducible signaling pathway protein 3 [Sourc	0.60787
ENSMUSG00000089791	Gm3555	predicted pseudogene 3555 [Source:MGI Symbol;A	0.60787
ENSMUSG00000039159	Ube2h	ubiquitin-conjugating enzyme E2H [Source:MGI Syr	0.60796
ENSMUSG00000021482	Aaed1	AhpC/TSA antioxidant enzyme domain containing 1	0.60799
ENSMUSG00000073586	Gm10543	predicted gene 10543 [Source:MGI Symbol;Acc:MG	0.608
ENSMUSG00000046480	Scn4b	sodium channel, type IV, beta [Source:MGI Symbol;	0.60811
ENSMUSG00000064315	Rpl21-ps10	ribosomal protein L21, pseudogene 10 [Source:MG	0.6082
ENSMUSG00000037405	Icam1	intercellular adhesion molecule 1 [Source:MGI Sym	0.60821
ENSMUSG00000020656	Grhl1	grainyhead-like 1 (Drosophila) [Source:MGI Symbol	0.60823
ENSMUSG00000023828	Slc22a3	solute carrier family 22 (organic cation transporter)	0.60823
ENSMUSG00000084769	Gm12061	predicted gene 12061 [Source:MGI Symbol;Acc:MG	0.60823
ENSMUSG00000086716	Gm11629	predicted gene 11629 [Source:MGI Symbol;Acc:MG	0.60823
ENSMUSG00000090146	4930405H06Rik	RIKEN cDNA 4930405H06 gene [Source:MGI Symbo	0.60825
ENSMUSG00000085327	Gm16104	predicted gene 16104 [Source:MGI Symbol;Acc:MG	0.60829
ENSMUSG00000044052	Ccr10	chemokine (C-C motif) receptor 10 [Source:MGI Syr	0.60836
ENSMUSG00000092515	C87198	expressed sequence C87198 [Source:MGI Symbol;A	0.60848
ENSMUSG00000083504	Gm12450	predicted gene 12450 [Source:MGI Symbol;Acc:MG	0.60862
ENSMUSG00000027483	Bpifa1	BPI fold containing family A, member 1 [Source:MG	0.60864
ENSMUSG00000040354	Mars	methionine-tRNA synthetase [Source:MGI Symbol;A	0.60865
ENSMUSG00000056185	Snx32	sorting nexin 32 [Source:MGI Symbol;Acc:MGI:2444	0.60868
ENSMUSG00000006906	Stambp	STAM binding protein [Source:MGI Symbol;Acc:MG	0.60875
ENSMUSG00000005682	Pan2	PAN2 polyA specific ribonuclease subunit homolog	0.60878
ENSMUSG00000020630	Rnaseh1	ribonuclease H1 [Source:MGI Symbol;Acc:MGI:1335	0.60888
ENSMUSG00000034981	Parm1	prostate androgen-regulated mucin-like protein 1 [	0.60892
ENSMUSG00000068877	Selenbp2	selenium binding protein 2 [Source:MGI Symbol;Ac	0.60913
ENSMUSG00000030562	Nox4	NADPH oxidase 4 [Source:MGI Symbol;Acc:MGI:135	0.60923
ENSMUSG00000096917	2500002B13Rik	RIKEN cDNA 2500002B13 gene [Source:MGI Symbo	0.60927
ENSMUSG00000027634	Ndrg3	N-myc downstream regulated gene 3 [Source:MGI S	0.60939
ENSMUSG00000081597	Gm14506	predicted gene 14506 [Source:MGI Symbol;Acc:MG	0.6094
ENSMUSG00000037730	Mynn	myoneurin [Source:MGI Symbol;Acc:MGI:1931415]	0.60942
ENSMUSG00000065990	Aurkaip1	aurora kinase A interacting protein 1 [Source:MGI S	0.60943
ENSMUSG00000036198	Arhgap36	Rho GTPase activating protein 36 [Source:MGI Sym	0.60962
ENSMUSG00000026803	Ttf1	transcription termination factor, RNA polymerase I	0.60974
ENSMUSG00000022400	Rbx1	ring-box 1 [Source:MGI Symbol;Acc:MGI:1891829]	0.60981
ENSMUSG00000042396	Rbm7	RNA binding motif protein 7 [Source:MGI Symbol;A	0.60995

ENSMUSG00000020133	2310011J03Rik	RIKEN cDNA 2310011J03 gene [Source:MGI Symbol	0.60996
ENSMUSG00000037935	Smarce1	SWI/SNF related, matrix associated, actin depender	0.60999
ENSMUSG00000021725	Parp8	poly (ADP-ribose) polymerase family, member 8 [Sc	0.61
ENSMUSG00000060376	Bckdha	branched chain ketoacid dehydrogenase E1, alpha	0.61
ENSMUSG00000048916	Gm5540	predicted pseudogene 5540 [Source:MGI Symbol;A	0.61001
ENSMUSG00000054715	Zscan22	zinc finger and SCAN domain containing 22 [Source	0.61007
ENSMUSG00000040424	Hipk4	homeodomain interacting protein kinase 4 [Source	0.61009
ENSMUSG00000046456	Tmem150b	transmembrane protein 150B [Source:MGI Symbol;	0.61022
ENSMUSG00000021290	2010107E04Rik	RIKEN cDNA 2010107E04 gene [Source:MGI Symbo	0.61032
ENSMUSG00000037440	Vnn1	vanin 1 [Source:MGI Symbol;Acc:MGI:108395]	0.61049
ENSMUSG00000057541	Pus7	pseudouridylate synthase 7 homolog (S. cerevisiae)	0.61051
ENSMUSG00000002341	Ncan	neurocan [Source:MGI Symbol;Acc:MGI:104694]	0.61053
ENSMUSG00000059751	Rps3a3	ribosomal protein S3A3 [Source:MGI Symbol;Acc:M	0.61065
ENSMUSG00000026589	Sec16b	SEC16 homolog B (S. cerevisiae) [Source:MGI Symb	0.61069
ENSMUSG00000037336	Mfsd2b	major facilitator superfamily domain containing 2B	0.61082
ENSMUSG00000032724	Abtb2	ankyrin repeat and BTB (POZ) domain containing 2	0.61083
ENSMUSG00000074476	Spc24	SPC24, NDC80 kinetochore complex component, ho	0.61094
ENSMUSG00000072664	Ugt3a1	UDP glycosyltransferases 3 family, polypeptide A1 [	0.611
ENSMUSG00000012819	Cdh23	cadherin 23 (otocadherin) [Source:MGI Symbol;Acc	0.61103
ENSMUSG00000086693	A730081D07Rik	RIKEN cDNA A730081D07 gene [Source:MGI Symbo	0.61111
ENSMUSG00000006587	Snai3	snail homolog 3 (Drosophila) [Source:MGI Symbol;	0.61115
ENSMUSG00000032383	Ppib	peptidylprolyl isomerase B [Source:MGI Symbol;Acc	0.61124
ENSMUSG00000097523	1700022N22Rik	RIKEN cDNA 1700022N22 gene [Source:MGI Symbo	0.61124
ENSMUSG00000041560	Gltscr2	glioma tumor suppressor candidate region gene 2 [	0.61129
ENSMUSG00000097273	C630004M23Rik	RIKEN cDNA C630004M23 gene [Source:MGI Symb	0.61129
ENSMUSG00000077575	Gm24888	predicted gene, 24888 [Source:MGI Symbol;Acc:M	0.61132
ENSMUSG00000038463	Olfml2b	olfactomedin-like 2B [Source:MGI Symbol;Acc:MGI	0.61147
ENSMUSG00000094486	Gm23268	predicted gene, 23268 [Source:MGI Symbol;Acc:M	0.61149
ENSMUSG00000043418	Lrit2	leucine-rich repeat, immunoglobulin-like and trans	0.61162
ENSMUSG00000087574	C030037D09Rik	RIKEN cDNA C030037D09 gene [Source:MGI Symbo	0.61172
ENSMUSG00000032440	Tgfbr2	transforming growth factor, beta receptor II [Sourc	0.61178
ENSMUSG00000036737	Oxsr1	oxidative-stress responsive 1 [Source:MGI Symbol;	0.6118
ENSMUSG00000023106	Denr	density-regulated protein [Source:MGI Symbol;Acc	0.61196
ENSMUSG00000030034	Ino80b	INO80 complex subunit B [Source:MGI Symbol;Acc:	0.61204
ENSMUSG00000020532	Acaca	acetyl-Coenzyme A carboxylase alpha [Source:MGI	0.61207
ENSMUSG00000076437	2700094K13Rik	RIKEN cDNA 2700094K13 gene [Source:MGI Symbo	0.61217
ENSMUSG00000038527	C1rl	complement component 1, r subcomponent-like [S	0.61219
ENSMUSG00000085153	B230314M03Rik	RIKEN cDNA B230314M03 gene [Source:MGI Symb	0.61225
ENSMUSG00000081730	Gm13785	predicted gene 13785 [Source:MGI Symbol;Acc:MG	0.61228
ENSMUSG00000074127	Cmtm2a	CKLF-like MARVEL transmembrane domain contain	0.61231
ENSMUSG00000032528	Vipr1	vasoactive intestinal peptide receptor 1 [Source:M	0.61239
ENSMUSG00000021572	Cep72	centrosomal protein 72 [Source:MGI Symbol;Acc:M	0.6124
ENSMUSG00000022821	Hgd	homogentisate 1, 2-dioxygenase [Source:MGI Symb	0.6124
ENSMUSG00000037437	Adam32	a disintegrin and metallopeptidase domain 32 [Sou	0.6124
ENSMUSG00000026923	Notch1	notch 1 [Source:MGI Symbol;Acc:MGI:97363]	0.61254
ENSMUSG00000056288	Gm11961	predicted gene 11961 [Source:MGI Symbol;Acc:MG	0.61264
ENSMUSG00000079179	1700012B15Rik	RIKEN cDNA 1700012B15 gene [Source:MGI Symbo	0.6127

ENSMUSG00000028661	Epha8	Eph receptor A8 [Source:MGI Symbol;Acc:MGI:109	0.61278
ENSMUSG00000060044	Tmem26	transmembrane protein 26 [Source:MGI Symbol;Ac	0.61285
ENSMUSG00000085897	Gm11604	predicted gene 11604 [Source:MGI Symbol;Acc:MG	0.61319
ENSMUSG00000039047	Pigk	phosphatidylinositol glycan anchor biosynthesis, cla	0.61329
ENSMUSG00000031443	F7	coagulation factor VII [Source:MGI Symbol;Acc:MG	0.61333
ENSMUSG00000054206	Gzmm	granzyme M (lymphocyte met-ase 1) [Source:MGI S	0.61354
ENSMUSG00000029752	Asns	asparagine synthetase [Source:MGI Symbol;Acc:MG	0.6136
ENSMUSG00000033237	Arid2	AT rich interactive domain 2 (ARID, RFX-like) [Sourc	0.6136
ENSMUSG00000026669	Mcm10	minichromosome maintenance deficient 10 (S. cere	0.61363
ENSMUSG00000031818	Cox4i1	cytochrome c oxidase subunit IV isoform 1 [Source:	0.61387
ENSMUSG00000029417	Cxcl9	chemokine (C-X-C motif) ligand 9 [Source:MGI Sym	0.61396
ENSMUSG00000090115	Usp49	ubiquitin specific peptidase 49 [Source:MGI Symbo	0.61396
ENSMUSG00000066278	Vps37b	vacuolar protein sorting 37B (yeast) [Source:MGI Sy	0.6142
ENSMUSG00000084876	Gm14965	predicted gene 14965 [Source:MGI Symbol;Acc:MG	0.61422
ENSMUSG00000040669	Phc1	polyhomeotic-like 1 (Drosophila) [Source:MGI Symb	0.61425
ENSMUSG00000002455	Prpf6	PRP6 pre-mRNA splicing factor 6 homolog (yeast) [S	0.61426
ENSMUSG00000026192	Atic	5-aminoimidazole-4-carboxamide ribonucleotide fo	0.61429
ENSMUSG00000085440	D330022K07Rik	RIKEN cDNA D330022K07 gene [Source:MGI Symbo	0.61438
ENSMUSG00000032376	Usp3	ubiquitin specific peptidase 3 [Source:MGI Symbol;	0.61452
ENSMUSG00000034226	Rhov	ras homolog gene family, member V [Source:MGI S	0.61453
ENSMUSG00000035121	Neil2	nei like 2 (E. coli) [Source:MGI Symbol;Acc:MGI:268	0.61466
ENSMUSG00000020823	Sec14l1	SEC14-like 1 (S. cerevisiae) [Source:MGI Symbol;Acc	0.61487
ENSMUSG00000039208	Metrnl	meteorin, glial cell differentiation regulator-like [So	0.61487
ENSMUSG00000031529	Tnks	tankyrase, TRF1-interacting ankyrin-related ADP-rib	0.61494
ENSMUSG00000060224	Pyroxd2	pyridine nucleotide-disulphide oxidoreductase dom	0.61495
ENSMUSG00000029428	Stx2	syntaxin 2 [Source:MGI Symbol;Acc:MGI:108059]	0.61496
ENSMUSG00000027463	Slc52a3	solute carrier protein family 52, member 3 [Source:	0.61507
ENSMUSG00000074218	Cox7a1	cytochrome c oxidase subunit VIIa 1 [Source:MGI Sy	0.61507
ENSMUSG00000019996	Map7	microtubule-associated protein 7 [Source:MGI Sym	0.61511
ENSMUSG00000020891	Alox8	arachidonate 8-lipoxygenase [Source:MGI Symbol;A	0.61516
ENSMUSG00000061894	Zscan20	zinc finger and SCAN domains 20 [Source:MGI Symb	0.61517
ENSMUSG00000039016	Timm8b	translocase of inner mitochondrial membrane 8B [S	0.61522
ENSMUSG00000000378	Ccm2	cerebral cavernous malformation 2 [Source:MGI Sy	0.61525
ENSMUSG00000047205	Dusp18	dual specificity phosphatase 18 [Source:MGI Symbo	0.61541
ENSMUSG00000020467	Efemp1	epidermal growth factor-containing fibulin-like extr	0.61544
ENSMUSG00000053819	Camk2d	calcium/calmodulin-dependent protein kinase II, de	0.61546
ENSMUSG00000050022	Amz1	archaelysin family metallopeptidase 1 [Source:MGI	0.61552
ENSMUSG00000021900	Btd	biotinidase [Source:MGI Symbol;Acc:MGI:1347001]	0.61565
ENSMUSG00000040093	Bmf	BCL2 modifying factor [Source:MGI Symbol;Acc:MG	0.61565
ENSMUSG00000019856	Fam184a	family with sequence similarity 184, member A [Sou	0.6157
ENSMUSG00000034117	Gpr44	G protein-coupled receptor 44 [Source:MGI Symbo	0.61575
ENSMUSG00000096914	Galntl6	UDP-N-acetyl-alpha-D-galactosamine:polypeptide N	0.61575
ENSMUSG00000075514	Gm13375	predicted gene 13375 [Source:MGI Symbol;Acc:MG	0.61581
ENSMUSG00000025511	Tspan4	tetraspanin 4 [Source:MGI Symbol;Acc:MGI:192809	0.61582
ENSMUSG00000018554	Ybx2	Y box protein 2 [Source:MGI Symbol;Acc:MGI:1096	0.61592
ENSMUSG00000067158	Col4a4	collagen, type IV, alpha 4 [Source:MGI Symbol;Acc:	0.61598
ENSMUSG00000000248	Clec2g	C-type lectin domain family 2, member g [Source:M	0.616

ENSMUSG00000042426	Dhx29	DEAH (Asp-Glu-Ala-His) box polypeptide 29 [Source	0.61607
ENSMUSG00000031429	Psmd10	proteasome (prosome, macropain) 26S subunit, no	0.61612
ENSMUSG00000030965	Fam175b	family with sequence similarity 175, member B [So	0.61615
ENSMUSG00000043940	Wdfy3	WD repeat and FYVE domain containing 3 [Source:I	0.61616
ENSMUSG00000030263	Lrmp	lymphoid-restricted membrane protein [Source:MG	0.61622
ENSMUSG00000097902	Gm26531	predicted gene, 26531 [Source:MGI Symbol;Acc:M(	0.61623
ENSMUSG00000029510	Gpc2	glypican 2 (cerebroglycan) [Source:MGI Symbol;Acc	0.6163
ENSMUSG00000088948	Gm23262	predicted gene, 23262 [Source:MGI Symbol;Acc:M(	0.61631
ENSMUSG00000097769	Snhg4	small nucleolar RNA host gene 4 (non-protein codir	0.61635
ENSMUSG00000034235	Usp54	ubiquitin specific peptidase 54 [Source:MGI Symbo	0.61644
ENSMUSG00000024587	Nars	asparaginyl-tRNA synthetase [Source:MGI Symbol;A	0.61663
ENSMUSG00000086291	Gm15513	predicted gene 15513 [Source:MGI Symbol;Acc:MG	0.61679
ENSMUSG00000083460	Gm12182	predicted gene 12182 [Source:MGI Symbol;Acc:MG	0.61681
ENSMUSG00000035238	Kcnk15	potassium channel, subfamily K, member 15 [Sourc	0.6171
ENSMUSG00000085517	Gm12963	predicted gene 12963 [Source:MGI Symbol;Acc:MG	0.61726
ENSMUSG00000097585	Gm26529	predicted gene, 26529 [Source:MGI Symbol;Acc:M(	0.61727
ENSMUSG00000022245	Skor1	SKI family transcriptional corepressor 1 [Source:MG	0.61728
ENSMUSG00000065258	Gm23969	predicted gene, 23969 [Source:MGI Symbol;Acc:M(	0.61729
ENSMUSG00000078671	Chd2	chromodomain helicase DNA binding protein 2 [Sou	0.61737
ENSMUSG00000060594	Layn	layilin [Source:MGI Symbol;Acc:MGI:2685357]	0.61751
ENSMUSG00000026377	Mki67ip	Mki67 (FHA domain) interacting nucleolar phospho	0.61752
ENSMUSG00000029098	Acox3	acyl-Coenzyme A oxidase 3, pristanoyl [Source:MGI	0.61764
ENSMUSG00000040046	Tph1	tryptophan hydroxylase 1 [Source:MGI Symbol;Acc	0.61766
ENSMUSG00000006445	Epha2	Eph receptor A2 [Source:MGI Symbol;Acc:MGI:952	0.61773
ENSMUSG00000067144	Slc22a7	solute carrier family 22 (organic anion transporter),	0.61777
ENSMUSG00000025041	Nt5c2	5'-nucleotidase, cytosolic II [Source:MGI Symbol;Ac	0.61802
ENSMUSG00000030346	Rad51ap1	RAD51 associated protein 1 [Source:MGI Symbol;A	0.61803
ENSMUSG00000052396	Mogat2	monoacylglycerol O-acyltransferase 2 [Source:MGI	0.61811
ENSMUSG00000010362	Rdm1	RAD52 motif 1 [Source:MGI Symbol;Acc:MGI:19138	0.61816
ENSMUSG00000077704	Snord89	small nucleolar RNA, C/D box 89 [Source:MGI Symb	0.61816
ENSMUSG00000070025	Gm10284	predicted pseudogene 10284 [Source:MGI Symbol;	0.61822
ENSMUSG00000065586	Mir96	microRNA 96 [Source:MGI Symbol;Acc:MGI:361944	0.61828
ENSMUSG00000022325	Pop1	processing of precursor 1, ribonuclease P/MRP fam	0.61853
ENSMUSG00000078532	Nkain1	Na+/K+ transporting ATPase interacting 1 [Source:I	0.61859
ENSMUSG00000093249	Gm24576	predicted gene, 24576 [Source:MGI Symbol;Acc:M(	0.61862
ENSMUSG00000005779	Psmb4	proteasome (prosome, macropain) subunit, beta ty	0.61863
ENSMUSG00000025139	Tollip	toll interacting protein [Source:MGI Symbol;Acc:M(	0.61867
ENSMUSG00000030505	Prmt3	protein arginine N-methyltransferase 3 [Source:MG	0.61867
ENSMUSG00000090270	Gm17226	predicted gene 17226 [Source:MGI Symbol;Acc:MG	0.61877
ENSMUSG00000097823	Gm16701	predicted gene, 16701 [Source:MGI Symbol;Acc:M(	0.61882
ENSMUSG00000079162	Trim43b	tripartite motif-containing 43B [Source:MGI Symbo	0.61884
ENSMUSG00000021013	Ttc8	tetratricopeptide repeat domain 8 [Source:MGI Syr	0.61887
ENSMUSG00000037474	Dtl	denticleless homolog (Drosophila) [Source:MGI Syn	0.61887
ENSMUSG00000050875	A730017C20Rik	RIKEN cDNA A730017C20 gene [Source:MGI Symbo	0.6189
ENSMUSG00000023345	Poc1a	POC1 centriolar protein homolog A (Chlamydomon	0.61894
ENSMUSG00000049882	Mettl21d	methyltransferase like 21D [Source:MGI Symbol;Ac	0.61895
ENSMUSG00000009549	Srp14	signal recognition particle 14 [Source:MGI Symbol;A	0.61896

ENSMUSG00000092099	Gm17530	predicted gene, 17530 [Source:MGI Symbol;Acc:M(	0.61907
ENSMUSG00000070291	Ddx43	DEAD (Asp-Glu-Ala-Asp) box polypeptide 43 [Sourc	0.6191
ENSMUSG00000071553	Cpa2	carboxypeptidase A2, pancreatic [Source:MGI Symb	0.61912
ENSMUSG00000055912	Tmem150a	transmembrane protein 150A [Source:MGI Symbol;	0.61918
ENSMUSG00000056592	Zfp658	zinc finger protein 658 [Source:MGI Symbol;Acc:M(	0.61925
ENSMUSG00000092509	Gm20394	predicted gene 20394 [Source:MGI Symbol;Acc:MG	0.61927
ENSMUSG00000046111	5830418K08Rik	RIKEN cDNA 5830418K08 gene [Source:MGI Symbo	0.61928
ENSMUSG00000032860	P2ry2	purinergic receptor P2Y, G-protein coupled 2 [Sour	0.6193
ENSMUSG00000080743	Gm7150	predicted gene 7150 [Source:MGI Symbol;Acc:MGI:	0.61931
ENSMUSG00000014030	Pax5	paired box gene 5 [Source:MGI Symbol;Acc:MGI:97	0.61932
ENSMUSG00000073485	Gm16067	predicted gene 16067 [Source:MGI Symbol;Acc:MG	0.61936
ENSMUSG00000029685	Asb15	ankyrin repeat and SOCS box-containing 15 [Source	0.6195
ENSMUSG00000015968	Cacna1d	calcium channel, voltage-dependent, L type, alpha	0.61952
ENSMUSG00000018698	Lhx1	LIM homeobox protein 1 [Source:MGI Symbol;Acc:I	0.61955
ENSMUSG00000056131	Pgm3	phosphoglucomutase 3 [Source:MGI Symbol;Acc:M	0.61959
ENSMUSG00000031683	Lsm6	LSM6 homolog, U6 small nuclear RNA associated (S	0.61971
ENSMUSG00000023806	Rsph3b	radial spoke 3B homolog (Chlamydomonas) [Source	0.61977
ENSMUSG00000097061	9330151L19Rik	RIKEN cDNA 9330151L19 gene [Source:MGI Symbo	0.6198
ENSMUSG00000050945	Zfp438	zinc finger protein 438 [Source:MGI Symbol;Acc:M(	0.61984
ENSMUSG00000036022	Fam122b	family with sequence similarity 122, member B [So	0.61988
ENSMUSG00000042349	Ikbke	inhibitor of kappaB kinase epsilon [Source:MGI Sym	0.61997
ENSMUSG00000009828	Ick	intestinal cell kinase [Source:MGI Symbol;Acc:MGI:	0.62002
ENSMUSG00000095279	Gm10067	predicted gene 10067 [Source:MGI Symbol;Acc:MG	0.62004
ENSMUSG00000030707	Coro1a	coronin, actin binding protein 1A [Source:MGI Sym	0.62025
ENSMUSG00000085427	6430710C18Rik	RIKEN cDNA 6430710C18 gene [Source:MGI Symbo	0.62028
ENSMUSG00000034168	Irf2bpl	interferon regulatory factor 2 binding protein-like [	0.62034
ENSMUSG00000025730	Rab40c	Rab40c, member RAS oncogene family [Source:MG	0.62047
ENSMUSG00000007783	Cpt1c	carnitine palmitoyltransferase 1c [Source:MGI Sym	0.62048
ENSMUSG00000037772	Mrpl23	mitochondrial ribosomal protein L23 [Source:MGI S	0.62064
ENSMUSG00000069053	Uba1y	ubiquitin-activating enzyme, Chr Y [Source:MGI Syr	0.62071
ENSMUSG00000025790	Slco3a1	solute carrier organic anion transporter family, mer	0.6211
ENSMUSG00000085710	Gm12676	predicted gene 12676 [Source:MGI Symbol;Acc:MG	0.62114
ENSMUSG00000087589	D430040D24Rik	RIKEN cDNA D430040D24 gene [Source:MGI Symbo	0.62126
ENSMUSG00000038528	BC021785	cDNA sequence BC021785 [Source:MGI Symbol;Acc	0.62128
ENSMUSG00000041923	Nol4	nucleolar protein 4 [Source:MGI Symbol;Acc:MGI:2	0.62129
ENSMUSG00000074634	Gm7120	predicted gene 7120 [Source:MGI Symbol;Acc:MGI:	0.62132
ENSMUSG00000029196	Tada2b	transcriptional adaptor 2B [Source:MGI Symbol;Acc	0.62148
ENSMUSG00000030089	Slc41a3	solute carrier family 41, member 3 [Source:MGI Syr	0.62149
ENSMUSG00000031737	Irx5	Iroquois related homeobox 5 (Drosophila) [Source:I	0.62161
ENSMUSG00000042694	Obfc1	oligonucleotide/oligosaccharide-binding fold conta	0.62172
ENSMUSG00000083992	Gm11478	predicted gene 11478 [Source:MGI Symbol;Acc:MG	0.62178
ENSMUSG00000015126	Tsr3	TSR3 20S rRNA accumulation [Source:MGI Symbol;/	0.62179
ENSMUSG00000020218	Wif1	Wnt inhibitory factor 1 [Source:MGI Symbol;Acc:M(	0.6219
ENSMUSG00000026554	Dcaf8	DDB1 and CUL4 associated factor 8 [Source:MGI Sy	0.62193
ENSMUSG00000060913	Trim55	tripartite motif-containing 55 [Source:MGI Symbol;	0.62196
ENSMUSG00000021456	Fbp2	fructose bisphosphatase 2 [Source:MGI Symbol;Acc	0.62197
ENSMUSG00000087366	BC042782	cDNA sequence BC042782 [Source:MGI Symbol;Acc	0.62201

ENSMUSG00000040829	Zmynd15	zinc finger, MYND-type containing 15 [Source:MGI S	0.62202
ENSMUSG00000034729	Mrps10	mitochondrial ribosomal protein S10 [Source:MGI S	0.62208
ENSMUSG00000085870	Gm12829	predicted gene 12829 [Source:MGI Symbol;Acc:MG	0.62218
ENSMUSG00000020440	Arf5	ADP-ribosylation factor 5 [Source:MGI Symbol;Acc:	0.6222
ENSMUSG00000082080	Gm9246	predicted gene 9246 [Source:MGI Symbol;Acc:MGI:	0.62231
ENSMUSG00000043833	2900005J15Rik	RIKEN cDNA 2900005J15 gene [Source:MGI Symbol	0.62232
ENSMUSG00000024948	Map4k2	mitogen-activated protein kinase kinase kinase kina	0.62247
ENSMUSG00000027663	Zmat3	zinc finger matrin type 3 [Source:MGI Symbol;Acc:M	0.6225
ENSMUSG00000061298	Agbl4	ATP/GTP binding protein-like 4 [Source:MGI Symbo	0.62266
ENSMUSG00000020638	Cmpk2	cytidine monophosphate (UMP-CMP) kinase 2, mito	0.62267
ENSMUSG00000031842	Pde4c	phosphodiesterase 4C, cAMP specific [Source:MGI S	0.62283
ENSMUSG00000026457	Adipor1	adiponectin receptor 1 [Source:MGI Symbol;Acc:MG	0.62286
ENSMUSG00000083166	Gm8648	predicted gene 8648 [Source:MGI Symbol;Acc:MGI:	0.62296
ENSMUSG00000046201	Scaf8	SR-related CTD-associated factor 8 [Source:MGI Syr	0.62301
ENSMUSG00000063558	Aox1	aldehyde oxidase 1 [Source:MGI Symbol;Acc:MGI:8	0.62304
ENSMUSG00000024537	Psmg2	proteasome (prosome, macropain) assembly chape	0.62309
ENSMUSG00000025255	Zfhx4	zinc finger homeodomain 4 [Source:MGI Symbol;Ac	0.62325
ENSMUSG00000061100	Retnla	resistin like alpha [Source:MGI Symbol;Acc:MGI:188	0.6233
ENSMUSG00000071076	Jund	Jun proto-oncogene related gene d [Source:MGI Sy	0.62333
ENSMUSG00000034616	Ssh3	slingshot homolog 3 (Drosophila) [Source:MGI Sym	0.62336
ENSMUSG00000020766	Galk1	galactokinase 1 [Source:MGI Symbol;Acc:MGI:9573	0.62338
ENSMUSG00000070284	Gmppb	GDP-mannose pyrophosphorylase B [Source:MGI Sy	0.62354
ENSMUSG00000097886	Gm12302	predicted gene 12302 [Source:MGI Symbol;Acc:MG	0.62355
ENSMUSG00000037601	Nme1	NME/NM23 nucleoside diphosphate kinase 1 [Sour	0.62375
ENSMUSG00000059891	Tsks	testis-specific serine kinase substrate [Source:MGI S	0.62377
ENSMUSG00000033082	Clec1a	C-type lectin domain family 1, member a [Source:M	0.62409
ENSMUSG00000029622	Arpc1b	actin related protein 2/3 complex, subunit 1B [Sour	0.62411
ENSMUSG00000048120	Entpd1	ectonucleoside triphosphate diphosphohydrolase 1	0.62427
ENSMUSG00000037010	Apln	apelin [Source:MGI Symbol;Acc:MGI:1353624]	0.62435
ENSMUSG00000077846	Gm22232	predicted gene, 22232 [Source:MGI Symbol;Acc:MG	0.62449
ENSMUSG00000036430	Tbcc	tubulin-specific chaperone C [Source:MGI Symbol;A	0.62457
ENSMUSG00000086083	Gm15829	predicted gene 15829 [Source:MGI Symbol;Acc:MG	0.62457
ENSMUSG00000024797	Vps51	vacuolar protein sorting 51 homolog (S. cerevisiae)	0.62461
ENSMUSG00000029761	Cald1	caldesmon 1 [Source:MGI Symbol;Acc:MGI:88250]	0.62466
ENSMUSG00000034023	Fancd2	Fanconi anemia, complementation group D2 [Sourc	0.62474
ENSMUSG00000082920	Gm13864	predicted gene 13864 [Source:MGI Symbol;Acc:MG	0.62474
ENSMUSG00000058793	Cds2	CDP-diacylglycerol synthase (phosphatidate cytidyl	0.62481
ENSMUSG00000049751	Rpl36al	ribosomal protein L36A-like [Source:MGI Symbol;Ac	0.62486
ENSMUSG00000056413	Adap1	ArfGAP with dual PH domains 1 [Source:MGI Symbo	0.62503
ENSMUSG00000087185	Gm13872	predicted gene 13872 [Source:MGI Symbol;Acc:MG	0.62507
ENSMUSG00000034675	Dbn1	drebrin 1 [Source:MGI Symbol;Acc:MGI:1931838]	0.62514
ENSMUSG00000038987	1700019L03Rik	RIKEN cDNA 1700019L03 gene [Source:MGI Symbo	0.62524
ENSMUSG00000027430	Dtd1	D-tyrosyl-tRNA deacylase 1 [Source:MGI Symbol;Ac	0.62525
ENSMUSG00000089665	Fcor	Foxo1 corepressor [Source:MGI Symbol;Acc:MGI:19	0.62525
ENSMUSG00000024866	Acy3	aspartoacylase (aminoacylase) 3 [Source:MGI Symb	0.6253
ENSMUSG00000038010	Ccdc138	coiled-coil domain containing 138 [Source:MGI Sym	0.62537
ENSMUSG00000038260	Trpm4	transient receptor potential cation channel, subfam	0.62537

ENSMUSG00000039660	D2Wsu81e	DNA segment, Chr 2, Wayne State University 81, ex	0.62542
ENSMUSG00000020190	Mknk2	MAP kinase-interacting serine/threonine kinase 2 [	0.62543
ENSMUSG00000025194	Abcc2	ATP-binding cassette, sub-family C (CFTR/MRP), me	0.62576
ENSMUSG00000044117	2900011O08Rik	RIKEN cDNA 2900011O08 gene [Source:MGI Symbo	0.62578
ENSMUSG00000088849	Gm26353	predicted gene, 26353 [Source:MGI Symbol;Acc:MG	0.62588
ENSMUSG00000019303	Psmc3ip	proteasome (prosome, macropain) 26S subunit, AT	0.62591
ENSMUSG00000052426	Gm9877	predicted gene 9877 [Source:MGI Symbol;Acc:MGI:	0.62591
ENSMUSG00000098224	Gm7985	predicted gene 7985 [Source:MGI Symbol;Acc:MGI:	0.62591
ENSMUSG00000074569	Gcnt7	glucosaminyl (N-acetyl) transferase family member	0.62596
ENSMUSG00000045930	Clec14a	C-type lectin domain family 14, member a [Source:	0.62598
ENSMUSG00000085303	Gm16751	predicted gene, 16751 [Source:MGI Symbol;Acc:MG	0.62609
ENSMUSG00000063656	Gm10135	predicted gene 10135 [Source:MGI Symbol;Acc:MG	0.6261
ENSMUSG00000032826	Ank2	ankyrin 2, brain [Source:MGI Symbol;Acc:MGI:8802	0.62618
ENSMUSG00000086555	Gm13446	predicted gene 13446 [Source:MGI Symbol;Acc:MG	0.62633
ENSMUSG00000036249	Rbm43	RNA binding motif protein 43 [Source:MGI Symbol;	0.62639
ENSMUSG00000025574	Tk1	thymidine kinase 1 [Source:MGI Symbol;Acc:MGI:9	0.6264
ENSMUSG00000084781	D930015M05Rik	RIKEN cDNA D930015M05 gene [Source:MGI Symb	0.62644
ENSMUSG00000056234	Ncoa4	nuclear receptor coactivator 4 [Source:MGI Symbol	0.62645
ENSMUSG00000047604	Frat2	frequently rearranged in advanced T cell lymphoma	0.62653
ENSMUSG00000034449	Dhrs11	dehydrogenase/reductase (SDR family) member 11	0.62655
ENSMUSG00000087588	Gm16355	predicted gene 16355 [Source:MGI Symbol;Acc:MG	0.62655
ENSMUSG00000025781	Atp5c1	ATP synthase, H+ transporting, mitochondrial F1 co	0.62658
ENSMUSG00000027536	Chmp4c	charged multivesicular body protein 4C [Source:MG	0.62669
ENSMUSG00000017390	Aldoc	aldolase C, fructose-bisphosphate [Source:MGI Sym	0.62675
ENSMUSG00000087106	Gm13964	predicted gene 13964 [Source:MGI Symbol;Acc:MG	0.62684
ENSMUSG00000022124	Fbxl3	F-box and leucine-rich repeat protein 3 [Source:MG	0.62687
ENSMUSG00000008682	Rpl10	ribosomal protein L10 [Source:MGI Symbol;Acc:MG	0.62697
ENSMUSG00000033427	Upb1	ureidopropionase, beta [Source:MGI Symbol;Acc:M	0.62719
ENSMUSG00000038540	Tmc3	transmembrane channel-like gene family 3 [Source	0.62719
ENSMUSG00000025078	Nhlrc2	NHL repeat containing 2 [Source:MGI Symbol;Acc:M	0.6272
ENSMUSG00000087314	Gm15552	predicted gene 15552 [Source:MGI Symbol;Acc:MG	0.62726
ENSMUSG00000020090	Npffr1	neuropeptide FF receptor 1 [Source:MGI Symbol;Ac	0.62755
ENSMUSG00000032842	Abcc10	ATP-binding cassette, sub-family C (CFTR/MRP), me	0.62776
ENSMUSG00000087036	Gm11748	predicted gene 11748 [Source:MGI Symbol;Acc:MG	0.62777
ENSMUSG00000075576	Gm12359	predicted gene 12359 [Source:MGI Symbol;Acc:MG	0.62792
ENSMUSG00000092592	Gm20449	predicted gene 20449 [Source:MGI Symbol;Acc:MG	0.62799
ENSMUSG00000029829	Tmem213	transmembrane protein 213 [Source:MGI Symbol;A	0.628
ENSMUSG00000062461	Gm5453	predicted gene 5453 [Source:MGI Symbol;Acc:MGI:	0.62802
ENSMUSG00000038866	Zcchc2	zinc finger, CCHC domain containing 2 [Source:MGI	0.62805
ENSMUSG00000096322	Gm3898	predicted gene 3898 [Source:MGI Symbol;Acc:MGI:	0.62808
ENSMUSG00000031380	Figf	c-fos induced growth factor [Source:MGI Symbol;Ac	0.62811
ENSMUSG00000026622	Nek2	NIMA (never in mitosis gene a)-related expressed k	0.6282
ENSMUSG00000022947	Cbr3	carbonyl reductase 3 [Source:MGI Symbol;Acc:MGI	0.62827
ENSMUSG00000067173	Mrgpra4	MAS-related GPR, member A4 [Source:MGI Symbol	0.62862
ENSMUSG00000081631	Gm12005	predicted gene 12005 [Source:MGI Symbol;Acc:MG	0.62862
ENSMUSG00000082408	Gm13545	predicted gene 13545 [Source:MGI Symbol;Acc:MG	0.62862
ENSMUSG00000022487	Gtsf1	gametocyte specific factor 1 [Source:MGI Symbol;A	0.62863

ENSMUSG00000064994	Gm22422	predicted gene, 22422 [Source:MGI Symbol;Acc:MG	0.62864
ENSMUSG00000076577	Igkv8-30	immunoglobulin kappa chain variable 8-30 [Source:	0.62864
ENSMUSG00000081319	Gm12936	predicted gene 12936 [Source:MGI Symbol;Acc:MG	0.62864
ENSMUSG00000079410	Gm2897	predicted gene 2897 [Source:MGI Symbol;Acc:MGI:	0.62865
ENSMUSG00000080739	Gm13795	predicted gene 13795 [Source:MGI Symbol;Acc:MG	0.62865
ENSMUSG00000081773	Gm8302	predicted gene 8302 [Source:MGI Symbol;Acc:MGI:	0.62865
ENSMUSG00000026343	Gpr39	G protein-coupled receptor 39 [Source:MGI Symbo	0.62866
ENSMUSG00000091906	1700099I09Rik	RIKEN cDNA 1700099I09 gene [Source:MGI Symbol	0.62866
ENSMUSG00000027573	Gid8	GID complex subunit 8 homolog (S. cerevisiae) [Sou	0.62867
ENSMUSG00000058613	Gm5062	predicted gene 5062 [Source:MGI Symbol;Acc:MGI:	0.62867
ENSMUSG00000082423	Gm15148	predicted gene 15148 [Source:MGI Symbol;Acc:MG	0.62867
ENSMUSG00000097185	Gm26596	predicted gene, 26596 [Source:MGI Symbol;Acc:MG	0.62867
ENSMUSG00000024034	Tmprss3	transmembrane protease, serine 3 [Source:MGI Syr	0.62868
ENSMUSG00000090319	Gm4462	predicted gene 4462 [Source:MGI Symbol;Acc:MGI:	0.62868
ENSMUSG00000093548	Gm6407	predicted gene 6407 [Source:MGI Symbol;Acc:MGI:	0.62869
ENSMUSG00000020700	Map3k3	mitogen-activated protein kinase kinase kinase 3 [S	0.6287
ENSMUSG00000055968	Gm9988	predicted gene 9988 [Source:MGI Symbol;Acc:MGI:	0.6287
ENSMUSG00000086356	Gm13441	predicted gene 13441 [Source:MGI Symbol;Acc:MG	0.6287
ENSMUSG00000092813	Mir3086	microRNA 3086 [Source:MGI Symbol;Acc:MGI:4834	0.6287
ENSMUSG00000096474	Gm5561	predicted gene 5561 [Source:MGI Symbol;Acc:MGI:	0.6287
ENSMUSG00000034854	Mfsd12	major facilitator superfamily domain containing 12	0.62871
ENSMUSG00000054128	H2-T3	histocompatibility 2, T region locus 3 [Source:MGI S	0.62871
ENSMUSG00000081059	Gm11945	predicted gene 11945 [Source:MGI Symbol;Acc:MG	0.62871
ENSMUSG00000082579	Gm11979	predicted gene 11979 [Source:MGI Symbol;Acc:MG	0.62871
ENSMUSG00000085073	Gm14562	predicted gene 14562 [Source:MGI Symbol;Acc:MG	0.62871
ENSMUSG00000086293	Gm11269	predicted gene 11269 [Source:MGI Symbol;Acc:MG	0.62871
ENSMUSG00000088494	Gm24399	predicted gene, 24399 [Source:MGI Symbol;Acc:MG	0.62871
ENSMUSG00000089438	Gm23194	predicted gene, 23194 [Source:MGI Symbol;Acc:MG	0.62871
ENSMUSG00000093242	Gm24574	predicted gene, 24574 [Source:MGI Symbol;Acc:MG	0.62871
ENSMUSG00000095788	Sirpb1a	signal-regulatory protein beta 1A [Source:MGI Sym	0.62871
ENSMUSG00000097503	3110045C21Rik	RIKEN cDNA 3110045C21 gene [Source:MGI Symbo	0.62871
ENSMUSG00000030162	Olr1	oxidized low density lipoprotein (lectin-like) recept	0.62872
ENSMUSG00000051490	Foxd4	forkhead box D4 [Source:MGI Symbol;Acc:MGI:134	0.62872
ENSMUSG00000081680	Gm14473	predicted gene 14473 [Source:MGI Symbol;Acc:MG	0.62872
ENSMUSG00000082032	Gm12271	predicted gene 12271 [Source:MGI Symbol;Acc:MG	0.62872
ENSMUSG00000084240	Gm15383	predicted gene 15383 [Source:MGI Symbol;Acc:MG	0.62872
ENSMUSG00000084659	Gm22720	predicted gene, 22720 [Source:MGI Symbol;Acc:MG	0.62872
ENSMUSG00000084706	Gm22987	predicted gene, 22987 [Source:MGI Symbol;Acc:MG	0.62872
ENSMUSG00000085222	Gm13974	predicted gene 13974 [Source:MGI Symbol;Acc:MG	0.62872
ENSMUSG00000085223	Gm11210	predicted gene 11210 [Source:MGI Symbol;Acc:MG	0.62872
ENSMUSG00000089015	Gm24996	predicted gene, 24996 [Source:MGI Symbol;Acc:MG	0.62872
ENSMUSG00000092748	Gm22175	predicted gene, 22175 [Source:MGI Symbol;Acc:MG	0.62872
ENSMUSG00000072768	Gm10418	predicted gene 10418 [Source:MGI Symbol;Acc:MG	0.62873
ENSMUSG00000080529	Gm23882	predicted gene, 23882 [Source:MGI Symbol;Acc:MG	0.62873
ENSMUSG00000081758	Gm11460	predicted gene 11460 [Source:MGI Symbol;Acc:MG	0.62873
ENSMUSG00000081797	Gm8662	predicted gene 8662 [Source:MGI Symbol;Acc:MGI:	0.62873
ENSMUSG00000083794	Gm16123	predicted gene 16123 [Source:MGI Symbol;Acc:MG	0.62873

ENSMUSG00000084153	Gm12186	predicted gene 12186 [Source:MGI Symbol;Acc:MG	0.62873
ENSMUSG00000089298	Gm24636	predicted gene, 24636 [Source:MGI Symbol;Acc:M(	0.62873
ENSMUSG00000093030	Gm23632	predicted gene, 23632 [Source:MGI Symbol;Acc:M(	0.62873
ENSMUSG00000098242	Gm26929	predicted gene, 26929 [Source:MGI Symbol;Acc:M(	0.62873
ENSMUSG00000020295	Hbq1a	hemoglobin, theta 1A [Source:MGI Symbol;Acc:MG	0.62874
ENSMUSG00000037738	Nek5	NIMA (never in mitosis gene a)-related expressed k	0.62874
ENSMUSG00000074602	Gm10728	predicted gene 10728 [Source:MGI Symbol;Acc:MG	0.62874
ENSMUSG00000085965	2310016D23Rik	RIKEN cDNA 2310016D23 gene [Source:MGI Symbo	0.62874
ENSMUSG00000029788	Cpa5	carboxypeptidase A5 [Source:MGI Symbol;Acc:MGI	0.62875
ENSMUSG00000035371	Ssx9	synovial sarcoma, X breakpoint 9 [Source:MGI Sym	0.62875
ENSMUSG00000073090	Gm7932	predicted gene 7932 [Source:MGI Symbol;Acc:MGI:	0.62875
ENSMUSG00000073980	Gm9574	predicted gene 9574 [Source:MGI Symbol;Acc:MGI:	0.62875
ENSMUSG00000078192	Gm7290	predicted pseudogene 7290 [Source:MGI Symbol;A	0.62875
ENSMUSG00000084419	Gm22275	predicted gene, 22275 [Source:MGI Symbol;Acc:M(	0.62875
ENSMUSG00000027694	Gm8325	predicted pseudogene 8325 [Source:MGI Symbol;A	0.62876
ENSMUSG00000010841	1700006E09Rik	RIKEN cDNA 1700006E09 gene [Source:MGI Symbo	0.62877
ENSMUSG00000024107	Lhcgr	luteinizing hormone/choriogonadotropin receptor	0.62877
ENSMUSG00000062064	Slc2a7	solute carrier family 2 (facilitated glucose transport	0.62877
ENSMUSG00000013562	4833413E03Rik	RIKEN cDNA 4833413E03 gene [Source:MGI Symbo	0.62878
ENSMUSG00000038806	Sde2	SDE2 telomere maintenance homolog (S. pombe) [	0.62878
ENSMUSG00000077202	Gm25612	predicted gene, 25612 [Source:MGI Symbol;Acc:M(	0.62878
ENSMUSG00000048398	Gm5065	predicted gene 5065 [Source:MGI Symbol;Acc:MGI:	0.62879
ENSMUSG00000039693	Msantd3	Myb/SANT-like DNA-binding domain containing 3 [	0.62881
ENSMUSG00000028841	Cnksr1	connector enhancer of kinase suppressor of Ras 1 [	0.62883
ENSMUSG00000034667	Xpot	exportin, tRNA (nuclear export receptor for tRNAs)	0.62883
ENSMUSG00000065078	Gm24536	predicted gene, 24536 [Source:MGI Symbol;Acc:M(	0.62883
ENSMUSG00000063932	Poteg	POTE ankyrin domain family, member G [Source:M	0.62886
ENSMUSG00000082284	H3f3a-ps1	H3 histone, family 3A, pseudogene 1 [Source:MGI S	0.62887
ENSMUSG00000081811	Gm12713	predicted gene 12713 [Source:MGI Symbol;Acc:MG	0.62888
ENSMUSG00000077785	Gm23910	predicted gene, 23910 [Source:MGI Symbol;Acc:M(	0.62889
ENSMUSG00000080849	Gm15702	predicted gene 15702 [Source:MGI Symbol;Acc:MG	0.62889
ENSMUSG00000081274	Gm15727	predicted gene 15727 [Source:MGI Symbol;Acc:MG	0.62889
ENSMUSG00000083340	Gm13156	predicted gene 13156 [Source:MGI Symbol;Acc:MG	0.62889
ENSMUSG00000065202	Gm24615	predicted gene, 24615 [Source:MGI Symbol;Acc:M(	0.6289
ENSMUSG00000082315	Gm16523	predicted gene, 16523 [Source:MGI Symbol;Acc:M(	0.6289
ENSMUSG00000084243	Gm13784	predicted gene 13784 [Source:MGI Symbol;Acc:MG	0.6289
ENSMUSG00000036743	Psma8	proteasome (prosome, macropain) subunit, alpha t	0.62891
ENSMUSG00000068957	4930589L23Rik	RIKEN cDNA 4930589L23 gene [Source:MGI Symbo	0.62891
ENSMUSG00000034192	Lsm3	LSM3 homolog, U6 small nuclear RNA associated (S	0.62892
ENSMUSG00000079633	Rhox4d	reproductive homeobox 4D [Source:MGI Symbol;A	0.62893
ENSMUSG00000083871	Gm16479	predicted gene 16479 [Source:MGI Symbol;Acc:MG	0.62893
ENSMUSG00000085810	Gm16325	predicted gene 16325 [Source:MGI Symbol;Acc:MG	0.62893
ENSMUSG00000067338	Tuba3b	tubulin, alpha 3B [Source:MGI Symbol;Acc:MGI:10	0.62894
ENSMUSG00000078010	Gm23489	predicted gene, 23489 [Source:MGI Symbol;Acc:M(	0.62894
ENSMUSG00000084760	Gm15472	predicted gene 15472 [Source:MGI Symbol;Acc:MG	0.62894
ENSMUSG00000086221	4930412B13Rik	RIKEN cDNA 4930412B13 gene [Source:MGI Symbo	0.62894
ENSMUSG00000064587	Gm25131	predicted gene, 25131 [Source:MGI Symbol;Acc:M(	0.62895

ENSMUSG00000074385	Gm10684	predicted gene 10684 [Source:MGI Symbol;Acc:MG	0.62895
ENSMUSG00000075325	Gm13582	predicted gene 13582 [Source:MGI Symbol;Acc:MG	0.62895
ENSMUSG00000081489	Gm14672	predicted gene 14672 [Source:MGI Symbol;Acc:MG	0.62895
ENSMUSG00000083772	Gm15566	predicted gene 15566 [Source:MGI Symbol;Acc:MG	0.62895
ENSMUSG00000091749	Tspy-ps	testis specific protein-Y encoded, pseudogene [Sou	0.62895
ENSMUSG00000092596	Gm20490	predicted gene 20490 [Source:MGI Symbol;Acc:MG	0.62895
ENSMUSG00000073408	Dpcr1	diffuse panbronchiolitis critical region 1 (human) [S	0.62896
ENSMUSG00000074555	Gm10714	predicted gene 10714 [Source:MGI Symbol;Acc:MG	0.62896
ENSMUSG00000077630	Gm22149	predicted gene, 22149 [Source:MGI Symbol;Acc:M(	0.62896
ENSMUSG00000080993	Gm12903	predicted gene 12903 [Source:MGI Symbol;Acc:MG	0.62896
ENSMUSG00000081139	Gm13333	predicted gene 13333 [Source:MGI Symbol;Acc:MG	0.62896
ENSMUSG00000084745	Gm25290	predicted gene, 25290 [Source:MGI Symbol;Acc:M(	0.62896
ENSMUSG00000064536	Gm23615	predicted gene, 23615 [Source:MGI Symbol;Acc:M(	0.62897
ENSMUSG00000074731	Zfp345	zinc finger protein 345 [Source:MGI Symbol;Acc:M(	0.62897
ENSMUSG00000081771	Gm15381	predicted gene 15381 [Source:MGI Symbol;Acc:MG	0.62897
ENSMUSG00000082550	Gm12344	predicted gene 12344 [Source:MGI Symbol;Acc:MG	0.62897
ENSMUSG00000084164	Gm13902	predicted gene 13902 [Source:MGI Symbol;Acc:MG	0.62897
ENSMUSG00000084783	Gm15419	predicted gene 15419 [Source:MGI Symbol;Acc:MG	0.62897
ENSMUSG00000086372	Gm16246	predicted gene 16246 [Source:MGI Symbol;Acc:MG	0.62897
ENSMUSG00000034785	Dio1	deiodinase, iodothyronine, type I [Source:MGI Sym	0.62898
ENSMUSG00000084642	Gm24384	predicted gene, 24384 [Source:MGI Symbol;Acc:M(	0.62898
ENSMUSG00000085237	Gm15406	predicted gene 15406 [Source:MGI Symbol;Acc:MG	0.62898
ENSMUSG00000089544	Gm25837	predicted gene, 25837 [Source:MGI Symbol;Acc:M(	0.62899
ENSMUSG00000023829	Slc22a1	solute carrier family 22 (organic cation transporter)	0.629
ENSMUSG00000059994	Fcrl1	Fc receptor-like 1 [Source:MGI Symbol;Acc:MGI:24	0.629
ENSMUSG00000091086	Gm5428	predicted gene 5428 [Source:MGI Symbol;Acc:MGI:	0.629
ENSMUSG00000093091	Mir3089	microRNA 3089 [Source:MGI Symbol;Acc:MGI:4834	0.629
ENSMUSG00000038791	Scgb3a2	secretoglobin, family 3A, member 2 [Source:MGI Sy	0.62901
ENSMUSG00000085937	4930535C22Rik	RIKEN cDNA 4930535C22 gene [Source:MGI Symbo	0.62901
ENSMUSG00000020562	Efcab10	EF-hand calcium binding domain 10 [Source:MGI Sy	0.62902
ENSMUSG00000021790	Dydc1	DPY30 domain containing 1 [Source:MGI Symbol;A(	0.62902
ENSMUSG00000028140	Mrpl9	mitochondrial ribosomal protein L9 [Source:MGI Sy	0.62905
ENSMUSG00000086612	Gm12064	predicted gene 12064 [Source:MGI Symbol;Acc:MG	0.62905
ENSMUSG00000087136	Gm15864	predicted gene 15864 [Source:MGI Symbol;Acc:MG	0.62905
ENSMUSG00000089676	B230217C12Rik	RIKEN cDNA B230217C12 gene [Source:MGI Symbo	0.62905
ENSMUSG00000020303	Stc2	stanniocalcin 2 [Source:MGI Symbol;Acc:MGI:1316	0.62906
ENSMUSG00000088958	Scarna8	small Cajal body-specific RNA 8 [Source:MGI Symbo	0.62906
ENSMUSG00000030638	Sh3gl3	SH3-domain GRB2-like 3 [Source:MGI Symbol;Acc:M	0.62907
ENSMUSG00000088777	Gm26039	predicted gene, 26039 [Source:MGI Symbol;Acc:M(	0.62907
ENSMUSG00000042625	Safb2	scaffold attachment factor B2 [Source:MGI Symbol;	0.62908
ENSMUSG00000088128	Gm25720	predicted gene, 25720 [Source:MGI Symbol;Acc:M(	0.62908
ENSMUSG00000089973	Vmn1r190-ps	vomeronasal 1 receptor 190, pseudogene [Source:M	0.62908
ENSMUSG00000090232	Gm16132	predicted gene 16132 [Source:MGI Symbol;Acc:MG	0.62912
ENSMUSG00000097764	Gm26635	predicted gene, 26635 [Source:MGI Symbol;Acc:M(	0.62921
ENSMUSG00000041936	Agrn	agrin [Source:MGI Symbol;Acc:MGI:87961]	0.62923
ENSMUSG00000097952	Gm4398	predicted gene 4398 [Source:MGI Symbol;Acc:MGI:	0.62923
ENSMUSG00000021376	Tpmt	thiopurine methyltransferase [Source:MGI Symbol;	0.62924

ENSMUSG00000081137	BC022960	cDNA sequence BC022960 [Source:MGI Symbol;Acc	0.62936
ENSMUSG00000083748	Gm11662	predicted gene 11662 [Source:MGI Symbol;Acc:MG	0.6294
ENSMUSG00000083310	Gm5760	predicted gene 5760 [Source:MGI Symbol;Acc:MGI:	0.62942
ENSMUSG00000081953	Gm12435	predicted gene 12435 [Source:MGI Symbol;Acc:MG	0.62943
ENSMUSG00000084343	Gm16198	predicted gene 16198 [Source:MGI Symbol;Acc:MG	0.62943
ENSMUSG00000086624	Gm16130	predicted gene 16130 [Source:MGI Symbol;Acc:MG	0.62943
ENSMUSG00000081898	Gm5754	predicted gene 5754 [Source:MGI Symbol;Acc:MGI:	0.62944
ENSMUSG00000074378	Bsph1	binder of sperm protein homolog 1 [Source:MGI Sy	0.62945
ENSMUSG00000085538	Gm11455	predicted gene 11455 [Source:MGI Symbol;Acc:MG	0.62945
ENSMUSG00000062518	Zfp534	zinc finger protein 534 [Source:MGI Symbol;Acc:M	0.62946
ENSMUSG00000064845	Gm26203	predicted gene, 26203 [Source:MGI Symbol;Acc:M	0.62946
ENSMUSG00000073430	Gm10505	predicted gene 10505 [Source:MGI Symbol;Acc:MG	0.62946
ENSMUSG00000081228	Gm16089	predicted gene 16089 [Source:MGI Symbol;Acc:MG	0.62946
ENSMUSG00000072576	Gm16020	predicted gene 16020 [Source:MGI Symbol;Acc:MG	0.62947
ENSMUSG00000080549	Gm22715	predicted gene, 22715 [Source:MGI Symbol;Acc:M	0.62947
ENSMUSG00000054757	Akr1c20	aldo-keto reductase family 1, member C20 [Source	0.62948
ENSMUSG00000086940	Gm12746	predicted gene 12746 [Source:MGI Symbol;Acc:MG	0.62948
ENSMUSG00000020469	Myl7	myosin, light polypeptide 7, regulatory [Source:MG	0.62949
ENSMUSG00000014813	Stc1	stanniocalcin 1 [Source:MGI Symbol;Acc:MGI:1091	0.6295
ENSMUSG00000021415	4933417A18Rik	RIKEN cDNA 4933417A18 gene [Source:MGI Symbo	0.6295
ENSMUSG00000027719	Adad1	adenosine deaminase domain containing 1 (testis s	0.6295
ENSMUSG00000051548	Gm6365	predicted pseudogene 6365 [Source:MGI Symbol;A	0.6295
ENSMUSG00000049230	Gm9833	predicted gene 9833 [Source:MGI Symbol;Acc:MGI:	0.62951
ENSMUSG00000050533	Gm9845	predicted pseudogene 9845 [Source:MGI Symbol;A	0.62951
ENSMUSG00000088853	Gm24730	predicted gene, 24730 [Source:MGI Symbol;Acc:M	0.62952
ENSMUSG00000087406	E130215H24Rik	RIKEN cDNA E130215H24 gene [Source:MGI Symbo	0.62955
ENSMUSG00000089968	4921518J05Rik	RIKEN cDNA 4921518J05 gene [Source:MGI Symbol	0.62956
ENSMUSG00000024325	Ring1	ring finger protein 1 [Source:MGI Symbol;Acc:MGI:	0.62958
ENSMUSG00000088855	Gm24724	predicted gene, 24724 [Source:MGI Symbol;Acc:M	0.6296
ENSMUSG00000091556	Gm14569	predicted gene 14569 [Source:MGI Symbol;Acc:MG	0.62961
ENSMUSG00000089369	Gm23819	predicted gene, 23819 [Source:MGI Symbol;Acc:M	0.62963
ENSMUSG00000092923	Gm24561	predicted gene, 24561 [Source:MGI Symbol;Acc:M	0.6297
ENSMUSG00000089069	Gm22840	predicted gene, 22840 [Source:MGI Symbol;Acc:M	0.62971
ENSMUSG00000088213	Gm22390	predicted gene, 22390 [Source:MGI Symbol;Acc:M	0.62973
ENSMUSG00000022687	Boc	biregional cell adhesion molecule-related/down-re	0.62974
ENSMUSG00000087596	Gm11715	predicted gene 11715 [Source:MGI Symbol;Acc:MG	0.62974
ENSMUSG00000095427	Rps2-ps6	ribosomal protein S2, pseudogene 6 [Source:MGI S	0.62974
ENSMUSG00000098245	Gm26927	predicted gene, 26927 [Source:MGI Symbol;Acc:M	0.62974
ENSMUSG00000087412	Gm15501	predicted pseudogene 15501 [Source:MGI Symbol;	0.62975
ENSMUSG00000083995	Gm12808	predicted gene 12808 [Source:MGI Symbol;Acc:MG	0.62977
ENSMUSG00000085638	Gm15521	predicted gene 15521 [Source:MGI Symbol;Acc:MG	0.62977
ENSMUSG00000083951	Gm13786	predicted gene 13786 [Source:MGI Symbol;Acc:MG	0.62978
ENSMUSG00000085158	Gm14264	predicted gene 14264 [Source:MGI Symbol;Acc:MG	0.62978
ENSMUSG00000013593	Ndufs2	NADH dehydrogenase (ubiquinone) Fe-S protein 2 [	0.6298
ENSMUSG00000072677	Gm10396	predicted gene 10396 [Source:MGI Symbol;Acc:MG	0.6298
ENSMUSG00000081111	Gm5913	predicted gene 5913 [Source:MGI Symbol;Acc:MGI:	0.6298
ENSMUSG00000081317	Gm12757	predicted gene 12757 [Source:MGI Symbol;Acc:MG	0.6298

ENSMUSG00000084969	Gm11205	predicted gene 11205 [Source:MGI Symbol;Acc:MG	0.6298
ENSMUSG00000080662	Mir1b	microRNA 1b [Source:MGI Symbol;Acc:MGI:378337	0.62981
ENSMUSG00000080757	Gm15029	predicted gene 15029 [Source:MGI Symbol;Acc:MG	0.62981
ENSMUSG00000033794	Lpcat2b	lysophosphatidylcholine acyltransferase 2B [Source	0.62982
ENSMUSG00000065642	Snora69	small nucleolar RNA, H/ACA box 69 [Source:MGI Sy	0.62982
ENSMUSG00000077921	Mir872	microRNA 872 [Source:MGI Symbol;Acc:MGI:37185	0.62982
ENSMUSG00000065331	Gm24927	predicted gene, 24927 [Source:MGI Symbol;Acc:M	0.62983
ENSMUSG00000077697	Gm25346	predicted gene, 25346 [Source:MGI Symbol;Acc:M	0.62983
ENSMUSG00000024670	Cd6	CD6 antigen [Source:MGI Symbol;Acc:MGI:103566]	0.62984
ENSMUSG00000029401	Rilpl2	Rab interacting lysosomal protein-like 2 [Source:M	0.62984
ENSMUSG00000054986	Sec14l3	SEC14-like 3 (S. cerevisiae) [Source:MGI Symbol;Ac	0.62985
ENSMUSG00000038973	Cldnd2	claudin domain containing 2 [Source:MGI Symbol;A	0.62986
ENSMUSG00000037683	Armc3	armadillo repeat containing 3 [Source:MGI Symbol;	0.62987
ENSMUSG00000048240	Gng7	guanine nucleotide binding protein (G protein), gar	0.62998
ENSMUSG00000002602	Axl	AXL receptor tyrosine kinase [Source:MGI Symbol;A	0.63003
ENSMUSG00000020167	Tcf3	transcription factor 3 [Source:MGI Symbol;Acc:MG	0.63018
ENSMUSG00000016510	Mtif3	mitochondrial translational initiation factor 3 [Sour	0.63021
ENSMUSG00000015120	Ube2i	ubiquitin-conjugating enzyme E2I [Source:MGI Sym	0.63023
ENSMUSG00000002820	Atg4d	autophagy related 4D, cysteine peptidase [Source:M	0.6303
ENSMUSG00000000730	Dnmt3l	DNA (cytosine-5-)-methyltransferase 3-like [Source	0.63031
ENSMUSG00000021068	Nin	ninein [Source:MGI Symbol;Acc:MGI:105108]	0.6304
ENSMUSG00000034173	2410018M08Rik	RIKEN cDNA 2410018M08 gene [Source:MGI Symb	0.6304
ENSMUSG00000027519	Rab22a	RAB22A, member RAS oncogene family [Source:M	0.63046
ENSMUSG00000051159	Cited1	Cbp/p300-interacting transactivator with Glu/Asp-r	0.63048
ENSMUSG00000020170	Frs2	fibroblast growth factor receptor substrate 2 [Sour	0.63053
ENSMUSG00000040629	Mael	maelstrom homolog (Drosophila) [Source:MGI Sym	0.63054
ENSMUSG00000045064	Zc2hc1c	zinc finger, C2HC-type containing 1C [Source:MGI S	0.63061
ENSMUSG00000085041	BB031773	expressed sequence BB031773 [Source:MGI Symbo	0.63077
ENSMUSG00000016194	Hsd11b1	hydroxysteroid 11-beta dehydrogenase 1 [Source:M	0.63094
ENSMUSG00000082895	Rpsa-ps9	ribosomal protein SA, pseudogene 9 [Source:MGI S	0.631
ENSMUSG00000028957	Per3	period circadian clock 3 [Source:MGI Symbol;Acc:M	0.63101
ENSMUSG00000028138	Adh5	alcohol dehydrogenase 5 (class III), chi polypeptide	0.63114
ENSMUSG00000093765	Gm20658	predicted gene 20658 [Source:MGI Symbol;Acc:MG	0.63117
ENSMUSG00000031170	Slc38a5	solute carrier family 38, member 5 [Source:MGI Syr	0.63119
ENSMUSG00000079484	Phyhd1	phytanoyl-CoA dioxygenase domain containing 1 [S	0.63133
ENSMUSG00000078451	Ppil6	peptidylprolyl isomerase (cyclophilin)-like 6 [Source	0.63134
ENSMUSG00000027244	Atg13	autophagy related 13 [Source:MGI Symbol;Acc:MG	0.63147
ENSMUSG00000037426	Depdc5	DEP domain containing 5 [Source:MGI Symbol;Acc:	0.63157
ENSMUSG00000028538	St3gal3	ST3 beta-galactoside alpha-2,3-sialyltransferase 3 [	0.63168
ENSMUSG00000065041	Gm24044	predicted gene, 24044 [Source:MGI Symbol;Acc:M	0.63168
ENSMUSG00000004902	Slc25a18	solute carrier family 25 (mitochondrial carrier), mer	0.6317
ENSMUSG00000097004	4731419I09Rik	RIKEN cDNA 4731419I09 gene [Source:MGI Symbol	0.63173
ENSMUSG00000054978	Kbtbd13	kelch repeat and BTB (POZ) domain containing 13 [	0.63175
ENSMUSG00000028613	Lrp8	low density lipoprotein receptor-related protein 8,	0.6318
ENSMUSG00000021500	Ddx46	DEAD (Asp-Glu-Ala-Asp) box polypeptide 46 [Sourc	0.63182
ENSMUSG00000086387	Gm15563	predicted gene 15563 [Source:MGI Symbol;Acc:MG	0.63185
ENSMUSG00000034118	Tpst1	protein-tyrosine sulfotransferase 1 [Source:MGI Sy	0.63187

ENSMUSG00000035831	Krt25	keratin 25 [Source:MGI Symbol;Acc:MGI:1918060]	0.63188
ENSMUSG00000075284	Wipf1	WAS/WASL interacting protein family, member 1 [S	0.63207
ENSMUSG00000061393	Acvr2b	activin receptor IIB [Source:MGI Symbol;Acc:MGI:8	0.63213
ENSMUSG00000097603	A430010J10Rik	RIKEN cDNA A430010J10 gene [Source:MGI Symbo	0.63216
ENSMUSG00000028558	Calr4	calreticulin 4 [Source:MGI Symbol;Acc:MGI:214043	0.63217
ENSMUSG00000030652	Coq7	demethyl-Q 7 [Source:MGI Symbol;Acc:MGI:10720	0.63219
ENSMUSG00000026618	Iars2	isoleucine-tRNA synthetase 2, mitochondrial [Sour	0.63224
ENSMUSG00000081997	Gm11886	predicted gene 11886 [Source:MGI Symbol;Acc:MG	0.63224
ENSMUSG00000021751	Acox2	acyl-Coenzyme A oxidase 2, branched chain [Source	0.63229
ENSMUSG00000032565	Nudt16	nudix (nucleoside diphosphate linked moiety X)-typ	0.6324
ENSMUSG00000022607	Ptk2	PTK2 protein tyrosine kinase 2 [Source:MGI Symbo	0.63241
ENSMUSG00000043964	Orai3	ORAI calcium release-activated calcium modulator	0.63249
ENSMUSG00000034673	Pbx2	pre B cell leukemia homeobox 2 [Source:MGI Symb	0.63252
ENSMUSG00000037321	Tap1	transporter 1, ATP-binding cassette, sub-family B (M	0.63252
ENSMUSG00000020399	Havcr2	hepatitis A virus cellular receptor 2 [Source:MGI Sy	0.63255
ENSMUSG00000062944	9130023H24Rik	RIKEN cDNA 9130023H24 gene [Source:MGI Symbo	0.63255
ENSMUSG00000030223	Ptpro	protein tyrosine phosphatase, receptor type, O [So	0.63258
ENSMUSG00000081103	Rps12-ps26	ribosomal protein S12, pseudogene 26 [Source:MG	0.63267
ENSMUSG00000025324	Atp10a	ATPase, class V, type 10A [Source:MGI Symbol;Acc:	0.63278
ENSMUSG00000030922	Lyrm1	LYR motif containing 1 [Source:MGI Symbol;Acc:MG	0.63278
ENSMUSG00000057322	Rpl38	ribosomal protein L38 [Source:MGI Symbol;Acc:MG	0.63279
ENSMUSG00000020738	Sumo2	SMT3 suppressor of mif two 3 homolog 2 (yeast) [S	0.63281
ENSMUSG00000008450	Nutf2	nuclear transport factor 2 [Source:MGI Symbol;Acc	0.63285
ENSMUSG00000025963	Mdh1b	malate dehydrogenase 1B, NAD (soluble) [Source:M	0.63293
ENSMUSG00000004032	Gstm5	glutathione S-transferase, mu 5 [Source:MGI Symb	0.63304
ENSMUSG00000030107	Usp18	ubiquitin specific peptidase 18 [Source:MGI Symbo	0.63308
ENSMUSG00000029168	Dpysl5	dihydropyrimidinase-like 5 [Source:MGI Symbol;Ac	0.63312
ENSMUSG00000023911	Flywch2	FLYWCH family member 2 [Source:MGI Symbol;Acc	0.63318
ENSMUSG00000086341	Gm15932	predicted gene 15932 [Source:MGI Symbol;Acc:MG	0.63318
ENSMUSG00000037851	Iars	isoleucine-tRNA synthetase [Source:MGI Symbol;Ac	0.63325
ENSMUSG00000003974	Grm3	glutamate receptor, metabotropic 3 [Source:MGI S	0.63333
ENSMUSG00000079278	Tmem233	transmembrane protein 233 [Source:MGI Symbol;A	0.63333
ENSMUSG00000028063	Lmna	lamin A [Source:MGI Symbol;Acc:MGI:96794]	0.63335
ENSMUSG00000047583	Tyw3	tRNA-yW synthesizing protein 3 homolog (S. cerevi	0.63337
ENSMUSG00000006728	Cdk4	cyclin-dependent kinase 4 [Source:MGI Symbol;Acc	0.63343
ENSMUSG00000031207	Msn	moesin [Source:MGI Symbol;Acc:MGI:97167]	0.63348
ENSMUSG00000037787	Apopt1	apoptogenic, mitochondrial 1 [Source:MGI Symbol;	0.63349
ENSMUSG00000046585	Ccdc147	coiled-coil domain containing 147 [Source:MGI Sym	0.63365
ENSMUSG00000029993	Nfu1	NFU1 iron-sulfur cluster scaffold homolog (S. cerev	0.63374
ENSMUSG00000029592	Usp30	ubiquitin specific peptidase 30 [Source:MGI Symbo	0.634
ENSMUSG00000054034	Tceal5	transcription elongation factor A (SII)-like 5 [Source	0.63409
ENSMUSG00000082286	Pisd-ps1	phosphatidylserine decarboxylase, pseudogene 1 [S	0.63409
ENSMUSG00000057606	Colq	collagen-like tail subunit (single strand of homotrim	0.63411
ENSMUSG00000030663	1110004F10Rik	RIKEN cDNA 1110004F10 gene [Source:MGI Symbo	0.63422
ENSMUSG00000091408	Gm6728	predicted gene 6728 [Source:MGI Symbol;Acc:MGI:	0.63434
ENSMUSG00000006021	Kptn	kaptin [Source:MGI Symbol;Acc:MGI:1890380]	0.6344
ENSMUSG00000006014	Prg4	proteoglycan 4 (megakaryocyte stimulating factor,	0.63454

ENSMUSG00000031730	Dhodh	dihydroorotate dehydrogenase [Source:MGI Symbo	0.63457
ENSMUSG00000039065	Fam173b	family with sequence similarity 173, member B [Sou	0.63462
ENSMUSG00000097534	Gm16675	predicted gene, 16675 [Source:MGI Symbol;Acc:MG	0.63465
ENSMUSG00000024191	Bnip1	BCL2/adenovirus E1B interacting protein 1 [Source:	0.63473
ENSMUSG00000022805	Maats1	MYCBP-associated, testis expressed 1 [Source:MGI	0.63477
ENSMUSG00000073403	Gm10499	predicted gene 10499 [Source:MGI Symbol;Acc:MG	0.6348
ENSMUSG00000039699	Batf2	basic leucine zipper transcription factor, ATF-like 2	0.63485
ENSMUSG00000061759	1700052N19Rik	RIKEN cDNA 1700052N19 gene [Source:MGI Symbo	0.63491
ENSMUSG00000054920	Klhl5	kelch-like 5 [Source:MGI Symbol;Acc:MGI:1919028]	0.63498
ENSMUSG00000098098	Gm20748	predicted gene, 20748 [Source:MGI Symbol;Acc:MG	0.63501
ENSMUSG00000046731	Kctd11	potassium channel tetramerisation domain contain	0.63513
ENSMUSG00000091345	Col6a5	collagen, type VI, alpha 5 [Source:MGI Symbol;Acc:	0.63513
ENSMUSG00000002949	Timm44	translocase of inner mitochondrial membrane 44 [S	0.63515
ENSMUSG00000003545	Fosb	FBJ osteosarcoma oncogene B [Source:MGI Symbol	0.63518
ENSMUSG00000081339	Gm16044	predicted gene 16044 [Source:MGI Symbol;Acc:MG	0.63527
ENSMUSG00000001946	Esam	endothelial cell-specific adhesion molecule [Source	0.63531
ENSMUSG00000084017	Rpl21-ps9	ribosomal protein L21, pseudogene 9 [Source:MGI	0.63531
ENSMUSG00000081829	Gm15460	predicted gene 15460 [Source:MGI Symbol;Acc:MG	0.6354
ENSMUSG00000030996	Art1	ADP-ribosyltransferase 1 [Source:MGI Symbol;Acc:I	0.63542
ENSMUSG00000081116	Gm13329	predicted gene 13329 [Source:MGI Symbol;Acc:MG	0.63544
ENSMUSG00000079470	Utp14b	UTP14, U3 small nucleolar ribonucleoprotein, homo	0.63568
ENSMUSG00000024304	Cdh2	cadherin 2 [Source:MGI Symbol;Acc:MGI:88355]	0.63575
ENSMUSG00000063605	Ccdc102a	coiled-coil domain containing 102A [Source:MGI Sy	0.6358
ENSMUSG00000015289	Lage3	L antigen family, member 3 [Source:MGI Symbol;Ac	0.63608
ENSMUSG00000029475	Kdm2b	lysine (K)-specific demethylase 2B [Source:MGI Sym	0.6361
ENSMUSG00000025008	Tctn3	tectonic family member 3 [Source:MGI Symbol;Acc	0.6364
ENSMUSG00000033316	Galnt9	UDP-N-acetyl-alpha-D-galactosamine:polypeptide N	0.63644
ENSMUSG00000087675	Gm11762	predicted gene 11762 [Source:MGI Symbol;Acc:MG	0.63654
ENSMUSG00000029490	Mfsd7a	major facilitator superfamily domain containing 7A	0.63656
ENSMUSG00000087556	Gm15764	predicted gene 15764 [Source:MGI Symbol;Acc:MG	0.63656
ENSMUSG00000029227	Fip1l1	FIP1 like 1 (S. cerevisiae) [Source:MGI Symbol;Acc:M	0.63664
ENSMUSG00000018727	Cpsf4l	cleavage and polyadenylation specific factor 4-like	0.63683
ENSMUSG00000025815	Dhtkd1	dehydrogenase E1 and transketolase domain conta	0.63683
ENSMUSG00000084088	Gm12941	predicted gene 12941 [Source:MGI Symbol;Acc:MG	0.63683
ENSMUSG00000046185	Zfp84	zinc finger protein 84 [Source:MGI Symbol;Acc:MGI	0.63697
ENSMUSG00000015247	Nipsnap3b	nipsnap homolog 3B (C. elegans) [Source:MGI Symb	0.637
ENSMUSG00000027111	Itga6	integrin alpha 6 [Source:MGI Symbol;Acc:MGI:9660	0.63706
ENSMUSG00000020992	4930512B01Rik	RIKEN cDNA 4930512B01 gene [Source:MGI Symbo	0.63709
ENSMUSG00000041429	Nthl1	nth (endonuclease III)-like 1 (E.coli) [Source:MGI Sy	0.63722
ENSMUSG00000067276	Capn6	calpain 6 [Source:MGI Symbol;Acc:MGI:1100850]	0.63733
ENSMUSG00000026173	Plcd4	phospholipase C, delta 4 [Source:MGI Symbol;Acc:M	0.63736
ENSMUSG00000033669	Zfp7	zinc finger protein 7 [Source:MGI Symbol;Acc:MGI:9	0.63739
ENSMUSG00000049641	Vgll2	vestigial like 2 homolog (Drosophila) [Source:MGI S	0.63749
ENSMUSG00000026141	Col19a1	collagen, type XIX, alpha 1 [Source:MGI Symbol;Acc	0.63762
ENSMUSG00000097888	Gm26682	predicted gene, 26682 [Source:MGI Symbol;Acc:MG	0.63763
ENSMUSG00000098120	Gm5914	predicted gene 5914 [Source:MGI Symbol;Acc:MGI:	0.63763
ENSMUSG00000031886	Ces2e	carboxylesterase 2E [Source:MGI Symbol;Acc:MGI:2	0.63766

ENSMUSG00000028687	Mutyh	mutY homolog (E. coli) [Source:MGI Symbol;Acc:MG	0.63778
ENSMUSG00000057191	AB124611	cDNA sequence AB124611 [Source:MGI Symbol;Acc	0.63781
ENSMUSG00000036046	5031439G07Rik	RIKEN cDNA 5031439G07 gene [Source:MGI Symbo	0.63782
ENSMUSG00000066629	Rpl36-ps3	ribosomal protein L36, pseudogene 3 [Source:MGI	0.6379
ENSMUSG00000025138	Sirt7	sirtuin 7 [Source:MGI Symbol;Acc:MGI:2385849]	0.63792
ENSMUSG00000024507	Hsd17b4	hydroxysteroid (17-beta) dehydrogenase 4 [Source	0.63794
ENSMUSG00000050075	Gpr171	G protein-coupled receptor 171 [Source:MGI Symb	0.63801
ENSMUSG00000086591	6030455H03Rik	RIKEN cDNA 6030455H03 gene [Source:MGI Symbo	0.63802
ENSMUSG00000020547	Bzw2	basic leucine zipper and W2 domains 2 [Source:MG	0.63811
ENSMUSG00000086504	4930511F01Rik	RIKEN cDNA 4930511F01 gene [Source:MGI Symbo	0.63813
ENSMUSG00000047507	Baiap3	BAI1-associated protein 3 [Source:MGI Symbol;Acc	0.63826
ENSMUSG00000022032	Scara5	scavenger receptor class A, member 5 (putative) [S	0.63833
ENSMUSG00000093634	Gm10860	predicted gene 10860 [Source:MGI Symbol;Acc:MG	0.63834
ENSMUSG00000004383	Large	like-glycosyltransferase [Source:MGI Symbol;Acc:M	0.63837
ENSMUSG00000019813	Cep57l1	centrosomal protein 57-like 1 [Source:MGI Symbol;	0.63896
ENSMUSG00000053841	Txlna	taxilin alpha [Source:MGI Symbol;Acc:MGI:105968]	0.63913
ENSMUSG00000080972	Gm16061	predicted gene 16061 [Source:MGI Symbol;Acc:MG	0.63915
ENSMUSG00000030538	Cib1	calcium and integrin binding 1 (calmyrin) [Source:M	0.63921
ENSMUSG00000024273	2700062C07Rik	RIKEN cDNA 2700062C07 gene [Source:MGI Symbo	0.63925
ENSMUSG00000098145	Gm26936	predicted gene, 26936 [Source:MGI Symbol;Acc:MG	0.63927
ENSMUSG00000090500	Gm17057	predicted gene 17057 [Source:MGI Symbol;Acc:MG	0.63933
ENSMUSG00000090939	Gm17147	predicted gene 17147 [Source:MGI Symbol;Acc:MG	0.63943
ENSMUSG00000022108	Itm2b	integral membrane protein 2B [Source:MGI Symbol	0.63946
ENSMUSG00000004748	Mtfp1	mitochondrial fission process 1 [Source:MGI Symbo	0.63952
ENSMUSG00000029763	Exoc4	exocyst complex component 4 [Source:MGI Symbo	0.63956
ENSMUSG00000039200	Atf7ip2	activating transcription factor 7 interacting protein	0.63978
ENSMUSG00000001729	Akt1	thymoma viral proto-oncogene 1 [Source:MGI Sym	0.63988
ENSMUSG00000048234	Rnf149	ring finger protein 149 [Source:MGI Symbol;Acc:MG	0.63989
ENSMUSG00000091176	Gm17678	predicted gene, 17678 [Source:MGI Symbol;Acc:MG	0.6399
ENSMUSG00000036591	Arhgap21	Rho GTPase activating protein 21 [Source:MGI Sym	0.63998
ENSMUSG00000035394	Ccdc11	coiled-coil domain containing 11 [Source:MGI Symb	0.64007
ENSMUSG00000015335	Zdhhc12	zinc finger, DHHC domain containing 12 [Source:MG	0.64008
ENSMUSG00000073551	Spink13	serine peptidase inhibitor, Kazal type 13 [Source:M	0.64012
ENSMUSG00000009394	Syn2	synapsin II [Source:MGI Symbol;Acc:MGI:103020]	0.6402
ENSMUSG00000028044	Cks1b	CDC28 protein kinase 1b [Source:MGI Symbol;Acc:M	0.6402
ENSMUSG00000028919	Arhgef19	Rho guanine nucleotide exchange factor (GEF) 19 [S	0.64029
ENSMUSG00000020720	Psmd12	proteasome (prosome, macropain) 26S subunit, no	0.64041
ENSMUSG00000029378	Areg	amphiregulin [Source:MGI Symbol;Acc:MGI:88068]	0.64057
ENSMUSG00000021268	Meg3	maternally expressed 3 [Source:MGI Symbol;Acc:M	0.64066
ENSMUSG00000006050	Sra1	steroid receptor RNA activator 1 [Source:MGI Symb	0.64071
ENSMUSG00000070385	Ampd1	adenosine monophosphate deaminase 1 [Source:M	0.64071
ENSMUSG00000097757	Gm17518	predicted gene, 17518 [Source:MGI Symbol;Acc:MG	0.64071
ENSMUSG00000007080	Pole	polymerase (DNA directed), epsilon [Source:MGI Sy	0.64078
ENSMUSG00000019822	Smpd2	sphingomyelin phosphodiesterase 2, neutral [Sourc	0.64086
ENSMUSG00000075520	Gm13318	predicted gene 13318 [Source:MGI Symbol;Acc:MG	0.64092
ENSMUSG00000038402	Foxf2	forkhead box F2 [Source:MGI Symbol;Acc:MGI:1347	0.64094
ENSMUSG00000022766	Serpind1	serine (or cysteine) peptidase inhibitor, clade D, me	0.64099

ENSMUSG00000091227	Gm3755	predicted gene 3755 [Source:MGI Symbol;Acc:MGI:	0.64113
ENSMUSG00000036639	Nudt1	nudix (nucleoside diphosphate linked moiety X)-typ	0.64114
ENSMUSG00000030337	Vamp1	vesicle-associated membrane protein 1 [Source:MG	0.64126
ENSMUSG00000026482	Rgl1	ral guanine nucleotide dissociation stimulator,-like	0.64129
ENSMUSG00000026750	Psmb7	proteasome (prosome, macropain) subunit, beta ty	0.64136
ENSMUSG00000078765	U2af1l4	U2 small nuclear RNA auxiliary factor 1-like 4 [Sour	0.64138
ENSMUSG00000029070	Mxra8	matrix-remodelling associated 8 [Source:MGI Symb	0.6415
ENSMUSG00000081400	Gm13680	predicted gene 13680 [Source:MGI Symbol;Acc:MG	0.6415
ENSMUSG00000051346	Spryd4	SPRY domain containing 4 [Source:MGI Symbol;Acc	0.64151
ENSMUSG00000029576	Radil	Ras association and DIL domains [Source:MGI Symb	0.64164
ENSMUSG00000090131	Gm16162	predicted gene 16162 [Source:MGI Symbol;Acc:MG	0.64168
ENSMUSG00000086458	Gm2639	predicted gene 2639 [Source:MGI Symbol;Acc:MGI:	0.64171
ENSMUSG00000049106	Dcaf5	DDB1 and CUL4 associated factor 5 [Source:MGI Sy	0.64199
ENSMUSG00000098146	Gm26935	predicted gene, 26935 [Source:MGI Symbol;Acc:MG	0.64199
ENSMUSG00000001054	Rmnd5b	required for meiotic nuclear division 5 homolog B (S	0.64208
ENSMUSG00000050966	Lin28a	lin-28 homolog A (C. elegans) [Source:MGI Symbol;	0.64208
ENSMUSG00000029778	Adcyap1r1	adenylate cyclase activating polypeptide 1 receptor	0.64212
ENSMUSG00000019864	Rtn4ip1	reticulon 4 interacting protein 1 [Source:MGI Symb	0.64222
ENSMUSG00000022992	Kansl2	KAT8 regulatory NSL complex subunit 2 [Source:MG	0.64236
ENSMUSG00000048149	Gm12663	predicted gene 12663 [Source:MGI Symbol;Acc:MG	0.64238
ENSMUSG00000021313	Ryr2	ryanodine receptor 2, cardiac [Source:MGI Symbol;	0.6424
ENSMUSG00000001123	Lgals9	lectin, galactose binding, soluble 9 [Source:MGI Syn	0.64243
ENSMUSG00000061983	Rps12	ribosomal protein S12 [Source:MGI Symbol;Acc:MG	0.64246
ENSMUSG00000070282	3000002C10Rik	RIKEN cDNA 3000002C10 gene [Source:MGI Symbo	0.64256
ENSMUSG00000028464	Tpm2	tropomyosin 2, beta [Source:MGI Symbol;Acc:MGI:	0.6426
ENSMUSG00000018428	Akap1	A kinase (PRKA) anchor protein 1 [Source:MGI Sym	0.64262
ENSMUSG00000091405	Hist2h4	histone cluster 2, H4 [Source:MGI Symbol;Acc:MGI:	0.64263
ENSMUSG00000091661	Gm17021	predicted gene 17021 [Source:MGI Symbol;Acc:MG	0.64276
ENSMUSG00000087198	Gm13097	predicted gene 13097 [Source:MGI Symbol;Acc:MG	0.64281
ENSMUSG00000006411	Pvrl4	poliovirus receptor-related 4 [Source:MGI Symbol;A	0.64294
ENSMUSG00000062742	Gm5239	predicted pseudogene 5239 [Source:MGI Symbol;A	0.64295
ENSMUSG00000097350	4732491K20Rik	RIKEN cDNA 4732491K20 gene [Source:MGI Symbo	0.64303
ENSMUSG00000097994	Gm26982	predicted gene, 26982 [Source:MGI Symbol;Acc:MG	0.64304
ENSMUSG00000085208	4632419I22Rik	RIKEN cDNA 4632419I22 gene [Source:MGI Symbol	0.64309
ENSMUSG00000097784	A430105J06Rik	RIKEN cDNA A430105J06 gene [Source:MGI Symbo	0.64332
ENSMUSG00000015656	Hspa8	heat shock protein 8 [Source:MGI Symbol;Acc:MGI:	0.64335
ENSMUSG00000079652	Fam71f2	family with sequence similarity 71, member F2 [Sou	0.64336
ENSMUSG00000032020	Ubash3b	ubiquitin associated and SH3 domain containing, B	0.64342
ENSMUSG00000037892	Pcdh18	protocadherin 18 [Source:MGI Symbol;Acc:MGI:192	0.64358
ENSMUSG00000020142	Slc1a4	solute carrier family 1 (glutamate/neutral amino ac	0.64371
ENSMUSG00000026676	Ccdc3	coiled-coil domain containing 3 [Source:MGI Symbo	0.64377
ENSMUSG00000090952	Gm17251	predicted gene, 17251 [Source:MGI Symbol;Acc:MG	0.64381
ENSMUSG00000082179	Gm11407	predicted gene 11407 [Source:MGI Symbol;Acc:MG	0.64393
ENSMUSG00000090050	Gm16339	predicted gene 16339 [Source:MGI Symbol;Acc:MG	0.64395
ENSMUSG00000073000	Gm10451	predicted gene 10451 [Source:MGI Symbol;Acc:MG	0.64399
ENSMUSG00000054910	4931415C17Rik	RIKEN cDNA 4931415C17 gene [Source:MGI Symbo	0.64405
ENSMUSG00000022895	Ets2	E26 avian leukemia oncogene 2, 3' domain [Source:	0.64406

ENSMUSG00000074635	3110070M22Rik	RIKEN cDNA 3110070M22 gene [Source:MGI Symbo	0.64416
ENSMUSG00000045365	Rbm15b	RNA binding motif protein 15B [Source:MGI Symbo	0.64425
ENSMUSG00000051390	Zbtb22	zinc finger and BTB domain containing 22 [Source:N	0.64439
ENSMUSG00000097020	Gm26613	predicted gene, 26613 [Source:MGI Symbol;Acc:MC	0.64443
ENSMUSG00000023348	Trip6	thyroid hormone receptor interactor 6 [Source:MG	0.64447
ENSMUSG00000037725	Ckap2	cytoskeleton associated protein 2 [Source:MGI Sym	0.64462
ENSMUSG00000070720	Tmem200b	transmembrane protein 200B [Source:MGI Symbol;	0.64474
ENSMUSG00000059326	Csf2ra	colony stimulating factor 2 receptor, alpha, low-affi	0.64488
ENSMUSG00000097657	Gm7389	predicted gene 7389 [Source:MGI Symbol;Acc:MGI:	0.64495
ENSMUSG00000002416	Ndufb2	NADH dehydrogenase (ubiquinone) 1 beta subcom	0.64498
ENSMUSG00000034641	Cd300ld	CD300 molecule-like family member d [Source:MGI	0.64509
ENSMUSG00000093231	Gm25297	predicted gene, 25297 [Source:MGI Symbol;Acc:MC	0.64509
ENSMUSG00000041596	Nlrp5-ps	NLR family, pyrin domain containing 5, pseudogene	0.64511
ENSMUSG00000022342	Kcnv1	potassium channel, subfamily V, member 1 [Source	0.64517
ENSMUSG00000097442	Gm26632	predicted gene, 26632 [Source:MGI Symbol;Acc:MC	0.64521
ENSMUSG00000087142	Gm12454	predicted gene 12454 [Source:MGI Symbol;Acc:MG	0.64522
ENSMUSG00000005553	Atp4a	ATPase, H+/K+ exchanging, gastric, alpha polypepti	0.64523
ENSMUSG00000022801	Lrch3	leucine-rich repeats and calponin homology (CH) do	0.64532
ENSMUSG00000082815	Gm5678	predicted gene 5678 [Source:MGI Symbol;Acc:MGI:	0.64534
ENSMUSG00000047462	A530099J19Rik	RIKEN cDNA A530099J19 gene [Source:MGI Symbo	0.64536
ENSMUSG00000037813	D630003M21Rik	RIKEN cDNA D630003M21 gene [Source:MGI Symb	0.64549
ENSMUSG00000022021	Diap3	diaphanous homolog 3 (Drosophila) [Source:MGI Sy	0.64554
ENSMUSG00000087066	Gm15518	predicted gene 15518 [Source:MGI Symbol;Acc:MG	0.64567
ENSMUSG00000086839	Gm11973	predicted gene 11973 [Source:MGI Symbol;Acc:MG	0.64569
ENSMUSG00000003882	Il7r	interleukin 7 receptor [Source:MGI Symbol;Acc:MG	0.64571
ENSMUSG00000094594	Tpd52-ps	tumor protein D52, pseudogene [Source:MGI Symb	0.64573
ENSMUSG00000029557	Ftsj2	FtsJ homolog 2 (E. coli) [Source:MGI Symbol;Acc:M	0.64578
ENSMUSG00000097414	B130046B21Rik	RIKEN cDNA B130046B21 gene [Source:MGI Symbo	0.6458
ENSMUSG00000021079	Timm9	translocase of inner mitochondrial membrane 9 [So	0.64583
ENSMUSG00000026857	Ntmt1	N-terminal Xaa-Pro-Lys N-methyltransferase 1 [Sou	0.64595
ENSMUSG00000023960	Enpp5	ectonucleotide pyrophosphatase/phosphodiesteras	0.64608
ENSMUSG00000090223	Pcp4	Purkinje cell protein 4 [Source:MGI Symbol;Acc:MG	0.64609
ENSMUSG00000025129	Ppp1r27	protein phosphatase 1, regulatory subunit 27 [Sour	0.64611
ENSMUSG00000032114	Slc37a4	solute carrier family 37 (glucose-6-phosphate trans	0.64619
ENSMUSG00000086121	Gm16068	predicted gene 16068 [Source:MGI Symbol;Acc:MG	0.64622
ENSMUSG00000031145	Prickle3	prickle homolog 3 (Drosophila) [Source:MGI Symbo	0.64626
ENSMUSG00000021565	Slc6a19	solute carrier family 6 (neurotransmitter transporte	0.64627
ENSMUSG00000043909	Trp53bp1	transformation related protein 53 binding protein 1	0.64631
ENSMUSG00000004043	Stat5a	signal transducer and activator of transcription 5A	0.64633
ENSMUSG00000005505	Kbtbd4	kelch repeat and BTB (POZ) domain containing 4 [S	0.64654
ENSMUSG00000032192	Gnb5	guanine nucleotide binding protein (G protein), bet	0.64682
ENSMUSG00000052331	Ankrd44	ankyrin repeat domain 44 [Source:MGI Symbol;Acc	0.64701
ENSMUSG00000085658	Gm15704	predicted gene 15704 [Source:MGI Symbol;Acc:MG	0.64701
ENSMUSG00000063447	Ube2d2b	ubiquitin-conjugating enzyme E2D 2B [Source:MGI	0.64711
ENSMUSG00000028328	Tmod1	tropomodulin 1 [Source:MGI Symbol;Acc:MGI:9877	0.64724
ENSMUSG00000031224	Magee2	melanoma antigen, family E, 2 [Source:MGI Symbol	0.64737
ENSMUSG00000031143	Ccdc22	coiled-coil domain containing 22 [Source:MGI Symb	0.64754

ENSMUSG00000075327	Zbtb2	zinc finger and BTB domain containing 2 [Source:M	0.64754
ENSMUSG00000044811	AF251705	cDNA sequence AF251705 [Source:MGI Symbol;Acc	0.64773
ENSMUSG00000082110	Gm11196	predicted gene 11196 [Source:MGI Symbol;Acc:MG	0.64774
ENSMUSG00000038957	Edc3	enhancer of mRNA decapping 3 homolog (S. cerevis	0.64782
ENSMUSG00000087177	E130307A14Rik	RIKEN cDNA E130307A14 gene [Source:MGI Symbo	0.64797
ENSMUSG00000062761	Zfp512	zinc finger protein 512 [Source:MGI Symbol;Acc:M	0.64799
ENSMUSG00000025086	Trub1	TruB pseudouridine (psi) synthase homolog 1 (E. co	0.64813
ENSMUSG00000026663	Atf6	activating transcription factor 6 [Source:MGI Symb	0.64815
ENSMUSG00000015889	Lta4h	leukotriene A4 hydrolase [Source:MGI Symbol;Acc:	0.64816
ENSMUSG00000031312	Itgb1bp2	integrin beta 1 binding protein 2 [Source:MGI Symb	0.64818
ENSMUSG00000006567	Atp7b	ATPase, Cu++ transporting, beta polypeptide [Sour	0.64821
ENSMUSG00000036873	2410004B18Rik	RIKEN cDNA 2410004B18 gene [Source:MGI Symbo	0.64842
ENSMUSG00000029613	Eif2ak1	eukaryotic translation initiation factor 2 alpha kinas	0.64848
ENSMUSG00000000244	Tspan32	tetraspanin 32 [Source:MGI Symbol;Acc:MGI:1350	0.64853
ENSMUSG00000020599	Rgs9	regulator of G-protein signaling 9 [Source:MGI Sym	0.64872
ENSMUSG00000091534	Gm17195	predicted gene 17195 [Source:MGI Symbol;Acc:MG	0.64873
ENSMUSG00000064032	Gm10143	predicted gene 10143 [Source:MGI Symbol;Acc:MG	0.6488
ENSMUSG00000057777	Mab21l2	mab-21-like 2 (C. elegans) [Source:MGI Symbol;Acc	0.64881
ENSMUSG00000074664	A830092H15Rik	RIKEN cDNA A830092H15 gene [Source:MGI Symbo	0.64887
ENSMUSG00000051652	Lrrc3	leucine rich repeat containing 3 [Source:MGI Symb	0.64892
ENSMUSG00000030067	Foxp1	forkhead box P1 [Source:MGI Symbol;Acc:MGI:191	0.64899
ENSMUSG00000031107	Rbmx2	RNA binding motif protein, X-linked 2 [Source:MGI	0.64903
ENSMUSG00000037942	Crp	C-reactive protein, pentraxin-related [Source:MGI S	0.64903
ENSMUSG00000078970	Wdr92	WD repeat domain 92 [Source:MGI Symbol;Acc:MG	0.64915
ENSMUSG00000047344	Lancl3	LanC lantibiotic synthetase component C-like 3 (ba	0.64922
ENSMUSG00000025425	St8sia5	ST8 alpha-N-acetyl-neuraminide alpha-2,8-sialyltra	0.64923
ENSMUSG00000053950	Adnp2	ADNP homeobox 2 [Source:MGI Symbol;Acc:MGI:2	0.6493
ENSMUSG00000039079	Trhr2	thyrotropin releasing hormone receptor 2 [Source:	0.64945
ENSMUSG00000097692	A230060F14Rik	RIKEN cDNA A230060F14 gene [Source:MGI Symbo	0.64945
ENSMUSG00000087698	Gm13031	predicted gene 13031 [Source:MGI Symbol;Acc:MG	0.64952
ENSMUSG00000085478	Gm11851	predicted gene 11851 [Source:MGI Symbol;Acc:MG	0.64955
ENSMUSG00000092210	A930009A15Rik	RIKEN cDNA A930009A15 gene [Source:MGI Symbo	0.64957
ENSMUSG00000056771	Gm10010	predicted gene 10010 [Source:MGI Symbol;Acc:MG	0.64982
ENSMUSG00000043061	Tmem18	transmembrane protein 18 [Source:MGI Symbol;Ac	0.6499
ENSMUSG00000021804	Rgr	retinal G protein coupled receptor [Source:MGI Sy	0.64999
ENSMUSG00000026187	Xrcc5	X-ray repair complementing defective repair in Chi	0.65001
ENSMUSG00000073590	3222401L13Rik	RIKEN cDNA 3222401L13 gene [Source:MGI Symbo	0.65001
ENSMUSG00000041710	Trpc5	transient receptor potential cation channel, subfam	0.65005
ENSMUSG00000085454	Gm16320	predicted gene 16320 [Source:MGI Symbol;Acc:MG	0.65006
ENSMUSG00000026848	Tor1b	torsin family 1, member B [Source:MGI Symbol;Acc	0.65007
ENSMUSG00000020258	Glyctk	glycerate kinase [Source:MGI Symbol;Acc:MGI:244	0.65025
ENSMUSG00000040952	Rps19	ribosomal protein S19 [Source:MGI Symbol;Acc:MG	0.65025
ENSMUSG00000044317	Gpr4	G protein-coupled receptor 4 [Source:MGI Symbol;	0.6504
ENSMUSG00000087163	Gm16230	predicted gene 16230 [Source:MGI Symbol;Acc:MG	0.6505
ENSMUSG00000078676	Casc3	cancer susceptibility candidate 3 [Source:MGI Symb	0.65054
ENSMUSG00000020604	Arsg	arylsulfatase G [Source:MGI Symbol;Acc:MGI:1921	0.65055
ENSMUSG00000025358	Cdk2	cyclin-dependent kinase 2 [Source:MGI Symbol;Acc	0.6506

ENSMUSG00000041343	Ankrd42	ankyrin repeat domain 42 [Source:MGI Symbol;Acc	0.65065
ENSMUSG00000071230	Npw	neuropeptide W [Source:MGI Symbol;Acc:MGI:268	0.65066
ENSMUSG00000029072	Tas1r3	taste receptor, type 1, member 3 [Source:MGI Sym	0.65074
ENSMUSG00000028073	Pear1	platelet endothelial aggregation receptor 1 [Source	0.65076
ENSMUSG00000030584	Dpf1	D4, zinc and double PHD fingers family 1 [Source:M	0.65079
ENSMUSG00000028347	Tmeff1	transmembrane protein with EGF-like and two follis	0.65089
ENSMUSG00000087020	Gm16283	predicted gene 16283 [Source:MGI Symbol;Acc:MG	0.65092
ENSMUSG00000070426	Rnf121	ring finger protein 121 [Source:MGI Symbol;Acc:MG	0.65097
ENSMUSG00000035239	Neu3	neuraminidase 3 [Source:MGI Symbol;Acc:MGI:135	0.65098
ENSMUSG00000031782	Coq9	coenzyme Q9 homolog (yeast) [Source:MGI Symbo	0.651
ENSMUSG00000043843	Tmem145	transmembrane protein 145 [Source:MGI Symbol;A	0.65104
ENSMUSG00000087390	Gm7598	predicted gene 7598 [Source:MGI Symbol;Acc:MGI:	0.65112
ENSMUSG00000027010	Slc25a12	solute carrier family 25 (mitochondrial carrier, Arala	0.65119
ENSMUSG00000030742	Lat	linker for activation of T cells [Source:MGI Symbol;A	0.65145
ENSMUSG00000038876	Rnf146	ring finger protein 146 [Source:MGI Symbol;Acc:MG	0.65147
ENSMUSG00000020523	Fam114a2	family with sequence similarity 114, member A2 [So	0.65154
ENSMUSG00000097867	1110054M08Rik	RIKEN cDNA 1110054M08 gene [Source:MGI Symbo	0.65156
ENSMUSG00000037801	Iqch	IQ motif containing H [Source:MGI Symbol;Acc:MG	0.65163
ENSMUSG00000057715	A830018L16Rik	RIKEN cDNA A830018L16 gene [Source:MGI Symbo	0.6517
ENSMUSG00000079071	Gm14085	predicted gene 14085 [Source:MGI Symbol;Acc:MG	0.65194
ENSMUSG00000038417	Fig4	FIG4 homolog (S. cerevisiae) [Source:MGI Symbol;A	0.65201
ENSMUSG00000028466	Creb3	cAMP responsive element binding protein 3 [Source	0.65205
ENSMUSG00000078554	Fam229a	family with sequence similarity 229, member A [Sou	0.65215
ENSMUSG00000025193	Cutc	cutC copper transporter homolog (E.coli) [Source:M	0.65217
ENSMUSG00000045102	Poln	DNA polymerase N [Source:MGI Symbol;Acc:MGI:2	0.65217
ENSMUSG00000044475	Ascc1	activating signal cointegrator 1 complex subunit 1 [	0.65229
ENSMUSG00000028626	Col9a2	collagen, type IX, alpha 2 [Source:MGI Symbol;Acc:	0.65231
ENSMUSG00000082100	Glns-ps1	glutamine synthetase pseudogene 1 [Source:MGI S	0.65244
ENSMUSG00000022761	Lztr1	leucine-zipper-like transcriptional regulator, 1 [Sou	0.65248
ENSMUSG00000040749	Siah1b	seven in absentia 1B [Source:MGI Symbol;Acc:MGI:	0.65259
ENSMUSG00000024030	Abcg1	ATP-binding cassette, sub-family G (WHITE), memb	0.6526
ENSMUSG00000028789	Adc	arginine decarboxylase [Source:MGI Symbol;Acc:M	0.65262
ENSMUSG00000060600	Eno3	enolase 3, beta muscle [Source:MGI Symbol;Acc:M	0.65268
ENSMUSG00000085564	Gm12198	predicted gene 12198 [Source:MGI Symbol;Acc:MG	0.65272
ENSMUSG00000002057	Foxn1	forkhead box N1 [Source:MGI Symbol;Acc:MGI:102	0.65274
ENSMUSG00000044786	Zfp36	zinc finger protein 36 [Source:MGI Symbol;Acc:MGI	0.65277
ENSMUSG00000073608	Gm6086	predicted gene 6086 [Source:MGI Symbol;Acc:MGI:	0.65278
ENSMUSG00000027406	Idh3b	isocitrate dehydrogenase 3 (NAD+) beta [Source:M	0.65279
ENSMUSG00000009406	Elk1	ELK1, member of ETS oncogene family [Source:MGI	0.65285
ENSMUSG00000032514	Ttc21a	tetratricopeptide repeat domain 21A [Source:MGI S	0.65297
ENSMUSG00000022335	Zfat	zinc finger and AT hook domain containing [Source:	0.65312
ENSMUSG00000027674	Pex5l	peroxisomal biogenesis factor 5-like [Source:MGI Sy	0.65318
ENSMUSG00000028033	Kcnq5	potassium voltage-gated channel, subfamily Q, mer	0.65322
ENSMUSG00000043789	Vwce	von Willebrand factor C and EGF domains [Source:M	0.65328
ENSMUSG00000086708	Gm15577	predicted gene 15577 [Source:MGI Symbol;Acc:MG	0.65328
ENSMUSG00000022178	Ajuba	ajuba LIM protein [Source:MGI Symbol;Acc:MGI:13	0.65329
ENSMUSG00000042408	Zmym6	zinc finger, MYM-type 6 [Source:MGI Symbol;Acc:M	0.65331

ENSMUSG00000039057	Myo16	myosin XVI [Source:MGI Symbol;Acc:MGI:2685951]	0.65337
ENSMUSG00000055676	Gm5069	predicted pseudogene 5069 [Source:MGI Symbol;A	0.65337
ENSMUSG00000035041	Creb3l3	cAMP responsive element binding protein 3-like 3 [	0.65341
ENSMUSG00000032526	Deb1	differentially expressed in B16F10 1 [Source:MGI Sy	0.65343
ENSMUSG00000085492	Trmt61b	tRNA methyltransferase 61B [Source:MGI Symbol;A	0.65372
ENSMUSG00000092301	Gm6887	predicted gene 6887 [Source:MGI Symbol;Acc:MGI:	0.65372
ENSMUSG00000042041	2010003K11Rik	RIKEN cDNA 2010003K11 gene [Source:MGI Symbo	0.65373
ENSMUSG00000026615	Eprs	glutamyl-prolyl-tRNA synthetase [Source:MGI Symb	0.65376
ENSMUSG00000047821	Trim16	tripartite motif-containing 16 [Source:MGI Symbol;	0.65378
ENSMUSG00000025266	Gnl3l	guanine nucleotide binding protein-like 3 (nucleola	0.65379
ENSMUSG00000063873	Slc24a3	solute carrier family 24 (sodium/potassium/calcium	0.65391
ENSMUSG00000064288	Hist1h4k	histone cluster 1, H4k [Source:MGI Symbol;Acc:MG	0.65391
ENSMUSG00000041870	Ankrd13a	ankyrin repeat domain 13a [Source:MGI Symbol;Ac	0.65392
ENSMUSG00000097583	6430590A07Rik	RIKEN cDNA 6430590A07 gene [Source:MGI Symbo	0.65406
ENSMUSG00000026240	Cops7b	COP9 (constitutive photomorphogenic) homolog, s	0.65407
ENSMUSG00000026603	Smyd2	SET and MYND domain containing 2 [Source:MGI Sy	0.65407
ENSMUSG00000041629	Fam104a	family with sequence similarity 104, member A [So	0.65411
ENSMUSG00000022885	St6gal1	beta galactoside alpha 2,6 sialyltransferase 1 [Sour	0.65412
ENSMUSG00000078995	Zfp456	zinc finger protein 456 [Source:MGI Symbol;Acc:M	0.65414
ENSMUSG00000097655	Gm26713	predicted gene, 26713 [Source:MGI Symbol;Acc:M	0.6542
ENSMUSG00000050270	Tmem220	transmembrane protein 220 [Source:MGI Symbol;A	0.65421
ENSMUSG00000090191	9230105E05Rik	RIKEN cDNA 9230105E05 gene [Source:MGI Symbo	0.65422
ENSMUSG00000053988	Gm9931	predicted gene 9931 [Source:MGI Symbol;Acc:MGI:	0.65423
ENSMUSG00000081480	Gm13608	predicted gene 13608 [Source:MGI Symbol;Acc:MG	0.65428
ENSMUSG00000056770	Setd3	SET domain containing 3 [Source:MGI Symbol;Acc:M	0.65447
ENSMUSG00000063488	Zkscan7	zinc finger with KRAB and SCAN domains 7 [Source:	0.65449
ENSMUSG00000052430	Bmpr1b	bone morphogenetic protein receptor, type 1B [Sou	0.65462
ENSMUSG00000074578	1500012F01Rik	RIKEN cDNA 1500012F01 gene [Source:MGI Symbo	0.65475
ENSMUSG00000059659	Gm10069	predicted gene 10069 [Source:MGI Symbol;Acc:MG	0.65478
ENSMUSG00000083152	Apc-ps1	adenomatosis polyposis coli, pseudogene 1 [Source	0.65481
ENSMUSG00000053475	Tnfaip6	tumor necrosis factor alpha induced protein 6 [Sou	0.65489
ENSMUSG00000020840	Blmh	bleomycin hydrolase [Source:MGI Symbol;Acc:MGI	0.65499
ENSMUSG00000041272	Tox	thymocyte selection-associated high mobility group	0.65504
ENSMUSG00000046415	B430212C06Rik	RIKEN cDNA B430212C06 gene [Source:MGI Symbo	0.65505
ENSMUSG00000074063	Osgin1	oxidative stress induced growth inhibitor 1 [Source	0.65507
ENSMUSG00000032555	Topbp1	topoisomerase (DNA) II binding protein 1 [Source:M	0.65508
ENSMUSG00000085030	2810455O05Rik	RIKEN cDNA 2810455O05 gene [Source:MGI Symbo	0.65509
ENSMUSG00000087181	Gm13570	predicted gene 13570 [Source:MGI Symbol;Acc:MG	0.65515
ENSMUSG00000020010	Vnn3	vanin 3 [Source:MGI Symbol;Acc:MGI:1347055]	0.65518
ENSMUSG00000038416	Cdc16	CDC16 cell division cycle 16 [Source:MGI Symbol;Ac	0.65528
ENSMUSG00000023004	Tuba1b	tubulin, alpha 1B [Source:MGI Symbol;Acc:MGI:107	0.65531
ENSMUSG00000046876	Atxn1	ataxin 1 [Source:MGI Symbol;Acc:MGI:104783]	0.65533
ENSMUSG00000001034	Mapk7	mitogen-activated protein kinase 7 [Source:MGI Sy	0.65542
ENSMUSG00000020333	Acsl6	acyl-CoA synthetase long-chain family member 6 [S	0.65545
ENSMUSG00000045004	Spata21	spermatogenesis associated 21 [Source:MGI Symbo	0.65555
ENSMUSG00000025153	Fasn	fatty acid synthase [Source:MGI Symbol;Acc:MGI:9	0.65572
ENSMUSG00000081817	Gm13590	predicted gene 13590 [Source:MGI Symbol;Acc:MG	0.65582

ENSMUSG00000073422	H2-Ke6	H2-K region expressed gene 6 [Source:MGI Symbol;	0.65598
ENSMUSG00000023456	Tpi1	triosephosphate isomerase 1 [Source:MGI Symbol;A	0.65601
ENSMUSG00000057378	Ryr3	ryanodine receptor 3 [Source:MGI Symbol;Acc:MGI	0.65616
ENSMUSG00000031998	1700128F08Rik	RIKEN cDNA 1700128F08 gene [Source:MGI Symbo	0.65618
ENSMUSG00000024131	Slc3a1	solute carrier family 3, member 1 [Source:MGI Sym	0.65621
ENSMUSG00000041415	Dicer1	dicer 1, ribonuclease type III [Source:MGI Symbol;A	0.65645
ENSMUSG00000015966	Il17rb	interleukin 17 receptor B [Source:MGI Symbol;Acc:	0.65651
ENSMUSG00000035024	Ncapd3	non-SMC condensin II complex, subunit D3 [Source	0.65651
ENSMUSG00000025236	Adpgk	ADP-dependent glucokinase [Source:MGI Symbol;A	0.65667
ENSMUSG00000002661	Alkbh7	alkB, alkylation repair homolog 7 (E. coli) [Source:M	0.65674
ENSMUSG00000029675	Eln	elastin [Source:MGI Symbol;Acc:MGI:95317]	0.65681
ENSMUSG00000054123	Gm9936	predicted gene 9936 [Source:MGI Symbol;Acc:MGI:	0.65697
ENSMUSG00000085156	Gm11974	predicted gene 11974 [Source:MGI Symbol;Acc:MG	0.657
ENSMUSG00000020846	Fam101b	family with sequence similarity 101, member B [Sou	0.65711
ENSMUSG00000097157	Gm26512	predicted gene, 26512 [Source:MGI Symbol;Acc:MG	0.65716
ENSMUSG00000087433	Gm14167	predicted gene 14167 [Source:MGI Symbol;Acc:MG	0.65732
ENSMUSG00000097644	Gm26862	predicted gene, 26862 [Source:MGI Symbol;Acc:MG	0.65748
ENSMUSG00000050064	Zfp697	zinc finger protein 697 [Source:MGI Symbol;Acc:MG	0.65755
ENSMUSG00000025226	Fbxl15	F-box and leucine-rich repeat protein 15 [Source:M	0.65758
ENSMUSG00000006095	Tbcb	tubulin folding cofactor B [Source:MGI Symbol;Acc:	0.65782
ENSMUSG00000097891	Gm3650	predicted gene 3650 [Source:MGI Symbol;Acc:MGI:	0.65782
ENSMUSG00000038507	Parp12	poly (ADP-ribose) polymerase family, member 12 [S	0.65788
ENSMUSG00000040620	Dhx33	DEAH (Asp-Glu-Ala-His) box polypeptide 33 [Source	0.65788
ENSMUSG00000094646	Gm10417	predicted gene 10417 [Source:MGI Symbol;Acc:MG	0.65801
ENSMUSG00000086536	Gm12264	predicted gene 12264 [Source:MGI Symbol;Acc:MG	0.65805
ENSMUSG00000091381	Vmn2r83	vomeronasal 2, receptor 83 [Source:MGI Symbol;Ac	0.65806
ENSMUSG00000086774	Gm11915	predicted gene 11915 [Source:MGI Symbol;Acc:MG	0.65808
ENSMUSG00000022898	Dscr3	Down syndrome critical region gene 3 [Source:MGI	0.65811
ENSMUSG00000020021	Fgd6	FYVE, RhoGEF and PH domain containing 6 [Source	0.65814
ENSMUSG00000050796	B3galt6	UDP-Gal:betaGal beta 1,3-galactosyltransferase, po	0.65861
ENSMUSG00000025228	Actr1a	ARP1 actin-related protein 1A, centractin alpha [So	0.65863
ENSMUSG00000085039	Gm15927	predicted gene 15927 [Source:MGI Symbol;Acc:MG	0.65881
ENSMUSG00000087523	Gm12319	predicted gene 12319 [Source:MGI Symbol;Acc:MG	0.65897
ENSMUSG00000044287	Nrn1l	neuritin 1-like [Source:MGI Symbol;Acc:MGI:24436	0.6591
ENSMUSG00000092540	Gm8738	predicted gene 8738 [Source:MGI Symbol;Acc:MGI:	0.65911
ENSMUSG00000054027	Nt5dc3	5'-nucleotidase domain containing 3 [Source:MGI S	0.65916
ENSMUSG00000090077	Lime1	Lck interacting transmembrane adaptor 1 [Source:M	0.65916
ENSMUSG00000027618	Nfs1	nitrogen fixation gene 1 (S. cerevisiae) [Source:MGI	0.6592
ENSMUSG00000065939	Snora2b	small nucleolar RNA, H/ACA box 2B [Source:MGI Sy	0.6592
ENSMUSG00000097037	Gm26818	predicted gene, 26818 [Source:MGI Symbol;Acc:MG	0.65937
ENSMUSG00000027489	Necab3	N-terminal EF-hand calcium binding protein 3 [Sour	0.65942
ENSMUSG00000024544	Ldlrad4	low density lipoprotein receptor class A domain co	0.65945
ENSMUSG00000070031	Sp140	Sp140 nuclear body protein [Source:MGI Symbol;Ac	0.65949
ENSMUSG00000021666	Gfm2	G elongation factor, mitochondrial 2 [Source:MGI S	0.65957
ENSMUSG00000000416	Cttnbp2	cortactin binding protein 2 [Source:MGI Symbol;Acc	0.65976
ENSMUSG00000006546	Cryba2	crystallin, beta A2 [Source:MGI Symbol;Acc:MGI:10	0.65978
ENSMUSG00000085709	Gm14211	predicted gene 14211 [Source:MGI Symbol;Acc:MG	0.66005

ENSMUSG00000047907	Tshz2	teashirt zinc finger family member 2 [Source:MGI S	0.66013
ENSMUSG00000044068	Zrsr1	zinc finger (CCCH type), RNA binding motif and seri	0.66014
ENSMUSG00000041161	Otud3	OTU domain containing 3 [Source:MGI Symbol;Acc:	0.66021
ENSMUSG00000026870	Cutal	cutA divalent cation tolerance homolog-like [Source	0.66028
ENSMUSG00000039307	Hexdc	hexosaminidase (glycosyl hydrolase family 20, catal	0.66048
ENSMUSG00000040272	Accs	1-aminocyclopropane-1-carboxylate synthase (non-	0.66054
ENSMUSG00000023885	Thbs2	thrombospondin 2 [Source:MGI Symbol;Acc:MGI:98	0.6606
ENSMUSG00000037361	0610009D07Rik	RIKEN cDNA 0610009D07 gene [Source:MGI Symbo	0.66063
ENSMUSG00000097343	9030407P20Rik	RIKEN cDNA 9030407P20 gene [Source:MGI Symbo	0.66071
ENSMUSG00000071176	Arhgef10	Rho guanine nucleotide exchange factor (GEF) 10 [S	0.66072
ENSMUSG00000096232	0610012G03Rik	RIKEN cDNA 0610012G03 gene [Source:MGI Symbo	0.66081
ENSMUSG00000032593	Amigo3	adhesion molecule with Ig like domain 3 [Source:M	0.66084
ENSMUSG00000054808	Actn4	actinin alpha 4 [Source:MGI Symbol;Acc:MGI:18907	0.66086
ENSMUSG00000038732	Mboat1	membrane bound O-acyltransferase domain contai	0.66088
ENSMUSG00000053916	Nanp	N-acetylneuraminic acid phosphatase [Source:MGI	0.66093
ENSMUSG00000022889	Mrpl39	mitochondrial ribosomal protein L39 [Source:MGI S	0.661
ENSMUSG00000001334	Fndc5	fibronectin type III domain containing 5 [Source:MG	0.66103
ENSMUSG00000030126	Tmcc1	transmembrane and coiled coil domains 1 [Source:	0.66104
ENSMUSG00000026480	Ncf2	neutrophil cytosolic factor 2 [Source:MGI Symbol;A	0.66109
ENSMUSG00000082321	Gm14253	predicted gene 14253 [Source:MGI Symbol;Acc:MG	0.66118
ENSMUSG00000078144	Capns2	calpain, small subunit 2 [Source:MGI Symbol;Acc:M	0.66164
ENSMUSG00000081800	Gm14200	predicted gene 14200 [Source:MGI Symbol;Acc:MG	0.66168
ENSMUSG00000044792	Isca1	iron-sulfur cluster assembly 1 homolog (S. cerevisia	0.66175
ENSMUSG00000038160	Atg5	autophagy related 5 [Source:MGI Symbol;Acc:MGI:	0.66181
ENSMUSG00000004891	Nes	nestin [Source:MGI Symbol;Acc:MGI:101784]	0.66184
ENSMUSG00000021754	Map3k1	mitogen-activated protein kinase kinase kinase 1 [S	0.66186
ENSMUSG00000022210	Dhrs4	dehydrogenase/reductase (SDR family) member 4 [	0.66189
ENSMUSG00000024892	Pcx	pyruvate carboxylase [Source:MGI Symbol;Acc:MGI	0.66193
ENSMUSG00000086707	Gm12088	predicted gene 12088 [Source:MGI Symbol;Acc:MG	0.66204
ENSMUSG00000048442	Smim5	small integral membrane protein 5 [Source:MGI Sy	0.66213
ENSMUSG00000066392	Nrxn3	neurexin III [Source:MGI Symbol;Acc:MGI:1096389]	0.6622
ENSMUSG00000050107	Gsg2	germ cell-specific gene 2 [Source:MGI Symbol;Acc:M	0.66224
ENSMUSG00000093416	Gm18294	predicted gene, 18294 [Source:MGI Symbol;Acc:MG	0.66239
ENSMUSG00000026058	Khdrbs2	KH domain containing, RNA binding, signal transdu	0.66256
ENSMUSG00000083853	Gm15696	predicted gene 15696 [Source:MGI Symbol;Acc:MG	0.66257
ENSMUSG00000020534	Shmt1	serine hydroxymethyltransferase 1 (soluble) [Sourc	0.66268
ENSMUSG00000086468	2900053O20Rik	RIKEN cDNA 2900053O20 gene [Source:MGI Symbo	0.6627
ENSMUSG00000082117	Gm4994	predicted gene 4994 [Source:MGI Symbol;Acc:MGI:	0.66275
ENSMUSG00000073686	Gm10564	predicted gene 10564 [Source:MGI Symbol;Acc:MG	0.66286
ENSMUSG00000039851	4932438H23Rik	RIKEN cDNA 4932438H23 gene [Source:MGI Symbo	0.66294
ENSMUSG00000027276	Jag1	jagged 1 [Source:MGI Symbol;Acc:MGI:1095416]	0.6631
ENSMUSG00000034203	Chchd4	coiled-coil-helix-coiled-coil-helix domain containing	0.66316
ENSMUSG00000026620	Mark1	MAP/microtubule affinity-regulating kinase 1 [Sour	0.66317
ENSMUSG00000055681	Cope	coatomer protein complex, subunit epsilon [Source	0.6632
ENSMUSG00000006930	Hap1	huntingtin-associated protein 1 [Source:MGI Symbo	0.66325
ENSMUSG00000033214	Slitrk5	SLIT and NTRK-like family, member 5 [Source:MGI S	0.66328
ENSMUSG00000049791	Fzd4	frizzled homolog 4 (Drosophila) [Source:MGI Symbo	0.66337

ENSMUSG00000042340	Ctf1	cardiotrophin 1 [Source:MGI Symbol;Acc:MGI:1051	0.66338
ENSMUSG00000078515	Ddi2	DNA-damage inducible protein 2 [Source:MGI Symb	0.66348
ENSMUSG00000005958	Ephb3	Eph receptor B3 [Source:MGI Symbol;Acc:MGI:1047	0.6635
ENSMUSG00000025145	Lrrc45	leucine rich repeat containing 45 [Source:MGI Symb	0.66353
ENSMUSG00000026755	Arpc5l	actin related protein 2/3 complex, subunit 5-like [S	0.66364
ENSMUSG00000085165	Gm12089	predicted gene 12089 [Source:MGI Symbol;Acc:MG	0.66373
ENSMUSG00000090124	Ugt1a7c	UDP glucuronosyltransferase 1 family, polypeptide	0.66375
ENSMUSG00000089734	Gm16108	predicted gene 16108 [Source:MGI Symbol;Acc:MG	0.66379
ENSMUSG00000062563	Cys1	cystin 1 [Source:MGI Symbol;Acc:MGI:2177632]	0.66388
ENSMUSG00000016481	Cr1l	complement component (3b/4b) receptor 1-like [S	0.66394
ENSMUSG00000027333	Smox	spermine oxidase [Source:MGI Symbol;Acc:MGI:24	0.66413
ENSMUSG00000079110	Capn3	calpain 3 [Source:MGI Symbol;Acc:MGI:107437]	0.66413
ENSMUSG00000025141	Myadml2	myeloid-associated differentiation marker-like 2 [S	0.66415
ENSMUSG00000086425	F730016J06Rik	RIKEN cDNA F730016J06 gene [Source:MGI Symbol	0.66419
ENSMUSG00000042557	Sin3a	transcriptional regulator, SIN3A (yeast) [Source:MG	0.6644
ENSMUSG00000083657	Gm12245	predicted gene 12245 [Source:MGI Symbol;Acc:MG	0.66454
ENSMUSG00000022682	Rrn3	RRN3 RNA polymerase I transcription factor homol	0.66461
ENSMUSG00000097912	6330403N20Rik	RIKEN cDNA 6330403N20 gene [Source:MGI Symbo	0.66468
ENSMUSG00000045912	C2cd4c	C2 calcium-dependent domain containing 4C [Sour	0.66484
ENSMUSG00000033847	Pla2g4c	phospholipase A2, group IVC (cytosolic, calcium-inc	0.66488
ENSMUSG00000040724	Kcna2	potassium voltage-gated channel, shaker-related su	0.66492
ENSMUSG00000031311	Nono	non-POU-domain-containing, octamer binding prot	0.66496
ENSMUSG00000058183	Mmel1	membrane metallo-endopeptidase-like 1 [Source:M	0.66496
ENSMUSG00000019773	Fbxo5	F-box protein 5 [Source:MGI Symbol;Acc:MGI:1914	0.66499
ENSMUSG00000032399	Rpl4	ribosomal protein L4 [Source:MGI Symbol;Acc:MGI	0.66499
ENSMUSG00000034334	Fam151b	family with sequence similarity 151, member B [Sou	0.66508
ENSMUSG00000069171	Nr2f1	nuclear receptor subfamily 2, group F, member 1 [S	0.66509
ENSMUSG00000027884	Clcc1	chloride channel CLIC-like 1 [Source:MGI Symbol;Ac	0.6652
ENSMUSG00000087640	Gm14798	predicted gene 14798 [Source:MGI Symbol;Acc:MG	0.66522
ENSMUSG00000087704	Gm14978	predicted gene 14978 [Source:MGI Symbol;Acc:MG	0.66533
ENSMUSG00000024132	Eci1	enoyl-Coenzyme A delta isomerase 1 [Source:MGI S	0.66544
ENSMUSG00000050017	Pitpnb	phosphatidylinositol transfer protein, beta [Source:	0.66548
ENSMUSG00000034460	Six4	sine oculis-related homeobox 4 [Source:MGI Symbo	0.66565
ENSMUSG00000032046	Abhd12	abhydrolase domain containing 12 [Source:MGI Syr	0.66584
ENSMUSG00000071662	Polr2g	polymerase (RNA) II (DNA directed) polypeptide G [	0.66584
ENSMUSG00000097160	A630072L19Rik	RIKEN cDNA A630072L19 gene [Source:MGI Symbo	0.66594
ENSMUSG00000042857	Gm9776	predicted gene 9776 [Source:MGI Symbol;Acc:MGI:	0.66606
ENSMUSG00000086644	Gm13470	predicted gene 13470 [Source:MGI Symbol;Acc:MG	0.66617
ENSMUSG00000030083	Abtb1	ankyrin repeat and BTB (POZ) domain containing 1	0.66626
ENSMUSG00000091735	Gpr62	G protein-coupled receptor 62 [Source:MGI Symbo	0.66627
ENSMUSG00000032596	Uba7	ubiquitin-like modifier activating enzyme 7 [Source	0.6663
ENSMUSG00000085175	Gm11423	predicted gene 11423 [Source:MGI Symbol;Acc:MG	0.66635
ENSMUSG00000022195	6030458C11Rik	RIKEN cDNA 6030458C11 gene [Source:MGI Symbo	0.66645
ENSMUSG00000030301	Ccdc91	coiled-coil domain containing 91 [Source:MGI Symb	0.66648
ENSMUSG00000092083	Kcnb2	potassium voltage gated channel, Shab-related sub	0.66662
ENSMUSG00000084803	5830444B04Rik	RIKEN cDNA 5830444B04 gene [Source:MGI Symbo	0.66668
ENSMUSG00000061802	Armc4	armadillo repeat containing 4 [Source:MGI Symbol;	0.66669

ENSMUSG00000024658	Gm9750	predicted gene 9750 [Source:MGI Symbol;Acc:MGI:	0.66671
ENSMUSG00000024177	Nme4	NME/NM23 nucleoside diphosphate kinase 4 [Sour	0.66674
ENSMUSG00000085923	Gm12781	predicted gene 12781 [Source:MGI Symbol;Acc:MG	0.66678
ENSMUSG00000002847	Pla1a	phospholipase A1 member A [Source:MGI Symbol;A	0.66681
ENSMUSG00000043110	Lrrn4	leucine rich repeat neuronal 4 [Source:MGI Symbol	0.66692
ENSMUSG00000085449	Gm15520	predicted gene 15520 [Source:MGI Symbol;Acc:MG	0.66692
ENSMUSG00000081922	Gm14646	predicted gene 14646 [Source:MGI Symbol;Acc:MG	0.66693
ENSMUSG00000087794	Gm25966	predicted gene, 25966 [Source:MGI Symbol;Acc:MG	0.66697
ENSMUSG00000006304	Arpc2	actin related protein 2/3 complex, subunit 2 [Sourc	0.66703
ENSMUSG00000084985	Gm16135	predicted gene 16135 [Source:MGI Symbol;Acc:MG	0.66714
ENSMUSG00000040759	Cmtm5	CKLF-like MARVEL transmembrane domain contain	0.66716
ENSMUSG00000044814	Olfr543	olfactory receptor 543 [Source:MGI Symbol;Acc:MG	0.66733
ENSMUSG00000057469	E2f6	E2F transcription factor 6 [Source:MGI Symbol;Acc:	0.66741
ENSMUSG00000031792	Usb1	U6 snRNA biogenesis 1 [Source:MGI Symbol;Acc:M	0.66764
ENSMUSG00000061762	Tac1	tachykinin 1 [Source:MGI Symbol;Acc:MGI:98474]	0.66777
ENSMUSG00000087108	Gm16093	predicted gene 16093 [Source:MGI Symbol;Acc:MG	0.66787
ENSMUSG00000075469	Gm17205	predicted gene 17205 [Source:MGI Symbol;Acc:MG	0.66788
ENSMUSG00000017146	Brca1	breast cancer 1 [Source:MGI Symbol;Acc:MGI:1045	0.66791
ENSMUSG00000035757	Selo	selenoprotein O [Source:MGI Symbol;Acc:MGI:1919	0.66791
ENSMUSG00000031323	Dmrtc1a	DMRT-like family C1a [Source:MGI Symbol;Acc:MG	0.66799
ENSMUSG00000055334	Snupn	snurportin 1 [Source:MGI Symbol;Acc:MGI:1913319	0.66802
ENSMUSG00000098183	Gm27010	predicted gene, 27010 [Source:MGI Symbol;Acc:MG	0.66807
ENSMUSG00000066867	Oas1e	2'-5' oligoadenylate synthetase 1E [Source:MGI Sym	0.66812
ENSMUSG00000084846	A730011C13Rik	RIKEN cDNA A730011C13 gene [Source:MGI Symbo	0.66823
ENSMUSG00000072494	Ppp1r3e	protein phosphatase 1, regulatory (inhibitor) subun	0.6683
ENSMUSG00000070780	Rbm47	RNA binding motif protein 47 [Source:MGI Symbol;	0.66833
ENSMUSG00000083890	Gm15703	predicted gene 15703 [Source:MGI Symbol;Acc:MG	0.66842
ENSMUSG00000092200	Tnxa	tenascin XA (pseudogene) [Source:MGI Symbol;Acc	0.66843
ENSMUSG00000026614	Slc30a10	solute carrier family 30, member 10 [Source:MGI Sy	0.66863
ENSMUSG00000044894	Uqcrq	ubiquinol-cytochrome c reductase, complex III subu	0.66869
ENSMUSG00000009108	Gnat2	guanine nucleotide binding protein, alpha transduc	0.66873
ENSMUSG00000048922	Cdca2	cell division cycle associated 2 [Source:MGI Symbol	0.66877
ENSMUSG00000058743	Kcnj14	potassium inwardly-rectifying channel, subfamily J,	0.66887
ENSMUSG00000087516	Gm2301	predicted gene 2301 [Source:MGI Symbol;Acc:MGI:	0.66891
ENSMUSG00000065226	Gm25791	predicted gene, 25791 [Source:MGI Symbol;Acc:MG	0.66894
ENSMUSG00000071847	Apcdd1	adenomatosis polyposis coli down-regulated 1 [Sou	0.669
ENSMUSG00000001988	Npas1	neuronal PAS domain protein 1 [Source:MGI Symbo	0.66908
ENSMUSG00000058934	Gm16176	predicted gene 16176 [Source:MGI Symbol;Acc:MG	0.66908
ENSMUSG00000057346	Apol9a	apolipoprotein L 9a [Source:MGI Symbol;Acc:MGI:3	0.6691
ENSMUSG00000022217	Emc9	ER membrane protein complex subunit 9 [Source:M	0.66914
ENSMUSG00000004626	Stxbp2	syntaxin binding protein 2 [Source:MGI Symbol;Acc	0.66949
ENSMUSG00000052188	Gm14964	predicted gene 14964 [Source:MGI Symbol;Acc:MG	0.66962
ENSMUSG00000056529	Ptafr	platelet-activating factor receptor [Source:MGI Sym	0.66962
ENSMUSG00000083087	Gm11249	predicted gene 11249 [Source:MGI Symbol;Acc:MG	0.66969
ENSMUSG00000059631	1500035N22Rik	RIKEN cDNA 1500035N22 gene [Source:MGI Symbo	0.6697
ENSMUSG00000087476	1810058N05Rik	RIKEN cDNA 1810058N05 gene [Source:MGI Symbo	0.6699
ENSMUSG00000031150	Ccdc120	coiled-coil domain containing 120 [Source:MGI Sym	0.66992

ENSMUSG00000063514	Gm6756	predicted gene 6756 [Source:MGI Symbol;Acc:MGI:	0.66992
ENSMUSG00000029331	4930522N08Rik	RIKEN cDNA 4930522N08 gene [Source:MGI Symbo	0.66994
ENSMUSG00000055994	Nod2	nucleotide-binding oligomerization domain contain	0.66997
ENSMUSG00000032322	Pstpip1	proline-serine-threonine phosphatase-interacting p	0.67002
ENSMUSG00000039748	Exo1	exonuclease 1 [Source:MGI Symbol;Acc:MGI:13494	0.67008
ENSMUSG00000023939	Mrpl14	mitochondrial ribosomal protein L14 [Source:MGI S	0.67021
ENSMUSG00000039230	Tbcd	tubulin-specific chaperone d [Source:MGI Symbol;A	0.67023
ENSMUSG00000085628	D630032N06Rik	RIKEN cDNA D630032N06 gene [Source:MGI Symbo	0.67042
ENSMUSG00000036550	Cnot1	CCR4-NOT transcription complex, subunit 1 [Source	0.67043
ENSMUSG00000051212	Gpr183	G protein-coupled receptor 183 [Source:MGI Symb	0.67048
ENSMUSG00000085767	Gm13563	predicted gene 13563 [Source:MGI Symbol;Acc:MG	0.67063
ENSMUSG00000065429	Mir345	microRNA 345 [Source:MGI Symbol;Acc:MGI:36193	0.67078
ENSMUSG00000086431	Gm14929	predicted gene 14929 [Source:MGI Symbol;Acc:MG	0.67079
ENSMUSG00000028680	Plk3	polo-like kinase 3 [Source:MGI Symbol;Acc:MGI:10	0.67097
ENSMUSG00000049620	Prss33	protease, serine, 33 [Source:MGI Symbol;Acc:MGI:2	0.671
ENSMUSG00000046434	Hnrnpa1	heterogeneous nuclear ribonucleoprotein A1 [Sour	0.67105
ENSMUSG00000020409	Slu7	SLU7 splicing factor homolog (S. cerevisiae) [Source	0.67111
ENSMUSG00000068105	Tnfrsf13c	tumor necrosis factor receptor superfamily, membe	0.67115
ENSMUSG00000014852	Adamts13	a disintegrin-like and metallopeptidase (reprolysin t	0.67123
ENSMUSG00000038521	C1s	complement component 1, s subcomponent [Sourc	0.67129
ENSMUSG00000041165	Spem1	sperm maturation 1 [Source:MGI Symbol;Acc:MGI:1	0.67129
ENSMUSG00000049241	Gpr81	G protein-coupled receptor 81 [Source:MGI Symbo	0.67142
ENSMUSG00000051041	Olfml1	olfactomedin-like 1 [Source:MGI Symbol;Acc:MGI:2	0.67142
ENSMUSG00000062382	Gm10116	predicted pseudogene 10116 [Source:MGI Symbol;	0.67142
ENSMUSG00000075590	Nrbp2	nuclear receptor binding protein 2 [Source:MGI Syr	0.67149
ENSMUSG00000032656	3-Mar	membrane-associated ring finger (C3HC4) 3 [Source	0.67151
ENSMUSG00000032042	Srpr	signal recognition particle receptor ('docking protei	0.67156
ENSMUSG00000031563	Wwc2	WW, C2 and coiled-coil domain containing 2 [Sourc	0.67165
ENSMUSG00000022639	5330426P16Rik	RIKEN cDNA 5330426P16 gene [Source:MGI Symbo	0.67178
ENSMUSG00000034997	Htr2a	5-hydroxytryptamine (serotonin) receptor 2A [Sour	0.67183
ENSMUSG00000020184	Mdm2	transformed mouse 3T3 cell double minute 2 [Sour	0.6719
ENSMUSG00000031448	Adprhl1	ADP-ribosylhydrolase like 1 [Source:MGI Symbol;Ac	0.67209
ENSMUSG00000047977	Synb	syncytin b [Source:MGI Symbol;Acc:MGI:3045308]	0.6721
ENSMUSG00000004610	Etfb	electron transferring flavoprotein, beta polypeptide	0.67214
ENSMUSG00000022144	Gdnf	glial cell line derived neurotrophic factor [Source:M	0.67221
ENSMUSG00000036594	H2-Aa	histocompatibility 2, class II antigen A, alpha [Sourc	0.67224
ENSMUSG00000025507	Lrdd	leucine-rich and death domain containing [Source:M	0.67234
ENSMUSG00000037885	Stk35	serine/threonine kinase 35 [Source:MGI Symbol;Ac	0.67235
ENSMUSG00000039108	Lsm14b	LSM14 homolog B (SCD6, S. cerevisiae) [Source:MG	0.67242
ENSMUSG00000031377	Bmx	BMX non-receptor tyrosine kinase [Source:MGI Syn	0.67249
ENSMUSG00000084166	Gm6451	predicted gene 6451 [Source:MGI Symbol;Acc:MGI:	0.67251
ENSMUSG00000024160	Spsb3	splA/ryanodine receptor domain and SOCS box con	0.67259
ENSMUSG00000021385	Ippk	inositol 1,3,4,5,6-pentakisphosphate 2-kinase [Sour	0.67263
ENSMUSG00000034101	Ctnnd1	catenin (cadherin associated protein), delta 1 [Sour	0.67266
ENSMUSG00000046714	Foxc2	forkhead box C2 [Source:MGI Symbol;Acc:MGI:134	0.67308
ENSMUSG00000031388	Naa10	N(alpha)-acetyltransferase 10, NatA catalytic subur	0.67324
ENSMUSG00000082985	Gm14042	predicted gene 14042 [Source:MGI Symbol;Acc:MG	0.67324

ENSMUSG00000023367	Tmem176a	transmembrane protein 176A [Source:MGI Symbol	0.67331
ENSMUSG00000026080	Chst10	carbohydrate sulfotransferase 10 [Source:MGI Sym	0.67347
ENSMUSG00000048000	Gigyf2	GRB10 interacting GYF protein 2 [Source:MGI Symb	0.67348
ENSMUSG00000030417	Pdcd5	programmed cell death 5 [Source:MGI Symbol;Acc:	0.67349
ENSMUSG00000085066	Gm4989	predicted pseudogene 4989 [Source:MGI Symbol;A	0.6735
ENSMUSG00000035297	Cops4	COP9 (constitutive photomorphogenic) homolog, s	0.67366
ENSMUSG00000064128	Cenpj	centromere protein J [Source:MGI Symbol;Acc:MGI	0.67368
ENSMUSG00000005267	Zfp287	zinc finger protein 287 [Source:MGI Symbol;Acc:M	0.67369
ENSMUSG00000089809	A930011G23Rik	RIKEN cDNA A930011G23 gene [Source:MGI Symbo	0.67369
ENSMUSG00000087470	A630031M04Rik	RIKEN cDNA A630031M04 gene [Source:MGI Symb	0.6738
ENSMUSG00000039126	Prune2	prune homolog 2 (Drosophila) [Source:MGI Symbol	0.67385
ENSMUSG00000088171	Gm25220	predicted gene, 25220 [Source:MGI Symbol;Acc:M	0.67386
ENSMUSG00000096958	Gm26573	predicted gene, 26573 [Source:MGI Symbol;Acc:M	0.67386
ENSMUSG00000063229	Ldha	lactate dehydrogenase A [Source:MGI Symbol;Acc:	0.67407
ENSMUSG00000030727	Rabep2	rabaptin, RAB GTPase binding effector protein 2 [S	0.67419
ENSMUSG00000084912	Gm16078	predicted gene 16078 [Source:MGI Symbol;Acc:MG	0.67429
ENSMUSG00000034493	4930556J24Rik	RIKEN cDNA 4930556J24 gene [Source:MGI Symbol	0.67434
ENSMUSG00000093693	Gm20719	predicted gene 20719 [Source:MGI Symbol;Acc:MG	0.67434
ENSMUSG00000044819	Oxgr1	oxoglutarate (alpha-ketoglutarate) receptor 1 [Sou	0.67437
ENSMUSG00000063506	Arhgap22	Rho GTPase activating protein 22 [Source:MGI Sym	0.67441
ENSMUSG00000032515	Csrnp1	cysteine-serine-rich nuclear protein 1 [Source:MGI	0.67443
ENSMUSG00000086213	A330040F15Rik	RIKEN cDNA A330040F15 gene [Source:MGI Symbo	0.67447
ENSMUSG00000035929	H2-Q4	histocompatibility 2, Q region locus 4 [Source:MGI	0.67451
ENSMUSG00000026489	Adck3	aarF domain containing kinase 3 [Source:MGI Symb	0.67501
ENSMUSG00000040554	Aipl1	aryl hydrocarbon receptor-interacting protein-like	0.67506
ENSMUSG00000096976	Gm26882	predicted gene, 26882 [Source:MGI Symbol;Acc:M	0.67516
ENSMUSG00000043301	Kcnj6	potassium inwardly-rectifying channel, subfamily J,	0.6752
ENSMUSG00000028708	Mknk1	MAP kinase-interacting serine/threonine kinase 1 [	0.67522
ENSMUSG00000061374	Fiz1	Flt3 interacting zinc finger protein 1 [Source:MGI S	0.67523
ENSMUSG00000087451	Gm12970	predicted gene 12970 [Source:MGI Symbol;Acc:MG	0.67531
ENSMUSG00000027349	Fam98b	family with sequence similarity 98, member B [Sou	0.67536
ENSMUSG00000068270	Shroom4	shroom family member 4 [Source:MGI Symbol;Acc:	0.67552
ENSMUSG00000022838	Eaf2	ELL associated factor 2 [Source:MGI Symbol;Acc:M	0.67553
ENSMUSG00000028621	Cyb5rl	cytochrome b5 reductase-like [Source:MGI Symbol	0.67553
ENSMUSG00000087059	Gm12339	predicted gene 12339 [Source:MGI Symbol;Acc:MG	0.67559
ENSMUSG00000018574	Acadvl	acyl-Coenzyme A dehydrogenase, very long chain [	0.67563
ENSMUSG00000051675	Trim32	tripartite motif-containing 32 [Source:MGI Symbol;	0.6757
ENSMUSG00000085745	Gm14372	predicted gene 14372 [Source:MGI Symbol;Acc:MG	0.6757
ENSMUSG00000095307	Gm12573	predicted gene 12573 [Source:MGI Symbol;Acc:MG	0.67571
ENSMUSG00000028532	Cachd1	cache domain containing 1 [Source:MGI Symbol;Ac	0.67574
ENSMUSG00000026478	Lamc1	laminin, gamma 1 [Source:MGI Symbol;Acc:MGI:99	0.67605
ENSMUSG00000093553	Gm20633	predicted gene 20633 [Source:MGI Symbol;Acc:MG	0.67606
ENSMUSG00000062646	Ganc	glucosidase, alpha; neutral C [Source:MGI Symbol;A	0.67608
ENSMUSG00000078713	Tomm5	translocase of outer mitochondrial membrane 5 ho	0.6761
ENSMUSG00000082064	Gm14217	predicted gene 14217 [Source:MGI Symbol;Acc:MG	0.67612
ENSMUSG00000054484	Tmem62	transmembrane protein 62 [Source:MGI Symbol;Ac	0.67613
ENSMUSG00000024942	Capn1	calpain 1 [Source:MGI Symbol;Acc:MGI:88263]	0.6762

ENSMUSG00000028441	1110017D15Rik	RIKEN cDNA 1110017D15 gene [Source:MGI Symbo	0.6762
ENSMUSG00000052337	Immt	inner membrane protein, mitochondrial [Source:M	0.67628
ENSMUSG00000056671	Prelid2	PRELI domain containing 2 [Source:MGI Symbol;Ac	0.67631
ENSMUSG00000097865	4932427H20Rik	RIKEN cDNA 4932427H20 gene [Source:MGI Symbo	0.67631
ENSMUSG00000043445	Pgp	phosphoglycolate phosphatase [Source:MGI Symbo	0.67637
ENSMUSG00000063019	Manbal	mannosidase, beta A, lysosomal-like [Source:MGI S	0.67645
ENSMUSG00000036275	9530068E07Rik	RIKEN cDNA 9530068E07 gene [Source:MGI Symbo	0.67647
ENSMUSG00000073854	AI427809	expressed sequence AI427809 [Source:MGI Symbol	0.67661
ENSMUSG00000045007	Tubg2	tubulin, gamma 2 [Source:MGI Symbol;Acc:MGI:21	0.67664
ENSMUSG00000069085	Dytn	dystrotelin [Source:MGI Symbol;Acc:MGI:2685061]	0.67665
ENSMUSG00000017830	Dhx58	DEXH (Asp-Glu-X-His) box polypeptide 58 [Source:M	0.67681
ENSMUSG00000033624	Pdpr	pyruvate dehydrogenase phosphatase regulatory su	0.67683
ENSMUSG00000035078	Mtmr9	myotubularin related protein 9 [Source:MGI Symbo	0.67693
ENSMUSG00000059150	Rpl26-ps4	ribosomal protein L26, pseudogene 4 [Source:MGI	0.67697
ENSMUSG00000097738	4930445N18Rik	RIKEN cDNA 4930445N18 gene [Source:MGI Symbo	0.67697
ENSMUSG00000016253	Nelfcd	negative elongation factor complex member C/D, T	0.67701
ENSMUSG00000024970	AI846148	expressed sequence AI846148 [Source:MGI Symbol	0.67717
ENSMUSG00000054934	Kcnmb4	potassium large conductance calcium-activated cha	0.67729
ENSMUSG00000097253	Gm26770	predicted gene, 26770 [Source:MGI Symbol;Acc:M	0.67736
ENSMUSG00000032398	Snapc5	small nuclear RNA activating complex, polypeptide	0.67741
ENSMUSG00000085360	Gm11647	predicted gene 11647 [Source:MGI Symbol;Acc:MG	0.67741
ENSMUSG00000049037	Clec4a1	C-type lectin domain family 4, member a1 [Source:	0.67747
ENSMUSG00000081187	3110067C02Rik	RIKEN cDNA 3110067C02 gene [Source:MGI Symbo	0.67754
ENSMUSG00000024525	Impa2	inositol (myo)-1(or 4)-monophosphatase 2 [Source:	0.67762
ENSMUSG00000042229	Rabif	RAB interacting factor [Source:MGI Symbol;Acc:MG	0.67765
ENSMUSG00000056018	1700008F21Rik	RIKEN cDNA 1700008F21 gene [Source:MGI Symbo	0.67771
ENSMUSG00000034825	Nrip3	nuclear receptor interacting protein 3 [Source:MGI	0.67772
ENSMUSG00000024073	Birc6	baculoviral IAP repeat-containing 6 [Source:MGI Sy	0.67793
ENSMUSG00000035032	Nek11	NIMA (never in mitosis gene a)-related expressed k	0.67797
ENSMUSG00000051671	Coa6	cytochrome c oxidase assembly factor 6 [Source:M	0.67817
ENSMUSG00000072487	Mroh5	maestro heat-like repeat family member 5 [Source:	0.67821
ENSMUSG00000050666	Vstm4	V-set and transmembrane domain containing 4 [So	0.67849
ENSMUSG00000019828	Grm1	glutamate receptor, metabotropic 1 [Source:MGI S	0.6785
ENSMUSG00000082927	Gm5863	predicted gene 5863 [Source:MGI Symbol;Acc:MGI	0.6786
ENSMUSG00000028907	Utp11l	UTP11-like, U3 small nucleolar ribonucleoprotein, (	0.67868
ENSMUSG00000059323	Tonsl	tonsoku-like, DNA repair protein [Source:MGI Symb	0.67872
ENSMUSG00000038502	Ptov1	prostate tumor over expressed gene 1 [Source:MGI	0.67875
ENSMUSG00000032883	Acsl3	acyl-CoA synthetase long-chain family member 3 [S	0.67878
ENSMUSG00000073610	Gm10549	predicted gene 10549 [Source:MGI Symbol;Acc:MG	0.67894
ENSMUSG00000051811	Cox6b2	cytochrome c oxidase subunit VIb polypeptide 2 [S	0.67895
ENSMUSG00000043192	Gm1840	predicted gene 1840 [Source:MGI Symbol;Acc:MGI	0.67899
ENSMUSG00000017009	Sdc4	syndecan 4 [Source:MGI Symbol;Acc:MGI:1349164]	0.67902
ENSMUSG00000085245	Gm11713	predicted gene 11713 [Source:MGI Symbol;Acc:MG	0.67908
ENSMUSG00000036892	Prodh2	proline dehydrogenase (oxidase) 2 [Source:MGI Syr	0.67917
ENSMUSG00000085945	2310014F06Rik	RIKEN cDNA 2310014F06 gene [Source:MGI Symbo	0.67927
ENSMUSG00000079467	Gm14966	predicted gene 14966 [Source:MGI Symbol;Acc:MG	0.67928
ENSMUSG00000087644	Gm14703	predicted gene 14703 [Source:MGI Symbol;Acc:MG	0.67928

ENSMUSG00000032497	Lrrfip2	leucine rich repeat (in FLII) interacting protein 2 [So	0.67937
ENSMUSG00000029148	Nrbp1	nuclear receptor binding protein 1 [Source:MGI Syr	0.67947
ENSMUSG00000024749	Tmc1	transmembrane channel-like gene family 1 [Source	0.67987
ENSMUSG00000044600	Smim7	small integral membrane protein 7 [Source:MGI Sy	0.67987
ENSMUSG00000021951	N6amt2	N-6 adenine-specific DNA methyltransferase 2 (put	0.68012
ENSMUSG00000035212	Leprot	leptin receptor overlapping transcript [Source:MGI	0.68012
ENSMUSG00000090322	Gm17090	predicted gene 17090 [Source:MGI Symbol;Acc:MG	0.68018
ENSMUSG00000055732	Gm9979	predicted gene 9979 [Source:MGI Symbol;Acc:MGI:	0.6802
ENSMUSG00000027709	Mccc1	methylcrotonoyl-Coenzyme A carboxylase 1 (alpha)	0.68022
ENSMUSG00000067058	Rps15a-ps5	ribosomal protein S15A, pseudogene 5 [Source:MG	0.68037
ENSMUSG00000090069	E430024P14Rik	RIKEN cDNA E430024P14 gene [Source:MGI Symbo	0.68042
ENSMUSG00000051515	Fam181b	family with sequence similarity 181, member B [Sou	0.68043
ENSMUSG00000032452	Clstn2	calsyntenin 2 [Source:MGI Symbol;Acc:MGI:192989	0.68051
ENSMUSG00000020610	Amz2	archaelysin family metallopeptidase 2 [Source:MGI	0.68053
ENSMUSG00000045751	Mms22l	MMS22-like, DNA repair protein [Source:MGI Symb	0.68075
ENSMUSG00000088088	Rmrp	RNA component of mitochondrial RNAase P [Source	0.68077
ENSMUSG00000059851	Suv420h2	suppressor of variegation 4-20 homolog 2 (Drosoph	0.68082
ENSMUSG00000093465	Gm20682	predicted gene 20682 [Source:MGI Symbol;Acc:MG	0.68112
ENSMUSG00000028337	Coro2a	coronin, actin binding protein 2A [Source:MGI Syml	0.68134
ENSMUSG00000091680	Klhdc7b	kelch domain containing 7B [Source:MGI Symbol;Ac	0.68139
ENSMUSG00000045179	Sox3	SRY-box containing gene 3 [Source:MGI Symbol;Acc	0.68142
ENSMUSG00000031480	Thsd1	thrombospondin, type I, domain 1 [Source:MGI Syn	0.68153
ENSMUSG00000047260	Emc6	ER membrane protein complex subunit 6 [Source:M	0.68169
ENSMUSG00000017692	Rhbdl3	rhomboid, veinlet-like 3 (Drosophila) [Source:MGI S	0.68179
ENSMUSG00000030553	Pgpep1l	pyroglutamyl-peptidase I-like [Source:MGI Symbol;	0.68183
ENSMUSG00000092459	Gm2302	predicted gene 2302 [Source:MGI Symbol;Acc:MGI:	0.6819
ENSMUSG00000049792	Bag5	BCL2-associated athanogene 5 [Source:MGI Symbo	0.68196
ENSMUSG00000021918	Nek4	NIMA (never in mitosis gene a)-related expressed k	0.68197
ENSMUSG00000027968	Larp7	La ribonucleoprotein domain family, member 7 [So	0.682
ENSMUSG00000038900	Rpl12	ribosomal protein L12 [Source:MGI Symbol;Acc:MG	0.68207
ENSMUSG00000040310	Alx4	aristaless-like homeobox 4 [Source:MGI Symbol;Acc	0.68209
ENSMUSG00000026698	Pigc	phosphatidylinositol glycan anchor biosynthesis, cla	0.68211
ENSMUSG00000027803	Wwtr1	WW domain containing transcription regulator 1 [S	0.68212
ENSMUSG00000021662	Arhgef28	Rho guanine nucleotide exchange factor (GEF) 28 [S	0.6822
ENSMUSG00000063623	C230062I16Rik	RIKEN cDNA C230062I16 gene [Source:MGI Symbol	0.68222
ENSMUSG00000093536	Smim17	small integral membrane protein 17 [Source:MGI S	0.68226
ENSMUSG00000087221	BC037032	cDNA Sequence BC037032 [Source:MGI Symbol;Acc	0.68261
ENSMUSG00000097934	6720483E21Rik	RIKEN cDNA 6720483E21 gene [Source:MGI Symbo	0.68265
ENSMUSG00000035860	Cdhr3	cadherin-related family member 3 [Source:MGI Syn	0.68272
ENSMUSG00000065406	Mirlet7i	microRNA let7i [Source:MGI Symbol;Acc:MGI:2676	0.68275
ENSMUSG00000097926	Gm26575	predicted gene, 26575 [Source:MGI Symbol;Acc:MG	0.6829
ENSMUSG00000038102	Trappc11	trafficking protein particle complex 11 [Source:MGI	0.68305
ENSMUSG00000096950	Gm9530	predicted gene 9530 [Source:MGI Symbol;Acc:MGI:	0.68305
ENSMUSG00000018449	Rpain	RPA interacting protein [Source:MGI Symbol;Acc:M	0.6831
ENSMUSG00000042572	Ube2q1	ubiquitin-conjugating enzyme E2Q (putative) 1 [Sou	0.68312
ENSMUSG00000001288	Rarg	retinoic acid receptor, gamma [Source:MGI Symbol	0.68315
ENSMUSG00000029134	Plb1	phospholipase B1 [Source:MGI Symbol;Acc:MGI:19	0.6832

ENSMUSG00000027963	Extl2	exostoses (multiple)-like 2 [Source:MGI Symbol;Acc	0.68331
ENSMUSG00000019235	Rps6kl1	ribosomal protein S6 kinase-like 1 [Source:MGI Sym	0.6835
ENSMUSG00000024896	Minpp1	multiple inositol polyphosphate histidine phosphata	0.68353
ENSMUSG00000084978	Gm11655	predicted gene 11655 [Source:MGI Symbol;Acc:MG	0.68365
ENSMUSG00000087259	2610035D17Rik	RIKEN cDNA 2610035D17 gene [Source:MGI Symbo	0.68365
ENSMUSG00000041736	Tspo	translocator protein [Source:MGI Symbol;Acc:MGI:	0.68377
ENSMUSG00000097567	Gm26637	predicted gene, 26637 [Source:MGI Symbol;Acc:M	0.68378
ENSMUSG00000086718	Gm16069	predicted gene 16069 [Source:MGI Symbol;Acc:MG	0.68381
ENSMUSG00000040560	Wdr7	WD repeat domain 7 [Source:MGI Symbol;Acc:MGI	0.68384
ENSMUSG00000097936	Gm4819	predicted gene 4819 [Source:MGI Symbol;Acc:MGI	0.68387
ENSMUSG00000042659	Arrdc4	arrestin domain containing 4 [Source:MGI Symbol;A	0.68394
ENSMUSG00000022956	Atp5o	ATP synthase, H+ transporting, mitochondrial F1 co	0.68397
ENSMUSG00000032340	Neo1	neogenin [Source:MGI Symbol;Acc:MGI:1097159]	0.68399
ENSMUSG00000064023	Klk8	kallikrein related-peptidase 8 [Source:MGI Symbol;	0.68404
ENSMUSG00000064307	Lrrc51	leucine rich repeat containing 51 [Source:MGI Sym	0.68431
ENSMUSG00000068606	Gm4841	predicted gene 4841 [Source:MGI Symbol;Acc:MGI	0.68432
ENSMUSG00000097471	5830432E09Rik	RIKEN cDNA 5830432E09 gene [Source:MGI Symbo	0.68435
ENSMUSG00000074918	5430417L22Rik	RIKEN cDNA 5430417L22 gene [Source:MGI Symbo	0.68439
ENSMUSG00000089743	Gm16226	predicted gene 16226 [Source:MGI Symbol;Acc:MG	0.68452
ENSMUSG00000069581	Tspear	thrombospondin type laminin G domain and EAR re	0.68455
ENSMUSG00000097620	4921514A10Rik	RIKEN cDNA 4921514A10 gene [Source:MGI Symbo	0.68456
ENSMUSG00000042724	Map3k9	mitogen-activated protein kinase kinase kinase 9 [S	0.68457
ENSMUSG00000030815	Phkg2	phosphorylase kinase, gamma 2 (testis) [Source:MG	0.68469
ENSMUSG00000092375	A730060N03Rik	RIKEN cDNA A730060N03 gene [Source:MGI Symbo	0.68486
ENSMUSG00000061740	Cyp2d22	cytochrome P450, family 2, subfamily d, polypeptid	0.68503
ENSMUSG00000032370	Lactb	lactamase, beta [Source:MGI Symbol;Acc:MGI:1933	0.68508
ENSMUSG00000024158	Hagh	hydroxyacyl glutathione hydrolase [Source:MGI Sym	0.68516
ENSMUSG00000034212	Ankmy1	ankyrin repeat and MYND domain containing 1 [Sou	0.68517
ENSMUSG00000047261	Gap43	growth associated protein 43 [Source:MGI Symbol;	0.68521
ENSMUSG00000041716	Gm20604	predicted gene 20604 [Source:MGI Symbol;Acc:MG	0.68522
ENSMUSG00000036450	Hif1an	hypoxia-inducible factor 1, alpha subunit inhibitor [	0.68527
ENSMUSG00000097993	Ptprv	protein tyrosine phosphatase, receptor type, V [Sou	0.68531
ENSMUSG00000034303	Ccdc15	coiled-coil domain containing 15 [Source:MGI Symb	0.68535
ENSMUSG00000031959	Wdr59	WD repeat domain 59 [Source:MGI Symbol;Acc:MG	0.68539
ENSMUSG00000085482	A230083N12Rik	RIKEN cDNA A230083N12 gene [Source:MGI Symbo	0.68563
ENSMUSG00000092569	Gm20544	predicted gene 20544 [Source:MGI Symbol;Acc:MG	0.68583
ENSMUSG00000026435	Slc45a3	solute carrier family 45, member 3 [Source:MGI Sym	0.68592
ENSMUSG00000055692	Tmem191c	transmembrane protein 191C [Source:MGI Symbol;	0.68593
ENSMUSG00000080971	Gm16128	predicted gene 16128 [Source:MGI Symbol;Acc:MG	0.68598
ENSMUSG00000092805	Gm26461	predicted gene, 26461 [Source:MGI Symbol;Acc:M	0.68598
ENSMUSG00000021259	Cyp46a1	cytochrome P450, family 46, subfamily a, polypepti	0.68603
ENSMUSG00000037358	4930578C19Rik	RIKEN cDNA 4930578C19 gene [Source:MGI Symbo	0.68606
ENSMUSG00000026305	Lrrfip1	leucine rich repeat (in FLII) interacting protein 1 [So	0.68615
ENSMUSG00000097976	Gm26918	predicted gene, 26918 [Source:MGI Symbol;Acc:M	0.68623
ENSMUSG00000094290	Gm23611	predicted gene, 23611 [Source:MGI Symbol;Acc:M	0.68626
ENSMUSG00000031731	Ap1g1	adaptor protein complex AP-1, gamma 1 subunit [S	0.68627
ENSMUSG00000052629	Gm9885	predicted gene 9885 [Source:MGI Symbol;Acc:MGI	0.68632

ENSMUSG00000026229	Psmd1	proteasome (prosome, macropain) 26S subunit, no	0.68633
ENSMUSG00000028517	Ppap2b	phosphatidic acid phosphatase type 2B [Source:MG	0.68636
ENSMUSG00000052005	Gm9864	predicted gene 9864 [Source:MGI Symbol;Acc:MGI:	0.68638
ENSMUSG00000055497	Gm9974	predicted gene 9974 [Source:MGI Symbol;Acc:MGI:	0.68639
ENSMUSG00000029029	Wrap73	WD repeat containing, antisense to Trp73 [Source:I	0.68641
ENSMUSG00000021411	Pxdc1	PX domain containing 1 [Source:MGI Symbol;Acc:M	0.68651
ENSMUSG00000026604	Ptpn14	protein tyrosine phosphatase, non-receptor type 14	0.68668
ENSMUSG00000031728	Zfp821	zinc finger protein 821 [Source:MGI Symbol;Acc:MG	0.68668
ENSMUSG00000041773	Enc1	ectodermal-neural cortex 1 [Source:MGI Symbol;Ac	0.6867
ENSMUSG00000032343	Impg1	interphotoreceptor matrix proteoglycan 1 [Source:I	0.68681
ENSMUSG00000023923	Tbc1d5	TBC1 domain family, member 5 [Source:MGI Symbo	0.6869
ENSMUSG00000097628	Gm2383	predicted gene 2383 [Source:MGI Symbol;Acc:MGI:	0.6869
ENSMUSG00000037015	Tmem185b	transmembrane protein 185B [Source:MGI Symbol;	0.68691
ENSMUSG00000039128	Cdc123	cell division cycle 123 [Source:MGI Symbol;Acc:MG	0.68692
ENSMUSG00000052143	Gm9869	predicted gene 9869 [Source:MGI Symbol;Acc:MGI:	0.68693
ENSMUSG00000028426	Rad23b	RAD23b homolog (S. cerevisiae) [Source:MGI Symb	0.68713
ENSMUSG00000059743	Fdps	farnesyl diphosphate synthetase [Source:MGI Symb	0.68717
ENSMUSG00000019854	Reps1	RalBP1 associated Eps domain containing protein [S	0.68721
ENSMUSG00000007950	Abhd8	abhydrolase domain containing 8 [Source:MGI Sym	0.68732
ENSMUSG00000070357	E030019B06Rik	RIKEN cDNA E030019B06 gene [Source:MGI Symbo	0.68733
ENSMUSG00000028519	Dab1	disabled 1 [Source:MGI Symbol;Acc:MGI:108554]	0.68736
ENSMUSG00000097203	4732419C18Rik	RIKEN cDNA 4732419C18 gene [Source:MGI Symbo	0.68745
ENSMUSG00000034829	Nxnl1	nucleoredoxin-like 1 [Source:MGI Symbol;Acc:MGI:	0.68754
ENSMUSG00000085723	Gm15915	predicted gene 15915 [Source:MGI Symbol;Acc:MG	0.68755
ENSMUSG00000026251	Chrnd	cholinergic receptor, nicotinic, delta polypeptide [S	0.68756
ENSMUSG00000019982	Myb	myeloblastosis oncogene [Source:MGI Symbol;Acc:	0.6876
ENSMUSG00000004929	Thop1	thimet oligopeptidase 1 [Source:MGI Symbol;Acc:M	0.68765
ENSMUSG00000078656	Vps25	vacuolar protein sorting 25 (yeast) [Source:MGI Syr	0.68767
ENSMUSG00000026490	Cdc42bpa	CDC42 binding protein kinase alpha [Source:MGI Sy	0.68778
ENSMUSG00000017300	Tnnc2	troponin C2, fast [Source:MGI Symbol;Acc:MGI:987	0.6878
ENSMUSG00000062691	1110001A16Rik	RIKEN cDNA 1110001A16 gene [Source:MGI Symbo	0.68783
ENSMUSG00000096780	Tmem181b-ps	transmembrane protein 181B, pseudogene [Source	0.68792
ENSMUSG00000034957	Cebpa	CCAAT/enhancer binding protein (C/EBP), alpha [So	0.68793
ENSMUSG00000024082	Ndufaf7	NADH dehydrogenase (ubiquinone) 1 alpha subcom	0.68794
ENSMUSG00000032556	Bfsp2	beaded filament structural protein 2, phakinin [Sou	0.68799
ENSMUSG00000082848	Gm16066	predicted gene 16066 [Source:MGI Symbol;Acc:MG	0.68802
ENSMUSG00000092392	Gm20546	predicted gene 20546 [Source:MGI Symbol;Acc:MG	0.68814
ENSMUSG00000086112	4930563I02Rik	RIKEN cDNA 4930563I02 gene [Source:MGI Symbol	0.68816
ENSMUSG00000018570	2810408A11Rik	RIKEN cDNA 2810408A11 gene [Source:MGI Symbo	0.68819
ENSMUSG00000086401	Gm15559	predicted gene 15559 [Source:MGI Symbol;Acc:MG	0.6883
ENSMUSG00000052139	Bre	brain and reproductive organ-expressed protein [So	0.68835
ENSMUSG00000029404	Arl6ip4	ADP-ribosylation factor-like 6 interacting protein 4	0.68855
ENSMUSG00000085220	Gm12521	predicted gene 12521 [Source:MGI Symbol;Acc:MG	0.68855
ENSMUSG00000047528	Als2cr12	amyotrophic lateral sclerosis 2 (juvenile) chromoso	0.6886
ENSMUSG00000034377	Tulp4	tubby like protein 4 [Source:MGI Symbol;Acc:MGI:1	0.68865
ENSMUSG00000001901	Kcnh6	potassium voltage-gated channel, subfamily H (eag	0.68874
ENSMUSG00000038215	Cep44	centrosomal protein 44 [Source:MGI Symbol;Acc:M	0.68882

ENSMUSG00000083844	2210015D19Rik	RIKEN cDNA 2210015D19 gene [Source:MGI Symbo	0.68882
ENSMUSG00000032215	Rsl24d1	ribosomal L24 domain containing 1 [Source:MGI Sy	0.68883
ENSMUSG00000001494	Sost	sclerostin [Source:MGI Symbol;Acc:MGI:1921749]	0.68894
ENSMUSG00000075410	Gm11744	predicted gene 11744 [Source:MGI Symbol;Acc:MG	0.68901
ENSMUSG00000035713	Usp35	ubiquitin specific peptidase 35 [Source:MGI Symbo	0.68908
ENSMUSG00000042759	Apobr	apolipoprotein B receptor [Source:MGI Symbol;Acc	0.68924
ENSMUSG00000047606	Ankrd34c	ankyrin repeat domain 34C [Source:MGI Symbol;Ac	0.68925
ENSMUSG00000097712	Gm10532	predicted gene 10532 [Source:MGI Symbol;Acc:MG	0.68925
ENSMUSG00000028116	Myoz2	myozenin 2 [Source:MGI Symbol;Acc:MGI:1913063	0.68949
ENSMUSG00000083796	Gm13369	predicted gene 13369 [Source:MGI Symbol;Acc:MG	0.68949
ENSMUSG00000021135	Slc10a1	solute carrier family 10 (sodium/bile acid cotransp	0.68968
ENSMUSG00000047592	Nxpe5	neurexophilin and PC-esterase domain family, mem	0.6897
ENSMUSG00000090327	Gm17111	predicted gene 17111 [Source:MGI Symbol;Acc:MG	0.68972
ENSMUSG00000018848	Rars	arginyl-tRNA synthetase [Source:MGI Symbol;Acc:M	0.68974
ENSMUSG00000063550	Nup98	nucleoporin 98 [Source:MGI Symbol;Acc:MGI:1094	0.68976
ENSMUSG00000015243	Abca1	ATP-binding cassette, sub-family A (ABC1), membe	0.68987
ENSMUSG00000045731	Pnoc	prepronociceptin [Source:MGI Symbol;Acc:MGI:10	0.6899
ENSMUSG00000028861	Mrps15	mitochondrial ribosomal protein S15 [Source:MGI S	0.68995
ENSMUSG00000024104	Fam21	family with sequence similarity 21 [Source:MGI Syn	0.69006
ENSMUSG00000050675	Gp1ba	glycoprotein 1b, alpha polypeptide [Source:MGI Sy	0.69013
ENSMUSG00000051435	Fhad1	forkhead-associated (FHA) phosphopeptide binding	0.69016
ENSMUSG00000036826	Igflr1	IGF-like family receptor 1 [Source:MGI Symbol;Acc:	0.69019
ENSMUSG00000093023	Gm25304	predicted gene, 25304 [Source:MGI Symbol;Acc:M	0.69019
ENSMUSG00000097775	4931440J10Rik	RIKEN cDNA 4931440J10 gene [Source:MGI Symbol	0.69019
ENSMUSG00000033016	Nfatc1	nuclear factor of activated T cells, cytoplasmic, calc	0.6902
ENSMUSG00000022445	Cyp2d26	cytochrome P450, family 2, subfamily d, polypeptid	0.69021
ENSMUSG00000092841	Gm24330	predicted gene, 24330 [Source:MGI Symbol;Acc:M	0.69021
ENSMUSG00000047676	Rpsa-ps10	ribosomal protein SA, pseudogene 10 [Source:MGI	0.69023
ENSMUSG00000085014	Gm13490	predicted gene 13490 [Source:MGI Symbol;Acc:MG	0.69025
ENSMUSG00000090374	Gm17232	predicted gene 17232 [Source:MGI Symbol;Acc:MG	0.69025
ENSMUSG00000036158	Prickle1	prickle homolog 1 (Drosophila) [Source:MGI Symbo	0.69027
ENSMUSG00000082398	Gm11927	predicted gene 11927 [Source:MGI Symbol;Acc:MG	0.69027
ENSMUSG00000085905	Gm14273	predicted gene 14273 [Source:MGI Symbol;Acc:MG	0.69027
ENSMUSG00000064495	Gm24313	predicted gene, 24313 [Source:MGI Symbol;Acc:M	0.69029
ENSMUSG00000081541	Gm13940	predicted gene 13940 [Source:MGI Symbol;Acc:MG	0.69029
ENSMUSG00000082248	Gm13161	predicted gene 13161 [Source:MGI Symbol;Acc:MG	0.69029
ENSMUSG00000049719	Prss46	protease, serine, 46 [Source:MGI Symbol;Acc:MGI:	0.69031
ENSMUSG00000023013	Aqp2	aquaporin 2 [Source:MGI Symbol;Acc:MGI:1096865	0.69032
ENSMUSG00000010057	Nprl2	nitrogen permease regulator-like 2 [Source:MGI Sy	0.69055
ENSMUSG00000034777	Vax2	ventral anterior homeobox containing gene 2 [Sour	0.69068
ENSMUSG00000074269	2410076I21Rik	RIKEN cDNA 2410076I21 gene [Source:MGI Symbol	0.69072
ENSMUSG00000031403	Dkc1	dyskeratosis congenita 1, dyskerin [Source:MGI Syn	0.69076
ENSMUSG00000002633	Shh	sonic hedgehog [Source:MGI Symbol;Acc:MGI:9829	0.69077
ENSMUSG00000078153	Psme2b	protease (prosome, macropain) activator subunit 2	0.69077
ENSMUSG00000081382	Rpl18-ps1	ribosomal protein L18, pseudogene 1 [Source:MGI	0.69081
ENSMUSG00000076271	Mir697	microRNA 697 [Source:MGI Symbol;Acc:MGI:36296	0.69083
ENSMUSG00000080370	Gm22952	predicted gene, 22952 [Source:MGI Symbol;Acc:M	0.69083

ENSMUSG00000092074	Dynlt1a	dynein light chain Tctex-type 1A [Source:MGI Symb	0.69083
ENSMUSG00000084817	Gm5526	predicted pseudogene 5526 [Source:MGI Symbol;A	0.69084
ENSMUSG00000070798	Psg25	pregnancy-specific glycoprotein 25 [Source:MGI Sy	0.69085
ENSMUSG00000090535	Gm8540	predicted gene 8540 [Source:MGI Symbol;Acc:MGI:	0.69085
ENSMUSG00000032816	Igdcc4	immunoglobulin superfamily, DCC subclass, membe	0.69086
ENSMUSG00000056824	Zfp663	zinc finger protein 663 [Source:MGI Symbol;Acc:M(	0.69086
ENSMUSG00000074919	Gm10787	predicted gene 10787 [Source:MGI Symbol;Acc:MG	0.69086
ENSMUSG00000084611	Gm24371	predicted gene, 24371 [Source:MGI Symbol;Acc:M(	0.69086
ENSMUSG00000086994	Gm13402	predicted gene 13402 [Source:MGI Symbol;Acc:MG	0.69086
ENSMUSG00000065499	Mir384	microRNA 384 [Source:MGI Symbol;Acc:MGI:36193	0.69087
ENSMUSG00000071818	4930417O22Rik	RIKEN cDNA 4930417O22 gene [Source:MGI Symbo	0.69087
ENSMUSG00000085074	Gm11494	predicted gene 11494 [Source:MGI Symbol;Acc:MG	0.69088
ENSMUSG00000023083	H2-M10.2	histocompatibility 2, M region locus 10.2 [Source:M	0.69089
ENSMUSG00000028684	Urod	uroporphyrinogen decarboxylase [Source:MGI Sym	0.69097
ENSMUSG00000024697	Gna14	guanine nucleotide binding protein, alpha 14 [Sourc	0.691
ENSMUSG00000032666	1700025G04Rik	RIKEN cDNA 1700025G04 gene [Source:MGI Symbo	0.69107
ENSMUSG00000085856	Gm12050	predicted gene 12050 [Source:MGI Symbol;Acc:MG	0.69118
ENSMUSG00000035561	Aldh1b1	aldehyde dehydrogenase 1 family, member B1 [Sou	0.69127
ENSMUSG00000062252	Lhfpl5	lipoma HMGIC fusion partner-like 5 [Source:MGI Sy	0.69133
ENSMUSG00000087357	Gm12498	predicted gene 12498 [Source:MGI Symbol;Acc:MG	0.69136
ENSMUSG00000039106	Htr5a	5-hydroxytryptamine (serotonin) receptor 5A [Sour	0.69142
ENSMUSG00000063439	B9d2	B9 protein domain 2 [Source:MGI Symbol;Acc:MGI:	0.69149
ENSMUSG00000090673	Gm340	predicted gene 340 [Source:MGI Symbol;Acc:MGI:2	0.69151
ENSMUSG00000043458	Pcdhb12	protocadherin beta 12 [Source:MGI Symbol;Acc:M(	0.6918
ENSMUSG00000041995	Zbed3	zinc finger, BED domain containing 3 [Source:MGI S	0.69183
ENSMUSG00000045094	Arhgef37	Rho guanine nucleotide exchange factor (GEF) 37 [S	0.69191
ENSMUSG00000036334	Igsf10	immunoglobulin superfamily, member 10 [Source:N	0.69207
ENSMUSG00000028514	Usp24	ubiquitin specific peptidase 24 [Source:MGI Symbo	0.69215
ENSMUSG00000051906	Cd209f	CD209f antigen [Source:MGI Symbol;Acc:MGI:1916	0.69221
ENSMUSG00000001918	Slc1a5	solute carrier family 1 (neutral amino acid transpor	0.69238
ENSMUSG00000076434	Wfdc3	WAP four-disulfide core domain 3 [Source:MGI Sym	0.69239
ENSMUSG00000086968	4933431E20Rik	RIKEN cDNA 4933431E20 gene [Source:MGI Symbo	0.69254
ENSMUSG00000007030	Vwa7	von Willebrand factor A domain containing 7 [Sourc	0.69275
ENSMUSG00000097085	Gm26634	predicted gene, 26634 [Source:MGI Symbol;Acc:M(	0.69277
ENSMUSG00000062515	Fabp4	fatty acid binding protein 4, adipocyte [Source:MGI	0.6928
ENSMUSG00000042807	Hecw2	HECT, C2 and WW domain containing E3 ubiquitin p	0.69284
ENSMUSG00000018209	Stk4	serine/threonine kinase 4 [Source:MGI Symbol;Acc	0.69289
ENSMUSG00000027386	Fbln7	fibulin 7 [Source:MGI Symbol;Acc:MGI:1917620]	0.69313
ENSMUSG00000040557	Wbscr27	Williams Beuren syndrome chromosome region 27	0.69322
ENSMUSG00000023027	Atf1	activating transcription factor 1 [Source:MGI Symbo	0.69336
ENSMUSG00000068417	Pnp2	purine-nucleoside phosphorylase 2 [Source:MGI Sy	0.69344
ENSMUSG00000093752	Gm20716	predicted gene 20716 [Source:MGI Symbol;Acc:MG	0.69351
ENSMUSG00000096966	Gm18336	predicted gene, 18336 [Source:MGI Symbol;Acc:M(	0.69351
ENSMUSG00000093134	Gm25996	predicted gene, 25996 [Source:MGI Symbol;Acc:M(	0.69353
ENSMUSG00000066060	Gm12866	predicted gene 12866 [Source:MGI Symbol;Acc:MG	0.69355
ENSMUSG00000077338	Gm23294	predicted gene, 23294 [Source:MGI Symbol;Acc:M(	0.69355
ENSMUSG00000065691	n-R5s77	nuclear encoded rRNA 5S 77 [Source:MGI Symbol;A	0.69357

ENSMUSG00000080967	Gm12706	predicted gene 12706 [Source:MGI Symbol;Acc:MG	0.69357
ENSMUSG00000097714	Gm20109	predicted gene, 20109 [Source:MGI Symbol;Acc:M(	0.69358
ENSMUSG00000029541	Cyp2w1	cytochrome P450, family 2, subfamily w, polypeptic	0.69359
ENSMUSG00000059706	A830035A12Rik	RIKEN cDNA A830035A12 gene [Source:MGI Symbo	0.69359
ENSMUSG00000078869	Gm14409	predicted gene 14409 [Source:MGI Symbol;Acc:MG	0.69359
ENSMUSG00000081239	Gm11836	predicted gene 11836 [Source:MGI Symbol;Acc:MG	0.6936
ENSMUSG00000085860	2410003L11Rik	RIKEN cDNA 2410003L11 gene [Source:MGI Symbo	0.6936
ENSMUSG00000085720	Gm7854	predicted gene 7854 [Source:MGI Symbol;Acc:MGI:	0.69361
ENSMUSG00000084202	Gm5398	predicted gene 5398 [Source:MGI Symbol;Acc:MGI:	0.69363
ENSMUSG00000020807	4933427D14Rik	RIKEN cDNA 4933427D14 gene [Source:MGI Symbo	0.69364
ENSMUSG00000083705	Gm8624	predicted gene 8624 [Source:MGI Symbol;Acc:MGI:	0.69365
ENSMUSG00000011427	Zfp790	zinc finger protein 790 [Source:MGI Symbol;Acc:M(	0.69367
ENSMUSG00000089907	Gm16169	predicted gene 16169 [Source:MGI Symbol;Acc:MG	0.69369
ENSMUSG00000095956	1700036A12Rik	RIKEN cDNA 1700036A12 gene [Source:MGI Symbo	0.69376
ENSMUSG00000097107	Gm6846	predicted gene 6846 [Source:MGI Symbol;Acc:MGI:	0.69377
ENSMUSG00000049565	Aknad1	AKNA domain containing 1 [Source:MGI Symbol;Ac	0.69378
ENSMUSG00000094988	Gm23728	predicted gene, 23728 [Source:MGI Symbol;Acc:M(	0.69378
ENSMUSG00000046372	Gm5590	predicted gene 5590 [Source:MGI Symbol;Acc:MGI:	0.69379
ENSMUSG00000088545	Gm25982	predicted gene, 25982 [Source:MGI Symbol;Acc:M(	0.6938
ENSMUSG00000029307	Dmp1	dentin matrix protein 1 [Source:MGI Symbol;Acc:M	0.69381
ENSMUSG00000055937	Krt28	keratin 28 [Source:MGI Symbol;Acc:MGI:1918093]	0.69381
ENSMUSG00000065873	n-R5s183	nuclear encoded rRNA 5S 183 [Source:MGI Symbol;	0.69381
ENSMUSG00000085707	Gm12212	predicted gene 12212 [Source:MGI Symbol;Acc:MG	0.69381
ENSMUSG00000087146	Gm15205	predicted gene 15205 [Source:MGI Symbol;Acc:MG	0.69381
ENSMUSG00000089924	Gm15689	predicted gene 15689 [Source:MGI Symbol;Acc:MG	0.69381
ENSMUSG00000093979	Gm2237	predicted gene 2237 [Source:MGI Symbol;Acc:MGI:	0.69381
ENSMUSG00000084878	Gm15817	predicted gene 15817 [Source:MGI Symbol;Acc:MG	0.69382
ENSMUSG00000091285	Gm17430	predicted gene, 17430 [Source:MGI Symbol;Acc:M(	0.69382
ENSMUSG00000082666	Gm13677	predicted gene 13677 [Source:MGI Symbol;Acc:MG	0.69383
ENSMUSG00000085563	2210411M09Rik	RIKEN cDNA 2210411M09 gene [Source:MGI Symb	0.69383
ENSMUSG00000081164	Gm8722	predicted gene 8722 [Source:MGI Symbol;Acc:MGI:	0.69385
ENSMUSG00000090665	Gad1-ps	glutamate decarboxylase 1, pseudogene [Source:M	0.69385
ENSMUSG00000067571	Ms4a15	membrane-spanning 4-domains, subfamily A, mem	0.69386
ENSMUSG00000081359	Cox20-ps	COX20 Cox2 chaperone, pseudogene [Source:MGI S	0.69387
ENSMUSG00000094443	Gm4975	predicted gene 4975 [Source:MGI Symbol;Acc:MGI:	0.69387
ENSMUSG00000047384	A730013G03Rik	RIKEN cDNA A730013G03 gene [Source:MGI Symbo	0.69388
ENSMUSG00000098033	Gm9381	predicted gene 9381 [Source:MGI Symbol;Acc:MGI:	0.69388
ENSMUSG00000077714	Snord17	small nucleolar RNA, C/D box 17 [Source:MGI Symb	0.69389
ENSMUSG00000022818	Cyp2ab1	cytochrome P450, family 2, subfamily ab, polypepti	0.6939
ENSMUSG00000070777	Ceacam20	carcinoembryonic antigen-related cell adhesion mo	0.6939
ENSMUSG00000074404	Gm1698	predicted gene 1698 [Source:MGI Symbol;Acc:MGI:	0.6939
ENSMUSG00000074417	Gm14548	predicted gene 14548 [Source:MGI Symbol;Acc:MG	0.6939
ENSMUSG00000031991	Spata19	spermatogenesis associated 19 [Source:MGI Symbo	0.69391
ENSMUSG00000083246	Gm11839	predicted gene 11839 [Source:MGI Symbol;Acc:MG	0.69394
ENSMUSG00000032594	Ip6k1	inositol hexaphosphate kinase 1 [Source:MGI Symb	0.69403
ENSMUSG00000035000	Dpp4	dipeptidylpeptidase 4 [Source:MGI Symbol;Acc:MG	0.69405
ENSMUSG00000098203	Gm9568	predicted gene 9568 [Source:MGI Symbol;Acc:MGI:	0.69412

ENSMUSG00000088442	Gm23553	predicted gene, 23553 [Source:MGI Symbol;Acc:MG	0.69415
ENSMUSG00000086277	4930558K02Rik	RIKEN cDNA 4930558K02 gene [Source:MGI Symbo	0.69416
ENSMUSG00000086889	Gm8619	predicted gene 8619 [Source:MGI Symbol;Acc:MGI:	0.69416
ENSMUSG00000087254	2310005A03Rik	RIKEN cDNA 2310005A03 gene [Source:MGI Symbo	0.69416
ENSMUSG00000086248	Gm16127	predicted gene 16127 [Source:MGI Symbol;Acc:MG	0.69417
ENSMUSG00000083184	Gm14969	predicted gene 14969 [Source:MGI Symbol;Acc:MG	0.69419
ENSMUSG00000044737	Klk14	kallikrein related-peptidase 14 [Source:MGI Symbo	0.69421
ENSMUSG00000086130	Gm16211	predicted gene 16211 [Source:MGI Symbol;Acc:MG	0.69423
ENSMUSG00000035522	Tsga8	testis specific gene A8 [Source:MGI Symbol;Acc:MG	0.69425
ENSMUSG00000095042	Gm12537	predicted gene 12537 [Source:MGI Symbol;Acc:MG	0.69429
ENSMUSG00000055177	Cstl1	cystatin-like 1 [Source:MGI Symbol;Acc:MGI:26528	0.6943
ENSMUSG00000064637	Snora20	small nucleolar RNA, H/ACA box 20 [Source:MGI Sy	0.69433
ENSMUSG00000006289	Osgep	O-sialoglycoprotein endopeptidase [Source:MGI Sy	0.69436
ENSMUSG00000074354	9230115E21Rik	RIKEN cDNA 9230115E21 gene [Source:MGI Symbo	0.69447
ENSMUSG00000039917	Rhbdd2	rhomboid domain containing 2 [Source:MGI Symbo	0.69451
ENSMUSG00000001156	Mxd1	MAX dimerization protein 1 [Source:MGI Symbol;A	0.69452
ENSMUSG00000071656	Lrrn4cl	LRRN4 C-terminal like [Source:MGI Symbol;Acc:MG	0.69452
ENSMUSG00000022680	Pdxdc1	pyridoxal-dependent decarboxylase domain contai	0.69454
ENSMUSG00000021745	Ptprg	protein tyrosine phosphatase, receptor type, G [So	0.69458
ENSMUSG00000025077	Dclre1a	DNA cross-link repair 1A, PSO2 homolog (S. cerevisi	0.69458
ENSMUSG00000039218	Srrm2	serine/arginine repetitive matrix 2 [Source:MGI Syr	0.6946
ENSMUSG00000086868	Gm15883	predicted gene 15883 [Source:MGI Symbol;Acc:MG	0.69498
ENSMUSG00000026675	Hsd17b7	hydroxysteroid (17-beta) dehydrogenase 7 [Source	0.69503
ENSMUSG00000086874	Gm13175	predicted gene 13175 [Source:MGI Symbol;Acc:MG	0.69506
ENSMUSG00000000817	Fasl	Fas ligand (TNF superfamily, member 6) [Source:MG	0.69507
ENSMUSG00000092182	Gm20407	predicted gene 20407 [Source:MGI Symbol;Acc:MG	0.69507
ENSMUSG00000028386	Slc46a2	solute carrier family 46, member 2 [Source:MGI Syr	0.69508
ENSMUSG00000087991	Gm25057	predicted gene, 25057 [Source:MGI Symbol;Acc:MG	0.69509
ENSMUSG00000086980	Gm13791	predicted gene 13791 [Source:MGI Symbol;Acc:MG	0.6951
ENSMUSG00000030365	Clec2i	C-type lectin domain family 2, member i [Source:M	0.69513
ENSMUSG00000040938	Slc16a11	solute carrier family 16 (monocarboxylic acid transp	0.69519
ENSMUSG00000085051	Gm11542	predicted gene 11542 [Source:MGI Symbol;Acc:MG	0.69521
ENSMUSG00000082049	Gm12105	predicted gene 12105 [Source:MGI Symbol;Acc:MG	0.69522
ENSMUSG00000080601	Gm23841	predicted gene, 23841 [Source:MGI Symbol;Acc:MG	0.69523
ENSMUSG00000055958	Gm9987	predicted gene 9987 [Source:MGI Symbol;Acc:MGI:	0.69524
ENSMUSG00000077358	Gm26293	predicted gene, 26293 [Source:MGI Symbol;Acc:MG	0.69524
ENSMUSG00000063821	Dupd1	dual specificity phosphatase and pro isomerase dor	0.69525
ENSMUSG00000065327	Gm22058	predicted gene, 22058 [Source:MGI Symbol;Acc:MG	0.69526
ENSMUSG00000094697	Gm10476	predicted gene 10476 [Source:MGI Symbol;Acc:MG	0.69526
ENSMUSG00000096597	Gm21886	predicted gene, 21886 [Source:MGI Symbol;Acc:MG	0.69529
ENSMUSG00000086841	2410006H16Rik	RIKEN cDNA 2410006H16 gene [Source:MGI Symbo	0.69536
ENSMUSG00000091020	Gm5828	predicted gene 5828 [Source:MGI Symbol;Acc:MGI:	0.69549
ENSMUSG00000089737	Gm15688	predicted gene 15688 [Source:MGI Symbol;Acc:MG	0.69555
ENSMUSG00000006638	Abhd1	abhydrolase domain containing 1 [Source:MGI Sym	0.69558
ENSMUSG00000064181	Rab3ip	RAB3A interacting protein [Source:MGI Symbol;Acc	0.69559
ENSMUSG00000020831	0610010K14Rik	RIKEN cDNA 0610010K14 gene [Source:MGI Symbo	0.69566
ENSMUSG00000036693	Nop14	NOP14 nucleolar protein [Source:MGI Symbol;Acc:I	0.69573

ENSMUSG00000027513	Pck1	phosphoenolpyruvate carboxykinase 1, cytosolic [S	0.6958
ENSMUSG00000029050	Ski	ski sarcoma viral oncogene homolog (avian) [Source	0.69585
ENSMUSG00000042549	Gm16516	predicted gene, Gm16516 [Source:MGI Symbol;Acc	0.6959
ENSMUSG00000072949	Acot1	acyl-CoA thioesterase 1 [Source:MGI Symbol;Acc:M	0.69596
ENSMUSG00000022546	Gpt	glutamic pyruvic transaminase, soluble [Source:MG	0.69598
ENSMUSG00000087443	5530401N12Rik	RIKEN cDNA 5530401N12 gene [Source:MGI Symbo	0.69611
ENSMUSG00000047844	Bex4	brain expressed gene 4 [Source:MGI Symbol;Acc:M	0.69613
ENSMUSG00000031897	Psmb10	proteasome (prosome, macropain) subunit, beta ty	0.69619
ENSMUSG00000005465	Il27ra	interleukin 27 receptor, alpha [Source:MGI Symbol;	0.69621
ENSMUSG00000090125	Pou3f1	POU domain, class 3, transcription factor 1 [Source	0.69621
ENSMUSG00000016255	Tubb1	tubulin, beta 1 class VI [Source:MGI Symbol;Acc:MG	0.69622
ENSMUSG00000097574	C920006O11Rik	RIKEN cDNA C920006O11 gene [Source:MGI Symbo	0.69626
ENSMUSG00000086247	Gm15787	predicted gene 15787 [Source:MGI Symbol;Acc:MG	0.69632
ENSMUSG00000072694	1500011B03Rik	RIKEN cDNA 1500011B03 gene [Source:MGI Symbo	0.69647
ENSMUSG00000057278	Snrpg	small nuclear ribonucleoprotein polypeptide G [Sou	0.69648
ENSMUSG00000035208	Slfn8	schlafen 8 [Source:MGI Symbol;Acc:MGI:2672859]	0.69655
ENSMUSG00000096957	E230013L22Rik	RIKEN cDNA E230013L22 gene [Source:MGI Symbo	0.69655
ENSMUSG00000079242	C730034F03Rik	RIKEN cDNA C730034F03 gene [Source:MGI Symbo	0.69658
ENSMUSG00000030094	Xpc	xeroderma pigmentosum, complementation group	0.69665
ENSMUSG00000038252	Ncapd2	non-SMC condensin I complex, subunit D2 [Source:	0.69667
ENSMUSG00000090038	Gm16573	predicted gene 16573 [Source:MGI Symbol;Acc:MG	0.69667
ENSMUSG00000092216	Gm19345	predicted gene, 19345 [Source:MGI Symbol;Acc:MG	0.69668
ENSMUSG00000032306	Mpi	mannose phosphate isomerase [Source:MGI Symbo	0.69686
ENSMUSG00000044339	Alkbh2	alkB, alkylation repair homolog 2 (E. coli) [Source:M	0.69698
ENSMUSG00000021902	Phf7	PHD finger protein 7 [Source:MGI Symbol;Acc:MGI:	0.69715
ENSMUSG00000068011	2510049J12Rik	RIKEN cDNA 2510049J12 gene [Source:MGI Symbol	0.69719
ENSMUSG00000068036	Mllt4	myeloid/lymphoid or mixed-lineage leukemia (trith	0.69745
ENSMUSG00000091996	BC049352	cDNA sequence BC049352 [Source:MGI Symbol;Acc	0.69748
ENSMUSG00000060727	Gm12571	predicted gene 12571 [Source:MGI Symbol;Acc:MG	0.6975
ENSMUSG00000000579	Dynlt1c	dynein light chain Tctex-type 1C [Source:MGI Symb	0.69753
ENSMUSG00000037349	Nudt22	nudix (nucleoside diphosphate linked moiety X)-typ	0.69754
ENSMUSG00000064272	Gpbar1	G protein-coupled bile acid receptor 1 [Source:MGI	0.69758
ENSMUSG00000065204	Gm24610	predicted gene, 24610 [Source:MGI Symbol;Acc:MG	0.69759
ENSMUSG00000037624	Kcnk2	potassium channel, subfamily K, member 2 [Source	0.6976
ENSMUSG00000044716	Dok7	docking protein 7 [Source:MGI Symbol;Acc:MGI:35	0.69763
ENSMUSG00000032121	Tmem218	transmembrane protein 218 [Source:MGI Symbol;A	0.69766
ENSMUSG00000028524	Sgip1	SH3-domain GRB2-like (endophilin) interacting prot	0.6977
ENSMUSG00000040356	Skiv2l	superkiller viralicidic activity 2-like (S. cerevisiae) [S	0.6977
ENSMUSG00000065623	Gm24762	predicted gene, 24762 [Source:MGI Symbol;Acc:MG	0.69774
ENSMUSG00000070834	Gm13015	predicted gene 13015 [Source:MGI Symbol;Acc:MG	0.69779
ENSMUSG00000048013	Krt35	keratin 35 [Source:MGI Symbol;Acc:MGI:1858899]	0.69782
ENSMUSG00000081740	Gm14279	predicted gene 14279 [Source:MGI Symbol;Acc:MG	0.69783
ENSMUSG00000083878	Gm12034	predicted gene 12034 [Source:MGI Symbol;Acc:MG	0.69783
ENSMUSG00000086147	Gm13150	predicted gene 13150 [Source:MGI Symbol;Acc:MG	0.69783
ENSMUSG00000081562	Gm11575	predicted gene 11575 [Source:MGI Symbol;Acc:MG	0.69785
ENSMUSG00000033854	Kcnk10	potassium channel, subfamily K, member 10 [Sourc	0.69786
ENSMUSG00000084772	Gm15473	predicted gene 15473 [Source:MGI Symbol;Acc:MG	0.69792

ENSMUSG00000094924	Cr2	complement receptor 2 [Source:MGI Symbol;Acc:M	0.69793
ENSMUSG00000043501	Lgals2	lectin, galactose-binding, soluble 2 [Source:MGI Syr	0.69794
ENSMUSG00000021890	Eaf1	ELL associated factor 1 [Source:MGI Symbol;Acc:M(	0.69801
ENSMUSG00000037815	Ctnna1	catenin (cadherin associated protein), alpha 1 [Sou	0.69811
ENSMUSG00000054021	Sirt5	sirtuin 5 [Source:MGI Symbol;Acc:MGI:1915596]	0.69823
ENSMUSG00000054191	Klf1	Kruppel-like factor 1 (erythroid) [Source:MGI Symb	0.69823
ENSMUSG00000022229	Atp12a	ATPase, H+/K+ transporting, nongastric, alpha poly	0.69836
ENSMUSG00000083507	Gm12842	predicted gene 12842 [Source:MGI Symbol;Acc:MG	0.69836
ENSMUSG00000060093	Hist1h4a	histone cluster 1, H4a [Source:MGI Symbol;Acc:MG	0.69837
ENSMUSG00000073652	Apol7d	apolipoprotein L 7d [Source:MGI Symbol;Acc:MGI:3	0.69837
ENSMUSG00000026405	C4bp	complement component 4 binding protein [Source:	0.69838
ENSMUSG00000053353	2310001K24Rik	RIKEN cDNA 2310001K24 gene [Source:MGI Symbo	0.6984
ENSMUSG00000038246	Fam50b	family with sequence similarity 50, member B [Sou	0.69841
ENSMUSG00000051879	Krt71	keratin 71 [Source:MGI Symbol;Acc:MGI:1861586]	0.69843
ENSMUSG00000050425	Mrgprb2	MAS-related GPR, member B2 [Source:MGI Symbol	0.69844
ENSMUSG00000041216	Clvs1	clavesin 1 [Source:MGI Symbol;Acc:MGI:1921688]	0.69845
ENSMUSG00000097429	Gm26520	predicted gene, 26520 [Source:MGI Symbol;Acc:M(	0.69845
ENSMUSG00000086786	Gm15908	predicted gene 15908 [Source:MGI Symbol;Acc:MG	0.69847
ENSMUSG00000038037	Socs1	suppressor of cytokine signaling 1 [Source:MGI Sym	0.69848
ENSMUSG00000030364	Clec2h	C-type lectin domain family 2, member h [Source:M	0.69861
ENSMUSG00000073433	Arhgdig	Rho GDP dissociation inhibitor (GDI) gamma [Sourc	0.69865
ENSMUSG00000089337	Gm24346	predicted gene, 24346 [Source:MGI Symbol;Acc:M(	0.69867
ENSMUSG00000078639	Gm12695	predicted gene 12695 [Source:MGI Symbol;Acc:MG	0.6987
ENSMUSG00000076338	Mir181d	microRNA 181d [Source:MGI Symbol;Acc:MGI:3709	0.69872
ENSMUSG00000075189	Olfr1055	olfactory receptor 1055 [Source:MGI Symbol;Acc:M	0.69873
ENSMUSG00000029650	Slc46a3	solute carrier family 46, member 3 [Source:MGI Syr	0.69876
ENSMUSG00000064158	Izumo1	izumo sperm-egg fusion 1 [Source:MGI Symbol;Acc	0.69876
ENSMUSG00000031295	Phka2	phosphorylase kinase alpha 2 [Source:MGI Symbol;	0.69888
ENSMUSG00000082849	Gm14053	predicted gene 14053 [Source:MGI Symbol;Acc:MG	0.69908
ENSMUSG00000097227	F630040L22Rik	RIKEN cDNA F630040L22 gene [Source:MGI Symbol	0.69924
ENSMUSG00000022043	Trim35	tripartite motif-containing 35 [Source:MGI Symbol;	0.69926
ENSMUSG00000000253	Gmpr	guanosine monophosphate reductase [Source:MGI	0.69933
ENSMUSG00000063171	Rps4y2	ribosomal protein S4, Y-linked 2 [Source:MGI Symb	0.69937
ENSMUSG00000048799	Cep120	centrosomal protein 120 [Source:MGI Symbol;Acc:I	0.69941
ENSMUSG00000027579	Srms	src-related kinase lacking C-terminal regulatory tyr	0.69945
ENSMUSG00000028240	Cyp7a1	cytochrome P450, family 7, subfamily a, polypeptid	0.69954
ENSMUSG00000024589	Nedd4l	neural precursor cell expressed, developmentally d	0.69955
ENSMUSG00000082387	Gm15724	predicted gene 15724 [Source:MGI Symbol;Acc:MG	0.69955
ENSMUSG00000085110	Gm13920	predicted gene 13920 [Source:MGI Symbol;Acc:MG	0.69955
ENSMUSG00000089699	Gm1992	predicted gene 1992 [Source:MGI Symbol;Acc:MGI:	0.69956
ENSMUSG00000084973	Gm13848	predicted gene 13848 [Source:MGI Symbol;Acc:MG	0.69957
ENSMUSG00000073102	Drc1	dynein regulatory complex subunit 1 [Source:MGI S	0.6996
ENSMUSG00000084400	Gm11576	predicted gene 11576 [Source:MGI Symbol;Acc:MG	0.6996
ENSMUSG00000085614	1700123M08Rik	RIKEN cDNA 1700123M08 gene [Source:MGI Symb	0.69961
ENSMUSG00000060038	Dhps	deoxyhypusine synthase [Source:MGI Symbol;Acc:M	0.69969
ENSMUSG00000061520	Olfr153	olfactory receptor 153 [Source:MGI Symbol;Acc:M(	0.69972
ENSMUSG00000067201	H2-M9	histocompatibility 2, M region locus 9 [Source:MGI	0.69973

ENSMUSG00000063586	Gm5513	predicted pseudogene 5513 [Source:MGI Symbol;A	0.69975
ENSMUSG00000089934	4930473D10Rik	RIKEN cDNA 4930473D10 gene [Source:MGI Symbo	0.69975
ENSMUSG00000092062	Gm7664	predicted gene 7664 [Source:MGI Symbol;Acc:MGI:	0.69975
ENSMUSG00000057037	Cfhr1	complement factor H-related 1 [Source:MGI Symbo	0.69976
ENSMUSG00000063021	Hist1h2ak	histone cluster 1, H2ak [Source:MGI Symbol;Acc:M	0.69976
ENSMUSG00000065100	Gm26132	predicted gene, 26132 [Source:MGI Symbol;Acc:M	0.69976
ENSMUSG00000077420	Gm24282	predicted gene, 24282 [Source:MGI Symbol;Acc:M	0.69976
ENSMUSG00000064525	Gm26440	predicted gene, 26440 [Source:MGI Symbol;Acc:M	0.69977
ENSMUSG00000087386	Gm15637	predicted gene 15637 [Source:MGI Symbol;Acc:MG	0.69977
ENSMUSG00000089493	Gm26361	predicted gene, 26361 [Source:MGI Symbol;Acc:M	0.69977
ENSMUSG00000073209	Klf14	Kruppel-like factor 14 [Source:MGI Symbol;Acc:MG	0.69978
ENSMUSG00000087364	Gm13398	predicted gene 13398 [Source:MGI Symbol;Acc:MG	0.69978
ENSMUSG00000089476	Gm25364	predicted gene, 25364 [Source:MGI Symbol;Acc:M	0.69978
ENSMUSG00000082876	Gm11889	predicted gene 11889 [Source:MGI Symbol;Acc:MG	0.69979
ENSMUSG00000082234	Gm16221	predicted gene 16221 [Source:MGI Symbol;Acc:MG	0.6998
ENSMUSG00000083818	Gm14908	predicted gene 14908 [Source:MGI Symbol;Acc:MG	0.69981
ENSMUSG00000082199	Gm15763	predicted gene 15763 [Source:MGI Symbol;Acc:MG	0.69982
ENSMUSG00000065423	Mir181a-2	microRNA 181a-2 [Source:MGI Symbol;Acc:MGI:26	0.69983
ENSMUSG00000068579	Rpl7a-ps3	ribosomal protein L7A, pseudogene 3 [Source:MGI	0.69983
ENSMUSG00000072753	9230020A06Rik	RIKEN cDNA 9230020A06 gene [Source:MGI Symbo	0.69983
ENSMUSG00000082925	Gm13135	predicted gene 13135 [Source:MGI Symbol;Acc:MG	0.69986
ENSMUSG00000091297	Gm8439	predicted gene 8439 [Source:MGI Symbol;Acc:MGI:	0.69987
ENSMUSG00000097874	C030010L15Rik	RIKEN cDNA C030010L15 gene [Source:MGI Symbo	0.69989
ENSMUSG00000078528	Gm17068	predicted gene 17068 [Source:MGI Symbol;Acc:MG	0.69993
ENSMUSG00000021115	Vrk1	vaccinia related kinase 1 [Source:MGI Symbol;Acc:M	0.69994
ENSMUSG00000085609	1700016P03Rik	RIKEN cDNA 1700016P03 gene [Source:MGI Symbo	0.69997
ENSMUSG00000037432	Fer1l5	fer-1-like 5 (C. elegans) [Source:MGI Symbol;Acc:M	0.70002
ENSMUSG00000087018	2900072N19Rik	RIKEN cDNA 2900072N19 gene [Source:MGI Symbo	0.70019
ENSMUSG00000018983	E2f2	E2F transcription factor 2 [Source:MGI Symbol;Acc:	0.70025
ENSMUSG00000033589	Reep4	receptor accessory protein 4 [Source:MGI Symbol;A	0.70031
ENSMUSG00000028950	Tas1r1	taste receptor, type 1, member 1 [Source:MGI Sym	0.7004
ENSMUSG00000025739	Gng13	guanine nucleotide binding protein (G protein), gar	0.70041
ENSMUSG00000033837	Foxh1	forkhead box H1 [Source:MGI Symbol;Acc:MGI:134	0.70045
ENSMUSG00000041308	Sntb2	syntrophin, basic 2 [Source:MGI Symbol;Acc:MGI:1	0.70052
ENSMUSG00000025200	Cwf19l1	CWF19-like 1, cell cycle control (S. pombe) [Source:	0.70056
ENSMUSG00000059395	Nkapl	NFKB activating protein-like [Source:MGI Symbol;A	0.70062
ENSMUSG00000059277	R74862	expressed sequence R74862 [Source:MGI Symbol;A	0.70063
ENSMUSG00000015536	Mocs2	molybdenum cofactor synthesis 2 [Source:MGI Sym	0.70067
ENSMUSG00000075040	Zfp408	zinc finger protein 408 [Source:MGI Symbol;Acc:M	0.7007
ENSMUSG00000036580	Spg20	spastic paraplegia 20, spartin (Troyer syndrome) ho	0.70097
ENSMUSG00000023452	Pisd	phosphatidylserine decarboxylase [Source:MGI Sym	0.701
ENSMUSG00000024981	Acsl5	acyl-CoA synthetase long-chain family member 5 [S	0.701
ENSMUSG00000048578	Mlec	malectin [Source:MGI Symbol;Acc:MGI:1924015]	0.70112
ENSMUSG00000031320	Rps4x	ribosomal protein S4, X-linked [Source:MGI Symbol	0.70113
ENSMUSG00000086820	Gm11465	predicted gene 11465 [Source:MGI Symbol;Acc:MG	0.70137
ENSMUSG00000069376	Gm5507	predicted gene 5507 [Source:MGI Symbol;Acc:MGI:	0.7014
ENSMUSG00000022061	Nkx3-1	NK-3 transcription factor, locus 1 (Drosophila) [Sou	0.70143

ENSMUSG00000063234	Gpr84	G protein-coupled receptor 84 [Source:MGI Symbo	0.70145
ENSMUSG00000097083	D930019O06Rik	RIKEN cDNA D930019O06 [Source:MGI Symbol;Acc	0.70148
ENSMUSG00000083219	Gm11410	predicted gene 11410 [Source:MGI Symbol;Acc:MG	0.70152
ENSMUSG00000024502	Jakmip2	janus kinase and microtubule interacting protein 2	0.70161
ENSMUSG00000072573	Gm10369	predicted gene 10369 [Source:MGI Symbol;Acc:MG	0.70162
ENSMUSG00000060621	Nkpd1	NTPase, KAP family P-loop domain containing 1 [So	0.70163
ENSMUSG00000032264	Zw10	zw10 kinetochore protein [Source:MGI Symbol;Acc	0.70176
ENSMUSG00000085227	6330418K02Rik	RIKEN cDNA 6330418K02 gene [Source:MGI Symbo	0.70177
ENSMUSG00000026036	Nif3l1	Ngg1 interacting factor 3-like 1 (S. pombe) [Source:	0.70192
ENSMUSG00000082163	Gm14276	predicted gene 14276 [Source:MGI Symbol;Acc:MG	0.70197
ENSMUSG00000031870	Pgr	progesterone receptor [Source:MGI Symbol;Acc:M	0.70204
ENSMUSG00000006642	Tcf23	transcription factor 23 [Source:MGI Symbol;Acc:M	0.70213
ENSMUSG00000002059	Rab34	RAB34, member of RAS oncogene family [Source:M	0.70215
ENSMUSG00000034648	Lrrn1	leucine rich repeat protein 1, neuronal [Source:MG	0.70232
ENSMUSG00000060591	Ifitm2	interferon induced transmembrane protein 2 [Sour	0.70233
ENSMUSG00000090628	Gm17083	predicted gene 17083 [Source:MGI Symbol;Acc:MG	0.70277
ENSMUSG00000026657	Frmd4a	FERM domain containing 4A [Source:MGI Symbol;A	0.70286
ENSMUSG00000051934	Spats2	spermatogenesis associated, serine-rich 2 [Source:I	0.70287
ENSMUSG00000048349	Pou4f1	POU domain, class 4, transcription factor 1 [Source	0.70294
ENSMUSG00000003559	As3mt	arsenic (+3 oxidation state) methyltransferase [Sou	0.70311
ENSMUSG00000022684	Bfar	bifunctional apoptosis regulator [Source:MGI Symb	0.70321
ENSMUSG00000023827	Agpat4	1-acylglycerol-3-phosphate O-acyltransferase 4 (lys	0.70329
ENSMUSG00000028744	Pqlc2	PQ loop repeat containing 2 [Source:MGI Symbol;A	0.70342
ENSMUSG00000016831	Tox4	TOX high mobility group box family member 4 [Sou	0.70343
ENSMUSG00000033083	Tbc1d4	TBC1 domain family, member 4 [Source:MGI Symb	0.70347
ENSMUSG00000085010	0610038P03Rik	RIKEN cDNA 0610038P03 gene [Source:MGI Symbo	0.70349
ENSMUSG00000090120	A730090N16Rik	RIKEN cDNA A730090N16 gene [Source:MGI Symb	0.70352
ENSMUSG00000097128	Gm26884	predicted gene, 26884 [Source:MGI Symbol;Acc:M	0.70357
ENSMUSG00000097176	Gm26830	predicted gene, 26830 [Source:MGI Symbol;Acc:M	0.70363
ENSMUSG00000024952	Rps6ka4	ribosomal protein S6 kinase, polypeptide 4 [Source	0.7037
ENSMUSG00000026313	Hdac4	histone deacetylase 4 [Source:MGI Symbol;Acc:MG	0.70377
ENSMUSG00000024957	Kcnk4	potassium channel, subfamily K, member 4 [Source	0.70393
ENSMUSG00000074280	Gm6166	predicted gene 6166 [Source:MGI Symbol;Acc:MGI:	0.70401
ENSMUSG00000025733	Rhot2	ras homolog gene family, member T2 [Source:MGI	0.70402
ENSMUSG00000016257	Slmo2	slowmo homolog 2 (Drosophila) [Source:MGI Symb	0.70405
ENSMUSG00000027752	Exosc8	exosome component 8 [Source:MGI Symbol;Acc:M	0.7042
ENSMUSG00000029186	Pi4k2b	phosphatidylinositol 4-kinase type 2 beta [Source:N	0.7044
ENSMUSG00000039716	Dock3	dedicator of cyto-kinesis 3 [Source:MGI Symbol;Acc	0.7044
ENSMUSG00000097650	4921507G05Rik	RIKEN cDNA 4921507G05 gene [Source:MGI Symbo	0.70446
ENSMUSG00000050240	Hic2	hypermethylated in cancer 2 [Source:MGI Symbol;A	0.70461
ENSMUSG00000022257	Laptm4b	lysosomal-associated protein transmembrane 4B [S	0.70462
ENSMUSG00000061787	Rps17	ribosomal protein S17 [Source:MGI Symbol;Acc:MG	0.70464
ENSMUSG00000024069	Slc30a6	solute carrier family 30 (zinc transporter), member	0.70473
ENSMUSG00000042684	Npl	N-acetylneuraminate pyruvate lyase [Source:MGI S	0.70473
ENSMUSG00000037005	Xpnpep2	X-prolyl aminopeptidase (aminopeptidase P) 2, me	0.70474
ENSMUSG00000024799	Tm7sf2	transmembrane 7 superfamily member 2 [Source:N	0.70475
ENSMUSG00000086453	Gm11457	predicted gene 11457 [Source:MGI Symbol;Acc:MG	0.7048

ENSMUSG00000072707	Olfr31	olfactory receptor 31 [Source:MGI Symbol;Acc:MGI	0.70497
ENSMUSG00000078441	Scamp4	secretory carrier membrane protein 4 [Source:MGI	0.70505
ENSMUSG00000030214	Plbd1	phospholipase B domain containing 1 [Source:MGI	0.70522
ENSMUSG00000090264	Eif4ebp3	eukaryotic translation initiation factor 4E binding p	0.7053
ENSMUSG00000051228	Nyx	nyctalopin [Source:MGI Symbol;Acc:MGI:2448607]	0.70537
ENSMUSG00000011958	Bnip2	BCL2/adenovirus E1B interacting protein 2 [Source:	0.70543
ENSMUSG00000041481	Serpina3g	serine (or cysteine) peptidase inhibitor, clade A, me	0.70543
ENSMUSG00000032318	Isl2	insulin related protein 2 (islet 2) [Source:MGI Symb	0.70549
ENSMUSG00000098199	Gm9358	predicted gene 9358 [Source:MGI Symbol;Acc:MGI:	0.7055
ENSMUSG00000031901	Dus2l	dihydrouridine synthase 2-like (SMM1, S. cerevisiae	0.70553
ENSMUSG00000084061	Gm4996	predicted gene 4996 [Source:MGI Symbol;Acc:MGI:	0.70555
ENSMUSG00000086446	4933426D04Rik	RIKEN cDNA 4933426D04 gene [Source:MGI Symbo	0.70566
ENSMUSG00000026894	Morn5	MORN repeat containing 5 [Source:MGI Symbol;Ac	0.70575
ENSMUSG00000086896	Gm12835	predicted gene 12835 [Source:MGI Symbol;Acc:MG	0.70589
ENSMUSG00000097444	Gm10489	predicted gene 10489 [Source:MGI Symbol;Acc:MG	0.70596
ENSMUSG00000055239	Kcmf1	potassium channel modulatory factor 1 [Source:MG	0.70597
ENSMUSG00000034796	Cpne7	copine VII [Source:MGI Symbol;Acc:MGI:2142747]	0.70602
ENSMUSG00000065968	Ifitm7	interferon induced transmembrane protein 7 [Sour	0.70602
ENSMUSG00000027643	Ghrh	growth hormone releasing hormone [Source:MGI S	0.70606
ENSMUSG00000040732	Erg	avian erythroblastosis virus E-26 (v-ets) oncogene r	0.70614
ENSMUSG00000043733	Ptpn11	protein tyrosine phosphatase, non-receptor type 1	0.7062
ENSMUSG00000072663	Spef2	sperm flagellar 2 [Source:MGI Symbol;Acc:MGI:244	0.70624
ENSMUSG00000040713	Creg1	cellular repressor of E1A-stimulated genes 1 [Sourc	0.70644
ENSMUSG00000018923	Med11	mediator of RNA polymerase II transcription, subur	0.70651
ENSMUSG00000003873	Bax	BCL2-associated X protein [Source:MGI Symbol;Acc	0.70656
ENSMUSG00000044066	Cep68	centrosomal protein 68 [Source:MGI Symbol;Acc:M	0.70671
ENSMUSG00000037216	Lipt1	lipoyltransferase 1 [Source:MGI Symbol;Acc:MGI:3	0.70673
ENSMUSG00000024982	Zdhhc6	zinc finger, DHHC domain containing 6 [Source:MG	0.70675
ENSMUSG00000087042	Gm11611	predicted gene 11611 [Source:MGI Symbol;Acc:MG	0.70681
ENSMUSG00000048905	4930539E08Rik	RIKEN cDNA 4930539E08 gene [Source:MGI Symbo	0.70685
ENSMUSG00000064140	Trim38	tripartite motif-containing 38 [Source:MGI Symbol;	0.70687
ENSMUSG00000091549	Gm6548	predicted gene 6548 [Source:MGI Symbol;Acc:MGI:	0.70689
ENSMUSG00000097374	Gm26874	predicted gene, 26874 [Source:MGI Symbol;Acc:M	0.70697
ENSMUSG00000042596	Tfap2d	transcription factor AP-2, delta [Source:MGI Symbo	0.70706
ENSMUSG00000042712	Wbp5	WW domain binding protein 5 [Source:MGI Symbol	0.70717
ENSMUSG00000024873	Cnih2	cornichon homolog 2 (Drosophila) [Source:MGI Syn	0.70721
ENSMUSG00000085508	Gm13965	predicted gene 13965 [Source:MGI Symbol;Acc:MG	0.70722
ENSMUSG00000006462	A530013C23Rik	RIKEN cDNA A530013C23 gene [Source:MGI Symbo	0.70729
ENSMUSG00000045838	A430105I19Rik	RIKEN cDNA A430105I19 gene [Source:MGI Symbol	0.70754
ENSMUSG00000047003	Zfp41	zinc finger protein 41 [Source:MGI Symbol;Acc:MG	0.7076
ENSMUSG00000090005	Gm16540	predicted gene 16540 [Source:MGI Symbol;Acc:MG	0.7076
ENSMUSG00000068264	Ap5s1	adaptor-related protein 5 complex, sigma 1 subunit	0.70763
ENSMUSG00000020434	4921536K21Rik	RIKEN cDNA 4921536K21 gene [Source:MGI Symbo	0.70772
ENSMUSG00000040694	Apobec2	apolipoprotein B mRNA editing enzyme, catalytic p	0.70778
ENSMUSG00000028633	Ctps	cytidine 5'-triphosphate synthase [Source:MGI Sym	0.70799
ENSMUSG00000038064	Cldn22	claudin 22 [Source:MGI Symbol;Acc:MGI:1922927]	0.70803
ENSMUSG00000053175	Bcl3	B cell leukemia/lymphoma 3 [Source:MGI Symbol;A	0.70805

ENSMUSG00000074219	Gm10644	predicted gene 10644 [Source:MGI Symbol;Acc:MG	0.70805
ENSMUSG00000040521	Tsfm	Ts translation elongation factor, mitochondrial [Sou	0.70809
ENSMUSG00000083838	Gm11957	predicted gene 11957 [Source:MGI Symbol;Acc:MG	0.7082
ENSMUSG00000078954	Arhgap8	Rho GTPase activating protein 8 [Source:MGI Symb	0.70846
ENSMUSG00000044595	Dnd1	dead end homolog 1 (zebrafish) [Source:MGI Symb	0.7087
ENSMUSG00000031972	Acta1	actin, alpha 1, skeletal muscle [Source:MGI Symbol	0.70887
ENSMUSG00000081343	Gm16071	predicted gene 16071 [Source:MGI Symbol;Acc:MG	0.70887
ENSMUSG00000071112	B230216G23Rik	RIKEN cDNA B230216G23 gene [Source:MGI Symbo	0.70889
ENSMUSG00000081211	Gm14886	predicted gene 14886 [Source:MGI Symbol;Acc:MG	0.70889
ENSMUSG00000021866	Anxa11	annexin A11 [Source:MGI Symbol;Acc:MGI:108481]	0.70901
ENSMUSG00000047293	Gpr15	G protein-coupled receptor 15 [Source:MGI Symbo	0.70912
ENSMUSG00000028617	Lrrc42	leucine rich repeat containing 42 [Source:MGI Sym	0.70917
ENSMUSG00000065954	Tacc1	transforming, acidic coiled-coil containing protein 1	0.70917
ENSMUSG00000061126	Cyp4f39	cytochrome P450, family 4, subfamily f, polypeptid	0.70918
ENSMUSG00000052852	Reep1	receptor accessory protein 1 [Source:MGI Symbol;A	0.7092
ENSMUSG00000085006	BC021767	cDNA sequence BC021767 [Source:MGI Symbol;Acc	0.7092
ENSMUSG00000035310	Lin54	lin-54 homolog (C. elegans) [Source:MGI Symbol;Ac	0.70931
ENSMUSG00000019867	Gje1	gap junction protein, epsilon 1 [Source:MGI Symbo	0.70936
ENSMUSG00000063275	Ptpla	protein tyrosine phosphatase-like (proline instead o	0.70954
ENSMUSG00000007207	Stx1a	syntaxin 1A (brain) [Source:MGI Symbol;Acc:MGI:1	0.70956
ENSMUSG00000018168	Ikzf3	IKAROS family zinc finger 3 [Source:MGI Symbol;Ac	0.70957
ENSMUSG00000014786	Slc9a5	solute carrier family 9 (sodium/hydrogen exchange	0.70958
ENSMUSG00000027748	Trpc4	transient receptor potential cation channel, subfam	0.70962
ENSMUSG00000037617	Spag1	sperm associated antigen 1 [Source:MGI Symbol;Ac	0.70964
ENSMUSG00000094772	Rpl29-ps2	ribosomal protein L29, pseudogene 2 [Source:MGI	0.70964
ENSMUSG00000049562	Ap5b1	adaptor-related protein complex 5, beta 1 subunit	0.70971
ENSMUSG00000074517	Gm10710	predicted gene 10710 [Source:MGI Symbol;Acc:MG	0.70973
ENSMUSG00000071866	Ppia	peptidylprolyl isomerase A [Source:MGI Symbol;Acc	0.70978
ENSMUSG00000044916	1700029I15Rik	RIKEN cDNA 1700029I15 gene [Source:MGI Symbol	0.70981
ENSMUSG00000028470	Hint2	histidine triad nucleotide binding protein 2 [Source	0.70985
ENSMUSG00000092203	1110038B12Rik	RIKEN cDNA 1110038B12 gene [Source:MGI Symbo	0.7099
ENSMUSG00000030342	Cd9	CD9 antigen [Source:MGI Symbol;Acc:MGI:88348]	0.71005
ENSMUSG00000043456	Zfp536	zinc finger protein 536 [Source:MGI Symbol;Acc:M(	0.71017
ENSMUSG00000091575	2010016I18Rik	RIKEN cDNA 2010016I18 gene [Source:MGI Symbol	0.71017
ENSMUSG00000049093	Il23r	interleukin 23 receptor [Source:MGI Symbol;Acc:M	0.71019
ENSMUSG00000051314	Ffar2	free fatty acid receptor 2 [Source:MGI Symbol;Acc:	0.71023
ENSMUSG00000001665	Gstt3	glutathione S-transferase, theta 3 [Source:MGI Sym	0.71036
ENSMUSG00000037824	Tspan14	tetraspanin 14 [Source:MGI Symbol;Acc:MGI:11963	0.71038
ENSMUSG00000078625	Gm12789	predicted gene 12789 [Source:MGI Symbol;Acc:MG	0.71039
ENSMUSG00000056508	1700001K19Rik	RIKEN cDNA 1700001K19 gene [Source:MGI Symbo	0.71042
ENSMUSG00000035459	Stab2	stabilin 2 [Source:MGI Symbol;Acc:MGI:2178743]	0.71057
ENSMUSG00000084882	Gm16047	predicted gene 16047 [Source:MGI Symbol;Acc:MG	0.7106
ENSMUSG00000028636	Ppcs	phosphopantothenoylcysteine synthetase [Source:	0.71072
ENSMUSG00000070529	Wfdc10	WAP four-disulfide core domain 10 [Source:MGI Sy	0.71076
ENSMUSG00000070167	Gm25822	predicted gene, 25822 [Source:MGI Symbol;Acc:M(	0.71078
ENSMUSG00000014633	Cmc2	COX assembly mitochondrial protein 2 [Source:MG	0.71086
ENSMUSG00000013158	4933405L10Rik	RIKEN cDNA 4933405L10 gene [Source:MGI Symbo	0.71087

ENSMUSG00000036435	Exoc1	exocyst complex component 1 [Source:MGI Symbo	0.71087
ENSMUSG00000002326	Gmpr2	guanosine monophosphate reductase 2 [Source:M(	0.71089
ENSMUSG00000063286	Gm8995	predicted gene 8995 [Source:MGI Symbol;Acc:MGI:	0.7109
ENSMUSG00000037014	Sstr4	somatostatin receptor 4 [Source:MGI Symbol;Acc:N	0.71105
ENSMUSG00000022911	Arl13b	ADP-ribosylation factor-like 13B [Source:MGI Symb	0.71106
ENSMUSG00000044787	Spata32	spermatogenesis associated 32 [Source:MGI Symbo	0.71111
ENSMUSG00000026281	Dtymk	deoxythymidylate kinase [Source:MGI Symbol;Acc:I	0.71124
ENSMUSG00000062683	Atp5g2	ATP synthase, H+ transporting, mitochondrial F0 co	0.71124
ENSMUSG00000042660	Wdr55	WD repeat domain 55 [Source:MGI Symbol;Acc:MG	0.7113
ENSMUSG00000071632	2510002D24Rik	RIKEN cDNA 2510002D24 gene [Source:MGI Symbo	0.71131
ENSMUSG00000089688	1600010F14Rik	RIKEN cDNA 1600010F14 gene [Source:MGI Symbo	0.71137
ENSMUSG00000004296	Il12b	interleukin 12b [Source:MGI Symbol;Acc:MGI:9654	0.71152
ENSMUSG00000051998	Lax1	lymphocyte transmembrane adaptor 1 [Source:MG	0.71152
ENSMUSG00000060671	Atp8b2	ATPase, class I, type 8B, member 2 [Source:MGI Syr	0.71152
ENSMUSG00000029757	Dync1i1	dynein cytoplasmic 1 intermediate chain 1 [Source:	0.71165
ENSMUSG00000030750	Nsmce1	non-SMC element 1 homolog (S. cerevisiae) [Source	0.71194
ENSMUSG00000026113	Inpp4a	inositol polyphosphate-4-phosphatase, type I [Sour	0.712
ENSMUSG00000029513	Prkab1	protein kinase, AMP-activated, beta 1 non-catalytic	0.71204
ENSMUSG00000064179	Tnnt1	troponin T1, skeletal, slow [Source:MGI Symbol;Acc	0.71204
ENSMUSG00000039156	Stim2	stromal interaction molecule 2 [Source:MGI Symbo	0.71206
ENSMUSG00000088326	Gm25667	predicted gene, 25667 [Source:MGI Symbol;Acc:M(	0.7121
ENSMUSG00000042895	Abra	actin-binding Rho activating protein [Source:MGI Sy	0.71221
ENSMUSG00000034485	Uaca	uveal autoantigen with coiled-coil domains and ank	0.71222
ENSMUSG00000081888	Spcs2-ps	signal peptidase complex subunit 2, pseudogene [S	0.71238
ENSMUSG00000061859	Inadl	InaD-like (Drosophila) [Source:MGI Symbol;Acc:MG	0.71239
ENSMUSG00000078592	Gm4609	predicted gene 4609 [Source:MGI Symbol;Acc:MGI:	0.71241
ENSMUSG00000042642	Flad1	RFad1, flavin adenine dinucleotide synthetase, hom	0.71242
ENSMUSG00000022241	Tars	threonyl-tRNA synthetase [Source:MGI Symbol;Acc	0.71256
ENSMUSG00000020099	Unc5b	unc-5 homolog B (C. elegans) [Source:MGI Symbol;/	0.71265
ENSMUSG00000093075	Gm25948	predicted gene, 25948 [Source:MGI Symbol;Acc:M(	0.71268
ENSMUSG00000078484	Klhl17	kelch-like 17 [Source:MGI Symbol;Acc:MGI:267894	0.7131
ENSMUSG00000029142	Mrpl33	mitochondrial ribosomal protein L33 [Source:MGI S	0.71314
ENSMUSG00000065676	Snord42b	small nucleolar RNA, C/D box 42B [Source:MGI Sym	0.71332
ENSMUSG00000026853	Crat	carnitine acetyltransferase [Source:MGI Symbol;Acc	0.71338
ENSMUSG00000030533	Unc45a	unc-45 homolog A (C. elegans) [Source:MGI Symbo	0.71351
ENSMUSG00000022449	Adamts20	a disintegrin-like and metallopeptidase (reprolysin	0.71353
ENSMUSG00000086127	Gm11934	predicted gene 11934 [Source:MGI Symbol;Acc:MG	0.71356
ENSMUSG00000030199	Etv6	ets variant gene 6 (TEL oncogene) [Source:MGI Sym	0.7136
ENSMUSG00000054641	Mmrn1	multimerin 1 [Source:MGI Symbol;Acc:MGI:191819	0.71369
ENSMUSG00000040054	Baz2a	bromodomain adjacent to zinc finger domain, 2A [S	0.71372
ENSMUSG00000085272	Gm1078	predicted gene 1078 [Source:MGI Symbol;Acc:MGI:	0.71376
ENSMUSG00000025340	Rabgef1	RAB guanine nucleotide exchange factor (GEF) 1 [So	0.71379
ENSMUSG00000027997	Casp6	caspase 6 [Source:MGI Symbol;Acc:MGI:1312921]	0.71384
ENSMUSG00000035775	Krt20	keratin 20 [Source:MGI Symbol;Acc:MGI:1914059]	0.71385
ENSMUSG00000057280	Musk	muscle, skeletal, receptor tyrosine kinase [Source:N	0.71385
ENSMUSG00000085704	4921531C22Rik	RIKEN cDNA 4921531C22 gene [Source:MGI Symbo	0.71387
ENSMUSG00000050846	Zfp623	zinc finger protein 623 [Source:MGI Symbol;Acc:M(	0.7139

ENSMUSG00000097271	Gm9903	predicted gene 9903 [Source:MGI Symbol;Acc:MGI:	0.71393
ENSMUSG00000078485	Plekhn1	pleckstrin homology domain containing, family N m	0.71397
ENSMUSG00000028836	Slc30a2	solute carrier family 30 (zinc transporter), member	0.71404
ENSMUSG00000047911	Npm2	nucleophosmin/nucleoplasmin 2 [Source:MGI Symb	0.71408
ENSMUSG00000058567	Gm2531	predicted gene 2531 [Source:MGI Symbol;Acc:MGI:	0.71413
ENSMUSG00000049907	Rasl11b	RAS-like, family 11, member B [Source:MGI Symbol	0.71445
ENSMUSG00000060176	Kif27	kinesin family member 27 [Source:MGI Symbol;Acc	0.71446
ENSMUSG00000030509	Asb7	ankyrin repeat and SOCS box-containing 7 [Source:	0.71472
ENSMUSG00000029410	Ppef2	protein phosphatase, EF hand calcium-binding dom	0.71478
ENSMUSG00000087610	Gm16253	predicted gene 16253 [Source:MGI Symbol;Acc:MG	0.71479
ENSMUSG00000091931	AK010878	cDNA sequence AK010878 [Source:MGI Symbol;Acc	0.71481
ENSMUSG00000026828	Galnt5	UDP-N-acetyl-alpha-D-galactosamine:polypeptide N	0.71482
ENSMUSG00000006782	Cnp	2',3'-cyclic nucleotide 3' phosphodiesterase [Source	0.71486
ENSMUSG00000042988	Notum	notum pectinacetylesterase homolog (Drosophila)	0.71495
ENSMUSG00000083935	Gm12294	predicted gene 12294 [Source:MGI Symbol;Acc:MG	0.71514
ENSMUSG00000097148	Gm3839	predicted pseudogene 3839 [Source:MGI Symbol;A	0.71518
ENSMUSG00000030225	Dera	2-deoxyribose-5-phosphate aldolase homolog (C. e	0.71524
ENSMUSG00000081594	Gm15467	predicted gene 15467 [Source:MGI Symbol;Acc:MG	0.71525
ENSMUSG00000087560	Gm15396	predicted gene 15396 [Source:MGI Symbol;Acc:MG	0.71527
ENSMUSG00000031970	Dbndd1	dysbindin (dystrobrevin binding protein 1) domain	0.7153
ENSMUSG00000033327	Tnxb	tenascin XB [Source:MGI Symbol;Acc:MGI:1932137	0.71533
ENSMUSG00000018322	Tomm34	translocase of outer mitochondrial membrane 34 [S	0.71549
ENSMUSG00000029851	Fam115c	family with sequence similarity 115, member C [Sou	0.71553
ENSMUSG00000029127	Zbtb49	zinc finger and BTB domain containing 49 [Source:M	0.71555
ENSMUSG00000054611	Kdm2a	lysine (K)-specific demethylase 2A [Source:MGI Sym	0.71566
ENSMUSG00000086801	Gm15943	predicted gene 15943 [Source:MGI Symbol;Acc:MG	0.71575
ENSMUSG00000045962	Wnk1	WNK lysine deficient protein kinase 1 [Source:MGI	0.71584
ENSMUSG00000074629	4930518I15Rik	RIKEN cDNA 4930518I15 gene [Source:MGI Symbol	0.71586
ENSMUSG00000021120	Pigh	phosphatidylinositol glycan anchor biosynthesis, cla	0.71593
ENSMUSG00000051391	Ywhag	tyrosine 3-monooxygenase/tryptophan 5-monooxy	0.71602
ENSMUSG00000064247	Plcxd1	phosphatidylinositol-specific phospholipase C, X do	0.71629
ENSMUSG00000027995	Tlr2	toll-like receptor 2 [Source:MGI Symbol;Acc:MGI:13	0.71634
ENSMUSG00000030834	Abcc6	ATP-binding cassette, sub-family C (CFTR/MRP), me	0.71642
ENSMUSG00000033885	Pxk	PX domain containing serine/threonine kinase [Sou	0.71647
ENSMUSG00000022383	Ppara	peroxisome proliferator activated receptor alpha [S	0.71669
ENSMUSG00000081915	Gm15174	predicted gene 15174 [Source:MGI Symbol;Acc:MG	0.71673
ENSMUSG00000097726	9530036O11Rik	RIKEN cDNA 9530036O11Rik [Source:MGI Symbol;A	0.71674
ENSMUSG00000018387	Shroom1	shroom family member 1 [Source:MGI Symbol;Acc:	0.71676
ENSMUSG00000031537	Ikbkb	inhibitor of kappaB kinase beta [Source:MGI Symbo	0.7168
ENSMUSG00000090761	Gm17201	predicted gene 17201 [Source:MGI Symbol;Acc:MG	0.71688
ENSMUSG00000073411	H2-D1	histocompatibility 2, D region locus 1 [Source:MGI S	0.71693
ENSMUSG00000045763	Basp1	brain abundant, membrane attached signal protein	0.71697
ENSMUSG00000063765	Chadl	chondroadherin-like [Source:MGI Symbol;Acc:MGI:	0.71698
ENSMUSG00000074183	Gsta1	glutathione S-transferase, alpha 1 (Ya) [Source:MGI	0.717
ENSMUSG00000021217	Tshz3	teashirt zinc finger family member 3 [Source:MGI S	0.71716
ENSMUSG00000020377	Ltc4s	leukotriene C4 synthase [Source:MGI Symbol;Acc:M	0.71718
ENSMUSG00000038403	Hfe2	hemochromatosis type 2 (juvenile) (human homolo	0.71722

ENSMUSG00000042210	Abhd14a	abhydrolase domain containing 14A [Source:MGI Sy	0.71727
ENSMUSG00000022439	Parvg	parvin, gamma [Source:MGI Symbol;Acc:MGI:2158	0.71729
ENSMUSG00000004897	Hdgf	hepatoma-derived growth factor [Source:MGI Syml	0.7173
ENSMUSG00000032026	Rexo2	REX2, RNA exonuclease 2 homolog (S. cerevisiae) [S	0.71746
ENSMUSG00000031839	Hsbp1	heat shock factor binding protein 1 [Source:MGI Sy	0.71747
ENSMUSG00000093594	Gm20707	predicted gene 20707 [Source:MGI Symbol;Acc:MG	0.71748
ENSMUSG00000097974	Gm10605	predicted gene 10605 [Source:MGI Symbol;Acc:MG	0.71751
ENSMUSG00000028409	Smu1	smu-1 suppressor of mec-8 and unc-52 homolog (C	0.71756
ENSMUSG00000064284	Cdpf1	cysteine rich, DPF motif domain containing 1 [Sourc	0.71758
ENSMUSG00000057751	Megf6	multiple EGF-like-domains 6 [Source:MGI Symbol;A	0.71763
ENSMUSG00000054967	Zfp647	zinc finger protein 647 [Source:MGI Symbol;Acc:MG	0.71764
ENSMUSG00000095651	Gm21817	predicted gene, 21817 [Source:MGI Symbol;Acc:MG	0.71768
ENSMUSG00000030590	Fam98c	family with sequence similarity 98, member C [Sour	0.71777
ENSMUSG00000020828	Pld2	phospholipase D2 [Source:MGI Symbol;Acc:MGI:89	0.71785
ENSMUSG00000026447	Pik3c2b	phosphoinositide-3-kinase, class 2, beta polypeptid	0.71798
ENSMUSG00000024712	Rfk	riboflavin kinase [Source:MGI Symbol;Acc:MGI:191	0.718
ENSMUSG00000021702	Thbs4	thrombospondin 4 [Source:MGI Symbol;Acc:MGI:1	0.71804
ENSMUSG00000050141	BC049635	cDNA sequence BC049635 [Source:MGI Symbol;Acc	0.71814
ENSMUSG00000026621	1-Mar	mitochondrial amidoxime reducing component 1 [S	0.71816
ENSMUSG00000020475	Pgam2	phosphoglycerate mutase 2 [Source:MGI Symbol;A	0.71821
ENSMUSG00000026516	Nvl	nuclear VCP-like [Source:MGI Symbol;Acc:MGI:191	0.71821
ENSMUSG00000097155	Gm26511	predicted gene, 26511 [Source:MGI Symbol;Acc:MG	0.71822
ENSMUSG00000018012	Rac3	RAS-related C3 botulinum substrate 3 [Source:MGI	0.71826
ENSMUSG00000097476	Gm26583	predicted gene, 26583 [Source:MGI Symbol;Acc:MG	0.71826
ENSMUSG00000027327	1700037H04Rik	RIKEN cDNA 1700037H04 gene [Source:MGI Symbo	0.71837
ENSMUSG00000025208	Mrpl43	mitochondrial ribosomal protein L43 [Source:MGI S	0.71846
ENSMUSG00000057880	Abat	4-aminobutyrate aminotransferase [Source:MGI Sy	0.7185
ENSMUSG00000021809	Nudt13	nudix (nucleoside diphosphate linked moiety X)-typ	0.7186
ENSMUSG00000031616	Ednra	endothelin receptor type A [Source:MGI Symbol;Ac	0.7186
ENSMUSG00000022498	Txndc11	thioredoxin domain containing 11 [Source:MGI Sym	0.71864
ENSMUSG00000037509	Arhgef4	Rho guanine nucleotide exchange factor (GEF) 4 [So	0.7187
ENSMUSG00000027833	Shox2	short stature homeobox 2 [Source:MGI Symbol;Acc	0.71878
ENSMUSG00000057899	Gpr111	G protein-coupled receptor 111 [Source:MGI Symb	0.71882
ENSMUSG00000022438	Parvb	parvin, beta [Source:MGI Symbol;Acc:MGI:2153063	0.71892
ENSMUSG00000044086	Lmod3	leiomodin 3 (fetal) [Source:MGI Symbol;Acc:MGI:24	0.71899
ENSMUSG00000028453	Fancg	Fanconi anemia, complementation group G [Source	0.719
ENSMUSG00000015647	Lama5	laminin, alpha 5 [Source:MGI Symbol;Acc:MGI:105	0.71923
ENSMUSG00000024309	H2-Ke2	H2-K region expressed gene 2 [Source:MGI Symbol;	0.71924
ENSMUSG00000078289	Gm10923	predicted gene 10923 [Source:MGI Symbol;Acc:MG	0.7193
ENSMUSG00000085184	4933439K11Rik	RIKEN cDNA 4933439K11 gene [Source:MGI Symbo	0.71932
ENSMUSG00000058174	Gm5148	predicted gene 5148 [Source:MGI Symbol;Acc:MGI	0.71933
ENSMUSG00000086869	Gm7809	predicted gene 7809 [Source:MGI Symbol;Acc:MGI	0.71933
ENSMUSG00000032105	Pdzd3	PDZ domain containing 3 [Source:MGI Symbol;Acc:	0.71935
ENSMUSG00000086844	B230206H07Rik	RIKEN cDNA B230206H07 gene [Source:MGI Symbo	0.71936
ENSMUSG00000064081	Gm8973	predicted gene 8973 [Source:MGI Symbol;Acc:MGI	0.71937
ENSMUSG00000037742	Eef1a1	eukaryotic translation elongation factor 1 alpha 1 [S	0.71961
ENSMUSG00000007122	Casq1	calsequestrin 1 [Source:MGI Symbol;Acc:MGI:1309	0.71965

ENSMUSG00000023084	Lrrc71	leucine rich repeat containing 71 [Source:MGI Syml	0.71965
ENSMUSG00000086039	Gm12227	predicted gene 12227 [Source:MGI Symbol;Acc:MG	0.71967
ENSMUSG00000054304	D130007C19Rik	RIKEN cDNA D130007C19 gene [Source:MGI Symbo	0.71969
ENSMUSG00000019880	Rspo3	R-spondin 3 homolog (Xenopus laevis) [Source:MGI	0.71972
ENSMUSG00000038482	Tfdp1	transcription factor Dp 1 [Source:MGI Symbol;Acc:M	0.71979
ENSMUSG00000047976	Kcna1	potassium voltage-gated channel, shaker-related su	0.71981
ENSMUSG00000039480	Nt5dc1	5'-nucleotidase domain containing 1 [Source:MGI S	0.71984
ENSMUSG00000061462	Obscn	obscurin, cytoskeletal calmodulin and titin-interact	0.71998
ENSMUSG00000056617	4931429L15Rik	RIKEN cDNA 4931429L15 gene [Source:MGI Symbo	0.72003
ENSMUSG00000034175	Rhbdd3	rhomboid domain containing 3 [Source:MGI Symbo	0.7201
ENSMUSG00000054013	Tmem179	transmembrane protein 179 [Source:MGI Symbol;A	0.7201
ENSMUSG00000048992	Prss32	protease, serine, 32 [Source:MGI Symbol;Acc:MGI:1	0.72016
ENSMUSG00000009210	2310007L24Rik	RIKEN cDNA 2310007L24 gene [Source:MGI Symbo	0.72022
ENSMUSG00000030268	Bcat1	branched chain aminotransferase 1, cytosolic [Sour	0.72035
ENSMUSG00000022101	Fgf17	fibroblast growth factor 17 [Source:MGI Symbol;Ac	0.72042
ENSMUSG00000044912	Syt16	synaptotagmin XVI [Source:MGI Symbol;Acc:MGI:2	0.72043
ENSMUSG00000053931	Cnn3	calponin 3, acidic [Source:MGI Symbol;Acc:MGI:191	0.72057
ENSMUSG00000085527	Gm15535	predicted gene 15535 [Source:MGI Symbol;Acc:MG	0.72058
ENSMUSG00000026409	Pfkfb2	6-phosphofructo-2-kinase/fructose-2,6-biphosphat	0.72062
ENSMUSG00000021066	Atl1	atlastin GTPase 1 [Source:MGI Symbol;Acc:MGI:192	0.72063
ENSMUSG00000061897	Gm14292	predicted gene 14292 [Source:MGI Symbol;Acc:MG	0.72063
ENSMUSG00000097977	Gm5652	predicted gene 5652 [Source:MGI Symbol;Acc:MGI:	0.72073
ENSMUSG00000049507	4930429B21Rik	RIKEN cDNA 4930429B21 gene [Source:MGI Symbo	0.72075
ENSMUSG00000083498	Gm14172	predicted gene 14172 [Source:MGI Symbol;Acc:MG	0.72082
ENSMUSG00000033350	Chst2	carbohydrate sulfotransferase 2 [Source:MGI Symb	0.72084
ENSMUSG00000025795	Rassf3	Ras association (RalGDS/AF-6) domain family meml	0.72087
ENSMUSG00000016386	Mpped2	metallophosphoesterase domain containing 2 [Sou	0.72089
ENSMUSG00000097415	AU020206	expressed sequence AU020206 [Source:MGI Symbo	0.72089
ENSMUSG00000031558	Slit2	slit homolog 2 (Drosophila) [Source:MGI Symbol;Ac	0.72091
ENSMUSG00000029790	Cep41	centrosomal protein 41 [Source:MGI Symbol;Acc:M	0.72096
ENSMUSG00000059830	Gm16380	predicted pseudogene 16380 [Source:MGI Symbol;	0.72099
ENSMUSG00000096215	Smim22	small integral membrane protein 22 [Source:MGI S	0.72099
ENSMUSG00000089694	Gm4477	predicted gene 4477 [Source:MGI Symbol;Acc:MGI:	0.72105
ENSMUSG00000097125	Gm26885	predicted gene, 26885 [Source:MGI Symbol;Acc:M(	0.72107
ENSMUSG00000021948	Prkcd	protein kinase C, delta [Source:MGI Symbol;Acc:M(	0.72119
ENSMUSG00000036934	4921524J17Rik	RIKEN cDNA 4921524J17 gene [Source:MGI Symbol	0.72119
ENSMUSG00000078349	AW011738	expressed sequence AW011738 [Source:MGI Symb	0.72125
ENSMUSG00000074811	Hps6	Hermansky-Pudlak syndrome 6 [Source:MGI Symbo	0.72126
ENSMUSG00000038543	BC028528	cDNA sequence BC028528 [Source:MGI Symbol;Acc	0.72129
ENSMUSG00000044770	Scml4	sex comb on midleg-like 4 (Drosophila) [Source:MG	0.7213
ENSMUSG00000022849	Hspbap1	Hspb associated protein 1 [Source:MGI Symbol;Acc	0.7214
ENSMUSG00000031668	Eif2ak3	eukaryotic translation initiation factor 2 alpha kinas	0.72145
ENSMUSG00000046440	Gm5564	predicted gene 5564 [Source:MGI Symbol;Acc:MGI:	0.72146
ENSMUSG00000049511	Htr1b	5-hydroxytryptamine (serotonin) receptor 1B [Sour	0.72148
ENSMUSG00000086448	9330162012Rik	cDNA RIKEN 9330162012 gene [Source:MGI Symbo	0.72158
ENSMUSG00000075534	Gm13262	predicted gene 13262 [Source:MGI Symbol;Acc:MG	0.72167
ENSMUSG00000020380	Rad50	RAD50 homolog (S. cerevisiae) [Source:MGI Symbo	0.72177

ENSMUSG00000004266	Ptpn6	protein tyrosine phosphatase, non-receptor type 6	0.72191
ENSMUSG00000089847	Timm10b	translocase of inner mitochondrial membrane 10B	0.72194
ENSMUSG00000037174	Elf2	E74-like factor 2 [Source:MGI Symbol;Acc:MGI:191	0.72197
ENSMUSG00000042569	Dhrs7b	dehydrogenase/reductase (SDR family) member 7B	0.72197
ENSMUSG00000097231	Gm26852	predicted gene, 26852 [Source:MGI Symbol;Acc:M	0.722
ENSMUSG00000073968	Trim68	tripartite motif-containing 68 [Source:MGI Symbol;	0.72208
ENSMUSG00000026627	Tmem206	transmembrane protein 206 [Source:MGI Symbol;A	0.7221
ENSMUSG00000041650	Pcca	propionyl-Coenzyme A carboxylase, alpha polypept	0.7222
ENSMUSG00000054412	Gm4793	predicted gene 4793 [Source:MGI Symbol;Acc:MGI	0.72221
ENSMUSG00000027742	Cog6	component of oligomeric golgi complex 6 [Source:M	0.72231
ENSMUSG00000030231	Plekha5	pleckstrin homology domain containing, family A m	0.72233
ENSMUSG00000096980	Gm26526	predicted gene, 26526 [Source:MGI Symbol;Acc:M	0.72238
ENSMUSG00000021752	Kctd6	potassium channel tetramerisation domain contain	0.72249
ENSMUSG00000028318	Polr1e	polymerase (RNA) I polypeptide E [Source:MGI Sym	0.72249
ENSMUSG00000081659	Gm14917	predicted gene 14917 [Source:MGI Symbol;Acc:MG	0.72257
ENSMUSG00000092994	Gm23918	predicted gene, 23918 [Source:MGI Symbol;Acc:M	0.72258
ENSMUSG00000027270	Lamp5	lysosomal-associated membrane protein family, me	0.7228
ENSMUSG00000082265	Gm15547	predicted gene 15547 [Source:MGI Symbol;Acc:MG	0.72302
ENSMUSG00000089663	Gm15651	predicted gene 15651 [Source:MGI Symbol;Acc:MG	0.72305
ENSMUSG00000020865	Abcc3	ATP-binding cassette, sub-family C (CFTR/MRP), me	0.72323
ENSMUSG00000056155	Nanos3	nanos homolog 3 (Drosophila) [Source:MGI Symbol	0.72334
ENSMUSG00000054237	Fra10ac1	FRA10AC1 homolog (human) [Source:MGI Symbol;	0.72348
ENSMUSG00000028139	Riiad1	regulatory subunit of type II PKA R-subunit (RIIa) do	0.72356
ENSMUSG00000094103	1700047I17Rik2	RIKEN cDNA 1700047I17 gene 2 [Source:MGI Symb	0.72365
ENSMUSG00000084776	Gm14951	predicted gene 14951 [Source:MGI Symbol;Acc:MG	0.72376
ENSMUSG00000051048	P4ha3	procollagen-proline, 2-oxoglutarate 4-dioxygenase	0.72384
ENSMUSG00000022897	Dyrk1a	dual-specificity tyrosine-(Y)-phosphorylation regula	0.72395
ENSMUSG00000037190	Cyb561d2	cytochrome b-561 domain containing 2 [Source:M	0.72412
ENSMUSG00000028963	Uts2	urotensin 2 [Source:MGI Symbol;Acc:MGI:1346329	0.72413
ENSMUSG00000022350	E430025E21Rik	RIKEN cDNA E430025E21 gene [Source:MGI Symbo	0.7243
ENSMUSG00000016252	Atp5e	ATP synthase, H+ transporting, mitochondrial F1 co	0.72431
ENSMUSG00000024524	Gnal	guanine nucleotide binding protein, alpha stimulati	0.72437
ENSMUSG00000086605	Gm14290	predicted gene 14290 [Source:MGI Symbol;Acc:MG	0.72438
ENSMUSG00000085279	Gm15965	predicted gene 15965 [Source:MGI Symbol;Acc:MG	0.72449
ENSMUSG00000033933	Vhl	von Hippel-Lindau tumor suppressor [Source:MGI S	0.72473
ENSMUSG00000000901	Mmp11	matrix metallopeptidase 11 [Source:MGI Symbol;Ac	0.72481
ENSMUSG00000022090	Pdlim2	PDZ and LIM domain 2 [Source:MGI Symbol;Acc:M	0.72487
ENSMUSG00000015451	C4a	complement component 4A (Rodgers blood group)	0.72503
ENSMUSG00000028370	Pappa	pregnancy-associated plasma protein A [Source:M	0.72504
ENSMUSG00000024454	Hdac3	histone deacetylase 3 [Source:MGI Symbol;Acc:MG	0.72505
ENSMUSG00000020526	Znhit3	zinc finger, HIT type 3 [Source:MGI Symbol;Acc:MG	0.7251
ENSMUSG00000097739	Gm2539	predicted gene, 2539 [Source:MGI Symbol;Acc:MGI	0.72511
ENSMUSG00000036103	Colec12	collectin sub-family member 12 [Source:MGI Symb	0.72513
ENSMUSG00000029191	Rfc1	replication factor C (activator 1) 1 [Source:MGI Sym	0.72518
ENSMUSG00000028971	Cort	cortistatin [Source:MGI Symbol;Acc:MGI:109538]	0.72519
ENSMUSG00000031577	Tti2	TELO2 interacting protein 2 [Source:MGI Symbol;Ac	0.72523
ENSMUSG00000005698	Ctcf	CCCTC-binding factor [Source:MGI Symbol;Acc:MGI	0.72524

ENSMUSG00000097518	Gm26694	predicted gene, 26694 [Source:MGI Symbol;Acc:MG	0.72538
ENSMUSG00000020941	Map3k14	mitogen-activated protein kinase kinase kinase 14 [	0.72539
ENSMUSG00000045679	Pqlc3	PQ loop repeat containing [Source:MGI Symbol;Acc	0.72539
ENSMUSG00000031665	Sall1	sal-like 1 (Drosophila) [Source:MGI Symbol;Acc:MG	0.72546
ENSMUSG00000035513	Ntng2	netrin G2 [Source:MGI Symbol;Acc:MGI:2159341]	0.72547
ENSMUSG00000079223	Gm8778	predicted gene 8778 [Source:MGI Symbol;Acc:MGI:	0.72553
ENSMUSG00000085890	2010012P19Rik	RIKEN cDNA 2010012P19 gene [Source:MGI Symbo	0.72554
ENSMUSG00000057666	Gapdh	glyceraldehyde-3-phosphate dehydrogenase [Sourc	0.72555
ENSMUSG00000041660	Bbox1	butyrobetaine (gamma), 2-oxoglutarate dioxygenas	0.72559
ENSMUSG00000035443	Thyn1	thymocyte nuclear protein 1 [Source:MGI Symbol;A	0.72572
ENSMUSG00000028348	Murc	muscle-related coiled-coil protein [Source:MGI Sym	0.72578
ENSMUSG00000000673	Haao	3-hydroxyanthranilate 3,4-dioxygenase [Source:MG	0.72584
ENSMUSG00000004270	Lpcat3	lysophosphatidylcholine acyltransferase 3 [Source:I	0.72593
ENSMUSG00000020282	Rhbdf1	rhomboid family 1 (Drosophila) [Source:MGI Symbo	0.72595
ENSMUSG00000023286	Ube2j2	ubiquitin-conjugating enzyme E2J 2 [Source:MGI Sy	0.72603
ENSMUSG00000006641	Slc5a6	solute carrier family 5 (sodium-dependent vitamin	0.72608
ENSMUSG00000044715	Gskip	GSK3B interacting protein [Source:MGI Symbol;Acc	0.72614
ENSMUSG00000026609	Ush2a	Usher syndrome 2A (autosomal recessive, mild) [So	0.72618
ENSMUSG00000019863	Qrsl1	glutaminyl-tRNA synthase (glutamine-hydrolyzing)-	0.72621
ENSMUSG00000097615	Gm2061	predicted gene 2061 [Source:MGI Symbol;Acc:MGI:	0.72621
ENSMUSG00000039671	Zmynd8	zinc finger, MYND-type containing 8 [Source:MGI Sy	0.72627
ENSMUSG00000026254	Eif4e2	eukaryotic translation initiation factor 4E member 2	0.72637
ENSMUSG00000085095	Gm15635	predicted gene 15635 [Source:MGI Symbol;Acc:MG	0.72637
ENSMUSG00000028405	Aco1	aconitase 1 [Source:MGI Symbol;Acc:MGI:87879]	0.72639
ENSMUSG00000085795	Zfp703	zinc finger protein 703 [Source:MGI Symbol;Acc:MG	0.72645
ENSMUSG00000068697	Myoz1	myozenin 1 [Source:MGI Symbol;Acc:MGI:1929471	0.72653
ENSMUSG00000003348	Mob3a	MOB kinase activator 3A [Source:MGI Symbol;Acc:M	0.72659
ENSMUSG00000028443	Nudt2	nudix (nucleoside diphosphate linked moiety X)-typ	0.72661
ENSMUSG00000011305	Plin5	perilipin 5 [Source:MGI Symbol;Acc:MGI:1914218]	0.72665
ENSMUSG00000086267	Gm12496	predicted gene 12496 [Source:MGI Symbol;Acc:MG	0.72674
ENSMUSG00000072571	Tmem253	transmembrane protein 253 [Source:MGI Symbol;A	0.72676
ENSMUSG00000044279	Crb3	crumbs homolog 3 (Drosophila) [Source:MGI Symbo	0.72684
ENSMUSG00000021356	Irf4	interferon regulatory factor 4 [Source:MGI Symbol;	0.72694
ENSMUSG00000044072	Eml6	echinoderm microtubule associated protein like 6 [	0.7272
ENSMUSG00000018501	Ncor1	nuclear receptor co-repressor 1 [Source:MGI Symbo	0.72727
ENSMUSG00000026452	Syt2	synaptotagmin II [Source:MGI Symbol;Acc:MGI:996	0.72756
ENSMUSG00000031497	Tnfsf13b	tumor necrosis factor (ligand) superfamily, membe	0.72765
ENSMUSG00000073623	5830472F04Rik	RIKEN cDNA 5830472F04 gene [Source:MGI Symbo	0.72769
ENSMUSG00000050304	Slc25a2	solute carrier family 25 (mitochondrial carrier, orni	0.72773
ENSMUSG00000028896	Rcc1	regulator of chromosome condensation 1 [Source:M	0.72776
ENSMUSG00000055072	Gm9964	predicted gene 9964 [Source:MGI Symbol;Acc:MGI:	0.72779
ENSMUSG00000083043	Gm13433	predicted gene 13433 [Source:MGI Symbol;Acc:MG	0.72779
ENSMUSG00000097209	AU022754	expressed sequence AU022754 [Source:MGI Symbo	0.72779
ENSMUSG00000000686	Abhd15	abhydrolase domain containing 15 [Source:MGI Syn	0.72782
ENSMUSG00000044285	Gm1821	predicted gene 1821 [Source:MGI Symbol;Acc:MGI:	0.72793
ENSMUSG00000030781	Slc5a2	solute carrier family 5 (sodium/glucose cotransport	0.72796
ENSMUSG00000026987	Baz2b	bromodomain adjacent to zinc finger domain, 2B [S	0.728

ENSMUSG00000030770	Parva	parvin, alpha [Source:MGI Symbol;Acc:MGI:193114	0.728
ENSMUSG00000065191	Gm23458	predicted gene, 23458 [Source:MGI Symbol;Acc:MG	0.72812
ENSMUSG00000030283	St8sia1	ST8 alpha-N-acetyl-neuraminide alpha-2,8-sialyltra	0.72814
ENSMUSG00000025069	Gsto2	glutathione S-transferase omega 2 [Source:MGI Syr	0.72845
ENSMUSG00000001323	Srr	serine racemase [Source:MGI Symbol;Acc:MGI:135	0.72856
ENSMUSG00000068874	Selenbp1	selenium binding protein 1 [Source:MGI Symbol;Ac	0.7286
ENSMUSG00000028005	Gucy1b3	guanylate cyclase 1, soluble, beta 3 [Source:MGI Sy	0.72861
ENSMUSG00000021798	Ldb3	LIM domain binding 3 [Source:MGI Symbol;Acc:MG	0.72865
ENSMUSG00000024420	Zfp521	zinc finger protein 521 [Source:MGI Symbol;Acc:MG	0.72866
ENSMUSG00000075027	4631405J19Rik	RIKEN cDNA 4631405J19 gene [Source:MGI Symbol	0.7287
ENSMUSG00000090006	Gm16227	predicted gene 16227 [Source:MGI Symbol;Acc:MG	0.7287
ENSMUSG00000052392	Acot4	acyl-CoA thioesterase 4 [Source:MGI Symbol;Acc:M	0.72873
ENSMUSG00000020849	Ywhae	tyrosine 3-monooxygenase/tryptophan 5-monooxy	0.72878
ENSMUSG00000050382	Kif7	kinesin family member 7 [Source:MGI Symbol;Acc:M	0.72881
ENSMUSG00000086915	Gm16365	predicted gene 16365 [Source:MGI Symbol;Acc:MG	0.72882
ENSMUSG00000010751	Tnfrsf22	tumor necrosis factor receptor superfamily, membe	0.72888
ENSMUSG00000019326	Aoc3	amine oxidase, copper containing 3 [Source:MGI Sy	0.72899
ENSMUSG00000030982	9030624J02Rik	RIKEN cDNA 9030624J02 gene [Source:MGI Symbol	0.72915
ENSMUSG00000097050	Gm9918	predicted gene 9918 [Source:MGI Symbol;Acc:MGI	0.7292
ENSMUSG00000049551	Fzd9	frizzled homolog 9 (Drosophila) [Source:MGI Symbo	0.72945
ENSMUSG00000049521	Cdc42ep1	CDC42 effector protein (Rho GTPase binding) 1 [Sou	0.72953
ENSMUSG00000055976	Cldn23	claudin 23 [Source:MGI Symbol;Acc:MGI:1919158]	0.72967
ENSMUSG00000037972	Snn	stannin [Source:MGI Symbol;Acc:MGI:1276549]	0.72968
ENSMUSG00000059406	Tmprss9	transmembrane protease, serine 9 [Source:MGI Syr	0.72971
ENSMUSG00000070705	Eid2b	EP300 interacting inhibitor of differentiation 2B [So	0.72972
ENSMUSG00000017288	Vps53	vacuolar protein sorting 53 (yeast) [Source:MGI Syr	0.72973
ENSMUSG00000037339	Fam53a	family with sequence similarity 53, member A [Sou	0.72983
ENSMUSG00000097248	Gm2694	predicted gene 2694 [Source:MGI Symbol;Acc:MGI	0.72996
ENSMUSG00000035545	Leng8	leukocyte receptor cluster (LRC) member 8 [Source	0.72997
ENSMUSG00000047669	Msl3l2	male-specific lethal 3-like 2 (Drosophila) [Source:M	0.73005
ENSMUSG00000066643	Wdr35	WD repeat domain 35 [Source:MGI Symbol;Acc:MG	0.73009
ENSMUSG00000018920	Cxcl16	chemokine (C-X-C motif) ligand 16 [Source:MGI Syn	0.73032
ENSMUSG00000025488	Cox8b	cytochrome c oxidase subunit VIIIb [Source:MGI Sy	0.73035
ENSMUSG00000046101	Mcmdc2	minichromosome maintenance domain containing	0.73036
ENSMUSG00000035237	Lcat	lecithin cholesterol acyltransferase [Source:MGI Sy	0.73041
ENSMUSG00000029469	Ift81	intraflagellar transport 81 [Source:MGI Symbol;Acc	0.73046
ENSMUSG00000081497	Gm15560	predicted gene 15560 [Source:MGI Symbol;Acc:MG	0.73047
ENSMUSG00000085437	1700047A11Rik	RIKEN cDNA 1700047A11 gene [Source:MGI Symbo	0.73051
ENSMUSG00000097533	Gm26590	predicted gene, 26590 [Source:MGI Symbol;Acc:MG	0.73051
ENSMUSG00000036825	Ssx2ip	synovial sarcoma, X breakpoint 2 interacting protei	0.73052
ENSMUSG00000084758	Gm12798	predicted gene 12798 [Source:MGI Symbol;Acc:MG	0.73056
ENSMUSG00000031861	Lpar2	lysophosphatidic acid receptor 2 [Source:MGI Symb	0.73072
ENSMUSG00000024256	Adcyap1	adenylate cyclase activating polypeptide 1 [Source:	0.73086
ENSMUSG00000048029	Eno4	enolase 4 [Source:MGI Symbol;Acc:MGI:2441717]	0.73093
ENSMUSG00000022704	Qtrtd1	queuine tRNA-ribosyltransferase domain containing	0.73097
ENSMUSG00000093720	Gm20635	predicted gene 20635 [Source:MGI Symbol;Acc:MG	0.73101
ENSMUSG00000067206	Lrrc66	leucine rich repeat containing 66 [Source:MGI Symb	0.73102

ENSMUSG00000095832	Supt20	suppressor of Ty 20 [Source:MGI Symbol;Acc:MGI:1	0.73104
ENSMUSG00000097479	Gm26582	predicted gene, 26582 [Source:MGI Symbol;Acc:MG	0.73122
ENSMUSG00000079478	Sssca1	Sjogren's syndrome/scleroderma autoantigen 1 ho	0.73129
ENSMUSG00000005262	Ufd1l	ubiquitin fusion degradation 1 like [Source:MGI Syr	0.7313
ENSMUSG00000038670	Mybpc2	myosin binding protein C, fast-type [Source:MGI Sy	0.73161
ENSMUSG00000095344	Gm6838	predicted gene 6838 [Source:MGI Symbol;Acc:MGI:	0.73168
ENSMUSG00000090222	Gm16340	predicted gene 16340 [Source:MGI Symbol;Acc:MG	0.73174
ENSMUSG00000025724	Sec11a	SEC11 homolog A (S. cerevisiae) [Source:MGI Symb	0.73184
ENSMUSG00000021311	Mtr	5-methyltetrahydrofolate-homocysteine methyltra	0.73185
ENSMUSG00000091881	Gm17146	predicted gene 17146 [Source:MGI Symbol;Acc:MG	0.73186
ENSMUSG00000005699	Pard6a	par-6 (partitioning defective 6,) homolog alpha (C.	0.73192
ENSMUSG00000034402	Kcnh5	potassium voltage-gated channel, subfamily H (eag	0.732
ENSMUSG00000018862	Otop3	otopetrin 3 [Source:MGI Symbol;Acc:MGI:1916852]	0.73201
ENSMUSG00000031074	Fgf3	fibroblast growth factor 3 [Source:MGI Symbol;Acc	0.73205
ENSMUSG00000069835	Sat2	spermidine/spermine N1-acetyl transferase 2 [Sour	0.73212
ENSMUSG00000074580	4931440P22Rik	RIKEN cDNA 4931440P22 gene [Source:MGI Symbo	0.7323
ENSMUSG00000039878	Slc39a5	solute carrier family 39 (metal ion transporter), me	0.73252
ENSMUSG00000086067	Gm16183	predicted gene 16183 [Source:MGI Symbol;Acc:MG	0.73258
ENSMUSG00000020793	Galr2	galanin receptor 2 [Source:MGI Symbol;Acc:MGI:13	0.73265
ENSMUSG00000031710	Ucp1	uncoupling protein 1 (mitochondrial, proton carrier	0.7327
ENSMUSG00000032249	Anp32a	acidic (leucine-rich) nuclear phosphoprotein 32 fam	0.7327
ENSMUSG00000051978	Erich1	glutamate rich 1 [Source:MGI Symbol;Acc:MGI:358	0.73277
ENSMUSG00000041445	Mmrn2	multimerin 2 [Source:MGI Symbol;Acc:MGI:238561	0.73281
ENSMUSG00000045382	Cxcr4	chemokine (C-X-C motif) receptor 4 [Source:MGI Sy	0.73291
ENSMUSG00000086976	Gm12316	predicted gene 12316 [Source:MGI Symbol;Acc:MG	0.73294
ENSMUSG00000093178	Snord87	small nucleolar RNA, C/D box 87 [Source:MGI Symb	0.73295
ENSMUSG00000086132	Gm13631	predicted gene 13631 [Source:MGI Symbol;Acc:MG	0.73296
ENSMUSG00000083223	Gm15016	predicted gene 15016 [Source:MGI Symbol;Acc:MG	0.73298
ENSMUSG00000085206	Gm13380	predicted gene 13380 [Source:MGI Symbol;Acc:MG	0.73299
ENSMUSG00000002992	Apoc2	apolipoprotein C-II [Source:MGI Symbol;Acc:MGI:8	0.733
ENSMUSG00000032053	Pou2af1	POU domain, class 2, associating factor 1 [Source:M	0.733
ENSMUSG00000065643	Gm25897	predicted gene, 25897 [Source:MGI Symbol;Acc:MG	0.733
ENSMUSG00000082786	Gm14489	predicted gene 14489 [Source:MGI Symbol;Acc:MG	0.733
ENSMUSG00000083603	Gm14810	predicted gene 14810 [Source:MGI Symbol;Acc:MG	0.733
ENSMUSG00000098099	Gm7456	predicted gene 7456 [Source:MGI Symbol;Acc:MGI:	0.733
ENSMUSG00000029553	Tfec	transcription factor EC [Source:MGI Symbol;Acc:MG	0.73301
ENSMUSG00000032606	Nicn1	nicolin 1 [Source:MGI Symbol;Acc:MGI:1913507]	0.73302
ENSMUSG00000082031	Gm8097	predicted gene 8097 [Source:MGI Symbol;Acc:MGI:	0.73302
ENSMUSG00000041439	Mfsd6	major facilitator superfamily domain containing 6 [	0.73305
ENSMUSG00000086810	Gm13110	predicted gene 13110 [Source:MGI Symbol;Acc:MG	0.73306
ENSMUSG00000047632	Fgfbp3	fibroblast growth factor binding protein 3 [Source:I	0.73317
ENSMUSG00000048038	4932418E24Rik	RIKEN cDNA 4932418E24 gene [Source:MGI Symbo	0.73318
ENSMUSG00000014402	Tsg101	tumor susceptibility gene 101 [Source:MGI Symbol;	0.73324
ENSMUSG00000026979	Psd4	pleckstrin and Sec7 domain containing 4 [Source:M	0.73331
ENSMUSG00000027506	Tpd52	tumor protein D52 [Source:MGI Symbol;Acc:MGI:1	0.73332
ENSMUSG00000010021	Kif19a	kinesin family member 19A [Source:MGI Symbol;Ac	0.73333
ENSMUSG00000091193	Gm17333	predicted gene, 17333 [Source:MGI Symbol;Acc:MG	0.73335

ENSMUSG00000082893	Gm11383	predicted gene 11383 [Source:MGI Symbol;Acc:MG	0.73337
ENSMUSG00000086936	Gm9697	predicted gene 9697 [Source:MGI Symbol;Acc:MGI:	0.73337
ENSMUSG00000093501	Vmn1r22	vomeronasal 1 receptor 22 [Source:MGI Symbol;Ac	0.73338
ENSMUSG00000037913	Tmem156	transmembrane protein 156 [Source:MGI Symbol;A	0.73339
ENSMUSG00000041717	Golt1a	golgi transport 1 homolog A (S. cerevisiae) [Source:	0.73339
ENSMUSG00000082431	Gm7110	predicted gene 7110 [Source:MGI Symbol;Acc:MGI:	0.73339
ENSMUSG00000080557	Gm24397	predicted gene, 24397 [Source:MGI Symbol;Acc:MG	0.7334
ENSMUSG00000030851	Ldhc	lactate dehydrogenase C [Source:MGI Symbol;Acc:M	0.73341
ENSMUSG00000052549	Arl13a	ADP-ribosylation factor-like 13A [Source:MGI Symb	0.73341
ENSMUSG00000038241	Cep250	centrosomal protein 250 [Source:MGI Symbol;Acc:M	0.73343
ENSMUSG00000097009	Gm26861	predicted gene, 26861 [Source:MGI Symbol;Acc:MG	0.73348
ENSMUSG00000018585	Atox1	ATX1 (antioxidant protein 1) homolog 1 (yeast) [Sou	0.73351
ENSMUSG00000030269	Mtmr14	myotubularin related protein 14 [Source:MGI Symb	0.73351
ENSMUSG00000038641	Akr1d1	aldo-keto reductase family 1, member D1 [Source:M	0.73355
ENSMUSG00000086928	Gm14210	predicted gene 14210 [Source:MGI Symbol;Acc:MG	0.73355
ENSMUSG00000087297	Gm15494	predicted gene 15494 [Source:MGI Symbol;Acc:MG	0.73355
ENSMUSG00000036449	Lcn8	lipocalin 8 [Source:MGI Symbol;Acc:MGI:2135945]	0.73356
ENSMUSG00000066538	Gm6254	predicted gene 6254 [Source:MGI Symbol;Acc:MGI:	0.73356
ENSMUSG00000083953	Gm14049	predicted gene 14049 [Source:MGI Symbol;Acc:MG	0.73357
ENSMUSG00000017688	Hnf4g	hepatocyte nuclear factor 4, gamma [Source:MGI S	0.73358
ENSMUSG00000074734	4933416C03Rik	RIKEN cDNA 4933416C03 gene [Source:MGI Symbo	0.73359
ENSMUSG00000001670	Tat	tyrosine aminotransferase [Source:MGI Symbol;Acc	0.7336
ENSMUSG00000009596	Taf7l	TAF7-like RNA polymerase II, TATA box binding prot	0.7336
ENSMUSG00000000216	Scnn1g	sodium channel, nonvoltage-gated 1 gamma [Sourc	0.73361
ENSMUSG00000001155	Ftcd	formiminotransferase cyclodeaminase [Source:MG	0.73361
ENSMUSG00000050633	1110035H17Rik	RIKEN cDNA 1110035H17 gene [Source:MGI Symbo	0.73364
ENSMUSG00000083250	Gm5403	predicted gene 5403 [Source:MGI Symbol;Acc:MGI:	0.73373
ENSMUSG00000075600	Zc3h3	zinc finger CCCH type containing 3 [Source:MGI Syn	0.73394
ENSMUSG00000024987	Cyp26a1	cytochrome P450, family 26, subfamily a, polypepti	0.73398
ENSMUSG00000040883	Tmem205	transmembrane protein 205 [Source:MGI Symbol;A	0.73401
ENSMUSG00000000318	Clec10a	C-type lectin domain family 10, member A [Source:	0.73409
ENSMUSG00000022757	Tfg	Trk-fused gene [Source:MGI Symbol;Acc:MGI:1338	0.73413
ENSMUSG00000087150	BC064078	cDNA sequence BC064078 [Source:MGI Symbol;Acc	0.73417
ENSMUSG00000098185	Gm9146	predicted gene 9146 [Source:MGI Symbol;Acc:MGI:	0.7342
ENSMUSG00000085310	Gm11491	predicted gene 11491 [Source:MGI Symbol;Acc:MG	0.73421
ENSMUSG00000085836	Gm13074	predicted gene 13074 [Source:MGI Symbol;Acc:MG	0.73421
ENSMUSG00000086109	Gm13391	predicted gene 13391 [Source:MGI Symbol;Acc:MG	0.73421
ENSMUSG00000087230	Mroh3	maestro heat-like repeat family member 3 [Source:	0.73421
ENSMUSG00000088993	Gm22770	predicted gene, 22770 [Source:MGI Symbol;Acc:MG	0.73422
ENSMUSG00000085228	Gm14376	predicted gene 14376 [Source:MGI Symbol;Acc:MG	0.73423
ENSMUSG00000085445	Gm16348	predicted gene 16348 [Source:MGI Symbol;Acc:MG	0.73423
ENSMUSG00000081447	Gm13870	predicted gene 13870 [Source:MGI Symbol;Acc:MG	0.73425
ENSMUSG00000082393	Gm15028	predicted gene 15028 [Source:MGI Symbol;Acc:MG	0.73425
ENSMUSG00000069267	Hist1h3b	histone cluster 1, H3b [Source:MGI Symbol;Acc:MG	0.73426
ENSMUSG00000057710	9630041A04Rik	RIKEN cDNA 9630041A04 gene [Source:MGI Symbo	0.73427
ENSMUSG00000053164	Gpr21	G protein-coupled receptor 21 [Source:MGI Symbo	0.73429
ENSMUSG00000035228	Ccdc106	coiled-coil domain containing 106 [Source:MGI Sym	0.73432

ENSMUSG00000033900	Map9	microtubule-associated protein 9 [Source:MGI Sym	0.73437
ENSMUSG00000084981	Gm15962	predicted gene 15962 [Source:MGI Symbol;Acc:MG	0.73437
ENSMUSG00000097498	Zfp850	zinc finger protein 850 [Source:MGI Symbol;Acc:M	0.73469
ENSMUSG00000031506	Ptpn7	protein tyrosine phosphatase, non-receptor type 7	0.73474
ENSMUSG00000036760	Kcnk9	potassium channel, subfamily K, member 9 [Source	0.73479
ENSMUSG00000044703	Phf11a	PHD finger protein 11A [Source:MGI Symbol;Acc:M	0.73485
ENSMUSG00000092981	Mir5125	microRNA 5125 [Source:MGI Symbol;Acc:MGI:4950	0.73498
ENSMUSG00000069255	Dusp22	dual specificity phosphatase 22 [Source:MGI Symbo	0.73505
ENSMUSG00000073728	Tmem51os1	Tmem51 opposite strand 1 [Source:MGI Symbol;Ac	0.7351
ENSMUSG00000042828	Trim72	tripartite motif-containing 72 [Source:MGI Symbol;	0.73526
ENSMUSG00000057706	Mex3b	mex3 homolog B (C. elegans) [Source:MGI Symbol;	0.73528
ENSMUSG00000051678	Pcdhb6	protocadherin beta 6 [Source:MGI Symbol;Acc:MGI	0.73534
ENSMUSG00000084893	Hba-ps4	hemoglobin alpha, pseudogene 4 [Source:MGI Sym	0.7357
ENSMUSG00000028412	Slc44a1	solute carrier family 44, member 1 [Source:MGI Sy	0.73576
ENSMUSG00000044199	S1pr4	sphingosine-1-phosphate receptor 4 [Source:MGI S	0.73602
ENSMUSG00000097098	9330111N05Rik	RIKEN cDNA 9330111N05 gene [Source:MGI Symbo	0.73605
ENSMUSG00000021998	Lcp1	lymphocyte cytosolic protein 1 [Source:MGI Symbo	0.73606
ENSMUSG00000020877	Scrn2	secernin 2 [Source:MGI Symbol;Acc:MGI:1343092]	0.73616
ENSMUSG00000024217	Snrpc	U1 small nuclear ribonucleoprotein C [Source:MGI	0.73622
ENSMUSG00000038374	Rbm8a	RNA binding motif protein 8a [Source:MGI Symbol;	0.73622
ENSMUSG00000031760	Mt3	metallothionein 3 [Source:MGI Symbol;Acc:MGI:97	0.73636
ENSMUSG00000030534	Vps33b	vacuolar protein sorting 33B (yeast) [Source:MGI Sy	0.73641
ENSMUSG00000092284	Gm8801	predicted gene 8801 [Source:MGI Symbol;Acc:MGI	0.73646
ENSMUSG00000039330	Tsga10ip	testis specific 10 interacting protein [Source:MGI Sy	0.73648
ENSMUSG00000019842	Traf3ip2	TRAF3 interacting protein 2 [Source:MGI Symbol;Ac	0.7365
ENSMUSG00000048096	Lmod1	leiomodin 1 (smooth muscle) [Source:MGI Symbol;	0.73652
ENSMUSG00000046668	Cxxc5	CXXC finger 5 [Source:MGI Symbol;Acc:MGI:191464	0.73672
ENSMUSG00000031578	Mak16	MAK16 homolog (S. cerevisiae) [Source:MGI Symbo	0.73675
ENSMUSG00000097058	4930556M19Rik	RIKEN cDNA 4930556M19 gene [Source:MGI Symbo	0.73675
ENSMUSG00000001755	Coasy	Coenzyme A synthase [Source:MGI Symbol;Acc:MG	0.73677
ENSMUSG00000069855	Slc47a2	solute carrier family 47, member 2 [Source:MGI Sy	0.73678
ENSMUSG00000024258	Polr2d	polymerase (RNA) II (DNA directed) polypeptide D [	0.73693
ENSMUSG00000097073	9430037G07Rik	RIKEN cDNA 9430037G07 gene [Source:MGI Symbo	0.73694
ENSMUSG00000086269	Gm16154	predicted gene 16154 [Source:MGI Symbol;Acc:MG	0.73729
ENSMUSG00000078695	Cisd3	CDGSH iron sulfur domain 3 [Source:MGI Symbol;A	0.73734
ENSMUSG00000023990	Tfeb	transcription factor EB [Source:MGI Symbol;Acc:M	0.73754
ENSMUSG00000087700	Gm15283	predicted gene 15283 [Source:MGI Symbol;Acc:MG	0.73756
ENSMUSG00000057818	1600029O15Rik	RIKEN cDNA 1600029O15 gene [Source:MGI Symbo	0.73763
ENSMUSG00000027774	Gfm1	G elongation factor, mitochondrial 1 [Source:MGI S	0.73767
ENSMUSG00000056486	Chn1	chimerin (chimaerin) 1 [Source:MGI Symbol;Acc:M	0.73771
ENSMUSG00000036473	Tbc1d24	TBC1 domain family, member 24 [Source:MGI Symb	0.73778
ENSMUSG00000090235	Gm16244	predicted gene 16244 [Source:MGI Symbol;Acc:MG	0.73783
ENSMUSG00000043753	Dmrta1	doublesex and mab-3 related transcription factor li	0.73786
ENSMUSG00000030737	Slco2b1	solute carrier organic anion transporter family, me	0.73791
ENSMUSG00000015355	Cd48	CD48 antigen [Source:MGI Symbol;Acc:MGI:88339]	0.73793
ENSMUSG00000050350	Gpr18	G protein-coupled receptor 18 [Source:MGI Symbo	0.73798
ENSMUSG00000026514	Cnih3	cornichon homolog 3 (Drosophila) [Source:MGI Syn	0.738

ENSMUSG00000087113	Gm11714	predicted gene 11714 [Source:MGI Symbol;Acc:MG	0.73814
ENSMUSG00000038286	Bphl	biphenyl hydrolase-like (serine hydrolase, breast ep	0.73816
ENSMUSG00000021051	Ppp2r5e	protein phosphatase 2, regulatory subunit B (B56),	0.73849
ENSMUSG00000091816	Gm17141	predicted gene 17141 [Source:MGI Symbol;Acc:MG	0.73855
ENSMUSG00000029661	Col1a2	collagen, type I, alpha 2 [Source:MGI Symbol;Acc:M	0.73863
ENSMUSG00000089942	Pira2	paired-Ig-like receptor A2 [Source:MGI Symbol;Acc	0.73882
ENSMUSG00000097260	Gm5706	predicted gene 5706 [Source:MGI Symbol;Acc:MGI:	0.73882
ENSMUSG00000061390	Gm7866	predicted gene 7866 [Source:MGI Symbol;Acc:MGI:	0.73884
ENSMUSG00000058147	Xlr3c	X-linked lymphocyte-regulated 3C [Source:MGI Sym	0.73885
ENSMUSG00000074737	C530025M09Rik	RIKEN cDNA C530025M09 gene [Source:MGI Symbo	0.73885
ENSMUSG00000080816	Gm12447	predicted gene 12447 [Source:MGI Symbol;Acc:MG	0.73885
ENSMUSG00000048310	Pskh1	protein serine kinase H1 [Source:MGI Symbol;Acc:M	0.73912
ENSMUSG00000061186	Sfmbt2	Scm-like with four mbt domains 2 [Source:MGI Sym	0.73912
ENSMUSG00000070699	Sars2	seryl-aminoacyl-tRNA synthetase 2 [Source:MGI Sy	0.73919
ENSMUSG00000083669	Gm10169	predicted pseudogene 10169 [Source:MGI Symbol;	0.73922
ENSMUSG00000064901	Snora21	small nucleolar RNA, H/ACA box 21 [Source:MGI Sy	0.73926
ENSMUSG00000064741	Snord14a	small nucleolar RNA, C/D box 14A [Source:MGI Sym	0.73928
ENSMUSG00000088689	Scarna17	small Cajal body-specific RNA 17 [Source:MGI Symb	0.73932
ENSMUSG00000024388	Myo7b	myosin VIIB [Source:MGI Symbol;Acc:MGI:107709]	0.73939
ENSMUSG00000027596	a	nonagouti [Source:MGI Symbol;Acc:MGI:87853]	0.73939
ENSMUSG00000030359	Pzp	pregnancy zone protein [Source:MGI Symbol;Acc:M	0.73939
ENSMUSG00000053333	Dis3l2	DIS3 mitotic control homolog (S. cerevisiae)-like 2 [	0.7394
ENSMUSG00000092353	Gm20539	predicted gene 20539 [Source:MGI Symbol;Acc:MG	0.73942
ENSMUSG00000043487	Acot6	acyl-CoA thioesterase 6 [Source:MGI Symbol;Acc:M	0.73943
ENSMUSG00000070306	Ccdc153	coiled-coil domain containing 153 [Source:MGI Sym	0.73943
ENSMUSG00000058669	Nkx2-9	NK2 homeobox 9 [Source:MGI Symbol;Acc:MGI:127	0.73946
ENSMUSG00000037568	Vash2	vasohibin 2 [Source:MGI Symbol;Acc:MGI:2444826	0.73951
ENSMUSG00000086474	9130204K15Rik	RIKEN cDNA 9130204K15 gene [Source:MGI Symbo	0.73966
ENSMUSG00000028480	Glipr2	GLI pathogenesis-related 2 [Source:MGI Symbol;Ac	0.73969
ENSMUSG00000088631	Gm22311	predicted gene, 22311 [Source:MGI Symbol;Acc:M	0.73971
ENSMUSG00000082128	Gm11824	predicted gene 11824 [Source:MGI Symbol;Acc:MG	0.73973
ENSMUSG00000096171	Gm8055	predicted pseudogene 8055 [Source:MGI Symbol;A	0.73977
ENSMUSG00000086750	Gm11770	predicted gene 11770 [Source:MGI Symbol;Acc:MG	0.73979
ENSMUSG00000097514	Gm17619	predicted gene, 17619 [Source:MGI Symbol;Acc:M	0.73981
ENSMUSG00000068587	Mgam	maltase-glucoamylase [Source:MGI Symbol;Acc:MG	0.73985
ENSMUSG00000088464	Mir1970	microRNA 1970 [Source:MGI Symbol;Acc:MGI:3837	0.73986
ENSMUSG00000096086	Gm4761	predicted gene 4761 [Source:MGI Symbol;Acc:MGI:	0.73986
ENSMUSG00000056729	Gm10006	predicted gene 10006 [Source:MGI Symbol;Acc:MG	0.73987
ENSMUSG00000087685	1700122E12Rik	RIKEN cDNA 1700122E12 gene [Source:MGI Symbo	0.73988
ENSMUSG00000027857	Tshb	thyroid stimulating hormone, beta subunit [Source:	0.73989
ENSMUSG00000086128	1700091E21Rik	RIKEN cDNA 1700091E21 gene [Source:MGI Symbo	0.73989
ENSMUSG00000096699	Rps19-ps4	ribosomal protein S19, pseudogene 4 [Source:MGI	0.73994
ENSMUSG00000097052	Snhg7	small nucleolar RNA host gene (non-protein coding	0.74013
ENSMUSG00000054280	Prr14l	proline rich 14-like [Source:MGI Symbol;Acc:MGI:2	0.74018
ENSMUSG00000097282	5031415H12Rik	RIKEN cDNA 5031415H12 gene [Source:MGI Symbo	0.74023
ENSMUSG00000018809	Smyd4	SET and MYND domain containing 4 [Source:MGI Sy	0.74024
ENSMUSG00000069310	Hist1h3c	histone cluster 1, H3c [Source:MGI Symbol;Acc:MG	0.74027

ENSMUSG00000097486	Gm17733	predicted gene, 17733 [Source:MGI Symbol;Acc:M(	0.74028
ENSMUSG00000097569	Gm26640	predicted gene, 26640 [Source:MGI Symbol;Acc:M(	0.74028
ENSMUSG00000051354	Samd3	sterile alpha motif domain containing 3 [Source:M(	0.7403
ENSMUSG00000089988	Gm16238	predicted gene 16238 [Source:MGI Symbol;Acc:MG	0.7403
ENSMUSG00000039264	Gimap3	GTPase, IMAP family member 3 [Source:MGI Symb	0.74032
ENSMUSG00000081285	Gm18859	predicted gene, 18859 [Source:MGI Symbol;Acc:M(	0.74032
ENSMUSG00000026398	Nr5a2	nuclear receptor subfamily 5, group A, member 2 [	0.74033
ENSMUSG00000081026	Gm11855	predicted gene 11855 [Source:MGI Symbol;Acc:MG	0.74033
ENSMUSG00000083875	Gm13831	predicted gene 13831 [Source:MGI Symbol;Acc:MG	0.74033
ENSMUSG00000028460	Sit1	suppression inducing transmembrane adaptor 1 [S(	0.74035
ENSMUSG00000025528	2010106E10Rik	RIKEN cDNA 2010106E10 gene [Source:MGI Symbo	0.74038
ENSMUSG00000079076	Gm3086	predicted gene 3086 [Source:MGI Symbol;Acc:MGI:	0.74038
ENSMUSG00000097358	Gm26773	predicted gene, 26773 [Source:MGI Symbol;Acc:M(	0.74042
ENSMUSG00000047767	Atg16l2	autophagy related 16-like 2 (S. cerevisiae) [Source:I	0.74054
ENSMUSG00000001379	Apbb3	amyloid beta (A4) precursor protein-binding, family	0.74065
ENSMUSG00000017718	Afmid	arylformamidase [Source:MGI Symbol;Acc:MGI:244	0.74068
ENSMUSG00000084004	Gm12047	predicted gene 12047 [Source:MGI Symbol;Acc:MG	0.74073
ENSMUSG00000035191	Rfpl4	ret finger protein-like 4 [Source:MGI Symbol;Acc:M	0.74074
ENSMUSG00000085875	Gm12905	predicted gene 12905 [Source:MGI Symbol;Acc:MG	0.7409
ENSMUSG00000020376	Rnf130	ring finger protein 130 [Source:MGI Symbol;Acc:M(	0.74092
ENSMUSG00000063895	Nupl1	nucleoporin like 1 [Source:MGI Symbol;Acc:MGI:19	0.74092
ENSMUSG00000030722	Nfatc2ip	nuclear factor of activated T cells, cytoplasmic, calc	0.74103
ENSMUSG00000030340	Scnn1a	sodium channel, nonvoltage-gated 1 alpha [Source:	0.74106
ENSMUSG00000047485	Klhl34	kelch-like 34 [Source:MGI Symbol;Acc:MGI:268523	0.74114
ENSMUSG00000031662	Snx20	sorting nexin 20 [Source:MGI Symbol;Acc:MGI:1918	0.74118
ENSMUSG00000081048	Gm11760	predicted gene 11760 [Source:MGI Symbol;Acc:MG	0.74118
ENSMUSG00000008845	Cd163	CD163 antigen [Source:MGI Symbol;Acc:MGI:21359	0.74126
ENSMUSG00000042354	Gnl3	guanine nucleotide binding protein-like 3 (nucleola	0.74131
ENSMUSG00000024437	Gm8615	predicted pseudogene 8615 [Source:MGI Symbol;A	0.74132
ENSMUSG00000051000	Fam160a1	family with sequence similarity 160, member A1 [S(	0.74143
ENSMUSG00000087075	A230065H16Rik	RIKEN cDNA A230065H16 gene [Source:MGI Symbo	0.74145
ENSMUSG00000088695	Gm22334	predicted gene, 22334 [Source:MGI Symbol;Acc:M(	0.74145
ENSMUSG00000022114	Spry2	sprouty homolog 2 (Drosophila) [Source:MGI Symb	0.74147
ENSMUSG00000064925	Snora62	small nucleolar RNA, H/ACA box 62 [Source:MGI Sy	0.74147
ENSMUSG00000083006	Gm15715	predicted gene 15715 [Source:MGI Symbol;Acc:MG	0.74147
ENSMUSG00000046764	A530053G22Rik	RIKEN cDNA A530053G22 gene [Source:MGI Symbo	0.74148
ENSMUSG00000079304	4933413G19Rik	RIKEN cDNA 4933413G19 gene [Source:MGI Symbo	0.74149
ENSMUSG00000097516	4930467D21Rik	RIKEN cDNA 4930467D21 gene [Source:MGI Symbo	0.74149
ENSMUSG00000064417	DQ267102	snoRNA DQ267102 [Source:MGI Symbol;Acc:MGI:5	0.7415
ENSMUSG00000002808	Epdr1	ependymin related protein 1 (zebrafish) [Source:M(	0.74158
ENSMUSG00000036890	Gtdc1	glycosyltransferase-like domain containing 1 [Sourc	0.74169
ENSMUSG00000041468	Gpr12	G-protein coupled receptor 12 [Source:MGI Symbo	0.74174
ENSMUSG00000064294	Aox3	aldehyde oxidase 3 [Source:MGI Symbol;Acc:MGI:1	0.74175
ENSMUSG00000081650	Gm16181	predicted gene 16181 [Source:MGI Symbol;Acc:MG	0.7418
ENSMUSG00000036913	Trim67	tripartite motif-containing 67 [Source:MGI Symbol;	0.74185
ENSMUSG00000072974	Gm4787	predicted gene 4787 [Source:MGI Symbol;Acc:MGI:	0.74189
ENSMUSG00000001119	Col6a1	collagen, type VI, alpha 1 [Source:MGI Symbol;Acc:	0.74195

ENSMUSG00000080746	Rpsa-ps12	ribosomal protein SA, pseudogene 12 [Source:MGI	0.74219
ENSMUSG00000022742	Cpox	coproporphyrinogen oxidase [Source:MGI Symbol;A	0.74221
ENSMUSG00000036162	1700066J24Rik	RIKEN cDNA 1700066J24 gene [Source:MGI Symbol	0.74221
ENSMUSG00000085816	Gm13052	predicted gene 13052 [Source:MGI Symbol;Acc:MG	0.74221
ENSMUSG00000081690	Gm12317	predicted gene 12317 [Source:MGI Symbol;Acc:MG	0.74222
ENSMUSG00000091685	Gm17359	predicted gene, 17359 [Source:MGI Symbol;Acc:M	0.74222
ENSMUSG00000083536	Gm15808	predicted gene 15808 [Source:MGI Symbol;Acc:MG	0.74223
ENSMUSG00000065806	n-R5s69	nuclear encoded rRNA 5S 69 [Source:MGI Symbol;A	0.74224
ENSMUSG00000081621	Gm12793	predicted gene 12793 [Source:MGI Symbol;Acc:MG	0.74224
ENSMUSG00000049530	Clrn2	clarin 2 [Source:MGI Symbol;Acc:MGI:3646230]	0.74227
ENSMUSG00000035364	4930524J08Rik	RIKEN cDNA 4930524J08 gene [Source:MGI Symbol	0.74229
ENSMUSG00000036214	Znrd1as	Znrd1 antisense [Source:MGI Symbol;Acc:MGI:1923	0.74234
ENSMUSG00000053080	2700081O15Rik	RIKEN cDNA 2700081O15 gene [Source:MGI Symbo	0.74234
ENSMUSG00000036985	Zdhhc9	zinc finger, DHHC domain containing 9 [Source:MG	0.74236
ENSMUSG00000055652	Klhl25	kelch-like 25 [Source:MGI Symbol;Acc:MGI:266803	0.74241
ENSMUSG00000022314	Rad21	RAD21 homolog (S. pombe) [Source:MGI Symbol;A	0.74256
ENSMUSG00000022550	Adck5	aarF domain containing kinase 5 [Source:MGI Symb	0.74263
ENSMUSG00000026023	Cdk15	cyclin-dependent kinase 15 [Source:MGI Symbol;Ac	0.74264
ENSMUSG00000046157	Tmem229b	transmembrane protein 229B [Source:MGI Symbol;	0.74264
ENSMUSG00000047888	Tnrc6b	trinucleotide repeat containing 6b [Source:MGI Sym	0.74269
ENSMUSG00000026189	Pecr	peroxisomal trans-2-enoyl-CoA reductase [Source:N	0.74279
ENSMUSG00000015750	Aph1a	anterior pharynx defective 1a homolog (C. elegans)	0.74284
ENSMUSG00000019767	Ccdc170	coiled-coil domain containing 170 [Source:MGI Sym	0.74285
ENSMUSG00000054181	A930012O16Rik	RIKEN cDNA A930012O16 gene [Source:MGI Symbo	0.74286
ENSMUSG00000087252	Gm14379	predicted gene 14379 [Source:MGI Symbol;Acc:MG	0.7429
ENSMUSG00000079659	Tmem243	transmembrane protein 243, mitochondrial [Source	0.74291
ENSMUSG00000042246	Tmc7	transmembrane channel-like gene family 7 [Source	0.74295
ENSMUSG00000025949	Pikfyve	phosphoinositide kinase, FYVE finger containing [So	0.74301
ENSMUSG00000060794	Tssk5	testis-specific serine kinase 5 [Source:MGI Symbol;	0.74307
ENSMUSG00000084149	Gm12944	predicted gene 12944 [Source:MGI Symbol;Acc:MG	0.74322
ENSMUSG00000052738	Suclg1	succinate-CoA ligase, GDP-forming, alpha subunit [	0.74324
ENSMUSG00000097399	Gm26555	predicted gene, 26555 [Source:MGI Symbol;Acc:M	0.74328
ENSMUSG00000071723	Gspt2	G1 to S phase transition 2 [Source:MGI Symbol;Acc	0.74345
ENSMUSG00000001416	Cct3	chaperonin containing Tcp1, subunit 3 (gamma) [So	0.74352
ENSMUSG00000029309	Sparcl1	SPARC-like 1 [Source:MGI Symbol;Acc:MGI:108110]	0.74354
ENSMUSG00000036864	BC053749	cDNA sequence BC053749 [Source:MGI Symbol;Acc	0.74361
ENSMUSG00000002329	Mdp1	magnesium-dependent phosphatase 1 [Source:MG	0.74391
ENSMUSG00000026255	Efhd1	EF hand domain containing 1 [Source:MGI Symbol;A	0.74393
ENSMUSG00000054003	Tdrd9	tudor domain containing 9 [Source:MGI Symbol;Acc	0.74397
ENSMUSG00000029467	Atp2a2	ATPase, Ca++ transporting, cardiac muscle, slow tw	0.74408
ENSMUSG00000031803	B3gnt3	UDP-GlcNAc:betaGal beta-1,3-N-acetylglucosaminy	0.74413
ENSMUSG00000030350	Prmt8	protein arginine N-methyltransferase 8 [Source:MG	0.74416
ENSMUSG00000091604	Gm17349	predicted gene, 17349 [Source:MGI Symbol;Acc:M	0.74416
ENSMUSG00000020107	Anapc16	anaphase promoting complex subunit 16 [Source:M	0.74419
ENSMUSG00000004894	Hapln2	hyaluronan and proteoglycan link protein 2 [Source	0.74442
ENSMUSG00000002804	Nudt14	nudix (nucleoside diphosphate linked moiety X)-typ	0.74444
ENSMUSG00000012187	Mogat1	monoacylglycerol O-acyltransferase 1 [Source:MGI	0.74446

ENSMUSG00000038462	Uqcrfs1	ubiquinol-cytochrome c reductase, Rieske iron-sulf	0.74454
ENSMUSG00000025408	Ddit3	DNA-damage inducible transcript 3 [Source:MGI Sy	0.74459
ENSMUSG00000082313	Gm11701	predicted gene 11701 [Source:MGI Symbol;Acc:MG	0.74467
ENSMUSG00000027716	Trpc3	transient receptor potential cation channel, subfam	0.74471
ENSMUSG00000045333	Zfp423	zinc finger protein 423 [Source:MGI Symbol;Acc:MG	0.74478
ENSMUSG00000087692	Gm11940	predicted gene 11940 [Source:MGI Symbol;Acc:MG	0.74479
ENSMUSG00000092262	Gm20434	predicted gene 20434 [Source:MGI Symbol;Acc:MG	0.7449
ENSMUSG00000019774	Mtrf1l	mitochondrial translational release factor 1-like [So	0.74499
ENSMUSG00000043415	Otud1	OTU domain containing 1 [Source:MGI Symbol;Acc:	0.74515
ENSMUSG00000097084	Foxl1	forkhead box L1 [Source:MGI Symbol;Acc:MGI:134	0.74531
ENSMUSG00000031949	Adat1	adenosine deaminase, tRNA-specific 1 [Source:MGI	0.7454
ENSMUSG00000085560	C230038L03Rik	RIKEN cDNA C230038L03 gene [Source:MGI Symbo	0.74559
ENSMUSG00000089621	Gm25445	predicted gene, 25445 [Source:MGI Symbol;Acc:MG	0.7456
ENSMUSG00000029247	Paics	phosphoribosylaminoimidazole carboxylase, phosp	0.74561
ENSMUSG00000018796	Acsl1	acyl-CoA synthetase long-chain family member 1 [S	0.74565
ENSMUSG00000053925	Gm9929	predicted gene 9929 [Source:MGI Symbol;Acc:MGI	0.7457
ENSMUSG00000017210	Med24	mediator complex subunit 24 [Source:MGI Symbol;	0.74578
ENSMUSG00000052407	Ccdc171	coiled-coil domain containing 171 [Source:MGI Sym	0.74578
ENSMUSG00000022377	Asap1	ArfGAP with SH3 domain, ankyrin repeat and PH do	0.74597
ENSMUSG00000022041	Chrna2	cholinergic receptor, nicotinic, alpha polypeptide 2	0.74599
ENSMUSG00000041491	Cep78	centrosomal protein 78 [Source:MGI Symbol;Acc:M	0.74607
ENSMUSG00000085888	Gm12224	predicted gene 12224 [Source:MGI Symbol;Acc:MG	0.74608
ENSMUSG00000044957	Pp2d1	protein phosphatase 2C-like domain containing 1 [S	0.74617
ENSMUSG00000026873	Phf19	PHD finger protein 19 [Source:MGI Symbol;Acc:MG	0.74629
ENSMUSG00000000402	Egfl6	EGF-like-domain, multiple 6 [Source:MGI Symbol;A	0.74633
ENSMUSG00000032218	Ccnb2	cyclin B2 [Source:MGI Symbol;Acc:MGI:88311]	0.74635
ENSMUSG00000078796	Zfp541	zinc finger protein 541 [Source:MGI Symbol;Acc:MG	0.74658
ENSMUSG00000020231	Dip2a	DIP2 disco-interacting protein 2 homolog A (Drosop	0.74661
ENSMUSG00000073702	Rpl31	ribosomal protein L31 [Source:MGI Symbol;Acc:MG	0.74664
ENSMUSG00000040289	Hey1	hairy/enhancer-of-split related with YRPW motif 1	0.74678
ENSMUSG00000000275	Trim25	tripartite motif-containing 25 [Source:MGI Symbol;	0.74697
ENSMUSG00000098175	Gm7545	predicted gene 7545 [Source:MGI Symbol;Acc:MGI	0.74704
ENSMUSG00000037375	Hhat	hedgehog acyltransferase [Source:MGI Symbol;Acc	0.74717
ENSMUSG00000061046	Haghl	hydroxyacylglutathione hydrolase-like [Source:MGI	0.74717
ENSMUSG00000073413	Ly6g6d	lymphocyte antigen 6 complex, locus G6D [Source:	0.74721
ENSMUSG00000087968	Gm25395	predicted gene, 25395 [Source:MGI Symbol;Acc:MG	0.74734
ENSMUSG00000032564	Cpne4	copine IV [Source:MGI Symbol;Acc:MGI:1921270]	0.74739
ENSMUSG00000043556	Fbxl7	F-box and leucine-rich repeat protein 7 [Source:MG	0.7474
ENSMUSG00000045409	Trim39	tripartite motif-containing 39 [Source:MGI Symbol;	0.7474
ENSMUSG00000062496	4930431F12Rik	RIKEN cDNA 4930431F12 gene [Source:MGI Symbo	0.74743
ENSMUSG00000090561	Gm17207	predicted gene 17207 [Source:MGI Symbol;Acc:MG	0.74745
ENSMUSG00000070858	Gm1673	predicted gene 1673 [Source:MGI Symbol;Acc:MGI	0.74751
ENSMUSG00000022994	Adcy6	adenylate cyclase 6 [Source:MGI Symbol;Acc:MGI:8	0.74753
ENSMUSG00000036123	Slc9a3	solute carrier family 9 (sodium/hydrogen exchange	0.74753
ENSMUSG00000046991	Wdr27	WD repeat domain 27 [Source:MGI Symbol;Acc:MG	0.74759
ENSMUSG00000024240	Epc1	enhancer of polycomb homolog 1 (Drosophila) [Sou	0.74767
ENSMUSG00000049892	Rasd1	RAS, dexamethasone-induced 1 [Source:MGI Symb	0.74769

ENSMUSG00000073388	A330017A19Rik	RIKEN cDNA A330017A19 gene [Source:MGI Symbo	0.74772
ENSMUSG00000022103	Gfra2	glial cell line derived neurotrophic factor family rec	0.74782
ENSMUSG00000024134	Six2	sine oculis-related homeobox 2 [Source:MGI Symbo	0.74793
ENSMUSG00000018669	Cdk5rap3	CDK5 regulatory subunit associated protein 3 [Sour	0.74807
ENSMUSG00000045989	4930451I11Rik	RIKEN cDNA 4930451I11 gene [Source:MGI Symbol	0.74819
ENSMUSG00000024122	Pdpk1	3-phosphoinositide dependent protein kinase 1 [So	0.7483
ENSMUSG00000037337	Map4k1	mitogen-activated protein kinase kinase kinase kina	0.74846
ENSMUSG00000074608	Zfp850	zinc finger protein 850 [Source:MGI Symbol;Acc:M	0.74869
ENSMUSG00000086567	Gm2830	predicted gene 2830 [Source:MGI Symbol;Acc:MGI:	0.74873
ENSMUSG00000055003	Lrtm2	leucine-rich repeats and transmembrane domains 2	0.74879
ENSMUSG00000036940	Kdm1a	lysine (K)-specific demethylase 1A [Source:MGI Sym	0.74881
ENSMUSG00000066979	Bub3	budding uninhibited by benzimidazoles 3 homolog	0.74885
ENSMUSG00000015652	Steap1	six transmembrane epithelial antigen of the prostat	0.74891
ENSMUSG00000025105	Bnc1	basonuclin 1 [Source:MGI Symbol;Acc:MGI:109716	0.74899
ENSMUSG00000019143	Hars2	histidyl-tRNA synthetase 2, mitochondrial (putative	0.74901
ENSMUSG00000000759	Tubgcp3	tubulin, gamma complex associated protein 3 [Sour	0.74905
ENSMUSG00000022389	Tef	thyrotroph embryonic factor [Source:MGI Symbol;A	0.7491
ENSMUSG00000097277	2900076A07Rik	RIKEN cDNA 2900076A07 gene [Source:MGI Symbo	0.7491
ENSMUSG00000087038	2900079G21Rik	RIKEN cDNA 2900079G21 gene [Source:MGI Symbo	0.74912
ENSMUSG00000086769	Gm15587	predicted gene 15587 [Source:MGI Symbol;Acc:MG	0.74921
ENSMUSG00000093499	Gm25442	predicted gene, 25442 [Source:MGI Symbol;Acc:M	0.74936
ENSMUSG00000032702	Kank1	KN motif and ankyrin repeat domains 1 [Source:MG	0.74951
ENSMUSG00000086537	Nespos	neuroendocrine secretory protein opposite strand	0.74956
ENSMUSG00000041062	Mslnl	mesothelin-like [Source:MGI Symbol;Acc:MGI:3607	0.74961
ENSMUSG00000095504	Gm22446	predicted gene, 22446 [Source:MGI Symbol;Acc:M	0.74963
ENSMUSG00000001520	Nrip2	nuclear receptor interacting protein 2 [Source:MGI	0.74972
ENSMUSG00000085109	Gm15356	predicted gene 15356 [Source:MGI Symbol;Acc:MG	0.74981
ENSMUSG00000035007	Rundc1	RUN domain containing 1 [Source:MGI Symbol;Acc:	0.74983
ENSMUSG00000085499	Gm15713	predicted gene 15713 [Source:MGI Symbol;Acc:MG	0.74984
ENSMUSG00000025290	Rps24	ribosomal protein S24 [Source:MGI Symbol;Acc:MG	0.74987
ENSMUSG00000055065	Ddx17	DEAD (Asp-Glu-Ala-Asp) box polypeptide 17 [Sourc	0.75003
ENSMUSG00000085894	Gm15832	predicted gene 15832 [Source:MGI Symbol;Acc:MG	0.75004
ENSMUSG00000078380	Gm6096	predicted gene 6096 [Source:MGI Symbol;Acc:MGI:	0.75019
ENSMUSG00000096753	Fam181a	family with sequence similarity 181, member A [Sou	0.75028
ENSMUSG00000032846	Zswim6	zinc finger SWIM-type containing 6 [Source:MGI Sy	0.75029
ENSMUSG00000030649	Anapc15	anaphase prompoting complex C subunit 15 [Sourc	0.75043
ENSMUSG00000049515	Espnl	espin-like [Source:MGI Symbol;Acc:MGI:2685402]	0.75052
ENSMUSG00000090160	4930480K15Rik	RIKEN cDNA 4930480K15 gene [Source:MGI Symbo	0.75054
ENSMUSG00000046108	Il17c	interleukin 17C [Source:MGI Symbol;Acc:MGI:2446	0.75058
ENSMUSG00000074388	Gm5544	predicted gene 5544 [Source:MGI Symbol;Acc:MGI:	0.75058
ENSMUSG00000087492	Gm15700	predicted gene 15700 [Source:MGI Symbol;Acc:MG	0.75064
ENSMUSG00000028179	Cth	cystathionase (cystathionine gamma-lyase) [Source	0.75073
ENSMUSG00000062309	Rpp25	ribonuclease P/MRP 25 subunit [Source:MGI Symbo	0.75081
ENSMUSG00000025190	Got1	glutamate oxaloacetate transaminase 1, soluble [So	0.75083
ENSMUSG00000025006	Sorbs1	sorbin and SH3 domain containing 1 [Source:MGI S	0.75087
ENSMUSG00000062175	Tgif2	TGFB-induced factor homeobox 2 [Source:MGI Sym	0.75087
ENSMUSG00000030525	Chrna7	cholinergic receptor, nicotinic, alpha polypeptide 7	0.75104

ENSMUSG00000093910	Zfp853	zinc finger protein 853 [Source:MGI Symbol;Acc:MG	0.75109
ENSMUSG00000007021	Syngr3	synaptogyrin 3 [Source:MGI Symbol;Acc:MGI:1341	0.75118
ENSMUSG00000022807	Osbpl11	oxysterol binding protein-like 11 [Source:MGI Symb	0.75118
ENSMUSG00000000247	Lhx2	LIM homeobox protein 2 [Source:MGI Symbol;Acc:I	0.75119
ENSMUSG00000025203	Scd2	stearoyl-Coenzyme A desaturase 2 [Source:MGI Syr	0.75119
ENSMUSG00000075587	Gm11531	predicted gene 11531 [Source:MGI Symbol;Acc:MG	0.75124
ENSMUSG00000001417	Rhbg	Rhesus blood group-associated B glycoprotein [Sou	0.75125
ENSMUSG00000022601	Rpl24	ribosomal protein L24 [Source:MGI Symbol;Acc:MG	0.75131
ENSMUSG00000085625	Gm8813	predicted gene 8813 [Source:MGI Symbol;Acc:MGI:	0.75136
ENSMUSG00000040429	Mterf	mitochondrial transcription termination factor [Sou	0.75157
ENSMUSG00000063856	Gpx1	glutathione peroxidase 1 [Source:MGI Symbol;Acc:I	0.75179
ENSMUSG00000021133	4933426M11Rik	RIKEN cDNA 4933426M11 gene [Source:MGI Symbo	0.7519
ENSMUSG00000003166	Dgcr2	DiGeorge syndrome critical region gene 2 [Source:N	0.75198
ENSMUSG00000026922	Agpat2	1-acylglycerol-3-phosphate O-acyltransferase 2 (lys	0.75199
ENSMUSG00000041671	Pyroxd1	pyridine nucleotide-disulphide oxidoreductase dom	0.752
ENSMUSG00000045251	Zfp688	zinc finger protein 688 [Source:MGI Symbol;Acc:MG	0.75231
ENSMUSG00000020940	1700023F06Rik	RIKEN cDNA 1700023F06 gene [Source:MGI Symbo	0.75243
ENSMUSG00000035923	Myf6	myogenic factor 6 [Source:MGI Symbol;Acc:MGI:97	0.75265
ENSMUSG00000025170	Rab40b	Rab40b, member RAS oncogene family [Source:MG	0.75275
ENSMUSG00000096056	Gm21986	predicted gene 21986 [Source:MGI Symbol;Acc:MG	0.75277
ENSMUSG00000039865	Slc44a3	solute carrier family 44, member 3 [Source:MGI Syr	0.7528
ENSMUSG00000038544	Inip	INTS3 and NABP interacting protein [Source:MGI Sy	0.75336
ENSMUSG00000097560	Gm26904	predicted gene, 26904 [Source:MGI Symbol;Acc:MG	0.75336
ENSMUSG00000039942	Ptger4	prostaglandin E receptor 4 (subtype EP4) [Source:N	0.75342
ENSMUSG00000039050	Osbpl2	oxysterol binding protein-like 2 [Source:MGI Symbo	0.75345
ENSMUSG00000004096	Cwc15	CWC15 homolog (S. cerevisiae) [Source:MGI Symbo	0.75346
ENSMUSG00000056394	Lig1	ligase I, DNA, ATP-dependent [Source:MGI Symbol;	0.75348
ENSMUSG00000029415	Sdad1	SDA1 domain containing 1 [Source:MGI Symbol;Acc	0.75349
ENSMUSG00000071506	Tmem139	transmembrane protein 139 [Source:MGI Symbol;A	0.75352
ENSMUSG00000058656	Samd12	sterile alpha motif domain containing 12 [Source:M	0.75359
ENSMUSG00000097474	Gm26584	predicted gene, 26584 [Source:MGI Symbol;Acc:MG	0.7538
ENSMUSG00000041594	Tmtc4	transmembrane and tetratricopeptide repeat conta	0.75381
ENSMUSG00000087627	A230059L01Rik	RIKEN cDNA A230059L01 gene [Source:MGI Symbo	0.75394
ENSMUSG00000066043	Phactr4	phosphatase and actin regulator 4 [Source:MGI Syr	0.75406
ENSMUSG00000063704	Mapk15	mitogen-activated protein kinase 15 [Source:MGI S	0.75418
ENSMUSG00000055653	Gpc3	glypican 3 [Source:MGI Symbol;Acc:MGI:104903]	0.75433
ENSMUSG00000015837	Sqstm1	sequestosome 1 [Source:MGI Symbol;Acc:MGI:107	0.7544
ENSMUSG00000001444	Tbx21	T-box 21 [Source:MGI Symbol;Acc:MGI:1888984]	0.75444
ENSMUSG00000021277	Traf3	TNF receptor-associated factor 3 [Source:MGI Symb	0.75444
ENSMUSG00000020256	Aldh1l2	aldehyde dehydrogenase 1 family, member L2 [Sou	0.75448
ENSMUSG00000004530	Coro1c	coronin, actin binding protein 1C [Source:MGI Symb	0.75481
ENSMUSG00000025650	Col7a1	collagen, type VII, alpha 1 [Source:MGI Symbol;Acc	0.7549
ENSMUSG00000019826	Zbtb24	zinc finger and BTB domain containing 24 [Source:N	0.75493
ENSMUSG00000067274	Rplp0	ribosomal protein, large, P0 [Source:MGI Symbol;A	0.75495
ENSMUSG00000017781	Pitpna	phosphatidylinositol transfer protein, alpha [Source	0.75543
ENSMUSG00000027470	Mylk2	myosin, light polypeptide kinase 2, skeletal muscle	0.75546
ENSMUSG00000048794	Ccdc37	coiled-coil domain containing 37 [Source:MGI Symb	0.75547

ENSMUSG00000031495	Cd209d	CD209d antigen [Source:MGI Symbol;Acc:MGI:215	0.75555
ENSMUSG00000051279	Gdf6	growth differentiation factor 6 [Source:MGI Symbo	0.7556
ENSMUSG00000074754	Gm561	predicted gene 561 [Source:MGI Symbol;Acc:MGI:2	0.75565
ENSMUSG00000086231	4732447D17Rik	RIKEN cDNA 4732447D17 gene [Source:MGI Symbo	0.75569
ENSMUSG00000042854	Trp53rk	transformation related protein 53 regulating kinase	0.75572
ENSMUSG00000034768	Asb16	ankyrin repeat and SOCS box-containing 16 [Source	0.75589
ENSMUSG00000032902	Slc16a1	solute carrier family 16 (monocarboxylic acid trans	0.75602
ENSMUSG00000031479	Vps36	vacuolar protein sorting 36 (yeast) [Source:MGI Sy	0.75623
ENSMUSG00000089993	Gm5822	predicted gene 5822 [Source:MGI Symbol;Acc:MGI	0.75637
ENSMUSG00000092172	Gm20523	predicted gene 20523 [Source:MGI Symbol;Acc:MG	0.7564
ENSMUSG00000093668	Pou5f2	POU domain class 5, transcription factor 2 [Source:	0.75652
ENSMUSG00000087350	Gm16254	predicted gene 16254 [Source:MGI Symbol;Acc:MG	0.75661
ENSMUSG00000087026	A230103J11Rik	RIKEN cDNA A230103J11 gene [Source:MGI Symbo	0.75664
ENSMUSG00000097220	Gm26599	predicted gene, 26599 [Source:MGI Symbol;Acc:M	0.75673
ENSMUSG00000004996	Mri1	methylthioribose-1-phosphate isomerase homolog	0.75679
ENSMUSG00000041769	Ppp2r2d	protein phosphatase 2, regulatory subunit B, delta	0.7568
ENSMUSG00000037031	Tspan15	tetraspanin 15 [Source:MGI Symbol;Acc:MGI:1917	0.75687
ENSMUSG00000065507	Mir204	microRNA 204 [Source:MGI Symbol;Acc:MGI:2676	0.75697
ENSMUSG00000081490	Gm11830	predicted gene 11830 [Source:MGI Symbol;Acc:MG	0.75706
ENSMUSG00000086382	Gm13705	predicted gene 13705 [Source:MGI Symbol;Acc:MG	0.75706
ENSMUSG00000018666	Cbx1	chromobox 1 [Source:MGI Symbol;Acc:MGI:10536	0.7573
ENSMUSG00000025213	Kazald1	Kazal-type serine peptidase inhibitor domain 1 [Sou	0.7574
ENSMUSG00000003119	Cdk12	cyclin-dependent kinase 12 [Source:MGI Symbol;A	0.75756
ENSMUSG00000028709	Mob3c	MOB kinase activator 3C [Source:MGI Symbol;Acc:	0.75761
ENSMUSG00000081041	Nlk-ps1	nemo like kinase, pseudogene 1 [Source:MGI Symb	0.75772
ENSMUSG00000022422	Dscc1	defective in sister chromatid cohesion 1 homolog (	0.75785
ENSMUSG00000005220	Corin	corin [Source:MGI Symbol;Acc:MGI:1349451]	0.75798
ENSMUSG00000069825	Spata22	spermatogenesis associated 22 [Source:MGI Symbo	0.75798
ENSMUSG00000031958	Ldhd	lactate dehydrogenase D [Source:MGI Symbol;Acc:	0.75807
ENSMUSG00000014550	Zfyve20	zinc finger, FYVE domain containing 20 [Source:MG	0.75824
ENSMUSG00000024371	C2	complement component 2 (within H-2S) [Source:M	0.75832
ENSMUSG00000036721	Zscan12	zinc finger and SCAN domain containing 12 [Source	0.75832
ENSMUSG00000066640	Fbxl18	F-box and leucine-rich repeat protein 18 [Source:M	0.75843
ENSMUSG00000086805	4932443L11Rik	RIKEN cDNA 4932443L11 gene [Source:MGI Symbo	0.7585
ENSMUSG00000005357	Slc1a6	solute carrier family 1 (high affinity aspartate/gluta	0.75852
ENSMUSG00000043155	Hpdl	4-hydroxyphenylpyruvate dioxygenase-like [Source	0.75871
ENSMUSG00000089633	A230009B12Rik	RIKEN cDNA A230009B12 gene [Source:MGI Symbo	0.75873
ENSMUSG00000034687	Fras1	Fraser syndrome 1 homolog (human) [Source:MGI	0.75875
ENSMUSG00000013418	B4galnt2	beta-1,4-N-acetyl-galactosaminyl transferase 2 [Sou	0.75877
ENSMUSG00000035064	Eef2k	eukaryotic elongation factor-2 kinase [Source:MGI	0.75884
ENSMUSG00000016495	Plgrkt	plasminogen receptor, C-terminal lysine transmem	0.75886
ENSMUSG00000095562	Gm21887	predicted gene, 21887 [Source:MGI Symbol;Acc:M	0.7589
ENSMUSG00000001128	Cfp	complement factor properdin [Source:MGI Symbol;	0.75903
ENSMUSG00000004815	Dgkq	diacylglycerol kinase, theta [Source:MGI Symbol;A	0.75904
ENSMUSG00000085439	Gm13634	predicted gene 13634 [Source:MGI Symbol;Acc:MG	0.75905
ENSMUSG00000035835	BC005764	cDNA sequence BC005764 [Source:MGI Symbol;Ac	0.75914
ENSMUSG00000021715	Cwc27	CWC27 spliceosome-associated protein homolog (	0.75947

ENSMUSG00000059119	Nap1l4	nucleosome assembly protein 1-like 4 [Source:MGI	0.75947
ENSMUSG00000007097	Atp1a2	ATPase, Na+/K+ transporting, alpha 2 polypeptide [	0.75954
ENSMUSG00000056313	1810011O10Rik	RIKEN cDNA 1810011O10 gene [Source:MGI Symbo	0.75955
ENSMUSG00000097320	C630016N16Rik	RIKEN cDNA C630016N16 gene [Source:MGI Symbo	0.75966
ENSMUSG00000038233	Fam198a	family with sequence similarity 198, member A [So	0.75975
ENSMUSG00000061950	Ppp4r1	protein phosphatase 4, regulatory subunit 1 [Sourc	0.7598
ENSMUSG00000046573	Lyrm4	LYR motif containing 4 [Source:MGI Symbol;Acc:MG	0.75989
ENSMUSG00000071793	2610005L07Rik	RIKEN cDNA 2610005L07 gene [Source:MGI Symbo	0.75989
ENSMUSG00000027889	Ampd2	adenosine monophosphate deaminase 2 [Source:M	0.75998
ENSMUSG00000007440	Pcdha2	protocadherin alpha 2 [Source:MGI Symbol;Acc:MG	0.76001
ENSMUSG00000059674	Cdh24	cadherin-like 24 [Source:MGI Symbol;Acc:MGI:1928	0.76016
ENSMUSG00000052566	Hook2	hook homolog 2 (Drosophila) [Source:MGI Symbol;A	0.76026
ENSMUSG00000086212	Mkln1os	Mkln1 opposite strand transcript [Source:MGI Sym	0.76037
ENSMUSG00000034354	Mtmr3	myotubularin related protein 3 [Source:MGI Symbo	0.76038
ENSMUSG00000094281	Gm8034	predicted gene 8034 [Source:MGI Symbol;Acc:MGI:	0.76051
ENSMUSG00000097086	Gm7672	predicted gene 7672 [Source:MGI Symbol;Acc:MGI:	0.7607
ENSMUSG00000083009	Gm1335	predicted gene 1335 [Source:MGI Symbol;Acc:MGI:	0.76071
ENSMUSG00000053654	Krt42	keratin 42 [Source:MGI Symbol;Acc:MGI:1915489]	0.76075
ENSMUSG00000031310	Zmym3	zinc finger, MYM-type 3 [Source:MGI Symbol;Acc:M	0.7609
ENSMUSG00000020284	1810043G02Rik	RIKEN cDNA 1810043G02 gene [Source:MGI Symbo	0.76097
ENSMUSG00000025421	Hdhd2	haloacid dehalogenase-like hydrolase domain conta	0.76102
ENSMUSG00000086589	Gm12915	predicted gene 12915 [Source:MGI Symbol;Acc:MG	0.76103
ENSMUSG00000001053	N4bp3	NEDD4 binding protein 3 [Source:MGI Symbol;Acc:	0.76104
ENSMUSG00000040767	Snrnp25	small nuclear ribonucleoprotein 25 (U11/U12) [Sou	0.76111
ENSMUSG00000094952	Mir3070b	microRNA 3070b [Source:MGI Symbol;Acc:MGI:483	0.76124
ENSMUSG00000027775	Mfsd1	major facilitator superfamily domain containing 1 [	0.76125
ENSMUSG00000091055	Siglec15	sialic acid binding Ig-like lectin 15 [Source:MGI Sym	0.76125
ENSMUSG00000093476	Gm22355	predicted gene, 22355 [Source:MGI Symbol;Acc:MG	0.76125
ENSMUSG00000084374	Gm13255	predicted gene 13255 [Source:MGI Symbol;Acc:MG	0.76127
ENSMUSG00000038132	Rbm24	RNA binding motif protein 24 [Source:MGI Symbol;	0.76129
ENSMUSG00000097995	Gm24362	predicted gene, 24362 [Source:MGI Symbol;Acc:MG	0.76131
ENSMUSG00000097661	Gm26763	predicted gene, 26763 [Source:MGI Symbol;Acc:MG	0.76132
ENSMUSG00000082693	Gm15190	predicted gene 15190 [Source:MGI Symbol;Acc:MG	0.76134
ENSMUSG00000086053	Gm15178	predicted gene 15178 [Source:MGI Symbol;Acc:MG	0.76134
ENSMUSG00000028262	Clca2	chloride channel calcium activated 2 [Source:MGI S	0.76135
ENSMUSG00000046620	Rps11-ps4	ribosomal protein S11, pseudogene 4 [Source:MGI	0.76135
ENSMUSG00000029389	Ddx55	DEAD (Asp-Glu-Ala-Asp) box polypeptide 55 [Source	0.76139
ENSMUSG00000032563	Mrpl3	mitochondrial ribosomal protein L3 [Source:MGI Sy	0.76148
ENSMUSG00000033628	Pik3c3	phosphoinositide-3-kinase, class 3 [Source:MGI Syn	0.76152
ENSMUSG00000003923	Tfam	transcription factor A, mitochondrial [Source:MGI S	0.76166
ENSMUSG00000029004	Kmt2e	lysine (K)-specific methyltransferase 2E [Source:MG	0.76166
ENSMUSG00000074151	Nlrc5	NLR family, CARD domain containing 5 [Source:MG	0.76168
ENSMUSG00000058318	Phf21a	PHD finger protein 21A [Source:MGI Symbol;Acc:M	0.76176
ENSMUSG00000007039	Ddah2	dimethylarginine dimethylaminohydrolase 2 [Sourc	0.76191
ENSMUSG00000031105	Slc25a14	solute carrier family 25 (mitochondrial carrier, brai	0.76191
ENSMUSG00000027584	Oprl1	opioid receptor-like 1 [Source:MGI Symbol;Acc:MG	0.76193
ENSMUSG00000036073	Galt	galactose-1-phosphate uridyl transferase [Source:M	0.76196

ENSMUSG00000026946	Nmi	N-myc (and STAT) interactor [Source:MGI Symbol;A	0.76197
ENSMUSG00000031119	Gpc4	glypican 4 [Source:MGI Symbol;Acc:MGI:104902]	0.76208
ENSMUSG00000026754	Golga1	golgi autoantigen, golgin subfamily a, 1 [Source:MG	0.76209
ENSMUSG00000026000	Lancl1	LanC (bacterial lantibiotic synthetase component C	0.76213
ENSMUSG00000082362	Gm5187	predicted gene 5187 [Source:MGI Symbol;Acc:MGI:	0.76215
ENSMUSG00000096140	Ankrd66	ankyrin repeat domain 66 [Source:MGI Symbol;Acc	0.76224
ENSMUSG00000026279	Thap4	THAP domain containing 4 [Source:MGI Symbol;Acc	0.76232
ENSMUSG00000049858	Suox	sulfite oxidase [Source:MGI Symbol;Acc:MGI:24461	0.76247
ENSMUSG00000030555	Ttc23	tetratricopeptide repeat domain 23 [Source:MGI Sy	0.76249
ENSMUSG00000068758	Il3ra	interleukin 3 receptor, alpha chain [Source:MGI Syr	0.76256
ENSMUSG00000027881	Prpf38b	PRP38 pre-mRNA processing factor 38 (yeast) doma	0.76259
ENSMUSG00000016262	Sertad4	SERTA domain containing 4 [Source:MGI Symbol;Ac	0.76261
ENSMUSG00000037627	Rgs22	regulator of G-protein signalling 22 [Source:MGI Sy	0.76264
ENSMUSG00000086580	Gm15280	predicted gene 15280 [Source:MGI Symbol;Acc:MG	0.76265
ENSMUSG00000024734	Zp1	zona pellucida glycoprotein 1 [Source:MGI Symbol;	0.7627
ENSMUSG00000029182	1700001C02Rik	RIKEN cDNA 1700001C02 gene [Source:MGI Symbo	0.7627
ENSMUSG00000031659	Adcy7	adenylate cyclase 7 [Source:MGI Symbol;Acc:MGI:1	0.7627
ENSMUSG00000031390	Avpr2	arginine vasopressin receptor 2 [Source:MGI Symbo	0.76271
ENSMUSG00000077614	Gm25500	predicted gene, 25500 [Source:MGI Symbol;Acc:MG	0.76271
ENSMUSG00000040329	Il7	interleukin 7 [Source:MGI Symbol;Acc:MGI:96561]	0.76272
ENSMUSG00000025723	Nmb	neuromedin B [Source:MGI Symbol;Acc:MGI:19152	0.76277
ENSMUSG00000043223	Gm4835	predicted pseudogene 4835 [Source:MGI Symbol;A	0.76289
ENSMUSG00000081751	Gm14251	predicted gene 14251 [Source:MGI Symbol;Acc:MG	0.76289
ENSMUSG00000094800	Gm9780	predicted gene 9780 [Source:MGI Symbol;Acc:MGI:	0.76294
ENSMUSG00000096810	Gm10481	predicted gene 10481 [Source:MGI Symbol;Acc:MG	0.76294
ENSMUSG00000092201	A530058N18Rik	RIKEN cDNA A530058N18 gene [Source:MGI Symbo	0.76295
ENSMUSG00000061356	Nuggc	nuclear GTPase, germinal center associated [Source	0.76309
ENSMUSG00000092261	Gm20519	predicted gene 20519 [Source:MGI Symbol;Acc:MG	0.76309
ENSMUSG00000086529	4930471I20Rik	RIKEN cDNA 4930471I20 gene [Source:MGI Symbol	0.7631
ENSMUSG00000096969	4930535I16Rik	RIKEN cDNA 4930535I16 gene [Source:MGI Symbol	0.76312
ENSMUSG00000024270	Slc39a6	solute carrier family 39 (metal ion transporter), me	0.7632
ENSMUSG00000079471	Gm7325	predicted gene 7325 [Source:MGI Symbol;Acc:MGI:	0.76329
ENSMUSG00000097900	Gm8652	predicted gene 8652 [Source:MGI Symbol;Acc:MGI:	0.76329
ENSMUSG00000035805	Mlc1	megalencephalic leukoencephalopathy with subcor	0.76331
ENSMUSG00000085755	Gm13496	predicted gene 13496 [Source:MGI Symbol;Acc:MG	0.76332
ENSMUSG00000086358	Gm13270	predicted gene 13270 [Source:MGI Symbol;Acc:MG	0.76332
ENSMUSG00000042200	Cdrt4	CMT1A duplicated region transcript 4 [Source:MGI	0.76333
ENSMUSG00000043681	Fam25c	family with sequence similarity 25, member C [Sour	0.76333
ENSMUSG00000082002	Gm6568	predicted pseudogene 6568 [Source:MGI Symbol;A	0.76334
ENSMUSG00000084504	Gm25323	predicted gene, 25323 [Source:MGI Symbol;Acc:MG	0.76334
ENSMUSG00000089781	Gm15756	predicted gene 15756 [Source:MGI Symbol;Acc:MG	0.76334
ENSMUSG00000042888	1700110M21Rik	RIKEN cDNA 1700110M21 gene [Source:MGI Symbo	0.76335
ENSMUSG00000097028	7530420F21Rik	RIKEN cDNA 7530420F21 gene [Source:MGI Symbo	0.76342
ENSMUSG00000037716	Ccdc33	coiled-coil domain containing 33 [Source:MGI Symb	0.76343
ENSMUSG00000039086	Ss18l1	synovial sarcoma translocation gene on chromosom	0.76346
ENSMUSG00000030222	Rerg	RAS-like, estrogen-regulated, growth-inhibitor [Sou	0.76359
ENSMUSG00000030287	Itpr2	inositol 1,4,5-triphosphate receptor 2 [Source:MGI	0.76375

ENSMUSG00000060036	Rpl3	ribosomal protein L3 [Source:MGI Symbol;Acc:MGI	0.76375
ENSMUSG00000097101	1810034E14Rik	RIKEN cDNA 1810034E14 gene [Source:MGI Symbo	0.7638
ENSMUSG00000001942	Siae	sialic acid acetylesterase [Source:MGI Symbol;Acc:M	0.76386
ENSMUSG00000082144	Gm12788	predicted gene 12788 [Source:MGI Symbol;Acc:MG	0.764
ENSMUSG00000060879	Gm6564	predicted pseudogene 6564 [Source:MGI Symbol;A	0.76402
ENSMUSG00000086167	Gm13827	predicted gene 13827 [Source:MGI Symbol;Acc:MG	0.76403
ENSMUSG00000087111	Gm11399	predicted gene 11399 [Source:MGI Symbol;Acc:MG	0.76412
ENSMUSG00000073435	Nme3	NME/NM23 nucleoside diphosphate kinase 3 [Sour	0.76414
ENSMUSG00000067870	Rpl31-ps8	ribosomal protein L31, pseudogene 8 [Source:MGI	0.76416
ENSMUSG00000089883	Gm3355	predicted gene 3355 [Source:MGI Symbol;Acc:MGI	0.76418
ENSMUSG00000062542	Syt9	synaptotagmin IX [Source:MGI Symbol;Acc:MGI:19	0.76432
ENSMUSG00000038459	Abhd17c	abhydrolase domain containing 17C [Source:MGI S	0.76434
ENSMUSG00000034820	Cpsf7	cleavage and polyadenylation specific factor 7 [Sou	0.7644
ENSMUSG00000021967	Mrp63	mitochondrial ribosomal protein 63 [Source:MGI S	0.76464
ENSMUSG00000052395	Rft1	RFT1 homolog (S. cerevisiae) [Source:MGI Symbol;	0.76471
ENSMUSG00000022948	Setd4	SET domain containing 4 [Source:MGI Symbol;Acc:M	0.76475
ENSMUSG00000046962	Zbtb21	zinc finger and BTB domain containing 21 [Source:M	0.76483
ENSMUSG00000026341	Actr3	ARP3 actin-related protein 3 [Source:MGI Symbol;A	0.76484
ENSMUSG00000020923	Ubtf	upstream binding transcription factor, RNA polyme	0.76492
ENSMUSG00000027883	Gpsm2	G-protein signalling modulator 2 (AGS3-like, C. eleg	0.76502
ENSMUSG00000070691	Runx3	runt related transcription factor 3 [Source:MGI Sym	0.76503
ENSMUSG00000021018	Polr2h	polymerase (RNA) II (DNA directed) polypeptide H [	0.76508
ENSMUSG00000081378	Rps13-ps4	ribosomal protein S13, pseudogene 4 [Source:MGI	0.76509
ENSMUSG00000050014	Apol10b	apolipoprotein L 10B [Source:MGI Symbol;Acc:MGI	0.76511
ENSMUSG00000026966	Ssna1	Sjogren's syndrome nuclear autoantigen 1 [Source:	0.7652
ENSMUSG00000065145	Vaultrc5	vault RNA component 5 [Source:MGI Symbol;Acc:M	0.76524
ENSMUSG00000084177	Gm13508	predicted gene 13508 [Source:MGI Symbol;Acc:MG	0.76527
ENSMUSG00000086275	1700121C08Rik	RIKEN cDNA 1700121C08 gene [Source:MGI Symbo	0.76527
ENSMUSG00000089945	Gm20459	predicted gene 20459 [Source:MGI Symbol;Acc:MG	0.76529
ENSMUSG00000087028	Gm13387	predicted gene 13387 [Source:MGI Symbol;Acc:MG	0.76532
ENSMUSG00000039908	Slc26a11	solute carrier family 26, member 11 [Source:MGI S	0.76537
ENSMUSG00000027274	Mkks	McKusick-Kaufman syndrome [Source:MGI Symbol;	0.76541
ENSMUSG00000064814	Gm23939	predicted gene, 23939 [Source:MGI Symbol;Acc:M	0.76546
ENSMUSG00000025081	Tdrd1	tudor domain containing 1 [Source:MGI Symbol;Ac	0.76547
ENSMUSG00000025075	Habp2	hyaluronic acid binding protein 2 [Source:MGI Sym	0.76548
ENSMUSG00000049107	Ntf3	neurotrophin 3 [Source:MGI Symbol;Acc:MGI:9738	0.76549
ENSMUSG00000057594	Arl16	ADP-ribosylation factor-like 16 [Source:MGI Symbo	0.76561
ENSMUSG00000000916	Nsun5	NOL1/NOP2/Sun domain family, member 5 [Source	0.76562
ENSMUSG00000031636	Pdlim3	PDZ and LIM domain 3 [Source:MGI Symbol;Acc:M	0.76562
ENSMUSG00000031736	4933436C20Rik	RIKEN cDNA 4933436C20 gene [Source:MGI Symbo	0.76564
ENSMUSG00000061315	Naca	nascent polypeptide-associated complex alpha poly	0.76585
ENSMUSG00000050321	Neto1	neuropilin (NRP) and tolloid (TLL)-like 1 [Source:MG	0.76588
ENSMUSG00000019845	Tube1	epsilon-tubulin 1 [Source:MGI Symbol;Acc:MGI:191	0.7659
ENSMUSG00000056598	Lrrc48	leucine rich repeat containing 48 [Source:MGI Sym	0.7659
ENSMUSG00000040811	Eml2	echinoderm microtubule associated protein like 2 [	0.76608
ENSMUSG00000089996	Tmsb15b2	thymosin beta 15b2 [Source:MGI Symbol;Acc:MGI:	0.76608
ENSMUSG00000036975	Tmem177	transmembrane protein 177 [Source:MGI Symbol;A	0.7662

ENSMUSG00000031487	Brf2	BRF2, subunit of RNA polymerase III transcription in	0.76628
ENSMUSG00000096740	Gm21743	predicted gene, 21743 [Source:MGI Symbol;Acc:MG	0.76632
ENSMUSG00000024747	Aldh1a7	aldehyde dehydrogenase family 1, subfamily A7 [So	0.76634
ENSMUSG00000058258	Idi1	isopentenyl-diphosphate delta isomerase [Source:M	0.76653
ENSMUSG00000029838	Ptn	pleiotrophin [Source:MGI Symbol;Acc:MGI:97804]	0.76654
ENSMUSG00000098112	Bin2	bridging integrator 2 [Source:MGI Symbol;Acc:MGI:	0.76657
ENSMUSG00000067370	B3galt4	UDP-Gal:betaGlcNAc beta 1,3-galactosyltransferase	0.76659
ENSMUSG00000018581	Dnah11	dynein, axonemal, heavy chain 11 [Source:MGI Sym	0.7666
ENSMUSG00000077524	n-R5s67	nuclear encoded rRNA 5S 67 [Source:MGI Symbol;A	0.76662
ENSMUSG00000083344	Gm7363	predicted gene 7363 [Source:MGI Symbol;Acc:MGI:	0.76662
ENSMUSG00000086297	Gm15632	predicted gene 15632 [Source:MGI Symbol;Acc:MG	0.76662
ENSMUSG00000016940	Kctd2	potassium channel tetramerisation domain contain	0.76667
ENSMUSG00000085908	Gm14209	predicted gene 14209 [Source:MGI Symbol;Acc:MG	0.76678
ENSMUSG00000083266	Gm12572	predicted gene 12572 [Source:MGI Symbol;Acc:MG	0.7668
ENSMUSG00000090282	Gm17688	predicted gene, 17688 [Source:MGI Symbol;Acc:MG	0.7668
ENSMUSG00000079146	Gm15353	predicted gene 15353 [Source:MGI Symbol;Acc:MG	0.76681
ENSMUSG00000082072	Gm15785	predicted gene 15785 [Source:MGI Symbol;Acc:MG	0.76681
ENSMUSG00000075480	Gm10840	predicted gene 10840 [Source:MGI Symbol;Acc:MG	0.76682
ENSMUSG00000064837	Snora75	small nucleolar RNA, H/ACA box 75 [Source:MGI Sy	0.76683
ENSMUSG00000064759	Gm25475	predicted gene, 25475 [Source:MGI Symbol;Acc:MG	0.76684
ENSMUSG00000056366	Fabp3-ps1	fatty acid binding protein 3, muscle and heart, pseu	0.76687
ENSMUSG00000088836	Gm23546	predicted gene, 23546 [Source:MGI Symbol;Acc:MG	0.76688
ENSMUSG00000079069	8430423G03Rik	RIKEN cDNA 8430423G03 gene [Source:MGI Symbo	0.76689
ENSMUSG00000064595	Gm22300	predicted gene, 22300 [Source:MGI Symbol;Acc:MG	0.7669
ENSMUSG00000026200	Glb1l	galactosidase, beta 1-like [Source:MGI Symbol;Acc:	0.76704
ENSMUSG00000033658	Ddx19b	DEAD (Asp-Glu-Ala-Asp) box polypeptide 19b [Sour	0.76707
ENSMUSG00000087269	D330023K18Rik	RIKEN cDNA D330023K18 gene [Source:MGI Symbo	0.76719
ENSMUSG00000093880	Tmem181c-ps	transmembrane protein 181C, pseudogene [Source	0.76726
ENSMUSG00000001666	Ddt	D-dopachrome tautomerase [Source:MGI Symbol;A	0.76733
ENSMUSG00000045757	Zfp764	zinc finger protein 764 [Source:MGI Symbol;Acc:MG	0.76735
ENSMUSG00000091803	Cox16	cytochrome c oxidase assembly protein 16 [Source:	0.7674
ENSMUSG00000026737	Pip4k2a	phosphatidylinositol-5-phosphate 4-kinase, type II,	0.76748
ENSMUSG00000064202	4430402I18Rik	RIKEN cDNA 4430402I18 gene [Source:MGI Symbol	0.76766
ENSMUSG00000063410	Stk24	serine/threonine kinase 24 [Source:MGI Symbol;Ac	0.76775
ENSMUSG00000021774	Ube2e1	ubiquitin-conjugating enzyme E2E 1 [Source:MGI Sy	0.76791
ENSMUSG00000017428	Psmd11	proteasome (prosome, macropain) 26S subunit, no	0.76799
ENSMUSG00000054409	Tmem74	transmembrane protein 74 [Source:MGI Symbol;Ac	0.76804
ENSMUSG00000029279	Brdt	bromodomain, testis-specific [Source:MGI Symbol;A	0.76805
ENSMUSG00000029804	Herc3	hect domain and RLD 3 [Source:MGI Symbol;Acc:M	0.76805
ENSMUSG00000044921	Rassf9	Ras association (RalGDS/AF-6) domain family (N-ter	0.76813
ENSMUSG00000021756	Il6st	interleukin 6 signal transducer [Source:MGI Symbol	0.76826
ENSMUSG00000090862	Rps13	ribosomal protein S13 [Source:MGI Symbol;Acc:MG	0.76832
ENSMUSG00000038068	Rnf144b	ring finger protein 144B [Source:MGI Symbol;Acc:M	0.76848
ENSMUSG00000014551	Mrps25	mitochondrial ribosomal protein S25 [Source:MGI S	0.76852
ENSMUSG00000086938	4930481A15Rik	RIKEN cDNA 4930481A15 gene [Source:MGI Symbo	0.76852
ENSMUSG00000021959	Lats2	large tumor suppressor 2 [Source:MGI Symbol;Acc:	0.76855
ENSMUSG00000094840	A630081J09Rik	RIKEN cDNA A630081J09 gene [Source:MGI Symbo	0.76857

ENSMUSG00000072620	Slfn2	schlafen 2 [Source:MGI Symbol;Acc:MGI:1313258]	0.76868
ENSMUSG00000045438	Cox19	cytochrome c oxidase assembly protein 19 [Source:	0.76869
ENSMUSG00000029725	Ppp1r35	protein phosphatase 1, regulatory subunit 35 [Sour	0.76874
ENSMUSG00000097165	2210008F06Rik	RIKEN cDNA 2210008F06 gene [Source:MGI Symbo	0.76877
ENSMUSG00000045929	Gm20715	predicted gene 20715 [Source:MGI Symbol;Acc:MG	0.76883
ENSMUSG00000060424	2610017I09Rik	RIKEN cDNA 2610017I09 gene [Source:MGI Symbol	0.76903
ENSMUSG00000019843	Fyn	Fyn proto-oncogene [Source:MGI Symbol;Acc:MGI:	0.76908
ENSMUSG00000020812	1810032O08Rik	RIKEN cDNA 1810032O08 gene [Source:MGI Symbo	0.76917
ENSMUSG00000024271	Elp2	elongator acetyltransferase complex subunit 2 [Sou	0.76918
ENSMUSG00000039168	Dap	death-associated protein [Source:MGI Symbol;Acc:	0.76928
ENSMUSG00000000088	Cox5a	cytochrome c oxidase subunit Va [Source:MGI Sym	0.76942
ENSMUSG00000074469	Gm15348	predicted gene 15348 [Source:MGI Symbol;Acc:MG	0.76949
ENSMUSG00000029924	Slc37a3	solute carrier family 37 (glycerol-3-phosphate trans	0.76959
ENSMUSG00000085033	Gm11646	predicted gene 11646 [Source:MGI Symbol;Acc:MG	0.76969
ENSMUSG00000020629	Adi1	acireductone dioxygenase 1 [Source:MGI Symbol;A	0.76984
ENSMUSG00000005373	Mlxipl	MLX interacting protein-like [Source:MGI Symbol;A	0.76993
ENSMUSG00000032481	Smarcc1	SWI/SNF related, matrix associated, actin depende	0.77
ENSMUSG00000090012	Gm16555	predicted gene 16555 [Source:MGI Symbol;Acc:MG	0.77008
ENSMUSG00000035759	Bbs10	Bardet-Biedl syndrome 10 (human) [Source:MGI Sy	0.77009
ENSMUSG00000032443	Zcwpw2	zinc finger, CW type with PWWP domain 2 [Source:	0.77017
ENSMUSG00000057967	Fgf18	fibroblast growth factor 18 [Source:MGI Symbol;Ac	0.77026
ENSMUSG00000091002	Tcerg1l	transcription elongation regulator 1-like [Source:M	0.77035
ENSMUSG00000021087	Rtn1	reticulon 1 [Source:MGI Symbol;Acc:MGI:1933947]	0.7704
ENSMUSG00000029426	Scarb2	scavenger receptor class B, member 2 [Source:MGI	0.77042
ENSMUSG00000061079	Zfp143	zinc finger protein 143 [Source:MGI Symbol;Acc:M	0.77051
ENSMUSG00000068407	Rnase12	ribonuclease, RNase A family, 12 (non-active) [Sour	0.77071
ENSMUSG00000071633	Gm4952	predicted gene 4952 [Source:MGI Symbol;Acc:MGI:	0.77073
ENSMUSG00000048429	1810026J23Rik	RIKEN cDNA 1810026J23 gene [Source:MGI Symbol	0.77078
ENSMUSG00000079316	Rab9	RAB9, member RAS oncogene family [Source:MGI S	0.77084
ENSMUSG00000040177	2310057M21Rik	RIKEN cDNA 2310057M21 gene [Source:MGI Symbo	0.77099
ENSMUSG00000079045	Gm17566	predicted gene, 17566 [Source:MGI Symbol;Acc:M	0.77099
ENSMUSG00000083429	Gm15198	predicted gene 15198 [Source:MGI Symbol;Acc:MG	0.77105
ENSMUSG00000001508	Sgca	sarcoglycan, alpha (dystrophin-associated glycopro	0.77106
ENSMUSG00000093751	Gm3307	predicted gene 3307 [Source:MGI Symbol;Acc:MGI:	0.77106
ENSMUSG00000080712	Hist3h2bb-ps	histone cluster 3, H2bb, pseudogene [Source:MGI S	0.7711
ENSMUSG00000074220	Zfp382	zinc finger protein 382 [Source:MGI Symbol;Acc:M	0.77113
ENSMUSG00000089853	Gm15853	predicted gene 15853 [Source:MGI Symbol;Acc:MG	0.77122
ENSMUSG00000038331	Satb2	special AT-rich sequence binding protein 2 [Source:	0.77125
ENSMUSG00000062312	Erbb2	v-erb-b2 erythroblastic leukemia viral oncogene ho	0.77127
ENSMUSG00000010660	Plcd1	phospholipase C, delta 1 [Source:MGI Symbol;Acc:M	0.77155
ENSMUSG00000078584	AU022252	expressed sequence AU022252 [Source:MGI Symbo	0.77159
ENSMUSG00000021772	Nkiras1	NFKB inhibitor interacting Ras-like protein 1 [Sourc	0.77167
ENSMUSG00000061559	Wdr61	WD repeat domain 61 [Source:MGI Symbol;Acc:MG	0.77182
ENSMUSG00000026840	Lamc3	laminin gamma 3 [Source:MGI Symbol;Acc:MGI:134	0.7719
ENSMUSG00000046447	Camk2n1	calcium/calmodulin-dependent protein kinase II inh	0.7719
ENSMUSG00000093589	Gm18367	predicted gene, 18367 [Source:MGI Symbol;Acc:M	0.77194
ENSMUSG00000060288	Ppih	peptidyl prolyl isomerase H [Source:MGI Symbol;Ac	0.77195

ENSMUSG00000051557	Pusl1	pseudouridylate synthase-like 1 [Source:MGI Symb	0.77211
ENSMUSG00000086657	4921530L18Rik	RIKEN cDNA 4921530L18 gene [Source:MGI Symbo	0.77212
ENSMUSG00000061882	Ccdc62	coiled-coil domain containing 62 [Source:MGI Symb	0.7722
ENSMUSG00000044636	Csrnp2	cysteine-serine-rich nuclear protein 2 [Source:MGI	0.77222
ENSMUSG00000036086	Zranb3	zinc finger, RAN-binding domain containing 3 [Sour	0.77224
ENSMUSG00000028603	Scp2	sterol carrier protein 2, liver [Source:MGI Symbol;A	0.77234
ENSMUSG00000005204	Senp3	SUMO/sentrin specific peptidase 3 [Source:MGI Sy	0.77245
ENSMUSG00000086207	Gm14717	predicted gene 14717 [Source:MGI Symbol;Acc:MG	0.77246
ENSMUSG00000093245	Mir677	microRNA 677 [Source:MGI Symbol;Acc:MGI:36298	0.77256
ENSMUSG00000092564	BC051226	cDNA sequence BC051226 [Source:MGI Symbol;Acc	0.77274
ENSMUSG00000097837	Gm26794	predicted gene, 26794 [Source:MGI Symbol;Acc:M	0.77274
ENSMUSG00000091922	Gm17409	predicted gene, 17409 [Source:MGI Symbol;Acc:M	0.77284
ENSMUSG00000045802	Hsf3	heat shock transcription factor 3 [Source:MGI Symb	0.77286
ENSMUSG00000059554	Ccdc28a	coiled-coil domain containing 28A [Source:MGI Sym	0.77298
ENSMUSG00000032401	Lctl	lactase-like [Source:MGI Symbol;Acc:MGI:2183549]	0.773
ENSMUSG00000086199	8430403D17Rik	RIKEN cDNA 8430403D17 gene [Source:MGI Symbo	0.77304
ENSMUSG00000022526	Zfp251	zinc finger protein 251 [Source:MGI Symbol;Acc:M	0.77309
ENSMUSG00000091271	Gm17104	predicted gene 17104 [Source:MGI Symbol;Acc:MG	0.77312
ENSMUSG00000020737	Hn1	hematological and neurological expressed sequenc	0.77313
ENSMUSG00000048699	4732456N10Rik	RIKEN cDNA 4732456N10 gene [Source:MGI Symbo	0.77313
ENSMUSG00000028873	Cdca8	cell division cycle associated 8 [Source:MGI Symbol	0.7732
ENSMUSG00000009563	Tor2a	torsin family 2, member A [Source:MGI Symbol;Acc	0.77328
ENSMUSG00000057234	Mettl15	methyltransferase like 15 [Source:MGI Symbol;Acc:	0.77337
ENSMUSG00000017499	Cdc6	cell division cycle 6 [Source:MGI Symbol;Acc:MGI:1	0.77355
ENSMUSG00000083161	Gm11427	predicted gene 11427 [Source:MGI Symbol;Acc:MG	0.77358
ENSMUSG00000089673	Gm16546	predicted gene 16546 [Source:MGI Symbol;Acc:MG	0.77358
ENSMUSG00000097301	AW121686	expressed sequence AW121686 [Source:MGI Symb	0.77363
ENSMUSG00000017734	Dbndd2	dysbindin (dystrobrevin binding protein 1) domain	0.77377
ENSMUSG00000044452	Zfp507	zinc finger protein 507 [Source:MGI Symbol;Acc:M	0.77377
ENSMUSG00000019210	Atp6v1e1	ATPase, H+ transporting, lysosomal V1 subunit E1 [	0.77385
ENSMUSG00000019789	Hey2	hairy/enhancer-of-split related with YRPW motif 2	0.77388
ENSMUSG00000063696	Gm8730	predicted pseudogene 8730 [Source:MGI Symbol;A	0.77392
ENSMUSG00000083442	Gm11187	predicted gene 11187 [Source:MGI Symbol;Acc:MG	0.77393
ENSMUSG00000053626	Tll1	tolloid-like [Source:MGI Symbol;Acc:MGI:106923]	0.77397
ENSMUSG00000020275	Rel	reticuloendotheliosis oncogene [Source:MGI Symbo	0.77403
ENSMUSG00000073437	D330041H03Rik	RIKEN cDNA D330041H03 gene [Source:MGI Symbo	0.77409
ENSMUSG00000063754	Gm10136	predicted pseudogene 10136 [Source:MGI Symbol;	0.77411
ENSMUSG00000069588	Gm11733	predicted gene 11733 [Source:MGI Symbol;Acc:MG	0.77419
ENSMUSG00000054545	Ugt1a6a	UDP glucuronosyltransferase 1 family, polypeptide	0.77434
ENSMUSG00000086315	4930555J06Rik	RIKEN cDNA 4930555J06 gene [Source:MGI Symbol	0.77434
ENSMUSG00000067543	Prb1	proline-rich protein BstNI subfamily 1 [Source:MGI	0.77439
ENSMUSG00000028989	Angptl7	angiopoietin-like 7 [Source:MGI Symbol;Acc:MGI:3	0.77447
ENSMUSG00000040866	Rsph6a	radial spoke head 6 homolog A (Chlamydomonas) [	0.77467
ENSMUSG00000055371	Stam2	signal transducing adaptor molecule (SH3 domain a	0.77478
ENSMUSG00000097381	A230087F16Rik	RIKEN cDNA A230087F16 gene [Source:MGI Symbo	0.7748
ENSMUSG00000022003	Slc25a30	solute carrier family 25, member 30 [Source:MGI S	0.77485
ENSMUSG00000063087	Gm10125	predicted gene 10125 [Source:MGI Symbol;Acc:MG	0.77486

ENSMUSG00000031557	Plekha2	pleckstrin homology domain-containing, family A (p	0.77487
ENSMUSG00000082900	Gm11557	predicted gene 11557 [Source:MGI Symbol;Acc:MG	0.77494
ENSMUSG00000034342	Cbl	Casitas B-lineage lymphoma [Source:MGI Symbol;A	0.775
ENSMUSG00000097703	Gm26623	predicted gene, 26623 [Source:MGI Symbol;Acc:MG	0.77504
ENSMUSG00000024154	Gtf2a1l	general transcription factor IIA, 1-like [Source:MGI	0.77528
ENSMUSG00000085133	B930095G15Rik	RIKEN cDNA B930095G15 gene [Source:MGI Symbo	0.77529
ENSMUSG00000026972	Arrdc1	arrestin domain containing 1 [Source:MGI Symbol;A	0.7753
ENSMUSG00000032501	Trib1	tribbles homolog 1 (Drosophila) [Source:MGI Symb	0.77539
ENSMUSG00000095468	Gm20379	predicted gene, 20379 [Source:MGI Symbol;Acc:MG	0.77541
ENSMUSG00000035575	Utp6	UTP6, small subunit (SSU) processome component,	0.77543
ENSMUSG00000081025	Gm11943	predicted gene 11943 [Source:MGI Symbol;Acc:MG	0.77543
ENSMUSG00000040209	Zfp704	zinc finger protein 704 [Source:MGI Symbol;Acc:MG	0.77544
ENSMUSG00000038022	Fam188b	family with sequence similarity 188, member B [Sou	0.77546
ENSMUSG00000097842	9330104G04Rik	RIKEN cDNA 9330104G04 gene [Source:MGI Symbo	0.77546
ENSMUSG00000030286	Emc3	ER membrane protein complex subunit 3 [Source:M	0.77551
ENSMUSG00000030522	Mtmr10	myotubularin related protein 10 [Source:MGI Symb	0.77561
ENSMUSG00000029343	Crybb1	crystallin, beta B1 [Source:MGI Symbol;Acc:MGI:10	0.77581
ENSMUSG00000004865	Srpk1	serine/arginine-rich protein specific kinase 1 [Sourc	0.77583
ENSMUSG00000047390	Defb9	defensin beta 9 [Source:MGI Symbol;Acc:MGI:2179	0.77588
ENSMUSG00000095068	Gm26189	predicted gene, 26189 [Source:MGI Symbol;Acc:MG	0.77591
ENSMUSG00000094143	B930078G14Rik	RIKEN cDNA B930078G14 gene [Source:MGI Symbo	0.77592
ENSMUSG00000089994	Gm16239	predicted gene 16239 [Source:MGI Symbol;Acc:MG	0.77594
ENSMUSG00000031629	Mlf1ip	myeloid leukemia factor 1 interacting protein [Sour	0.776
ENSMUSG00000038880	Mrps34	mitochondrial ribosomal protein S34 [Source:MGI S	0.77619
ENSMUSG00000080708	Ccnd3-ps	cyclin D3, pseudogene [Source:MGI Symbol;Acc:MG	0.77629
ENSMUSG00000033872	Best4-ps	bestrophin 4, pseudogene [Source:MGI Symbol;Acc	0.77633
ENSMUSG00000068966	Zbtb34	zinc finger and BTB domain containing 34 [Source:M	0.77633
ENSMUSG00000097884	Gm26543	predicted gene, 26543 [Source:MGI Symbol;Acc:MG	0.77634
ENSMUSG00000029564	4930519G04Rik	RIKEN cDNA 4930519G04 gene [Source:MGI Symbo	0.77636
ENSMUSG00000025555	Farp1	FERM, RhoGEF (Arhgef) and pleckstrin domain prot	0.77648
ENSMUSG00000038775	Vill	villin-like [Source:MGI Symbol;Acc:MGI:1201781]	0.7767
ENSMUSG00000026564	Dusp27	dual specificity phosphatase 27 (putative) [Source:M	0.77691
ENSMUSG00000069867	Pabpn1l	poly(A)binding protein nuclear 1-like [Source:MGI S	0.77715
ENSMUSG00000030795	Fus	fused in sarcoma [Source:MGI Symbol;Acc:MGI:135	0.77719
ENSMUSG00000018583	G3bp1	GTPase activating protein (SH3 domain) binding pro	0.77726
ENSMUSG00000073598	1700066B19Rik	RIKEN cDNA 1700066B19 gene [Source:MGI Symbo	0.77739
ENSMUSG00000059070	Rpl18	ribosomal protein L18 [Source:MGI Symbol;Acc:MG	0.77744
ENSMUSG00000071347	C1qtnf9	C1q and tumor necrosis factor related protein 9 [So	0.77749
ENSMUSG00000048677	Tpcn2	two pore segment channel 2 [Source:MGI Symbol;A	0.77751
ENSMUSG00000032604	Qars	glutaminyl-tRNA synthetase [Source:MGI Symbol;A	0.77752
ENSMUSG00000024215	Spdef	SAM pointed domain containing ets transcription fa	0.77768
ENSMUSG00000022433	Csnk1e	casein kinase 1, epsilon [Source:MGI Symbol;Acc:M	0.77792
ENSMUSG00000037111	Setd7	SET domain containing (lysine methyltransferase) 7	0.77814
ENSMUSG00000028528	Dnajc6	DnaJ (Hsp40) homolog, subfamily C, member 6 [Sou	0.77817
ENSMUSG00000084824	Gm16344	predicted gene 16344 [Source:MGI Symbol;Acc:MG	0.7783
ENSMUSG00000041926	Rnpep	arginyl aminopeptidase (aminopeptidase B) [Source	0.77832
ENSMUSG00000018593	Sparc	secreted acidic cysteine rich glycoprotein [Source:M	0.77833

ENSMUSG00000036602	Alx1	ALX homeobox 1 [Source:MGI Symbol;Acc:MGI:104	0.77833
ENSMUSG00000068876	Cgn	cingulin [Source:MGI Symbol;Acc:MGI:1927237]	0.77835
ENSMUSG00000058952	Cfi	complement component factor i [Source:MGI Symb	0.77845
ENSMUSG00000026031	Cflar	CASP8 and FADD-like apoptosis regulator [Source:M	0.7785
ENSMUSG00000078974	Sec61g	SEC61, gamma subunit [Source:MGI Symbol;Acc:M	0.7785
ENSMUSG00000015947	Fcgr1	Fc receptor, IgG, high affinity I [Source:MGI Symbol	0.77859
ENSMUSG00000086216	Gm13610	predicted gene 13610 [Source:MGI Symbol;Acc:MG	0.7786
ENSMUSG00000030177	Ccdc77	coiled-coil domain containing 77 [Source:MGI Symb	0.77862
ENSMUSG00000024182	Axin1	axin 1 [Source:MGI Symbol;Acc:MGI:1096327]	0.77866
ENSMUSG00000080115	Mettl21b	methyltransferase like 21B [Source:MGI Symbol;Ac	0.77873
ENSMUSG00000066148	Prpf4	PRP4 pre-mRNA processing factor 4 homolog (yeas	0.77883
ENSMUSG00000037262	Kin	antigenic determinant of rec-A protein [Source:MG	0.77892
ENSMUSG00000028863	Meaf6	MYST/Esa1-associated factor 6 [Source:MGI Symbo	0.77898
ENSMUSG00000023707	Ogfod2	2-oxoglutarate and iron-dependent oxygenase dom	0.77909
ENSMUSG00000037737	Actrt3	actin related protein T3 [Source:MGI Symbol;Acc:M	0.77911
ENSMUSG00000022246	Rai14	retinoic acid induced 14 [Source:MGI Symbol;Acc:M	0.77915
ENSMUSG00000021699	Pde4d	phosphodiesterase 4D, cAMP specific [Source:MGI	0.77917
ENSMUSG00000074452	Pate2	prostate and testis expressed 2 [Source:MGI Symbo	0.77919
ENSMUSG00000070902	Zfp352	zinc finger protein 352 [Source:MGI Symbol;Acc:M	0.77929
ENSMUSG00000057572	Zbtb8os	zinc finger and BTB domain containing 8 opposite s	0.7793
ENSMUSG00000033213	AA467197	expressed sequence AA467197 [Source:MGI Symbo	0.77936
ENSMUSG00000031298	Gpr64	G protein-coupled receptor 64 [Source:MGI Symbo	0.77956
ENSMUSG00000083415	Gm14690	predicted gene 14690 [Source:MGI Symbol;Acc:MG	0.77968
ENSMUSG00000035864	Syt1	synaptotagmin I [Source:MGI Symbol;Acc:MGI:996	0.77973
ENSMUSG00000022244	Amacr	alpha-methylacyl-CoA racemase [Source:MGI Symb	0.77996
ENSMUSG00000051034	Zfp11	zinc finger protein 11 [Source:MGI Symbol;Acc:MG	0.77996
ENSMUSG00000041669	Prima1	proline rich membrane anchor 1 [Source:MGI Symb	0.78004
ENSMUSG00000015224	Cyp2j9	cytochrome P450, family 2, subfamily j, polypeptide	0.78008
ENSMUSG00000097287	D130017N08Rik	RIKEN cDNA D130017N08 gene [Source:MGI Symbo	0.78013
ENSMUSG00000089798	1700028K03Rik	RIKEN cDNA 1700028K03 gene [Source:MGI Symbo	0.78031
ENSMUSG00000031444	F10	coagulation factor X [Source:MGI Symbol;Acc:MGI:	0.78043
ENSMUSG00000090559	Gm17137	predicted gene 17137 [Source:MGI Symbol;Acc:MG	0.78044
ENSMUSG00000026890	Lhx6	LIM homeobox protein 6 [Source:MGI Symbol;Acc:I	0.78063
ENSMUSG00000049760	2410015M20Rik	RIKEN cDNA 2410015M20 gene [Source:MGI Symbo	0.78066
ENSMUSG00000021186	Fbln5	fibulin 5 [Source:MGI Symbol;Acc:MGI:1346091]	0.78069
ENSMUSG00000028012	Rrh	retinal pigment epithelium derived rhodopsin hom	0.7807
ENSMUSG00000021416	Eci3	enoyl-Coenzyme A delta isomerase 3 [Source:MGI S	0.78075
ENSMUSG00000020806	Rhbdf2	rhomboid 5 homolog 2 (Drosophila) [Source:MGI Sy	0.78094
ENSMUSG00000027175	Tcp11l1	t-complex 11 like 1 [Source:MGI Symbol;Acc:MGI:2	0.78102
ENSMUSG00000086933	4933426K07Rik	RIKEN cDNA 4933426K07 gene [Source:MGI Symbo	0.78104
ENSMUSG00000030747	Dgat2	diacylglycerol O-acyltransferase 2 [Source:MGI Sym	0.78107
ENSMUSG00000020788	Atp2a3	ATPase, Ca++ transporting, ubiquitous [Source:MGI	0.78109
ENSMUSG00000044125	9530080O11Rik	RIKEN cDNA 9530080O11 gene [Source:MGI Symbo	0.78123
ENSMUSG00000091071	1700030C10Rik	RIKEN cDNA 1700030C10 gene [Source:MGI Symbo	0.78125
ENSMUSG00000050930	Map10	microtubule-associated protein 10 [Source:MGI Sym	0.78147
ENSMUSG00000051510	Mafg	v-maf musculoaponeurotic fibrosarcoma oncogene	0.7817
ENSMUSG00000033419	Snap91	synaptosomal-associated protein 91 [Source:MGI S	0.78179

ENSMUSG00000045316	Fahd1	fumarylacetoacetate hydrolase domain containing	0.78183
ENSMUSG00000032412	Atp1b3	ATPase, Na+/K+ transporting, beta 3 polypeptide [S	0.78186
ENSMUSG00000022803	Popdc2	popeye domain containing 2 [Source:MGI Symbol;A	0.78193
ENSMUSG00000028933	Xrcc2	X-ray repair complementing defective repair in Chi	0.78195
ENSMUSG00000085246	Gm15893	predicted gene 15893 [Source:MGI Symbol;Acc:MG	0.78198
ENSMUSG00000028028	Alpk1	alpha-kinase 1 [Source:MGI Symbol;Acc:MGI:19187	0.78202
ENSMUSG00000022842	Ece2	endothelin converting enzyme 2 [Source:MGI Symb	0.78219
ENSMUSG00000009741	Ubp1	upstream binding protein 1 [Source:MGI Symbol;Ac	0.78222
ENSMUSG00000037257	Aagab	alpha- and gamma-adaptin binding protein [Source	0.78227
ENSMUSG00000063297	Luzp2	leucine zipper protein 2 [Source:MGI Symbol;Acc:M	0.78241
ENSMUSG00000020271	Fbxw11	F-box and WD-40 domain protein 11 [Source:MGI S	0.78249
ENSMUSG00000022551	Cyc1	cytochrome c-1 [Source:MGI Symbol;Acc:MGI:1913	0.78251
ENSMUSG00000040740	Slc25a34	solute carrier family 25, member 34 [Source:MGI Sy	0.78258
ENSMUSG00000092545	Gm20319	predicted gene, 20319 [Source:MGI Symbol;Acc:MG	0.78268
ENSMUSG00000086162	Gm16342	predicted gene 16342 [Source:MGI Symbol;Acc:MG	0.78277
ENSMUSG00000089661	Mia	melanoma inhibitory activity [Source:MGI Symbol;A	0.78277
ENSMUSG00000034467	Dynlrb2	dynein light chain roadblock-type 2 [Source:MGI Sy	0.78279
ENSMUSG00000090785	Gm17116	predicted gene 17116 [Source:MGI Symbol;Acc:MG	0.78279
ENSMUSG00000047636	Cdcp2	CUB domain containing protein 2 [Source:MGI Sym	0.7828
ENSMUSG00000082990	Gm14052	predicted gene 14052 [Source:MGI Symbol;Acc:MG	0.7828
ENSMUSG00000026275	Ppp1r7	protein phosphatase 1, regulatory (inhibitor) subun	0.78287
ENSMUSG00000042644	Itpr3	inositol 1,4,5-triphosphate receptor 3 [Source:MGI	0.78298
ENSMUSG00000024421	Lama3	laminin, alpha 3 [Source:MGI Symbol;Acc:MGI:9990	0.78301
ENSMUSG00000049881	2810025M15Rik	RIKEN cDNA 2810025M15 gene [Source:MGI Symbo	0.78302
ENSMUSG00000057354	4930430O22Rik	RIKEN cDNA 4930430O22 gene [Source:MGI Symbo	0.78303
ENSMUSG00000086806	Gm13054	predicted gene 13054 [Source:MGI Symbol;Acc:MG	0.78304
ENSMUSG00000074059	Fbxw18	F-box and WD-40 domain protein 18 [Source:MGI S	0.78322
ENSMUSG00000035296	Sgcg	sarcoglycan, gamma (dystrophin-associated glycopr	0.78324
ENSMUSG00000096635	Gm22174	predicted gene, 22174 [Source:MGI Symbol;Acc:MG	0.78328
ENSMUSG00000044827	Tlr1	toll-like receptor 1 [Source:MGI Symbol;Acc:MGI:13	0.78329
ENSMUSG00000047171	Helt	helt bHLH transcription factor [Source:MGI Symbol;	0.78329
ENSMUSG00000051777	Iqcj	IQ motif containing J [Source:MGI Symbol;Acc:MGI	0.78329
ENSMUSG00000067001	Serpinb7	serine (or cysteine) peptidase inhibitor, clade B, me	0.78329
ENSMUSG00000034438	Gbp8	guanylate-binding protein 8 [Source:MGI Symbol;A	0.7833
ENSMUSG00000090164	BC035044	cDNA sequence BC035044 [Source:MGI Symbol;Acc	0.78331
ENSMUSG00000084422	Gm25108	predicted gene, 25108 [Source:MGI Symbol;Acc:MG	0.78332
ENSMUSG00000020310	Madcam1	mucosal vascular addressin cell adhesion molecule	0.78335
ENSMUSG00000033004	Mycbp2	MYC binding protein 2 [Source:MGI Symbol;Acc:MG	0.78337
ENSMUSG00000094350	Gm10931	predicted gene 10931 [Source:MGI Symbol;Acc:MG	0.78337
ENSMUSG00000038572	Bpifb5	BPI fold containing family B, member 5 [Source:MG	0.78338
ENSMUSG00000024664	Fads3	fatty acid desaturase 3 [Source:MGI Symbol;Acc:MG	0.78356
ENSMUSG00000025473	Adam8	a disintegrin and metallopeptidase domain 8 [Sourc	0.78358
ENSMUSG00000019808	Adat2	adenosine deaminase, tRNA-specific 2 [Source:MGI	0.78359
ENSMUSG00000050323	Ndufaf6	NADH dehydrogenase (ubiquinone) complex I, asse	0.78359
ENSMUSG00000088769	Gm22637	predicted gene, 22637 [Source:MGI Symbol;Acc:MG	0.78369
ENSMUSG00000096941	Gm26720	predicted gene, 26720 [Source:MGI Symbol;Acc:MG	0.78374
ENSMUSG00000036766	Dner	delta/notch-like EGF-related receptor [Source:MGI	0.78378

ENSMUSG00000024426	Atat1	alpha tubulin acetyltransferase 1 [Source:MGI Syml	0.78382
ENSMUSG00000032308	Ulk3	unc-51-like kinase 3 [Source:MGI Symbol;Acc:MGI:1	0.78382
ENSMUSG00000031168	Ebp	phenylalkylamine Ca2+ antagonist (emopamil) binc	0.78384
ENSMUSG00000094685	Gm5900	predicted pseudogene 5900 [Source:MGI Symbol;A	0.78385
ENSMUSG00000026274	Pask	PAS domain containing serine/threonine kinase [So	0.78403
ENSMUSG00000020696	Rffl	ring finger and FYVE like domain containing protein	0.78404
ENSMUSG00000027978	Prss12	protease, serine, 12 neurotrypsin (motopsin) [Sourc	0.78414
ENSMUSG00000027843	Ptpn22	protein tyrosine phosphatase, non-receptor type 2	0.78416
ENSMUSG00000001228	Uhrf1	ubiquitin-like, containing PHD and RING finger dom	0.78424
ENSMUSG00000024049	Myom1	myomesin 1 [Source:MGI Symbol;Acc:MGI:134143(	0.78424
ENSMUSG00000066037	Hnrnpr	heterogeneous nuclear ribonucleoprotein R [Sourc	0.78424
ENSMUSG00000010476	Ebf3	early B cell factor 3 [Source:MGI Symbol;Acc:MGI:8	0.7843
ENSMUSG00000025477	Inpp5a	inositol polyphosphate-5-phosphatase A [Source:M	0.7844
ENSMUSG00000022768	Ccdc116	coiled-coil domain containing 116 [Source:MGI Sym	0.78462
ENSMUSG00000046856	Gpr1	G protein-coupled receptor 1 [Source:MGI Symbol;	0.78474
ENSMUSG00000031465	Angpt2	angiopoietin 2 [Source:MGI Symbol;Acc:MGI:12028	0.78479
ENSMUSG00000041836	Ptpre	protein tyrosine phosphatase, receptor type, E [Sou	0.78482
ENSMUSG00000039873	Neurl2	neuralized-like 2 (Drosophila) [Source:MGI Symbol;	0.78488
ENSMUSG00000023977	Ubr2	ubiquitin protein ligase E3 component n-recognin 2	0.78495
ENSMUSG00000007338	Mrpl49	mitochondrial ribosomal protein L49 [Source:MGI S	0.78512
ENSMUSG00000024725	Ostf1	osteoclast stimulating factor 1 [Source:MGI Symbol	0.78515
ENSMUSG00000028954	Nub1	negative regulator of ubiquitin-like proteins 1 [Sour	0.78519
ENSMUSG00000065475	Mir27b	microRNA 27b [Source:MGI Symbol;Acc:MGI:26769	0.78524
ENSMUSG00000035152	Ap2b1	adaptor-related protein complex 2, beta 1 subunit	0.7853
ENSMUSG00000027583	Zbtb46	zinc finger and BTB domain containing 46 [Source:N	0.78542
ENSMUSG00000021650	Ptcd2	pentatricopeptide repeat domain 2 [Source:MGI Sy	0.78559
ENSMUSG00000024818	Slc25a45	solute carrier family 25, member 45 [Source:MGI Sy	0.78566
ENSMUSG00000054619	Mettl7a1	methyltransferase like 7A1 [Source:MGI Symbol;Ac	0.78569
ENSMUSG00000037362	Nov	nephroblastoma overexpressed gene [Source:MGI S	0.78573
ENSMUSG00000042708	Shcbp1l	Shc SH2-domain binding protein 1-like [Source:MGI	0.78573
ENSMUSG00000095115	Itpripl2	inositol 1,4,5-triphosphate receptor interacting pro	0.78579
ENSMUSG00000046733	Gprc5a	G protein-coupled receptor, family C, group 5, mem	0.7858
ENSMUSG00000055200	Sertad3	SERTA domain containing 3 [Source:MGI Symbol;Ac	0.786
ENSMUSG00000097540	1700037F24Rik	RIKEN cDNA 1700037F24 gene [Source:MGI Symbo	0.78604
ENSMUSG00000045176	2310047M10Rik	RIKEN cDNA 2310047M10 gene [Source:MGI Symb	0.78608
ENSMUSG00000068706	Gm10250	predicted pseudogene 10250 [Source:MGI Symbol;	0.78608
ENSMUSG00000040139	9430038I01Rik	RIKEN cDNA 9430038I01 gene [Source:MGI Symbol	0.78618
ENSMUSG00000076123	Mir700	microRNA 700 [Source:MGI Symbol;Acc:MGI:36296	0.78627
ENSMUSG00000089698	Gm2541	predicted pseudogene 2541 [Source:MGI Symbol;A	0.78627
ENSMUSG00000045515	Pou3f3	POU domain, class 3, transcription factor 3 [Source	0.78629
ENSMUSG00000073486	Gm10518	predicted gene 10518 [Source:MGI Symbol;Acc:MG	0.78629
ENSMUSG00000037573	Tob1	transducer of ErbB-2.1 [Source:MGI Symbol;Acc:M(	0.78633
ENSMUSG00000082605	Gm16148	predicted gene 16148 [Source:MGI Symbol;Acc:MG	0.78633
ENSMUSG00000032056	Btg4	B cell translocation gene 4 [Source:MGI Symbol;Acc	0.78634
ENSMUSG00000021927	Gm5803	predicted gene 5803 [Source:MGI Symbol;Acc:MGI:	0.78636
ENSMUSG00000081223	Gm12247	predicted gene 12247 [Source:MGI Symbol;Acc:MG	0.78645
ENSMUSG00000039676	Capsl	calcyphosine-like [Source:MGI Symbol;Acc:MGI:192	0.78649

ENSMUSG00000074128	Gm10631	predicted gene 10631 [Source:MGI Symbol;Acc:MG	0.78649
ENSMUSG00000026818	Cel	carboxyl ester lipase [Source:MGI Symbol;Acc:MGI:	0.7865
ENSMUSG00000054362	BC055111	cDNA sequence BC055111 [Source:MGI Symbol;Acc	0.78651
ENSMUSG00000041921	Metap1d	methionyl aminopeptidase type 1D (mitochondrial)	0.78654
ENSMUSG00000059689	Zfp637	zinc finger protein 637 [Source:MGI Symbol;Acc:MG	0.78654
ENSMUSG00000062365	Gm4968	predicted gene 4968 [Source:MGI Symbol;Acc:MGI:	0.78662
ENSMUSG00000096890	Gm20797	predicted gene, 20797 [Source:MGI Symbol;Acc:MG	0.78663
ENSMUSG00000014686	Ceacam16	carcinoembryonic antigen-related cell adhesion mo	0.78664
ENSMUSG00000073174	Magi2	membrane associated guanylate kinase, WW and P	0.78667
ENSMUSG00000029624	Ptcd1	pentatricopeptide repeat domain 1 [Source:MGI Sy	0.78668
ENSMUSG00000035226	Rims4	regulating synaptic membrane exocytosis 4 [Source	0.78676
ENSMUSG00000023886	Smoc2	SPARC related modular calcium binding 2 [Source:M	0.78683
ENSMUSG00000053889	A130066N16Rik	RIKEN cDNA A130066N16 gene [Source:MGI Symbo	0.78684
ENSMUSG00000050747	Trim15	tripartite motif-containing 15 [Source:MGI Symbol;	0.78688
ENSMUSG00000021815	Mss51	MSS51 mitochondrial translational activator [Sourc	0.78691
ENSMUSG00000075267	Dfnb59	deafness, autosomal recessive 59 (human) [Source:	0.78694
ENSMUSG00000097991	Gm7656	predicted gene 7656 [Source:MGI Symbol;Acc:MGI:	0.78696
ENSMUSG00000051527	Usp29	ubiquitin specific peptidase 29 [Source:MGI Symbo	0.78698
ENSMUSG00000087013	2610027K06Rik	RIKEN cDNA 2610027K06 gene [Source:MGI Symbo	0.78707
ENSMUSG00000053153	Spag16	sperm associated antigen 16 [Source:MGI Symbol;A	0.78716
ENSMUSG00000086732	Gm6117	predicted gene 6117 [Source:MGI Symbol;Acc:MGI:	0.78717
ENSMUSG00000094244	Gm23967	predicted gene, 23967 [Source:MGI Symbol;Acc:MG	0.78717
ENSMUSG00000097654	Gm26714	predicted gene, 26714 [Source:MGI Symbol;Acc:MG	0.78717
ENSMUSG00000021694	Ercc8	excision repaiross-complementing rodent repair de	0.78736
ENSMUSG00000087683	Gm11712	predicted gene 11712 [Source:MGI Symbol;Acc:MG	0.78741
ENSMUSG00000093490	Gm19932	predicted gene, 19932 [Source:MGI Symbol;Acc:MG	0.78742
ENSMUSG00000032495	Lrrc2	leucine rich repeat containing 2 [Source:MGI Symbo	0.78745
ENSMUSG00000018986	Slfn3	schlafen 3 [Source:MGI Symbol;Acc:MGI:1329005]	0.78752
ENSMUSG00000090307	1700071M16Rik	RIKEN cDNA 1700071M16 gene [Source:MGI Symbo	0.78757
ENSMUSG00000057315	Arhgap24	Rho GTPase activating protein 24 [Source:MGI Sym	0.78767
ENSMUSG00000056367	Actr3b	ARP3 actin-related protein 3B [Source:MGI Symbol;	0.78776
ENSMUSG00000022464	Slc38a4	solute carrier family 38, member 4 [Source:MGI Sym	0.78779
ENSMUSG00000063885	Gm6498	predicted gene 6498 [Source:MGI Symbol;Acc:MGI:	0.78781
ENSMUSG00000039795	Zfand1	zinc finger, AN1-type domain 1 [Source:MGI Symbo	0.78791
ENSMUSG00000036833	Pnpla7	patatin-like phospholipase domain containing 7 [So	0.78802
ENSMUSG00000003476	Crhr2	corticotropin releasing hormone receptor 2 [Source	0.78813
ENSMUSG00000058586	Serhl	serine hydrolase-like [Source:MGI Symbol;Acc:MGI	0.78814
ENSMUSG00000083157	Gm13511	predicted gene 13511 [Source:MGI Symbol;Acc:MG	0.78817
ENSMUSG00000052833	Sae1	SUMO1 activating enzyme subunit 1 [Source:MGI S	0.78818
ENSMUSG00000023073	Slc10a2	solute carrier family 10, member 2 [Source:MGI Sym	0.78819
ENSMUSG00000045215	Asxl3	additional sex combs like 3 (Drosophila) [Source:MG	0.78819
ENSMUSG00000086049	Gm12735	predicted gene 12735 [Source:MGI Symbol;Acc:MG	0.78819
ENSMUSG00000085087	Gm13528	predicted gene 13528 [Source:MGI Symbol;Acc:MG	0.7882
ENSMUSG00000086946	Gm15527	predicted gene 15527 [Source:MGI Symbol;Acc:MG	0.78827
ENSMUSG00000081219	Bambi-ps1	BMP and activin membrane-bound inhibitor, pseud	0.78828
ENSMUSG00000093067	Gm23156	predicted gene, 23156 [Source:MGI Symbol;Acc:MG	0.78828
ENSMUSG00000079263	Gm6614	predicted gene 6614 [Source:MGI Symbol;Acc:MGI:	0.78829

ENSMUSG00000087282	Gm15317	predicted gene 15317 [Source:MGI Symbol;Acc:MG	0.78829
ENSMUSG00000094947	Gm3944	predicted gene 3944 [Source:MGI Symbol;Acc:MGI:	0.78829
ENSMUSG00000096052	9930004E17Rik	RIKEN cDNA 9930004E17 gene [Source:MGI Symbo	0.7883
ENSMUSG00000015316	Slamf1	signaling lymphocytic activation molecule family m	0.78835
ENSMUSG00000065619	Mir183	microRNA 183 [Source:MGI Symbol;Acc:MGI:26768	0.78835
ENSMUSG00000092248	Vmn1r-ps63	vomeronasal 1 receptor, pseudogene 63 [Source:M	0.78837
ENSMUSG00000090957	Gm7535	predicted gene 7535 [Source:MGI Symbol;Acc:MGI:	0.78838
ENSMUSG00000080406	Mir1188	microRNA 1188 [Source:MGI Symbol;Acc:MGI:3783	0.78839
ENSMUSG00000087474	Gm12100	predicted gene 12100 [Source:MGI Symbol;Acc:MG	0.78839
ENSMUSG00000078580	E430018J23Rik	RIKEN cDNA E430018J23 gene [Source:MGI Symbol	0.78845
ENSMUSG00000032294	Pkm	pyruvate kinase, muscle [Source:MGI Symbol;Acc:M	0.78846
ENSMUSG00000085277	Gm13943	predicted gene 13943 [Source:MGI Symbol;Acc:MG	0.78857
ENSMUSG00000020085	Aifm2	apoptosis-inducing factor, mitochondrion-associate	0.78865
ENSMUSG00000036819	Jmjd4	jumonji domain containing 4 [Source:MGI Symbol;A	0.78866
ENSMUSG00000097797	Gm26901	predicted gene, 26901 [Source:MGI Symbol;Acc:M	0.7889
ENSMUSG00000026826	Nr4a2	nuclear receptor subfamily 4, group A, member 2 [S	0.789
ENSMUSG00000005054	Cstb	cystatin B [Source:MGI Symbol;Acc:MGI:109514]	0.78902
ENSMUSG00000089964	Gm16582	predicted gene 16582 [Source:MGI Symbol;Acc:MG	0.78907
ENSMUSG00000054764	Mtnr1a	melatonin receptor 1A [Source:MGI Symbol;Acc:M	0.78918
ENSMUSG00000008348	Ubc	ubiquitin C [Source:MGI Symbol;Acc:MGI:98889]	0.7892
ENSMUSG00000097296	Gm26532	predicted gene, 26532 [Source:MGI Symbol;Acc:M	0.78935
ENSMUSG00000031101	Sash3	SAM and SH3 domain containing 3 [Source:MGI Sym	0.78943
ENSMUSG00000038168	Leprel1	leprecan-like 1 [Source:MGI Symbol;Acc:MGI:2146	0.78946
ENSMUSG00000070366	Ppapdc1a	phosphatidic acid phosphatase type 2 domain cont	0.78951
ENSMUSG00000025366	Esyt1	extended synaptotagmin-like protein 1 [Source:MG	0.78961
ENSMUSG00000097730	Gm26588	predicted gene, 26588 [Source:MGI Symbol;Acc:M	0.79003
ENSMUSG00000093565	2610019E17Rik	RIKEN cDNA 2610019E17 gene [Source:MGI Symbo	0.79012
ENSMUSG00000086363	A330102I10Rik	RIKEN cDNA A330102I10 gene [Source:MGI Symbol	0.79014
ENSMUSG00000070683	Lactbl1	lactamase, beta-like 1 [Source:MGI Symbol;Acc:MG	0.79018
ENSMUSG00000026807	Ak8	adenylate kinase 8 [Source:MGI Symbol;Acc:MGI:1	0.7903
ENSMUSG00000054723	Vmac	vimentin-type intermediate filament associated coi	0.79037
ENSMUSG00000029175	Slc35f6	solute carrier family 35, member F6 [Source:MGI S	0.79044
ENSMUSG00000097597	Gm26674	predicted gene, 26674 [Source:MGI Symbol;Acc:M	0.79046
ENSMUSG00000068015	Lrch1	leucine-rich repeats and calponin homology (CH) d	0.7905
ENSMUSG00000027832	Ptx3	pentraxin related gene [Source:MGI Symbol;Acc:M	0.79052
ENSMUSG00000052056	Zfp217	zinc finger protein 217 [Source:MGI Symbol;Acc:M	0.79055
ENSMUSG00000017765	Slc12a4	solute carrier family 12, member 4 [Source:MGI Sy	0.79058
ENSMUSG00000061517	Sox21	SRY-box containing gene 21 [Source:MGI Symbol;A	0.79058
ENSMUSG00000075254	Heg1	HEG homolog 1 (zebrafish) [Source:MGI Symbol;Ac	0.79061
ENSMUSG00000076190	Gm23256	predicted gene, 23256 [Source:MGI Symbol;Acc:M	0.79069
ENSMUSG00000048807	Slc35e4	solute carrier family 35, member E4 [Source:MGI S	0.79071
ENSMUSG00000019837	Gtf3c6	general transcription factor IIIC, polypeptide 6, alp	0.7909
ENSMUSG00000053194	Cib4	calcium and integrin binding family member 4 [Sou	0.79111
ENSMUSG00000087176	D230022J07Rik	RIKEN cDNA D230022J07 gene [Source:MGI Symbo	0.79116
ENSMUSG00000025747	Tyms	thymidylate synthase [Source:MGI Symbol;Acc:MG	0.79117
ENSMUSG00000054385	Ceacam2	carcinoembryonic antigen-related cell adhesion mo	0.79117
ENSMUSG00000023094	Msrb2	methionine sulfoxide reductase B2 [Source:MGI Sy	0.79123

ENSMUSG00000043923	Ccdc84	coiled-coil domain containing 84 [Source:MGI Symb	0.79143
ENSMUSG00000079489	C030013D06Rik	RIKEN cDNA C030013D06 gene [Source:MGI Symbo	0.79144
ENSMUSG00000029723	Tsc22d4	TSC22 domain family, member 4 [Source:MGI Symb	0.79158
ENSMUSG00000031740	Mmp2	matrix metallopeptidase 2 [Source:MGI Symbol;Acc	0.7916
ENSMUSG00000093256	Gm22933	predicted gene, 22933 [Source:MGI Symbol;Acc:MG	0.79166
ENSMUSG00000091007	D630036H23Rik	RIKEN cDNA D630036H23 gene [Source:MGI Symbo	0.79175
ENSMUSG00000031851	Ntpcr	nucleoside-triphosphatase, cancer-related [Source:	0.79187
ENSMUSG00000047123	Ticam1	toll-like receptor adaptor molecule 1 [Source:MGI S	0.7919
ENSMUSG00000091351	Gm17135	predicted gene 17135 [Source:MGI Symbol;Acc:MG	0.7919
ENSMUSG00000007833	Aldh16a1	aldehyde dehydrogenase 16 family, member A1 [So	0.79205
ENSMUSG00000018567	Gabarap	gamma-aminobutyric acid receptor associated prot	0.79213
ENSMUSG00000034255	Arhgap27	Rho GTPase activating protein 27 [Source:MGI Sym	0.79223
ENSMUSG00000048285	Frmd6	FERM domain containing 6 [Source:MGI Symbol;Ac	0.79226
ENSMUSG00000087253	Gm12043	predicted gene 12043 [Source:MGI Symbol;Acc:MG	0.79229
ENSMUSG00000091155	Serpine3	serpin peptidase inhibitor, clade E (nexin, plasmino	0.7923
ENSMUSG00000081747	Rpl7a-ps9	ribosomal protein L7A, pseudogene 9 [Source:MGI	0.79234
ENSMUSG00000038429	Usp5	ubiquitin specific peptidase 5 (isopeptidase T) [Sou	0.79236
ENSMUSG00000096544	Gm4617	predicted pseudogene 4617 [Source:MGI Symbol;A	0.79238
ENSMUSG00000022774	Ncbp2	nuclear cap binding protein subunit 2 [Source:MGI	0.79239
ENSMUSG00000022587	Ly6e	lymphocyte antigen 6 complex, locus E [Source:MG	0.79248
ENSMUSG00000063767	S100a7a	S100 calcium binding protein A7A [Source:MGI Sym	0.79248
ENSMUSG00000052854	Nrk	Nik related kinase [Source:MGI Symbol;Acc:MGI:13	0.79254
ENSMUSG00000042351	Grap2	GRB2-related adaptor protein 2 [Source:MGI Symb	0.79263
ENSMUSG00000034522	Zfp395	zinc finger protein 395 [Source:MGI Symbol;Acc:MG	0.79264
ENSMUSG00000096299	Gm21814	predicted gene, 21814 [Source:MGI Symbol;Acc:MG	0.79267
ENSMUSG00000088208	Gm23751	predicted gene, 23751 [Source:MGI Symbol;Acc:MG	0.79273
ENSMUSG00000031292	Cdkl5	cyclin-dependent kinase-like 5 [Source:MGI Symbol	0.79275
ENSMUSG00000081436	Gm5912	predicted gene 5912 [Source:MGI Symbol;Acc:MGI:	0.79286
ENSMUSG00000027344	Fsip1	fibrous sheath-interacting protein 1 [Source:MGI Sy	0.79287
ENSMUSG00000079254	Itprip	inositol 1,4,5-triphosphate receptor interacting pro	0.79287
ENSMUSG00000097789	Gm2115	predicted gene 2115 [Source:MGI Symbol;Acc:MGI:	0.79287
ENSMUSG00000045534	Kcna5	potassium voltage-gated channel, shaker-related su	0.7929
ENSMUSG00000042814	Mcts2	malignant T cell amplified sequence 2 [Source:MGI	0.79294
ENSMUSG00000086031	Gm12348	predicted gene 12348 [Source:MGI Symbol;Acc:MG	0.79299
ENSMUSG00000021216	Tubal3	tubulin, alpha-like 3 [Source:MGI Symbol;Acc:MGI:3	0.793
ENSMUSG00000006763	Saal1	serum amyloid A-like 1 [Source:MGI Symbol;Acc:M	0.79302
ENSMUSG00000062087	4930423O20Rik	RIKEN cDNA 4930423O20 gene [Source:MGI Symbo	0.79306
ENSMUSG00000026114	Cnga3	cyclic nucleotide gated channel alpha 3 [Source:MG	0.79314
ENSMUSG00000014554	Dguok	deoxyguanosine kinase [Source:MGI Symbol;Acc:M	0.7933
ENSMUSG00000031767	Nudt7	nudix (nucleoside diphosphate linked moiety X)-typ	0.79331
ENSMUSG00000031428	Zcchc18	zinc finger, CCHC domain containing 18 [Source:MG	0.79353
ENSMUSG00000011148	Adssl1	adenylosuccinate synthetase like 1 [Source:MGI Sy	0.79356
ENSMUSG00000079737	3110001I22Rik	RIKEN cDNA 3110001I22 gene [Source:MGI Symbol	0.79357
ENSMUSG00000029073	Gltpd1	glycolipid transfer protein domain containing 1 [Sou	0.79376
ENSMUSG00000037816	Fbxw17	F-box and WD-40 domain protein 17 [Source:MGI S	0.79381
ENSMUSG00000028407	2010003O02Rik	RIKEN cDNA 2010003O02 gene [Source:MGI Symbo	0.79386
ENSMUSG00000031772	Cntnap4	contactin associated protein-like 4 [Source:MGI Syr	0.79402

ENSMUSG00000037295	Ldlrap1	low density lipoprotein receptor adaptor protein 1	0.79406
ENSMUSG00000026940	4921530D09Rik	RIKEN cDNA 4921530D09 gene [Source:MGI Symbo	0.79421
ENSMUSG00000024754	Tmem2	transmembrane protein 2 [Source:MGI Symbol;Acc	0.79423
ENSMUSG00000020186	Csrp2	cysteine and glycine-rich protein 2 [Source:MGI Syr	0.79429
ENSMUSG00000050709	4932442E05Rik	RIKEN cDNA 4932442E05 gene [Source:MGI Symbo	0.79445
ENSMUSG00000024346	Pfdn1	prefoldin 1 [Source:MGI Symbol;Acc:MGI:1914449]	0.7945
ENSMUSG00000056316	Gm9996	predicted gene 9996 [Source:MGI Symbol;Acc:MGI:	0.79464
ENSMUSG00000020642	Rnf144a	ring finger protein 144A [Source:MGI Symbol;Acc:M	0.79467
ENSMUSG00000025855	Prkar1b	protein kinase, cAMP dependent regulatory, type I	0.79475
ENSMUSG00000026027	Stradb	STE20-related kinase adaptor beta [Source:MGI Syr	0.7948
ENSMUSG00000035530	Eif1	eukaryotic translation initiation factor 1 [Source:M	0.79481
ENSMUSG00000092399	4930556N13Rik	RIKEN cDNA 4930556N13 gene [Source:MGI Symbo	0.79488
ENSMUSG00000097033	Gm26819	predicted gene, 26819 [Source:MGI Symbol;Acc:M	0.79488
ENSMUSG00000024793	Tnfrsf25	tumor necrosis factor receptor superfamily, membe	0.79492
ENSMUSG00000035165	Kcne3	potassium voltage-gated channel, Isk-related subfa	0.79494
ENSMUSG00000090828	Sox2ot	SOX2 overlapping transcript (non-protein coding) [S	0.79494
ENSMUSG00000046058	Eid2	EP300 interacting inhibitor of differentiation 2 [Sou	0.79498
ENSMUSG00000006542	Prkag3	protein kinase, AMP-activated, gamma 3 non-catat	0.79503
ENSMUSG00000027886	1700013F07Rik	RIKEN cDNA 1700013F07 gene [Source:MGI Symbo	0.79513
ENSMUSG00000067399	Trim43c	tripartite motif-containing 43C [Source:MGI Symbo	0.79522
ENSMUSG00000027245	Hypk	huntingtin interacting protein K [Source:MGI Symbo	0.79526
ENSMUSG00000040584	Abcb1a	ATP-binding cassette, sub-family B (MDR/TAP), mer	0.79533
ENSMUSG00000070563	Spaca4	sperm acrosome associated 4 [Source:MGI Symbol;	0.79542
ENSMUSG00000028692	Akr1a1	aldo-keto reductase family 1, member A1 (aldehyd	0.79565
ENSMUSG00000078763	Slfn1	schlafen 1 [Source:MGI Symbol;Acc:MGI:1313259]	0.79577
ENSMUSG00000020415	Pttg1	pituitary tumor-transforming gene 1 [Source:MGI S	0.79584
ENSMUSG00000038056	Kmt2c	lysine (K)-specific methyltransferase 2C [Source:MG	0.79585
ENSMUSG00000018160	Med1	mediator complex subunit 1 [Source:MGI Symbol;A	0.79603
ENSMUSG00000049115	Agtr1a	angiotensin II receptor, type 1a [Source:MGI Symbo	0.79616
ENSMUSG00000024235	Map3k8	mitogen-activated protein kinase kinase kinase 8 [S	0.79617
ENSMUSG00000071424	Grid2	glutamate receptor, ionotropic, delta 2 [Source:MG	0.79625
ENSMUSG00000019796	Lrp11	low density lipoprotein receptor-related protein 11	0.79627
ENSMUSG00000090263	D730045A05Rik	RIKEN cDNA D730045A05 gene [Source:MGI Symbo	0.79627
ENSMUSG00000040282	BC052040	cDNA sequence BC052040 [Source:MGI Symbol;Acc	0.79629
ENSMUSG00000030647	Ndufc2	NADH dehydrogenase (ubiquinone) 1, subcomplex	0.79637
ENSMUSG00000087924	Gm23231	predicted gene, 23231 [Source:MGI Symbol;Acc:M	0.79643
ENSMUSG00000054256	Msi1	musashi RNA-binding protein 1 [Source:MGI Symbo	0.79651
ENSMUSG00000053091	Lins	lines homolog (Drosophila) [Source:MGI Symbol;Ac	0.79665
ENSMUSG00000068742	Cry2	cryptochrome 2 (photolyase-like) [Source:MGI Sym	0.79667
ENSMUSG00000034591	Slc41a2	solute carrier family 41, member 2 [Source:MGI Syr	0.79673
ENSMUSG00000038725	Pkhd1l1	polycystic kidney and hepatic disease 1-like 1 [Sour	0.79675
ENSMUSG00000081732	Gm15495	predicted gene 15495 [Source:MGI Symbol;Acc:MG	0.79677
ENSMUSG00000029998	Pcyox1	prenylcysteine oxidase 1 [Source:MGI Symbol;Acc:M	0.79705
ENSMUSG00000030279	C2cd5	C2 calcium-dependent domain containing 5 [Source	0.79707
ENSMUSG00000082394	Gm4596	predicted gene 4596 [Source:MGI Symbol;Acc:MGI:	0.79708
ENSMUSG00000038766	Gabpb2	GA repeat binding protein, beta 2 [Source:MGI Sym	0.79714
ENSMUSG00000054381	Zfp747	zinc finger protein 747 [Source:MGI Symbol;Acc:MG	0.7972

ENSMUSG00000020079	Supv3l1	suppressor of var1, 3-like 1 (S. cerevisiae) [Source:M	0.79727
ENSMUSG00000037747	Phyhipl	phytanoyl-CoA hydroxylase interacting protein-like	0.79727
ENSMUSG00000055567	Unc80	unc-80 homolog (C. elegans) [Source:MGI Symbol;A	0.79731
ENSMUSG00000071691	Gm960	predicted gene 960 [Source:MGI Symbol;Acc:MGI:2	0.79741
ENSMUSG00000032936	Camkv	CaM kinase-like vesicle-associated [Source:MGI Syr	0.7977
ENSMUSG00000058740	Kcnt1	potassium channel, subfamily T, member 1 [Source	0.79777
ENSMUSG00000084572	Gm23657	predicted gene, 23657 [Source:MGI Symbol;Acc:MG	0.79789
ENSMUSG00000071650	Ganab	alpha glucosidase 2 alpha neutral subunit [Source:M	0.79802
ENSMUSG00000024066	Xdh	xanthine dehydrogenase [Source:MGI Symbol;Acc:I	0.79825
ENSMUSG00000029769	Ccdc136	coiled-coil domain containing 136 [Source:MGI Sym	0.79833
ENSMUSG00000018906	P4ha2	procollagen-proline, 2-oxoglutarate 4-dioxygenase	0.79837
ENSMUSG00000032540	Abhd5	abhydrolase domain containing 5 [Source:MGI Sym	0.79837
ENSMUSG00000029536	Gatc	glutamyl-tRNA(Gln) amidotransferase, subunit C ho	0.79842
ENSMUSG00000018169	Mfng	MFNG O-fucosylpeptide 3-beta-N-acetylglucosamir	0.79863
ENSMUSG00000025198	Erlin1	ER lipid raft associated 1 [Source:MGI Symbol;Acc:N	0.79874
ENSMUSG00000022586	Ly6i	lymphocyte antigen 6 complex, locus I [Source:MGI	0.79879
ENSMUSG00000022991	Lalba	lactalbumin, alpha [Source:MGI Symbol;Acc:MGI:96	0.79883
ENSMUSG00000086866	4930512H18Rik	RIKEN cDNA 4930512H18 gene [Source:MGI Symbo	0.79884
ENSMUSG00000089146	Gm25266	predicted gene, 25266 [Source:MGI Symbol;Acc:MG	0.79885
ENSMUSG00000085885	Gm11906	predicted gene 11906 [Source:MGI Symbol;Acc:MG	0.79887
ENSMUSG00000059562	Ccdc154	coiled-coil domain containing 154 [Source:MGI Sym	0.79891
ENSMUSG00000089810	Gm16536	predicted gene 16536 [Source:MGI Symbol;Acc:MG	0.79891
ENSMUSG00000038257	Glra3	glycine receptor, alpha 3 subunit [Source:MGI Symb	0.79892
ENSMUSG00000073290	Gm10490	predicted gene 10490 [Source:MGI Symbol;Acc:MG	0.79892
ENSMUSG00000028825	Rhd	Rh blood group, D antigen [Source:MGI Symbol;Acc	0.79893
ENSMUSG00000015668	Pdzd11	PDZ domain containing 11 [Source:MGI Symbol;Acc	0.79894
ENSMUSG00000086670	Gm13194	predicted gene 13194 [Source:MGI Symbol;Acc:MG	0.79894
ENSMUSG00000023905	Tnfrsf12a	tumor necrosis factor receptor superfamily, membe	0.79897
ENSMUSG00000069920	B3gnt9	UDP-GlcNAc:betaGal beta-1,3-N-acetylglucosaminy	0.79901
ENSMUSG00000020917	Acly	ATP citrate lyase [Source:MGI Symbol;Acc:MGI:103	0.79911
ENSMUSG00000037138	Aff3	AF4/FMR2 family, member 3 [Source:MGI Symbol;A	0.79913
ENSMUSG00000027678	Ncoa3	nuclear receptor coactivator 3 [Source:MGI Symbol	0.79927
ENSMUSG00000044949	Ubtd2	ubiquitin domain containing 2 [Source:MGI Symbol	0.79929
ENSMUSG00000005237	Dnah2	dynein, axonemal, heavy chain 2 [Source:MGI Symb	0.7993
ENSMUSG00000031568	Rwdd4a	RWD domain containing 4A [Source:MGI Symbol;Ac	0.79931
ENSMUSG00000007411	Mark3	MAP/microtubule affinity-regulating kinase 3 [Sour	0.79932
ENSMUSG00000031758	Cdyl2	chromodomain protein, Y chromosome-like 2 [Sour	0.79936
ENSMUSG00000038201	Kcna7	potassium voltage-gated channel, shaker-related su	0.79953
ENSMUSG00000022843	Clcn2	chloride channel 2 [Source:MGI Symbol;Acc:MGI:10	0.79955
ENSMUSG00000057072	1700022P22Rik	RIKEN cDNA 1700022P22 gene [Source:MGI Symbo	0.79956
ENSMUSG00000009013	Dynll1	dynein light chain LC8-type 1 [Source:MGI Symbol;A	0.79961
ENSMUSG00000027249	F2	coagulation factor II [Source:MGI Symbol;Acc:MGI:8	0.79967
ENSMUSG00000033715	Akr1c14	aldo-keto reductase family 1, member C14 [Source	0.79969
ENSMUSG00000020873	Slc35b1	solute carrier family 35, member B1 [Source:MGI Sy	0.79972
ENSMUSG00000082140	Gm13094	predicted gene 13094 [Source:MGI Symbol;Acc:MG	0.79978
ENSMUSG00000091784	Gm17022	predicted gene 17022 [Source:MGI Symbol;Acc:MG	0.7998
ENSMUSG00000097464	Gm26736	predicted gene, 26736 [Source:MGI Symbol;Acc:MG	0.8

ENSMUSG00000097365	C030034L19Rik	RIKEN cDNA C030034L19 gene [Source:MGI Symbo	0.80003
ENSMUSG00000084967	Gm12296	predicted gene 12296 [Source:MGI Symbol;Acc:MG	0.80009
ENSMUSG00000094845	Tmem95	transmembrane protein 95 [Source:MGI Symbol;Ac	0.80011
ENSMUSG00000098164	Gm5493	predicted gene 5493 [Source:MGI Symbol;Acc:MGI:	0.80014
ENSMUSG00000086442	Gm11722	predicted gene 11722 [Source:MGI Symbol;Acc:MG	0.80015
ENSMUSG00000003477	Inmt	indolethylamine N-methyltransferase [Source:MGI	0.80032
ENSMUSG00000024095	Hnrnpll	heterogeneous nuclear ribonucleoprotein L-like [So	0.80035
ENSMUSG00000019942	Cdk1	cyclin-dependent kinase 1 [Source:MGI Symbol;Acc	0.8005
ENSMUSG00000019820	Utrn	utrophin [Source:MGI Symbol;Acc:MGI:104631]	0.80066
ENSMUSG00000074643	Cpne1	copine I [Source:MGI Symbol;Acc:MGI:2386621]	0.80066
ENSMUSG00000005846	Rsl1d1	ribosomal L1 domain containing 1 [Source:MGI Sym	0.80071
ENSMUSG00000036964	Trim17	tripartite motif-containing 17 [Source:MGI Symbol;	0.80071
ENSMUSG00000019518	Ap4m1	adaptor-related protein complex AP-4, mu 1 [Sourc	0.80077
ENSMUSG00000042312	S100a13	S100 calcium binding protein A13 [Source:MGI Sym	0.80083
ENSMUSG00000019579	D17Wsu104e	DNA segment, Chr 17, Wayne State University 104,	0.80089
ENSMUSG00000087541	Gm15830	predicted gene 15830 [Source:MGI Symbol;Acc:MG	0.80092
ENSMUSG00000032094	Cd3d	CD3 antigen, delta polypeptide [Source:MGI Symbo	0.80095
ENSMUSG00000082446	Gm13863	predicted gene 13863 [Source:MGI Symbol;Acc:MG	0.80095
ENSMUSG00000098031	Gm9341	predicted gene 9341 [Source:MGI Symbol;Acc:MGI:	0.80095
ENSMUSG00000048709	Gm8666	predicted gene 8666 [Source:MGI Symbol;Acc:MGI:	0.80097
ENSMUSG00000079164	Tlr5	toll-like receptor 5 [Source:MGI Symbol;Acc:MGI:18	0.80109
ENSMUSG00000071858	Gm94	predicted gene 94 [Source:MGI Symbol;Acc:MGI:26	0.80116
ENSMUSG00000031362	Xlr4c	X-linked lymphocyte-regulated 4C [Source:MGI Sym	0.80117
ENSMUSG00000027718	Il21	interleukin 21 [Source:MGI Symbol;Acc:MGI:18904	0.8012
ENSMUSG00000097870	Gm26868	predicted gene, 26868 [Source:MGI Symbol;Acc:MG	0.80124
ENSMUSG00000027233	Patl2	protein associated with topoisomerase II homolog	0.80125
ENSMUSG00000042976	9930038B18Rik	RIKEN cDNA 9930038B18 gene [Source:MGI Symbo	0.80127
ENSMUSG00000057130	Txnl4a	thioredoxin-like 4A [Source:MGI Symbol;Acc:MGI:1	0.80132
ENSMUSG00000029061	Mmp23	matrix metallopeptidase 23 [Source:MGI Symbol;A	0.80141
ENSMUSG00000097163	BC051077	cDNA sequence BC051077 [Source:MGI Symbol;Acc	0.80145
ENSMUSG00000029177	Cenpa	centromere protein A [Source:MGI Symbol;Acc:MG	0.8016
ENSMUSG00000022010	Tsc22d1	TSC22 domain family, member 1 [Source:MGI Symb	0.80167
ENSMUSG00000027574	Nkain4	Na+/K+ transporting ATPase interacting 4 [Source:M	0.80167
ENSMUSG00000032204	Aqp9	aquaporin 9 [Source:MGI Symbol;Acc:MGI:1891066	0.8017
ENSMUSG00000025428	Atp5a1	ATP synthase, H+ transporting, mitochondrial F1 co	0.80172
ENSMUSG00000027861	Casq2	calsequestrin 2 [Source:MGI Symbol;Acc:MGI:1309	0.80177
ENSMUSG00000081266	Gm15820	predicted gene 15820 [Source:MGI Symbol;Acc:MG	0.80182
ENSMUSG00000060530	A930017M01Rik	RIKEN cDNA A930017M01 gene [Source:MGI Symb	0.80196
ENSMUSG00000042626	Shc1	src homology 2 domain-containing transforming pr	0.80198
ENSMUSG00000031627	Irf2	interferon regulatory factor 2 [Source:MGI Symbol;	0.8022
ENSMUSG00000028523	Tctex1d1	Tctex1 domain containing 1 [Source:MGI Symbol;A	0.80221
ENSMUSG00000031023	Akip1	A kinase (PRKA) interacting protein 1 [Source:MGI S	0.80224
ENSMUSG00000000861	Bcl11a	B cell CLL/lymphoma 11A (zinc finger protein) [Sour	0.80226
ENSMUSG00000097885	5031434O11Rik	RIKEN cDNA 5031434O11 gene [Source:MGI Symbo	0.80228
ENSMUSG00000065765	Gm23851	predicted gene, 23851 [Source:MGI Symbol;Acc:MG	0.80232
ENSMUSG00000042842	Serpinb6b	serine (or cysteine) peptidase inhibitor, clade B, me	0.80234
ENSMUSG00000085817	Gm12668	predicted gene 12668 [Source:MGI Symbol;Acc:MG	0.80244

ENSMUSG00000082118	Gm13862	predicted gene 13862 [Source:MGI Symbol;Acc:MG	0.80254
ENSMUSG00000016758	Bik	BCL2-interacting killer [Source:MGI Symbol;Acc:MG	0.80255
ENSMUSG00000074749	Plk1s1	polo-like kinase 1 substrate 1 [Source:MGI Symbol;	0.80264
ENSMUSG00000056069	Fam105a	family with sequence similarity 105, member A [Sou	0.80274
ENSMUSG00000029440	Psmd9	proteasome (prosome, macropain) 26S subunit, no	0.80279
ENSMUSG00000083282	Ctsf	cathepsin F [Source:MGI Symbol;Acc:MGI:1861434	0.8028
ENSMUSG00000021219	Rgs6	regulator of G-protein signaling 6 [Source:MGI Sym	0.80283
ENSMUSG00000052374	Actn2	actinin alpha 2 [Source:MGI Symbol;Acc:MGI:10919	0.80284
ENSMUSG00000021223	Papln	papilin, proteoglycan-like sulfated glycoprotein [So	0.80286
ENSMUSG00000038525	Armc10	armadillo repeat containing 10 [Source:MGI Symbo	0.80291
ENSMUSG00000030879	Mrpl17	mitochondrial ribosomal protein L17 [Source:MGI S	0.80296
ENSMUSG00000045345	9530056K15Rik	RIKEN cDNA 9530056K15 gene [Source:MGI Symbo	0.8031
ENSMUSG00000045973	Slc25a51	solute carrier family 25, member 51 [Source:MGI Sy	0.80311
ENSMUSG00000047407	Tgif1	TGFB-induced factor homeobox 1 [Source:MGI Sym	0.80324
ENSMUSG00000034538	Zfp418	zinc finger protein 418 [Source:MGI Symbol;Acc:MG	0.80325
ENSMUSG00000041219	Arhgap11a	Rho GTPase activating protein 11A [Source:MGI Syr	0.80329
ENSMUSG00000023044	Csad	cysteine sulfinic acid decarboxylase [Source:MGI Sy	0.80337
ENSMUSG00000027751	Supt20	suppressor of Ty 20 [Source:MGI Symbol;Acc:MGI:1	0.8034
ENSMUSG00000025591	Tma16	translation machinery associated 16 homolog (S. ce	0.80356
ENSMUSG00000006392	Med8	mediator of RNA polymerase II transcription, subur	0.8036
ENSMUSG00000050174	Nudt6	nudix (nucleoside diphosphate linked moiety X)-typ	0.80366
ENSMUSG00000020297	Nsg2	neuron specific gene family member 2 [Source:MGI	0.80368
ENSMUSG00000030204	Ddx47	DEAD (Asp-Glu-Ala-Asp) box polypeptide 47 [Source	0.80375
ENSMUSG00000078489	Gm17106	predicted gene 17106 [Source:MGI Symbol;Acc:MG	0.80381
ENSMUSG00000072660	Gm6288	predicted gene 6288 [Source:MGI Symbol;Acc:MGI:	0.8039
ENSMUSG00000097724	Gm26850	predicted gene, 26850 [Source:MGI Symbol;Acc:MG	0.80391
ENSMUSG00000006736	Tspan31	tetraspanin 31 [Source:MGI Symbol;Acc:MGI:19143	0.80403
ENSMUSG00000026726	Cubn	cubilin (intrinsic factor-cobalamin receptor) [Source	0.80405
ENSMUSG00000058625	Gm17383	predicted gene, 17383 [Source:MGI Symbol;Acc:MG	0.80405
ENSMUSG00000062319	Gm10115	predicted gene 10115 [Source:MGI Symbol;Acc:MG	0.80405
ENSMUSG00000085335	Gm13684	predicted gene 13684 [Source:MGI Symbol;Acc:MG	0.80427
ENSMUSG00000056755	Grm7	glutamate receptor, metabotropic 7 [Source:MGI Sy	0.80433
ENSMUSG00000033044	Dhrs7c	dehydrogenase/reductase (SDR family) member 7C	0.80435
ENSMUSG00000042506	Usp22	ubiquitin specific peptidase 22 [Source:MGI Symbo	0.80436
ENSMUSG00000020169	Best3	bestrophin 3 [Source:MGI Symbol;Acc:MGI:358029	0.80441
ENSMUSG00000091475	2810468N07Rik	RIKEN cDNA 2810468N07 gene [Source:MGI Symbo	0.80451
ENSMUSG00000027304	Rtf1	Rtf1, Paf1/RNA polymerase II complex component,	0.80461
ENSMUSG00000053965	Pde5a	phosphodiesterase 5A, cGMP-specific [Source:MGI	0.80461
ENSMUSG00000010392	Gosr1	golgi SNAP receptor complex member 1 [Source:MG	0.80463
ENSMUSG00000027079	Clp1	CLP1, cleavage and polyadenylation factor I subunit	0.80465
ENSMUSG00000085846	Gm15597	predicted gene 15597 [Source:MGI Symbol;Acc:MG	0.80474
ENSMUSG00000078920	Ifi47	interferon gamma inducible protein 47 [Source:MG	0.8048
ENSMUSG00000029534	St7	suppression of tumorigenicity 7 [Source:MGI Symbo	0.80481
ENSMUSG00000054822	1700041G16Rik	RIKEN cDNA 1700041G16 gene [Source:MGI Symbo	0.80481
ENSMUSG00000087598	Zfp111	zinc finger protein 111 [Source:MGI Symbol;Acc:MG	0.80484
ENSMUSG00000015755	Tab2	TGF-beta activated kinase 1/MAP3K7 binding prote	0.80508
ENSMUSG00000030056	Isy1	ISY1 splicing factor homolog (S. cerevisiae) [Source:	0.8052

ENSMUSG00000031711	Zfp330	zinc finger protein 330 [Source:MGI Symbol;Acc:MG	0.8052
ENSMUSG00000085112	A530072M11Rik	RIKEN cDNA gene A530072M11 [Source:MGI Symbo	0.80523
ENSMUSG00000087632	Gm6058	predicted gene 6058 [Source:MGI Symbol;Acc:MGI:	0.80526
ENSMUSG00000020405	Fabp6	fatty acid binding protein 6, ileal (gastrotropin) [Sou	0.80528
ENSMUSG00000067787	Blcap	bladder cancer associated protein homolog (human	0.80533
ENSMUSG00000020263	Appl2	adaptor protein, phosphotyrosine interaction, PH d	0.80539
ENSMUSG00000039308	Ndst2	N-deacetylase/N-sulfotransferase (heparan glucosa	0.80539
ENSMUSG00000040522	Tlr8	toll-like receptor 8 [Source:MGI Symbol;Acc:MGI:21	0.8054
ENSMUSG00000034853	Acot11	acyl-CoA thioesterase 11 [Source:MGI Symbol;Acc:M	0.80542
ENSMUSG00000071665	Foxr2	forkhead box R2 [Source:MGI Symbol;Acc:MGI:351	0.80551
ENSMUSG00000069439	Gm8444	predicted gene 8444 [Source:MGI Symbol;Acc:MGI:	0.80562
ENSMUSG00000038624	Nepn	nephrocan [Source:MGI Symbol;Acc:MGI:1913900]	0.80569
ENSMUSG00000052388	Gm9874	predicted gene 9874 [Source:MGI Symbol;Acc:MGI:	0.80583
ENSMUSG00000034893	Cog3	component of oligomeric golgi complex 3 [Source:M	0.80586
ENSMUSG00000096192	Gm18856	predicted gene, 18856 [Source:MGI Symbol;Acc:MG	0.80592
ENSMUSG00000036655	Colec11	collectin sub-family member 11 [Source:MGI Symbo	0.80593
ENSMUSG00000044952	Kctd21	potassium channel tetramerisation domain contain	0.80599
ENSMUSG00000017801	Mlx	MAX-like protein X [Source:MGI Symbol;Acc:MGI:1	0.806
ENSMUSG00000086233	Gm11816	predicted gene 11816 [Source:MGI Symbol;Acc:MG	0.80601
ENSMUSG00000055421	Pcdh9	protocadherin 9 [Source:MGI Symbol;Acc:MGI:130	0.80602
ENSMUSG00000036586	Grifin	galectin-related inter-fiber protein [Source:MGI Sym	0.80604
ENSMUSG00000038422	Hdhd3	haloacid dehalogenase-like hydrolase domain conta	0.80615
ENSMUSG00000060143	Gm10076	predicted gene 10076 [Source:MGI Symbol;Acc:MG	0.80617
ENSMUSG00000042233	2010015L04Rik	RIKEN cDNA 2010015L04 gene [Source:MGI Symbo	0.80619
ENSMUSG00000051855	Mest	mesoderm specific transcript [Source:MGI Symbol;	0.8065
ENSMUSG00000042359	Osbpl6	oxysterol binding protein-like 6 [Source:MGI Symbo	0.80664
ENSMUSG00000051599	Pcdhb2	protocadherin beta 2 [Source:MGI Symbol;Acc:MGI	0.80667
ENSMUSG00000024786	1700123I01Rik	RIKEN cDNA 1700123I01 gene [Source:MGI Symbol	0.8067
ENSMUSG00000005718	Tfap4	transcription factor AP4 [Source:MGI Symbol;Acc:M	0.80676
ENSMUSG00000078933	Lrrc70	leucine rich repeat containing 70 [Source:MGI Symb	0.80682
ENSMUSG00000097217	Gm26549	predicted gene, 26549 [Source:MGI Symbol;Acc:MG	0.80688
ENSMUSG00000031853	BC021891	cDNA sequence BC021891 [Source:MGI Symbol;Acc	0.80698
ENSMUSG00000086166	Gm14342	predicted gene 14342 [Source:MGI Symbol;Acc:MG	0.80698
ENSMUSG00000019797	1700021F05Rik	RIKEN cDNA 1700021F05 gene [Source:MGI Symbo	0.80699
ENSMUSG00000087060	2810442I21Rik	RIKEN cDNA 2810442I21 gene [Source:MGI Symbol	0.80702
ENSMUSG00000041961	Znrf3	zinc and ring finger 3 [Source:MGI Symbol;Acc:MGI	0.80706
ENSMUSG00000000766	Oprm1	opioid receptor, mu 1 [Source:MGI Symbol;Acc:MG	0.80713
ENSMUSG00000070327	Rnf213	ring finger protein 213 [Source:MGI Symbol;Acc:MG	0.80726
ENSMUSG00000039115	Itga9	integrin alpha 9 [Source:MGI Symbol;Acc:MGI:1047	0.80743
ENSMUSG00000063594	Gng8	guanine nucleotide binding protein (G protein), gar	0.80747
ENSMUSG00000078384	Gm10935	predicted gene 10935 [Source:MGI Symbol;Acc:MG	0.80754
ENSMUSG00000040770	Il25	interleukin 25 [Source:MGI Symbol;Acc:MGI:21558	0.8077
ENSMUSG00000028194	Ddah1	dimethylarginine dimethylaminohydrolase 1 [Sourc	0.80776
ENSMUSG00000027601	Mtfr1	mitochondrial fission regulator 1 [Source:MGI Symb	0.80778
ENSMUSG00000037669	1110057K04Rik	RIKEN cDNA 1110057K04 gene [Source:MGI Symbo	0.80787
ENSMUSG00000066850	Vmn1r65	vomeronasal 1 receptor 65 [Source:MGI Symbol;Ac	0.80794
ENSMUSG00000076144	Mir709	microRNA 709 [Source:MGI Symbol;Acc:MGI:36297	0.80794

ENSMUSG00000001349	Cnn1	calponin 1 [Source:MGI Symbol;Acc:MGI:104979]	0.80796
ENSMUSG00000026077	Npas2	neuronal PAS domain protein 2 [Source:MGI Symbo	0.80796
ENSMUSG00000024064	Galnt14	UDP-N-acetyl-alpha-D-galactosamine:polypeptide N	0.80798
ENSMUSG00000036568	Gltscr1l	GLTSCR1-like [Source:MGI Symbol;Acc:MGI:267385	0.80801
ENSMUSG00000074457	S100a16	S100 calcium binding protein A16 [Source:MGI Sym	0.80804
ENSMUSG00000092852	Mir5103	microRNA 5103 [Source:MGI Symbol;Acc:MGI:4950	0.8081
ENSMUSG00000086399	Gm16144	predicted gene 16144 [Source:MGI Symbol;Acc:MG	0.80811
ENSMUSG00000004359	Spic	Spi-C transcription factor (Spi-1/PU.1 related) [Sour	0.80813
ENSMUSG00000086064	4930439A04Rik	RIKEN cDNA 4930439A04 gene [Source:MGI Symbo	0.80813
ENSMUSG00000044361	BC024139	cDNA sequence BC024139 [Source:MGI Symbol;Acc	0.80819
ENSMUSG00000002688	Prkd1	protein kinase D1 [Source:MGI Symbol;Acc:MGI:99	0.80834
ENSMUSG00000031770	Herpud1	homocysteine-inducible, endoplasmic reticulum str	0.80845
ENSMUSG00000039239	Tgfb2	transforming growth factor, beta 2 [Source:MGI Sy	0.80849
ENSMUSG00000083542	Olfr1379-ps1	olfactory receptor 1379, pseudogene 1 [Source:MG	0.80856
ENSMUSG00000020522	Mfap3	microfibrillar-associated protein 3 [Source:MGI Sym	0.80876
ENSMUSG00000072647	Adam1a	a disintegrin and metallopeptidase domain 1a [Sou	0.80876
ENSMUSG00000087143	A830082K12Rik	RIKEN cDNA A830082K12 gene [Source:MGI Symbo	0.80881
ENSMUSG00000041319	Thoc6	THO complex 6 homolog (Drosophila) [Source:MGI	0.80894
ENSMUSG00000076361	Mir182	microRNA 182 [Source:MGI Symbol;Acc:MGI:26768	0.8091
ENSMUSG00000026172	Bcs1l	BCS1-like (yeast) [Source:MGI Symbol;Acc:MGI:191	0.80933
ENSMUSG00000073821	8030451A03Rik	RIKEN cDNA 8030451A03 gene [Source:MGI Symbo	0.80944
ENSMUSG00000026273	Mterfd2	MTERF domain containing 2 [Source:MGI Symbol;A	0.80946
ENSMUSG00000029212	Gabrb1	gamma-aminobutyric acid (GABA) A receptor, subu	0.80947
ENSMUSG00000043340	6530409C15Rik	RIKEN cDNA 6530409C15 gene [Source:MGI Symbo	0.80949
ENSMUSG00000067547	Gm7666	predicted pseudogene 7666 [Source:MGI Symbol;A	0.80956
ENSMUSG00000004668	Abca13	ATP-binding cassette, sub-family A (ABC1), membe	0.8096
ENSMUSG00000037962	Fam101a	family with sequence similarity 101, member A [Sou	0.80967
ENSMUSG00000045466	Zfp956	zinc finger protein 956 [Source:MGI Symbol;Acc:MG	0.80974
ENSMUSG00000031974	Abcb10	ATP-binding cassette, sub-family B (MDR/TAP), mer	0.80982
ENSMUSG00000061702	Tmem91	transmembrane protein 91 [Source:MGI Symbol;Ac	0.80989
ENSMUSG00000038347	Tcte2	t-complex-associated testis expressed 2 [Source:MG	0.80995
ENSMUSG00000018263	Tbx5	T-box 5 [Source:MGI Symbol;Acc:MGI:102541]	0.81018
ENSMUSG00000024921	Smarca2	SWI/SNF related, matrix associated, actin depender	0.81034
ENSMUSG00000047786	Lix1	limb expression 1 homolog (chicken) [Source:MGI S	0.81053
ENSMUSG00000038965	Ube2l3	ubiquitin-conjugating enzyme E2L 3 [Source:MGI Sy	0.81054
ENSMUSG00000051396	Hspa14	heat shock protein 14 [Source:MGI Symbol;Acc:MG	0.8106
ENSMUSG00000021614	Vcan	versican [Source:MGI Symbol;Acc:MGI:102889]	0.81068
ENSMUSG00000079144	A130010J15Rik	RIKEN cDNA A130010J15 gene [Source:MGI Symbo	0.81069
ENSMUSG00000002732	Fkbp7	FK506 binding protein 7 [Source:MGI Symbol;Acc:M	0.81074
ENSMUSG00000027397	Slc20a1	solute carrier family 20, member 1 [Source:MGI Sym	0.81084
ENSMUSG00000070368	Prok1	prokineticin 1 [Source:MGI Symbol;Acc:MGI:21803	0.81097
ENSMUSG00000050605	Zfp61	zinc finger protein 61 [Source:MGI Symbol;Acc:MG	0.81102
ENSMUSG00000063888	Rpl7l1	ribosomal protein L7-like 1 [Source:MGI Symbol;Ac	0.81103
ENSMUSG00000079283	2310009B15Rik	RIKEN cDNA 2310009B15 gene [Source:MGI Symbo	0.81109
ENSMUSG00000038759	Nup205	nucleoporin 205 [Source:MGI Symbol;Acc:MGI:214	0.81135
ENSMUSG00000034706	Dnaic2	dynein, axonemal, intermediate chain 2 [Source:MG	0.81145
ENSMUSG00000048997	Atxn7l2	ataxin 7-like 2 [Source:MGI Symbol;Acc:MGI:19197	0.81149

ENSMUSG00000086119	Gm2415	predicted gene 2415 [Source:MGI Symbol;Acc:MGI:	0.81166
ENSMUSG00000080893	Gm15920	predicted gene 15920 [Source:MGI Symbol;Acc:MG	0.8117
ENSMUSG00000057054	Inca1	inhibitor of CDK, cyclin A1 interacting protein 1 [Sou	0.81171
ENSMUSG00000021750	Fam107a	family with sequence similarity 107, member A [Sou	0.81174
ENSMUSG00000047221	Fam185a	family with sequence similarity 185, member A [Sou	0.81183
ENSMUSG00000084786	Ubl5	ubiquitin-like 5 [Source:MGI Symbol;Acc:MGI:1913	0.81183
ENSMUSG00000080303	Gm12419	predicted gene 12419 [Source:MGI Symbol;Acc:MG	0.81185
ENSMUSG00000023908	Pkmyt1	protein kinase, membrane associated tyrosine/thre	0.81194
ENSMUSG00000097357	Gm16793	predicted gene, 16793 [Source:MGI Symbol;Acc:MG	0.81196
ENSMUSG00000093119	Gm25640	predicted gene, 25640 [Source:MGI Symbol;Acc:MG	0.81201
ENSMUSG00000055041	Commd5	COMM domain containing 5 [Source:MGI Symbol;A	0.81204
ENSMUSG00000035168	Tanc1	tetratricopeptide repeat, ankyrin repeat and coiled	0.81205
ENSMUSG00000021884	Hacl1	2-hydroxyacyl-CoA lyase 1 [Source:MGI Symbol;Acc	0.81208
ENSMUSG00000050558	Prokr2	prokineticin receptor 2 [Source:MGI Symbol;Acc:M	0.81223
ENSMUSG00000097208	Gm26698	predicted gene, 26698 [Source:MGI Symbol;Acc:MG	0.8123
ENSMUSG00000084031	Gm7416	predicted gene 7416 [Source:MGI Symbol;Acc:MGI:	0.81232
ENSMUSG00000076320	Gm26340	predicted gene, 26340 [Source:MGI Symbol;Acc:MG	0.81233
ENSMUSG00000054520	Sh3bp2	SH3-domain binding protein 2 [Source:MGI Symbol	0.81235
ENSMUSG00000020964	Sel1l	sel-1 suppressor of lin-12-like (C. elegans) [Source:M	0.8124
ENSMUSG00000093445	Lrch4	leucine-rich repeats and calponin homology (CH) do	0.8124
ENSMUSG00000049653	Spatc1	spermatogenesis and centriole associated 1 [Source	0.81242
ENSMUSG00000090186	Gm7592	predicted gene 7592 [Source:MGI Symbol;Acc:MGI:	0.81246
ENSMUSG00000083569	Gm13666	predicted gene 13666 [Source:MGI Symbol;Acc:MG	0.81247
ENSMUSG00000081642	Gm13532	predicted gene 13532 [Source:MGI Symbol;Acc:MG	0.81248
ENSMUSG00000038170	Pde4dip	phosphodiesterase 4D interacting protein (myomeg	0.81264
ENSMUSG00000001248	Gramd1a	GRAM domain containing 1A [Source:MGI Symbol;A	0.81267
ENSMUSG00000047090	Tmem198b	transmembrane protein 198b [Source:MGI Symbol;	0.81281
ENSMUSG00000047631	Apof	apolipoprotein F [Source:MGI Symbol;Acc:MGI:104	0.8129
ENSMUSG00000081865	Gm15484	predicted gene 15484 [Source:MGI Symbol;Acc:MG	0.81293
ENSMUSG00000027068	Dhrs9	dehydrogenase/reductase (SDR family) member 9 [	0.81294
ENSMUSG00000037949	Ano10	anoctamin 10 [Source:MGI Symbol;Acc:MGI:21431	0.81295
ENSMUSG00000046460	Sh2d7	SH2 domain containing 7 [Source:MGI Symbol;Acc:	0.81295
ENSMUSG00000097242	Gm16907	predicted gene, 16907 [Source:MGI Symbol;Acc:MG	0.81307
ENSMUSG00000088626	Gm23034	predicted gene, 23034 [Source:MGI Symbol;Acc:MG	0.81311
ENSMUSG00000031303	Map3k15	mitogen-activated protein kinase kinase kinase 15 [	0.81329
ENSMUSG00000038368	Focad	focadhesin [Source:MGI Symbol;Acc:MGI:2676921]	0.81338
ENSMUSG00000028106	Rprd2	regulation of nuclear pre-mRNA domain containing	0.81342
ENSMUSG00000047298	Kcnv2	potassium channel, subfamily V, member 2 [Source	0.81354
ENSMUSG00000081453	Gm6767	predicted gene 6767 [Source:MGI Symbol;Acc:MGI:	0.81358
ENSMUSG00000001018	Snapin	SNAP-associated protein [Source:MGI Symbol;Acc:M	0.81379
ENSMUSG00000097283	Gm26686	predicted gene, 26686 [Source:MGI Symbol;Acc:MG	0.81382
ENSMUSG00000025264	Tsr2	TSR2 20S rRNA accumulation [Source:MGI Symbol;A	0.81384
ENSMUSG00000032113	Chek1	checkpoint kinase 1 [Source:MGI Symbol;Acc:MGI:1	0.81394
ENSMUSG00000033845	Mrpl15	mitochondrial ribosomal protein L15 [Source:MGI S	0.81401
ENSMUSG00000082481	Rps12-ps19	ribosomal protein S12, pseudogene 19 [Source:MG	0.81405
ENSMUSG00000024831	Ighmbp2	immunoglobulin mu binding protein 2 [Source:MGI	0.81415
ENSMUSG00000075265	Gm10818	predicted gene 10818 [Source:MGI Symbol;Acc:MG	0.81415

ENSMUSG00000008658	Rbfox1	RNA binding protein, fox-1 homolog (C. elegans) 1 [	0.81418
ENSMUSG00000085282	Gm15663	predicted gene 15663 [Source:MGI Symbol;Acc:MG	0.81425
ENSMUSG00000081720	Gm12391	predicted gene 12391 [Source:MGI Symbol;Acc:MG	0.81436
ENSMUSG00000059974	Ntm	neurotrimin [Source:MGI Symbol;Acc:MGI:2446259	0.81441
ENSMUSG00000039497	Dse	dermatan sulfate epimerase [Source:MGI Symbol;A	0.81452
ENSMUSG00000024334	H2-Oa	histocompatibility 2, O region alpha locus [Source:M	0.81458
ENSMUSG00000075529	BC028471	cDNA sequence BC028471 [Source:MGI Symbol;Acc	0.81463
ENSMUSG00000029384	2010109A12Rik	RIKEN cDNA 2010109A12 gene [Source:MGI Symbo	0.81471
ENSMUSG00000022809	Nr1i2	nuclear receptor subfamily 1, group I, member 2 [S	0.81472
ENSMUSG00000027525	Phactr3	phosphatase and actin regulator 3 [Source:MGI Syn	0.81475
ENSMUSG00000085081	A430071A18Rik	RIKEN cDNA A430071A18 gene [Source:MGI Symbo	0.81476
ENSMUSG00000072612	Gm10382	predicted gene 10382 [Source:MGI Symbol;Acc:MG	0.81513
ENSMUSG00000097047	1110020A21Rik	RIKEN cDNA 1110020A21 gene [Source:MGI Symbo	0.81521
ENSMUSG00000027956	Tmem144	transmembrane protein 144 [Source:MGI Symbol;A	0.81524
ENSMUSG00000031953	Tmem170	transmembrane protein 170 [Source:MGI Symbol;A	0.81525
ENSMUSG00000081608	Gm16210	predicted gene 16210 [Source:MGI Symbol;Acc:MG	0.81528
ENSMUSG00000092741	Mir3074-1	microRNA 3074-1 [Source:MGI Symbol;Acc:MGI:48	0.81537
ENSMUSG00000085024	C230035I16Rik	RIKEN cDNA C230035I16 gene [Source:MGI Symbol	0.8154
ENSMUSG00000082544	Gm11667	predicted gene 11667 [Source:MGI Symbol;Acc:MG	0.81541
ENSMUSG00000088793	Gm22540	predicted gene, 22540 [Source:MGI Symbol;Acc:MG	0.81543
ENSMUSG00000074628	Tldc2	TBC/LysM associated domain containing 2 [Source:	0.81544
ENSMUSG00000026039	Sgol2	shugoshin-like 2 (S. pombe) [Source:MGI Symbol;Ac	0.81551
ENSMUSG00000060679	Mrps9	mitochondrial ribosomal protein S9 [Source:MGI Sy	0.8156
ENSMUSG00000029446	Psph	phosphoserine phosphatase [Source:MGI Symbol;A	0.81565
ENSMUSG00000040950	Mgl2	macrophage galactose N-acetyl-galactosamine spec	0.81586
ENSMUSG00000081136	Gm12990	predicted gene 12990 [Source:MGI Symbol;Acc:MG	0.81592
ENSMUSG00000020641	Rsad2	radical S-adenosyl methionine domain containing 2	0.81599
ENSMUSG00000054514	2610204G22Rik	RIKEN cDNA 2610204G22 gene [Source:MGI Symbo	0.816
ENSMUSG00000037736	Limch1	LIM and calponin homology domains 1 [Source:MG	0.81604
ENSMUSG00000039089	L3mbtl3	l(3)mbt-like 3 (Drosophila) [Source:MGI Symbol;Acc	0.81606
ENSMUSG00000047495	Dlgap2	discs, large (Drosophila) homolog-associated protei	0.81614
ENSMUSG00000032309	Fbxo22	F-box protein 22 [Source:MGI Symbol;Acc:MGI:192	0.81615
ENSMUSG00000046169	Adamts6	a disintegrin-like and metallopeptidase (reprolysin t	0.81616
ENSMUSG00000038671	Arfrp1	ADP-ribosylation factor related protein 1 [Source:M	0.81618
ENSMUSG00000094245	Gm23966	predicted gene, 23966 [Source:MGI Symbol;Acc:MG	0.81621
ENSMUSG00000009356	Lpo	lactoperoxidase [Source:MGI Symbol;Acc:MGI:1923	0.81624
ENSMUSG00000074895	Eif4e1b	eukaryotic translation initiation factor 4E family me	0.81626
ENSMUSG00000056912	1700017N19Rik	RIKEN cDNA 1700017N19 gene [Source:MGI Symbo	0.81627
ENSMUSG00000024905	Mtl5	metallothionein-like 5, testis-specific (tesmin) [Sour	0.81634
ENSMUSG00000052125	F730043M19Rik	RIKEN cDNA F730043M19 gene [Source:MGI Symbo	0.81636
ENSMUSG00000095871	Gm25931	predicted gene, 25931 [Source:MGI Symbol;Acc:MG	0.81639
ENSMUSG00000087403	2900056M20Rik	RIKEN cDNA 2900056M20 gene [Source:MGI Symbo	0.81646
ENSMUSG00000019952	Poc1b	POC1 centriolar protein homolog B (Chlamydomona	0.8165
ENSMUSG00000094411	Snord16a	small nucleolar RNA, C/D box 16A [Source:MGI Sym	0.81656
ENSMUSG00000034664	Itga2b	integrin alpha 2b [Source:MGI Symbol;Acc:MGI:966	0.81657
ENSMUSG00000098037	Gm7765	predicted gene 7765 [Source:MGI Symbol;Acc:MGI:	0.81658
ENSMUSG00000030546	Plin1	perilipin 1 [Source:MGI Symbol;Acc:MGI:1890505]	0.81667

ENSMUSG00000022952	Runx1	runt related transcription factor 1 [Source:MGI Sym	0.81673
ENSMUSG00000087338	4930405P13Rik	RIKEN cDNA 4930405P13 gene [Source:MGI Symbo	0.81673
ENSMUSG00000097360	9430065F17Rik	RIKEN cDNA 9430065F17 gene [Source:MGI Symbo	0.81673
ENSMUSG00000097294	Gm26888	predicted gene, 26888 [Source:MGI Symbol;Acc:M	0.81677
ENSMUSG00000029918	Mrps33	mitochondrial ribosomal protein S33 [Source:MGI S	0.81686
ENSMUSG00000085205	Gm13185	predicted gene 13185 [Source:MGI Symbol;Acc:MG	0.81694
ENSMUSG00000046807	Fam211b	family with sequence similarity 211, member B [So	0.81695
ENSMUSG00000022962	Gart	phosphoribosylglycinamide formyltransferase [Sou	0.81698
ENSMUSG00000047603	Zfp235	zinc finger protein 235 [Source:MGI Symbol;Acc:M	0.81706
ENSMUSG00000040701	Ap1g2	adaptor protein complex AP-1, gamma 2 subunit [S	0.81707
ENSMUSG00000003778	Brd8	bromodomain containing 8 [Source:MGI Symbol;Ac	0.81708
ENSMUSG00000074071	Fam169b	family with sequence similarity 169, member B [So	0.81711
ENSMUSG00000081255	Gm5380	predicted gene 5380 [Source:MGI Symbol;Acc:MGI	0.81712
ENSMUSG00000028053	Ash1l	ash1 (absent, small, or homeotic)-like (Drosophila)	0.81713
ENSMUSG00000036223	Ska1	spindle and kinetochore associated complex subun	0.81713
ENSMUSG00000078894	2210418O10Rik	RIKEN cDNA 2210418O10 gene [Source:MGI Symbo	0.81724
ENSMUSG00000089958	Gm16165	predicted gene 16165 [Source:MGI Symbol;Acc:MG	0.8173
ENSMUSG00000040621	Gemin8	gem (nuclear organelle) associated protein 8 [Sour	0.81733
ENSMUSG00000086697	4933427G23Rik	RIKEN cDNA 4933427G23 gene [Source:MGI Symbo	0.81737
ENSMUSG00000037070	Rbmxl1	RNA binding motif protein, X linked-like-1 [Source:M	0.81739
ENSMUSG00000022346	Myc	myelocytomatosis oncogene [Source:MGI Symbol;A	0.8175
ENSMUSG00000075596	B130006D01Rik	RIKEN cDNA B130006D01 gene [Source:MGI Symbo	0.81756
ENSMUSG00000077222	Gm22270	predicted gene, 22270 [Source:MGI Symbol;Acc:M	0.81759
ENSMUSG00000024812	Tjp2	tight junction protein 2 [Source:MGI Symbol;Acc:M	0.81764
ENSMUSG00000086417	Gm12996	predicted gene 12996 [Source:MGI Symbol;Acc:MG	0.81764
ENSMUSG00000020429	Igfbp1	insulin-like growth factor binding protein 1 [Source	0.81776
ENSMUSG00000078942	Naip6	NLR family, apoptosis inhibitory protein 6 [Source:M	0.81777
ENSMUSG00000087022	9130024F11Rik	RIKEN cDNA 9130024F11 gene [Source:MGI Symbo	0.81778
ENSMUSG00000074357	AA386476	expressed sequence AA386476 [Source:MGI Symbo	0.81809
ENSMUSG00000028029	Aimp1	aminoacyl tRNA synthetase complex-interacting mu	0.81812
ENSMUSG00000038437	Mllt6	myeloid/lymphoid or mixed-lineage leukemia (trith	0.81827
ENSMUSG00000097515	1700040D17Rik	RIKEN cDNA 1700040D17 gene [Source:MGI Symbo	0.81834
ENSMUSG00000097767	Miat	myocardial infarction associated transcript (non-pr	0.81835
ENSMUSG00000021962	Dcp1a	DCP1 decapping enzyme homolog A (S. cerevisiae)	0.81847
ENSMUSG00000098002	Gm9658	predicted gene 9658 [Source:MGI Symbol;Acc:MGI	0.81853
ENSMUSG00000034416	Pkd1l2	polycystic kidney disease 1 like 2 [Source:MGI Symb	0.81859
ENSMUSG00000056941	Commd7	COMM domain containing 7 [Source:MGI Symbol;A	0.81866
ENSMUSG00000041616	Nppa	natriuretic peptide type A [Source:MGI Symbol;Acc	0.81891
ENSMUSG00000024846	Cst6	cystatin E/M [Source:MGI Symbol;Acc:MGI:192097	0.81892
ENSMUSG00000015377	Dennd6b	DENN/MADD domain containing 6B [Source:MGI Sy	0.81895
ENSMUSG00000038268	Ovca2	candidate tumor suppressor in ovarian cancer 2 [So	0.81899
ENSMUSG00000019437	Tlcd1	TLC domain containing 1 [Source:MGI Symbol;Acc:M	0.81906
ENSMUSG00000075511	1700001L05Rik	RIKEN cDNA 1700001L05 gene [Source:MGI Symbo	0.8191
ENSMUSG00000021340	Gpld1	glycosylphosphatidylinositol specific phospholipase	0.81924
ENSMUSG00000025239	Limd1	LIM domains containing 1 [Source:MGI Symbol;Acc	0.81932
ENSMUSG00000078786	BC024978	cDNA sequence BC024978 [Source:MGI Symbol;Acc	0.81934
ENSMUSG00000053137	Mapk11	mitogen-activated protein kinase 11 [Source:MGI S	0.81937

ENSMUSG00000024833	Pola2	polymerase (DNA directed), alpha 2 [Source:MGI Sy	0.81939
ENSMUSG00000026766	Mmadhc	methylmalonic aciduria (cobalamin deficiency) cblD	0.81941
ENSMUSG00000053388	Trim50	tripartite motif-containing 50 [Source:MGI Symbol;	0.81949
ENSMUSG00000093026	Gm25301	predicted gene, 25301 [Source:MGI Symbol;Acc:MG	0.81958
ENSMUSG00000024910	Ctsw	cathepsin W [Source:MGI Symbol;Acc:MGI:1338045	0.81961
ENSMUSG00000027358	Bmp2	bone morphogenetic protein 2 [Source:MGI Symbo	0.81979
ENSMUSG00000031688	Pou4f2	POU domain, class 4, transcription factor 2 [Source	0.81997
ENSMUSG00000056043	Rgs9bp	regulator of G-protein signalling 9 binding protein [	0.81998
ENSMUSG00000029695	Aass	aminoadipate-semialdehyde synthase [Source:MGI	0.82002
ENSMUSG00000063646	Jakmip1	janus kinase and microtubule interacting protein 1	0.82011
ENSMUSG00000019762	Iyd	iodotyrosine deiodinase [Source:MGI Symbol;Acc:M	0.82013
ENSMUSG00000066233	Tmem42	transmembrane protein 42 [Source:MGI Symbol;Ac	0.82013
ENSMUSG00000049940	Pgrmc2	progesterone receptor membrane component 2 [So	0.82016
ENSMUSG00000097069	Gm16998	predicted gene, 16998 [Source:MGI Symbol;Acc:MG	0.82024
ENSMUSG00000006218	Fam131c	family with sequence similarity 131, member C [Sou	0.82027
ENSMUSG00000026509	Capn2	calpain 2 [Source:MGI Symbol;Acc:MGI:88264]	0.8203
ENSMUSG00000082965	Gm6754	predicted pseudogene 6754 [Source:MGI Symbol;A	0.82038
ENSMUSG00000052865	Gm13619	predicted gene 13619 [Source:MGI Symbol;Acc:MG	0.82039
ENSMUSG00000026526	Fh1	fumarate hydratase 1 [Source:MGI Symbol;Acc:MG	0.82041
ENSMUSG00000086991	Gm15334	predicted gene 15334 [Source:MGI Symbol;Acc:MG	0.82049
ENSMUSG00000066760	Psg16	pregnancy specific glycoprotein 16 [Source:MGI Syn	0.8205
ENSMUSG00000097877	Gm26703	predicted gene, 26703 [Source:MGI Symbol;Acc:MG	0.82058
ENSMUSG00000039834	Zfp335	zinc finger protein 335 [Source:MGI Symbol;Acc:MG	0.82061
ENSMUSG00000029017	Pmpcb	peptidase (mitochondrial processing) beta [Source:	0.82067
ENSMUSG00000031028	Tub	tubby candidate gene [Source:MGI Symbol;Acc:MG	0.82067
ENSMUSG00000021069	Pygl	liver glycogen phosphorylase [Source:MGI Symbol;A	0.82073
ENSMUSG00000041264	Uspl1	ubiquitin specific peptidase like 1 [Source:MGI Sym	0.82074
ENSMUSG00000042379	Esm1	endothelial cell-specific molecule 1 [Source:MGI Sy	0.82076
ENSMUSG00000027610	Gss	glutathione synthetase [Source:MGI Symbol;Acc:M	0.82097
ENSMUSG00000068566	Myadm	myeloid-associated differentiation marker [Source:	0.82101
ENSMUSG00000055945	Prr18	proline rich region 18 [Source:MGI Symbol;Acc:MG	0.82107
ENSMUSG00000051427	Ccdc157	coiled-coil domain containing 157 [Source:MGI Sym	0.82108
ENSMUSG00000048965	Mrgpre	MAS-related GPR, member E [Source:MGI Symbol;A	0.82115
ENSMUSG00000068246	Apol9b	apolipoprotein L 9b [Source:MGI Symbol;Acc:MGI:1	0.8212
ENSMUSG00000082668	Gm13258	predicted gene 13258 [Source:MGI Symbol;Acc:MG	0.82124
ENSMUSG00000084722	Gm24140	predicted gene, 24140 [Source:MGI Symbol;Acc:MG	0.82127
ENSMUSG00000055320	Tead1	TEA domain family member 1 [Source:MGI Symbol;	0.82132
ENSMUSG00000019945	1700040L02Rik	RIKEN cDNA 1700040L02 gene [Source:MGI Symbo	0.82147
ENSMUSG00000020988	L2hgdh	L-2-hydroxyglutarate dehydrogenase [Source:MGI S	0.82147
ENSMUSG00000065287	Gm24451	predicted gene, 24451 [Source:MGI Symbol;Acc:MG	0.82151
ENSMUSG00000083132	Gm14422	predicted gene 14422 [Source:MGI Symbol;Acc:MG	0.82152
ENSMUSG00000015202	Cnksr3	Cnksr family member 3 [Source:MGI Symbol;Acc:M	0.82156
ENSMUSG00000092192	Dyx1c1	dyslexia susceptibility 1 candidate 1 homolog (hum	0.82159
ENSMUSG00000037709	Fam13a	family with sequence similarity 13, member A [Sou	0.82173
ENSMUSG00000095159	Tubb4b-ps1	tubulin, beta 4B class IVB, pseudogene 1 [Source:M	0.82184
ENSMUSG00000006301	Tmbim1	transmembrane BAX inhibitor motif containing 1 [S	0.82185
ENSMUSG00000084050	Gm14439	predicted gene 14439 [Source:MGI Symbol;Acc:MG	0.82186

ENSMUSG00000020444	Guk1	guanylate kinase 1 [Source:MGI Symbol;Acc:MGI:95	0.82198
ENSMUSG00000059436	Max	Max protein [Source:MGI Symbol;Acc:MGI:96921]	0.82225
ENSMUSG00000058715	Fcer1g	Fc receptor, IgE, high affinity I, gamma polypeptide	0.82247
ENSMUSG00000051056	Gja10	gap junction protein, alpha 10 [Source:MGI Symbol	0.82252
ENSMUSG00000006717	Acot13	acyl-CoA thioesterase 13 [Source:MGI Symbol;Acc:I	0.82263
ENSMUSG00000029699	Srcrb4d	scavenger receptor cysteine rich domain containing	0.82278
ENSMUSG00000004040	Stat3	signal transducer and activator of transcription 3 [S	0.82301
ENSMUSG00000017188	Coa3	cytochrome C oxidase assembly factor 3 [Source:M	0.82311
ENSMUSG00000084729	Gm24139	predicted gene, 24139 [Source:MGI Symbol;Acc:MC	0.82323
ENSMUSG00000032705	Exd2	exonuclease 3'-5' domain containing 2 [Source:MGI	0.82328
ENSMUSG00000097423	Gm26522	predicted gene, 26522 [Source:MGI Symbol;Acc:MC	0.82328
ENSMUSG00000043531	Sorcs1	VPS10 domain receptor protein SORCS 1 [Source:M	0.82336
ENSMUSG00000031340	Gabre	gamma-aminobutyric acid (GABA) A receptor, subu	0.82341
ENSMUSG00000083409	Gm11340	predicted gene 11340 [Source:MGI Symbol;Acc:MG	0.82349
ENSMUSG00000061461	Smim20	small integral membrane protein 20 [Source:MGI S	0.82351
ENSMUSG00000005683	Cs	citrate synthase [Source:MGI Symbol;Acc:MGI:885	0.82352
ENSMUSG00000037287	Tbcel	tubulin folding cofactor E-like [Source:MGI Symbol;	0.82354
ENSMUSG00000034584	Exph5	exophilin 5 [Source:MGI Symbol;Acc:MGI:2443248]	0.82361
ENSMUSG00000089937	Gm7281	predicted gene 7281 [Source:MGI Symbol;Acc:MGI	0.82363
ENSMUSG00000079357	Gm11100	predicted gene 11100 [Source:MGI Symbol;Acc:MG	0.82367
ENSMUSG00000027313	Chac1	ChaC, cation transport regulator 1 [Source:MGI Syn	0.82368
ENSMUSG00000028385	Snx30	sorting nexin family member 30 [Source:MGI Symb	0.82368
ENSMUSG00000032314	Etfa	electron transferring flavoprotein, alpha polypeptic	0.8237
ENSMUSG00000083090	Gm11736	predicted gene 11736 [Source:MGI Symbol;Acc:MG	0.82377
ENSMUSG00000025219	Fgf8	fibroblast growth factor 8 [Source:MGI Symbol;Acc	0.82382
ENSMUSG00000050195	Scd4	stearoyl-coenzyme A desaturase 4 [Source:MGI Syr	0.82382
ENSMUSG00000058945	Gm10056	predicted gene 10056 [Source:MGI Symbol;Acc:MG	0.82388
ENSMUSG00000000031	H19	H19 fetal liver mRNA [Source:MGI Symbol;Acc:MGI	0.82389
ENSMUSG00000021431	Snrnp48	small nuclear ribonucleoprotein 48 (U11/U12) [Sou	0.82402
ENSMUSG00000080059	Rps19-ps3	ribosomal protein S19, pseudogene 3 [Source:MGI	0.82416
ENSMUSG00000072789	Gm10420	predicted gene 10420 [Source:MGI Symbol;Acc:MG	0.82419
ENSMUSG00000073752	Gm10570	predicted gene 10570 [Source:MGI Symbol;Acc:MG	0.8243
ENSMUSG00000096517	Gm6919	predicted gene 6919 [Source:MGI Symbol;Acc:MGI	0.82433
ENSMUSG00000037022	Mmaa	methylmalonic aciduria (cobalamin deficiency) type	0.82434
ENSMUSG00000062132	Gm1082	predicted gene 1082 [Source:MGI Symbol;Acc:MGI	0.82434
ENSMUSG00000050578	Mmp13	matrix metallopeptidase 13 [Source:MGI Symbol;A	0.82437
ENSMUSG00000034518	Hmgxb4	HMG box domain containing 4 [Source:MGI Symbo	0.82438
ENSMUSG00000065734	Snord49a	small nucleolar RNA, C/D box 49A [Source:MGI Sym	0.8244
ENSMUSG00000093598	A730085K08Rik	RIKEN cDNA A730085K08 gene [Source:MGI Symbo	0.82442
ENSMUSG00000072944	Nup62cl	nucleoporin 62 C-terminal like [Source:MGI Symbol	0.82443
ENSMUSG00000028167	Bdh2	3-hydroxybutyrate dehydrogenase, type 2 [Source:	0.82446
ENSMUSG00000048938	Nr1h5	nuclear receptor subfamily 1, group H, member 5 [	0.82451
ENSMUSG00000021621	Zcchc9	zinc finger, CCHC domain containing 9 [Source:MGI	0.82454
ENSMUSG00000036764	Dnajc12	DnaJ (Hsp40) homolog, subfamily C, member 12 [Sc	0.82468
ENSMUSG00000071332	E230015B07Rik	RIKEN cDNA E230015B07 gene [Source:MGI Symbo	0.82478
ENSMUSG00000046491	C1qtnf2	C1q and tumor necrosis factor related protein 2 [Sc	0.82479
ENSMUSG00000049154	Fam183b	family with sequence similarity 183, member B [Sou	0.82479

ENSMUSG00000097756	A730056A06Rik	RIKEN cDNA A730056A06 gene [Source:MGI Symbo	0.82482
ENSMUSG00000048752	Prss50	protease, serine, 50 [Source:MGI Symbol;Acc:MGI:2	0.82488
ENSMUSG00000028072	Ntrk1	neurotrophic tyrosine kinase, receptor, type 1 [Sou	0.82493
ENSMUSG00000036206	Sh3bp4	SH3-domain binding protein 4 [Source:MGI Symbol	0.82498
ENSMUSG00000041879	Ipo9	importin 9 [Source:MGI Symbol;Acc:MGI:1918944]	0.82507
ENSMUSG00000080709	Gm14324	predicted gene 14324 [Source:MGI Symbol;Acc:MG	0.82524
ENSMUSG00000022436	Sh3bp1	SH3-domain binding protein 1 [Source:MGI Symbol	0.82539
ENSMUSG00000062727	Hist1h2bk	histone cluster 1, H2bk [Source:MGI Symbol;Acc:M	0.82544
ENSMUSG00000046219	C230012O17Rik	RIKEN cDNA C230012O17 gene [Source:MGI Symbo	0.82552
ENSMUSG00000032035	Ets1	E26 avian leukemia oncogene 1, 5' domain [Source	0.82568
ENSMUSG00000085593	4930589P08Rik	RIKEN cDNA 4930589P08 gene [Source:MGI Symbo	0.82586
ENSMUSG00000052295	8030423F21Rik	RIKEN cDNA 8030423F21 gene [Source:MGI Symbo	0.82589
ENSMUSG00000058396	Gpr182	G protein-coupled receptor 182 [Source:MGI Symb	0.82602
ENSMUSG00000070034	Sp110	Sp110 nuclear body protein [Source:MGI Symbol;A	0.82608
ENSMUSG00000098143	Gm26937	predicted gene, 26937 [Source:MGI Symbol;Acc:M	0.82608
ENSMUSG00000042215	Bag2	BCL2-associated athanogene 2 [Source:MGI Symbo	0.82615
ENSMUSG00000079001	4930404H24Rik	RIKEN cDNA 4930404H24 gene [Source:MGI Symbo	0.82623
ENSMUSG00000087555	Gm13997	predicted gene 13997 [Source:MGI Symbol;Acc:MG	0.82631
ENSMUSG00000071659	Hnrnpul2	heterogeneous nuclear ribonucleoprotein U-like 2 [	0.8264
ENSMUSG00000029597	Sds	serine dehydratase [Source:MGI Symbol;Acc:MGI:9	0.82641
ENSMUSG00000056329	1810010K12Rik	RIKEN cDNA 1810010K12 gene [Source:MGI Symbo	0.82644
ENSMUSG00000081873	Gm15197	predicted gene 15197 [Source:MGI Symbol;Acc:MG	0.82659
ENSMUSG00000085276	Gm15812	predicted gene 15812 [Source:MGI Symbol;Acc:MG	0.82661
ENSMUSG00000046378	Asphd1	aspartate beta-hydroxylase domain containing 1 [S	0.82674
ENSMUSG00000041406	BC055324	cDNA sequence BC055324 [Source:MGI Symbol;Ac	0.82689
ENSMUSG00000025089	Gfra1	glial cell line derived neurotrophic factor family rec	0.82716
ENSMUSG00000046167	Gldn	gliomedin [Source:MGI Symbol;Acc:MGI:2388361]	0.82717
ENSMUSG00000028487	Bnc2	basonuclin 2 [Source:MGI Symbol;Acc:MGI:244380	0.82722
ENSMUSG00000054901	Arhgef33	Rho guanine nucleotide exchange factor (GEF) 33 [S	0.82731
ENSMUSG00000036995	Asap3	ArfGAP with SH3 domain, ankyrin repeat and PH do	0.82733
ENSMUSG00000040751	Lat2	linker for activation of T cells family, member 2 [So	0.82737
ENSMUSG00000052684	Jun	Jun oncogene [Source:MGI Symbol;Acc:MGI:96646]	0.82752
ENSMUSG00000046861	Hectd3	HECT domain containing 3 [Source:MGI Symbol;Acc	0.82758
ENSMUSG00000086279	Gm15634	predicted gene 15634 [Source:MGI Symbol;Acc:MG	0.82762
ENSMUSG00000092242	Gm20515	predicted gene 20515 [Source:MGI Symbol;Acc:MG	0.82764
ENSMUSG00000079669	Gm17396	predicted gene, 17396 [Source:MGI Symbol;Acc:M	0.82765
ENSMUSG00000029720	Gm20605	predicted gene 20605 [Source:MGI Symbol;Acc:MG	0.8277
ENSMUSG00000058594	Fbxo18	F-box protein 18 [Source:MGI Symbol;Acc:MGI:135	0.82774
ENSMUSG00000083512	Gm12749	predicted gene 12749 [Source:MGI Symbol;Acc:MG	0.82788
ENSMUSG00000045817	Zfp36l2	zinc finger protein 36, C3H type-like 2 [Source:MGI	0.8279
ENSMUSG00000005947	Itgae	integrin alpha E, epithelial-associated [Source:MGI	0.82801
ENSMUSG00000026988	Wdsub1	WD repeat, SAM and U-box domain containing 1 [S	0.82804
ENSMUSG00000045087	S1pr5	sphingosine-1-phosphate receptor 5 [Source:MGI S	0.82808
ENSMUSG00000095400	Muc2	mucin 2 [Source:MGI Symbol;Acc:MGI:1339364]	0.82808
ENSMUSG00000073415	Gm10501	predicted gene 10501 [Source:MGI Symbol;Acc:MG	0.82812
ENSMUSG00000096221	1500002C15Rik	RIKEN cDNA 1500002C15 gene [Source:MGI Symbo	0.82813
ENSMUSG00000040729	AK129341	cDNA sequence AK129341 [Source:MGI Symbol;Ac	0.82819

ENSMUSG00000082964	Rpl13-ps5	ribosomal protein L13, pseudogene 5 [Source:MGI	0.8282
ENSMUSG00000097717	1700066J03Rik	RIKEN cDNA 1700066J03 gene [Source:MGI Symbol	0.82823
ENSMUSG00000072066	6720489N17Rik	RIKEN cDNA 6720489N17 gene [Source:MGI Symbo	0.82837
ENSMUSG00000057446	Cts8	cathepsin 8 [Source:MGI Symbol;Acc:MGI:1860275	0.82849
ENSMUSG00000046550	Spin2c	spindlin family, member 2C [Source:MGI Symbol;Ac	0.8285
ENSMUSG00000047843	Bri3	brain protein I3 [Source:MGI Symbol;Acc:MGI:1933	0.82852
ENSMUSG00000026159	Agfg1	ArfGAP with FG repeats 1 [Source:MGI Symbol;Acc:	0.8286
ENSMUSG00000005107	Slc2a9	solute carrier family 2 (facilitated glucose transport	0.82861
ENSMUSG00000021025	Nfkbia	nuclear factor of kappa light polypeptide gene enha	0.82862
ENSMUSG00000040407	Akap9	A kinase (PRKA) anchor protein (yotiao) 9 [Source:M	0.82862
ENSMUSG00000029014	Dnajc2	DnaJ (Hsp40) homolog, subfamily C, member 2 [Sou	0.82863
ENSMUSG00000029082	Bst1	bone marrow stromal cell antigen 1 [Source:MGI Sy	0.82867
ENSMUSG00000029784	Ssmem1	serine-rich single-pass membrane protein 1 [Source	0.82869
ENSMUSG00000081448	Gm13086	predicted gene 13086 [Source:MGI Symbol;Acc:MG	0.8288
ENSMUSG00000053647	Gper1	G protein-coupled estrogen receptor 1 [Source:MG	0.82882
ENSMUSG00000022014	Epsti1	epithelial stromal interaction 1 (breast) [Source:MG	0.82887
ENSMUSG00000075270	Pde11a	phosphodiesterase 11A [Source:MGI Symbol;Acc:M	0.82891
ENSMUSG00000033770	Clcnka	chloride channel Ka [Source:MGI Symbol;Acc:MGI:1	0.82894
ENSMUSG00000038623	Tm6sf1	transmembrane 6 superfamily member 1 [Source:M	0.82896
ENSMUSG00000086047	9530046B11Rik	RIKEN cDNA 9530046B11 gene [Source:MGI Symbo	0.82907
ENSMUSG00000041583	Obox6	oocyte specific homeobox 6 [Source:MGI Symbol;A	0.82925
ENSMUSG00000036564	Ndrg4	N-myc downstream regulated gene 4 [Source:MGI S	0.8295
ENSMUSG00000022109	Med4	mediator of RNA polymerase II transcription, subun	0.82969
ENSMUSG00000020061	Mybpc1	myosin binding protein C, slow-type [Source:MGI Sy	0.82971
ENSMUSG00000087600	Gm14454	predicted gene 14454 [Source:MGI Symbol;Acc:MG	0.82977
ENSMUSG00000085168	Gm16252	predicted gene 16252 [Source:MGI Symbol;Acc:MG	0.82984
ENSMUSG00000003423	Pih1d1	PIH1 domain containing 1 [Source:MGI Symbol;Acc	0.82985
ENSMUSG00000046408	1700067K01Rik	RIKEN cDNA 1700067K01 gene [Source:MGI Symbo	0.82985
ENSMUSG00000032221	Mns1	meiosis-specific nuclear structural protein 1 [Sourc	0.83011
ENSMUSG00000029304	Spp1	secreted phosphoprotein 1 [Source:MGI Symbol;Ac	0.83013
ENSMUSG00000029063	Nadk	NAD kinase [Source:MGI Symbol;Acc:MGI:2183149	0.83047
ENSMUSG00000020253	Ppm1m	protein phosphatase 1M [Source:MGI Symbol;Acc:M	0.83049
ENSMUSG00000025192	Entpd7	ectonucleoside triphosphate diphosphohydrolase 7	0.83053
ENSMUSG00000026167	Wnt10a	wingless related MMTV integration site 10a [Source	0.83056
ENSMUSG00000039253	Fn3krp	fructosamine 3 kinase related protein [Source:MGI	0.83061
ENSMUSG00000075704	Txnrd2	thioredoxin reductase 2 [Source:MGI Symbol;Acc:M	0.83067
ENSMUSG00000008136	Fhl2	four and a half LIM domains 2 [Source:MGI Symbol	0.83069
ENSMUSG00000015291	Gdi1	guanosine diphosphate (GDP) dissociation inhibitor	0.83085
ENSMUSG00000027332	Ivd	isovaleryl coenzyme A dehydrogenase [Source:MGI	0.83088
ENSMUSG00000072553	Gm525	predicted gene 525 [Source:MGI Symbol;Acc:MGI:2	0.83106
ENSMUSG00000026111	Unc50	unc-50 homolog (C. elegans) [Source:MGI Symbol;A	0.83117
ENSMUSG00000085666	Gm9855	predicted pseudogene 9855 [Source:MGI Symbol;A	0.83119
ENSMUSG00000033467	Crlf2	cytokine receptor-like factor 2 [Source:MGI Symbol	0.83125
ENSMUSG00000006241	Ccdc159	coiled-coil domain containing 159 [Source:MGI Sym	0.83144
ENSMUSG00000082226	Gm715	predicted gene 715 [Source:MGI Symbol;Acc:MGI:2	0.83147
ENSMUSG00000029517	Ankrd7	ankyrin repeat domain 7 [Source:MGI Symbol;Acc:M	0.83148
ENSMUSG00000035069	Oma1	OMA1 homolog, zinc metallopeptidase (S. cerevisia	0.83158

ENSMUSG00000031488	Rab11fip1	RAB11 family interacting protein 1 (class I) [Source:	0.83159
ENSMUSG00000018395	Kif3a	kinesin family member 3A [Source:MGI Symbol;Acc	0.83164
ENSMUSG00000005615	Pcyt1a	phosphate cytidylyltransferase 1, choline, alpha iso	0.83168
ENSMUSG00000022993	4930415O20Rik	RIKEN cDNA 4930415O20 gene [Source:MGI Symbo	0.83173
ENSMUSG00000042353	Frem3	Fras1 related extracellular matrix protein 3 [Source	0.83175
ENSMUSG00000038059	Smim3	small integral membrane protein 3 [Source:MGI Sy	0.83182
ENSMUSG00000031954	Cfdp1	craniofacial development protein 1 [Source:MGI Sy	0.83186
ENSMUSG00000028608	0610037L13Rik	RIKEN cDNA 0610037L13 gene [Source:MGI Symbo	0.832
ENSMUSG00000045322	Tlr9	toll-like receptor 9 [Source:MGI Symbol;Acc:MGI:1	0.83206
ENSMUSG00000019088	Dnase1l1	deoxyribonuclease 1-like 1 [Source:MGI Symbol;Ac	0.83229
ENSMUSG00000046139	Patl1	protein associated with topoisomerase II homolog	0.83257
ENSMUSG00000032373	Car12	carbonic anyhydrase 12 [Source:MGI Symbol;Acc:M	0.83261
ENSMUSG00000029030	Tprgl	transformation related protein 63 regulated like [S	0.83265
ENSMUSG00000015133	Lrrk1	leucine-rich repeat kinase 1 [Source:MGI Symbol;A	0.83267
ENSMUSG00000014668	Chfr	checkpoint with forkhead and ring finger domains [	0.83268
ENSMUSG00000097460	Gm26734	predicted gene, 26734 [Source:MGI Symbol;Acc:M	0.83273
ENSMUSG00000087371	Gm15541	predicted gene 15541 [Source:MGI Symbol;Acc:MG	0.833
ENSMUSG00000055850	Rnf181	ring finger protein 181 [Source:MGI Symbol;Acc:M	0.8331
ENSMUSG00000091583	Gm17033	predicted gene 17033 [Source:MGI Symbol;Acc:MG	0.83321
ENSMUSG00000027030	Stk39	serine/threonine kinase 39 [Source:MGI Symbol;Ac	0.8334
ENSMUSG00000082088	Gm15753	predicted gene 15753 [Source:MGI Symbol;Acc:MG	0.83356
ENSMUSG00000037762	Slc16a9	solute carrier family 16 (monocarboxylic acid trans	0.83357
ENSMUSG00000029311	Hsd17b11	hydroxysteroid (17-beta) dehydrogenase 11 [Sourc	0.83364
ENSMUSG00000062694	Cav3	caveolin 3 [Source:MGI Symbol;Acc:MGI:107570]	0.83376
ENSMUSG00000028369	Svep1	sushi, von Willebrand factor type A, EGF and pentra	0.83379
ENSMUSG00000090523	Gypc	glycophorin C [Source:MGI Symbol;Acc:MGI:10985	0.83379
ENSMUSG00000003032	Klf4	Kruppel-like factor 4 (gut) [Source:MGI Symbol;Acc	0.83381
ENSMUSG00000040389	Wdr47	WD repeat domain 47 [Source:MGI Symbol;Acc:MG	0.83382
ENSMUSG00000030677	Kif22	kinesin family member 22 [Source:MGI Symbol;Acc	0.83387
ENSMUSG00000040613	Apobec1	apolipoprotein B mRNA editing enzyme, catalytic p	0.83398
ENSMUSG00000087538	Gm16341	predicted gene 16341 [Source:MGI Symbol;Acc:MG	0.83399
ENSMUSG00000038530	Rgs4	regulator of G-protein signaling 4 [Source:MGI Sym	0.83406
ENSMUSG00000031397	Tktl1	transketolase-like 1 [Source:MGI Symbol;Acc:MGI:1	0.83407
ENSMUSG00000036330	Slc18a1	solute carrier family 18 (vesicular monoamine), me	0.83425
ENSMUSG00000088338	Gm22849	predicted gene, 22849 [Source:MGI Symbol;Acc:M	0.83451
ENSMUSG00000028159	Dapp1	dual adaptor for phosphotyrosine and 3-phosphoin	0.83452
ENSMUSG00000041133	Smc1a	structural maintenance of chromosomes 1A [Sourc	0.83453
ENSMUSG00000038204	Asb10	ankyrin repeat and SOCS box-containing 10 [Source	0.83459
ENSMUSG00000041372	B4galnt3	beta-1,4-N-acetyl-galactosaminyl transferase 3 [So	0.83462
ENSMUSG00000041702	Btbd7	BTB (POZ) domain containing 7 [Source:MGI Symbo	0.83462
ENSMUSG00000001380	Hars	histidyl-tRNA synthetase [Source:MGI Symbol;Acc:	0.83466
ENSMUSG00000027984	Hadh	hydroxyacyl-Coenzyme A dehydrogenase [Source:M	0.83468
ENSMUSG00000087480	Gm15910	predicted gene 15910 [Source:MGI Symbol;Acc:MG	0.83474
ENSMUSG00000030144	Clec4d	C-type lectin domain family 4, member d [Source:M	0.83491
ENSMUSG00000025353	Ormdl2	ORM1-like 2 (S. cerevisiae) [Source:MGI Symbol;Ac	0.83496
ENSMUSG00000056184	Olfr283	olfactory receptor 283 [Source:MGI Symbol;Acc:M	0.83496
ENSMUSG00000059089	Fcgr4	Fc receptor, IgG, low affinity IV [Source:MGI Symbo	0.83496

ENSMUSG00000089645	Gm5766	predicted gene 5766 [Source:MGI Symbol;Acc:MGI:	0.83502
ENSMUSG00000038848	Ythdf1	YTH domain family 1 [Source:MGI Symbol;Acc:MGI:	0.83521
ENSMUSG00000033065	Pfkm	phosphofructokinase, muscle [Source:MGI Symbol;	0.83522
ENSMUSG00000061533	Cep128	centrosomal protein 128 [Source:MGI Symbol;Acc:I	0.83524
ENSMUSG00000016946	Kctd5	potassium channel tetramerisation domain contain	0.83534
ENSMUSG00000026149	Tm4sf20	transmembrane 4 L six family member 20 [Source:I	0.83542
ENSMUSG00000057580	Gm10012	predicted pseudogene 10012 [Source:MGI Symbol;	0.83543
ENSMUSG00000083832	Gm13771	predicted gene 13771 [Source:MGI Symbol;Acc:MG	0.83544
ENSMUSG00000096623	Gm8112	predicted gene 8112 [Source:MGI Symbol;Acc:MGI:	0.83544
ENSMUSG00000047565	Acot10	acyl-CoA thioesterase 10 [Source:MGI Symbol;Acc:I	0.83545
ENSMUSG00000036131	Frmd7	FERM domain containing 7 [Source:MGI Symbol;Ac	0.83546
ENSMUSG00000038011	Dnah10	dynein, axonemal, heavy chain 10 [Source:MGI Sym	0.83548
ENSMUSG00000078238	Gm12854	predicted gene 12854 [Source:MGI Symbol;Acc:MG	0.83553
ENSMUSG00000097298	A130057D12Rik	RIKEN cDNA A130057D12 gene [Source:MGI Symbo	0.83553
ENSMUSG00000037860	Aim2	absent in melanoma 2 [Source:MGI Symbol;Acc:MG	0.83555
ENSMUSG00000063816	Gm4987	predicted gene 4987 [Source:MGI Symbol;Acc:MGI:	0.83555
ENSMUSG00000070778	Rpl7a-ps8	ribosomal protein L7A, pseudogene 8 [Source:MGI	0.8356
ENSMUSG00000025465	Echs1	enoyl Coenzyme A hydratase, short chain, 1, mitoch	0.83568
ENSMUSG00000098235	Gm7497	predicted gene 7497 [Source:MGI Symbol;Acc:MGI:	0.83569
ENSMUSG00000090387	Gm17056	predicted gene 17056 [Source:MGI Symbol;Acc:MG	0.83571
ENSMUSG00000020319	Wdpcp	WD repeat containing planar cell polarity effector [	0.83595
ENSMUSG00000086648	Gm15511	predicted gene 15511 [Source:MGI Symbol;Acc:MG	0.83607
ENSMUSG00000033717	Adra2a	adrenergic receptor, alpha 2a [Source:MGI Symbol;	0.8361
ENSMUSG00000043807	Ly6g5b	lymphocyte antigen 6 complex, locus G5B [Source:I	0.8362
ENSMUSG00000033307	Mif	macrophage migration inhibitory factor [Source:MG	0.83629
ENSMUSG00000084632	Gm23887	predicted gene, 23887 [Source:MGI Symbol;Acc:MG	0.83646
ENSMUSG00000022629	Kif21a	kinesin family member 21A [Source:MGI Symbol;Ac	0.8366
ENSMUSG00000078627	10-Mar	membrane-associated ring finger (C3HC4) 10 [Sour	0.83668
ENSMUSG00000086680	Gm11592	predicted gene 11592 [Source:MGI Symbol;Acc:MG	0.83688
ENSMUSG00000078941	Taf9	TAF9 RNA polymerase II, TATA box binding protein	0.83693
ENSMUSG00000020323	Prss57	protease, serine 57 [Source:MGI Symbol;Acc:MGI:1	0.83695
ENSMUSG00000089780	Gm16306	predicted gene 16306 [Source:MGI Symbol;Acc:MG	0.83701
ENSMUSG00000045509	Gpr150	G protein-coupled receptor 150 [Source:MGI Symb	0.83702
ENSMUSG00000027761	Aadac	arylacetamide deacetylase (esterase) [Source:MGI S	0.83703
ENSMUSG00000056476	Med12l	mediator of RNA polymerase II transcription, subun	0.83704
ENSMUSG00000036875	Dna2	DNA replication helicase 2 homolog (yeast) [Source	0.83714
ENSMUSG00000068615	Gjd2	gap junction protein, delta 2 [Source:MGI Symbol;A	0.83714
ENSMUSG00000063314	Gm12657	predicted gene 12657 [Source:MGI Symbol;Acc:MG	0.8372
ENSMUSG00000057596	Trim30d	tripartite motif-containing 30D [Source:MGI Symbo	0.83721
ENSMUSG00000088913	Gm22611	predicted gene, 22611 [Source:MGI Symbol;Acc:MG	0.83724
ENSMUSG00000059336	Slc14a1	solute carrier family 14 (urea transporter), member	0.83741
ENSMUSG00000056820	Tsnax	translin-associated factor X [Source:MGI Symbol;Ac	0.83748
ENSMUSG00000015354	Pcolce2	procollagen C-endopeptidase enhancer 2 [Source:M	0.8375
ENSMUSG00000044847	Lsm11	U7 snRNP-specific Sm-like protein LSM11 [Source:M	0.8375
ENSMUSG00000020928	Higd1b	HIG1 domain family, member 1B [Source:MGI Symb	0.83755
ENSMUSG00000025702	8-Mar	membrane-associated ring finger (C3HC4) 8 [Source	0.83767
ENSMUSG00000005836	Gata6	GATA binding protein 6 [Source:MGI Symbol;Acc:M	0.83768

ENSMUSG00000028773	Fabp3	fatty acid binding protein 3, muscle and heart [Sour	0.83776
ENSMUSG00000003464	Pex19	peroxisomal biogenesis factor 19 [Source:MGI Sym	0.83777
ENSMUSG00000014606	Slc25a11	solute carrier family 25 (mitochondrial carrier oxog	0.83786
ENSMUSG00000038152	5033430I15Rik	RIKEN cDNA 5033430I15 gene [Source:MGI Symbol	0.83786
ENSMUSG00000034394	Lif	leukemia inhibitory factor [Source:MGI Symbol;Acc	0.83792
ENSMUSG00000097580	Gm5432	predicted gene 5432 [Source:MGI Symbol;Acc:MGI:	0.83794
ENSMUSG00000039629	Strip2	striatin interacting protein 2 [Source:MGI Symbol;A	0.83795
ENSMUSG00000035351	Nup37	nucleoporin 37 [Source:MGI Symbol;Acc:MGI:1919	0.83796
ENSMUSG00000041688	Amot	angiomotin [Source:MGI Symbol;Acc:MGI:108440]	0.83797
ENSMUSG00000050954	Zfp169	zinc finger protein 169 [Source:MGI Symbol;Acc:M	0.83797
ENSMUSG00000066975	Cryba4	crystallin, beta A4 [Source:MGI Symbol;Acc:MGI:10	0.83801
ENSMUSG00000040345	Arhgap9	Rho GTPase activating protein 9 [Source:MGI Symb	0.83811
ENSMUSG00000024338	Psmb8	proteasome (prosome, macropain) subunit, beta ty	0.83833
ENSMUSG00000021720	Rnf180	ring finger protein 180 [Source:MGI Symbol;Acc:M	0.83838
ENSMUSG00000051220	Ercc6l	excision repair cross-complementing rodent repair	0.83838
ENSMUSG00000086891	Gm15963	predicted gene 15963 [Source:MGI Symbol;Acc:MG	0.83842
ENSMUSG00000045326	Fndc7	fibronectin type III domain containing 7 [Source:M	0.83844
ENSMUSG00000038676	Ucn	urocortin [Source:MGI Symbol;Acc:MGI:1276123]	0.83848
ENSMUSG00000034035	Ccdc17	coiled-coil domain containing 17 [Source:MGI Symb	0.83852
ENSMUSG00000084880	Gm14872	predicted gene 14872 [Source:MGI Symbol;Acc:MG	0.83852
ENSMUSG00000022580	Rhpn1	rhophilin, Rho GTPase binding protein 1 [Source:M	0.83855
ENSMUSG00000086893	Gm11365	predicted gene 11365 [Source:MGI Symbol;Acc:MG	0.83856
ENSMUSG00000025497	Cdhr5	cadherin-related family member 5 [Source:MGI Syn	0.83857
ENSMUSG00000088901	Mir1943	microRNA 1943 [Source:MGI Symbol;Acc:MGI:3836	0.83857
ENSMUSG00000026432	Avpr1b	arginine vasopressin receptor 1B [Source:MGI Symb	0.83858
ENSMUSG00000097422	Gm26521	predicted gene, 26521 [Source:MGI Symbol;Acc:M	0.83859
ENSMUSG00000025355	Mmp19	matrix metallopeptidase 19 [Source:MGI Symbol;A	0.83881
ENSMUSG00000025732	Fam195a	family with sequence similarity 195, member A [So	0.83882
ENSMUSG00000041354	Rgl2	ral guanine nucleotide dissociation stimulator-like 2	0.83886
ENSMUSG00000041440	Gk5	glycerol kinase 5 (putative) [Source:MGI Symbol;Ac	0.83906
ENSMUSG00000026437	Cdk18	cyclin-dependent kinase 18 [Source:MGI Symbol;Ac	0.83917
ENSMUSG00000013936	Myl2	myosin, light polypeptide 2, regulatory, cardiac, slo	0.83923
ENSMUSG00000075416	Gm14488	predicted gene 14488 [Source:MGI Symbol;Acc:MG	0.83929
ENSMUSG00000018427	Ypel2	yippee-like 2 (Drosophila) [Source:MGI Symbol;Acc	0.83931
ENSMUSG00000021810	Ecd	ecdysoneless homolog (Drosophila) [Source:MGI Sy	0.83941
ENSMUSG00000086753	Gm15751	predicted gene 15751 [Source:MGI Symbol;Acc:MG	0.83942
ENSMUSG00000071586	Gm10337	predicted gene 10337 [Source:MGI Symbol;Acc:MG	0.83943
ENSMUSG00000086965	Gm16314	predicted gene 16314 [Source:MGI Symbol;Acc:MG	0.83955
ENSMUSG00000036983	Tfb1m	transcription factor B1, mitochondrial [Source:MGI	0.83958
ENSMUSG00000037536	Fbxo34	F-box protein 34 [Source:MGI Symbol;Acc:MGI:192	0.83969
ENSMUSG00000097075	D830044I16Rik	RIKEN cDNA D830044I16 gene [Source:MGI Symbol	0.83978
ENSMUSG00000083962	Gm15707	predicted gene 15707 [Source:MGI Symbol;Acc:MG	0.83981
ENSMUSG00000097353	A430046D13Rik	Riken cDNA A430046D13 gene [Source:MGI Symbo	0.83987
ENSMUSG00000039114	Nrn1	neuritin 1 [Source:MGI Symbol;Acc:MGI:1915654]	0.83997
ENSMUSG00000005609	Ctr9	Ctr9, Paf1/RNA polymerase II complex component,	0.84009
ENSMUSG00000056735	A930024E05Rik	RIKEN cDNA A930024E05 gene [Source:MGI Symbo	0.84011
ENSMUSG00000031698	Mylk3	myosin light chain kinase 3 [Source:MGI Symbol;Ac	0.84014

ENSMUSG00000063875	Rps6-ps1	ribosomal protein S6, pseudogene 1 [Source:MGI S	0.84016
ENSMUSG00000016494	Cd34	CD34 antigen [Source:MGI Symbol;Acc:MGI:88329]	0.8403
ENSMUSG00000093629	Gm19357	predicted gene, 19357 [Source:MGI Symbol;Acc:MG	0.84048
ENSMUSG00000021573	Tppp	tubulin polymerization promoting protein [Source:I	0.84072
ENSMUSG00000037393	Nmur2	neuromedin U receptor 2 [Source:MGI Symbol;Acc:	0.84083
ENSMUSG00000097403	9230116N13Rik	RIKEN cDNA 9230116N13 gene [Source:MGI Symbo	0.84089
ENSMUSG00000043969	Emx2	empty spiracles homeobox 2 [Source:MGI Symbol;A	0.84091
ENSMUSG00000089834	Gm16303	predicted gene 16303 [Source:MGI Symbol;Acc:MG	0.84095
ENSMUSG00000021287	Xrcc3	X-ray repair complementing defective repair in Chir	0.84098
ENSMUSG00000096945	Gm26546	predicted gene, 26546 [Source:MGI Symbol;Acc:MG	0.84099
ENSMUSG00000027863	Cd2	CD2 antigen [Source:MGI Symbol;Acc:MGI:88320]	0.84104
ENSMUSG00000024979	Tectb	tectorin beta [Source:MGI Symbol;Acc:MGI:109574	0.84113
ENSMUSG00000067215	Usp51	ubiquitin specific protease 51 [Source:MGI Symbol;	0.84123
ENSMUSG00000050511	Oprd1	opioid receptor, delta 1 [Source:MGI Symbol;Acc:M	0.84144
ENSMUSG00000001999	Blvra	biliverdin reductase A [Source:MGI Symbol;Acc:MG	0.84145
ENSMUSG00000026062	Slc9a2	solute carrier family 9 (sodium/hydrogen exchange	0.84145
ENSMUSG00000024425	Ndfip1	Nedd4 family interacting protein 1 [Source:MGI Syr	0.84165
ENSMUSG00000042489	Clspn	claspin [Source:MGI Symbol;Acc:MGI:2445153]	0.8417
ENSMUSG00000027469	Tpx2	TPX2, microtubule-associated protein homolog (Xe	0.84177
ENSMUSG00000054568	Usp17la	ubiquitin specific peptidase 17-like A [Source:MGI S	0.84182
ENSMUSG00000020515	Cnot8	CCR4-NOT transcription complex, subunit 8 [Source	0.84183
ENSMUSG00000061742	Slc22a12	solute carrier family 22 (organic anion/cation trans	0.84183
ENSMUSG00000035509	Fbxl21	F-box and leucine-rich repeat protein 21 [Source:M	0.84193
ENSMUSG00000027196	4921507L20Rik	RIKEN cDNA 4921507L20 gene [Source:MGI Symbo	0.84197
ENSMUSG00000071203	Naip5	NLR family, apoptosis inhibitory protein 5 [Source:N	0.84198
ENSMUSG00000032172	Olfm2	olfactomedin 2 [Source:MGI Symbol;Acc:MGI:3045	0.84207
ENSMUSG00000083773	Gm13394	predicted gene 13394 [Source:MGI Symbol;Acc:MG	0.84215
ENSMUSG00000044937	BC030307	cDNA sequence BC030307 [Source:MGI Symbol;Acc	0.84225
ENSMUSG00000024644	Cndp2	CNDP dipeptidase 2 (metallopeptidase M20 family)	0.84226
ENSMUSG00000089628	Gm9727	predicted gene 9727 [Source:MGI Symbol;Acc:MGI:	0.84226
ENSMUSG00000037016	Frem2	Fras1 related extracellular matrix protein 2 [Source	0.84232
ENSMUSG00000080773	Gm12955	predicted gene 12955 [Source:MGI Symbol;Acc:MG	0.84232
ENSMUSG00000087461	C230014O12Rik	RIKEN cDNA C230014O12 gene [Source:MGI Symbo	0.84234
ENSMUSG00000071713	Csf2rb	colony stimulating factor 2 receptor, beta, low-affir	0.84244
ENSMUSG00000097970	Gm27028	predicted gene, 27028 [Source:MGI Symbol;Acc:MG	0.84244
ENSMUSG00000044794	9330133O14Rik	RIKEN cDNA 9330133O14 gene [Source:MGI Symbo	0.84248
ENSMUSG00000092241	Gm20522	predicted gene 20522 [Source:MGI Symbol;Acc:MG	0.84253
ENSMUSG00000080951	Gm12208	predicted gene 12208 [Source:MGI Symbol;Acc:MG	0.8426
ENSMUSG00000074682	Zcchc3	zinc finger, CCHC domain containing 3 [Source:MGI	0.84273
ENSMUSG00000032034	Kcnj5	potassium inwardly-rectifying channel, subfamily J,	0.84275
ENSMUSG00000033970	Rfc3	replication factor C (activator 1) 3 [Source:MGI Sym	0.84282
ENSMUSG00000064377	Gm24966	predicted gene, 24966 [Source:MGI Symbol;Acc:MG	0.84282
ENSMUSG00000028334	Nans	N-acetylneuraminic acid synthase (sialic acid syntha	0.84286
ENSMUSG00000010607	Pigyl	phosphatidylinositol glycan anchor biosynthesis, cla	0.84295
ENSMUSG00000082625	Gm15204	predicted gene 15204 [Source:MGI Symbol;Acc:MG	0.84296
ENSMUSG00000028807	Zbtb8a	zinc finger and BTB domain containing 8a [Source:N	0.84299
ENSMUSG00000045877	4933415A04Rik	RIKEN cDNA 4933415A04 gene [Source:MGI Symbo	0.84308

ENSMUSG00000049354	Dcaf7	DDB1 and CUL4 associated factor 7 [Source:MGI Sy	0.8432
ENSMUSG00000074232	Gm10647	predicted gene 10647 [Source:MGI Symbol;Acc:MG	0.84328
ENSMUSG00000093886	Gm23158	predicted gene, 23158 [Source:MGI Symbol;Acc:M(	0.84328
ENSMUSG00000034324	Tmem132c	transmembrane protein 132C [Source:MGI Symbol;	0.84344
ENSMUSG00000070448	Vmn2r89	vomeronasal 2, receptor 89 [Source:MGI Symbol;A	0.84348
ENSMUSG00000072893	4933439C10Rik	RIKEN cDNA 4933439C10 gene [Source:MGI Symbo	0.84353
ENSMUSG00000089343	Gm22667	predicted gene, 22667 [Source:MGI Symbol;Acc:M(	0.84355
ENSMUSG00000081499	Gm12517	predicted gene 12517 [Source:MGI Symbol;Acc:MG	0.84359
ENSMUSG00000033701	Acbd6	acyl-Coenzyme A binding domain containing 6 [Sou	0.84366
ENSMUSG00000030724	Cd19	CD19 antigen [Source:MGI Symbol;Acc:MGI:88319]	0.84391
ENSMUSG00000023206	Il15ra	interleukin 15 receptor, alpha chain [Source:MGI Sy	0.844
ENSMUSG00000021140	Pcnx	pecanex homolog (Drosophila) [Source:MGI Symbo	0.84401
ENSMUSG00000090667	Gm765	predicted gene 765 [Source:MGI Symbol;Acc:MGI:2	0.84411
ENSMUSG00000097845	A230020J21Rik	RIKEN cDNA A230020J21 gene [Source:MGI Symbo	0.84416
ENSMUSG00000069744	Psmb3	proteasome (prosome, macropain) subunit, beta ty	0.84417
ENSMUSG00000090008	Gm16111	predicted gene 16111 [Source:MGI Symbol;Acc:MG	0.84417
ENSMUSG00000042787	Exog	endo/exonuclease (5'-3'), endonuclease G-like [Sou	0.84432
ENSMUSG00000032477	Cdc25a	cell division cycle 25A [Source:MGI Symbol;Acc:MG	0.84434
ENSMUSG00000028234	Rps20	ribosomal protein S20 [Source:MGI Symbol;Acc:MG	0.84449
ENSMUSG00000081861	Gm14441	predicted gene 14441 [Source:MGI Symbol;Acc:MG	0.84455
ENSMUSG00000020007	Il20ra	interleukin 20 receptor, alpha [Source:MGI Symbol;	0.84478
ENSMUSG00000079507	H2-Q1	histocompatibility 2, Q region locus 1 [Source:MGI S	0.84494
ENSMUSG00000032415	Ube2cbp	ubiquitin-conjugating enzyme E2C binding protein [	0.84511
ENSMUSG00000038009	Dnajc22	DnaJ (Hsp40) homolog, subfamily C, member 22 [So	0.84512
ENSMUSG00000046031	Fam26f	family with sequence similarity 26, member F [Sour	0.84518
ENSMUSG00000073674	Gm6644	predicted gene 6644 [Source:MGI Symbol;Acc:MGI:	0.84526
ENSMUSG00000027210	Meis2	Meis homeobox 2 [Source:MGI Symbol;Acc:MGI:10	0.84532
ENSMUSG00000074884	Serf2	small EDRK-rich factor 2 [Source:MGI Symbol;Acc:M	0.84542
ENSMUSG00000094083	Gm1604A	predicted gene 1604A [Source:MGI Symbol;Acc:MG	0.84544
ENSMUSG00000001687	Ubl3	ubiquitin-like 3 [Source:MGI Symbol;Acc:MGI:1344	0.84546
ENSMUSG00000033029	1700088E04Rik	RIKEN cDNA 1700088E04 gene [Source:MGI Symbo	0.84557
ENSMUSG00000043183	Simc1	SUMO-interacting motifs containing 1 [Source:MGI	0.84559
ENSMUSG00000038390	Gpr162	G protein-coupled receptor 162 [Source:MGI Symb	0.84561
ENSMUSG00000029144	Gtf3c2	general transcription factor IIIC, polypeptide 2, beta	0.84566
ENSMUSG00000006649	Nphs1	nephrosis 1, nephrin [Source:MGI Symbol;Acc:MGI:	0.84572
ENSMUSG00000075395	A630010A05Rik	RIKEN cDNA A630010A05 gene [Source:MGI Symbo	0.84574
ENSMUSG00000028007	Snx7	sorting nexin 7 [Source:MGI Symbol;Acc:MGI:1923	0.84582
ENSMUSG00000028032	Papss1	3'-phosphoadenosine 5'-phosphosulfate synthase 1	0.84593
ENSMUSG00000069713	4933406P04Rik	RIKEN cDNA 4933406P04 gene [Source:MGI Symbo	0.84601
ENSMUSG00000012282	Wnt8a	wingless-related MMTV integration site 8A [Source	0.84605
ENSMUSG00000050147	F2rl3	coagulation factor II (thrombin) receptor-like 3 [Sou	0.84605
ENSMUSG00000033565	Rbfox2	RNA binding protein, fox-1 homolog (C. elegans) 2 [	0.84617
ENSMUSG00000025268	Maged2	melanoma antigen, family D, 2 [Source:MGI Symbo	0.84623
ENSMUSG00000018845	Unc45b	unc-45 homolog B (C. elegans) [Source:MGI Symbo	0.84634
ENSMUSG00000027303	Ptpra	protein tyrosine phosphatase, receptor type, A [Sou	0.84648
ENSMUSG00000030703	Gdpd3	glycerophosphodiester phosphodiesterase domain	0.84667
ENSMUSG00000021176	Efcab11	EF-hand calcium binding domain 11 [Source:MGI Sy	0.8467

ENSMUSG00000086688	Gm11560	predicted gene 11560 [Source:MGI Symbol;Acc:MG	0.84683
ENSMUSG00000097325	Gm16897	predicted gene, 16897 [Source:MGI Symbol;Acc:M(	0.84684
ENSMUSG00000030159	Clec1b	C-type lectin domain family 1, member b [Source:M	0.84688
ENSMUSG00000028876	Epha10	Eph receptor A10 [Source:MGI Symbol;Acc:MGI:35	0.84692
ENSMUSG00000005611	Mrvi1	MRV integration site 1 [Source:MGI Symbol;Acc:M(	0.84701
ENSMUSG00000086798	Gm16222	predicted gene 16222 [Source:MGI Symbol;Acc:MG	0.84702
ENSMUSG00000087638	Gm11773	predicted gene 11773 [Source:MGI Symbol;Acc:MG	0.84702
ENSMUSG00000084869	Gm13063	predicted gene 13063 [Source:MGI Symbol;Acc:MG	0.84717
ENSMUSG00000048439	Nupl2	nucleoporin like 2 [Source:MGI Symbol;Acc:MGI:23	0.84724
ENSMUSG00000039960	Rhou	ras homolog gene family, member U [Source:MGI S	0.84727
ENSMUSG00000082507	Gm9378	predicted gene 9378 [Source:MGI Symbol;Acc:MGI:	0.84737
ENSMUSG00000096014	Sox1	SRY-box containing gene 1 [Source:MGI Symbol;Acc	0.84742
ENSMUSG00000036114	Rpp25l	ribonuclease P/MRP 25 subunit-like [Source:MGI Sy	0.84752
ENSMUSG00000033436	Armcx2	armadillo repeat containing, X-linked 2 [Source:MG	0.84762
ENSMUSG00000028629	Exo5	exonuclease 5 [Source:MGI Symbol;Acc:MGI:19204	0.84771
ENSMUSG00000086421	Gm14091	predicted gene 14091 [Source:MGI Symbol;Acc:MG	0.84772
ENSMUSG00000029735	Tpk1	thiamine pyrophosphokinase [Source:MGI Symbol;/	0.84774
ENSMUSG00000048833	Slc39a9	solute carrier family 39 (zinc transporter), member	0.84782
ENSMUSG00000087605	Gm15742	predicted gene 15742 [Source:MGI Symbol;Acc:MG	0.84786
ENSMUSG00000058729	Lin9	lin-9 homolog (C. elegans) [Source:MGI Symbol;Acc	0.84788
ENSMUSG00000047638	Nr1h4	nuclear receptor subfamily 1, group H, member 4 [	0.84792
ENSMUSG00000005534	Insr	insulin receptor [Source:MGI Symbol;Acc:MGI:9657	0.84819
ENSMUSG00000073468	Sft2d1	SFT2 domain containing 1 [Source:MGI Symbol;Acc	0.84822
ENSMUSG00000021594	Srd5a1	steroid 5 alpha-reductase 1 [Source:MGI Symbol;Ac	0.84833
ENSMUSG00000046580	Gm7862	predicted gene 7862 [Source:MGI Symbol;Acc:MGI:	0.84846
ENSMUSG00000024486	Hbegf	heparin-binding EGF-like growth factor [Source:MG	0.84849
ENSMUSG00000090087	Gm15939	predicted gene 15939 [Source:MGI Symbol;Acc:MG	0.8486
ENSMUSG00000097472	Gm26586	predicted gene, 26586 [Source:MGI Symbol;Acc:M(	0.8486
ENSMUSG00000027831	Veph1	ventricular zone expressed PH domain-containing 1	0.84879
ENSMUSG00000087272	Gm15409	predicted gene 15409 [Source:MGI Symbol;Acc:MG	0.84886
ENSMUSG00000000282	Mnt	max binding protein [Source:MGI Symbol;Acc:MGI:	0.84889
ENSMUSG00000029916	Agk	acylglycerol kinase [Source:MGI Symbol;Acc:MGI:1	0.84894
ENSMUSG00000024885	Aldh3b1	aldehyde dehydrogenase 3 family, member B1 [Sou	0.84896
ENSMUSG00000021636	Marveld2	MARVEL (membrane-associating) domain containin	0.84898
ENSMUSG00000042121	Ssh1	slingshot homolog 1 (Drosophila) [Source:MGI Sym	0.849
ENSMUSG00000070972	Dnajc25	DnaJ (Hsp40) homolog, subfamily C, member 25 [Sc	0.84912
ENSMUSG00000087041	Gm16295	predicted gene 16295 [Source:MGI Symbol;Acc:MG	0.84916
ENSMUSG00000026174	Rqcd1	rcd1 (required for cell differentiation) homolog 1 (S	0.8492
ENSMUSG00000065273	Gm25128	predicted gene, 25128 [Source:MGI Symbol;Acc:M(	0.84925
ENSMUSG00000081401	Gm14889	predicted gene 14889 [Source:MGI Symbol;Acc:MG	0.84925
ENSMUSG00000043993	2900052L18Rik	RIKEN cDNA 2900052L18 gene [Source:MGI Symbo	0.84926
ENSMUSG00000092903	Gm25734	predicted gene, 25734 [Source:MGI Symbol;Acc:M(	0.84927
ENSMUSG00000048410	Zfp407	zinc finger protein 407 [Source:MGI Symbol;Acc:M(	0.8493
ENSMUSG00000059173	Pde1a	phosphodiesterase 1A, calmodulin-dependent [Sou	0.84933
ENSMUSG00000051116	Gm8121	predicted pseudogene 8121 [Source:MGI Symbol;A	0.84935
ENSMUSG00000097180	2700038G22Rik	RIKEN cDNA 2700038G22 gene [Source:MGI Symbo	0.84945
ENSMUSG00000023942	Slc29a1	solute carrier family 29 (nucleoside transporters), n	0.84962

ENSMUSG00000097524	Gm26846	predicted gene, 26846 [Source:MGI Symbol;Acc:MG	0.84969
ENSMUSG00000033106	Slc7a6os	solute carrier family 7, member 6 opposite strand [	0.84972
ENSMUSG00000091102	5830462I19Rik	RIKEN cDNA 5830462I19 gene [Source:MGI Symbol	0.84973
ENSMUSG00000002500	Rpl3l	ribosomal protein L3-like [Source:MGI Symbol;Acc:	0.84977
ENSMUSG00000084493	Gm22106	predicted gene, 22106 [Source:MGI Symbol;Acc:MG	0.8498
ENSMUSG00000098019	Gm2546	predicted gene 2546 [Source:MGI Symbol;Acc:MGI:	0.8498
ENSMUSG00000072919	Noxred1	NADP+ dependent oxidoreductase domain containi	0.84994
ENSMUSG00000004558	Ndrg2	N-myc downstream regulated gene 2 [Source:MGI S	0.85015
ENSMUSG00000026879	Gsn	gelsolin [Source:MGI Symbol;Acc:MGI:95851]	0.85025
ENSMUSG00000046487	Mospd4	motile sperm domain containing 4 [Source:MGI Syn	0.85029
ENSMUSG00000024907	Gal	galanin [Source:MGI Symbol;Acc:MGI:95637]	0.8503
ENSMUSG00000078607	1810010H24Rik	RIKEN cDNA 1810010H24 gene [Source:MGI Symbo	0.85032
ENSMUSG00000031349	Nsdhl	NAD(P) dependent steroid dehydrogenase-like [Sou	0.85034
ENSMUSG00000023020	Cox14	cytochrome c oxidase assembly protein 14 [Source:	0.85055
ENSMUSG00000020956	Dtd2	D-tyrosyl-tRNA deacylase 2 [Source:MGI Symbol;Ac	0.85056
ENSMUSG00000056215	Lrguk	leucine-rich repeats and guanylate kinase domain c	0.85066
ENSMUSG00000045114	Prrt2	proline-rich transmembrane protein 2 [Source:MGI	0.85067
ENSMUSG00000036611	Eepd1	endonuclease/exonuclease/phosphatase family do	0.8507
ENSMUSG00000077565	Gm23201	predicted gene, 23201 [Source:MGI Symbol;Acc:MG	0.85075
ENSMUSG00000021492	F12	coagulation factor XII (Hageman factor) [Source:MG	0.85077
ENSMUSG00000025485	Ric8	resistance to inhibitors of cholinesterase 8 homolog	0.8509
ENSMUSG00000090075	Gm16251	predicted gene 16251 [Source:MGI Symbol;Acc:MG	0.85104
ENSMUSG00000002664	Pspn	persephin [Source:MGI Symbol;Acc:MGI:1201684]	0.85107
ENSMUSG00000027166	Dnajc24	DnaJ (Hsp40) homolog, subfamily C, member 24 [So	0.8511
ENSMUSG00000036023	Parp2	poly (ADP-ribose) polymerase family, member 2 [So	0.85122
ENSMUSG00000002578	Ikzf4	IKAROS family zinc finger 4 [Source:MGI Symbol;Ac	0.85123
ENSMUSG00000056458	Stk30	serine/threonine kinase 30 [Source:MGI Symbol;Ac	0.85134
ENSMUSG00000083012	Fam220a	family with sequence similarity 220, member A [Sou	0.85136
ENSMUSG00000002963	Pnkp	polynucleotide kinase 3'- phosphatase [Source:MGI	0.85154
ENSMUSG00000037646	Vps13b	vacuolar protein sorting 13B (yeast) [Source:MGI Sy	0.85162
ENSMUSG00000089886	Gm16184	predicted gene 16184 [Source:MGI Symbol;Acc:MG	0.85164
ENSMUSG00000036381	P2ry14	purinergic receptor P2Y, G-protein coupled, 14 [Sou	0.85172
ENSMUSG00000036545	Adamts2	a disintegrin-like and metallopeptidase (reprolysin	0.85173
ENSMUSG00000036792	Mbd5	methyl-CpG binding domain protein 5 [Source:MGI	0.85185
ENSMUSG00000028455	Stoml2	stomatin (Epb7.2)-like 2 [Source:MGI Symbol;Acc:M	0.85189
ENSMUSG00000036459	Wtip	WT1-interacting protein [Source:MGI Symbol;Acc:M	0.85196
ENSMUSG00000045404	Kcnk13	potassium channel, subfamily K, member 13 [Sourc	0.85201
ENSMUSG00000025503	Taldo1	transaldolase 1 [Source:MGI Symbol;Acc:MGI:1274	0.85205
ENSMUSG00000022574	Naprt1	nicotinate phosphoribosyltransferase domain conta	0.85211
ENSMUSG00000062762	Ei24	etoposide induced 2.4 mRNA [Source:MGI Symbol;	0.85213
ENSMUSG00000051726	Kcnf1	potassium voltage-gated channel, subfamily F, men	0.85222
ENSMUSG00000032180	Tmed1	transmembrane emp24 domain containing 1 [Sourc	0.85247
ENSMUSG00000033557	Fam20b	family with sequence similarity 20, member B [Sour	0.85252
ENSMUSG00000090565	4930405O22Rik	RIKEN cDNA 4930405O22 gene [Source:MGI Symbo	0.85252
ENSMUSG00000027860	Vangl1	vang-like 1 (van gogh, Drosophila) [Source:MGI Sym	0.85272
ENSMUSG00000066319	Rtp3	receptor transporter protein 3 [Source:MGI Symbo	0.85284
ENSMUSG00000097081	Gm10425	predicted gene 10425 [Source:MGI Symbol;Acc:MG	0.85292

ENSMUSG00000000127	Fer	fer (fms/fps related) protein kinase [Source:MGI Sy	0.853
ENSMUSG00000039131	Gipc2	GIPC PDZ domain containing family, member 2 [Sou	0.85305
ENSMUSG00000018541	Cwc25	CWC25 spliceosome-associated protein homolog (S	0.85313
ENSMUSG00000003380	Rabac1	Rab acceptor 1 (prenylated) [Source:MGI Symbol;A	0.85318
ENSMUSG00000021986	Amer2	APC membrane recruitment 2 [Source:MGI Symbol	0.85319
ENSMUSG00000029388	Eif2b1	eukaryotic translation initiation factor 2B, subunit 1	0.85328
ENSMUSG00000031586	Rbpms	RNA binding protein gene with multiple splicing [So	0.85334
ENSMUSG00000005506	Celf1	CUGBP, Elav-like family member 1 [Source:MGI Syn	0.85343
ENSMUSG00000027429	Sec23b	SEC23B (S. cerevisiae) [Source:MGI Symbol;Acc:MG	0.85346
ENSMUSG00000038770	Kpna7	karyopherin alpha 7 (importin alpha 8) [Source:MG	0.85347
ENSMUSG00000046814	Gchfr	GTP cyclohydrolase I feedback regulator [Source:M	0.8535
ENSMUSG00000022215	Fitm1	fat storage-inducing transmembrane protein 1 [Sou	0.85351
ENSMUSG00000038932	Tcfl5	transcription factor-like 5 (basic helix-loop-helix) [S	0.85353
ENSMUSG00000027394	Ttl	tubulin tyrosine ligase [Source:MGI Symbol;Acc:MG	0.85365
ENSMUSG00000040822	1700123O20Rik	RIKEN cDNA 1700123O20 gene [Source:MGI Symbo	0.85373
ENSMUSG00000044744	1700001G17Rik	RIKEN cDNA 1700001G17 gene [Source:MGI Symbo	0.85378
ENSMUSG00000007989	Fzd3	frizzled homolog 3 (Drosophila) [Source:MGI Symbo	0.85379
ENSMUSG00000062729	Ppox	protoporphyrinogen oxidase [Source:MGI Symbol;A	0.85387
ENSMUSG00000084411	Gm11510	predicted gene 11510 [Source:MGI Symbol;Acc:MG	0.85389
ENSMUSG00000083538	Gm16016	predicted gene 16016 [Source:MGI Symbol;Acc:MG	0.85393
ENSMUSG00000059183	Mtfmt	mitochondrial methionyl-tRNA formyltransferase [S	0.85396
ENSMUSG00000097131	D230017M19Rik	RIKEN cDNA D230017M19 gene [Source:MGI Symb	0.85405
ENSMUSG00000022787	Wdr53	WD repeat domain 53 [Source:MGI Symbol;Acc:MG	0.85422
ENSMUSG00000025154	Arhgap19	Rho GTPase activating protein 19 [Source:MGI Sym	0.85424
ENSMUSG00000085386	Gm13630	predicted gene 13630 [Source:MGI Symbol;Acc:MG	0.85425
ENSMUSG00000085415	Gm12060	predicted gene 12060 [Source:MGI Symbol;Acc:MG	0.85425
ENSMUSG00000060716	Plekhh1	pleckstrin homology domain containing, family H (w	0.85429
ENSMUSG00000035722	Abca7	ATP-binding cassette, sub-family A (ABC1), member	0.8543
ENSMUSG00000005897	Nr2c1	nuclear receptor subfamily 2, group C, member 1 [S	0.85444
ENSMUSG00000040164	Kcns1	K+ voltage-gated channel, subfamily S, 1 [Source:M	0.85463
ENSMUSG00000078552	Dcdc2b	doublecortin domain containing 2b [Source:MGI Sy	0.85464
ENSMUSG00000058655	Eif4b	eukaryotic translation initiation factor 4B [Source:N	0.85469
ENSMUSG00000087502	Gm16091	predicted gene 16091 [Source:MGI Symbol;Acc:MG	0.85478
ENSMUSG00000033918	Parl	presenilin associated, rhomboid-like [Source:MGI S	0.85485
ENSMUSG00000083207	Gm14780	predicted gene 14780 [Source:MGI Symbol;Acc:MG	0.85494
ENSMUSG00000036390	Gadd45a	growth arrest and DNA-damage-inducible 45 alpha	0.85496
ENSMUSG00000039488	Cntn5	contactin 5 [Source:MGI Symbol;Acc:MGI:3042287]	0.85496
ENSMUSG00000021532	Fastkd3	FAST kinase domains 3 [Source:MGI Symbol;Acc:M	0.85512
ENSMUSG00000053214	Gm9899	predicted gene 9899 [Source:MGI Symbol;Acc:MGI:	0.85515
ENSMUSG00000071392	Ect2l	epithelial cell transforming sequence 2 oncogene-li	0.8552
ENSMUSG00000020092	Pald1	phosphatase domain containing, paladin 1 [Source:	0.85521
ENSMUSG00000020871	Dlx4	distal-less homeobox 4 [Source:MGI Symbol;Acc:M	0.85521
ENSMUSG00000029605	Oas1b	2'-5' oligoadenylate synthetase 1B [Source:MGI Syn	0.85523
ENSMUSG00000097463	4930448E22Rik	RIKEN cDNA 4930448E22 gene [Source:MGI Symbo	0.85536
ENSMUSG00000039382	Wdr45	WD repeat domain 45 [Source:MGI Symbol;Acc:MG	0.85545
ENSMUSG00000033952	Aspm	asp (abnormal spindle)-like, microcephaly associate	0.85553
ENSMUSG00000017832	Hspb9	heat shock protein, alpha-crystallin-related, B9 [Sou	0.85567

ENSMUSG00000036120	Rfxank	regulatory factor X-associated ankyrin-containing p	0.85569
ENSMUSG00000073843	Gm12542	predicted gene 12542 [Source:MGI Symbol;Acc:MG	0.85573
ENSMUSG00000090188	Gm7418	predicted pseudogene 7418 [Source:MGI Symbol;A	0.85581
ENSMUSG00000084381	AA413626	expressed sequence AA413626 [Source:MGI Symbo	0.856
ENSMUSG00000040102	Klhl42	kelch-like 42 [Source:MGI Symbol;Acc:MGI:244478	0.85607
ENSMUSG00000024588	Fech	ferrochelatase [Source:MGI Symbol;Acc:MGI:95513	0.85621
ENSMUSG00000040658	Dnph1	2'-deoxynucleoside 5'-phosphate N-hydrolase 1 [So	0.85623
ENSMUSG00000055897	Ppp4r1l-ps	protein phosphatase 4, regulatory subunit 1-like, ps	0.85628
ENSMUSG00000086674	Gm12283	predicted gene 12283 [Source:MGI Symbol;Acc:MG	0.85634
ENSMUSG00000093031	Gm22662	predicted gene, 22662 [Source:MGI Symbol;Acc:M	0.85642
ENSMUSG00000039062	Anpep	alanyl (membrane) aminopeptidase [Source:MGI Sy	0.85644
ENSMUSG00000092492	B230208B08Rik	RIKEN cDNA B230208B08 gene [Source:MGI Symbo	0.85672
ENSMUSG00000027288	Zfp106	zinc finger protein 106 [Source:MGI Symbol;Acc:M	0.85678
ENSMUSG00000083245	Gm11185	predicted gene 11185 [Source:MGI Symbol;Acc:MG	0.85687
ENSMUSG00000035299	Mid1	midline 1 [Source:MGI Symbol;Acc:MGI:1100537]	0.8569
ENSMUSG00000054843	Atrnl1	attractin like 1 [Source:MGI Symbol;Acc:MGI:2147	0.85692
ENSMUSG00000000056	Narf	nuclear prelamin A recognition factor [Source:MGI	0.85699
ENSMUSG00000087389	Gm15592	predicted gene 15592 [Source:MGI Symbol;Acc:MG	0.85703
ENSMUSG00000097548	Gm26748	predicted gene, 26748 [Source:MGI Symbol;Acc:M	0.85704
ENSMUSG00000024352	Spata24	spermatogenesis associated 24 [Source:MGI Symbo	0.85708
ENSMUSG00000095010	Gm23490	predicted gene, 23490 [Source:MGI Symbol;Acc:M	0.85719
ENSMUSG00000084249	Gm12267	predicted gene 12267 [Source:MGI Symbol;Acc:MG	0.8572
ENSMUSG00000001517	Foxm1	forkhead box M1 [Source:MGI Symbol;Acc:MGI:134	0.85729
ENSMUSG00000063683	Glyat	glycine-N-acyltransferase [Source:MGI Symbol;Acc:	0.85733
ENSMUSG00000097380	Gm26816	predicted gene, 26816 [Source:MGI Symbol;Acc:M	0.8574
ENSMUSG00000097159	Gm26517	predicted gene, 26517 [Source:MGI Symbol;Acc:M	0.85744
ENSMUSG00000041845	Rhod	ras homolog gene family, member D [Source:MGI S	0.85752
ENSMUSG00000055254	Ntrk2	neurotrophic tyrosine kinase, receptor, type 2 [Sou	0.85778
ENSMUSG00000024534	Sncaip	synuclein, alpha interacting protein (synphilin) [Sou	0.85784
ENSMUSG00000031776	Arl2bp	ADP-ribosylation factor-like 2 binding protein [Sour	0.85791
ENSMUSG00000067148	Polr1c	polymerase (RNA) I polypeptide C [Source:MGI Sym	0.858
ENSMUSG00000037465	Klf10	Kruppel-like factor 10 [Source:MGI Symbol;Acc:MG	0.85806
ENSMUSG00000059263	Usp47	ubiquitin specific peptidase 47 [Source:MGI Symbo	0.85807
ENSMUSG00000053985	Zfp14	zinc finger protein 14 [Source:MGI Symbol;Acc:MG	0.85815
ENSMUSG00000028102	Pex11b	peroxisomal biogenesis factor 11 beta [Source:MGI	0.85823
ENSMUSG00000020519	Sap30l	SAP30-like [Source:MGI Symbol;Acc:MGI:1354709]	0.85825
ENSMUSG00000022401	Xpnpep3	X-prolyl aminopeptidase (aminopeptidase P) 3, put	0.85836
ENSMUSG00000031402	Mpp1	membrane protein, palmitoylated [Source:MGI Sym	0.85839
ENSMUSG00000028884	Rpa2	replication protein A2 [Source:MGI Symbol;Acc:MG	0.85854
ENSMUSG00000028972	Car6	carbonic anhydrase 6 [Source:MGI Symbol;Acc:MG	0.85872
ENSMUSG00000065118	Gm23297	predicted gene, 23297 [Source:MGI Symbol;Acc:M	0.85888
ENSMUSG00000030278	Cidec	cell death-inducing DFFA-like effector c [Source:MG	0.85892
ENSMUSG00000047875	Gpr157	G protein-coupled receptor 157 [Source:MGI Symb	0.85897
ENSMUSG00000021318	Gli3	GLI-Kruppel family member GLI3 [Source:MGI Symb	0.85902
ENSMUSG00000044176	Spink10	serine peptidase inhibitor, Kazal type 10 [Source:M	0.85905
ENSMUSG00000006574	Slc4a1	solute carrier family 4 (anion exchanger), member	0.85907
ENSMUSG00000052751	Repin1	replication initiator 1 [Source:MGI Symbol;Acc:MGI	0.85915

ENSMUSG00000029538	Srsf9	serine/arginine-rich splicing factor 9 [Source:MGI S	0.85921
ENSMUSG00000040187	Arntl2	aryl hydrocarbon receptor nuclear translocator-like	0.85944
ENSMUSG00000040033	Stat2	signal transducer and activator of transcription 2 [S	0.85946
ENSMUSG00000042682	Selk	selenoprotein K [Source:MGI Symbol;Acc:MGI:1931	0.85955
ENSMUSG00000057551	Zfp317	zinc finger protein 317 [Source:MGI Symbol;Acc:M	0.85955
ENSMUSG00000071324	Armc2	armadillo repeat containing 2 [Source:MGI Symbol;	0.85958
ENSMUSG00000097336	Fendrr	Foxf1 adjacent non-coding developmental regulato	0.85978
ENSMUSG00000098129	Gm27040	predicted gene, 27040 [Source:MGI Symbol;Acc:M	0.85978
ENSMUSG00000020234	4930404N11Rik	RIKEN cDNA 4930404N11 gene [Source:MGI Symbo	0.85979
ENSMUSG00000027566	Psma7	proteasome (prosome, macropain) subunit, alpha t	0.85986
ENSMUSG00000068327	Tlx2	T cell leukemia, homeobox 2 [Source:MGI Symbol;A	0.85989
ENSMUSG00000097827	5330439K02Rik	RIKEN cDNA 5330439K02 gene [Source:MGI Symbo	0.85991
ENSMUSG00000060572	Mfap2	microfibrillar-associated protein 2 [Source:MGI Sym	0.85997
ENSMUSG00000086496	Gm14204	predicted gene 14204 [Source:MGI Symbol;Acc:MG	0.85997
ENSMUSG00000039449	Prpf18	PRP18 pre-mRNA processing factor 18 homolog (ye	0.85998
ENSMUSG00000080152	Gm12260	predicted gene 12260 [Source:MGI Symbol;Acc:MG	0.86002
ENSMUSG00000085957	Syna	syncytin a [Source:MGI Symbol;Acc:MGI:2684898]	0.86004
ENSMUSG00000085125	Gm16070	predicted gene 16070 [Source:MGI Symbol;Acc:MG	0.86009
ENSMUSG00000032601	Prkar2a	protein kinase, cAMP dependent regulatory, type II	0.86025
ENSMUSG00000042249	Adrbk2	adrenergic receptor kinase, beta 2 [Source:MGI Sym	0.86032
ENSMUSG00000034709	Ppp1r21	protein phosphatase 1, regulatory subunit 21 [Sour	0.8604
ENSMUSG00000079111	Kdelr2	KDEL (Lys-Asp-Glu-Leu) endoplasmic reticulum prot	0.86046
ENSMUSG00000002324	Rec8	REC8 homolog (yeast) [Source:MGI Symbol;Acc:MG	0.86053
ENSMUSG00000027750	Postn	periostin, osteoblast specific factor [Source:MGI Sy	0.86074
ENSMUSG00000028402	Mpdz	multiple PDZ domain protein [Source:MGI Symbol;A	0.86081
ENSMUSG00000027324	Rpusd2	RNA pseudouridylate synthase domain containing 2	0.86084
ENSMUSG00000039067	Psmd7	proteasome (prosome, macropain) 26S subunit, no	0.86084
ENSMUSG00000026805	Barhl1	BarH-like 1 (Drosophila) [Source:MGI Symbol;Acc:M	0.86093
ENSMUSG00000031509	1700016D06Rik	RIKEN cDNA 1700016D06 gene [Source:MGI Symbo	0.86096
ENSMUSG00000064558	Gm24620	predicted gene, 24620 [Source:MGI Symbol;Acc:M	0.86096
ENSMUSG00000029614	Rpl6	ribosomal protein L6 [Source:MGI Symbol;Acc:MGI	0.86106
ENSMUSG00000029607	Ankrd61	ankyrin repeat domain 61 [Source:MGI Symbol;Acc	0.86108
ENSMUSG00000027133	Nop10	NOP10 ribonucleoprotein [Source:MGI Symbol;Acc	0.8612
ENSMUSG00000016239	Lonrf3	LON peptidase N-terminal domain and ring finger 3	0.86122
ENSMUSG00000024667	Tmem216	transmembrane protein 216 [Source:MGI Symbol;A	0.86123
ENSMUSG00000024301	Kifc5b	kinesin family member C5B [Source:MGI Symbol;Ac	0.86128
ENSMUSG00000032290	Ptpn9	protein tyrosine phosphatase, non-receptor type 9	0.86133
ENSMUSG00000087430	Gm13405	predicted gene 13405 [Source:MGI Symbol;Acc:MG	0.86137
ENSMUSG00000082697	Gm12913	predicted gene 12913 [Source:MGI Symbol;Acc:MG	0.86138
ENSMUSG00000032498	Mlh1	mutL homolog 1 (E. coli) [Source:MGI Symbol;Acc:M	0.86147
ENSMUSG00000096954	Gdap10	ganglioside-induced differentiation-associated-prot	0.86148
ENSMUSG00000079037	Prnp	prion protein [Source:MGI Symbol;Acc:MGI:97769]	0.86155
ENSMUSG00000027364	Usp50	ubiquitin specific peptidase 50 [Source:MGI Symbo	0.86168
ENSMUSG00000030890	Ilk	integrin linked kinase [Source:MGI Symbol;Acc:MG	0.8617
ENSMUSG00000070942	Il1rl2	interleukin 1 receptor-like 2 [Source:MGI Symbol;A	0.86176
ENSMUSG00000021156	Zmynd11	zinc finger, MYND domain containing 11 [Source:M	0.86177
ENSMUSG00000023571	Fam132a	family with sequence similarity 132, member A [So	0.86179

ENSMUSG00000029346	Srrd	SRR1 domain containing [Source:MGI Symbol;Acc:N	0.86185
ENSMUSG00000035745	Grin3b	glutamate receptor, ionotropic, NMDA3B [Source:N	0.86186
ENSMUSG00000059301	Gm5434	predicted gene 5434 [Source:MGI Symbol;Acc:MGI:	0.86191
ENSMUSG00000077552	Gm23709	predicted gene, 23709 [Source:MGI Symbol;Acc:MG	0.86204
ENSMUSG00000051984	Sec31b	Sec31 homolog B (S. cerevisiae) [Source:MGI Symbo	0.86219
ENSMUSG00000026856	Dolpp1	dolichyl pyrophosphate phosphatase 1 [Source:MG	0.8623
ENSMUSG00000031534	Smim19	small integral membrane protein 19 [Source:MGI Sy	0.86234
ENSMUSG00000097133	Gm26628	predicted gene, 26628 [Source:MGI Symbol;Acc:MG	0.86245
ENSMUSG00000075334	Rprm	reprimo, TP53 dependent G2 arrest mediator candi	0.86256
ENSMUSG00000025432	Avil	advillin [Source:MGI Symbol;Acc:MGI:1333798]	0.86257
ENSMUSG00000034686	Prr7	proline rich 7 (synaptic) [Source:MGI Symbol;Acc:M	0.86264
ENSMUSG00000003752	Itpkc	inositol 1,4,5-trisphosphate 3-kinase C [Source:MG	0.86272
ENSMUSG00000031015	Swap70	SWA-70 protein [Source:MGI Symbol;Acc:MGI:1298	0.86275
ENSMUSG00000021981	Cab39l	calcium binding protein 39-like [Source:MGI Symbo	0.86279
ENSMUSG00000054169	Ceacam10	carcinoembryonic antigen-related cell adhesion mo	0.86298
ENSMUSG00000045555	Mettl24	methyltransferase like 24 [Source:MGI Symbol;Acc:	0.86302
ENSMUSG00000056947	Mab21l1	mab-21-like 1 (C. elegans) [Source:MGI Symbol;Acc	0.86309
ENSMUSG00000036139	Hoxc9	homeobox C9 [Source:MGI Symbol;Acc:MGI:96199]	0.86311
ENSMUSG00000040165	Cd209c	CD209c antigen [Source:MGI Symbol;Acc:MGI:2157	0.86324
ENSMUSG00000064109	Hcst	hematopoietic cell signal transducer [Source:MGI S	0.86336
ENSMUSG00000024575	Pde6a	phosphodiesterase 6A, cGMP-specific, rod, alpha [S	0.86337
ENSMUSG00000097348	Rmst	rhabdomyosarcoma 2 associated transcript (non-co	0.86347
ENSMUSG00000038365	Fbxo25	F-box protein 25 [Source:MGI Symbol;Acc:MGI:191	0.86351
ENSMUSG00000028114	Mettl14	methyltransferase like 14 [Source:MGI Symbol;Acc:	0.86352
ENSMUSG00000041712	Ubr7	ubiquitin protein ligase E3 component n-recognin 7	0.86353
ENSMUSG00000081862	Gm12413	predicted gene 12413 [Source:MGI Symbol;Acc:MG	0.86358
ENSMUSG00000005370	Msh6	mutS homolog 6 (E. coli) [Source:MGI Symbol;Acc:N	0.8636
ENSMUSG00000004931	Apba3	amyloid beta (A4) precursor protein-binding, family	0.86362
ENSMUSG00000017286	Glod4	glyoxalase domain containing 4 [Source:MGI Symbo	0.86393
ENSMUSG00000000827	Tpd52l2	tumor protein D52-like 2 [Source:MGI Symbol;Acc:I	0.86396
ENSMUSG00000087385	AA415398	expressed sequence AA415398 [Source:MGI Symbo	0.86396
ENSMUSG00000035891	Cerk	ceramide kinase [Source:MGI Symbol;Acc:MGI:238	0.86413
ENSMUSG00000097892	Gm26801	predicted gene, 26801 [Source:MGI Symbol;Acc:MG	0.86425
ENSMUSG00000080956	Gm16179	predicted gene 16179 [Source:MGI Symbol;Acc:MG	0.86433
ENSMUSG00000004151	Etv1	ets variant gene 1 [Source:MGI Symbol;Acc:MGI:99	0.86448
ENSMUSG00000038213	Tapbpl	TAP binding protein-like [Source:MGI Symbol;Acc:N	0.86456
ENSMUSG00000038784	Cnot4	CCR4-NOT transcription complex, subunit 4 [Source	0.86478
ENSMUSG00000023505	Cdca3	cell division cycle associated 3 [Source:MGI Symbol	0.86482
ENSMUSG00000021965	Ska3	spindle and kinetochore associated complex subun	0.86484
ENSMUSG00000027673	Ndufb5	NADH dehydrogenase (ubiquinone) 1 beta subcom	0.86488
ENSMUSG00000019564	Arid3a	AT rich interactive domain 3A (BRIGHT-like) [Source	0.86497
ENSMUSG00000048772	Tmem53	transmembrane protein 53 [Source:MGI Symbol;Ac	0.86518
ENSMUSG00000054517	Trim65	tripartite motif-containing 65 [Source:MGI Symbol;	0.86525
ENSMUSG00000018634	Crhr1	corticotropin releasing hormone receptor 1 [Source	0.8653
ENSMUSG00000086331	Gm16310	predicted gene 16310 [Source:MGI Symbol;Acc:MG	0.86531
ENSMUSG00000093739	Gm20692	predicted gene 20692 [Source:MGI Symbol;Acc:MG	0.86551
ENSMUSG00000052558	Gm9884	predicted gene 9884 [Source:MGI Symbol;Acc:MGI:	0.86552

ENSMUSG00000004110	Cacna1e	calcium channel, voltage-dependent, R type, alpha	0.86558
ENSMUSG00000008200	Fnbp4	formin binding protein 4 [Source:MGI Symbol;Acc:M	0.86561
ENSMUSG00000032355	Mlip	muscular LMNA-interacting protein [Source:MGI Sy	0.866
ENSMUSG00000055128	Cgrrf1	cell growth regulator with ring finger domain 1 [Sou	0.86615
ENSMUSG00000017868	Sgk2	serum/glucocorticoid regulated kinase 2 [Source:M	0.86633
ENSMUSG00000035456	Prdm8	PR domain containing 8 [Source:MGI Symbol;Acc:M	0.86637
ENSMUSG00000093404	Gm25190	predicted gene, 25190 [Source:MGI Symbol;Acc:MG	0.86639
ENSMUSG00000092118	Fancf	Fanconi anemia, complementation group F [Source	0.86641
ENSMUSG00000045005	Fzd5	frizzled homolog 5 (Drosophila) [Source:MGI Symbo	0.86645
ENSMUSG00000097071	4930544I03Rik	RIKEN cDNA 4930544I03 gene [Source:MGI Symbol	0.86664
ENSMUSG00000034792	Gna15	guanine nucleotide binding protein, alpha 15 [Sour	0.86667
ENSMUSG00000078776	9530053A07Rik	RIKEN cDNA 9530053A07 gene [Source:MGI Symbo	0.86672
ENSMUSG00000043587	Acpl2	acid phosphatase-like 2 [Source:MGI Symbol;Acc:M	0.86677
ENSMUSG00000026102	Inpp1	inositol polyphosphate-1-phosphatase [Source:MG	0.86679
ENSMUSG00000053714	4732471J01Rik	RIKEN cDNA 4732471J01 gene [Source:MGI Symbol	0.86684
ENSMUSG00000020818	Mfsd11	major facilitator superfamily domain containing 11	0.86685
ENSMUSG00000089806	Gm16570	predicted gene 16570 [Source:MGI Symbol;Acc:MG	0.86687
ENSMUSG00000091730	Gm17230	predicted gene 17230 [Source:MGI Symbol;Acc:MG	0.86691
ENSMUSG00000044122	Proca1	protein interacting with cyclin A1 [Source:MGI Sym	0.86693
ENSMUSG00000029131	Dnajb6	DnaJ (Hsp40) homolog, subfamily B, member 6 [Sou	0.86701
ENSMUSG00000040140	Tdrd6	tudor domain containing 6 [Source:MGI Symbol;Acc	0.86705
ENSMUSG00000032985	5730522E02Rik	RIKEN cDNA 5730522E02 gene [Source:MGI Symbo	0.86712
ENSMUSG00000021555	Naa35	N(alpha)-acetyltransferase 35, NatC auxiliary subun	0.86714
ENSMUSG00000019977	Hbs1l	Hbs1-like (S. cerevisiae) [Source:MGI Symbol;Acc:M	0.86722
ENSMUSG00000028411	Aptx	aprataxin [Source:MGI Symbol;Acc:MGI:1913658]	0.8673
ENSMUSG00000039977	Ccdc67	coiled-coil domain containing 67 [Source:MGI Symb	0.8673
ENSMUSG00000050944	Efcab5	EF-hand calcium binding domain 5 [Source:MGI Syr	0.86733
ENSMUSG00000096210	H1f0	H1 histone family, member 0 [Source:MGI Symbol;	0.86735
ENSMUSG00000073739	Gm16287	predicted gene 16287 [Source:MGI Symbol;Acc:MG	0.86745
ENSMUSG00000026605	Cenpf	centromere protein F [Source:MGI Symbol;Acc:MG	0.86756
ENSMUSG00000074899	Sptbn5	spectrin beta, non-erythrocytic 5 [Source:MGI Sym	0.86756
ENSMUSG00000017453	Pipox	pipecolic acid oxidase [Source:MGI Symbol;Acc:MG	0.86762
ENSMUSG00000021972	Hmbox1	homeobox containing 1 [Source:MGI Symbol;Acc:M	0.86782
ENSMUSG00000028341	Nr4a3	nuclear receptor subfamily 4, group A, member 3 [S	0.86795
ENSMUSG00000029363	Rfc5	replication factor C (activator 1) 5 [Source:MGI Sym	0.86808
ENSMUSG00000054582	Pabpc1l	poly(A) binding protein, cytoplasmic 1-like [Source:	0.86808
ENSMUSG00000030745	Il21r	interleukin 21 receptor [Source:MGI Symbol;Acc:M	0.86822
ENSMUSG00000066952	Myo1h	myosin 1H [Source:MGI Symbol;Acc:MGI:1914674]	0.86822
ENSMUSG00000054161	Fam83e	family with sequence similarity 83, member E [Sour	0.86837
ENSMUSG00000041679	Lrrc29	leucine rich repeat containing 29 [Source:MGI Sym	0.86842
ENSMUSG00000086539	Gm16759	predicted gene, 16759 [Source:MGI Symbol;Acc:MG	0.86845
ENSMUSG00000039670	Oxld1	oxidoreductase like domain containing 1 [Source:M	0.86854
ENSMUSG00000024935	Slc1a1	solute carrier family 1 (neuronal/epithelial high affi	0.86883
ENSMUSG00000032329	Hmg20a	high mobility group 20A [Source:MGI Symbol;Acc:M	0.86884
ENSMUSG00000097501	Gm26536	predicted gene, 26536 [Source:MGI Symbol;Acc:MG	0.86903
ENSMUSG00000080976	Gm11490	predicted gene 11490 [Source:MGI Symbol;Acc:MG	0.86928
ENSMUSG00000059923	Grb2	growth factor receptor bound protein 2 [Source:MG	0.86931

ENSMUSG00000044364	Tmem74b	transmembrane protein 74b [Source:MGI Symbol;A	0.86948
ENSMUSG00000082463	Gm15765	predicted gene 15765 [Source:MGI Symbol;Acc:MG	0.86948
ENSMUSG00000009551	6330409D20Rik	RIKEN cDNA 6330409D20 gene [Source:MGI Symbo	0.86951
ENSMUSG00000003762	Adck4	aarF domain containing kinase 4 [Source:MGI Symb	0.86956
ENSMUSG00000048482	Bdnf	brain derived neurotrophic factor [Source:MGI Sym	0.86956
ENSMUSG00000096987	Gm26525	predicted gene, 26525 [Source:MGI Symbol;Acc:M	0.86979
ENSMUSG00000034343	Ube2f	ubiquitin-conjugating enzyme E2F (putative) [Sourc	0.86998
ENSMUSG00000040717	Il17rd	interleukin 17 receptor D [Source:MGI Symbol;Acc:	0.8701
ENSMUSG00000027796	Smad9	SMAD family member 9 [Source:MGI Symbol;Acc:M	0.87026
ENSMUSG00000001983	Taco1	translational activator of mitochondrially encoded	0.87033
ENSMUSG00000037855	Zfp365	zinc finger protein 365 [Source:MGI Symbol;Acc:M	0.87046
ENSMUSG00000028893	Sesn2	sestrin 2 [Source:MGI Symbol;Acc:MGI:2651874]	0.87058
ENSMUSG00000010097	Nxf1	nuclear RNA export factor 1 [Source:MGI Symbol;A	0.8708
ENSMUSG00000042816	Gpr151	G protein-coupled receptor 151 [Source:MGI Symb	0.87084
ENSMUSG00000090470	2410012M07Rik	RIKEN cDNA 2410012M07 gene [Source:MGI Symb	0.87101
ENSMUSG00000029158	Yipf7	Yip1 domain family, member 7 [Source:MGI Symbo	0.87102
ENSMUSG00000044014	Npy5r	neuropeptide Y receptor Y5 [Source:MGI Symbol;A	0.87104
ENSMUSG00000086502	B130055M24Rik	RIKEN cDNA B130055M24 gene [Source:MGI Symb	0.87117
ENSMUSG00000085385	9430008C03Rik	RIKEN cDNA 9430008C03 gene [Source:MGI Symbo	0.8712
ENSMUSG00000035367	Rmi1	RMI1, RecQ mediated genome instability 1, homol	0.87123
ENSMUSG00000049657	Zbtb5	zinc finger and BTB domain containing 5 [Source:M	0.87131
ENSMUSG00000041700	Lhfpl1	lipoma HMGIC fusion partner-like 1 [Source:MGI Sy	0.87141
ENSMUSG00000075510	Fam187a	family with sequence similarity 187, member A [So	0.87142
ENSMUSG00000026778	Prkcq	protein kinase C, theta [Source:MGI Symbol;Acc:M	0.87157
ENSMUSG00000032462	Pik3cb	phosphatidylinositol 3-kinase, catalytic, beta polyp	0.87171
ENSMUSG00000060509	Xcr1	chemokine (C motif) receptor 1 [Source:MGI Symb	0.87175
ENSMUSG00000066057	Gm1976	predicted gene 1976 [Source:MGI Symbol;Acc:MGI	0.87177
ENSMUSG00000078630	Tomt	transmembrane O-methyltransferase [Source:MGI	0.8718
ENSMUSG00000010175	Prox1	prospero-related homeobox 1 [Source:MGI Symbol	0.87204
ENSMUSG00000047379	B3gnt1	UDP-GlcNAc:betaGal beta-1,3-N-acetylglucosaminy	0.87206
ENSMUSG00000045948	Mrps12	mitochondrial ribosomal protein S12 [Source:MGI S	0.87208
ENSMUSG00000011114	Tbrg1	transforming growth factor beta regulated gene 1 [	0.8723
ENSMUSG00000079442	St6galnac4	ST6 (alpha-N-acetyl-neuraminyl-2,3-beta-galactosy	0.87245
ENSMUSG00000048076	Arf1	ADP-ribosylation factor 1 [Source:MGI Symbol;Acc:	0.87247
ENSMUSG00000020230	Prmt2	protein arginine N-methyltransferase 2 [Source:MG	0.87255
ENSMUSG00000058145	Adamts17	a disintegrin-like and metallopeptidase (reprolysin	0.87271
ENSMUSG00000016520	Lnx2	ligand of numb-protein X 2 [Source:MGI Symbol;Ac	0.87276
ENSMUSG00000089984	Fbxo24	F-box protein 24 [Source:MGI Symbol;Acc:MGI:191	0.87276
ENSMUSG00000031546	Gins4	GINS complex subunit 4 (Sld5 homolog) [Source:M	0.87298
ENSMUSG00000046027	Stard5	StAR-related lipid transfer (START) domain contain	0.87302
ENSMUSG00000024781	Lipa	lysosomal acid lipase A [Source:MGI Symbol;Acc:M	0.87315
ENSMUSG00000037759	Ptger2	prostaglandin E receptor 2 (subtype EP2) [Source:M	0.87327
ENSMUSG00000037344	Slc12a9	solute carrier family 12 (potassium/chloride transp	0.8733
ENSMUSG00000051401	Kctd16	potassium channel tetramerisation domain contain	0.87333
ENSMUSG00000054823	Whsc1l1	Wolf-Hirschhorn syndrome candidate 1-like 1 (hum	0.87333
ENSMUSG00000081692	Gm15971	predicted gene 15971 [Source:MGI Symbol;Acc:MG	0.87344
ENSMUSG00000045689	Pcdhb4	protocadherin beta 4 [Source:MGI Symbol;Acc:MGI	0.87345

ENSMUSG00000024912	Fosl1	fos-like antigen 1 [Source:MGI Symbol;Acc:MGI:107	0.87353
ENSMUSG00000032512	Wdr48	WD repeat domain 48 [Source:MGI Symbol;Acc:MG	0.87372
ENSMUSG00000030075	Cntn3	contactin 3 [Source:MGI Symbol;Acc:MGI:99534]	0.8738
ENSMUSG00000020495	Smg8	smg-8 homolog, nonsense mediated mRNA decay f	0.87381
ENSMUSG00000022066	Gm21685	predicted gene, 21685 [Source:MGI Symbol;Acc:MG	0.87381
ENSMUSG00000097682	4930554H23Rik	RIKEN cDNA 4930554H23 gene [Source:MGI Symbo	0.87384
ENSMUSG00000073125	Xlr3b	X-linked lymphocyte-regulated 3B [Source:MGI Sym	0.87401
ENSMUSG00000071456	1110002L01Rik	RIKEN cDNA 1110002L01 gene [Source:MGI Symbo	0.87408
ENSMUSG00000020227	Irak3	interleukin-1 receptor-associated kinase 3 [Source:	0.87415
ENSMUSG00000046572	Zfp518b	zinc finger protein 518B [Source:MGI Symbol;Acc:M	0.87419
ENSMUSG00000090722	Gm8378	predicted gene 8378 [Source:MGI Symbol;Acc:MGI:	0.87425
ENSMUSG00000073129	Gm10472	predicted gene 10472 [Source:MGI Symbol;Acc:MG	0.87427
ENSMUSG00000036731	2310002J15Rik	RIKEN cDNA 2310002J15 gene [Source:MGI Symbol	0.87429
ENSMUSG00000033499	Larp4b	La ribonucleoprotein domain family, member 4B [S	0.8744
ENSMUSG00000026556	Vangl2	vang-like 2 (van gogh, Drosophila) [Source:MGI Sym	0.87442
ENSMUSG00000053914	Kdm4d	lysine (K)-specific demethylase 4D [Source:MGI Sym	0.87444
ENSMUSG00000024515	Smad4	SMAD family member 4 [Source:MGI Symbol;Acc:M	0.87448
ENSMUSG00000074715	Ccl28	chemokine (C-C motif) ligand 28 [Source:MGI Symb	0.87453
ENSMUSG00000021032	Ngb	neuroglobin [Source:MGI Symbol;Acc:MGI:2151886	0.87455
ENSMUSG00000023832	Acat2	acetyl-Coenzyme A acetyltransferase 2 [Source:MG	0.87457
ENSMUSG00000087660	B230398E01Rik	RIKEN cDNA B230398E01 gene [Source:MGI Symbo	0.87459
ENSMUSG00000021534	1700001L19Rik	RIKEN cDNA 1700001L19 gene [Source:MGI Symbo	0.87472
ENSMUSG00000026934	Lhx3	LIM homeobox protein 3 [Source:MGI Symbol;Acc:I	0.87474
ENSMUSG00000068394	Cep152	centrosomal protein 152 [Source:MGI Symbol;Acc:I	0.87481
ENSMUSG00000092622	Khdc3	KH domain containing 3, subcortical maternal comp	0.87495
ENSMUSG00000085185	BC028777	cDNA sequence BC028777 [Source:MGI Symbol;Acc	0.87499
ENSMUSG00000020832	Eral1	Era (G-protein)-like 1 (E. coli) [Source:MGI Symbol;/	0.87523
ENSMUSG00000043065	Spice1	spindle and centriole associated protein 1 [Source:I	0.87534
ENSMUSG00000097680	Gm26642	predicted gene, 26642 [Source:MGI Symbol;Acc:MG	0.87544
ENSMUSG00000000738	Spg7	spastic paraplegia 7 homolog (human) [Source:MGI	0.87559
ENSMUSG00000049349	Gm5105	predicted gene 5105 [Source:MGI Symbol;Acc:MGI:	0.87565
ENSMUSG00000038533	Cbfa2t2	core-binding factor, runt domain, alpha subunit 2, t	0.87581
ENSMUSG00000085768	Gm13707	predicted gene 13707 [Source:MGI Symbol;Acc:MG	0.87581
ENSMUSG00000039728	Slc6a5	solute carrier family 6 (neurotransmitter transporte	0.87586
ENSMUSG00000019838	Slc16a10	solute carrier family 16 (monocarboxylic acid transp	0.87589
ENSMUSG00000097089	Gm26879	predicted gene, 26879 [Source:MGI Symbol;Acc:MG	0.87589
ENSMUSG00000030757	Zkscan2	zinc finger with KRAB and SCAN domains 2 [Source:	0.87592
ENSMUSG00000041540	Sox5	SRY-box containing gene 5 [Source:MGI Symbol;Acc	0.87594
ENSMUSG00000050106	Tmc8	transmembrane channel-like gene family 8 [Source	0.87599
ENSMUSG00000056310	Tyw1	tRNA-yW synthesizing protein 1 homolog (S. cerevis	0.87606
ENSMUSG00000097873	Gm26867	predicted gene, 26867 [Source:MGI Symbol;Acc:MG	0.87606
ENSMUSG00000029464	Gpn3	GPN-loop GTPase 3 [Source:MGI Symbol;Acc:MGI:1	0.87614
ENSMUSG00000092901	Mir5130	microRNA 5130 [Source:MGI Symbol;Acc:MGI:4950	0.87631
ENSMUSG00000024096	Ralbp1	ralA binding protein 1 [Source:MGI Symbol;Acc:MG	0.87634
ENSMUSG00000081619	Gm13351	predicted gene 13351 [Source:MGI Symbol;Acc:MG	0.87638
ENSMUSG00000029178	Klf3	Kruppel-like factor 3 (basic) [Source:MGI Symbol;Ac	0.8765
ENSMUSG00000005470	Asf1b	ASF1 anti-silencing function 1 homolog B (S. cerevis	0.87667

ENSMUSG00000000346	Dazap2	DAZ associated protein 2 [Source:MGI Symbol;Acc:	0.87676
ENSMUSG00000030968	Pdilt	protein disulfide isomerase-like, testis expressed [S	0.87684
ENSMUSG00000020279	Il9r	interleukin 9 receptor [Source:MGI Symbol;Acc:MG	0.87688
ENSMUSG00000021765	Fst	follistatin [Source:MGI Symbol;Acc:MGI:95586]	0.877
ENSMUSG00000026617	Bpnt1	bisphosphate 3'-nucleotidase 1 [Source:MGI Symbo	0.87703
ENSMUSG00000056130	Ticam2	toll-like receptor adaptor molecule 2 [Source:MGI S	0.8772
ENSMUSG00000026628	Atf3	activating transcription factor 3 [Source:MGI Symbo	0.87721
ENSMUSG00000030826	Bcat2	branched chain aminotransferase 2, mitochondrial	0.87726
ENSMUSG00000044465	Fam160a2	family with sequence similarity 160, member A2 [So	0.87733
ENSMUSG00000062519	Zfp398	zinc finger protein 398 [Source:MGI Symbol;Acc:MG	0.87737
ENSMUSG00000021686	Ap3b1	adaptor-related protein complex 3, beta 1 subunit	0.87741
ENSMUSG00000047759	Hs3st3a1	heparan sulfate (glucosamine) 3-O-sulfotransferase	0.87752
ENSMUSG00000063652	Slc22a21	solute carrier family 22 (organic cation transporter)	0.87755
ENSMUSG00000096188	Cmtm4	CKLF-like MARVEL transmembrane domain contain	0.87758
ENSMUSG00000046610	Oacyl	O-acyltransferase like [Source:MGI Symbol;Acc:MG	0.87763
ENSMUSG00000028965	Tnfrsf9	tumor necrosis factor receptor superfamily, membe	0.87779
ENSMUSG00000028822	Tmem50a	transmembrane protein 50A [Source:MGI Symbol;A	0.87781
ENSMUSG00000021576	Pdcd6	programmed cell death 6 [Source:MGI Symbol;Acc:	0.87791
ENSMUSG00000097694	G730013B05Rik	RIKEN cDNA G730013B05 gene [Source:MGI Symbo	0.87794
ENSMUSG00000068284	Gm608	predicted gene 608 [Source:MGI Symbol;Acc:MGI:2	0.87801
ENSMUSG00000097621	Gm26562	predicted gene, 26562 [Source:MGI Symbol;Acc:MG	0.87802
ENSMUSG00000078722	Gm12394	predicted gene 12394 [Source:MGI Symbol;Acc:MG	0.87814
ENSMUSG00000032607	Amt	aminomethyltransferase [Source:MGI Symbol;Acc:M	0.87823
ENSMUSG00000098055	Gm26947	predicted gene, 26947 [Source:MGI Symbol;Acc:MG	0.87827
ENSMUSG00000030994	D7Ertd443e	DNA segment, Chr 7, ERATO Doi 443, expressed [So	0.87848
ENSMUSG00000063687	Pcdhb5	protocadherin beta 5 [Source:MGI Symbol;Acc:MGI	0.87851
ENSMUSG00000036835	Psenen	presenilin enhancer 2 homolog (C. elegans) [Source	0.87884
ENSMUSG00000042401	Crtac1	cartilage acidic protein 1 [Source:MGI Symbol;Acc:M	0.87884
ENSMUSG00000020878	Lrrc46	leucine rich repeat containing 46 [Source:MGI Syml	0.87893
ENSMUSG00000053930	Shisa6	shisa homolog 6 (Xenopus laevis) [Source:MGI Sym	0.87902
ENSMUSG00000038312	Edem2	ER degradation enhancer, mannosidase alpha-like 2	0.87907
ENSMUSG00000028920	Fbxo42	F-box protein 42 [Source:MGI Symbol;Acc:MGI:192	0.87917
ENSMUSG00000029591	Ung	uracil DNA glycosylase [Source:MGI Symbol;Acc:MG	0.8792
ENSMUSG00000051444	Bbs12	Bardet-Biedl syndrome 12 (human) [Source:MGI Sy	0.87922
ENSMUSG00000097327	E030030I06Rik	RIKEN cDNA E030030I06 gene [Source:MGI Symbol	0.87923
ENSMUSG00000008874	Clec3a	C-type lectin domain family 3, member a [Source:M	0.87934
ENSMUSG00000018932	Map2k3	mitogen-activated protein kinase kinase 3 [Source:	0.87937
ENSMUSG00000022802	Lmln	leishmanolysin-like (metallopeptidase M8 family) [S	0.87951
ENSMUSG00000067577	A430093F15Rik	RIKEN cDNA A430093F15 gene [Source:MGI Symbo	0.87959
ENSMUSG00000078601	Gm12525	predicted gene 12525 [Source:MGI Symbol;Acc:MG	0.87959
ENSMUSG00000085192	Gm12195	predicted gene 12195 [Source:MGI Symbol;Acc:MG	0.87959
ENSMUSG00000083193	4930595D18Rik	RIKEN cDNA 4930595D18 gene [Source:MGI Symbo	0.8796
ENSMUSG00000087095	Emx2os	Emx2 opposite strand/antisense transcript (non-pro	0.8796
ENSMUSG00000073530	Pappa2	pappalysin 2 [Source:MGI Symbol;Acc:MGI:305164	0.87963
ENSMUSG00000092190	Gm20470	predicted gene 20470 [Source:MGI Symbol;Acc:MG	0.87966
ENSMUSG00000065738	Gm24494	predicted gene, 24494 [Source:MGI Symbol;Acc:MG	0.8797
ENSMUSG00000055546	Timd4	T cell immunoglobulin and mucin domain containin	0.87973

ENSMUSG00000072772	Grcc10	gene rich cluster, C10 gene [Source:MGI Symbol;Ac	0.88028
ENSMUSG00000066892	Fbxl12	F-box and leucine-rich repeat protein 12 [Source:M	0.88032
ENSMUSG00000028431	Ikbkap	inhibitor of kappa light polypeptide enhancer in B c	0.8804
ENSMUSG00000083674	Zfp133-ps	zinc finger protein 133, pseudogene [Source:MGI Sy	0.8804
ENSMUSG00000087261	Gm15477	predicted gene 15477 [Source:MGI Symbol;Acc:MG	0.8804
ENSMUSG00000000094	Tbx4	T-box 4 [Source:MGI Symbol;Acc:MGI:102556]	0.88053
ENSMUSG00000032224	Fam81a	family with sequence similarity 81, member A [Sou	0.88054
ENSMUSG00000092448	Gm20387	predicted gene 20387 [Source:MGI Symbol;Acc:MG	0.88056
ENSMUSG00000070315	4930581F22Rik	RIKEN cDNA 4930581F22 gene [Source:MGI Symbo	0.88064
ENSMUSG00000086105	Gm11636	predicted gene 11636 [Source:MGI Symbol;Acc:MG	0.88068
ENSMUSG00000045576	St7l	suppression of tumorigenicity 7-like [Source:MGI Sy	0.8807
ENSMUSG00000000889	Dbh	dopamine beta hydroxylase [Source:MGI Symbol;Ac	0.88076
ENSMUSG00000081666	Gm11677	predicted gene 11677 [Source:MGI Symbol;Acc:MG	0.88076
ENSMUSG00000026580	Selp	selectin, platelet [Source:MGI Symbol;Acc:MGI:982	0.88077
ENSMUSG00000046844	Vat1l	vesicle amine transport protein 1 homolog-like (T. c	0.88094
ENSMUSG00000085058	8030453O22Rik	RIKEN cDNA 8030453O22 gene [Source:MGI Symbo	0.88099
ENSMUSG00000021501	Caml	calcium modulating ligand [Source:MGI Symbol;Acc	0.88104
ENSMUSG00000079355	Ackr4	atypical chemokine receptor 4 [Source:MGI Symbo	0.88111
ENSMUSG00000005540	Fcer2a	Fc receptor, IgE, low affinity II, alpha polypeptide [S	0.88114
ENSMUSG00000027967	Neurog2	neurogenin 2 [Source:MGI Symbol;Acc:MGI:109619	0.88132
ENSMUSG00000078486	2310042D19Rik	RIKEN cDNA 2310042D19 gene [Source:MGI Symbo	0.88134
ENSMUSG00000028101	Pias3	protein inhibitor of activated STAT 3 [Source:MGI S	0.88151
ENSMUSG00000026024	Als2	amyotrophic lateral sclerosis 2 (juvenile) [Source:M	0.88153
ENSMUSG00000026999	Nup35	nucleoporin 35 [Source:MGI Symbol;Acc:MGI:1916	0.88153
ENSMUSG00000043939	A530064D06Rik	RIKEN cDNA A530064D06 gene [Source:MGI Symbo	0.88153
ENSMUSG00000028832	Stmn1	stathmin 1 [Source:MGI Symbol;Acc:MGI:96739]	0.88158
ENSMUSG00000056966	Gjc3	gap junction protein, gamma 3 [Source:MGI Symbo	0.88159
ENSMUSG00000028865	Cd164l2	CD164 sialomucin-like 2 [Source:MGI Symbol;Acc:M	0.88175
ENSMUSG00000046805	Mpeg1	macrophage expressed gene 1 [Source:MGI Symbo	0.88196
ENSMUSG00000019756	Prl8a1	prolactin family 8, subfamily a, member 1 [Source:N	0.88209
ENSMUSG00000019732	Calr3	calreticulin 3 [Source:MGI Symbol;Acc:MGI:192056	0.88213
ENSMUSG00000024055	Cyp4f13	cytochrome P450, family 4, subfamily f, polypeptide	0.88227
ENSMUSG00000037579	Kcnh3	potassium voltage-gated channel, subfamily H (eag	0.88227
ENSMUSG00000038503	Mesdc2	mesoderm development candidate 2 [Source:MGI S	0.88237
ENSMUSG00000033149	Phldb2	pleckstrin homology-like domain, family B, member	0.88239
ENSMUSG00000021906	Oxnad1	oxidoreductase NAD-binding domain containing 1 [	0.88249
ENSMUSG00000032469	Dbr1	debranching enzyme homolog 1 (S. cerevisiae) [Sou	0.88263
ENSMUSG00000023047	Amhr2	anti-Mullerian hormone type 2 receptor [Source:M	0.88268
ENSMUSG00000098056	Gm8100	predicted gene 8100 [Source:MGI Symbol;Acc:MGI:	0.88274
ENSMUSG00000029136	Rbks	ribokinase [Source:MGI Symbol;Acc:MGI:1918586]	0.88282
ENSMUSG00000070423	Olfr558	olfactory receptor 558 [Source:MGI Symbol;Acc:MG	0.88284
ENSMUSG00000032392	Parp16	poly (ADP-ribose) polymerase family, member 16 [S	0.88289
ENSMUSG00000081783	Gm15599	predicted gene 15599 [Source:MGI Symbol;Acc:MG	0.88299
ENSMUSG00000092103	Gm17039	predicted gene 17039 [Source:MGI Symbol;Acc:MG	0.88299
ENSMUSG00000022620	Arsa	arylsulfatase A [Source:MGI Symbol;Acc:MGI:88077	0.88303
ENSMUSG00000028911	Srsf4	serine/arginine-rich splicing factor 4 [Source:MGI Sy	0.8831
ENSMUSG00000085642	3110053B16Rik	RIKEN cDNA 3110053B16 gene [Source:MGI Symbo	0.88312

ENSMUSG00000024498	Tcerg1	transcription elongation regulator 1 (CA150) [Sourc	0.88318
ENSMUSG00000032135	Mcam	melanoma cell adhesion molecule [Source:MGI Syn	0.88318
ENSMUSG00000022362	9130401M01Rik	RIKEN cDNA 9130401M01 gene [Source:MGI Symbo	0.88323
ENSMUSG00000023066	Rttn	rotatin [Source:MGI Symbol;Acc:MGI:2179288]	0.88326
ENSMUSG00000029648	Flt1	FMS-like tyrosine kinase 1 [Source:MGI Symbol;Acc	0.88329
ENSMUSG00000019494	Cops6	COP9 (constitutive photomorphogenic) homolog, s	0.88359
ENSMUSG00000025500	1600016N20Rik	RIKEN cDNA 1600016N20 gene [Source:MGI Symbo	0.88366
ENSMUSG00000039740	Alg2	asparagine-linked glycosylation 2 (alpha-1,3-manno	0.88376
ENSMUSG00000086321	Gm11413	predicted gene 11413 [Source:MGI Symbol;Acc:MG	0.88377
ENSMUSG00000087362	Gm13710	predicted gene 13710 [Source:MGI Symbol;Acc:MG	0.88384
ENSMUSG00000027854	Sike1	suppressor of IKBKE 1 [Source:MGI Symbol;Acc:MG	0.88394
ENSMUSG00000036395	Glb1l2	galactosidase, beta 1-like 2 [Source:MGI Symbol;Ac	0.88402
ENSMUSG00000087356	Gm13856	predicted gene 13856 [Source:MGI Symbol;Acc:MG	0.88413
ENSMUSG00000093093	Gm22642	predicted gene, 22642 [Source:MGI Symbol;Acc:M	0.88416
ENSMUSG00000087534	Gm11418	predicted gene 11418 [Source:MGI Symbol;Acc:MG	0.88419
ENSMUSG00000086902	Gm11728	predicted gene 11728 [Source:MGI Symbol;Acc:MG	0.88424
ENSMUSG00000029199	Lias	lipoic acid synthetase [Source:MGI Symbol;Acc:MG	0.88425
ENSMUSG00000026353	Ubxn4	UBX domain protein 4 [Source:MGI Symbol;Acc:MG	0.88432
ENSMUSG00000020912	Krt12	keratin 12 [Source:MGI Symbol;Acc:MGI:96687]	0.88437
ENSMUSG00000038267	Slc22a23	solute carrier family 22, member 23 [Source:MGI Sy	0.88438
ENSMUSG00000031458	Coprs	coordinator of PRMT5, differentiation stimulator [S	0.88447
ENSMUSG00000095597	Gm6472	predicted pseudogene 6472 [Source:MGI Symbol;A	0.88456
ENSMUSG00000064037	Gpn1	GPN-loop GTPase 1 [Source:MGI Symbol;Acc:MGI:1	0.8847
ENSMUSG00000057948	Unc13d	unc-13 homolog D (C. elegans) [Source:MGI Symbo	0.88471
ENSMUSG00000028047	Thbs3	thrombospondin 3 [Source:MGI Symbol;Acc:MGI:9	0.88483
ENSMUSG00000029414	Kntc1	kinetochore associated 1 [Source:MGI Symbol;Acc:	0.88487
ENSMUSG00000032595	Cdhr4	cadherin-related family member 4 [Source:MGI Syn	0.88487
ENSMUSG00000070822	Zscan18	zinc finger and SCAN domain containing 18 [Source	0.88501
ENSMUSG00000020803	Txndc17	thioredoxin domain containing 17 [Source:MGI Sym	0.88509
ENSMUSG00000004896	Rrnad1	ribosomal RNA adenine dimethylase domain contai	0.88516
ENSMUSG00000039754	Alkbh4	alkB, alkylation repair homolog 4 (E. coli) [Source:M	0.88522
ENSMUSG00000086719	Gm16275	predicted gene 16275 [Source:MGI Symbol;Acc:MG	0.88534
ENSMUSG00000034566	Atp5h	ATP synthase, H+ transporting, mitochondrial F0 co	0.88535
ENSMUSG00000044313	Mab21l3	mab-21-like 3 (C. elegans) [Source:MGI Symbol;Acc	0.88541
ENSMUSG00000028295	Smim8	small integral membrane protein 8 [Source:MGI Sy	0.88544
ENSMUSG00000059901	Adamts14	a disintegrin-like and metallopeptidase (reprolysin	0.88553
ENSMUSG00000053436	Mapk14	mitogen-activated protein kinase 14 [Source:MGI S	0.88563
ENSMUSG00000056972	Magel2	melanoma antigen, family L, 2 [Source:MGI Symbol	0.88564
ENSMUSG00000068329	Htra2	HtrA serine peptidase 2 [Source:MGI Symbol;Acc:M	0.88566
ENSMUSG00000021822	Plau	plasminogen activator, urokinase [Source:MGI Sym	0.88575
ENSMUSG00000035189	Ano4	anoctamin 4 [Source:MGI Symbol;Acc:MGI:244334	0.88578
ENSMUSG00000053025	Sv2b	synaptic vesicle glycoprotein 2 b [Source:MGI Symb	0.88588
ENSMUSG00000005973	Rcn1	reticulocalbin 1 [Source:MGI Symbol;Acc:MGI:1045	0.88589
ENSMUSG00000036111	Lmo1	LIM domain only 1 [Source:MGI Symbol;Acc:MGI:1	0.88593
ENSMUSG00000038633	Degs1	degenerative spermatocyte homolog 1 (Drosophila	0.88603
ENSMUSG00000097030	Gm26693	predicted gene, 26693 [Source:MGI Symbol;Acc:M	0.88609
ENSMUSG00000091722	Siah3	seven in absentia homolog 3 (Drosophila) [Source:M	0.8861

ENSMUSG00000037306	Man1c1	mannosidase, alpha, class 1C, member 1 [Source:M	0.88611
ENSMUSG00000040987	Mill2	MHC I like leukocyte 2 [Source:MGI Symbol;Acc:MG	0.88629
ENSMUSG00000039328	Rnf122	ring finger protein 122 [Source:MGI Symbol;Acc:MG	0.8863
ENSMUSG00000026429	Ube2t	ubiquitin-conjugating enzyme E2T (putative) [Sourc	0.88636
ENSMUSG00000006457	Actn3	actinin alpha 3 [Source:MGI Symbol;Acc:MGI:99678	0.88645
ENSMUSG00000054342	Kcnn4	potassium intermediate/small conductance calcium	0.88647
ENSMUSG00000030116	Mfap5	microfibrillar associated protein 5 [Source:MGI Sym	0.88648
ENSMUSG00000086495	Gm13778	predicted gene 13778 [Source:MGI Symbol;Acc:MG	0.88657
ENSMUSG00000024730	Ms4a8a	membrane-spanning 4-domains, subfamily A, mem	0.88672
ENSMUSG00000038194	Lhb	luteinizing hormone beta [Source:MGI Symbol;Acc:	0.88672
ENSMUSG00000043144	Aqp6	aquaporin 6 [Source:MGI Symbol;Acc:MGI:1341204	0.88698
ENSMUSG00000082364	Gm11955	predicted gene 11955 [Source:MGI Symbol;Acc:MG	0.88699
ENSMUSG00000006711	D130043K22Rik	RIKEN cDNA D130043K22 gene [Source:MGI Symbo	0.88707
ENSMUSG00000090150	Acad11	acyl-Coenzyme A dehydrogenase family, member 1	0.88708
ENSMUSG00000081818	Gm12652	predicted gene 12652 [Source:MGI Symbol;Acc:MG	0.88714
ENSMUSG00000062328	Rpl17	ribosomal protein L17 [Source:MGI Symbol;Acc:MG	0.88719
ENSMUSG00000013787	Ehmt2	euchromatic histone lysine N-methyltransferase 2 [	0.88723
ENSMUSG00000063428	Ddo	D-aspartate oxidase [Source:MGI Symbol;Acc:MGI:	0.88723
ENSMUSG00000060579	Fhit	fragile histidine triad gene [Source:MGI Symbol;Acc	0.88727
ENSMUSG00000070336	Fbxo47	F-box protein 47 [Source:MGI Symbol;Acc:MGI:192	0.88742
ENSMUSG00000052605	Isoc2b	isochorismatase domain containing 2b [Source:MG	0.88746
ENSMUSG00000054203	Ifi205	interferon activated gene 205 [Source:MGI Symbol;	0.88747
ENSMUSG00000053286	Trmt1l	tRNA methyltransferase 1 like [Source:MGI Symbol	0.88768
ENSMUSG00000049173	Myoz3	myozenin 3 [Source:MGI Symbol;Acc:MGI:2179296	0.88792
ENSMUSG00000021127	Zfp36l1	zinc finger protein 36, C3H type-like 1 [Source:MGI	0.88795
ENSMUSG00000049891	Gm7984	predicted gene 7984 [Source:MGI Symbol;Acc:MGI:	0.88801
ENSMUSG00000070427	Il18bp	interleukin 18 binding protein [Source:MGI Symbol;	0.88805
ENSMUSG00000019577	Pdk4	pyruvate dehydrogenase kinase, isoenzyme 4 [Sour	0.88825
ENSMUSG00000018415	Gid4	GID complex subunit 4, VID24 homolog (S. cerevisia	0.88833
ENSMUSG00000019803	Nr2e1	nuclear receptor subfamily 2, group E, member 1 [S	0.88857
ENSMUSG00000020101	4632428N05Rik	RIKEN cDNA 4632428N05 gene [Source:MGI Symbo	0.88857
ENSMUSG00000032301	Psma4	proteasome (prosome, macropain) subunit, alpha t	0.88858
ENSMUSG00000085612	Gm15868	predicted gene 15868 [Source:MGI Symbol;Acc:MG	0.88864
ENSMUSG00000032083	Apoa1	apolipoprotein A-I [Source:MGI Symbol;Acc:MGI:88	0.88865
ENSMUSG00000020486	4-Sep	septin 4 [Source:MGI Symbol;Acc:MGI:1270156]	0.88877
ENSMUSG00000084821	Gm15880	predicted gene 15880 [Source:MGI Symbol;Acc:MG	0.88892
ENSMUSG00000014592	Camta1	calmodulin binding transcription activator 1 [Sourc	0.88896
ENSMUSG00000086894	Gm15708	predicted gene 15708 [Source:MGI Symbol;Acc:MG	0.88897
ENSMUSG00000063659	Zbtb18	zinc finger and BTB domain containing 18 [Source:M	0.88903
ENSMUSG00000097806	Gm6556	predicted gene 6556 [Source:MGI Symbol;Acc:MGI:	0.88916
ENSMUSG00000019710	Mrpl24	mitochondrial ribosomal protein L24 [Source:MGI S	0.88929
ENSMUSG00000055204	Ankrd17	ankyrin repeat domain 17 [Source:MGI Symbol;Acc	0.88933
ENSMUSG00000025967	Eef1b2	eukaryotic translation elongation factor 1 beta 2 [S	0.8894
ENSMUSG00000006932	Ctnnb1	catenin (cadherin associated protein), beta 1 [Sourc	0.88942
ENSMUSG00000033917	Gde1	glycerophosphodiester phosphodiesterase 1 [Sourc	0.88949
ENSMUSG00000087374	Gm15457	predicted gene 15457 [Source:MGI Symbol;Acc:MG	0.8895
ENSMUSG00000035067	Xkr6	X Kell blood group precursor related family membe	0.88959

ENSMUSG00000027384	Ndufaf5	NADH dehydrogenase (ubiquinone) complex I, asse	0.88964
ENSMUSG00000097433	Gm26781	predicted gene, 26781 [Source:MGI Symbol;Acc:MG	0.88971
ENSMUSG00000035901	Dennd5a	DENN/MADD domain containing 5A [Source:MGI Sy	0.88972
ENSMUSG00000089525	Gm23833	predicted gene, 23833 [Source:MGI Symbol;Acc:MG	0.88972
ENSMUSG00000085012	Gm15492	predicted gene 15492 [Source:MGI Symbol;Acc:MG	0.88974
ENSMUSG00000022685	Parn	poly(A)-specific ribonuclease (deadenylation nuclea	0.88975
ENSMUSG00000096932	5033403F01Rik	RIKEN cDNA 5033403F01 gene [Source:MGI Symbo	0.88979
ENSMUSG00000020422	Tns3	tensin 3 [Source:MGI Symbol;Acc:MGI:2443012]	0.88982
ENSMUSG00000081142	Gm15497	predicted gene 15497 [Source:MGI Symbol;Acc:MG	0.88987
ENSMUSG00000097440	Gm6277	predicted gene 6277 [Source:MGI Symbol;Acc:MGI:	0.88987
ENSMUSG00000079445	B3gnt7	UDP-GlcNAc:betaGal beta-1,3-N-acetylglucosaminy	0.88988
ENSMUSG00000058886	Deaf1	deformed epidermal autoregulatory factor 1 (Droso	0.89
ENSMUSG00000084408	Gm11870	predicted gene 11870 [Source:MGI Symbol;Acc:MG	0.89007
ENSMUSG00000037731	Themis2	thymocyte selection associated family member 2 [S	0.89013
ENSMUSG00000085390	Gm13115	predicted gene 13115 [Source:MGI Symbol;Acc:MG	0.89024
ENSMUSG00000056596	Trnp1	TMF1-regulated nuclear protein 1 [Source:MGI Sym	0.89025
ENSMUSG00000028653	Trit1	tRNA isopentenyltransferase 1 [Source:MGI Symbo	0.8903
ENSMUSG00000085925	Rtl1	retrotransposon-like 1 [Source:MGI Symbol;Acc:MG	0.89044
ENSMUSG00000002844	Adprh	ADP-ribosylarginine hydrolase [Source:MGI Symbol	0.8905
ENSMUSG00000049488	Tmem67	transmembrane protein 67 [Source:MGI Symbol;Ac	0.89053
ENSMUSG00000093930	Hmgcs1	3-hydroxy-3-methylglutaryl-Coenzyme A synthase 1	0.89057
ENSMUSG00000084849	Gm16105	predicted gene 16105 [Source:MGI Symbol;Acc:MG	0.8906
ENSMUSG00000040899	Ccr6	chemokine (C-C motif) receptor 6 [Source:MGI Sym	0.89088
ENSMUSG00000055874	Foxi3	forkhead box I3 [Source:MGI Symbol;Acc:MGI:3511	0.89091
ENSMUSG00000022671	Mzt2	mitotic spindle organizing protein 2 [Source:MGI Sy	0.89093
ENSMUSG00000074305	C230081A13Rik	RIKEN cDNA C230081A13 gene [Source:MGI Symbo	0.89094
ENSMUSG00000074874	Ctla2b	cytotoxic T lymphocyte-associated protein 2 beta [S	0.89098
ENSMUSG00000058503	Fam133b	family with sequence similarity 133, member B [Sou	0.89102
ENSMUSG00000015217	Hmgb3	high mobility group box 3 [Source:MGI Symbol;Acc:	0.89108
ENSMUSG00000056899	Immp2l	IMP2 inner mitochondrial membrane peptidase-like	0.89111
ENSMUSG00000095795	Park2	Parkinson disease (autosomal recessive, juvenile) 2	0.89117
ENSMUSG00000032009	Sesn3	sestrin 3 [Source:MGI Symbol;Acc:MGI:1922997]	0.89121
ENSMUSG00000007827	Ankrd26	ankyrin repeat domain 26 [Source:MGI Symbol;Acc	0.89127
ENSMUSG00000057219	Armc7	armadillo repeat containing 7 [Source:MGI Symbol;	0.89139
ENSMUSG00000082596	Gm14227	predicted gene 14227 [Source:MGI Symbol;Acc:MG	0.8914
ENSMUSG00000083397	Gm5466	predicted gene 5466 [Source:MGI Symbol;Acc:MGI:	0.8914
ENSMUSG00000049676	Catsperg1	catsper channel auxiliary subunit gamma 1 [Source	0.89142
ENSMUSG00000000171	Sdhd	succinate dehydrogenase complex, subunit D, integ	0.89146
ENSMUSG00000020052	Ascl1	achaete-scute complex homolog 1 (Drosophila) [So	0.89151
ENSMUSG00000034501	Pcnxl4	pecanex-like 4 (Drosophila) [Source:MGI Symbol;Ac	0.89151
ENSMUSG00000070604	Vsig10l	ZV-set and immunoglobulin domain containing 10 l	0.89152
ENSMUSG00000068921	Dap3	death associated protein 3 [Source:MGI Symbol;Acc	0.89158
ENSMUSG00000017652	Cd40	CD40 antigen [Source:MGI Symbol;Acc:MGI:88336]	0.89165
ENSMUSG00000029192	Tbc1d14	TBC1 domain family, member 14 [Source:MGI Symb	0.89166
ENSMUSG00000033059	Pygb	brain glycogen phosphorylase [Source:MGI Symbol	0.89168
ENSMUSG00000041377	Ninj2	ninjurin 2 [Source:MGI Symbol;Acc:MGI:1352751]	0.89173
ENSMUSG00000025207	Sema4g	sema domain, immunoglobulin domain (Ig), transm	0.89178

ENSMUSG00000058325	Dock1	dedicator of cytokinesis 1 [Source:MGI Symbol;Acc:	0.89186
ENSMUSG00000046159	Chrm3	cholinergic receptor, muscarinic 3, cardiac [Source:	0.89189
ENSMUSG00000040498	Igsf23	immunoglobulin superfamily, member 23 [Source:M	0.89193
ENSMUSG00000022236	Ropn1l	ropporin 1-like [Source:MGI Symbol;Acc:MGI:2182	0.89206
ENSMUSG00000023723	Mrps23	mitochondrial ribosomal protein S23 [Source:MGI S	0.89211
ENSMUSG00000061723	Tnnt3	troponin T3, skeletal, fast [Source:MGI Symbol;Acc:	0.89218
ENSMUSG00000030894	Tpp1	tripeptidyl peptidase I [Source:MGI Symbol;Acc:MG	0.8922
ENSMUSG00000037896	Rcor1	REST corepressor 1 [Source:MGI Symbol;Acc:MGI:1	0.89224
ENSMUSG00000089889	0610040B10Rik	RIKEN cDNA 0610040B10 gene [Source:MGI Symbo	0.89227
ENSMUSG00000041747	Utp15	UTP15, U3 small nucleolar ribonucleoprotein, hom	0.89232
ENSMUSG00000034063	4930590J08Rik	RIKEN cDNA 4930590J08 gene [Source:MGI Symbol	0.89233
ENSMUSG00000038717	Atp5l	ATP synthase, H+ transporting, mitochondrial F0 co	0.89235
ENSMUSG00000000125	Wnt3	wingless-related MMTV integration site 3 [Source:M	0.89257
ENSMUSG00000066568	Lsm14a	LSM14 homolog A (SCD6, S. cerevisiae) [Source:MG	0.89263
ENSMUSG00000028899	Taf12	TAF12 RNA polymerase II, TATA box binding protei	0.89274
ENSMUSG00000028901	Gmeb1	glucocorticoid modulatory element binding protein	0.8928
ENSMUSG00000049411	Tmem241	transmembrane protein 241 [Source:MGI Symbol;A	0.8928
ENSMUSG00000038216	Pnmt	phenylethanolamine-N-methyltransferase [Source:	0.89283
ENSMUSG00000097735	D930032P07Rik	RIKEN cDNA D930032P07 gene [Source:MGI Symbo	0.89283
ENSMUSG00000022450	Ndufa6	NADH dehydrogenase (ubiquinone) 1 alpha subcom	0.89286
ENSMUSG00000006390	Elovl1	elongation of very long chain fatty acids (FEN1/Elo2	0.89297
ENSMUSG00000082969	Gm12630	predicted gene 12630 [Source:MGI Symbol;Acc:MG	0.893
ENSMUSG00000097848	Gm807	predicted gene 807 [Source:MGI Symbol;Acc:MGI:2	0.89303
ENSMUSG00000028423	Nfx1	nuclear transcription factor, X-box binding 1 [Sourc	0.8931
ENSMUSG00000041134	Cyyr1	cysteine and tyrosine-rich protein 1 [Source:MGI Sy	0.89322
ENSMUSG00000010797	Wnt2	wingless-related MMTV integration site 2 [Source:M	0.89325
ENSMUSG00000091558	Gm17227	predicted gene 17227 [Source:MGI Symbol;Acc:MG	0.89333
ENSMUSG00000018965	Ywhah	tyrosine 3-monooxygenase/tryptophan 5-monooxy	0.89338
ENSMUSG00000024190	Dusp1	dual specificity phosphatase 1 [Source:MGI Symbol	0.89344
ENSMUSG00000052707	Tnrc6a	trinucleotide repeat containing 6a [Source:MGI Syn	0.89362
ENSMUSG00000086193	Gm11508	predicted gene 11508 [Source:MGI Symbol;Acc:MG	0.89392
ENSMUSG00000054200	Ffar4	free fatty acid receptor 4 [Source:MGI Symbol;Acc:	0.89398
ENSMUSG00000020581	Agr2	anterior gradient 2 [Source:MGI Symbol;Acc:MGI:1	0.89401
ENSMUSG00000085961	Gm15904	predicted gene 15904 [Source:MGI Symbol;Acc:MG	0.89415
ENSMUSG00000040532	Abhd11	abhydrolase domain containing 11 [Source:MGI Syn	0.89421
ENSMUSG00000029352	Crybb3	crystallin, beta B3 [Source:MGI Symbol;Acc:MGI:10	0.89424
ENSMUSG00000065037	Rn7sk	RNA, 7SK, nuclear [Source:MGI Symbol;Acc:MGI:10	0.89429
ENSMUSG00000087439	Gm15788	predicted gene 15788 [Source:MGI Symbol;Acc:MG	0.89433
ENSMUSG00000033781	Asb13	ankyrin repeat and SOCS box-containing 13 [Source	0.89442
ENSMUSG00000056305	Usp39	ubiquitin specific peptidase 39 [Source:MGI Symbo	0.89442
ENSMUSG00000030516	Tjp1	tight junction protein 1 [Source:MGI Symbol;Acc:M	0.89449
ENSMUSG00000028936	Rpl22	ribosomal protein L22 [Source:MGI Symbol;Acc:MG	0.89452
ENSMUSG00000063354	Slc39a4	solute carrier family 39 (zinc transporter), member	0.89471
ENSMUSG00000023953	Polh	polymerase (DNA directed), eta (RAD 30 related) [S	0.89483
ENSMUSG00000028737	Aldh4a1	aldehyde dehydrogenase 4 family, member A1 [Sou	0.89495
ENSMUSG00000098141	Gm6944	predicted gene 6944 [Source:MGI Symbol;Acc:MGI:	0.895
ENSMUSG00000032128	Robo3	roundabout homolog 3 (Drosophila) [Source:MGI Sy	0.89508

ENSMUSG00000024687	Osbp	oxysterol binding protein [Source:MGI Symbol;Acc:	0.89523
ENSMUSG00000037161	Mgarp	mitochondria localized glutamic acid rich protein [S	0.89528
ENSMUSG00000060538	Tmem219	transmembrane protein 219 [Source:MGI Symbol;A	0.89528
ENSMUSG00000025429	Pstpip2	proline-serine-threonine phosphatase-interacting p	0.89529
ENSMUSG00000024666	Tmem138	transmembrane protein 138 [Source:MGI Symbol;A	0.89536
ENSMUSG00000060808	3110043A19Rik	RIKEN cDNA 3110043A19 gene [Source:MGI Symbo	0.89539
ENSMUSG00000028701	Lurap1	leucine rich adaptor protein 1 [Source:MGI Symbol;	0.89544
ENSMUSG00000074250	Gm10653	predicted gene 10653 [Source:MGI Symbol;Acc:MG	0.89553
ENSMUSG00000030209	Grin2b	glutamate receptor, ionotropic, NMDA2B (epsilon 2	0.89554
ENSMUSG00000079324	4932414N04Rik	RIKEN cDNA 4932414N04 gene [Source:MGI Symbo	0.89566
ENSMUSG00000028688	Toe1	target of EGR1, member 1 (nuclear) [Source:MGI Sy	0.89579
ENSMUSG00000031925	Maml2	mastermind like 2 (Drosophila) [Source:MGI Symbo	0.89581
ENSMUSG00000078684	5830417I10Rik	RIKEN cDNA 5830417I10 gene [Source:MGI Symbol	0.89582
ENSMUSG00000097267	1700109K24Rik	RIKEN cDNA 1700109K24 gene [Source:MGI Symbo	0.89586
ENSMUSG00000038167	Plekhg6	pleckstrin homology domain containing, family G (w	0.89587
ENSMUSG00000096449	Gm4076	predicted gene 4076 [Source:MGI Symbol;Acc:MGI:	0.89591
ENSMUSG00000044216	Kcnj4	potassium inwardly-rectifying channel, subfamily J,	0.89598
ENSMUSG00000046192	Iqub	IQ motif and ubiquitin domain containing [Source:N	0.89618
ENSMUSG00000098132	Rassf10	Ras association (RalGDS/AF-6) domain family (N-ter	0.89629
ENSMUSG00000031877	Ces2g	carboxylesterase 2G [Source:MGI Symbol;Acc:MGI:	0.8963
ENSMUSG00000078780	Gm5150	predicted gene 5150 [Source:MGI Symbol;Acc:MGI:	0.89633
ENSMUSG00000092325	Gm18284	predicted gene, 18284 [Source:MGI Symbol;Acc:MG	0.89634
ENSMUSG00000070532	1700084C01Rik	RIKEN cDNA 1700084C01 gene [Source:MGI Symbo	0.89635
ENSMUSG00000080731	Rps2-ps9	ribosomal protein S2, pseudogene 9 [Source:MGI Sy	0.89645
ENSMUSG00000054364	Rhob	ras homolog gene family, member B [Source:MGI S	0.89651
ENSMUSG00000029640	Usp12	ubiquitin specific peptidase 12 [Source:MGI Symbo	0.89654
ENSMUSG00000017491	Rarb	retinoic acid receptor, beta [Source:MGI Symbol;Ac	0.89659
ENSMUSG00000030844	Rgs10	regulator of G-protein signalling 10 [Source:MGI Sy	0.89669
ENSMUSG00000045761	Fam179a	family with sequence similarity 179, member A [Sou	0.89676
ENSMUSG00000034872	Gipc3	GIPC PDZ domain containing family, member 3 [Sou	0.89685
ENSMUSG00000021241	Isca2	iron-sulfur cluster assembly 2 homolog (S. cerevisia	0.8969
ENSMUSG00000030088	Aldh1l1	aldehyde dehydrogenase 1 family, member L1 [Sou	0.89704
ENSMUSG00000026656	Fcgr2b	Fc receptor, IgG, low affinity IIb [Source:MGI Symbo	0.8971
ENSMUSG00000065343	Gm25580	predicted gene, 25580 [Source:MGI Symbol;Acc:MG	0.89713
ENSMUSG00000097954	Gm4217	predicted gene 4217 [Source:MGI Symbol;Acc:MGI:	0.89713
ENSMUSG00000027263	Tubgcp4	tubulin, gamma complex associated protein 4 [Sour	0.89716
ENSMUSG00000075227	Znhit2	zinc finger, HIT domain containing 2 [Source:MGI Sy	0.89719
ENSMUSG00000034161	Scx	scleraxis [Source:MGI Symbol;Acc:MGI:102934]	0.89727
ENSMUSG00000058013	11-Sep	septin 11 [Source:MGI Symbol;Acc:MGI:1277214]	0.89735
ENSMUSG00000021139	Gm20498	predicted gene 20498 [Source:MGI Symbol;Acc:MG	0.89739
ENSMUSG00000036299	BC031181	cDNA sequence BC031181 [Source:MGI Symbol;Acc	0.89741
ENSMUSG00000055409	Nell1	NEL-like 1 [Source:MGI Symbol;Acc:MGI:2443902]	0.89754
ENSMUSG00000018347	Zkscan6	zinc finger with KRAB and SCAN domains 6 [Source:	0.89763
ENSMUSG00000024039	Cbs	cystathionine beta-synthase [Source:MGI Symbol;A	0.89772
ENSMUSG00000043372	Hexim2	hexamethylene bis-acetamide inducible 2 [Source:N	0.89784
ENSMUSG00000043366	Olfr78	olfactory receptor 78 [Source:MGI Symbol;Acc:MGI	0.89786
ENSMUSG00000066877	Nck2	non-catalytic region of tyrosine kinase adaptor prot	0.89786

ENSMUSG00000006958	Chrd	chordin [Source:MGI Symbol;Acc:MGI:1313268]	0.89787
ENSMUSG00000028545	Bend5	BEN domain containing 5 [Source:MGI Symbol;Acc:	0.89789
ENSMUSG00000037613	Tnfrsf23	tumor necrosis factor receptor superfamily, membe	0.89801
ENSMUSG00000095477	Gm25076	predicted gene, 25076 [Source:MGI Symbol;Acc:M	0.89806
ENSMUSG00000097907	Raxos1	Rax overlapping opposite strand transcript 1 [Sourc	0.89815
ENSMUSG00000081512	Gm15821	predicted gene 15821 [Source:MGI Symbol;Acc:MG	0.89817
ENSMUSG00000029472	Anapc5	anaphase-promoting complex subunit 5 [Source:M	0.89824
ENSMUSG00000079036	Alkbh1	alkB, alkylation repair homolog 1 (E. coli) [Source:M	0.89826
ENSMUSG00000084780	Gm15350	predicted gene 15350 [Source:MGI Symbol;Acc:MG	0.8984
ENSMUSG00000041915	Ammecr1l	AMME chromosomal region gene 1-like [Source:M	0.89851
ENSMUSG00000033450	Tagap	T cell activation Rho GTPase activating protein [Sou	0.89852
ENSMUSG00000036138	Acaa1a	acetyl-Coenzyme A acyltransferase 1A [Source:MGI	0.89867
ENSMUSG00000022822	Abcc5	ATP-binding cassette, sub-family C (CFTR/MRP), me	0.89888
ENSMUSG00000050471	Fam118b	family with sequence similarity 118, member B [Sou	0.89893
ENSMUSG00000085514	9530048J24Rik	RIKEN cDNA 9530048J24 gene [Source:MGI Symbol	0.89896
ENSMUSG00000046034	Fam105b	family with sequence similarity 105, member B [Sou	0.89912
ENSMUSG00000030981	Mmp21	matrix metallopeptidase 21 [Source:MGI Symbol;Ac	0.89921
ENSMUSG00000059796	Eif4a1	eukaryotic translation initiation factor 4A1 [Source:	0.89922
ENSMUSG00000090104	Gm16241	predicted gene 16241 [Source:MGI Symbol;Acc:MG	0.8994
ENSMUSG00000049721	Gal3st1	galactose-3-O-sulfotransferase 1 [Source:MGI Symb	0.89941
ENSMUSG00000020553	Pctp	phosphatidylcholine transfer protein [Source:MGI S	0.89942
ENSMUSG00000053838	Nudcd3	NudC domain containing 3 [Source:MGI Symbol;Acc	0.89957
ENSMUSG00000034110	Kctd7	potassium channel tetramerisation domain contain	0.89977
ENSMUSG00000036241	Ube2r2	ubiquitin-conjugating enzyme E2R 2 [Source:MGI S	0.89978
ENSMUSG00000032547	Ryk	receptor-like tyrosine kinase [Source:MGI Symbol;A	0.89982
ENSMUSG00000041117	Ccdc8	coiled-coil domain containing 8 [Source:MGI Symbo	0.89984
ENSMUSG00000093593	Gm20683	predicted gene 20683 [Source:MGI Symbol;Acc:MG	0.89985
ENSMUSG00000081600	Gm12286	predicted gene 12286 [Source:MGI Symbol;Acc:MG	0.90001
ENSMUSG00000027219	Slc28a2	solute carrier family 28 (sodium-coupled nucleoside	0.90004
ENSMUSG00000067713	Prkag1	protein kinase, AMP-activated, gamma 1 non-cataly	0.90004
ENSMUSG00000024136	Dnase1l2	deoxyribonuclease 1-like 2 [Source:MGI Symbol;Ac	0.90006
ENSMUSG00000047409	Ctdspl	CTD (carboxy-terminal domain, RNA polymerase II,	0.90031
ENSMUSG00000096141	Dnah7a	dynein, axonemal, heavy chain 7A [Source:MGI Sym	0.90032
ENSMUSG00000061911	Myt1l	myelin transcription factor 1-like [Source:MGI Symb	0.90036
ENSMUSG00000039384	Dusp10	dual specificity phosphatase 10 [Source:MGI Symbo	0.90038
ENSMUSG00000046997	Spsb4	splA/ryanodine receptor domain and SOCS box con	0.90042
ENSMUSG00000046523	Kctd4	potassium channel tetramerisation domain contain	0.90043
ENSMUSG00000026087	Mrpl30	mitochondrial ribosomal protein L30 [Source:MGI S	0.90062
ENSMUSG00000069072	Slc7a14	solute carrier family 7 (cationic amino acid transpo	0.90067
ENSMUSG00000012396	Nanog	Nanog homeobox [Source:MGI Symbol;Acc:MGI:19	0.90071
ENSMUSG00000036931	Nfkbid	nuclear factor of kappa light polypeptide gene enha	0.90074
ENSMUSG00000020038	Cry1	cryptochrome 1 (photolyase-like) [Source:MGI Sym	0.90084
ENSMUSG00000039068	Zzz3	zinc finger, ZZ domain containing 3 [Source:MGI Sy	0.9009
ENSMUSG00000097927	Gm6999	predicted gene 6999 [Source:MGI Symbol;Acc:MGI:	0.901
ENSMUSG00000044328	Trp53i13	transformation related protein 53 inducible protein	0.90105
ENSMUSG00000039313	AF529169	cDNA sequence AF529169 [Source:MGI Symbol;Acc	0.90114
ENSMUSG00000041396	Mettl18	methyltransferase like 18 [Source:MGI Symbol;Acc:	0.90128

ENSMUSG00000021226	Acot2	acyl-CoA thioesterase 2 [Source:MGI Symbol;Acc:M	0.9013
ENSMUSG00000044252	Osbpl1a	oxysterol binding protein-like 1A [Source:MGI Symb	0.90163
ENSMUSG00000092212	Slc22a13b-ps	solute carrier family 22 (organic cation transporter)	0.9017
ENSMUSG00000023947	Nfkbie	nuclear factor of kappa light polypeptide gene enha	0.90177
ENSMUSG00000039037	St6galnac5	ST6 (alpha-N-acetyl-neuraminyl-2,3-beta-galactosyl	0.90192
ENSMUSG00000042761	Mrap2	melanocortin 2 receptor accessory protein 2 [Sourc	0.90205
ENSMUSG00000029781	Fkbp9	FK506 binding protein 9 [Source:MGI Symbol;Acc:M	0.90208
ENSMUSG00000036961	Wnt8b	wingless related MMTV integration site 8b [Source:	0.90225
ENSMUSG00000038718	Pbx3	pre B cell leukemia homeobox 3 [Source:MGI Symb	0.90235
ENSMUSG00000027368	Dusp2	dual specificity phosphatase 2 [Source:MGI Symbol	0.90242
ENSMUSG00000086816	Gm16726	predicted gene, 16726 [Source:MGI Symbol;Acc:MC	0.9025
ENSMUSG00000030323	Ift122	intraflagellar transport 122 [Source:MGI Symbol;Ac	0.90256
ENSMUSG00000034906	Ncaph	non-SMC condensin I complex, subunit H [Source:M	0.90257
ENSMUSG00000024209	1700061G19Rik	RIKEN cDNA 1700061G19 gene [Source:MGI Symbo	0.90264
ENSMUSG00000024370	Cdc23	CDC23 cell division cycle 23 [Source:MGI Symbol;Ac	0.90287
ENSMUSG00000086454	4930500J02Rik	RIKEN cDNA 4930500J02 gene [Source:MGI Symbol	0.90291
ENSMUSG00000034259	Exosc4	exosome component 4 [Source:MGI Symbol;Acc:M	0.90293
ENSMUSG00000026974	Zmynd19	zinc finger, MYND domain containing 19 [Source:M	0.90301
ENSMUSG00000063458	1700112E06Rik	RIKEN cDNA 1700112E06 gene [Source:MGI Symbo	0.90305
ENSMUSG00000026670	Uap1	UDP-N-acetylglucosamine pyrophosphorylase 1 [So	0.90307
ENSMUSG00000027330	Cdc25b	cell division cycle 25B [Source:MGI Symbol;Acc:MG	0.90314
ENSMUSG00000051007	Pddc1	Parkinson disease 7 domain containing 1 [Source:M	0.90318
ENSMUSG00000028794	A3galt2	alpha 1,3-galactosyltransferase 2 (isoglobotriaosylc	0.90322
ENSMUSG00000085241	Snhg3	small nucleolar RNA host gene (non-protein coding	0.90326
ENSMUSG00000034602	Mon2	MON2 homolog (yeast) [Source:MGI Symbol;Acc:M	0.90328
ENSMUSG00000031539	Ap3m2	adaptor-related protein complex 3, mu 2 subunit [S	0.90336
ENSMUSG00000021374	Nup153	nucleoporin 153 [Source:MGI Symbol;Acc:MGI:238	0.90339
ENSMUSG00000089710	Gm15810	predicted gene 15810 [Source:MGI Symbol;Acc:MG	0.90339
ENSMUSG00000041020	Map7d2	MAP7 domain containing 2 [Source:MGI Symbol;Ac	0.90341
ENSMUSG00000095730	Vmn2r29	vomeronasal 2, receptor 29 [Source:MGI Symbol;A	0.90348
ENSMUSG00000097276	4930525G20Rik	RIKEN cDNA 4930525G20 gene [Source:MGI Symbo	0.90351
ENSMUSG00000029427	Zcchc8	zinc finger, CCHC domain containing 8 [Source:MGI	0.9038
ENSMUSG00000020257	Wdr82	WD repeat domain containing 82 [Source:MGI Sym	0.90383
ENSMUSG00000033233	Trim45	tripartite motif-containing 45 [Source:MGI Symbol;	0.90393
ENSMUSG00000027208	Fgf7	fibroblast growth factor 7 [Source:MGI Symbol;Acc	0.90394
ENSMUSG00000022044	Stmn4	stathmin-like 4 [Source:MGI Symbol;Acc:MGI:1931	0.90406
ENSMUSG00000027363	Usp8	ubiquitin specific peptidase 8 [Source:MGI Symbol;	0.90406
ENSMUSG00000020640	Itsn2	intersectin 2 [Source:MGI Symbol;Acc:MGI:133804	0.90423
ENSMUSG00000024869	Nudt8	nudix (nucleoside diphosphate linked moiety X)-typ	0.90424
ENSMUSG00000043668	Tox3	TOX high mobility group box family member 3 [Sou	0.90433
ENSMUSG00000078937	Cpt1b	carnitine palmitoyltransferase 1b, muscle [Source:N	0.90435
ENSMUSG00000054477	Kcnn2	potassium intermediate/small conductance calcium	0.90436
ENSMUSG00000001366	Fbxo9	f-box protein 9 [Source:MGI Symbol;Acc:MGI:1918	0.9045
ENSMUSG00000087054	Gm12405	predicted gene 12405 [Source:MGI Symbol;Acc:MG	0.90457
ENSMUSG00000072572	Slc39a2	solute carrier family 39 (zinc transporter), member	0.90459
ENSMUSG00000019301	Hsd17b1	hydroxysteroid (17-beta) dehydrogenase 1 [Source	0.90471
ENSMUSG00000034563	Ccpg1	cell cycle progression 1 [Source:MGI Symbol;Acc:M	0.90472

ENSMUSG00000028923	Necap2	NECAP endocytosis associated 2 [Source:MGI Symb	0.90482
ENSMUSG00000036036	Zfp57	zinc finger protein 57 [Source:MGI Symbol;Acc:MGI	0.90501
ENSMUSG00000060657	Marf1	meiosis arrest female 1 [Source:MGI Symbol;Acc:M	0.90502
ENSMUSG00000047361	Gm973	predicted gene 973 [Source:MGI Symbol;Acc:MGI:2	0.90504
ENSMUSG00000059208	Hnrnpm	heterogeneous nuclear ribonucleoprotein M [Sourc	0.90506
ENSMUSG00000029059	Fam213b	family with sequence similarity 213, member B [Sou	0.90512
ENSMUSG00000052889	Prkcb	protein kinase C, beta [Source:MGI Symbol;Acc:MG	0.90513
ENSMUSG00000060862	Zbtb40	zinc finger and BTB domain containing 40 [Source:M	0.90522
ENSMUSG00000096924	Gm26824	predicted gene, 26824 [Source:MGI Symbol;Acc:MG	0.90522
ENSMUSG00000074797	Itpa	inosine triphosphatase (nucleoside triphosphate py	0.90531
ENSMUSG00000081305	Gm12879	predicted gene 12879 [Source:MGI Symbol;Acc:MG	0.90548
ENSMUSG00000001134	Uxt	ubiquitously expressed transcript [Source:MGI Sym	0.9055
ENSMUSG00000037370	Enpp1	ectonucleotide pyrophosphatase/phosphodiesteras	0.90561
ENSMUSG00000076433	BC100451	cDNA sequence BC100451 [Source:MGI Symbol;Acc	0.90562
ENSMUSG00000025314	Ptprj	protein tyrosine phosphatase, receptor type, J [Sou	0.90567
ENSMUSG00000073460	Pnldc1	poly(A)-specific ribonuclease (PARN)-like domain co	0.90573
ENSMUSG00000040631	Dok4	docking protein 4 [Source:MGI Symbol;Acc:MGI:21	0.90574
ENSMUSG00000081850	Gm15667	predicted gene 15667 [Source:MGI Symbol;Acc:MG	0.906
ENSMUSG00000017615	Tnfaip1	tumor necrosis factor, alpha-induced protein 1 (end	0.90604
ENSMUSG00000032122	Slc37a2	solute carrier family 37 (glycerol-3-phosphate trans	0.90613
ENSMUSG00000028417	Tal2	T cell acute lymphocytic leukemia 2 [Source:MGI Sy	0.9063
ENSMUSG00000038943	Prc1	protein regulator of cytokinesis 1 [Source:MGI Sym	0.9063
ENSMUSG00000028271	Gtf2b	general transcription factor IIB [Source:MGI Symbo	0.90654
ENSMUSG00000079177	Fam228a	family with sequence similarity 228, member A [So	0.90654
ENSMUSG00000090118	Gm16163	predicted gene 16163 [Source:MGI Symbol;Acc:MG	0.90655
ENSMUSG00000085372	Gm16145	predicted gene 16145 [Source:MGI Symbol;Acc:MG	0.90663
ENSMUSG00000037872	Darc	Duffy blood group, chemokine receptor [Source:MG	0.90664
ENSMUSG00000041215	Yeats2	YEATS domain containing 2 [Source:MGI Symbol;Ac	0.90676
ENSMUSG00000047490	4932411E22Rik	RIKEN cDNA 4932411E22 gene [Source:MGI Symbo	0.90676
ENSMUSG00000000131	Xpo6	exportin 6 [Source:MGI Symbol;Acc:MGI:2429950]	0.90678
ENSMUSG00000051367	Six1	sine oculis-related homeobox 1 [Source:MGI Symbo	0.90682
ENSMUSG00000030792	Dkkl1	dickkopf-like 1 [Source:MGI Symbol;Acc:MGI:13549	0.90692
ENSMUSG00000026616	Cr2	complement receptor 2 [Source:MGI Symbol;Acc:M	0.907
ENSMUSG00000042046	Dstyk	dual serine/threonine and tyrosine protein kinase [	0.907
ENSMUSG00000030825	Hsd17b14	hydroxysteroid (17-beta) dehydrogenase 14 [Sourc	0.90702
ENSMUSG00000031722	Hp	haptoglobin [Source:MGI Symbol;Acc:MGI:96211]	0.90711
ENSMUSG00000021514	Zfp369	zinc finger protein 369 [Source:MGI Symbol;Acc:MG	0.90718
ENSMUSG00000005907	Pex1	peroxisomal biogenesis factor 1 [Source:MGI Symb	0.90735
ENSMUSG00000085069	Gm13111	predicted gene 13111 [Source:MGI Symbol;Acc:MG	0.90737
ENSMUSG00000006920	Ezh1	enhancer of zeste homolog 1 (Drosophila) [Source:I	0.90739
ENSMUSG00000034951	Cog7	component of oligomeric golgi complex 7 [Source:M	0.90743
ENSMUSG00000004707	Ly9	lymphocyte antigen 9 [Source:MGI Symbol;Acc:MG	0.90747
ENSMUSG00000021124	Vti1b	vesicle transport through interaction with t-SNAREs	0.90757
ENSMUSG00000030450	Oca2	oculocutaneous albinism II [Source:MGI Symbol;Acc	0.90759
ENSMUSG00000036751	Cox6b1	cytochrome c oxidase, subunit VIb polypeptide 1 [S	0.90775
ENSMUSG00000022365	Derl1	Der1-like domain family, member 1 [Source:MGI Sy	0.90777
ENSMUSG00000010142	Tnfrsf13b	tumor necrosis factor receptor superfamily, membe	0.90797

ENSMUSG00000039031	Arhgap18	Rho GTPase activating protein 18 [Source:MGI Sym	0.90799
ENSMUSG00000031536	Polb	polymerase (DNA directed), beta [Source:MGI Symb	0.90813
ENSMUSG00000017386	Traf4	TNF receptor associated factor 4 [Source:MGI Symb	0.90824
ENSMUSG00000051663	Pcdhb1	protocadherin beta 1 [Source:MGI Symbol;Acc:MGI	0.90827
ENSMUSG00000040888	Gfer	growth factor, erv1 (S. cerevisiae)-like (augmenter	0.90828
ENSMUSG00000021286	Zfyve21	zinc finger, FYVE domain containing 21 [Source:MG	0.90848
ENSMUSG00000041075	Fzd7	frizzled homolog 7 (Drosophila) [Source:MGI Symbo	0.90855
ENSMUSG00000097928	Gm26578	predicted gene, 26578 [Source:MGI Symbol;Acc:MG	0.90855
ENSMUSG00000055125	M5C1000I18Rik	RIKEN cDNA M5C1000I18 gene [Source:MGI Symbo	0.90861
ENSMUSG00000021591	Glrx	glutaredoxin [Source:MGI Symbol;Acc:MGI:213562	0.90866
ENSMUSG00000071341	Egr4	early growth response 4 [Source:MGI Symbol;Acc:N	0.90869
ENSMUSG00000072244	Trim6	tripartite motif-containing 6 [Source:MGI Symbol;A	0.90872
ENSMUSG00000059049	Frem1	Fras1 related extracellular matrix protein 1 [Source	0.90911
ENSMUSG00000081788	Gm5898	predicted gene 5898 [Source:MGI Symbol;Acc:MGI:	0.90923
ENSMUSG00000062077	Trim54	tripartite motif-containing 54 [Source:MGI Symbol;	0.90929
ENSMUSG00000078440	Dohh	deoxyhypusine hydroxylase/monooxygenase [Sour	0.90944
ENSMUSG00000037110	Ralgapa2	Ral GTPase activating protein, alpha subunit 2 (cata	0.90963
ENSMUSG00000074507	Gm14340	predicted gene 14340 [Source:MGI Symbol;Acc:MG	0.90978
ENSMUSG00000037712	Fermt2	fermitin family homolog 2 (Drosophila) [Source:MG	0.9098
ENSMUSG00000022489	Pde1b	phosphodiesterase 1B, Ca2+-calmodulin dependen	0.90991
ENSMUSG00000028093	Acp6	acid phosphatase 6, lysophosphatidic [Source:MGI	0.90993
ENSMUSG00000097378	B230208H11Rik	RIKEN cDNA B230208H11 gene [Source:MGI Symbo	0.91
ENSMUSG00000036928	Stag3	stromal antigen 3 [Source:MGI Symbol;Acc:MGI:13	0.91012
ENSMUSG00000032715	Trib3	tribbles homolog 3 (Drosophila) [Source:MGI Symb	0.91032
ENSMUSG00000029263	Pigg	phosphatidylinositol glycan anchor biosynthesis, cla	0.91036
ENSMUSG00000048602	Morc2b	microrchidia 2B [Source:MGI Symbol;Acc:MGI:3045	0.91037
ENSMUSG00000014355	Anapc1	anaphase promoting complex subunit 1 [Source:M	0.91052
ENSMUSG00000001383	Zmat2	zinc finger, matrin type 2 [Source:MGI Symbol;Acc:	0.91069
ENSMUSG00000084861	1700095J07Rik	RIKEN cDNA 1700095J07 gene [Source:MGI Symbol	0.91072
ENSMUSG00000079547	H2-DMb1	histocompatibility 2, class II, locus Mb1 [Source:MG	0.9108
ENSMUSG00000029504	Ddx51	DEAD (Asp-Glu-Ala-Asp) box polypeptide 51 [Sourc	0.91086
ENSMUSG00000047117	Ankdd1b	ankyrin repeat and death domain containing 1B [So	0.91086
ENSMUSG00000087651	1500009L16Rik	RIKEN cDNA 1500009L16 gene [Source:MGI Symbo	0.91087
ENSMUSG00000043505	Gimap5	GTPase, IMAP family member 5 [Source:MGI Symb	0.91099
ENSMUSG00000069996	Gm7125	predicted pseudogene 7125 [Source:MGI Symbol;A	0.91104
ENSMUSG00000085765	Gm12333	predicted gene 12333 [Source:MGI Symbol;Acc:MG	0.91108
ENSMUSG00000031109	Enox2	ecto-NOX disulfide-thiol exchanger 2 [Source:MGI S	0.91112
ENSMUSG00000056895	Hist3h2ba	histone cluster 3, H2ba [Source:MGI Symbol;Acc:M	0.91116
ENSMUSG00000039358	Drd5	dopamine receptor D5 [Source:MGI Symbol;Acc:M	0.91131
ENSMUSG00000042015	Wdr41	WD repeat domain 41 [Source:MGI Symbol;Acc:MG	0.91144
ENSMUSG00000049323	Smcr8	Smith-Magenis syndrome chromosome region, can	0.91151
ENSMUSG00000029530	Ccr9	chemokine (C-C motif) receptor 9 [Source:MGI Sym	0.91157
ENSMUSG00000032849	Abcc4	ATP-binding cassette, sub-family C (CFTR/MRP), me	0.91158
ENSMUSG00000097638	4933439G12Rik	RIKEN cDNA 4933439G12 gene [Source:MGI Symbo	0.91162
ENSMUSG00000069893	9930111J21Rik1	RIKEN cDNA 9930111J21 gene 1 [Source:MGI Symb	0.91163
ENSMUSG00000055523	Gucy2g	guanylate cyclase 2g [Source:MGI Symbol;Acc:MGI:	0.91172
ENSMUSG00000029217	Tec	tec protein tyrosine kinase [Source:MGI Symbol;Ac	0.91181

ENSMUSG00000087611	4930458D05Rik	RIKEN cDNA 4930458D05 gene [Source:MGI Symbo	0.91196
ENSMUSG00000048755	Mcat	malonyl CoA:ACP acyltransferase (mitochondrial) [S	0.91213
ENSMUSG00000085672	Gm16193	predicted gene 16193 [Source:MGI Symbol;Acc:MG	0.91218
ENSMUSG00000033126	Ybey	ybeY metallopeptidase [Source:MGI Symbol;Acc:M	0.91228
ENSMUSG00000027635	Dsn1	DSN1, MIND kinetochore complex component, hon	0.91247
ENSMUSG00000029503	P2rx2	purinergic receptor P2X, ligand-gated ion channel,	0.91253
ENSMUSG00000034612	Chst11	carbohydrate sulfotransferase 11 [Source:MGI Sym	0.91253
ENSMUSG00000087344	Gm15627	predicted gene 15627 [Source:MGI Symbol;Acc:MG	0.91262
ENSMUSG00000029999	Tgfa	transforming growth factor alpha [Source:MGI Sym	0.91279
ENSMUSG00000056904	Gm5620	predicted gene 5620 [Source:MGI Symbol;Acc:MGI	0.91305
ENSMUSG00000033318	Gstt2	glutathione S-transferase, theta 2 [Source:MGI Sym	0.91311
ENSMUSG00000038522	AI317395	expressed sequence AI317395 [Source:MGI Symbol	0.91312
ENSMUSG00000061603	Akap6	A kinase (PRKA) anchor protein 6 [Source:MGI Sym	0.91335
ENSMUSG00000028359	Orm3	orosomucoid 3 [Source:MGI Symbol;Acc:MGI:9744	0.91336
ENSMUSG00000017858	Ift52	intraflagellar transport 52 [Source:MGI Symbol;Acc	0.9134
ENSMUSG00000047084	Ngrn	neugrin, neurite outgrowth associated [Source:MG	0.91369
ENSMUSG00000083816	Gm13033	predicted gene 13033 [Source:MGI Symbol;Acc:MG	0.91377
ENSMUSG00000037606	Osbpl5	oxysterol binding protein-like 5 [Source:MGI Symbo	0.9138
ENSMUSG00000039652	Cpeb3	cytoplasmic polyadenylation element binding prote	0.91386
ENSMUSG00000069170	Gpr98	G protein-coupled receptor 98 [Source:MGI Symbo	0.91397
ENSMUSG00000079437	Tmem179b	transmembrane protein 179B [Source:MGI Symbol;	0.91407
ENSMUSG00000019992	Mtfr2	mitochondrial fission regulator 2 [Source:MGI Symb	0.9141
ENSMUSG00000002007	Srpk3	serine/arginine-rich protein specific kinase 3 [Sourc	0.91429
ENSMUSG00000026928	Card9	caspase recruitment domain family, member 9 [Sou	0.9143
ENSMUSG00000024497	Pou4f3	POU domain, class 4, transcription factor 3 [Source	0.91434
ENSMUSG00000085669	Gm14817	predicted gene 14817 [Source:MGI Symbol;Acc:MG	0.91435
ENSMUSG00000097303	3110083C13Rik	RIKEN cDNA 3110083C13 gene [Source:MGI Symbo	0.91436
ENSMUSG00000036377	C530008M17Rik	RIKEN cDNA C530008M17 gene [Source:MGI Symb	0.91448
ENSMUSG00000004637	Wwox	WW domain-containing oxidoreductase [Source:M	0.91454
ENSMUSG00000090061	3110047P20Rik	RIKEN cDNA 3110047P20 gene [Source:MGI Symbo	0.91459
ENSMUSG00000011486	Slc25a41	solute carrier family 25, member 41 [Source:MGI S	0.91463
ENSMUSG00000028017	Egf	epidermal growth factor [Source:MGI Symbol;Acc:M	0.9147
ENSMUSG00000023008	Fmnl3	formin-like 3 [Source:MGI Symbol;Acc:MGI:109569	0.91483
ENSMUSG00000000902	Smarcb1	SWI/SNF related, matrix associated, actin depender	0.91489
ENSMUSG00000063179	Pstk	phosphoseryl-tRNA kinase [Source:MGI Symbol;Acc	0.91489
ENSMUSG00000067724	Gbx1	gastrulation brain homeobox 1 [Source:MGI Symbo	0.91492
ENSMUSG00000020621	Rdh14	retinol dehydrogenase 14 (all-trans and 9-cis) [Sour	0.91507
ENSMUSG00000031802	Phxr4	per-hexamer repeat gene 4 [Source:MGI Symbol;Ac	0.91507
ENSMUSG00000038496	Slc19a3	solute carrier family 19, member 3 [Source:MGI Sy	0.91509
ENSMUSG00000038582	Pptc7	PTC7 protein phosphatase homolog (S. cerevisiae)	0.91511
ENSMUSG00000024645	Timm21	tranlocase of inner mitochondrial membrane 21 [So	0.91532
ENSMUSG00000024042	Sik1	salt inducible kinase 1 [Source:MGI Symbol;Acc:MG	0.91541
ENSMUSG00000030125	Lrrc23	leucine rich repeat containing 23 [Source:MGI Syml	0.91555
ENSMUSG00000023153	Tmem52	transmembrane protein 52 [Source:MGI Symbol;Ac	0.91571
ENSMUSG00000029621	Arpc1a	actin related protein 2/3 complex, subunit 1A [Sour	0.91574
ENSMUSG00000021468	Sptlc1	serine palmitoyltransferase, long chain base subuni	0.91587
ENSMUSG00000022771	Ppil2	peptidylprolyl isomerase (cyclophilin)-like 2 [Source	0.91596

ENSMUSG00000025184	R3hcc1l	R3H domain and coiled-coil containing 1 like [Sourc	0.91604
ENSMUSG00000028614	Ndc1	NDC1 transmembrane nucleoporin [Source:MGI Sy	0.91608
ENSMUSG00000085363	Gm15478	predicted gene 15478 [Source:MGI Symbol;Acc:MG	0.91608
ENSMUSG00000094724	Rnaset2b	ribonuclease T2B [Source:MGI Symbol;Acc:MGI:370	0.91608
ENSMUSG00000078439	2210404O07Rik	RIKEN cDNA 2210404O07 gene [Source:MGI Symbo	0.9161
ENSMUSG00000057841	Rpl32	ribosomal protein L32 [Source:MGI Symbol;Acc:MG	0.91611
ENSMUSG00000035370	Adat3	adenosine deaminase, tRNA-specific 3 [Source:MGI	0.91612
ENSMUSG00000090248	Gm14027	predicted gene 14027 [Source:MGI Symbol;Acc:MG	0.91613
ENSMUSG00000042055	Wdr11	WD repeat domain 11 [Source:MGI Symbol;Acc:MG	0.91619
ENSMUSG00000030577	Cd22	CD22 antigen [Source:MGI Symbol;Acc:MGI:88322]	0.91628
ENSMUSG00000053964	Lgals4	lectin, galactose binding, soluble 4 [Source:MGI Syr	0.91632
ENSMUSG00000032118	Fez1	fasciculation and elongation protein zeta 1 (zygin I)	0.91635
ENSMUSG00000020023	Tmcc3	transmembrane and coiled coil domains 3 [Source:	0.9165
ENSMUSG00000035107	Dcbld2	discoidin, CUB and LCCL domain containing 2 [Sour	0.91664
ENSMUSG00000031667	Aktip	thymoma viral proto-oncogene 1 interacting protei	0.91677
ENSMUSG00000085354	Gm2044	predicted gene 2044 [Source:MGI Symbol;Acc:MGI	0.91677
ENSMUSG00000038664	Herc1	hect (homologous to the E6-AP (UBE3A) carboxyl te	0.91681
ENSMUSG00000040466	Blvrb	biliverdin reductase B (flavin reductase (NADPH)) [S	0.91686
ENSMUSG00000053559	Smagp	small cell adhesion glycoprotein [Source:MGI Symb	0.91707
ENSMUSG00000027284	Cdan1	congenital dyserythropoietic anemia, type I (humar	0.91711
ENSMUSG00000041975	Mettl8	methyltransferase like 8 [Source:MGI Symbol;Acc:N	0.91717
ENSMUSG00000014547	Wdfy2	WD repeat and FYVE domain containing 2 [Source:N	0.91723
ENSMUSG00000002017	Fam98a	family with sequence similarity 98, member A [Sour	0.91731
ENSMUSG00000034413	Neurl1b	neuralized homolog 1b (Drosophila) [Source:MGI Sy	0.91741
ENSMUSG00000043157	Arl11	ADP-ribosylation factor-like 11 [Source:MGI Symbo	0.91753
ENSMUSG00000053181	A830005F24Rik	RIKEN cDNA A830005F24 gene [Source:MGI Symbo	0.91753
ENSMUSG00000024787	Snx15	sorting nexin 15 [Source:MGI Symbol;Acc:MGI:191	0.9176
ENSMUSG00000042992	Loh12cr1	loss of heterozygosity, 12, chromosomal region 1 h	0.91761
ENSMUSG00000013997	Nit1	nitrilase 1 [Source:MGI Symbol;Acc:MGI:1350916]	0.91763
ENSMUSG00000031706	Rfx1	regulatory factor X, 1 (influences HLA class II expres	0.91764
ENSMUSG00000035142	Nubpl	nucleotide binding protein-like [Source:MGI Symbo	0.91764
ENSMUSG00000031575	Ash2l	ash2 (absent, small, or homeotic)-like (Drosophila)	0.91766
ENSMUSG00000038058	Nod1	nucleotide-binding oligomerization domain contain	0.91768
ENSMUSG00000037300	Ttc13	tetratricopeptide repeat domain 13 [Source:MGI Sy	0.91773
ENSMUSG00000074766	Ism1	isthmin 1 homolog (zebrafish) [Source:MGI Symbol	0.91773
ENSMUSG00000054204	Fam150b	family with sequence similarity 150, member B [Sou	0.9178
ENSMUSG00000096199	Ptrhd1	peptidyl-tRNA hydrolase domain containing 1 [Sour	0.9179
ENSMUSG00000037638	Zbtb42	zinc finger and BTB domain containing 42 [Source:N	0.91798
ENSMUSG00000021488	Nsd1	nuclear receptor-binding SET-domain protein 1 [So	0.91804
ENSMUSG00000030730	Atp2a1	ATPase, Ca++ transporting, cardiac muscle, fast twi	0.91814
ENSMUSG00000091313	Fth-ps2	ferritin heavy chain, pseudogene 2 [Source:MGI Syr	0.91825
ENSMUSG00000047033	Pcdhb15	protocadherin beta 15 [Source:MGI Symbol;Acc:MG	0.91864
ENSMUSG00000052915	Msl1	male-specific lethal 1 homolog (Drosophila) [Source	0.91876
ENSMUSG00000034442	Trmt5	TRM5 tRNA methyltransferase 5 [Source:MGI Symb	0.91879
ENSMUSG00000086424	Gm15569	predicted gene 15569 [Source:MGI Symbol;Acc:MG	0.91882
ENSMUSG00000045136	Tubb2b	tubulin, beta 2B class IIB [Source:MGI Symbol;Acc:N	0.91883
ENSMUSG00000023043	Krt18	keratin 18 [Source:MGI Symbol;Acc:MGI:96692]	0.91884

ENSMUSG00000031521	Aga	aspartylglucosaminidase [Source:MGI Symbol;Acc:N	0.9189
ENSMUSG00000047539	Fbxo28	F-box protein 28 [Source:MGI Symbol;Acc:MGI:126	0.91906
ENSMUSG00000049396	Gemin4	gem (nuclear organelle) associated protein 4 [Sourc	0.91906
ENSMUSG00000043003	Rasef	RAS and EF hand domain containing [Source:MGI Sy	0.91911
ENSMUSG00000068744	Psrc1	proline/serine-rich coiled-coil 1 [Source:MGI Symbo	0.91916
ENSMUSG00000067336	Bmpr2	bone morphogenetic protein receptor, type II (serin	0.91917
ENSMUSG00000034601	2700049A03Rik	RIKEN cDNA 2700049A03 gene [Source:MGI Symbo	0.91931
ENSMUSG00000005886	Ncoa2	nuclear receptor coactivator 2 [Source:MGI Symbol	0.91932
ENSMUSG00000073073	Gm8098	predicted gene 8098 [Source:MGI Symbol;Acc:MGI:	0.91933
ENSMUSG00000054150	Syne3	spectrin repeat containing, nuclear envelope family	0.91957
ENSMUSG00000081909	Gm11341	predicted gene 11341 [Source:MGI Symbol;Acc:MG	0.91959
ENSMUSG00000031750	Il34	interleukin 34 [Source:MGI Symbol;Acc:MGI:19237	0.91979
ENSMUSG00000019874	Fabp7	fatty acid binding protein 7, brain [Source:MGI Sym	0.9198
ENSMUSG00000000339	Rtca	RNA 3'-terminal phosphate cyclase [Source:MGI Sy	0.91993
ENSMUSG00000085555	2610035F20Rik	RIKEN cDNA 2610035F20 gene [Source:MGI Symbo	0.91997
ENSMUSG00000028459	Cd72	CD72 antigen [Source:MGI Symbol;Acc:MGI:88345]	0.92004
ENSMUSG00000087445	Gm14286	predicted gene 14286 [Source:MGI Symbol;Acc:MG	0.92017
ENSMUSG00000021846	Peli2	pellino 2 [Source:MGI Symbol;Acc:MGI:1891445]	0.92018
ENSMUSG00000069804	Gm10277	predicted gene 10277 [Source:MGI Symbol;Acc:MG	0.92023
ENSMUSG00000085886	D030047H15Rik	RIKEN cDNA D030047H15 gene [Source:MGI Symbo	0.92024
ENSMUSG00000095276	Gm581	predicted gene 581 [Source:MGI Symbol;Acc:MGI:2	0.92032
ENSMUSG00000020181	Nav3	neuron navigator 3 [Source:MGI Symbol;Acc:MGI:2	0.92054
ENSMUSG00000047394	Odf3b	outer dense fiber of sperm tails 3B [Source:MGI Syr	0.92059
ENSMUSG00000057286	St6galnac2	ST6 (alpha-N-acetyl-neuraminyl-2,3-beta-galactosyl	0.92067
ENSMUSG00000036427	Gpi1	glucose phosphate isomerase 1 [Source:MGI Symbo	0.92075
ENSMUSG00000029101	Rgs12	regulator of G-protein signaling 12 [Source:MGI Syr	0.92088
ENSMUSG00000038871	Bpgm	2,3-bisphosphoglycerate mutase [Source:MGI Symb	0.92089
ENSMUSG00000016018	Skiv2l2	superkiller viralicidic activity 2-like 2 (S. cerevisiae)	0.92098
ENSMUSG00000024168	Tmem204	transmembrane protein 204 [Source:MGI Symbol;A	0.92114
ENSMUSG00000031841	Cdh13	cadherin 13 [Source:MGI Symbol;Acc:MGI:99551]	0.92134
ENSMUSG00000086477	Gm15506	predicted gene 15506 [Source:MGI Symbol;Acc:MG	0.92136
ENSMUSG00000081194	Gm8424	predicted gene 8424 [Source:MGI Symbol;Acc:MGI:	0.92137
ENSMUSG00000022468	Endou	endonuclease, polyU-specific [Source:MGI Symbol;	0.92144
ENSMUSG00000081516	Gm12470	predicted gene 12470 [Source:MGI Symbol;Acc:MG	0.92155
ENSMUSG00000032998	Foxj3	forkhead box J3 [Source:MGI Symbol;Acc:MGI:2443	0.92164
ENSMUSG00000039109	F13a1	coagulation factor XIII, A1 subunit [Source:MGI Sym	0.92164
ENSMUSG00000064652	Gm24165	predicted gene, 24165 [Source:MGI Symbol;Acc:MG	0.92167
ENSMUSG00000018001	Cyth3	cytohesin 3 [Source:MGI Symbol;Acc:MGI:1335107	0.92206
ENSMUSG00000015879	Fam184b	family with sequence similarity 184, member B [Sou	0.92213
ENSMUSG00000037994	Slc9b2	solute carrier family 9, subfamily B (NHA2, cation p	0.92217
ENSMUSG00000090207	4930524O07Rik	RIKEN cDNA 4930524O07 gene [Source:MGI Symbo	0.92217
ENSMUSG00000048271	Rbm33	RNA binding motif protein 33 [Source:MGI Symbol;	0.92222
ENSMUSG00000048503	Tmem136	transmembrane protein 136 [Source:MGI Symbol;A	0.92232
ENSMUSG00000042707	Dnali1	dynein, axonemal, light intermediate polypeptide 1	0.92234
ENSMUSG00000055833	1700034H15Rik	RIKEN cDNA 1700034H15 gene [Source:MGI Symbo	0.92238
ENSMUSG00000085962	Gm16984	predicted gene, 16984 [Source:MGI Symbol;Acc:MG	0.92242
ENSMUSG00000061132	Blnk	B cell linker [Source:MGI Symbol;Acc:MGI:96878]	0.92266

ENSMUSG00000025439	Clns1a	chloride channel, nucleotide-sensitive, 1A [Source:I	0.92279
ENSMUSG00000081996	Gm7153	predicted gene 7153 [Source:MGI Symbol;Acc:MGI:	0.92283
ENSMUSG00000049091	Sephs2	selenophosphate synthetase 2 [Source:MGI Symbo	0.92292
ENSMUSG00000030270	Cpne9	copine family member IX [Source:MGI Symbol;Acc:	0.92293
ENSMUSG00000024642	Tle4	transducin-like enhancer of split 4, homolog of Dro	0.92294
ENSMUSG00000097869	C230021G24Rik	RIKEN cDNA C230021G24 gene [Source:MGI Symbo	0.92299
ENSMUSG00000034379	Wdr5b	WD repeat domain 5B [Source:MGI Symbol;Acc:MG	0.92309
ENSMUSG00000022848	Dirc2	disrupted in renal carcinoma 2 (human) [Source:MG	0.92311
ENSMUSG00000022808	Snx4	sorting nexin 4 [Source:MGI Symbol;Acc:MGI:19164	0.92315
ENSMUSG00000005687	Bcas2	breast carcinoma amplified sequence 2 [Source:MG	0.92324
ENSMUSG00000002477	Snrpd1	small nuclear ribonucleoprotein D1 [Source:MGI Sy	0.92354
ENSMUSG00000019984	Med23	mediator complex subunit 23 [Source:MGI Symbol;	0.92357
ENSMUSG00000026321	Tnfrsf11a	tumor necrosis factor receptor superfamily, membe	0.92364
ENSMUSG00000012519	Mlkl	mixed lineage kinase domain-like [Source:MGI Sym	0.92371
ENSMUSG00000031490	Eif4ebp1	eukaryotic translation initiation factor 4E binding p	0.92371
ENSMUSG00000038949	Cnst	consortin, connexin sorting protein [Source:MGI Sy	0.92381
ENSMUSG00000039763	Dnajc28	DnaJ (Hsp40) homolog, subfamily C, member 28 [So	0.92391
ENSMUSG00000073889	Il11ra1	interleukin 11 receptor, alpha chain 1 [Source:MGI	0.92392
ENSMUSG00000069917	Hba-a2	hemoglobin alpha, adult chain 2 [Source:MGI Symb	0.92393
ENSMUSG00000027361	Gabpb1	GA repeat binding protein, beta 1 [Source:MGI Sym	0.92395
ENSMUSG00000027312	Atrn	attractin [Source:MGI Symbol;Acc:MGI:1341628]	0.92397
ENSMUSG00000035547	Capn5	calpain 5 [Source:MGI Symbol;Acc:MGI:1100859]	0.92402
ENSMUSG00000054051	Ercc6	excision repair cross-complementing rodent repair	0.92405
ENSMUSG00000028671	Gale	galactose-4-epimerase, UDP [Source:MGI Symbol;A	0.92418
ENSMUSG00000075389	2810410L24Rik	RIKEN cDNA 2810410L24 gene [Source:MGI Symbo	0.92437
ENSMUSG00000054957	Gm9959	predicted gene 9959 [Source:MGI Symbol;Acc:MGI:	0.92444
ENSMUSG00000069763	Tmem100	transmembrane protein 100 [Source:MGI Symbol;A	0.92444
ENSMUSG00000033356	Pus7l	pseudouridylate synthase 7 homolog (S. cerevisiae)	0.92451
ENSMUSG00000017667	Zfp334	zinc finger protein 334 [Source:MGI Symbol;Acc:MG	0.92452
ENSMUSG00000050440	Hamp	hepcidin antimicrobial peptide [Source:MGI Symbo	0.92456
ENSMUSG00000029141	Slc4a1ap	solute carrier family 4 (anion exchanger), member 1	0.92458
ENSMUSG00000066438	Plekhd1	pleckstrin homology domain containing, family D (w	0.9246
ENSMUSG00000087497	2810001G20Rik	RIKEN cDNA 2810001G20 gene [Source:MGI Symbo	0.9247
ENSMUSG00000091277	Gm1818	predicted gene 1818 [Source:MGI Symbol;Acc:MGI:	0.92475
ENSMUSG00000021993	Mipep	mitochondrial intermediate peptidase [Source:MGI	0.92515
ENSMUSG00000030347	D6Wsu163e	DNA segment, Chr 6, Wayne State University 163, e	0.92519
ENSMUSG00000031963	Bmper	BMP-binding endothelial regulator [Source:MGI Syr	0.92535
ENSMUSG00000052957	Gas1	growth arrest specific 1 [Source:MGI Symbol;Acc:M	0.92536
ENSMUSG00000038569	Rad9b	RAD9 homolog B [Source:MGI Symbol;Acc:MGI:238	0.92547
ENSMUSG00000041258	Zfp236	zinc finger protein 236 [Source:MGI Symbol;Acc:MG	0.92557
ENSMUSG00000089944	Gm8292	predicted gene 8292 [Source:MGI Symbol;Acc:MGI:	0.92586
ENSMUSG00000030254	Rad18	RAD18 homolog (S. cerevisiae) [Source:MGI Symbo	0.92588
ENSMUSG00000025102	3110040N11Rik	RIKEN cDNA 3110040N11 gene [Source:MGI Symbo	0.92593
ENSMUSG00000028654	Mycl	v-myc myelocytomatosis viral oncogene homolog, l	0.92594
ENSMUSG00000039936	Pik3cd	phosphatidylinositol 3-kinase catalytic delta polype	0.92618
ENSMUSG00000022364	Tbc1d31	TBC1 domain family, member 31 [Source:MGI Symb	0.92619
ENSMUSG00000040364	Sec1	secretory blood group 1 [Source:MGI Symbol;Acc:M	0.92625

ENSMUSG00000005320	Fgfr4	fibroblast growth factor receptor 4 [Source:MGI Sy	0.92635
ENSMUSG00000029408	Abcb9	ATP-binding cassette, sub-family B (MDR/TAP), mer	0.92638
ENSMUSG00000045034	Ankrd34b	ankyrin repeat domain 34B [Source:MGI Symbol;Ac	0.92649
ENSMUSG00000085213	Gm13091	predicted gene 13091 [Source:MGI Symbol;Acc:MG	0.92651
ENSMUSG00000078491	Gm13090	predicted gene 13090 [Source:MGI Symbol;Acc:MG	0.92654
ENSMUSG00000034940	Synrg	synergin, gamma [Source:MGI Symbol;Acc:MGI:135	0.92667
ENSMUSG00000081769	Gm12216	predicted gene 12216 [Source:MGI Symbol;Acc:MG	0.92678
ENSMUSG00000027409	1700020A23Rik	RIKEN cDNA 1700020A23 gene [Source:MGI Symbo	0.92684
ENSMUSG00000000296	Tpd52l1	tumor protein D52-like 1 [Source:MGI Symbol;Acc:	0.92687
ENSMUSG00000079662	Ntn3	netrin 3 [Source:MGI Symbol;Acc:MGI:1341188]	0.92703
ENSMUSG00000048895	Cdk5r1	cyclin-dependent kinase 5, regulatory subunit 1 (p3	0.92718
ENSMUSG00000023952	Gtpbp2	GTP binding protein 2 [Source:MGI Symbol;Acc:MG	0.92719
ENSMUSG00000097198	9330159N05Rik	RIKEN cDNA 9330159N05 gene [Source:MGI Symbo	0.92733
ENSMUSG00000029233	Srd5a3	steroid 5 alpha-reductase 3 [Source:MGI Symbol;Ac	0.92738
ENSMUSG00000068099	1500009C09Rik	RIKEN cDNA 1500009C09 gene [Source:MGI Symbo	0.92738
ENSMUSG00000055210	Foxd2	forkhead box D2 [Source:MGI Symbol;Acc:MGI:134	0.92741
ENSMUSG00000079505	Gm11131	predicted gene 11131 [Source:MGI Symbol;Acc:MG	0.92759
ENSMUSG00000041775	Mapk1ip1	mitogen-activated protein kinase 1 interacting prot	0.92764
ENSMUSG00000085505	Gm12868	predicted gene 12868 [Source:MGI Symbol;Acc:MG	0.92768
ENSMUSG00000056204	Pgpep1	pyroglutamyl-peptidase I [Source:MGI Symbol;Acc:	0.92777
ENSMUSG00000053329	D10Jhu81e	DNA segment, Chr 10, Johns Hopkins University 81	0.92781
ENSMUSG00000069678	Pcgf1	polycomb group ring finger 1 [Source:MGI Symbol;	0.92793
ENSMUSG00000022203	Efs	embryonal Fyn-associated substrate [Source:MGI S	0.92797
ENSMUSG00000039046	Usp6nl	USP6 N-terminal like [Source:MGI Symbol;Acc:MGI	0.92812
ENSMUSG00000020226	Slc5a4b	solute carrier family 5 (neutral amino acid transpor	0.92814
ENSMUSG00000021338	Lrrc16a	leucine rich repeat containing 16A [Source:MGI Syr	0.9283
ENSMUSG00000046826	Fam187b	family with sequence similarity 187, member B [Sou	0.92832
ENSMUSG00000025509	Pnpla2	patatin-like phospholipase domain containing 2 [So	0.92851
ENSMUSG00000031266	Gla	galactosidase, alpha [Source:MGI Symbol;Acc:MGI:	0.92858
ENSMUSG00000025911	Adhfe1	alcohol dehydrogenase, iron containing, 1 [Source:	0.92862
ENSMUSG00000022053	Ebf2	early B cell factor 2 [Source:MGI Symbol;Acc:MGI:8	0.92874
ENSMUSG00000034154	Ino80	INO80 homolog (S. cerevisiae) [Source:MGI Symbol	0.92879
ENSMUSG00000021423	Ly86	lymphocyte antigen 86 [Source:MGI Symbol;Acc:M	0.92892
ENSMUSG00000072770	Acrbp	proacrosin binding protein [Source:MGI Symbol;Ac	0.92917
ENSMUSG00000097090	Gm26724	predicted gene, 26724 [Source:MGI Symbol;Acc:M	0.92923
ENSMUSG00000031574	Star	steroidogenic acute regulatory protein [Source:MG	0.92925
ENSMUSG00000044534	Ackr2	atypical chemokine receptor 2 [Source:MGI Symbol	0.92928
ENSMUSG00000049526	Tmem202	transmembrane protein 202 [Source:MGI Symbol;A	0.92942
ENSMUSG00000029337	Fgf5	fibroblast growth factor 5 [Source:MGI Symbol;Acc	0.92945
ENSMUSG00000029771	Irf5	interferon regulatory factor 5 [Source:MGI Symbol;	0.92946
ENSMUSG00000062421	Arf2	ADP-ribosylation factor 2 [Source:MGI Symbol;Acc:	0.92947
ENSMUSG00000022856	Tmem41a	transmembrane protein 41a [Source:MGI Symbol;A	0.92965
ENSMUSG00000044340	Phlpp1	PH domain and leucine rich repeat protein phospha	0.9297
ENSMUSG00000080797	Gm15760	predicted gene 15760 [Source:MGI Symbol;Acc:MG	0.92973
ENSMUSG00000063445	Nmral1	NmrA-like family domain containing 1 [Source:MGI	0.92974
ENSMUSG00000032059	Alg9	asparagine-linked glycosylation 9 (alpha 1,2 manno	0.9298
ENSMUSG00000097443	Gm17529	predicted gene, 17529 [Source:MGI Symbol;Acc:M	0.92986

ENSMUSG00000026014	Raph1	Ras association (RalGDS/AF-6) and pleckstrin homo	0.92988
ENSMUSG00000068154	Insm1	insulinoma-associated 1 [Source:MGI Symbol;Acc:M	0.92988
ENSMUSG00000093587	Gm20554	predicted gene, 20554 [Source:MGI Symbol;Acc:MG	0.92993
ENSMUSG00000085687	Gm16153	predicted gene 16153 [Source:MGI Symbol;Acc:MG	0.92999
ENSMUSG00000097147	Gm26780	predicted gene, 26780 [Source:MGI Symbol;Acc:MG	0.93006
ENSMUSG00000031700	Gpt2	glutamic pyruvate transaminase (alanine aminotran	0.93018
ENSMUSG00000028330	Ncbp1	nuclear cap binding protein subunit 1 [Source:MGI	0.9303
ENSMUSG00000000290	Itgb2	integrin beta 2 [Source:MGI Symbol;Acc:MGI:9661	0.93031
ENSMUSG00000057229	Atp5sl	ATP5S-like [Source:MGI Symbol;Acc:MGI:1913599]	0.93032
ENSMUSG00000008482	Rnf151	ring finger protein 151 [Source:MGI Symbol;Acc:MG	0.93042
ENSMUSG00000073627	C130036L24Rik	RIKEN cDNA C130036L24 gene [Source:MGI Symbo	0.93047
ENSMUSG00000041278	Ttc1	tetratricopeptide repeat domain 1 [Source:MGI Sym	0.93056
ENSMUSG00000049130	C5ar1	complement component 5a receptor 1 [Source:MG	0.93061
ENSMUSG00000057156	Homez	homeodomain leucine zipper-encoding gene [Sourc	0.93098
ENSMUSG00000029830	Svopl	SV2 related protein homolog (rat)-like [Source:MGI	0.93116
ENSMUSG00000020801	Med31	mediator of RNA polymerase II transcription, subun	0.93117
ENSMUSG00000044835	Ankrd45	ankyrin repeat domain 45 [Source:MGI Symbol;Acc	0.93122
ENSMUSG00000096553	Gm10097	predicted gene 10097 [Source:MGI Symbol;Acc:MG	0.93126
ENSMUSG00000003206	Ebi3	Epstein-Barr virus induced gene 3 [Source:MGI Sym	0.93131
ENSMUSG00000063260	Syt10	synaptotagmin X [Source:MGI Symbol;Acc:MGI:185	0.93135
ENSMUSG00000024991	Eif3a	eukaryotic translation initiation factor 3, subunit A	0.93138
ENSMUSG00000033728	Lrrc14	leucine rich repeat containing 14 [Source:MGI Syml	0.9315
ENSMUSG00000097561	4632411P08Rik	RIKEN cDNA 4632411P08 gene [Source:MGI Symbo	0.93156
ENSMUSG00000028553	Angptl3	angiopoietin-like 3 [Source:MGI Symbol;Acc:MGI:1	0.93162
ENSMUSG00000020115	Tbk1	TANK-binding kinase 1 [Source:MGI Symbol;Acc:MG	0.93168
ENSMUSG00000000399	Ndufa9	NADH dehydrogenase (ubiquinone) 1 alpha subcom	0.93173
ENSMUSG00000020659	Cbll1	Casitas B-lineage lymphoma-like 1 [Source:MGI Syn	0.93198
ENSMUSG00000047635	2810006K23Rik	RIKEN cDNA 2810006K23 gene [Source:MGI Symbo	0.93221
ENSMUSG00000096687	AA474331	expressed sequence AA474331 [Source:MGI Symbo	0.93223
ENSMUSG00000021192	Golga5	golgi autoantigen, golgin subfamily a, 5 [Source:MG	0.93224
ENSMUSG00000008167	Fbxw9	F-box and WD-40 domain protein 9 [Source:MGI Sy	0.93234
ENSMUSG00000098022	Zfp82	zinc finger protein 82 [Source:MGI Symbol;Acc:MGI	0.93234
ENSMUSG00000004415	Col26a1	collagen, type XXVI, alpha 1 [Source:MGI Symbol;A	0.93247
ENSMUSG00000087501	Gm12116	predicted gene 12116 [Source:MGI Symbol;Acc:MG	0.93248
ENSMUSG00000098128	Gm3693	predicted gene 3693 [Source:MGI Symbol;Acc:MGI	0.93254
ENSMUSG00000000881	Dlg3	discs, large homolog 3 (Drosophila) [Source:MGI Sy	0.93272
ENSMUSG00000021943	Gdf10	growth differentiation factor 10 [Source:MGI Symb	0.93274
ENSMUSG00000051136	Ghsr	growth hormone secretagogue receptor [Source:M	0.93283
ENSMUSG00000034292	Traf3ip1	TRAF3 interacting protein 1 [Source:MGI Symbol;A	0.93309
ENSMUSG00000036923	Stox1	storkhead box 1 [Source:MGI Symbol;Acc:MGI:268	0.93333
ENSMUSG00000085111	Ascl4	achaete-scute complex homolog 4 (Drosophila) [So	0.93337
ENSMUSG00000033501	Crygs	crystallin, gamma S [Source:MGI Symbol;Acc:MGI:1	0.93339
ENSMUSG00000097813	A830035O19Rik	RIKEN cDNA A830035O19 gene [Source:MGI Symbo	0.93339
ENSMUSG00000040675	Mthfd1l	methylenetetrahydrofolate dehydrogenase (NADP-	0.93345
ENSMUSG00000089760	D030046N08Rik	RIKEN cDNA D030046N08 gene [Source:MGI Symbo	0.9336
ENSMUSG00000027048	Abcb11	ATP-binding cassette, sub-family B (MDR/TAP), me	0.93368
ENSMUSG00000009995	Taz	tafazzin [Source:MGI Symbol;Acc:MGI:109626]	0.93388

ENSMUSG00000008540	Mgst1	microsomal glutathione S-transferase 1 [Source:MG	0.93389
ENSMUSG00000082936	Gm16166	predicted gene 16166 [Source:MGI Symbol;Acc:MG	0.93411
ENSMUSG00000079051	Gm14025	predicted gene 14025 [Source:MGI Symbol;Acc:MG	0.93425
ENSMUSG00000042289	Hsd3b7	hydroxy-delta-5-steroid dehydrogenase, 3 beta- an	0.9343
ENSMUSG00000021361	Tmem14c	transmembrane protein 14C [Source:MGI Symbol;A	0.93433
ENSMUSG00000046750	BC089491	cDNA sequence BC089491 [Source:MGI Symbol;Acc	0.93437
ENSMUSG00000035578	Iqcg	IQ motif containing G [Source:MGI Symbol;Acc:MG	0.9344
ENSMUSG00000020973	Dnaaf2	dynein, axonemal assembly factor 2 [Source:MGI Sy	0.93441
ENSMUSG00000045328	Cenpe	centromere protein E [Source:MGI Symbol;Acc:MG	0.93464
ENSMUSG00000052262	Prpmp5	proline-rich protein MP5 [Source:MGI Symbol;Acc:I	0.93464
ENSMUSG00000032435	Dync1li1	dynein cytoplasmic 1 light intermediate chain 1 [So	0.93471
ENSMUSG00000097531	Gm26591	predicted gene, 26591 [Source:MGI Symbol;Acc:MG	0.93486
ENSMUSG00000034157	2310044G17Rik	RIKEN cDNA 2310044G17 gene [Source:MGI Symbo	0.93488
ENSMUSG00000026980	Ly75	lymphocyte antigen 75 [Source:MGI Symbol;Acc:M	0.93489
ENSMUSG00000022724	Mina	myc induced nuclear antigen [Source:MGI Symbol;A	0.93496
ENSMUSG00000046605	B3gntl1	UDP-GlcNAc:betaGal beta-1,3-N-acetylglucosaminy	0.93498
ENSMUSG00000036585	Fgf1	fibroblast growth factor 1 [Source:MGI Symbol;Acc	0.93511
ENSMUSG00000039518	Cdsn	corneodesmosin [Source:MGI Symbol;Acc:MGI:350	0.93516
ENSMUSG00000013539	Tango2	transport and golgi organization 2 [Source:MGI Sym	0.93523
ENSMUSG00000063919	Srrm4	serine/arginine repetitive matrix 4 [Source:MGI Syr	0.93523
ENSMUSG00000040856	Dlk1	delta-like 1 homolog (Drosophila) [Source:MGI Sym	0.9354
ENSMUSG00000047067	Dusp28	dual specificity phosphatase 28 [Source:MGI Symbo	0.9354
ENSMUSG00000064302	Clasp1	CLIP associating protein 1 [Source:MGI Symbol;Acc:	0.93542
ENSMUSG00000042050	Wdr60	WD repeat domain 60 [Source:MGI Symbol;Acc:MG	0.93546
ENSMUSG00000097622	A330033J07Rik	RIKEN cDNA A330033J07 gene [Source:MGI Symbo	0.93546
ENSMUSG00000097816	Gm26811	predicted gene, 26811 [Source:MGI Symbol;Acc:MG	0.93559
ENSMUSG00000053839	Gm9924	predicted gene 9924 [Source:MGI Symbol;Acc:MGI:	0.93563
ENSMUSG00000053110	Yap1	yes-associated protein 1 [Source:MGI Symbol;Acc:N	0.93565
ENSMUSG00000016995	Matn4	matrilin 4 [Source:MGI Symbol;Acc:MGI:1328314]	0.93592
ENSMUSG00000096991	Gm26789	predicted gene, 26789 [Source:MGI Symbol;Acc:MG	0.93608
ENSMUSG00000046182	Gsg1l	GSG1-like [Source:MGI Symbol;Acc:MGI:2685483]	0.93621
ENSMUSG00000004035	Gstm7	glutathione S-transferase, mu 7 [Source:MGI Symb	0.93653
ENSMUSG00000059540	Tcea2	transcription elongation factor A (SII), 2 [Source:MG	0.93656
ENSMUSG00000026288	Inpp5d	inositol polyphosphate-5-phosphatase D [Source:M	0.93662
ENSMUSG00000045441	Gprin3	GPRIN family member 3 [Source:MGI Symbol;Acc:N	0.93662
ENSMUSG00000049134	Nrap	nebulin-related anchoring protein [Source:MGI Sym	0.93667
ENSMUSG00000020131	Pcsk4	proprotein convertase subtilisin/kexin type 4 [Sour	0.93671
ENSMUSG00000033594	Spata2l	spermatogenesis associated 2-like [Source:MGI Syn	0.93672
ENSMUSG00000043448	Gjc2	gap junction protein, gamma 2 [Source:MGI Symbo	0.93675
ENSMUSG00000097760	6030442K20Rik	RIKEN cDNA 6030442K20 gene [Source:MGI Symbo	0.93689
ENSMUSG00000026504	Sdccag8	serologically defined colon cancer antigen 8 [Sourc	0.93692
ENSMUSG00000096472	Cdkn2d	cyclin-dependent kinase inhibitor 2D (p19, inhibits	0.93706
ENSMUSG00000034187	Nsf	N-ethylmaleimide sensitive fusion protein [Source:I	0.9372
ENSMUSG00000030051	Aplf	aprataxin and PNKP like factor [Source:MGI Symbol	0.93739
ENSMUSG00000029461	Fam168a	family with sequence similarity 168, member A [Sou	0.93753
ENSMUSG00000079559	Gm684	predicted gene 684 [Source:MGI Symbol;Acc:MGI:2	0.93775
ENSMUSG00000030493	C230052I12Rik	RIKEN cDNA C230052I12 gene [Source:MGI Symbol	0.93776

ENSMUSG00000040540	Gm9770	predicted gene 9770 [Source:MGI Symbol;Acc:MGI:	0.93792
ENSMUSG00000029064	Gnb1	guanine nucleotide binding protein (G protein), bet	0.93799
ENSMUSG00000032330	Cox7a2	cytochrome c oxidase subunit VIIa 2 [Source:MGI Sy	0.93799
ENSMUSG00000091512	Lamtor3	late endosomal/lysosomal adaptor, MAPK and MTO	0.93804
ENSMUSG00000097000	Gm17435	predicted gene, 17435 [Source:MGI Symbol;Acc:MG	0.93809
ENSMUSG00000063564	Col23a1	collagen, type XXIII, alpha 1 [Source:MGI Symbol;Ac	0.93811
ENSMUSG00000036432	Siah2	seven in absentia 2 [Source:MGI Symbol;Acc:MGI:1	0.93813
ENSMUSG00000055184	Fam72a	family with sequence similarity 72, member A [Sou	0.93814
ENSMUSG00000027560	Dok5	docking protein 5 [Source:MGI Symbol;Acc:MGI:19	0.93815
ENSMUSG00000056938	Acbd4	acyl-Coenzyme A binding domain containing 4 [Sou	0.93817
ENSMUSG00000025316	Banp	BTG3 associated nuclear protein [Source:MGI Symb	0.9382
ENSMUSG00000074466	Gm15417	predicted gene 15417 [Source:MGI Symbol;Acc:MG	0.93851
ENSMUSG00000084898	Gm12371	predicted gene 12371 [Source:MGI Symbol;Acc:MG	0.93882
ENSMUSG00000019785	Clvs2	clavesin 2 [Source:MGI Symbol;Acc:MGI:2443223]	0.93917
ENSMUSG00000073406	H2-Bl	histocompatibility 2, blastocyst [Source:MGI Symbo	0.9392
ENSMUSG00000025909	Sntg1	syntrophin, gamma 1 [Source:MGI Symbol;Acc:MGI	0.93927
ENSMUSG00000095332	Gm9821	predicted gene 9821 [Source:MGI Symbol;Acc:MGI:	0.93928
ENSMUSG00000090290	Gm17296	predicted gene, 17296 [Source:MGI Symbol;Acc:MG	0.93931
ENSMUSG00000054793	Cadm4	cell adhesion molecule 4 [Source:MGI Symbol;Acc:M	0.93933
ENSMUSG00000019080	Mfsd3	major facilitator superfamily domain containing 3 [S	0.93935
ENSMUSG00000074807	Gm10762	predicted gene 10762 [Source:MGI Symbol;Acc:MG	0.93935
ENSMUSG00000083889	E530001F21Rik	RIKEN cDNA E530001F21 gene [Source:MGI Symbo	0.93936
ENSMUSG00000024985	Tcf7l2	transcription factor 7 like 2, T cell specific, HMG bo	0.93948
ENSMUSG00000024044	Epb4.1l3	erythrocyte protein band 4.1-like 3 [Source:MGI Sy	0.93949
ENSMUSG00000089998	A130049A11Rik	RIKEN cDNA A130049A11 gene [Source:MGI Symbo	0.93963
ENSMUSG00000028894	Inpp5b	inositol polyphosphate-5-phosphatase B [Source:M	0.9398
ENSMUSG00000042240	Crybb2	crystallin, beta B2 [Source:MGI Symbol;Acc:MGI:88	0.93999
ENSMUSG00000037017	Zscan21	zinc finger and SCAN domain containing 21 [Source	0.94011
ENSMUSG00000061175	Fnip2	folliculin interacting protein 2 [Source:MGI Symbol;	0.94012
ENSMUSG00000009876	Cox4i2	cytochrome c oxidase subunit IV isoform 2 [Source:	0.94015
ENSMUSG00000029153	Ociad2	OCIA domain containing 2 [Source:MGI Symbol;Acc	0.9402
ENSMUSG00000018821	Avpi1	arginine vasopressin-induced 1 [Source:MGI Symbo	0.94024
ENSMUSG00000085419	Gm11734	predicted gene 11734 [Source:MGI Symbol;Acc:MG	0.9403
ENSMUSG00000020327	Fgf22	fibroblast growth factor 22 [Source:MGI Symbol;Ac	0.9404
ENSMUSG00000015085	Entpd2	ectonucleoside triphosphate diphosphohydrolase 2	0.94051
ENSMUSG00000029463	Fam216a	family with sequence similarity 216, member A [So	0.94055
ENSMUSG00000074497	A430078G23Rik	RIKEN cDNA A430078G23 gene [Source:MGI Symbo	0.9406
ENSMUSG00000078762	Haus5	HAUS augmin-like complex, subunit 5 [Source:MGI	0.94067
ENSMUSG00000026433	Rab7l1	RAB7, member RAS oncogene family-like 1 [Source	0.94076
ENSMUSG00000033540	Idua	iduronidase, alpha-L- [Source:MGI Symbol;Acc:MGI	0.94079
ENSMUSG00000025735	Rhbdl1	rhomboid, veinlet-like 1 (Drosophila) [Source:MGI S	0.94092
ENSMUSG00000087368	BC065397	cDNA sequence BC065397 [Source:MGI Symbol;Acc	0.94093
ENSMUSG00000033080	Vsx1	visual system homeobox 1 homolog (zebrafish) [So	0.94121
ENSMUSG00000073617	Hdac4	histone deacetylase 4 [Source:MGI Symbol;Acc:MG	0.94122
ENSMUSG00000042766	Trim46	tripartite motif-containing 46 [Source:MGI Symbol;	0.9413
ENSMUSG00000025017	Pik3ap1	phosphoinositide-3-kinase adaptor protein 1 [Sourc	0.94137
ENSMUSG00000056612	Ppp1r14b	protein phosphatase 1, regulatory (inhibitor) subun	0.94138

ENSMUSG00000068551	Zfp467	zinc finger protein 467 [Source:MGI Symbol;Acc:MG	0.94139
ENSMUSG00000014195	Dnajc7	DnaJ (Hsp40) homolog, subfamily C, member 7 [Sou	0.94141
ENSMUSG00000097979	Gm4691	predicted gene 4691 [Source:MGI Symbol;Acc:MGI:	0.94147
ENSMUSG00000047557	Lxn	latexin [Source:MGI Symbol;Acc:MGI:107633]	0.94153
ENSMUSG00000058979	Cecr5	cat eye syndrome chromosome region, candidate 5	0.94163
ENSMUSG00000036775	Decr2	2-4-dienoyl-Coenzyme A reductase 2, peroxisomal	0.94168
ENSMUSG00000038884	A230050P20Rik	RIKEN cDNA A230050P20 gene [Source:MGI Symbo	0.94175
ENSMUSG00000086486	2310043D23Rik	RIKEN cDNA 2310043D23 gene [Source:MGI Symbo	0.94175
ENSMUSG00000022883	Robo1	roundabout homolog 1 (Drosophila) [Source:MGI Sy	0.94183
ENSMUSG00000032051	Fdx1	ferredoxin 1 [Source:MGI Symbol;Acc:MGI:103224]	0.94187
ENSMUSG00000089670	Gm16581	predicted gene 16581 [Source:MGI Symbol;Acc:MG	0.94199
ENSMUSG00000033993	1700018A14Rik	RIKEN cDNA 1700018A14 gene [Source:MGI Symbo	0.94202
ENSMUSG00000018654	Ikzf1	IKAROS family zinc finger 1 [Source:MGI Symbol;Ac	0.94213
ENSMUSG00000025178	Pi4k2a	phosphatidylinositol 4-kinase type 2 alpha [Source:	0.94229
ENSMUSG00000017417	Plxdc1	plexin domain containing 1 [Source:MGI Symbol;Ac	0.94253
ENSMUSG00000027845	Dclre1b	DNA cross-link repair 1B, PSO2 homolog (S. cerevisi	0.9426
ENSMUSG00000020857	Nme2	NME/NM23 nucleoside diphosphate kinase 2 [Sour	0.94265
ENSMUSG00000026385	Dbi	diazepam binding inhibitor [Source:MGI Symbol;Ac	0.94285
ENSMUSG00000085852	Gm13807	predicted gene 13807 [Source:MGI Symbol;Acc:MG	0.94294
ENSMUSG00000071451	Psmg4	proteasome (prosome, macropain) assembly chape	0.94299
ENSMUSG00000003934	Efnb3	ephrin B3 [Source:MGI Symbol;Acc:MGI:109196]	0.94308
ENSMUSG00000002032	Tmem25	transmembrane protein 25 [Source:MGI Symbol;Ac	0.94322
ENSMUSG00000032908	Sgpp2	sphingosine-1-phosphate phosphotase 2 [Source:M	0.94334
ENSMUSG00000034209	Rasl10a	RAS-like, family 10, member A [Source:MGI Symbol	0.94365
ENSMUSG00000047330	Kcne4	potassium voltage-gated channel, Isk-related subfa	0.94387
ENSMUSG00000045282	Tmem86b	transmembrane protein 86B [Source:MGI Symbol;A	0.94399
ENSMUSG00000071036	Gm10309	predicted gene 10309 [Source:MGI Symbol;Acc:MG	0.94421
ENSMUSG00000041798	Gck	glucokinase [Source:MGI Symbol;Acc:MGI:1270854	0.94428
ENSMUSG00000051323	Pcdh19	protocadherin 19 [Source:MGI Symbol;Acc:MGI:268	0.94441
ENSMUSG00000032101	Ddx25	DEAD (Asp-Glu-Ala-Asp) box polypeptide 25 [Sourc	0.94453
ENSMUSG00000040482	Dxo	decapping exoribonuclease [Source:MGI Symbol;Ac	0.94488
ENSMUSG00000019831	Wasf1	WAS protein family, member 1 [Source:MGI Symbo	0.9451
ENSMUSG00000005968	Tuft1	tuftelin 1 [Source:MGI Symbol;Acc:MGI:109572]	0.94519
ENSMUSG00000019850	Tnfaip3	tumor necrosis factor, alpha-induced protein 3 [Sou	0.94519
ENSMUSG00000034684	Sema3f	sema domain, immunoglobulin domain (Ig), short b	0.94524
ENSMUSG00000025044	Msr1	macrophage scavenger receptor 1 [Source:MGI Syn	0.94536
ENSMUSG00000021215	Net1	neuroepithelial cell transforming gene 1 [Source:M	0.9454
ENSMUSG00000010830	Kdelr3	KDEL (Lys-Asp-Glu-Leu) endoplasmic reticulum prot	0.9455
ENSMUSG00000019947	Arid5b	AT rich interactive domain 5B (MRF1-like) [Source:M	0.9455
ENSMUSG00000028040	Efna4	ephrin A4 [Source:MGI Symbol;Acc:MGI:106643]	0.94558
ENSMUSG00000078700	D030028A08Rik	RIKEN cDNA D030028A08 gene [Source:MGI Symbo	0.94584
ENSMUSG00000074284	Gm10658	predicted gene 10658 [Source:MGI Symbol;Acc:MG	0.94588
ENSMUSG00000060843	Ctnna3	catenin (cadherin associated protein), alpha 3 [Sou	0.946
ENSMUSG00000090564	A430057M04Rik	RIKEN cDNA A430057M04 gene [Source:MGI Symb	0.94608
ENSMUSG00000062006	Rpl34	ribosomal protein L34 [Source:MGI Symbol;Acc:MG	0.94619
ENSMUSG00000030979	Uros	uroporphyrinogen III synthase [Source:MGI Symbol	0.9465
ENSMUSG00000007041	Clic1	chloride intracellular channel 1 [Source:MGI Symbo	0.94679

ENSMUSG00000029600	1110008J03Rik	RIKEN cDNA 1110008J03 gene [Source:MGI Symbol	0.94679
ENSMUSG00000039376	Synpo2l	synaptopodin 2-like [Source:MGI Symbol;Acc:MGI:1	0.9468
ENSMUSG00000015522	Arnt	aryl hydrocarbon receptor nuclear translocator [Sou	0.94686
ENSMUSG00000047045	Tmem164	transmembrane protein 164 [Source:MGI Symbol;A	0.94692
ENSMUSG00000029185	Fam114a1	family with sequence similarity 114, member A1 [S	0.94702
ENSMUSG00000085733	Gm11641	predicted gene 11641 [Source:MGI Symbol;Acc:MG	0.94745
ENSMUSG00000056148	Rdh9	retinol dehydrogenase 9 [Source:MGI Symbol;Acc:M	0.94751
ENSMUSG00000073733	Rsg1	REM2 and RAB-like small GTPase 1 [Source:MGI Sym	0.94778
ENSMUSG00000070632	Btbd8	BTB (POZ) domain containing 8 [Source:MGI Symbo	0.94794
ENSMUSG00000043439	E130012A19Rik	RIKEN cDNA E130012A19 gene [Source:MGI Symbo	0.94797
ENSMUSG00000051306	Usp42	ubiquitin specific peptidase 42 [Source:MGI Symbo	0.94798
ENSMUSG00000054115	Skp2	S-phase kinase-associated protein 2 (p45) [Source:M	0.94806
ENSMUSG00000091697	Eif3s6-ps2	eukaryotic translation initiation factor 3, subunit 6,	0.94809
ENSMUSG00000084052	Gm11745	predicted gene 11745 [Source:MGI Symbol;Acc:MG	0.9481
ENSMUSG00000013707	Tnfaip8l2	tumor necrosis factor, alpha-induced protein 8-like	0.94812
ENSMUSG00000004565	Pnpla6	patatin-like phospholipase domain containing 6 [So	0.94823
ENSMUSG00000006386	Tek	endothelial-specific receptor tyrosine kinase [Sourc	0.94862
ENSMUSG00000047909	Ankrd16	ankyrin repeat domain 16 [Source:MGI Symbol;Acc	0.94863
ENSMUSG00000026414	Tnnt2	troponin T2, cardiac [Source:MGI Symbol;Acc:MGI:	0.94878
ENSMUSG00000036552	Ermard	ER membrane associated RNA degradation [Source	0.9488
ENSMUSG00000021359	Tfap2a	transcription factor AP-2, alpha [Source:MGI Symbo	0.94898
ENSMUSG00000078866	Gm14420	predicted gene 14420 [Source:MGI Symbol;Acc:MG	0.94898
ENSMUSG00000092600	Gm20442	predicted gene 20442 [Source:MGI Symbol;Acc:MG	0.94907
ENSMUSG00000030095	Tmem43	transmembrane protein 43 [Source:MGI Symbol;Ac	0.94937
ENSMUSG00000037890	Wdr19	WD repeat domain 19 [Source:MGI Symbol;Acc:MG	0.94937
ENSMUSG00000022048	Dpysl2	dihydropyrimidinase-like 2 [Source:MGI Symbol;Ac	0.94966
ENSMUSG00000039842	Mcph1	microcephaly, primary autosomal recessive 1 [Sour	0.94998
ENSMUSG00000086350	B230369F24Rik	RIKEN cDNA B230369F24 gene [Source:MGI Symbo	0.95004
ENSMUSG00000024048	Myl12a	myosin, light chain 12A, regulatory, non-sarcomeric	0.95005
ENSMUSG00000042419	Nfkbil1	nuclear factor of kappa light polypeptide gene enha	0.9502
ENSMUSG00000040812	Agbl2	ATP/GTP binding protein-like 2 [Source:MGI Symbo	0.9505
ENSMUSG00000030868	Dctn5	dynactin 5 [Source:MGI Symbol;Acc:MGI:1891689]	0.95053
ENSMUSG00000025515	Muc2	mucin 2 [Source:MGI Symbol;Acc:MGI:1339364]	0.95066
ENSMUSG00000097637	4933417D19Rik	RIKEN cDNA 4933417D19 gene [Source:MGI Symbo	0.9507
ENSMUSG00000035781	R3hdm4	R3H domain containing 4 [Source:MGI Symbol;Acc:	0.95079
ENSMUSG00000031709	Tbc1d9	TBC1 domain family, member 9 [Source:MGI Symbo	0.9508
ENSMUSG00000031637	Lrp2bp	Lrp2 binding protein [Source:MGI Symbol;Acc:MGI:	0.95089
ENSMUSG00000032942	Ucp3	uncoupling protein 3 (mitochondrial, proton carrier	0.95097
ENSMUSG00000055078	Gabra5	gamma-aminobutyric acid (GABA) A receptor, subu	0.95109
ENSMUSG00000097715	Gpr137b-ps	G protein-coupled receptor 137B, pseudogene [Sou	0.95114
ENSMUSG00000028030	Tbck	TBC1 domain containing kinase [Source:MGI Symbo	0.95123
ENSMUSG00000035799	Twist1	twist basic helix-loop-helix transcription factor 1 [S	0.95138
ENSMUSG00000038020	Rapgefl1	Rap guanine nucleotide exchange factor (GEF)-like	0.95144
ENSMUSG00000003849	Nqo1	NAD(P)H dehydrogenase, quinone 1 [Source:MGI S	0.95147
ENSMUSG00000089975	Gm7420	predicted gene 7420 [Source:MGI Symbol;Acc:MGI:	0.95147
ENSMUSG00000092412	Gm20507	predicted gene 20507 [Source:MGI Symbol;Acc:MG	0.9516
ENSMUSG00000032841	Prr5l	proline rich 5 like [Source:MGI Symbol;Acc:MGI:191	0.95189

ENSMUSG00000004561	Mettl17	methyltransferase like 17 [Source:MGI Symbol;Acc:	0.95191
ENSMUSG00000028759	Hp1bp3	heterochromatin protein 1, binding protein 3 [Sour	0.9522
ENSMUSG00000085882	2610507I01Rik	RIKEN cDNA 2610507I01 gene [Source:MGI Symbol	0.95226
ENSMUSG00000020782	Llgl2	lethal giant larvae homolog 2 (Drosophila) [Source:	0.95257
ENSMUSG00000033653	Vps8	vacuolar protein sorting 8 homolog (S. cerevisiae) [	0.95289
ENSMUSG00000061613	U2af1	U2 small nuclear ribonucleoprotein auxiliary factor	0.95296
ENSMUSG00000036904	Fzd8	frizzled homolog 8 (Drosophila) [Source:MGI Symbo	0.95297
ENSMUSG00000068117	Mei1	meiosis defective 1 [Source:MGI Symbol;Acc:MGI:3	0.95299
ENSMUSG00000030117	Gdf3	growth differentiation factor 3 [Source:MGI Symbo	0.95304
ENSMUSG00000079017	Ifi27l2a	interferon, alpha-inducible protein 27 like 2A [Sour	0.95309
ENSMUSG00000008932	Slc1a7	solute carrier family 1 (glutamate transporter), mer	0.95319
ENSMUSG00000053519	Kcnip1	Kv channel-interacting protein 1 [Source:MGI Symb	0.95332
ENSMUSG00000022214	Dcaf11	DDB1 and CUL4 associated factor 11 [Source:MGI S	0.95333
ENSMUSG00000025221	Kcnip2	Kv channel-interacting protein 2 [Source:MGI Symb	0.95335
ENSMUSG00000091712	Sec14l5	SEC14-like 5 (S. cerevisiae) [Source:MGI Symbol;Ac	0.95335
ENSMUSG00000040940	Arhgef1	Rho guanine nucleotide exchange factor (GEF) 1 [S	0.95336
ENSMUSG00000078201	Tmem203	transmembrane protein 203 [Source:MGI Symbol;A	0.95339
ENSMUSG00000036322	H2-Ea-ps	histocompatibility 2, class II antigen E alpha, pseud	0.95347
ENSMUSG00000051113	Fam71e1	family with sequence similarity 71, member E1 [Sou	0.95347
ENSMUSG00000037526	Atg14	autophagy related 14 [Source:MGI Symbol;Acc:MG	0.95349
ENSMUSG00000091514	Gm17484	predicted gene, 17484 [Source:MGI Symbol;Acc:M	0.95354
ENSMUSG00000054196	Cthrc1	collagen triple helix repeat containing 1 [Source:M	0.95359
ENSMUSG00000040640	Erc2	ELKS/RAB6-interacting/CAST family member 2 [Sou	0.95363
ENSMUSG00000022211	Lrrc16b	leucine rich repeat containing 16B [Source:MGI Sym	0.95375
ENSMUSG00000028099	Polr3c	polymerase (RNA) III (DNA directed) polypeptide C	0.95385
ENSMUSG00000021738	Atxn7	ataxin 7 [Source:MGI Symbol;Acc:MGI:2179277]	0.95414
ENSMUSG00000032946	Rasgrp2	RAS, guanyl releasing protein 2 [Source:MGI Symbo	0.95423
ENSMUSG00000027670	Ocstamp	osteoclast stimulatory transmembrane protein [Sou	0.95426
ENSMUSG00000004127	Trmt10a	tRNA methyltransferase 10A [Source:MGI Symbol;A	0.95427
ENSMUSG00000084259	Gm12040	predicted gene 12040 [Source:MGI Symbol;Acc:MG	0.9543
ENSMUSG00000026932	Nacc2	nucleus accumbens associated 2, BEN and BTB (PO	0.95436
ENSMUSG00000023932	Cdc5l	cell division cycle 5-like (S. pombe) [Source:MGI Sy	0.95437
ENSMUSG00000024013	Fgd2	FYVE, RhoGEF and PH domain containing 2 [Source	0.9544
ENSMUSG00000039476	Prrx2	paired related homeobox 2 [Source:MGI Symbol;Ac	0.95465
ENSMUSG00000024668	Sdhaf2	succinate dehydrogenase complex assembly factor	0.95467
ENSMUSG00000026566	Mpzl1	myelin protein zero-like 1 [Source:MGI Symbol;Acc	0.9548
ENSMUSG00000030330	Ing4	inhibitor of growth family, member 4 [Source:MGI	0.9548
ENSMUSG00000086455	Gm11815	predicted gene 11815 [Source:MGI Symbol;Acc:MG	0.95501
ENSMUSG00000032257	Ankk1	ankyrin repeat and kinase domain containing 1 [Sou	0.95503
ENSMUSG00000053898	Ech1	enoyl coenzyme A hydratase 1, peroxisomal [Sourc	0.95504
ENSMUSG00000032418	Me1	malic enzyme 1, NADP(+)-dependent, cytosolic [Sou	0.95506
ENSMUSG00000052248	Gm13476	predicted gene 13476 [Source:MGI Symbol;Acc:MG	0.95517
ENSMUSG00000035295	Wdr38	WD repeat domain 38 [Source:MGI Symbol;Acc:MG	0.95519
ENSMUSG00000025856	Pdgfa	platelet derived growth factor, alpha [Source:MGI	0.95531
ENSMUSG00000003134	Tbc1d8	TBC1 domain family, member 8 [Source:MGI Symb	0.95535
ENSMUSG00000058360	Gm10040	predicted gene 10040 [Source:MGI Symbol;Acc:MG	0.95539
ENSMUSG00000037847	Nmrk1	nicotinamide riboside kinase 1 [Source:MGI Symbo	0.9554

ENSMUSG00000028859	Csf3r	colony stimulating factor 3 receptor (granulocyte) [	0.95559
ENSMUSG00000033618	Map3k13	mitogen-activated protein kinase kinase kinase 13 [	0.95561
ENSMUSG00000040564	Apoc1	apolipoprotein C-I [Source:MGI Symbol;Acc:MGI:88	0.95564
ENSMUSG00000027309	4930402H24Rik	RIKEN cDNA 4930402H24 gene [Source:MGI Symbo	0.95565
ENSMUSG00000021891	Mettl6	methyltransferase like 6 [Source:MGI Symbol;Acc:N	0.95576
ENSMUSG00000038276	Asic3	acid-sensing (proton-gated) ion channel 3 [Source:N	0.95584
ENSMUSG00000078931	Pdf	peptide deformylase (mitochondrial) [Source:MGI S	0.95586
ENSMUSG00000040857	Erf	Ets2 repressor factor [Source:MGI Symbol;Acc:MGI	0.95595
ENSMUSG00000030598	Fbxo17	F-box protein 17 [Source:MGI Symbol;Acc:MGI:135	0.95602
ENSMUSG00000033595	Lgi3	leucine-rich repeat LGI family, member 3 [Source:M	0.95608
ENSMUSG00000031791	Tmem38a	transmembrane protein 38A [Source:MGI Symbol;A	0.95635
ENSMUSG00000003123	Lipe	lipase, hormone sensitive [Source:MGI Symbol;Acc:	0.95636
ENSMUSG00000085566	A730017L22Rik	RIKEN cDNA A730017L22 gene [Source:MGI Symbo	0.9566
ENSMUSG00000024983	Vti1a	vesicle transport through interaction with t-SNAREs	0.95687
ENSMUSG00000025821	Zfp282	zinc finger protein 282 [Source:MGI Symbol;Acc:MG	0.9569
ENSMUSG00000021178	Psmc1	protease (prosome, macropain) 26S subunit, ATPas	0.95711
ENSMUSG00000036513	Commd2	COMM domain containing 2 [Source:MGI Symbol;A	0.95719
ENSMUSG00000033313	Fbxl8	F-box and leucine-rich repeat protein 8 [Source:MG	0.95727
ENSMUSG00000028948	Nol9	nucleolar protein 9 [Source:MGI Symbol;Acc:MGI:1	0.95729
ENSMUSG00000034892	Rps29	ribosomal protein S29 [Source:MGI Symbol;Acc:MG	0.9573
ENSMUSG00000036955	2510003E04Rik	RIKEN cDNA 2510003E04 gene [Source:MGI Symbo	0.95735
ENSMUSG00000030872	Gga2	golgi associated, gamma adaptin ear containing, AR	0.95755
ENSMUSG00000002897	Il17ra	interleukin 17 receptor A [Source:MGI Symbol;Acc:	0.95759
ENSMUSG00000072082	Ccnf	cyclin F [Source:MGI Symbol;Acc:MGI:102551]	0.95762
ENSMUSG00000097032	4930539J05Rik	RIKEN cDNA 4930539J05 gene [Source:MGI Symbol	0.95762
ENSMUSG00000004231	Pax2	paired box gene 2 [Source:MGI Symbol;Acc:MGI:97	0.95765
ENSMUSG00000084845	Tmem240	transmembrane protein 240 [Source:MGI Symbol;A	0.95786
ENSMUSG00000054404	Slfn5	schlafen 5 [Source:MGI Symbol;Acc:MGI:1329004]	0.95791
ENSMUSG00000039099	Wdr93	WD repeat domain 93 [Source:MGI Symbol;Acc:MG	0.95793
ENSMUSG00000054850	6330419J24Rik	RIKEN cDNA 6330419J24 gene [Source:MGI Symbol	0.958
ENSMUSG00000033769	Exoc6b	exocyst complex component 6B [Source:MGI Symb	0.95811
ENSMUSG00000038781	Stap2	signal transducing adaptor family member 2 [Sourc	0.95815
ENSMUSG00000028163	Nfkb1	nuclear factor of kappa light polypeptide gene enha	0.95816
ENSMUSG00000023088	Abcc1	ATP-binding cassette, sub-family C (CFTR/MRP), me	0.9582
ENSMUSG00000059434	Gckr	glucokinase regulatory protein [Source:MGI Symbo	0.9582
ENSMUSG00000019505	Ubb	ubiquitin B [Source:MGI Symbol;Acc:MGI:98888]	0.95847
ENSMUSG00000024548	Setbp1	SET binding protein 1 [Source:MGI Symbol;Acc:MGI	0.95869
ENSMUSG00000002325	Irf9	interferon regulatory factor 9 [Source:MGI Symbol;	0.95877
ENSMUSG00000037108	Zcwpw1	zinc finger, CW type with PWWP domain 1 [Source:	0.95878
ENSMUSG00000000440	Pparg	peroxisome proliferator activated receptor gamma	0.9589
ENSMUSG00000032611	1700102P08Rik	RIKEN cDNA 1700102P08 gene [Source:MGI Symbo	0.95892
ENSMUSG00000020032	Nuak1	NUAK family, SNF1-like kinase, 1 [Source:MGI Symb	0.95922
ENSMUSG00000092558	Med20	mediator complex subunit 20 [Source:MGI Symbol;	0.95936
ENSMUSG00000025812	Pard3	par-3 (partitioning defective 3) homolog (C. elegans	0.95938
ENSMUSG00000098024	Gm27003	predicted gene, 27003 [Source:MGI Symbol;Acc:MG	0.95938
ENSMUSG00000046982	Tshz1	teashirt zinc finger family member 1 [Source:MGI S	0.95943
ENSMUSG00000097752	Gm26688	predicted gene, 26688 [Source:MGI Symbol;Acc:MG	0.95948

ENSMUSG00000097221	1810049J17Rik	RIKEN cDNA 1810049J17 gene [Source:MGI Symbol	0.95951
ENSMUSG00000030801	Kat8	K(lysine) acetyltransferase 8 [Source:MGI Symbol;A	0.95953
ENSMUSG00000046312	AI464131	expressed sequence AI464131 [Source:MGI Symbol	0.95957
ENSMUSG00000036339	Tmem260	transmembrane protein 260 [Source:MGI Symbol;A	0.95961
ENSMUSG00000030271	Ogg1	8-oxoguanine DNA-glycosylase 1 [Source:MGI Symb	0.95999
ENSMUSG00000026354	Lct	lactase [Source:MGI Symbol;Acc:MGI:104576]	0.96003
ENSMUSG00000084372	Gm13988	predicted gene 13988 [Source:MGI Symbol;Acc:MG	0.96022
ENSMUSG00000075000	Nrbf2	nuclear receptor binding factor 2 [Source:MGI Sym	0.96032
ENSMUSG00000037029	Zfp146	zinc finger protein 146 [Source:MGI Symbol;Acc:M	0.96059
ENSMUSG00000047153	Khnyn	KH and NYN domain containing [Source:MGI Symbo	0.96083
ENSMUSG00000020722	Cacng1	calcium channel, voltage-dependent, gamma subur	0.96086
ENSMUSG00000034771	Tle2	transducin-like enhancer of split 2, homolog of Dro	0.96089
ENSMUSG00000006134	Crkl	v-crk sarcoma virus CT10 oncogene homolog (avian	0.96094
ENSMUSG00000030309	Caprin2	caprin family member 2 [Source:MGI Symbol;Acc:M	0.96118
ENSMUSG00000097007	4930432B10Rik	RIKEN cDNA 4930432B10 gene [Source:MGI Symbo	0.96122
ENSMUSG00000053334	Ficd	FIC domain containing [Source:MGI Symbol;Acc:M	0.96126
ENSMUSG00000003279	Dlgap1	discs, large (Drosophila) homolog-associated protei	0.96153
ENSMUSG00000025085	Ablim1	actin-binding LIM protein 1 [Source:MGI Symbol;Ac	0.96168
ENSMUSG00000022434	Fam118a	family with sequence similarity 118, member A [Sou	0.96198
ENSMUSG00000069539	Scyl2	SCY1-like 2 (S. cerevisiae) [Source:MGI Symbol;Acc:	0.96208
ENSMUSG00000062590	Armc9	armadillo repeat containing 9 [Source:MGI Symbol;	0.96221
ENSMUSG00000085436	Gm11458	predicted gene 11458 [Source:MGI Symbol;Acc:MG	0.96223
ENSMUSG00000041840	Haus1	HAUS augmin-like complex, subunit 1 [Source:MGI	0.96232
ENSMUSG00000021260	Hhipl1	hedgehog interacting protein-like 1 [Source:MGI Sy	0.96235
ENSMUSG00000079197	Psme2	proteasome (prosome, macropain) activator subun	0.96246
ENSMUSG00000025478	Dpysl4	dihydropyrimidinase-like 4 [Source:MGI Symbol;Ac	0.96275
ENSMUSG00000096617	Gm5559	predicted gene 5559 [Source:MGI Symbol;Acc:MGI	0.96279
ENSMUSG00000012848	Rps5	ribosomal protein S5 [Source:MGI Symbol;Acc:MGI	0.96283
ENSMUSG00000045629	Sh3tc2	SH3 domain and tetratricopeptide repeats 2 [Sourc	0.96291
ENSMUSG00000034088	Hdlbp	high density lipoprotein (HDL) binding protein [Sou	0.96292
ENSMUSG00000073565	Prr16	proline rich 16 [Source:MGI Symbol;Acc:MGI:19186	0.96299
ENSMUSG00000014747	Ankrd53	ankyrin repeat domain 53 [Source:MGI Symbol;Acc	0.96305
ENSMUSG00000024542	Cep192	centrosomal protein 192 [Source:MGI Symbol;Acc:I	0.96308
ENSMUSG00000044617	Zbtb39	zinc finger and BTB domain containing 39 [Source:N	0.9631
ENSMUSG00000025872	Thoc3	THO complex 3 [Source:MGI Symbol;Acc:MGI:1920	0.96319
ENSMUSG00000003355	Fkbp11	FK506 binding protein 11 [Source:MGI Symbol;Acc:	0.96321
ENSMUSG00000001493	Meox1	mesenchyme homeobox 1 [Source:MGI Symbol;Acc	0.96365
ENSMUSG00000024598	Fbn2	fibrillin 2 [Source:MGI Symbol;Acc:MGI:95490]	0.96375
ENSMUSG00000068732	Tmem167b	transmembrane protein 167B [Source:MGI Symbol;	0.96382
ENSMUSG00000025558	Dock9	dedicator of cytokinesis 9 [Source:MGI Symbol;Acc:	0.96384
ENSMUSG00000060923	Acyp2	acylphosphatase 2, muscle type [Source:MGI Symb	0.96386
ENSMUSG00000039286	Fndc3b	fibronectin type III domain containing 3B [Source:M	0.96397
ENSMUSG00000042680	Garem	GRB2 associated, regulator of MAPK1 [Source:MGI	0.96397
ENSMUSG00000040478	Prdm13	PR domain containing 13 [Source:MGI Symbol;Acc:I	0.96409
ENSMUSG00000025427	Rnf165	ring finger protein 165 [Source:MGI Symbol;Acc:M	0.9641
ENSMUSG00000040605	Bace2	beta-site APP-cleaving enzyme 2 [Source:MGI Symb	0.96414
ENSMUSG00000084950	Gm5577	predicted gene 5577 [Source:MGI Symbol;Acc:MGI	0.96423

ENSMUSG00000046707	Csnk2a2	casein kinase 2, alpha prime polypeptide [Source:M	0.96465
ENSMUSG00000039357	Fut11	fucosyltransferase 11 [Source:MGI Symbol;Acc:MG	0.96469
ENSMUSG00000038065	2410066E13Rik	RIKEN cDNA 2410066E13 gene [Source:MGI Symbo	0.96478
ENSMUSG00000087535	Zmiz1os1	Zmiz1 opposite strand 1 [Source:MGI Symbol;Acc:M	0.96482
ENSMUSG00000028530	Jak1	Janus kinase 1 [Source:MGI Symbol;Acc:MGI:96628	0.96498
ENSMUSG00000002489	Tiam1	T cell lymphoma invasion and metastasis 1 [Source:	0.96522
ENSMUSG00000075486	Commd6	COMM domain containing 6 [Source:MGI Symbol;A	0.96527
ENSMUSG00000014776	Nol3	nucleolar protein 3 (apoptosis repressor with CARD	0.96532
ENSMUSG00000015290	Ubl4	ubiquitin-like 4 [Source:MGI Symbol;Acc:MGI:9504	0.9654
ENSMUSG00000022040	Ephx2	epoxide hydrolase 2, cytoplasmic [Source:MGI Sym	0.9654
ENSMUSG00000074873	AI606181	expressed sequence AI606181 [Source:MGI Symbol	0.96558
ENSMUSG00000005069	Pex5	peroxisomal biogenesis factor 5 [Source:MGI Symb	0.96569
ENSMUSG00000032560	Dnajc13	DnaJ (Hsp40) homolog, subfamily C, member 13 [Sc	0.96583
ENSMUSG00000045657	Pcdhb10	protocadherin beta 10 [Source:MGI Symbol;Acc:MC	0.96593
ENSMUSG00000028438	Kif24	kinesin family member 24 [Source:MGI Symbol;Acc	0.96602
ENSMUSG00000024076	Vit	vitrin [Source:MGI Symbol;Acc:MGI:1921449]	0.96612
ENSMUSG00000093507	Gm20627	predicted gene 20627 [Source:MGI Symbol;Acc:MG	0.96617
ENSMUSG00000036061	Smug1	single-strand selective monofunctional uracil DNA g	0.96629
ENSMUSG00000027777	Schip1	schwannomin interacting protein 1 [Source:MGI Sy	0.9663
ENSMUSG00000030738	Eif3c	eukaryotic translation initiation factor 3, subunit C	0.96641
ENSMUSG00000036528	Ppfibp2	PTPRF interacting protein, binding protein 2 (liprin	0.96665
ENSMUSG00000003585	Sec14l2	SEC14-like 2 (S. cerevisiae) [Source:MGI Symbol;Acc	0.9667
ENSMUSG00000021113	Snapc1	small nuclear RNA activating complex, polypeptide	0.96672
ENSMUSG00000018861	Fdxr	ferredoxin reductase [Source:MGI Symbol;Acc:MGI	0.96681
ENSMUSG00000033590	Myo5c	myosin VC [Source:MGI Symbol;Acc:MGI:2442485]	0.96681
ENSMUSG00000028247	Coq3	coenzyme Q3 homolog, methyltransferase (yeast) [	0.96682
ENSMUSG00000097251	5033417F24Rik	RIKEN cDNA 5033417F24 gene [Source:MGI Symbo	0.96684
ENSMUSG00000097136	Gm26772	predicted gene, 26772 [Source:MGI Symbol;Acc:MC	0.96685
ENSMUSG00000029862	Clcn1	chloride channel 1 [Source:MGI Symbol;Acc:MGI:88	0.96692
ENSMUSG00000032366	Tpm1	tropomyosin 1, alpha [Source:MGI Symbol;Acc:MGI	0.96707
ENSMUSG00000039294	BC017643	cDNA sequence BC017643 [Source:MGI Symbol;Acc	0.96714
ENSMUSG00000035486	Plk5	polo-like kinase 5 [Source:MGI Symbol;Acc:MGI:302	0.9672
ENSMUSG00000027848	Olfml3	olfactomedin-like 3 [Source:MGI Symbol;Acc:MGI:1	0.96727
ENSMUSG00000068151	A230006K03Rik	RIKEN cDNA A230006K03 gene [Source:MGI Symbo	0.96751
ENSMUSG00000024513	Mbd2	methyl-CpG binding domain protein 2 [Source:MGI	0.96753
ENSMUSG00000032278	Paqr5	progestin and adipoQ receptor family member V [S	0.96768
ENSMUSG00000021458	2010111I01Rik	RIKEN cDNA 2010111I01 gene [Source:MGI Symbol	0.96783
ENSMUSG00000028990	Lzic	leucine zipper and CTNNBIP1 domain containing [S	0.96783
ENSMUSG00000040451	Sgms1	sphingomyelin synthase 1 [Source:MGI Symbol;Acc	0.9679
ENSMUSG00000037788	Vopp1	vesicular, overexpressed in cancer, prosurvival prot	0.96794
ENSMUSG00000020453	Patz1	POZ (BTB) and AT hook containing zinc finger 1 [Sou	0.96797
ENSMUSG00000096988	A930029G22Rik	RIKEN cDNA A930029G22 gene [Source:MGI Symbo	0.968
ENSMUSG00000048264	Dip2c	DIP2 disco-interacting protein 2 homolog C (Drosop	0.96827
ENSMUSG00000031971	Ccsap	centriole, cilia and spindle associated protein [Sour	0.96829
ENSMUSG00000073434	Wdr90	WD repeat domain 90 [Source:MGI Symbol;Acc:MC	0.96834
ENSMUSG00000039234	Sec24d	Sec24 related gene family, member D (S. cerevisiae	0.96872
ENSMUSG00000026042	Col5a2	collagen, type V, alpha 2 [Source:MGI Symbol;Acc:M	0.96884

ENSMUSG00000028111	Ctsk	cathepsin K [Source:MGI Symbol;Acc:MGI:107823]	0.9689
ENSMUSG00000028456	Unc13b	unc-13 homolog B (C. elegans) [Source:MGI Symbo	0.96892
ENSMUSG00000074746	Pdzd8	PDZ domain containing 8 [Source:MGI Symbol;Acc:	0.96901
ENSMUSG00000015575	Atp6v0e	ATPase, H+ transporting, lysosomal V0 subunit E [S	0.9692
ENSMUSG00000039242	B3galnt2	UDP-GalNAc:betaGlcNAc beta 1,3-galactosaminyltr	0.96924
ENSMUSG00000041515	Irf8	interferon regulatory factor 8 [Source:MGI Symbol;	0.96954
ENSMUSG00000079104	Prps1l3	phosphoribosyl pyrophosphate synthetase 1-like 3	0.96959
ENSMUSG00000018736	Ndel1	nuclear distribution gene E-like homolog 1 (A. nidul	0.96966
ENSMUSG00000039496	Cdnf	cerebral dopamine neurotrophic factor [Source:MG	0.96966
ENSMUSG00000032372	Plscr2	phospholipid scramblase 2 [Source:MGI Symbol;Acc	0.96982
ENSMUSG00000005774	Rfx5	regulatory factor X, 5 (influences HLA class II expres	0.97
ENSMUSG00000046318	Ccbe1	collagen and calcium binding EGF domains 1 [Sourc	0.97004
ENSMUSG00000085169	Gm10785	predicted gene 10785 [Source:MGI Symbol;Acc:MG	0.97016
ENSMUSG00000023176	Cpn2	carboxypeptidase N, polypeptide 2 [Source:MGI Sy	0.97026
ENSMUSG00000000724	Cryba1	crystallin, beta A1 [Source:MGI Symbol;Acc:MGI:88	0.97033
ENSMUSG00000002308	Cd320	CD320 antigen [Source:MGI Symbol;Acc:MGI:18600	0.97033
ENSMUSG00000060519	Tor3a	torsin family 3, member A [Source:MGI Symbol;Acc	0.97053
ENSMUSG00000026632	Tatdn3	TatD DNase domain containing 3 [Source:MGI Syml	0.97067
ENSMUSG00000038175	Mylip	myosin regulatory light chain interacting protein [S	0.97067
ENSMUSG00000093734	Gm25082	predicted gene, 25082 [Source:MGI Symbol;Acc:M	0.97085
ENSMUSG00000046718	Bst2	bone marrow stromal cell antigen 2 [Source:MGI Sy	0.97088
ENSMUSG00000043088	Il17re	interleukin 17 receptor E [Source:MGI Symbol;Acc:	0.97117
ENSMUSG00000038214	Bend3	BEN domain containing 3 [Source:MGI Symbol;Acc:	0.97125
ENSMUSG00000053580	Tanc2	tetratricopeptide repeat, ankyrin repeat and coiled	0.97134
ENSMUSG00000038305	Spats2l	spermatogenesis associated, serine-rich 2-like [Sou	0.97141
ENSMUSG00000024845	Tmem134	transmembrane protein 134 [Source:MGI Symbol;A	0.97145
ENSMUSG00000027937	Jtb	jumping translocation breakpoint [Source:MGI Sym	0.97166
ENSMUSG00000051890	Klhdc1	kelch domain containing 1 [Source:MGI Symbol;Acc	0.97184
ENSMUSG00000035948	Acss3	acyl-CoA synthetase short-chain family member 3 [	0.9719
ENSMUSG00000039194	Rlbp1	retinaldehyde binding protein 1 [Source:MGI Symb	0.97198
ENSMUSG00000030045	Mrpl19	mitochondrial ribosomal protein L19 [Source:MGI S	0.97206
ENSMUSG00000038860	Garnl3	GTPase activating RANGAP domain-like 3 [Source:M	0.97211
ENSMUSG00000056687	Gm11696	predicted gene 11696 [Source:MGI Symbol;Acc:MG	0.97231
ENSMUSG00000026072	Il1r1	interleukin 1 receptor, type I [Source:MGI Symbol;A	0.97237
ENSMUSG00000026817	Ak1	adenylate kinase 1 [Source:MGI Symbol;Acc:MGI:87	0.97241
ENSMUSG00000046417	Fam211a	family with sequence similarity 211, member A [Sou	0.97241
ENSMUSG00000033542	Arhgef5	Rho guanine nucleotide exchange factor (GEF) 5 [So	0.97276
ENSMUSG00000039396	Neil3	nei like 3 (E. coli) [Source:MGI Symbol;Acc:MGI:238	0.97287
ENSMUSG00000029449	Rhof	ras homolog gene family, member f [Source:MGI Sy	0.97292
ENSMUSG00000044646	Zbtb7c	zinc finger and BTB domain containing 7C [Source:M	0.97294
ENSMUSG00000028078	Dclk2	doublecortin-like kinase 2 [Source:MGI Symbol;Acc	0.97306
ENSMUSG00000038506	Dcun1d2	DCN1, defective in cullin neddylation 1, domain con	0.97312
ENSMUSG00000039182	AW209491	expressed sequence AW209491 [Source:MGI Symb	0.97314
ENSMUSG00000022408	Fam83f	family with sequence similarity 83, member F [Sour	0.97327
ENSMUSG00000029056	Pank4	pantothenate kinase 4 [Source:MGI Symbol;Acc:M	0.97331
ENSMUSG00000026117	Zap70	zeta-chain (TCR) associated protein kinase [Source:	0.97335
ENSMUSG00000032523	Hhatl	hedgehog acyltransferase-like [Source:MGI Symbol	0.97337

ENSMUSG00000030763	Lcmt1	leucine carboxyl methyltransferase 1 [Source:MGI S	0.9734
ENSMUSG00000022555	Dgat1	diacylglycerol O-acyltransferase 1 [Source:MGI Sym	0.97344
ENSMUSG00000032279	Idh3a	isocitrate dehydrogenase 3 (NAD+) alpha [Source:M	0.97348
ENSMUSG00000042647	Acad12	acyl-Coenzyme A dehydrogenase family, member 1	0.97357
ENSMUSG00000027405	Nop56	NOP56 ribonucleoprotein [Source:MGI Symbol;Acc	0.97368
ENSMUSG00000056724	Nbeal2	neurobeachin-like 2 [Source:MGI Symbol;Acc:MGI:	0.9737
ENSMUSG00000031389	Arhgap4	Rho GTPase activating protein 4 [Source:MGI Symb	0.97383
ENSMUSG00000097526	Gm26847	predicted gene, 26847 [Source:MGI Symbol;Acc:M	0.97391
ENSMUSG00000031097	Tnni2	troponin I, skeletal, fast 2 [Source:MGI Symbol;Acc:	0.97411
ENSMUSG00000072653	Zfp783	zinc finger protein 783 [Source:MGI Symbol;Acc:M	0.97434
ENSMUSG00000075602	Ly6a	lymphocyte antigen 6 complex, locus A [Source:MG	0.97436
ENSMUSG00000035539	E230008N13Rik	RIKEN cDNA E230008N13 gene [Source:MGI Symbo	0.97438
ENSMUSG00000044702	Palb2	partner and localizer of BRCA2 [Source:MGI Symbo	0.9744
ENSMUSG00000021003	Galc	galactosylceramidase [Source:MGI Symbol;Acc:MG	0.9746
ENSMUSG00000045083	Lingo2	leucine rich repeat and Ig domain containing 2 [Sou	0.9747
ENSMUSG00000049580	Tsku	tsukushi [Source:MGI Symbol;Acc:MGI:2443855]	0.97471
ENSMUSG00000047656	Trpt1	tRNA phosphotransferase 1 [Source:MGI Symbol;A	0.97476
ENSMUSG00000032342	Mto1	mitochondrial translation optimization 1 homolog (	0.97482
ENSMUSG00000040666	Sh3bgr	SH3-binding domain glutamic acid-rich protein [Sou	0.97482
ENSMUSG00000019189	Rnf145	ring finger protein 145 [Source:MGI Symbol;Acc:M	0.97502
ENSMUSG00000056656	Apol8	apolipoprotein L 8 [Source:MGI Symbol;Acc:MGI:24	0.97506
ENSMUSG00000097431	Gm26782	predicted gene, 26782 [Source:MGI Symbol;Acc:M	0.97512
ENSMUSG00000035933	Cog5	component of oligomeric golgi complex 5 [Source:M	0.97524
ENSMUSG00000001348	Acp5	acid phosphatase 5, tartrate resistant [Source:MGI	0.97534
ENSMUSG00000044749	Abca6	ATP-binding cassette, sub-family A (ABC1), membe	0.97544
ENSMUSG00000071711	Mpst	mercaptopyruvate sulfurtransferase [Source:MGI S	0.97548
ENSMUSG00000025173	Wdr45b	WD repeat domain 45B [Source:MGI Symbol;Acc:M	0.9756
ENSMUSG00000020448	Rnf185	ring finger protein 185 [Source:MGI Symbol;Acc:M	0.97561
ENSMUSG00000048920	Fkrp	fukutin related protein [Source:MGI Symbol;Acc:M	0.97571
ENSMUSG00000020088	Sar1a	SAR1 gene homolog A (S. cerevisiae) [Source:MGI S	0.97576
ENSMUSG00000044367	Slc16a13	solute carrier family 16 (monocarboxylic acid trans	0.97601
ENSMUSG00000001158	Snrnp27	small nuclear ribonucleoprotein 27 (U4/U6.U5) [So	0.97607
ENSMUSG00000066441	Rdh11	retinol dehydrogenase 11 [Source:MGI Symbol;Acc	0.97617
ENSMUSG00000020778	Ten1	TEN1 telomerase capping complex subunit [Source	0.97628
ENSMUSG00000055991	Zkscan5	zinc finger with KRAB and SCAN domains 5 [Source:	0.97709
ENSMUSG00000063785	Utp14a	UTP14, U3 small nucleolar ribonucleoprotein, hom	0.97717
ENSMUSG00000057788	Ddx49	DEAD (Asp-Glu-Ala-Asp) box polypeptide 49 [Sourc	0.9772
ENSMUSG00000029312	Klhl8	kelch-like 8 [Source:MGI Symbol;Acc:MGI:2179430]	0.97727
ENSMUSG00000031633	Slc25a4	solute carrier family 25 (mitochondrial carrier, ade	0.97736
ENSMUSG00000034152	Exoc3	exocyst complex component 3 [Source:MGI Symbo	0.97738
ENSMUSG00000019124	Scrn1	secernin 1 [Source:MGI Symbol;Acc:MGI:1917188]	0.97742
ENSMUSG00000023913	Pla2g7	phospholipase A2, group VII (platelet-activating fac	0.97755
ENSMUSG00000024875	Yif1a	Yip1 interacting factor homolog A (S. cerevisiae) [So	0.97782
ENSMUSG00000049439	Cyp20a1	cytochrome P450, family 20, subfamily a, polypepti	0.97782
ENSMUSG00000041774	Ydjc	YdjC homolog (bacterial) [Source:MGI Symbol;Acc:M	0.97786
ENSMUSG00000024186	Rgs11	regulator of G-protein signaling 11 [Source:MGI Sy	0.97788
ENSMUSG00000046441	Cmtr2	cap methyltransferase 2 [Source:MGI Symbol;Acc:M	0.9779

ENSMUSG00000002012	Pnck	pregnancy upregulated non-ubiquitously expressed	0.97793
ENSMUSG00000073609	D2hgdh	D-2-hydroxyglutarate dehydrogenase [Source:MGI	0.97814
ENSMUSG00000036814	Slc6a20a	solute carrier family 6 (neurotransmitter transporte	0.97815
ENSMUSG00000039137	Whrn	whirlin [Source:MGI Symbol;Acc:MGI:2682003]	0.97821
ENSMUSG00000071369	Map3k5	mitogen-activated protein kinase kinase kinase 5 [S	0.97821
ENSMUSG00000001555	Fkbp10	FK506 binding protein 10 [Source:MGI Symbol;Acc:	0.97822
ENSMUSG00000045917	6330416G13Rik	RIKEN cDNA 6330416G13 gene [Source:MGI Symbo	0.97825
ENSMUSG00000035033	Tbr1	T-box brain gene 1 [Source:MGI Symbol;Acc:MGI:1	0.9784
ENSMUSG00000028902	Sf3a3	splicing factor 3a, subunit 3 [Source:MGI Symbol;A	0.97842
ENSMUSG00000068749	Psma5	proteasome (prosome, macropain) subunit, alpha t	0.97853
ENSMUSG00000027985	Lef1	lymphoid enhancer binding factor 1 [Source:MGI Sy	0.97861
ENSMUSG00000070469	Adamtsl3	ADAMTS-like 3 [Source:MGI Symbol;Acc:MGI:3028	0.97862
ENSMUSG00000097893	1700034P13Rik	RIKEN cDNA 1700034P13 gene [Source:MGI Symbo	0.97869
ENSMUSG00000069874	Irgm2	immunity-related GTPase family M member 2 [Sou	0.9788
ENSMUSG00000041779	Tram2	translocating chain-associating membrane protein	0.97885
ENSMUSG00000039887	Alg14	asparagine-linked glycosylation 14 [Source:MGI Syr	0.97899
ENSMUSG00000020836	Coro6	coronin 6 [Source:MGI Symbol;Acc:MGI:2183448]	0.97906
ENSMUSG00000021905	Dph3	diphthamine biosynthesis 3 [Source:MGI Symbol;A	0.97912
ENSMUSG00000017969	Ptgis	prostaglandin I2 (prostacyclin) synthase [Source:M	0.97942
ENSMUSG00000060373	Hnrnpc	heterogeneous nuclear ribonucleoprotein C [Sourc	0.97949
ENSMUSG00000025010	Ccnj	cyclin J [Source:MGI Symbol;Acc:MGI:2443297]	0.97955
ENSMUSG00000019763	Rmnd1	required for meiotic nuclear division 1 homolog (S.	0.97959
ENSMUSG00000049999	Ppp1r3d	protein phosphatase 1, regulatory subunit 3D [Sour	0.97961
ENSMUSG00000028619	Tceanc2	transcription elongation factor A (SII) N-terminal ar	0.97964
ENSMUSG00000079610	Ankrd39	ankyrin repeat domain 39 [Source:MGI Symbol;Acc	0.9797
ENSMUSG00000062526	Mppe1	metallophosphoesterase 1 [Source:MGI Symbol;Ac	0.97974
ENSMUSG00000005672	Kit	kit oncogene [Source:MGI Symbol;Acc:MGI:96677]	0.97977
ENSMUSG00000020743	Mif4gd	MIF4G domain containing [Source:MGI Symbol;Acc	0.97979
ENSMUSG00000064215	Ifi27	interferon, alpha-inducible protein 27 [Source:MGI	0.97988
ENSMUSG00000004951	Hspb1	heat shock protein 1 [Source:MGI Symbol;Acc:MGI:	0.98006
ENSMUSG00000030335	Mrpl51	mitochondrial ribosomal protein L51 [Source:MGI S	0.98016
ENSMUSG00000027086	Fastkd1	FAST kinase domains 1 [Source:MGI Symbol;Acc:M	0.98062
ENSMUSG00000031446	Cul4a	cullin 4A [Source:MGI Symbol;Acc:MGI:1914487]	0.9807
ENSMUSG00000057375	Yipf1	Yip1 domain family, member 1 [Source:MGI Symbo	0.98081
ENSMUSG00000020848	Doc2b	double C2, beta [Source:MGI Symbol;Acc:MGI:110	0.98085
ENSMUSG00000049764	Zfp280b	zinc finger protein 280B [Source:MGI Symbol;Acc:M	0.98098
ENSMUSG00000031980	Agt	angiotensinogen (serpin peptidase inhibitor, clade	0.98102
ENSMUSG00000022814	Umps	uridine monophosphate synthetase [Source:MGI Sy	0.98107
ENSMUSG00000095742	CAAA01147332.1	CD99 antigen (Cd99), mRNA [Source:RefSeq mRNA	0.98159
ENSMUSG00000038683	Pak1ip1	PAK1 interacting protein 1 [Source:MGI Symbol;Ac	0.98168
ENSMUSG00000038145	Snrk	SNF related kinase [Source:MGI Symbol;Acc:MGI:1	0.98189
ENSMUSG00000025225	Nfkb2	nuclear factor of kappa light polypeptide gene enha	0.982
ENSMUSG00000032567	Aste1	asteroid homolog 1 (Drosophila) [Source:MGI Symb	0.98206
ENSMUSG00000039914	Coq10a	coenzyme Q10 homolog A (yeast) [Source:MGI Sym	0.98219
ENSMUSG00000028085	Pet112	PET112 homolog (S. cerevisiae) [Source:MGI Symb	0.98228
ENSMUSG00000034243	Golgb1	golgi autoantigen, golgin subfamily b, macrogolgin	0.98281
ENSMUSG00000025413	Ttc4	tetratricopeptide repeat domain 4 [Source:MGI Syr	0.98295

ENSMUSG00000045733	Sprn	shadow of prion protein [Source:MGI Symbol;Acc:M	0.98312
ENSMUSG00000058498	Rnf207	ring finger protein 207 [Source:MGI Symbol;Acc:MG	0.98346
ENSMUSG00000037572	Wdhd1	WD repeat and HMG-box DNA binding protein 1 [So	0.98367
ENSMUSG00000052906	Ubxn8	UBX domain protein 8 [Source:MGI Symbol;Acc:MG	0.98367
ENSMUSG00000037353	Letmd1	LETM1 domain containing 1 [Source:MGI Symbol;A	0.9837
ENSMUSG00000027722	Spata5	spermatogenesis associated 5 [Source:MGI Symbol	0.98378
ENSMUSG00000073542	Cep76	centrosomal protein 76 [Source:MGI Symbol;Acc:M	0.98391
ENSMUSG00000035776	Cd99l2	CD99 antigen-like 2 [Source:MGI Symbol;Acc:MGI:2	0.98406
ENSMUSG00000039828	Wdr70	WD repeat domain 70 [Source:MGI Symbol;Acc:MG	0.98415
ENSMUSG00000009647	Mcu	mitochondrial calcium uniporter [Source:MGI Symb	0.98433
ENSMUSG00000050627	Gpd1l	glycerol-3-phosphate dehydrogenase 1-like [Source	0.98454
ENSMUSG00000047842	Diras2	DIRAS family, GTP-binding RAS-like 2 [Source:MGI S	0.98456
ENSMUSG00000052336	Cx3cr1	chemokine (C-X3-C) receptor 1 [Source:MGI Symbo	0.98463
ENSMUSG00000022790	Igsf11	immunoglobulin superfamily, member 11 [Source:M	0.9847
ENSMUSG00000001948	Spa17	sperm autoantigenic protein 17 [Source:MGI Symbo	0.98487
ENSMUSG00000028886	Eya3	eyes absent 3 homolog (Drosophila) [Source:MGI Sy	0.98502
ENSMUSG00000035704	Alg8	asparagine-linked glycosylation 8 (alpha-1,3-glucosy	0.98514
ENSMUSG00000019907	Ppp1r12a	protein phosphatase 1, regulatory (inhibitor) subun	0.98515
ENSMUSG00000038844	Kif16b	kinesin family member 16B [Source:MGI Symbol;Ac	0.98515
ENSMUSG00000029659	Medag	mesenteric estrogen dependent adipogenesis [Sour	0.98517
ENSMUSG00000003235	Eif2b5	eukaryotic translation initiation factor 2B, subunit 5	0.98523
ENSMUSG00000025188	Hps1	Hermansky-Pudlak syndrome 1 homolog (human) [	0.98525
ENSMUSG00000027331	Knstrn	kinetochore-localized astrin/SPAG5 binding [Source	0.98539
ENSMUSG00000008226	Scrn3	secernin 3 [Source:MGI Symbol;Acc:MGI:1921866]	0.9855
ENSMUSG00000075033	Nxpe3	neurexophilin and PC-esterase domain family, mem	0.98551
ENSMUSG00000035642	Aamdc	adipogenesis associated Mth938 domain containing	0.98569
ENSMUSG00000028607	Cpt2	carnitine palmitoyltransferase 2 [Source:MGI Symb	0.9857
ENSMUSG00000034587	8430429K09Rik	RIKEN cDNA 8430429K09 gene [Source:MGI Symbo	0.98585
ENSMUSG00000022617	Chkb	choline kinase beta [Source:MGI Symbol;Acc:MGI:1	0.98592
ENSMUSG00000073755	5730409E04Rik	RIKEN cDNA 5730409E04Rik gene [Source:MGI Sym	0.98599
ENSMUSG00000040875	Osbpl10	oxysterol binding protein-like 10 [Source:MGI Symb	0.98607
ENSMUSG00000035671	Zswim4	zinc finger SWIM-type containing 4 [Source:MGI Sy	0.98615
ENSMUSG00000026427	Eif2d	eukaryotic translation initiation factor 2D [Source:M	0.98621
ENSMUSG00000020528	Prpsap2	phosphoribosyl pyrophosphate synthetase-associat	0.98656
ENSMUSG00000020728	Cep112	centrosomal protein 112 [Source:MGI Symbol;Acc:M	0.98666
ENSMUSG00000023019	Gpd1	glycerol-3-phosphate dehydrogenase 1 (soluble) [So	0.98687
ENSMUSG00000020777	Acox1	acyl-Coenzyme A oxidase 1, palmitoyl [Source:MGI	0.98708
ENSMUSG00000027487	Cdk5rap1	CDK5 regulatory subunit associated protein 1 [Sour	0.98712
ENSMUSG00000031090	Nadsyn1	NAD synthetase 1 [Source:MGI Symbol;Acc:MGI:19	0.98715
ENSMUSG00000006273	Atp6v1b2	ATPase, H+ transporting, lysosomal V1 subunit B2 [	0.98717
ENSMUSG00000031988	Vps26b	vacuolar protein sorting 26 homolog B (yeast) [Sour	0.98717
ENSMUSG00000022987	Zfp641	zinc finger protein 641 [Source:MGI Symbol;Acc:MG	0.98723
ENSMUSG00000021108	Prkch	protein kinase C, eta [Source:MGI Symbol;Acc:MGI:	0.9873
ENSMUSG00000067567	Hdac8	histone deacetylase 8 [Source:MGI Symbol;Acc:MG	0.98732
ENSMUSG00000028909	Ptpru	protein tyrosine phosphatase, receptor type, U [Sou	0.98755
ENSMUSG00000011884	Gltp	glycolipid transfer protein [Source:MGI Symbol;Acc	0.98758
ENSMUSG00000064254	Ethe1	ethylmalonic encephalopathy 1 [Source:MGI Symbo	0.98758

ENSMUSG00000067242	Lgi1	leucine-rich repeat LGI family, member 1 [Source:M	0.98783
ENSMUSG00000026468	Lhx4	LIM homeobox protein 4 [Source:MGI Symbol;Acc:I	0.98802
ENSMUSG00000048612	Myof	myoferlin [Source:MGI Symbol;Acc:MGI:1919192]	0.98806
ENSMUSG00000034674	Tdg	thymine DNA glycosylase [Source:MGI Symbol;Acc:	0.9881
ENSMUSG00000043962	Thrap3	thyroid hormone receptor associated protein 3 [So	0.98813
ENSMUSG00000049804	Armcx4	armadillo repeat containing, X-linked 4 [Source:MG	0.98821
ENSMUSG00000032297	Celf6	CUGBP, Elav-like family member 6 [Source:MGI Syn	0.98831
ENSMUSG00000068299	1700019G17Rik	RIKEN cDNA 1700019G17 gene [Source:MGI Symbo	0.98846
ENSMUSG00000053317	Sec61b	Sec61 beta subunit [Source:MGI Symbol;Acc:MGI:1	0.98847
ENSMUSG00000043999	Gpr75	G protein-coupled receptor 75 [Source:MGI Symbo	0.9885
ENSMUSG00000019891	Dcbld1	discoidin, CUB and LCCL domain containing 1 [Sour	0.98897
ENSMUSG00000052763	Zfp212	Zinc finger protein 212 [Source:MGI Symbol;Acc:M	0.98905
ENSMUSG00000022212	Cpne6	copine VI [Source:MGI Symbol;Acc:MGI:1334445]	0.98909
ENSMUSG00000034848	Ttc21b	tetratricopeptide repeat domain 21B [Source:MGI S	0.9892
ENSMUSG00000040234	Tm7sf3	transmembrane 7 superfamily member 3 [Source:N	0.98921
ENSMUSG00000037958	Ccdc55	coiled-coil domain containing 55 [Source:MGI Symb	0.98937
ENSMUSG00000029708	Gcc1	golgi coiled coil 1 [Source:MGI Symbol;Acc:MGI:192	0.9895
ENSMUSG00000051062	Fbll1	fibrillarin-like 1 [Source:MGI Symbol;Acc:MGI:3034	0.9897
ENSMUSG00000012535	Tnpo3	transportin 3 [Source:MGI Symbol;Acc:MGI:119641	0.98991
ENSMUSG00000068854	Hist2h2be	histone cluster 2, H2be [Source:MGI Symbol;Acc:M	0.98997
ENSMUSG00000046613	Vwa5b2	von Willebrand factor A domain containing 5B2 [So	0.99011
ENSMUSG00000029231	Pdgfra	platelet derived growth factor receptor, alpha poly	0.99014
ENSMUSG00000020056	Ccdc53	coiled-coil domain containing 53 [Source:MGI Symb	0.99018
ENSMUSG00000028322	Exosc3	exosome component 3 [Source:MGI Symbol;Acc:M	0.99025
ENSMUSG00000005686	Ampd3	adenosine monophosphate deaminase 3 [Source:M	0.99041
ENSMUSG00000025470	Zfp511	zinc finger protein 511 [Source:MGI Symbol;Acc:M	0.99048
ENSMUSG00000028800	Hdac1	histone deacetylase 1 [Source:MGI Symbol;Acc:MG	0.99048
ENSMUSG00000024493	Lars	leucyl-tRNA synthetase [Source:MGI Symbol;Acc:M	0.99072
ENSMUSG00000017843	Ppp2r5c	protein phosphatase 2, regulatory subunit B (B56),	0.99073
ENSMUSG00000004113	Cacna1b	calcium channel, voltage-dependent, N type, alpha	0.99111
ENSMUSG00000027692	Tnik	TRAF2 and NCK interacting kinase [Source:MGI Sym	0.99126
ENSMUSG00000047810	Ccdc88b	coiled-coil domain containing 88B [Source:MGI Sym	0.99146
ENSMUSG00000026584	Scyl3	SCY1-like 3 (S. cerevisiae) [Source:MGI Symbol;Acc:	0.99165
ENSMUSG00000024018	Ccdc167	coiled-coil domain containing 167 [Source:MGI Sym	0.99166
ENSMUSG00000001089	Luzp1	leucine zipper protein 1 [Source:MGI Symbol;Acc:M	0.99209
ENSMUSG00000024526	Cidea	cell death-inducing DNA fragmentation factor, alph	0.99245
ENSMUSG00000010554	Mettl16	methyltransferase like 16 [Source:MGI Symbol;Acc:	0.99254
ENSMUSG00000034274	Thoc5	THO complex 5 [Source:MGI Symbol;Acc:MGI:1351	0.99258
ENSMUSG00000039001	Rps21	ribosomal protein S21 [Source:MGI Symbol;Acc:MG	0.99268
ENSMUSG00000022234	Cct5	chaperonin containing Tcp1, subunit 5 (epsilon) [So	0.99281
ENSMUSG00000097944	A130014A01Rik	RIKEN cDNA A130014A01 gene [Source:MGI Symbo	0.99339
ENSMUSG00000037242	Clic4	chloride intracellular channel 4 (mitochondrial) [So	0.99349
ENSMUSG00000070730	Rmdn3	regulator of microtubule dynamics 3 [Source:MGI S	0.99369
ENSMUSG00000060798	Intu	inturned planar cell polarity effector homolog (Dros	0.99388
ENSMUSG00000031924	Cyb5b	cytochrome b5 type B [Source:MGI Symbol;Acc:MG	0.994
ENSMUSG00000033096	Apmap	adipocyte plasma membrane associated protein [So	0.99408
ENSMUSG00000033361	Prrg3	proline rich Gla (G-carboxyglutamic acid) 3 (transm	0.99429

ENSMUSG00000020154	Ptprb	protein tyrosine phosphatase, receptor type, B [Sou	0.99441
ENSMUSG00000051985	Igfn1	immunoglobulin-like and fibronectin type III domai	0.99469
ENSMUSG00000037211	Spry1	sprouty homolog 1 (Drosophila) [Source:MGI Symb	0.99533
ENSMUSG00000020044	Timp3	tissue inhibitor of metalloproteinase 3 [Source:MG	0.99545
ENSMUSG00000025646	Atrip	ATR interacting protein [Source:MGI Symbol;Acc:M	0.99561
ENSMUSG00000021245	Mlh3	mutL homolog 3 (E coli) [Source:MGI Symbol;Acc:M	0.99579
ENSMUSG00000097815	Gm26809	predicted gene, 26809 [Source:MGI Symbol;Acc:M	0.99587
ENSMUSG00000004455	Ppp1cc	protein phosphatase 1, catalytic subunit, gamma is	0.99601
ENSMUSG00000096054	Syne1	spectrin repeat containing, nuclear envelope 1 [Sou	0.9962
ENSMUSG00000044676	Zfp612	zinc finger protein 612 [Source:MGI Symbol;Acc:M	0.9965
ENSMUSG00000035566	Pcdh17	protocadherin 17 [Source:MGI Symbol;Acc:MGI:26	0.99656
ENSMUSG00000090626	Tex9	testis expressed gene 9 [Source:MGI Symbol;Acc:M	0.99689
ENSMUSG00000066357	Wdr6	WD repeat domain 6 [Source:MGI Symbol;Acc:MGI	0.99704
ENSMUSG00000024135	Srbd1	S1 RNA binding domain 1 [Source:MGI Symbol;Acc:	0.99729
ENSMUSG00000015747	Vps45	vacuolar protein sorting 45 (yeast) [Source:MGI Sy	0.9975
ENSMUSG00000005936	Kctd20	potassium channel tetramerisation domain contain	0.99765
ENSMUSG00000045411	2410002F23Rik	RIKEN cDNA 2410002F23 gene [Source:MGI Symbo	0.99789
ENSMUSG00000048732	Klhl11	kelch-like 11 [Source:MGI Symbol;Acc:MGI:238864	0.99791
ENSMUSG00000021179	Nrde2	nrde-2 necessary for RNA interference, domain con	0.998
ENSMUSG00000026466	Tor1aip1	torsin A interacting protein 1 [Source:MGI Symbol;	0.99809
ENSMUSG00000067071	Hes6	hairy and enhancer of split 6 [Source:MGI Symbol;	0.99809
ENSMUSG00000011171	Vipr2	vasoactive intestinal peptide receptor 2 [Source:M	0.99812
ENSMUSG00000030672	Mylpf	myosin light chain, phosphorylatable, fast skeletal	0.99853
ENSMUSG00000009630	Ppp2cb	protein phosphatase 2 (formerly 2A), catalytic subu	0.99857
ENSMUSG00000037710	Cisd1	CDGSH iron sulfur domain 1 [Source:MGI Symbol;A	0.99877
ENSMUSG00000045967	Gpr158	G protein-coupled receptor 158 [Source:MGI Symb	0.99924
ENSMUSG00000004789	Dlst	dihydrolipoamide S-succinyltransferase (E2 compo	0.99932
ENSMUSG00000042010	Acacb	acetyl-Coenzyme A carboxylase beta [Source:MGI S	0.99983
ENSMUSG00000062232	Rapgef2	Rap guanine nucleotide exchange factor (GEF) 2 [S	0.99986
ENSMUSG00000057133	Chd6	chromodomain helicase DNA binding protein 6 [Sou	0.99987
ENSMUSG00000019467	Arhgef25	Rho guanine nucleotide exchange factor (GEF) 25 [	0.99998
ENSMUSG00000000049	Apoh	apolipoprotein H [Source:MGI Symbol;Acc:MGI:880	1
ENSMUSG00000000159	Igsf5	immunoglobulin superfamily, member 5 [Source:M	1
ENSMUSG00000000182	Fgf23	fibroblast growth factor 23 [Source:MGI Symbol;Ac	1
ENSMUSG00000000204	Slfn4	schlafen 4 [Source:MGI Symbol;Acc:MGI:1329010]	1
ENSMUSG00000000386	Mx1	myxovirus (influenza virus) resistance 1 [Source:M	1
ENSMUSG00000000435	Myf5	myogenic factor 5 [Source:MGI Symbol;Acc:MGI:97	1
ENSMUSG00000000544	Gpa33	glycoprotein A33 (transmembrane) [Source:MGI Sy	1
ENSMUSG00000000706	Btn1a1	butyrophilin, subfamily 1, member A1 [Source:MGI	1
ENSMUSG00000000982	Ccl3	chemokine (C-C motif) ligand 3 [Source:MGI Symbo	1
ENSMUSG00000001076	C1ql4	complement component 1, q subcomponent-like 4	1
ENSMUSG00000001225	Slc26a3	solute carrier family 26, member 3 [Source:MGI Sy	1
ENSMUSG00000001333	Sync	syncoilin [Source:MGI Symbol;Acc:MGI:1916078]	1
ENSMUSG00000001403	Ube2c	ubiquitin-conjugating enzyme E2C [Source:MGI Sy	1
ENSMUSG00000001510	Dlx3	distal-less homeobox 3 [Source:MGI Symbol;Acc:M	1
ENSMUSG00000001657	Hoxc8	homeobox C8 [Source:MGI Symbol;Acc:MGI:96198	1
ENSMUSG00000001865	Cpa3	carboxypeptidase A3, mast cell [Source:MGI Symbo	1

ENSMUSG00000002033	Cd3g	CD3 antigen, gamma polypeptide [Source:MGI Sym	1
ENSMUSG00000002100	Mybpc3	myosin binding protein C, cardiac [Source:MGI Sym	1
ENSMUSG00000002384	Bmp8b	bone morphogenetic protein 8b [Source:MGI Symb	1
ENSMUSG00000002814	Top3a	topoisomerase (DNA) III alpha [Source:MGI Symbol	1
ENSMUSG00000003341	Atp8b3	ATPase, class I, type 8B, member 3 [Source:MGI Syı	1
ENSMUSG00000003379	Cd79a	CD79A antigen (immunoglobulin-associated alpha)	1
ENSMUSG00000004328	Hif3a	hypoxia inducible factor 3, alpha subunit [Source:M	1
ENSMUSG00000004341	Gpx6	glutathione peroxidase 6 [Source:MGI Symbol;Acc:	1
ENSMUSG00000004473	Clec11a	C-type lectin domain family 11, member a [Source:	1
ENSMUSG00000004542	Psg19	pregnancy specific glycoprotein 19 [Source:MGI Syı	1
ENSMUSG00000004612	Nkg7	natural killer cell group 7 sequence [Source:MGI Sy	1
ENSMUSG00000004814	Ccl24	chemokine (C-C motif) ligand 24 [Source:MGI Symb	1
ENSMUSG00000004864	Mapk13	mitogen-activated protein kinase 13 [Source:MGI S	1
ENSMUSG00000005131	4930550C14Rik	RIKEN cDNA 4930550C14 gene [Source:MGI Symbo	1
ENSMUSG00000005493	Msh4	mutS homolog 4 (E. coli) [Source:MGI Symbol;Acc:N	1
ENSMUSG00000005510	Ndufs3	NADH dehydrogenase (ubiquinone) Fe-S protein 3 [	1
ENSMUSG00000005640	Insrr	insulin receptor-related receptor [Source:MGI Syml	1
ENSMUSG00000005883	Spo11	SPO11 meiotic protein covalently bound to DSB hoı	1
ENSMUSG00000005891	Prl4a1	prolactin family 4, subfamily a, member 1 [Source:N	1
ENSMUSG00000006056	Calcoco2	calcium binding and coiled-coil domain 2 [Source:M	1
ENSMUSG00000006389	Mpl	myeloproliferative leukemia virus oncogene [Sourc	1
ENSMUSG00000007591	Tssk4	testis-specific serine kinase 4 [Source:MGI Symbol;/	1
ENSMUSG00000007646	Rad51c	RAD51 homolog C [Source:MGI Symbol;Acc:MGI:21	1
ENSMUSG00000009070	Rtdr1	rhabdoid tumor deletion region gene 1 [Source:MG	1
ENSMUSG00000009075	Cabp7	calcium binding protein 7 [Source:MGI Symbol;Acc:	1
ENSMUSG00000009350	Mpo	myeloperoxidase [Source:MGI Symbol;Acc:MGI:971	1
ENSMUSG00000009646	Pla2g12b	phospholipase A2, group XIIB [Source:MGI Symbol;	1
ENSMUSG00000009654	Oit3	oncoprotein induced transcript 3 [Source:MGI Syml	1
ENSMUSG00000010086	Rnf112	ring finger protein 112 [Source:MGI Symbol;Acc:MC	1
ENSMUSG00000011118	Panx3	pannexin 3 [Source:MGI Symbol;Acc:MGI:1918881]	1
ENSMUSG00000012705	Retn	resistin [Source:MGI Symbol;Acc:MGI:1888506]	1
ENSMUSG00000013928	Thap8	THAP domain containing 8 [Source:MGI Symbol;Acc	1
ENSMUSG00000014243	Zswim7	zinc finger SWIM-type containing 7 [Source:MGI Sy	1
ENSMUSG00000014453	Blk	B lymphoid kinase [Source:MGI Symbol;Acc:MGI:88	1
ENSMUSG00000014609	Chrne	cholinergic receptor, nicotinic, epsilon polypeptide	1
ENSMUSG00000015083	C8g	complement component 8, gamma polypeptide [Sc	1
ENSMUSG00000015314	Slamf6	SLAM family member 6 [Source:MGI Symbol;Acc:M	1
ENSMUSG00000015854	Cd5l	CD5 antigen-like [Source:MGI Symbol;Acc:MGI:133	1
ENSMUSG00000016283	H2-M2	histocompatibility 2, M region locus 2 [Source:MGI	1
ENSMUSG00000016458	Wt1	Wilms tumor 1 homolog [Source:MGI Symbol;Acc:N	1
ENSMUSG00000016498	Pdcd1lg2	programmed cell death 1 ligand 2 [Source:MGI Sym	1
ENSMUSG00000017002	Slpi	secretory leukocyte peptidase inhibitor [Source:MG	1
ENSMUSG00000017007	Rbpjl	recombination signal binding protein for immunogl	1
ENSMUSG00000017195	Zpbp2	zona pellucida binding protein 2 [Source:MGI Symb	1
ENSMUSG00000017204	Gsdma	gasdermin A [Source:MGI Symbol;Acc:MGI:188950	1
ENSMUSG00000017720	Trp53tg5	transformation related protein 53 target 5 [Source:	1
ENSMUSG00000018238	Gdf9	growth differentiation factor 9 [Source:MGI Symbo	1

ENSMUSG00000018566	Slc2a4	solute carrier family 2 (facilitated glucose transport	1
ENSMUSG00000018656	Fam115e	family with sequence similarity 115, member E [Sou	1
ENSMUSG00000018925	Gm11435	predicted gene 11435 [Source:MGI Symbol;Acc:MG	1
ENSMUSG00000019027	Dnah1	dynein, axonemal, heavy chain 1 [Source:MGI Symb	1
ENSMUSG00000019913	Sim1	single-minded homolog 1 (Drosophila) [Source:MG	1
ENSMUSG00000020081	Tacr2	tachykinin receptor 2 [Source:MGI Symbol;Acc:MG	1
ENSMUSG00000020123	Avpr1a	arginine vasopressin receptor 1A [Source:MGI Symb	1
ENSMUSG00000020155	Kcnmb1	potassium large conductance calcium-activated cha	1
ENSMUSG00000020392	Cdkn2aipnl	CDKN2A interacting protein N-terminal like [Source	1
ENSMUSG00000020419	Hormad2	HORMA domain containing 2 [Source:MGI Symbol;	1
ENSMUSG00000020566	Atp6v1c2	ATPase, H+ transporting, lysosomal V1 subunit C2 [	1
ENSMUSG00000020583	Matn3	matrilin 3 [Source:MGI Symbol;Acc:MGI:1328350]	1
ENSMUSG00000020600	Slc7a15	solute carrier family 7 (cationic amino acid transpo	1
ENSMUSG00000020660	Pomc	pro-opiomelanocortin-alpha [Source:MGI Symbol;A	1
ENSMUSG00000020686	Gas2l2	growth arrest-specific 2 like 2 [Source:MGI Symbol;	1
ENSMUSG00000020690	Efcab3	EF-hand calcium binding domain 3 [Source:MGI Syr	1
ENSMUSG00000020734	Grin2c	glutamate receptor, ionotropic, NMDA2C (epsilon 3	1
ENSMUSG00000020826	Nos2	nitric oxide synthase 2, inducible [Source:MGI Symb	1
ENSMUSG00000020872	Tac4	tachykinin 4 [Source:MGI Symbol;Acc:MGI:193113C	1
ENSMUSG00000020950	Foxg1	forkhead box G1 [Source:MGI Symbol;Acc:MGI:134	1
ENSMUSG00000021041	2700073G19Rik	RIKEN cDNA 2700073G19 gene [Source:MGI Symbo	1
ENSMUSG00000021055	Esr2	estrogen receptor 2 (beta) [Source:MGI Symbol;Acc	1
ENSMUSG00000021056	Tex21	testis expressed gene 21 [Source:MGI Symbol;Acc:N	1
ENSMUSG00000021070	Bdkrb2	bradykinin receptor, beta 2 [Source:MGI Symbol;Ac	1
ENSMUSG00000021101	4930408O17Rik	RIKEN cDNA 4930408O17 gene [Source:MGI Symbo	1
ENSMUSG00000021213	Akr1c13	aldo-keto reductase family 1, member C13 [Source:	1
ENSMUSG00000021278	Amn	amnionless [Source:MGI Symbol;Acc:MGI:1934943]	1
ENSMUSG00000021322	Aoah	acyloxyacyl hydrolase [Source:MGI Symbol;Acc:MG	1
ENSMUSG00000021403	Serpinb9b	serine (or cysteine) peptidase inhibitor, clade B, me	1
ENSMUSG00000021432	Slc35b3	solute carrier family 35, member B3 [Source:MGI Sy	1
ENSMUSG00000021464	Ror2	receptor tyrosine kinase-like orphan receptor 2 [So	1
ENSMUSG00000021481	Zfp346	zinc finger protein 346 [Source:MGI Symbol;Acc:MC	1
ENSMUSG00000021536	Adcy2	adenylate cyclase 2 [Source:MGI Symbol;Acc:MGI:9	1
ENSMUSG00000021539	Lect2	leukocyte cell-derived chemotaxin 2 [Source:MGI S	1
ENSMUSG00000021545	1700067P10Rik	RIKEN cDNA 1700067P10 gene [Source:MGI Symbo	1
ENSMUSG00000021611	Tert	telomerase reverse transcriptase [Source:MGI Sym	1
ENSMUSG00000021620	Acot12	acyl-CoA thioesterase 12 [Source:MGI Symbol;Acc:I	1
ENSMUSG00000021697	Depdc1b	DEP domain containing 1B [Source:MGI Symbol;Acc	1
ENSMUSG00000021718	4933425L06Rik	RIKEN cDNA 4933425L06 gene [Source:MGI Symbo	1
ENSMUSG00000021872	Rnase10	ribonuclease, RNase A family, 10 (non-active) [Sour	1
ENSMUSG00000021886	Gpr65	G-protein coupled receptor 65 [Source:MGI Symbo	1
ENSMUSG00000021933	Gucy1b2	guanylate cyclase 1, soluble, beta 2 [Source:MGI Sy	1
ENSMUSG00000021953	Tdh	L-threonine dehydrogenase [Source:MGI Symbol;A	1
ENSMUSG00000021966	Prss52	protease, serine, 52 [Source:MGI Symbol;Acc:MGI:	1
ENSMUSG00000021997	Lrrc63	leucine rich repeat containing 63 [Source:MGI Syml	1
ENSMUSG00000022018	Rgcc	regulator of cell cycle [Source:MGI Symbol;Acc:MG	1
ENSMUSG00000022039	Adam2	a disintegrin and metallopeptidase domain 2 [Sour	1

ENSMUSG00000022057	Adamdec1	ADAM-like, decysin 1 [Source:MGI Symbol;Acc:MGI	1
ENSMUSG00000022085	Pebp4	phosphatidylethanolamine binding protein 4 [Sourc	1
ENSMUSG00000022102	Dok2	docking protein 2 [Source:MGI Symbol;Acc:MGI:13	1
ENSMUSG00000022187	Gm5546	predicted gene 5546 [Source:MGI Symbol;Acc:MGI	1
ENSMUSG00000022219	Cideb	cell death-inducing DNA fragmentation factor, alph	1
ENSMUSG00000022296	Baalc	brain and acute leukemia, cytoplasmic [Source:MG	1
ENSMUSG00000022322	Shcbp1	Shc SH2-domain binding protein 1 [Source:MGI Syn	1
ENSMUSG00000022388	Ttll8	tubulin tyrosine ligase-like family, member 8 [Sourc	1
ENSMUSG00000022455	Wbp2nl	WBP2 N-terminal like [Source:MGI Symbol;Acc:MG	1
ENSMUSG00000022589	Cyp11b2	cytochrome P450, family 11, subfamily b, polypepti	1
ENSMUSG00000022652	Morc1	microrchidia 1 [Source:MGI Symbol;Acc:MGI:13167	1
ENSMUSG00000022657	Cd96	CD96 antigen [Source:MGI Symbol;Acc:MGI:193436	1
ENSMUSG00000022696	Sidt1	SID1 transmembrane family, member 1 [Source:MG	1
ENSMUSG00000022750	Klhl22	kelch-like 22 [Source:MGI Symbol;Acc:MGI:133799	1
ENSMUSG00000022857	Tmprss15	transmembrane protease, serine 15 [Source:MGI Sy	1
ENSMUSG00000022915	1700093J21Rik	RIKEN cDNA 1700093J21 gene [Source:MGI Symbol	1
ENSMUSG00000022945	Chaf1b	chromatin assembly factor 1, subunit B (p60) [Sour	1
ENSMUSG00000023070	Rgn	regucalcin [Source:MGI Symbol;Acc:MGI:108024]	1
ENSMUSG00000023341	Mx2	myxovirus (influenza virus) resistance 2 [Source:MG	1
ENSMUSG00000023391	Dlx2	distal-less homeobox 2 [Source:MGI Symbol;Acc:M	1
ENSMUSG00000023902	Zscan10	zinc finger and SCAN domain containing 10 [Source	1
ENSMUSG00000023903	Mmp25	matrix metallopeptidase 25 [Source:MGI Symbol;Ac	1
ENSMUSG00000023926	Rhag	Rhesus blood group-associated A glycoprotein [Sou	1
ENSMUSG00000023964	Calcr	calcitonin receptor [Source:MGI Symbol;Acc:MGI:1	1
ENSMUSG00000024029	Tff3	trefoil factor 3, intestinal [Source:MGI Symbol;Acc:	1
ENSMUSG00000024056	Ndc80	NDC80 homolog, kinetochore complex component	1
ENSMUSG00000024114	Prss41	protease, serine, 41 [Source:MGI Symbol;Acc:MGI:	1
ENSMUSG00000024155	Meiob	meiosis specific with OB domains [Source:MGI Sym	1
ENSMUSG00000024175	Tekt4	tektin 4 [Source:MGI Symbol;Acc:MGI:1919090]	1
ENSMUSG00000024184	Pdia2	protein disulfide isomerase associated 2 [Source:M	1
ENSMUSG00000024366	Gfra3	glial cell line derived neurotrophic factor family rec	1
ENSMUSG00000024409	Psors1c2	psoriasis susceptibility 1 candidate 2 (human) [Sour	1
ENSMUSG00000024535	Snx24	sorting nexing 24 [Source:MGI Symbol;Acc:MGI:191	1
ENSMUSG00000024552	Slc14a2	solute carrier family 14 (urea transporter), member	1
ENSMUSG00000024553	Galr1	galanin receptor 1 [Source:MGI Symbol;Acc:MGI:10	1
ENSMUSG00000024650	Slc22a6	solute carrier family 22 (organic anion transporter),	1
ENSMUSG00000024675	Ms4a4c	membrane-spanning 4-domains, subfamily A, mem	1
ENSMUSG00000024682	Gif	gastric intrinsic factor [Source:MGI Symbol;Acc:MG	1
ENSMUSG00000024863	Mbl2	mannose-binding lectin (protein C) 2 [Source:MGI S	1
ENSMUSG00000024868	Dkk1	dickkopf homolog 1 (Xenopus laevis) [Source:MGI S	1
ENSMUSG00000024883	Rin1	Ras and Rab interactor 1 [Source:MGI Symbol;Acc:M	1
ENSMUSG00000025140	Pycr1	pyrroline-5-carboxylate reductase 1 [Source:MGI Sy	1
ENSMUSG00000025163	Cd7	CD7 antigen [Source:MGI Symbol;Acc:MGI:88344]	1
ENSMUSG00000025197	Cyp2c44	cytochrome P450, family 2, subfamily c, polypeptid	1
ENSMUSG00000025383	Il23a	interleukin 23, alpha subunit p19 [Source:MGI Sym	1
ENSMUSG00000025418	Bsnd	Bartter syndrome, infantile, with sensorineural dea	1
ENSMUSG00000025521	Tmem192	transmembrane protein 192 [Source:MGI Symbol;A	1

ENSMUSG00000025716	Myo3a	myosin IIIA [Source:MGI Symbol;Acc:MGI:2183924]	1
ENSMUSG00000025726	Slc28a1	solute carrier family 28 (sodium-coupled nucleoside	1
ENSMUSG00000025946	Pth2r	parathyroid hormone 2 receptor [Source:MGI Symb	1
ENSMUSG00000025977	Boll	bol, boule-like (Drosophila) [Source:MGI Symbol;Ac	1
ENSMUSG00000026009	Icos	inducible T cell co-stimulator [Source:MGI Symbol;A	1
ENSMUSG00000026065	Slc9a4	solute carrier family 9 (sodium/hydrogen exchange	1
ENSMUSG00000026068	Il18rap	interleukin 18 receptor accessory protein [Source:M	1
ENSMUSG00000026069	Il1rl1	interleukin 1 receptor-like 1 [Source:MGI Symbol;A	1
ENSMUSG00000026175	Vil1	villin 1 [Source:MGI Symbol;Acc:MGI:98930]	1
ENSMUSG00000026196	Bard1	BRCA1 associated RING domain 1 [Source:MGI Sym	1
ENSMUSG00000026224	4933407L21Rik	RIKEN cDNA 4933407L21 gene [Source:MGI Symbo	1
ENSMUSG00000026237	Nmur1	neuromedin U receptor 1 [Source:MGI Symbol;Acc:	1
ENSMUSG00000026295	Spp2	secreted phosphoprotein 2 [Source:MGI Symbol;Ac	1
ENSMUSG00000026459	Myog	myogenin [Source:MGI Symbol;Acc:MGI:97276]	1
ENSMUSG00000026479	Lamc2	laminin, gamma 2 [Source:MGI Symbol;Acc:MGI:99	1
ENSMUSG00000026567	Adcy10	adenylate cyclase 10 [Source:MGI Symbol;Acc:MGI:	1
ENSMUSG00000026592	Tex35	testis expressed 35 [Source:MGI Symbol;Acc:MGI:1	1
ENSMUSG00000026645	Olah	oleoyl-ACP hydrolase [Source:MGI Symbol;Acc:MGI	1
ENSMUSG00000026683	Nuf2	NUF2, NDC80 kinetochore complex component, ho	1
ENSMUSG00000026691	Fmo3	flavin containing monooxygenase 3 [Source:MGI Sy	1
ENSMUSG00000026736	4930426L09Rik	RIKEN cDNA 4930426L09 gene [Source:MGI Symbo	1
ENSMUSG00000026751	Nr5a1	nuclear receptor subfamily 5, group A, member 1 [	1
ENSMUSG00000026786	Apbb1ip	amyloid beta (A4) precursor protein-binding, family	1
ENSMUSG00000026832	Cytip	cytohesin 1 interacting protein [Source:MGI Symbo	1
ENSMUSG00000026874	Hc	hemolytic complement [Source:MGI Symbol;Acc:M	1
ENSMUSG00000026943	Lcn12	lipocalin 12 [Source:MGI Symbol;Acc:MGI:1924951	1
ENSMUSG00000026955	Sapcd2	suppressor APC domain containing 2 [Source:MGI S	1
ENSMUSG00000027225	Duoxa2	dual oxidase maturation factor 2 [Source:MGI Symb	1
ENSMUSG00000027246	Ell3	elongation factor RNA polymerase II-like 3 [Source:	1
ENSMUSG00000027261	Hao1	hydroxyacid oxidase 1, liver [Source:MGI Symbol;Ac	1
ENSMUSG00000027296	Itpka	inositol 1,4,5-trisphosphate 3-kinase A [Source:MG	1
ENSMUSG00000027338	Prnd	prion protein dublet [Source:MGI Symbol;Acc:MGI:	1
ENSMUSG00000027359	Slc27a2	solute carrier family 27 (fatty acid transporter), me	1
ENSMUSG00000027376	Prom2	prominin 2 [Source:MGI Symbol;Acc:MGI:2138997]	1
ENSMUSG00000027496	Aurka	aurora kinase A [Source:MGI Symbol;Acc:MGI:8946	1
ENSMUSG00000027505	Fam209	family with sequence similarity 209 [Source:MGI Sy	1
ENSMUSG00000027654	Fam83d	family with sequence similarity 83, member D [Sou	1
ENSMUSG00000027713	1810062G17Rik	RIKEN cDNA 1810062G17 gene [Source:MGI Symbo	1
ENSMUSG00000027744	Stoml3	stomatin (Epb7.2)-like 3 [Source:MGI Symbol;Acc:M	1
ENSMUSG00000027880	Slc25a54	solute carrier family 25, member 54 [Source:MGI Sy	1
ENSMUSG00000027938	Creb3l4	cAMP responsive element binding protein 3-like 4 [	1
ENSMUSG00000028004	Npy2r	neuropeptide Y receptor Y2 [Source:MGI Symbol;Ac	1
ENSMUSG00000028141	Oaz3	ornithine decarboxylase antizyme 3 [Source:MGI Sy	1
ENSMUSG00000028238	Atp6v0d2	ATPase, H+ transporting, lysosomal V0 subunit D2 [	1
ENSMUSG00000028259	Fhl5	four and a half LIM domains 5 [Source:MGI Symbol	1
ENSMUSG00000028457	Atp8b5	ATPase, class I, type 8B, member 5 [Source:MGI Syr	1
ENSMUSG00000028536	2610528J11Rik	RIKEN cDNA 2610528J11 gene [Source:MGI Symbol	1

ENSMUSG00000028575	Eqtn	equatorin, sperm acrosome associated [Source:MG	1
ENSMUSG00000028591	Pramef12	PRAME family member 12 [Source:MGI Symbol;Acc	1
ENSMUSG00000028610	Dmrtb1	DMRT-like family B with proline-rich C-terminal, 1 [	1
ENSMUSG00000028612	4930430J20Rik	RIKEN cDNA 4930430J20 gene [Source:MGI Symbol	1
ENSMUSG00000028712	Cyp4a31	cytochrome P450, family 4, subfamily a, polypeptid	1
ENSMUSG00000028715	Cyp4a14	cytochrome P450, family 4, subfamily a, polypeptid	1
ENSMUSG00000028718	Stil	Scl/Tal1 interrupting locus [Source:MGI Symbol;Acc	1
ENSMUSG00000028784	Spocd1	SPOC domain containing 1 [Source:MGI Symbol;Acc	1
ENSMUSG00000028801	Stpg1	sperm tail PG rich repeat containing 1 [Source:MGI	1
ENSMUSG00000028813	CK137956	cDNA sequence CK137956 [Source:MGI Symbol;Acc	1
ENSMUSG00000028850	Gpatch3	G patch domain containing 3 [Source:MGI Symbol;A	1
ENSMUSG00000028940	Hes2	hairy and enhancer of split 2 (Drosophila) [Source:N	1
ENSMUSG00000029015	Slc26a5	solute carrier family 26, member 5 [Source:MGI Syr	1
ENSMUSG00000029049	Morn1	MORN repeat containing 1 [Source:MGI Symbol;Acc	1
ENSMUSG00000029102	Hgfac	hepatocyte growth factor activator [Source:MGI Sy	1
ENSMUSG00000029193	Cckar	cholecystokinin A receptor [Source:MGI Symbol;Acc	1
ENSMUSG00000029195	Klb	klotho beta [Source:MGI Symbol;Acc:MGI:1932466	1
ENSMUSG00000029204	Rhoh	ras homolog gene family, member H [Source:MGI S	1
ENSMUSG00000029248	1700023E05Rik	RIKEN cDNA 1700023E05 gene [Source:MGI Symbo	1
ENSMUSG00000029288	Ambn	ameloblastin [Source:MGI Symbol;Acc:MGI:104655	1
ENSMUSG00000029321	Slc10a6	solute carrier family 10 (sodium/bile acid cotranspo	1
ENSMUSG00000029423	Piwil1	piwi-like RNA-mediated gene silencing 1 [Source:M	1
ENSMUSG00000029431	B3gnt4	UDP-GlcNAc:betaGal beta-1,3-N-acetylglucosaminy	1
ENSMUSG00000029445	Hpd	4-hydroxyphenylpyruvic acid dioxygenase [Source:I	1
ENSMUSG00000029595	Lhx5	LIM homeobox protein 5 [Source:MGI Symbol;Acc:I	1
ENSMUSG00000029711	Epo	erythropoietin [Source:MGI Symbol;Acc:MGI:95407	1
ENSMUSG00000029736	Nobox	NOBOX oogenesis homeobox [Source:MGI Symbol;	1
ENSMUSG00000029882	2210010C04Rik	RIKEN cDNA 2210010C04 gene [Source:MGI Symbo	1
ENSMUSG00000030041	M1ap	meiosis 1 associated protein [Source:MGI Symbol;A	1
ENSMUSG00000030054	Gp9	glycoprotein 9 (platelet) [Source:MGI Symbol;Acc:N	1
ENSMUSG00000030068	Gm20696	predicted gene 20696 [Source:MGI Symbol;Acc:MG	1
ENSMUSG00000030143	Gm8882	predicted gene 8882 [Source:MGI Symbol;Acc:MGI:	1
ENSMUSG00000030149	Klrk1	killer cell lectin-like receptor subfamily K, member	1
ENSMUSG00000030172	Erc1	ELKS/RAB6-interacting/CAST family member 1 [Sou	1
ENSMUSG00000030200	Bcl2l14	BCL2-like 14 (apoptosis facilitator) [Source:MGI Syn	1
ENSMUSG00000030316	Tamm41	TAM41, mitochondrial translocator assembly and m	1
ENSMUSG00000030321	Efcab12	EF-hand calcium binding domain 12 [Source:MGI Sy	1
ENSMUSG00000030336	Cd27	CD27 antigen [Source:MGI Symbol;Acc:MGI:88326]	1
ENSMUSG00000030344	Akap3	A kinase (PRKA) anchor protein 3 [Source:MGI Sym	1
ENSMUSG00000030345	Dyrk4	dual-specificity tyrosine-(Y)-phosphorylation regula	1
ENSMUSG00000030361	Klrb1a	killer cell lectin-like receptor subfamily B member 1	1
ENSMUSG00000030406	Gipr	gastric inhibitory polypeptide receptor [Source:MG	1
ENSMUSG00000030491	Tdrd12	tudor domain containing 12 [Source:MGI Symbol;A	1
ENSMUSG00000030607	Acan	aggrecan [Source:MGI Symbol;Acc:MGI:99602]	1
ENSMUSG00000030617	Ccdc83	coiled-coil domain containing 83 [Source:MGI Symb	1
ENSMUSG00000030623	1700019G06Rik	RIKEN cDNA 1700019G06 gene [Source:MGI Symbo	1
ENSMUSG00000030666	Calcb	calcitonin-related polypeptide, beta [Source:MGI Sy	1

ENSMUSG00000030669	Calca	calcitonin/calcitonin-related polypeptide, alpha [So	1
ENSMUSG00000030769	Slc5a11	solute carrier family 5 (sodium/glucose cotransport	1
ENSMUSG00000030771	Micalcl	MICAL C-terminal like [Source:MGI Symbol;Acc:MG	1
ENSMUSG00000030775	Trat1	T cell receptor associated transmembrane adaptor	1
ENSMUSG00000030804	Gm21974	predicted gene 21974 [Source:MGI Symbol;Acc:MG	1
ENSMUSG00000030823	9130019O22Rik	RIKEN cDNA 9130019O22 gene [Source:MGI Symbo	1
ENSMUSG00000030827	Fgf21	fibroblast growth factor 21 [Source:MGI Symbol;Ac	1
ENSMUSG00000030858	1700007K09Rik	RIKEN cDNA 1700007K09 gene [Source:MGI Symbo	1
ENSMUSG00000030859	Fam24a	family with sequence similarity 24, member A [Sour	1
ENSMUSG00000030873	Scnn1b	sodium channel, nonvoltage-gated 1 beta [Source:N	1
ENSMUSG00000030972	Acsm5	acyl-CoA synthetase medium-chain family member	1
ENSMUSG00000031073	Fgf15	fibroblast growth factor 15 [Source:MGI Symbol;Ac	1
ENSMUSG00000031098	Syt8	synaptotagmin VIII [Source:MGI Symbol;Acc:MGI:1	1
ENSMUSG00000031104	Rab33a	RAB33A, member of RAS oncogene family [Source:	1
ENSMUSG00000031111	Igsf1	immunoglobulin superfamily, member 1 [Source:M	1
ENSMUSG00000031146	Plp2	proteolipid protein 2 [Source:MGI Symbol;Acc:MGI	1
ENSMUSG00000031147	Magix	MAGI family member, X-linked [Source:MGI Symbo	1
ENSMUSG00000031156	Slc35a2	solute carrier family 35 (UDP-galactose transporter	1
ENSMUSG00000031162	Gata1	GATA binding protein 1 [Source:MGI Symbol;Acc:M	1
ENSMUSG00000031163	Glod5	glyoxalase domain containing 5 [Source:MGI Symb	1
ENSMUSG00000031294	D630029K05Rik	RIKEN cDNA D630029K05 gene [Source:MGI Symbo	1
ENSMUSG00000031330	Zcchc13	zinc finger, CCHC domain containing 13 [Source:MG	1
ENSMUSG00000031364	Grpr	gastrin releasing peptide receptor [Source:MGI Sym	1
ENSMUSG00000031478	Nek3	NIMA (never in mitosis gene a)-related expressed k	1
ENSMUSG00000031594	Fgl1	fibrinogen-like protein 1 [Source:MGI Symbol;Acc:N	1
ENSMUSG00000031610	Scrg1	scrapie responsive gene 1 [Source:MGI Symbol;Acc	1
ENSMUSG00000031660	Brd7	bromodomain containing 7 [Source:MGI Symbol;Ac	1
ENSMUSG00000031712	Il15	interleukin 15 [Source:MGI Symbol;Acc:MGI:10301	1
ENSMUSG00000031766	Slc12a3	solute carrier family 12, member 3 [Source:MGI Syr	1
ENSMUSG00000031779	Ccl22	chemokine (C-C motif) ligand 22 [Source:MGI Symb	1
ENSMUSG00000031880	Rrad	Ras-related associated with diabetes [Source:MGI S	1
ENSMUSG00000031881	Cdh16	cadherin 16 [Source:MGI Symbol;Acc:MGI:106671]	1
ENSMUSG00000031898	Dpep3	dipeptidase 3 [Source:MGI Symbol;Acc:MGI:19191	1
ENSMUSG00000031932	Gpr83	G protein-coupled receptor 83 [Source:MGI Symbo	1
ENSMUSG00000031937	Vstm5	V-set and transmembrane domain containing 5 [So	1
ENSMUSG00000031981	Capn9	calpain 9 [Source:MGI Symbol;Acc:MGI:1920897]	1
ENSMUSG00000032015	Pou2f3	POU domain, class 2, transcription factor 3 [Source	1
ENSMUSG00000032062	2310030G06Rik	RIKEN cDNA 2310030G06 gene [Source:MGI Symbo	1
ENSMUSG00000032066	Bco2	beta-carotene oxygenase 2 [Source:MGI Symbol;Ac	1
ENSMUSG00000032207	Lipc	lipase, hepatic [Source:MGI Symbol;Acc:MGI:96216	1
ENSMUSG00000032226	Gcnt3	glucosaminyl (N-acetyl) transferase 3, mucin type [	1
ENSMUSG00000032269	Htr3a	5-hydroxytryptamine (serotonin) receptor 3A [Sour	1
ENSMUSG00000032360	Hcrtr2	hypocretin (orexin) receptor 2 [Source:MGI Symbol	1
ENSMUSG00000032417	Rwdd2a	RWD domain containing 2A [Source:MGI Symbol;Ac	1
ENSMUSG00000032484	Ngp	neutrophilic granule protein [Source:MGI Symbol;A	1
ENSMUSG00000032487	Ptgs2	prostaglandin-endoperoxide synthase 2 [Source:MG	1
ENSMUSG00000032494	Tdgf1	teratocarcinoma-derived growth factor 1 [Source:N	1

ENSMUSG00000032503	Arpp21	cyclic AMP-regulated phosphoprotein, 21 [Source:M	1
ENSMUSG00000032530	Lyzl4	lysozyme-like 4 [Source:MGI Symbol;Acc:MGI:1916	1
ENSMUSG00000032572	Col6a4	collagen, type VI, alpha 4 [Source:MGI Symbol;Acc:	1
ENSMUSG00000032584	Mst1r	macrophage stimulating 1 receptor (c-met-related	1
ENSMUSG00000032591	Mst1	macrophage stimulating 1 (hepatocyte growth fact	1
ENSMUSG00000032680	6820408C15Rik	RIKEN cDNA 6820408C15 gene [Source:MGI Symbo	1
ENSMUSG00000032726	Bmp8a	bone morphogenetic protein 8a [Source:MGI Symb	1
ENSMUSG00000032921	Odf4	outer dense fiber of sperm tails 4 [Source:MGI Sym	1
ENSMUSG00000032999	Nlrp4f	NLR family, pyrin domain containing 4F [Source:MG	1
ENSMUSG00000033036	Gm7879	predicted pseudogene 7879 [Source:MGI Symbol;A	1
ENSMUSG00000033207	Mamdc2	MAM domain containing 2 [Source:MGI Symbol;Acc	1
ENSMUSG00000033227	Wnt6	wingless-related MMTV integration site 6 [Source:M	1
ENSMUSG00000033409	Syce1l	synaptonemal complex central element protein 1 li	1
ENSMUSG00000033470	Cysltr2	cysteinyl leukotriene receptor 2 [Source:MGI Symb	1
ENSMUSG00000033486	Catsper2	cation channel, sperm associated 2 [Source:MGI Sy	1
ENSMUSG00000033491	Prss35	protease, serine, 35 [Source:MGI Symbol;Acc:MGI:2	1
ENSMUSG00000033726	Emx1	empty spiracles homeobox 1 [Source:MGI Symbol;A	1
ENSMUSG00000033731	3300002A11Rik	RIKEN cDNA 3300002A11 gene [Source:MGI Symbo	1
ENSMUSG00000033987	Dnah17	dynein, axonemal, heavy chain 17 [Source:MGI Sym	1
ENSMUSG00000034028	Cd226	CD226 antigen [Source:MGI Symbol;Acc:MGI:30396	1
ENSMUSG00000034064	Poglut1	protein O-glucosyltransferase 1 [Source:MGI Symbo	1
ENSMUSG00000034112	Atp2c2	ATPase, Ca++ transporting, type 2C, member 2 [Sou	1
ENSMUSG00000034266	Batf	basic leucine zipper transcription factor, ATF-like [S	1
ENSMUSG00000034290	Nek9	NIMA (never in mitosis gene a)-related expressed k	1
ENSMUSG00000034353	Ramp1	receptor (calcitonin) activity modifying protein 1 [S	1
ENSMUSG00000034623	Prss55	protease, serine, 55 [Source:MGI Symbol;Acc:MGI:1	1
ENSMUSG00000034764	1700006J14Rik	RIKEN cDNA 1700006J14 gene [Source:MGI Symbol	1
ENSMUSG00000034871	Fam151a	family with sequence simliarity 151, member A [Sou	1
ENSMUSG00000034959	5031414D18Rik	RIKEN cDNA 5031414D18 gene [Source:MGI Symbo	1
ENSMUSG00000035031	C8a	complement component 8, alpha polypeptide [Sou	1
ENSMUSG00000035277	Arx	aristaless related homeobox [Source:MGI Symbol;A	1
ENSMUSG00000035352	Ccl12	chemokine (C-C motif) ligand 12 [Source:MGI Symb	1
ENSMUSG00000035373	Ccl7	chemokine (C-C motif) ligand 7 [Source:MGI Symbo	1
ENSMUSG00000035435	Abca17	ATP-binding cassette, sub-family A (ABC1), member	1
ENSMUSG00000035458	Tnni3	troponin I, cardiac 3 [Source:MGI Symbol;Acc:MGI:9	1
ENSMUSG00000035842	Ddx11	DEAD/H (Asp-Glu-Ala-Asp/His) box helicase 11 [Sou	1
ENSMUSG00000035875	AI182371	expressed sequence AI182371 [Source:MGI Symbol	1
ENSMUSG00000036117	Il5	interleukin 5 [Source:MGI Symbol;Acc:MGI:96557]	1
ENSMUSG00000036208	BC027231	cDNA sequence BC027231 [Source:MGI Symbol;Acc	1
ENSMUSG00000036216	Leap2	liver-expressed antimicrobial peptide 2 [Source:MG	1
ENSMUSG00000036551	Akap14	A kinase (PRKA) anchor protein 14 [Source:MGI Syn	1
ENSMUSG00000036557	1700011E24Rik	RIKEN cDNA 1700011E24 gene [Source:MGI Symbo	1
ENSMUSG00000036587	Fut7	fucosyltransferase 7 [Source:MGI Symbol;Acc:MGI:	1
ENSMUSG00000036855	Gjd4	gap junction protein, delta 4 [Source:MGI Symbol;A	1
ENSMUSG00000036858	Ptcra	pre T cell antigen receptor alpha [Source:MGI Symb	1
ENSMUSG00000036925	Spt1	salivary protein 1 [Source:MGI Symbol;Acc:MGI:983	1
ENSMUSG00000037139	Myom3	myomesin family, member 3 [Source:MGI Symbol;A	1

ENSMUSG00000037157	Il22ra1	interleukin 22 receptor, alpha 1 [Source:MGI Symb	1
ENSMUSG00000037167	Spaca5	sperm acrosome associated 5 [Source:MGI Symbol;	1
ENSMUSG00000037171	Nodal	nodal [Source:MGI Symbol;Acc:MGI:97359]	1
ENSMUSG00000037204	9430023L20Rik	RIKEN cDNA 9430023L20 gene [Source:MGI Symbo	1
ENSMUSG00000037406	Htra4	HtrA serine peptidase 4 [Source:MGI Symbol;Acc:M	1
ENSMUSG00000037544	Dlgap5	discs, large (Drosophila) homolog-associated prote	1
ENSMUSG00000037636	Slc25a43	solute carrier family 25, member 43 [Source:MGI S	1
ENSMUSG00000037686	Aspg	asparaginase homolog (S. cerevisiae) [Source:MGI S	1
ENSMUSG00000037798	Mat1a	methionine adenosyltransferase I, alpha [Source:M	1
ENSMUSG00000037944	Ccr7	chemokine (C-C motif) receptor 7 [Source:MGI Sym	1
ENSMUSG00000037973	Ccdc129	coiled-coil domain containing 129 [Source:MGI Sym	1
ENSMUSG00000037982	Gm9725	predicted gene 9725 [Source:MGI Symbol;Acc:MGI	1
ENSMUSG00000037997	Parp11	poly (ADP-ribose) polymerase family, member 11 [	1
ENSMUSG00000038044	Cct8l1	chaperonin containing TCP1, subunit 8 (theta)-like	1
ENSMUSG00000038086	Hspb2	heat shock protein 2 [Source:MGI Symbol;Acc:MGI	1
ENSMUSG00000038141	Tmem181a	transmembrane protein 181A [Source:MGI Symbol	1
ENSMUSG00000038192	Cer1	cerberus 1 homolog (Xenopus laevis) [Source:MGI S	1
ENSMUSG00000038259	Gdf5	growth differentiation factor 5 [Source:MGI Symbo	1
ENSMUSG00000038393	Txnip	thioredoxin interacting protein [Source:MGI Symbo	1
ENSMUSG00000038421	Fcrla	Fc receptor-like A [Source:MGI Symbol;Acc:MGI:21	1
ENSMUSG00000038491	4930520K10Rik	RIKEN cDNA 4930520K10 gene [Source:MGI Symbo	1
ENSMUSG00000038560	Sp6	trans-acting transcription factor 6 [Source:MGI Sym	1
ENSMUSG00000038600	Atp6v0a4	ATPase, H+ transporting, lysosomal V0 subunit A4 [	1
ENSMUSG00000038607	Gng10	guanine nucleotide binding protein (G protein), gar	1
ENSMUSG00000038651	Sycp2l	synaptonemal complex protein 2-like [Source:MGI S	1
ENSMUSG00000038692	Hoxb4	homeobox B4 [Source:MGI Symbol;Acc:MGI:96185	1
ENSMUSG00000038745	Nlrp6	NLR family, pyrin domain containing 6 [Source:MGI	1
ENSMUSG00000038768	9130409I23Rik	RIKEN cDNA 9130409I23 gene [Source:MGI Symbol	1
ENSMUSG00000038782	1700028J19Rik	RIKEN cDNA 1700028J19 gene [Source:MGI Symbol	1
ENSMUSG00000038859	Baiap2l1	BAI1-associated protein 2-like 1 [Source:MGI Symb	1
ENSMUSG00000038925	E330034G19Rik	RIKEN cDNA E330034G19 gene [Source:MGI Symbo	1
ENSMUSG00000039000	Ube3c	ubiquitin protein ligase E3C [Source:MGI Symbol;A	1
ENSMUSG00000039063	Echdc3	enoyl Coenzyme A hydratase domain containing 3 [	1
ENSMUSG00000039070	Cpa4	carboxypeptidase A4 [Source:MGI Symbol;Acc:MGI	1
ENSMUSG00000039098	Gm9767	predicted gene 9767 [Source:MGI Symbol;Acc:MGI	1
ENSMUSG00000039187	Fanci	Fanconi anemia, complementation group I [Source:	1
ENSMUSG00000039315	Clnk	cytokine-dependent hematopoietic cell linker [Sour	1
ENSMUSG00000039329	Tex19.1	testis expressed gene 19.1 [Source:MGI Symbol;Ac	1
ENSMUSG00000039339	2010001E11Rik	RIKEN cDNA 2010001E11 gene [Source:MGI Symbo	1
ENSMUSG00000039492	Ccdc27	coiled-coil domain containing 27 [Source:MGI Symb	1
ENSMUSG00000039639	Kcne1	potassium voltage-gated channel, Isk-related subfa	1
ENSMUSG00000039814	Xkr5	X Kell blood group precursor-related family, memb	1
ENSMUSG00000039981	Zc3h12d	zinc finger CCCH type containing 12D [Source:MGI S	1
ENSMUSG00000040013	Fkbp6	FK506 binding protein 6 [Source:MGI Symbol;Acc:M	1
ENSMUSG00000040017	Saa4	serum amyloid A 4 [Source:MGI Symbol;Acc:MGI:9	1
ENSMUSG00000040026	Saa3	serum amyloid A 3 [Source:MGI Symbol;Acc:MGI:9	1
ENSMUSG00000040078	Gm9769	predicted pseudogene 9769 [Source:MGI Symbol;A	1

ENSMUSG00000040328	Olfr56	olfactory receptor 56 [Source:MGI Symbol;Acc:MGI	1
ENSMUSG00000040485	Lrrc52	leucine rich repeat containing 52 [Source:MGI Syml	1
ENSMUSG00000040505	Abcg5	ATP-binding cassette, sub-family G (WHITE), memb	1
ENSMUSG00000040552	C3ar1	complement component 3a receptor 1 [Source:MG	1
ENSMUSG00000040600	Eps8l3	EPS8-like 3 [Source:MGI Symbol;Acc:MGI:2139743]	1
ENSMUSG00000040601	Nlrp4a	NLR family, pyrin domain containing 4A [Source:MG	1
ENSMUSG00000040808	S100g	S100 calcium binding protein G [Source:MGI Symbo	1
ENSMUSG00000040904	Gm21988	predicted gene 21988 [Source:MGI Symbol;Acc:MG	1
ENSMUSG00000040919	4930505A04Rik	RIKEN cDNA 4930505A04 gene [Source:MGI Symbo	1
ENSMUSG00000040985	Sun3	Sad1 and UNC84 domain containing 3 [Source:MGI	1
ENSMUSG00000041237	Pklr	pyruvate kinase liver and red blood cell [Source:MG	1
ENSMUSG00000041255	Tmco5b	transmembrane and coiled-coil domains 5B [Source	1
ENSMUSG00000041287	Sox15	SRY-box containing gene 15 [Source:MGI Symbol;Ac	1
ENSMUSG00000041324	Inhba	inhibin beta-A [Source:MGI Symbol;Acc:MGI:96570	1
ENSMUSG00000041347	Bdkrb1	bradykinin receptor, beta 1 [Source:MGI Symbol;Ac	1
ENSMUSG00000041358	Nutm1	NUT midline carcinoma, family member 1 [Source:M	1
ENSMUSG00000041565	L3mbtl4	l(3)mbt-like 4 (Drosophila) [Source:MGI Symbol;Acc	1
ENSMUSG00000041673	Lrrc18	leucine rich repeat containing 18 [Source:MGI Syml	1
ENSMUSG00000041750	Cd1d2	CD1d2 antigen [Source:MGI Symbol;Acc:MGI:10767	1
ENSMUSG00000041945	Mfsd9	major facilitator superfamily domain containing 9 [S	1
ENSMUSG00000042102	Dmgdh	dimethylglycine dehydrogenase precursor [Source:	1
ENSMUSG00000042165	Gm9774	predicted pseudogene 9774 [Source:MGI Symbol;A	1
ENSMUSG00000042195	Slc35f2	solute carrier family 35, member F2 [Source:MGI Sy	1
ENSMUSG00000042360	4930433N12Rik	RIKEN cDNA 4930433N12 gene [Source:MGI Symbo	1
ENSMUSG00000042414	Prdm14	PR domain containing 14 [Source:MGI Symbol;Acc:	1
ENSMUSG00000042638	Gucy2c	guanylate cyclase 2c [Source:MGI Symbol;Acc:MGI:	1
ENSMUSG00000042788	Fam166b	family with sequence similarity 166, member B [Sou	1
ENSMUSG00000042812	Foxf1	forkhead box F1 [Source:MGI Symbol;Acc:MGI:134	1
ENSMUSG00000042817	Flt3	FMS-like tyrosine kinase 3 [Source:MGI Symbol;Acc	1
ENSMUSG00000042962	Gm5436	predicted pseudogene 5436 [Source:MGI Symbol;A	1
ENSMUSG00000043050	Tnp2	transition protein 2 [Source:MGI Symbol;Acc:MGI:9	1
ENSMUSG00000043085	Tmem82	transmembrane protein 82 [Source:MGI Symbol;Ac	1
ENSMUSG00000043177	Gm11993	predicted gene 11993 [Source:MGI Symbol;Acc:MG	1
ENSMUSG00000043243	Fam129c	family with sequence similarity 129, member C [Sou	1
ENSMUSG00000043333	Rhbdl2	rhomboid, veinlet-like 2 (Drosophila) [Source:MGI S	1
ENSMUSG00000043727	F830045P16Rik	RIKEN cDNA F830045P16 gene [Source:MGI Symbo	1
ENSMUSG00000043747	1520401A03Rik	RIKEN cDNA 1520401A03 gene [Source:MGI Symbo	1
ENSMUSG00000043787	Defb12	defensin beta 12 [Source:MGI Symbol;Acc:MGI:192	1
ENSMUSG00000043832	Clec4a3	C-type lectin domain family 4, member a3 [Source:	1
ENSMUSG00000043931	Gimap7	GTPase, IMAP family member 7 [Source:MGI Symbo	1
ENSMUSG00000043932	Klri2	killer cell lectin-like receptor family I member 2 [So	1
ENSMUSG00000043948	Olfr691	olfactory receptor 691 [Source:MGI Symbol;Acc:MG	1
ENSMUSG00000043953	Ccrl2	chemokine (C-C motif) receptor-like 2 [Source:MGI	1
ENSMUSG00000044056	Efcab9	EF-hand calcium binding domain 9 [Source:MGI Syn	1
ENSMUSG00000044083	Efcab8	EF-hand calcium binding domain 8 [Source:MGI Syn	1
ENSMUSG00000044141	E130201H02Rik	RIKEN cDNA E130201H02 gene [Source:MGI Symbo	1
ENSMUSG00000044156	Hepacam2	HEPACAM family member 2 [Source:MGI Symbol;A	1

ENSMUSG00000044162	Tnip3	TNFAIP3 interacting protein 3 [Source:MGI Symbol;	1
ENSMUSG00000044172	Ptx4	pentraxin 4 [Source:MGI Symbol;Acc:MGI:1915759]	1
ENSMUSG00000044243	Bhlha9	basic helix-loop-helix family, member a9 [Source:M	1
ENSMUSG00000044303	Cdkn2a	cyclin-dependent kinase inhibitor 2A [Source:MGI S	1
ENSMUSG00000044338	Aplnr	apelin receptor [Source:MGI Symbol;Acc:MGI:1346	1
ENSMUSG00000044341	Gm5601	predicted pseudogene 5601 [Source:MGI Symbol;A	1
ENSMUSG00000044378	Slc15a5	solute carrier family 15, member 5 [Source:MGI Sy	1
ENSMUSG00000044522	A730020M07Rik	RIKEN cDNA A730020M07 gene [Source:MGI Symb	1
ENSMUSG00000044544	4921513I03Rik	RIKEN cDNA 4921513I03 gene [Source:MGI Symbol	1
ENSMUSG00000044574	5031434C07Rik	RIKEN cDNA 5031434C07 gene [Source:MGI Symbo	1
ENSMUSG00000044624	Gm4922	predicted gene 4922 [Source:MGI Symbol;Acc:MGI:	1
ENSMUSG00000044678	Ly6k	lymphocyte antigen 6 complex, locus K [Source:MG	1
ENSMUSG00000044719	E230025N22Rik	Riken cDNA E230025N22 gene [Source:MGI Symbo	1
ENSMUSG00000044751	Gm12231	predicted gene 12231 [Source:MGI Symbol;Acc:MG	1
ENSMUSG00000044860	Gm1123	predicted gene 1123 [Source:MGI Symbol;Acc:MGI:	1
ENSMUSG00000044867	Gm3345	predicted gene 3345 [Source:MGI Symbol;Acc:MGI:	1
ENSMUSG00000045022	1700024P04Rik	RIKEN cDNA 1700024P04 gene [Source:MGI Symbo	1
ENSMUSG00000045091	Aqp12	aquaporin 12 [Source:MGI Symbol;Acc:MGI:266463	1
ENSMUSG00000045165	AI467606	expressed sequence AI467606 [Source:MGI Symbol	1
ENSMUSG00000045178	4933411O13Rik	RIKEN cDNA 4933411O13 gene [Source:MGI Symbo	1
ENSMUSG00000045231	BC106179	cDNA sequence BC106179 [Source:MGI Symbol;Acc	1
ENSMUSG00000045236	Krtap5-4	keratin associated protein 5-4 [Source:MGI Symbol	1
ENSMUSG00000045281	Gpr20	G protein-coupled receptor 20 [Source:MGI Symbo	1
ENSMUSG00000045362	Tnfrsf26	tumor necrosis factor receptor superfamily, memb	1
ENSMUSG00000045378	Pxt1	peroxisomal, testis specific 1 [Source:MGI Symbol;A	1
ENSMUSG00000045464	2810002D19Rik	RIKEN cDNA 2810002D19 gene [Source:MGI Symbo	1
ENSMUSG00000045569	Mc2r	melanocortin 2 receptor [Source:MGI Symbol;Acc:N	1
ENSMUSG00000045620	Odf3l1	outer dense fiber of sperm tails 3-like 1 [Source:MG	1
ENSMUSG00000045680	Tcf21	transcription factor 21 [Source:MGI Symbol;Acc:MG	1
ENSMUSG00000045699	Gm21411	predicted gene, 21411 [Source:MGI Symbol;Acc:MG	1
ENSMUSG00000045746	B230317F23Rik	RIKEN cDNA B230317F23 gene [Source:MGI Symbo	1
ENSMUSG00000045799	Gm9800	predicted gene 9800 [Source:MGI Symbol;Acc:MGI:	1
ENSMUSG00000045813	Gm9801	predicted gene 9801 [Source:MGI Symbol;Acc:MGI:	1
ENSMUSG00000045942	BC049762	cDNA sequence BC049762 [Source:MGI Symbol;Acc	1
ENSMUSG00000045999	AY036118	cDNA sequence AY036118 [Source:MGI Symbol;Acc	1
ENSMUSG00000046005	D830044D21Rik	RIKEN cDNA D830044D21 gene [Source:MGI Symbo	1
ENSMUSG00000046070	Igfals	insulin-like growth factor binding protein, acid labil	1
ENSMUSG00000046119	1110008E08Rik	RIKEN cDNA 1110008E08 gene [Source:MGI Symbo	1
ENSMUSG00000046213	Cym	chymosin [Source:MGI Symbol;Acc:MGI:2684977]	1
ENSMUSG00000046245	Pilra	paired immunoglobin-like type 2 receptor alpha [So	1
ENSMUSG00000046341	Gm11223	predicted gene 11223 [Source:MGI Symbol;Acc:MG	1
ENSMUSG00000046413	D230002A01Rik	RIKEN cDNA D230002A01 gene [Source:MGI Symbo	1
ENSMUSG00000046561	Arsj	arylsulfatase J [Source:MGI Symbol;Acc:MGI:24435	1
ENSMUSG00000046589	Lrrc8e	leucine rich repeat containing 8 family, member E [	1
ENSMUSG00000046694	Fam46b	family with sequence similarity 46, member B [Sour	1
ENSMUSG00000046721	Rpl14-ps1	ribosomal protein L14, pseudogene 1 [Source:MGI	1
ENSMUSG00000046748	2310005G13Rik	RIKEN cDNA 2310005G13 gene [Source:MGI Symbo	1

ENSMUSG00000046916	Myct1	myc target 1 [Source:MGI Symbol;Acc:MGI:191588	1
ENSMUSG00000046971	Pla2g4f	phospholipase A2, group IVF [Source:MGI Symbol;A	1
ENSMUSG00000047053	Teddm1	transmembrane epididymal protein 1 [Source:MGI	1
ENSMUSG00000047061	Gm9817	predicted gene 9817 [Source:MGI Symbol;Acc:MGI:	1
ENSMUSG00000047109	Cldn14	claudin 14 [Source:MGI Symbol;Acc:MGI:1860425]	1
ENSMUSG00000047150	1700001C19Rik	RIKEN cDNA 1700001C19 gene [Source:MGI Symbo	1
ENSMUSG00000047197	Gjd3	gap junction protein, delta 3 [Source:MGI Symbol;A	1
ENSMUSG00000047220	Ccdc36	coiled-coil domain containing 36 [Source:MGI Symb	1
ENSMUSG00000047420	Fam180a	family with sequence similarity 180, member A [Sou	1
ENSMUSG00000047441	Antxrl	anthrax toxin receptor-like [Source:MGI Symbol;Ac	1
ENSMUSG00000047492	Inhbe	inhibin beta E [Source:MGI Symbol;Acc:MGI:10926	1
ENSMUSG00000047511	Olfr1396	olfactory receptor 1396 [Source:MGI Symbol;Acc:M	1
ENSMUSG00000047619	Ddi1	DDI1, DNA-damage inducible 1, homolog 1 (S. cere	1
ENSMUSG00000047794	Olfr685	olfactory receptor 685 [Source:MGI Symbol;Acc:M	1
ENSMUSG00000047798	Cd300lf	CD300 antigen like family member F [Source:MGI S	1
ENSMUSG00000047884	Klk9	kallikrein related-peptidase 9 [Source:MGI Symbol;	1
ENSMUSG00000047938	4930483J18Rik	RIKEN cDNA 4930483J18 gene [Source:MGI Symbol	1
ENSMUSG00000047940	Stpg2	sperm tail PG rich repeat containing 2 [Source:MGI	1
ENSMUSG00000047988	4933428G20Rik	RIKEN cDNA 4933428G20 gene [Source:MGI Symbo	1
ENSMUSG00000047990	C2cd4a	C2 calcium-dependent domain containing 4A [Sour	1
ENSMUSG00000048107	Kctd12	potassium channel tetramerisation domain contain	1
ENSMUSG00000048155	H2bfm	H2B histone family, member M [Source:MGI Symbo	1
ENSMUSG00000048206	Dnajb8	DnaJ (Hsp40) homolog, subfamily B, member 8 [Sou	1
ENSMUSG00000048337	Npy4r	neuropeptide Y receptor Y4 [Source:MGI Symbol;A	1
ENSMUSG00000048473	Gm766	predicted gene 766 [Source:MGI Symbol;Acc:MGI:2	1
ENSMUSG00000048537	Phldb1	pleckstrin homology-like domain, family B, membe	1
ENSMUSG00000048544	5930422O12Rik	RIKEN cDNA 5930422O12 gene [Source:MGI Symbo	1
ENSMUSG00000048621	Gm6377	predicted gene 6377 [Source:MGI Symbol;Acc:MGI:	1
ENSMUSG00000048665	Gm27015	predicted gene, 27015 [Source:MGI Symbol;Acc:M	1
ENSMUSG00000048852	Gm12185	predicted gene 12185 [Source:MGI Symbol;Acc:MG	1
ENSMUSG00000048982	Gphb5	glycoprotein hormone beta 5 [Source:MGI Symbol;	1
ENSMUSG00000049119	Fam110b	family with sequence similarity 110, member B [Sou	1
ENSMUSG00000049123	Catsperg2	catsper channel auxiliary subunit gamma 2 [Source	1
ENSMUSG00000049128	Ivl	involucrin [Source:MGI Symbol;Acc:MGI:96626]	1
ENSMUSG00000049214	Skint7	selection and upkeep of intraepithelial T cells 7 [Sou	1
ENSMUSG00000049246	Hspe1-ps2	heat shock protein 1 (chaperonin 10), pseudogene	1
ENSMUSG00000049291	Prss38	protease, serine, 38 [Source:MGI Symbol;Acc:MGI:	1
ENSMUSG00000049404	Rarres1	retinoic acid receptor responder (tazarotene induce	1
ENSMUSG00000049409	Prokr1	prokineticin receptor 1 [Source:MGI Symbol;Acc:M	1
ENSMUSG00000049576	Zfa-ps	zinc finger protein, autosomal, pseudogene [Source	1
ENSMUSG00000049741	Rxfp4	relaxin family peptide receptor 4 [Source:MGI Symb	1
ENSMUSG00000049830	Gm9839	predicted gene 9839 [Source:MGI Symbol;Acc:MGI:	1
ENSMUSG00000049971	Glt1d1	glycosyltransferase 1 domain containing 1 [Source:	1
ENSMUSG00000049988	Lrrc25	leucine rich repeat containing 25 [Source:MGI Symb	1
ENSMUSG00000050010	Shisa3	shisa homolog 3 (Xenopus laevis) [Source:MGI Sym	1
ENSMUSG00000050058	Prm3	protamine 3 [Source:MGI Symbol;Acc:MGI:106601]	1
ENSMUSG00000050108	Bpifc	BPI fold containing family C [Source:MGI Symbol;Ac	1

ENSMUSG00000050122	Vwa3b	von Willebrand factor A domain containing 3B [Sou	1
ENSMUSG00000050189	Etos1	ectopic ossification 1 [Source:MGI Symbol;Acc:MGI	1
ENSMUSG00000050345	4930486L24Rik	RIKEN cDNA 4930486L24 gene [Source:MGI Symbo	1
ENSMUSG00000050395	Tnfsf15	tumor necrosis factor (ligand) superfamily, membe	1
ENSMUSG00000050410	Tcf19	transcription factor 19 [Source:MGI Symbol;Acc:MC	1
ENSMUSG00000050534	Htr5b	5-hydroxytryptamine (serotonin) receptor 5B [Sour	1
ENSMUSG00000050623	Tex40	testis expressed 40 [Source:MGI Symbol;Acc:MGI:1	1
ENSMUSG00000050700	Emilin3	elastin microfibril interfacer 3 [Source:MGI Symbol;	1
ENSMUSG00000050799	Hist1h2ba	histone cluster 1, H2ba [Source:MGI Symbol;Acc:M	1
ENSMUSG00000050824	Sstr5	somatostatin receptor 5 [Source:MGI Symbol;Acc:N	1
ENSMUSG00000050926	Dcaf12l2	DDB1 and CUL4 associated factor 12-like 2 [Source:	1
ENSMUSG00000050959	Olfr1392	olfactory receptor 1392 [Source:MGI Symbol;Acc:M	1
ENSMUSG00000050994	Adgb	androglobin [Source:MGI Symbol;Acc:MGI:3605549	1
ENSMUSG00000051169	Rpusd3	RNA pseudouridylate synthase domain containing 3	1
ENSMUSG00000051361	6030498E09Rik	RIKEN cDNA 6030498E09 gene [Source:MGI Symbo	1
ENSMUSG00000051378	Kif18b	kinesin family member 18B [Source:MGI Symbol;Ac	1
ENSMUSG00000051439	Cd14	CD14 antigen [Source:MGI Symbol;Acc:MGI:88318]	1
ENSMUSG00000051452	Gm11437	predicted gene 11437 [Source:MGI Symbol;Acc:MG	1
ENSMUSG00000051456	Hspb3	heat shock protein 3 [Source:MGI Symbol;Acc:MGI:	1
ENSMUSG00000051498	Tlr6	toll-like receptor 6 [Source:MGI Symbol;Acc:MGI:13	1
ENSMUSG00000051517	Arhgef39	Rho guanine nucleotide exchange factor (GEF) 39 [S	1
ENSMUSG00000051582	Otud6a	OTU domain containing 6A [Source:MGI Symbol;Ac	1
ENSMUSG00000051590	Map3k19	mitogen-activated protein kinase kinase kinase 19 [	1
ENSMUSG00000051616	C230029F24Rik	RIKEN cDNA C230029F24 gene [Source:MGI Symbo	1
ENSMUSG00000051617	Krt9	keratin 9 [Source:MGI Symbol;Acc:MGI:96696]	1
ENSMUSG00000051805	A530095I07Rik	RIKEN cDNA A530095I07 gene [Source:MGI Symbol	1
ENSMUSG00000051839	Gypa	glycophorin A [Source:MGI Symbol;Acc:MGI:95880]	1
ENSMUSG00000051965	Nanos2	nanos homolog 2 (Drosophila) [Source:MGI Symbol	1
ENSMUSG00000052098	Gm9867	predicted gene 9867 [Source:MGI Symbol;Acc:MGI:	1
ENSMUSG00000052154	Gm9870	predicted gene 9870 [Source:MGI Symbol;Acc:MGI:	1
ENSMUSG00000052157	Gm650	predicted pseudogene 650 [Source:MGI Symbol;Ac	1
ENSMUSG00000052212	Cd177	CD177 antigen [Source:MGI Symbol;Acc:MGI:19161	1
ENSMUSG00000052221	Ppp1r36	protein phosphatase 1, regulatory subunit 36 [Sour	1
ENSMUSG00000052241	A930035D04Rik	RIKEN cDNA A930035D04 gene [Source:MGI Symbo	1
ENSMUSG00000052270	Fpr2	formyl peptide receptor 2 [Source:MGI Symbol;Acc	1
ENSMUSG00000052281	Gm15024	predicted gene 15024 [Source:MGI Symbol;Acc:MG	1
ENSMUSG00000052363	Zdhhc19	zinc finger, DHHC domain containing 19 [Source:MC	1
ENSMUSG00000052471	Gm9881	predicted gene 9881 [Source:MGI Symbol;Acc:MGI:	1
ENSMUSG00000052477	C130026I21Rik	RIKEN cDNA C130026I21 gene [Source:MGI Symbol	1
ENSMUSG00000052482	Gm8174	predicted gene 8174 [Source:MGI Symbol;Acc:MGI:	1
ENSMUSG00000052485	Tmem171	transmembrane protein 171 [Source:MGI Symbol;A	1
ENSMUSG00000052776	Oas1a	2'-5' oligoadenylate synthetase 1A [Source:MGI Syr	1
ENSMUSG00000052979	6530403G13Rik	RIKEN cDNA 6530403G13 gene [Source:MGI Symbo	1
ENSMUSG00000053030	Spink2	serine peptidase inhibitor, Kazal type 2 [Source:MG	1
ENSMUSG00000053044	Cd8b1	CD8 antigen, beta chain 1 [Source:MGI Symbol;Acc	1
ENSMUSG00000053129	Gsx1	GS homeobox 1 [Source:MGI Symbol;Acc:MGI:9584	1
ENSMUSG00000053219	Raet1e	retinoic acid early transcript 1E [Source:MGI Symbo	1

ENSMUSG00000053244	Gm9901	predicted gene 9901 [Source:MGI Symbol;Acc:MGI:	1
ENSMUSG00000053318	Slamf8	SLAM family member 8 [Source:MGI Symbol;Acc:M	1
ENSMUSG00000053358	Gm9905	predicted gene 9905 [Source:MGI Symbol;Acc:MGI:	1
ENSMUSG00000053367	Gm6792	predicted gene 6792 [Source:MGI Symbol;Acc:MGI:	1
ENSMUSG00000053368	Rxfp2	relaxin/insulin-like family peptide receptor 2 [Sourc	1
ENSMUSG00000053461	Hhipl2	hedgehog interacting protein-like 2 [Source:MGI Sy	1
ENSMUSG00000053467	A530088E08Rik	RIKEN cDNA A530088E08 gene [Source:MGI Symbo	1
ENSMUSG00000053583	Gm9912	predicted gene 9912 [Source:MGI Symbol;Acc:MGI:	1
ENSMUSG00000053706	B430305J03Rik	RIKEN cDNA B430305J03 gene [Source:MGI Symbol	1
ENSMUSG00000053719	Klk1b26	kallikrein 1-related petidase b26 [Source:MGI Symb	1
ENSMUSG00000053746	Ptrh1	peptidyl-tRNA hydrolase 1 homolog (S. cerevisiae) [	1
ENSMUSG00000053783	1700016K19Rik	RIKEN cDNA 1700016K19 gene [Source:MGI Symbo	1
ENSMUSG00000053861	Gm9925	predicted gene 9925 [Source:MGI Symbol;Acc:MGI:	1
ENSMUSG00000053957	Gm12474	predicted gene 12474 [Source:MGI Symbol;Acc:MG	1
ENSMUSG00000053980	Gm9930	predicted gene 9930 [Source:MGI Symbol;Acc:MGI:	1
ENSMUSG00000054005	Mill1	MHC I like leukocyte 1 [Source:MGI Symbol;Acc:MG	1
ENSMUSG00000054006	D630008O14Rik	RIKEN cDNA D630008O14 gene [Source:MGI Symbo	1
ENSMUSG00000054052	Rdh19	retinol dehydrogenase 19 [Source:MGI Symbol;Acc	1
ENSMUSG00000054156	Gm4894	predicted gene 4894 [Source:MGI Symbol;Acc:MGI:	1
ENSMUSG00000054320	Lrrc36	leucine rich repeat containing 36 [Source:MGI Sym	1
ENSMUSG00000054325	Lce3a	late cornified envelope 3A [Source:MGI Symbol;Acc	1
ENSMUSG00000054453	Sytl5	synaptotagmin-like 5 [Source:MGI Symbol;Acc:MGI	1
ENSMUSG00000054457	9430021M05Rik	RIKEN cDNA 9430021M05 gene [Source:MGI Symbo	1
ENSMUSG00000054474	Thnsl2	threonine synthase-like 2 (bacterial) [Source:MGI S	1
ENSMUSG00000054555	Adam12	a disintegrin and metallopeptidase domain 12 (mel	1
ENSMUSG00000054588	Gbp10	guanylate-binding protein 10 [Source:MGI Symbol;	1
ENSMUSG00000054622	D730045B01Rik	RIKEN cDNA D730045B01 gene [Source:MGI Symbo	1
ENSMUSG00000054662	Ano9	anoctamin 9 [Source:MGI Symbol;Acc:MGI:191859	1
ENSMUSG00000054667	Irs4	insulin receptor substrate 4 [Source:MGI Symbol;Ac	1
ENSMUSG00000054745	Gm9955	predicted gene 9955 [Source:MGI Symbol;Acc:MGI:	1
ENSMUSG00000054746	Abca15	ATP-binding cassette, sub-family A (ABC1), membe	1
ENSMUSG00000054763	Defb42	defensin beta 42 [Source:MGI Symbol;Acc:MGI:303	1
ENSMUSG00000054966	Ifltd1	intermediate filament tail domain containing 1 [Sou	1
ENSMUSG00000054988	Agtr1b	angiotensin II receptor, type 1b [Source:MGI Symbo	1
ENSMUSG00000055015	Gm9961	predicted gene 9961 [Source:MGI Symbol;Acc:MGI:	1
ENSMUSG00000055048	Gm9962	predicted gene 9962 [Source:MGI Symbol;Acc:MGI:	1
ENSMUSG00000055109	Gm15155	predicted gene 15155 [Source:MGI Symbol;Acc:MG	1
ENSMUSG00000055114	Anxa13	annexin A13 [Source:MGI Symbol;Acc:MGI:191703	1
ENSMUSG00000055134	9130017K11Rik	RIKEN cDNA 9130017K11 gene [Source:MGI Symbo	1
ENSMUSG00000055159	4930583K01Rik	RIKEN cDNA 4930583K01 gene [Source:MGI Symbo	1
ENSMUSG00000055271	9330161L09Rik	RIKEN cDNA 9330161L09 gene [Source:MGI Symbo	1
ENSMUSG00000055357	4933400A11Rik	RIKEN cDNA 4933400A11 gene [Source:MGI Symbo	1
ENSMUSG00000055413	H2-Q5	histocompatibility 2, Q region locus 5 [Source:MGI	1
ENSMUSG00000055547	Apobec4	apolipoprotein B mRNA editing enzyme, catalytic p	1
ENSMUSG00000055657	E030030I06Rik	RIKEN cDNA E030030I06 gene [Source:MGI Symbol	1
ENSMUSG00000055691	Gja6	gap junction protein, alpha 6 [Source:MGI Symbol;	1
ENSMUSG00000055972	2810407A14Rik	RIKEN cDNA 2810407A14 gene [Source:MGI Symbo	1

ENSMUSG00000055978	Fut2	fucosyltransferase 2 [Source:MGI Symbol;Acc:MGI:	1
ENSMUSG00000056054	S100a8	S100 calcium binding protein A8 (calgranulin A) [So	1
ENSMUSG00000056061	Gm14318	predicted gene 14318 [Source:MGI Symbol;Acc:MG	1
ENSMUSG00000056332	Gm16294	predicted gene 16294 [Source:MGI Symbol;Acc:MG	1
ENSMUSG00000056412	Gm12396	predicted gene 12396 [Source:MGI Symbol;Acc:MG	1
ENSMUSG00000056501	Cebpb	CCAAT/enhancer binding protein (C/EBP), beta [Sou	1
ENSMUSG00000056718	Gm13199	predicted gene 13199 [Source:MGI Symbol;Acc:MG	1
ENSMUSG00000056797	Gm4889	predicted gene 4889 [Source:MGI Symbol;Acc:MGI:	1
ENSMUSG00000056849	Gm16415	predicted pseudogene 16415 [Source:MGI Symbol;	1
ENSMUSG00000057000	Nxf3	nuclear RNA export factor 3 [Source:MGI Symbol;A	1
ENSMUSG00000057068	Fam47e	family with sequence similarity 47, member E [Sour	1
ENSMUSG00000057135	Scimp	SLP adaptor and CSK interacting membrane protein	1
ENSMUSG00000057157	Gm6054	predicted gene 6054 [Source:MGI Symbol;Acc:MGI:	1
ENSMUSG00000057465	Saa2	serum amyloid A 2 [Source:MGI Symbol;Acc:MGI:98	1
ENSMUSG00000057499	Gm6177	predicted gene 6177 [Source:MGI Symbol;Acc:MGI:	1
ENSMUSG00000057802	Gm10030	predicted gene 10030 [Source:MGI Symbol;Acc:MG	1
ENSMUSG00000057913	Gm10032	predicted gene 10032 [Source:MGI Symbol;Acc:MG	1
ENSMUSG00000057945	Gm17136	predicted gene 17136 [Source:MGI Symbol;Acc:MG	1
ENSMUSG00000058028	Zscan5b	zinc finger and SCAN domain containing 5B [Source	1
ENSMUSG00000058050	Gm9234	predicted pseudogene 9234 [Source:MGI Symbol;A	1
ENSMUSG00000058064	Gm10036	predicted gene 10036 [Source:MGI Symbol;Acc:MG	1
ENSMUSG00000058126	Tpm3-rs7	tropomyosin 3, related sequence 7 [Source:MGI Sy	1
ENSMUSG00000058159	T2	brachyury 2 [Source:MGI Symbol;Acc:MGI:104658]	1
ENSMUSG00000058281	Gm10038	predicted gene 10038 [Source:MGI Symbol;Acc:MG	1
ENSMUSG00000058542	Gm15590	predicted gene 15590 [Source:MGI Symbol;Acc:MG	1
ENSMUSG00000058579	Cela2a	chymotrypsin-like elastase family, member 2A [Sou	1
ENSMUSG00000058603	Rpl28-ps1	ribosomal protein L28, pseudogene 1 [Source:MGI	1
ENSMUSG00000058809	Gm12141	predicted gene 12141 [Source:MGI Symbol;Acc:MG	1
ENSMUSG00000058838	Rps27a-ps2	ribosomal protein S27A, pseudogene 2 [Source:MG	1
ENSMUSG00000058953	Gm4822	predicted pseudogene 4822 [Source:MGI Symbol;A	1
ENSMUSG00000058976	Usp17lc	ubiquitin specific peptidase 17-like C [Source:MGI S	1
ENSMUSG00000058997	Vwa8	von Willebrand factor A domain containing 8 [Sour	1
ENSMUSG00000059022	Kcp	kielin/chordin-like protein [Source:MGI Symbol;Acc	1
ENSMUSG00000059027	9630013D21Rik	RIKEN cDNA 9630013D21 gene [Source:MGI Symbo	1
ENSMUSG00000059060	Rad51b	RAD51 homolog B [Source:MGI Symbol;Acc:MGI:10	1
ENSMUSG00000059195	Gm12715	predicted gene 12715 [Source:MGI Symbol;Acc:MG	1
ENSMUSG00000059305	Vpreb1	pre-B lymphocyte gene 1 [Source:MGI Symbol;Acc:	1
ENSMUSG00000059383	Gfral	GDNF family receptor alpha like [Source:MGI Symb	1
ENSMUSG00000059408	Mrgprh	MAS-related GPR, member H [Source:MGI Symbol;	1
ENSMUSG00000059455	Plac8l1	PLAC8-like 1 [Source:MGI Symbol;Acc:MGI:191665	1
ENSMUSG00000059481	Plg	plasminogen [Source:MGI Symbol;Acc:MGI:97620]	1
ENSMUSG00000059483	Gm5528	predicted gene 5528 [Source:MGI Symbol;Acc:MGI:	1
ENSMUSG00000059625	Sohlh1	spermatogenesis and oogenesis specific basic helix-	1
ENSMUSG00000059854	Hydin	HYDIN, axonemal central pair apparatus protein [So	1
ENSMUSG00000059883	Irak4	interleukin-1 receptor-associated kinase 4 [Source:	1
ENSMUSG00000059970	Hspa2	heat shock protein 2 [Source:MGI Symbol;Acc:MGI:	1
ENSMUSG00000060034	Ctf2	cardiotrophin 2 [Source:MGI Symbol;Acc:MGI:2684	1

ENSMUSG00000060081	Hist1h2aa	histone cluster 1, H2aa [Source:MGI Symbol;Acc:M	1
ENSMUSG00000060096	Amd-ps3	S-adenosylmethionine decarboxylase, pseudogene	1
ENSMUSG00000060131	Atp8b4	ATPase, class I, type 8B, member 4 [Source:MGI Sy	1
ENSMUSG00000060183	Cxcl11	chemokine (C-X-C motif) ligand 11 [Source:MGI Syn	1
ENSMUSG00000060198	Gm11353	predicted gene 11353 [Source:MGI Symbol;Acc:MG	1
ENSMUSG00000060201	Spink7	serine peptidase inhibitor, Kazal type 7 (putative) [S	1
ENSMUSG00000060209	Gm6570	predicted gene 6570 [Source:MGI Symbol;Acc:MGI:	1
ENSMUSG00000060268	Gm1661	predicted gene 1661 [Source:MGI Symbol;Acc:MGI:	1
ENSMUSG00000060332	Tmc2	transmembrane channel-like gene family 2 [Source	1
ENSMUSG00000060416	Gm839	predicted gene 839 [Source:MGI Symbol;Acc:MGI:2	1
ENSMUSG00000060438	Rps10-ps1	ribosomal protein S10, pseudogene 1 [Source:MGI	1
ENSMUSG00000060461	Dppa5a	developmental pluripotency associated 5A [Source	1
ENSMUSG00000060508	Nlrp9b	NLR family, pyrin domain containing 9B [Source:MG	1
ENSMUSG00000060512	0610040J01Rik	RIKEN cDNA 0610040J01 gene [Source:MGI Symbol	1
ENSMUSG00000060566	Rpl21-ps3	ribosomal protein L21, pseudogene 3 [Source:MGI	1
ENSMUSG00000060615	Ang4	angiogenin, ribonuclease A family, member 4 [Sour	1
ENSMUSG00000060630	Zfp735	zinc finger protein 735 [Source:MGI Symbol;Acc:MG	1
ENSMUSG00000060730	Gm10086	predicted pseudogene 10086 [Source:MGI Symbol;	1
ENSMUSG00000060878	Olfr1420	olfactory receptor 1420 [Source:MGI Symbol;Acc:M	1
ENSMUSG00000060962	Dmkn	dermokine [Source:MGI Symbol;Acc:MGI:1920962]	1
ENSMUSG00000061531	Tmem236	transmembrane protein 236 [Source:MGI Symbol;A	1
ENSMUSG00000061577	Gpr114	G protein-coupled receptor 114 [Source:MGI Symb	1
ENSMUSG00000061615	Hist1h2ab	histone cluster 1, H2ab [Source:MGI Symbol;Acc:M	1
ENSMUSG00000061616	Olfr12	olfactory receptor 12 [Source:MGI Symbol;Acc:MGI	1
ENSMUSG00000061619	Vma21-ps	VMA21 vacuolar H+-ATPase homolog (S. cerevisiae	1
ENSMUSG00000061728	Btnl7-ps	butyrophilin-like 7, pseudogene [Source:MGI Symb	1
ENSMUSG00000061815	Rufy4	RUN and FYVE domain containing 4 [Source:MGI Sy	1
ENSMUSG00000061843	Vmn1r-ps32	vomeronasal 1 receptor, pseudogene 32 [Source:M	1
ENSMUSG00000061877	BC048679	cDNA sequence BC048679 [Source:MGI Symbol;Acc	1
ENSMUSG00000061947	Serpina10	serine (or cysteine) peptidase inhibitor, clade A (alp	1
ENSMUSG00000061952	Olfr1377	olfactory receptor 1377 [Source:MGI Symbol;Acc:M	1
ENSMUSG00000061988	Rpl10a-ps2	ribosomal protein L10A, pseudogene 2 [Source:MG	1
ENSMUSG00000062007	Hsh2d	hematopoietic SH2 domain containing [Source:MG	1
ENSMUSG00000062152	Rpl21-ps14	ribosomal protein L21, pseudogene 14 [Source:MG	1
ENSMUSG00000062235	Gm5341	predicted pseudogene 5341 [Source:MGI Symbol;A	1
ENSMUSG00000062279	Gm10237	predicted gene 10237 [Source:MGI Symbol;Acc:MG	1
ENSMUSG00000062383	B130024G19Rik	RIKEN cDNA B130024G19 gene [Source:MGI Symbo	1
ENSMUSG00000062393	Dgkk	diacylglycerol kinase kappa [Source:MGI Symbol;Ac	1
ENSMUSG00000062410	Hsd3b3	hydroxy-delta-5-steroid dehydrogenase, 3 beta- an	1
ENSMUSG00000062501	4930425F17Rik	RIKEN cDNA 4930425F17 gene [Source:MGI Symbo	1
ENSMUSG00000062585	Cnr2	cannabinoid receptor 2 (macrophage) [Source:MGI	1
ENSMUSG00000062588	Gm6104	predicted gene 6104 [Source:MGI Symbol;Acc:MGI:	1
ENSMUSG00000062783	Csprs	component of Sp100-rs [Source:MGI Symbol;Acc:M	1
ENSMUSG00000062896	Rpl31-ps11	ribosomal protein L31, pseudogene 11 [Source:MG	1
ENSMUSG00000062939	Stat4	signal transducer and activator of transcription 4 [S	1
ENSMUSG00000063011	Msln	mesothelin [Source:MGI Symbol;Acc:MGI:1888992]	1
ENSMUSG00000063018	2010204K13Rik	RIKEN cDNA 2010204K13 gene [Source:MGI Symbo	1

ENSMUSG00000063177	Klk1b27	kallikrein 1-related peptidase b27 [Source:MGI Sym	1
ENSMUSG00000063442	Gm7117	predicted pseudogene 7117 [Source:MGI Symbol;A	1
ENSMUSG00000063529	Stmnd1	stathmin domain containing 1 [Source:MGI Symbol	1
ENSMUSG00000063535	Zfp773	zinc finger protein 773 [Source:MGI Symbol;Acc:MG	1
ENSMUSG00000063651	Cnfn	cornifelin [Source:MGI Symbol;Acc:MGI:1919633]	1
ENSMUSG00000063886	Gm10138	predicted gene 10138 [Source:MGI Symbol;Acc:MG	1
ENSMUSG00000063902	Gm7964	predicted gene 7964 [Source:MGI Symbol;Acc:MGI:	1
ENSMUSG00000063975	Slco1a5	solute carrier organic anion transporter family, mer	1
ENSMUSG00000064036	Mro	maestro [Source:MGI Symbol;Acc:MGI:2152817]	1
ENSMUSG00000064052	Gm5089	predicted gene 5089 [Source:MGI Symbol;Acc:MGI:	1
ENSMUSG00000064231	Gm5321	predicted gene 5321 [Source:MGI Symbol;Acc:MGI:	1
ENSMUSG00000064308	2410137M14Rik	RIKEN cDNA 2410137M14 gene [Source:MGI Symbo	1
ENSMUSG00000064371	mt-Tt	mitochondrially encoded tRNA threonine [Source:M	1
ENSMUSG00000064380	Gm26448	predicted gene, 26448 [Source:MGI Symbol;Acc:MG	1
ENSMUSG00000064427	Gm22748	predicted gene, 22748 [Source:MGI Symbol;Acc:MG	1
ENSMUSG00000064441	Snord37	small nucleolar RNA, C/D box 37 [Source:MGI Symb	1
ENSMUSG00000064443	Gm26226	predicted gene, 26226 [Source:MGI Symbol;Acc:MG	1
ENSMUSG00000064476	Gm23405	predicted gene, 23405 [Source:MGI Symbol;Acc:MG	1
ENSMUSG00000064491	Gm22061	predicted gene, 22061 [Source:MGI Symbol;Acc:MG	1
ENSMUSG00000064494	Gm22063	predicted gene, 22063 [Source:MGI Symbol;Acc:MG	1
ENSMUSG00000064508	n-R5s96	nuclear encoded rRNA 5S 96 [Source:MGI Symbol;A	1
ENSMUSG00000064519	n-R5s97	nuclear encoded rRNA 5S 97 [Source:MGI Symbol;A	1
ENSMUSG00000064542	Gm22980	predicted gene, 22980 [Source:MGI Symbol;Acc:MG	1
ENSMUSG00000064565	Gm24145	predicted gene, 24145 [Source:MGI Symbol;Acc:MG	1
ENSMUSG00000064600	Gm25636	predicted gene, 25636 [Source:MGI Symbol;Acc:MG	1
ENSMUSG00000064624	Gm22305	predicted gene, 22305 [Source:MGI Symbol;Acc:MG	1
ENSMUSG00000064655	Gm25788	predicted gene, 25788 [Source:MGI Symbol;Acc:MG	1
ENSMUSG00000064669	Gm24461	predicted gene, 24461 [Source:MGI Symbol;Acc:MG	1
ENSMUSG00000064671	Gm22807	predicted gene, 22807 [Source:MGI Symbol;Acc:MG	1
ENSMUSG00000064704	n-R5s180	nuclear encoded rRNA 5S 180 [Source:MGI Symbol;	1
ENSMUSG00000064722	Gm25857	predicted gene, 25857 [Source:MGI Symbol;Acc:MG	1
ENSMUSG00000064738	Gm23054	predicted gene, 23054 [Source:MGI Symbol;Acc:MG	1
ENSMUSG00000064770	n-R5s29	nuclear encoded rRNA 5S 29 [Source:MGI Symbol;A	1
ENSMUSG00000064811	Gm22358	predicted gene, 22358 [Source:MGI Symbol;Acc:MG	1
ENSMUSG00000064851	Gm24525	predicted gene, 24525 [Source:MGI Symbol;Acc:MG	1
ENSMUSG00000064857	n-R5s25	nuclear encoded rRNA 5S 25 [Source:MGI Symbol;A	1
ENSMUSG00000064871	Snord58b	small nucleolar RNA, C/D box 58B [Source:MGI Sym	1
ENSMUSG00000064919	Gm22572	predicted gene, 22572 [Source:MGI Symbol;Acc:MG	1
ENSMUSG00000064952	Gm24920	predicted gene, 24920 [Source:MGI Symbol;Acc:MG	1
ENSMUSG00000064960	Gm24406	predicted gene, 24406 [Source:MGI Symbol;Acc:MG	1
ENSMUSG00000064999	Gm26035	predicted gene, 26035 [Source:MGI Symbol;Acc:MG	1
ENSMUSG00000065130	Gm24457	predicted gene, 24457 [Source:MGI Symbol;Acc:MG	1
ENSMUSG00000065162	Gm23645	predicted gene, 23645 [Source:MGI Symbol;Acc:MG	1
ENSMUSG00000065196	Snord85	small nucleolar RNA, C/D box 85 [Source:MGI Symb	1
ENSMUSG00000065219	Snord32a	small nucleolar RNA, C/D box 32A [Source:MGI Sym	1
ENSMUSG00000065315	Gm26064	predicted gene, 26064 [Source:MGI Symbol;Acc:MG	1
ENSMUSG00000065361	Gm24413	predicted gene, 24413 [Source:MGI Symbol;Acc:MG	1

ENSMUSG00000065399	Mir133a-1	microRNA 133a-1 [Source:MGI Symbol;Acc:MGI:26	1
ENSMUSG00000065421	Mirlet7a-1	microRNA let7a-1 [Source:MGI Symbol;Acc:MGI:26	1
ENSMUSG00000065430	Mir26a-2	microRNA 26a-2 [Source:MGI Symbol;Acc:MGI:361	1
ENSMUSG00000065433	Mir370	microRNA 370 [Source:MGI Symbol;Acc:MGI:36193	1
ENSMUSG00000065435	Mir135b	microRNA 135b [Source:MGI Symbol;Acc:MGI:3618	1
ENSMUSG00000065436	Mir342	microRNA 342 [Source:MGI Symbol;Acc:MGI:36193	1
ENSMUSG00000065479	Mir125a	microRNA 125a [Source:MGI Symbol;Acc:MGI:2676	1
ENSMUSG00000065483	Mir181c	microRNA 181c [Source:MGI Symbol;Acc:MGI:3618	1
ENSMUSG00000065485	Mir219-2	microRNA 219-2 [Source:MGI Symbol;Acc:MGI:361	1
ENSMUSG00000065490	Mir30c-1	microRNA 30c-1 [Source:MGI Symbol;Acc:MGI:267	1
ENSMUSG00000065514	Mir106b	microRNA 106b [Source:MGI Symbol;Acc:MGI:3619	1
ENSMUSG00000065523	Mir192	microRNA 192 [Source:MGI Symbol;Acc:MGI:26768	1
ENSMUSG00000065526	Mir337	microRNA 337 [Source:MGI Symbol;Acc:MGI:36193	1
ENSMUSG00000065528	Mir320	microRNA 320 [Source:MGI Symbol;Acc:MGI:36193	1
ENSMUSG00000065544	Mir32	microRNA 32 [Source:MGI Symbol;Acc:MGI:361933	1
ENSMUSG00000065547	Mir199a-1	microRNA 199a-1 [Source:MGI Symbol;Acc:MGI:26	1
ENSMUSG00000065562	Mir215	microRNA 215 [Source:MGI Symbol;Acc:MGI:26768	1
ENSMUSG00000065565	Mir181a-1	microRNA 181a-1 [Source:MGI Symbol;Acc:MGI:36	1
ENSMUSG00000065568	Mir344	microRNA 344 [Source:MGI Symbol;Acc:MGI:36193	1
ENSMUSG00000065579	Mir425	microRNA 425 [Source:MGI Symbol;Acc:MGI:36194	1
ENSMUSG00000065581	Mir194-1	microRNA 194-1 [Source:MGI Symbol;Acc:MGI:267	1
ENSMUSG00000065593	Mir339	microRNA 339 [Source:MGI Symbol;Acc:MGI:36193	1
ENSMUSG00000065599	Mir23b	microRNA 23b [Source:MGI Symbol;Acc:MGI:26768	1
ENSMUSG00000065600	Mir338	microRNA 338 [Source:MGI Symbol;Acc:MGI:36193	1
ENSMUSG00000065603	Mir218-1	microRNA 218-1 [Source:MGI Symbol;Acc:MGI:361	1
ENSMUSG00000065608	Mirlet7c-2	microRNA let7c-2 [Source:MGI Symbol;Acc:MGI:36	1
ENSMUSG00000065612	Mir151	microRNA 151 [Source:MGI Symbol;Acc:MGI:26768	1
ENSMUSG00000065651	Gm25973	predicted gene, 25973 [Source:MGI Symbol;Acc:M	1
ENSMUSG00000065657	Gm25337	predicted gene, 25337 [Source:MGI Symbol;Acc:M	1
ENSMUSG00000065663	Gm22579	predicted gene, 22579 [Source:MGI Symbol;Acc:M	1
ENSMUSG00000065669	Gm22581	predicted gene, 22581 [Source:MGI Symbol;Acc:M	1
ENSMUSG00000065687	Gm23734	predicted gene, 23734 [Source:MGI Symbol;Acc:M	1
ENSMUSG00000065725	Gm26165	predicted gene, 26165 [Source:MGI Symbol;Acc:M	1
ENSMUSG00000065728	Gm26175	predicted gene, 26175 [Source:MGI Symbol;Acc:M	1
ENSMUSG00000065750	Gm23346	predicted gene, 23346 [Source:MGI Symbol;Acc:M	1
ENSMUSG00000065808	Gm23037	predicted gene, 23037 [Source:MGI Symbol;Acc:M	1
ENSMUSG00000065817	Gm24698	predicted gene, 24698 [Source:MGI Symbol;Acc:M	1
ENSMUSG00000065824	Gm26315	predicted gene, 26315 [Source:MGI Symbol;Acc:M	1
ENSMUSG00000065825	Gm26314	predicted gene, 26314 [Source:MGI Symbol;Acc:M	1
[truncated: 442,240 more chars]
